# Comparative mangrove metagenome reveals global prevalence of heavy metals and antibiotic resistome across different ecosystems

**DOI:** 10.1038/s41598-018-29521-4

**Published:** 2018-07-25

**Authors:** Madangchanok Imchen, Ranjith Kumavath, Debmalya Barh, Aline Vaz, Aristóteles Góes-Neto, Sandeep Tiwari, Preetam Ghosh, Alice R. Wattam, Vasco Azevedo

**Affiliations:** 1 Department of Genomic Science, School of Biological Sciences, Central University of Kerala, Tejaswini Hills, Periya P.O, Kasaragod, Kerala 671316 India; 2 Centre for Genomics and Applied Gene Technology, Institute of Integrative Omics and Applied Biotechnology (IIOAB), Nonakuri, Purba Medinipur, West Bengal India; 3 Division of Bioinformatics and Computational Genomics, NITTE University Center for Science Education and Research (NUCSER), NITTE (Deemed to be University), Deralakatte, Mangaluru, Karnataka India; 4 Molecular and Computational Biology of Fungi Laboratory, Department of Microbiology, Institute of Biological Sciences (ICB), Federal University of Minas Gerais (UFMG), Pampulha, Belo Horizonte, Minas Gerais Brazil; 5 Laboratório de Genética Celular e Molecular, Departamento de Biologia Geral, Instituto de Ciências Biológicas (ICB), Universidade Federal de Minas Gerais, Pampulha, Belo Horizonte, Minas Gerais Brazil; 6 Department of Computer Science Virginia Commonwealth University, Virginia, 23284 USA; 7 Biocomplexity Institute, Virginia Tech University, Blacksburg, Virginia, 24061 USA

## Abstract

The mangrove ecosystem harbors a complex microbial community that plays crucial role in biogeochemical cycles. In this study, we analyzed mangrove sediments from India using *de novo* whole metagenome next generation sequencing (NGS) and compared their taxonomic and functional community structures to mangrove metagenomics samples from Brazil and Saudi Arabia. The most abundant phyla in the mangroves of all three countries was Proteobacteria, followed by Firmicutes and Bacteroidetes. A total of 1,942 genes were found to be common across all the mangrove sediments from each of the three countries. The mangrove resistome consistently showed high resistance to fluoroquinolone and acriflavine. A comparative study of the mangrove resistome with other ecosystems shows a higher frequency of heavy metal resistance in mangrove and terrestrial samples. Ocean samples had a higher abundance of drug resistance genes with fluoroquinolone and methicillin resistance genes being as high as 28.178% ± 3.619 and 10.776% ± 1.823. Genes involved in cobalt-zinc-cadmium resistance were higher in the mangrove (23.495% ± 4.701) and terrestrial (27.479% ± 4.605) ecosystems. Our comparative analysis of samples collected from a variety of habitats shows that genes involved in resistance to both heavy metals and antibiotics are ubiquitous, irrespective of the ecosystem examined.

## Introduction

Mangroves are estuarine ecosystems composed of saline tolerant plants and are found in 60–70% of the coastal areas, exclusively in tropical and subtropical regions^1^. They are exposed to fresh and oceanic water, experiencing a wide variation of salinity throughout the tidal cycles^2^. Mangroves are important as they are a rich reservoir of microbial diversity and act as a buffer zone between land and sea. Furthermore, mangroves are also a source of novel enzymes and small biomolecules such as LipA-like lipase^3^, aspergilumamide-A peptide^4^, pyrrolizidine alkaloid penibruguieramine-A^5^, GH44 family endoglucanase^6^, pullularins E, F peptides^7^ and salt-tolerant endo-β-1, 4-glucanase Cel5A^8^. They also serve as a potential phytostabilizer to absorb heavy metal pollutants in industrial areas^9^. In addition, recent studies have shown that mangroves can enhance fish abundance^10^ and provide an optimal environment for microbial communities, which, in turn, help in nutrient recycling, by sulphate-reducing bacteria (SRB), methanogenic archaea^11^. Unfortunately, mangroves are under the threat of extinction, having experienced 35% in habitat loss in the last quarter century due to human activities^12,13^. In spite of the need for extensive studies on mangroves microbial community, they have largely been neglected^14,15^.

As most microorganisms are unculturable, traditional culture-dependent and fingerprinting methods have been inadequate in accessing the taxonomic and functional diversity of these ecosystems^1,16^. There are few metagenomic studies about the microbial communities in mangrove from the Brazil^17^, India^18^ and the Red Sea region of Saudi Arabia of the grey mangrove *Avicennia marina*^19^. Our recent report on mangrove ecosystem has focused on microbial community structure and an overview on functional capabilities^18^.

The primary objective of our work is to compare the structure and function of the biotic communities of mangroves in India, Brazil, and Saudi Arabia. The following topics are addressed by our study: (i) the comparison of the taxa composition, richness and relative abundance among the study areas; and (ii) functional diversity analyses for the gene composition, richness and relative abundance of genes among the study areas. A robust analysis was performed for preferential metabolic process, drug and heavy metal resistomes, that were further compared among distinct ecosystems.

## Materials and Methods

### Sampling

The samples were collected in 2015 during the month of mid-December in the following four sites Kumbla (KMA) (N12°35′39.101″, E74°56′47.842″), Valpadananam (VPM) (N9° 59′ 47.636″, E76° 14′ 49.882″), Kavayi (KAY) (N12° 5′ 17.83″, E75° 10′ 33.706″) and Bangramanjeshwar (BHN) (N12° 42′ 29.998″, E74° 54′ 2.716″), all of which are located within Kerala, India. All the samples had indirect exposure to anthropogenic activities resulting from household drainage. Three soil subsamples (~250 g) of the mangrove rhizosphere from the upper 20 cm depth were collected from each site using both hand gloves and sterilized polythene bags. All the samples were transported to the laboratory and processed within 48 hours.

### Metagenomic DNA extraction

Metagenomic DNA from the samples was extracted using soil extraction kits (MoBio PowerSoil). There was deviation from the manufacturer’s instructions, with the elution time extended to 30 minutes at 37 °C. The subsamples in triplicates from the same collection site were pooled and were sequenced using Illumina HiSeq platform 2500 at SciGenome Labs Pvt Ltd, Cochin (India).

### Quality control and annotation pipeline of the indian samples

The raw fastQ reads of Kumbla (KMA), Valpadananam (VPM), Kavayi (KAY) and Bangramanjeshwar (BHN) samples were uploaded to the Metagenome Rapid Annotation using Subsystem Technology (MG-RAST) server (http://metagenomics.anl.gov/)^20^ for analysis. The pipeline, in brief, joins the mate pairs and trims off low quality regions using SolexaQA^21^ followed by dereplication and artificially duplicated reads (ADRs) analysis^22^ using DRISEE (Duplicate Read Inferred Sequencing Error Estimation)^23^. Sequences showing similarity to fly, mouse, cow and humans were removed using Bowtie^24^. Annotation was done against the RefSeq^25^ and Subsystems database^26,27^ for diversity and functional analysis, respectively.

### Comparative analysis of metagenomes across distinct mangrove areas

The metagenomic data from this study was compared to samples from Brazil^17^ and Saudi Arabia^19^, both of which are available at MG-RAST server (Table 1). Brazilian mangrove data^17^ consisted of four different samples that had different anthropogenic impacts. The BrMgv01 and BrMgv02 samples were obtained from two different sites that had experienced an oil spill in 1983. The first sample did not show any strong effects from the oil spill but the second sample still show oil effects. BrMgv3 was collected in a site near an urban area while the last sample, BrMgv04, was isolated from what was determined to be pristine conditions. A second dataset collected in Saudi Arabia also had a total of four samples (RSMgr01, RSMgr02, RSMgr03 and RSMgr04) collected from the rhizosphere of *Avicennia marina*, commonly known as the grey mangrove, in the Red Sea^19^. All samples are available at MG-RAST.

**Table 1:** Samples from this study and our previous study along with other publicly available datasets were compared for diversity and functional analysis.

Sample ID	MG-RAST ID	Location	Country	Sample Type	Sample Info	Reference
MG_KAY	mgm4667575.3	Valapattanam	India	Mangrove	Mangrove rhizosphere from Arabian Sea coast with moderate impact from anthropogenic activities.	This study
MG_VPM	mgm4667708.3	Kumbla	India	Mangrove		
MG_BNH	mgm4667773.3	Kavvayi	India	Mangrove		
MG_KMA	mgm4667861.3	Bangramanjeshwar	India	Mangrove		
PGD	mgm4671368.3	Panangod	India	Mangrove		Imchen *et al*.^18^
MAL	mgm4671369.3	Madakal	India	Mangrove		
PYN	mgm4671370.3	Pyannur	India	Mangrove		
VL1	mgm4671371.3	Vallarpadam	India	Mangrove		
BRMgv-1	mgm4451033.3	Bertioga	Brazil	Mangrove	Area free of oil contamination	Andreote *et al*.^17^
BRMgv-2	mgm4451034.3	Bertioga	Brazil	Mangrove	Area highly impacted by the oil contamination	
BrMgv-3	mgm4451035.3	Bertioga	Brazil	Mangrove	Mangrove near the city, under anthropogenic pressure	
BrMgv-4	mgm4451036.3	Cananeia	Brazil	Mangrove	Located in a preservation area, under pristine conditions	
RSMgr01	mgm4523017.3	Thuwal	Saudi Arabia	Mangrove	Gray mangroves (*Avicennia marina*) samples collected from a 10 cm depth in Red Sea	Alzubaidy *et al*.^19^
RSMgr02	mgm4523018.3	Thuwal	Saudi Arabia	Mangrove		
RSMgr03	mgm4523019.3	Thuwal	Saudi Arabia	Mangrove		
RSMgr04	mgm4523020.3	Thuwal	Saudi Arabia	Mangrove		
Sargasso Station 11 (GS000a)	mgm4441570.3	Sargasso Sea	Bermuda	Ocean	Ocean Sample	Global Ocean Sampling Expedition (Rusch *et al*.^31^; Williamson *et al*.^32^)
North American East Coast (GS013)	mgm4441585.3	Off Nags Head, NC	United States of America	Ocean		
Panama Canal (GS020)	mgm4441590.3	Lake Gatun, Panama	Panama	Ocean		
Eastern Tropical Pacific (GS021)	mgm4441591.3	Eastern Tropical Pacific, Gulf of Panama	Panama	Ocean		
Galapagos Islands (GS027)	mgm4441595.3	Galapagos Islands, Devil	Ecuador	Ocean		
Galapagos Islands (GS028)	mgm4441596.4	Galapagos Islands, Coastal Floreana	Ecuador	Ocean		
Hypersaline Lagoon (GS033)	mgm4441599.3	Punta Cormorant, Hypersaline Lagoon, Floreana Island	Ecuador	Ocean		
Wolf Island (GS035)	mgm4441601.3	Galapagos Islands - Wolf Island	Ecuador	Ocean		
Indian Ocean (GS113)	mgm4441610.3	Indian Ocean	NA	Ocean		
West of the Seychelles (GS114)	mgm4441611.3	Indian Ocean - 500 Miles west of the Seychelles in the Indian Ocean	NA	Ocean		
St. Anne Island (GS117a)	mgm4441613.3	Indian Ocean - St. Anne Island, Seychelles	Seychelles	Ocean		
Indian Ocean (GS121)	mgm4441614.3	Indian Ocean - International water between Madagascar and South Africa	NA	Ocean		
West coast Zanzibar (GS149)	mgm4441618.3	Indian Ocean - West coast Zanzibar (Tanzania), harbour region	Tanzania	Ocean		
Eastern Tropical Pacific (GS023)	mgm4441661.3	Eastern Tropical Pacific, 30 miles from Cocos Island	Costa Rica	Ocean		
Warm seep, Roca Redonda (GS030)	mgm4441662.3	Galapagos Islands - Warm seep, Roca Redonda	Ecuador	Ocean		
Fernandina Island (GS030)	mgm4442626.3	Upwelling, Fernandina Island	Ecuador	Ocean		
Forest Soil, Puerto Rico	mgm4446153.3	subtropical lower montane wet forest in the Luquillo experimental forest	Puerto Rico	Forest	Luquillo Experimental Forest soil	DeAngelis *et al*.^29^
PE6_r1	mgm4477807.3	Manu national park, Peru	USA	Forest	Tropical forest	Fierer *et al*.^30^
AR3_r1	mgm4477875.3	Misiones, Argentina	USA	Forest		
BZ1_r1	mgm4477876.3	Bonanza creek lter, Alaska, USA	USA	Forest	Boreal forest	
CL1_r1	mgm4477877.3	Calhoun experimental forest, south Carolina, USA	USA	Forest	Temperate deciduous forest	
DF1_r1	mgm4477899.3	Duke forest, north Carolina, USA	USA	Forest		
WS-8	mgm4528934.3	Bjornstorp	Sweden	Agriculture	Winter wheat field	Manoharan *et al*.^28^
WS-16	mgm4529786.3	Bjornstorp	Sweden	Agriculture		
WS-24	mgm4529373.3	Bjornstorp	Sweden	Agriculture		
WS-72	mgm4527652.3	Bjornstorp	Sweden	Agriculture		
GL-8	mgm4528937.3	Bjornstorp	Sweden	Grassland	Grassland nearby the wheat field	
GL-16	mgm4529787.3	Bjornstorp	Sweden	Grassland		
GL-24	mgm4529374.3	Bjornstorp	Sweden	Grassland		
GL-72	mgm4527653.3	Bjornstorp	Sweden	Grassland		

### Comparative analysis of resistance to antibiotics and heavy metals in various ecosystems

For resistome analysis, the twelve mangrove datasets as described above and four mangrove datasets from our previous study^18^ were included. In addition, datasets collected from soil samples in four agricultural and adjacent grassland samples from Sweden^28^ were added. Six different forest soil samples from Puerto Rico^29^ and USA^30^ were included, as were sixteen oceanic soil samples from the Global Ocean Sampling Expedition^31,32^ (Table 1).

### Analysis of metagenomic data

Venn diagrams were generated using the Venny 2.0 program^33^. Normality testing using the Shapiro-Wilk test and the Kruskal-Wallis “Nemenyi” tests was performed to evaluate whether the OTU’s and functional genes abundances were different within and between the ecosystems using R^34^ and PAST^35^. The dataset was standardized dividing each OTU abundance value by the sum of all abundances in each sample. A Principal Component Analysis (PCA) was used to compare the microbiota of each site using the vegan package^36^. Multiple linear regressions were conducted with the first two PCs obtained in the PCA analysis and the site as an independent variable. All the analyses were performed using R software^34^.

### Availability of data and materials

The raw fastQ files for the Kerala India samples were uploaded in MG-RAST server and publicly available from the MG-RAST server under the following IDs: mgm4667575.3, mgm4667708.3, mgm4667773.3, and mgm4667861.3. The other publicly available samples used in this study were obtained from the MG-RAST server under the following ids: mgm4671368.3, mgm4671369.3, mgm4671370.3, mgm4671371.3, mgm4451033.3, mgm4451034.3, mgm4451035.3, mgm4451036.3, mgm4523017.3, mgm4523018.3, mgm4523019.3, mgm4523020.3, mgm4441570.3, mgm4441585.3, mgm4441590.3, mgm4441591.3, mgm4441595.3, mgm4441596.4, mgm4441599.3, mgm4441601.3, mgm4441610.3, mgm4441611.3, mgm4441613.3, mgm4441614.3, mgm4441618.3, mgm4441661.3, mgm4441662.3, mgm4442626.3, mgm4446153.3, mgm4477807.3, mgm4477875.3, mgm4477876.3, mgm4477877.3, mgm4477899.3, mgm4528934.3, mgm4529786.3, mgm4529373.3, mgm4527652.3, mgm4528937.3, mgm4529787.3, mgm4529374.3, mgm4527653.3.

## Results

The sequencing from the four India datasets resulted in a total of 9 GB, of which 32,080,253 reads were obtained with an average length ranging from 252 ± 9 bp to 409 ± 139 bp. After quality control pipeline, 13 ± 3.08% reads were assigned to ribosomal RNA genes, 38.56 ± 4.41% to predicted proteins with known functions, and 48.62 ± 6.45% to predicted proteins with unknown function (hypothetical proteins) (Table 2).

**Table 2:** Statistical analysis of the annotation results for all metagenomic samples used from MG-RAST.

Sample ID	Upload: bp Count	Upload: Sequences Count	Upload: Mean Sequence Length	Upload: Mean GC percent	Artificial Duplicate Reads: Sequence Count	Post QC: bp Count	Post QC: Sequences Count	Post QC: Mean Sequence Length	Post QC: Mean GC percent	Processed: Predicted Protein Features	Processed: Predicted rRNA Features	Alignment: Identified Protein Features	Alignment: Identified rRNA Features	Annotation: Identified Functional Categories
MG_KAY	1,063,207,094 bp	2,600,216	409 ± 139 bp	54 ± 9 %	30,782	478,643,511 bp	2,330,736	205 ± 68 bp	56 ± 10 %	1,880,502	219,146	609,089	1,433	484,220
MGVPM	1,206,841,116 bp	2,956,271	408 ± 139 bp	48 ± 9 %	13,768	479,789,043 bp	2,607,133	184 ± 65 bp	51 ± 12 %	2,103,517	258,212	623,629	1,734	485,588
MG_BNH	3,317,193,870 bp	13,142,449	252 ± 9 bp	49 ± 8 %	1,597,912	2,254,033,431 bp	10,931,395	206 ± 56 bp	50 ± 9 %	8,394,321	566,490	3,028,744	3,641	2,396,156
MGKMA	3,416,879,669 bp	13,381,317	255 ± 17 bp	48 ± 10 %	1,219,884	2,423,281,285 bp	11,516,112	210 ± 59 bp	49 ± 11 %	9,956,122	701,720	3,190,526	4,184	2,497,249
PGD	1,987,586,930 bp	7,886,422	252 ± 7 bp	56 ± 10 %	1,108,461	1,148,618,254 bp	5,928,096	194 ± 64 bp	57 ± 11 %	3,750,581	487,269	1,459,319	3,286	1,214,414
MAL	1,845,380,076 bp	7,217,730	256 ± 16 bp	53 ± 11 %	76,558	1,303,758,395 bp	6,457,605	202 ± 68 bp	54 ± 11 %	4,608,323	525,993	1,625,595	3,056	1,271,359
PYN	2,241,809,528 bp	8,803,250	255 ± 14 bp	52 ± 10 %	90,953	1,525,223,003 bp	7,849,891	194 ± 66 bp	53 ± 11 %	5,937,271	623,171	1,843,305	2,986	1,439,895
VL1	2,156,410,938 bp	8,425,778	256 ± 17 bp	54 ± 9 %	251,689	1,474,646,474 bp	7,362,146	200 ± 68 bp	54 ± 10 %	5,545,975	589,227	1,727,829	3,016	1,356,152
BRMgv-1	58,801,025 bp	249,993	235 ± 111 bp	56 ± 10 %	12,048	53,144,117 bp	231,702	229 ± 105 bp	56 ± 10 %	213,268	1	91,085	0	85,285
BRMgv-2	55,077,381 bp	231,233	238 ± 107 bp	55 ± 11 %	12,273	49,736,607 bp	213,348	233 ± 101 bp	54 ± 11 %	198,445	1	79,094	123	73,440
BrMgv-3	53,292,298 bp	214,921	248 ± 112 bp	56 ± 11 %	30,624	43,781,595 bp	179,384	244 ± 108 bp	56 ± 11 %	164,169	22,118	79,074	134	72,636
BrMgv-4	48,522,914 bp	217,605	223 ± 107 bp	55 ± 11 %	16,401	42,070,955 bp	194,797	216 ± 101 bp	54 ± 11 %	175,067	1	70,045	104	65,144
RSMgr01	717,402,333 bp	1,267,409	566 ± 86 bp	51 ± 10 %	76,696	270,309,281 bp	1,089,202	248 ± 100 bp	51 ± 10 %	1,011,211	131,532	256,422	769	202,924
RSMgr02	799,123,615 bp	1,416,928	564 ± 87 bp	52 ± 11 %	102,162	306,144,677 bp	1,211,004	253 ± 100 bp	52 ± 11 %	1,144,572	146,256	350,658	856	272,608
RSMgr03	477,029,111 bp	854,451	558 ± 72 bp	51 ± 12 %	45,503	214,120,294 bp	762,883	281 ± 106 bp	52 ± 11 %	733,494	87,862	243,816	553	188,420
RSMgr04	566,715,841 bp	1,045,353	542 ± 61 bp	52 ± 11 %	94,741	268,607,839 bp	894,444	300 ± 117 bp	52 ± 11 %	863,547	99,227	334,041	866	262,564
Sargasso Station 11 (GS000a)	658,755,696 bp	644,551	1,022 ± 73 bp	52 ± 15 %	0	658,755,696 bp	644,551	1,022 ± 73 bp	52 ± 15 %	509,297	12	416,666	1,839	390,782
North American East Coast (GS013)	149,007,574 bp	138,033	1,080 ± 107 bp	44 ± 11 %	0	149,007,574 bp	138,033	1,080 ± 107 bp	44 ± 11 %	179,606	2	110,197	475	100,559
Panama Canal (GS020)	315,151,139 bp	296,355	1,063 ± 88 bp	47 ± 13 %	0	315,151,139 bp	296,355	1,063 ± 88 bp	47 ± 13 %	340,669	6	197,162	661	184,372
Eastern Tropical Pacific (GS021)	143,454,700 bp	131,798	1,088 ± 70 bp	39 ± 11 %	0	143,454,700 bp	131,798	1,088 ± 70 bp	39 ± 11 %	164,215	3	104,924	356	98,140
Galapagos Islands (GS027)	237,326,008 bp	222,080	1,069 ± 81 bp	37 ± 9 %	0	237,326,008 bp	222,080	1,069 ± 81 bp	37 ± 9 %	279,783	4	202,252	766	189,356
Galapagos Islands (GS028)	205,008,796 bp	189,052	1,084 ± 79 bp	36 ± 8 %	0	205,008,796 bp	189,052	1,084 ± 79 bp	36 ± 8 %	238,061	4	169,294	580	158,043
Hypersaline Lagoon (GS033)	729,708,089 bp	692,255	1,054 ± 96 bp	59 ± 8 %	0	729,708,089 bp	692,255	1,054 ± 96 bp	59 ± 8 %	572,130	13	316,623	1,326	298,336
Wolf Island (GS035)	151,840,270 bp	140,814	1,078 ± 102 bp	36 ± 8 %	0	151,840,270 bp	140,814	1,078 ± 102 bp	36 ± 8 %	173,705	3	130,000	407	122,564
Indian Ocean (GS113)	118,339,154 bp	109,700	1,079 ± 63 bp	35 ± 8 %	0	118,339,154 bp	109,700	1,079 ± 63 bp	35 ± 8 %	144,686	2	103,473	384	96,803
West of the Seychelles (GS114)	345,285,679 bp	348,823	990 ± 73 bp	35 ± 8 %	0	345,285,679 bp	348,823	990 ± 73 bp	35 ± 8 %	426,217	6	287,233	940	265,580
St. Anne Island (GS117a)	339,868,195 bp	346,952	980 ± 71 bp	35 ± 8 %	0	339,868,195 bp	346,952	980 ± 71 bp	35 ± 8 %	429,855	6	285,584	949	266,630
Indian Ocean (GS121)	119,426,081 bp	110,720	1,079 ± 58 bp	35 ± 8 %	0	119,426,081 bp	110,720	1,079 ± 58 bp	35 ± 8 %	144,413	2	106,487	390	100,199
West coast Zanzibar (GS149)	111,178,553 bp	110,984	1,002 ± 62 bp	38 ± 11 %	0	111,178,553 bp	110,984	1,002 ± 62 bp	38 ± 11 %	142,538	2	104,081	419	97,687
Eastern Tropical Pacific (GS023)	143,626,589 bp	133,051	1,079 ± 76 bp	36 ± 9 %	0	143,626,589 bp	133,051	1,079 ± 76 bp	36 ± 9 %	171,111	3	123,834	468	114,444
Warm seep, Roca Redonda (GS030)	391,694,924 bp	359,152	1,091 ± 92 bp	35 ± 7 %	0	391,694,924 bp	359,152	1,091 ± 92 bp	35 ± 7 %	379,822	7	294,116	1,379	278,212
Fernandina Island (GS030)	461,671,889 bp	436,401	1,058 ± 87 bp	34 ± 8 %	0	461,671,889 bp	436,401	1,058 ± 87 bp	34 ± 8 %	520,676	8	386,514	1,407	366,844
Forest Soil, Puerto Rico	322,213,082 bp	782,404	412 ± 103 bp	60 ± 6 %	83,075	279,379,947 bp	642,197	435 ± 74 bp	60 ± 6 %	677,007	39,548	341,249	178	314,106
PE6_r1	920,666,200 bp	9,206,662	100 ± 0 bp	61 ± 8 %	116,635	909,002,500 bp	9,090,025	100 ± 0 bp	61 ± 8 %	8,458,471	2,165,606	4,213,331	3,188	3,664,108
AR3_r1	523,535,200 bp	5,235,352	100 ± 0 bp	62 ± 10 %	58,738	517,661,200 bp	5,176,612	100 ± 0 bp	62 ± 9 %	4,773,078	1,295,718	2,400,106	1,612	2,082,380
BZ1_r1	654,390,300 bp	6,543,903	100 ± 0 bp	58 ± 10 %	113,593	643,030,800 bp	6,430,308	100 ± 0 bp	58 ± 10 %	5,855,737	1,515,096	2,880,748	3,408	2,512,398
CL1_r1	640,294,000 bp	6,402,940	100 ± 0 bp	61 ± 9 %	154,353	624,858,500 bp	6,248,585	100 ± 0 bp	61 ± 9 %	5,776,093	1,516,288	2,985,408	2,845	2,611,586
DF1_r1	389,004,400 bp	3,890,044	100 ± 0 bp	62 ± 9 %	39,901	385,014,100 bp	3,850,141	100 ± 0 bp	62 ± 9 %	3,581,817	958,747	1,893,449	1,743	1,656,393
WS-8	36,416,512 bp	99,966	364 ± 227 bp	64 ± 6 %	5,720	33,518,514 bp	92,955	361 ± 221 bp	64 ± 6 %	82,947	12,831	35,828	28	31,106
WS-16	44,629,285 bp	124,818	358 ± 221 bp	64 ± 6 %	6,924	41,098,149 bp	116,225	354 ± 215 bp	64 ± 6 %	102,141	15,895	68,141	49	57,581
WS-24	48,270,036 bp	138,970	347 ± 219 bp	64 ± 6 %	7,798	44,281,009 bp	129,198	343 ± 213 bp	64 ± 6 %	109,800	17,914	74,327	44	63,362
WS-72	39,046,366 bp	113,014	346 ± 214 bp	64 ± 6 %	6,213	35,885,214 bp	105,190	341 ± 208 bp	64 ± 6 %	89,121	14,670	63,643	42	55,032
GL-8	36,451,314 bp	105,580	345 ± 220 bp	64 ± 6 %	5,824	33,387,051 bp	98,160	340 ± 213 bp	64 ± 6 %	84,918	13,712	42,369	26	34,713
GL-16	39,044,729 bp	113,862	343 ± 211 bp	64 ± 5 %	6,438	35,527,706 bp	105,402	337 ± 203 bp	64 ± 5 %	91,874	14,318	57,268	33	47,623
GL-24	42,416,465 bp	122,694	346 ± 217 bp	64 ± 5 %	7,070	38,835,959 bp	113,806	341 ± 211 bp	64 ± 5 %	95,376	15,530	61,173	33	51,459
GL-72	32,394,105 bp	96,092	337 ± 206 bp	65 ± 5 %	5,175	29,613,637 bp	89,281	332 ± 198 bp	65 ± 5 %	75,136	12,050	50,296	22	42,990

### Analysis at domain level

**Figure 1:**
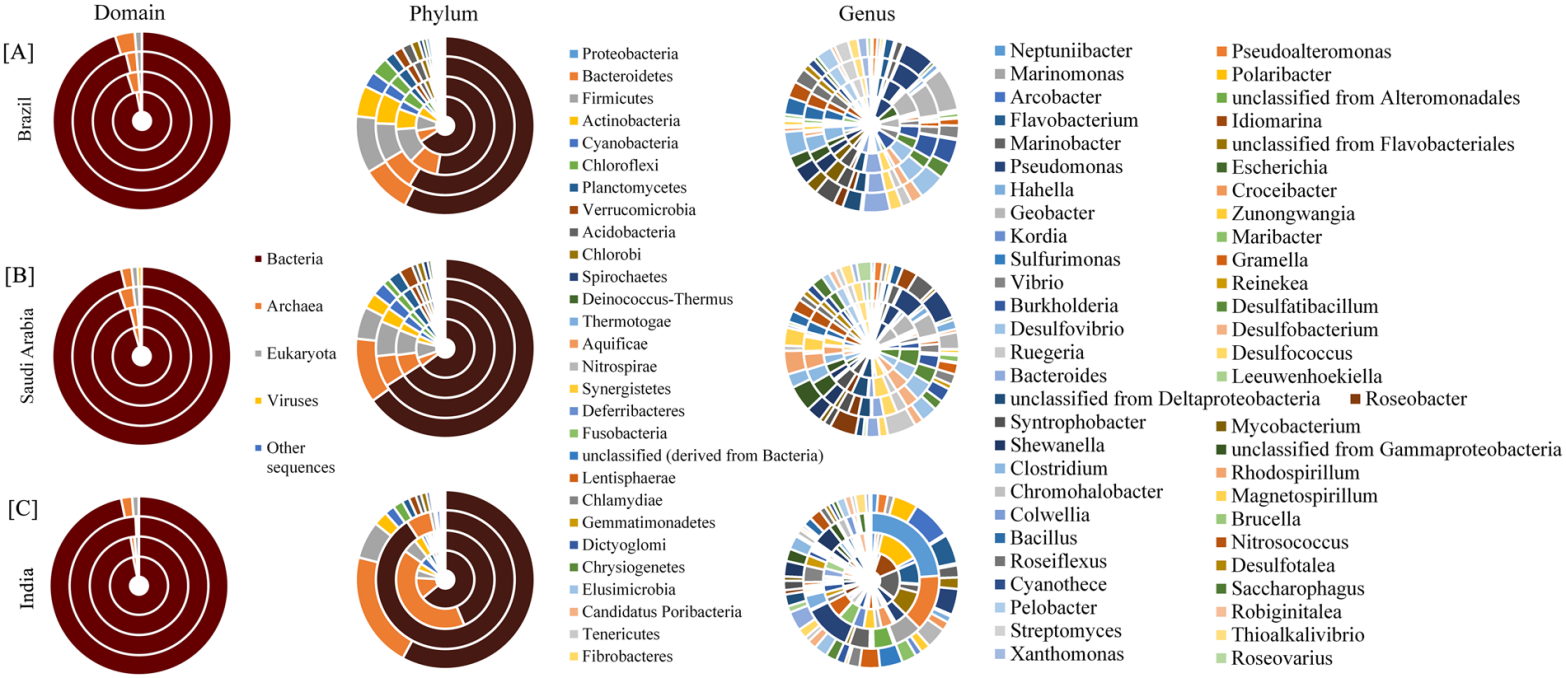
Doughnut chart representing the distribution of domain, phyla and genus of (**A**) Brazil (BRMgv-1, BRMgv-2, BrMgv-3 and BrMgv-4) (**B**) Saudi Arabia (RSMgr01, RSMgr02, RSMgr03 and RSMgr04) and (**C**) India (MG_KAY, MG_VPM, MG_BNH and MG_KMA) (sample labels from inside to outside in doughnut wheels).

All the samples had sequences that map to the Bacteria, Archaea, Eukarya and Viruses. A small percentage of the sequences (0.011 to 0.75%) were not assigned to any organism. Bacteria were the most abundant domain recovered from all the mangrove datasets, ranging from 94.8 to 99.2% of the total. Regardless of the low sequence proportion compared to other domains, the number of sequences affiliated with viruses was the highest in Saudi Arabia samples (Fig. 1B).

**Figure 2:**
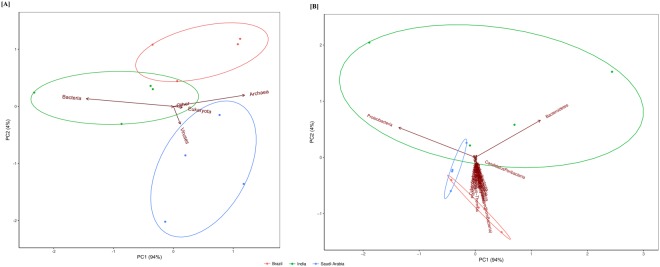
PCA plot of domain and bacterial phyla.

The first two components in the PCA explained more than 98% of variation and there was a clear separation among the samples (Fig. 2A). To determine if the separation among mangrove samples isolated from the different countries were statistically significant, the scores of the first two PC (Principal Component) were used as dependent variables in the multiple linear regression. The clustering effect in the first PC was due to the community abundance at domain level from India. On the other hand, all communities were different when analyzed by the second PC (Table 3). Although the reads mean frequency of Bacteria was not statistically different among the countries, the higher proportion in Indian samples (96.7–99.2%) could explain the separation of this country from the other two (Brazil: 95.1–96.7%; Saudi Arabia: 94.7–96.2%) in the first PC.

**Table 3:** A multiple regression analysis of the first two principal components (PCs) performed at the domain level.

	Variable	Estimates	Std. Error	T value	*P*
PC1					
R^2^ = 53.4%	Saudi Arabia	0.0079	0.00599	1.319	0.2198
STD = 0.0119	Brazil	0.0074	0.00599	1.251	0.2425
*P* = 0.071	India	−0.0154	0.00599	−2.570	**0.0302**
PC2					
R^2^ = 77.2%	Saudi Arabia	−0.0035	0.00085	−4.157	**0.0024**
STD = 0.0017	Brazil	0.0066	0.00121	5.481	**0.0004**
*P* = 0.001	India	0.0040	0.00121	3.337	**0.0087**

### Analysis at phylum level

A total of 66 phyla were recovered from all samples. The richness at phylum level was quite similar across the geographic localities, except for the following Eukarya phyla: Annelida, Brachiopoda, Chytridiomycota, Echiura, Entoprocta, Glomeromycota and Xenoturbellida, which were found exclusively in the Indian samples. The Kruskal-Wallis comparison of the reads abundances among the countries indicated that Brazil (Fig. 1A) had 23 and 11 phyla statistically different from Saudi Arabia and India (Fig. 1C), respectively. Only 5 phyla were statistically different between Saudi Arabia and India. Twenty-eight bacterial phyla were retrieved from all mangroves of the three different countries. The most abundant phylum among the samples was Proteobacteria, which accounted for 50.7 to 64.28% of the sequences in Brazil, 62.6 to 64.2% in Saudi Arabia and 56.7 to 90.5% in India. Firmicutes and Bacteroidetes were the second or the third most frequent bacterial phyla recovered. The PCA using only the frequency of the bacteria phyla showed a clear separation of India from Brazil and Saudi Arabia mainly due to Proteobacteria and Bacteroidetes reads abundance (Fig. 2B).

**Figure 3:**
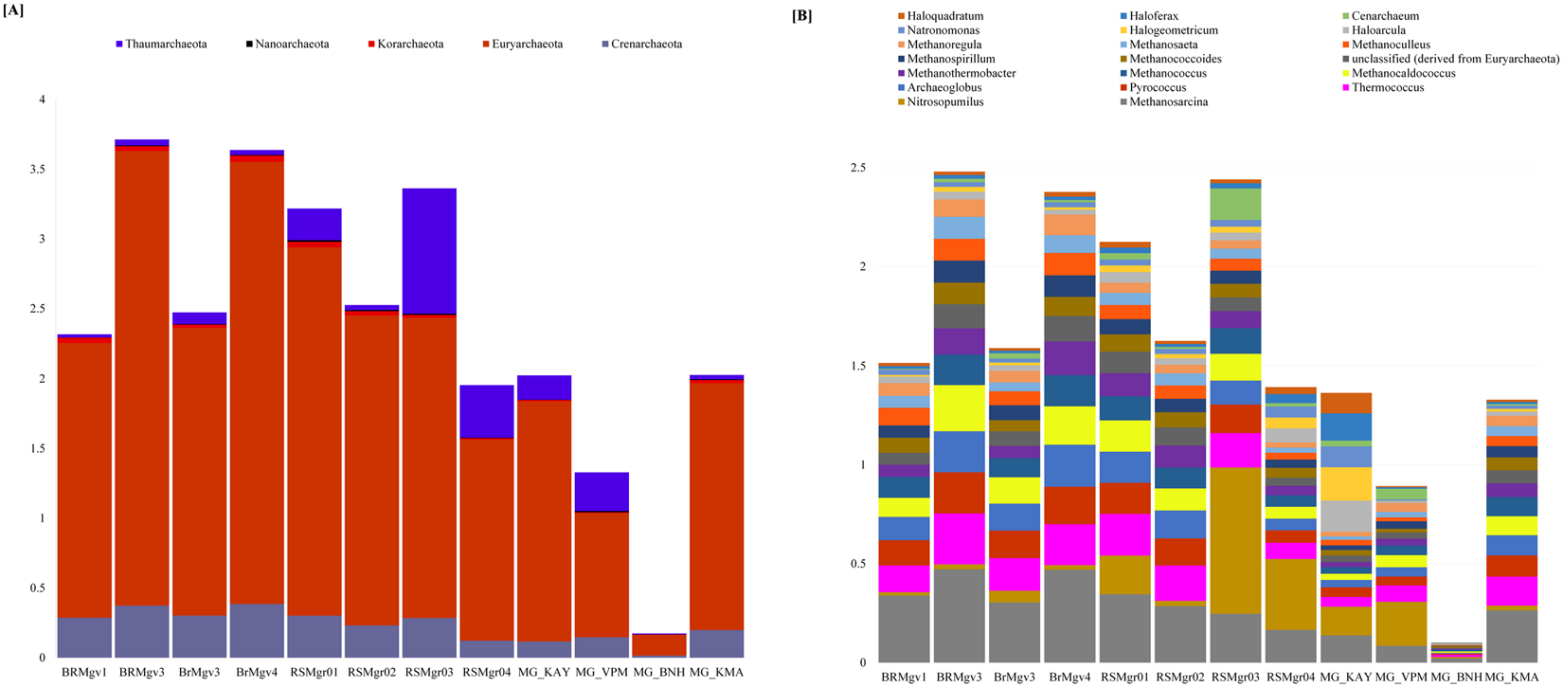
Bar chart of archaeal (**A**) phyla and (**B**) genera.

Five archaeal phyla (Crenarchaeota, Korarchaeota, Thaumarchaeota and Nanoarchaeota) were recovered from all samples in all countries (Fig. 3A). The Crenarchaeota abundance was statistically higher in Brazilian samples than in the other countries (BR-SA: *P* = 0.043; BR- SA: *P* = 0.021) while Euryarchaeota (*P* = 0.021) and Korarchaeota (*P* = 0.043) abundances were statistically lower in India than in Brazil. Euryarchaeota was the most abundant among all archaeal phyla between and within all samples.

### Overview of the dominant bacterial and archaeal genera

**Figure 4:**
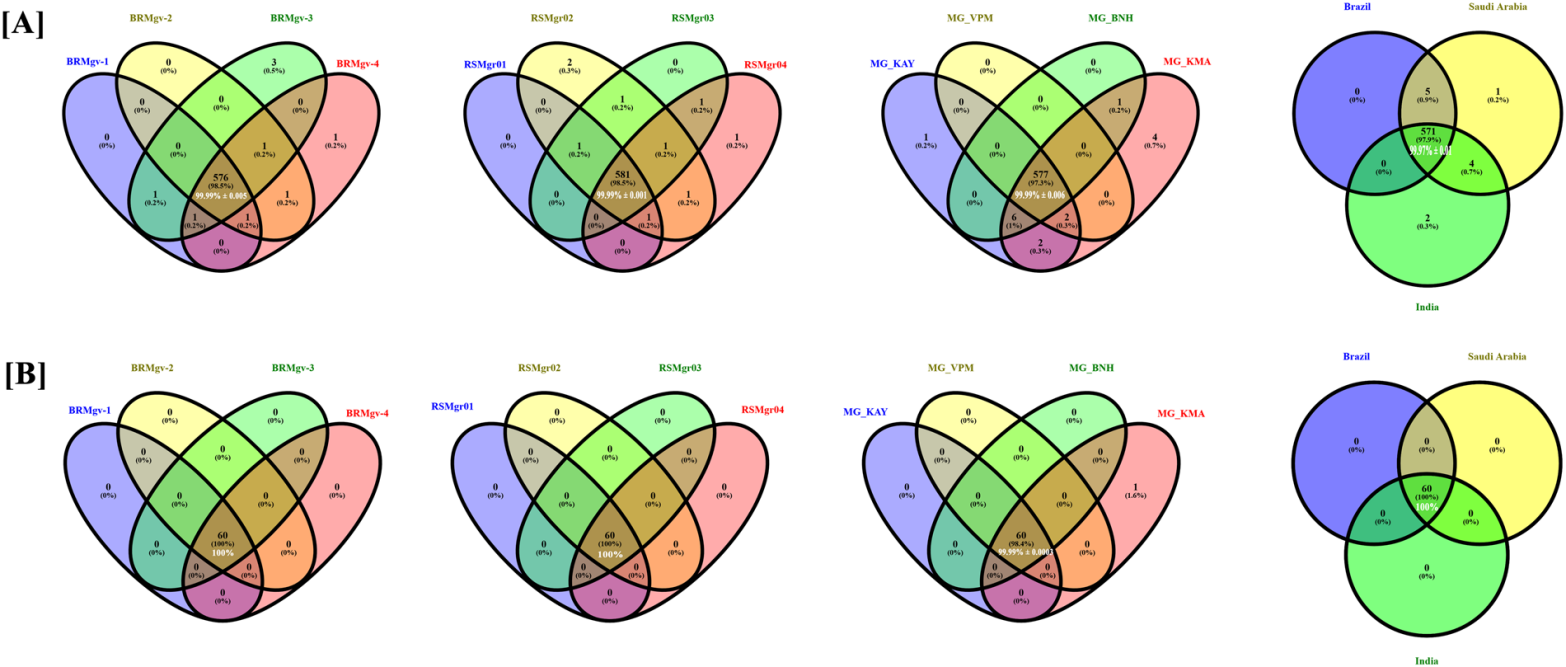
Venn diagram representing the common microbiome diversity within the samples of (**A**) Brazil (**B**) Saudi Arabia (**C**) Kerala and (**D**) among the entire three sample group.

A total of 593 bacterial and 61 archaeal genera were recovered from all the collection sites (Supplementary Data 1). Most of the bacterial genera were present in all samples (Fig. 4), and all archaeal genera were obtained from all of the geographic locations.

To get a clear picture of the dominant bacterial community, only those bacterial genera with more than 1% abundance in at least one sample were selected for further analysis. Fifty-four bacterial genera met this criterion (Fig. 1A–C). Most of these genera belonged to *Proteobacteria* followed by *Bacteroidetes* and *Firmicutes*. Other phyla detected in decreasing order of abundance were *Actinobacteria*, *Chloroflexi* and *Cyanobacteria*. Since Archaea were found less frequently compared to bacteria, dominant archaeal genera were examined when they were 0.1% or more of the total sample population. Twenty archaeal genera (Fig. 3B) met this criterion, with *Methanosarcina*, *Nitrosopumilus*, *Thermococcus*, *Pyrococcus*, *Archaeoglobus* and *Methanocaldococcs* being the most abundant. The Indian sample MG_BNH did not have any archaeal genera with abundance higher than 0.1%.

### Comparative functional analysis of mangrove sediments

**Figure 5:**
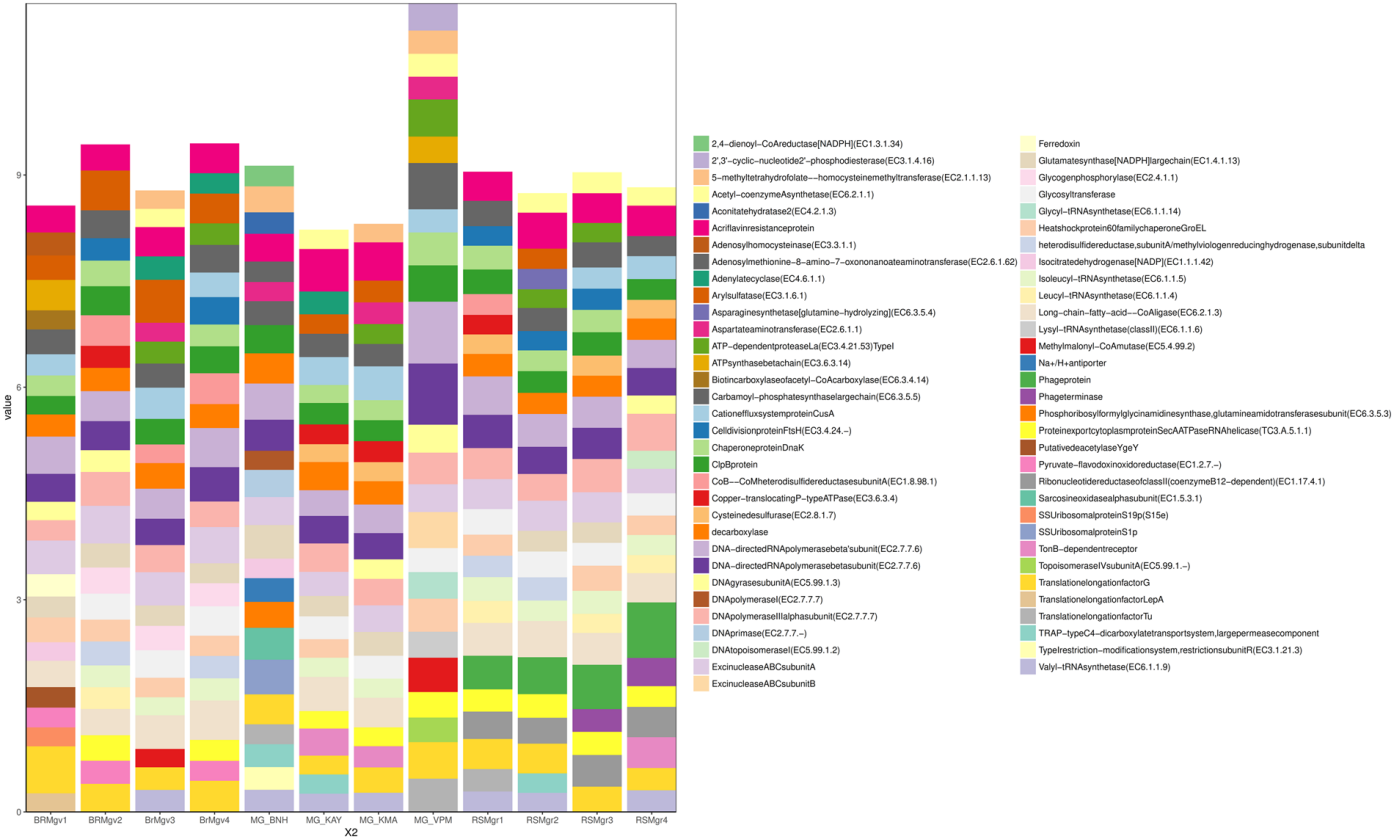
The top 25 most abundant functions from each sample annotated against subsystems.

A total of 7410 protein coding genes were annotated, with 1942 found in all samples and 1023 in only one sample (Supplementary Data 2). The comparison of the top 25 most abundant functional genes from all the samples consisted of 65 genes (Fig. 5). Protein metabolism was the most diversified function with 11 different sub-functions, which includes ATP-dependent protease La (EC 3.4.21.53) (0.282% ± 0.077) and diverse tRNA synthetase, such as valyl (EC 6.1.1.9) (0.266% ± 0.037), glycyl (EC 6.1.1.14) (0.089% ± 0.08), leucyl (EC 6.1.1.4) (0.246% ± 0.049) and lysyl (class II) (EC 6.1.1.6) (0.154% ± 0.068). Within the DNA replication and transcription functional category, DNA primase (EC 2.7.7.-) (0.13% ± 0.08) and DNA-directed RNA polymerase beta subunit (EC 2.7.7.6) (0.471% ± 0.13) were found most frequently. The most common gene in the Cell Division and Cell Cycle functional category was the carbamoyl-phosphate synthase large chain (EC 6.3.5.5) (0.369% ± 0.09). The metabolism of aromatic compounds through Long-chain-fatty-acid-CoA ligase (EC 6.2.1.3) exhibited a similar pattern of low frequency between MG_VPM (0.165%) and the MG_BNH (0.206%) while the remaining samples showed higher level of abundance (0.45% ± 0.05). Kruskal-Wallis comparison of the most abundant functional genes also indicated statistically significant (*p* < 0.05) differences between AS, BR and ID (Supplementary Data 3).

There were also three genes related to antibiotics resistance and toxic compounds: Cation efflux system protein CusA (0.311% ± 0.101), Acriflavine resistance protein (0.428% ± 0.0843) and Topoisomerase IV subunit A (EC 5.99.1.3) (0.136% ± 0.073). Cation efflux system protein CusA is involved in resistance to copper and silver while Acriflavine resistance protein and Topoisomerase IV subunit A are involved in resistance to antiseptic Acriflavine and fluoroquinolones antibiotics respectively, which are both clinically relevant^37–39^.

Overall, the DNA-directed RNA polymerase beta subunit (EC 2.7.7.6) (0.471% ± 0.131) was the most abundant gene followed by Acriflavine resistance protein (0.42% ± 0.08). Within the resistome, Acriflavine resistance protein was the most abundant followed by Cation efflux system protein CusA and Topoisomerase IV subunit A as the 2^nd^ and 3^rd^ most abundant features. All the samples between and within the group of India, Brazil and Saudi Arabia showed high abundance (0.428% ± 0.08) of Acriflavine resistance protein with no significant difference within the samples (*p* 0.4433).

### Comparative functional analysis across different ecosystems

**Figure 6:**
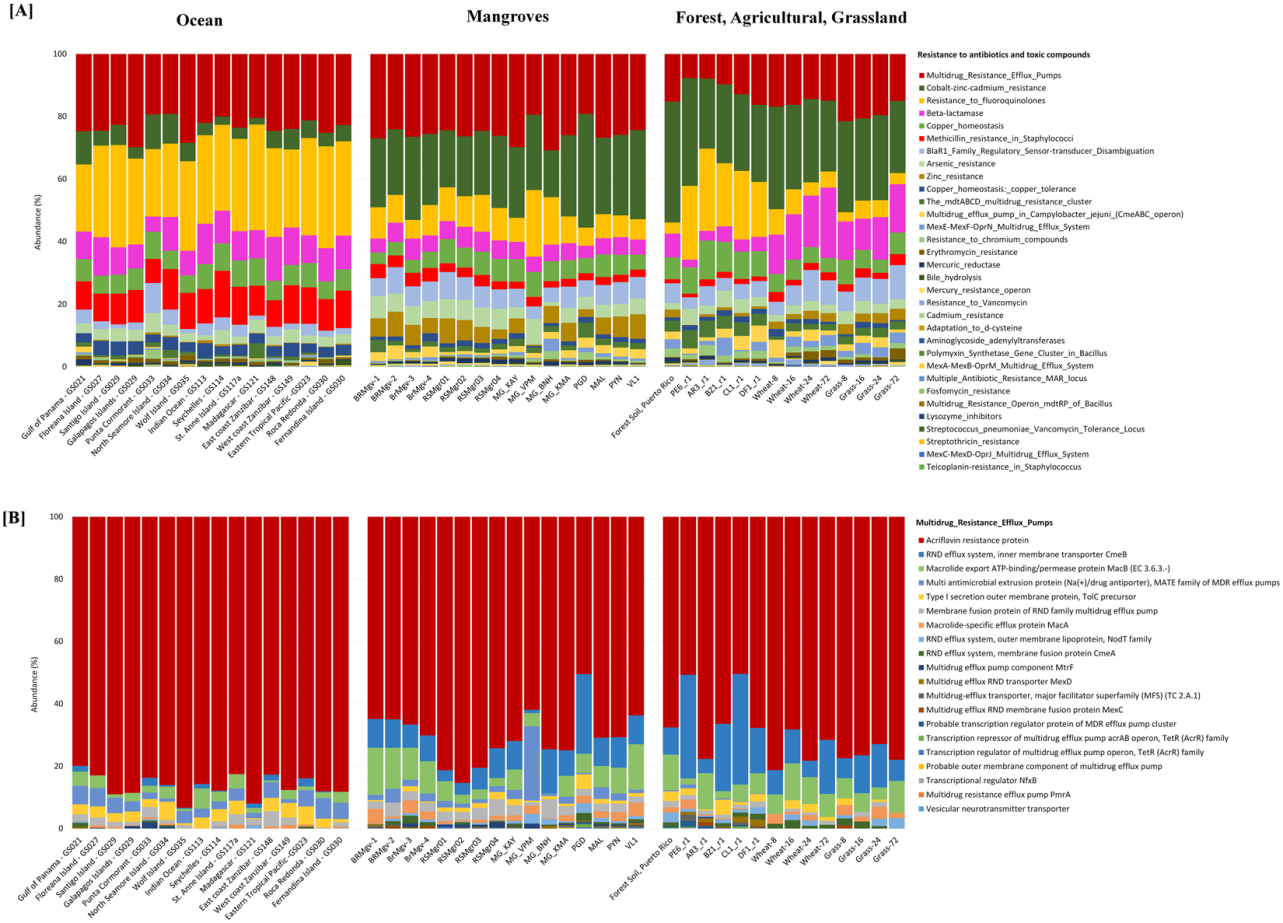
(**A**) Bar chart of resistance to antibiotics and toxic compounds and (**B**) Multidrug Resistance Efflux Pumps in Ocean, Mangroves and Land (Forest, Agriculture and Grassland).

The high abundance of acriflavine resistance protein and the widespread presence of genes related to fluoroquinolone resistance in all the mangroves samples was intriguing and resulted in a deeper examination of the resistance to antibiotics and toxic compounds in the mangroves and other ecosystems. Publicly available metagenomes of marine and terrestrial (forest, grassland and agricultural soil samples) ecosystems were compared to the mangroves sediments (Table 1). Remarkable patterns were observed across the ecosystems showing sharp distinction between the terrestrial and aquatic sites. Enrichment of Multidrug Resistance Efflux Pumps were similar in oceans (23.274% ± 2.931) and mangroves (25.406% ± 2.922) although statistically different (*p* = *0*.*028)*. Interestingly, terrestrial samples had a much lower abundance (14.897% ± 4.116) (*p* < *0*.*005)* of Multidrug Resistance Efflux Pumps (Fig. 6A). However, a deeper look into the functional level of Multidrug Resistance Efflux Pumps shows acriflavine resistance protein to be highly enriched in all the samples irrespective of ecosystem and anthropogenic activities. The relative abundance of the acriflavine resistance was similar in mangroves and terrestrial which were in turn significantly different from Ocean (*p* < *0*.*005)* (Fig. 6B). Similarly, other clinically relevant antibiotic resistance genes (ARGs) such as those related to resistance to fluoroquinolones and beta-lactamase were significantly higher (*p* < 0.005) in ocean (28.178% ± 3.619, 9.913% ± 2.208) compared to mangroves (9.82% ± 3.776, 5.489% ± 0.742) and terrestrial (11.18% ± 8.327, 10.247% ± 5.826). Methicillin resistance was found to be statistically different in all the ecosystems although the relative percentage was more similar between mangrove (3.034% ± 0.808) and terrestrial (2.159% ± 0.682) as compared to Ocean (10.776% ± 1.823) respectively. In addition, resistance genes related to heavy metals such as cobalt, zinc and cadmium were significantly (*p* < 0.015) different in Ocean, Mangroves, and Terrestrial ecosystems (Supplementary data 4).

## Discussion

In this study, whole-metagenome of Indian mangrove samples were sequenced to examine the community structure and functional content using the Illumina technology. These were compared to samples isolated from mangroves in Brazil^17^ and Saudi Arabia^19^, which were sequenced by a different platform (Roche 454). Although the three datasets were generated by two different NGS platforms, our analysis showed that they had similar taxonomic diversity in their microbial communites^40^.

### Bacterial and archaeal diversity in the mangrove sediments

The bacterial phyla, Proteobacteria (61.2% ± 10.82), was the most abundant in the samples examined from each of the geographic locations (Fig. 1A–C). Proteobacteria had previously been noted as being highly abundant in mangrove samples^41^. This phylum has a high metabolic diversity, with a wide distribution in marine environments, playing an important role in nutrient cycling^42^. The second most frequently found phyla within the mangrove metagenomics samples we examined was Archaea. Members belonging to this phylum inhabit extreme environments, playing important roles in the biogeochemical cycles. However, our knowledge as to the niche they occupy, and the role they play in the mangrove microbial community is still limited^43^. Within the archaeal kingdom, Euryarchaeota (81.29% ± 7.993) were found most frequently within and between all the samples when compared to other Archaeal phyla (Fig. 3), and were also found to be highly abundant in mangrove sediments of Sundarbans (India)^43^ and other ecosystems such as marine sediment (North Sea of Atlantic Ocean)^44^ and German bight^45^ (a shallow region of the North Sea that borders Germany). The archaeal community we found in the mangroves had many methanogen genera, including *Methanothermobacter*, *Methanocaldococcus*, *Methanococcus*, *Methanosarcina*, *Methanococcoides*, *Methanospirillum*, *Methanoculleus*, *Methanosaeta*, and *Methanoregula*. The diversity and presence of these specific genera is an indication that methane metabolism is important in mangrove ecosystems. The archaeal ammonia oxidizer *Nitrosopumilus* (0.15% ± 0.2) and *Cenarchaeum* (0.03% ± 0.04) of the phylum Thaumarchaeota was also observed in all samples.

At the bacterial genus level, *Pseudomonas* (2.61% ± 1.7) was found to be most abundant in MG_BNH (8.06%) (India). *Pseudomonas spp*. thrives in many diverse environments that range from individual humans to the rhizosphere^46^. *Pseudomonas* has been found to influence plant growth^19,46,47^ by releasing siderophores, antibiotics, biosurfactants and solubilization of potassium into forms that are accessible for plants, and has also been noted for its critical nitrogen fixation role in the mangrove ecosystem^48,49^. In addition, they are also tolerant to aromatic hydrocarbons, organic and heavy metal contaminants^50,51^. Another aromatic hydrocarbons degrader, *Geobacte*r, was found at a similar frequency in the samples from Brazil (2.65% ± 0.08) and Saudi Arabia (2.05% ± 0.6). Its presence in the Indian samples (1.03% ± 0.53) differed statistically (*p* *0*.*02*) from the samples of the other two countries. *Geobacter* has also been found in petroleum contaminated environments and pristine deep aquifers capable of Fe (III) reduction^52^ and are also strong candidates for immobilization uranium^53^ (U_(VI)_) and bioremediation of aromatic hydrocarbon contaminants. *Neptuniibacter*, a copiotrophic microorganism which can degrade aromatic hydrocarbon such as carbazole^54^, was the 3^rd^ most abundant genus in all the samples (1.61% ± 4.61). The high standard deviation was due to the overwhelmingly high frequency in MG_BNH (16.91%) (Indian sample) that could be due to this region having higher anthropogenic activity when compared to the other samples. In addition, *Marinobacter*, a ubiquitous marine aromatic hydrocarbon degradation genus^55^ of the Proteobacteria phylum, was significantly (*p* 0.0124) abundant in the Indian (3.15% ± 2.23) samples compared to Brazil (0.44% ± 0.04) and Saudi Arabia (0.82% ± 0.35). Our previous study also showed a dominance of *Marinobacter* in mangrove samples^18^. Genome analysis of *Marinobacter* has revealed its potentiality to survive in oil-polluted water^55^ and has suggested that it could be used for bio-monitoring of oil spills in mangroves^56^. Two important genera from the phylum Actinobacteria, Streptomyces and Mycobacterium, were found. *Mycobacterium* were found at more significant frequency (*p* 0.0209) in Brazil (1.17% ± 0.34) than in Saudi Arabia (0.35% ± 0.03) or India (0.35% ± 0.16). Interestingly, a pristine Brazilian sample (BRMgv-1: 1.72%) and one that was highly impacted by human activity (BRMgv-2: 0.98%) had a higher frequency of *Mycobacterium* compared to the samples associated with anthropogenic activity (BRMgv-3: 1.19%), or to the sample from pristine (BRMgv-4: 0.79%) environment. It is interesting that *Mycobacterium spp*., such as *M*. *chlorophenolicum*, *M*. *farcinogenes* and *M*. *austroafricanum*, were observed in samples from mangrove sediments that were contaminated with PAH (Polycyclic aromatic hydrocarbon)^57^. Seven genera belonging to order *Desulfobacterales* were found in all samples and showed similar frequency across all samples. *Desulfotalea* is a psychrophilic genus^58^. *Desulfovibrio* is known to be aerotolerant^59^. *Geobacter* is recognized as an aromatic hydrocarbons degrader^60^ and *Pelobacter* is an iron and sulfur-reducing mesophilic anaerobe^61^. These were significantly less abundant (*p* 0.0209, 0.034, 0.026 and 0.037 respectively) in Indian samples when compared to Brazil and Saudi Arabia. An ammonia oxidizing bacteria, *Nitrosococcus* (0.97% ± 0.28), was present in all the samples with no significant difference within or between the groups. Samples collected from mangroves in each of these countries shared a total of 97.9% of the OTUs, which accounted for 99.97% of the total reads abundance (Fig. 4). Similar results were obtained when compared to forest and vineyard soils^62^. The high number of shared OTUs between the mangroves corroborates the functional genes statistical analysis (Supplementary data 3) between the samples.

### Resistance to antibiotics and heavy metals in various ecosystems

High abundance of fluoroquinolones and acriflavine resistance proteins were found in the mangrove samples of India, Brazil and Saudi Arabia irrespective of the collection site. In order to examine the consistency of these resistance genes in other ecosystems, whole metagenomic datasets from others studies, including^18^, ocean^31,32^, forest^29,30^ agricultural and grassland soil samples^28^ were compared, specifically targeting marine and terrestrial environments with and without anthropogenic activity (Table 1).

### Heavy metal resistome in diverse ecosystems

The presence of genes involved in heavy metal resistance in rivers, activated sludges, aquaculture farm sediments, etc.^63,64^ have been previously described. In our study, genes that play a role in the resistance to antibiotics and toxic compounds were found across all of the ecosystems. Cobalt-zinc-cadmium resistance were found in all the samples, but the percentage of reads that mapped to these genes was found to be significantly lower (*p* < 0.01) in the samples collected from the ocean (5.713% ± 2.589) compared to those collected from mangroves (23.495% ± 4.701) or terrestrial (27.479% ± 4.605) ecosystems (Fig. 6). Among the genes that determine cobalt-zinc-cadmium resistance, the cation efflux system protein *CusA* was the most abundant gene. *CusA* and the cation efflux system provide bacteria with resistance to copper and silver. Although copper is an essential element, it can be lethal to plants even at low concentrations^65^ and can lead to several ill effects such as chlorosis, yellow coloration, and retardation of growth^66^. This copper resistance symbiotic bacterium is associated with plants found in mine tailings^67^. Metal ion solubility generally increases with decreasing pH^68^, and the presence of *CusA* has been found to be associated with soil types with low pH^68^. Marine samples showed significantly lesser enrichment of *CusA* (5.37% ± 3.55), which could be due to the higher pH of marine water^69^ and the lack of plants in this ecosystem. Another annotated function that was seen involved copper homeostasis, but all the ecosystems exhibited comparable level of this particular functionality (7.068% ± 1.154, 6.058% ± 1.343 and 7.238% ± 2.116 for marine, mangroves and terrestrial, respectively). Arsenic resistance genes were also found consistently across all the samples (Fig. 6) having significant difference in ocean vs. mangrove (*p* < 0.001) and terrestrial vs. mangrove (*p* < 0.002) ecosystem (Supplementary data 4). A recent study by Xiao *et al*.^70^ demonstrated a similar presence of genes involved in arsenic metabolism in paddy soil, with the authors concluding that these genes play an important role in avoiding arsenic risk through biotransformation.

### Antibiotic resistance genes (ARGs) patterns across ecosystems

Genes involved in antibiotic resistance have been observed in distinct patterns across different ecosystems^71^. In our study, the Multidrug Resistance Efflux Pumps functional category was the most abundant drug resistance function across all the ecosystems (Fig. 6A). Interestingly, among the subtypes within this functional category, Acriflavine resistance proteins were significantly abundant in every sample (Fig. 6B). Acriflavine has antibacterial properties and is used as an antibiotic^72^. It has been shown to have antiviral and antitumor activities through its topoisomerase inhibition properties^73,74^. The widespread prevalence of acriflavine resistance was also observed in the past from clinical samples in 11 Asian countries^75^. In the recent years, metagenomic analyses showed that acriflavine resistance genes were highly abundant in South China paddy soil^76^ and aerobic activated sludge and anaerobically digested sludge^77^. In our analysis, we found that the acriflavine resistance genes were widespread in aquatic and terrestrial ecosystems that had significant human activity or were from pristine environments (Fig. 6A and B). Fluoroquinolone drugs, which target DNA gyrase and topoisomerase IV, are widely used as the first line for nosocomial infections^78,79^. Fluoroquinolone as well as methicillin resistance gene were found to be significantly higher in marine (28.178% ± 3.619 and 10.776% ± 1.823 respectively, *p* < 0.001) as compared to mangroves (9.82% ± 3.776 and 3.034% ± 0.808, respectively) and terrestrial (11.18% ± 8.327 and 2.159% ± 0.682, respectively) ecosystems.

Beta-lactamase was highly abundant in the marine (9.913% ± 2.208) and terrestrial (10.247% ± 5.826) ecosystems. Within the terrestrial samples, forest (4.38% ± 1.66) had similar abundance comparable to mangroves (5.489% ± 0.742) while the agricultural and grassland samples were found to be highly enriched (14.64% ± 3.54). Antibiotic-resistant genes have been found to be similarly abundant in soils that contain or lack manure^80^. Similarly, our result showed a high abundance of β-lactamases genes in agricultural and adjacent grasslands samples with comparable frequency while the forest and mangroves samples had relatively lesser abundance. The high abundance of β-lactamases in agricultural soil have been demonstrated in a recent functional metagenomic study by Lau *et al*.^81^ who identified 34 new antibiotic resistance genes that were related to multi-drug efflux systems, indicating a potential high-level resistance towards aminoglycosides, sulfonamides, and a broad range of beta-lactams. As β-lactamases have been hypothesized to play a vital role in the survival of the bacteria in its natural habitat^82^, the presence of the genes involved in this resistance have been noted in metagenomic samples from a variety of habitats. For instance, a proficient β-lactamase enzyme was isolated from *Oceanobacillus iheyensis* in the ocean sediments at a depth of 1050 meters^83^. Recently, a novel β-lactamase gene was discovered from *Pelagibacterium halotolerans* B2T, which was isolated from the East China Sea^84^. The high abundance of β-lactamases in oceans also indicates the rich diversity of enzymes and the promising prospects of novel antibiotic discoveries.

Antibiotics and antibiotic resistance genes have been found in diverse environments that include deep terrestrial subsurface, glacier ice core and samples collected from deep sea that have not been in contact with humans^83,85–88^, but they are mostly present at non-inhibitory concentrations^89–91^. The antibiotic resistance genes were dominant in the resistome having significant differences among the ecosystems with the ocean having highest relative abundance compared to mangrove and terrestrial ecosystems (Supplementary data 4). It has been hypothesized that the function of such resistance genes in the natural environment could be related to some basic physiological processes such as biosynthesis of the cell wall^92,93^, trafficking of signaling molecules, detoxification of metabolic intermediates^88^ or antibiotic detoxification^88,94–96^. Untouched environments can have novel antibiotic resistance genes^97^ that can give rise to more multidrug resistant strains via horizontal transfer when human activities encroach upon them. For instance, when soil samples from pre-and post-antibiotic areas were compared, plasmids from the earlier era had fewer antibiotic resistance genes^97,98^ and this was followed by a significant rise in their presence in later sampes^99^. The notion of clinically relevant pathogens acquiring resistance genes from the environment is a likely possibility^97,100,101^.

## Conclusion

We have analysed the metagenomic profiles of mangrove sediments across India and compared them with publicly available samples from Brazil and Saudi Arabia mangrove. Distinct patterns unique to the Brazilian and Saudi Arabian mangroves were observed which differentiated them from samples collected in India. Although there were differences, a significant number of microbial genera were found to be present across all of the three geographic regions. Proteobacteria and Euryarchaeota were the most abundant phyla within and between all the mangroves for bacteria and archaea, respectively. A functional analysis that compared the mangroves samples with metagenomic sample taken from ocean, forest, agriculture and grassland showed the presence of highly enriched acrylflavine, copper, fluoroquinolone, β-lactamase and methilicin resistant genes distributed consistently in patterns throughout all the examined ecosystems. Further, our study showed that heavy metals and antibiotic resistance genes are founnd in microbial populations from mangroves and the other ecosystems, including both pristine areas and environments that experience significant human activity. The widespread existence of antibiotic resistance genes could be a warning bell, indicating a source of new genes that could further increase the rise in antimicrobial resistance that could have clinical significance.

## Electronic supplementary material

Dataset 1, 2, 3, and, 4
