# Supplementary material for: Comparative mangrove metagenome reveals global prevalence of heavy metals and antibiotic resistome across different ecosystems
Source: Sci Rep. 2018 Jul 25;8:11187. doi: 10.1038/s41598-018-29521-4 (PMC6060162; doi:10.1038/s41598-018-29521-4)
Supplement: Supplementary file 1 — Dataset 1, 2, 3, and, 4 [file 41598_2018_29521_MOESM1_ESM.pdf]

Comparative mangrove metagenome reveals global prevalence of heavy metals and antibiotic resistome across different ecosystems

Madangchanok Imchen[1#], Ranjith Kumavath[1#*], Debmalya Barh[2,3], Aline Vaz[4], Aristóteles Góes-Neto[4], Sandeep Tiwari[5], Preetam Ghosh[6], Alice R. Wattam[7], Vasco Azevedo[5]

Supplementary data 1: Overall Archaeal and bacterial genus level diversity in Brazil, Saudi Arabai and India

Domain	Phylum	Class	Order	Family	Genus	BRMgv-1	BRMgv-2	BrMgv-3	BrMgv-4	RSMgr01	RSMgr02	RSMgr03	RSMgr04	MG_KAY	MG_VPM	MG_BNH	MG_KMA
Archaea	Crenarchaeota	Thermoprotei	Acidilobales	Acidilobaceae	Acidilobus	0.005954	0.007529	0.010594	0.012584	0.005404	0.006147	0.004455	0.003478	0.001861	0.009736	0.000379	0.004563
Archaea	Euryarchaeota	unclassified (derived from Euryarchaeota)	unclassified (derived from Euryarchaeota)	unclassified (derived from Euryarchaeota)	Aciduliprofundum	0.037763	0.060234	0.045554	0.057676	0.070001	0.068964	0.05346	0.020052	0.011908	0.026287	0.004312	0.05085
Archaea	Crenarchaeota	Thermoprotei	Desulfurococcales	Desulfurococcaceae	Aeropyrum	0.009866	0.019764	0.021188	0.022022	0.018272	0.015752	0.020493	0.010231	0.010606	0.010709	0.0009	0.011679
Archaea	Euryarchaeota	Archaeoglobi	Archaeoglobales	Archaeoglobaceae	Archaeoglobus	0.118223	0.207053	0.136663	0.212875	0.157501	0.141193	0.121473	0.059543	0.037584	0.046732	0.003744	0.101832
Archaea	Crenarchaeota	Thermoprotei	Thermoproteales	Thermoproteaceae	Caldivirga	0.010036	0.013176	0.009535	0.016778	0.010552	0.015176	0.009801	0.002251	0.004465	0.004868	0.000474	0.008434
Archaea	Korarchaeota	unclassified (derived from Korarchaeota)	unclassified (derived from Korarchaeota)	unclassified (derived from Korarchaeota)	Candidatus Korarchaeum	0.039804	0.034823	0.022247	0.045092	0.039118	0.031696	0.02079	0.011663	0.011257	0.004868	0.002038	0.022831
Archaea	Thaumarchaeota	unclassified (derived from Thaumarchaeota)	Cenarchaeales	Cenarchaeaceae	Cenarchaeum	0.004423	0.017882	0.024366	0.011535	0.031912	0.009797	0.159192	0.017188	0.029212	0.053547	0.000995	0.008417
Archaea	Crenarchaeota	Thermoprotei	Desulfurococcales	Desulfurococcaceae	Desulfurococcus	0.006804	0.013176	0.014832	0.017827	0.012868	0.004995	0.010098	0.004911	0.002512	0.006815	0.000474	0.006688
Archaea	Euryarchaeota	Archaeoglobi	Archaeoglobales	Archaeoglobaceae	Ferroglobus	0.044397	0.071527	0.061445	0.056627	0.068971	0.067811	0.046926	0.019643	0.012187	0.014604	0.00199	0.036783
Archaea	Euryarchaeota	Thermoplasmata	Thermoplasmatales	Ferroplasmaceae	Ferroplasma	0.001021	0.004706	0.005297	0.012584	0.008235	0.006723	0.008019	0.002865	0.002698	0.010709	0.000569	0.006128
Archaea	Euryarchaeota	Halobacteria	Halobacteriales	Halobacteriaceae	Halalkalicoccus	0.005613	0.010353	0.010594	0.01573	0.012868	0.013255	0.015444	0.022303	0.047446	0.003894	0.001422	0.007594
Archaea	Euryarchaeota	Halobacteria	Halobacteriales	Halobacteriaceae	Haloarcula	0.03283	0.038587	0.028604	0.024119	0.053273	0.032465	0.039204	0.07141	0.157873	0.009736	0.001185	0.021793
Archaea	Euryarchaeota	Halobacteria	Halobacteriales	Halobacteriaceae	Halobacterium	0.026536	0.034823	0.021188	0.029362	0.033971	0.019018	0.029106	0.032329	0.077588	0.006815	0.001327	0.012914
Archaea	Euryarchaeota	Halobacteria	Halobacteriales	Halobacteriaceae	Haloferax	0.013098	0.018823	0.013772	0.016778	0.028566	0.014792	0.02673	0.046038	0.137593	0.007789	0.002085	0.010773
Archaea	Euryarchaeota	Halobacteria	Halobacteriales	Halobacteriaceae	Halogeometricum	0.008675	0.02447	0.013772	0.012584	0.033714	0.019978	0.0297	0.054427	0.1682	0.000974	0.000995	0.013705
Archaea	Euryarchaeota	Halobacteria	Halobacteriales	Halobacteriaceae	Halomicrobium	0.019052	0.016	0.01695	0.019924	0.021875	0.012679	0.027027	0.026191	0.057586	0.007789	0.001185	0.009735
Archaea	Euryarchaeota	Halobacteria	Halobacteriales	Halobacteriaceae	Haloquadratum	0.01633	0.016941	0.012713	0.024119	0.028566	0.016136	0.019008	0.035194	0.103357	0.005841	0.000711	0.010855
Archaea	Euryarchaeota	Halobacteria	Halobacteriales	Halobacteriaceae	Halorhabdus	0.006124	0.025411	0.013772	0.019924	0.02625	0.014792	0.021384	0.028237	0.042701	0.004868	0.001185	0.01214
Archaea	Euryarchaeota	Halobacteria	Halobacteriales	Halobacteriaceae	Halorubrum	0.009526	0.013176	0.011653	0.011535	0.01853	0.009989	0.016335	0.027009	0.055074	0.000974	0.001043	0.007594
Archaea	Euryarchaeota	Halobacteria	Halobacteriales	Halobacteriaceae	Haloterrigena	0.019222	0.030117	0.028604	0.034605	0.027022	0.024589	0.032076	0.031101	0.0614	0.009736	0.005781	0.014825
Archaea	Crenarchaeota	Thermoprotei	Desulfurococcales	Pyrodictiaceae	Hyperthermus	0.020242	0.016941	0.01801	0.034605	0.019816	0.018057	0.023166	0.00798	0.006326	0.009736	0.001232	0.014282
Archaea	Crenarchaeota	Thermoprotei	Desulfurococcales	Desulfurococcaceae	Ignicoccus	0.021263	0.025411	0.011653	0.016778	0.022133	0.013447	0.015444	0.011458	0.006047	0.002921	0.000758	0.010559
Archaea	Crenarchaeota	Thermoprotei	Desulfurococcales	Desulfurococcaceae	Ignisphaera	0.006634	0.00847	0.003178	0.010486	0.006691	0.004802	0.005049	0.004706	0.001954	0.003894	0.000758	0.006391
Archaea	Crenarchaeota	Thermoprotei	Sulfolobales	Sulfolobaceae	Metallosphaera	0.018711	0.021646	0.014832	0.016778	0.013897	0.011526	0.013068	0.005525	0.005861	0.010709	0.000569	0.010065
Archaea	Euryarchaeota	Methanobacteria	Methanobacteriales	Methanobacteriaceae	Methanobrevibacter	0.03317	0.058351	0.03496	0.052432	0.042206	0.044375	0.047817	0.020666	0.010792	0.009736	0.002749	0.033505
Archaea	Euryarchaeota	Methanococci	Methanococcales	Methanocaldococcaceae	Methanocaldococcus	0.096789	0.233405	0.133484	0.194	0.157501	0.111802	0.133353	0.060156	0.031072	0.061335	0.007108	0.095721
Archaea	Euryarchaeota	Methanomicrobia	Methanocellales	Methanocellaceae	Methanocella	0.027557	0.061175	0.041317	0.062919	0.047868	0.041685	0.024354	0.016369	0.018141	0.031154	0.003459	0.034707
Archaea	Euryarchaeota	Methanomicrobia	Methanosarcinales	Methanosarcinaceae	Methanococcoides	0.076887	0.109173	0.056148	0.096475	0.087243	0.076455	0.069201	0.05013	0.02856	0.018498	0.008009	0.065725
Archaea	Euryarchaeota	Methanococci	Methanococcales	Methanococcaceae	Methanococcus	0.105635	0.153407	0.096405	0.157297	0.122244	0.105654	0.129195	0.057496	0.033398	0.048679	0.009715	0.097401
Archaea	Euryarchaeota	Methanomicrobia	Methanomicrobiales	Methanocorpusculaceae	Methanocorpusculum	0.023134	0.035764	0.021188	0.034605	0.017243	0.021707	0.024651	0.010435	0.006326	0.005841	0.001327	0.018548
Archaea	Euryarchaeota	Methanomicrobia	Methanomicrobiales	Methanomicrobiaceae	Methanoculleus	0.089135	0.110115	0.07098	0.113254	0.071545	0.067427	0.060885	0.035194	0.027537	0.020445	0.002512	0.050702
Archaea	Euryarchaeota	Methanomicrobia	Methanosarcinales	Methanosarcinaceae	Methanohalobium	0.015139	0.046116	0.019069	0.034605	0.037831	0.03573	0.02673	0.020871	0.008838	0.015577	0.00417	0.02321
Archaea	Euryarchaeota	Methanomicrobia	Methanosarcinales	Methanosarcinaceae	Methanohalophilus	0.027387	0.055528	0.038138	0.047189	0.051471	0.043991	0.042768	0.027009	0.012001	0.019472	0.008151	0.03166
Archaea	Euryarchaeota	Methanomicrobia	Methanomicrobiales	Methanomicrobiaceae	Methanoplanus	0.013778	0.049881	0.030723	0.05453	0.030111	0.025357	0.022869	0.015551	0.010233	0.01363	0.004597	0.022337
Archaea	Euryarchaeota	Methanopyri	Methanopyrales	Methanopyraceae	Methanopyrus	0.041335	0.05741	0.031782	0.072357	0.040405	0.036499	0.036234	0.017597	0.008745	0.012657	0.000995	0.025499
Archaea	Euryarchaeota	Methanomicrobia	Methanomicrobiales	unclassified (derived from Methanomicrobiales)	Methanoregula	0.06498	0.088468	0.058267	0.104865	0.051728	0.043222	0.041283	0.025577	0.023444	0.044785	0.003459	0.051427
Archaea	Euryarchaeota	Methanomicrobia	Methanosarcinales	Methanosaetaceae	Methanosaeta	0.059707	0.111056	0.044495	0.089135	0.060993	0.061087	0.051381	0.025986	0.017769	0.02726	0.001848	0.050982
Archaea	Euryarchaeota	Methanomicrobia	Methanosarcinales	Methanosarcinaceae	Methanosarcina	0.338848	0.471516	0.304048	0.469794	0.345628	0.285843	0.247104	0.165737	0.137593	0.084701	0.021799	0.264909
Archaea	Euryarchaeota	Methanobacteria	Methanobacteriales	Methanobacteriaceae	Methanosphaera	0.013268	0.035764	0.015891	0.027265	0.022905	0.02017	0.019305	0.011049	0.006977	0.003894	0.001232	0.016654
Archaea	Euryarchaeota	Methanomicrobia	Methanomicrobiales	unclassified (derived from Methanomicrobiales)	Methanosphaerula	0.03215	0.058351	0.038138	0.068162	0.050184	0.038804	0.024354	0.024349	0.017676	0.031154	0.003507	0.039386
Archaea	Euryarchaeota	Methanomicrobia	Methanomicrobiales	Methanospirillaceae	Methanospirillum	0.062088	0.111056	0.075217	0.109059	0.077464	0.068003	0.065637	0.041127	0.023444	0.03797	0.002369	0.056846
Archaea	Euryarchaeota	Methanobacteria	Methanobacteriales	Methanobacteriaceae	Methanothermobacter	0.061748	0.13082	0.061445	0.167783	0.116325	0.111033	0.086427	0.049107	0.025676	0.035049	0.004549	0.06981
Archaea	Euryarchaeota	Methanococci	Methanococcales	Methanococcaceae	Methanothermococcus	0.010036	0.009411	0.007416	0.012584	0.012353	0.010758	0.012177	0.004092	0.002419	0.005841	0.001043	0.0085
Archaea	Euryarchaeota	Methanobacteria	Methanobacteriales	Methanothermaceae	Methanothermus	0.009016	0.021646	0.012713	0.022022	0.030368	0.019402	0.01485	0.006957	0.005489	0.010709	0.002843	0.016374
Archaea	Nanoarchaeota	unclassified (derived from Nanoarchaeota)	unclassified (derived from Nanoarchaeota)	unclassified (derived from Nanoarchaeota)	Nanoarchaeum	0.001531	0.005647	0.004238	0.005243	0.012353	0.008644	0.008019	0.004297	0.001582	0.009736	0.000284	0.005617
Archaea	Euryarchaeota	Halobacteria	Halobacteriales	Halobacteriaceae	Natrialba	0.01548	0.012235	0.015891	0.018876	0.019816	0.013447	0.018711	0.020666	0.039166	0.004868	0.000711	0.008516
Archaea	Euryarchaeota	Halobacteria	Halobacteriales	Halobacteriaceae	Natronomonas	0.026536	0.023529	0.020129	0.025168	0.030111	0.025741	0.034452	0.055655	0.105776	0.007789	0.001327	0.015814
Archaea	Thaumarchaeota	unclassified (derived from Thaumarchaeota)	Nitrosopumilales	Nitrosopumilaceae	Nitrosopumilus	0.017691	0.025411	0.059326	0.02307	0.19559	0.026125	0.739826	0.358688	0.145128	0.222949	0.006397	0.023243
Archaea	Euryarchaeota	Thermoplasmata	Thermoplasmatales	Picrophilaceae	Picrophilus	0.013268	0.014117	0.009535	0.018876	0.014412	0.013255	0.012474	0.005525	0.004838	0.008762	0.00109	0.010411
Archaea	Crenarchaeota	Thermoprotei	Thermoproteales	Thermoproteaceae	Pyrobaculum	0.047799	0.067763	0.045554	0.067113	0.044008	0.030928	0.043956	0.018824	0.022327	0.017524	0.002464	0.031199
Archaea	Euryarchaeota	Thermococci	Thermococcales	Thermococcaceae	Pyrococcus	0.127748	0.208935	0.138781	0.189805	0.157501	0.137351	0.144045	0.062612	0.048376	0.044785	0.006018	0.107664
Archaea	Crenarchaeota	Thermoprotei	Desulfurococcales	Desulfurococcaceae	Staphylothermus	0.018031	0.031999	0.026485	0.035654	0.036802	0.021707	0.027918	0.010026	0.006605	0.014604	0.001564	0.019454
Archaea	Crenarchaeota	Thermoprotei	Sulfolobales	Sulfolobaceae	Sulfolobus	0.06549	0.070586	0.06992	0.070259	0.054045	0.044759	0.062964	0.027009	0.0267	0.032128	0.00308	0.037738
Archaea	Euryarchaeota	Thermococci	Thermococcales	Thermococcaceae	Thermococcus	0.134212	0.256934	0.165266	0.206583	0.210517	0.178652	0.173448	0.081641	0.049771	0.082754	0.01161	0.146374
Archaea	Crenarchaeota	Thermoprotei	Thermoproteales	Thermofilaceae	Thermofilum	0.034531	0.050822	0.029663	0.036703	0.036802	0.02651	0.031779	0.010231	0.014048	0.011683	0.001232	0.022633
Archaea	Euryarchaeota	Thermoplasmata	Thermoplasmatales	Thermoplasmataceae	Thermoplasma	0.028578	0.037646	0.023307	0.040897	0.035	0.026894	0.031185	0.012481	0.011071	0.015577	0.001848	0.024429
Archaea	Crenarchaeota	Thermoprotei	Thermoproteales	Thermoproteaceae	Thermoproteus	0.009526	0.004706	0.004238	0.008389	0.003088	0.00365	0.003267	0.001842	0.001582	0.001947	0.000237	0.002899
Archaea	Crenarchaeota	Thermoprotei	Desulfurococcales	Desulfurococcaceae	Thermosphaera	0.006804	0.00847	0.011653	0.010486	0.009265	0.006339	0.005643	0.000818	0.002419	0.001947	0.000332	0.006358
Archaea	Crenarchaeota	Thermoprotei	Thermoproteales	Thermoproteaceae	Vulcanisaeta	0.005273	0.013176	0.011653	0.008389	0.008493	0.007684	0.007425	0.003274	0.003442	0.007789	0.000474	0.005419
Archaea	Euryarchaeota	unclassified (derived from Euryarchaeota)	unclassified (derived from Euryarchaeota)	unclassified (derived from Euryarchaeota)	unclassified (derived from Euryarchaeota)	0.059537	0.122349	0.074158	0.128984	0.106802	0.091823	0.069201	0.040104	0.032375	0.031154	0.004312	0.064967
Archaea	Crenarchaeota	Thermoprotei	Sulfolobales	Sulfolobaceae	Acidianus	0	0	0	0	0	0	0	0	0	0	0	1.65E-05
Bacteria	Firmicutes	Bacilli	Lactobacillales	Aerococcaceae	Abiotrophia	0.008165	0.020705	0.008475	0.025168	0.010037	0.007108	0.012771	0.004501	0.005861	0.005841	0.000853	0.008566

Bacteria	Cyanobacteria	unclassified (derived from Cyanobacteria)	unclassified (derived from Cyanobacteria)	unclassified (derived from Cyanobacteria)	Acaryochloris	0.067872	0.101644	0.098524	0.099621	0.144891	0.128322	0.118503	0.116834	0.176945	0.052573	0.030471	0.078442
Bacteria	Firmicutes	Clostridia	Clostridiales	Ruminococcaceae	Acetivibrio	0.017691	0.056469	0.040257	0.056627	0.05842	0.053211	0.041283	0.0266	0.017397	0.05452	0.008009	0.046024
Bacteria	Proteobacteria	Alphaproteobacteria	Rhodospirillales	Acetobacteraceae	Acetobacter	0.09764	0.023529	0.026485	0.026216	0.028824	0.029583	0.042174	0.046038	0.032561	0.026287	0.002559	0.012255
Bacteria	Firmicutes	Clostridia	Halanaerobiales	Halobacteroidaceae	Acetohalobium	0.036913	0.079998	0.060386	0.073405	0.090074	0.083947	0.070092	0.049516	0.035259	0.024339	0.005781	0.052827
Bacteria	Tenericutes	Mollicutes	Acholeplasmatales	Acholeplasmataceae	Acholeplasma	0.007485	0.013176	0.006356	0.010486	0.005662	0.014215	0.019602	0.019029	0.004745	0.001947	0.002512	0.00789
Bacteria	Proteobacteria	Betaproteobacteria	Burkholderiales	Alcaligenaceae	Achromobacter	0.104444	0.129879	0.173742	0.126886	0.104744	0.104694	0.126522	0.09801	0.157036	0.11975	0.076201	0.078705
Bacteria	Firmicutes	Negativicutes	Selenomonadales	Acidaminococcaceae	Acidaminococcus	0.026026	0.051763	0.043435	0.035654	0.030368	0.035346	0.03861	0.020666	0.019816	0.020445	0.005118	0.028431
Bacteria	Actinobacteria	Actinobacteria (class)	Acidimicrobiales	Acidimicrobiaceae	Acidimicrobium	0.026706	0.029176	0.040257	0.030411	0.031397	0.016328	0.021384	0.017188	0.037212	0.025313	0.002749	0.018037
Bacteria	Proteobacteria	Alphaproteobacteria	Rhodospirillales	Acetobacteraceae	Acidiphilium	0.158707	0.143996	0.14196	0.131081	0.147465	0.168663	0.211761	0.205227	0.197783	0.105146	0.015212	0.072001
Bacteria	Proteobacteria	Gammaproteobacteria	Acidithiobacillales	Acidithiobacillaceae	Acidithiobacillus	0.301595	0.182583	0.204464	0.18561	0.118898	0.098163	0.112563	0.077753	0.107544	0.086648	0.045588	0.12351
Bacteria	Acidobacteria	Acidobacteria (class)	Acidobacteriales	Acidobacteriaceae	Acidobacterium	0.229981	0.253169	0.292395	0.238043	0.159303	0.146764	0.14553	0.148345	0.235647	0.294994	0.022794	0.140081
Bacteria	Actinobacteria	Actinobacteria (class)	Actinomycetales	Acidothermaceae	Acidothermus	0.087774	0.121408	0.116534	0.095427	0.068714	0.046872	0.056133	0.033147	0.062238	0.016551	0.008151	0.037211
Bacteria	Proteobacteria	Betaproteobacteria	Burkholderiales	Comamonadaceae	Acidovorax	0.284245	0.338814	0.391978	0.397437	0.210002	0.238971	0.272942	0.22753	0.254718	0.235606	0.15207	0.187884
Bacteria	Proteobacteria	Gammaproteobacteria	Pseudomonadales	Moraxellaceae	Acinetobacter	0.366235	0.217406	0.30087	0.247481	0.19559	0.19767	0.296702	0.258018	0.199086	0.251183	0.600412	0.274859
Bacteria	Proteobacteria	Gammaproteobacteria	Pasteurellales	Pasteurellaceae	Actinobacillus	0.129109	0.07341	0.094287	0.070259	0.057905	0.068387	0.119097	0.079799	0.055446	0.073018	0.099516	0.081868
Bacteria	Actinobacteria	Actinobacteria (class)	Actinomycetales	Actinomycetaceae	Actinomyces	0.030789	0.059292	0.047673	0.04614	0.022905	0.021131	0.03564	0.016983	0.025211	0.020445	0.003886	0.019602
Bacteria	Actinobacteria	Actinobacteria (class)	Actinomycetales	Actinosynnemataceae	Actinosynnema	0.043036	0.054587	0.062505	0.044043	0.033971	0.02382	0.038907	0.033966	0.036933	0.039917	0.004455	0.024939
Bacteria	Firmicutes	Bacilli	Lactobacillales	Aerococcaceae	Aerococcus	0.002722	0.001882	0.005297	0.003146	0.003603	0.003458	0.001782	0.004501	0.003256	0.006815	0.001137	0.003739
Bacteria	Actinobacteria	Actinobacteria (class)	Actinomycetales	Nocardioidaceae	Aeromicrobium	0.009356	0.022588	0.019069	0.019924	0.01261	0.012679	0.011286	0.011663	0.010512	0.011683	0.00308	0.006885
Bacteria	Proteobacteria	Gammaproteobacteria	Aeromonadales	Aeromonadaceae	Aeromonas	0.240358	0.141172	0.17692	0.191902	0.143347	0.154255	0.174933	0.167374	0.171642	0.236579	0.448011	0.215657
Bacteria	Proteobacteria	Alphaproteobacteria	Rhizobiales	Bradyrhizobiaceae	Afipia	0.029258	0.016941	0.048732	0.031459	0.03603	0.030544	0.046035	0.043992	0.050144	0.0516	0.004075	0.021579
Bacteria	Proteobacteria	Gammaproteobacteria	Pasteurellales	Pasteurellaceae	Aggregatibacter	0.024665	0.029176	0.025426	0.025168	0.016213	0.017865	0.024948	0.022712	0.019443	0.033102	0.040991	0.025961
Bacteria	Proteobacteria	Alphaproteobacteria	Rhizobiales	Rhizobiaceae	Agrobacterium	0.241718	0.200465	0.254256	0.233848	0.272797	0.227061	0.271457	0.290961	0.307746	0.185953	0.126433	0.141086
Bacteria	Proteobacteria	Alphaproteobacteria	Rhodobacterales	Rhodobacteraceae	Ahrensia	0.021773	0.030117	0.029663	0.028313	0.067942	0.061472	0.073359	0.081641	0.070331	0.033102	0.008246	0.019092
Bacteria	Verrucomicrobia	Verrucomicrobiae	Verrucomicrobiales	Verrucomicrobiaceae	Akkermansia	0.046949	0.069645	0.061445	0.057676	0.045037	0.053019	0.041877	0.076935	0.058237	0.04089	0.010899	0.046567
Bacteria	Proteobacteria	Betaproteobacteria	Burkholderiales	Comamonadaceae	Albidiferax	0.246822	0.209876	0.262731	0.261113	0.147722	0.141385	0.135432	0.102921	0.15992	0.082754	0.090986	0.149273
Bacteria	Proteobacteria	Gammaproteobacteria	Oceanospirillales	Alcanivoracaceae	Alcanivorax	0.264342	0.197641	0.248959	0.235946	0.293128	0.337134	0.382535	0.54018	0.497343	0.208346	0.795321	0.367499
Bacteria	Bacteroidetes	Cytophagia	Cytophagales	Cyclobacteriaceae	Algoriphagus	0.106826	0.15529	0.143019	0.13947	0.131251	0.192675	0.162162	0.330451	0.321607	0.351461	0.320631	0.218194
Bacteria	Proteobacteria	Betaproteobacteria	Burkholderiales	Comamonadaceae	Alicycliphilus	0.034021	0.049881	0.055089	0.070259	0.036802	0.032657	0.040392	0.038467	0.046515	0.042837	0.021893	0.027937
Bacteria	Firmicutes	Bacilli	Bacillales	Alicyclobacillaceae	Alicyclobacillus	0.062769	0.083762	0.067802	0.08494	0.061765	0.057245	0.058806	0.056883	0.050423	0.055494	0.008814	0.04833
Bacteria	Proteobacteria	Gammaproteobacteria	Vibrionales	Vibrionaceae	Aliivibrio	0.127068	0.08188	0.099584	0.094378	0.082611	0.092976	0.102762	0.09801	0.10038	0.152852	0.421473	0.150492
Bacteria	Bacteroidetes	Bacteroidia	Bacteroidales	Rikenellaceae	Alistipes	0.020923	0.046116	0.03496	0.035654	0.031912	0.032465	0.040689	0.048084	0.022886	0.035049	0.011705	0.075411
Bacteria	Proteobacteria	Gammaproteobacteria	Chromatiales	Ectothiorhodospiraceae	Alkalilimnicola	0.453499	0.287992	0.358077	0.360734	0.495666	0.482745	0.427976	0.441965	0.338911	0.149931	0.235189	0.36918
Bacteria	Firmicutes	Clostridia	Clostridiales	Clostridiaceae	Alkaliphilus	0.181672	0.24564	0.208702	0.279989	0.238826	0.250113	0.203445	0.146299	0.106613	0.124618	0.023599	0.232459
Bacteria	Proteobacteria	Gammaproteobacteria	Chromatiales	Chromatiaceae	Allochromatium	0.363683	0.606101	0.431176	0.37961	0.798316	0.647374	0.409562	0.312445	0.20811	0.237553	0.281583	0.691512
Bacteria	Proteobacteria	Gammaproteobacteria	Alteromonadales	Alteromonadaceae	Alteromonas	0.102913	0.071527	0.098524	0.12374	0.136398	0.148685	0.172557	0.258018	0.228856	0.258972	0.613966	0.165712
Bacteria	Synergistetes	Synergistia	Synergistales	Synergistaceae	Aminobacterium	0.039975	0.058351	0.031782	0.065016	0.051728	0.053596	0.032076	0.027214	0.019536	0.025313	0.00327	0.037672
Bacteria	Synergistetes	Synergistia	Synergistales	Synergistaceae	Aminomonas	0.014629	0.04894	0.030723	0.026216	0.030111	0.027854	0.025839	0.017188	0.013396	0.016551	0.006587	0.022139
Bacteria	Firmicutes	Clostridia	Thermoanaerobacterales	Thermoanaerobacteraceae	Ammonifex	0.085052	0.137408	0.085811	0.122692	0.099854	0.082026	0.056133	0.044606	0.038329	0.036022	0.005165	0.063616
Bacteria	Actinobacteria	Actinobacteria (class)	Actinomycetales	Pseudonocardiaceae	Amycolatopsis	0.043036	0.060234	0.077336	0.04614	0.040405	0.032465	0.043065	0.033147	0.053586	0.028234	0.004691	0.031182
Bacteria	Cyanobacteria	unclassified (derived from Cyanobacteria)	Nostocales	Nostocaceae	Anabaena	0.142888	0.208935	0.206583	0.197146	0.208458	0.179036	0.182952	0.162668	0.350912	0.073018	0.053881	0.119079
Bacteria	Synergistetes	Synergistia	Synergistales	Synergistaceae	Anaerobaculum	0.036743	0.080939	0.038138	0.051384	0.069486	0.075303	0.054351	0.028851	0.025304	0.033102	0.003365	0.043009
Bacteria	Firmicutes	Clostridia	Clostridiales	Clostridiales Family XI. Incertae Sedis	Anaerococcus	0.01599	0.036705	0.025426	0.029362	0.028052	0.026702	0.034452	0.024963	0.014141	0.016551	0.003033	0.024725
Bacteria	Firmicutes	Clostridia	Clostridiales	Eubacteriaceae	Anaerofustis	0.004763	0.009411	0.005297	0.007341	0.01261	0.011334	0.010989	0.010435	0.006419	0.006815	0.001801	0.010081
Bacteria	Proteobacteria	Deltaproteobacteria	Myxococcales	Myxococcaceae	Anaeromyxobacter	0.82977	0.92515	0.987361	0.887155	0.665778	0.611451	0.682208	0.50069	0.659867	0.269681	0.049379	0.552353
Bacteria	Firmicutes	Clostridia	Clostridiales	Lachnospiraceae	Anaerostipes	0.012418	0.018823	0.01801	0.016778	0.016985	0.01556	0.013068	0.008594	0.005117	0.008762	0.001469	0.009603
Bacteria	Firmicutes	Clostridia	Clostridiales	Ruminococcaceae	Anaerotruncus	0.011397	0.022588	0.024366	0.014681	0.023419	0.020555	0.018414	0.015141	0.007815	0.017524	0.001848	0.013557
Bacteria	Proteobacteria	Alphaproteobacteria	Rickettsiales	Anaplasmataceae	Anaplasma	0.005613	0.004706	0.003178	0.007341	0.008235	0.009221	0.012771	0.012481	0.008001	0.002921	0.000237	0.00481
Bacteria	Firmicutes	Bacilli	Bacillales	Bacillaceae	Anoxybacillus	0.037763	0.051763	0.033901	0.037751	0.033714	0.028623	0.029997	0.027623	0.027909	0.028234	0.007345	0.026076
Bacteria	Aquificae	Aquificae (class)	Aquificales	Aquificaceae	Aquifex	0.074506	0.135526	0.149375	0.128984	0.11581	0.098739	0.088209	0.080209	0.054981	0.024339	0.005213	0.078079
Bacteria	Actinobacteria	Actinobacteria (class)	Actinomycetales	Actinomycetaceae	Arcanobacterium	0.006464	0.010353	0.006356	0.005243	0.005147	0.004226	0.004455	0.004092	0.002791	0.000974	0.000758	0.00341
Bacteria	Proteobacteria	Epsilonproteobacteria	Campylobacterales	Campylobacteraceae	Arcobacter	0.079099	0.092233	0.131366	0.063967	0.07592	0.132164	0.079893	0.087575	0.047167	0.7185	0.04483	3.440295
Bacteria	Proteobacteria	Betaproteobacteria	Rhodocyclales	Rhodocyclaceae	Aromatoleum	0.225899	0.255993	0.343246	0.250627	0.220039	0.200167	0.182952	0.152642	0.226995	0.157719	0.158135	0.184639
Bacteria	Actinobacteria	Actinobacteria (class)	Actinomycetales	Micrococcaceae	Arthrobacter	0.088114	0.17976	0.208702	0.13947	0.081582	0.085484	0.076923	0.077139	0.09731	0.073992	0.027485	0.065494
Bacteria	Cyanobacteria	unclassified (derived from Cyanobacteria)	Oscillatoriales	unclassified (derived from Oscillatoriales)	Arthrospira	0.038444	0.082821	0.066742	0.063967	0.059964	0.063008	0.069201	0.054223	0.097217	0.034075	0.031039	0.040786
Bacteria	Proteobacteria	Alphaproteobacteria	Caulobacterales	Caulobacteraceae	Asticcacaulis	0.043377	0.045175	0.044495	0.035654	0.030368	0.033425	0.051381	0.049107	0.046515	0.033102	0.008483	0.022386
Bacteria	Actinobacteria	Actinobacteria (class)	Coriobacteriales	Coriobacteriaceae	Atopobium	0.013268	0.035764	0.01695	0.027265	0.0175	0.012871	0.019602	0.011663	0.010792	0.020445	0.001706	0.013145
Bacteria	Proteobacteria	Alphaproteobacteria	Rhizobiales	Aurantimonadaceae	Aurantimonas	0.059707	0.062116	0.102762	0.074454	0.103714	0.109689	0.117612	0.13218	0.154152	0.06523	0.037626	0.046864
Bacteria	Proteobacteria	Betaproteobacteria	Rhodocyclales	Rhodocyclaceae	Azoarcus	0.245121	0.207053	0.324177	0.245383	0.191472	0.175002	0.188001	0.152846	0.228577	0.159667	0.33698	0.207124
Bacteria	Proteobacteria	Alphaproteobacteria	Rhizobiales	Xanthobacteraceae	Azorhizobium	0.06515	0.072469	0.094287	0.081794	0.161619	0.121791	0.155331	0.157348	0.149687	0.082754	0.024832	0.049187
Bacteria	Proteobacteria	Alphaproteobacteria	Rhodospirillales	Rhodospirillaceae	Azospirillum	0.066681	0.08188	0.100643	0.088086	0.37368	0.452009	0.578258	0.647193	0.364681	0.14701	0.205856	0.090219
Bacteria	Proteobacteria	Gammaproteobacteria	Pseudomonadales	Pseudomonadaceae	Azotobacter	0.14663	0.101644	0.131366	0.131081	0.121986	0.119678	0.149391	0.148754	0.13843	0.142142	0.868631	0.159173
Bacteria	Firmicutes	Bacilli	Bacillales	Bacillaceae	Bacillus	1.337021	1.221613	1.128262	1.122052	0.898942	0.866366	0.953071	0.758912	0.689265	0.632825	0.157046	0.753415
Bacteria	Bacteroidetes	Bacteroidia	Bacteroidales	Bacteroidaceae	Bacteroides	1.625008	2.092176	1.457735	1.736559	0.677102	0.817189	1.003858	0.887205	0.694289	1.395136	0.235094	1.692589
Bacteria	Proteobacteria	Alphaproteobacteria	Rhizobiales	Bartonellaceae	Bartonella	0.04916	0.04894	0.044495	0.057676	0.066912	0.048793	0.092367	0.074684	0.046608	0.047705	0.006871	0.026241
Bacteria	Proteobacteria	Gammaproteobacteria	Pasteurellales	Pasteurellaceae	Basfia	0.031299	0.026352	0.022247	0.028313	0.022647	0.016713	0.026136	0.021894	0.021211	0.020445	0.038479	0.028085
Bacteria	Proteobacteria	Deltaproteobacteria	Bdellovibrionales	Bdellovibrionaceae	Bdellovibrio	0.061748	0.095056	0.114415	0.102767	0.118126	0.146379	0.162459	0.249833	0.11387	0.069124	0.013885	0.08055

Bacteria	Proteobacteria	Gammaproteobacteria	Thiotrichales	Thiotrichaceae	Beggiatoa	0.139996	0.159995	0.17692	0.16254	0.284635	0.242237	0.206118	0.143843	0.123173	0.062309	0.074826	0.212856
Bacteria	Proteobacteria	Alphaproteobacteria	Rhizobiales	Beijerinckiaceae	Beijerinckia	0.043036	0.038587	0.06992	0.04614	0.048383	0.053019	0.079299	0.062407	0.067075	0.058415	0.018434	0.027657
Bacteria	Proteobacteria	Gammaproteobacteria	Oceanospirillales	Oceanospirillaceae	Bermanella	0.060217	0.047057	0.049792	0.05453	0.079523	0.084524	0.120285	0.154074	0.088844	0.242421	0.366076	0.205938
Bacteria	Actinobacteria	Actinobacteria (class)	Actinomycetales	Beutenbergiaceae	Beutenbergia	0.044737	0.085645	0.064623	0.062919	0.035515	0.028239	0.028512	0.026395	0.033026	0.014604	0.002796	0.029568
Bacteria	Actinobacteria	Actinobacteria (class)	Bifidobacteriales	Bifidobacteriaceae	Bifidobacterium	0.08131	0.09035	0.073099	0.05453	0.048125	0.035154	0.061776	0.042969	0.028002	0.038943	0.006066	0.037112
Bacteria	Planctomycetes	Planctomycetacia	Planctomycetales	Planctomycetaceae	Blastopirellula	0.220626	0.380225	0.447067	0.287329	0.485887	0.463535	0.388772	0.357255	0.405521	0.121697	0.019429	0.226825
Bacteria	Bacteroidetes	Flavobacteria	Flavobacteriales	Blattabacteriaceae	Blattabacterium	0.008165	0.018823	0.01801	0.008389	0.005147	0.007492	0.010395	0.009821	0.012466	0.021419	0.00436	0.023523
Bacteria	Firmicutes	Clostridia	Clostridiales	unclassified (derived from Clostridiales)	Blautia	0.013268	0.034823	0.021188	0.027265	0.024706	0.025549	0.019305	0.015551	0.00921	0.011683	0.002085	0.019372
Bacteria	Proteobacteria	Betaproteobacteria	Burkholderiales	Alcaligenaceae	Bordetella	0.359261	0.305874	0.3888	0.349199	0.227759	0.244926	0.308285	0.264565	0.354912	0.264813	0.25097	0.200815
Bacteria	Spirochaetes	Spirochaetes (class)	Spirochaetales	Spirochaetaceae	Borrelia	0.08216	0.082821	0.067802	0.060822	0.04478	0.057245	0.064449	0.051153	0.021025	0.020445	0.003128	0.038397
Bacteria	Actinobacteria	Actinobacteria (class)	Actinomycetales	Dermabacteraceae	Brachybacterium	0.025346	0.027293	0.021188	0.01573	0.011324	0.013063	0.01485	0.012277	0.015443	0.008762	0.001801	0.010328
Bacteria	Spirochaetes	Spirochaetes (class)	Spirochaetales	Brachyspiraceae	Brachyspira	0.043036	0.06588	0.054029	0.055578	0.063309	0.048793	0.068013	0.05013	0.027723	0.049652	0.005924	0.05197
Bacteria	Proteobacteria	Alphaproteobacteria	Rhizobiales	Bradyrhizobiaceae	Bradyrhizobium	0.706954	0.543985	0.83057	0.649112	0.520115	0.564003	0.661418	0.595426	0.848627	0.472185	0.195667	0.328328
Bacteria	Firmicutes	Bacilli	Bacillales	Paenibacillaceae	Brevibacillus	0.042186	0.068704	0.063564	0.05453	0.061765	0.056285	0.065934	0.045833	0.044655	0.047705	0.011563	0.048116
Bacteria	Actinobacteria	Actinobacteria (class)	Actinomycetales	Brevibacteriaceae	Brevibacterium	0.014119	0.035764	0.025426	0.022022	0.01853	0.013831	0.014553	0.014732	0.021862	0.015577	0.003838	0.012799
Bacteria	Proteobacteria	Alphaproteobacteria	Caulobacterales	Caulobacteraceae	Brevundimonas	0.035042	0.058351	0.058267	0.050335	0.064854	0.090479	0.120582	0.113561	0.079913	0.04089	0.026253	0.031083
Bacteria	Proteobacteria	Alphaproteobacteria	Rhizobiales	Brucellaceae	Brucella	1.211994	0.186348	0.339008	0.242237	0.139487	0.129091	0.250668	0.203386	0.191923	0.125591	0.090796	0.092526
Bacteria	Proteobacteria	Gammaproteobacteria	Enterobacteriales	Enterobacteriaceae	Buchnera	0.011397	0.006588	0.011653	0.019924	0.013897	0.01095	0.014553	0.012277	0.011443	0.004868	0.003033	0.013837
Bacteria	Firmicutes	Erysipelotrichi	Erysipelotrichales	Erysipelotrichaceae	Bulleidia	0.001021	0.002823	0.005297	0.002097	0.002316	0.002497	0.00297	0.002251	0.001209	0	0.000711	0.002224
Bacteria	Proteobacteria	Betaproteobacteria	Burkholderiales	Burkholderiaceae	Burkholderia	3.237599	1.488899	1.835941	1.583457	0.901773	0.880966	1.136617	0.935494	1.292477	1.329906	0.865599	0.79028
Bacteria	Firmicutes	Clostridia	Clostridiales	Lachnospiraceae	Butyrivibrio	0.013949	0.038587	0.015891	0.030411	0.020074	0.019978	0.023166	0.018415	0.012094	0.015577	0.001753	0.022353
Bacteria	Firmicutes	Clostridia	Thermoanaerobacterales	Thermoanaerobacteraceae	Caldanaerobacter	0.196301	0.282345	0.215058	0.254821	0.228789	0.189217	0.170181	0.111719	0.088751	0.088596	0.012084	0.15148
Bacteria	Firmicutes	Clostridia	Thermoanaerobacterales	Thermoanaerobacterales Family III. Incertae Sedis	Caldicellulosiruptor	0.190687	0.358578	0.266969	0.273697	0.21798	0.20228	0.198693	0.134227	0.093682	0.11975	0.020519	0.177704
Bacteria	Deferribacteres	Deferribacteres (class)	Deferribacterales	Deferribacteraceae	Calditerrivibrio	0.029768	0.044234	0.045554	0.072357	0.073346	0.066466	0.052272	0.039695	0.028653	0.056467	0.006445	0.052761
Bacteria	Proteobacteria	Epsilonproteobacteria	Nautiliales	Nautiliaceae	Caminibacter	0.008845	0.014117	0.01801	0.017827	0.01261	0.014023	0.012474	0.012891	0.006233	0.002921	0.002417	0.034543
Bacteria	Proteobacteria	Epsilonproteobacteria	Campylobacterales	Campylobacteraceae	Campylobacter	0.182862	0.205171	0.210821	0.131081	0.114266	0.123135	0.150579	0.102102	0.062145	0.094437	0.032224	0.457175
Bacteria	Proteobacteria	Betaproteobacteria	unclassified (derived from Betaproteobacteria)	unclassified (derived from Betaproteobacteria)	Candidatus Accumulibacter	0.187965	0.189171	0.283919	0.191902	0.164965	0.151374	0.134838	0.099033	0.185596	0.16064	0.076864	0.161265
Bacteria	Bacteroidetes	unclassified (derived from Bacteroidetes)	unclassified (derived from Bacteroidetes)	unclassified (derived from Bacteroidetes)	Candidatus Amoebophilus	0.021093	0.039528	0.045554	0.031459	0.02728	0.030736	0.040095	0.047061	0.051446	0.034075	0.012084	0.037442
Bacteria	Bacteroidetes	Bacteroidia	Bacteroidales	unclassified (derived from Bacteroidales)	Candidatus Azobacteroides	0.013438	0.043293	0.027544	0.030411	0.010037	0.012871	0.019305	0.013709	0.01321	0.023366	0.006634	0.035679
Bacteria	Proteobacteria	Gammaproteobacteria	Enterobacteriales	Enterobacteriaceae	Candidatus Blochmannia	0.007485	0.004706	0.010594	0.006292	0.004375	0.002881	0.004158	0.002865	0.002977	0	0.00109	0.004777
Bacteria	Proteobacteria	Gammaproteobacteria	unclassified (derived from Gammaproteobacteria)	unclassified (derived from Gammaproteobacteria)	Candidatus Carsonella	0	0	0	0	0	0	0	0	9.3E-05	0	0	0.000165
Bacteria	unclassified (derived from Bacteria)	unclassified (derived from Bacteria)	unclassified (derived from Bacteria)	unclassified (derived from Bacteria)	Candidatus Cloacamonas	0.035722	0.086586	0.039198	0.051384	0.057905	0.067427	0.059697	0.056269	0.022606	0.034075	0.005829	0.056517
Bacteria	Firmicutes	Clostridia	Clostridiales	Peptococcaceae	Candidatus Desulforudis	0.119243	0.148702	0.111237	0.132129	0.121472	0.09797	0.07425	0.055655	0.052562	0.034075	0.004549	0.071095
Bacteria	Proteobacteria	Gammaproteobacteria	Enterobacteriales	Enterobacteriaceae	Candidatus Hamiltonella	0.002381	0.005647	0.006356	0.004195	0.008493	0.011718	0.013068	0.012072	0.00614	0.012657	0.010283	0.006144
Bacteria	Proteobacteria	Alphaproteobacteria	Rhizobiales	unclassified (derived from Rhizobiales)	Candidatus Hodgkinia	0	0	0	0	0	0	0	0.000205	9.3E-05	0	0	6.59E-05
Bacteria	Acidobacteria	unclassified (derived from Acidobacteria)	unclassified (derived from Acidobacteria)	unclassified (derived from Acidobacteria)	Candidatus Koribacter	0.467447	0.465869	0.513809	0.45721	0.319893	0.294296	0.281258	0.228963	0.370169	0.196663	0.015543	0.251649
Bacteria	Proteobacteria	Alphaproteobacteria	Rhizobiales	Rhizobiaceae	Candidatus Liberibacter	0.002722	0.004706	0.004238	0.003146	0.00386	0.005187	0.006831	0.005525	0.003256	0.001947	0.016349	0.003179
Bacteria	Proteobacteria	Alphaproteobacteria	Rickettsiales	unclassified (derived from Rickettsiales)	Candidatus Pelagibacter	0.053923	0.031999	0.047673	0.044043	0.06614	0.073382	0.098901	0.080209	0.070331	0.080807	0.016112	0.039303
Bacteria	Tenericutes	Mollicutes	Acholeplasmatales	Acholeplasmataceae	Candidatus Phytoplasma	0.001021	0.000941	0.001059	0.001049	0.003088	0.006531	0.011583	0.005525	0.002512	0.003894	0.000948	0.00308
Bacteria	Chlamydiae	Chlamydiae (class)	Chlamydiales	Parachlamydiaceae	Candidatus Protochlamydia	0.03334	0.064939	0.039198	0.059773	0.083898	0.05398	0.081972	0.087779	0.030886	0.034075	0.003459	0.031561
Bacteria	Proteobacteria	Alphaproteobacteria	unclassified (derived from Alphaproteobacteria)	unclassified (derived from Alphaproteobacteria)	Candidatus Puniceispirillum	0.035552	0.028234	0.033901	0.042995	0.118383	0.139464	0.165429	0.1907	0.118521	0.036996	0.032461	0.034246
Bacteria	Proteobacteria	Gammaproteobacteria	Enterobacteriales	Enterobacteriaceae	Candidatus Regiella	0.004763	0.003765	0.006356	0.003146	0.005147	0.003074	0.003267	0.003888	0.004652	0.004868	0.001043	0.004678
Bacteria	Proteobacteria	Gammaproteobacteria	Enterobacteriales	Enterobacteriaceae	Candidatus Riesia	0.001021	0	0.001059	0.001049	0.002316	0.000576	0.001188	0.000409	0.000651	0.001947	4.74E-05	0.000791
Bacteria	Acidobacteria	Solibacteres	Solibacterales	Solibacteraceae	Candidatus Solibacter	0.812419	0.863034	0.981005	0.923858	0.664491	0.604343	0.505493	0.475113	0.930773	0.269681	0.026632	0.487962
Bacteria	Bacteroidetes	Flavobacteria	Flavobacteriales	unclassified (derived from Flavobacteriales)	Candidatus Sulcia	0.006124	0.004706	0.002119	0.008389	0.002574	0.00461	0.003861	0.004911	0.004745	0.011683	0.002654	0.010147
Bacteria	Proteobacteria	Betaproteobacteria	Burkholderiales	Oxalobacteraceae	Candidatus Zinderia	0	0	0.002119	0	0	0.000384	0	0	9.3E-05	0	9.48E-05	9.88E-05
Bacteria	Bacteroidetes	Flavobacteria	Flavobacteriales	Flavobacteriaceae	Capnocytophaga	0.090155	0.147761	0.138781	0.112205	0.059706	0.087789	0.106623	0.130748	0.12801	0.785677	0.089564	0.453304
Bacteria	Firmicutes	Clostridia	Thermoanaerobacterales	Thermoanaerobacteraceae	Carboxydothermus	0.158367	0.259757	0.187514	0.25587	0.219009	0.183839	0.163944	0.107218	0.079634	0.053547	0.005876	0.135189
Bacteria	Proteobacteria	Gammaproteobacteria	Cardiobacteriales	Cardiobacteriaceae	Cardiobacterium	0.015309	0.020705	0.013772	0.02307	0.014155	0.016136	0.016929	0.018415	0.01628	0.017524	0.01289	0.017988
Bacteria	Firmicutes	Bacilli	Lactobacillales	Carnobacteriaceae	Carnobacterium	0.004933	0.00847	0.006356	0.010486	0.008493	0.005955	0.006534	0.00532	0.003907	0.005841	0.001469	0.005452
Bacteria	Firmicutes	Erysipelotrichi	Erysipelotrichales	Erysipelotrichaceae	Catenibacterium	0.001531	0.005647	0.001059	0.010486	0.005404	0.006723	0.005346	0.004092	0.003628	0.002921	0.001185	0.005074
Bacteria	Actinobacteria	Actinobacteria (class)	Actinomycetales	Catenulisporaceae	Catenulispora	0.054093	0.095997	0.080514	0.089135	0.049927	0.036115	0.047223	0.04215	0.053121	0.029207	0.008956	0.032731
Bacteria	Firmicutes	Clostridia	Clostridiales	Lachnospiraceae	Catonella	0.003062	0.000941	0.003178	0.006292	0.003088	0.004418	0.005346	0.004706	0.002047	0.003894	0.000663	0.002899
Bacteria	Proteobacteria	Alphaproteobacteria	Caulobacterales	Caulobacteraceae	Caulobacter	0.307889	0.22117	0.250019	0.288378	0.212575	0.24896	0.341846	0.398997	0.320119	0.229764	0.063927	0.137182
Bacteria	Actinobacteria	Actinobacteria (class)	Actinomycetales	Cellulomonadaceae	Cellulomonas	0.031639	0.04894	0.058267	0.026216	0.020588	0.018442	0.016335	0.018415	0.018978	0.009736	0.003838	0.016752
Bacteria	Firmicutes	Clostridia	Clostridiales	Lachnospiraceae	Cellulosilyticum	0.01633	0.022588	0.025426	0.027265	0.020074	0.022283	0.023166	0.016164	0.011815	0.016551	0.003412	0.020212
Bacteria	Proteobacteria	Gammaproteobacteria	Pseudomonadales	Pseudomonadaceae	Cellvibrio	0.159048	0.123291	0.177979	0.136324	0.29287	0.223987	0.34838	0.3947	0.285791	0.230738	0.798733	0.254647
Bacteria	Proteobacteria	Alphaproteobacteria	Rhizobiales	Phyllobacteriaceae	Chelativorans	0.204125	0.127055	0.206583	0.16254	0.219267	0.200551	0.222156	0.268453	0.266719	0.129486	0.070609	0.088078
Bacteria	Bacteroidetes	Sphingobacteria	Sphingobacteriales	unclassified (derived from Sphingobacteriales)	Chitinophaga	0.158707	0.26164	0.220355	0.245383	0.139744	0.176347	0.184437	0.309785	0.309048	0.315439	0.056534	0.281233
Bacteria	Chlamydiae	Chlamydiae (class)	Chlamydiales	Chlamydiaceae	Chlamydia	0.067531	0.031999	0.021188	0.025168	0.020588	0.016905	0.033561	0.02128	0.012559	0.005841	0.001374	0.011728
Bacteria	Chlamydiae	Chlamydiae (class)	Chlamydiales	Chlamydiaceae	Chlamydophila	0.03198	0.03294	0.030723	0.035654	0.035258	0.024012	0.046629	0.03192	0.016653	0.024339	0.002275	0.018861
Bacteria	Chlorobi	Chlorobia	Chlorobiales	Chlorobiaceae	Chlorobaculum	0.19477	0.328461	0.21294	0.225459	0.178347	0.178268	0.174339	0.171262	0.158059	0.107093	0.027391	0.168084
Bacteria	Chlorobi	Chlorobia	Chlorobiales	Chlorobiaceae	Chlorobium	0.512185	0.800919	0.589027	0.624993	0.60427	0.525391	0.512027	0.468565	0.382077	0.379695	0.081319	0.456829
Bacteria	Chloroflexi	Chloroflexi (class)	Chloroflexales	Chloroflexaceae	Chloroflexus	0.497896	0.56469	0.468255	0.445675	0.352834	0.247231	0.25245	0.190291	0.299838	0.143116	0.058857	0.284462

Bacteria	Chlorobi	Chlorobia	Chlorobiales	Chlorobiaceae	Chloroherpeton	0.126558	0.189171	0.165266	0.179319	0.188641	0.193444	0.188595	0.23285	0.207924	0.156746	0.03502	0.16759
Bacteria	Proteobacteria	Betaproteobacteria	Neisseriales	Neisseriaceae	Chromobacterium	0.194429	0.125173	0.161029	0.184562	0.110148	0.113723	0.118206	0.101079	0.128382	0.125591	0.194103	0.126409
Bacteria	Proteobacteria	Gammaproteobacteria	Oceanospirillales	Halomonadaceae	Chromohalobacter	0.207868	0.137408	0.182217	0.149956	0.192759	0.202472	0.239382	0.326359	0.466828	0.180112	1.26769	0.457587
Bacteria	Bacteroidetes	Flavobacteria	Flavobacteriales	Flavobacteriaceae	Chryseobacterium	0.027897	0.05741	0.029663	0.048238	0.028052	0.034578	0.051084	0.067113	0.06754	0.134354	0.028244	0.100943
Bacteria	Verrucomicrobia	Spartobacteria	unclassified (derived from Spartobacteria)	unclassified (derived from Spartobacteria)	Chthoniobacter	0.153945	0.227758	0.286038	0.222313	0.216178	0.203049	0.178497	0.189472	0.265696	0.165508	0.017581	0.174608
Bacteria	Proteobacteria	Alphaproteobacteria	Rhodobacterales	Rhodobacteraceae	Citreicella	0.055624	0.052704	0.048732	0.076551	0.061765	0.098547	0.101277	0.151823	0.173316	0.099305	0.063595	0.044937
Bacteria	Proteobacteria	Gammaproteobacteria	Enterobacteriales	Enterobacteriaceae	Citrobacter	0.076717	0.070586	0.057208	0.057676	0.043493	0.048985	0.052569	0.046652	0.046981	0.045758	0.080039	0.046469
Bacteria	Proteobacteria	Alphaproteobacteria	Sphingomonadales	Sphingomonadaceae	Citromicrobium	0.036232	0.038587	0.051911	0.052432	0.024449	0.024397	0.034155	0.045424	0.040934	0.038943	0.010662	0.022534
Bacteria	Actinobacteria	Actinobacteria (class)	Actinomycetales	Microbacteriaceae	Clavibacter	0.029768	0.04894	0.050851	0.048238	0.020331	0.019786	0.027621	0.024349	0.024467	0.023366	0.006445	0.018531
Bacteria	Firmicutes	Clostridia	Clostridiales	Clostridiaceae	Clostridium	1.291093	1.693129	1.320013	1.609673	1.349571	1.282453	1.267	0.928946	0.587117	0.74576	0.169509	1.214412
Bacteria	Proteobacteria	Betaproteobacteria	Burkholderiales	Oxalobacteraceae	Collimonas	0	0	0	0	0	0.000192	0.000297	0	0	0	0	1.65E-05
Bacteria	Actinobacteria	Actinobacteria (class)	Coriobacteriales	Coriobacteriaceae	Collinsella	0.013268	0.018823	0.012713	0.017827	0.016471	0.012294	0.017226	0.009821	0.00921	0.006815	0.002512	0.011382
Bacteria	Proteobacteria	Gammaproteobacteria	Alteromonadales	Colwelliaceae	Colwellia	0.187965	0.129879	0.204464	0.165686	0.15956	0.191331	0.194832	0.200726	0.184201	0.140195	1.219354	0.246559
Bacteria	Proteobacteria	Betaproteobacteria	Burkholderiales	Comamonadaceae	Comamonas	0.059877	0.071527	0.073099	0.082843	0.051728	0.055325	0.07425	0.067522	0.056935	0.06815	0.045493	0.047243
Bacteria	Actinobacteria	Actinobacteria (class)	Solirubrobacterales	Conexibacteraceae	Conexibacter	0.084202	0.13082	0.156791	0.08494	0.076177	0.069156	0.068013	0.060156	0.086612	0.072045	0.008009	0.050307
Bacteria	Proteobacteria	Gammaproteobacteria	unclassified (derived from Gammaproteobacteria)	unclassified (derived from Gammaproteobacteria)	Congregibacter	0.24359	0.205171	0.270147	0.267405	0.284892	0.511175	0.494207	0.589492	0.265324	0.182059	0.337311	0.283737
Bacteria	Firmicutes	Erysipelotrichi	Erysipelotrichales	Erysipelotrichaceae	Coprobacillus	0.010376	0.003765	0.002119	0.004195	0.003346	0.003842	0.006237	0.006343	0.002605	0.008762	0.000379	0.004695
Bacteria	Firmicutes	Clostridia	Clostridiales	Lachnospiraceae	Coprococcus	0.008165	0.013176	0.007416	0.024119	0.010809	0.0146	0.009207	0.011868	0.004652	0.004868	0.002085	0.010789
Bacteria	Firmicutes	Clostridia	Thermoanaerobacterales	Thermodesulfobiaceae	Coprothermobacter	0.034021	0.041411	0.024366	0.032508	0.040662	0.026702	0.027621	0.016778	0.012745	0.012657	0.001659	0.025285
Bacteria	Verrucomicrobia	Opitutae	Puniceicoccales	Puniceicoccaceae	Coraliomargarita	0.115161	0.152466	0.133484	0.128984	0.171399	0.219185	0.181764	0.829094	0.129406	0.071071	0.046772	0.099444
Bacteria	Actinobacteria	Actinobacteria (class)	Actinomycetales	Corynebacteriaceae	Corynebacterium	0.141187	0.194818	0.180098	0.171978	0.11478	0.094513	0.136323	0.116221	0.106613	0.06815	0.033362	0.084915
Bacteria	Proteobacteria	Gammaproteobacteria	Legionellales	Coxiellaceae	Coxiella	0.274549	0.093174	0.118653	0.136324	0.152097	0.131204	0.174339	0.143025	0.08196	0.099305	0.045635	0.1032
Bacteria	Bacteroidetes	Flavobacteria	Flavobacteriales	Flavobacteriaceae	Croceibacter	0.111589	0.131761	0.129247	0.130032	0.059449	0.114107	0.108702	0.21955	0.322631	2.199311	0.302907	0.784828
Bacteria	Cyanobacteria	unclassified (derived from Cyanobacteria)	Chroococcales	unclassified (derived from Chroococcales)	Crocosphaera	0.033681	0.063057	0.039198	0.041946	0.069229	0.062624	0.063558	0.057292	0.097682	0.023366	0.023694	0.033653
Bacteria	Proteobacteria	Gammaproteobacteria	Enterobacteriales	Enterobacteriaceae	Cronobacter	0.038274	0.059292	0.050851	0.049286	0.032169	0.041109	0.057024	0.053404	0.044841	0.073018	0.093687	0.044245
Bacteria	Actinobacteria	Actinobacteria (class)	Coriobacteriales	Coriobacteriaceae	Cryptobacterium	0.010887	0.022588	0.013772	0.018876	0.013897	0.008068	0.010692	0.009821	0.006791	0.005841	0.001043	0.009274
Bacteria	Proteobacteria	Betaproteobacteria	Burkholderiales	Burkholderiaceae	Cupriavidus	0.518989	0.509162	0.696026	0.578853	0.347687	0.319461	0.368873	0.317765	0.522926	0.397219	0.493077	0.333912
Bacteria	Cyanobacteria	unclassified (derived from Cyanobacteria)	Chroococcales	unclassified (derived from Chroococcales)	Cyanobium	0.021433	0.036705	0.030723	0.047189	0.016471	0.017673	0.016632	0.014937	0.026049	0.017524	0.007772	0.017016
Bacteria	Cyanobacteria	unclassified (derived from Cyanobacteria)	Chroococcales	unclassified (derived from Chroococcales)	Cyanothece	0.452478	0.544926	0.449186	0.50335	0.623829	0.649487	0.561626	0.524834	1.082878	0.237553	0.216423	0.361734
Bacteria	Cyanobacteria	unclassified (derived from Cyanobacteria)	Nostocales	Nostocaceae	Cylindrospermopsis	0.010036	0.023529	0.01801	0.022022	0.017243	0.016905	0.024354	0.009617	0.026886	0.019472	0.013032	0.01298
Bacteria	Cyanobacteria	unclassified (derived from Cyanobacteria)	Nostocales	Nostocaceae	Cylindrospermum	0	0	0	0	0	0	0	0	0.000279	0	0	0
Bacteria	Bacteroidetes	Cytophagia	Cytophagales	Cytophagaceae	Cytophaga	0.185754	0.296462	0.263791	0.297816	0.173715	0.256644	0.223344	0.446262	0.440222	0.299862	0.051701	0.343862
Bacteria	Proteobacteria	Betaproteobacteria	Rhodocyclales	Rhodocyclaceae	Dechloromonas	0.30942	0.263522	0.362315	0.242237	0.212575	0.193828	0.17523	0.160826	0.213227	0.165508	0.419673	0.239657
Bacteria	Deferribacteres	Deferribacteres (class)	Deferribacterales	Deferribacteraceae	Deferribacter	0.054944	0.080939	0.078396	0.102767	0.131509	0.111994	0.098307	0.072024	0.046701	0.090543	0.01071	0.082428
Bacteria	Chloroflexi	Dehalococcoidetes	unclassified (derived from Dehalococcoidetes)	unclassified (derived from Dehalococcoidetes)	Dehalococcoides	0.459452	0.784919	0.383503	0.637577	0.558718	0.2407	0.226611	0.092281	0.08317	0.152852	0.015875	0.279866
Bacteria	Chloroflexi	Dehalococcoidetes	unclassified (derived from Dehalococcoidetes)	unclassified (derived from Dehalococcoidetes)	Dehalogenimonas	0.101382	0.189171	0.102762	0.148908	0.196362	0.089518	0.068904	0.037854	0.034235	0.058415	0.005734	0.089742
Bacteria	Deinococcus-Thermus	Deinococci	Deinococcales	Deinococcaceae	Deinococcus	0.176398	0.201406	0.182217	0.183513	0.145148	0.125441	0.161865	0.15878	0.149128	0.070098	0.022367	0.106527
Bacteria	Proteobacteria	Betaproteobacteria	Burkholderiales	Comamonadaceae	Delftia	0.066511	0.079998	0.10594	0.082843	0.063052	0.062816	0.069201	0.076525	0.07796	0.052573	0.052696	0.049763
Bacteria	Deferribacteres	Deferribacteres (class)	Deferribacterales	Deferribacteraceae	Denitrovibrio	0.089305	0.111997	0.099584	0.106962	0.147465	0.126017	0.10692	0.081845	0.049865	0.044785	0.013837	0.095738
Bacteria	Actinobacteria	Actinobacteria (class)	Actinomycetales	Dermacoccaceae	Dermacoccus	0.009866	0.013176	0.01801	0.016778	0.005147	0.006147	0.006237	0.006343	0.007442	0.008762	0.001374	0.005255
Bacteria	Proteobacteria	Deltaproteobacteria	Desulfarculales	Desulfarculaceae	Desulfarculus	0.191027	0.317168	0.238365	0.308302	0.389636	0.360762	0.254528	0.126451	0.074239	0.08178	0.012558	0.205329
Bacteria	Proteobacteria	Deltaproteobacteria	Desulfobacterales	Desulfobacteraceae	Desulfatibacillum	0.841337	1.273376	0.921679	1.000409	2.254432	2.443307	1.411935	0.663971	0.207087	0.207372	0.029191	0.971608
Bacteria	Firmicutes	Clostridia	Clostridiales	Peptococcaceae	Desulfitobacterium	0.239337	0.472457	0.350662	0.319837	0.318606	0.251265	0.187407	0.123382	0.085681	0.080807	0.014501	0.19785
Bacteria	Proteobacteria	Deltaproteobacteria	Desulfobacterales	Desulfobacteraceae	Desulfobacterium	0.631938	0.953385	0.67166	0.762366	1.793251	1.950573	1.138993	0.588469	0.162246	0.22587	0.116576	0.787282
Bacteria	Proteobacteria	Deltaproteobacteria	Desulfobacterales	Desulfobacteraceae	Desulfococcus	0.692325	1.089852	0.792432	0.808507	1.997076	2.129609	1.185028	0.5588	0.181782	0.204451	0.029097	0.862989
Bacteria	Proteobacteria	Deltaproteobacteria	Desulfovibrionales	Desulfohalobiaceae	Desulfohalobium	0.177589	0.216464	0.19387	0.22441	0.285407	0.296985	0.222453	0.146913	0.062145	0.058415	0.0127	0.142948
Bacteria	Proteobacteria	Deltaproteobacteria	Desulfovibrionales	Desulfomicrobiaceae	Desulfomicrobium	0.200213	0.295521	0.278622	0.261113	0.373165	0.387464	0.278288	0.175968	0.076285	0.064256	0.061131	0.211489
Bacteria	Proteobacteria	Deltaproteobacteria	Desulfovibrionales	Desulfohalobiaceae	Desulfonatronospira	0.101212	0.20423	0.149375	0.171978	0.239598	0.256836	0.168102	0.091667	0.044283	0.038943	0.006729	0.105967
Bacteria	Proteobacteria	Deltaproteobacteria	Desulfobacterales	Desulfobulbaceae	Desulfotalea	0.415735	0.454575	0.500037	0.404778	1.143687	0.875971	0.47906	0.390198	0.125126	0.127539	0.041986	0.389029
Bacteria	Firmicutes	Clostridia	Clostridiales	Peptococcaceae	Desulfotomaculum	0.287817	0.44987	0.360196	0.417361	0.388606	0.340207	0.272942	0.183129	0.118428	0.105146	0.020661	0.230152
Bacteria	Proteobacteria	Deltaproteobacteria	Desulfovibrionales	Desulfovibrionaceae	Desulfovibrio	1.41595	1.93124	1.589101	1.67364	1.934796	2.171295	1.678047	1.107164	0.532229	0.496524	0.123494	1.17043
Bacteria	Chrysiogenetes	Chrysiogenetes (class)	Chrysiogenales	Chrysiogenaceae	Desulfurispirillum	0.050011	0.060234	0.083693	0.066065	0.099339	0.082795	0.080784	0.076525	0.049399	0.05452	0.035304	0.080221
Bacteria	Proteobacteria	Deltaproteobacteria	Desulfobacterales	Desulfobulbaceae	Desulfurivibrio	0.303807	0.252228	0.272266	0.297816	0.872434	0.573608	0.266705	0.193769	0.0881	0.117803	0.04336	0.247877
Bacteria	Proteobacteria	Deltaproteobacteria	Desulfuromonadales	Desulfuromonadaceae	Desulfuromonas	0.204295	0.271992	0.268028	0.269502	0.300334	0.27547	0.23166	0.16676	0.109218	0.110014	0.121504	0.26962
Bacteria	Firmicutes	Clostridia	Clostridiales	Syntrophomonadaceae	Dethiobacter	0.052052	0.084703	0.081574	0.079697	0.100626	0.085676	0.065637	0.04215	0.034421	0.033102	0.007487	0.05935
Bacteria	Synergistetes	Synergistia	Synergistales	Synergistaceae	Dethiosulfovibrio	0.041165	0.088468	0.055089	0.079697	0.066912	0.06224	0.058806	0.038058	0.02856	0.020445	0.00218	0.049533
Bacteria	Firmicutes	Negativicutes	Selenomonadales	Veillonellaceae	Dialister	0.006294	0.013176	0.004238	0.010486	0.021103	0.016521	0.024948	0.021075	0.007442	0.009736	0.001848	0.009669
Bacteria	Proteobacteria	Gammaproteobacteria	Cardiobacteriales	Cardiobacteriaceae	Dichelobacter	0.039804	0.017882	0.024366	0.035654	0.028052	0.030352	0.02673	0.031101	0.023537	0.026287	0.024832	0.027064
Bacteria	Proteobacteria	Gammaproteobacteria	Enterobacteriales	Enterobacteriaceae	Dickeya	0.073315	0.074351	0.092168	0.079697	0.063567	0.061472	0.085536	0.062816	0.069215	0.102226	0.150364	0.077914
Bacteria	Dictyoglomi	Dictyoglomia	Dictyoglomales	Dictyoglomaceae	Dictyoglomus	0.104784	0.174113	0.125009	0.151005	0.139744	0.130243	0.10989	0.060361	0.054981	0.055494	0.010947	0.101816
Bacteria	Proteobacteria	Alphaproteobacteria	Rhodobacterales	Rhodobacteraceae	Dinoroseobacter	0.141357	0.127996	0.146197	0.184562	0.184266	0.311969	0.303236	0.531995	0.46841	0.16064	0.093687	0.134514
Bacteria	Bacteroidetes	Flavobacteria	Flavobacteriales	Flavobacteriaceae	Dokdonia	0.085733	0.093174	0.099584	0.096475	0.044522	0.084331	0.091179	0.150596	0.177503	0.990128	0.151406	0.418943
Bacteria	Firmicutes	Clostridia	Clostridiales	Lachnospiraceae	Dorea	0.01565	0.016	0.013772	0.018876	0.013897	0.012294	0.011583	0.011663	0.006698	0.004868	0.001659	0.012041
Bacteria	Bacteroidetes	Cytophagia	Cytophagales	Cytophagaceae	Dyadobacter	0.158537	0.260699	0.21294	0.269502	0.167024	0.20132	0.201663	0.298122	0.329422	0.25313	0.05317	0.261862
Bacteria	Proteobacteria	Gammaproteobacteria	Enterobacteriales	Enterobacteriaceae	Edwardsiella	0.057665	0.043293	0.047673	0.047189	0.04478	0.038804	0.046332	0.05013	0.03805	0.062309	0.073737	0.049401
Bacteria	Actinobacteria	Actinobacteria (class)	Coriobacteriales	Coriobacteriaceae	Eggerthella	0.023815	0.026352	0.029663	0.034605	0.021103	0.020747	0.015147	0.014323	0.008838	0.007789	0.001564	0.018844

Bacteria	Proteobacteria	Alphaproteobacteria	Rickettsiales	Anaplasmataceae	Ehrlichia	0.019222	0.012235	0.012713	0.011535	0.011838	0.014407	0.031779	0.022917	0.010326	0.015577	0.001422	0.00962
Bacteria	Proteobacteria	Betaproteobacteria	Neisseriales	Neisseriaceae	Eikenella	0.013949	0.00847	0.009535	0.013632	0.007721	0.007492	0.008613	0.009208	0.009024	0.003894	0.014217	0.009307
Bacteria	Elusimicrobia	Elusimicrobia (class)	Elusimicrobiales	Elusimicrobiaceae	Elusimicrobium	0.013438	0.035764	0.03496	0.039849	0.046324	0.037843	0.038907	0.031715	0.015722	0.01363	0.002322	0.02433
Bacteria	Proteobacteria	Gammaproteobacteria	unclassified (derived from Gammaproteobacteria)	unclassified (derived from Gammaproteobacteria)	Endoriftia	0.058856	0.09035	0.144078	0.102767	0.468644	0.320997	0.140481	0.103944	0.050795	0.066203	0.055776	0.265799
Bacteria	Proteobacteria	Gammaproteobacteria	Pseudomonadales	Moraxellaceae	Enhydrobacter	0.01565	0.016	0.015891	0.008389	0.009779	0.012679	0.01485	0.020052	0.013582	0.008762	0.016728	0.016028
Bacteria	Proteobacteria	Gammaproteobacteria	Enterobacteriales	Enterobacteriaceae	Enterobacter	0.223347	0.084703	0.082633	0.08494	0.076692	0.092592	0.118206	0.080004	0.057028	0.073018	0.121078	0.068739
Bacteria	Firmicutes	Bacilli	Lactobacillales	Enterococcaceae	Enterococcus	0.21161	0.096938	0.060386	0.071308	0.045809	0.052059	0.072765	0.05545	0.035166	0.048679	0.006824	0.049648
Bacteria	Firmicutes	Clostridia	Clostridiales	unclassified (derived from Clostridiales)	Epulopiscium	0.002381	0.014117	0.009535	0.008389	0.012868	0.016328	0.010989	0.008389	0.003814	0.002921	0.001185	0.008533
Bacteria	Firmicutes	Bacilli	Lactobacillales	Aerococcaceae	Eremococcus	0.004423	0.004706	0.002119	0.001049	0.003088	0.002497	0.002673	0.004297	0.002512	0.001947	0.000521	0.002372
Bacteria	Proteobacteria	Gammaproteobacteria	Enterobacteriales	Enterobacteriaceae	Erwinia	0.045078	0.041411	0.044495	0.052432	0.03989	0.041493	0.048411	0.041946	0.048469	0.056467	0.0862	0.043915
Bacteria	Firmicutes	Erysipelotrichi	Erysipelotrichales	Erysipelotrichaceae	Erysipelothrix	0.00051	0.005647	0.003178	0.004195	0.003088	0.003458	0.003564	0.003478	0.001675	0.000974	0.000663	0.00285
Bacteria	Proteobacteria	Alphaproteobacteria	Sphingomonadales	Erythrobacteraceae	Erythrobacter	0.381714	0.302109	0.364434	0.391145	0.164193	0.202088	0.256904	0.316537	0.281604	0.323228	0.076201	0.180439
Bacteria	Proteobacteria	Gammaproteobacteria	Enterobacteriales	Enterobacteriaceae	Escherichia	2.505639	0.210818	0.218237	0.214973	0.162391	0.17385	0.256607	0.206251	0.132104	0.147984	0.236137	0.170127
Bacteria	Firmicutes	Clostridia	Clostridiales	Ruminococcaceae	Ethanoligenens	0.017351	0.031058	0.021188	0.032508	0.021618	0.017097	0.018711	0.015551	0.01135	0.009736	0.001896	0.020525
Bacteria	Firmicutes	Clostridia	Clostridiales	Eubacteriaceae	Eubacterium	0.063109	0.159054	0.14196	0.142616	0.129964	0.115836	0.124146	0.095759	0.058609	0.077886	0.014122	0.110859
Bacteria	Firmicutes	Bacilli	Bacillales	unclassified (derived from Bacillales)	Exiguobacterium	0.041335	0.042352	0.03496	0.039849	0.037317	0.038612	0.040986	0.036626	0.037119	0.021419	0.005544	0.033604
Bacteria	Firmicutes	Clostridia	Clostridiales	Ruminococcaceae	Faecalibacterium	0.009016	0.020705	0.019069	0.01573	0.015184	0.017481	0.020196	0.019234	0.006326	0.009736	0.001137	0.012107
Bacteria	Proteobacteria	Gammaproteobacteria	Alteromonadales	Ferrimonadaceae	Ferrimonas	0.054433	0.052704	0.07098	0.081794	0.076692	0.086445	0.093555	0.105581	0.096566	0.085675	0.237037	0.098653
Bacteria	Thermotogae	Thermotogae (class)	Thermotogales	Thermotogaceae	Fervidobacterium	0.060387	0.069645	0.055089	0.060822	0.061251	0.060511	0.054945	0.041946	0.025397	0.015577	0.002559	0.042136
Bacteria	Fibrobacteres	Fibrobacteres (class)	Fibrobacterales	Fibrobacteraceae	Fibrobacter	0.035722	0.045175	0.033901	0.045092	0.04375	0.050714	0.035343	0.049107	0.023537	0.012657	0.003459	0.034361
Bacteria	Firmicutes	Clostridia	Clostridiales	Peptostreptococcaceae	Filifactor	0.003572	0.00847	0.005297	0.006292	0.005919	0.003842	0.005049	0.005525	0.004838	0.002921	0.000995	0.007627
Bacteria	Firmicutes	Clostridia	Clostridiales	Clostridiales Family XI. Incertae Sedis	Finegoldia	0.004763	0.018823	0.012713	0.017827	0.021618	0.018826	0.018117	0.018211	0.010792	0.024339	0.00218	0.018301
Bacteria	Bacteroidetes	Flavobacteria	Flavobacteriales	Flavobacteriaceae	Flavobacterium	0.370828	0.592924	0.590086	0.585145	0.231877	0.37248	0.370358	0.640031	0.674101	3.984851	0.343898	2.567933
Bacteria	Proteobacteria	Gammaproteobacteria	Legionellales	Legionellaceae	Fluoribacter	0	0	0.001059	0	0.000257	0.000192	0.000297	0	0	0	9.48E-05	0.000148
Bacteria	Proteobacteria	Gammaproteobacteria	Thiotrichales	Francisellaceae	Francisella	0.193919	0.069645	0.075217	0.083892	0.079523	0.095089	0.152658	0.112537	0.05647	0.089569	0.047057	0.09045
Bacteria	Actinobacteria	Actinobacteria (class)	Actinomycetales	Frankiaceae	Frankia	0.332214	0.427282	0.569958	0.397437	0.23059	0.202088	0.244431	0.199703	0.316584	0.175244	0.028338	0.188609
Bacteria	Proteobacteria	Alphaproteobacteria	Rhizobiales	Aurantimonadaceae	Fulvimarina	0.029938	0.023529	0.037079	0.035654	0.046839	0.037651	0.06237	0.068341	0.074053	0.032128	0.025353	0.02036
Bacteria	Fusobacteria	Fusobacteria (class)	Fusobacteriales	Fusobacteriaceae	Fusobacterium	0.116862	0.153407	0.120772	0.126886	0.111435	0.115644	0.121176	0.099442	0.0614	0.073992	0.019192	0.104682
Bacteria	Proteobacteria	Betaproteobacteria	Gallionellales	Gallionellaceae	Gallionella	0.116351	0.098821	0.154672	0.113254	0.069743	0.0705	0.061479	0.048698	0.074983	0.132406	0.057245	0.088292
Bacteria	Actinobacteria	Actinobacteria (class)	Bifidobacteriales	Bifidobacteriaceae	Gardnerella	0.004083	0.007529	0.002119	0.007341	0.005919	0.004995	0.005346	0.00266	0.003628	0.005841	0.000616	0.003822
Bacteria	Firmicutes	Bacilli	Bacillales	unclassified (derived from Bacillales)	Gemella	0.000851	0.001882	0.004238	0.005243	0.00386	0.005187	0.00297	0.005115	0.002977	0.004868	0.000569	0.003542
Bacteria	Planctomycetes	Planctomycetacia	Planctomycetales	Planctomycetaceae	Gemmata	0.060047	0.162819	0.186455	0.142616	0.158273	0.149645	0.140778	0.118881	0.182619	0.082754	0.010236	0.087996
Bacteria	Gemmatimonadetes	Gemmatimonadetes (class)	Gemmatimonadales	Gemmatimonadaceae	Gemmatimonas	0.12979	0.133643	0.169504	0.146811	0.195333	0.173273	0.180279	0.205432	0.236391	0.112935	0.015259	0.102014
Bacteria	Firmicutes	Bacilli	Bacillales	Bacillaceae	Geobacillus	0.348204	0.387754	0.378206	0.361783	0.265848	0.24435	0.290168	0.202977	0.199086	0.165508	0.042934	0.213417
Bacteria	Proteobacteria	Deltaproteobacteria	Desulfuromonadales	Geobacteraceae	Geobacter	2.522139	2.683218	2.669689	2.751649	2.75962	2.395474	1.928714	1.134583	0.986871	1.041728	0.305609	1.818158
Bacteria	Actinobacteria	Actinobacteria (class)	Actinomycetales	Geodermatophilaceae	Geodermatophilus	0.050011	0.066822	0.076277	0.063967	0.042978	0.037843	0.040392	0.032124	0.050144	0.039917	0.019098	0.029041
Bacteria	Proteobacteria	Gammaproteobacteria	Alteromonadales	Alteromonadaceae	Glaciecola	0	0	0	0	0	0.000768	0.001188	0.001432	0.001116	0.000974	0.000616	0.000675
Bacteria	Cyanobacteria	Gloeobacteria	Gloeobacterales	unclassified (derived from Gloeobacterales)	Gloeobacter	0.117882	0.143996	0.169504	0.189805	0.157501	0.143306	0.141966	0.118881	0.174619	0.049652	0.009193	0.09923
Bacteria	Proteobacteria	Alphaproteobacteria	Rhodospirillales	Acetobacteraceae	Gluconacetobacter	0.060047	0.056469	0.050851	0.065016	0.101913	0.087981	0.15444	0.136887	0.093496	0.063283	0.008103	0.032385
Bacteria	Proteobacteria	Alphaproteobacteria	Rhodospirillales	Acetobacteraceae	Gluconobacter	0.050861	0.043293	0.057208	0.037751	0.047611	0.05494	0.08613	0.088598	0.061772	0.022392	0.003744	0.023473
Bacteria	Actinobacteria	Actinobacteria (class)	Actinomycetales	Gordoniaceae	Gordonia	0.019222	0.040469	0.026485	0.027265	0.013125	0.011526	0.012177	0.0133	0.018885	0.023366	0.003838	0.012173
Bacteria	Bacteroidetes	Flavobacteria	Flavobacteriales	Flavobacteriaceae	Gramella	0.254306	0.396224	0.401513	0.447772	0.239855	0.384966	0.334718	0.74152	1.153675	3.051191	0.588328	1.790172
Bacteria	Proteobacteria	Alphaproteobacteria	Rhodospirillales	Acetobacteraceae	Granulibacter	0.046268	0.053646	0.061445	0.056627	0.090846	0.107191	0.149094	0.154074	0.114056	0.06523	0.019477	0.036948
Bacteria	Firmicutes	Bacilli	Lactobacillales	Carnobacteriaceae	Granulicatella	0.005954	0.011294	0.008475	0.008389	0.007463	0.007108	0.006534	0.006957	0.005396	0.005841	0.001706	0.005205
Bacteria	Proteobacteria	Gammaproteobacteria	Pasteurellales	Pasteurellaceae	Haemophilus	0.166192	0.09035	0.130306	0.1164	0.076692	0.083755	0.116721	0.094327	0.080844	0.097358	0.117429	0.107038
Bacteria	Proteobacteria	Gammaproteobacteria	Oceanospirillales	Hahellaceae	Hahella	0.431045	0.287992	0.330533	0.350248	0.490776	0.470066	0.574991	0.634711	0.555301	0.349514	2.394447	0.56983
Bacteria	Firmicutes	Clostridia	Halanaerobiales	Halanaerobiaceae	Halanaerobium	0.017861	0.05741	0.039198	0.053481	0.0525	0.046104	0.051084	0.051153	0.024839	0.032128	0.003412	0.038035
Bacteria	Proteobacteria	Deltaproteobacteria	Myxococcales	Haliangiaceae	Haliangium	0.333745	0.314344	0.360196	0.303059	0.371364	0.327913	0.359963	0.435622	0.532043	0.129486	0.03284	0.240629
Bacteria	Proteobacteria	Gammaproteobacteria	Oceanospirillales	Halomonadaceae	Halomonas	0.047459	0.05741	0.06992	0.065016	0.120185	0.106615	0.150876	0.205841	0.384775	0.157719	0.790962	0.298315
Bacteria	Proteobacteria	Gammaproteobacteria	Chromatiales	Ectothiorhodospiraceae	Halorhodospira	0.294621	0.185407	0.261672	0.238043	0.211031	0.222451	0.247104	0.237352	0.174619	0.082754	0.094019	0.212329
Bacteria	Firmicutes	Clostridia	Halanaerobiales	Halanaerobiaceae	Halothermothrix	0.076037	0.132702	0.103821	0.138421	0.137942	0.115836	0.119691	0.108854	0.057865	0.046732	0.007866	0.090499
Bacteria	Proteobacteria	Gammaproteobacteria	Chromatiales	Halothiobacillaceae	Halothiobacillus	0.126728	0.070586	0.082633	0.0776	0.07103	0.067811	0.060885	0.065886	0.059354	0.074965	0.095677	0.102409
Bacteria	Proteobacteria	Epsilonproteobacteria	Campylobacterales	Helicobacteraceae	Helicobacter	0.082671	0.117644	0.112296	0.08494	0.064854	0.077992	0.106029	0.073252	0.037212	0.059388	0.030044	0.223119
Bacteria	Firmicutes	Clostridia	Clostridiales	Heliobacteriaceae	Heliobacterium	0.090836	0.136467	0.118653	0.115351	0.112464	0.092208	0.087615	0.057087	0.045492	0.04089	0.00853	0.070518
Bacteria	Proteobacteria	Betaproteobacteria	Burkholderiales	Oxalobacteraceae	Herbaspirillum	0.045928	0.050822	0.073099	0.056627	0.051728	0.042838	0.048708	0.043378	0.072192	0.090543	0.037674	0.045151
Bacteria	Proteobacteria	Betaproteobacteria	Burkholderiales	Oxalobacteraceae	Herminiimonas	0.082841	0.066822	0.132425	0.121643	0.068714	0.066658	0.076626	0.058929	0.088658	0.07886	0.051511	0.075477
Bacteria	Chloroflexi	Chloroflexi (class)	Herpetosiphonales	Herpetosiphonaceae	Herpetosiphon	0.160068	0.262581	0.191752	0.210778	0.228531	0.145419	0.138402	0.123791	0.166339	0.089569	0.01488	0.162912
Bacteria	Proteobacteria	Alphaproteobacteria	Rhodobacterales	Hyphomonadaceae	Hirschia	0.03249	0.034823	0.046614	0.037751	0.041692	0.048409	0.064746	0.110491	0.060005	0.024339	0.020567	0.028547
Bacteria	Proteobacteria	Gammaproteobacteria	Pasteurellales	Pasteurellaceae	Histophilus	0.031299	0.025411	0.030723	0.026216	0.023677	0.024781	0.031482	0.033557	0.019723	0.024339	0.031039	0.030441
Bacteria	Proteobacteria	Alphaproteobacteria	Rhizobiales	Phyllobacteriaceae	Hoeflea	0.041676	0.050822	0.043435	0.060822	0.100369	0.082795	0.105435	0.124814	0.115172	0.07886	0.059473	0.091389
Bacteria	Firmicutes	Erysipelotrichi	Erysipelotrichales	Erysipelotrichaceae	Holdemania	0.007144	0.016941	0.015891	0.014681	0.014412	0.018634	0.019008	0.010845	0.009024	0.011683	0.002275	0.013277
Bacteria	Aquificae	Aquificae (class)	Aquificales	Aquificaceae	Hydrogenivirga	0.031639	0.039528	0.031782	0.051384	0.045295	0.039764	0.043659	0.028851	0.023351	0.026287	0.004691	0.037952
Bacteria	Aquificae	Aquificae (class)	Aquificales	Aquificaceae	Hydrogenobacter	0.021603	0.016941	0.032841	0.037751	0.028052	0.02017	0.028215	0.024963	0.016839	0.010709	0.003554	0.020146
Bacteria	Aquificae	Aquificae (class)	Aquificales	Aquificaceae	Hydrogenobaculum	0.017861	0.030117	0.020129	0.022022	0.027794	0.015368	0.021978	0.018211	0.011722	0.009736	0.001422	0.019602
Bacteria	Proteobacteria	Alphaproteobacteria	Rhizobiales	Hyphomicrobiaceae	Hyphomicrobium	0.055624	0.063998	0.08899	0.072357	0.096766	0.068003	0.097119	0.092485	0.075634	0.057441	0.046109	0.033357
Bacteria	Proteobacteria	Alphaproteobacteria	Rhodobacterales	Hyphomonadaceae	Hyphomonas	0.08148	0.115761	0.112296	0.120594	0.114266	0.129091	0.164538	0.298122	0.157594	0.042837	0.040944	0.069678

Bacteria	Proteobacteria	Gammaproteobacteria	Alteromonadales	Idiomarinaceae	Idiomarina	0.170785	0.113879	0.148316	0.149956	0.159818	0.165205	0.215622	1.035754	5.405275	0.207372	0.341103	0.189301
Bacteria	Fusobacteria	Fusobacteria (class)	Fusobacteriales	Fusobacteriaceae	Ilyobacter	0.024155	0.044234	0.033901	0.039849	0.059706	0.056669	0.049896	0.037649	0.024467	0.025313	0.005023	0.042466
Bacteria	Actinobacteria	Actinobacteria (class)	Actinomycetales	Intrasporangiaceae	Intrasporangium	0.021263	0.060234	0.075217	0.040897	0.021618	0.015752	0.018414	0.018824	0.030886	0.02726	0.003649	0.020558
Bacteria	Actinobacteria	Actinobacteria (class)	Actinomycetales	Intrasporangiaceae	Janibacter	0.040315	0.066822	0.072039	0.070259	0.0175	0.013831	0.015147	0.014732	0.026793	0.032128	0.002843	0.019404
Bacteria	Proteobacteria	Alphaproteobacteria	Rhodobacterales	Rhodobacteraceae	Jannaschia	0.147991	0.134584	0.17692	0.192951	0.178347	0.304861	0.249777	0.472044	0.473527	0.138248	0.066297	0.10287
Bacteria	Proteobacteria	Betaproteobacteria	Burkholderiales	Oxalobacteraceae	Janthinobacterium	0.153434	0.110115	0.136663	0.130032	0.096251	0.090287	0.103356	0.102511	0.106985	0.124618	0.036963	0.081489
Bacteria	Actinobacteria	Actinobacteria (class)	Actinomycetales	Jonesiaceae	Jonesia	0.014799	0.022588	0.020129	0.02307	0.00875	0.0073	0.013662	0.011868	0.01014	0.006815	0.001564	0.007544
Bacteria	Synergistetes	Synergistia	Synergistales	Synergistaceae	Jonquetella	0.009866	0.013176	0.013772	0.011535	0.012868	0.016713	0.014256	0.010231	0.005954	0.005841	0.000663	0.006754
Bacteria	Proteobacteria	Gammaproteobacteria	Oceanospirillales	Alcanivoracaceae	Kangiella	0.139656	0.101644	0.091108	0.1164	0.141545	0.142922	0.16335	0.201135	0.142802	0.13338	0.288928	0.154182
Bacteria	Proteobacteria	Alphaproteobacteria	Rhodobacterales	Rhodobacteraceae	Ketogulonicigenium	0.021603	0.026352	0.027544	0.035654	0.034228	0.046296	0.051678	0.086961	0.076843	0.056467	0.009857	0.025368
Bacteria	Actinobacteria	Actinobacteria (class)	Actinomycetales	Kineosporiaceae	Kineococcus	0.051201	0.102585	0.097465	0.081794	0.032169	0.032081	0.033858	0.032329	0.046515	0.026287	0.004502	0.029601
Bacteria	Proteobacteria	Betaproteobacteria	Neisseriales	Neisseriaceae	Kingella	0.011227	0.006588	0.005297	0.017827	0.009265	0.009221	0.010989	0.020871	0.008094	0.004868	0.004786	0.008632
Bacteria	Proteobacteria	Gammaproteobacteria	Enterobacteriales	Enterobacteriaceae	Klebsiella	0.14697	0.086586	0.113356	0.120594	0.065111	0.080105	0.112266	0.075502	0.066982	0.098331	0.100369	0.074917
Bacteria	Actinobacteria	Actinobacteria (class)	Actinomycetales	Micrococcaceae	Kocuria	0.005613	0.013176	0.01695	0.012584	0.009007	0.009221	0.007425	0.00798	0.017583	0.005841	0.001943	0.005979
Bacteria	Bacteroidetes	Flavobacteria	Flavobacteriales	Flavobacteriaceae	Kordia	0.067021	0.070586	0.060386	0.058724	0.044008	0.080105	0.078408	0.141183	0.151175	2.047433	0.270399	0.595774
Bacteria	Thermotogae	Thermotogae (class)	Thermotogales	Thermotogaceae	Kosmotoga	0.03334	0.053646	0.031782	0.057676	0.056361	0.048601	0.036531	0.028237	0.017025	0.014604	0.003838	0.037574
Bacteria	Actinobacteria	Actinobacteria (class)	Actinomycetales	Nocardioidaceae	Kribbella	0.064129	0.093174	0.100643	0.075503	0.033199	0.033617	0.038016	0.032124	0.048934	0.02726	0.00744	0.030326
Bacteria	Chloroflexi	Ktedonobacteria	Ktedonobacterales	Ktedonobacteraceae	Ktedonobacter	0.105295	0.225876	0.154672	0.144713	0.177575	0.139464	0.11583	0.083073	0.137779	0.103199	0.013458	0.123181
Bacteria	Actinobacteria	Actinobacteria (class)	Actinomycetales	Dermacoccaceae	Kytococcus	0.021943	0.021646	0.039198	0.020973	0.011581	0.009605	0.015147	0.012891	0.015164	0.009736	0.002796	0.010262
Bacteria	Proteobacteria	Alphaproteobacteria	Rhodobacterales	Rhodobacteraceae	Labrenzia	0.137104	0.124232	0.150435	0.160443	0.319893	0.348852	0.434213	0.408204	0.300582	0.167455	0.076106	0.110563
Bacteria	Firmicutes	Bacilli	Lactobacillales	Lactobacillaceae	Lactobacillus	0.19528	0.231523	0.198108	0.189805	0.158788	0.14734	0.194535	0.146913	0.100752	0.098331	0.019951	0.119293
Bacteria	Firmicutes	Bacilli	Lactobacillales	Streptococcaceae	Lactococcus	0.022454	0.028234	0.025426	0.024119	0.019559	0.01921	0.031185	0.025986	0.017397	0.018498	0.006871	0.019108
Bacteria	Proteobacteria	Betaproteobacteria	Neisseriales	Neisseriaceae	Laribacter	0.06481	0.047999	0.06992	0.071308	0.051214	0.049562	0.060291	0.058315	0.054981	0.060362	0.052317	0.051394
Bacteria	Proteobacteria	Deltaproteobacteria	Desulfovibrionales	Desulfovibrionaceae	Lawsonia	0.038103	0.058351	0.061445	0.041946	0.045037	0.04572	0.045441	0.030487	0.016001	0.016551	0.002417	0.032928
Bacteria	Bacteroidetes	Cytophagia	Cytophagales	Cytophagaceae	Leadbetterella	0.060047	0.095997	0.083693	0.098573	0.065111	0.06685	0.079002	0.128293	0.138523	0.131433	0.031892	0.119853
Bacteria	Bacteroidetes	Flavobacteria	Flavobacteriales	Flavobacteriaceae	Leeuwenhoekiella	0.108697	0.160937	0.14196	0.153102	0.075405	0.116604	0.120285	0.218732	0.304118	1.535331	0.25206	0.568825
Bacteria	Proteobacteria	Gammaproteobacteria	Legionellales	Legionellaceae	Legionella	0.468128	0.213641	0.284979	0.26321	0.2939	0.237819	0.343925	0.32063	0.195458	0.126565	0.157282	0.234666
Bacteria	Actinobacteria	Actinobacteria (class)	Actinomycetales	Microbacteriaceae	Leifsonia	0.020072	0.036705	0.039198	0.025168	0.015441	0.008644	0.013662	0.009412	0.013769	0.018498	0.001043	0.010328
Bacteria	Lentisphaerae	unclassified (derived from Lentisphaerae)	Lentisphaerales	Lentisphaeraceae	Lentisphaera	0.08131	0.116703	0.104881	0.105913	0.135884	0.174234	0.137511	0.161849	0.075913	0.053547	0.015875	0.074917
Bacteria	Spirochaetes	Spirochaetes (class)	Spirochaetales	Leptospiraceae	Leptospira	0.199192	0.215523	0.201286	0.214973	0.198164	0.188065	0.243837	0.19602	0.170525	0.101252	0.048478	0.157657
Bacteria	Nitrospirae	Nitrospira (class)	Nitrospirales	Nitrospiraceae	Leptospirillum	0	0	0	0.001049	0	0.000576	0	0	9.3E-05	0.000974	0	0.000115
Bacteria	Proteobacteria	Betaproteobacteria	Burkholderiales	unclassified (derived from Burkholderiales)	Leptothrix	0.103934	0.112938	0.132425	0.169881	0.070515	0.071077	0.065637	0.058315	0.096938	0.102226	0.131029	0.085245
Bacteria	Fusobacteria	Fusobacteria (class)	Fusobacteriales	Fusobacteriaceae	Leptotrichia	0.012077	0.028234	0.01695	0.037751	0.02625	0.027278	0.020196	0.025577	0.016094	0.018498	0.003744	0.023819
Bacteria	Firmicutes	Bacilli	Lactobacillales	Leuconostocaceae	Leuconostoc	0.013098	0.015058	0.010594	0.017827	0.017243	0.013255	0.012771	0.014937	0.011722	0.012657	0.002227	0.011267
Bacteria	Proteobacteria	Betaproteobacteria	Burkholderiales	Burkholderiaceae	Limnobacter	0.034871	0.037646	0.044495	0.050335	0.031912	0.027086	0.03564	0.033557	0.040747	0.059388	0.049426	0.031512
Bacteria	Firmicutes	Bacilli	Bacillales	Listeriaceae	Listeria	0.181501	0.113879	0.086871	0.117448	0.060221	0.063777	0.091179	0.065272	0.052748	0.045758	0.010189	0.064407
Bacteria	Proteobacteria	Gammaproteobacteria	Vibrionales	Vibrionaceae	Listonella	0.00068	0	0.001059	0	0	0.000192	0	0.000409	0	0	0	8.24E-05
Bacteria	Proteobacteria	Alphaproteobacteria	Rhodobacterales	Rhodobacteraceae	Loktanella	0.047969	0.036705	0.058267	0.055578	0.050956	0.072037	0.084348	0.137705	0.141686	0.060362	0.049095	0.037788
Bacteria	Proteobacteria	Betaproteobacteria	Neisseriales	Neisseriaceae	Lutiella	0.06464	0.04894	0.064623	0.065016	0.06228	0.05494	0.056133	0.051153	0.074611	0.094437	0.107477	0.063468
Bacteria	Cyanobacteria	unclassified (derived from Cyanobacteria)	Oscillatoriales	unclassified (derived from Oscillatoriales)	Lyngbya	0.047969	0.059292	0.060386	0.051384	0.086471	0.081258	0.07425	0.062407	0.121777	0.032128	0.026016	0.046222
Bacteria	Firmicutes	Bacilli	Bacillales	Bacillaceae	Lysinibacillus	0.01701	0.030117	0.025426	0.022022	0.02625	0.025549	0.031482	0.0266	0.017118	0.018498	0.007582	0.023341
Bacteria	Firmicutes	Bacilli	Bacillales	Staphylococcaceae	Macrococcus	0.004423	0.010353	0.007416	0.008389	0.006691	0.009989	0.014256	0.008594	0.008838	0.007789	0.001801	0.007083
Bacteria	Proteobacteria	unclassified (derived from Proteobacteria)	unclassified (derived from Proteobacteria)	unclassified (derived from Proteobacteria)	Magnetococcus	0.289008	0.247522	0.251078	0.256918	0.361327	0.328873	0.303236	0.253107	0.149035	0.067177	0.113116	0.224799
Bacteria	Proteobacteria	Alphaproteobacteria	Rhodospirillales	Rhodospirillaceae	Magnetospirillum	0.21127	0.27858	0.363374	0.299913	0.814529	0.994496	1.226905	1.21254	0.676055	0.246315	0.167708	0.247646
Bacteria	Proteobacteria	Gammaproteobacteria	Pasteurellales	Pasteurellaceae	Mannheimia	0.017181	0.013176	0.007416	0.010486	0.015441	0.013063	0.021978	0.016983	0.010326	0.007789	0.007203	0.016143
Bacteria	Bacteroidetes	Flavobacteria	Flavobacteriales	Flavobacteriaceae	Maribacter	0.230492	0.256934	0.226712	0.268454	0.156472	0.313313	0.223641	0.466314	0.357052	2.74062	0.331388	1.293035
Bacteria	Proteobacteria	Alphaproteobacteria	Rhodobacterales	Hyphomonadaceae	Maricaulis	0.116692	0.068704	0.12395	0.099621	0.144119	0.197286	0.226017	0.576192	0.170432	0.063283	0.013932	0.06752
Bacteria	Proteobacteria	Gammaproteobacteria	Alteromonadales	Alteromonadaceae	Marinobacter	0.489561	0.369872	0.468255	0.445675	0.545593	0.569958	0.764773	1.417972	6.682122	1.397083	3.457039	1.064579
Bacteria	Proteobacteria	Gammaproteobacteria	Oceanospirillales	Oceanospirillaceae	Marinomonas	0.226069	0.157172	0.217177	0.206583	0.273826	0.252802	0.305909	0.354595	0.306722	0.685398	4.787614	0.560457
Bacteria	Proteobacteria	Zetaproteobacteria	Mariprofundales	Mariprofundaceae	Mariprofundus	0.176739	0.082821	0.103821	0.102767	0.134339	0.131011	0.174636	0.094327	0.085495	0.177191	0.044356	0.12066
Bacteria	Proteobacteria	Alphaproteobacteria	Rhodobacterales	Rhodobacteraceae	Maritimibacter	0.100192	0.092233	0.103821	0.108011	0.168053	0.180765	0.184734	0.430712	0.255649	0.145063	0.069092	0.09157
Bacteria	Bacteroidetes	Cytophagia	Cytophagales	Flammeovirgaceae	Marivirga	0.054604	0.149643	0.135603	0.119546	0.162391	0.245695	0.230769	0.495165	0.509809	0.289152	0.092076	0.340748
Bacteria	Firmicutes	Negativicutes	Selenomonadales	Veillonellaceae	Megasphaera	0.014969	0.017882	0.019069	0.01573	0.023934	0.014984	0.021087	0.011254	0.007256	0.012657	0.002322	0.012931
Bacteria	Deinococcus-Thermus	Deinococci	Thermales	Thermaceae	Meiothermus	0.138975	0.133643	0.144078	0.144713	0.134854	0.122943	0.116721	0.118676	0.134523	0.057441	0.010331	0.094173
Bacteria	Tenericutes	Mollicutes	Entomoplasmatales	Entomoplasmataceae	Mesoplasma	0	0.002823	0	0.002097	0.001801	0.001537	0.003861	0.004092	0.00093	0.000974	0.000379	0.001697
Bacteria	Proteobacteria	Alphaproteobacteria	Rhizobiales	Phyllobacteriaceae	Mesorhizobium	0.271657	0.299286	0.364434	0.376464	0.357209	0.34136	0.425303	0.434804	0.477992	0.276496	0.081129	0.169913
Bacteria	Verrucomicrobia	unclassified (derived from Verrucomicrobia)	Methylacidiphilales	Methylacidiphilaceae	Methylacidiphilum	0.053413	0.068704	0.066742	0.083892	0.04864	0.047641	0.039798	0.046243	0.060377	0.060362	0.003222	0.047721
Bacteria	Proteobacteria	Betaproteobacteria	Burkholderiales	unclassified (derived from Burkholderiales)	Methylibium	0.132852	0.142114	0.202346	0.208681	0.094449	0.087597	0.110484	0.094327	0.154431	0.110014	0.237796	0.097714
Bacteria	Proteobacteria	Betaproteobacteria	Methylophilales	Methylophilaceae	Methylobacillus	0.197661	0.13929	0.21188	0.200292	0.135369	0.116796	0.120879	0.094736	0.116195	0.109041	0.069045	0.134794
Bacteria	Proteobacteria	Gammaproteobacteria	Methylococcales	Methylococcaceae	Methylobacter	0.108527	0.135526	0.174801	0.167783	0.242686	0.203625	0.156519	0.138933	0.119358	0.124618	0.165433	0.255701
Bacteria	Proteobacteria	Alphaproteobacteria	Rhizobiales	Methylobacteriaceae	Methylobacterium	0.47289	0.398106	0.506393	0.403729	0.436732	0.437986	0.596375	0.533428	0.609259	0.370933	0.109515	0.21167
Bacteria	Proteobacteria	Alphaproteobacteria	Rhizobiales	Beijerinckiaceae	Methylocella	0.068212	0.062116	0.080514	0.0776	0.070001	0.063585	0.065934	0.080209	0.09424	0.059388	0.049947	0.03848
Bacteria	Proteobacteria	Gammaproteobacteria	Methylococcales	Methylococcaceae	Methylococcus	0.520009	0.373637	0.47673	0.450918	0.483828	0.441251	0.36026	0.317151	0.285232	0.130459	0.20813	0.426767
Bacteria	Proteobacteria	Gammaproteobacteria	Thiotrichales	Piscirickettsiaceae	Methylophaga	0.110568	0.088468	0.108059	0.09333	0.21798	0.159634	0.18414	0.199294	0.080379	0.181085	0.183488	0.198229
Bacteria	Proteobacteria	Alphaproteobacteria	Rhizobiales	Methylocystaceae	Methylosinus	0.03249	0.05741	0.041317	0.051384	0.047353	0.044759	0.065637	0.05811	0.055818	0.053547	0.008103	0.022699
Bacteria	Proteobacteria	Betaproteobacteria	Methylophilales	Methylophilaceae	Methylotenera	0.144078	0.10635	0.121831	0.148908	0.084155	0.064353	0.079893	0.057087	0.080379	0.135327	0.10198	0.104863

Bacteria	Proteobacteria	Betaproteobacteria	Methylophilales	Methylophilaceae	Methylovorus	0.109547	0.070586	0.115475	0.106962	0.065883	0.063585	0.07722	0.050335	0.065215	0.116829	0.044166	0.082691
Bacteria	Actinobacteria	Actinobacteria (class)	Actinomycetales	Micrococcaceae	Micrococcus	0.0165	0.02447	0.032841	0.020973	0.009779	0.008644	0.013959	0.008389	0.014513	0.004868	0.001516	0.006918
Bacteria	Cyanobacteria	unclassified (derived from Cyanobacteria)	Oscillatoriales	unclassified (derived from Oscillatoriales)	Microcoleus	0.063279	0.079998	0.084752	0.080746	0.121472	0.122175	0.105138	0.098419	0.189969	0.047705	0.030281	0.065527
Bacteria	Cyanobacteria	unclassified (derived from Cyanobacteria)	Chroococcales	unclassified (derived from Chroococcales)	Microcystis	0.040315	0.067763	0.054029	0.075503	0.075148	0.071653	0.071577	0.069364	0.09545	0.020445	0.026253	0.04352
Bacteria	Actinobacteria	Actinobacteria (class)	Actinomycetales	Micromonosporaceae	Micromonospora	0.079609	0.096938	0.106999	0.078649	0.048897	0.042262	0.062073	0.042355	0.063633	0.043811	0.010283	0.037541
Bacteria	Bacteroidetes	Cytophagia	Cytophagales	Cytophagaceae	Microscilla	0.087264	0.118585	0.127128	0.1164	0.154413	0.192099	0.196614	0.324313	0.293884	0.250209	0.052412	0.234551
Bacteria	Firmicutes	Negativicutes	Selenomonadales	Veillonellaceae	Mitsuokella	0.014969	0.022588	0.012713	0.019924	0.015184	0.014023	0.013662	0.010231	0.007535	0.014604	0.001564	0.012025
Bacteria	Actinobacteria	Actinobacteria (class)	Actinomycetales	Actinomycetaceae	Mobiluncus	0.006294	0.02447	0.022247	0.01573	0.014669	0.014215	0.013662	0.009003	0.008745	0.009736	0.002132	0.009933
Bacteria	Firmicutes	Clostridia	Thermoanaerobacterales	Thermoanaerobacteraceae	Moorella	0.256688	0.36799	0.262731	0.331372	0.286179	0.243005	0.18117	0.108036	0.090891	0.063283	0.008246	0.160309
Bacteria	Proteobacteria	Gammaproteobacteria	Pseudomonadales	Moraxellaceae	Moraxella	0.009016	0.00847	0.009535	0.011535	0.007206	0.008452	0.012771	0.013504	0.010792	0.015577	0.022746	0.012865
Bacteria	Proteobacteria	Gammaproteobacteria	Alteromonadales	Moritellaceae	Moritella	0.022454	0.018823	0.026485	0.026216	0.064854	0.054556	0.047817	0.050744	0.040282	0.048679	0.176901	0.06416
Bacteria	Bacteroidetes	Sphingobacteria	Sphingobacteriales	Sphingobacteriaceae	Mucilaginibacter	0.04899	0.109173	0.074158	0.079697	0.061508	0.07319	0.072765	0.120313	0.125499	0.162587	0.035731	0.140724
Bacteria	Actinobacteria	Actinobacteria (class)	Actinomycetales	Mycobacteriaceae	Mycobacterium	1.72622	0.986325	1.193944	0.795923	0.372908	0.320997	0.400949	0.330656	0.529996	0.414744	0.085157	0.382753
Bacteria	Tenericutes	Mollicutes	Mycoplasmatales	Mycoplasmataceae	Mycoplasma	0.011057	0.023529	0.023307	0.016778	0.025993	0.021899	0.032967	0.027623	0.014513	0.012657	0.002559	0.017955
Bacteria	Proteobacteria	Deltaproteobacteria	Myxococcales	Myxococcaceae	Myxococcus	0.364704	0.487516	0.510631	0.456161	0.433644	0.369022	0.395306	0.418844	0.413243	0.151878	0.027912	0.275205
Bacteria	Actinobacteria	Actinobacteria (class)	Actinomycetales	Nakamurellaceae	Nakamurella	0.04882	0.079057	0.097465	0.06187	0.037059	0.025549	0.035046	0.02926	0.043073	0.017524	0.009004	0.025895
Bacteria	Firmicutes	Clostridia	Natranaerobiales	Natranaerobiaceae	Natranaerobius	0.070423	0.093174	0.061445	0.066065	0.071287	0.06685	0.050193	0.047061	0.032561	0.030181	0.003886	0.053931
Bacteria	Proteobacteria	Epsilonproteobacteria	Nautiliales	Nautiliaceae	Nautilia	0.017861	0.028234	0.031782	0.028313	0.017758	0.018634	0.021087	0.018415	0.009117	0.019472	0.003838	0.061689
Bacteria	Proteobacteria	Betaproteobacteria	Neisseriales	Neisseriaceae	Neisseria	0.252775	0.159054	0.189633	0.183513	0.085442	0.083371	0.129195	0.115197	0.08931	0.126565	0.106908	0.115488
Bacteria	Proteobacteria	Alphaproteobacteria	Rickettsiales	Anaplasmataceae	Neorickettsia	0.009016	0.004706	0.004238	0.005243	0.005662	0.009605	0.008316	0.008389	0.00521	0.000974	0.000379	0.003673
Bacteria	Proteobacteria	Gammaproteobacteria	Oceanospirillales	Oceanospirillaceae	Neptuniibacter	0.088454	0.06588	0.074158	0.076551	0.159046	0.149453	0.1782	0.194383	0.136662	0.757443	16.91102	0.58029
Bacteria	Proteobacteria	Epsilonproteobacteria	unclassified (derived from Epsilonproteobacteria)	unclassified (derived from Epsilonproteobacteria)	Nitratiruptor	0.057325	0.063057	0.063564	0.060822	0.059192	0.055132	0.05346	0.049107	0.025211	0.034075	0.006634	0.22979
Bacteria	Proteobacteria	Alphaproteobacteria	Rhizobiales	Bradyrhizobiaceae	Nitrobacter	0.330853	0.17976	0.328414	0.219167	0.193274	0.182878	0.255122	0.237556	0.25202	0.222949	0.028433	0.107482
Bacteria	Proteobacteria	Gammaproteobacteria	Chromatiales	Ectothiorhodospiraceae	Nitrococcus	0.221136	0.206112	0.241543	0.212875	0.24912	0.234361	0.215325	0.197248	0.210157	0.084701	0.132593	0.200947
Bacteria	Proteobacteria	Gammaproteobacteria	Chromatiales	Chromatiaceae	Nitrosococcus	1.472084	0.841388	1.039272	1.033966	1.382512	1.146255	1.106917	0.8604	0.797274	0.499445	0.482368	1.051664
Bacteria	Proteobacteria	Betaproteobacteria	Nitrosomonadales	Nitrosomonadaceae	Nitrosomonas	0.40706	0.313403	0.447067	0.415264	0.314745	0.248384	0.264329	0.216072	0.26765	0.261892	0.16169	0.284429
Bacteria	Proteobacteria	Betaproteobacteria	Nitrosomonadales	Nitrosomonadaceae	Nitrosospira	0.26094	0.174113	0.259553	0.241189	0.162648	0.135046	0.147015	0.109673	0.191736	0.131433	0.048621	0.157624
Bacteria	Nitrospirae	Nitrospira (class)	Nitrospirales	Nitrospiraceae	Nitrospira	0.090325	0.178818	0.182217	0.174075	0.240627	0.165397	0.177012	0.112947	0.215552	0.16064	0.012558	0.116674
Bacteria	Actinobacteria	Actinobacteria (class)	Actinomycetales	Nocardiaceae	Nocardia	0.054604	0.083762	0.076277	0.074454	0.04375	0.038996	0.057618	0.056678	0.052097	0.035049	0.015401	0.031759
Bacteria	Actinobacteria	Actinobacteria (class)	Actinomycetales	Nocardioidaceae	Nocardioides	0.131321	0.191053	0.185395	0.130032	0.080037	0.074342	0.081081	0.062612	0.092752	0.063283	0.009999	0.061014
Bacteria	Actinobacteria	Actinobacteria (class)	Actinomycetales	Nocardiopsaceae	Nocardiopsis	0.038444	0.051763	0.048732	0.045092	0.029596	0.020747	0.025839	0.025167	0.035166	0.032128	0.003128	0.01975
Bacteria	Cyanobacteria	unclassified (derived from Cyanobacteria)	Nostocales	Nostocaceae	Nodularia	0.027557	0.033881	0.026485	0.042995	0.036544	0.041301	0.039204	0.034375	0.062331	0.020445	0.023979	0.022715
Bacteria	Cyanobacteria	unclassified (derived from Cyanobacteria)	Nostocales	Nostocaceae	Nostoc	0.188646	0.253169	0.273325	0.275794	0.2939	0.279312	0.255419	0.230804	0.514181	0.088596	0.075869	0.177144
Bacteria	Proteobacteria	Alphaproteobacteria	Sphingomonadales	Sphingomonadaceae	Novosphingobium	0.285435	0.166584	0.206583	0.209729	0.10706	0.09797	0.135729	0.161236	0.130801	0.141169	0.049189	0.084849
Bacteria	Proteobacteria	Alphaproteobacteria	Rhodobacterales	Rhodobacteraceae	Oceanibulbus	0.035212	0.031999	0.024366	0.039849	0.031397	0.043991	0.062667	0.087165	0.078425	0.058415	0.020282	0.028695
Bacteria	Proteobacteria	Alphaproteobacteria	Rhodobacterales	Hyphomonadaceae	Oceanicaulis	0.033511	0.043293	0.063564	0.04614	0.070258	0.102773	0.137808	0.368918	0.100752	0.032128	0.012368	0.03194
Bacteria	Proteobacteria	Alphaproteobacteria	Rhodobacterales	Rhodobacteraceae	Oceanicola	0.141697	0.09035	0.139841	0.112205	0.141031	0.230711	0.23463	0.412706	0.402916	0.178165	0.061747	0.100663
Bacteria	Deinococcus-Thermus	Deinococci	Thermales	Thermaceae	Oceanithermus	0.041505	0.044234	0.047673	0.035654	0.055331	0.051483	0.044253	0.040104	0.039538	0.015577	0.002512	0.035959
Bacteria	Firmicutes	Bacilli	Bacillales	Bacillaceae	Oceanobacillus	0.036402	0.074351	0.064623	0.06187	0.064081	0.061472	0.053163	0.045833	0.046515	0.026287	0.006634	0.043108
Bacteria	Proteobacteria	Alphaproteobacteria	Rhizobiales	Brucellaceae	Ochrobactrum	0.106996	0.076233	0.110178	0.091232	0.127391	0.109304	0.150282	0.154688	0.13322	0.089569	0.04137	0.051641
Bacteria	Proteobacteria	Alphaproteobacteria	Rhodobacterales	Rhodobacteraceae	Octadecabacter	0.08165	0.042352	0.068861	0.057676	0.063052	0.082026	0.090288	0.142206	0.119079	0.069124	0.027675	0.037063
Bacteria	Firmicutes	Bacilli	Lactobacillales	Leuconostocaceae	Oenococcus	0.021943	0.00847	0.004238	0.013632	0.004118	0.007492	0.009801	0.004706	0.005675	0.003894	0.000711	0.005634
Bacteria	Proteobacteria	Alphaproteobacteria	Rhizobiales	Bradyrhizobiaceae	Oligotropha	0.073145	0.055528	0.068861	0.06187	0.054559	0.060703	0.076923	0.077958	0.090519	0.072045	0.016539	0.028711
Bacteria	Actinobacteria	Actinobacteria (class)	Coriobacteriales	Coriobacteriaceae	Olsenella	0.002552	0.00847	0.009535	0.009438	0.006949	0.005571	0.007722	0.006957	0.005396	0.003894	0.000616	0.006655
Bacteria	Verrucomicrobia	Opitutae	unclassified (derived from Opitutae)	Opitutaceae	Opitutus	0.267915	0.370813	0.35278	0.347102	0.271767	0.289685	0.27116	0.498234	0.309234	0.136301	0.022841	0.245669
Bacteria	Firmicutes	Clostridia	Clostridiales	Lachnospiraceae	Oribacterium	0.008335	0.021646	0.009535	0.009438	0.010809	0.014023	0.010098	0.009208	0.005861	0.012657	0.003128	0.010921
Bacteria	Proteobacteria	Alphaproteobacteria	Rickettsiales	Rickettsiaceae	Orientia	0.005613	0.009411	0.006356	0.006292	0.00875	0.008068	0.01188	0.009003	0.006419	0.004868	0.000853	0.004448
Bacteria	Bacteroidetes	Flavobacteria	Flavobacteriales	Flavobacteriaceae	Ornithobacterium	0	0	0	0	0	0	0.000297	0.000205	0.000186	0.000974	9.48E-05	1.65E-05
Bacteria	Cyanobacteria	unclassified (derived from Cyanobacteria)	Oscillatoriales	unclassified (derived from Oscillatoriales)	Oscillatoria	0.014629	0.053646	0.056148	0.051384	0.072059	0.066658	0.052272	0.049926	0.100101	0.021419	0.027438	0.039649
Bacteria	Chloroflexi	Chloroflexi (class)	Chloroflexales	Oscillochloridaceae	Oscillochloris	0.061408	0.13929	0.133484	0.126886	0.117097	0.082987	0.075735	0.059952	0.090426	0.062309	0.008814	0.090302
Bacteria	Proteobacteria	Betaproteobacteria	Burkholderiales	Oxalobacteraceae	Oxalobacter	0.028237	0.039528	0.051911	0.04614	0.030883	0.028431	0.04158	0.028441	0.029491	0.042837	0.014643	0.027871
Bacteria	Firmicutes	Bacilli	Bacillales	Paenibacillaceae	Paenibacillus	0.203955	0.350108	0.316761	0.312497	0.291069	0.296793	0.294326	0.228963	0.188294	0.192768	0.045066	0.23824
Bacteria	Bacteroidetes	Bacteroidia	Bacteroidales	Porphyromonadaceae	Paludibacter	0.053583	0.190112	0.118653	0.152054	0.071287	0.103926	0.100089	0.105376	0.090519	0.206398	0.026585	0.250875
Bacteria	Proteobacteria	Gammaproteobacteria	Enterobacteriales	Enterobacteriaceae	Pantoea	0.060217	0.05741	0.076277	0.071308	0.048383	0.046488	0.057618	0.057087	0.068843	0.07886	0.102928	0.059581
Bacteria	Bacteroidetes	Bacteroidia	Bacteroidales	Porphyromonadaceae	Parabacteroides	0.224538	0.43387	0.322058	0.332421	0.151068	0.203241	0.194832	0.214435	0.150431	0.215161	0.044261	0.378569
Bacteria	Chlamydiae	Chlamydiae (class)	Chlamydiales	Parachlamydiaceae	Parachlamydia	0.020583	0.031058	0.021188	0.020973	0.049155	0.035923	0.048114	0.051563	0.024281	0.044785	0.002606	0.018828
Bacteria	Proteobacteria	Alphaproteobacteria	Rhodobacterales	Rhodobacteraceae	Paracoccus	0.166022	0.125173	0.146197	0.126886	0.16059	0.159442	0.189486	0.30426	0.275743	0.145063	0.085536	0.088589
Bacteria	Actinobacteria	Actinobacteria (class)	Bifidobacteriales	Bifidobacteriaceae	Parascardovia	0.002722	0.004706	0.003178	0.004195	0.002059	0.001537	0.00297	0.000818	0.000744	0	0.000521	0.002026
Bacteria	Proteobacteria	Alphaproteobacteria	Rhizobiales	Phyllobacteriaceae	Parvibaculum	0.21195	0.13929	0.253197	0.203437	0.379342	0.411476	0.552716	0.66704	0.394916	0.17135	0.051037	0.117267
Bacteria	Proteobacteria	Alphaproteobacteria	Parvularculales	Parvularculaceae	Parvularcula	0.035552	0.036705	0.033901	0.036703	0.062023	0.068964	0.089991	0.121745	0.08317	0.021419	0.007156	0.025664
Bacteria	Proteobacteria	Gammaproteobacteria	Pasteurellales	Pasteurellaceae	Pasteurella	0.043036	0.040469	0.043435	0.052432	0.032941	0.037267	0.03267	0.037035	0.03805	0.057441	0.07312	0.042894
Bacteria	Proteobacteria	Gammaproteobacteria	Enterobacteriales	Enterobacteriaceae	Pectobacterium	0.096279	0.087527	0.104881	0.094378	0.084927	0.079913	0.091773	0.082664	0.087077	0.142142	0.254524	0.092789
Bacteria	Firmicutes	Bacilli	Lactobacillales	Lactobacillaceae	Pediococcus	0.010206	0.020705	0.010594	0.017827	0.009007	0.012486	0.010098	0.008389	0.008187	0.008762	0.001043	0.010312
Bacteria	Bacteroidetes	Sphingobacteria	Sphingobacteriales	Sphingobacteriaceae	Pedobacter	0.197491	0.373637	0.324177	0.346053	0.19559	0.256836	0.299078	0.449945	0.427476	0.488736	0.123115	0.45589
Bacteria	Proteobacteria	Alphaproteobacteria	Rhodobacterales	Rhodobacteraceae	Pelagibaca	0.070083	0.049881	0.045554	0.047189	0.077207	0.102581	0.111672	0.183948	0.204854	0.088596	0.039901	0.050373
Bacteria	Proteobacteria	Deltaproteobacteria	Desulfuromonadales	Pelobacteraceae	Pelobacter	0.869574	0.973149	0.928035	1.031868	1.104311	0.984315	0.743093	0.509079	0.369332	0.249236	0.129323	0.739479

Bacteria	Chlorobi	Chlorobia	Chlorobiales	Chlorobiaceae	Pelodictyon	0.182352	0.271051	0.200227	0.223362	0.155185	0.156945	0.168102	0.138319	0.118242	0.104173	0.011942	0.142635
Bacteria	Firmicutes	Clostridia	Clostridiales	Peptococcaceae	Pelotomaculum	0.173336	0.355755	0.213999	0.289426	0.250664	0.215151	0.186219	0.114993	0.079262	0.045758	0.022983	0.140724
Bacteria	Firmicutes	Clostridia	Clostridiales	Clostridiales Family XI. Incertae Sedis	Peptoniphilus	0.014629	0.022588	0.029663	0.032508	0.024191	0.020362	0.025542	0.023121	0.015815	0.017524	0.003222	0.022254
Bacteria	Firmicutes	Clostridia	Clostridiales	Peptostreptococcaceae	Peptostreptococcus	0.003402	0.014117	0.005297	0.010486	0.015184	0.012871	0.011286	0.009208	0.007908	0.008762	0.001422	0.014183
Bacteria	Aquificae	Aquificae (class)	Aquificales	Hydrogenothermaceae	Persephonella	0.039124	0.041411	0.041317	0.05453	0.05842	0.044951	0.04752	0.029669	0.02363	0.026287	0.003365	0.044706
Bacteria	Thermotogae	Thermotogae (class)	Thermotogales	Thermotogaceae	Petrotoga	0.038444	0.047057	0.048732	0.045092	0.044265	0.046488	0.037125	0.033147	0.021304	0.012657	0.001848	0.038282
Bacteria	Proteobacteria	Alphaproteobacteria	Rhodobacterales	Rhodobacteraceae	Phaeobacter	0.047799	0.029176	0.037079	0.055578	0.037574	0.047641	0.069795	0.084301	0.070703	0.043811	0.022083	0.027114
Bacteria	Proteobacteria	Alphaproteobacteria	Caulobacterales	Caulobacteraceae	Phenylobacterium	0.058346	0.069645	0.083693	0.069211	0.074376	0.09336	0.13365	0.14548	0.117126	0.082754	0.009051	0.042614
Bacteria	Proteobacteria	Gammaproteobacteria	Vibrionales	Vibrionaceae	Photobacterium	0.19528	0.178818	0.19493	0.194	0.226473	0.209196	0.226908	0.218732	0.200481	0.299862	0.911518	0.294922
Bacteria	Proteobacteria	Gammaproteobacteria	Enterobacteriales	Enterobacteriaceae	Photorhabdus	0.055964	0.06588	0.062505	0.072357	0.083898	0.067811	0.076626	0.075093	0.061028	0.082754	0.330345	0.071276
Bacteria	Planctomycetes	Planctomycetacia	Planctomycetales	Planctomycetaceae	Pirellula	0.192558	0.264463	0.345365	0.247481	0.456806	0.443749	0.331748	0.311627	0.383566	0.104173	0.023979	0.201556
Bacteria	Planctomycetes	Planctomycetacia	Planctomycetales	Planctomycetaceae	Planctomyces	0.416586	0.641864	0.709798	0.572561	0.819162	0.771854	0.760616	0.790013	0.819787	0.349514	0.066581	0.428744
Bacteria	Proteobacteria	Deltaproteobacteria	Myxococcales	Nannocystaceae	Plesiocystis	0.136254	0.135526	0.191752	0.127935	0.17706	0.177884	0.227502	0.241444	0.197039	0.046732	0.013127	0.121369
Bacteria	Bacteroidetes	Flavobacteria	Flavobacteriales	Flavobacteriaceae	Polaribacter	0.140676	0.210818	0.146197	0.191902	0.077464	0.15099	0.150579	0.243899	0.28914	7.235625	0.295278	2.141677
Bacteria	Proteobacteria	Betaproteobacteria	Burkholderiales	Comamonadaceae	Polaromonas	0.307039	0.298345	0.398335	0.410021	0.235223	0.216496	0.224235	0.179446	0.275557	0.184006	0.230213	0.176716
Bacteria	Proteobacteria	Betaproteobacteria	Burkholderiales	Burkholderiaceae	Polynucleobacter	0.08131	0.06588	0.096405	0.095427	0.048125	0.044951	0.051678	0.043583	0.05647	0.042837	0.04791	0.051723
Bacteria	Bacteroidetes	Bacteroidia	Bacteroidales	Porphyromonadaceae	Porphyromonas	0.142207	0.207053	0.15891	0.197146	0.082354	0.10431	0.128304	0.139751	0.10159	0.164535	0.030139	0.216958
Bacteria	Bacteroidetes	Bacteroidia	Bacteroidales	Prevotellaceae	Prevotella	0.198682	0.413165	0.291335	0.339762	0.136913	0.18941	0.24057	0.2306	0.177875	0.306677	0.070372	0.367928
Bacteria	Cyanobacteria	unclassified (derived from Cyanobacteria)	Prochlorales	Prochlorococcaceae	Prochlorococcus	0.09696	0.126114	0.132425	0.109059	0.092391	0.073382	0.125631	0.072638	0.077123	0.064256	0.017865	0.07294
Bacteria	Actinobacteria	Actinobacteria (class)	Actinomycetales	Propionibacteriaceae	Propionibacterium	0.134212	0.085645	0.084752	0.06187	0.030111	0.031504	0.025839	0.022507	0.024467	0.019472	0.005639	0.025944
Bacteria	Chlorobi	Chlorobia	Chlorobiales	Chlorobiaceae	Prosthecochloris	0.056815	0.092233	0.068861	0.082843	0.076692	0.065506	0.064449	0.062816	0.048097	0.043811	0.019714	0.053947
Bacteria	Proteobacteria	Gammaproteobacteria	Enterobacteriales	Enterobacteriaceae	Proteus	0.040145	0.034823	0.033901	0.045092	0.040919	0.038228	0.048411	0.047061	0.040096	0.045758	0.051132	0.04604
Bacteria	Proteobacteria	Gammaproteobacteria	Enterobacteriales	Enterobacteriaceae	Providencia	0.040145	0.035764	0.041317	0.037751	0.047353	0.041301	0.049005	0.054018	0.038236	0.057441	0.05843	0.044426
Bacteria	Proteobacteria	Gammaproteobacteria	Alteromonadales	Pseudoalteromonadaceae	Pseudoalteromonas	0.356369	0.273875	0.305107	0.312497	0.352062	0.409171	0.442232	0.552457	0.522275	0.567595	9.63072	0.866251
Bacteria	Proteobacteria	Gammaproteobacteria	Pseudomonadales	Pseudomonadaceae	Pseudomonas	3.438662	1.697834	2.109267	2.009207	1.556999	1.692968	2.262839	2.062301	1.92276	2.281091	8.064672	2.298823
Bacteria	Firmicutes	Clostridia	Clostridiales	Eubacteriaceae	Pseudoramibacter	0.003402	0.014117	0.004238	0.004195	0.011066	0.006723	0.008019	0.004706	0.003721	0.000974	0.000853	0.005074
Bacteria	Proteobacteria	Alphaproteobacteria	Rhodobacterales	Rhodobacteraceae	Pseudovibrio	0.042356	0.03294	0.050851	0.062919	0.098567	0.098547	0.13068	0.132794	0.096845	0.067177	0.071841	0.036289
Bacteria	Proteobacteria	Gammaproteobacteria	Pseudomonadales	Moraxellaceae	Psychrobacter	0.126218	0.095997	0.081574	0.104865	0.080295	0.087405	0.127413	0.122768	0.106985	0.120724	0.220783	0.127678
Bacteria	Bacteroidetes	Flavobacteria	Flavobacteriales	Flavobacteriaceae	Psychroflexus	0.053073	0.083762	0.067802	0.080746	0.061765	0.093552	0.097119	0.188654	0.155082	0.481921	0.08947	0.20719
Bacteria	Proteobacteria	Gammaproteobacteria	Alteromonadales	Psychromonadaceae	Psychromonas	0.165342	0.123291	0.134544	0.144713	0.175259	0.162324	0.158598	0.170443	0.148198	0.181085	0.695521	0.218506
Bacteria	Synergistetes	Synergistia	Synergistales	Synergistaceae	Pyramidobacter	0.008845	0.021646	0.012713	0.013632	0.018272	0.022668	0.027324	0.012481	0.009117	0.006815	0.001422	0.011794
Bacteria	Proteobacteria	Betaproteobacteria	Burkholderiales	Burkholderiaceae	Ralstonia	0.338678	0.374578	0.473552	0.369124	0.183752	0.228214	0.255419	0.208706	0.299652	0.354382	0.205287	0.193534
Bacteria	Cyanobacteria	unclassified (derived from Cyanobacteria)	Nostocales	Nostocaceae	Raphidiopsis	0.008505	0.013176	0.012713	0.011535	0.012353	0.009413	0.01485	0.010845	0.020653	0.003894	0.006587	0.0099
Bacteria	Proteobacteria	Gammaproteobacteria	unclassified (derived from Gammaproteobacteria)	unclassified (derived from Gammaproteobacteria)	Reinekea	0.08182	0.064939	0.08899	0.092281	0.190443	0.143114	0.207306	0.343137	0.442082	0.982339	1.78304	0.256179
Bacteria	Actinobacteria	Actinobacteria (class)	Actinomycetales	Micrococcaceae	Renibacterium	0.007825	0.025411	0.024366	0.022022	0.007721	0.006339	0.008613	0.00532	0.008931	0.007789	0.000995	0.006424
Bacteria	Proteobacteria	Alphaproteobacteria	Rhizobiales	Rhizobiaceae	Rhizobium	0.404338	0.479045	0.507453	0.497059	0.460408	0.388616	0.488267	0.428256	0.544974	0.380669	0.180929	0.267001
Bacteria	Proteobacteria	Alphaproteobacteria	Rhodobacterales	Rhodobacteraceae	Rhodobacter	0.628536	0.424459	0.488384	0.488669	0.405592	0.546906	0.616868	0.973347	0.958775	0.836303	0.226328	0.314442
Bacteria	Actinobacteria	Actinobacteria (class)	Actinomycetales	Nocardiaceae	Rhodococcus	0.249033	0.240934	0.344305	0.222313	0.134597	0.115836	0.162459	0.141593	0.197876	0.342699	0.025448	0.115801
Bacteria	Proteobacteria	Alphaproteobacteria	Rhizobiales	Hyphomicrobiaceae	Rhodomicrobium	0.06532	0.063998	0.05297	0.078649	0.106288	0.082603	0.091179	0.110901	0.091821	0.076913	0.017155	0.035679
Bacteria	Planctomycetes	Planctomycetacia	Planctomycetales	Planctomycetaceae	Rhodopirellula	0.367426	0.617394	0.778659	0.586194	0.758683	0.782803	0.64241	0.735995	0.600142	0.089569	0.024784	0.344487
Bacteria	Proteobacteria	Alphaproteobacteria	Rhizobiales	Bradyrhizobiaceae	Rhodopseudomonas	0.92826	0.58916	0.832689	0.687912	0.550998	0.555166	0.719333	0.645761	0.732152	0.551045	0.163538	0.325412
Bacteria	Proteobacteria	Alphaproteobacteria	Rhodospirillales	Rhodospirillaceae	Rhodospirillum	0.304657	0.232464	0.334771	0.262162	0.878611	1.138955	1.384314	1.576753	0.839138	0.232685	0.099421	0.196862
Bacteria	Bacteroidetes	Sphingobacteria	Sphingobacteriales	Rhodothermaceae	Rhodothermus	0.273358	0.287992	0.292395	0.276843	0.344341	0.367485	0.389663	0.77569	0.6661	0.170376	0.023836	0.214668
Bacteria	Proteobacteria	Alphaproteobacteria	Rickettsiales	Rickettsiaceae	Rickettsia	0.054774	0.056469	0.064623	0.067113	0.079523	0.075879	0.126819	0.10333	0.056284	0.039917	0.008009	0.040275
Bacteria	Proteobacteria	Gammaproteobacteria	Legionellales	Coxiellaceae	Rickettsiella	0.009696	0.012235	0.010594	0.006292	0.02239	0.012486	0.019899	0.020461	0.010326	0.017524	0.001896	0.014117
Bacteria	Bacteroidetes	Flavobacteria	Flavobacteriales	Flavobacteriaceae	Riemerella	0.018711	0.027293	0.026485	0.033557	0.015956	0.026702	0.034452	0.057701	0.054144	0.139222	0.021704	0.111551
Bacteria	Bacteroidetes	Flavobacteria	Flavobacteriales	Flavobacteriaceae	Robiginitalea	0.208378	0.2127	0.269088	0.251675	0.153898	0.299098	0.248589	0.433372	0.342632	1.063146	0.14245	0.567787
Bacteria	Firmicutes	Clostridia	Clostridiales	Lachnospiraceae	Roseburia	0.017691	0.016941	0.014832	0.024119	0.016985	0.017097	0.016335	0.017188	0.009303	0.015577	0.002132	0.015962
Bacteria	Proteobacteria	Alphaproteobacteria	Rhodobacterales	Rhodobacteraceae	Roseibium	0.031639	0.039528	0.047673	0.057676	0.106545	0.096242	0.140481	0.140979	0.089217	0.052573	0.041749	0.030754
Bacteria	Chloroflexi	Chloroflexi (class)	Chloroflexales	Chloroflexaceae	Roseiflexus	0.718691	1.151026	0.884599	0.957414	0.779014	0.614909	0.557765	0.411478	0.571488	0.211266	0.04336	0.533854
Bacteria	Proteobacteria	Alphaproteobacteria	Rhodobacterales	Rhodobacteraceae	Roseobacter	0.542293	0.412224	0.486265	0.606118	0.553828	0.910165	0.89278	1.763565	1.241496	0.500419	0.41536	0.37959
Bacteria	Proteobacteria	Alphaproteobacteria	Rhodospirillales	Acetobacteraceae	Roseomonas	0.023815	0.039528	0.041317	0.041946	0.087243	0.108152	0.153252	0.146503	0.105497	0.072045	0.009809	0.025746
Bacteria	Proteobacteria	Alphaproteobacteria	Rhodobacterales	Rhodobacteraceae	Roseovarius	0.235765	0.15529	0.192811	0.279989	0.244745	0.42281	0.457676	1.004653	0.562464	0.229764	0.127286	0.17754
Bacteria	Actinobacteria	Actinobacteria (class)	Actinomycetales	Micrococcaceae	Rothia	0.009526	0.025411	0.012713	0.018876	0.008493	0.007492	0.010989	0.009003	0.015815	0.005841	0.005402	0.007577
Bacteria	Actinobacteria	Actinobacteria (class)	Rubrobacterales	Rubrobacteraceae	Rubrobacter	0.143228	0.223053	0.241543	0.200292	0.206656	0.175578	0.152658	0.145685	0.187364	0.064256	0.006729	0.111683
Bacteria	Proteobacteria	Alphaproteobacteria	Rhodobacterales	Rhodobacteraceae	Ruegeria	0.605232	0.412224	0.566779	0.644918	0.628204	0.961455	0.969703	1.935236	1.295919	0.510154	0.326223	0.426981
Bacteria	Firmicutes	Clostridia	Clostridiales	Ruminococcaceae	Ruminococcus	0.052732	0.115761	0.064623	0.089135	0.071802	0.079145	0.08613	0.057292	0.033677	0.03797	0.007961	0.063007
Bacteria	Actinobacteria	Actinobacteria (class)	Actinomycetales	Pseudonocardiaceae	Saccharomonospora	0.029088	0.039528	0.028604	0.034605	0.023419	0.020362	0.029106	0.027623	0.033119	0.022392	0.005592	0.019059
Bacteria	Proteobacteria	Gammaproteobacteria	Alteromonadales	Alteromonadaceae	Saccharophagus	0.286456	0.239052	0.302989	0.256918	0.477651	0.424731	0.727055	0.80495	0.408312	0.285258	1.070459	0.395074
Bacteria	Actinobacteria	Actinobacteria (class)	Actinomycetales	Pseudonocardiaceae	Saccharopolyspora	0.079779	0.127996	0.149375	0.138421	0.073604	0.075495	0.08019	0.077958	0.10866	0.061335	0.013648	0.056912
Bacteria	Proteobacteria	Alphaproteobacteria	Rhodobacterales	Rhodobacteraceae	Sagittula	0.066001	0.034823	0.049792	0.059773	0.064854	0.11161	0.086427	0.184357	0.218529	0.103199	0.046062	0.040753
Bacteria	Bacteroidetes	Sphingobacteria	Sphingobacteriales	Rhodothermaceae	Salinibacter	0.186264	0.229641	0.278622	0.27894	0.307797	0.321574	0.371249	0.989512	0.735874	0.109041	0.014596	0.170753
Bacteria	Actinobacteria	Actinobacteria (class)	Actinomycetales	Micromonosporaceae	Salinispora	0.09781	0.166584	0.12395	0.120594	0.083126	0.064161	0.081081	0.076116	0.093217	0.047705	0.008625	0.065494
Bacteria	Proteobacteria	Gammaproteobacteria	Enterobacteriales	Enterobacteriaceae	Salmonella	0.615778	0.118585	0.10594	0.125838	0.085957	0.08875	0.123255	0.110696	0.07675	0.087622	0.110557	0.096627
Bacteria	Actinobacteria	Actinobacteria (class)	Actinomycetales	Sanguibacteraceae	Sanguibacter	0.023304	0.043293	0.045554	0.035654	0.014155	0.01095	0.020196	0.011049	0.017583	0.011683	0.00834	0.014133
Bacteria	Actinobacteria	Actinobacteria (class)	Bifidobacteriales	Bifidobacteriaceae	Scardovia	0	0.002823	0.003178	0.005243	0.002059	0.00096	0.000297	0.000409	0.001395	0.000974	0.000284	0.001944
Bacteria	Fusobacteria	Fusobacteria (class)	Fusobacteriales	Fusobacteriaceae	Sebaldella	0.03232	0.049881	0.03602	0.028313	0.035515	0.036883	0.027324	0.034989	0.019257	0.022392	0.003365	0.030639

Bacteria	Actinobacteria	Actinobacteria (class)	Actinomycetales	Segniliparaceae	Segniliparus	0.002722	0.014117	0.015891	0.005243	0.006949	0.004418	0.00891	0.004092	0.007256	0.002921	0.001137	0.004909
Bacteria	Firmicutes	Negativicutes	Selenomonadales	Veillonellaceae	Selenomonas	0.043887	0.070586	0.054029	0.057676	0.055846	0.048985	0.050787	0.039695	0.028281	0.032128	0.009288	0.04151
Bacteria	Proteobacteria	Gammaproteobacteria	Enterobacteriales	Enterobacteriaceae	Serratia	0.09832	0.098821	0.090049	0.087038	0.063824	0.068195	0.088209	0.072638	0.083542	0.074965	0.138659	0.083993
Bacteria	Proteobacteria	Gammaproteobacteria	Alteromonadales	Shewanellaceae	Shewanella	1.604766	0.983502	1.145212	1.14722	0.911552	0.963376	1.159783	1.04353	1.083064	1.130323	3.297577	1.353044
Bacteria	Proteobacteria	Gammaproteobacteria	Enterobacteriales	Enterobacteriaceae	Shigella	0.084712	0.030117	0.03602	0.035654	0.020074	0.02651	0.04158	0.035194	0.020932	0.036996	0.048099	0.029436
Bacteria	Firmicutes	Clostridia	Clostridiales	Lachnospiraceae	Shuttleworthia	0.00051	0.009411	0.006356	0.006292	0.005919	0.005379	0.008316	0.006138	0.002791	0.003894	0.002132	0.004431
Bacteria	Proteobacteria	Betaproteobacteria	Gallionellales	Gallionellaceae	Sideroxydans	0.22794	0.145878	0.19387	0.210778	0.157759	0.124672	0.110781	0.085324	0.128196	0.246315	0.146336	0.161858
Bacteria	Proteobacteria	Betaproteobacteria	Neisseriales	Neisseriaceae	Simonsiella	0.008505	0.006588	0.007416	0.002097	0.006434	0.004995	0.008316	0.011254	0.006047	0.010709	0.002843	0.006803
Bacteria	Proteobacteria	Alphaproteobacteria	Rhizobiales	Rhizobiaceae	Sinorhizobium	0.514906	0.459281	0.60174	0.539004	0.514968	0.489852	0.55925	0.545091	0.591025	0.316413	0.20832	0.28886
Bacteria	Actinobacteria	Actinobacteria (class)	Coriobacteriales	Coriobacteriaceae	Slackia	0.026026	0.041411	0.020129	0.027265	0.02728	0.021707	0.023166	0.013914	0.013582	0.006815	0.00199	0.021529
Bacteria	Proteobacteria	Gammaproteobacteria	Enterobacteriales	Enterobacteriaceae	Sodalis	0.028407	0.02447	0.03602	0.034605	0.020846	0.026318	0.024948	0.027214	0.024839	0.016551	0.035494	0.023276
Bacteria	Proteobacteria	Deltaproteobacteria	Myxococcales	Polyangiaceae	Sorangium	0.329322	0.379283	0.409988	0.309351	0.328643	0.292375	0.340955	0.312036	0.424127	0.137274	0.042365	0.252374
Bacteria	Chloroflexi	Thermomicrobia (class)	Sphaerobacterales	Sphaerobacteraceae	Sphaerobacter	0.180481	0.250346	0.187514	0.243286	0.247576	0.166166	0.157707	0.111105	0.18234	0.066203	0.012084	0.134118
Bacteria	Bacteroidetes	Sphingobacteria	Sphingobacteriales	Sphingobacteriaceae	Sphingobacterium	0.063109	0.086586	0.083693	0.074454	0.044008	0.072229	0.089397	0.121745	0.121126	0.180112	0.046062	0.134003
Bacteria	Proteobacteria	Alphaproteobacteria	Sphingomonadales	Sphingomonadaceae	Sphingobium	0.118053	0.09035	0.125009	0.115351	0.071802	0.071653	0.108999	0.108241	0.096566	0.093463	0.028433	0.050636
Bacteria	Proteobacteria	Alphaproteobacteria	Sphingomonadales	Sphingomonadaceae	Sphingomonas	0.273528	0.183524	0.26591	0.247481	0.171656	0.159442	0.25245	0.252493	0.214994	0.154799	0.061889	0.102738
Bacteria	Proteobacteria	Alphaproteobacteria	Sphingomonadales	Sphingomonadaceae	Sphingopyxis	0.185244	0.09788	0.169504	0.141567	0.078493	0.092015	0.131571	0.145685	0.114428	0.069124	0.011373	0.056402
Bacteria	Spirochaetes	Spirochaetes (class)	Spirochaetales	Spirochaetaceae	Spirochaeta	0.152584	0.295521	0.22989	0.276843	0.374709	0.48044	0.323432	0.403498	0.096566	0.126565	0.024595	0.236807
Bacteria	Bacteroidetes	Cytophagia	Cytophagales	Cytophagaceae	Spirosoma	0.205826	0.298345	0.277563	0.310399	0.196877	0.235129	0.223938	0.350912	0.405614	0.256051	0.070182	0.311032
Bacteria	Actinobacteria	Actinobacteria (class)	Actinomycetales	Glycomycetaceae	Stackebrandtia	0.059366	0.054587	0.064623	0.041946	0.035772	0.027086	0.039204	0.030487	0.043352	0.023366	0.003128	0.0254
Bacteria	Firmicutes	Bacilli	Bacillales	Staphylococcaceae	Staphylococcus	0.370147	0.156231	0.10594	0.117448	0.08081	0.091631	0.124443	0.097396	0.094705	0.057441	0.016728	0.098258
Bacteria	Proteobacteria	Alphaproteobacteria	Rhizobiales	Xanthobacteraceae	Starkeya	0.070253	0.060234	0.090049	0.078649	0.08081	0.098547	0.123255	0.12686	0.130243	0.086648	0.037532	0.039188
Bacteria	Proteobacteria	Gammaproteobacteria	Xanthomonadales	Xanthomonadaceae	Stenotrophomonas	0.145609	0.140231	0.163148	0.1552	0.083126	0.096818	0.133353	0.13218	0.13536	0.125591	0.061889	0.10885
Bacteria	Proteobacteria	Deltaproteobacteria	Myxococcales	Cystobacteraceae	Stigmatella	0.163981	0.240934	0.225652	0.196097	0.219781	0.193252	0.220968	0.194178	0.236763	0.176217	0.027106	0.159189
Bacteria	Fusobacteria	Fusobacteria (class)	Fusobacteriales	Fusobacteriaceae	Streptobacillus	0.007995	0.003765	0.007416	0.013632	0.012353	0.005571	0.007128	0.00798	0.004093	0	0.00109	0.006638
Bacteria	Firmicutes	Bacilli	Lactobacillales	Streptococcaceae	Streptococcus	0.270806	0.216464	0.14196	0.153102	0.108089	0.116604	0.144342	0.112128	0.075076	0.087622	0.029002	0.110678
Bacteria	Actinobacteria	Actinobacteria (class)	Actinomycetales	Streptomycetaceae	Streptomyces	0.572402	1.041853	1.075292	0.894496	0.467357	0.406097	0.519155	0.412501	0.573442	0.389431	0.085157	0.348622
Bacteria	Actinobacteria	Actinobacteria (class)	Actinomycetales	Streptosporangiaceae	Streptosporangium	0.068382	0.089409	0.083693	0.076551	0.058934	0.051483	0.055539	0.051563	0.071355	0.036996	0.012463	0.044673
Bacteria	Firmicutes	Clostridia	Clostridiales	Ruminococcaceae	Subdoligranulum	0.010206	0.018823	0.009535	0.014681	0.012353	0.006339	0.009801	0.00798	0.004559	0.004868	0.000569	0.00761
Bacteria	Proteobacteria	Alphaproteobacteria	Rhodobacterales	Rhodobacteraceae	Sulfitobacter	0.071444	0.054587	0.078396	0.091232	0.073346	0.097586	0.120285	0.167374	0.179177	0.080807	0.083593	0.06617
Bacteria	Proteobacteria	Epsilonproteobacteria	Campylobacterales	Helicobacteraceae	Sulfuricurvum	0.018201	0.041411	0.05297	0.0388	0.026508	0.031696	0.028512	0.028237	0.010047	0.022392	0.019192	0.328526
Bacteria	Aquificae	Aquificae (class)	Aquificales	Hydrogenothermaceae	Sulfurihydrogenibium	0.053583	0.107291	0.098524	0.090184	0.088788	0.069348	0.083457	0.053609	0.040468	0.075939	0.007203	0.071787
Bacteria	Proteobacteria	Epsilonproteobacteria	Campylobacterales	Helicobacteraceae	Sulfurimonas	0.086753	0.086586	0.119712	0.075503	0.054559	0.087021	0.085239	0.071615	0.032003	0.150904	0.058004	2.007097
Bacteria	Proteobacteria	Epsilonproteobacteria	Campylobacterales	Campylobacteraceae	Sulfurospirillum	0.031639	0.043293	0.03496	0.035654	0.025478	0.029583	0.029403	0.024554	0.010792	0.046732	0.01796	0.189993
Bacteria	Proteobacteria	Epsilonproteobacteria	unclassified (derived from Epsilonproteobacteria)	unclassified (derived from Epsilonproteobacteria)	Sulfurovum	0.151053	0.148702	0.224593	0.109059	0.064339	0.106231	0.067419	0.126656	0.030886	0.044785	0.029097	0.46411
Bacteria	Firmicutes	Clostridia	Clostridiales	Clostridiales Family XVIII. Incertae Sedis	Symbiobacterium	0.163981	0.235287	0.220355	0.206583	0.203311	0.169623	0.147906	0.109059	0.107916	0.052573	0.012416	0.120726
Bacteria	Cyanobacteria	unclassified (derived from Cyanobacteria)	Chroococcales	unclassified (derived from Chroococcales)	Synechococcus	0.514906	0.554337	0.51169	0.535858	0.463497	0.447783	0.488267	0.413933	0.673357	0.209319	0.122262	0.32645
Bacteria	Cyanobacteria	unclassified (derived from Cyanobacteria)	Chroococcales	unclassified (derived from Chroococcales)	Synechocystis	0.095769	0.160937	0.149375	0.156248	0.153126	0.174042	0.150282	0.138728	0.227181	0.050626	0.021325	0.101734
Bacteria	Proteobacteria	Deltaproteobacteria	Syntrophobacterales	Syntrophobacteraceae	Syntrophobacter	0.996302	1.387255	1.04351	1.333879	1.796082	1.785368	0.968812	0.485344	0.289605	0.165508	0.039806	0.816257
Bacteria	Firmicutes	Clostridia	Clostridiales	Syntrophomonadaceae	Syntrophomonas	0.11363	0.158113	0.132425	0.145762	0.142575	0.110073	0.089991	0.066295	0.051074	0.035049	0.006871	0.089
Bacteria	Firmicutes	Clostridia	Clostridiales	Syntrophomonadaceae	Syntrophothermus	0.052562	0.099762	0.064623	0.091232	0.080037	0.057438	0.049896	0.025372	0.025583	0.044785	0.005355	0.047457
Bacteria	Proteobacteria	Deltaproteobacteria	Syntrophobacterales	Syntrophaceae	Syntrophus	0.540592	0.75292	0.616571	0.699447	0.936516	0.884039	0.504008	0.298736	0.182433	0.124618	0.018955	0.470567
Bacteria	Proteobacteria	Gammaproteobacteria	Alteromonadales	unclassified (derived from Alteromonadales)	Teredinibacter	0.165512	0.116703	0.143019	0.133178	0.254782	0.238779	0.475496	0.441556	0.210994	0.222949	0.71604	0.227435
Bacteria	Acidobacteria	Acidobacteria (class)	Acidobacteriales	Acidobacteriaceae	Terriglobus	0.06481	0.074351	0.102762	0.085989	0.056618	0.049754	0.053757	0.037649	0.079913	0.079833	0.004834	0.05034
Bacteria	Firmicutes	Bacilli	Lactobacillales	Enterococcaceae	Tetragenococcus	0	0	0	0	0	0	0	0	0	0	0	1.65E-05
Bacteria	Proteobacteria	Alphaproteobacteria	Rhodobacterales	Rhodobacteraceae	Thalassobium	0.045078	0.020705	0.03602	0.029362	0.027794	0.043799	0.039501	0.075502	0.064284	0.036996	0.015923	0.01947
Bacteria	Proteobacteria	Betaproteobacteria	Rhodocyclales	Rhodocyclaceae	Thauera	0.123666	0.123291	0.179039	0.144713	0.109119	0.120254	0.127116	0.103125	0.144291	0.129486	0.169272	0.124548
Bacteria	Firmicutes	Clostridia	Clostridiales	Clostridiales Family XVII. Incertae Sedis	Thermaerobacter	0.050691	0.115761	0.116534	0.074454	0.147465	0.118333	0.117018	0.088802	0.113311	0.104173	0.012037	0.080534
Bacteria	Synergistetes	Synergistia	Synergistales	Synergistaceae	Thermanaerovibrio	0.047629	0.095997	0.06992	0.056627	0.058934	0.050714	0.048114	0.02926	0.02363	0.024339	0.004028	0.037854
Bacteria	Firmicutes	Clostridia	Clostridiales	Peptococcaceae	Thermincola	0.087264	0.13929	0.094287	0.127935	0.144376	0.111994	0.101277	0.056678	0.038236	0.05452	0.015259	0.080155
Bacteria	Firmicutes	Clostridia	Thermoanaerobacterales	Thermoanaerobacteraceae	Thermoanaerobacter	0.327621	0.283286	0.182217	0.246432	0.161362	0.153487	0.169587	0.08737	0.072564	0.129486	0.016586	0.144463
Bacteria	Firmicutes	Clostridia	Thermoanaerobacterales	Thermoanaerobacterales Family III. Incertae Sedis	Thermoanaerobacterium	0.033511	0.064939	0.062505	0.078649	0.054817	0.036691	0.043065	0.034375	0.019629	0.026287	0.004218	0.040423
Bacteria	unclassified (derived from Bacteria)	unclassified (derived from Bacteria)	unclassified (derived from Bacteria)	unclassified (derived from Bacteria)	Thermobaculum	0.1468	0.190112	0.147257	0.183513	0.15956	0.111225	0.122364	0.085528	0.10559	0.056467	0.006682	0.101783
Bacteria	Actinobacteria	Actinobacteria (class)	Actinomycetales	Nocardiopsaceae	Thermobifida	0.070423	0.103526	0.112296	0.090184	0.056876	0.047448	0.053757	0.051767	0.053865	0.030181	0.005592	0.039402
Bacteria	Actinobacteria	Actinobacteria (class)	Actinomycetales	Pseudonocardiaceae	Thermobispora	0.059707	0.060234	0.074158	0.039849	0.032941	0.032657	0.03564	0.030283	0.03777	0.02726	0.005829	0.027147
Bacteria	Aquificae	Aquificae (class)	Aquificales	Aquificaceae	Thermocrinis	0.022284	0.031999	0.020129	0.028313	0.024706	0.017865	0.014553	0.01064	0.015164	0.014604	0.002369	0.016687
Bacteria	Nitrospirae	Nitrospira (class)	Nitrospirales	Nitrospiraceae	Thermodesulfovibrio	0.106826	0.145878	0.117593	0.132129	0.327871	0.198246	0.114048	0.066909	0.056377	0.089569	0.006682	0.095853
Bacteria	Chloroflexi	Thermomicrobia (class)	Thermomicrobiales	Thermomicrobiaceae	Thermomicrobium	0.117032	0.137408	0.131366	0.117448	0.129707	0.102197	0.09207	0.062816	0.103171	0.038943	0.005639	0.079249
Bacteria	Actinobacteria	Actinobacteria (class)	Actinomycetales	Thermomonosporaceae	Thermomonospora	0.059707	0.076233	0.080514	0.072357	0.038346	0.036691	0.043065	0.039695	0.058423	0.047705	0.009146	0.033291
Bacteria	Firmicutes	Clostridia	Thermoanaerobacterales	Thermoanaerobacterales Family III. Incertae Sedis	Thermosediminibacter	0.047969	0.079057	0.063564	0.088086	0.100626	0.074342	0.055836	0.048289	0.032747	0.030181	0.006303	0.056714
Bacteria	Firmicutes	Negativicutes	Selenomonadales	Veillonellaceae	Thermosinus	0.055114	0.084703	0.066742	0.083892	0.103972	0.078376	0.066528	0.049107	0.028747	0.0516	0.007582	0.053387
Bacteria	Thermotogae	Thermotogae (class)	Thermotogales	Thermotogaceae	Thermosipho	0.066001	0.129879	0.097465	0.097524	0.08364	0.092015	0.081972	0.065886	0.041492	0.04089	0.005829	0.070222
Bacteria	Cyanobacteria	unclassified (derived from Cyanobacteria)	Chroococcales	unclassified (derived from Chroococcales)	Thermosynechococcus	0.06515	0.086586	0.092168	0.085989	0.068199	0.072998	0.062964	0.057906	0.106706	0.036996	0.013079	0.046979
Bacteria	Thermotogae	Thermotogae (class)	Thermotogales	Thermotogaceae	Thermotoga	0.241548	0.35199	0.226712	0.291524	0.207943	0.189602	0.203445	0.119904	0.108102	0.105146	0.013221	0.176914
Bacteria	Deinococcus-Thermus	Deinococci	Thermales	Thermaceae	Thermus	0.161089	0.197641	0.174801	0.190854	0.154928	0.152334	0.155331	0.123996	0.113684	0.056467	0.014074	0.102755

Bacteria	Proteobacteria	Gammaproteobacteria	Chromatiales	Ectothiorhodospiraceae	Thioalkalivibrio	0.796259	0.54869	0.625046	0.617653	1.020671	1.075946	0.844963	0.734768	0.491203	0.286232	0.389865	0.921845
Bacteria	Proteobacteria	Betaproteobacteria	Hydrogenophilales	Hydrogenophilaceae	Thiobacillus	0.45571	0.352931	0.402572	0.486572	0.282319	0.270475	0.247995	0.191109	0.210808	0.192768	0.118234	0.357204
Bacteria	Proteobacteria	Gammaproteobacteria	Thiotrichales	Piscirickettsiaceae	Thiomicrospira	0.222837	0.134584	0.137722	0.163589	0.160847	0.182878	0.165132	0.188245	0.102985	0.181085	0.369773	0.393098
Bacteria	Proteobacteria	Betaproteobacteria	Burkholderiales	unclassified (derived from Burkholderiales)	Thiomonas	0.068552	0.062116	0.067802	0.089135	0.031912	0.030736	0.034749	0.0266	0.056005	0.06523	0.029286	0.047704
Bacteria	Proteobacteria	Gammaproteobacteria	Aeromonadales	Aeromonadaceae	Tolumonas	0.059877	0.034823	0.045554	0.063967	0.049927	0.048601	0.055836	0.05279	0.055818	0.07886	0.229929	0.071655
Bacteria	Spirochaetes	Spirochaetes (class)	Spirochaetales	Spirochaetaceae	Treponema	0.086243	0.181642	0.106999	0.124789	0.171399	0.216111	0.159192	0.191928	0.046888	0.039917	0.013601	0.108751
Bacteria	Cyanobacteria	unclassified (derived from Cyanobacteria)	Oscillatoriales	unclassified (derived from Oscillatoriales)	Trichodesmium	0.115331	0.110115	0.114415	0.113254	0.1382	0.144266	0.121176	0.113561	0.203923	0.034075	0.019003	0.072561
Bacteria	Actinobacteria	Actinobacteria (class)	Actinomycetales	unclassified (derived from Actinomycetales)	Tropheryma	0.001701	0.009411	0.008475	0.014681	0.006434	0.003458	0.006534	0.004501	0.004372	0.003894	0.000426	0.003311
Bacteria	Deinococcus-Thermus	Deinococci	Deinococcales	Trueperaceae	Truepera	0.096449	0.087527	0.097465	0.094378	0.10217	0.105078	0.104544	0.157757	0.092194	0.043811	0.007772	0.061607
Bacteria	Actinobacteria	Actinobacteria (class)	Actinomycetales	Tsukamurellaceae	Tsukamurella	0.037253	0.033881	0.029663	0.022022	0.010294	0.007492	0.015444	0.014732	0.015629	0.010709	0.002559	0.011794
Bacteria	Firmicutes	Erysipelotrichi	Erysipelotrichales	Erysipelotrichaceae	Turicibacter	0.007655	0.015058	0.008475	0.010486	0.008235	0.016136	0.013662	0.010026	0.004745	0.006815	0.00545	0.010822
Bacteria	Tenericutes	Mollicutes	Mycoplasmatales	Mycoplasmataceae	Ureaplasma	0.00017	0.002823	0.001059	0.007341	0.002831	0.001921	0.001782	0.00266	0.001395	0	0.001043	0.001697
Bacteria	Proteobacteria	Betaproteobacteria	Burkholderiales	Comamonadaceae	Variovorax	0.068382	0.075292	0.101702	0.104865	0.054817	0.051483	0.06534	0.048698	0.101497	0.07886	0.041749	0.048495
Bacteria	Firmicutes	Negativicutes	Selenomonadales	Veillonellaceae	Veillonella	0.037933	0.075292	0.043435	0.060822	0.040147	0.039572	0.053163	0.033761	0.024095	0.022392	0.011089	0.036733
Bacteria	Proteobacteria	Betaproteobacteria	Burkholderiales	Comamonadaceae	Verminephrobacter	0.104614	0.124232	0.120772	0.161492	0.111177	0.110841	0.108108	0.088188	0.129778	0.09249	0.050232	0.067257
Bacteria	Verrucomicrobia	Verrucomicrobiae	Verrucomicrobiales	Verrucomicrobiaceae	Verrucomicrobium	0.060727	0.145878	0.155732	0.127935	0.129964	0.119678	0.130086	0.144253	0.150431	0.061335	0.024547	0.09918
Bacteria	Proteobacteria	Gammaproteobacteria	Vibrionales	Vibrionaceae	Vibrio	1.973892	0.663511	0.722511	0.839966	0.574674	0.675612	0.766852	0.707963	0.629911	0.992075	2.813077	1.044005
Bacteria	Lentisphaerae	unclassified (derived from Lentisphaerae)	unclassified (derived from Lentisphaerae)	Victivallaceae	Victivallis	0.050691	0.083762	0.092168	0.071308	0.099339	0.128706	0.110187	0.093918	0.033026	0.017524	0.008009	0.058625
Bacteria	Chlamydiae	Chlamydiae (class)	Chlamydiales	Waddliaceae	Waddlia	0.019732	0.033881	0.032841	0.029362	0.068971	0.047448	0.057915	0.067113	0.021862	0.035049	0.003744	0.022715
Bacteria	Firmicutes	Bacilli	Lactobacillales	Leuconostocaceae	Weissella	0.001191	0.001882	0.001059	0.001049	0.002831	0.001921	0.002079	0.001842	0.002791	0.001947	0.000332	0.001927
Bacteria	Proteobacteria	Gammaproteobacteria	Enterobacteriales	Enterobacteriaceae	Wigglesworthia	0.00017	0.001882	0	0.004195	0.003088	0.004226	0.002079	0.00266	0.001023	0.000974	0.000569	0.002487
Bacteria	Proteobacteria	Alphaproteobacteria	Rickettsiales	Anaplasmataceae	Wolbachia	0.014969	0.028234	0.023307	0.024119	0.031397	0.038612	0.065934	0.048084	0.022234	0.016551	0.002701	0.016719
Bacteria	Proteobacteria	Epsilonproteobacteria	Campylobacterales	Helicobacteraceae	Wolinella	0.045418	0.047057	0.057208	0.048238	0.038603	0.053788	0.042174	0.035194	0.022234	0.026287	0.009241	0.18258
Bacteria	Proteobacteria	Alphaproteobacteria	Rhizobiales	Xanthobacteraceae	Xanthobacter	0.135063	0.159054	0.226712	0.174075	0.180663	0.168663	0.208494	0.2173	0.221785	0.144089	0.087621	0.084438
Bacteria	Proteobacteria	Gammaproteobacteria	Xanthomonadales	Xanthomonadaceae	Xanthomonas	1.00923	0.549632	0.619749	0.61241	0.271252	0.312545	0.3959	0.371374	0.41101	0.309598	0.225095	0.341457
Bacteria	Proteobacteria	Gammaproteobacteria	Enterobacteriales	Enterobacteriaceae	Xenorhabdus	0.01633	0.017882	0.030723	0.026216	0.036544	0.03112	0.036234	0.036421	0.024839	0.047705	0.113069	0.030194
Bacteria	Actinobacteria	Actinobacteria (class)	Actinomycetales	Promicromonosporaceae	Xylanimonas	0.027897	0.037646	0.03602	0.032508	0.014669	0.010758	0.016632	0.011663	0.018606	0.011683	0.002038	0.012371
Bacteria	Proteobacteria	Gammaproteobacteria	Xanthomonadales	Xanthomonadaceae	Xylella	0.230321	0.102585	0.131366	0.119546	0.060736	0.06954	0.081378	0.077344	0.07368	0.056467	0.02668	0.076201
Bacteria	Proteobacteria	Gammaproteobacteria	Enterobacteriales	Enterobacteriaceae	Yersinia	0.941868	0.207994	0.199167	0.219167	0.113751	0.133509	0.180576	0.153256	0.143081	0.173297	0.31049	0.178413
Bacteria	Bacteroidetes	Flavobacteria	Flavobacteriales	Flavobacteriaceae	Zunongwangia	0.101552	0.166584	0.139841	0.163589	0.08467	0.118141	0.119097	0.254949	0.615492	2.16913	0.47308	0.836139
Bacteria	Proteobacteria	Alphaproteobacteria	Sphingomonadales	Sphingomonadaceae	Zymomonas	0.131831	0.063057	0.074158	0.069211	0.035515	0.046104	0.071577	0.072638	0.042701	0.031154	0.007061	0.026718
Bacteria	Actinobacteria	Actinobacteria (class)	unclassified (derived from Actinobacteria (class))	unclassified (derived from Actinobacteria (class))	unclassified (derived from Actinobacteria (class))	0.007144	0.031999	0.027544	0.019924	0.012096	0.011526	0.012177	0.009821	0.012001	0.014604	0.001469	0.011794
Bacteria	Firmicutes	Bacilli	Bacillales	Alicyclobacillaceae	unclassified (derived from Alicyclobacillaceae)	0.062939	0.089409	0.072039	0.076551	0.059964	0.058782	0.053163	0.037854	0.055539	0.05452	0.009809	0.041889
Bacteria	Proteobacteria	Alphaproteobacteria	unclassified (derived from Alphaproteobacteria)	unclassified (derived from Alphaproteobacteria)	unclassified (derived from Alphaproteobacteria)	0.074506	0.091292	0.132425	0.094378	0.470445	0.639882	0.700028	0.741111	0.533439	0.152852	0.059046	0.084586
Bacteria	Proteobacteria	Gammaproteobacteria	Alteromonadales	unclassified (derived from Alteromonadales)	unclassified (derived from Alteromonadales)	0.028407	0.021646	0.029663	0.031459	0.030625	0.046104	0.058806	0.051767	0.0534	0.072045	3.428322	0.15942
Bacteria	unclassified (derived from Bacteria)	unclassified (derived from Bacteria)	unclassified (derived from Bacteria)	unclassified (derived from Bacteria)	unclassified (derived from Bacteria)	0.001361	0.021646	0.015891	0.007341	0.050442	0.038036	0.073062	0.05054	0.008001	0.03797	0.003317	0.01102
Bacteria	Bacteroidetes	unclassified (derived from Bacteroidetes)	unclassified (derived from Bacteroidetes)	unclassified (derived from Bacteroidetes)	unclassified (derived from Bacteroidetes)	0.037253	0.05741	0.056148	0.078649	0.039633	0.066658	0.067419	0.099647	0.102334	0.733104	0.102217	0.330387
Bacteria	Proteobacteria	Betaproteobacteria	unclassified (derived from Betaproteobacteria)	unclassified (derived from Betaproteobacteria)	unclassified (derived from Betaproteobacteria)	0.005784	0.006588	0.008475	0.011535	0.009779	0.006916	0.013068	0.011458	0.008094	0.018498	0.010046	0.009307
Bacteria	Proteobacteria	Betaproteobacteria	Burkholderiales	unclassified (derived from Burkholderiales)	unclassified (derived from Burkholderiales)	0.006464	0.019764	0.01801	0.012584	0.017243	0.014023	0.012474	0.014323	0.008466	0.019472	0.002701	0.012091
Bacteria	Proteobacteria	Epsilonproteobacteria	Campylobacterales	unclassified (derived from Campylobacterales)	unclassified (derived from Campylobacterales)	0.033681	0.029176	0.041317	0.033557	0.02728	0.039188	0.038016	0.032124	0.009396	0.025313	0.033788	0.927479
Bacteria	Candidatus Poribacteria	unclassified (derived from Candidatus Poribacteria)	unclassified (derived from Candidatus Poribacteria)	unclassified (derived from Candidatus Poribacteria)	unclassified (derived from Candidatus Poribacteria)	0.01548	0.03294	0.023307	0.029362	0.068971	0.05129	0.058509	0.03724	0.055446	0.059388	0.002938	0.026537
Bacteria	Cyanobacteria	unclassified (derived from Cyanobacteria)	Chroococcales	unclassified (derived from Chroococcales)	unclassified (derived from Chroococcales)	0.005784	0.007529	0.004238	0.007341	0.005919	0.006916	0.006831	0.005525	0.009582	0.005841	0.001327	0.003739
Bacteria	Firmicutes	Clostridia	Clostridiales	Clostridiales Family XI. Incertae Sedis	unclassified (derived from Clostridiales Family XI. Incertae Sedis)	0.002041	0.003765	0.003178	0.008389	0.005662	0.004802	0.006237	0.006138	0.00214	0.006815	0.000663	0.005979
Bacteria	Firmicutes	Clostridia	Clostridiales	unclassified (derived from Clostridiales)	unclassified (derived from Clostridiales)	0.025346	0.053646	0.024366	0.052432	0.031912	0.029007	0.029106	0.014528	0.012001	0.010709	0.002749	0.026241
Bacteria	Proteobacteria	Deltaproteobacteria	unclassified (derived from Deltaproteobacteria)	unclassified (derived from Deltaproteobacteria)	unclassified (derived from Deltaproteobacteria)	0.602	1.174555	0.926976	1.156657	2.412962	2.15535	1.095631	0.50294	0.25937	0.443951	0.10634	1.038025
Bacteria	Elusimicrobia	unclassified (derived from Elusimicrobia)	unclassified (derived from Elusimicrobia)	unclassified (derived from Elusimicrobia)	unclassified (derived from Elusimicrobia)	0.010717	0.020705	0.021188	0.01573	0.032684	0.021899	0.021978	0.010435	0.009303	0.01363	0.00218	0.014875
Bacteria	Firmicutes	Erysipelotrichi	Erysipelotrichales	Erysipelotrichaceae	unclassified (derived from Erysipelotrichaceae)	0.01667	0.042352	0.01801	0.028313	0.023934	0.023628	0.035046	0.023531	0.014699	0.02726	0.011184	0.023737
Bacteria	Bacteroidetes	Flavobacteria	unclassified (derived from Flavobacteria)	unclassified (derived from Flavobacteria)	unclassified (derived from Flavobacteria)	0.122986	0.133643	0.144078	0.173027	0.061251	0.121599	0.114048	0.216072	0.224018	0.946317	0.14771	0.448033
Bacteria	Bacteroidetes	Flavobacteria	Flavobacteriales	Flavobacteriaceae	unclassified (derived from Flavobacteriaceae)	0.034361	0.043293	0.044495	0.0388	0.021875	0.032273	0.039798	0.057701	0.067726	0.175244	0.017865	0.123164
Bacteria	Bacteroidetes	Flavobacteria	Flavobacteriales	unclassified (derived from Flavobacteriales)	unclassified (derived from Flavobacteriales)	0.117882	0.145878	0.115475	0.13947	0.076434	0.152911	0.135729	0.23326	0.241508	4.495979	0.680167	1.016446
Bacteria	Proteobacteria	Gammaproteobacteria	unclassified (derived from Gammaproteobacteria)	unclassified (derived from Gammaproteobacteria)	unclassified (derived from Gammaproteobacteria)	0.767511	0.701157	0.911085	0.835771	0.979494	1.266509	1.496283	1.762542	0.795692	0.841171	1.367253	1.09843
Bacteria	Firmicutes	Clostridia	Clostridiales	Lachnospiraceae	unclassified (derived from Lachnospiraceae)	0.012928	0.02447	0.019069	0.016778	0.012353	0.015944	0.016038	0.013504	0.007349	0.008762	0.001469	0.012338
Bacteria	Proteobacteria	Betaproteobacteria	Methylophilales	unclassified (derived from Methylophilales)	unclassified (derived from Methylophilales)	0.006294	0.010353	0.008475	0.011535	0.010037	0.010373	0.010098	0.009821	0.007349	0.014604	0.003554	0.009488
Bacteria	Verrucomicrobia	Opitutae	unclassified (derived from Opitutae)	Opitutaceae	unclassified (derived from Opitutaceae)	0.052222	0.087527	0.074158	0.0776	0.071545	0.083563	0.091773	0.120517	0.063261	0.046732	0.008577	0.05459
Bacteria	Proteobacteria	unclassified (derived from Proteobacteria)	unclassified (derived from Proteobacteria)	unclassified (derived from Proteobacteria)	unclassified (derived from Proteobacteria)	0	0	0	0	0.000257	0.000384	0.001485	0.000205	0.001116	0.000974	0	0.000609
Bacteria	Proteobacteria	Alphaproteobacteria	Rhodobacterales	Rhodobacteraceae	unclassified (derived from Rhodobacteraceae)	0.121455	0.095056	0.103821	0.143665	0.124303	0.165013	0.166617	0.232236	0.207924	0.128512	0.110463	0.076185
Bacteria	Proteobacteria	Alphaproteobacteria	Rhodobacterales	unclassified (derived from Rhodobacterales)	unclassified (derived from Rhodobacterales)	0.076717	0.05741	0.077336	0.068162	0.089302	0.104886	0.116127	0.178218	0.144849	0.099305	0.09672	0.073368
Bacteria	Proteobacteria	Alphaproteobacteria	Rickettsiales	unclassified (derived from Rickettsiales)	unclassified (derived from Rickettsiales)	0.008335	0.005647	0.010594	0.009438	0.019816	0.019018	0.016335	0.019029	0.010326	0.008762	0.003222	0.006062
Bacteria	Firmicutes	Clostridia	Clostridiales	Ruminococcaceae	unclassified (derived from Ruminococcaceae)	0.004763	0.010353	0.006356	0.006292	0.013382	0.008837	0.008316	0.006138	0.004931	0.008762	0.001232	0.00822

Bacteria	Thermotogae	Thermotogae (class)	Thermotogales	unclassified (derived from Thermotogales)	unclassified (derived from Thermotogales)	0.014289	0.042352	0.030723	0.04614	0.035515	0.032081	0.0297	0.02128	0.011815	0.010709	0.001801	0.024956
Bacteria	Proteobacteria	Gammaproteobacteria	Thiotrichales	unclassified (derived from Thiotrichales)	unclassified (derived from Thiotrichales)	0	0	0.001059	0	0.000257	0.001153	0	0.000614	0	0	0	9.88E-05
Bacteria	Verrucomicrobia	Verrucomicrobiae	Verrucomicrobiales	Verrucomicrobia subdivision 3	unclassified (derived from Verrucomicrobia subdivision 3)	0.222667	0.401871	0.444948	0.489718	0.227245	0.217648	0.182655	0.178628	0.406172	0.216134	0.024358	0.380298
Bacteria	Verrucomicrobia	Verrucomicrobiae	Verrucomicrobiales	unclassified (derived from Verrucomicrobiales)	unclassified (derived from Verrucomicrobiales)	0.121795	0.141172	0.15891	0.182465	0.169854	0.204009	0.199584	0.33618	0.181131	0.105146	0.02758	0.132026
Bacteria	Proteobacteria	Gammaproteobacteria	Vibrionales	Vibrionaceae	unclassified (derived from Vibrionaceae)	0.031299	0.038587	0.024366	0.013632	0.038089	0.048793	0.046332	0.046857	0.041213	0.032128	0.14046	0.049961
Bacteria	Proteobacteria	Gammaproteobacteria	Vibrionales	unclassified (derived from Vibrionales)	unclassified (derived from Vibrionales)	0.018031	0.016941	0.014832	0.014681	0.011838	0.014407	0.014553	0.015551	0.00921	0.023366	0.046867	0.018136

Comparative mangrove metagenome reveals global prevalence of heavy metals and antibiotic resistome across different ecosystems

Madangchanok Imchen[1#], Ranjith Kumavath[1#]*, Debmalya Barh[2,3], Aline Vaz[4], Aristóteles Góes-Neto[4], Sandeep Tiwari[5], Preetam Ghosh[6], Alice R. Wattam[7], Vasco Azevedo[5]

Supplementary data 2: Functional level classifion of mangrove sediments from Brazil, Saudi Arabai and Kerala. The fonts in green color indicates the functions present in all samples, fonts in black indicates functions present in more than 1 sample but not in all samples and font in blue indicates functions present only in one sample.

level1	level2	level3	function	BRMgv-1	BRMgv-2	BrMgv-3	BrMgv-4	RSMgr01	RSMgr02	RSMgr03	RSMgr04	MG_KAY	MG_VPM	MG_BNH	MG_KMA
Fatty Acids, Lipids, and Isoprenoids	Fatty acids	Fatty Acid Biosynthesis FASII.	(3R)-hydroxymyristoyl-[acyl carrier protein] dehydratase (EC 4.2.1.-)	0.032379	0.074645	0.056206	0.054781	0.032171	0.036528	0.053434	0.047344	0.039848	0.143181	0.037957	0.063575273
Amino Acids and Derivatives	Branched-chain amino acids	Branched-Chain Amino Acid Biosynthesis	(R)-citramalate synthase (EC 2.3.1.182)	0.064757	0.108575	0.06898	0.117032	0.110301	0.08231	0.072208	0.068275	0.043133	0.048461	0.002445	0.058448235
Respiration	Electron donating reactions	Hydrogenases	[Ni/Fe] hydrogenase, group 1, large subunit	0.028201	0.018096	0.012774	0.02739	0.048585	0.031171	0.022385	0.007475	0.004598	0.006608	0.039937	0.025595752
Respiration	Electron donating reactions	Hydrogenases	[Ni/Fe] hydrogenase, group 1, small subunit	0.005919	0.013572	0.012774	0.01494	0.013131	0.016559	0.009387	0.006479	0.002846	0.002203	0.003842	0.014828972
Protein Metabolism	Protein processing and modification	G3E family of P-loop GTPases (metallocenter biosynthesis)	[NiFe] hydrogenase metallocenter assembly protein HypD	0.051876	0.058812	0.061315	0.122012	0.055151	0.056984	0.063543	0.037875	0.033499	0.03965	0.084997	0.066927567
Respiration	Electron donating reactions	NiFe hydrogenase maturation	[NiFe] hydrogenase metallocenter assembly protein HypE	0.040038	0.070121	0.038322	0.044821	0.063686	0.072082	0.051268	0.031895	0.02693	0.01542	0.065669	0.055095941
Respiration	Electron donating reactions	NiFe hydrogenase maturation	[NiFe] hydrogenase metallocenter assembly protein HypF	0.04909	0.128933	0.117521	0.097112	0.086665	0.102765	0.079429	0.046846	0.040724	0.004406	0.006055	0.07982404
Respiration	Electron donating reactions	NiFe hydrogenase maturation	[NiFe] hydrogenase nickel incorporation-associated protein HypB	0.040038	0.047502	0.015329	0.064741	0.040706	0.036041	0.033216	0.012459	0.017735	0.059475	0.05682	0.041213499
Clustering-based subsystems	NULL	CBSS-312309.3.peg.1965	[Protein-PII] uridylyltransferase (EC 2.7.7.59)	0.071372	0.063336	0.084308	0.067231	0.085352	0.082797	0.070042	0.089207	0.111225	0.022028	0.199102	0.067952975
Clustering-based subsystems	NULL	Glycogen metabolism cluster	1,4-alpha-glucan (glycogen) branching enzyme, GH-13-type (EC 2.4.1.18)	0.113499	0.108575	0.222268	0.149402	0.146412	0.123708	0.111923	0.088708	0.136404	0.140978	0.043313	0.140125896
Cofactors, Vitamins, Prosthetic Groups, Pigments	Quinone cofactors	Menaquinone and Phylloquinone Biosynthesis	1,4-dihydroxy-2-naphthoate octaprenyltransferase (EC 2.5.1.-)	0.030638	0.03393	0.020438	0.0249	0.011818	0.025813	0.015886	0.015449	0.025836	0.013217	0.002445	0.028869169
Virulence, Disease and Defense	Adhesion	Mediator of hyperadherence YidE in Enterobacteria and its conserved region	16 kDa heat shock protein A	0.004178	0.004524	0.007664	0.01743	0.005252	0.006332	0.006499	0.017443	0.012699	0.008811	0.009315	0.004219947
Protein Metabolism	Protein biosynthesis	Ribosome biogenesis bacterial	16S rRNA processing protein RimM	0.00383	0.004524	0.00511	0.00747	0.008535	0.011689	0.009387	0.009967	0.018173	0.074895	0.012575	0.01995601
Fatty Acids, Lipids, and Isoprenoids	Phospholipids	Glycerolipid and Glycerophospholipid Metabolism in Bacteria	1-acyl-sn-glycerol-3-phosphate acyltransferase (EC 2.3.1.51)	0.022282	0.018096	0.012774	0.01494	0.027575	0.03458	0.029605	0.035384	0.03744	0.037447	0.025965	0.040424724
Miscellaneous	Plant-Prokaryote DOE project	Experimental-COG2515	1-aminocyclopropane-1-carboxylate deaminase (EC 3.5.99.7)	0.002089	0.002262	0.00511	0.00747	0.00197	0.003409	0.002166	0.004984	0.005255	0.017622	0.001863	0.019206674
Fatty Acids, Lipids, and Isoprenoids	Isoprenoids	Isoprenoid Biosynthesis	1-deoxy-D-xylulose 5-phosphate reductoisomerase (EC 1.1.1.267)	0.071024	0.079169	0.086863	0.089641	0.077474	0.075491	0.056323	0.070767	0.063714	0.057273	0.021075	0.072803942
Cofactors, Vitamins, Prosthetic Groups, Pigments	Pyridoxine	Pyridoxin (Vitamin B6) Biosynthesis	1-deoxy-D-xylulose 5-phosphate synthase (EC 2.2.1.7)	0.226303	0.147029	0.158398	0.102092	0.126715	0.166568	0.153082	0.136551	0.139688	0.105734	0.096058	0.136970796
Clustering-based subsystems	Isoprenoid/cell wall biosynthesis: PREDICTED UNDECAPRENYL DIPHOSPHATE PHOSPHATASE	CBSS-83331.1.peg.3039	1-hydroxy-2-methyl-2-(E)-butenyl 4-diphosphate synthase (EC 1.17.7.1)	0.101662	0.115361	0.097082	0.074701	0.086009	0.085719	0.084484	0.087213	0.07269	0.063881	0.012109	0.075761848
Amino Acids and Derivatives	Proline and 4-hydroxyproline	Proline, 4-hydroxyproline uptake and utilization	1-pyrroline-4-hydroxy-2-carboxylate deaminase (EC 3.5.4.22)	0.002785	0.006786	0.012774	0.00498	0.000657	0.000974	0.002888	0.006977	0.007225	0.006608	0.001747	0.008676526
Amino Acids and Derivatives	Lysine, threonine, methionine, and cysteine	Lysine Biosynthesis DAP Pathway	2,3,4,5-tetrahydropyridine-2,6-dicarboxylate N-acetyltransferase (EC 2.3.1.89)	0.008704	0.009048	0.028103	0.01494	0.018384	0.018508	0.009387	0.014951	0.011823	0.041853	0.000349	0.01806295
Amino Acids and Derivatives	Lysine, threonine, methionine, and cysteine	Lysine Biosynthesis DAP Pathway	2,3,4,5-tetrahydropyridine-2,6-dicarboxylate N-succinyltransferase (EC 2.3.1.117)	0.065454	0.031668	0.017884	0.042331	0.039393	0.03458	0.036826	0.055318	0.050796	0.041853	0.074168	0.044013651
Carbohydrates	Central carbohydrate metabolism	Glycolysis and Gluconeogenesis	2,3-bisphosphoglycerate-independent phosphoglycerate mutase (EC 5.4.2.1)	0.105144	0.101789	0.066425	0.104582	0.133281	0.115915	0.126365	0.136551	0.090863	0.09472	0.088723	0.129990136
Nucleosides and Nucleotides	Pyrimidines	pyrimidine conversions	2',3'-cyclic-nucleotide 2'-phosphodiesterase (EC 3.1.4.16)	0.01323	0.02262	0.015329	0.02739	0.007222	0.007793	0.009387	0.014951	0.022333	0.385488	0.022588	0.036559726
Amino Acids and Derivatives	Lysine, threonine, methionine, and cysteine	Methionine Salvage	2,3-diketo-5-methylthiopentyl-1-phosphate enolase	0.002785	0.002262	0.002555	0.00996	0.002626	0.000974	0.000722	0.001993	0.005255	0.002203	0.004192	0.002760713
Fatty Acids, Lipids, and Isoprenoids	Fatty acids	Fatty acid degradation regulons	2,4-dienoyl-CoA reductase [NADPH] (EC 1.3.1.34)	0.058142	0.097265	0.091973	0.057271	0.072221	0.075978	0.067154	0.063292	0.074661	0.03965	0.289687	0.067834659
Metabolism of Aromatic Compounds	Metabolism of central aromatic intermediates	4-Hydroxyphenylacetic acid catabolic pathway	2,4-dihydroxyhept-2-ene-1,7-dioic acid aldolase (EC 4.1.2.-)	0.010445	0.013572	0.022993	0.00498	0.01904	0.010228	0.008665	0.00598	0.01029	0.011014	0.033882	0.009938566
RNA Metabolism	RNA processing and modification	rRNA modification Bacteria	23S rRNA (guanosine-2'-O-) -methyltransferase rlmB (EC 2.1.1.-)	0.016015	0.01131	0.043432	0.0249	0.016414	0.016072	0.025273	0.023921	0.026274	0.008811	0.01665	0.029105801
RNA Metabolism	RNA processing and modification	RNA methylation	23S rRNA (Uracil-5-) -methyltransferase RumA (EC 2.1.1.-)	0.017408	0.01131	0.015329	0.02241	0.011818	0.011689	0.014442	0.018938	0.021238	0.004406	0.003493	0.014355707
Amino Acids and Derivatives	Alanine, serine, and glycine	Glycine Biosynthesis	2-amino-3-ketobutyrate coenzyme A ligase (EC 2.3.1.29)	0.084254	0.124409	0.099637	0.089641	0.050555	0.086693	0.067876	0.080236	0.079478	0.129965	0.089887	0.096743266
Miscellaneous	Plant-Prokaryote DOE project	COG4319	2-amino-4-hydroxy-6-hydroxymethyldihydropteridine pyrophosphokinase (EC 2.7.6.3)	0.021934	0.029406	0.020438	0.01494	0.018384	0.027274	0.019496	0.019436	0.02912	0.019825	0.042848	0.031748198
Phosphorus Metabolism	NULL	Phosphoenolpyruvate phosphomutase	2-aminoethylphosphonate:pyruvate aminotransferase (EC 2.6.1.37)	0.012882	0.002262	0.007664	0.00249	0.007879	0.01315	0.014442	0.006977	0.008539	0.03965	0.071723	0.004693212
Clustering-based subsystems	NULL	CBSS-211586.1.peg.3133	2-C-methyl-D-erythritol 2,4-cyclodiphosphate synthase (EC 4.6.1.12)	0.035164	0.03393	0.022993	0.034861	0.022979	0.029222	0.02094	0.022925	0.020143	0.011014	0.006287	0.022361774
Carbohydrates	Monosaccharides	D-Galacturonate and D-Glucuronate Utilization	2-dehydro-3-deoxygluconate kinase (EC 2.7.1.45)	0.013578	0.063336	0.051096	0.042331	0.02101	0.02143	0.014442	0.022925	0.024522	0.002203	0.001164	0.027725445
Carbohydrates	Monosaccharides	D-Galacturonate and D-Glucuronate Utilization	2-dehydro-3-deoxyphosphogluconate aldolase (EC 4.1.2.14)	0.014971	0.01131	0.020438	0.01992	0.013788	0.011202	0.01372	0.019436	0.020143	0.008811	0.023985	0.016564277
Cofactors, Vitamins, Prosthetic Groups, Pigments	Coenzyme A	Coenzyme A Biosynthesis	2-dehydropantoate 2-reductase (EC 1.1.1.169)	0.040734	0.015834	0.025548	0.042331	0.028232	0.044321	0.014442	0.021928	0.033718	0.026433	0.013157	0.024373151
Carbohydrates	Monosaccharides	D-Galacturonate and D-Glucuronate Utilization	2-deoxy-D-gluconate 3-dehydrogenase (EC 1.1.1.125)	0.011141	0.013572	0.015329	0.01494	0.013788	0.020943	0.007943	0.011961	0.021019	0.01542	0.001863	0.017234736
Clustering-based subsystems	Hypothetical in Lysine biosynthetic cluster	CBSS-323850.3.peg.3269	2-haloalkanoic acid dehalogenase (EC 3.8.1.2)	0.002089	0.006786	0.00511	0.00747	0.001313	0.003409	0.002166	0.006977	0.005912	0.002203	0.000233	0.008992036
Carbohydrates	CO2 fixation	Photorespiration (oxidative C2 cycle)	2-hydroxy-3-oxopropionate reductase (EC 1.1.1.60)	0.03029	0.063336	0.015329	0.039841	0.040706	0.052113	0.049824	0.042859	0.057145	0.011014	0.066251	0.032260902
Metabolism of Aromatic Compounds	Peripheral pathways for catabolism of aromatic compounds	Naphtalene and antracene degradation	2-hydroxychromene-2-carboxylate isomerase	0.002785	0.013572	0.012774	0.00996	0.011818	0.012663	0.014442	0.019436	0.016202	0.01542	0.001281	0.00378612
Amino Acids and Derivatives	Aromatic amino acids and derivatives	Aromatic amino acid degradation	2-hydroxyhepta-2,4-diene-1,7-dioate isomerase (EC 5.3.3.-)	0.000696	0.01131	0.002555	0.00747	0.003283	0.000974	0.005777	0.001495	0.006349	0.013217	0.001397	0.00378612
Amino Acids and Derivatives	Branched-chain amino acids	Leucine Biosynthesis	2-isopropylmalate synthase (EC 2.3.3.13)	0.162938	0.171911	0.173726	0.144422	0.212067	0.167055	0.174744	0.114125	0.125457	0.167412	0.231936	0.1602791
Amino Acids and Derivatives	Aromatic amino acids and derivatives	Common Pathway For Synthesis of Aromatic Compounds (DAHP synthase to chorismate)	2-keto-3-deoxy-D-arabino-heptulosonate-7-phosphate synthase I alpha (EC 2.5.1.54)	0.021586	0.020358	0.045986	0.039841	0.038737	0.043834	0.03466	0.035882	0.025836	0.033042	0.056703	0.033838452
Amino Acids and Derivatives	Aromatic amino acids and derivatives	Chorismate Synthesis	2-keto-3-deoxy-D-arabino-heptulosonate-7-phosphate synthase I beta (EC 2.5.1.54)	0.061972	0.090479	0.056206	0.069721	0.075504	0.071108	0.0556	0.047344	0.037221	0.033042	0.014787	0.063930222
Amino Acids and Derivatives	Aromatic amino acids and derivatives	Chorismate Synthesis	2-keto-3-deoxy-D-arabino-heptulosonate-7-phosphate synthase II (EC 2.5.1.54)	0.021238	0.029406	0.053651	0.01743	0.020353	0.030684	0.025273	0.046348	0.048825	0.028636	0.110729	0.028238149
Cell Wall and Capsule	Gram-Negative cell wall components	KDO2-Lipid A biosynthesis	2-Keto-3-deoxy-D-manno-octulosonate-8-phosphate synthase (EC 2.5.1.55)	0.060231	0.05655	0.06387	0.034861	0.07813	0.075491	0.088816	0.072262	0.05605	0.154195	0.062525	0.069412209
Carbohydrates	Organic acids	Methylcitrate cycle	2-methylcitrate dehydratase (EC 4.2.1.79)	0.0094	0.015834	0.00511	0.01245	0.015101	0.012663	0.018774	0.017941	0.028244	0.037447	0.002445	0.006152446
Carbohydrates	Organic acids	Methylcitrate cycle	2-methylcitrate synthase (EC 2.3.3.5)	0.059883	0.02262	0.020438	0.00747	0.012475	0.011202	0.010831	0.018938	0.031528	0.006608	0.078826	0.016209328
Carbohydrates	Organic acids	Propionate-CoA to Succinate Module	2-methylisocitrate dehydratase (EC 4.2.1.99)	0.020193	0.013572	0.017884	0.01245	0.024949	0.018508	0.030328	0.022925	0.048825	0.022028	0.153809	0.022401213
Cofactors, Vitamins, Prosthetic Groups, Pigments	Quinone cofactors	Ubiquinone Biosynthesis	2-octaprenyl-3-methyl-6-methoxy-1,4-benzoquinol hydroxylase (EC 1.14.13.-)	0.03029	0.018096	0.010219	0.02241	0.013131	0.018508	0.016608	0.025915	0.035031	0.017622	0.084415	0.025161926

Carbohydrates	Central carbohydrate metabolism	Dehydrogenase complexes	2-oxoglutarate dehydrogenase E1 component (EC 1.2.4.2)	0.24754	0.085955	0.160952	0.134462	0.10111	0.148547	0.148749	0.174426	0.179098	0.224684	0.212841	0.151523697
Metabolism of Aromatic Compounds	Metabolism of central aromatic intermediates	Central meta-cleavage pathway of aromatic compound degradation	2-polyprenylphenol hydroxylase and related flavodoxin oxidoreductases	0.030986	0.024882	0.010219	0.00747	0.009848	0.018508	0.007943	0.006479	0.007225	0.006608	0.005589	0.014316268
Cofactors, Vitamins, Prosthetic Groups, Pigments	Quinone cofactors	Menaquinone and Phylloquinone Biosynthesis	2-succinyl-5-enolpyruvyl-6-hydroxy-3-cyclohexene-1-carboxylic-acid synthase (EC 2.2.1.9)	0.014274	0.009048	0.015329	0.01245	0.007879	0.010715	0.015164	0.013954	0.014232	0.011014	0.003959	0.013251422
Sulfur Metabolism	Inorganic sulfur assimilation	Inorganic Sulfur Assimilation	3'(2'),5'-bisphosphate nucleotidase (EC 3.1.3.7)	0.023327	0.020358	0.035767	0.00996	0.014444	0.024839	0.029605	0.032393	0.032404	0.006608	0.027595	0.023899886
Cofactors, Vitamins, Prosthetic Groups, Pigments	Riboflavin, FMN, FAD	riboflavin to FAD	3,4-dihydroxy-2-butanone 4-phosphate synthase	0.037601	0.065598	0.040877	0.059761	0.03808	0.040911	0.056323	0.03937	0.043789	0.066084	0.035396	0.050797116
Amino Acids and Derivatives	Aromatic amino acids and derivatives	Common Pathway For Synthesis of Aromatic Compounds (DAHP synthase to chorismate)	3,7-dideoxy-D-threo-hepto-2, 6-diulosonate synthase (EC 4.2.3.-)	0.003133	0.002262	0.002555	0.00747	0.003283	0.001461	0.00361	0.000997	0.001533	0.013217	0.000349	0.00347061
Amino Acids and Derivatives	Aromatic amino acids and derivatives	Chorismate Synthesis	3-dehydroquinate dehydratase II (EC 4.2.1.10)	0.039342	0.04524	0.028103	0.0249	0.027575	0.038476	0.03466	0.033889	0.029777	0.059475	0.024451	0.031945392
Amino Acids and Derivatives	Aromatic amino acids and derivatives	Chorismate Synthesis	3-dehydroquinate synthase (EC 4.2.3.4)	0.049438	0.054288	0.06898	0.077191	0.059747	0.060393	0.056323	0.055816	0.052985	0.041853	0.061244	0.061839968
Miscellaneous	Plant-Prokaryote DOE project	Experimental-Ubiquinone BiosynthesisVDC	3-demethylubiquinone-9 3-methyltransferase (EC 2.1.1.64)	0.06232	0.040716	0.033212	0.044821	0.026919	0.034093	0.040437	0.051829	0.038754	0.004406	0.03854	0.027212741
Cell Wall and Capsule	Gram-Negative cell wall components	KDO2-Lipid A biosynthesis	3-deoxy-D-manno-octulosonate 8-phosphate phosphatase (EC 3.1.3.45)	0.029593	0.002262	0.007664	0.01245	0.011818	0.018995	0.014442	0.012459	0.01029	0.008811	0.014321	0.027922639
Miscellaneous	Plant-Prokaryote DOE project	COG0451	3-deoxy-D-manno-octulosonic-acid transferase (EC 2.-.-.-)	0.015319	0.03393	0.022993	0.0249	0.034797	0.027761	0.024551	0.0304	0.036345	0.013217	0.008383	0.032379218
Cell Wall and Capsule	Gram-Negative cell wall components	KDO2-Lipid A biosynthesis	3-deoxy-manno-octulosonate cytidylyltransferase (EC 2.7.7.38)	0.024371	0.042978	0.040877	0.049801	0.047929	0.0526	0.058489	0.061797	0.038973	0.061678	0.039122	0.049298444
Amino Acids and Derivatives	Branched-chain amino acids	Isoleucine degradation	3-hydroxyacyl-CoA dehydrogenase (EC 1.1.1.35)	0.06998	0.083693	0.091973	0.052291	0.082726	0.09546	0.08665	0.099672	0.141878	0.237901	0.088257	0.096861582
Carbohydrates	Organic acids	Isobutyryl-CoA to Propionyl-CoA Module	3-hydroxyacyl-CoA dehydrogenase [isoleucine degradation] (EC 1.1.1.35)	0.003133	0.002262	0.007664	0.00747	0.000657	0.000974	0.002888	0.005482	0.007006	0.006608	0.017349	0.003864998
Carbohydrates	Fermentation	Acetone Butanol Ethanol Synthesis	3-hydroxybutyryl-CoA dehydratase (EC 4.2.1.55)	0.006267	0.020358	0.030658	0.02988	0.03808	0.033119	0.032494	0.020931	0.021019	0.008811	0.000582	0.022716723
Carbohydrates	Fermentation	Acetone Butanol Ethanol Synthesis	3-hydroxybutyryl-CoA dehydrogenase (EC 1.1.1.157)	0.041083	0.047502	0.040877	0.054781	0.066969	0.07403	0.071486	0.065285	0.054737	0.05507	0.049252	0.060262418
Carbohydrates	Fermentation	Acetyl-CoA fermentation to Butyrate	3-hydroxybutyryl-CoA epimerase (EC 5.1.2.3)	0.038646	0.006786	0.017884	0.02241	0.011818	0.014124	0.018052	0.034387	0.04576	0.013217	0.047156	0.016761471
Fatty Acids, Lipids, and Isoprenoids	Fatty acids	Fatty Acid Biosynthesis FASII	3-hydroxydecanoyl-[acyl-carrier-protein] dehydratase (EC 4.2.1.60)	0.069632	0.020358	0.007664	0.00996	0.009848	0.015585	0.010109	0.021928	0.013575	0.019825	0.032019	0.014513462
Amino Acids and Derivatives	Branched-chain amino acids	Valine degradation	3-hydroxyisobutyrate dehydrogenase (EC 1.1.1.31)	0.012186	0.020358	0.020438	0.00996	0.013788	0.023378	0.023107	0.025915	0.027587	0.002203	0.049252	0.011042851
Amino Acids and Derivatives	Branched-chain amino acids	Branched-Chain Amino Acid Biosynthesis	3-isopropylmalate dehydratase large subunit (EC 4.2.1.33)	0.11524	0.160601	0.140514	0.164343	0.157573	0.139293	0.122032	0.117115	0.112758	0.226887	0.140768	0.137207429
Amino Acids and Derivatives	Branched-chain amino acids	Branched-Chain Amino Acid Biosynthesis	3-isopropylmalate dehydratase small subunit (EC 4.2.1.33)	0.051527	0.074645	0.066425	0.054781	0.045302	0.045295	0.0556	0.052826	0.040943	0.066084	0.041683	0.048825179
Amino Acids and Derivatives	Branched-chain amino acids	Branched-Chain Amino Acid Biosynthesis	3-isopropylmalate dehydrogenase (EC 1.1.1.85)	0.097136	0.117623	0.125185	0.117032	0.12015	0.126143	0.107591	0.095685	0.093709	0.140978	0.089305	0.118395142
Amino Acids and Derivatives	Branched-chain amino acids	Isoleucine degradation	3-ketoacyl-CoA thiolase (EC 2.3.1.16)	0.065454	0.088217	0.074089	0.074701	0.083383	0.069647	0.075819	0.083226	0.092614	0.03965	0.095592	0.068702311
Amino Acids and Derivatives	Branched-chain amino acids	Branched chain amino acid degradation regulons	3-ketoacyl-CoA thiolase [isoleucine degradation] (EC 2.3.1.16)	0.011837	0.004524	0.00511	0.00498	0.002626	0.002922	0.002166	0.004984	0.020581	0.002203	0.050416	0.006389078
Clustering-based subsystems	Clustering-based subsystems	CBSS-280355.3.peg.2835	3-methyl-2-oxobutanoate hydroxymethyltransferase (EC 2.1.2.11)	0.067194	0.081431	0.056206	0.069721	0.067625	0.064289	0.076541	0.073757	0.064808	0.041853	0.030273	0.071817973
Fatty Acids, Lipids, and Isoprenoids	Fatty acids	Fatty Acid Biosynthesis FASII	3-oxoacyl-[acyl-carrier protein] reductase (EC 1.1.1.100)	0.11907	0.183221	0.176281	0.214143	0.168078	0.150008	0.166079	0.147515	0.230551	0.132167	0.12668	0.171834655
Fatty Acids, Lipids, and Isoprenoids	Fatty acids	Fatty Acid Biosynthesis FASII	3-oxoacyl-[acyl-carrier-protein] synthase, KASI (EC 2.3.1.41)	0.014623	0.006786	0.038322	0.02241	0.02101	0.024352	0.036826	0.029403	0.03328	0.019825	0.110729	0.027370496
Fatty Acids, Lipids, and Isoprenoids	Fatty acids	Fatty Acid Biosynthesis FASII	3-oxoacyl-[acyl-carrier-protein] synthase, KASII (EC 2.3.1.41)	0.188005	0.167387	0.153288	0.139442	0.15823	0.140755	0.155248	0.12459	0.134214	0.145384	0.13483	0.146869923
Fatty Acids, Lipids, and Isoprenoids	Fatty acids	Fatty Acid Biosynthesis FASII	3-oxoacyl-[acyl-carrier-protein] synthase, KASIII (EC 2.3.1.41)	0.096092	0.128933	0.086863	0.124502	0.100453	0.111045	0.093871	0.111134	0.100934	0.202657	0.007917	0.136418653
Metabolism of Aromatic Compounds	Metabolism of central aromatic intermediates	Protocatechuate branch of beta-ketoadipate pathway	3-oxoadipate CoA-transferase subunit A (EC 2.8.3.6)	0.003133	0.02262	0.010219	0.034861	0.010505	0.007793	0.009387	0.00897	0.01445	0.008811	0.001979	0.005324232
Metabolism of Aromatic Compounds	Peripheral pathways for catabolism of aromatic compounds	Chloroaromatic degradation pathway	3-oxoadipate CoA-transferase subunit B (EC 2.8.3.6)	0.009748	0.015834	0.010219	0.00249	0.013788	0.013637	0.006499	0.008472	0.014013	0.006608	0.000815	0.006231323
Miscellaneous	Plant-Prokaryote DOE project	Experimental-Ubiquinone BiosynthesisVDC	3-polyprenyl-4-hydroxybenzoate carboxy-lyase (EC 4.1.1.-)	0.107581	0.063336	0.084308	0.052291	0.082069	0.0789	0.088816	0.053823	0.088673	0.052867	0.139371	0.082939702
Cofactors, Vitamins, Prosthetic Groups, Pigments	Quinone cofactors	Menaquinone Biosynthesis via Futalosine -- gjo	3-polyprenyl-4-hydroxybenzoate carboxy-lyase UbiX (EC 4.1.1.-)	0.081469	0.015834	0.035767	0.00498	0.016414	0.016072	0.024551	0.017443	0.018173	0.028636	0.006287	0.022440652
RNA Metabolism	RNA processing and modification	RNA processing and degradation, bacterial	3'-to-5' exoribonuclease RNase R	0.063017	0.054288	0.051096	0.084661	0.092574	0.069647	0.085206	0.12459	0.118231	0.138776	0.168014	0.115516113
RNA Metabolism	RNA processing and modification	RNA processing and degradation, bacterial	3'-to-5' oligoribonuclease (orn)	0.041431	0.013572	0.015329	0.02739	0.011818	0.012663	0.012998	0.015449	0.016859	0.002203	0.039587	0.016209328
Clustering-based subsystems	NULL	Glycogen metabolism cluster	4-alpha-glucanotransferase (amylomaltase) (EC 2.4.1.25)	0.066498	0.067859	0.079199	0.059761	0.054494	0.04286	0.038993	0.03638	0.044008	0.008811	0.017232	0.044723548
Amino Acids and Derivatives	Arginine; urea cycle, polyamines	Polyamine Metabolism	4-aminobutyraldehyde dehydrogenase (EC 1.2.1.19)	0.019497	0.002262	0.010219	0.00498	0.003939	0.002435	0.00361	0.00299	0.008101	0.01542	0.000931	0.003076223
Carbohydrates	Monosaccharides	D-Galacturonate and D-Glucuronate Utilization	4-deoxy-L-threo-5-hexosulose-uronate ketol-isomerase (EC 5.3.1.17)	0.002785	0.01131	0.020438	0.01245	0.006566	0.007793	0.004333	0.00299	0.007444	0.026433	0.001281	0.008321577
Fatty Acids, Lipids, and Isoprenoids	Isoprenoids	polyprenyl synthesis	4-diphosphocytidyl-2-C-methyl-D-erythritol kinase (EC 2.7.1.148)	0.020889	0.01131	0.028103	0.01245	0.032828	0.02143	0.022385	0.03339	0.021238	0.024231	0.001863	0.024333712
Sulfur Metabolism	Inorganic sulfur assimilation	Inorganic Sulfur Assimilation	4Fe-4S ferredoxin, iron-sulfur binding	0.048394	0.052026	0.048541	0.054781	0.047929	0.051626	0.052712	0.041862	0.030653	0.070489	0.040403	0.057738337
Nitrogen Metabolism	NULL	Nitrogen fixation	4Fe-4S ferredoxin, nitrogenase-associated	0.003482	0.009048	0.012774	0.00249	0.006566	0.002435	0.001444	0.001495	0.001095	0.002203	0.015369	0.003194539
Metabolism of Aromatic Compounds	Peripheral pathways for catabolism of aromatic compounds	Biphenyl Degradation	4-hydroxy-2-oxovalerate aldolase (EC 4.1.3.-)	0.01323	0.018096	0.022993	0.00747	0.020353	0.011202	0.010831	0.010964	0.008977	0.033042	0.186061	0.01743193
Fatty Acids, Lipids, and Isoprenoids	Isoprenoids	polyprenyl synthesis	4-hydroxy-3-methylbut-2-enyl diphosphate reductase (EC 1.17.1.2)	0.042823	0.054288	0.061315	0.059761	0.063029	0.058932	0.049102	0.06678	0.068749	0.041853	0.022821	0.053833901
Miscellaneous	Plant-Prokaryote DOE project	COG1836	4-hydroxybenzoate polyprenyltransferase (EC 2.5.1.-)	0.042127	0.018096	0.030658	0.0249	0.029545	0.032632	0.03466	0.03937	0.03744	0.013217	0.038423	0.032694728
Membrane Transport	NULL	Ton and Tol transport systems	4-hydroxybenzoyl-CoA thioesterase family active site	0.005222	0.013572	0.017884	0.02241	0.016414	0.012663	0.02094	0.020433	0.012699	0.002203	0.011411	0.018575654
Metabolism of Aromatic Compounds	Metabolism of central aromatic intermediates	Homogentisate pathway of aromatic compound degradation	4-hydroxyphenylpyruvate dioxygenase (EC 1.13.11.27)	0.018801	0.02262	0.017884	0.047311	0.026262	0.030196	0.03466	0.042361	0.072909	0.074895	0.120742	0.032773605
Amino Acids and Derivatives	Proline and 4-hydroxyproline	Proline, 4-hydroxyproline uptake and utilization	4-hydroxyproline epimerase (EC 5.1.1.8)	0.018104	0.006786	0.015329	0.01245	0.003283	0.005357	0.00361	0.007974	0.009415	0.050664	0.000466	0.009899127
Clustering-based subsystems	NULL	EC49-61	4-hydroxythreonine-4-phosphate dehydrogenase (EC 1.1.1.262)	0.053268	0.063336	0.05876	0.062251	0.044646	0.054548	0.071486	0.064787	0.04357	0.107937	0.061826	0.065073946
Protein Metabolism	Protein processing and modification	N-linked Glycosylation in Bacteria	4-keto-6-deoxy-N-Acetyl-D-hexosaminyl-(Lipid carrier) aminotransferase	0.010097	0.02262	0.017884	0.032371	0.015757	0.0263	0.023107	0.015948	0.016859	0.037447	0.002562	0.037821766
Amino Acids and Derivatives	Lysine, threonine, methionine, and cysteine	Methionine Biosynthesis	5,10-methylenetetrahydrofolate reductase (EC 1.5.1.20)	0.111411	0.122147	0.130295	0.134462	0.145099	0.158288	0.06932	0.085718	0.072033	0.050664	0.042731	0.102540763
Cofactors, Vitamins, Prosthetic Groups, Pigments	Riboflavin, FMN, FAD	Riboflavin, FMN and FAD metabolism	5-amino-6-(5-phosphoribosylamino)uracil reductase (EC 1.1.1.193)	0.023327	0.061074	0.081754	0.049801	0.060403	0.047243	0.044769	0.068275	0.052766	0.052867	0.035745	0.052492983
Cofactors, Vitamins, Prosthetic Groups, Pigments	Tetrapyrroles	Heme and Siroheme Biosynthesis	5-aminolevulinate synthase (EC 2.3.1.37)	0.010793	0.004524	0.010219	0.00249	0.015757	0.027274	0.026717	0.048839	0.026493	0.008811	0.00326	0.004101631
Metabolism of Aromatic Compounds	Metabolism of central aromatic intermediates	Central meta-cleavage pathway of aromatic compound degradation	5-carboxymethyl-2-hydroxymuconate semialdehyde dehydrogenase (EC 1.2.1.60)	0.000348	0.006786	0.010219	0.01743	0.007222	0.006332	0.00361	0.009967	0.019267	0.008811	0.15346	0.017195297
Carbohydrates	Sugar alcohols	Inositol catabolism	5-deoxy-glucuronate isomerase (EC 5.3.1.-)	0.002785	0.002262	0.007664	0.00498	0.007222	0.004383	0.002166	0.00299	0.008101	0.002203	0.000349	0.002445203

Amino Acids and Derivatives	Aromatic amino acids and derivatives	Common Pathway For Synthesis of Aromatic Compounds (DAHP synthase to chorismate)	5-Enolpyruvylshikimate-3-phosphate synthase (EC 2.5.1.19)	0.065802	0.083693	0.043432	0.057271	0.077474	0.064776	0.057045	0.081731	0.079478	0.059475	0.087675	0.074775879
Cofactors, Vitamins, Prosthetic Groups, Pigments	Folate and pterines	5-FCL-like protein	5-formyltetrahydrofolate cyclo-ligase (EC 6.3.3.2)	0.004178	0.01131	0.010219	0.00996	0.012475	0.014611	0.01372	0.016944	0.01248	0.008811	0.001048	0.010017444
Carbohydrates	Sugar alcohols	Inositol catabolism	5-keto-2-deoxygluconokinase (EC 2.7.1.92)	0.002785	0.004524	0.00511	0.00498	0.003283	0.004383	0.000722	0.00299	0.008101	0.004406	0.000699	0.002602958
Carbohydrates	Monosaccharides	D-gluconate and ketogluconates metabolism	5-keto-D-gluconate 5-reductase (EC 1.1.1.69)	0.002785	0.027144	0.012774	0.00498	0.007879	0.005357	0.005777	0.004485	0.008758	0.002203	0.000466	0.005915813
Amino Acids and Derivatives	Lysine, threonine, methionine, and cysteine	Methionine Biosynthesis	5-methyltetrahydrofolate--homocysteine methyltransferase (EC 2.1.1.13)	0.218295	0.196793	0.258035	0.231574	0.193684	0.179718	0.20435	0.242702	0.241717	0.328216	0.367465	0.261163435
Amino Acids and Derivatives	Lysine, threonine, methionine, and cysteine	Methionine Biosynthesis	5-methyltetrahydropteroyltriglutamate--homocysteine methyltransferase (EC 2.1.1.14)	0.024719	0.01131	0.020438	0.00747	0.024949	0.013637	0.026717	0.0304	0.036126	0.017622	0.06986	0.03166932
Miscellaneous	Plant-Prokaryote DOE project	Omega-amidase	5'-methylthioadenosine phosphorylase (EC 2.4.2.28)	0.047698	0.058812	0.053651	0.057271	0.046615	0.048217	0.043325	0.033889	0.034375	0.024231	0.006171	0.029973454
Nucleosides and Nucleotides	Purines	Purine conversions	5'-nucleotidase (EC 3.1.3.5)	0.013926	0.018096	0.022993	0.01494	0.027575	0.02143	0.025995	0.035384	0.040943	0.019825	0.028992	0.051507014
Nucleosides and Nucleotides	Detoxification	Housecleaning nucleoside triphosphate pyrophosphatases	5-nucleotidase SurE (EC 3.1.3.5)	0.041779	0.03393	0.015329	0.039841	0.028232	0.032632	0.02094	0.032393	0.029996	0.006608	0.023403	0.040266969
Stress Response	Oxidative stress	Glutathione: Biosynthesis and gamma-glutamyl cycle	5-oxoprolinase (EC 3.5.2.9)	0.039342	0.038454	0.06898	0.039841	0.068282	0.052113	0.081596	0.067777	0.071158	0.019825	0.00163	0.022361774
Cofactors, Vitamins, Prosthetic Groups, Pigments	Riboflavin, FMN, FAD	Riboflavin, FMN and FAD metabolism	6,7-dimethyl-8-ribityllumazine synthase (EC 2.5.1.9)	0.076595	0.03393	0.045986	0.039841	0.040706	0.03945	0.033938	0.03937	0.02912	0.037447	0.02678	0.041134621
Carbohydrates	Monosaccharides	D-Tagatose and Galactitol Utilization	6-phosphofructokinase (EC 2.7.1.11)	0.118722	0.117623	0.194165	0.144422	0.114897	0.101304	0.085206	0.10017	0.094585	0.129965	0.023054	0.113938563
Carbohydrates	Central carbohydrate metabolism	Pentose phosphate pathway	6-phosphogluconate dehydrogenase, decarboxylating (EC 1.1.1.44)	0.051179	0.074645	0.061315	0.052291	0.060403	0.059906	0.043325	0.056315	0.053204	0.026433	0.022122	0.054662114
Carbohydrates	Central carbohydrate metabolism	Pentose phosphate pathway	6-phosphogluconolactonase (EC 3.1.1.31), eukaryotic type	0.000348	0.018096	0.017884	0.00498	0.005909	0.00828	0.00361	0.003987	0.004598	0.004406	0.000233	0.004850967
Cofactors, Vitamins, Prosthetic Groups, Pigments	Coenzyme F420	Coenzyme F420 synthesis	7,8-didemethyl-8-hydroxy-5-deazariboflavin synthase subunit 1	0.001044	0.009048	0.007664	0.00747	0.001313	0.005844	0.012275	0.00598	0.01029	0.006608	0.000931	0.001932499
Cofactors, Vitamins, Prosthetic Groups, Pigments	Coenzyme F420	Coenzyme F420 synthesis	7,8-didemethyl-8-hydroxy-5-deazariboflavin synthase subunit 2	0.005222	0.002262	0.007664	0.00498	0.003283	0.006332	0.010831	0.011462	0.016859	0.01542	0.000699	0.003628365
Cofactors, Vitamins, Prosthetic Groups, Pigments	Biotin	Biotin biosynthesis	8-amino-7-oxononanoate synthase (EC 2.3.1.47)	0.101314	0.04524	0.045986	0.059761	0.047272	0.054548	0.041159	0.05781	0.051671	0.050664	0.145077	0.055293134
Miscellaneous	Plant-Prokaryote DOE project	YggW	A/G-specific adenine glycosylase (EC 3.2.2.-)	0.033075	0.040716	0.030658	0.052291	0.04005	0.041885	0.05199	0.056315	0.04992	0.022028	0.043313	0.044960181
Membrane Transport	ABC transporters	ATP-dependent efflux pump transporter Ybh	ABC transport system, permease component YbhR	0.015319	0.038454	0.012774	0.01245	0.010505	0.006332	0.005777	0.000997	0.013794	0.008811	0.001397	0.019877133
Membrane Transport	ABC transporters	ATP-dependent efflux pump transporter Ybh	ABC transport system, permease component YbhS	0.028549	0.013572	0.025548	0.01992	0.010505	0.003896	0.005777	0.002492	0.013794	0.004406	0.000116	0.021099734
Respiration	NULL	Biogenesis of c-type cytochromes	ABC transporter involved in cytochrome c biogenesis, ATPase component CcmA	0.01323	0.013572	0.00511	0.01245	0.007879	0.016072	0.012998	0.014951	0.018829	0.006608	0.009082	0.01108229
Respiration	NULL	Biogenesis of c-type cytochromes	ABC transporter involved in cytochrome c biogenesis, CcmB subunit	0.005222	0.01131	0.007664	0.00996	0.01707	0.014124	0.016608	0.011462	0.018173	0.011014	0.042615	0.013685248
Membrane Transport	ABC transporters	ATP-dependent efflux pump transporter Ybh	ABC transporter multidrug efflux pump, fused ATP-binding domains	0.054661	0.031668	0.043432	0.034861	0.010505	0.007793	0.005055	0.012957	0.027587	0.004406	0.000233	0.037585133
Amino Acids and Derivatives	Arginine; urea cycle, polyamines	Polyamine Metabolism	ABC transporter, periplasmic spermidine putrescine-binding protein PotD (TC 3.A.1.11.1)	0.022978	0.020358	0.028103	0.00747	0.022979	0.0263	0.02094	0.016446	0.031966	0.022028	0.001863	0.019522184
Clustering-based subsystems	Putative GGDEF domain protein related to agglutinin secretion	CBSS-323850.3.peg.3284	ABC transporter, transmembrane region:ABC transporter:Peptidase C39, bacteriocin processing	0.001741	0.002262	0.00511	0.00249	0.010505	0.006332	0.006499	0.008472	0.003722	0.002203	0.005589	0.015183921
Clustering-based subsystems	Cytochrome biogenesis	CBSS-196164.1.peg.1690	ABC-type multidrug transport system, ATPase component	0.052572	0.099527	0.086863	0.077191	0.051211	0.061367	0.059211	0.037377	0.098964	0.085909	0.115037	0.097295409
Clustering-based subsystems	Cytochrome biogenesis	CBSS-196164.1.peg.1690	ABC-type multidrug transport system, permease component	0.090869	0.038454	0.043432	0.039841	0.027575	0.0263	0.025273	0.030898	0.069625	0.035245	0.117831	0.066927567
Sulfur Metabolism	Organic sulfur assimilation	Alkanesulfonate assimilation	ABC-type nitrate/sulfonate/bicarbonate transport system, ATPase component	0.006963	0.013572	0.010219	0.00249	0.010505	0.010715	0.007221	0.006977	0.016202	0.011014	0.000699	0.007098976
Clustering-based subsystems	Translation	CBSS-326442.4.peg.1852	ABC-type polar amino acid transport system, ATPase component	0.002437	0.009048	0.007664	0.00747	0.007222	0.006819	0.005777	0.010964	0.019048	0.035245	0.039122	0.015420553
Membrane Transport	ABC transporters	ABC transporter tungstate (TC 3.A.1.6.2)	ABC-type tungstate transport system, periplasmic binding protein	0.02263	0.031668	0.007664	0.049801	0.031515	0.037015	0.023829	0.022426	0.024303	0.011014	0.000349	0.023584375
Membrane Transport	ABC transporters	ABC transporter tungstate (TC 3.A.1.6.2)	ABC-type tungstate transport system, permease protein	0.01706	0.006786	0.025548	0.02739	0.022323	0.027274	0.016608	0.012459	0.017954	0.019825	0.001979	0.015341676
Amino Acids and Derivatives	Aromatic amino acids and derivatives	Tryptophan catabolism	Acetaldehyde dehydrogenase (EC 1.2.1.10)	0.003133	0.009048	0.012774	0.00249	0.005909	0.008767	0.002888	0.00598	0.005036	0.004406	0.032951	0.014197952
Carbohydrates	Sugar alcohols	Ethanolamine utilization	Acetaldehyde dehydrogenase, ethanolamine utilization cluster	0.009748	0.01131	0.010219	0.00498	0.010505	0.005357	0.00361	0.007475	0.009634	0.004406	0.000349	0.008715965
Carbohydrates	Central carbohydrate metabolism	Pyruvate metabolism II: acetyl-CoA, acetogenesis from pyruvate	Acetate kinase (EC 2.7.2.1)	0.060579	0.063336	0.048541	0.054781	0.037424	0.058445	0.059211	0.044852	0.037221	0.048461	0.027129	0.082900263
Protein Metabolism	Protein processing and modification	Protein Acetylation and Deacetylation in Bacteria	Acetate permease ActP (cation/acetate symporter)	0.047001	0.027144	0.056206	0.054781	0.050555	0.053087	0.059211	0.075751	0.083419	0.048461	0.070675	0.099858928
Carbohydrates	Fermentation	Acetone Butanol Ethanol Synthesis	Acetoacetate metabolism regulatory protein AtoC	0.058839	0.02262	0.002555	0.037351	0.020353	0.029222	0.005055	0.006479	0.014669	0.002203	0.006986	0.023860447
Fatty Acids, Lipids, and Isoprenoids	NULL	Polyhydroxybutyrate metabolism	Acetoacetyl-CoA reductase (EC 1.1.1.36)	0.019497	0.013572	0.020438	0.01992	0.019697	0.024352	0.024551	0.030898	0.017297	0.057273	0.023054	0.015459992
Amino Acids and Derivatives	Branched-chain amino acids	Branched chain amino acid degradation regulons	Acetoacetyl-CoA synthetase (EC 6.2.1.16)	0.052224	0.067859	0.071534	0.062251	0.070908	0.079875	0.063543	0.0608	0.071596	0.026433	0.026663	0.053360635
Amino Acids and Derivatives	Branched-chain amino acids	HMG CoA Synthesis	Acetoacetyl-CoA synthetase [leucine] (EC 6.2.1.16)	0.007659	0.01131	0.012774	0.00249	0.006566	0.00828	0.015886	0.010964	0.012699	0.01542	0.000582	0.006783466
Carbohydrates	Central carbohydrate metabolism	Dehydrogenase complexes	Acetoin dehydrogenase E1 component alpha-subunit (EC 1.2.4.-)	0.041083	0.05655	0.066425	0.032371	0.032828	0.045295	0.039715	0.028905	0.028901	0.026433	0.066134	0.031117178
Carbohydrates	Fermentation	Acetoin, butanediol metabolism	Acetoin dehydrogenase E1 component beta-subunit (EC 1.2.4.-)	0.019845	0.054288	0.051096	0.072211	0.031515	0.040424	0.021663	0.014452	0.031309	0.028636	0.071025	0.0227956
Carbohydrates	Fermentation	Acetoin, butanediol metabolism	Acetolactate synthase large subunit (EC 2.2.1.6)	0.160501	0.171911	0.143069	0.164343	0.165452	0.168029	0.129253	0.125088	0.124362	0.143181	0.175815	0.164814557
Carbohydrates	Fermentation	Acetoin, butanediol metabolism	Acetolactate synthase small subunit (EC 2.2.1.6)	0.042127	0.031668	0.020438	0.01743	0.034797	0.028735	0.028161	0.028407	0.025836	0.048461	0.037957	0.034784982
Carbohydrates	Fermentation	Acetone Butanol Ethanol Synthesis	Acetyl-CoA acetyltransferase (EC 2.3.1.9)	0.144486	0.20584	0.206939	0.181773	0.201563	0.191894	0.18702	0.185888	0.211284	0.193845	0.209581	0.184178986
Carbohydrates	Central carbohydrate metabolism	Pyruvate metabolism II: acetyl-CoA, acetogenesis from pyruvate	Acetyl-CoA synthetase (ADP-forming) alpha and beta chains, putative	0.11907	0.158339	0.114966	0.114542	0.078787	0.086693	0.068598	0.044354	0.04773	0.041853	0.000582	0.0868047
Carbohydrates	Central carbohydrate metabolism	Pyruvate metabolism II: acetyl-CoA, acetogenesis from pyruvate	Acetyl-CoA synthetase (ADP-forming) alpha chain (EC 6.2.1.13)	0.013926	0.031668	0.025548	0.02739	0.01904	0.022891	0.006499	0.006479	0.005036	0.004406	0.000233	0.016209328
Fatty Acids, Lipids, and Isoprenoids	Fatty acids	Fatty Acid Biosynthesis FASII	Acetyl-coenzyme A carboxyl transferase alpha chain (EC 6.4.1.2)	0.064061	0.095003	0.094528	0.067231	0.089292	0.088641	0.10037	0.087213	0.07685	0.123356	0.094777	0.09694046
Fatty Acids, Lipids, and Isoprenoids	Fatty acids	Fatty Acid Biosynthesis FASII	Acetyl-coenzyme A carboxyl transferase beta chain (EC 6.4.1.2)	0.068239	0.058812	0.079199	0.062251	0.05909	0.059419	0.073653	0.076249	0.069625	0.083706	0.0418	0.063772467
Amino Acids and Derivatives	Branched-chain amino acids	Ketoisovalerate oxidoreductase	Acetyl-coenzyme A synthetase (EC 6.2.1.1)	0.246496	0.264652	0.258035	0.181773	0.256713	0.276152	0.29461	0.257652	0.272589	0.32381	0.16359	0.22996738
Amino Acids and Derivatives	Arginine; urea cycle, polyamines	Arginine Biosynthesis extended	Acetylglutamate kinase (EC 2.7.2.8)	0.077987	0.047502	0.079199	0.054781	0.062373	0.056984	0.044047	0.064787	0.039191	0.074895	0.049601	0.059789153
Miscellaneous	Plant-Prokaryote DOE project	PROSC	Acetylornithine aminotransferase (EC 2.6.1.11)	0.069632	0.090479	0.076644	0.131972	0.089292	0.096434	0.08304	0.105652	0.107065	0.101328	0.134597	0.118197949
Amino Acids and Derivatives	Arginine; urea cycle, polyamines	Arginine Biosynthesis extended	Acetylornithine deacetylase (EC 3.5.1.16)	0.076247	0.04524	0.025548	0.049801	0.049898	0.06088	0.062821	0.055318	0.063495	0.099125	0.068696	0.04878574
Carbohydrates	Organic acids	Propionate-CoA to Succinate Module	Aconitate hydratase (EC 4.2.1.3)	0.16816	0.21715	0.224822	0.176793	0.193027	0.186049	0.200739	0.199344	0.221574	0.17402	0.13844	0.161107314
Carbohydrates	Central carbohydrate metabolism	Glyoxylate bypass	Aconitate hydratase 2 (EC 4.2.1.3)	0.025416	0.015834	0.017884	0.02988	0.030858	0.028248	0.033938	0.040866	0.053642	0.061678	0.302844	0.05099431
Virulence, Disease and Defense	Resistance to antibiotics and toxic compounds	Multidrug Resistance Efflux Pumps	Acriflavin resistance protein	0.37984	0.368703	0.413878	0.420817	0.411004	0.507496	0.420975	0.431082	0.600571	0.257726	0.3918	0.541494106

Fatty Acids, Lipids, and Isoprenoids	Fatty acids	Fatty Acid Biosynthesis FASII	Acyl carrier protein	0.072765	0.047502	0.05876	0.034861	0.04005	0.048704	0.043325	0.042859	0.044008	0.033042	0.017232	0.052532422
Clustering-based subsystems	Fatty acid metabolic cluster	CBSS-246196.1.peg.364	Acyl dehydratase	0.005571	0.018096	0.012774	0.00249	0.003939	0.007793	0.008665	0.011961	0.011604	0.006608	0.001863	0.005127038
Cell Wall and Capsule	Gram-Negative cell wall components	KDO2-Lipid A biosynthesis	Acyl-[acyl-carrier-protein]--UDP-N-acetylglucosamine O-acyltransferase (EC 2.3.1.129)	0.040734	0.054288	0.104747	0.049801	0.049242	0.046269	0.044047	0.042361	0.050577	0.059475	0.039937	0.053597268
Amino Acids and Derivatives	Branched-chain amino acids	Isoleucine degradation	Acyl-CoA dehydrogenase, short-chain specific (EC 1.3.99.2)	0.093654	0.104051	0.104747	0.112052	0.090605	0.10861	0.109757	0.123593	0.182164	0.178426	0.128077	0.130345085
Cell Wall and Capsule	Cell wall of Mycobacteria	mycolic acid synthesis	acyl-CoA synthetase	0.010097	0.013572	0.017884	0.01743	0.013131	0.013637	0.007943	0.012957	0.024522	0.008811	0.003144	0.01329086
Miscellaneous	Plant-Prokaryote DOE project	At5g04520 AT1G06240	Acyl-CoA thioesterase II (EC 3.1.2.-)	0.016363	0.006786	0.00511	0.00996	0.011161	0.009741	0.023107	0.016446	0.022114	0.008811	0.012808	0.008321577
Fatty Acids, Lipids, and Isoprenoids	Phospholipids	Glycerolipid and Glycerophospholipid Metabolism in Bacteria	Acyl-phosphate:glycerol-3-phosphate O-acyltransferase PlsY	0.024023	0.03393	0.020438	0.037351	0.022979	0.023865	0.023107	0.034885	0.030653	0.024231	0.003726	0.021809631
Clustering-based subsystems	NULL	CBSS-393124.3.peg.2657	ADA regulatory protein	0.011489	0.004524	0.017884	0.01494	0.008535	0.015585	0.011553	0.00897	0.012699	0.004406	0.001397	0.007651118
Nucleosides and Nucleotides	Purines	Purine conversions	Adenine deaminase (EC 3.5.4.2)	0.023327	0.042978	0.017884	0.01743	0.027575	0.036041	0.026717	0.025416	0.01445	0.052867	0.006986	0.023899886
Regulation and Cell signaling	NULL	cAMP signaling in bacteria	Adenine phosphoribosyltransferase (EC 2.4.2.7)	0.01706	0.052026	0.022993	0.037351	0.035454	0.025326	0.035382	0.026911	0.031966	0.030839	0.022472	0.027764883
Nucleosides and Nucleotides	Detoxification	Nudix proteins (nucleoside triphosphate hydrolases)	Adenosine (5')-pentaphospho-(5'')-adenosine pyrophosphohydrolase (EC 3.6.1.-)	0.004874	0.002262	0.010219	0.01743	0.007879	0.007793	0.015164	0.025416	0.020143	0.011014	0.02317	0.016524838
Nucleosides and Nucleotides	Purines	Purine conversions	Adenosine deaminase (EC 3.5.4.4)	0.02263	0.047502	0.038322	0.037351	0.019697	0.020456	0.025273	0.031895	0.041162	0.048461	0.051114	0.03324687
Amino Acids and Derivatives	Lysine, threonine, methionine, and cysteine	Methionine Biosynthesis	Adenosylhomocysteinase (EC 3.3.1.1)	0.323439	0.192269	0.25548	0.181773	0.183179	0.198225	0.220958	0.177915	0.165086	0.202657	0.043197	0.172031849
Cofactors, Vitamins, Prosthetic Groups, Pigments	Biotin	Biotin biosynthesis	Adenosylmethionine-8-amino-7-oxononanoate aminotransferase (EC 2.6.1.62)	0.037253	0.05655	0.05876	0.02988	0.066312	0.05601	0.062099	0.05482	0.066998	0.061678	0.290619	0.064245732
Clustering-based subsystems	Nucleotidyl-phosphate metabolic cluster	CBSS-222523.1.peg.1311	Adenylate cyclase	0.079032	0.144767	0.12774	0.136952	0.107019	0.109584	0.080151	0.056315	0.090644	0.017622	0.001979	0.071541902
Regulation and Cell signaling	NULL	cAMP signaling in bacteria	Adenylate cyclase (EC 4.6.1.1)	0.23431	0.26239	0.332124	0.288845	0.244895	0.229396	0.151638	0.170439	0.322509	0.041853	0.019444	0.175423582
Nucleosides and Nucleotides	Purines	Purine conversions	Adenylate kinase (EC 2.7.4.3)	0.097484	0.047502	0.056206	0.032371	0.050555	0.045782	0.049102	0.055318	0.052985	0.074895	0.043663	0.079035265
Nucleosides and Nucleotides	Purines	Purine conversions	Adenylosuccinate lyase (EC 4.3.2.2)	0.093306	0.126671	0.097082	0.109562	0.123432	0.125656	0.101814	0.08821	0.094147	0.200454	0.138556	0.118079632
Clustering-based subsystems	Clustering-based subsystems	CBSS-262719.3.peg.410	Adenylosuccinate synthetase (EC 6.3.4.4)	0.184872	0.088217	0.12263	0.156873	0.110301	0.101304	0.141529	0.114125	0.08933	0.138776	0.113639	0.11721198
Amino Acids and Derivatives	Lysine, threonine, methionine, and cysteine	Cysteine Biosynthesis	Adenylylsulfate kinase (EC 2.7.1.25)	0.222821	0.101789	0.081754	0.084661	0.111614	0.109097	0.089538	0.095187	0.091301	0.026433	0.114571	0.075682971
Cell Wall and Capsule	Gram-Negative cell wall components	LOS core oligosaccharide biosynthesis	ADP-heptose synthase (EC 2.7.-.-)	0.027853	0.013572	0.030658	0.037351	0.033484	0.043347	0.045491	0.024918	0.01664	0.013217	0.017116	0.032813044
Miscellaneous	Plant-Prokaryote DOE project	COG0451	ADP-heptose--lipooligosaccharide heptosyltransferase II (EC 2.4.1.-)	0.019149	0.027144	0.022993	0.02988	0.022979	0.028735	0.018052	0.02741	0.018392	0.002203	0.001863	0.027212741
Cell Wall and Capsule	Gram-Negative cell wall components	LOS core oligosaccharide biosynthesis	ADP-L-glycero-D-manno-heptose-6-epimerase (EC 5.1.3.20)	0.012534	0.013572	0.033212	0.01992	0.026919	0.020943	0.041881	0.038872	0.016421	0.017622	0.004308	0.029657944
Nucleosides and Nucleotides	Detoxification	Nudix proteins (nucleoside triphosphate hydrolases)	ADP-ribose pyrophosphatase (EC 3.6.1.13)	0.016363	0.018096	0.022993	0.01245	0.067625	0.0263	0.042603	0.034885	0.02277	0.024231	0.028177	0.022558968
Cofactors, Vitamins, Prosthetic Groups, Pigments	Tetrapyrroles	Coenzyme B12 biosynthesis	Aerobic cobaltochelatase CobS subunit (EC 6.6.1.2)	0.008356	0.004524	0.010219	0.00249	0.011818	0.018508	0.027439	0.020931	0.023208	0.008811	0.000466	0.003431172
Cofactors, Vitamins, Prosthetic Groups, Pigments	Tetrapyrroles	Coenzyme B12 biosynthesis	Aerobic cobaltochelatase CobT subunit (EC 6.6.1.2)	0.01323	0.013572	0.010219	0.00498	0.01904	0.015585	0.01372	0.018938	0.01861	0.006608	0.001747	0.003036784
Fatty Acids, Lipids, and Isoprenoids	Phospholipids	Glycerolipid and Glycerophospholipid Metabolism in Bacteria	Aerobic glycerol-3-phosphate dehydrogenase (EC 1.1.5.3)	0.029593	0.047502	0.033212	0.042331	0.026919	0.038963	0.040437	0.051331	0.052985	0.024231	0.058333	0.023663253
Amino Acids and Derivatives	Arginine; urea cycle, polyamines	Arginine and Ornithine Degradation	Agmatinase (EC 3.5.3.11)	0.025067	0.042978	0.010219	0.037351	0.035454	0.043834	0.042603	0.03937	0.060648	0.019825	0.037492	0.050166096
Carbohydrates	Central carbohydrate metabolism	Pyruvate Alanine Serine Interconversions	Alanine dehydrogenase (EC 1.4.1.1)	0.126729	0.063336	0.074089	0.047311	0.069595	0.086693	0.102536	0.084223	0.082762	0.037447	0.08686	0.079390214
Amino Acids and Derivatives	Alanine, serine, and glycine	Alanine biosynthesis	Alanine racemase (EC 5.1.1.1)	0.018452	0.058812	0.038322	0.059761	0.044646	0.036528	0.03466	0.025915	0.015545	0.013217	0.030622	0.042514978
Amino Acids and Derivatives	Alanine, serine, and glycine	Alanine biosynthesis	Alanine racemase, biosynthetic (EC 5.1.1.1)	0.060579	0.004524	0.007664	0.00498	0.005909	0.003409	0.010109	0.003489	0.008101	0.002203	0.001514	0.006822905
Clustering-based subsystems	Hypothetical protein possible functionally linked with Alanyl-tRNA synthetase	CBSS-257314.1.peg.488	Alanyl-tRNA synthetase (EC 6.1.1.7)	0.197406	0.246556	0.235042	0.191733	0.204189	0.194816	0.206516	0.230741	0.189827	0.185034	0.206903	0.220028814
Carbohydrates	Fermentation	Fermentations: Mixed acid	Alcohol dehydrogenase (EC 1.1.1.1)	0.110714	0.122147	0.13285	0.117032	0.149038	0.129066	0.15236	0.148013	0.153044	0.081503	0.080689	0.1013576
Carbohydrates	Central carbohydrate metabolism	Methylglyoxal Metabolism	Aldehyde dehydrogenase (EC 1.2.1.3)	0.089477	0.054288	0.091973	0.092131	0.081413	0.066724	0.109035	0.1216	0.187637	0.180629	0.228094	0.106879026
Metabolism of Aromatic Compounds	NULL	Aromatic Amin Catabolism	Aldehyde dehydrogenase (EC 1.2.1.3), PaaZ	0.000348	0.002262	0.002555	0.00249	0.003283	0.001461	0.002888	0.000498	0.002189	0.002203	0.000233	0.00189306
Fatty Acids, Lipids, and Isoprenoids	Phospholipids	Glycerolipid and Glycerophospholipid Metabolism in Bacteria	Aldehyde dehydrogenase B (EC 1.2.1.22)	0.040038	0.029406	0.05876	0.037351	0.033484	0.036528	0.049102	0.045351	0.059991	0.01542	0.132269	0.061051193
Carbohydrates	Di- and oligosaccharides	Maltose and Maltodextrin Utilization	Aldose 1-epimerase (EC 5.1.3.3)	0.008008	0.015834	0.00511	0.00747	0.007879	0.006819	0.006499	0.006977	0.007444	0.011014	0.003959	0.010254076
Carbohydrates	Monosaccharides	L-rhamnose utilization	Alfa-L-rhamnosidase (EC 3.2.1.40)	0.036208	0.113099	0.074089	0.067231	0.032171	0.025326	0.01372	0.026911	0.033718	0.004406	0.002445	0.041529009
Phosphorus Metabolism	NULL	Phosphate metabolism	Alkaline phosphatase (EC 3.1.3.1)	0.027156	0.038454	0.056206	0.039841	0.055807	0.077926	0.075819	0.096682	0.094804	0.024231	0.169528	0.068071291
Phosphorus Metabolism	NULL	High affinity phosphate transporter and control of PHO regulon	Alkaline phosphatase synthesis transcriptional regulatory protein PhoP	0.0094	0.006786	0.00511	0.01494	0.011161	0.003896	0.005055	0.001993	0.007225	0.011014	0.001281	0.008006067
Sulfur Metabolism	NULL	Thioredoxin-disulfide reductase	Alkyl hydroperoxide reductase protein C (EC 1.6.4.-)	0.013926	0.027144	0.012774	0.02241	0.010505	0.00828	0.015886	0.018439	0.012042	0.002203	0.030971	0.015617747
Sulfur Metabolism	NULL	Thioredoxin-disulfide reductase	Alkyl hydroperoxide reductase protein F (EC 1.6.4.-)	0.005571	0.004524	0.017884	0.00498	0.004596	0.012176	0.023829	0.018938	0.014888	0.002203	0.172322	0.014355707
Sulfur Metabolism	NULL	Thioredoxin-disulfide reductase	Alkyl hydroperoxide reductase subunit C-like protein	0.041083	0.05655	0.045986	0.02739	0.036111	0.057471	0.043325	0.054321	0.060648	0.147587	0.05682	0.066138792
Phosphorus Metabolism	NULL	Alkylphosphonate utilization	Alkylphosphonate utilization operon protein PhnA	0.003133	0.004524	0.002555	0.00498	0.006566	0.003409	0.011553	0.00598	0.007882	0.041853	0.033882	0.022558968
Amino Acids and Derivatives	Arginine; urea cycle, polyamines	Urea carboxylase and Allophanate hydrolase cluster	Allophanate hydrolase (EC 3.5.1.54)	0.005919	0.002262	0.010219	0.01494	0.003939	0.010715	0.015164	0.011961	0.009196	0.004406	0.000582	0.010490709
Clustering-based subsystems	NULL	EC699-706	Allophanate hydrolase 2 subunit 1 (EC 3.5.1.54)	0.003482	0.004524	0.015329	0.00498	0.003939	0.01315	0.006499	0.011462	0.016859	0.011014	0.027711	0.010648464
Carbohydrates	Di- and oligosaccharides	Trehalose Biosynthesis	Alpha,alpha-trehalose-phosphate synthase [UDP-forming] (EC 2.4.1.15)	0.045957	0.047502	0.051096	0.039841	0.049898	0.041398	0.046213	0.053325	0.057583	0.030839	0.003144	0.023308304
Carbohydrates	Monosaccharides	Mannose Metabolism	Alpha-1,2-mannosidase	0.043868	0.05655	0.051096	0.044821	0.016414	0.019482	0.021663	0.017941	0.02912	0.185034	0.018629	0.067203638
Protein Metabolism	Protein processing and modification	N-linked Glycosylation in Bacteria	Alpha-1,3-N-acetylgalactosamine transferase PglA (EC 2.4.1.-)	0.004526	0.009048	0.007664	0.00498	0.002626	0.003896	0.007221	0.00299	0.006568	0.002203	0.000233	0.006704588
Carbohydrates	Di- and oligosaccharides	Maltose and Maltodextrin Utilization	Alpha-amylase (EC 3.2.1.1)	0.020193	0.054288	0.06898	0.057271	0.028232	0.023378	0.03466	0.026911	0.044446	0.052867	0.018629	0.04148957
Miscellaneous	Plant-Prokaryote DOE project	COG3533	Alpha-galactosidase (EC 3.2.1.22)	0.021934	0.054288	0.045986	0.044821	0.030858	0.028248	0.027439	0.027908	0.020143	0.017622	0.001747	0.022637845
Carbohydrates	Monosaccharides	D-Galacturonate and D-Glucuronate Utilization	Alpha-glucosidase (EC 3.2.1.20)	0.013926	0.052026	0.043432	0.037351	0.015101	0.021917	0.02094	0.016446	0.02693	0.028636	0.006753	0.050205535
Membrane Transport	ABC transporters	Periplasmic-Binding-Protein-Dependent Transport System for -Glucosides	Alpha-glucoside transport ATP-binding protein AglK	0.000696	0.006786	0.00511	0.00996	0.005252	0.001948	0.002166	0.005482	0.007444	0.004406	0.011294	0.003312855
Membrane Transport	ABC transporters	Periplasmic-Binding-Protein-Dependent Transport System for -Glucosides	Alpha-glucoside transport system permease protein AglG	0.004526	0.015834	0.015329	0.01245	0.010505	0.015585	0.010109	0.012957	0.010947	0.006608	0.012691	0.00634964
Membrane Transport	ABC transporters	Periplasmic-Binding-Protein-Dependent Transport System for -Glucosides	Alpha-glucosides-binding periplasmic protein AglE precursor	0.001393	0.013572	0.002555	0.00747	0.01707	0.00828	0.006499	0.00897	0.015326	0.006608	0.000349	0.004219947
Virulence, Disease and Defense	Resistance to antibiotics and toxic compounds	Bile hydrolysis	Alpha-ketoglutarate-dependent taurine dioxygenase (EC 1.14.11.17)	0.003482	0.013572	0.012774	0.00747	0.004596	0.006332	0.011553	0.008472	0.036564	0.006608	0.000349	0.004377702

Miscellaneous	Plant-Prokaryote DOE project	COG3533	Alpha-L-arabinofuranosidase II precursor (EC 3.2.1.55)	0.008008	0.01131	0.002555	0.00498	0.000657	0.002435	0.006499	0.004984	0.006349	0.008811	0.016534	0.007887751
Carbohydrates	Monosaccharides	L-fucose utilization temp	Alpha-L-fucosidase (EC 3.2.1.51)	0.036208	0.067859	0.071534	0.069721	0.030858	0.054061	0.040437	0.032892	0.024303	0.022028	0.002794	0.052138034
Carbohydrates	Di- and oligosaccharides	Fructooligosaccharides(FOS) and Raffinose Utilization	Alpha-mannosidase (EC 3.2.1.24)	0.012882	0.015834	0.012774	0.01245	0.008535	0.012663	0.016608	0.00598	0.003503	0.002203	0.001281	0.00887372
Carbohydrates	NULL	Sugar utilization in Thermotogales	Alpha-N-arabinofuranosidase (EC 3.2.1.55)	0.015319	0.03393	0.028103	0.042331	0.017727	0.006819	0.01372	0.007974	0.020143	0.004406	0.003027	0.022637845
Miscellaneous	Plant-Prokaryote DOE project	COG3533	Alpha-N-arabinofuranosidase 2 (EC 3.2.1.55)	0.015319	0.013572	0.017884	0.01743	0.008535	0.00828	0.008665	0.010964	0.019486	0.024231	0.000116	0.010924535
Carbohydrates	Monosaccharides	Xylose utilization	Alpha-xylosidase (EC 3.2.1.-)	0.017408	0.02262	0.028103	0.047311	0.009848	0.009741	0.014442	0.007974	0.017735	0.011014	0.00163	0.016248767
Respiration	Electron accepting reactions	Terminal cytochrome C oxidases	Alternative cytochrome c oxidase polypeptide CoxN (EC 1.9.3.1)	0.003482	0.002262	0.022993	0.00498	0.005252	0.004383	0.015886	0.006977	0.012261	0.004406	0.00815	0.007256731
Carbohydrates	Monosaccharides	D-Galacturonate and D-Glucuronate Utilization	Altronate hydrolase (EC 4.2.1.7)	0.035164	0.038454	0.035767	0.047311	0.032171	0.0526	0.044047	0.018938	0.048606	0.024231	0.005123	0.022125142
Cofactors, Vitamins, Prosthetic Groups, Pigments	Folate and pterines	YgfZ	Amidophosphoribosyltransferase (EC 2.4.2.14)	0.135782	0.133457	0.150733	0.164343	0.141159	0.139293	0.1177	0.120603	0.109254	0.196048	0.069627	0.138272275
Cell Wall and Capsule	NULL	Recycling of Peptidoglycan Amino Acids	Aminoacyl-histidine dipeptidase (Peptidase D) (EC 3.4.13.3)	0.021586	0.027144	0.028103	0.01245	0.012475	0.021917	0.015886	0.014951	0.011604	0.03965	0.019095	0.040779673
Carbohydrates	CO2 fixation	Photorespiration (oxidative C2 cycle)	Aminomethyltransferase (glycine cleavage system T protein) (EC 2.1.2.10)	0.093654	0.074645	0.030658	0.077191	0.055151	0.072082	0.078707	0.084721	0.067436	0.127762	0.18152	0.073750472
Protein Metabolism	Protein degradation	Protein degradation	Aminopeptidase YpdF (MP-, MA-, MS-, AP-, NP-specific)	0.013926	0.015834	0.022993	0.02241	0.026919	0.016559	0.020218	0.015948	0.019924	0.006608	0.000699	0.01616989
Nitrogen Metabolism	NULL	Ammonia assimilation	Ammonium transporter	0.150404	0.196793	0.194165	0.141932	0.190401	0.175821	0.158136	0.145521	0.176471	0.072692	0.201896	0.19163291
Nucleosides and Nucleotides	Purines	Purine conversions	AMP nucleosidase (EC 3.2.2.4)	0.016363	0.015834	0.022993	0.0249	0.013131	0.014124	0.014442	0.02741	0.020362	0.006608	0.003609	0.012541524
Cell Wall and Capsule	NULL	Recycling of Peptidoglycan Amino Acids	AmpG permease	0.021934	0.03393	0.028103	0.062251	0.03808	0.048704	0.031772	0.023423	0.03109	0.006608	0.063107	0.026976108
Respiration	Electron accepting reactions	Anaerobic respiratory reductases	Anaerobic dehydrogenases, typically selenocysteine-containing	0.086343	0.169649	0.137959	0.107072	0.116867	0.12176	0.064988	0.036879	0.070282	0.013217	0.001048	0.133973451
Respiration	Electron accepting reactions	Anaerobic respiratory reductases	Anaerobic dimethyl sulfoxide reductase chain A (EC 1.8.99.-)	0.062668	0.076907	0.086863	0.097112	0.07616	0.076465	0.05199	0.043856	0.031528	0.002203	0.000699	0.053084564
Carbohydrates	Sugar alcohols	Glycerol and Glycerol-3-phosphate Uptake and Utilization	Anaerobic glycerol-3-phosphate dehydrogenase subunit A (EC 1.1.5.3)	0.012534	0.013572	0.007664	0.00747	0.01707	0.017533	0.005055	0.00299	0.003065	0.006608	0.000116	0.007848312
Amino Acids and Derivatives	Aromatic amino acids and derivatives	Chorismate: Intermediate for synthesis of Tryptophan, PAPA antibiotics, PABA, 3-hydroxyanthranilate and more.	Anthranilate phosphoribosyltransferase (EC 2.4.2.18)	0.049438	0.092741	0.06898	0.094622	0.091261	0.071595	0.077985	0.048341	0.061524	0.116748	0.062991	0.07315889
Amino Acids and Derivatives	Aromatic amino acids and derivatives	Chorismate: Intermediate for synthesis of Tryptophan, PAPA antibiotics, PABA, 3-hydroxyanthranilate and more.	Anthranilate synthase, amidotransferase component (EC 4.1.3.27)	0.051876	0.024882	0.020438	0.0249	0.030202	0.031171	0.049102	0.039869	0.032185	0.041853	0.027013	0.030762229
Amino Acids and Derivatives	Aromatic amino acids and derivatives	Chorismate: Intermediate for synthesis of Tryptophan, PAPA antibiotics, PABA, 3-hydroxyanthranilate and more.	Anthranilate synthase, aminase component (EC 4.1.3.27)	0.081817	0.113099	0.143069	0.087151	0.091918	0.097408	0.105424	0.091698	0.082981	0.063881	0.066949	0.099188469
Stress Response	NULL	SigmaB stress responce regulation	anti sigma b factor antagonist RsbV	0.001393	0.004524	0.00511	0.00498	0.003283	0.002435	0.002166	0.009469	0.007444	0.002203	0.000582	0.003036784
Clustering-based subsystems	NULL	EC49-61	ApaG protein	0.029245	0.002262	0.010219	0.034861	0.008535	0.015585	0.012275	0.013456	0.007663	0.01542	0.015835	0.015262798
Clustering-based subsystems	Biosynthesis of galactoglycans and related lipopolysacharides	CBSS-376686.6.peg.291	Apolipoprotein N-acyltransferase (EC 2.3.1.-)	0.008704	0.01131	0.022993	0.00747	0.017727	0.019482	0.01733	0.015449	0.017297	0.002203	0.041567	0.015657186
Stress Response	Osmotic stress	Osmoregulation	Aquaporin Z	0.012186	0.03393	0.053651	0.01743	0.004596	0.008767	0.012275	0.010964	0.014669	0.017622	0.003027	0.013488054
Clustering-based subsystems	NULL	Lipopolysaccharide assembly cluster	Arabinose 5-phosphate isomerase (EC 5.3.1.13)	0.046305	0.03393	0.020438	0.049801	0.024293	0.026787	0.03827	0.047843	0.034375	0.050664	0.068696	0.048588546
DNA Metabolism	DNA replication	DNA replication, archaeal	Archaeal DNA polymerase II large subunit (EC 2.7.7.7)	0.003133	0.004524	0.002555	0.01494	0.013131	0.006819	0.015164	0.010466	0.008758	0.01542	0.000349	0.01013576
Stress Response	Acid stress	Acid resistance mechanisms	Arginine decarboxylase (EC 4.1.1.19)	0.012534	0.02262	0.017884	0.042331	0.011818	0.013637	0.010109	0.016446	0.022552	0.019825	0.056703	0.035889267
Amino Acids and Derivatives	Arginine; urea cycle, polyamines	Arginine and Ornithine Degradation	Arginine utilization regulatory protein RocR	0.005919	0.002262	0.002555	0.00747	0.006566	0.006332	0.002888	0.003987	0.004817	0.004406	0.000233	0.007887751
Amino Acids and Derivatives	Arginine; urea cycle, polyamines	Arginine Biosynthesis extended	Argininosuccinate lyase (EC 4.3.2.1)	0.079728	0.092741	0.112411	0.084661	0.089292	0.092051	0.080873	0.082229	0.095242	0.132167	0.120742	0.099503979
Amino Acids and Derivatives	Arginine; urea cycle, polyamines	Arginine Biosynthesis extended	Argininosuccinate synthase (EC 6.3.4.5)	0.163634	0.090479	0.143069	0.122012	0.126059	0.096921	0.123476	0.091698	0.096774	0.151992	0.126796	0.100056121
Protein Metabolism	Protein biosynthesis	tRNA aminoacylation, Arg	Arginyl-tRNA synthetase (EC 6.1.1.19)	0.103055	0.119885	0.140514	0.131972	0.107019	0.130527	0.143695	0.132066	0.11845	0.096923	0.07999	0.128767535
Secondary Metabolism	Plant Hormones	Auxin biosynthesis	Aromatic-L-amino-acid decarboxylase (EC 4.1.1.28)	0.011489	0.013572	0.012774	0.01245	0.007879	0.013637	0.002888	0.017941	0.015326	0.002203	0.000466	0.008952597
Virulence, Disease and Defense	Resistance to antibiotics and toxic compounds	Arsenic resistance	Arsenate reductase (EC 1.20.4.1)	0.036557	0.061074	0.045986	0.037351	0.04202	0.048704	0.046213	0.054321	0.042695	0.066084	0.003377	0.041883958
Virulence, Disease and Defense	Resistance to antibiotics and toxic compounds	Arsenic resistance	Arsenical pump-driving ATPase (EC 3.6.3.16)	0.012186	0.024882	0.033212	0.01245	0.014444	0.024839	0.012998	0.016446	0.016202	0.004406	0.001281	0.011871065
Virulence, Disease and Defense	Resistance to antibiotics and toxic compounds	Arsenic resistance	Arsenical-resistance protein ACR3	0.117329	0.079169	0.081754	0.089641	0.086665	0.093999	0.090261	0.093692	0.054299	0.127762	0.019561	0.083531283
Clustering-based subsystems	Sulfatases and sulfatase modifying factor 1 (and a hypothetical)	Sulfatases and sulfatase modifying factor 1	Arylsulfatase (EC 3.1.6.1)	0.346765	0.563234	0.608043	0.420817	0.227825	0.287354	0.213737	0.184393	0.275654	0.050664	0.005589	0.308016676
Amino Acids and Derivatives	Glutamine, glutamate, aspartate, asparagine; ammonia assimilation	Glutamine, Glutamate, Aspartate and Asparagine Biosynthesis	Asparagine synthetase [glutamine-hydrolyzing] (EC 6.3.5.4)	0.186961	0.158339	0.176281	0.194223	0.248835	0.286866	0.246953	0.18838	0.167713	0.107937	0.028643	0.192342808
Protein Metabolism	Protein biosynthesis	tRNA aminoacylation, Asp and Asn	Asparaginyl-tRNA synthetase (EC 6.1.1.22)	0.086343	0.088217	0.079199	0.082171	0.109645	0.079387	0.080151	0.072761	0.078383	0.28416	0.038656	0.085029956
Cofactors, Vitamins, Prosthetic Groups, Pigments	Coenzyme A	Coenzyme A Biosynthesis	Aspartate 1-decarboxylase (EC 4.1.1.11)	0.010793	0.004524	0.012774	0.00249	0.006566	0.005844	0.006499	0.00897	0.005255	0.004406	0.014088	0.01234433
Amino Acids and Derivatives	Glutamine, glutamate, aspartate, asparagine; ammonia assimilation	Glutamine, Glutamate, Aspartate and Asparagine Biosynthesis	Aspartate aminotransferase (EC 2.6.1.1)	0.206458	0.203578	0.265699	0.221614	0.225199	0.213811	0.254896	0.205823	0.225953	0.321607	0.270825	0.305532035
Amino Acids and Derivatives	Glutamine, glutamate, aspartate, asparagine; ammonia assimilation	Glutamine, Glutamate, Aspartate and Asparagine Biosynthesis	Aspartate ammonia-lyase (EC 4.3.1.1)	0.0094	0.009048	0.012774	0.01743	0.008535	0.014124	0.015164	0.009469	0.009634	0.01542	0.076613	0.018496776
Nucleosides and Nucleotides	Pyrimidines	De Novo Pyrimidine Synthesis	Aspartate carbamoyltransferase (EC 2.1.3.2)	0.090869	0.074645	0.130295	0.124502	0.074191	0.089615	0.109035	0.086216	0.103562	0.154195	0.076264	0.09567842
Amino Acids and Derivatives	Glutamine, glutamate, aspartate, asparagine; ammonia assimilation	Glutamine, Glutamate, Aspartate and Asparagine Biosynthesis	Aspartate--ammonia ligase (EC 6.3.1.1)	0.006267	0.018096	0.035767	0.042331	0.014444	0.015098	0.008665	0.004485	0.01861	0.002203	0.009664	0.014197952
Amino Acids and Derivatives	Lysine, threonine, methionine, and cysteine	Threonine and Homoserine Biosynthesis	Aspartate-semialdehyde dehydrogenase (EC 1.2.1.11)	0.159108	0.099527	0.086863	0.089641	0.094544	0.08231	0.087372	0.093193	0.080135	0.09472	0.108633	0.093272655
Miscellaneous	Plant-Prokaryote DOE project	PROSC	Aspartokinase (EC 2.7.2.4)	0.150752	0.153815	0.13285	0.094622	0.144442	0.124195	0.133586	0.141534	0.121734	0.105734	0.181637	0.157005684
Protein Metabolism	Protein biosynthesis	tRNA aminoacylation, Asp and Asn	Aspartyl-tRNA synthetase (EC 6.1.1.12)	0.109322	0.140243	0.12263	0.146912	0.126715	0.110558	0.116978	0.126085	0.109473	0.286363	0.105489	0.137444061
Miscellaneous	Plant-Prokaryote DOE project	At3g21300	Aspartyl-tRNA(Asn) amidotransferase subunit A (EC 6.3.5.6)	0.055009	0.081431	0.025548	0.052291	0.063029	0.064289	0.052712	0.055318	0.052985	0.033042	0.059381	0.050797116
Protein Metabolism	Protein biosynthesis	tRNA aminoacylation, Asp and Asn	Aspartyl-tRNA(Asn) amidotransferase subunit B (EC 6.3.5.6)	0.052224	0.052026	0.040877	0.054781	0.084696	0.058932	0.068598	0.065784	0.052766	0.048461	0.035047	0.061169509
Miscellaneous	Plant-Prokaryote DOE project	At3g21300	Aspartyl-tRNA(Asn) amidotransferase subunit C (EC 6.3.5.6)	0.002437	0.006786	0.002555	0.00249	0.006566	0.001948	0.004333	0.007974	0.003503	0.004406	0.000349	0.002563519
Protein Metabolism	Protein biosynthesis	tRNA aminoacylation, Asp and Asn	Aspartyl-tRNA(Asn) synthetase (EC 6.1.1.23)	0.038994	0.03393	0.066425	0.049801	0.041363	0.045782	0.068598	0.06678	0.038097	0.041853	0.02678	0.040937428
Protein Metabolism	Protein degradation	Protein degradation	Asp-X dipeptidase	0.006615	0.004524	0.007664	0.00747	0.005252	0.005357	0.006499	0.009469	0.009853	0.006608	0.006986	0.008479332
Nitrogen Metabolism	NULL	Nitrate and nitrite ammonification	Assimilatory nitrate reductase large subunit (EC:1.7.99.4)	0.011489	0.013572	0.035767	0.00996	0.024949	0.018995	0.036104	0.033889	0.035469	0.028636	0.06171	0.051073188
Amino Acids and Derivatives	Histidine Metabolism	Histidine Biosynthesis	ATP phosphoribosyltransferase (EC 2.4.2.17)	0.035512	0.05655	0.071534	0.057271	0.05712	0.071595	0.041881	0.032393	0.039848	0.070489	0.015136	0.051901402
Amino Acids and Derivatives	Histidine Metabolism	Histidine Biosynthesis	ATP phosphoribosyltransferase catalytic subunit (EC 2.4.2.17)	0.079032	0.013572	0.007664	0.02739	0.013131	0.025326	0.037548	0.030898	0.020143	0.011014	0.025615	0.022006825

Amino Acids and Derivatives	Histidine Metabolism	Histidine Biosynthesis	ATP phosphoribosyltransferase regulatory subunit (EC 2.4.2.17)	0.047349	0.013572	0.017884	0.01743	0.007879	0.019969	0.016608	0.028407	0.020362	0.011014	0.021657	0.019679939
Respiration	ATP synthases	F0F1-type ATP synthase	ATP synthase A chain (EC 3.6.3.14)	0.166071	0.106313	0.094528	0.057271	0.052524	0.077926	0.049824	0.070269	0.063714	0.03965	0.061826	0.073553278
Respiration	ATP synthases	F0F1-type ATP synthase	ATP synthase alpha chain (EC 3.6.3.14)	0.214117	0.223936	0.217158	0.156873	0.185149	0.17436	0.203628	0.180905	0.175157	0.187237	0.173486	0.175463021
Respiration	ATP synthases	F0F1-type ATP synthase	ATP synthase B chain (EC 3.6.3.14)	0.007311	0.015834	0.002555	0.01245	0.016414	0.01802	0.012998	0.028905	0.018173	0.011014	0.019794	0.026857792
Respiration	ATP synthases	F0F1-type ATP synthase	ATP synthase beta chain (EC 3.6.3.14)	0.430671	0.180959	0.19161	0.201693	0.172018	0.150008	0.176911	0.189377	0.180193	0.374474	0.178493	0.175147511
Miscellaneous	Plant-Prokaryote DOE project	Iojap	ATP synthase C chain (EC 3.6.3.14)	0.042475	0.027144	0.035767	0.047311	0.043989	0.033606	0.037548	0.03638	0.03525	0.004406	0.002445	0.028671975
Respiration	ATP synthases	F0F1-type ATP synthase	ATP synthase epsilon chain (EC 3.6.3.14)	0.03969	0.018096	0.015329	0.01743	0.021666	0.02143	0.015164	0.019436	0.025836	0.024231	0.039005	0.024097079
Respiration	ATP synthases	F0F1-type ATP synthase	ATP synthase gamma chain (EC 3.6.3.14)	0.089128	0.063336	0.040877	0.069721	0.079443	0.088641	0.093871	0.095685	0.072033	0.035245	0.070675	0.073474401
Membrane Transport	NULL	ECF class transporters	ATPase component NikO of energizing module of nickel ECF transporter	0.019497	0.02262	0.038322	0.02988	0.01904	0.023865	0.004333	0.006479	0.006568	0.002203	0.000349	0.016564277
Cell Wall and Capsule	NULL	YjeE	ATPase YjeE, predicted to have essential role in cell wall biosynthesis	0.010097	0.018096	0.017884	0.039841	0.018384	0.023865	0.01733	0.021429	0.015326	0.022028	0.04343	0.016564277
Clustering-based subsystems	NULL	ClpS and metallo-protease cluster in Cyanos	ATP-dependent Clp protease adaptor protein ClpS	0.004874	0.024882	0.007664	0.01245	0.019697	0.020456	0.028161	0.021429	0.026712	0.008811	0.009082	0.017629123
Clustering-based subsystems	NULL	ClpAS cluster	ATP-dependent Clp protease ATP-binding subunit ClpA	0.151449	0.133457	0.178836	0.146912	0.156917	0.152931	0.179077	0.173928	0.171654	0.158601	0.186643	0.137641255
Protein Metabolism	Protein degradation	Proteasome bacterial	ATP-dependent Clp protease ATP-binding subunit ClpX	0.18905	0.190007	0.166062	0.196713	0.160856	0.160236	0.111923	0.135056	0.113852	0.26874	0.144844	0.156295786
Protein Metabolism	Protein degradation	Proteasome bacterial	ATP-dependent Clp protease proteolytic subunit (EC 3.4.21.92)	0.090173	0.04524	0.091973	0.084661	0.102423	0.0789	0.123476	0.102662	0.086703	0.160804	0.090586	0.072370115
Protein Metabolism	Protein degradation	Proteolysis in bacteria, ATP-dependent	ATP-dependent Clp protease, ATP-binding subunit ClpC	0.064757	0.104051	0.038322	0.099602	0.073534	0.058445	0.072931	0.065784	0.057145	0.121153	0.023403	0.061169509
Clustering-based subsystems	Ribosomal Protein L28P relates to a set of uncharacterized proteins	A Gram-positive cluster that relates ribosomal protein L28P to a set of uncharacterized proteins	ATP-dependent DNA helicase RecG (EC 3.6.1.-)	0.12847	0.106313	0.130295	0.072211	0.0801	0.072569	0.103258	0.101666	0.109911	0.14979	0.068463	0.103368977
DNA Metabolism	DNA repair	DNA repair, bacterial RecFOR pathway	ATP-dependent DNA helicase RecQ	0.096788	0.099527	0.074089	0.077191	0.061716	0.075491	0.079429	0.101666	0.111882	0.281957	0.094661	0.129950698
DNA Metabolism	DNA repair	DNA repair, bacterial UvrD and related helicases	ATP-dependent DNA helicase Rep	0.042127	0.009048	0.020438	0.02739	0.025606	0.029709	0.03466	0.03339	0.041162	0.035245	0.13157	0.03864998
Clustering-based subsystems	NULL	CBSS-393121.3.peg.1913	ATP-dependent DNA helicase UvrD/PcrA	0.162242	0.190007	0.217158	0.224104	0.230451	0.201635	0.205072	0.224761	0.167932	0.18944	0.088606	0.200033365
DNA Metabolism	DNA replication	DNA replication, archaeal	ATP-dependent DNA ligase (EC 6.5.1.1)	0.012882	0.018096	0.012774	0.02739	0.015101	0.024352	0.028161	0.024918	0.03525	0.011014	0.001281	0.01202882
DNA Metabolism	DNA repair	DNA Repair Base Excision	ATP-dependent DNA ligase (EC 6.5.1.1) clustered with Ku protein, LigD	0.004178	0.006786	0.015329	0.00747	0.003939	0.004383	0.004333	0.006479	0.01664	0.002203	0.000233	0.002445203
DNA Metabolism	DNA repair	DNA Repair Base Excision	ATP-dependent DNA ligase (EC 6.5.1.1) LigC	0.004526	0.006786	0.00511	0.00249	0.011161	0.014611	0.01372	0.014951	0.017735	0.002203	0.00163	0.003036784
Protein Metabolism	Protein degradation	Proteolysis in bacteria, ATP-dependent	ATP-dependent hsl protease ATP-binding subunit HslU	0.102707	0.070121	0.076644	0.109562	0.112928	0.107636	0.124198	0.099174	0.117137	0.068286	0.134481	0.078483122
Protein Metabolism	Protein degradation	Proteasome bacterial	ATP-dependent protease HslV (EC 3.4.25.-)	0.041779	0.027144	0.028103	0.02739	0.041363	0.058932	0.028883	0.040367	0.028244	0.022028	0.023752	0.031511565
Protein Metabolism	Protein degradation	Proteolysis in bacteria, ATP-dependent	ATP-dependent protease La (EC 3.4.21.53)	0.086343	0.128933	0.145624	0.184263	0.173331	0.157314	0.103258	0.112629	0.119545	0.035245	0.039355	0.123916568
Protein Metabolism	Protein degradation	Proteasome bacterial	ATP-dependent protease La (EC 3.4.21.53) Type I	0.247888	0.244294	0.309131	0.303785	0.258026	0.265437	0.272948	0.21878	0.228143	0.524264	0.23671	0.281356078
Protein Metabolism	Protein degradation	Proteasome bacterial	ATP-dependent protease La (EC 3.4.21.53) Type II	0.048046	0.029406	0.012774	0.02241	0.022323	0.018995	0.008665	0.010466	0.008977	0.011014	0.000582	0.014631778
RNA Metabolism	RNA processing and modification	ATP-dependent RNA helicases, bacterial	ATP-dependent RNA helicase DbpA	0.00383	0.006786	0.007664	0.00996	0.00197	0.001461	0.001444	0.002492	0.009196	0.017622	0.077428	0.021730754
RNA Metabolism	RNA processing and modification	ATP-dependent RNA helicases, bacterial	ATP-dependent RNA helicase RhlB	0.066498	0.040716	0.033212	0.042331	0.042676	0.035067	0.035382	0.029403	0.030434	0.022028	0.072422	0.035337124
RNA Metabolism	RNA processing and modification	ATP-dependent RNA helicases, bacterial	ATP-dependent RNA helicase RhlE	0.058142	0.052026	0.061315	0.052291	0.056464	0.053574	0.073653	0.062793	0.063714	0.176223	0.098736	0.088658321
Miscellaneous	Plant-Prokaryote DOE project	At5g04520 AT1G06240	Bacterioferritin	0.013926	0.03393	0.028103	0.01992	0.021666	0.019482	0.027439	0.034885	0.027587	0.019825	0.013157	0.02184907
Regulation and Cell signaling	Regulation of virulence	BarA-UvrY(SirA) two-component regulatory system	BarA sensory histidine kinase (= VarS = GacS)	0.007311	0.004524	0.010219	0.00498	0.005909	0.008767	0.018052	0.011462	0.023865	0.006608	0.037841	0.038295031
Clustering-based subsystems	NULL	Aerotolerance operon in Bacteroides and potentially orthologous operons in other organisms	BatA (Bacteroides aerotolerance operon)	0.024719	0.027144	0.028103	0.02739	0.036767	0.03458	0.03466	0.028407	0.036126	0.022028	0.00652	0.042160029
Clustering-based subsystems	NULL	Aerotolerance operon in Bacteroides and potentially orthologous operons in other organisms	BatB	0.000696	0.004524	0.010219	0.00249	0.008535	0.009741	0.005777	0.004485	0.007444	0.002203	0.00163	0.021967387
Metabolism of Aromatic Compounds	NULL	Benzoate transport and degradation cluster	Benzoate transport, extracellular ligand-binding receptor	0.002437	0.002262	0.002555	0.00249	0.004596	0.004383	0.002888	0.003987	0.005255	0.004406	0.010013	0.001695866
Metabolism of Aromatic Compounds	Anaerobic degradation of aromatic compounds	Anaerobic benzoate metabolism	Benzoyl-CoA reductase subunit BadD (EC 1.3.99.15)	0.002089	0.002262	0.002555	0.01245	0.005252	0.005357	0.002888	0.003489	0.005255	0.002203	0.000116	0.001774744
Metabolism of Aromatic Compounds	Anaerobic degradation of aromatic compounds	Anaerobic benzoate metabolism	Benzoyl-CoA reductase subunit BadG (EC 1.3.99.15)	0.016363	0.027144	0.028103	0.037351	0.022979	0.017046	0.015164	0.000997	0.003065	0.004406	0.000349	0.016367083
Metabolism of Aromatic Compounds	NULL	Benzoate transport and degradation cluster	benzoyl-CoA-dihydrodiol lyase (BoxC)	0.011837	0.006786	0.022993	0.01245	0.005909	0.010715	0.007943	0.010964	0.022114	0.011014	0.000233	0.005797497
Cell Wall and Capsule	NULL	Recycling of Peptidoglycan Amino Sugars	Beta N-acetyl-glucosaminidase (EC 3.2.1.52)	0.011141	0.020358	0.020438	0.00249	0.012475	0.018508	0.015164	0.022925	0.015764	0.006608	0.027478	0.017471368
Fatty Acids, Lipids, and Isoprenoids	Isoprenoids	Carotenoids	Beta-carotene ketolase (EC 1.14.-.-)	0.019149	0.020358	0.038322	0.044821	0.015757	0.02143	0.036826	0.032892	0.038316	0.002203	0.000466	0.011752749
Carbohydrates	Di- and oligosaccharides	Lactose and Galactose Uptake and Utilization	Beta-galactosidase (EC 3.2.1.23)	0.071721	0.221674	0.171172	0.179283	0.082726	0.084258	0.087372	0.081731	0.08517	0.028636	0.010246	0.171282513
Carbohydrates	Di- and oligosaccharides	Beta-Glucoside Metabolism	Beta-glucosidase (EC 3.2.1.21)	0.054661	0.108575	0.102192	0.134462	0.050555	0.044321	0.046213	0.047344	0.073566	0.052867	0.00163	0.076077358
Carbohydrates	NULL	Sugar utilization in Thermotogales	Beta-hexosaminidase (EC 3.2.1.52)	0.020889	0.05655	0.038322	0.049801	0.036111	0.031658	0.041159	0.029902	0.037659	0.03965	0.007801	0.049929464
Metabolism of Aromatic Compounds	Metabolism of central aromatic intermediates	Catechol branch of beta-ketoadipate pathway	Beta-ketoadipate enol-lactone hydrolase (EC 3.1.1.24)	0.010445	0.004524	0.012774	0.00498	0.009848	0.01315	0.011553	0.011462	0.015107	0.008811	0.038307	0.010963974
Metabolism of Aromatic Compounds	Metabolism of central aromatic intermediates	Protocatechuate branch of beta-ketoadipate pathway	Beta-ketoadipyl CoA thiolase (EC 2.3.1.-)	0.013926	0.004524	0.007664	0.01494	0.004596	0.005357	0.008665	0.006977	0.009415	0.002203	0.011294	0.006113007
Virulence, Disease and Defense	Resistance to antibiotics and toxic compounds	Beta-lactamase	Beta-lactamase	0.068935	0.063336	0.089418	0.084661	0.055151	0.075978	0.068598	0.072262	0.08933	0.085909	0.011294	0.092759952
Virulence, Disease and Defense	Resistance to antibiotics and toxic compounds	BlaR1 Family Regulatory Sensor-transducer Disambiguation	Beta-lactamase (EC 3.5.2.6)	0.010793	0.031668	0.020438	0.01494	0.015101	0.014124	0.014442	0.012459	0.034594	0.004406	0.02189	0.018772847
Virulence, Disease and Defense	Resistance to antibiotics and toxic compounds	Beta-lactamase	Beta-lactamase class C and other penicillin binding proteins	0.009052	0.020358	0.017884	0.00249	0.026262	0.024839	0.023829	0.026911	0.026493	0.008811	0.053792	0.021651876
Carbohydrates	Monosaccharides	Mannose Metabolism	Beta-mannosidase (EC 3.2.1.25)	0.012186	0.031668	0.010219	0.0249	0.002626	0.015098	0.010831	0.017443	0.006349	0.002203	0.000233	0.011673871
Nucleosides and Nucleotides	Pyrimidines	Novel non-oxidative pathway of Uracil catabolism	Beta-ureidopropionase (EC 3.5.1.6)	0.016015	0.013572	0.015329	0.01494	0.007222	0.010228	0.015164	0.011462	0.021457	0.008811	0.000931	0.006704588
Carbohydrates	NULL	Sugar utilization in Thermotogales	Beta-xylosidase (EC 3.2.1.37)	0.037253	0.074645	0.05876	0.034861	0.022979	0.017046	0.021663	0.015948	0.044665	0.019825	0.001979	0.04117406
Membrane Transport	NULL	Ton and Tol transport systems	Biopolymer transport protein ExbD/TolR	0.015319	0.024882	0.017884	0.037351	0.040706	0.037015	0.035382	0.053325	0.038316	0.013217	0.015719	0.032458095
Stress Response	Acid stress	Acid resistance mechanisms	Biosynthetic arginine decarboxylase (EC 4.1.1.19)	0.097484	0.038454	0.043432	0.062251	0.036111	0.030684	0.041881	0.052826	0.08101	0.008811	0.057053	0.066099353
Amino Acids and Derivatives	Aromatic amino acids and derivatives	Phenylalanine and Tyrosine Branches from Chorismate	Biosynthetic Aromatic amino acid aminotransferase alpha (EC 2.6.1.57)	0.069632	0.018096	0.033212	0.01494	0.01707	0.015098	0.010109	0.006479	0.014669	0.006608	0.001281	0.019127796
Amino Acids and Derivatives	Aromatic amino acids and derivatives	Phenylalanine and Tyrosine Branches from Chorismate	Biosynthetic Aromatic amino acid aminotransferase beta (EC 2.6.1.57)	0.034119	0.036192	0.043432	0.049801	0.033484	0.040424	0.043325	0.046846	0.048825	0.008811	0.02154	0.043303753

Fatty Acids, Lipids, and Isoprenoids	Fatty acids	Fatty Acid Biosynthesis FASII	Biotin carboxyl carrier protein	0.029245	0.029406	0.040877	0.01992	0.035454	0.028248	0.037548	0.053823	0.052109	0.026433	0.015253	0.043698141
Fatty Acids, Lipids, and Isoprenoids	Fatty acids	Fatty Acid Biosynthesis FASII	Biotin carboxylase of acetyl-CoA carboxylase (EC 6.3.4.14)	0.269126	0.074645	0.102192	0.102092	0.093887	0.097408	0.107591	0.091698	0.113633	0.105734	0.121091	0.122654528
Amino Acids and Derivatives	Branched-chain amino acids	HMG CoA Synthesis	Biotin carboxylase of methylcrotonyl-CoA carboxylase (EC 6.3.4.14)	0.000348	0.002262	0.010219	0.00747	0.003939	0.000487	0.001444	0.003987	0.003722	0.008811	0.001397	0.002129693
Miscellaneous	Plant-Prokaryote DOE project	Biotin biosynthesis Experimental	Biotin synthase (EC 2.8.1.6)	0.035512	0.018096	0.022993	0.034861	0.030858	0.023865	0.045491	0.044852	0.040286	0.068286	0.083599	0.03770345
Cofactors, Vitamins, Prosthetic Groups, Pigments	Biotin	Biotin biosynthesis	Biotin-protein ligase (EC 6.3.4.15)	0.008356	0.009048	0.010219	0.01743	0.01707	0.01802	0.014442	0.016446	0.015107	0.004406	0.01176	0.014710656
Amino Acids and Derivatives	Branched-chain amino acids	Branched chain amino acid degradation regulons	Branched-chain alpha-keto acid dehydrogenase, E1 component, alpha subunit (EC 1.2.4.4)	0.051876	0.029406	0.033212	0.042331	0.021666	0.023378	0.028161	0.048341	0.05189	0.017622	0.05845	0.031551004
Amino Acids and Derivatives	Branched-chain amino acids	Branched chain amino acid degradation regulons	Branched-chain alpha-keto acid dehydrogenase, E1 component, beta subunit (EC 1.2.4.4)	0.070328	0.042978	0.048541	0.067231	0.02101	0.035554	0.037548	0.063292	0.105313	0.072692	0.060313	0.060341295
Virulence, Disease and Defense	NULL	C jejuni colonization of chick caeca	Branched-chain amino acid ABC transporter, amino acid-binding protein (TC 3.A.1.4.1)	0.026112	0.058812	0.056206	0.044821	0.077474	0.058932	0.039715	0.03638	0.039848	0.017622	0.010712	0.035218808
Amino Acids and Derivatives	Branched-chain amino acids	Leucine Degradation and HMG-CoA Metabolism	Branched-chain amino acid aminotransferase (EC 2.6.1.42)	0.071372	0.099527	0.102192	0.102092	0.096514	0.091563	0.103258	0.101167	0.110787	0.09472	0.102811	0.10293515
Membrane Transport	ABC transporters	ABC transporter branched-chain amino acid (TC 3.A.1.4.1)	Branched-chain amino acid transport ATP-binding protein LivF (TC 3.A.1.4.1)	0.156671	0.160601	0.178836	0.109562	0.153634	0.138319	0.098203	0.058308	0.096556	0.110139	0.080106	0.108377699
Membrane Transport	ABC transporters	ABC transporter branched-chain amino acid (TC 3.A.1.4.1)	Branched-chain amino acid transport ATP-binding protein LivG (TC 3.A.1.4.1)	0.080076	0.101789	0.13285	0.129482	0.137877	0.163645	0.087372	0.059803	0.098526	0.052867	0.05519	0.10739173
Membrane Transport	ABC transporters	ABC transporter branched-chain amino acid (TC 3.A.1.4.1)	Branched-chain amino acid transport system permease protein LivM (TC 3.A.1.4.1)	0.10967	0.101789	0.081754	0.099602	0.122776	0.121273	0.085928	0.041364	0.076193	0.01542	0.100017	0.078443684
Amino Acids and Derivatives	Branched-chain amino acids	Isoleucine degradation	Butyrate kinase (EC 2.7.2.7)	0.005919	0.002262	0.00511	0.01494	0.004596	0.005357	0.005777	0.005482	0.001971	0.002203	0.002329	0.010372393
Carbohydrates	Organic acids	Isobutyryl-CoA to Propionyl-CoA Module	Butyryl-CoA dehydrogenase (EC 1.3.99.2)	0.166419	0.248818	0.201829	0.256474	0.196967	0.204557	0.175466	0.173429	0.198366	0.317202	0.088373	0.187925667
Miscellaneous	Plant-Prokaryote DOE project	COG0523	C4-type zinc finger protein, DksA/TraR family	0.106884	0.020358	0.022993	0.01743	0.022323	0.022404	0.029605	0.038374	0.020581	0.01542	0.030971	0.021020856
Virulence, Disease and Defense	Resistance to antibiotics and toxic compounds	Cadmium resistance	Cadmium-transporting ATPase (EC 3.6.3.3)	0.000348	0.004524	0.00511	0.00249	0.005909	0.005357	0.005777	0.004485	0.007006	0.004406	0.003027	0.006507395
Regulation and Cell signaling	NULL	cAMP signaling in bacteria	cAMP-binding proteins - catabolite gene activator and regulatory subunit of cAMP-dependent protein kinases	0.016712	0.013572	0.015329	0.01245	0.022979	0.028735	0.016608	0.03638	0.021238	0.028636	0.002212	0.019246112
Cell Wall and Capsule	Capsular and extracellular polysacchrides	Rhamnose containing glycans	capsular polysaccharide biosynthesis protein	0.010793	0.020358	0.015329	0.00996	0.010505	0.016559	0.004333	0.012459	0.011166	0.002203	0.000233	0.01013576
Amino Acids and Derivatives	Arginine; urea cycle, polyamines	Polyamine Metabolism	Carbamate kinase (EC 2.7.2.2)	0.012186	0.036192	0.020438	0.079681	0.032171	0.027274	0.025273	0.010964	0.015545	0.004406	0.000466	0.024294273
Cell Division and Cell Cycle	NULL	Macromolecular synthesis operon	Carbamoyl-phosphate synthase large chain (EC 6.3.5.5)	0.350943	0.393585	0.342343	0.390936	0.357166	0.326804	0.355988	0.283567	0.32842	0.654228	0.338706	0.316219937
Clustering-based subsystems	Probably Pyrimidine biosynthesis-related	CBSS-306254.1.peg.1508	Carbamoyl-phosphate synthase small chain (EC 6.3.5.5)	0.141352	0.108575	0.091973	0.119522	0.099796	0.081823	0.124198	0.104157	0.097212	0.116748	0.089188	0.107233975
Carbohydrates	Glycoside hydrolases	Predicted carbohydrate hydrolases	Carbohydrate Esterase Family 4	0.002785	0.004524	0.002555	0.00996	0.00197	0.003896	0.006499	0.00299	0.002846	0.013217	0.000233	0.002800152
Respiration	NULL	Carbon monoxide induced hydrogenase	Carbon monoxide dehydrogenase CooS subunit (EC 1.2.99.2)	0.031334	0.047502	0.061315	0.062251	0.055151	0.060393	0.039715	0.011961	0.005474	0.008811	0.001514	0.027528251
Respiration	NULL	Carbon monoxide dehydrogenase maturation factors	carbon monoxide dehydrogenase D protein	0.010097	0.015834	0.015329	0.02241	0.009192	0.012663	0.014442	0.010466	0.028463	0.008811	0.001863	0.009662495
Respiration	Electron donating reactions	CO Dehydrogenase	carbon monoxide dehydrogenase E protein	0.008704	0.004524	0.002555	0.00498	0.006566	0.010228	0.006499	0.015449	0.02496	0.002203	0.001281	0.005087599
Respiration	Electron donating reactions	CO Dehydrogenase	Carbon monoxide dehydrogenase large chain (EC 1.2.99.2)	0.03969	0.02262	0.074089	0.059761	0.045302	0.03458	0.038993	0.037875	0.117137	0.019825	0.001281	0.013921881
Clustering-based subsystems	NULL	CBSS-314269.3.peg.1840	Carbon monoxide dehydrogenase medium chain (EC 1.2.99.2)	0.01706	0.009048	0.010219	0.00747	0.008535	0.011689	0.014442	0.020931	0.021895	0.013217	0.001514	0.002800152
Respiration	Electron donating reactions	CO Dehydrogenase	Carbon monoxide dehydrogenase small chain (EC 1.2.99.2)	0.088432	0.01131	0.043432	0.00747	0.013131	0.009254	0.01733	0.015948	0.03941	0.008811	0.000699	0.010845658
Miscellaneous	Plant-Prokaryote DOE project	COG0523	Carbon starvation protein A	0.025067	0.047502	0.033212	0.032371	0.031515	0.037015	0.021663	0.037875	0.035688	0.006608	0.029225	0.020192643
Virulence, Disease and Defense	NULL	Bacterial cyanide production and tolerance mechanisms	Carbonic anhydrase (EC 4.2.1.1)	0.020541	0.031668	0.045986	0.049801	0.040706	0.036041	0.033216	0.041364	0.034375	0.026433	0.087675	0.039162684
Clustering-based subsystems	NULL	CBSS-224911.1.peg.435	Carboxyl-terminal protease (EC 3.4.21.102)	0.022282	0.02262	0.033212	0.02241	0.048585	0.035554	0.039715	0.034885	0.03744	0.013217	0.013623	0.040740234
Fatty Acids, Lipids, and Isoprenoids	Phospholipids	Cardiolipin synthesis	Cardiolipin synthetase (EC 2.7.8.-)	0.042127	0.054288	0.045986	0.052291	0.050555	0.056984	0.031772	0.035882	0.051671	0.024231	0.00524	0.049259005
Stress Response	Oxidative stress	Protection from Reactive Oxygen Species	Catalase (EC 1.11.1.6)	0.108277	0.135719	0.076644	0.094622	0.0801	0.094973	0.088094	0.083725	0.128084	0.066084	0.107468	0.104315507
Virulence, Disease and Defense	Resistance to antibiotics and toxic compounds	Cobalt-zinc-cadmium resistance	Cation efflux system protein CusA	0.300112	0.294058	0.44198	0.348606	0.198936	0.245468	0.300387	0.324433	0.39498	0.328216	0.080805	0.478707608
Clustering-based subsystems	Two related proteases	CBSS-257314.1.peg.676	CDP-diacylglycerol--glycerol-3-phosphate 3-phosphatidyltransferase (EC 2.7.8.5)	0.040386	0.040716	0.053651	0.049801	0.032828	0.041885	0.035382	0.034885	0.030215	0.024231	0.022472	0.029263556
Fatty Acids, Lipids, and Isoprenoids	Phospholipids	Glycerolipid and Glycerophospholipid Metabolism in Bacteria	CDP-diacylglycerol--serine O-phosphatidyltransferase (EC 2.7.8.8)	0.027504	0.024882	0.040877	0.034861	0.02101	0.030684	0.01733	0.03339	0.026274	0.026433	0.011178	0.031393249
Dormancy and Sporulation	NULL	Persister Cells	Cell division inhibitor	0.021934	0.047502	0.030658	0.01992	0.01904	0.016072	0.024551	0.026413	0.032185	0.019825	0.001863	0.03040728
Clustering-based subsystems	Cell Division	Cell Division Cluster	Cell division protein FtsA	0.143093	0.081431	0.06898	0.084661	0.123432	0.084258	0.068598	0.118111	0.080791	0.215873	0.164987	0.097334847
Clustering-based subsystems	NULL	Cell division-ribosomal stress proteins cluster	Cell division protein FtsH (EC 3.4.24.-)	0.200539	0.316678	0.245261	0.385956	0.275753	0.273229	0.298943	0.232734	0.238214	0.26874	0.173836	0.238446713
Motility and Chemotaxis	Flagellar motility in Prokaryota	Flagellum in Campylobacter	Cell division protein FtsI [Peptidoglycan synthetase] (EC 2.4.1.129)	0.044216	0.054288	0.066425	0.054781	0.067625	0.086693	0.077985	0.083725	0.07904	0.006608	0.044478	0.094850206
Clustering-based subsystems	NULL	Bacterial Cell Division	Cell division protein FtsJ	0.016363	0.004524	0.002555	0.00498	0.007222	0.004383	0.012998	0.008472	0.007882	0.002203	0.020143	0.006546833
Clustering-based subsystems	NULL	Bacterial RNA-metabolizing Zn-dependent hydrolases	Cell division protein FtsK	0.099225	0.092741	0.150733	0.174303	0.147725	0.164132	0.156692	0.12758	0.138155	0.147587	0.135296	0.156414102
Clustering-based subsystems	NULL	Bacterial Cell Division	Cell division protein FtsW	0.041431	0.05655	0.048541	0.047311	0.047929	0.061854	0.056323	0.045849	0.049482	0.068286	0.02643	0.059710275
Clustering-based subsystems	Protein export?	CBSS-393121.3.peg.2760	Cell division protein FtsX	0.016712	0.018096	0.030658	0.034861	0.028232	0.024352	0.033216	0.021429	0.032842	0.035245	0.021191	0.035573757
Clustering-based subsystems	NULL	Bacterial Cell Division	Cell division protein FtsZ (EC 3.4.24.-)	0.085995	0.165125	0.148178	0.139442	0.126715	0.11689	0.122754	0.110636	0.097869	0.178426	0.101763	0.105538108
RNA Metabolism	RNA processing and modification	16S rRNA modification within P site of ribosome	Cell division protein MraZ	0.014623	0.009048	0.017884	0.00996	0.015101	0.012663	0.015886	0.015449	0.014013	0.019825	0.020842	0.018102389
Clustering-based subsystems	NULL	Bacterial Cell Division	Cell division transporter, ATP-binding protein FtsE (TC 3.A.5.1.1)	0.070328	0.052026	0.043432	0.057271	0.047272	0.049191	0.050546	0.0608	0.053423	0.083706	0.041101	0.074026543
Clustering-based subsystems	NULL	Bacterial Cell Division	Cell division trigger factor (EC 5.2.1.8)	0.036905	0.036192	0.017884	0.01245	0.035454	0.036528	0.033216	0.044852	0.038316	0.008811	0.079757	0.067755781
Stress Response	Heat shock	Heat shock dnaK gene cluster extended	Chaperone protein DnaJ	0.140656	0.092741	0.125185	0.124502	0.11818	0.115428	0.127809	0.130072	0.09984	0.041853	0.04145	0.113899124
Protein Metabolism	Protein folding	Protein chaperones	Chaperone protein DnaK	0.290016	0.361917	0.247816	0.308765	0.336813	0.292711	0.315551	0.235724	0.255511	0.464788	0.256038	0.284077352
Protein Metabolism	Protein folding	Protein chaperones	Chaperone protein HscA	0.018801	0.015834	0.020438	0.039841	0.025606	0.018995	0.025273	0.02741	0.007444	0.004406	0.108633	0.023032233
Protein Metabolism	Protein folding	Protein chaperones	Chaperone protein HtpG	0.080424	0.052026	0.074089	0.057271	0.104392	0.110558	0.111923	0.12459	0.110787	0.185034	0.181287	0.12513917
Motility and Chemotaxis	NULL	Bacterial Chemotaxis	Chemotaxis protein CheD	0.003482	0.006786	0.002555	0.00996	0.007222	0.013637	0.010109	0.006977	0.003503	0.011014	0.000699	0.011516116
Motility and Chemotaxis	NULL	Bacterial Chemotaxis	Chemotaxis protein methyltransferase CheR (EC 2.1.1.80)	0.041431	0.05655	0.038322	0.039841	0.083383	0.076465	0.102536	0.081233	0.125894	0.026433	0.039471	0.096348878
Motility and Chemotaxis	NULL	Bacterial Chemotaxis	Chemotaxis regulator - transmits chemoreceptor signals to flagelllar motor components CheY	0.035164	0.024882	0.00511	0.01743	0.032828	0.037502	0.064265	0.041364	0.04992	0.03965	0.02515	0.052650738
Motility and Chemotaxis	NULL	Bacterial Chemotaxis	Chemotaxis response regulator protein-glutamate methylesterase CheB (EC 3.1.1.61)	0.025764	0.036192	0.038322	0.054781	0.091918	0.066237	0.077263	0.076249	0.063932	0.01542	0.03004	0.084675007

Carbohydrates	Aminosugars	Chitin and N-acetylglucosamine utilization	Chitinase (EC 3.2.1.14)	0.006615	0.027144	0.028103	0.01245	0.013131	0.02143	0.018052	0.011462	0.009196	0.004406	0.001397	0.014237391
Miscellaneous	Plant-Prokaryote DOE project	Niacin-Choline transport and metabolism	Choline dehydrogenase (EC 1.1.99.1)	0.02263	0.01131	0.022993	0.02739	0.02101	0.022891	0.019496	0.045849	0.069187	0.01542	0.212259	0.033325748
Membrane Transport	NULL	Choline Transport	Choline-sulfatase (EC 3.1.6.6)	0.082165	0.058812	0.081754	0.052291	0.094544	0.099356	0.073653	0.084223	0.043351	0.008811	0.001514	0.044210844
Amino Acids and Derivatives	Aromatic amino acids and derivatives	Chorismate Synthesis	Chorismate mutase I (EC 5.4.99.5)	0.02263	0.01131	0.022993	0.00996	0.024949	0.017533	0.018052	0.026413	0.022333	0.024231	0.014904	0.01900948
Amino Acids and Derivatives	Aromatic amino acids and derivatives	Common Pathway For Synthesis of Aromatic Compounds (DAHP synthase to chorismate)	Chorismate synthase (EC 4.2.3.5)	0.077639	0.085955	0.071534	0.094622	0.084039	0.092051	0.098203	0.095685	0.077726	0.035245	0.077545	0.080967764
Clustering-based subsystems	NULL	DNA replication cluster 1	Chromosomal replication initiator protein DnaA	0.087039	0.147029	0.112411	0.077191	0.113584	0.093025	0.137918	0.150006	0.091082	0.277551	0.090353	0.114609022
DNA Metabolism	DNA replication	Plasmid replication	Chromosome (plasmid) partitioning protein ParA	0.153189	0.088217	0.071534	0.067231	0.086009	0.072569	0.090261	0.076249	0.078821	0.03965	0.066717	0.076471746
Cell Division and Cell Cycle	NULL	Bacterial Cytoskeleton	Chromosome (plasmid) partitioning protein ParB	0.025764	0.070121	0.05876	0.047311	0.050555	0.048704	0.041881	0.048839	0.040943	0.019825	0.023752	0.052729615
Clustering-based subsystems	Fatty acid metabolic cluster	COG1399	Chromosome partition protein smc	0.117329	0.115361	0.074089	0.112052	0.150351	0.151956	0.124198	0.154492	0.109473	0.090314	0.039471	0.08743572
Cell Division and Cell Cycle	NULL	Cyanobacterial Circadian Clock	Circadian input kinase A	0.007659	0.018096	0.017884	0.00747	0.023636	0.013637	0.019496	0.015948	0.041162	0.004406	0.000582	0.026739476
Carbohydrates	One-carbon Metabolism	Serine-glyoxylate cycle	Citrate synthase (si) (EC 2.3.3.1)	0.118374	0.108575	0.143069	0.109562	0.126059	0.128579	0.129253	0.138046	0.124581	0.169615	0.150432	0.148960178
Fatty Acids, Lipids, and Isoprenoids	Isoprenoids	Acyclic terpenes utilization	Citronellyl-CoA dehydrogenase	0.008356	0.009048	0.012774	0.01494	0.005252	0.00828	0.005055	0.00598	0.010728	0.002203	0.000116	0.007098976
Membrane Transport	Protein secretion system, Type VI	Type VI secretion systems	ClpB protein	0.261815	0.411681	0.355117	0.380976	0.346661	0.305374	0.329992	0.292538	0.302366	0.51325	0.398669	0.29109745
Respiration	NULL	Carbon monoxide induced hydrogenase	CO dehydrogenase accessory protein CooC (nickel insertion)	0.021934	0.074645	0.012774	0.049801	0.054494	0.04286	0.026717	0.009469	0.001752	0.004406	0.001164	0.027094425
Carbohydrates	Sugar alcohols	Propanediol utilization	CoA-acylating propionaldehyde dehydrogenase	0.006267	0.002262	0.00511	0.00249	0.006566	0.001948	0.00361	0.000997	0.001752	0.002203	0.000116	0.003391733
Stress Response	Oxidative stress	CoA disulfide thiol-disulfide redox system	CoA-disulfide reductase (EC 1.8.1.14)	0.01323	0.013572	0.00511	0.01494	0.005252	0.007306	0.005777	0.003987	0.003503	0.006608	0.000931	0.00761168
Cofactors, Vitamins, Prosthetic Groups, Pigments	Tetrapyrroles	Cobalamin synthesis	Cob(I)alamin adenosyltransferase (EC 2.5.1.17)	0.021238	0.038454	0.035767	0.02739	0.016414	0.015585	0.018774	0.011961	0.017954	0.019825	0.009315	0.017274175
Protein Metabolism	Protein processing and modification	G3E family of P-loop GTPases (metallocenter biosynthesis)	Cob(I)alamin adenosyltransferase PduO (EC 2.5.1.17)	0.016712	0.009048	0.035767	0.00747	0.021666	0.021917	0.022385	0.038872	0.028901	0.01542	0.005007	0.019995449
Cofactors, Vitamins, Prosthetic Groups, Pigments	Tetrapyrroles	Coenzyme B12 biosynthesis	Cobalt-precorrin-4 C11-methyltransferase (EC 2.1.1.133)	0.011141	0.004524	0.010219	0.00996	0.007222	0.008767	0.018052	0.010466	0.009196	0.01542	0.001863	0.003943875
Virulence, Disease and Defense	Resistance to antibiotics and toxic compounds	Cobalt-zinc-cadmium resistance	Cobalt-zinc-cadmium resistance protein	0.075898	0.072383	0.05876	0.064741	0.046615	0.051626	0.037548	0.052328	0.07072	0.044056	0.071723	0.070161545
Virulence, Disease and Defense	Resistance to antibiotics and toxic compounds	Cobalt-zinc-cadmium resistance	Cobalt-zinc-cadmium resistance protein CzcD	0.018801	0.013572	0.012774	0.01992	0.015101	0.015098	0.01372	0.016446	0.026055	0.01542	0.004774	0.026818353
Respiration	Electron donating reactions	H2:CoM-S-S-HTP oxidoreductase	CoB--CoM heterodisulfide reductase subunit A (EC 1.8.98.1)	0.208895	0.432039	0.263144	0.435757	0.294794	0.257157	0.143695	0.056813	0.022333	0.006608	0.000349	0.170690931
Carbohydrates	One-carbon Metabolism	Methanogenesis	CoB--CoM heterodisulfide reductase subunit B (EC 1.8.98.1)	0.017408	0.074645	0.028103	0.064741	0.032171	0.027761	0.018052	0.004485	0.011823	0.002203	0.000582	0.017471368
Cofactors, Vitamins, Prosthetic Groups, Pigments	Tetrapyrroles	Coenzyme B12 biosynthesis	CobN component of cobalt chelatase involved in B12 biosynthesis	0.014971	0.02262	0.012774	0.00747	0.020353	0.017046	0.023107	0.037377	0.030871	0.006608	0.002096	0.00603413
Protein Metabolism	Protein processing and modification	G3E family of P-loop GTPases (metallocenter biosynthesis)	CobW GTPase involved in cobalt insertion for B12 biosynthesis	0.006267	0.004524	0.00511	0.00249	0.00197	0.006819	0.010109	0.009469	0.007882	0.004406	0.000466	0.000907091
Cofactors, Vitamins, Prosthetic Groups, Pigments	Tetrapyrroles	Coenzyme B12 biosynthesis	Cobyric acid synthase	0.026112	0.018096	0.025548	0.02739	0.04202	0.024839	0.03827	0.023423	0.02693	0.006608	0.004424	0.028317026
Cofactors, Vitamins, Prosthetic Groups, Pigments	Tetrapyrroles	Cobalamin synthesis	Cobyrinic acid A,C-diamide synthase	0.010097	0.031668	0.033212	0.034861	0.028232	0.038476	0.035382	0.018938	0.016421	0.002203	0.000815	0.022835039
Cofactors, Vitamins, Prosthetic Groups, Pigments	Coenzyme F420	Coenzyme F420 synthesis	Coenzyme F420-0:L-glutamate ligase	0.005919	0.004524	0.00511	0.00996	0.003283	0.001461	0.007943	0.002492	0.006131	0.002203	0.000116	0.003076223
Cofactors, Vitamins, Prosthetic Groups, Pigments	Quinone cofactors	Pyrroloquinoline Quinone biosynthesis	Coenzyme PQQ synthesis protein B	0.001044	0.002262	0.012774	0.00249	0.00197	0.00487	0.001444	0.004485	0.006131	0.002203	0.028061	0.009544179
Cofactors, Vitamins, Prosthetic Groups, Pigments	Quinone cofactors	Coenzyme PQQ synthesis	Coenzyme PQQ synthesis protein E	0.000348	0.004524	0.007664	0.00747	0.003939	0.005357	0.007943	0.008472	0.012261	0.011014	0.073004	0.012304892
RNA Metabolism	RNA processing and modification	tRNA modification yeast cytoplasmic	COG0009 Sua5 subfamily, required for N6-threonylcarbamoyl adenosine t(6)A37 modification in tRNA	0.006963	0.01131	0.010219	0.01245	0.010505	0.008767	0.018052	0.032892	0.01445	0.006608	0.000466	0.005797497
Miscellaneous	Plant-Prokaryote DOE project	YrdC-YciO-Sua5 protein family	COG0009 YrdC subfamily, required for N6-threonylcarbamoyl adenosine t(6)A37 modification in tRNA	0.024719	0.020358	0.015329	0.01992	0.007222	0.016559	0.010109	0.008472	0.015107	0.006608	0.006171	0.021336366
Miscellaneous	Plant-Prokaryote DOE project	COG0398	COG0398: uncharacterized membrane protein	0.005222	0.009048	0.015329	0.00747	0.022979	0.023378	0.015886	0.013954	0.017954	0.01542	0.029458	0.015854379
Protein Metabolism	Protein biosynthesis	Universal GTPases	COG0536: GTP-binding protein Obg	0.055357	0.042978	0.051096	0.049801	0.04202	0.043347	0.059933	0.05482	0.047949	0.070489	0.048436	0.053360635
Miscellaneous	Plant-Prokaryote DOE project	YrdC-YciO-Sua5 protein family	COG0613, Predicted metal-dependent phosphoesterases (PHP family)	0.01706	0.024882	0.002555	0.00498	0.013131	0.015585	0.009387	0.006977	0.010509	0.004406	0.01176	0.010963974
RNA Metabolism	RNA processing and modification	tRNA modification Archaea	COG1180: Radical SAM, Pyruvate-formate lyase-activating enzyme like	0.06615	0.081431	0.094528	0.052291	0.071565	0.060393	0.049102	0.042859	0.030871	0.013217	0.086161	0.053755023
Cofactors, Vitamins, Prosthetic Groups, Pigments	Folate and pterines	YgfZ-Iron	COG1272: Predicted membrane protein hemolysin III homolog	0.012186	0.004524	0.010219	0.00996	0.007879	0.011202	0.005055	0.014452	0.01445	0.011014	0.000233	0.013803564
RNA Metabolism	RNA processing and modification	tRNA modification Archaea	COG1355, Predicted dioxygenase	0.032379	0.018096	0.035767	0.01743	0.034141	0.029709	0.029605	0.013954	0.007006	0.004406	0.000466	0.015459992
Clustering-based subsystems	Three hypotheticals linked to lipoprotein biosynthesis	CBSS-188.1.peg.9880	COG1496: Uncharacterized conserved protein	0.026808	0.009048	0.033212	0.00996	0.01707	0.013637	0.015886	0.028905	0.013794	0.011014	0.011061	0.014237391
Miscellaneous	Plant-Prokaryote DOE project	Experimental-PTPS	COG1565: Uncharacterized conserved protein	0.017408	0.01131	0.017884	0.0249	0.017727	0.017533	0.010109	0.015948	0.012918	0.008811	0.001397	0.015459992
Miscellaneous	Plant-Prokaryote DOE project	At1g69340 At2g40600	COG2110, Macro domain, possibly ADP-ribose binding module	0.033423	0.036192	0.025548	0.057271	0.035454	0.035067	0.022385	0.032892	0.021019	0.006608	0.0085	0.018891164
Carbohydrates	Glycoside hydrolases	Predicted carbohydrate hydrolases	COG2152 predicted glycoside hydrolase	0.011489	0.031668	0.010219	0.01992	0.021666	0.01315	0.01733	0.011961	0.011604	0.004406	0.002096	0.025043609
Miscellaneous	Plant-Prokaryote DOE project	At5g04520 AT1G06240	COG2833: uncharacterized protein	0.011141	0.01131	0.007664	0.00249	0.003283	0.005844	0.012275	0.012459	0.005474	0.008811	0.007335	0.010017444
Miscellaneous	Plant-Prokaryote DOE project	COG3380 COG2907	COG2907: Amine oxidase, flavin-containing	0.018801	0.018096	0.020438	0.032371	0.028232	0.050652	0.032494	0.047843	0.035688	0.013217	0.009548	0.026818353
Cell Wall and Capsule	NULL	YjeE	COG3178: Predicted phosphotransferase related to Ser/Thr protein kinases	0.011141	0.002262	0.015329	0.01245	0.009848	0.011689	0.022385	0.021429	0.021238	0.002203	0.000699	0.016998103
Miscellaneous	Plant-Prokaryote DOE project	COG3533	COG3866 Pectate lyase	0.005222	0.018096	0.007664	0.02739	0.01707	0.019969	0.007221	0.007974	0.008758	0.002203	0.000233	0.011871065
Cofactors, Vitamins, Prosthetic Groups, Pigments	Folate and pterines	Pterin metabolism	COGs COG3146	0.008356	0.009048	0.033212	0.02739	0.026262	0.018508	0.01372	0.014452	0.01664	0.004406	0.013506	0.015183921
Stress Response	Cold shock	Cold shock, CspA family of proteins	Cold shock protein CspA	0.026808	0.020358	0.022993	0.02241	0.01904	0.027761	0.03466	0.016446	0.023208	0.013217	0.000233	0.0310383
Stress Response	Cold shock	Cold shock, CspA family of proteins	Cold shock protein CspE	0.000348	0.013572	0.015329	0.01743	0.004596	0.004383	0.00361	0.004984	0.008101	0.006608	0.00163	0.00855821
RNA Metabolism	RNA processing and modification	ATP-dependent RNA helicases, bacterial	Cold-shock DEAD-box protein A	0.029245	0.027144	0.035767	0.044821	0.04202	0.038476	0.03827	0.061298	0.051671	0.154195	0.099318	0.088579444
Phages, Prophages, Transposable elements, Plasmids	Transposable elements	CBSS-203122.12.peg.188	ComM-related protein	0.043172	0.076907	0.089418	0.054781	0.058433	0.049191	0.064988	0.044354	0.047074	0.017622	0.014088	0.060380734
Miscellaneous	Plant-Prokaryote DOE project	Competence or DNA damage-inducible protein CinA and related protein families	Competence/damage-inducible protein CinA	0.035164	0.052026	0.022993	0.01743	0.039393	0.040424	0.032494	0.029902	0.031528	0.011014	0.001514	0.045472884
Virulence, Disease and Defense	Resistance to antibiotics and toxic compounds	Copper homeostasis: copper tolerance	Copper homeostasis protein CutE	0.016712	0.027144	0.033212	0.039841	0.034141	0.034093	0.029605	0.017941	0.029996	0.006608	0.003144	0.022913917
Regulation and Cell signaling	NULL	Orphan regulatory proteins	Copper-sensing two-component system response regulator CpxR	0.001044	0.020358	0.002555	0.01494	0.009192	0.002922	0.00361	0.001993	0.004379	0.017622	0.001281	0.006507395
Clustering-based subsystems	NULL	CBSS-196620.1.peg.2477	Copper-translocating P-type ATPase (EC 3.6.3.4)	0.17199	0.309892	0.201829	0.268924	0.27641	0.2518	0.230345	0.216787	0.274997	0.207062	0.101996	0.29650056

Cofactors, Vitamins, Prosthetic Groups, Pigments	Tetrapyrroles	Heme and Siroheme Biosynthesis	Coproporphyrinogen III oxidase, aerobic (EC 1.3.3.3)	0.051876	0.009048	0.012774	0.039841	0.014444	0.027274	0.03466	0.051829	0.041162	0.037447	0.066949	0.030288964
Cofactors, Vitamins, Prosthetic Groups, Pigments	Tetrapyrroles	Heme and Siroheme Biosynthesis	Coproporphyrinogen III oxidase, oxygen-independent (EC 1.3.99.22)	0.037601	0.020358	0.030658	0.042331	0.045302	0.031171	0.042603	0.049338	0.047074	0.01542	0.06171	0.058448235
DNA Metabolism	CRISPs	CRISPRs	CRISPR-associated helicase Cas3	0.006963	0.006786	0.012774	0.01245	0.016414	0.012176	0.014442	0.010466	0.011823	0.004406	0.000815	0.008203261
Clustering-based subsystems	CRISPRs and associated hypotheticals	CBSS-216592.1.peg.3534	CRISPR-associated protein Cas1	0.019845	0.024882	0.015329	0.02988	0.021666	0.012176	0.014442	0.018439	0.014232	0.01542	0.107468	0.012423208
DNA Metabolism	DNA replication	DNA-replication	Crossover junction endodeoxyribonuclease RuvC (EC 3.1.22.4)	0.020193	0.027144	0.025548	0.02988	0.028232	0.024352	0.021663	0.023423	0.021019	0.048461	0.025383	0.02914524
Carbohydrates	Central carbohydrate metabolism	Ethylmalonyl-CoA pathway of C2 assimilation	Crotonyl-CoA carboxylase/reductase, ethylmalonyl-CoA producing	0.005571	0.006786	0.00511	0.00498	0.005909	0.011689	0.006499	0.012957	0.013356	0.006608	0.001514	0.0031551
Clustering-based subsystems	NULL	CTP synthase (EC 6.3.4.2) cluster	CTP synthase (EC 6.3.4.2)	0.12847	0.142505	0.160952	0.122012	0.208785	0.190433	0.194963	0.164957	0.14144	0.253321	0.128194	0.171282513
Nitrogen Metabolism	NULL	Cyanate hydrolysis	Cyanate hydratase (EC 4.2.1.104)	0.006267	0.004524	0.015329	0.00498	0.011161	0.006819	0.00361	0.004984	0.007006	0.004406	0.044594	0.006744027
Amino Acids and Derivatives	Arginine; urea cycle, polyamines	Cyanophycin Metabolism	Cyanophycin synthase (EC 6.3.2.29)(EC 6.3.2.30)	0.045609	0.027144	0.017884	0.0249	0.02101	0.044321	0.026717	0.02741	0.033061	0.004406	0.001979	0.015538869
Stress Response	Osmotic stress	Synthesis of osmoregulated periplasmic glucans	Cyclic beta-1,2-glucan synthase (EC 2.4.1.-)	0.015667	0.015834	0.015329	0.01992	0.007879	0.002922	0.005055	0.016944	0.025617	0.013217	0.001514	0.007414486
Amino Acids and Derivatives	Aromatic amino acids and derivatives	Chorismate Synthesis	Cyclohexadienyl dehydrogenase (EC 1.3.1.12)(EC 1.3.1.43)	0.020541	0.018096	0.012774	0.01245	0.01904	0.018508	0.030328	0.017941	0.016859	0.011014	0.023752	0.015262798
Amino Acids and Derivatives	Lysine, threonine, methionine, and cysteine	Methionine Biosynthesis	Cystathionine beta-lyase (EC 4.4.1.8)	0.012882	0.004524	0.00511	0.00996	0.012475	0.013637	0.023107	0.019436	0.01445	0.004406	0.013157	0.007256731
Amino Acids and Derivatives	Lysine, threonine, methionine, and cysteine	Methionine Degradation	Cystathionine beta-synthase (EC 4.2.1.22)	0.033771	0.020358	0.015329	0.00996	0.021666	0.023865	0.027439	0.039869	0.057145	0.123356	0.020143	0.047681455
Amino Acids and Derivatives	Lysine, threonine, methionine, and cysteine	Methionine Biosynthesis	Cystathionine gamma-lyase (EC 4.4.1.1)	0.032031	0.024882	0.030658	0.049801	0.034141	0.034093	0.041159	0.061298	0.087798	0.019825	0.003377	0.038847174
Amino Acids and Derivatives	Lysine, threonine, methionine, and cysteine	Methionine Biosynthesis	Cystathionine gamma-synthase (EC 2.5.1.48)	0.023327	0.009048	0.012774	0.01245	0.017727	0.023378	0.021663	0.020433	0.040286	0.004406	0.003144	0.017905195
Amino Acids and Derivatives	Alanine, serine, and glycine	Alanine biosynthesis	Cysteine desulfurase (EC 2.8.1.7)	0.207502	0.298582	0.206939	0.266434	0.275753	0.257644	0.284501	0.265128	0.251351	0.235698	0.252661	0.2698794
Cofactors, Vitamins, Prosthetic Groups, Pigments	Folate and pterines	YgfZ	Cysteine desulfurase (EC 2.8.1.7), IscS subfamily	0.032031	0.013572	0.020438	0.01494	0.007879	0.011202	0.018774	0.019934	0.017516	0.019825	0.016999	0.015933257
Miscellaneous	Plant-Prokaryote DOE project	At5g37530	Cysteine desulfurase (EC 2.8.1.7), NifS subfamily	0.021238	0.009048	0.00511	0.01245	0.015757	0.016559	0.00361	0.005482	0.004379	0.002203	0.024335	0.008755404
Amino Acids and Derivatives	Alanine, serine, and glycine	Alanine biosynthesis	Cysteine desulfurase (EC 2.8.1.7), SufS subfamily	0.000696	0.013572	0.015329	0.01494	0.005909	0.00828	0.018774	0.014452	0.011604	0.004406	0.001747	0.0082427
Amino Acids and Derivatives	Lysine, threonine, methionine, and cysteine	Methionine Biosynthesis	Cysteine synthase (EC 2.5.1.47)	0.093654	0.190007	0.137959	0.151892	0.144442	0.110558	0.116256	0.087711	0.112101	0.143181	0.116201	0.106642393
Amino Acids and Derivatives	Lysine, threonine, methionine, and cysteine	Cysteine Biosynthesis	Cysteine synthase B (EC 2.5.1.47)	0.057098	0.013572	0.033212	0.02241	0.014444	0.01802	0.01733	0.023423	0.019486	0.011014	0.048902	0.024175957
Miscellaneous	Plant-Prokaryote DOE project	Conserved gene cluster possibly involved in RNA metabolism	Cysteinyl-tRNA synthetase (EC 6.1.1.16)	0.102358	0.156077	0.099637	0.141932	0.137877	0.111532	0.142973	0.126085	0.103343	0.101328	0.049484	0.130542279
Nucleosides and Nucleotides	Pyrimidines	pyrimidine conversions	Cytidine deaminase (EC 3.5.4.5)	0.00383	0.01131	0.002555	0.032371	0.009192	0.005357	0.007221	0.004485	0.007225	0.011014	0.000815	0.014552901
Nucleosides and Nucleotides	Pyrimidines	pyrimidine conversions	Cytidylate kinase (EC 2.7.4.14)	0.037253	0.05655	0.056206	0.047311	0.045959	0.044321	0.033216	0.044852	0.028244	0.035245	0.044361	0.04910125
Respiration	NULL	Soluble cytochromes and functionally related electron carriers	cytochrome b-5	0.001393	0.002262	0.010219	0.00498	0.004596	0.002922	0.006499	0.008472	0.005036	0.002203	0.000116	0.002997345
Virulence, Disease and Defense	Resistance to antibiotics and toxic compounds	Copper homeostasis	Cytochrome c heme lyase subunit CcmF	0.069632	0.061074	0.076644	0.039841	0.064342	0.0526	0.075819	0.078741	0.079697	0.019825	0.118297	0.052059157
Respiration	Electron accepting reactions	Terminal cytochrome C oxidases	Cytochrome c oxidase polypeptide I (EC 1.9.3.1)	0.137871	0.092741	0.137959	0.082171	0.097827	0.082797	0.090983	0.128577	0.178442	0.156398	0.153693	0.114411828
Clustering-based subsystems	NULL	CBSS-316057.3.peg.563	Cytochrome c oxidase polypeptide II (EC 1.9.3.1)	0.028201	0.020358	0.030658	0.0249	0.035454	0.031171	0.028883	0.056315	0.074223	0.022028	0.041916	0.051546453
Respiration	Electron accepting reactions	Terminal cytochrome C oxidases	Cytochrome c oxidase polypeptide III (EC 1.9.3.1)	0.022282	0.024882	0.045986	0.034861	0.021666	0.027761	0.035382	0.049338	0.057364	0.033042	0.037259	0.031156616
Respiration	Electron accepting reactions	Terminal cytochrome C oxidases	Cytochrome c oxidase subunit CcoN (EC 1.9.3.1)	0.105144	0.054288	0.107302	0.057271	0.054494	0.091076	0.085928	0.091698	0.085389	0.185034	0.123769	0.097019337
Respiration	Electron accepting reactions	Terminal cytochrome C oxidases	Cytochrome c oxidase subunit CcoO (EC 1.9.3.1)	0.040386	0.024882	0.030658	0.02988	0.038737	0.042372	0.040437	0.055816	0.042038	0.01542	0.037608	0.040661356
Respiration	Electron accepting reactions	Terminal cytochrome C oxidases	Cytochrome c oxidase subunit CcoP (EC 1.9.3.1)	0.023675	0.027144	0.035767	0.01743	0.023636	0.020456	0.021663	0.038872	0.035031	0.033042	0.028643	0.030801668
Respiration	NULL	Soluble cytochromes and functionally related electron carriers	Cytochrome c4	0.020193	0.006786	0.010219	0.00747	0.013788	0.012663	0.011553	0.013456	0.014669	0.002203	0.03167	0.01013576
Stress Response	Oxidative stress	Protection from Reactive Oxygen Species	Cytochrome c551 peroxidase (EC 1.11.1.5)	0.006615	0.040716	0.035767	0.032371	0.013788	0.014611	0.01733	0.013456	0.013794	0.028636	0.011993	0.03707243
Cofactors, Vitamins, Prosthetic Groups, Pigments	Tetrapyrroles	Heme biosynthesis orphans	Cytochrome cd1 nitrite reductase (EC:1.7.2.1)	0.019497	0.01131	0.015329	0.00996	0.005909	0.009741	0.01733	0.010964	0.008758	0.011014	0.004657	0.018102389
Respiration	NULL	Biogenesis of c-type cytochromes	Cytochrome c-type biogenesis protein CcmC, putative heme lyase for CcmE	0.07555	0.01131	0.017884	0.0249	0.017727	0.022891	0.020218	0.026911	0.026712	0.01542	0.06171	0.020034888
Respiration	NULL	Biogenesis of c-type cytochromes	Cytochrome c-type biogenesis protein CcmE, heme chaperone	0.007311	0.01131	0.00511	0.02241	0.003939	0.012176	0.016608	0.013456	0.019705	0.008811	0.005007	0.01013576
Protein Metabolism	Protein folding	Periplasmic disulfide interchange	Cytochrome c-type biogenesis protein CcmG/DsbE, thiol:disulfide oxidoreductase	0.011489	0.009048	0.025548	0.00747	0.010505	0.005357	0.008665	0.012459	0.01861	0.004406	0.005589	0.01045127
Respiration	NULL	Biogenesis of c-type cytochromes	Cytochrome c-type biogenesis protein CcsA/ResC	0.016015	0.015834	0.030658	0.00747	0.024949	0.036041	0.01733	0.008472	0.006568	0.011014	0.000233	0.015578308
Protein Metabolism	Protein folding	Periplasmic disulfide interchange	Cytochrome c-type biogenesis protein DsbD, protein-disulfide reductase (EC 1.8.1.8)	0.02263	0.036192	0.038322	0.01743	0.022323	0.038963	0.024551	0.041862	0.053861	0.006608	0.036444	0.05225635
Respiration	Electron accepting reactions	Terminal cytochrome d ubiquinol oxidases	Cytochrome d ubiquinol oxidase subunit I (EC 1.10.3.-)	0.144137	0.061074	0.05876	0.054781	0.064999	0.049191	0.054878	0.042859	0.045541	0.037447	0.107585	0.075761848
Clustering-based subsystems	NULL	Conserved gene cluster associated with Met-tRNA formyltransferase	Cytochrome d ubiquinol oxidase subunit II (EC 1.10.3.-)	0.042127	0.058812	0.040877	0.0249	0.040706	0.04773	0.028883	0.017941	0.026055	0.011014	0.041916	0.046695486
Respiration	Electron accepting reactions	Terminal cytochrome oxidases	Cytochrome O ubiquinol oxidase subunit I (EC 1.10.3.-)	0.028897	0.009048	0.012774	0.01494	0.003283	0.001948	0.002888	0.002492	0.016421	0.022028	0.027595	0.00792719
Respiration	Electron accepting reactions	Terminal cytochrome O ubiquinol oxidase	Cytochrome O ubiquinol oxidase subunit II (EC 1.10.3.-)	0.005919	0.004524	0.012774	0.00249	0.000657	0.000487	0.001444	0.000997	0.003941	0.011014	0.009198	0.00378612
Respiration	NULL	Biogenesis of cytochrome c oxidases	Cytochrome oxidase biogenesis protein Sco1/SenC/PrrC, putative copper metallochaperone	0.017756	0.009048	0.020438	0.01494	0.01707	0.014611	0.023107	0.018439	0.020581	0.030839	0.002562	0.020823663
Respiration	Electron donating reactions	Hydrogenases	cytochrome-c3 hydrogenase delta chain	0.008356	0.004524	0.010219	0.00249	0.003939	0.000487	0.00361	0.001495	0.004598	0.004406	0.000582	0.00252408
Clustering-based subsystems	NULL	Bacterial Cell Division	Cytoplasmic axial filament protein CafA and Ribonuclease G (EC 3.1.4.-)	0.067891	0.097265	0.097082	0.067231	0.084696	0.108123	0.08665	0.098675	0.086046	0.14979	0.080456	0.084832762
Nucleosides and Nucleotides	Pyrimidines	pyrimidine conversions	Cytosine deaminase (EC 3.5.4.1)	0.010793	0.024882	0.040877	0.00747	0.013788	0.014124	0.010109	0.012459	0.022333	0.011014	0.061593	0.008715965
Carbohydrates	Central carbohydrate metabolism	Dehydrogenase complexes	Cytosol aminopeptidase PepA (EC 3.4.11.1)	0.083558	0.049764	0.079199	0.059761	0.063686	0.062341	0.079429	0.083725	0.07904	0.037447	0.091168	0.063535834
Miscellaneous	Plant-Prokaryote DOE project	Scaffold proteins for [4Fe-4S] cluster assembly (MRP family)	Cytosolic Fe-S cluster assembling factor NBP35	0.022282	0.049764	0.020438	0.02739	0.013131	0.012663	0.024551	0.00897	0.001971	0.002203	0.000931	0.013369738
Cell Wall and Capsule	Capsular and extracellular polysacchrides	Capsular heptose biosynthesis	D,D-heptose 7-phosphate kinase	0.006963	0.024882	0.038322	0.032371	0.026262	0.031171	0.010109	0.010466	0.012918	0.011014	0.000931	0.011989382
Miscellaneous	Plant-Prokaryote DOE project	COG0277	D-2-hydroxyglutarate dehydrogenase	0.010097	0.004524	0.00511	0.032371	0.007222	0.011689	0.011553	0.028905	0.027806	0.048461	0.002794	0.010254076
Cofactors, Vitamins, Prosthetic Groups, Pigments	Pyridoxine	Pyridoxin (Vitamin B6) Biosynthesis	D-3-phosphoglycerate dehydrogenase (EC 1.1.1.95)	0.124292	0.151553	0.181391	0.236554	0.195654	0.178257	0.172578	0.162465	0.179536	0.297376	0.203992	0.157715581
Carbohydrates	Central carbohydrate metabolism	Pyruvate Alanine Serine Interconversions	D-alanine aminotransferase (EC 2.6.1.21)	0.016363	0.027144	0.010219	0.02241	0.007222	0.019969	0.028161	0.020433	0.017297	0.004406	0.000582	0.011437239
Cell Wall and Capsule	NULL	Peptidoglycan Biosynthesis	D-alanine--D-alanine ligase (EC 6.3.2.4)	0.067194	0.110837	0.097082	0.114542	0.087978	0.053087	0.064265	0.0608	0.075099	0.107937	0.039937	0.090709136
Miscellaneous	Plant-Prokaryote DOE project	COG0523	D-alanyl-D-alanine carboxypeptidase (EC 3.4.16.4)	0.050483	0.027144	0.043432	0.067231	0.049898	0.057471	0.054878	0.081233	0.082105	0.024231	0.07184	0.04783921

Fatty Acids, Lipids, and Isoprenoids	NULL	Polyhydroxybutyrate metabolism	D-beta-hydroxybutyrate dehydrogenase (EC 1.1.1.30)	0.015319	0.018096	0.020438	0.0249	0.028888	0.033119	0.027439	0.040866	0.03328	0.011014	0.015369	0.013093667
Nucleosides and Nucleotides	NULL	Hydantoin metabolism	Deacetylases, including yeast histone deacetylase and acetoin utilization protein	0.038297	0.029406	0.045986	0.052291	0.036767	0.037989	0.038993	0.018439	0.036345	0.022028	0.005007	0.035770951
Protein Metabolism	Protein degradation	Protein degradation	Deblocking aminopeptidase (EC 3.4.11.-)	0.014971	0.013572	0.007664	0.01245	0.013788	0.016559	0.012998	0.013954	0.016859	0.006608	0.001164	0.009623056
Carbohydrates	One-carbon Metabolism	Serine-glyoxylate cycle	decarboxylase	0.307075	0.330249	0.362782	0.338645	0.31843	0.300017	0.297499	0.300511	0.399797	0.288565	0.427778	0.3288009
Stress Response	Detoxification	Uptake of selenate and selenite	DedA protein	0.018104	0.036192	0.010219	0.00747	0.002626	0.001461	0.001444	0.001495	0.006568	0.017622	0.000699	0.008755404
Clustering-based subsystems	NULL	CBSS-196620.1.peg.2477	Delta-1-pyrroline-5-carboxylate dehydrogenase (EC 1.5.1.12)	0.047698	0.061074	0.112411	0.054781	0.092574	0.083284	0.078707	0.073757	0.09568	0.101328	0.18513	0.092286687
Nucleosides and Nucleotides	Purines	Purine conversions	Deoxyadenosine kinase (EC 2.7.1.76)	0.003133	0.02262	0.007664	0.01992	0.007879	0.010228	0.019496	0.012459	0.008539	0.057273	0.000233	0.014986727
Nucleosides and Nucleotides	Pyrimidines	pyrimidine conversions	Deoxycytidine triphosphate deaminase (EC 3.5.4.13)	0.097832	0.040716	0.030658	0.044821	0.03808	0.028735	0.050546	0.05482	0.049044	0.017622	0.037841	0.023781569
Nucleosides and Nucleotides	Purines	Purine conversions	Deoxyguanosine kinase (EC 2.7.1.113)	0.005919	0.02262	0.00511	0.01494	0.009848	0.00828	0.006499	0.020931	0.007663	0.044056	0.000815	0.019285551
Secondary Metabolism	Plant Alkaloids	Alkaloid biosynthesis from L-lysine	Deoxyhypusine synthase	0.018104	0.03393	0.025548	0.044821	0.018384	0.021917	0.01733	0.022925	0.03328	0.057273	0.004424	0.036954113
Clustering-based subsystems	NULL	EC699-706	Deoxyribodipyrimidine photolyase (EC 4.1.99.3)	0.041083	0.052026	0.048541	0.074701	0.041363	0.049191	0.066432	0.081233	0.057364	0.030839	0.035396	0.049061811
DNA Metabolism	DNA repair	DNA repair, bacterial photolyase	Deoxyribodipyrimidine photolyase, type II (EC 4.1.99.3)	0.010445	0.04524	0.030658	0.069721	0.05909	0.06916	0.036104	0.03339	0.029339	0.013217	0.000931	0.023268865
Carbohydrates	Monosaccharides	Deoxyribose and Deoxynucleoside Catabolism	Deoxyribose-phosphate aldolase (EC 4.1.2.4)	0.03586	0.049764	0.020438	0.044821	0.022979	0.028248	0.025273	0.02741	0.042038	0.048461	0.008034	0.026581721
Phages, Prophages, Transposable elements, Plasmids	Phages, Prophages	r1t-like streptococcal phages	Deoxyuridine 5'-triphosphate nucleotidohydrolase (EC 3.6.1.23)	0.047001	0.038454	0.028103	0.062251	0.041363	0.060393	0.047658	0.059305	0.021676	0.033042	0.006637	0.02914524
Cofactors, Vitamins, Prosthetic Groups, Pigments	Coenzyme A	Coenzyme A Biosynthesis	Dephospho-CoA kinase (EC 2.7.1.24)	0.028549	0.013572	0.015329	0.00498	0.013131	0.019969	0.016608	0.025416	0.019924	0.024231	0.022006	0.019443306
Cofactors, Vitamins, Prosthetic Groups, Pigments	Biotin	Biotin biosynthesis	Dethiobiotin synthetase (EC 6.3.3.3)	0.004874	0.015834	0.012774	0.00747	0.013131	0.011202	0.023107	0.013954	0.013356	0.006608	0.037375	0.015854379
Carbohydrates	Organic acids	Glycerate metabolism	D-glycerate 2-kinase (EC 2.7.1.-)	0.034468	0.015834	0.038322	0.02988	0.04005	0.042372	0.027439	0.022925	0.024084	0.013217	0.001048	0.0164854
Cell Wall and Capsule	Gram-Negative cell wall components	LOS core oligosaccharide biosynthesis	D-glycero-beta-D-manno-heptose 7-phosphate kinase	0.034468	0.029406	0.028103	0.042331	0.030858	0.035554	0.033938	0.035384	0.014232	0.033042	0.028293	0.027212741
Cell Wall and Capsule	Capsular and extracellular polysacchrides	Capsular heptose biosynthesis	D-glycero-D-manno-heptose 1,7-bisphosphate phosphatase (EC 3.1.1.-)	0.001044	0.004524	0.012774	0.00747	0.005252	0.005357	0.005777	0.005482	0.004817	0.006608	0.00978	0.009780811
Membrane Transport	Uni- Sym- and Antiporters	Proton-dependent Peptide Transporters	Di/tripeptide permease DtpA	0.001393	0.002262	0.002555	0.00249	0.000657	0.000974	0.002166	0.000997	0.002189	0.002203	0.000116	0.002602958
Membrane Transport	Uni- Sym- and Antiporters	Proton-dependent Peptide Transporters	Di-/tripeptide transporter	0.016363	0.018096	0.038322	0.034861	0.010505	0.01802	0.024551	0.037875	0.019705	0.022028	0.000582	0.037861205
Fatty Acids, Lipids, and Isoprenoids	Phospholipids	Glycerolipid and Glycerophospholipid Metabolism in Bacteria	Diacylglycerol kinase (EC 2.7.1.107)	0.010793	0.002262	0.010219	0.00996	0.008535	0.010715	0.007943	0.005482	0.006787	0.002203	0.00489	0.015262798
Stress Response	Osmotic stress	Ectoine biosynthesis and regulation	Diaminobutyrate-pyruvate aminotransferase (EC 2.6.1.46)	0.006963	0.002262	0.00511	0.00747	0.024293	0.01802	0.077985	0.049836	0.029996	0.01542	0.092332	0.025319681
Amino Acids and Derivatives	Lysine, threonine, methionine, and cysteine	Lysine Biosynthesis DAP Pathway	Diaminopimelate decarboxylase (EC 4.1.1.20)	0.089825	0.081431	0.081754	0.094622	0.084039	0.089128	0.081596	0.080734	0.085827	0.03965	0.088257	0.095520665
Amino Acids and Derivatives	Lysine, threonine, methionine, and cysteine	Lysine Biosynthesis DAP Pathway	Diaminopimelate epimerase (EC 5.1.1.7)	0.037949	0.058812	0.048541	0.057271	0.043333	0.049191	0.044047	0.050833	0.032404	0.061678	0.039238	0.052414105
Clustering-based subsystems	Chemotaxis, response regulators	CBSS-323850.3.peg.3142	diguanylate cyclase (GGDEF domain) with PAS/PAC sensor	0.004874	0.002262	0.002555	0.00996	0.02101	0.016559	0.014442	0.007475	0.034594	0.006608	0.059614	0.068781189
Stress Response	NULL	Bacterial hemoglobins	diguanylate cyclase/phosphodiesterase (GGDEF & EAL domains) with PAS/PAC sensor(s)	0.038994	0.03393	0.038322	0.057271	0.057777	0.056984	0.054156	0.042361	0.111444	0.033042	0.16487	0.184376179
Clustering-based subsystems	Clustering-based subsystems	CBSS-280355.3.peg.2835	Dihydrodipicolinate reductase (EC 1.3.1.26)	0.033075	0.036192	0.061315	0.0249	0.04202	0.033119	0.035382	0.034387	0.034375	0.03965	0.066134	0.046458853
Amino Acids and Derivatives	Lysine, threonine, methionine, and cysteine	Lysine Biosynthesis DAP Pathway	Dihydrodipicolinate synthase (EC 4.2.1.52)	0.050135	0.061074	0.051096	0.079681	0.068282	0.061367	0.064988	0.078741	0.06021	0.13437	0.091866	0.068150169
Clustering-based subsystems	NULL	EC49-61	Dihydrofolate reductase (EC 1.5.1.3)	0.092262	0.006786	0.007664	0.02739	0.017727	0.01315	0.030328	0.0304	0.023427	0.004406	0.021075	0.024609783
Cofactors, Vitamins, Prosthetic Groups, Pigments	Folate and pterines	Folate Biosynthesis	Dihydrofolate synthase (EC 6.3.2.12)	0.017756	0.031668	0.022993	0.0249	0.023636	0.017533	0.025995	0.023921	0.023208	0.026433	0.0085	0.024333712
Carbohydrates	Central carbohydrate metabolism	Dehydrogenase complexes	Dihydrolipoamide acetyltransferase component (E2) of acetoin dehydrogenase complex (EC 2.3.1.-)	0.007311	0.02262	0.010219	0.00249	0.005252	0.007793	0.005777	0.005482	0.007225	0.006608	0.000466	0.006546833
Carbohydrates	Central carbohydrate metabolism	Dehydrogenase complexes	Dihydrolipoamide acetyltransferase component of pyruvate dehydrogenase complex (EC 2.3.1.12)	0.058839	0.052026	0.05876	0.059761	0.061716	0.068186	0.067154	0.090203	0.111882	0.061678	0.077196	0.073277207
Amino Acids and Derivatives	Branched-chain amino acids	Isoleucine degradation	Dihydrolipoamide acyltransferase component of branched-chain alpha-keto acid dehydrogenase complex (EC 2.3.1.168)	0.034119	0.042978	0.043432	0.037351	0.011818	0.025813	0.035382	0.047843	0.073128	0.01542	0.044827	0.04306712
Amino Acids and Derivatives	Alanine, serine, and glycine	Glycine cleavage system	Dihydrolipoamide dehydrogenase (EC 1.8.1.4)	0.027156	0.054288	0.061315	0.034861	0.056464	0.0789	0.052712	0.050833	0.044008	0.160804	0.025732	0.05734395
Carbohydrates	Central carbohydrate metabolism	Dehydrogenase complexes	Dihydrolipoamide dehydrogenase of 2-oxoglutarate dehydrogenase (EC 1.8.1.4)	0.099921	0.036192	0.033212	0.02739	0.043333	0.057471	0.064265	0.06678	0.057583	0.028636	0.083949	0.055569206
Amino Acids and Derivatives	Branched-chain amino acids	Isoleucine degradation	Dihydrolipoamide dehydrogenase of branched-chain alpha-keto acid dehydrogenase (EC 1.8.1.4)	0.021934	0.009048	0.015329	0.01245	0.015757	0.011689	0.018052	0.015449	0.01445	0.011014	0.000582	0.017786878
Carbohydrates	Central carbohydrate metabolism	Pyruvate metabolism II: acetyl-CoA, acetogenesis from pyruvate	Dihydrolipoamide dehydrogenase of pyruvate dehydrogenase complex (EC 1.8.1.4)	0.033423	0.027144	0.06898	0.044821	0.035454	0.050165	0.057767	0.060302	0.058021	0.013217	0.031088	0.032379218
Carbohydrates	Central carbohydrate metabolism	Dehydrogenase complexes	Dihydrolipoamide succinyltransferase component (E2) of 2-oxoglutarate dehydrogenase complex (EC 2.3.1.61)	0.087736	0.05655	0.071534	0.062251	0.047272	0.066237	0.094593	0.096682	0.087141	0.123356	0.097572	0.070437616
Cofactors, Vitamins, Prosthetic Groups, Pigments	Folate and pterines	Folate Biosynthesis	Dihydroneopterin aldolase (EC 4.1.2.25)	0.021238	0.027144	0.002555	0.00747	0.010505	0.004383	0.005055	0.015449	0.012261	0.013217	0.012226	0.016406522
Regulation and Cell signaling	NULL	Zinc regulated enzymes	Dihydroorotase (EC 3.5.2.3)	0.093306	0.122147	0.145624	0.104582	0.103736	0.107149	0.094593	0.121102	0.112101	0.154195	0.144611	0.131015544
Clustering-based subsystems	Hypothetical Related to Dihydroorate Dehydrogenase	Hypothetical Related to Dihydroorotate dehydrogenase	Dihydroorotate dehydrogenase (EC 1.3.3.1)	0.038646	0.020358	0.045986	0.01992	0.035454	0.035554	0.046935	0.057311	0.055175	0.041853	0.048786	0.053991656
Nucleosides and Nucleotides	Pyrimidines	De Novo Pyrimidine Synthesis	Dihydroorotate dehydrogenase electron transfer subunit (EC 1.3.3.1)	0.008008	0.015834	0.017884	0.01494	0.015757	0.022404	0.004333	0.007475	0.004379	0.004406	0.000116	0.01171331
Miscellaneous	Plant-Prokaryote DOE project	Experimental-PTPS	Dihydropteroate synthase (EC 2.5.1.15)	0.032727	0.042978	0.045986	0.037351	0.056464	0.04286	0.036826	0.040367	0.047074	0.030839	0.030273	0.057107317
Nucleosides and Nucleotides	Pyrimidines	Novel non-oxidative pathway of Uracil catabolism	Dihydropyrimidinase (EC 3.5.2.2)	0.011837	0.013572	0.010219	0.01743	0.016414	0.022404	0.010831	0.014452	0.02277	0.019825	0.002329	0.011318923
Amino Acids and Derivatives	Branched-chain amino acids	Branched-Chain Amino Acid Biosynthesis	Dihydroxy-acid dehydratase (EC 4.2.1.9)	0.238488	0.176435	0.204384	0.179283	0.191058	0.199199	0.198573	0.145521	0.172968	0.26874	0.194561	0.185795975
Clustering-based subsystems	NULL	EC49-61	Dimethyladenosine transferase (EC 2.1.1.-)	0.021238	0.024882	0.022993	0.039841	0.033484	0.032145	0.033216	0.050833	0.037659	0.028636	0.037375	0.045867272
Fatty Acids, Lipids, and Isoprenoids	Isoprenoids	Archaeal lipids	Dimethylallyltransferase (EC 2.5.1.1)	0.064061	0.009048	0.015329	0.00996	0.015757	0.024839	0.01372	0.020931	0.028244	0.035245	0.02154	0.02184907
DNA Metabolism	DNA repair	DNA repair, bacterial DinG and relatives	DinG family ATP-dependent helicase YoaA	0.044912	0.05655	0.051096	0.074701	0.046615	0.04773	0.054156	0.062295	0.047511	0.006608	0.0248	0.037861205
Membrane Transport	ABC transporters	ABC transporter dipeptide (TC 3.A.1.5.2)	Dipeptide transport ATP-binding protein DppD (TC 3.A.1.5.2)	0.012882	0.027144	0.00511	0.032371	0.025606	0.018508	0.020218	0.020433	0.03109	0.006608	0.014321	0.036875236
Membrane Transport	ABC transporters	ABC transporter dipeptide (TC 3.A.1.5.2)	Dipeptide transport ATP-binding protein DppF (TC 3.A.1.5.2)	0.062668	0.024882	0.028103	0.01494	0.042676	0.043347	0.033938	0.014951	0.033937	0.057273	0.004075	0.02882973
Membrane Transport	ABC transporters	ABC transporter dipeptide (TC 3.A.1.5.2)	Dipeptide transport system permease protein DppB (TC 3.A.1.5.2)	0.040734	0.061074	0.043432	0.059761	0.043989	0.059906	0.046213	0.031895	0.073785	0.024231	0.014787	0.049337882
Membrane Transport	ABC transporters	ABC transporter dipeptide (TC 3.A.1.5.2)	Dipeptide transport system permease protein DppC (TC 3.A.1.5.2)	0.049787	0.070121	0.051096	0.02739	0.068938	0.085719	0.054878	0.047344	0.07269	0.033042	0.004424	0.061563897
Membrane Transport	ABC transporters	ABC transporter dipeptide (TC 3.A.1.5.2)	Dipeptide-binding ABC transporter, periplasmic	0.039342	0.052026	0.045986	0.052291	0.069595	0.054548	0.042603	0.047843	0.079478	0.026433	0.034232	0.067045883

			substrate-binding component (TC 3.A.1.5.2)												
Protein Metabolism	Protein degradation	Protein degradation	Dipeptidyl carboxypeptidase Dcp (EC 3.4.15.5)	0.017756	0.027144	0.022993	0.032371	0.020353	0.027274	0.032494	0.065784	0.053204	0.127762	0.061011	0.072488432
Carbohydrates	Fermentation	Fermentations: Lactate	D-lactate dehydrogenase (EC 1.1.1.28)	0.007311	0.006786	0.002555	0.00498	0.019697	0.017533	0.01372	0.022426	0.012261	0.035245	0.009897	0.017313613
Clustering-based subsystems	Choline bitartrate degradation, putative	CBSS-344610.3.peg.2335	D-malic enzyme (EC 1.1.1.83)	0.00383	0.009048	0.048541	0.01743	0.007879	0.009254	0.010831	0.010466	0.011823	0.013217	0.000233	0.005048161
Carbohydrates	Monosaccharides	D-Galacturonate and D-Glucuronate Utilization	D-mannonate oxidoreductase (EC 1.1.1.57)	0.008356	0.004524	0.00511	0.01992	0.008535	0.014611	0.008665	0.011961	0.011823	0.002203	0.000815	0.011949943
Clustering-based subsystems	NULL	DNA gyrase subunits	DNA gyrase subunit A (EC 5.99.1.3)	0.262859	0.30763	0.217158	0.249004	0.266562	0.26495	0.25995	0.258649	0.216976	0.392096	0.233916	0.275874091
Clustering-based subsystems	NULL	DNA gyrase subunits	DNA gyrase subunit B (EC 5.99.1.3)	0.145182	0.28501	0.235042	0.201693	0.263935	0.236701	0.265005	0.190872	0.170997	0.224684	0.192232	0.194038674
Phages, Prophages, Transposable elements, Plasmids	Phages, Prophages	Phage replication	DNA helicase (EC 3.6.1.-)	0.136478	0.052026	0.056206	0.062251	0.057777	0.062341	0.072208	0.089705	0.053423	0.081503	0.044827	0.048075842
Cofactors, Vitamins, Prosthetic Groups, Pigments	NAD and NADP	NAD consumption	DNA ligase (EC 6.5.1.2)	0.09261	0.147029	0.222268	0.107072	0.173331	0.125169	0.172578	0.131567	0.141878	0.224684	0.149035	0.154599919
DNA Metabolism	DNA repair	DNA repair, bacterial MutL-MutS system	DNA mismatch repair protein MutL	0.129167	0.074645	0.091973	0.082171	0.100453	0.099843	0.093871	0.120603	0.095023	0.019825	0.067997	0.108101627
DNA Metabolism	DNA repair	DNA repair, bacterial MutL-MutS system	DNA mismatch repair protein MutS	0.135085	0.226198	0.13285	0.164343	0.211411	0.163645	0.180521	0.194361	0.153263	0.240104	0.162658	0.196365561
DNA Metabolism	DNA repair	DNA Repair Base Excision	DNA polymerase I (EC 2.7.7.7)	0.231525	0.174173	0.206939	0.201693	0.211411	0.233292	0.234677	0.224761	0.209751	0.09472	0.27001	0.214704582
Clustering-based subsystems	NULL	EC49-61	DNA polymerase II (EC 2.7.7.7)	0.011489	0.002262	0.007664	0.01494	0.011161	0.012663	0.020218	0.024918	0.012918	0.006608	0.035862	0.006152446
Clustering-based subsystems	NULL	CBSS-350688.3.peg.1509	DNA polymerase III alpha subunit (EC 2.7.7.7)	0.285489	0.477278	0.38322	0.361056	0.439236	0.376969	0.469355	0.518295	0.402424	0.447166	0.212841	0.374904805
Clustering-based subsystems	NULL	DNA replication cluster 1	DNA polymerase III beta subunit (EC 2.7.7.7)	0.065802	0.047502	0.048541	0.032371	0.063686	0.071595	0.056323	0.084721	0.066122	0.083706	0.117365	0.07635343
DNA Metabolism	DNA replication	DNA-replication	DNA polymerase III delta prime subunit (EC 2.7.7.7)	0.010793	0.01131	0.015329	0.0249	0.01707	0.015098	0.012275	0.026413	0.021676	0.002203	0.004424	0.026542282
Miscellaneous	Plant-Prokaryote DOE project	At4g10620 At3g57180 At3g47450	DNA polymerase III delta subunit (EC 2.7.7.7)	0.006267	0.013572	0.010219	0.0249	0.01707	0.012663	0.012275	0.014951	0.021895	0.046259	0.010363	0.026976108
Clustering-based subsystems	DNA polymerase III epsilon cluster	CBSS-342610.3.peg.1536	DNA polymerase III epsilon subunit (EC 2.7.7.7)	0.040386	0.03393	0.022993	0.02988	0.025606	0.030196	0.038993	0.054321	0.04773	0.044056	0.053327	0.04626166
DNA Metabolism	DNA uptake, competence	DNA processing cluster	DNA polymerase III subunits gamma and tau (EC 2.7.7.7)	0.098181	0.052026	0.071534	0.069721	0.089292	0.077926	0.087372	0.090203	0.063057	0.116748	0.052512	0.077063327
DNA Metabolism	DNA repair	DNA repair, bacterial	DNA polymerase IV (EC 2.7.7.7)	0.041431	0.067859	0.056206	0.057271	0.061716	0.061854	0.044769	0.076748	0.065246	0.059475	0.033882	0.068229046
DNA Metabolism	DNA replication	DNA-replication	DNA primase (EC 2.7.7.-)	0.076595	0.119885	0.074089	0.102092	0.128685	0.113967	0.109035	0.104656	0.088017	0.167412	0.383999	0.099030714
Clustering-based subsystems	NULL	DNA replication cluster 1	DNA recombination and repair protein RecF	0.025416	0.013572	0.020438	0.01992	0.026262	0.023378	0.028161	0.035882	0.031528	0.13437	0.025615	0.035692073
Clustering-based subsystems	Probably GTP or GMP signaling related	CBSS-176299.4.peg.1292	DNA recombination and repair protein RecO	0.042475	0.01131	0.017884	0.0249	0.009848	0.009254	0.008665	0.014452	0.013137	0.024231	0.001747	0.013014789
DNA Metabolism	DNA repair	DNA repair, bacterial	DNA recombination protein RmuC	0.047001	0.054288	0.040877	0.02739	0.041363	0.040911	0.029605	0.054321	0.056707	0.048461	0.05845	0.056673491
DNA Metabolism	DNA repair	DNA repair, bacterial	DNA repair protein RadA	0.097832	0.097265	0.081754	0.059761	0.070908	0.080849	0.092427	0.0912	0.073566	0.09472	0.093729	0.101633672
DNA Metabolism	DNA repair	DNA repair, bacterial	DNA repair protein RadC	0.030638	0.049764	0.045986	0.042331	0.033484	0.057471	0.061377	0.053823	0.04576	0.059475	0.039937	0.053478952
DNA Metabolism	DNA repair	DNA repair, bacterial	DNA repair protein RecN	0.069283	0.058812	0.056206	0.069721	0.058433	0.061367	0.05199	0.077246	0.058459	0.037447	0.018164	0.068583995
DNA Metabolism	DNA replication	DNA replication, archaeal	DNA replication helicase protein MCM	0.004178	0.01131	0.00511	0.00249	0.011161	0.008767	0.010831	0.012957	0.003065	0.011014	0.000233	0.005758058
Clustering-based subsystems	NULL	Conserved gene cluster associated with Met-tRNA formyltransferase	DNA topoisomerase I (EC 5.99.1.2)	0.203324	0.201316	0.240151	0.184263	0.172018	0.20407	0.222402	0.255659	0.20778	0.207062	0.255922	0.205436475
DNA Metabolism	DNA replication	DNA topoisomerases, Type I, ATP-independent	DNA topoisomerase III (EC 5.99.1.2)	0.004178	0.020358	0.010219	0.00498	0.012475	0.008767	0.010831	0.015449	0.019924	0.035245	0.088723	0.039359877
DNA Metabolism	DNA replication	DNA topoisomerases, Type I, ATP-independent	DNA topoisomerase III, Burkholderia type (EC 5.99.1.2)	0.005571	0.006786	0.020438	0.00747	0.00197	0.000974	0.001444	0.003987	0.007444	0.013217	0.000582	0.002800152
DNA Metabolism	DNA replication	DNA topoisomerases, Type I, ATP-independent	DNA topoisomerase V	0.009052	0.03393	0.053651	0.01743	0.025606	0.017533	0.023829	0.010466	0.017297	0.002203	0.000116	0.01265984
DNA Metabolism	DNA repair	DNA Repair Base Excision	DNA-3-methyladenine glycosylase (EC 3.2.2.20)	0.055357	0.015834	0.053651	0.037351	0.04005	0.033119	0.044769	0.043357	0.040724	0.099125	0.042382	0.038847174
DNA Metabolism	DNA repair	DNA Repair Base Excision	DNA-3-methyladenine glycosylase II (EC 3.2.2.21)	0.006963	0.013572	0.015329	0.00747	0.009192	0.003409	0.009387	0.004984	0.015983	0.002203	0.000349	0.005560864
Cofactors, Vitamins, Prosthetic Groups, Pigments	Folate and pterines	Molybdenum cofactor biosynthesis	DNA-binding domain of ModE	0.002785	0.002262	0.002555	0.00747	0.005909	0.002435	0.002888	0.000498	0.001752	0.004406	0.012691	0.005758058
Virulence, Disease and Defense	Resistance to antibiotics and toxic compounds	Cobalt-zinc-cadmium resistance	DNA-binding heavy metal response regulator	0.002089	0.004524	0.007664	0.00249	0.003283	0.003409	0.000722	0.004485	0.01248	0.006608	0.000116	0.009228669
DNA Metabolism	NULL	DNA structural proteins, bacterial	DNA-binding protein HU	0.066498	0.02262	0.030658	0.00747	0.028232	0.023865	0.03105	0.025416	0.020143	0.013217	0.020027	0.036559726
Regulation and Cell signaling	NULL	The Chv regulatory system of Alphaproteobacteria	DNA-binding response regulator ChvI	0.004874	0.002262	0.010219	0.01494	0.004596	0.008767	0.007221	0.005482	0.007882	0.011014	0.000931	0.002997345
DNA Metabolism	DNA repair	DNA repair, bacterial	DNA-cytosine methyltransferase (EC 2.1.1.37)	0.011141	0.009048	0.017884	0.00249	0.05712	0.075004	0.096037	0.084223	0.01664	0.052867	0.00326	0.023899886
RNA Metabolism	Transcription	RNA polymerase bacterial	DNA-directed RNA polymerase alpha subunit (EC 2.7.7.6)	0.142397	0.117623	0.081754	0.102092	0.123432	0.115428	0.129975	0.105154	0.090644	0.209265	0.079874	0.105735302
RNA Metabolism	Transcription	RNA polymerase bacterial	DNA-directed RNA polymerase beta subunit (EC 2.7.7.6)	0.394115	0.407157	0.373001	0.485558	0.464841	0.384274	0.435417	0.388721	0.389068	0.863493	0.434065	0.37060598
RNA Metabolism	Transcription	RNA polymerase bacterial	DNA-directed RNA polymerase beta' subunit (EC 2.7.7.6)	0.526415	0.422991	0.424097	0.547809	0.542972	0.459279	0.440471	0.397692	0.362357	0.874507	0.510679	0.404878258
RNA Metabolism	Transcription	RNA polymerase bacterial	DNA-directed RNA polymerase omega subunit (EC 2.7.7.6)	0.04909	0.009048	0.007664	0.00249	0.01904	0.018508	0.01372	0.024918	0.009853	0.004406	0.001514	0.009741372
Clustering-based subsystems	Putative associate of RNA polymerase sigma-54 factor rpoN	CBSS-316057.3.peg.1308	DNA-directed RNA polymerase specialized sigma subunit, sigma24-like	0.013926	0.004524	0.007664	0.00249	0.000657	0.004383	0.001444	0.00598	0.007225	0.002203	0.000349	0.00283959
RNA Metabolism	Transcription	RNA polymerase archaeal	DNA-directed RNA polymerase subunit A' (EC 2.7.7.6)	0.008704	0.024882	0.007664	0.01743	0.033484	0.026787	0.029605	0.009967	0.00832	0.019825	0.001048	0.012226014
RNA Metabolism	Transcription	RNA polymerase archaeal	DNA-directed RNA polymerase subunit A'' (EC 2.7.7.6)	0.002785	0.009048	0.007664	0.01245	0.004596	0.005844	0.018774	0.003987	0.001314	0.006608	0.001747	0.004535457
RNA Metabolism	Transcription	RNA polymerase archaeal	DNA-directed RNA polymerase subunit B' (EC 2.7.7.6)	0.009748	0.004524	0.002555	0.00249	0.01904	0.007306	0.010831	0.005482	0.000876	0.002203	0.000582	0.005442548
RNA Metabolism	Transcription	RNA polymerase archaeal	DNA-directed RNA polymerase subunit B'' (EC 2.7.7.6)	0.004874	0.002262	0.00511	0.02241	0.011161	0.003409	0.010109	0.003489	0.002846	0.002203	0.000116	0.001774744
Protein Metabolism	Protein folding	Protein chaperones	DnaJ-class molecular chaperone CbpA	0.031334	0.054288	0.038322	0.039841	0.046615	0.04773	0.051268	0.034387	0.036345	0.048461	0.027246	0.046537731
Clustering-based subsystems	NULL	EC49-61	DnaJ-like protein DjlA	0.028201	0.002262	0.002555	0.00249	0.024949	0.012176	0.019496	0.019934	0.025836	0.01542	0.000815	0.024452028
Amino Acids and Derivatives	Arginine; urea cycle, polyamines	Anaerobic Oxidative Degradation of L-Ornithine	D-Ornithine 4,5-aminomutase E subunit (EC 5.4.3.5)	0.004178	0.004524	0.00511	0.00498	0.008535	0.005357	0.005055	0.002492	0.000438	0.006608	0.000349	0.005600303
Cell Wall and Capsule	Capsular and extracellular polysacchrides	dTDP-rhamnose synthesis	dTDP-4-dehydrorhamnose 3,5-epimerase (EC 5.1.3.13)	0.026112	0.029406	0.030658	0.044821	0.03808	0.032632	0.03466	0.038872	0.038316	0.077098	0.078244	0.042633294
Cell Wall and Capsule	Capsular and extracellular polysacchrides	Rhamnose containing glycans	dTDP-4-dehydrorhamnose reductase (EC 1.1.1.133)	0.023675	0.020358	0.035767	0.02988	0.034797	0.040424	0.028161	0.025416	0.023865	0.024231	0.023869	0.032379218
Cell Wall and Capsule	Capsular and extracellular polysacchrides	Rhamnose containing glycans	dTDP-glucose 4,6-dehydratase (EC 4.2.1.46)	0.102707	0.167387	0.178836	0.156873	0.175957	0.160236	0.168968	0.168944	0.140564	0.24451	0.162425	0.165563893
Clustering-based subsystems	D-tyrosyl-tRNA(Tyr) deacylase (EC 3.1.-.-) cluster	CBSS-342610.3.peg.283	D-tyrosyl-tRNA(Tyr) deacylase	0.023327	0.024882	0.017884	0.02739	0.027575	0.024839	0.028883	0.02741	0.026274	0.028636	0.02154	0.033049677
Carbohydrates	Monosaccharides	Xylose utilization	D-xylose proton-symporter XylE	0.06998	0.009048	0.012774	0.01743	0.005909	0.005357	0.002888	0.014452	0.009415	0.059475	0.000466	0.02248009
Carbohydrates	Monosaccharides	Xylose utilization	D-xylose transport ATP-binding protein XylG	0.020193	0.013572	0.015329	0.00996	0.007222	0.007306	0.005055	0.006479	0.01861	0.017622	0.005472	0.011003413

Amino Acids and Derivatives	Branched-chain amino acids	Branched chain amino acid degradation regulons	Electron transfer flavoprotein, alpha subunit	0.085647	0.106313	0.076644	0.092131	0.099796	0.129066	0.087372	0.077246	0.052109	0.072692	0.13483	0.087080771
Amino Acids and Derivatives	Branched-chain amino acids	Branched chain amino acid degradation regulons	Electron transfer flavoprotein, beta subunit	0.054661	0.076907	0.053651	0.067231	0.0801	0.077439	0.075819	0.063292	0.055175	0.116748	0.111776	0.065862721
Respiration	Electron accepting reactions	Anaerobic respiratory reductases	Electron transfer flavoprotein-ubiquinone oxidoreductase (EC 1.5.5.1)	0.046305	0.029406	0.038322	0.057271	0.04202	0.048217	0.059933	0.052328	0.067217	0.17402	0.165452	0.030565035
Respiration	Electron donating reactions	Na(+)-translocating NADH-quinone oxidoreductase and rnf-like group of electron transport complexes	Electron transport complex protein RnfA	0.099225	0.047502	0.038322	0.032371	0.036111	0.053087	0.039715	0.022925	0.015983	0.024231	0.082319	0.049968903
Respiration	Electron donating reactions	Na(+)-translocating NADH-quinone oxidoreductase and rnf-like group of electron transport complexes	Electron transport complex protein RnfB	0.029245	0.029406	0.025548	0.02739	0.030202	0.024839	0.035382	0.020433	0.021457	0.011014	0.039355	0.033799013
Respiration	Electron donating reactions	Na(+)-translocating NADH-quinone oxidoreductase and rnf-like group of electron transport complexes	Electron transport complex protein RnfC	0.047349	0.040716	0.015329	0.037351	0.043333	0.051626	0.036104	0.041862	0.03525	0.019825	0.037608	0.070516494
Respiration	Electron donating reactions	Na(+)-translocating NADH-quinone oxidoreductase and rnf-like group of electron transport complexes	Electron transport complex protein RnfD	0.086691	0.049764	0.051096	0.042331	0.037424	0.036528	0.035382	0.03339	0.022552	0.01542	0.089654	0.050797116
Respiration	Electron donating reactions	Na(+)-translocating NADH-quinone oxidoreductase and rnf-like group of electron transport complexes	Electron transport complex protein RnfE	0.050135	0.049764	0.022993	0.047311	0.038737	0.037989	0.028883	0.026413	0.017516	0.01542	0.120393	0.054583237
Respiration	Electron donating reactions	Na(+)-translocating NADH-quinone oxidoreductase and rnf-like group of electron transport complexes	Electron transport complex protein RnfG	0.006267	0.004524	0.020438	0.01494	0.013788	0.013637	0.004333	0.00897	0.007006	0.004406	0.022239	0.017550246
Miscellaneous	Plant-Prokaryote DOE project	COG3533	Endo-1,4-beta-xylanase A precursor (EC 3.2.1.8)	0.032727	0.099527	0.028103	0.039841	0.038737	0.023865	0.051268	0.029403	0.048825	0.002203	0.005356	0.038137276
Cell Division and Cell Cycle	NULL	Control of cell elongation - division cycle in Bacilli	Endonuclease III (EC 4.2.99.18)	0.092262	0.067859	0.061315	0.057271	0.068938	0.05601	0.06932	0.071266	0.047511	0.061678	0.051231	0.063811906
DNA Metabolism	DNA repair	DNA repair, bacterial	Endonuclease IV (EC 3.1.21.2)	0.019497	0.02262	0.007664	0.01992	0.030858	0.023378	0.023107	0.024918	0.009196	0.008811	0.018979	0.027962077
DNA Metabolism	DNA repair	DNA repair, bacterial	Endonuclease V (EC 3.1.21.7)	0.005919	0.031668	0.010219	0.00996	0.014444	0.014124	0.01372	0.007475	0.007882	0.011014	0.000466	0.008045506
Miscellaneous	Plant-Prokaryote DOE project	Iojap	Endoribonuclease L-PSP	0.032727	0.027144	0.051096	0.0249	0.026919	0.019969	0.027439	0.029403	0.035907	0.063881	0.040519	0.03738794
Carbohydrates	Central carbohydrate metabolism	Glycolysis and Gluconeogenesis	Enolase (EC 4.2.1.11)	0.195665	0.176435	0.237596	0.169323	0.159543	0.146599	0.159581	0.150006	0.131587	0.158601	0.247538	0.142886609
Fatty Acids, Lipids, and Isoprenoids	Fatty acids	Fatty Acid Biosynthesis FASII	Enoyl-[acyl-carrier-protein] reductase [FMN] (EC 1.3.1.9)	0.038994	0.05655	0.056206	0.042331	0.048585	0.057471	0.044047	0.037875	0.028463	0.044056	0.037259	0.054977624
Fatty Acids, Lipids, and Isoprenoids	Fatty acids	Polyunsaturated Fatty Acids synthesis	Enoyl-[acyl-carrier-protein] reductase [FMN] (EC 1.3.1.9), inferred for PFA pathway	0.009748	0.002262	0.007664	0.00498	0.013131	0.010228	0.010109	0.007974	0.003284	0.002203	0.000815	0.007966629
Fatty Acids, Lipids, and Isoprenoids	Fatty acids	Fatty Acid Biosynthesis FASII	Enoyl-[acyl-carrier-protein] reductase [NADH] (EC 1.3.1.9)	0.050135	0.020358	0.040877	0.042331	0.042676	0.041398	0.050546	0.062793	0.072252	0.103531	0.017698	0.060538489
Clustering-based subsystems	NULL	Butyrate metabolism cluster	Enoyl-CoA hydratase (EC 4.2.1.17)	0.133344	0.117623	0.150733	0.099602	0.105049	0.118838	0.129253	0.162964	0.161145	0.154195	0.213772	0.104276068
Amino Acids and Derivatives	Branched-chain amino acids	Isoleucine degradation	Enoyl-CoA hydratase [isoleucine degradation] (EC 4.2.1.17)	0.012882	0.031668	0.020438	0.00996	0.036767	0.040911	0.031772	0.031895	0.027806	0.041853	0.015835	0.025398558
Amino Acids and Derivatives	Branched-chain amino acids	Branched chain amino acid degradation regulons	Enoyl-CoA hydratase [valine degradation] (EC 4.2.1.17)	0.002437	0.002262	0.002555	0.00747	0.008535	0.005844	0.014442	0.010466	0.014013	0.008811	0.022821	0.011161168
Carbohydrates	Sugar alcohols	Inositol catabolism	Epi-inositol hydrolase (EC 3.7.1.-)	0.010793	0.006786	0.002555	0.00996	0.004596	0.009254	0.002166	0.010466	0.017078	0.008811	0.00163	0.006546833
Carbohydrates	Sugar alcohols	Ethanolamine utilization	Ethanolamine utilization protein similar to PduA/PduJ	0.034119	0.006786	0.017884	0.00498	0.00197	0.005357	0.005055	0.00299	0.003722	0.008811	0.000582	0.002760713
Carbohydrates	One-carbon Metabolism	Serine-glyoxylate cycle	ethylmalonyl-CoA mutase	0.114544	0.115361	0.097082	0.079681	0.087322	0.089128	0.067876	0.078243	0.075537	0.046259	0.010712	0.088816076
Carbohydrates	Central carbohydrate metabolism	Ethylmalonyl-CoA pathway of C2 assimilation	Ethylmalonyl-CoA mutase, methylsuccinyl-CoA-forming	0.002437	0.013572	0.022993	0.00498	0.011161	0.010228	0.012998	0.02442	0.026712	0.026433	0.001514	0.006310201
Protein Metabolism	Protein biosynthesis	Translation initiation factors eukaryotic and archaeal	Eukaryotic translation initiation factor 2 gamma subunit	0.007311	0.004524	0.002555	0.00747	0.011161	0.013637	0.022385	0.004485	0.002189	0.01542	0.000466	0.007059537
DNA Metabolism	DNA repair	DNA repair, UvrABC system	Excinuclease ABC subunit A	0.477324	0.531566	0.470083	0.512948	0.422822	0.425186	0.426752	0.345364	0.340681	0.392096	0.396108	0.37474705
DNA Metabolism	DNA repair	DNA repair, UvrABC system	Excinuclease ABC subunit A, dimeric form	0.022282	0.009048	0.017884	0.01992	0.023636	0.019969	0.022385	0.015948	0.017735	0.004406	0.000582	0.016209328
DNA Metabolism	DNA repair	DNA repair, UvrABC system	Excinuclease ABC subunit B	0.230132	0.23977	0.235042	0.234064	0.245552	0.215272	0.231067	0.226754	0.202526	0.508844	0.237059	0.218332947
Regulation and Cell signaling	Regulation of virulence	BarA-UvrY(SirA) two-component regulatory system	Excinuclease ABC subunit C	0.098877	0.081431	0.143069	0.131972	0.114241	0.099843	0.116256	0.133561	0.12896	0.154195	0.108982	0.129201361
DNA Metabolism	DNA repair	DNA Repair Base Excision	Exodeoxyribonuclease I (EC 3.1.11.1)	0.012882	0.002262	0.010219	0.01494	0.013131	0.015585	0.010109	0.013954	0.019048	0.002203	0.04145	0.016564277
DNA Metabolism	DNA repair	DNA repair, bacterial	Exodeoxyribonuclease III (EC 3.1.11.2)	0.058142	0.083693	0.06898	0.074701	0.064999	0.06916	0.080151	0.075252	0.062619	0.030839	0.064039	0.069293892
DNA Metabolism	DNA repair	DNA repair, bacterial RecBCD pathway	Exodeoxyribonuclease V alpha chain (EC 3.1.11.5)	0.010097	0.015834	0.012774	0.00996	0.022979	0.01802	0.01372	0.014452	0.022552	0.002203	0.01176	0.015144482
DNA Metabolism	DNA replication	DNA-replication	Exodeoxyribonuclease V beta chain (EC 3.1.11.5)	0.016015	0.029406	0.00511	0.02988	0.041363	0.039937	0.03105	0.021429	0.042038	0.024231	0.057868	0.024412589
DNA Metabolism	DNA repair	DNA repair, bacterial RecBCD pathway	Exodeoxyribonuclease V gamma chain (EC 3.1.11.5)	0.008356	0.015834	0.012774	0.01245	0.023636	0.017533	0.01733	0.016944	0.031309	0.01542	0.038307	0.015814941
DNA Metabolism	DNA repair	DNA repair, bacterial	Exodeoxyribonuclease VII large subunit (EC 3.1.11.6)	0.044564	0.054288	0.06387	0.064741	0.068282	0.056984	0.057045	0.06977	0.063276	0.026433	0.038423	0.052138034
DNA Metabolism	DNA repair	DNA repair, bacterial	Exodeoxyribonuclease VII small subunit (EC 3.1.11.6)	0.008356	0.004524	0.010219	0.00249	0.011818	0.00828	0.006499	0.012957	0.01445	0.004406	0.005822	0.006191885
DNA Metabolism	DNA repair	DNA repair, bacterial	Exonuclease SbcC	0.01323	0.027144	0.022993	0.037351	0.035454	0.020456	0.025273	0.012957	0.034594	0.004406	0.087908	0.029066362
DNA Metabolism	DNA repair	DNA repair, bacterial	Exonuclease SbcD	0.005571	0.020358	0.010219	0.01494	0.009192	0.006332	0.009387	0.007475	0.015764	0.004406	0.055655	0.015578308
Phosphorus Metabolism	NULL	Phosphate metabolism	Exopolyphosphatase (EC 3.6.1.11)	0.021934	0.024882	0.05876	0.01992	0.03808	0.031658	0.031772	0.050334	0.052109	0.083706	0.025615	0.048627985
Carbohydrates	One-carbon Metabolism	Methanogenesis	F420-dependent N(5),N(10)-methylenetetrahydromethanopterin reductase (EC 1.5.99.11)	0.009052	0.01131	0.007664	0.00747	0.011818	0.004383	0.007943	0.007475	0.007225	0.002203	0.000582	0.002642397
Sulfur Metabolism	Inorganic sulfur assimilation	Inorganic Sulfur Assimilation	Ferredoxin	0.315431	0.190007	0.176281	0.224104	0.23833	0.227448	0.202906	0.168446	0.186762	0.24451	0.119578	0.200782702
Nitrogen Metabolism	NULL	Ammonia assimilation	Ferredoxin-dependent glutamate synthase (EC 1.4.7.1)	0.016015	0.01131	0.015329	0.00747	0.009848	0.018995	0.024551	0.028407	0.044446	0.05507	0.052162	0.024215396
Iron acquisition and metabolism	NULL	Transport of Iron	Ferric iron ABC transporter, ATP-binding protein	0.018801	0.006786	0.015329	0.02739	0.026262	0.01802	0.018774	0.024918	0.026712	0.022028	0.026663	0.020468714
Iron acquisition and metabolism	NULL	Campylobacter Iron Metabolism	Ferric iron ABC transporter, iron-binding protein	0.004178	0.027144	0.017884	0.02739	0.034797	0.043834	0.032494	0.043357	0.037221	0.017622	0.052861	0.021651876
Iron acquisition and metabolism	NULL	Transport of Iron	Ferric iron ABC transporter, permease protein	0.011837	0.027144	0.007664	0.0249	0.029545	0.042372	0.03827	0.041862	0.051671	0.006608	0.049601	0.020863101
Membrane Transport	NULL	Ton and Tol transport systems	Ferric siderophore transport system, biopolymer transport protein ExbB	0.023327	0.013572	0.043432	0.02739	0.034797	0.032632	0.035382	0.040866	0.040724	0.019825	0.001397	0.023899886
Iron acquisition and metabolism	NULL	Hemin transport system	Ferric siderophore transport system, periplasmic binding protein TonB	0.008008	0.01131	0.002555	0.00747	0.009848	0.012663	0.014442	0.027908	0.025179	0.002203	0.002096	0.01297535
Iron acquisition and metabolism	NULL	Transport of Iron	Ferric uptake regulation protein FUR	0.019497	0.015834	0.017884	0.02739	0.011161	0.020943	0.02094	0.02741	0.018173	0.022028	0.033999	0.022361774
Miscellaneous	Plant-Prokaryote DOE project	At4g38090	Ferrochelatase, protoheme ferro-lyase (EC 4.99.1.1)	0.049787	0.058812	0.079199	0.032371	0.03808	0.050165	0.052712	0.067279	0.062181	0.024231	0.032485	0.063890783
Clustering-based subsystems	NULL	CBSS-196620.1.peg.2477	Ferrous iron transport protein B	0.109322	0.180959	0.12263	0.151892	0.129341	0.149034	0.096759	0.08522	0.082762	0.191643	0.037142	0.151287064
RNA Metabolism	Transcription	Transcription factors bacterial	FIG000325: clustered with transcription termination protein NusA	0.019149	0.004524	0.015329	0.00996	0.019697	0.021917	0.015886	0.022925	0.021676	0.008811	0.027478	0.023860447
Clustering-based subsystems	NULL	Cluster containing CofD-like protein and co-occuring with DNA repair	FIG000506: Predicted P-loop-containing kinase	0.055357	0.018096	0.030658	0.01992	0.034141	0.031658	0.044769	0.020931	0.034375	0.006608	0.027129	0.02121805

DNA Metabolism	DNA uptake, competence	DNA processing cluster	FIG000557: hypothetical protein co-occurring with RecR	0.019149	0.031668	0.020438	0.032371	0.02101	0.025326	0.028883	0.017443	0.023427	0.002203	0.01176	0.017313613
Clustering-based subsystems	NULL	Cluster co-expressed with butyrate metabolism cluster	FIG000605: protein co-occurring with transport systems (COG1739)	0.003133	0.009048	0.00511	0.00249	0.005909	0.005357	0.00361	0.010466	0.006131	0.006608	0.01991	0.013882442
DNA Metabolism	DNA recombination	RuvABC plus a hypothetical	FIG000859: hypothetical protein YebC	0.060231	0.070121	0.081754	0.102092	0.072221	0.070621	0.078707	0.099174	0.069625	0.041853	0.066717	0.073789911
Miscellaneous	Plant-Prokaryote DOE project	YggW	FIG001341: Probable Fe(2+)-trafficking protein YggX	0.004178	0.002262	0.010219	0.01245	0.003939	0.003896	0.005777	0.003489	0.010072	0.004406	0.01176	0.00698066
Miscellaneous	Plant-Prokaryote DOE project	COG3380 COG2907	FIG001571: Hypothetical protein	0.002785	0.004524	0.010219	0.01494	0.014444	0.020943	0.016608	0.018439	0.01664	0.004406	0.000349	0.008400455
Miscellaneous	NULL	Broadly distributed proteins not in subsystems	FIG001943: hypothetical protein YajQ	0.008704	0.009048	0.022993	0.01245	0.007879	0.006819	0.012998	0.011462	0.011604	0.008811	0.008616	0.015775502
Clustering-based subsystems	Two related proteases	CBSS-257314.1.peg.676	FIG002344: Hydrolase (HAD superfamily)	0.025416	0.042978	0.020438	0.049801	0.049898	0.035554	0.042603	0.013456	0.014669	0.013217	0.001048	0.030210086
Clustering-based subsystems	TldD cluster	CBSS-354.1.peg.2917	FIG003879: Predicted amidohydrolase	0.015319	0.009048	0.020438	0.00498	0.017727	0.009254	0.007943	0.009967	0.01861	0.008811	0.002329	0.012935912
Clustering-based subsystems	NULL	CBSS-281090.3.peg.464	FIG004453: protein YceG like	0.032727	0.031668	0.022993	0.034861	0.034141	0.033119	0.036826	0.03937	0.031309	0.041853	0.035512	0.045117936
Clustering-based subsystems	Two related proteases	CBSS-257314.1.peg.676	FIG006542: Phosphoesterase	0.005919	0.015834	0.002555	0.00747	0.002626	0.011202	0.007221	0.00598	0.007225	0.006608	0.002445	0.007414486
Clustering-based subsystems	NULL	CBSS-350688.3.peg.1509	FIG007959: peptidase, M16 family	0.008356	0.006786	0.010219	0.01494	0.029545	0.014611	0.005055	0.004984	0.010728	0.008811	0.00163	0.012186575
Clustering-based subsystems	alpha-proteobacterial cluster of hypotheticals	CBSS-52598.3.peg.2843	FIG017823: ATPase, MoxR family	0.007311	0.006786	0.002555	0.00747	0.006566	0.007793	0.01372	0.016944	0.025398	0.004406	0.000582	0.010530148
Clustering-based subsystems	NULL	Cell wall related cluster	FIG040338: Glycosyl transferase	0.002437	0.01131	0.007664	0.00747	0.00197	0.006819	0.005777	0.00299	0.005474	0.002203	0.000349	0.004298824
Clustering-based subsystems	NULL	EC699-706	FIG042796: Hypothetical protein	0.005919	0.006786	0.012774	0.01743	0.007879	0.009741	0.010831	0.011462	0.01029	0.022028	0.001281	0.027173302
Clustering-based subsystems	NULL	CBSS-316273.3.peg.2378	FIG045374: Type II restriction enzyme, methylase subunit YeeA	0.005571	0.004524	0.007664	0.00498	0.005252	0.006819	0.005777	0.009967	0.011604	0.022028	0.000582	0.006901782
Clustering-based subsystems	NULL	Cell wall related cluster	FIG070318: hypothetical protein	0.007311	0.013572	0.002555	0.00747	0.010505	0.004383	0.00361	0.004485	0.004598	0.002203	0.000582	0.008084945
Clustering-based subsystems	NULL	Cell wall related cluster	FIG071646: Sugar transferase	0.009052	0.013572	0.00511	0.00249	0.011818	0.006819	0.000722	0.00598	0.012261	0.01542	0.001164	0.011042851
RNA Metabolism	RNA processing and modification	tRNA modification Bacteria	FIG137478: Hypothetical protein	0.016363	0.015834	0.020438	0.01494	0.008535	0.012663	0.010831	0.014452	0.018173	0.026433	0.034115	0.042199468
Miscellaneous	Plant-Prokaryote DOE project	COG3380 COG2907	FIG143828: Hypothetical protein YbgA	0.016712	0.03393	0.022993	0.059761	0.020353	0.022891	0.018052	0.015948	0.015764	0.004406	0.004541	0.018733409
Miscellaneous	Plant-Prokaryote DOE project	Biotin biosynthesis Experimental	FIG147869: Carbon-nitrogen hydrolase	0.001044	0.004524	0.012774	0.00747	0.002626	0.005357	0.011553	0.012957	0.007882	0.008811	0.000466	0.001616989
Protein Metabolism	Protein folding	Peptidyl-prolyl cis-trans isomerase	FKBP-type peptidyl-prolyl cis-trans isomerase fklB (EC 5.2.1.8)	0.012534	0.004524	0.012774	0.00996	0.013131	0.011689	0.007221	0.015948	0.013356	0.017622	0.022355	0.017076981
Protein Metabolism	Protein processing and modification	G3E family of P-loop GTPases (metallocenter biosynthesis)	FKBP-type peptidyl-prolyl cis-trans isomerase SlyD (EC 5.2.1.8)	0.022282	0.02262	0.015329	0.01743	0.023636	0.023865	0.029605	0.034387	0.028463	0.026433	0.058333	0.028001516
Motility and Chemotaxis	Flagellar motility in Prokaryota	Flagellum	Flagellar basal-body rod protein FlgC	0.024371	0.004524	0.012774	0.00498	0.025606	0.015098	0.024551	0.020433	0.011823	0.011014	0.008616	0.014395146
Motility and Chemotaxis	Flagellar motility in Prokaryota	Flagellum	Flagellar basal-body rod protein FlgG	0.01706	0.020358	0.025548	0.01992	0.047929	0.043347	0.046935	0.055816	0.042476	0.013217	0.0418	0.031274933
Motility and Chemotaxis	Flagellar motility in Prokaryota	Flagellum	Flagellar biosynthesis protein FlhA	0.029593	0.049764	0.022993	0.047311	0.089292	0.093512	0.088816	0.105652	0.076412	0.061678	0.125981	0.067716342
Motility and Chemotaxis	Flagellar motility in Prokaryota	Flagellum	Flagellar biosynthesis protein FlhB	0.018104	0.013572	0.015329	0.00498	0.027575	0.032145	0.031772	0.038374	0.032185	0.05507	0.023752	0.029224117
Motility and Chemotaxis	Flagellar motility in Prokaryota	Flagellar motility	Flagellar biosynthesis protein FlhF	0.007311	0.004524	0.00511	0.00498	0.014444	0.010715	0.010109	0.011961	0.018173	0.006608	0.005123	0.01806295
Motility and Chemotaxis	Flagellar motility in Prokaryota	Flagellum	Flagellar biosynthesis protein FliP	0.007659	0.018096	0.020438	0.02241	0.03808	0.040911	0.040437	0.037377	0.032623	0.046259	0.039471	0.024452028
Motility and Chemotaxis	Flagellar motility in Prokaryota	Flagellum	Flagellar hook protein FlgE	0.030638	0.013572	0.007664	0.01245	0.030858	0.046269	0.036104	0.045849	0.031309	0.01542	0.001397	0.031314371
Virulence, Disease and Defense	NULL	C jejuni colonization of chick caeca	Flagellar hook-associated protein FlgK	0.008704	0.01131	0.010219	0.00498	0.019697	0.0263	0.028883	0.021928	0.024741	0.004406	0.02317	0.018417899
Motility and Chemotaxis	Flagellar motility in Prokaryota	Flagellum	Flagellar hook-associated protein FliD	0.002785	0.009048	0.00511	0.00747	0.013131	0.009254	0.012998	0.014951	0.011385	0.011014	0.001397	0.005560864
Motility and Chemotaxis	Flagellar motility in Prokaryota	Flagellum	Flagellar hook-length control protein FliK	0.028549	0.054288	0.033212	0.039841	0.044646	0.090589	0.06571	0.039869	0.039191	0.008811	0.000233	0.039714826
Virulence, Disease and Defense	NULL	C jejuni colonization of chick caeca	Flagellar motor rotation protein MotA	0.007311	0.02262	0.010219	0.02241	0.048585	0.04773	0.057045	0.063292	0.04773	0.026433	0.075333	0.036204777
Virulence, Disease and Defense	NULL	C jejuni colonization of chick caeca	Flagellar motor rotation protein MotB	0.027853	0.03393	0.030658	0.01245	0.056464	0.063315	0.0556	0.076249	0.052985	0.033042	0.036677	0.038452786
Motility and Chemotaxis	NULL	Bacterial Chemotaxis	Flagellar motor switch protein FliG	0.047349	0.01131	0.022993	0.00747	0.038737	0.040911	0.052712	0.054321	0.036345	0.004406	0.078011	0.034469472
Motility and Chemotaxis	Flagellar motility in Prokaryota	Flagellar motility	Flagellar motor switch protein FliM	0.038994	0.004524	0.017884	0.00747	0.036111	0.027274	0.03105	0.032393	0.031966	0.024231	0.044245	0.023821008
Motility and Chemotaxis	NULL	Bacterial Chemotaxis	Flagellar motor switch protein FliN	0.014274	0.004524	0.012774	0.00747	0.015101	0.018995	0.015886	0.016944	0.016859	0.008811	0.001747	0.016879787
Motility and Chemotaxis	Flagellar motility in Prokaryota	Flagellum	Flagellar M-ring protein FliF	0.002785	0.006786	0.002555	0.00747	0.035454	0.024839	0.023829	0.045849	0.0416	0.006608	0.050998	0.028711414
Motility and Chemotaxis	Flagellar motility in Prokaryota	Flagellum	Flagellar P-ring protein FlgI	0.022282	0.04524	0.033212	0.02988	0.038737	0.064776	0.052712	0.051331	0.0416	0.013217	0.057984	0.035928706
Motility and Chemotaxis	Flagellar motility in Prokaryota	Flagellum	Flagellar regulatory protein FleQ	0.071372	0.04524	0.030658	0.059761	0.049898	0.040424	0.037548	0.040367	0.059772	0.050664	0.074401	0.060735683
Motility and Chemotaxis	Flagellar motility in Prokaryota	Flagellum	Flagellar sensor histidine kinase FleS	0.011141	0.006786	0.007664	0.00249	0.004596	0.005357	0.008665	0.007475	0.013794	0.006608	0.035745	0.013330299
Motility and Chemotaxis	Flagellar motility in Prokaryota	Flagellar motility	Flagellar synthesis regulator FleN	0.018452	0.006786	0.015329	0.01494	0.028888	0.028735	0.03105	0.035384	0.021019	0.013217	0.039937	0.019127796
Motility and Chemotaxis	Flagellar motility in Prokaryota	Archaeal Flagellum	Flagella-related protein FlaI	0.002785	0.002262	0.002555	0.01245	0.008535	0.004383	0.010831	0.002492	0.006787	0.006608	0.001164	0.006231323
Motility and Chemotaxis	Flagellar motility in Prokaryota	Flagellar motility	Flagellin	0.008704	0.01131	0.00511	0.00249	0.023636	0.021917	0.015164	0.032892	0.014888	0.028636	0.027013	0.016919226
Motility and Chemotaxis	Flagellar motility in Prokaryota	Flagellum in Campylobacter	Flagellin protein FlaA	0.00383	0.013572	0.002555	0.01245	0.039393	0.036041	0.051268	0.046846	0.019705	0.022028	0.010712	0.024294273
Motility and Chemotaxis	Flagellar motility in Prokaryota	Flagellar motility	Flagellum-specific ATP synthase FliI	0.032031	0.027144	0.007664	0.034861	0.061716	0.0526	0.06571	0.061298	0.058021	0.046259	0.084182	0.038492225
Cofactors, Vitamins, Prosthetic Groups, Pigments	Riboflavin, FMN, FAD	Flavodoxin	Flavodoxin	0.014971	0.024882	0.002555	0.01743	0.020353	0.014611	0.012998	0.012459	0.025617	0.01542	0.043313	0.018772847
Respiration	Electron accepting reactions	Anaerobic respiratory reductases	Flavodoxin reductases (ferredoxin-NADPH reductases) family 1	0.007659	0.002262	0.00511	0.00249	0.004596	0.005357	0.006499	0.012957	0.009853	0.013217	0.000233	0.012778157
Membrane Transport	Protein secretion system, Type II	Widespread colonization island	Flp pilus assembly protein RcpC/CpaB	0.000348	0.004524	0.00511	0.00498	0.003283	0.003896	0.010109	0.001993	0.002627	0.004406	0.000582	0.002366325
Membrane Transport	Protein secretion system, Type II	Widespread colonization island	Flp pilus assembly protein TadB	0.006963	0.009048	0.010219	0.02739	0.011818	0.009741	0.007943	0.014452	0.009196	0.004406	0.000699	0.006822905
Cofactors, Vitamins, Prosthetic Groups, Pigments	Riboflavin, FMN, FAD	riboflavin to FAD	FMN adenylyltransferase (EC 2.7.7.2)	0.019149	0.03393	0.030658	0.01245	0.023636	0.030196	0.015164	0.02741	0.035688	0.013217	0.029923	0.033049677
Cofactors, Vitamins, Prosthetic Groups, Pigments	Folate and pterines	Folate Biosynthesis	FolM Alternative dihydrofolate reductase 1	0.016363	0.004524	0.012774	0.02241	0.012475	0.017046	0.021663	0.013456	0.024084	0.019825	0.027246	0.013251422
Cofactors, Vitamins, Prosthetic Groups, Pigments	Folate and pterines	Folate Biosynthesis	Folylpolyglutamate synthase (EC 6.3.2.17)	0.006963	0.031668	0.012774	0.00747	0.030202	0.029222	0.024551	0.023921	0.01861	0.017622	0.028759	0.027449373
DNA Metabolism	DNA repair	DNA Repair Base Excision	Formamidopyrimidine-DNA glycosylase (EC 3.2.2.23)	0.059535	0.029406	0.051096	0.047311	0.029545	0.030684	0.047658	0.040367	0.044446	0.008811	0.043313	0.024925293
Respiration	NULL	Formate hydrogenase	Formate dehydrogenase chain D (EC 1.2.1.2)	0.011489	0.02262	0.015329	0.034861	0.025606	0.022891	0.018774	0.020433	0.026055	0.017622	0.007685	0.020705346
Respiration	Electron donating reactions	Formate dehydrogenase	Formate dehydrogenase O alpha subunit (EC 1.2.1.2)	0.012882	0.020358	0.007664	0.034861	0.016414	0.028735	0.016608	0.003489	0.004379	0.006608	0.001514	0.017984072
Cofactors, Vitamins, Prosthetic Groups, Pigments	Folate and pterines	YgfZ	Formate dehydrogenase-O, major subunit (EC 1.2.1.2)	0.110018	0.149291	0.099637	0.119522	0.110958	0.106175	0.064265	0.054321	0.094147	0.03965	0.006753	0.087317403

Carbohydrates	Fermentation	Fermentations: Mixed acid	Formate efflux transporter (TC 2.A.44 family)	0.005222	0.009048	0.007664	0.00249	0.003283	0.001948	0.007221	0.003489	0.004598	0.004406	0.001164	0.004298824
Respiration	NULL	Formate hydrogenase	Formate hydrogenlyase subunit 3	0.033771	0.015834	0.033212	0.039841	0.013131	0.005357	0.007221	0.002492	0.021457	0.019825	0.000699	0.013961319
Respiration	NULL	Formate hydrogenase	Formate hydrogenlyase subunit 5	0.003133	0.004524	0.002555	0.00249	0.003939	0.002435	0.002888	0.000498	0.004598	0.002203	0.000815	0.0031551
Respiration	NULL	Formate hydrogenase	Formate hydrogenlyase transcriptional activator	0.039342	0.040716	0.051096	0.037351	0.068282	0.078413	0.040437	0.025416	0.031966	0.022028	0.000699	0.064127416
Carbohydrates	One-carbon Metabolism	Serine-glyoxylate cycle	Formate--tetrahydrofolate ligase (EC 6.3.4.3)	0.067543	0.083693	0.051096	0.124502	0.083383	0.086693	0.082318	0.047344	0.051234	0.022028	0.042149	0.071975728
Miscellaneous	Plant-Prokaryote DOE project	COG4319	Formyltetrahydrofolate deformylase (EC 3.5.1.10)	0.044216	0.02262	0.015329	0.01743	0.014444	0.014611	0.020218	0.029403	0.025179	0.019825	0.161843	0.02662116
Carbohydrates	Monosaccharides	Fructose utilization	Fructokinase (EC 2.7.1.4)	0.0094	0.009048	0.028103	0.0249	0.022323	0.022404	0.012998	0.018938	0.020362	0.008811	0.020376	0.012778157
Carbohydrates	CO2 fixation	Calvin-Benson cycle	Fructose-1,6-bisphosphatase, GlpX type (EC 3.1.3.11)	0.014623	0.015834	0.020438	0.00996	0.01904	0.01802	0.023829	0.025416	0.025179	0.01542	0.008616	0.008006067
Carbohydrates	Central carbohydrate metabolism	Glycolysis and Gluconeogenesis	Fructose-1,6-bisphosphatase, type I (EC 3.1.3.11)	0.050135	0.03393	0.038322	0.01992	0.034797	0.024352	0.03105	0.029403	0.046855	0.154195	0.062991	0.045630639
Carbohydrates	Fermentation	Fermentations: Lactate	Fructose-6-phosphate phosphoketolase (EC 4.1.2.22)	0.025416	0.031668	0.020438	0.042331	0.011161	0.011202	0.007221	0.005482	0.038316	0.006608	0.000466	0.016209328
Carbohydrates	NULL	Unknown carbohydrate utilization (cluster Yeg)	Fructose-bisphosphate aldolase class I (EC 4.1.2.13)	0.045609	0.040716	0.06387	0.049801	0.036767	0.04773	0.044047	0.055816	0.0624	0.03965	0.005123	0.026345088
Carbohydrates	CO2 fixation	Calvin-Benson cycle	Fructose-bisphosphate aldolase class II (EC 4.1.2.13)	0.073461	0.079169	0.061315	0.079681	0.073534	0.068186	0.067154	0.059803	0.04576	0.204859	0.115735	0.095796736
Carbohydrates	Monosaccharides	L-fucose utilization	Fucose permease	0.010793	0.009048	0.010219	0.01743	0.005252	0.002435	0.002888	0.00897	0.003065	0.004406	0.000349	0.007375047
Carbohydrates	One-carbon Metabolism	Serine-glyoxylate cycle	Fumarate hydratase class I, aerobic (EC 4.2.1.2)	0.067543	0.088217	0.074089	0.077191	0.055807	0.061367	0.06571	0.048839	0.06853	0.116748	0.086277	0.062116039
Carbohydrates	One-carbon Metabolism	Serine-glyoxylate cycle	Fumarate hydratase class I, anaerobic (EC 4.2.1.2)	0.007659	0.01131	0.015329	0.00498	0.004596	0.005357	0.008665	0.002492	0.002189	0.002203	0.001164	0.006428517
Carbohydrates	Central carbohydrate metabolism	TCA Cycle	Fumarate hydratase class II (EC 4.2.1.2)	0.129515	0.049764	0.097082	0.129482	0.104392	0.092051	0.095315	0.106649	0.124362	0.13437	0.07999	0.100134999
Cofactors, Vitamins, Prosthetic Groups, Pigments	Folate and pterines	Pterin carbinolamine dehydratase	Fumarylacetoacetase (EC 3.7.1.2)	0.005919	0.020358	0.033212	0.032371	0.015757	0.020456	0.028883	0.051331	0.051015	0.207062	0.064621	0.034311717
Metabolism of Aromatic Compounds	NULL	Gentisare degradation	Fumarylacetoacetate hydrolase family protein	0.020541	0.027144	0.025548	0.01992	0.022979	0.024839	0.021663	0.024918	0.044008	0.019825	0.052861	0.034430033
Nitrogen Metabolism	NULL	Nitrosative stress	Functional role page for Anaerobic nitric oxide reductase transcription regulator NorR	0.007311	0.015834	0.007664	0.01494	0.026262	0.012176	0.010109	0.012459	0.009415	0.013217	0.014088	0.014907849
Dormancy and Sporulation	NULL	Spore Core Dehydration	Fused spore maturation proteins A and B	0.001741	0.013572	0.002555	0.0249	0.005909	0.005844	0.004333	0.002492	0.000657	0.004406	0.05682	0.007966629
Carbohydrates	NULL	Sugar utilization in Thermotogales	Galactokinase (EC 2.7.1.6)	0.005919	0.018096	0.033212	0.032371	0.018384	0.01315	0.012998	0.013456	0.021238	0.002203	0.002212	0.028632536
Carbohydrates	Di- and oligosaccharides	Lactose and Galactose Uptake and Utilization	Galactose-1-phosphate uridylyltransferase (EC 2.7.7.10)	0.005222	0.054288	0.038322	0.037351	0.015757	0.015585	0.024551	0.017443	0.009196	0.002203	0.003726	0.019916571
Amino Acids and Derivatives	Arginine; urea cycle, polyamines	Putrescine utilization pathways	Gamma-aminobutyrate:alpha-ketoglutarate aminotransferase (EC 2.6.1.19)	0.021238	0.027144	0.015329	0.037351	0.019697	0.019969	0.015886	0.021429	0.031747	0.004406	0.01339	0.019601061
Miscellaneous	Plant-Prokaryote DOE project	PROSC	Gamma-glutamyl phosphate reductase (EC 1.2.1.41)	0.088084	0.095003	0.066425	0.079681	0.062373	0.093999	0.068598	0.081731	0.063495	0.116748	0.083716	0.074539247
Miscellaneous	Plant-Prokaryote DOE project	At3g50560	Gamma-glutamyltranspeptidase (EC 2.3.2.2)	0.08878	0.108575	0.099637	0.134462	0.129341	0.161697	0.155248	0.186387	0.179317	0.099125	0.034464	0.106879026
Cell Wall and Capsule	Capsular and extracellular polysacchrides	Capsular heptose biosynthesis	GDP-L-fucose synthetase (EC 1.1.1.271)	0.040386	0.038454	0.045986	0.02241	0.036767	0.045295	0.04838	0.03638	0.028025	0.105734	0.077312	0.034469472
Cell Wall and Capsule	Gram-Negative cell wall components	Perosamine Synthesis Vibrio	GDP-mannose 4,6-dehydratase (EC 4.2.1.47)	0.044216	0.01131	0.017884	0.02241	0.010505	0.019969	0.01733	0.024918	0.020362	0.035245	0.077428	0.024767538
Cofactors, Vitamins, Prosthetic Groups, Pigments	Quinone cofactors	Menaquinone Biosynthesis via Futalosine -- gjo	Gene SCO4494, often clustered with other genes in menaquinone via futalosine pathway	0.003482	0.006786	0.028103	0.01245	0.019697	0.013637	0.010109	0.017941	0.012042	0.008811	0.000815	0.018102389
Membrane Transport	Protein secretion system, Type II	General Secretion Pathway	General secretion pathway protein A	0.04352	0.027144	0.043432	0.044821	0.061716	0.060393	0.020218	0.027908	0.027587	0.028636	0.028643	0.028869169
Membrane Transport	Protein secretion system, Type II	General Secretion Pathway	General secretion pathway protein E	0.239184	0.088217	0.117521	0.109562	0.170048	0.156827	0.139362	0.101167	0.11232	0.050664	0.105722	0.114096318
Membrane Transport	Protein secretion system, Type II	General Secretion Pathway	General secretion pathway protein F	0.017408	0.038454	0.028103	0.01992	0.035454	0.029222	0.028883	0.028407	0.038535	0.008811	0.000931	0.029500189
Membrane Transport	Protein secretion system, Type II	General Secretion Pathway	General secretion pathway protein G	0.058839	0.01131	0.028103	0.02241	0.024949	0.020456	0.02094	0.022925	0.011823	0.011014	0.006287	0.01711642
Fatty Acids, Lipids, and Isoprenoids	Isoprenoids	Archaeal lipids	Geranylgeranyl pyrophosphate synthetase (EC 2.5.1.29)	0.012186	0.02262	0.020438	0.01743	0.016414	0.02143	0.019496	0.02442	0.017735	0.024231	0.024335	0.018220705
Fatty Acids, Lipids, and Isoprenoids	Isoprenoids	Carotenoids	Geranyltranstransferase (farnesyldiphosphate synthase) (EC 2.5.1.10)	0.017408	0.02262	0.033212	0.02241	0.026919	0.032145	0.025995	0.032393	0.031966	0.033042	0.036793	0.034311717
Carbohydrates	Monosaccharides	D-galactarate, D-glucarate and D-glycerate catabolism	Glucarate dehydratase (EC 4.2.1.40)	0.005222	0.004524	0.002555	0.00498	0.001313	0.001461	0.004333	0.00299	0.002408	0.002203	0.001863	0.004732651
Carbohydrates	Di- and oligosaccharides	Maltose and Maltodextrin Utilization	Glucoamylase (EC 3.2.1.3)	0.029245	0.03393	0.038322	0.042331	0.01707	0.010715	0.011553	0.010964	0.046855	0.011014	0.003842	0.015854379
Carbohydrates	Central carbohydrate metabolism	Peripheral Glucose Catabolism Pathways	Glucokinase (EC 2.7.1.2)	0.018104	0.020358	0.022993	0.00996	0.015757	0.019969	0.012275	0.016944	0.02693	0.002203	0.000466	0.012462647
Carbohydrates	Monosaccharides	D-gluconate and ketogluconates metabolism	Gluconate dehydratase (EC 4.2.1.39)	0.010097	0.027144	0.025548	0.01992	0.025606	0.026787	0.028161	0.013954	0.029996	0.006608	0.001048	0.014750094
Carbohydrates	Central carbohydrate metabolism	Entner-Doudoroff Pathway	Gluconolactonase (EC 3.1.1.17)	0.011141	0.040716	0.022993	0.037351	0.020353	0.014611	0.020218	0.014951	0.033061	0.01542	0.000699	0.01329086
Cell Wall and Capsule	Capsular and extracellular polysacchrides	Sialic Acid Metabolism	Glucosamine-1-phosphate N-acetyltransferase (EC 2.3.1.157)	0.023675	0.02262	0.025548	0.01992	0.024949	0.020456	0.02094	0.022426	0.028463	0.01542	0.056703	0.026345088
Carbohydrates	Aminosugars	Chitin and N-acetylglucosamine utilization	Glucosamine-6-phosphate deaminase (EC 3.5.99.6)	0.051527	0.013572	0.017884	0.01494	0.022979	0.014611	0.01372	0.020931	0.020362	0.041853	0.02352	0.031235494
Carbohydrates	NULL	Sugar utilization in Thermotogales	Glucosamine-6-phosphate deaminase [isomerizing], alternative (EC 3.5.99.6)	0.007659	0.002262	0.00511	0.00498	0.005909	0.000974	0.00361	0.001993	0.004379	0.004406	0.000582	0.0031551
Miscellaneous	Plant-Prokaryote DOE project	PROSC	Glucosamine--fructose-6-phosphate aminotransferase [isomerizing] (EC 2.6.1.16)	0.193228	0.223936	0.194165	0.206673	0.230451	0.207966	0.196407	0.18539	0.181069	0.198251	0.132735	0.196444438
Carbohydrates	Monosaccharides	D-gluconate and ketogluconates metabolism	Glucose 1-dehydrogenase (EC 1.1.1.47)	0.002089	0.020358	0.002555	0.00996	0.012475	0.013637	0.007943	0.002492	0.017297	0.006608	0.000349	0.010056883
Carbohydrates	Central carbohydrate metabolism	Entner-Doudoroff Pathway	Glucose dehydrogenase, PQQ-dependent (EC 1.1.5.2)	0.000696	0.004524	0.012774	0.01494	0.009848	0.006332	0.008665	0.013954	0.032404	0.01542	0.000466	0.006231323
Clustering-based subsystems	NULL	Glycogen metabolism cluster	Glucose-1-phosphate adenylyltransferase (EC 2.7.7.27)	0.113151	0.108575	0.102192	0.097112	0.070251	0.067212	0.054156	0.048341	0.070939	0.143181	0.035047	0.094258624
Cell Wall and Capsule	Capsular and extracellular polysacchrides	dTDP-rhamnose synthesis	Glucose-1-phosphate cytidylyltransferase (EC 2.7.7.33)	0.011141	0.047502	0.030658	0.0249	0.022979	0.018508	0.027439	0.032892	0.02277	0.022028	0.002329	0.025043609
Cell Wall and Capsule	Capsular and extracellular polysacchrides	Rhamnose containing glycans	Glucose-1-phosphate thymidylyltransferase (EC 2.7.7.24)	0.178605	0.095003	0.148178	0.114542	0.092574	0.091076	0.085206	0.107147	0.089549	0.176223	0.115269	0.115161165
Carbohydrates	NULL	Sugar utilization in Thermotogales	Glucose-6-phosphate 1-dehydrogenase (EC 1.1.1.49)	0.05675	0.099527	0.102192	0.109562	0.059747	0.068673	0.049824	0.047344	0.101153	0.044056	0.054724	0.068229046
Carbohydrates	Central carbohydrate metabolism	Glycolysis and Gluconeogenesis	Glucose-6-phosphate isomerase (EC 5.3.1.9)	0.097832	0.113099	0.099637	0.114542	0.108332	0.124682	0.103258	0.103161	0.099621	0.081503	0.228327	0.115910501
Respiration	Electron donating reactions	Respiratory dehydrogenases 1	Glucose-methanol-choline (GMC) oxidoreductase:NAD binding site	0.034468	0.029406	0.038322	0.0249	0.032828	0.018508	0.019496	0.052826	0.07488	0.006608	0.048436	0.027804322
Miscellaneous	Plant-Prokaryote DOE project	Iojap	Glutamate 5-kinase (EC 2.7.2.11)	0.223169	0.065598	0.066425	0.044821	0.070251	0.066237	0.044047	0.053823	0.058021	0.057273	0.061477	0.066927567
Stress Response	Acid stress	Acid resistance mechanisms	Glutamate decarboxylase (EC 4.1.1.15)	0.018104	0.015834	0.00511	0.01992	0.007222	0.005357	0.005055	0.000498	0.012918	0.002203	0.002096	0.005639742
Miscellaneous	Plant-Prokaryote DOE project	Experimental - Histidine Degradation	Glutamate formiminotransferase (EC 2.1.2.5)	0.010793	0.020358	0.015329	0.01245	0.003939	0.009741	0.012998	0.005482	0.003941	0.006608	0.000582	0.009149791
Amino Acids and Derivatives	Arginine; urea cycle, polyamines	Arginine Biosynthesis extended	Glutamate N-acetyltransferase (EC 2.3.1.35)	0.077639	0.040716	0.043432	0.054781	0.041363	0.031658	0.044047	0.03339	0.032185	0.022028	0.051347	0.032852483
Cell Wall and Capsule	NULL	Peptidoglycan Biosynthesis	Glutamate racemase (EC 5.1.1.3)	0.012882	0.029406	0.040877	0.0249	0.015101	0.017046	0.024551	0.029902	0.029558	0.037447	0.006171	0.036283655
Nitrogen Metabolism	NULL	Ammonia assimilation	Glutamate synthase [NADPH] large chain (EC	0.295586	0.341559	0.288692	0.281375	0.267875	0.293198	0.289556	0.25217	0.287696	0.068286	0.474002	0.336175947

			1.4.1.13)												
Nitrogen Metabolism	NULL	Ammonia assimilation	Glutamate synthase [NADPH] small chain (EC 1.4.1.13)	0.206458	0.264652	0.194165	0.234064	0.166109	0.184588	0.145861	0.108643	0.112101	0.127762	0.080223	0.17708001
Cofactors, Vitamins, Prosthetic Groups, Pigments	Tetrapyrroles	Heme and Siroheme Biosynthesis	Glutamate-1-semialdehyde aminotransferase (EC 5.4.3.8)	0.110714	0.095003	0.137959	0.131972	0.104392	0.115428	0.098203	0.093692	0.117793	0.264335	0.12144	0.113504737
Nitrogen Metabolism	NULL	Ammonia assimilation	Glutamate-ammonia-ligase adenylyltransferase (EC 2.7.7.42)	0.088084	0.038454	0.05876	0.069721	0.059747	0.08231	0.059211	0.045849	0.077507	0.011014	0.11853	0.058211602
Cofactors, Vitamins, Prosthetic Groups, Pigments	Folate and pterines	YgfZ	Glutamate--cysteine ligase (EC 6.3.2.2)	0.0094	0.02262	0.028103	0.037351	0.027575	0.03458	0.03827	0.049338	0.04992	0.011014	0.075798	0.02977626
Amino Acids and Derivatives	Glutamine, glutamate, aspartate, asparagine; ammonia assimilation	Glutamine, Glutamate, Aspartate and Asparagine Biosynthesis	Glutaminase (EC 3.5.1.2)	0.020541	0.018096	0.020438	0.01992	0.009848	0.011202	0.004333	0.00897	0.027149	0.011014	0.051813	0.027607128
Cofactors, Vitamins, Prosthetic Groups, Pigments	NAD and NADP	NAD and NADP cofactor biosynthesis global	Glutamine amidotransferase chain of NAD synthetase	0.023675	0.02262	0.022993	0.042331	0.024949	0.023378	0.027439	0.020931	0.024741	0.011014	0.001514	0.01869397
Amino Acids and Derivatives	Glutamine, glutamate, aspartate, asparagine; ammonia assimilation	Glutamine, Glutamate, Aspartate and Asparagine Biosynthesis	Glutamine synthetase (EC 6.3.1.2)	0.005222	0.004524	0.007664	0.00498	0.005252	0.012176	0.007943	0.004984	0.011166	0.002203	0.000466	0.003194539
Amino Acids and Derivatives	Glutamine, glutamate, aspartate, asparagine; ammonia assimilation	Glutamine, Glutamate, Aspartate and Asparagine Biosynthesis	glutamine synthetase family protein	0.028897	0.018096	0.020438	0.034861	0.019697	0.024352	0.029605	0.047843	0.042476	0.03965	0.124468	0.024767538
Amino Acids and Derivatives	Glutamine, glutamate, aspartate, asparagine; ammonia assimilation	Glutamine synthetases	Glutamine synthetase type I (EC 6.3.1.2)	0.133693	0.183221	0.178836	0.231574	0.17727	0.131988	0.149471	0.132564	0.149103	0.096923	0.239854	0.145765638
Nitrogen Metabolism	NULL	Ammonia assimilation	Glutamine synthetase type II, eukaryotic (EC 6.3.1.2)	0.006615	0.006786	0.010219	0.01743	0.008535	0.014611	0.015886	0.019436	0.014888	0.026433	0.004541	0.013645809
Nitrogen Metabolism	NULL	Ammonia assimilation	Glutamine synthetase type III, GlnN (EC 6.3.1.2)	0.020193	0.038454	0.030658	0.02739	0.017727	0.030196	0.024551	0.040367	0.031966	0.193845	0.110496	0.054701553
Protein Metabolism	Protein biosynthesis	tRNA aminoacylation, Glu and Gln	Glutaminyl-tRNA synthetase (EC 6.1.1.18)	0.130907	0.180959	0.158398	0.171813	0.130655	0.150982	0.129253	0.106649	0.113852	0.074895	0.160213	0.16631323
Cofactors, Vitamins, Prosthetic Groups, Pigments	Tetrapyrroles	Heme and Siroheme Biosynthesis	Glutamyl-tRNA reductase (EC 1.2.1.70)	0.038994	0.024882	0.035767	0.049801	0.050555	0.053087	0.045491	0.03937	0.046636	0.09472	0.075216	0.057541143
Cofactors, Vitamins, Prosthetic Groups, Pigments	Tetrapyrroles	Heme and Siroheme Biosynthesis	Glutamyl-tRNA synthetase (EC 6.1.1.17)	0.055357	0.097265	0.071534	0.069721	0.098483	0.090102	0.10398	0.103659	0.08736	0.03965	0.022472	0.101712549
Miscellaneous	Plant-Prokaryote DOE project	At3g21300	Glutamyl-tRNA(Gln) amidotransferase subunit A (EC 6.3.5.7)	0.05292	0.072383	0.061315	0.047311	0.060403	0.056984	0.067876	0.054321	0.046636	0.052867	0.025965	0.037466817
Miscellaneous	Plant-Prokaryote DOE project	At3g21300	Glutamyl-tRNA(Gln) amidotransferase subunit B (EC 6.3.5.7)	0.054661	0.061074	0.045986	0.069721	0.053838	0.064776	0.059933	0.072761	0.057364	0.037447	0.068347	0.055332573
Miscellaneous	Plant-Prokaryote DOE project	At3g21300	Glutamyl-tRNA(Gln) amidotransferase subunit C (EC 6.3.5.7)	0.004526	0.002262	0.007664	0.00996	0.009192	0.009741	0.005055	0.013456	0.005255	0.008811	0.001164	0.007966629
Protein Metabolism	Protein biosynthesis	tRNA aminoacylation, Glu and Gln	Glutamyl-tRNA(Gln) synthetase (EC 6.1.1.24)	0.034468	0.015834	0.030658	0.039841	0.024293	0.030196	0.046935	0.041364	0.027587	0.017622	0.028643	0.026187333
Amino Acids and Derivatives	Lysine, threonine, methionine, and cysteine	Lysine degradation	Glutarate-semialdehyde dehydrogenase (EC 1.2.1.20)	0.002785	0.002262	0.00511	0.00498	0.007222	0.007793	0.007221	0.007475	0.011604	0.006608	0.032485	0.007572241
Phages, Prophages, Transposable elements, Plasmids	Phages, Prophages	Phage DNA synthesis	Glutaredoxin	0.006615	0.02262	0.007664	0.00747	0.013131	0.01315	0.012998	0.010964	0.022114	0.002203	0.032718	0.014040197
Metabolism of Aromatic Compounds	Anaerobic degradation of aromatic compounds	Anaerobic benzoate metabolism	Glutaryl-CoA dehydrogenase (EC 1.3.99.7)	0.053616	0.029406	0.06387	0.067231	0.030858	0.040911	0.047658	0.055318	0.084076	0.057273	0.080922	0.043698141
Stress Response	Oxidative stress	Glutathione: Redox cycle	Glutathione reductase (EC 1.8.1.7)	0.085647	0.01131	0.028103	0.02739	0.019697	0.022891	0.03827	0.044852	0.055831	0.013217	0.084997	0.030998861
Stress Response	Oxidative stress	Glutathione: Non-redox reactions	Glutathione S-transferase (EC 2.5.1.18)	0.041083	0.029406	0.053651	0.044821	0.034797	0.036528	0.062099	0.086216	0.075975	0.050664	0.166151	0.024570344
Miscellaneous	Plant-Prokaryote DOE project	At3g50560	Glutathione S-transferase family protein	0.008704	0.006786	0.012774	0.00498	0.009848	0.006819	0.019496	0.022426	0.013137	0.013217	0.000815	0.004929844
Clustering-based subsystems	tRNA sulfuration	CBSS-89187.3.peg.2957	Glutathione S-transferase, omega (EC 2.5.1.18)	0.012882	0.009048	0.015329	0.00996	0.023636	0.019482	0.026717	0.032892	0.048606	0.019825	0.109681	0.019167235
Stress Response	Heat shock	Heat shock dnaK gene cluster extended	Glutathione synthetase (EC 6.3.2.3)	0.096092	0.036192	0.05876	0.064741	0.035454	0.033119	0.03466	0.053823	0.050796	0.024231	0.078011	0.034469472
Potassium metabolism	NULL	Glutathione-regulated potassium-efflux system and associated functions	Glutathione-regulated potassium-efflux system protein KefB	0.031682	0.03393	0.038322	0.032371	0.05909	0.068186	0.064988	0.042859	0.050577	0.01542	0.07836	0.048509669
Potassium metabolism	NULL	Potassium homeostasis	Glutathione-regulated potassium-efflux system protein KefC	0.005222	0.006786	0.015329	0.02988	0.036111	0.026787	0.027439	0.029403	0.033937	0.004406	0.002212	0.017668562
Carbohydrates	NULL	Sugar utilization in Thermotogales	Glycerol kinase (EC 2.7.1.30)	0.087388	0.088217	0.091973	0.074701	0.0801	0.085232	0.096037	0.103659	0.104657	0.099125	0.172438	0.070753126
Carbohydrates	Sugar alcohols	Glycerol and Glycerol-3-phosphate Uptake and Utilization	Glycerol-3-phosphate ABC transporter, ATP-binding protein UgpC (TC 3.A.1.1.3)	0.012534	0.01131	0.033212	0.044821	0.042676	0.051626	0.038993	0.037377	0.048606	0.048461	0.017931	0.019482745
Carbohydrates	Sugar alcohols	Glycerol and Glycerol-3-phosphate Uptake and Utilization	Glycerol-3-phosphate ABC transporter, periplasmic glycerol-3-phosphate-binding protein (TC 3.A.1.1.3)	0.004178	0.01131	0.00511	0.00498	0.009192	0.017533	0.018052	0.019436	0.021895	0.030839	0.000931	0.007453925
Carbohydrates	NULL	Sugar utilization in Thermotogales	Glycerol-3-phosphate ABC transporter, permease protein UgpA (TC 3.A.1.1.3)	0.012534	0.018096	0.017884	0.02241	0.044646	0.068186	0.037548	0.053823	0.063276	0.035245	0.02515	0.021809631
Carbohydrates	NULL	Sugar utilization in Thermotogales	Glycerol-3-phosphate ABC transporter, permease protein UgpE (TC 3.A.1.1.3)	0.004526	0.013572	0.020438	0.01494	0.021666	0.0263	0.016608	0.017443	0.027149	0.011014	0.012342	0.008676526
Fatty Acids, Lipids, and Isoprenoids	Phospholipids	Glycerolipid and Glycerophospholipid Metabolism in Bacteria	Glycerol-3-phosphate acyltransferase (EC 2.3.1.15)	0.003133	0.004524	0.007664	0.00996	0.01707	0.025813	0.016608	0.023921	0.047074	0.013217	0.045991	0.016288206
Carbohydrates	NULL	Sugar utilization in Thermotogales	Glycerol-3-phosphate dehydrogenase (EC 1.1.5.3)	0.018104	0.031668	0.033212	0.032371	0.013788	0.01802	0.014442	0.016944	0.020581	0.011014	0.016417	0.012107698
Clustering-based subsystems	NULL	Glutaredoxin 3 containing cluster	Glycerol-3-phosphate dehydrogenase [NAD(P)+] (EC 1.1.1.94)	0.041431	0.052026	0.125185	0.082171	0.045959	0.064776	0.041881	0.066282	0.054299	0.081503	0.044711	0.064640119
Clustering-based subsystems	NULL	CBSS-176299.4.peg.1996A	Glycerophosphoryl diester phosphodiesterase (EC 3.1.4.46)	0.01706	0.027144	0.056206	0.02241	0.036767	0.033606	0.050546	0.046348	0.042476	0.01542	0.016883	0.022401213
Carbohydrates	CO2 fixation	Photorespiration (oxidative C2 cycle)	Glycine cleavage system H protein	0.018104	0.03393	0.025548	0.047311	0.039393	0.038963	0.053434	0.041862	0.037659	0.024231	0.041916	0.037348501
Amino Acids and Derivatives	Alanine, serine, and glycine	Glycine and Serine Utilization	Glycine dehydrogenase [decarboxylating] (glycine cleavage system P protein) (EC 1.4.4.2)	0.082862	0.05655	0.076644	0.052291	0.027575	0.025813	0.064265	0.075252	0.079916	0.017622	0.181753	0.063575273
Amino Acids and Derivatives	Alanine, serine, and glycine	Glycine cleavage system	Glycine dehydrogenase [decarboxylating] (glycine cleavage system P1 protein) (EC 1.4.4.2)	0.041083	0.065598	0.061315	0.064741	0.061716	0.060393	0.057767	0.043357	0.019705	0.022028	0.002212	0.039281
Amino Acids and Derivatives	Alanine, serine, and glycine	Glycine cleavage system	Glycine dehydrogenase [decarboxylating] (glycine cleavage system P2 protein) (EC 1.4.4.2)	0.096092	0.079169	0.094528	0.062251	0.080756	0.083771	0.107591	0.064787	0.033718	0.035245	0.004657	0.065389456
Cofactors, Vitamins, Prosthetic Groups, Pigments	NULL	Thiamin biosynthesis	Glycine oxidase ThiO (EC 1.4.3.19)	0.004874	0.006786	0.015329	0.00747	0.011161	0.009254	0.012275	0.013954	0.005474	0.004406	0.022122	0.012423208
Carbohydrates	Polysaccharides	Glycogen metabolism	Glycogen branching enzyme, GH-57-type, archaeal (EC 2.4.1.18)	0.005222	0.020358	0.025548	0.01992	0.015757	0.01802	0.020218	0.013456	0.006787	0.011014	0.000116	0.011003413
Clustering-based subsystems	NULL	Glycogen metabolism cluster	Glycogen debranching enzyme (EC 3.2.1.-)	0.041083	0.097265	0.107302	0.119522	0.056464	0.050652	0.041881	0.039869	0.102248	0.048461	0.025033	0.042712172
Carbohydrates	Di- and oligosaccharides	Maltose and Maltodextrin Utilization	Glycogen phosphorylase (EC 2.4.1.1)	0.212376	0.368703	0.344898	0.326195	0.228481	0.188484	0.148749	0.119108	0.16224	0.068286	0.051697	0.214941215
Carbohydrates	Polysaccharides	Glycogen metabolism	Glycogen synthase, ADP-glucose transglucosylase (EC 2.4.1.21)	0.077987	0.110837	0.104747	0.094622	0.066969	0.081336	0.053434	0.059305	0.050796	0.019825	0.002096	0.079626846
Carbohydrates	Central carbohydrate metabolism	Glycolate, glyoxylate interconversions	Glycolate dehydrogenase (EC 1.1.99.14), FAD-binding subunit GlcE	0.019149	0.006786	0.010219	0.00996	0.011818	0.015585	0.010109	0.009469	0.016421	0.002203	0.001164	0.007335608
Carbohydrates	CO2 fixation	Photorespiration (oxidative C2 cycle)	Glycolate dehydrogenase (EC 1.1.99.14), iron-sulfur subunit GlcF	0.06232	0.015834	0.022993	0.01992	0.022979	0.031171	0.022385	0.017443	0.028463	0.008811	0.003027	0.015972696
Carbohydrates	CO2 fixation	Photorespiration (oxidative C2 cycle)	Glycolate dehydrogenase (EC 1.1.99.14), subunit GlcD	0.043868	0.031668	0.056206	0.037351	0.038737	0.025326	0.03105	0.025915	0.04357	0.035245	0.009431	0.033759574
Clustering-based subsystems	Biosynthesis of galactoglycans and related lipopolysacharides	CBSS-376686.6.peg.291	Glycosyl transferase, family 2	0.046653	0.076907	0.06387	0.084661	0.082069	0.056497	0.070042	0.054321	0.06437	0.019825	0.056121	0.059315887
Clustering-based subsystems	Biosynthesis of galactoglycans and related lipopolysacharides	CBSS-376686.6.peg.291	glycosyl transferase, family 2	0.011489	0.029406	0.033212	0.01992	0.030202	0.030684	0.025273	0.017941	0.026712	0.017622	0.001048	0.025319681
Clustering-based subsystems	Biosynthesis of galactoglycans and related lipopolysacharides	CBSS-258594.1.peg.3339	Glycosyl transferase, group 1	0.044912	0.063336	0.053651	0.064741	0.053181	0.043347	0.044047	0.037377	0.071815	0.033042	0.00489	0.053991656
Cell Wall and Capsule	Capsular and extracellular polysacchrides	Exopolysaccharide Biosynthesis	Glycosyl transferase, group 2 family protein	0.055705	0.031668	0.038322	0.044821	0.047929	0.047243	0.039715	0.057311	0.059335	0.077098	0.0418	0.080691693
Clustering-based subsystems	Biosynthesis of galactoglycans and related lipopolysacharides	CBSS-258594.1.peg.3339	Glycosyltransferase	0.235355	0.368703	0.38833	0.415837	0.361762	0.36528	0.322772	0.314964	0.322509	0.339229	0.189321	0.323910495

Clustering-based subsystems	Biosynthesis of galactoglycans and related lipopolysacharides	CBSS-376686.6.peg.291	Glycosyltransferase (EC 2.4.1.-)	0.027853	0.031668	0.030658	0.01992	0.028232	0.031658	0.02094	0.016446	0.024084	0.006608	0.001747	0.024175957
Protein Metabolism	Protein biosynthesis	tRNA aminoacylation, Gly	Glycyl-tRNA synthetase (EC 6.1.1.14)	0.09261	0.072383	0.061315	0.034861	0.063686	0.055036	0.083762	0.075252	0.067655	0.376677	0.028643	0.059513081
Protein Metabolism	Protein biosynthesis	tRNA aminoacylation, Gly	Glycyl-tRNA synthetase alpha chain (EC 6.1.1.14)	0.152145	0.101789	0.053651	0.087151	0.064999	0.077926	0.051268	0.056813	0.057364	0.052867	0.070908	0.05829048
Protein Metabolism	Protein biosynthesis	tRNA aminoacylation, Gly	Glycyl-tRNA synthetase beta chain (EC 6.1.1.14)	0.095743	0.067859	0.094528	0.072211	0.091918	0.075491	0.076541	0.090702	0.077069	0.028636	0.119112	0.081362151
Carbohydrates	Central carbohydrate metabolism	Glycolate, glyoxylate interconversions	Glyoxylate reductase (EC 1.1.1.26)	0.011837	0.002262	0.007664	0.00747	0.011818	0.010228	0.018052	0.00897	0.007663	0.004406	0.000815	0.004811528
Nucleosides and Nucleotides	Purines	Purine conversions	GMP synthase [glutamine-hydrolyzing] (EC 6.3.5.2)	0.198102	0.176435	0.148178	0.184263	0.142473	0.158775	0.1733	0.155987	0.165743	0.140978	0.164987	0.167141444
Regulation and Cell signaling	NULL	Zinc regulated enzymes	GTP cyclohydrolase I (EC 3.5.4.16) type 1	0.037253	0.047502	0.05876	0.064741	0.072221	0.057958	0.085928	0.065784	0.059991	0.022028	0.05647	0.055214257
Cofactors, Vitamins, Prosthetic Groups, Pigments	Folate and pterines	Folate Biosynthesis	GTP cyclohydrolase I (EC 3.5.4.16) type 2	0.030986	0.04524	0.020438	0.01743	0.034797	0.027761	0.028883	0.031397	0.028244	0.017622	0.001164	0.027054986
Cofactors, Vitamins, Prosthetic Groups, Pigments	Riboflavin, FMN, FAD	Riboflavin, FMN and FAD metabolism	GTP cyclohydrolase II (EC 3.5.4.25)	0.04909	0.072383	0.061315	0.077191	0.07616	0.073543	0.081596	0.075751	0.068749	0.061678	0.051231	0.066296547
Clustering-based subsystems	Nucleotidyl-phosphate metabolic cluster	CBSS-222523.1.peg.1311	GTP pyrophosphokinase	0.026808	0.018096	0.035767	0.039841	0.027575	0.025326	0.021663	0.029403	0.028901	0.013217	0.053327	0.028395904
Clustering-based subsystems	Nucleotidyl-phosphate metabolic cluster	CBSS-222523.1.peg.1311	GTP pyrophosphokinase (EC 2.7.6.5)	0.024371	0.029406	0.010219	0.02241	0.026919	0.037502	0.036826	0.030898	0.023208	0.008811	0.016766	0.024175957
Clustering-based subsystems	Probably GTP or GMP signaling related	CBSS-176299.4.peg.1292	GTP pyrophosphokinase (EC 2.7.6.5), (p)ppGpp synthetase I	0.113151	0.03393	0.040877	0.092131	0.047929	0.058445	0.062821	0.070767	0.049263	0.017622	0.137392	0.080218428
Clustering-based subsystems	Probably GTP or GMP signaling related	CBSS-176299.4.peg.1292	GTP pyrophosphokinase (EC 2.7.6.5), (p)ppGpp synthetase II	0.010097	0.009048	0.015329	0.01992	0.01707	0.020456	0.015164	0.025915	0.019048	0.004406	0.000466	0.015814941
Cofactors, Vitamins, Prosthetic Groups, Pigments	Folate and pterines	YgfZ	GTPase and tRNA-U34 5-formylation enzyme TrmE	0.082862	0.081431	0.081754	0.074701	0.047929	0.056984	0.053434	0.093692	0.063276	0.160804	0.076846	0.081874855
Protein Metabolism	Protein biosynthesis	Universal GTPases	GTP-binding and nucleic acid-binding protein YchF	0.137174	0.088217	0.06387	0.079681	0.091261	0.10861	0.109035	0.120105	0.086484	0.218076	0.096291	0.094968522
Clustering-based subsystems	NULL	CBSS-235.1.peg.567	GTP-binding protein	0.175472	0.21715	0.25548	0.186753	0.181209	0.16949	0.191352	0.214793	0.208656	0.288565	0.179075	0.242863853
Clustering-based subsystems	NULL	CBSS-290633.1.peg.1906	GTP-binding protein EngA	0.064409	0.067859	0.089418	0.104582	0.055151	0.081336	0.070042	0.079738	0.071815	0.030839	0.083599	0.089920361
Protein Metabolism	Protein biosynthesis	Universal GTPases	GTP-binding protein EngB	0.024023	0.009048	0.007664	0.00747	0.01707	0.012663	0.014442	0.018938	0.017078	0.030839	0.012458	0.014434584
Clustering-based subsystems	Fatty acid metabolic cluster	COG1399	GTP-binding protein Era	0.037601	0.040716	0.022993	0.049801	0.042676	0.048217	0.046213	0.043856	0.053642	0.013217	0.082319	0.06081456
Stress Response	NULL	Hfl operon	GTP-binding protein HflX	0.032031	0.047502	0.033212	0.044821	0.053838	0.044808	0.041881	0.041364	0.043351	0.125559	0.058683	0.039833143
Protein Metabolism	Protein biosynthesis	Universal GTPases	GTP-binding protein TypA/BipA	0.068587	0.088217	0.120076	0.064741	0.080756	0.075004	0.074375	0.098675	0.093928	0.083706	0.109331	0.072291238
Nucleosides and Nucleotides	Purines	Purine conversions	Guanine deaminase (EC 3.5.4.3)	0.018452	0.02262	0.015329	0.0249	0.016414	0.01315	0.022385	0.014452	0.02277	0.033042	0.108516	0.015144482
Clustering-based subsystems	Probably GTP or GMP signaling related	CBSS-176299.4.peg.1292	Guanosine-3',5'-bis(diphosphate) 3'-pyrophosphohydrolase (EC 3.1.7.2)	0.035512	0.013572	0.025548	0.039841	0.035454	0.044321	0.058489	0.05482	0.063932	0.01542	0.069045	0.054425482
Clustering-based subsystems	NULL	CBSS-323097.3.peg.2594	Guanylate kinase (EC 2.7.4.8)	0.163982	0.020358	0.048541	0.037351	0.033484	0.028248	0.039715	0.039869	0.038754	0.176223	0.045293	0.043895334
Phages, Prophages, Transposable elements, Plasmids	Pathogenicity islands	Staphylococcal pathogenicity islands SaPI	Heat shock protein 60 family chaperone GroEL	0.349899	0.30763	0.275918	0.286355	0.298076	0.263002	0.355265	0.274098	0.262955	0.466991	0.178726	0.23821008
Protein Metabolism	Protein folding	GroEL GroES	Heat shock protein 60 family co-chaperone GroES	0.067891	0.061074	0.071534	0.057271	0.043989	0.048217	0.057045	0.074256	0.055394	0.019825	0.017465	0.050126658
Protein Metabolism	Protein folding	Protein chaperones	Heat shock protein GrpE	0.027504	0.009048	0.015329	0.034861	0.026262	0.019969	0.016608	0.026413	0.025836	0.026433	0.032369	0.028908607
Stress Response	Heat shock	Heat shock dnaK gene cluster extended	Heat-inducible transcription repressor HrcA	0.047698	0.063336	0.025548	0.02739	0.022323	0.031171	0.036104	0.02741	0.020581	0.026433	0.012924	0.017037542
Clustering-based subsystems	NULL	Conserved gene cluster associated with Met-tRNA formyltransferase	Helicase PriA essential for oriC/DnaA-independent DNA replication	0.064061	0.101789	0.104747	0.084661	0.068282	0.090102	0.081596	0.082229	0.081667	0.068286	0.072655	0.092128932
Nitrogen Metabolism	NULL	Dissimilatory nitrite reductase	Heme d1 biosynthesis protein NirF	0.007659	0.004524	0.017884	0.00249	0.009192	0.006332	0.015886	0.004485	0.007006	0.004406	0.001514	0.009465301
Clustering-based subsystems	NULL	CBSS-316057.3.peg.563	Heme O synthase, protoheme IX farnesyltransferase (EC 2.5.1.-) COX10-CtaB	0.013578	0.031668	0.033212	0.02988	0.024293	0.025326	0.023829	0.055318	0.046855	0.057273	0.034697	0.031827075
Cofactors, Vitamins, Prosthetic Groups, Pigments	Tetrapyrroles	Heme and Siroheme Biosynthesis	HemX protein, negative effector of steady-state concentration of glutamyl-tRNA reductase	0.011489	0.006786	0.002555	0.00249	0.007879	0.011689	0.002888	0.000997	0.001314	0.002203	0.000233	0.004772089
Respiration	Electron accepting reactions	Anaerobic respiratory reductases	Heterodisulfide reductase, cytochrome reductase subunit	0.004874	0.040716	0.012774	0.02739	0.02101	0.027274	0.011553	0.004485	0.000876	0.006608	0.000349	0.012107698
Cell Wall and Capsule	Capsular and extracellular polysacchrides	Alginate metabolism	Hexuronate transporter	0.008008	0.006786	0.007664	0.00996	0.00197	0.000974	0.002166	0.000498	0.003065	0.006608	0.001397	0.007375047
Stress Response	NULL	Hfl operon	HflC protein	0.026112	0.042978	0.038322	0.034861	0.047272	0.04773	0.04838	0.039869	0.040286	0.035245	0.041101	0.032260902
Miscellaneous	Plant-Prokaryote DOE project	Scaffold proteins for [4Fe-4S] cluster assembly (MRP family)	HflK protein	0.024023	0.031668	0.045986	0.067231	0.049898	0.066237	0.047658	0.040367	0.045103	0.022028	0.027129	0.036954113
Regulation and Cell signaling	Programmed Cell Death and Toxin-antitoxin Systems	Toxin-antitoxin systems (other than RelBE and MazEF)	HigA protein (antitoxin to HigB)	0.016363	0.020358	0.017884	0.00996	0.024949	0.022404	0.031772	0.011462	0.022333	0.008811	0.018862	0.014276829
Regulation and Cell signaling	Programmed Cell Death and Toxin-antitoxin Systems	Toxin-antitoxin systems (other than RelBE and MazEF)	HigB toxin protein	0.008356	0.013572	0.010219	0.00747	0.030202	0.023378	0.015164	0.016944	0.009634	0.002203	0.000116	0.009425862
Membrane Transport	ABC transporters	ABC transporter branched-chain amino acid (TC 3.A.1.4.1)	High-affinity branched-chain amino acid transport system permease protein LivH (TC 3.A.1.4.1)	0.083906	0.117623	0.079199	0.112052	0.13722	0.153418	0.088816	0.077744	0.090425	0.050664	0.080106	0.083649599
Membrane Transport	ABC transporters	ABC transporter branched-chain amino acid (TC 3.A.1.4.1)	High-affinity leucine-specific transport system, periplasmic binding protein LivK (TC 3.A.1.4.1)	0.002785	0.013572	0.007664	0.00996	0.020353	0.013637	0.012998	0.011961	0.01248	0.013217	0.022705	0.016998103
Dormancy and Sporulation	NULL	Persister Cells	HipA protein	0.002437	0.002262	0.00511	0.00996	0.002626	0.005844	0.014442	0.006977	0.007444	0.002203	0.000815	0.002681835
Miscellaneous	Plant-Prokaryote DOE project	Experimental - Histidine Degradation	Histidine ammonia-lyase (EC 4.3.1.3)	0.058839	0.070121	0.048541	0.034861	0.054494	0.047243	0.057045	0.059305	0.076193	0.160804	0.119345	0.06113007
Amino Acids and Derivatives	Histidine Metabolism	Histidine Biosynthesis	Histidinol dehydrogenase (EC 1.1.1.23)	0.084602	0.144767	0.114966	0.129482	0.100453	0.100817	0.084484	0.093692	0.075975	0.165209	0.099784	0.098005306
Amino Acids and Derivatives	Histidine Metabolism	Histidine Biosynthesis	Histidinol-phosphatase (EC 3.1.3.15)	0.129863	0.02262	0.020438	0.01245	0.018384	0.028248	0.018052	0.014452	0.031966	0.017622	0.023636	0.042199468
Amino Acids and Derivatives	Histidine Metabolism	Histidine Biosynthesis	Histidinol-phosphate aminotransferase (EC 2.6.1.9)	0.049438	0.072383	0.056206	0.062251	0.053181	0.050165	0.043325	0.058308	0.05189	0.035245	0.055422	0.065547211
Protein Metabolism	Protein biosynthesis	tRNA aminoacylation, His	Histidyl-tRNA synthetase (EC 6.1.1.21)	0.099573	0.113099	0.091973	0.092131	0.092574	0.09546	0.094593	0.106151	0.099621	0.163006	0.11818	0.096230562
Miscellaneous	Plant-Prokaryote DOE project	At2g23840	HNH endonuclease family protein	0.021586	0.02262	0.028103	0.00996	0.024949	0.022891	0.036826	0.035384	0.021895	0.006608	0.001863	0.013133105
DNA Metabolism	DNA replication	DNA-replication	Holliday junction DNA helicase RuvA	0.050831	0.015834	0.017884	0.02241	0.022979	0.02143	0.025273	0.026911	0.03328	0.046259	0.014787	0.03072279
DNA Metabolism	DNA replication	DNA-replication	Holliday junction DNA helicase RuvB	0.091217	0.097265	0.125185	0.072211	0.108332	0.089128	0.111201	0.095685	0.081886	0.118951	0.081853	0.096072807
Clustering-based subsystems	Probably GTP or GMP signaling related	CBSS-176299.4.peg.1292	Holo-[acyl-carrier protein] synthase (EC 2.7.8.7)	0.087039	0.006786	0.007664	0.00747	0.009192	0.005844	0.006499	0.012459	0.007663	0.002203	0.005705	0.008597649
Amino Acids and Derivatives	Lysine, threonine, methionine, and cysteine	Lysine biosynthesis AAA pathway 2	Homocitrate synthase (EC 2.3.3.14)	0.009052	0.042978	0.025548	0.01494	0.024949	0.018995	0.022385	0.009967	0.004817	0.002203	0.053676	0.021296928
Amino Acids and Derivatives	Lysine, threonine, methionine, and cysteine	Methionine Biosynthesis	Homocysteine S-methyltransferase (EC 2.1.1.10)	0.005571	0.002262	0.012774	0.00249	0.003283	0.005357	0.004333	0.008472	0.003941	0.004406	0.000815	0.002918468
Miscellaneous	Plant-Prokaryote DOE project	COG3146 experimental	Homogentisate 1,2-dioxygenase (EC 1.13.11.5)	0.014623	0.018096	0.012774	0.01245	0.014444	0.014611	0.03466	0.050334	0.058678	0.006608	0.014438	0.027686006
Amino Acids and Derivatives	Lysine, threonine, methionine, and cysteine	Methionine Biosynthesis	Homoserine dehydrogenase (EC 1.1.1.3)	0.050483	0.076907	0.086863	0.057271	0.078787	0.06575	0.077985	0.06678	0.071596	0.154195	0.090818	0.079784601
Amino Acids and Derivatives	Lysine, threonine, methionine, and cysteine	Methionine Biosynthesis	Homoserine kinase (EC 2.7.1.39)	0.024023	0.01131	0.022993	0.02241	0.019697	0.038476	0.030328	0.037377	0.030871	0.024231	0.032834	0.041331815
Miscellaneous	Plant-Prokaryote DOE project	PROSC	Homoserine O-acetyltransferase (EC 2.3.1.31)	0.040038	0.042978	0.056206	0.067231	0.055151	0.072569	0.042603	0.062295	0.080353	0.046259	0.071723	0.053124003

Amino Acids and Derivatives	Lysine, threonine, methionine, and cysteine	Methionine Biosynthesis	Homoserine O-succinyltransferase (EC 2.3.1.46)	0.018104	0.029406	0.043432	0.00996	0.008535	0.014124	0.014442	0.020931	0.015545	0.028636	0.013041	0.019877133
Carbohydrates	Sugar alcohols	Mannitol Utilization	HPr kinase/phosphorylase (EC 2.7.1.-) (EC 2.7.4.-)	0.020541	0.009048	0.028103	0.00747	0.017727	0.009741	0.010109	0.023921	0.021019	0.006608	0.000699	0.014986727
Miscellaneous	Plant-Prokaryote DOE project	Scaffold proteins for [4Fe-4S] cluster assembly (MRP family)	HtrA protease/chaperone protein	0.036905	0.063336	0.06387	0.067231	0.068938	0.076465	0.059933	0.050833	0.069625	0.022028	0.090353	0.066454302
Regulation and Cell signaling	NULL	DNA-binding regulatory proteins, strays	Hydrogen peroxide-inducible genes activator	0.018104	0.013572	0.015329	0.02241	0.024293	0.024839	0.032494	0.043357	0.045541	0.041853	0.076846	0.035416002
Respiration	Electron donating reactions	Hydrogenases	hydrogenase, subunit gamma related protein	0.007659	0.020358	0.012774	0.034861	0.011161	0.013637	0.010831	0.003987	0.002189	0.002203	0.000699	0.013566932
Miscellaneous	Plant-Prokaryote DOE project	At1g52510 AT4G12830 (COG0596)	Hydrolase, alpha/beta fold family protein, in hypothetical gene cluster	0.011141	0.013572	0.007664	0.00249	0.008535	0.016072	0.009387	0.004485	0.003284	0.002203	0.000349	0.004456579
Carbohydrates	Central carbohydrate metabolism	Methylglyoxal Metabolism	Hydroxyacylglutathione hydrolase (EC 3.1.2.6)	0.010445	0.040716	0.030658	0.01494	0.023636	0.023378	0.03105	0.032393	0.038535	0.011014	0.004075	0.019916571
Cofactors, Vitamins, Prosthetic Groups, Pigments	Folate and pterines	5-FCL-like protein	Hydroxyethylthiazole kinase (EC 2.7.1.50)	0.006963	0.013572	0.00511	0.00747	0.010505	0.013637	0.006499	0.003987	0.001533	0.004406	0.001164	0.006270762
Nitrogen Metabolism	NULL	Nitrosative stress	Hydroxylamine reductase (EC 1.7.-.-)	0.014971	0.061074	0.040877	0.02739	0.10111	0.096921	0.059933	0.032393	0.004379	0.026433	0.00524	0.054543798
Amino Acids and Derivatives	Branched-chain amino acids	Branched chain amino acid degradation regulons	Hydroxymethylglutaryl-CoA lyase (EC 4.1.3.4)	0.026808	0.029406	0.05876	0.0249	0.030858	0.03945	0.044769	0.044354	0.04576	0.083706	0.060895	0.035455441
Fatty Acids, Lipids, and Isoprenoids	Isoprenoids	Isoprenoid Biosynthesis	Hydroxymethylglutaryl-CoA reductase (EC 1.1.1.34)	0.010793	0.03393	0.038322	0.02241	0.028888	0.03458	0.022385	0.0304	0.028901	0.041853	0.033882	0.040779673
Cofactors, Vitamins, Prosthetic Groups, Pigments	NULL	Thiamin biosynthesis	Hydroxymethylpyrimidine ABC transporter, substrate-binding component	0.005222	0.013572	0.020438	0.00996	0.013788	0.012176	0.009387	0.009967	0.013137	0.019825	0.006055	0.006822905
Cofactors, Vitamins, Prosthetic Groups, Pigments	NULL	Thiamin biosynthesis	Hydroxymethylpyrimidine ABC transporter, transmembrane component	0.002437	0.004524	0.00511	0.00996	0.007222	0.011202	0.011553	0.011462	0.01248	0.006608	0.002329	0.004850967
Carbohydrates	Organic acids	Glycerate metabolism	Hydroxypyruvate isomerase (EC 5.3.1.22)	0.016015	0.009048	0.030658	0.01743	0.010505	0.015585	0.018774	0.017443	0.020362	0.002203	0.001863	0.01045127
Carbohydrates	Central carbohydrate metabolism	Glycolate, glyoxylate interconversions	Hydroxypyruvate reductase (EC 1.1.1.81)	0.015319	0.027144	0.035767	0.039841	0.024949	0.031658	0.041159	0.023921	0.034156	0.011014	0.014088	0.019167235
Clustering-based subsystems	NULL	Aerotolerance operon in Bacteroides and potentially orthologous operons in other organisms	hypothetical protein PA3071	0.022282	0.020358	0.053651	0.00996	0.033484	0.031171	0.022385	0.027908	0.026493	0.103531	0.005356	0.04306712
Protein Metabolism	Protein biosynthesis	Ribosome biogenesis bacterial	hypothetical protein sometimes fused to ribosomal protein S6 glutaminyl transferase	0.014971	0.004524	0.020438	0.00498	0.007879	0.011202	0.02094	0.019436	0.012699	0.033042	0.052628	0.027370496
Protein Metabolism	Protein biosynthesis	Translation termination factors bacterial	Hypothetical protein YaeJ with similarity to translation release factor	0.008704	0.006786	0.007664	0.00996	0.010505	0.017046	0.011553	0.010964	0.018392	0.006608	0.013623	0.018417899
Miscellaneous	Plant-Prokaryote DOE project	COG2509	Hypothetical protein YbbP, contains nucleotide-binding domain of DisA bacterial checkpoint controller	0.012882	0.013572	0.020438	0.00996	0.010505	0.013637	0.014442	0.005482	0.01248	0.013217	0.000931	0.02121805
Miscellaneous	Plant-Prokaryote DOE project	COG2302	Hypothetical protein YggS, proline synthase co-transcribed bacterial homolog PROSC	0.05675	0.024882	0.061315	0.042331	0.034797	0.048217	0.042603	0.043357	0.033718	0.013217	0.04506	0.04275161
Miscellaneous	Plant-Prokaryote DOE project	At1g33290 At1g73170 At3g10420	Hypothetical protein, contains AAA+ NTPase domain and putative R3H ssDNA-binding domain	0.019149	0.036192	0.030658	0.00996	0.011161	0.00487	0.002166	0.00299	0.009196	0.01542	0.00163	0.007493363
Clustering-based subsystems	NULL	Cell division-ribosomal stress proteins cluster	Hypoxanthine-guanine phosphoribosyltransferase (EC 2.4.2.8)	0.028897	0.038454	0.022993	0.049801	0.035454	0.020943	0.018052	0.030898	0.031309	0.081503	0.079059	0.03072279
Amino Acids and Derivatives	Histidine Metabolism	Histidine Biosynthesis	Imidazole glycerol phosphate synthase amidotransferase subunit (EC 2.4.2.-)	0.02263	0.040716	0.040877	0.044821	0.049242	0.051139	0.052712	0.048341	0.041162	0.033042	0.039587	0.054740992
Amino Acids and Derivatives	Histidine Metabolism	Histidine Biosynthesis	Imidazole glycerol phosphate synthase cyclase subunit (EC 4.1.3.-)	0.060579	0.083693	0.071534	0.114542	0.071565	0.073543	0.096037	0.055816	0.062619	0.05507	0.065552	0.080888886
Amino Acids and Derivatives	Histidine Metabolism	Histidine Biosynthesis	Imidazoleglycerol-phosphate dehydratase (EC 4.2.1.19)	0.036208	0.052026	0.028103	0.059761	0.040706	0.045295	0.036104	0.038374	0.032185	0.022028	0.044245	0.029224117
Amino Acids and Derivatives	Histidine Metabolism	Histidine Degradation	Imidazolonepropionase (EC 3.5.2.7)	0.028201	0.036192	0.045986	0.02739	0.019697	0.033606	0.033938	0.034885	0.048387	0.14979	0.068114	0.044802426
Nucleosides and Nucleotides	Purines	De Novo Purine Biosynthesis	IMP cyclohydrolase (EC 3.5.4.10)	0.07555	0.047502	0.051096	0.049801	0.044646	0.036528	0.039715	0.045849	0.037878	0.01542	0.020492	0.039320439
Cell Division and Cell Cycle	NULL	YgjD and YeaZ	Inactive homolog of metal-dependent proteases, putative molecular chaperone	0.014623	0.013572	0.015329	0.01992	0.022979	0.009741	0.018052	0.011961	0.015545	0.028636	0.001863	0.020113765
Amino Acids and Derivatives	Aromatic amino acids and derivatives	Tryptophan synthesis	Indole-3-glycerol phosphate synthase (EC 4.1.1.48)	0.034816	0.047502	0.028103	0.044821	0.037424	0.037015	0.052712	0.050833	0.038973	0.044056	0.078011	0.05765946
Amino Acids and Derivatives	Aromatic amino acids and derivatives	Aromatic amino acid interconversions with aryl acids	Indolepyruvate ferredoxin oxidoreductase, alpha and beta subunits	0.027504	0.029406	0.045986	0.054781	0.027575	0.02143	0.05199	0.059803	0.079478	0.030839	0.205156	0.026976108
Amino Acids and Derivatives	Aromatic amino acids and derivatives	Aromatic amino acid interconversions with aryl acids	Indolepyruvate oxidoreductase subunit IorA (EC 1.2.7.8)	0.080076	0.126671	0.099637	0.144422	0.091918	0.105201	0.067876	0.026413	0.018173	0.002203	0.043896	0.072172922
Amino Acids and Derivatives	Aromatic amino acids and derivatives	Indole-pyruvate oxidoreductase complex	Indolepyruvate oxidoreductase subunit IorB (EC 1.2.7.8)	0.013926	0.02262	0.010219	0.00747	0.02101	0.014124	0.012998	0.004984	0.001752	0.017622	0.007335	0.016091012
Virulence, Disease and Defense	Bacteriocins, ribosomally synthesized antibacterial peptides	Tolerance to colicin E2	Inner membrane protein CreD	0.002785	0.004524	0.002555	0.00249	0.007879	0.004383	0.001444	0.002492	0.002627	0.006608	0.000466	0.0031551
Clustering-based subsystems	NULL	CTP synthase (EC 6.3.4.2) cluster	Inner membrane protein translocase component YidC, long form	0.084254	0.049764	0.045986	0.037351	0.072878	0.069647	0.068598	0.064787	0.065246	0.105734	0.082435	0.088421689
Miscellaneous	NULL	YbbK	Inner membrane protein YqiK	0.021586	0.018096	0.033212	0.01992	0.028888	0.0263	0.026717	0.03339	0.037002	0.019825	0.014321	0.037900644
Cell Wall and Capsule	Gram-Negative cell wall components	Lipopolysaccharide assembly	Inner membrane protein YrbG, predicted calcium/sodium:proton antiporter	0.036557	0.036192	0.028103	0.01245	0.01707	0.028735	0.03105	0.055318	0.042257	0.004406	0.014088	0.03707243
Miscellaneous	Plant-Prokaryote DOE project	Iojap	Inorganic pyrophosphatase (EC 3.6.1.1)	0.041083	0.038454	0.043432	0.02988	0.026919	0.030684	0.045491	0.041862	0.042476	0.028636	0.055073	0.039635949
Nucleosides and Nucleotides	Purines	Purine conversions	Inosine-5'-monophosphate dehydrogenase (EC 1.1.1.205)	0.256941	0.126671	0.163507	0.144422	0.179896	0.14806	0.157414	0.152498	0.141659	0.123356	0.135995	0.138232836
Carbohydrates	Sugar alcohols	Inositol catabolism	Inositol transport system ATP-binding protein	0.00383	0.009048	0.002555	0.00249	0.007879	0.007793	0.004333	0.007974	0.005036	0.002203	0.002445	0.005718619
Carbohydrates	Sugar alcohols	Inositol catabolism	Inositol transport system permease protein	0.002785	0.009048	0.00511	0.01494	0.000657	0.002922	0.002166	0.001993	0.002627	0.004406	0.000233	0.002090254
Carbohydrates	NULL	Sugar utilization in Thermotogales	Inosose isomerase (EC 5.3.99.-)	0.010097	0.029406	0.020438	0.01992	0.011161	0.01315	0.007221	0.013456	0.019924	0.004406	0.004774	0.011752749
Phages, Prophages, Transposable elements, Plasmids	Phages, Prophages	Phage integration and excision	Integrase	0.131952	0.162863	0.204384	0.169323	0.145099	0.131014	0.123476	0.136052	0.11429	0.114545	0.069395	0.144424721
Miscellaneous	Plant-Prokaryote DOE project	At5g48545 and At3g56490 At1g31160	Integration host factor alpha subunit	0.021238	0.009048	0.012774	0.01245	0.023636	0.023378	0.03105	0.022426	0.018392	0.008811	0.010246	0.017037542
DNA Metabolism	NULL	DNA structural proteins, bacterial	Integration host factor beta subunit	0.010445	0.015834	0.020438	0.00249	0.020353	0.0263	0.035382	0.032892	0.019486	0.006608	0.034232	0.019285551
Protein Metabolism	Protein processing and modification	Inteins	intein-containing	0.019845	0.02262	0.043432	0.0249	0.045302	0.036041	0.041881	0.032892	0.020143	0.022028	0.000582	0.019798255
Clustering-based subsystems	Isoprenoid/cell wall biosynthesis: PREDICTED UNDECAPRENYL DIPHOSPHATE PHOSPHATASE	CBSS-83331.1.peg.3039	Intramembrane protease RasP/YluC, implicated in cell division based on FtsL cleavage	0.043172	0.038454	0.033212	0.047311	0.056464	0.0526	0.068598	0.075252	0.052985	0.030839	0.060429	0.054977624
Clustering-based subsystems	NULL	CBSS-316279.3.peg.746	Iojap protein	0.020889	0.015834	0.017884	0.00747	0.020353	0.017533	0.015886	0.015948	0.02496	0.004406	0.012109	0.028356465
Stress Response	Oxidative stress	Oxidative stress	Iron-binding ferritin-like antioxidant protein	0.005919	0.002262	0.00511	0.00498	0.00197	0.011202	0.018052	0.028407	0.027587	0.011014	0.002096	0.01013576
Miscellaneous	Plant-Prokaryote DOE project	Iron-sulfur cluster assembly	Iron-sulfur cluster assembly ATPase protein SufC	0.040734	0.083693	0.040877	0.062251	0.062373	0.056984	0.057767	0.070269	0.079916	0.099125	0.043197	0.054346604
Clustering-based subsystems	Cytochrome biogenesis	CBSS-196164.1.peg.1690	Iron-sulfur cluster assembly protein SufB	0.150056	0.101789	0.097082	0.112052	0.086665	0.090102	0.132141	0.147515	0.132463	0.187237	0.113989	0.104749333
Clustering-based subsystems	Cytochrome biogenesis	CBSS-196164.1.peg.1690	Iron-sulfur cluster assembly protein SufD	0.012186	0.020358	0.020438	0.01494	0.017727	0.020456	0.026717	0.031895	0.042914	0.061678	0.01339	0.032852483
RNA Metabolism	RNA processing and modification	Wyeosine-MimG Biosynthesis	Iron-sulfur cluster assembly scaffold protein IscU	0.020541	0.03393	0.012774	0.02739	0.023636	0.021917	0.028883	0.014951	0.007225	0.011014	0.026314	0.026384527
Nitrogen Metabolism	NULL	Nitrogen fixation	Iron-sulfur cluster assembly scaffold protein NifU	0.025764	0.03393	0.028103	0.01245	0.034797	0.035554	0.021663	0.008472	0.003503	0.002203	0.046574	0.01774744
Miscellaneous	Plant-Prokaryote DOE project	Scaffold proteins for [4Fe-4S] cluster assembly (MRP family)	Iron-sulfur cluster-binding protein	0.129863	0.113099	0.114966	0.114542	0.147068	0.158288	0.122754	0.111134	0.099183	0.081503	0.106188	0.100292754
Amino Acids and Derivatives	Aromatic amino acids and derivatives	Chorismate: Intermediate for synthesis of Tryptophan, PAPA antibiotics, PABA, 3-	Isochorismatase (EC 3.3.2.1)	0.019845	0.02262	0.020438	0.01494	0.02101	0.029222	0.026717	0.020433	0.025617	0.013217	0.012226	0.018536215

		hydroxyanthranilate and more.													
Carbohydrates	Central carbohydrate metabolism	TCA Cycle	Isocitrate dehydrogenase [NAD] (EC 1.1.1.41)	0.044216	0.04524	0.030658	0.02241	0.018384	0.013637	0.025995	0.027908	0.025836	0.006608	0.021424	0.01297535
Carbohydrates	Central carbohydrate metabolism	TCA Cycle	Isocitrate dehydrogenase [NADP] (EC 1.1.1.42)	0.262859	0.133457	0.135404	0.149402	0.127372	0.157314	0.166079	0.200341	0.16837	0.257726	0.27688	0.162132722
Carbohydrates	Central carbohydrate metabolism	Glyoxylate bypass	Isocitrate dehydrogenase phosphatase (EC 2.7.11.5)/kinase (EC 3.1.3.-)	0.020889	0.009048	0.017884	0.00747	0.021666	0.016559	0.01733	0.017941	0.015764	0.004406	0.052512	0.009268107
Carbohydrates	Central carbohydrate metabolism	Glyoxylate bypass	Isocitrate lyase (EC 4.1.3.1)	0.047349	0.038454	0.030658	0.044821	0.041363	0.030196	0.047658	0.043357	0.063276	0.044056	0.229375	0.038137276
Clustering-based subsystems	Cell Division	Cell Division Cluster	Isoleucyl-tRNA synthetase (EC 6.1.1.5)	0.210984	0.30763	0.25548	0.313745	0.336813	0.29125	0.324216	0.286059	0.271275	0.277551	0.146124	0.271733022
Fatty Acids, Lipids, and Isoprenoids	Isoprenoids	polyprenyl synthesis	Isopentenyl-diphosphate delta-isomerase (EC 5.3.3.2)	0.028549	0.013572	0.012774	0.01743	0.023636	0.012663	0.016608	0.017443	0.01248	0.044056	0.003609	0.017826317
Fatty Acids, Lipids, and Isoprenoids	Isoprenoids	Archaeal lipids	Isopentenyl-diphosphate delta-isomerase, FMN-dependent (EC 5.3.3.2)	0.011141	0.020358	0.015329	0.01245	0.012475	0.006819	0.007943	0.009967	0.005912	0.002203	0.025266	0.011831626
Clustering-based subsystems	Putative Isoquinoline 1-oxidoreductase subunit	CBSS-314267.3.peg.390	Isoquinoline 1-oxidoreductase alpha subunit (EC 1.3.99.16)	0.037601	0.024882	0.038322	0.039841	0.027575	0.03945	0.029605	0.032393	0.037878	0.019825	0.004192	0.029421311
Clustering-based subsystems	Putative Isoquinoline 1-oxidoreductase subunit	CBSS-314267.3.peg.390	Isoquinoline 1-oxidoreductase beta subunit (EC 1.3.99.16)	0.077987	0.074645	0.148178	0.089641	0.091261	0.099356	0.093149	0.118111	0.112758	0.011014	0.003726	0.08617368
Amino Acids and Derivatives	Branched-chain amino acids	Leucine Degradation and HMG-CoA Metabolism	Isovaleryl-CoA dehydrogenase (EC 1.3.99.10)	0.065106	0.05655	0.076644	0.042331	0.066312	0.070621	0.063543	0.056813	0.066779	0.037447	0.044943	0.047089873
Potassium metabolism	NULL	Potassium homeostasis	KdpE	0.000696	0.006786	0.017884	0.01494	0.013131	0.003896	0.005055	0.004485	0.021019	0.008811	0.032951	0.015775502
Amino Acids and Derivatives	Proline and 4-hydroxyproline	Proline, 4-hydroxyproline uptake and utilization	Ketoglutarate semialdehyde dehydrogenase (EC 1.2.1.26)	0.003482	0.006786	0.007664	0.00996	0.001313	0.002922	0.005055	0.008472	0.013575	0.017622	0.002678	0.021730754
Amino Acids and Derivatives	Branched-chain amino acids	Branched-Chain Amino Acid Biosynthesis	Ketol-acid reductoisomerase (EC 1.1.1.86)	0.120114	0.128933	0.109856	0.151892	0.093887	0.088641	0.103258	0.084223	0.065465	0.132167	0.149268	0.106642393
Clustering-based subsystems	NULL	KH domain RNA binding protein YlqC	KH domain RNA binding protein YlqC	0.006267	0.02262	0.010219	0.02241	0.020353	0.028248	0.012275	0.007974	0.005474	0.008811	0.000349	0.011792188
Potassium metabolism	NULL	Potassium homeostasis	Kup system potassium uptake protein	0.033771	0.054288	0.033212	0.039841	0.003283	0.000487	0.002888	0.007974	0.047293	0.083706	0.002096	0.025516874
Amino Acids and Derivatives	Aromatic amino acids and derivatives	Tryptophan catabolism	Kynureninase (EC 3.7.1.3)	0.014274	0.01131	0.015329	0.01245	0.018384	0.017533	0.019496	0.039869	0.036126	0.107937	0.001514	0.012620402
Amino Acids and Derivatives	Lysine, threonine, methionine, and cysteine	Lysine Biosynthesis DAP Pathway	L,L-diaminopimelate aminotransferase (EC 2.6.1.83)	0.023327	0.027144	0.084308	0.039841	0.041363	0.061854	0.033938	0.025915	0.0208	0.022028	0.003493	0.029421311
Clustering-based subsystems	NULL	Allophanate hydrolase 2 and Biotin carboxylase cluster	Lactam utilization protein LamB	0.008356	0.01131	0.010219	0.01743	0.011161	0.008767	0.009387	0.00598	0.018829	0.004406	0.057402	0.014868411
Stress Response	Oxidative stress	Glutathione: Non-redox reactions	Lactoylglutathione lyase (EC 4.4.1.5)	0.024023	0.009048	0.020438	0.01494	0.011161	0.01802	0.026717	0.028905	0.031528	0.033042	0.020609	0.012620402
Carbohydrates	Central carbohydrate metabolism	Pyruvate Alanine Serine Interconversions	L-alanine:glyoxylate aminotransferase (EC 2.6.1.44)	0.038994	0.052026	0.022993	0.02241	0.04202	0.037502	0.047658	0.023423	0.025836	0.01542	0.08651	0.036520287
Carbohydrates	Monosaccharides	L-Arabinose utilization	L-arabinose isomerase (EC 5.3.1.4)	0.021934	0.054288	0.022993	0.032371	0.011818	0.01802	0.007943	0.017443	0.024084	0.050664	0.020027	0.0227956
Miscellaneous	Plant-Prokaryote DOE project	COG3533	L-arabinose transport ATP-binding protein AraG (TC 3.A.1.2.2)	0.00383	0.006786	0.00511	0.00996	0.004596	0.006819	0.002888	0.003987	0.005912	0.01542	0.001514	0.005876374
Potassium metabolism	NULL	Potassium homeostasis	Large-conductance mechanosensitive channel	0.015319	0.054288	0.033212	0.01992	0.008535	0.00828	0.005055	0.009967	0.014232	0.017622	0.001979	0.020153204
Amino Acids and Derivatives	Glutamine, glutamate, aspartate, asparagine; ammonia assimilation	Glutamine, Glutamate, Aspartate and Asparagine Biosynthesis	L-asparaginase (EC 3.5.1.1)	0.012534	0.029406	0.020438	0.02241	0.015757	0.018508	0.018052	0.019436	0.027587	0.002203	0.009897	0.018772847
Cofactors, Vitamins, Prosthetic Groups, Pigments	NAD and NADP	NAD and NADP cofactor biosynthesis global	L-aspartate oxidase (EC 1.4.3.16)	0.081121	0.128933	0.097082	0.124502	0.112928	0.101304	0.096759	0.095187	0.070501	0.057273	0.12016	0.112006064
Miscellaneous	Plant-Prokaryote DOE project	At4g10620 At3g57180 At3g47450	Late competence protein ComEB	0.009748	0.009048	0.00511	0.00747	0.009848	0.008767	0.00361	0.007974	0.001971	0.004406	0.000466	0.007375047
Cell Wall and Capsule	Capsular and extracellular polysacchrides	Legionaminic Acid Biosynthesis	Legionaminic acid synthase (EC 2.5.1.56)	0.002437	0.002262	0.017884	0.00498	0.01904	0.014611	0.005777	0.016446	0.003503	0.004406	0.001048	0.012620402
Amino Acids and Derivatives	Branched-chain amino acids	Leucine Degradation and HMG-CoA Metabolism	Leucine dehydrogenase (EC 1.4.1.9)	0.02263	0.004524	0.002555	0.02739	0.011818	0.019969	0.023829	0.041862	0.038535	0.022028	0.058217	0.015617747
Carbohydrates	Central carbohydrate metabolism	Dehydrogenase complexes	Leucine-, isoleucine-, valine-, threonine-, and alanine-binding protein	0.002437	0.002262	0.007664	0.00747	0.005252	0.004383	0.006499	0.011961	0.01861	0.01542	0.000466	0.009307546
Protein Metabolism	Protein degradation	Protein degradation	Leucyl/phenylalanyl-tRNA--protein transferase (EC 2.3.2.6)	0.042823	0.027144	0.010219	0.02241	0.029545	0.037502	0.03105	0.035882	0.042257	0.026433	0.036444	0.037151307
Protein Metabolism	Protein biosynthesis	tRNA aminoacylation, Leu	Leucyl-tRNA synthetase (EC 6.1.1.4)	0.259378	0.30763	0.247816	0.256474	0.309894	0.247416	0.270059	0.254662	0.235587	0.116748	0.187808	0.259073181
Carbohydrates	Monosaccharides	L-fucose utilization	L-fucose isomerase (EC 5.3.1.25)	0.018452	0.031668	0.015329	0.01743	0.009192	0.008767	0.007221	0.009469	0.007444	0.004406	0.000349	0.011831626
Metabolism of Aromatic Compounds	Peripheral pathways for catabolism of aromatic compounds	Phenylpropanoid compound degradation	Lignostilbene-alpha,beta-dioxygenase and related enzymes	0.002785	0.020358	0.00511	0.00747	0.009848	0.005357	0.002888	0.006479	0.010072	0.002203	0.000699	0.001419795
Miscellaneous	NULL	ZZ gjo need homes	Lipid A export ATP-binding/permease protein MsbA	0.01706	0.018096	0.038322	0.02988	0.029545	0.031658	0.01733	0.017443	0.035469	0.017622	0.058799	0.039202122
Cell Wall and Capsule	Gram-Negative cell wall components	Lipopolysaccharide-related cluster in Alphaproteobacteria	Lipid A export ATP-binding/permease protein MsbA (EC 3.6.3.25)	0.023327	0.031668	0.06387	0.037351	0.046615	0.0526	0.049102	0.049338	0.057364	0.081503	0.102578	0.048746301
Clustering-based subsystems	NULL	CBSS-296591.1.peg.2330	Lipid carrier : UDP-N-acetylgalactosaminyltransferase (EC 2.4.1.-)	0.016712	0.01131	0.012774	0.01494	0.015101	0.010228	0.012998	0.009967	0.009853	0.017622	0.021773	0.018299582
Cell Wall and Capsule	Gram-Negative cell wall components	KDO2-Lipid A biosynthesis	Lipid-A-disaccharide synthase (EC 2.4.1.182)	0.028549	0.036192	0.030658	0.039841	0.037424	0.03458	0.045491	0.032393	0.046855	0.022028	0.04995	0.056831246
Miscellaneous	Plant-Prokaryote DOE project	Competence or DNA damage-inducible protein CinA and related protein families	Lipoate synthase	0.244407	0.106313	0.051096	0.059761	0.057777	0.080849	0.070764	0.087711	0.089768	0.063881	0.113756	0.072448993
Cell Wall and Capsule	Gram-Negative cell wall components	Lipopolysaccharide assembly	Lipopolysaccharide ABC transporter, ATP-binding protein LptB	0.024023	0.006786	0.020438	0.01494	0.012475	0.016559	0.029605	0.013954	0.019048	0.035245	0.041683	0.021967387
Clustering-based subsystems	NULL	CBSS-211586.1.peg.3133	Lipoprotein NlpD	0.01706	0.013572	0.012774	0.00498	0.011161	0.010715	0.002166	0.011462	0.010509	0.004406	0.012458	0.020074326
Cell Wall and Capsule	Gram-Negative cell wall components	Lipopolysaccharide assembly	Lipoprotein releasing system ATP-binding protein LolD	0.054661	0.040716	0.035767	0.01743	0.033484	0.048217	0.041881	0.036879	0.038973	0.037447	0.024568	0.047287067
Cell Wall and Capsule	Gram-Negative cell wall components	Lipopolysaccharide assembly	Lipoprotein releasing system transmembrane protein LolC	0.038646	0.02262	0.022993	0.02739	0.032171	0.028735	0.03105	0.029403	0.032842	0.011014	0.013273	0.020981418
Regulation and Cell signaling	NULL	Sex pheromones in Enterococcus faecalis and other Firmicutes	Lipoprotein signal peptidase (EC 3.4.23.36)	0.020193	0.024882	0.043432	0.037351	0.01707	0.023378	0.02094	0.020433	0.024303	0.006608	0.011294	0.032536973
Carbohydrates	Fermentation	Fermentations: Lactate	L-lactate dehydrogenase (EC 1.1.1.27)	0.006267	0.009048	0.00511	0.01245	0.009848	0.0263	0.014442	0.014951	0.00832	0.011014	0.001514	0.004969283
Carbohydrates	Organic acids	Lactate utilization	L-lactate permease	0.01706	0.015834	0.002555	0.02241	0.023636	0.01315	0.021663	0.023423	0.01445	0.024231	0.01013	0.013251422
Amino Acids and Derivatives	Lysine, threonine, methionine, and cysteine	Lysine Biosynthesis DAP Pathway	LL-diaminopimelate aminotransferase, predicted alternative	0.005571	0.015834	0.025548	0.01494	0.009192	0.006819	0.010109	0.002492	0.003941	0.002203	0.000582	0.008361016
Metabolism of Aromatic Compounds	Peripheral pathways for catabolism of aromatic compounds	n-Phenylalkanoic acid degradation	Long-chain-fatty-acid--CoA ligase (EC 6.2.1.3)	0.371484	0.370965	0.475193	0.557769	0.464841	0.511392	0.451303	0.413141	0.482997	0.165209	0.206786	0.418326874
Clustering-based subsystems	NULL	LMPTP YfkJ cluster	Low molecular weight protein tyrosine phosphatase (EC 3.1.3.48)	0.001044	0.002262	0.007664	0.00996	0.009192	0.00828	0.012275	0.011462	0.010728	0.026433	0.005356	0.013409177
Amino Acids and Derivatives	Lysine, threonine, methionine, and cysteine	Threonine degradation	Low-specificity L-threonine aldolase (EC 4.1.2.5)	0.029942	0.04524	0.045986	0.052291	0.031515	0.053574	0.03105	0.040866	0.059116	0.048461	0.068347	0.033089115
Amino Acids and Derivatives	Lysine, threonine, methionine, and cysteine	Lysine degradation	L-pipecolate dehydrogenase (EC 1.5.99.3)	0.004874	0.006786	0.00511	0.00747	0.004596	0.006332	0.005777	0.004485	0.006787	0.006608	0.001979	0.002681835
Stress Response	Osmotic stress	Choline and Betaine Uptake and Betaine Biosynthesis	L-proline glycine betaine ABC transport system permease protein ProV (TC 3.A.1.12.1)	0.017408	0.018096	0.017884	0.01743	0.029545	0.042372	0.041881	0.039869	0.039629	0.052867	0.123536	0.034075084
Stress Response	Osmotic stress	Choline and Betaine Uptake and Betaine Biosynthesis	L-proline glycine betaine ABC transport system permease protein ProW (TC 3.A.1.12.1)	0.004874	0.006786	0.007664	0.01245	0.026262	0.028735	0.01733	0.0304	0.022989	0.041853	0.138207	0.024728099
Stress Response	Osmotic stress	Choline and Betaine Uptake and Betaine Biosynthesis	L-proline glycine betaine binding ABC transporter protein ProX (TC 3.A.1.12.1)	0.014274	0.01131	0.010219	0.00996	0.032171	0.038476	0.033938	0.0304	0.024522	0.024231	0.087908	0.025477436
Carbohydrates	Monosaccharides	L-Arabinose utilization	L-ribulose-5-phosphate 4-epimerase (EC 5.1.3.4)	0.014623	0.02262	0.012774	0.02241	0.009192	0.007306	0.007221	0.009469	0.007006	0.008811	0.003959	0.00918923
Amino Acids and Derivatives	Alanine, serine, and glycine	Glycine and Serine Utilization	L-serine dehydratase (EC 4.3.1.17)	0.043172	0.009048	0.015329	0.02241	0.026262	0.025813	0.018052	0.035882	0.042038	0.024231	0.106304	0.021770193

Protein Metabolism	Selenoproteins	Selenocysteine metabolism	L-seryl-tRNA(Sec) selenium transferase (EC 2.9.1.1)	0.040038	0.018096	0.043432	0.077191	0.051868	0.03945	0.032494	0.010964	0.017078	0.030839	0.002096	0.030288964
RNA Metabolism	RNA processing and modification	rRNA modification Bacteria	LSU m3Psi1915 methyltransferase RlmH	0.031682	0.02262	0.00511	0.01245	0.009848	0.012663	0.012275	0.011961	0.010072	0.035245	0.017931	0.022243458
Protein Metabolism	Protein biosynthesis	Ribosome biogenesis bacterial	LSU m5C1962 methyltransferase RlmI	0.035164	0.027144	0.012774	0.037351	0.015757	0.013637	0.015164	0.018938	0.019486	0.030839	0.033999	0.033128554
Protein Metabolism	Protein biosynthesis	Ribosome LSU bacterial	LSU ribosomal protein L10p (P0)	0.02646	0.015834	0.015329	0.02241	0.037424	0.01802	0.030328	0.036879	0.02277	0.022028	0.069162	0.037269623
Protein Metabolism	Protein biosynthesis	Ribosome LSU bacterial	LSU ribosomal protein L11p (L12e)	0.07555	0.067859	0.048541	0.044821	0.051211	0.051139	0.0556	0.056315	0.049263	0.061678	0.05845	0.047326506
Protein Metabolism	Protein biosynthesis	Ribosome LSU bacterial	LSU ribosomal protein L13p (L13Ae)	0.207502	0.047502	0.033212	0.039841	0.045302	0.03458	0.062821	0.044852	0.036126	0.059475	0.035745	0.048075842
Protein Metabolism	Protein biosynthesis	Ribosome LSU bacterial	LSU ribosomal protein L14p (L23e)	0.116285	0.065598	0.043432	0.042331	0.049242	0.046756	0.051268	0.055816	0.042038	0.074895	0.043081	0.038886612
Protein Metabolism	Protein biosynthesis	Ribosome LSU bacterial	LSU ribosomal protein L15p (L27Ae)	0.027156	0.061074	0.030658	0.01494	0.028232	0.031171	0.03466	0.038374	0.03328	0.019825	0.029458	0.036796358
Protein Metabolism	Protein biosynthesis	Ribosome LSU bacterial	LSU ribosomal protein L16p (L10e)	0.053268	0.067859	0.022993	0.032371	0.048585	0.044321	0.045491	0.048341	0.039848	0.046259	0.031204	0.044171406
Protein Metabolism	Protein biosynthesis	Ribosome LSU bacterial	LSU ribosomal protein L17p	0.237096	0.058812	0.040877	0.039841	0.051868	0.043834	0.044047	0.044852	0.028463	0.066084	0.025965	0.035021614
Protein Metabolism	Protein biosynthesis	Ribosome LSU bacterial	LSU ribosomal protein L18p (L5e)	0.042475	0.042978	0.051096	0.039841	0.033484	0.044321	0.037548	0.029403	0.026712	0.057273	0.024218	0.028119832
Protein Metabolism	Protein biosynthesis	Ribosome LSU bacterial	LSU ribosomal protein L19p	0.243711	0.036192	0.051096	0.057271	0.04202	0.048217	0.05199	0.049338	0.035688	0.017622	0.037841	0.032379218
Clustering-based subsystems	NULL	LSU ribosomal proteins cluster	LSU ribosomal protein L1p (L10Ae)	0.189398	0.070121	0.033212	0.077191	0.069595	0.067212	0.080873	0.074256	0.062619	0.099125	0.070675	0.076274552
Protein Metabolism	Protein biosynthesis	Ribosome LSU bacterial	LSU ribosomal protein L20p	0.047001	0.038454	0.020438	0.039841	0.030202	0.035067	0.041159	0.043856	0.038754	0.013217	0.022588	0.02946075
Protein Metabolism	Protein biosynthesis	Ribosome LSU bacterial	LSU ribosomal protein L21p	0.020541	0.027144	0.00511	0.0249	0.022979	0.024352	0.025273	0.018938	0.026712	0.066084	0.00652	0.034193401
Protein Metabolism	Protein biosynthesis	Ribosome LSU bacterial	LSU ribosomal protein L22p (L17e)	0.125337	0.031668	0.010219	0.01245	0.023636	0.016559	0.038993	0.039869	0.018173	0.050664	0.020958	0.02851422
Protein Metabolism	Protein biosynthesis	Ribosome LSU bacterial	LSU ribosomal protein L23p (L23Ae)	0.006267	0.002262	0.017884	0.00747	0.019697	0.025813	0.019496	0.028905	0.025398	0.017622	0.020492	0.026345088
Protein Metabolism	Protein biosynthesis	Ribosome LSU bacterial	LSU ribosomal protein L24p (L26e)	0.091217	0.024882	0.010219	0.02988	0.025606	0.023378	0.022385	0.020931	0.021676	0.008811	0.018862	0.024175957
Protein Metabolism	Protein biosynthesis	Ribosome LSU bacterial	LSU ribosomal protein L25p	0.008356	0.018096	0.025548	0.01494	0.01904	0.024839	0.019496	0.034885	0.027806	0.026433	0.016534	0.030565035
Clustering-based subsystems	NULL	CBSS-176279.3.peg.868	LSU ribosomal protein L27p	0.036557	0.04524	0.033212	0.02988	0.020353	0.022891	0.033938	0.026911	0.029996	0.017622	0.019444	0.025911262
Protein Metabolism	Protein biosynthesis	Ribosome LSU bacterial	LSU ribosomal protein L28p	0.050135	0.013572	0.00511	0.00747	0.013131	0.012176	0.012998	0.014452	0.01861	0.013217	0.022006	0.019127796
Protein Metabolism	Protein biosynthesis	Ribosome LSU bacterial	LSU ribosomal protein L29p (L35e)	0.008356	0.015834	0.007664	0.01992	0.005252	0.009741	0.010109	0.011462	0.01029	0.008811	0.000699	0.014907849
Protein Metabolism	Protein biosynthesis	Ribosome LSU bacterial	LSU ribosomal protein L2p (L8e)	0.101662	0.090479	0.086863	0.107072	0.074847	0.082797	0.080151	0.083226	0.074661	0.262132	0.041916	0.074105421
Miscellaneous	Plant-Prokaryote DOE project	COG0523	LSU ribosomal protein L31p	0.038297	0.03393	0.030658	0.02241	0.021666	0.01315	0.036826	0.028407	0.021238	0.013217	0.001048	0.020468714
Protein Metabolism	Protein biosynthesis	Ribosome LSU bacterial	LSU ribosomal protein L35p	0.018104	0.013572	0.025548	0.01245	0.011818	0.012663	0.015164	0.011961	0.013356	0.004406	0.000582	0.013251422
Protein Metabolism	Protein biosynthesis	Ribosome LSU bacterial	LSU ribosomal protein L3p (L3e)	0.104796	0.04524	0.05876	0.042331	0.058433	0.051626	0.068598	0.053325	0.053642	0.072692	0.034697	0.056870685
Protein Metabolism	Protein biosynthesis	Ribosome LSU bacterial	LSU ribosomal protein L4p (L1e)	0.06232	0.015834	0.043432	0.037351	0.032828	0.042372	0.030328	0.050334	0.047949	0.077098	0.030622	0.041686764
Protein Metabolism	Protein biosynthesis	Ribosome LSU bacterial	LSU ribosomal protein L5p (L11e)	0.123944	0.058812	0.045986	0.092131	0.066312	0.055523	0.061377	0.058807	0.046417	0.057273	0.071025	0.066059915
Protein Metabolism	Protein biosynthesis	Ribosome LSU bacterial	LSU ribosomal protein L6p (L9e)	0.113151	0.067859	0.086863	0.02739	0.057777	0.047243	0.049102	0.05482	0.044665	0.099125	0.041218	0.048549107
Clustering-based subsystems	NULL	LSU ribosomal proteins cluster	LSU ribosomal protein L7/L12 (P1/P2)	0.040734	0.047502	0.007664	0.069721	0.050555	0.045295	0.072208	0.058308	0.040505	0.037447	0.014088	0.040819111
Clustering-based subsystems	Clustering-based subsystems	CBSS-262719.3.peg.410	LSU ribosomal protein L9p	0.039342	0.049764	0.007664	0.0249	0.035454	0.04286	0.046213	0.035384	0.033061	0.046259	0.030273	0.03198483
Protein Metabolism	Protein biosynthesis	rRNAs	LSU rRNA	0.064409	0.05655	0.056206	0.032371	0.057777	0.062341	0.058489	0.063292	0.059554	0.05507	0.078476	0.069648841
Amino Acids and Derivatives	Alanine, serine, and glycine	Glycine Biosynthesis	L-threonine 3-dehydrogenase (EC 1.1.1.103)	0.014623	0.040716	0.045986	0.042331	0.037424	0.037502	0.033938	0.032393	0.03525	0.01542	0.080339	0.029263556
Amino Acids and Derivatives	Lysine, threonine, methionine, and cysteine	Lysine fermentation	Lysine 2,3-aminomutase (EC 5.4.3.2)	0.095047	0.133457	0.130295	0.159363	0.097827	0.127604	0.06571	0.075751	0.075537	0.037447	0.032019	0.0931149
Clustering-based subsystems	Lysine, threonine, methionine, and cysteine	YeiH	LysR family transcriptional regulator YeiE	0.011489	0.006786	0.010219	0.01494	0.004596	0.006332	0.00361	0.005482	0.002846	0.002203	0.000233	0.008282139
Protein Metabolism	Protein biosynthesis	tRNA aminoacylation, Lys	Lysyl-tRNA synthetase (class I) (EC 6.1.1.6)	0.004178	0.024882	0.015329	0.00996	0.019697	0.022404	0.036104	0.037377	0.024084	0.006608	0.002562	0.010096321
Protein Metabolism	Protein biosynthesis	tRNA aminoacylation, Lys	Lysyl-tRNA synthetase (class II) (EC 6.1.1.6)	0.179301	0.119885	0.150733	0.097112	0.148382	0.119812	0.132863	0.119108	0.113633	0.367866	0.162658	0.137404622
Virulence, Disease and Defense	Resistance to antibiotics and toxic compounds	Multidrug Resistance Efflux Pumps	Macrolide export ATP-binding/permease protein MacB (EC 3.6.3.-)	0.088432	0.074645	0.06387	0.062251	0.032171	0.017046	0.016608	0.014951	0.054737	0.017622	0.000349	0.048549107
Virulence, Disease and Defense	Resistance to antibiotics and toxic compounds	Multidrug Resistance Efflux Pumps	Macrolide-specific efflux protein MacA	0.028549	0.004524	0.025548	0.01494	0.004596	0.002922	0.005777	0.008472	0.01445	0.011014	0.001164	0.013843003
Virulence, Disease and Defense	Resistance to antibiotics and toxic compounds	Copper homeostasis: copper tolerance	Magnesium and cobalt efflux protein CorC	0.027504	0.040716	0.033212	0.049801	0.029545	0.035067	0.038993	0.040367	0.047074	0.004406	0.026314	0.031866514
Iron acquisition and metabolism	NULL	Campylobacter Iron Metabolism	Magnesium and cobalt transport protein CorA	0.04352	0.061074	0.061315	0.052291	0.047929	0.036528	0.036104	0.037377	0.048168	0.006608	0.000582	0.052847932
Carbohydrates	One-carbon Metabolism	Serine-glyoxylate cycle	Malate dehydrogenase (EC 1.1.1.37)	0.067543	0.081431	0.102192	0.087151	0.080756	0.090589	0.108313	0.103659	0.093709	0.105734	0.058683	0.079429653
Carbohydrates	Central carbohydrate metabolism	Glyoxylate bypass	Malate synthase (EC 2.3.3.9)	0.014971	0.015834	0.028103	0.02739	0.023636	0.018508	0.019496	0.02741	0.035907	0.041853	0.002445	0.01234433
Carbohydrates	Central carbohydrate metabolism	Glyoxylate bypass	Malate synthase G (EC 2.3.3.9)	0.027156	0.009048	0.043432	0.054781	0.034141	0.029222	0.049824	0.042859	0.044008	0.059475	0.145193	0.039478194
Fatty Acids, Lipids, and Isoprenoids	Fatty acids	Fatty Acid Biosynthesis FASII	Malonyl CoA-acyl carrier protein transacylase (EC 2.3.1.39)	0.029942	0.054288	0.056206	0.112052	0.049898	0.050165	0.072931	0.076249	0.084295	0.048461	0.054724	0.081914294
Carbohydrates	NULL	Sugar utilization in Thermotogales	Maltodextrin glucosidase (EC 3.2.1.20)	0.02263	0.040716	0.040877	0.02739	0.013131	0.015585	0.028161	0.024918	0.034375	0.044056	0.021307	0.02882973
Carbohydrates	Di- and oligosaccharides	Maltose and Maltodextrin Utilization	Malto-oligosyltrehalose synthase (EC 5.4.99.15)	0.027504	0.03393	0.040877	0.044821	0.038737	0.023865	0.012275	0.012459	0.03941	0.017622	0.000349	0.014434584
Carbohydrates	Di- and oligosaccharides	Trehalose Biosynthesis	Malto-oligosyltrehalose trehalohydrolase (EC 3.2.1.141)	0.020193	0.02262	0.022993	0.01743	0.01707	0.00828	0.010109	0.006977	0.027368	0.011014	0.000349	0.011516116
Carbohydrates	Di- and oligosaccharides	Maltose and Maltodextrin Utilization	Maltose phosphorylase (EC 2.4.1.8)	0.013578	0.009048	0.010219	0.00249	0.004596	0.009254	0.004333	0.004984	0.004817	0.008811	0.002445	0.018102389
Carbohydrates	Di- and oligosaccharides	Maltose and Maltodextrin Utilization	Maltose/maltodextrin ABC transporter, permease protein MalF	0.017756	0.013572	0.025548	0.01245	0.012475	0.017046	0.014442	0.012957	0.027587	0.017622	0.037259	0.012186575
Carbohydrates	Di- and oligosaccharides	Maltose and Maltodextrin Utilization	Maltose/maltodextrin ABC transporter, permease protein MalG	0.011141	0.015834	0.012774	0.01494	0.009848	0.015585	0.009387	0.011961	0.025617	0.017622	0.023752	0.014474023
Carbohydrates	Di- and oligosaccharides	Maltose and Maltodextrin Utilization	Maltose/maltodextrin transport ATP-binding protein MalK (EC 3.6.3.19)	0.0094	0.020358	0.017884	0.032371	0.022323	0.022891	0.02094	0.009967	0.030215	0.037447	0.022472	0.016722032
Carbohydrates	CO2 fixation	Photorespiration (oxidative C2 cycle)	Malyl-CoA lyase (EC 4.1.3.24)	0.022282	0.004524	0.015329	0.00747	0.007222	0.009254	0.009387	0.013456	0.01445	0.008811	0.001514	0.008282139
Metabolism of Aromatic Compounds	Metabolism of central aromatic intermediates	Catechol branch of beta-ketoadipate pathway	mandelate racemase/muconate lactonizing enzyme family protein	0.01323	0.029406	0.015329	0.00996	0.015757	0.018508	0.020218	0.012957	0.024303	0.008811	0.00687	0.017905195
Membrane Transport	NULL	Transport of Manganese	Manganese ABC transporter, inner membrane permease protein SitD	0.012186	0.020358	0.010219	0.00747	0.015757	0.014124	0.014442	0.017941	0.014669	0.002203	0.003842	0.005679181
Stress Response	Oxidative stress	Oxidative stress	Manganese superoxide dismutase (EC 1.15.1.1)	0.017408	0.040716	0.020438	0.02241	0.026262	0.024352	0.03105	0.038374	0.025179	0.019825	0.007685	0.027331057
Membrane Transport	NULL	Transport of Manganese	Manganese transport protein MntH	0.012186	0.040716	0.06898	0.052291	0.007879	0.010228	0.007221	0.012459	0.033937	0.129965	0.003609	0.02599014

Phosphorus Metabolism	NULL	Phosphate metabolism	Manganese-dependent inorganic pyrophosphatase (EC 3.6.1.1)	0.008704	0.006786	0.00511	0.00498	0.010505	0.009254	0.002888	0.011961	0.003941	0.004406	0.005356	0.01108229
Carbohydrates	Monosaccharides	D-Galacturonate and D-Glucuronate Utilization	Mannonate dehydratase (EC 4.2.1.8)	0.008356	0.01131	0.020438	0.01743	0.019697	0.015098	0.02094	0.010964	0.019486	0.017622	0.001281	0.012186575
Carbohydrates	Monosaccharides	Mannose Metabolism	Mannose-1-phosphate guanylyltransferase (EC 2.7.7.13)	0.003482	0.004524	0.002555	0.00498	0.004596	0.006332	0.00361	0.002492	0.005036	0.008811	0.000699	0.005994691
Carbohydrates	Monosaccharides	Mannose Metabolism	Mannose-1-phosphate guanylyltransferase (GDP) (EC 2.7.7.22)	0.057446	0.072383	0.033212	0.039841	0.052524	0.048704	0.062821	0.059803	0.054956	0.057273	0.038423	0.063496395
Carbohydrates	NULL	Sugar utilization in Thermotogales	Mannose-6-phosphate isomerase (EC 5.3.1.8)	0.024023	0.020358	0.033212	0.01743	0.030202	0.030196	0.016608	0.024918	0.023208	0.022028	0.003609	0.029657944
Protein Metabolism	Protein degradation	Aminopeptidases (EC 3.4.11.-)	Membrane alanine aminopeptidase N (EC 3.4.11.2)	0.040734	0.024882	0.048541	0.039841	0.040706	0.05601	0.054156	0.089705	0.07904	0.066084	0.151597	0.070358739
Virulence, Disease and Defense	Resistance to antibiotics and toxic compounds	Multidrug Resistance Efflux Pumps	Membrane fusion protein of RND family multidrug efflux pump	0.013578	0.024882	0.017884	0.01743	0.015757	0.015585	0.019496	0.030898	0.02693	0.004406	0.031903	0.020941979
Miscellaneous	NULL	YbbK	Membrane protease family protein BA0301	0.011141	0.015834	0.002555	0.0249	0.004596	0.005357	0.010109	0.004485	0.010728	0.074895	0.002329	0.02027152
Clustering-based subsystems	NULL	CBSS-56780.10.peg.1536	Membrane protein containing HD superfamily hydrolase domain, YQFF ortholog	0.020193	0.036192	0.035767	0.02241	0.034797	0.035554	0.049824	0.023921	0.024741	0.017622	0.002445	0.046932118
Clustering-based subsystems	Protein export?	CBSS-393121.3.peg.2760	Membrane proteins related to metalloendopeptidases	0.0094	0.020358	0.015329	0.0249	0.024949	0.0263	0.016608	0.019436	0.019486	0.002203	0.004075	0.021257489
Protein Metabolism	Protein degradation	Proteolysis in bacteria, ATP-dependent	Membrane-associated protein containing a homolog of PilT-like ATPase N-terminal domain, YACL B.subtilis ortholog	0.000696	0.024882	0.00511	0.00996	0.011818	0.006819	0.002888	0.002492	0.009853	0.013217	0.000349	0.007256731
Miscellaneous	Plant-Prokaryote DOE project	At5g37530	Membrane-bound lytic murein transglycosylase A precursor (EC 3.2.1.-)	0.004874	0.009048	0.017884	0.00996	0.008535	0.015098	0.008665	0.011961	0.011604	0.006608	0.000233	0.008952597
Cell Wall and Capsule	NULL	Murein Hydrolases	Membrane-bound lytic murein transglycosylase B precursor (EC 3.2.1.-)	0.021586	0.013572	0.028103	0.02988	0.026262	0.028248	0.032494	0.022925	0.034594	0.013217	0.022705	0.026266211
Clustering-based subsystems	NULL	CBSS-228410.1.peg.134	Membrane-bound lytic murein transglycosylase D precursor (EC 3.2.1.-)	0.036208	0.061074	0.06387	0.054781	0.053181	0.051626	0.03466	0.055816	0.047949	0.125559	0.036327	0.068860066
Cofactors, Vitamins, Prosthetic Groups, Pigments	Quinone cofactors	Menaquinone Biosynthesis via Futalosine -- gjo	Menaquinone via futalosine step 3	0.008356	0.018096	0.028103	0.01245	0.024293	0.01802	0.020218	0.011961	0.009853	0.013217	0.002794	0.017392491
Cofactors, Vitamins, Prosthetic Groups, Pigments	Quinone cofactors	Menaquinone Biosynthesis via Futalosine -- gjo	Menaquinone via futalosine step 4	0.012186	0.020358	0.033212	0.01245	0.010505	0.014611	0.014442	0.007475	0.005693	0.008811	0.001048	0.013369738
Carbohydrates	Central carbohydrate metabolism	Ethylmalonyl-CoA pathway of C2 assimilation	Mesaconyl-CoA hydratase	0.00383	0.006786	0.007664	0.00249	0.013131	0.010715	0.008665	0.021429	0.01248	0.008811	0.000466	0.005876374
Clustering-based subsystems	NULL	CBSS-56780.10.peg.1536	Metal-dependent hydrolase YbeY, involved in rRNA and/or ribosome maturation and assembly	0.083558	0.013572	0.025548	0.00996	0.012475	0.012176	0.028883	0.020931	0.017078	0.057273	0.018397	0.023347743
Virulence, Disease and Defense	Resistance to antibiotics and toxic compounds	Beta-lactamase	Metal-dependent hydrolases of the beta-lactamase superfamily I	0.004874	0.027144	0.028103	0.02241	0.015757	0.027274	0.024551	0.015449	0.013575	0.004406	0.000815	0.017629123
Clustering-based subsystems	NULL	Bacterial RNA-metabolizing Zn-dependent hydrolases	Metallo-beta-lactamase family protein, RNA-specific	0.099921	0.104051	0.081754	0.064741	0.092574	0.097408	0.093871	0.089705	0.094804	0.037447	0.024568	0.079942356
Respiration	Electron donating reactions	Respiratory dehydrogenases 1	Methanol dehydrogenase large subunit protein (EC 1.1.99.8)	0.006267	0.006786	0.002555	0.00996	0.005252	0.004383	0.007943	0.001495	0.011823	0.006608	0.002445	0.00220857
Carbohydrates	One-carbon Metabolism	One-carbon metabolism by tetrahydropterines	Methenyltetrahydrofolate cyclohydrolase (EC 3.5.4.9)	0.015667	0.042978	0.053651	0.01743	0.055151	0.033119	0.035382	0.026413	0.029996	0.044056	0.038656	0.03072279
Amino Acids and Derivatives	Lysine, threonine, methionine, and cysteine	Methionine Degradation	Methionine ABC transporter ATP-binding protein	0.043172	0.027144	0.020438	0.02241	0.017727	0.038963	0.036104	0.02442	0.057802	0.030839	0.027362	0.048904056
Protein Metabolism	Protein biosynthesis	Translation termination factors bacterial	Methionine aminopeptidase (EC 3.4.11.18)	0.029942	0.070121	0.043432	0.087151	0.084039	0.086206	0.080873	0.078741	0.061962	0.178426	0.080689	0.081835417
Amino Acids and Derivatives	Lysine, threonine, methionine, and cysteine	Methionine Degradation	Methionine gamma-lyase (EC 4.4.1.11)	0.009748	0.018096	0.00511	0.02739	0.016414	0.022891	0.02094	0.018938	0.030215	0.022028	0.011178	0.03293136
Clustering-based subsystems	tRNA sulfuration	CBSS-89187.3.peg.2957	Methionyl-tRNA formyltransferase (EC 2.1.2.9)	0.069283	0.03393	0.066425	0.034861	0.051211	0.048217	0.045491	0.058308	0.048606	0.129965	0.023403	0.053715584
Protein Metabolism	Protein biosynthesis	tRNA aminoacylation, Met	Methionyl-tRNA synthetase (EC 6.1.1.10)	0.202628	0.208102	0.163507	0.186753	0.149038	0.199199	0.194963	0.172433	0.1456	0.240104	0.116434	0.165524455
Motility and Chemotaxis	NULL	Bacterial Chemotaxis	Methyl-accepting chemotaxis protein I (serine chemoreceptor protein)	0.003133	0.01131	0.010219	0.00498	0.004596	0.011689	0.010831	0.00299	0.007663	0.03965	0.00489	0.014434584
Clustering-based subsystems	NULL	CBSS-393124.3.peg.2657	Methylated-DNA--protein-cysteine methyltransferase (EC 2.1.1.63)	0.033075	0.052026	0.020438	0.01245	0.024293	0.027274	0.03105	0.026911	0.030871	0.011014	0.004657	0.029342434
Amino Acids and Derivatives	Branched-chain amino acids	HMG CoA Synthesis	Methylcrotonyl-CoA carboxylase biotin-containing subunit (EC 6.4.1.4)	0.10584	0.027144	0.043432	0.02988	0.023636	0.022404	0.028883	0.043357	0.068092	0.070489	0.088723	0.026581721
Carbohydrates	One-carbon Metabolism	Serine-glyoxylate cycle	Methylcrotonyl-CoA carboxylase carboxyl transferase subunit (EC 6.4.1.4)	0.073113	0.072383	0.094528	0.074701	0.065656	0.069647	0.074375	0.090203	0.098526	0.28416	0.132269	0.079626846
Carbohydrates	One-carbon Metabolism	Serine-glyoxylate cycle	Methylenetetrahydrofolate dehydrogenase (NADP+) (EC 1.5.1.5)	0.027504	0.02262	0.048541	0.02241	0.036111	0.040911	0.040437	0.037377	0.021457	0.059475	0.048436	0.028908607
Cofactors, Vitamins, Prosthetic Groups, Pigments	Folate and pterines	5-FCL-like protein	Methylenetetrahydrofolate dehydrogenase (NADP+) (EC 1.5.1.5) / Methenyltetrahydrofolate cyclohydrolase (EC 3.5.4.9)	0.059187	0.018096	0.033212	0.00498	0.012475	0.014124	0.015886	0.019934	0.016202	0.066084	0.065319	0.020153204
Amino Acids and Derivatives	Branched-chain amino acids	HMG CoA Synthesis	Methylglutaconyl-CoA hydratase (EC 4.2.1.18)	0.009052	0.009048	0.012774	0.00747	0.016414	0.023378	0.026717	0.033889	0.032623	0.050664	0.012808	0.02662116
Carbohydrates	Organic acids	Methylcitrate cycle	Methylisocitrate lyase (EC 4.1.3.30)	0.010793	0.006786	0.00511	0.00249	0.007222	0.010228	0.015164	0.013456	0.029558	0.050664	0.043779	0.014513462
Amino Acids and Derivatives	Branched-chain amino acids	Valine degradation	Methylmalonate-semialdehyde dehydrogenase (EC 1.2.1.27)	0.052224	0.009048	0.043432	0.02739	0.029545	0.030684	0.038993	0.034885	0.056707	0.017622	0.185712	0.035218808
Carbohydrates	Sugar alcohols	Inositol catabolism	Methylmalonate-semialdehyde dehydrogenase [inositol] (EC 1.2.1.27)	0.005919	0.015834	0.010219	0.00498	0.007879	0.012663	0.005777	0.00598	0.007882	0.013217	0.001048	0.00855821
Respiration	Sodium Ion-Coupled Energetics	Na+ translocating decarboxylases and related biotin-dependent enzymes	Methylmalonyl-CoA decarboxylase, alpha chain (EC 4.1.1.41)	0.005919	0.024882	0.00511	0.00747	0.013131	0.010715	0.010109	0.004485	0.005036	0.004406	0.001514	0.014750094
Carbohydrates	Organic acids	Propionyl-CoA to Succinyl-CoA Module	Methylmalonyl-CoA decarboxylase, beta chain (EC 4.1.1.41)	0.006267	0.03393	0.017884	0.01245	0.021666	0.023378	0.010109	0.009967	0.005255	0.004406	0.006637	0.013172544
Carbohydrates	Organic acids	Propionyl-CoA to Succinyl-CoA Module	Methylmalonyl-CoA epimerase (EC 5.1.99.1)	0.058839	0.013572	0.015329	0.00249	0.015757	0.014611	0.010109	0.023423	0.013356	0.002203	0.000699	0.020389836
Carbohydrates	Organic acids	Propionyl-CoA to Succinyl-CoA Module	Methylmalonyl-CoA mutase (EC 5.4.99.2)	0.189398	0.25108	0.26059	0.169323	0.183179	0.204557	0.19063	0.187882	0.172311	0.484614	0.06858	0.189937044
RNA Metabolism	RNA processing and modification	tRNA modification Archaea	Methylthioadenosine deaminase	0.032379	0.04524	0.025548	0.057271	0.046615	0.046756	0.025995	0.02442	0.0208	0.011014	0.001048	0.034824421
Amino Acids and Derivatives	Lysine, threonine, methionine, and cysteine	Methionine Salvage	Methylthioribose-1-phosphate isomerase (EC 5.3.1.23)	0.071372	0.124409	0.053651	0.109562	0.108332	0.075978	0.064265	0.048839	0.050577	0.030839	0.023403	0.055293134
Fatty Acids, Lipids, and Isoprenoids	Isoprenoids	Archaeal lipids	Mevalonate kinase (EC 2.7.1.36)	0.000348	0.006786	0.010219	0.00747	0.003939	0.005357	0.005777	0.007475	0.003503	0.088112	0.004308	0.019443306
Miscellaneous	Plant-Prokaryote DOE project	At5g38900	MFS family multidrug efflux protein, similarity to bicyclomycin resistance protein Bcr	0.003133	0.009048	0.020438	0.00249	0.005252	0.002922	0.006499	0.00897	0.008101	0.01542	0.001747	0.009110352
Phages, Prophages, Transposable elements, Plasmids	Transposable elements	CBSS-203122.12.peg.188	MG(2+) CHELATASE FAMILY PROTEIN	0.04352	0.038454	0.06898	0.052291	0.047272	0.047243	0.082318	0.049836	0.048168	0.013217	0.006171	0.049337882
RNA Metabolism	RNA processing and modification	Methylthiotransferases	MiaB family protein, possibly involved in tRNA or rRNA modification	0.033423	0.040716	0.028103	0.049801	0.040706	0.044808	0.044047	0.057311	0.037221	0.070489	0.00489	0.047957526
Cofactors, Vitamins, Prosthetic Groups, Pigments	Quinone cofactors	Pyrroloquinoline Quinone biosynthesis	Microsomal dipeptidase (EC 3.4.13.19)	0.006963	0.004524	0.022993	0.00747	0.007879	0.011202	0.006499	0.007475	0.009196	0.008811	0.090818	0.010648464
Cofactors, Vitamins, Prosthetic Groups, Pigments	Folate and pterines	YgfZ	Molybdenum cofactor biosynthesis protein MoaA	0.07555	0.081431	0.084308	0.079681	0.066312	0.068186	0.060655	0.050334	0.057583	0.0793	0.059265	0.072803942
Cofactors, Vitamins, Prosthetic Groups, Pigments	Folate and pterines	Molybdenum cofactor biosynthesis	Molybdenum cofactor biosynthesis protein MoaB	0.012534	0.018096	0.022993	0.01992	0.018384	0.023378	0.022385	0.016944	0.024741	0.033042	0.01502	0.017195297
Cofactors, Vitamins, Prosthetic Groups, Pigments	Folate and pterines	Molybdenum cofactor biosynthesis	Molybdenum cofactor biosynthesis protein MoaC	0.06998	0.027144	0.028103	0.01992	0.021666	0.033119	0.03105	0.023921	0.030871	0.022028	0.020376	0.034627227
Cofactors, Vitamins, Prosthetic Groups, Pigments	Folate and pterines	Molybdenum cofactor biosynthesis	Molybdenum cofactor biosynthesis protein MoaE	0.011837	0.01131	0.022993	0.00249	0.009192	0.027274	0.010109	0.013456	0.014669	0.002203	0.007917	0.018930602
Cofactors, Vitamins, Prosthetic Groups, Pigments	Folate and pterines	Molybdenum cofactor biosynthesis	Molybdenum transport ATP-binding protein ModC (TC 3.A.1.8.1)	0.036557	0.047502	0.00511	0.032371	0.035454	0.029709	0.036826	0.033889	0.024084	0.019825	0.023403	0.036796358
Clustering-based subsystems	Molybdopterin oxidoreductase	CBSS-269799.3.peg.2220	Molybdenum transport protein, putative	0.00383	0.002262	0.002555	0.00249	0.00197	0.001948	0.00361	0.00299	0.005693	0.008811	0.00326	0.005758058
Membrane Transport	NULL	Transport of Molybdenum	Molybdenum transport system permease	0.060928	0.029406	0.015329	0.042331	0.022323	0.022891	0.040437	0.03339	0.03328	0.030839	0.047039	0.035258247

			protein ModB (TC 3.A.1.8.1)												
Cofactors, Vitamins, Prosthetic Groups, Pigments	Folate and pterines	Molybdenum cofactor biosynthesis	Molybdopterin biosynthesis Mog protein, molybdochelatase	0.002437	0.002262	0.00511	0.01494	0.001313	0.003409	0.005777	0.001993	0.003722	0.011014	0.001164	0.00698066
Cofactors, Vitamins, Prosthetic Groups, Pigments	Folate and pterines	Molybdenum cofactor biosynthesis	Molybdopterin biosynthesis protein MoeA	0.048394	0.070121	0.06898	0.102092	0.081413	0.076465	0.060655	0.043856	0.054737	0.059475	0.030971	0.078719755
Cofactors, Vitamins, Prosthetic Groups, Pigments	Folate and pterines	Molybdenum cofactor biosynthesis	Molybdopterin biosynthesis protein MoeB	0.018801	0.004524	0.015329	0.00498	0.008535	0.012176	0.012275	0.008472	0.009196	0.004406	0.008383	0.01171331
Clustering-based subsystems	Molybdopterin oxidoreductase	CBSS-269799.3.peg.2220	Molybdopterin oxidoreductase (EC 1.2.7.-)	0.037601	0.029406	0.051096	0.042331	0.043333	0.050165	0.03466	0.051331	0.064151	0.085909	0.008849	0.056436858
Cofactors, Vitamins, Prosthetic Groups, Pigments	Folate and pterines	Molybdenum cofactor biosynthesis	Molybdopterin-guanine dinucleotide biosynthesis protein MobA	0.002089	0.013572	0.015329	0.01494	0.005909	0.016559	0.018774	0.010964	0.008539	0.03965	0.002212	0.017195297
Cofactors, Vitamins, Prosthetic Groups, Pigments	Folate and pterines	Molybdenum cofactor biosynthesis	Molybdopterin-guanine dinucleotide biosynthesis protein MobB	0.012534	0.029406	0.015329	0.00996	0.02101	0.012663	0.005777	0.004984	0.007225	0.004406	0.000466	0.013882442
Clustering-based subsystems	NULL	CBSS-176280.1.peg.1561	Monofunctional biosynthetic peptidoglycan transglycosylase (EC 2.4.2.-)	0.012186	0.024882	0.033212	0.02739	0.010505	0.023865	0.016608	0.021928	0.023646	0.136573	0.033766	0.053124003
Membrane Transport	NULL	Ton and Tol transport systems	MotA/TolQ/ExbB proton channel family protein	0.059883	0.052026	0.061315	0.057271	0.086009	0.079387	0.063543	0.113128	0.094147	0.211468	0.100832	0.073908227
Clustering-based subsystems	NULL	Aerotolerance operon in Bacteroides and potentially orthologous operons in other organisms	MoxR-like ATPase in aerotolerance operon	0.081121	0.081431	0.071534	0.072211	0.046615	0.055036	0.067876	0.056315	0.066341	0.017622	0.011876	0.078798632
Miscellaneous	NULL	Muconate lactonizing enzyme family	Muconate cycloisomerase (EC 5.5.1.1)	0.011837	0.009048	0.007664	0.01494	0.013788	0.012176	0.01372	0.011462	0.013794	0.008811	0.001164	0.010214638
Virulence, Disease and Defense	Resistance to antibiotics and toxic compounds	Multidrug Resistance Efflux Pumps	Multi antimicrobial extrusion protein (Na(+)/drug antiporter), MATE family of MDR efflux pumps	0.009748	0.024882	0.012774	0.01494	0.010505	0.007793	0.006499	0.010964	0.024522	0.099125	0.003959	0.015736063
Virulence, Disease and Defense	Resistance to antibiotics and toxic compounds	Copper homeostasis	Multicopper oxidase	0.007311	0.013572	0.010219	0.01245	0.055151	0.054548	0.047658	0.044852	0.06437	0.070489	0.007801	0.053755023
Virulence, Disease and Defense	Resistance to antibiotics and toxic compounds	MexE-MexF-OprN Multidrug Efflux System	Multidrug efflux transporter MexF	0.011141	0.006786	0.012774	0.0249	0.004596	0.017533	0.008665	0.008472	0.027587	0.006608	0.0666	0.013921881
Cell Wall and Capsule	NULL	Peptidoglycan Biosynthesis	Multimodular transpeptidase-transglycosylase (EC 2.4.1.129) (EC 3.4.-.-)	0.200539	0.201316	0.160952	0.164343	0.232421	0.209914	0.164635	0.178413	0.178442	0.05507	0.158117	0.155309817
Carbohydrates	Sugar alcohols	Mannitol Utilization	Multiple polyol-specific dehydrogenase (EC 1.1.1.-)	0.001741	0.006786	0.00511	0.00747	0.009192	0.00828	0.005777	0.007974	0.009415	0.013217	0.001514	0.003391733
Carbohydrates	Di- and oligosaccharides	Fructooligosaccharides(FOS) and Raffinose Utilization	Multiple sugar ABC transporter, ATP-binding protein	0.021238	0.027144	0.010219	0.044821	0.022323	0.023378	0.009387	0.007974	0.014232	0.022028	0.004541	0.011161168
Carbohydrates	Di- and oligosaccharides	Fructooligosaccharides(FOS) and Raffinose Utilization	Multiple sugar ABC transporter, membrane-spanning permease protein MsmF	0.001741	0.002262	0.002555	0.00498	0.005909	0.005844	0.00361	0.002492	0.00416	0.006608	0.009548	0.003549488
Carbohydrates	Di- and oligosaccharides	Fructooligosaccharides(FOS) and Raffinose Utilization	Multiple sugar ABC transporter, substrate-binding protein	0.000696	0.004524	0.002555	0.00249	0.006566	0.003896	0.002888	0.002492	0.003722	0.006608	0.008383	0.002050815
Protein Metabolism	Protein degradation	Metallocarboxypeptidases (EC 3.4.17.-)	Muramoyltetrapeptide carboxypeptidase (EC 3.4.17.13)	0.003482	0.009048	0.002555	0.01245	0.012475	0.015585	0.012275	0.010964	0.012918	0.002203	0.004424	0.01932499
DNA Metabolism	DNA repair	DNA repair, bacterial MutL-MutS system	MutS-related protein, family 1	0.004178	0.009048	0.012774	0.01245	0.005252	0.003896	0.002888	0.00598	0.010072	0.050664	0.000349	0.017037542
Carbohydrates	Sugar alcohols	Inositol catabolism	Myo-inositol 2-dehydrogenase (EC 1.1.1.18)	0.013578	0.03393	0.043432	0.042331	0.038737	0.048217	0.033216	0.032892	0.058678	0.006608	0.01665	0.039241561
Carbohydrates	Sugar alcohols	Inositol catabolism	Myo-inositol 2-dehydrogenase 1 (EC 1.1.1.18)	0.006963	0.013572	0.002555	0.00996	0.005909	0.006819	0.000722	0.001993	0.004598	0.011014	0.000931	0.006073568
Membrane Transport	Uni- Sym- and Antiporters	Multi-subunit cation antiporter	Na(+) H(+) antiporter subunit A	0.002437	0.013572	0.010219	0.01743	0.024949	0.036041	0.022385	0.032892	0.038316	0.046259	0.054957	0.014040197
Membrane Transport	Uni- Sym- and Antiporters	Multi-subunit cation antiporter	Na(+) H(+) antiporter subunit B	0.00383	0.018096	0.010219	0.00996	0.007222	0.023378	0.019496	0.025416	0.027806	0.004406	0.001048	0.01869397
Membrane Transport	Uni- Sym- and Antiporters	Multi-subunit cation antiporter	Na(+) H(+) antiporter subunit C	0.00383	0.009048	0.007664	0.00249	0.013131	0.010228	0.011553	0.011961	0.015107	0.013217	0.000233	0.007020098
Membrane Transport	Uni- Sym- and Antiporters	Multi-subunit cation antiporter	Na(+) H(+) antiporter subunit D	0.010097	0.015834	0.007664	0.02988	0.027575	0.049191	0.029605	0.032892	0.03744	0.002203	0.020492	0.021296928
Membrane Transport	Uni- Sym- and Antiporters	Multi-subunit cation antiporter	Na(+) H(+) antiporter subunit E	0.004526	0.006786	0.002555	0.01494	0.007222	0.01315	0.005055	0.007475	0.009415	0.004406	0.002096	0.005639742
Respiration	Electron donating reactions	Na(+)-translocating NADH-quinone oxidoreductase and rnf-like group of electron transport complexes	Na(+)-translocating NADH-quinone reductase subunit A (EC 1.6.5.-)	0.017408	0.024882	0.020438	0.00498	0.025606	0.031658	0.021663	0.037377	0.034375	0.050664	0.070675	0.043461508
Respiration	Electron donating reactions	Na(+)-translocating NADH-quinone oxidoreductase and rnf-like group of electron transport complexes	Na(+)-translocating NADH-quinone reductase subunit B (EC 1.6.5.-)	0.031682	0.031668	0.017884	0.0249	0.042676	0.044321	0.046213	0.046348	0.054299	0.114545	0.072189	0.049890025
Respiration	Electron donating reactions	Na(+)-translocating NADH-quinone oxidoreductase and rnf-like group of electron transport complexes	Na(+)-translocating NADH-quinone reductase subunit C (EC 1.6.5.-)	0.000696	0.013572	0.020438	0.00498	0.016414	0.016072	0.01733	0.011961	0.018392	0.002203	0.001397	0.023899886
Respiration	Electron donating reactions	Na(+)-translocating NADH-quinone oxidoreductase and rnf-like group of electron transport complexes	Na(+)-translocating NADH-quinone reductase subunit D (EC 1.6.5.-)	0.073461	0.01131	0.010219	0.01992	0.037424	0.040424	0.043325	0.0304	0.02912	0.072692	0.084065	0.029815699
Respiration	Electron donating reactions	Na(+)-translocating NADH-quinone oxidoreductase and rnf-like group of electron transport complexes	Na(+)-translocating NADH-quinone reductase subunit E (EC 1.6.5.-)	0.023327	0.018096	0.017884	0.049801	0.028888	0.036528	0.047658	0.032892	0.029777	0.041853	0.059265	0.034311717
Respiration	Electron donating reactions	Na(+)-translocating NADH-quinone oxidoreductase and rnf-like group of electron transport complexes	Na(+)-translocating NADH-quinone reductase subunit F (EC 1.6.5.-)	0.058142	0.04524	0.053651	0.044821	0.036111	0.052113	0.068598	0.068275	0.058459	0.193845	0.146008	0.058093286
Miscellaneous	NULL	ZZ gjo need homes	Na+/H+ antiporter	0.216902	0.119885	0.12774	0.164343	0.161513	0.172899	0.157414	0.153495	0.180193	0.059475	0.334747	0.199717855
Membrane Transport	Uni- Sym- and Antiporters	NhaA, NhaD and Sodium-dependent phosphate transporters	Na+/H+ antiporter NhaA type	0.036905	0.015834	0.010219	0.02988	0.01904	0.016559	0.015164	0.020931	0.014669	0.046259	0.012924	0.024649222
Miscellaneous	NULL	ZZ gjo need homes	Na+/H+ antiporter NhaB	0.006615	0.002262	0.010219	0.00747	0.001313	0.000974	0.00361	0.000997	0.007006	0.024231	0.061011	0.005600303
Membrane Transport	Uni- Sym- and Antiporters	NhaA, NhaD and Sodium-dependent phosphate transporters	Na+/H+ antiporter NhaD type	0.017756	0.02262	0.035767	0.02739	0.015757	0.009254	0.010831	0.010964	0.010728	0.008811	0.044827	0.033799013
Stress Response	Oxidative stress	Glutathione analogs: mycothiol	N-acetyl-1-D-myo-inosityl-2-amino-2-deoxy-alpha-D-glucopyranoside deacetylase MshB	0.000696	0.009048	0.00511	0.00996	0.002626	0.003896	0.00361	0.001993	0.001533	0.002203	0.000233	0.001774744
Carbohydrates	Aminosugars	Chitin and N-acetylglucosamine utilization	N-Acetyl-D-glucosamine ABC transport system, permease protein 1	0.003133	0.009048	0.00511	0.00249	0.007222	0.005844	0.00361	0.002492	0.006349	0.006608	0.002445	0.004141069
Miscellaneous	Plant-Prokaryote DOE project	COG3533	N-Acetyl-D-glucosamine ABC transport system, sugar-binding protein	0.003482	0.004524	0.010219	0.00498	0.005909	0.005357	0.004333	0.008472	0.009415	0.002203	0.007917	0.004614334
Amino Acids and Derivatives	Arginine; urea cycle, polyamines	Arginine Biosynthesis extended	N-acetyl-gamma-glutamyl-phosphate reductase (EC 1.2.1.38)	0.038646	0.047502	0.056206	0.032371	0.079443	0.064776	0.057045	0.062295	0.057364	0.037447	0.092332	0.073711033
Carbohydrates	Aminosugars	Chitin and N-acetylglucosamine utilization	N-acetylglucosamine related transporter, NagX	0.004178	0.01131	0.017884	0.01245	0.003939	0.003409	0.010109	0.007974	0.009415	0.002203	0.003842	0.010175199
Cell Wall and Capsule	NULL	UDP-N-acetylmuramate from Fructose-6-phosphate Biosynthesis	N-acetylglucosamine-1-phosphate uridyltransferase (EC 2.7.7.23)	0.045609	0.047502	0.025548	0.01992	0.018384	0.025326	0.035382	0.03339	0.041381	0.048461	0.034115	0.036283655
Cell Wall and Capsule	Capsular and extracellular polysacchrides	Sialic Acid Metabolism	N-acetylglucosamine-6-phosphate deacetylase (EC 3.5.1.25)	0.000696	0.002262	0.002555	0.01245	0.006566	0.00487	0.00361	0.012459	0.01861	0.004406	0.007219	0.004732651
Amino Acids and Derivatives	Arginine; urea cycle, polyamines	Arginine Biosynthesis extended	N-acetylglutamate synthase (EC 2.3.1.1)	0.040734	0.042978	0.051096	0.044821	0.063029	0.050165	0.052712	0.031895	0.039629	0.01542	0.07836	0.064679558
Amino Acids and Derivatives	Lysine, threonine, methionine, and cysteine	Lysine Biosynthesis DAP Pathway	N-acetyl-L,L-diaminopimelate deacetylase (EC 3.5.1.47)	0.004526	0.006786	0.012774	0.00996	0.010505	0.007793	0.007943	0.006977	0.012918	0.008811	0.000233	0.014868411
Cell Wall and Capsule	NULL	Recycling of Peptidoglycan Amino Sugars	N-acetylmuramic acid 6-phosphate etherase (EC 4.2.-.-)	0.018104	0.01131	0.010219	0.00747	0.007222	0.006819	0.009387	0.012459	0.020362	0.140978	0.016534	0.018733409
Cell Wall and Capsule	NULL	Murein Hydrolases	N-acetylmuramoyl-L-alanine amidase (EC 3.5.1.28)	0.049438	0.047502	0.038322	0.02739	0.05909	0.069647	0.075819	0.071764	0.053861	0.048461	0.018746	0.063496395
Virulence, Disease and Defense	NULL	Streptococcus agalactiae virulome	N-acetylneuraminate synthase (EC 2.5.1.56)	0.022282	0.054288	0.025548	0.0249	0.092574	0.076465	0.084484	0.063292	0.031747	0.077098	0.043197	0.059789153
Cell Wall and Capsule	Capsular and extracellular polysacchrides	Sialic Acid Metabolism	N-acylglucosamine 2-epimerase (EC 5.1.3.8)	0.000696	0.01131	0.007664	0.01494	0.005909	0.007793	0.008665	0.004984	0.003284	0.011014	0.000699	0.008282139
Cofactors, Vitamins, Prosthetic Groups, Pigments	NAD and NADP	NAD and NADP cofactor biosynthesis global	NAD kinase (EC 2.7.1.23)	0.02263	0.05655	0.030658	0.01743	0.033484	0.044321	0.088094	0.055318	0.048606	0.017622	0.062758	0.043500947

Cofactors, Vitamins, Prosthetic Groups, Pigments	NAD and NADP	NAD regulation	NAD synthetase (EC 6.3.1.5)	0.094699	0.128933	0.097082	0.117032	0.098483	0.11348	0.110479	0.109639	0.083857	0.107937	0.062525	0.107983311
Miscellaneous	Plant-Prokaryote DOE project	COG2509	NAD(FAD)-utilizing dehydrogenase, sll0175 homolog	0.007311	0.018096	0.007664	0.00498	0.003283	0.004383	0.005055	0.003987	0.003722	0.006608	0.085346	0.014907849
Phosphorus Metabolism	NULL	Phosphate metabolism	NAD(P) transhydrogenase alpha subunit (EC 1.6.1.2)	0.051179	0.074645	0.06387	0.089641	0.04005	0.067699	0.078707	0.073757	0.088454	0.028636	0.005472	0.04941676
Phosphorus Metabolism	NULL	Phosphate metabolism	NAD(P) transhydrogenase subunit beta (EC 1.6.1.2)	0.102707	0.061074	0.076644	0.057271	0.074847	0.075004	0.087372	0.083725	0.07488	0.074895	0.004424	0.057698899
Clustering-based subsystems	NULL	CBSS-296591.1.peg.2330	NAD-dependent epimerase/dehydratase	0.080424	0.076907	0.125185	0.099602	0.108988	0.098382	0.094593	0.071764	0.061086	0.059475	0.038074	0.070595371
Clustering-based subsystems	NULL	CBSS-296591.1.peg.2330	NAD-dependent epimerase/dehydratase family protein	0.015319	0.002262	0.030658	0.00747	0.011818	0.011202	0.012998	0.011462	0.005255	0.004406	0.01665	0.0082427
Respiration	NULL	Formate hydrogenase	NAD-dependent formate dehydrogenase alpha subunit	0.028897	0.067859	0.038322	0.074701	0.054494	0.036041	0.039715	0.02741	0.030215	0.017622	0.115852	0.061800529
Respiration	NULL	Formate hydrogenase	NAD-dependent formate dehydrogenase beta subunit	0.016015	0.018096	0.015329	0.01245	0.011161	0.006819	0.011553	0.012957	0.009415	0.01542	0.077545	0.008992036
Respiration	NULL	Formate hydrogenase	NAD-dependent formate dehydrogenase gamma subunit	0.000348	0.02262	0.007664	0.01494	0.005252	0.004383	0.005055	0.004485	0.004598	0.011014	0.02154	0.003628365
Carbohydrates	Central carbohydrate metabolism	Glycolysis and Gluconeogenesis	NAD-dependent glyceraldehyde-3-phosphate dehydrogenase (EC 1.2.1.12)	0.139611	0.079169	0.12774	0.169323	0.1602	0.134423	0.127809	0.117613	0.123924	0.178426	0.178726	0.160515733
Carbohydrates	Central carbohydrate metabolism	Pyruvate metabolism I: anaplerotic reactions, PEP	NAD-dependent malic enzyme (EC 1.1.1.38)	0.044564	0.02262	0.048541	0.034861	0.038737	0.0263	0.03105	0.02741	0.038097	0.019825	0.041916	0.018141827
Protein Metabolism	Protein processing and modification	Protein Acetylation and Deacetylation in Bacteria	NAD-dependent protein deacetylase of SIR2 family	0.047349	0.081431	0.074089	0.052291	0.075504	0.073056	0.08304	0.078741	0.049701	0.004406	0.041916	0.053636707
Carbohydrates	CO2 fixation	Carboxysome	NADH dehydrogenase (EC 1.6.99.3)	0.046305	0.049764	0.061315	0.0249	0.043333	0.026787	0.039715	0.045351	0.06437	0.163006	0.057053	0.052019718
Nucleosides and Nucleotides	Detoxification	Nudix proteins (nucleoside triphosphate hydrolases)	NADH pyrophosphatase (EC 3.6.1.22)	0.014274	0.006786	0.002555	0.01245	0.01707	0.015098	0.019496	0.020931	0.0208	0.004406	0.026081	0.009899127
Respiration	Electron donating reactions	Respiratory Complex I	NADH ubiquinone oxidoreductase chain A (EC 1.6.5.3)	0.044216	0.018096	0.025548	0.049801	0.028888	0.01802	0.015164	0.022426	0.026055	0.024231	0.003959	0.02756769
Carbohydrates	Fermentation	Butanol Biosynthesis	NADH-dependent butanol dehydrogenase A (EC 1.1.1.-)	0.008356	0.006786	0.012774	0.01494	0.019697	0.011689	0.012998	0.00598	0.001752	0.008811	0.002096	0.032063708
Respiration	Electron donating reactions	Respiratory Complex I	NADH-ubiquinone oxidoreductase chain B (EC 1.6.5.3)	0.04352	0.076907	0.06387	0.074701	0.051868	0.051139	0.042603	0.049836	0.044884	0.026433	0.004308	0.054228288
Respiration	Electron donating reactions	Respiratory Complex I	NADH-ubiquinone oxidoreductase chain C (EC 1.6.5.3)	0.035164	0.027144	0.028103	0.037351	0.040706	0.027274	0.024551	0.028407	0.032404	0.008811	0.001863	0.036323093
Respiration	Electron donating reactions	Respiratory Complex I	NADH-ubiquinone oxidoreductase chain D (EC 1.6.5.3)	0.122552	0.147029	0.130295	0.122012	0.095201	0.080849	0.063543	0.090702	0.113414	0.14979	0.012924	0.124429272
Respiration	Electron donating reactions	Respiratory Complex I	NADH-ubiquinone oxidoreductase chain E (EC 1.6.5.3)	0.080424	0.038454	0.035767	0.042331	0.028232	0.029709	0.031772	0.027908	0.023208	0.004406	0.00163	0.024452028
Cofactors, Vitamins, Prosthetic Groups, Pigments	Folate and pterines	YgfZ	NADH-ubiquinone oxidoreductase chain F (EC 1.6.5.3)	0.104796	0.126671	0.084308	0.094622	0.087978	0.091563	0.093149	0.068275	0.084513	0.059475	0.00815	0.076629501
Respiration	Electron donating reactions	Respiratory Complex I	NADH-ubiquinone oxidoreductase chain G (EC 1.6.5.3)	0.095395	0.076907	0.102192	0.117032	0.072878	0.073056	0.073653	0.079738	0.080353	0.046259	0.009664	0.085739853
Respiration	Electron donating reactions	Respiratory Complex I	NADH-ubiquinone oxidoreductase chain H (EC 1.6.5.3)	0.139263	0.072383	0.112411	0.092131	0.074847	0.072569	0.058489	0.071266	0.061305	0.05507	0.006287	0.090314749
Respiration	Electron donating reactions	Respiratory Complex I	NADH-ubiquinone oxidoreductase chain I (EC 1.6.5.3)	0.044216	0.024882	0.06387	0.057271	0.04202	0.030684	0.030328	0.021429	0.031747	0.052867	0.004075	0.032852483
Respiration	Electron donating reactions	Respiratory Complex I	NADH-ubiquinone oxidoreductase chain J (EC 1.6.5.3)	0.022978	0.024882	0.017884	0.032371	0.024949	0.019969	0.014442	0.018439	0.027587	0.006608	0.002911	0.028869169
Respiration	Electron donating reactions	Respiratory Complex I	NADH-ubiquinone oxidoreductase chain K (EC 1.6.5.3)	0.019149	0.031668	0.00511	0.057271	0.022979	0.029222	0.025995	0.028407	0.028244	0.022028	0.003726	0.024964732
Respiration	Electron donating reactions	Respiratory Complex I	NADH-ubiquinone oxidoreductase chain L (EC 1.6.5.3)	0.180694	0.174173	0.160952	0.159363	0.161513	0.161697	0.179077	0.13057	0.128741	0.171818	0.018397	0.148526351
Respiration	Electron donating reactions	Respiratory Complex I	NADH-ubiquinone oxidoreductase chain M (EC 1.6.5.3)	0.131604	0.135719	0.13285	0.161853	0.087322	0.09546	0.095315	0.092197	0.104	0.0793	0.019095	0.11752749
Respiration	Electron donating reactions	Respiratory Complex I	NADH-ubiquinone oxidoreductase chain N (EC 1.6.5.3)	0.106536	0.079169	0.099637	0.064741	0.100453	0.080849	0.090983	0.092197	0.088017	0.112342	0.010246	0.093627604
Carbohydrates	Central carbohydrate metabolism	Pyruvate metabolism I: anaplerotic reactions, PEP	NADP-dependent malic enzyme (EC 1.1.1.40)	0.103403	0.133457	0.155843	0.126992	0.095201	0.08231	0.115533	0.146518	0.145162	0.233496	0.127379	0.126795597
RNA Metabolism	RNA processing and modification	Queuosine-Archaeosine Biosynthesis	NADPH-dependent 7-cyano-7-deazaguanine reductase (EC 1.7.1.-)	0.01706	0.024882	0.028103	0.01992	0.051868	0.026787	0.030328	0.03339	0.032842	0.019825	0.026198	0.024294273
Carbohydrates	CO2 fixation	Calvin-Benson cycle	NADPH-dependent glyceraldehyde-3-phosphate dehydrogenase (EC 1.2.1.13)	0.005919	0.01131	0.025548	0.01992	0.007879	0.014124	0.032494	0.025416	0.030653	0.019825	0.069278	0.018102389
Respiration	Electron donating reactions	Hydrogenases	NADP-reducing hydrogenase, subunit C	0.012882	0.029406	0.012774	0.02241	0.015757	0.019969	0.009387	0.005482	0.003065	0.002203	0.010246	0.010569586
Amino Acids and Derivatives	Glutamine, glutamate, aspartate, asparagine; ammonia assimilation	Glutamate dehydrogenases	NADP-specific glutamate dehydrogenase (EC 1.4.1.4)	0.100618	0.128933	0.089418	0.119522	0.126059	0.121273	0.102536	0.099174	0.11429	0.17402	0.043081	0.124705343
Respiration	Electron donating reactions	Hydrogenases	NAD-reducing hydrogenase subunit HoxE (EC 1.12.1.2)	0.00383	0.018096	0.00511	0.02241	0.013131	0.007306	0.005055	0.004984	0.000438	0.004406	0.000116	0.00918923
Respiration	Electron donating reactions	Hydrogenases	NAD-reducing hydrogenase subunit HoxF (EC 1.12.1.2)	0.125685	0.20584	0.109856	0.159363	0.107019	0.109097	0.08304	0.042361	0.019705	0.050664	0.055772	0.115240042
Respiration	Electron donating reactions	Hydrogenases	NAD-reducing hydrogenase subunit HoxH (EC 1.12.1.2)	0.03029	0.106313	0.048541	0.064741	0.025606	0.032632	0.027439	0.019436	0.008977	0.013217	0.138906	0.047168751
Respiration	Electron donating reactions	Hydrogenases	NAD-reducing hydrogenase subunit HoxY (EC 1.12.1.2)	0.017408	0.024882	0.012774	0.02241	0.006566	0.009254	0.010831	0.009469	0.002846	0.002203	0.047971	0.017629123
Amino Acids and Derivatives	Glutamine, glutamate, aspartate, asparagine; ammonia assimilation	Glutamine, Glutamate, Aspartate and Asparagine Biosynthesis	NAD-specific glutamate dehydrogenase (EC 1.4.1.2)	0.045609	0.05655	0.076644	0.072211	0.049898	0.070134	0.08665	0.113626	0.137061	0.03965	0.177911	0.068662872
Amino Acids and Derivatives	Glutamine, glutamate, aspartate, asparagine; ammonia assimilation	Glutamate dehydrogenases	NAD-specific glutamate dehydrogenase (EC 1.4.1.2), large form	0.005571	0.004524	0.012774	0.00747	0.007222	0.009741	0.01372	0.009967	0.00832	0.002203	0.006986	0.005048161
Cofactors, Vitamins, Prosthetic Groups, Pigments	Quinone cofactors	Menaquinone and Phylloquinone Biosynthesis	Naphthoate synthase (EC 4.1.3.36)	0.070676	0.031668	0.015329	0.042331	0.008535	0.014611	0.025273	0.020931	0.020581	0.05507	0.00978	0.021927948
Amino Acids and Derivatives	Arginine; urea cycle, polyamines	Polyamine Metabolism	N-carbamoylputrescine amidase (3.5.1.53)	0.029245	0.031668	0.017884	0.02241	0.013788	0.017533	0.016608	0.022925	0.027368	0.096923	0.037142	0.026818353
Carbohydrates	Di- and oligosaccharides	Maltose and Maltodextrin Utilization	Neopullulanase (EC 3.2.1.135)	0.032031	0.040716	0.030658	0.044821	0.014444	0.017533	0.021663	0.031397	0.029339	0.017622	0.007102	0.029934015
Amino Acids and Derivatives	Histidine Metabolism	Histidine Degradation	N-formylglutamate deformylase (EC 3.5.1.68)	0.001393	0.004524	0.002555	0.00996	0.003283	0.002922	0.007221	0.012957	0.008977	0.004406	0.000233	0.000867653
Stress Response	NULL	Dimethylarginine metabolism	NG,NG-dimethylarginine dimethylaminohydrolase 1 (EC 3.5.3.18)	0.006963	0.009048	0.00511	0.01245	0.004596	0.010715	0.008665	0.009469	0.010072	0.030839	0.002678	0.018417899
Cofactors, Vitamins, Prosthetic Groups, Pigments	NAD and NADP	NAD regulation	Nicotinamidase (EC 3.5.1.19)	0.018104	0.047502	0.033212	0.00996	0.029545	0.035554	0.029605	0.022426	0.021238	0.008811	0.001048	0.014237391
Cofactors, Vitamins, Prosthetic Groups, Pigments	NAD and NADP	NAD and NADP cofactor biosynthesis global	Nicotinate phosphoribosyltransferase (EC 2.4.2.11)	0.045957	0.031668	0.035767	0.034861	0.052524	0.040911	0.06932	0.050334	0.053204	0.022028	0.007335	0.026502843
Cofactors, Vitamins, Prosthetic Groups, Pigments	NAD and NADP	NAD and NADP cofactor biosynthesis global	Nicotinate-nucleotide adenylyltransferase (EC 2.7.7.18)	0.013578	0.015834	0.017884	0.02988	0.014444	0.017046	0.018774	0.023423	0.018829	0.059475	0.002212	0.019088357
Cofactors, Vitamins, Prosthetic Groups, Pigments	Tetrapyrroles	Coenzyme B12 biosynthesis	Nicotinate-nucleotide--dimethylbenzimidazole phosphoribosyltransferase (EC 2.4.2.21)	0.029593	0.029406	0.022993	0.01992	0.03808	0.035067	0.026717	0.019436	0.024741	0.01542	0.005822	0.024728099
Nitrogen Metabolism	NULL	Nitrate and nitrite ammonification	Nitrate ABC transporter, ATP-binding protein	0.005222	0.01131	0.017884	0.00498	0.006566	0.009254	0.010109	0.009967	0.019705	0.019825	0.063107	0.018812286
Nitrogen Metabolism	NULL	Nitrate and nitrite ammonification	Nitrate ABC transporter, nitrate-binding protein	0.008008	0.01131	0.035767	0.039841	0.015101	0.018995	0.009387	0.021429	0.0208	0.011014	0.098619	0.03261585
Nitrogen Metabolism	NULL	Nitrate and nitrite ammonification	Nitrate ABC transporter, permease protein	0.010793	0.004524	0.012774	0.01992	0.008535	0.006819	0.005777	0.00897	0.01445	0.013217	0.063456	0.016958665
Nitrogen Metabolism	NULL	Nitrate and nitrite ammonification	Nitrate/nitrite sensor protein (EC 2.7.3.-)	0.002089	0.006786	0.002555	0.00498	0.005252	0.002435	0.002166	0.002492	0.002408	0.002203	0.000466	0.004772089
Nitrogen Metabolism	NULL	Nitrate and nitrite ammonification	Nitrate/nitrite transporter	0.017756	0.004524	0.030658	0.01245	0.024949	0.017533	0.026717	0.02741	0.03525	0.013217	0.079059	0.014395146
Nitrogen Metabolism	NULL	Denitrification	Nitric oxide reductase activation protein NorQ	0.003482	0.002262	0.012774	0.00996	0.005909	0.004383	0.010109	0.003489	0.006131	0.004406	0.033649	0.010096321
Stress Response	NULL	Flavohaemoglobin	Nitric-oxide reductase (EC 1.7.99.7), quinol-	0.01323	0.002262	0.00511	0.00996	0.04005	0.024352	0.012998	0.010466	0.016859	0.008811	0.000466	0.016209328

			dependent												
Nitrogen Metabolism	NULL	Denitrification	Nitric-oxide reductase subunit B (EC 1.7.99.7)	0.004178	0.018096	0.010219	0.00498	0.006566	0.009254	0.007943	0.010466	0.006349	0.01542	0.120975	0.017510807
Nitrogen Metabolism	NULL	Nitrate and nitrite ammonification	Nitrite reductase [NAD(P)H] large subunit (EC 1.7.1.4)	0.035164	0.013572	0.035767	0.02988	0.032828	0.025326	0.044047	0.031397	0.053423	0.059475	0.11655	0.05671293
Nitrogen Metabolism	NULL	Ammonia assimilation	Nitrogen regulation protein NR(I)	0.153886	0.02262	0.030658	0.01743	0.031515	0.022891	0.026717	0.023921	0.055612	0.030839	0.11981	0.050481606
Nitrogen Metabolism	NULL	Ammonia assimilation	Nitrogen regulatory protein P-II	0.064757	0.061074	0.155843	0.072211	0.066312	0.064776	0.071486	0.070767	0.053423	0.044056	0.058683	0.065231701
Nitrogen Metabolism	NULL	Nitrogen fixation	Nitrogenase (molybdenum-iron) alpha chain (EC 1.18.6.1)	0.023675	0.024882	0.022993	0.032371	0.028888	0.020943	0.025273	0.018938	0.006131	0.017622	0.108051	0.044329161
Nitrogen Metabolism	NULL	Nitrogen fixation	Nitrogenase (molybdenum-iron) beta chain (EC 1.18.6.1)	0.016015	0.01131	0.028103	0.01743	0.04005	0.025326	0.015886	0.015948	0.005912	0.008811	0.165918	0.047996965
Nitrogen Metabolism	NULL	Nitrogen fixation	Nitrogenase (molybdenum-iron) reductase and maturation protein NifH	0.013578	0.004524	0.017884	0.02988	0.022979	0.016072	0.008665	0.008472	0.003503	0.01542	0.061011	0.01774744
Nitrogen Metabolism	NULL	Nitrogen fixation	Nitrogenase (molybdenum-iron)-specific transcriptional regulator NifA	0.013578	0.049764	0.053651	0.052291	0.025606	0.026787	0.030328	0.014951	0.024522	0.011014	0.000116	0.045315129
Nitrogen Metabolism	NULL	Nitrogen fixation	Nitrogenase FeMo-cofactor scaffold and assembly protein NifE	0.018801	0.03393	0.012774	0.02241	0.028232	0.017533	0.014442	0.015449	0.003941	0.019825	0.112941	0.039004929
Nitrogen Metabolism	NULL	Nitrogen fixation	Nitrogenase FeMo-cofactor scaffold and assembly protein NifN	0.008008	0.015834	0.017884	0.01245	0.02101	0.015098	0.01372	0.00897	0.004598	0.004406	0.014554	0.021691315
Nitrogen Metabolism	NULL	Nitrogen fixation	Nitrogenase FeMo-cofactor synthesis FeS core scaffold and assembly protein NifB	0.015319	0.013572	0.017884	0.01743	0.01707	0.018508	0.010831	0.013456	0.00832	0.01542	0.080339	0.026581721
Nitrogen Metabolism	NULL	Denitrification	Nitrous oxide reductase maturation protein NosF (ATPase)	0.001044	0.002262	0.002555	0.00996	0.001313	0.002435	0.00361	0.004984	0.006568	0.004406	0.000233	0.007453925
Amino Acids and Derivatives	NULL	Creatine and Creatinine Degradation	N-methylhydantoinase (ATP-hydrolyzing) (EC 3.5.2.14)	0.003133	0.01131	0.012774	0.00747	0.02101	0.036041	0.015886	0.013456	0.009634	0.006608	0.000233	0.015814941
Nucleosides and Nucleotides	NULL	Hydantoin metabolism	N-methylhydantoinase A (EC 3.5.2.14)	0.065454	0.049764	0.094528	0.049801	0.074191	0.103252	0.076541	0.043856	0.076193	0.033042	0.148337	0.055095941
Amino Acids and Derivatives	NULL	Creatine and Creatinine Degradation	N-methylhydantoinase B (EC 3.5.2.14)	0.028897	0.015834	0.06387	0.059761	0.055151	0.035554	0.03466	0.034885	0.05824	0.046259	0.100715	0.032891922
Nitrogen Metabolism	NULL	Denitrification	NnrS protein involved in response to NO	0.01323	0.004524	0.010219	0.00249	0.001313	0.00828	0.010109	0.020433	0.015983	0.004406	0.000466	0.009070914
Amino Acids and Derivatives	Proline and 4-hydroxyproline	Proline, 4-hydroxyproline uptake and utilization	Not a Proline racemase, nor 4-hydroxyproline epimerase [missing catalytic residues]	0.008704	0.009048	0.010219	0.00498	0.008535	0.013637	0.005055	0.00598	0.006131	0.002203	0.000116	0.003943875
Amino Acids and Derivatives	Lysine, threonine, methionine, and cysteine	Lysine Biosynthesis DAP Pathway	N-succinyl-L,L-diaminopimelate aminotransferase alternative (EC 2.6.1.17)	0.012186	0.024882	0.030658	0.01743	0.013788	0.014611	0.016608	0.017941	0.015983	0.017622	0.043779	0.02977626
Clustering-based subsystems	Lysine Biosynthesis	A Glutathione-dependent Thiol Reductase Associated with a Step in Lysine Biosynthesis	N-succinyl-L,L-diaminopimelate desuccinylase (EC 3.5.1.18)	0.022978	0.018096	0.022993	0.039841	0.018384	0.035067	0.03827	0.045351	0.044446	0.033042	0.06986	0.036875236
Stress Response	Heat shock	Heat shock dnaK gene cluster extended	Nucleoside 5-triphosphatase RdgB (dHAPTP, dITP, XTP-specific) (EC 3.6.1.15)	0.031682	0.067859	0.028103	0.064741	0.04005	0.048217	0.046935	0.051829	0.040286	0.05507	0.029225	0.043895334
Nucleosides and Nucleotides	Purines	Purine conversions	Nucleoside diphosphate kinase (EC 2.7.4.6)	0.027156	0.05655	0.035767	0.0249	0.038737	0.037015	0.057767	0.046348	0.048606	0.050664	0.059498	0.039162684
Carbohydrates	Monosaccharides	Deoxyribose and Deoxynucleoside Catabolism	Nucleoside permease NupC	0.0094	0.004524	0.00511	0.01245	0.009848	0.012663	0.007943	0.013456	0.028463	0.004406	0.024451	0.007966629
Nucleosides and Nucleotides	Detoxification	Nucleoside triphosphate pyrophosphohydrolase MazG	Nucleoside triphosphate pyrophosphohydrolase MazG	0.028897	0.05655	0.015329	0.054781	0.032171	0.028735	0.036104	0.045351	0.046636	0.14979	0.029225	0.047799771
Clustering-based subsystems	NULL	CBSS-296591.1.peg.2330	Nucleoside-diphosphate sugar epimerase/dehydratase	0.004526	0.006786	0.00511	0.00498	0.005252	0.014124	0.016608	0.013456	0.019705	0.008811	0.013273	0.014434584
Clustering-based subsystems	NULL	CBSS-296591.1.peg.2330	Nucleoside-diphosphate-sugar epimerases	0.016363	0.042978	0.028103	0.01992	0.029545	0.031171	0.036104	0.041862	0.044446	0.004406	0.011294	0.02027152
Nucleosides and Nucleotides	Purines	Purine conversions	Nucleotide pyrophosphatase (EC 3.6.1.9)	0.002437	0.002262	0.010219	0.00498	0.003939	0.000487	0.000722	0.004485	0.01029	0.002203	0.003959	0.002957907
Clustering-based subsystems	NULL	CBSS-296591.1.peg.2330	Nucleotide sugar epimerase	0.004526	0.015834	0.007664	0.00996	0.010505	0.009741	0.011553	0.009469	0.012699	0.017622	0.010828	0.0082427
Phages, Prophages, Transposable elements, Plasmids	Phages, Prophages	Phage regulation of gene expression	Nudix hydrolase	0.015319	0.031668	0.015329	0.01743	0.01707	0.016559	0.018052	0.023921	0.032623	0.026433	0.000699	0.032497534
Amino Acids and Derivatives	Lysine, threonine, methionine, and cysteine	Methionine Biosynthesis	O-acetylhomoserine sulfhydrylase (EC 2.5.1.49)	0.060231	0.042978	0.051096	0.044821	0.05909	0.073543	0.056323	0.081731	0.08101	0.063881	0.091401	0.062037162
Cell Wall and Capsule	Gram-Negative cell wall components	Perosamine Synthesis Vibrio	O-antigen export system, permease protein	0.020193	0.03393	0.025548	0.044821	0.01707	0.010228	0.01372	0.006479	0.020362	0.024231	0.003726	0.025359119
Respiration	Electron accepting reactions	Tetrathionate respiration	Octaheme tetrathionate reductase	0.022978	0.054288	0.028103	0.042331	0.049898	0.041885	0.036104	0.019934	0.006349	0.002203	0.000466	0.029342434
Cofactors, Vitamins, Prosthetic Groups, Pigments	Lipoic acid	Lipoic acid metabolism	Octanoate-[acyl-carrier-protein]-protein-N-octanoyltransferase	0.033771	0.015834	0.05876	0.032371	0.029545	0.029222	0.051268	0.051829	0.030215	0.033042	0.03656	0.029381872
Photosynthesis	NULL	Proteorhodopsin	Octaprenyl-diphosphate synthase (EC 2.5.1.-)	0.027156	0.029406	0.038322	0.032371	0.028888	0.022404	0.016608	0.031397	0.028244	0.013217	0.016883	0.030959423
Protein Metabolism	Protein degradation	Protein degradation	Oligopeptidase A (EC 3.4.24.70)	0.035164	0.01131	0.025548	0.034861	0.024949	0.019482	0.026717	0.042859	0.059116	0.026433	0.132152	0.053045125
Membrane Transport	ABC transporters	ABC transporter oligopeptide (TC 3.A.1.5.1)	Oligopeptide ABC transporter, periplasmic oligopeptide-binding protein OppA (TC 3.A.1.5.1)	0.027156	0.042978	0.061315	0.034861	0.045959	0.041885	0.039715	0.051829	0.032842	0.017622	0.020725	0.028632536
Membrane Transport	ABC transporters	ABC transporter oligopeptide (TC 3.A.1.5.1)	Oligopeptide transport ATP-binding protein OppD (TC 3.A.1.5.1)	0.121159	0.095003	0.102192	0.124502	0.122776	0.138806	0.099648	0.073757	0.100497	0.041853	0.009315	0.081598784
Membrane Transport	ABC transporters	ABC transporter oligopeptide (TC 3.A.1.5.1)	Oligopeptide transport ATP-binding protein OppF (TC 3.A.1.5.1)	0.09261	0.126671	0.099637	0.112052	0.118837	0.125656	0.088816	0.053325	0.07269	0.063881	0.008267	0.066888128
Membrane Transport	ABC transporters	ABC transporter oligopeptide (TC 3.A.1.5.1)	Oligopeptide transport system permease protein OppB (TC 3.A.1.5.1)	0.087736	0.029406	0.061315	0.067231	0.070251	0.063802	0.070042	0.074754	0.088017	0.063881	0.14007	0.060853999
Membrane Transport	ABC transporters	ABC transporter oligopeptide (TC 3.A.1.5.1)	Oligopeptide transport system permease protein OppC (TC 3.A.1.5.1)	0.028549	0.015834	0.015329	0.01992	0.028232	0.030196	0.023829	0.029403	0.036564	0.008811	0.006637	0.025083048
Miscellaneous	Plant-Prokaryote DOE project	At1g52510 AT4G12830 (COG0596)	omega-3 polyunsaturated fatty acid synthase subunit, PfaA	0.021586	0.024882	0.017884	0.01245	0.04005	0.04286	0.029605	0.018439	0.012699	0.004406	0.000466	0.022401213
Miscellaneous	Plant-Prokaryote DOE project	At1g52510 AT4G12830 (COG0596)	omega-3 polyunsaturated fatty acid synthase subunit, PfaC	0.004874	0.020358	0.002555	0.00498	0.029545	0.029709	0.023829	0.011961	0.005474	0.006608	0.000349	0.010845658
Clustering-based subsystems	Putrescine/GABA utilization cluster-temporal,to add to SSs	GABA and putrescine metabolism from cluters	Omega-amino acid--pyruvate aminotransferase (EC 2.6.1.18)	0.04352	0.020358	0.007664	0.01245	0.009848	0.020456	0.025995	0.018938	0.036564	0.037447	0.098387	0.018023511
Clustering-based subsystems	NULL	EC49-61	Organic solvent tolerance protein precursor	0.004874	0.006786	0.00511	0.00498	0.012475	0.015098	0.010109	0.022925	0.02277	0.004406	0.00652	0.01329086
Miscellaneous	Plant-Prokaryote DOE project	PROSC	Ornithine aminotransferase (EC 2.6.1.13)	0.041431	0.052026	0.025548	0.02241	0.020353	0.033119	0.028161	0.033889	0.038097	0.05507	0.007917	0.042869927
Amino Acids and Derivatives	Arginine; urea cycle, polyamines	Arginine Biosynthesis extended	Ornithine carbamoyltransferase (EC 2.1.3.3)	0.165723	0.122147	0.137959	0.097112	0.104392	0.082797	0.105424	0.074256	0.074004	0.081503	0.095126	0.110152443
Miscellaneous	Plant-Prokaryote DOE project	PROSC	Ornithine cyclodeaminase (EC 4.3.1.12)	0.017408	0.020358	0.040877	0.00996	0.050555	0.037502	0.028161	0.03339	0.026274	0.011014	0.091633	0.015538869
Cofactors, Vitamins, Prosthetic Groups, Pigments	Folate and pterines	YgfZ	Orotate phosphoribosyltransferase (EC 2.4.2.10)	0.079032	0.042978	0.045986	0.02988	0.041363	0.058445	0.041159	0.037377	0.051452	0.099125	0.047039	0.048115281
Nucleosides and Nucleotides	Pyrimidines	De Novo Pyrimidine Synthesis	Orotidine 5'-phosphate decarboxylase (EC 4.1.1.23)	0.016363	0.027144	0.035767	0.01245	0.022323	0.018995	0.025995	0.021429	0.017297	0.013217	0.021075	0.028040955
Potassium metabolism	NULL	Potassium homeostasis	Osmosensitive K+ channel histidine kinase KdpD (EC 2.7.3.-)	0.004874	0.013572	0.015329	0.01992	0.015757	0.015098	0.008665	0.011462	0.03941	0.022028	0.002562	0.023623814
Amino Acids and Derivatives	Lysine, threonine, methionine, and cysteine	Methionine Biosynthesis	O-succinylhomoserine sulfhydrylase (EC 2.5.1.48)	0.050483	0.065598	0.061315	0.044821	0.057777	0.044321	0.06571	0.049338	0.058459	0.0793	0.067415	0.068820627
Membrane Transport	NULL	Ton and Tol transport systems	Outer membrane lipoprotein omp16 precursor	0.068935	0.002262	0.00511	0.00747	0.009192	0.011202	0.014442	0.012459	0.020581	0.028636	0.015253	0.029263556
Cell Wall and Capsule	Gram-Negative cell wall components	Outer membrane	Outer membrane protein assembly factor YaeT precursor	0.034816	0.070121	0.056206	0.049801	0.066312	0.068673	0.064988	0.092197	0.08101	0.013217	0.025732	0.088342811
Cell Wall and Capsule	Gram-Negative cell wall components	Lipopolysaccharide assembly	Outer membrane protein Imp, required for envelope biogenesis	0.003482	0.002262	0.007664	0.01494	0.011818	0.005357	0.00361	0.007974	0.012261	0.006608	0.000931	0.005363671
Clustering-based subsystems	NULL	CBSS-290633.1.peg.1906	Outer membrane protein YfgL, lipoprotein component of the protein assembly complex	0.006615	0.01131	0.010219	0.00996	0.013788	0.007793	0.010831	0.012459	0.01445	0.01542	0.011178	0.004890406

			(forms a complex with YaeT, YfiO, and NlpB)												
Stress Response	Periplasmic Stress	Periplasmic Stress Response	Outer membrane stress sensor protease DegS	0.008008	0.009048	0.010219	0.01992	0.007222	0.013637	0.007943	0.007974	0.013575	0.002203	0.036793	0.014828972
Cofactors, Vitamins, Prosthetic Groups, Pigments	Tetrapyrroles	Coenzyme B12 biosynthesis	Outer membrane vitamin B12 receptor BtuB	0.022282	0.013572	0.017884	0.00747	0.020353	0.015585	0.023829	0.025416	0.013575	0.033042	0.003493	0.020074326
Carbohydrates	Central carbohydrate metabolism	Pyruvate metabolism I: anaplerotic reactions, PEP	Oxaloacetate decarboxylase alpha chain (EC 4.1.1.3)	0.004874	0.004524	0.010219	0.00996	0.007879	0.012663	0.015164	0.021928	0.032623	0.017622	0.128077	0.028987485
Carbohydrates	Central carbohydrate metabolism	Pyruvate metabolism I: anaplerotic reactions, PEP	Oxaloacetate decarboxylase beta chain (EC 4.1.1.3)	0.018452	0.063336	0.020438	0.02739	0.028232	0.037015	0.04838	0.026413	0.03109	0.008811	0.069045	0.040661356
Cell Wall and Capsule	Capsular and extracellular polysacchrides	Capsular Polysaccharides Biosynthesis and Assembly	Oxidoreductase, short-chain dehydrogenase/reductase family (EC 1.1.1.-)	0.004526	0.004524	0.007664	0.00498	0.013131	0.009741	0.015886	0.019436	0.024522	0.002203	0.022588	0.007335608
Clustering-based subsystems	Shiga toxin cluster	CBSS-194948.1.peg.143	P protein	0.087736	0.156077	0.160952	0.164343	0.147068	0.118351	0.109035	0.135554	0.135528	0.136573	0.190602	0.126598403
Miscellaneous	Plant-Prokaryote DOE project	Scaffold proteins for [4Fe-4S] cluster assembly (MRP family)	PaaD-like protein (DUF59) involved in Fe-S cluster assembly	0.019497	0.015834	0.00511	0.00498	0.011161	0.006819	0.015886	0.019934	0.024522	0.03965	0.010363	0.01743193
Cofactors, Vitamins, Prosthetic Groups, Pigments	Coenzyme A	Coenzyme A Biosynthesis	Pantoate--beta-alanine ligase (EC 6.3.2.1)	0.072765	0.04524	0.074089	0.089641	0.064999	0.037989	0.062099	0.059803	0.03941	0.030839	0.033649	0.057422827
Cofactors, Vitamins, Prosthetic Groups, Pigments	Coenzyme A	Coenzyme A Biosynthesis	Pantothenate kinase type III, CoaX-like (EC 2.7.1.33)	0.033771	0.036192	0.033212	0.042331	0.028888	0.031171	0.03466	0.016446	0.02693	0.024231	0.007102	0.029736821
Cofactors, Vitamins, Prosthetic Groups, Pigments	Coenzyme A	Coenzyme A Biosynthesis	Pantothenate:Na+ symporter (TC 2.A.21.1.1)	0.005222	0.009048	0.010219	0.00747	0.007879	0.008767	0.002888	0.007974	0.014669	0.002203	0.132385	0.010096321
Amino Acids and Derivatives	Aromatic amino acids and derivatives	Chorismate: Intermediate for synthesis of Tryptophan, PAPA antibiotics, PABA, 3-hydroxyanthranilate and more.	Para-aminobenzoate synthase, amidotransferase component (EC 2.6.1.85)	0.033423	0.031668	0.028103	0.01743	0.017727	0.018508	0.020218	0.025915	0.024084	0.019825	0.008965	0.023899886
Cofactors, Vitamins, Prosthetic Groups, Pigments	Folate and pterines	Folate Biosynthesis	Para-aminobenzoate synthase, aminase component (EC 2.6.1.85)	0.022282	0.04524	0.025548	0.054781	0.036111	0.037989	0.029605	0.038374	0.037659	0.063881	0.042731	0.051349259
Iron acquisition and metabolism	NULL	Heme, hemin uptake and utilization systems in GramNegatives	Paraquat-inducible protein A	0.011837	0.015834	0.022993	0.00498	0.011818	0.015098	0.014442	0.010466	0.01861	0.002203	0.017116	0.006941221
Iron acquisition and metabolism	NULL	Heme, hemin uptake and utilization systems in GramNegatives	Paraquat-inducible protein B	0.009052	0.029406	0.028103	0.01743	0.010505	0.018995	0.006499	0.004984	0.012918	0.006608	0.017931	0.01045127
Clustering-based subsystems	Isoprenoid/cell wall biosynthesis: PREDICTED UNDECAPRENYL DIPHOSPHATE PHOSPHATASE	CBSS-83331.1.peg.3039	Penicillin-binding protein 2 (PBP-2)	0.037949	0.047502	0.056206	0.047311	0.039393	0.058445	0.070042	0.071266	0.065903	0.136573	0.06008	0.06302313
Membrane Transport	Protein secretion system, Type II	Predicted secretion system W clustering with cell division proteins	Peptidase M23B precursor	0.006963	0.006786	0.010219	0.00249	0.010505	0.014611	0.005777	0.015948	0.013794	0.008811	0.000466	0.016012135
Virulence, Disease and Defense	Resistance to antibiotics and toxic compounds	BlaR1 Family Regulatory Sensor-transducer Disambiguation	Peptidase M48, Ste24p precursor	0.025764	0.006786	0.022993	0.02739	0.02101	0.009741	0.016608	0.011961	0.008539	0.002203	0.001048	0.015144482
Miscellaneous	Plant-Prokaryote DOE project	At5g04520 AT1G06240	Peptidase, M23/M37 family	0.027504	0.01131	0.022993	0.01494	0.024949	0.030684	0.030328	0.050334	0.048606	0.006608	0.027595	0.048351914
Miscellaneous	Plant-Prokaryote DOE project	At2g23840	Peptide chain release factor 1	0.119418	0.088217	0.099637	0.092131	0.105049	0.083284	0.095315	0.112131	0.089549	0.125559	0.08977	0.094731889
Protein Metabolism	Protein biosynthesis	Translation termination factors bacterial	Peptide chain release factor 2	0.073461	0.092741	0.079199	0.074701	0.083383	0.066237	0.074375	0.06379	0.05824	0.107937	0.081271	0.065626088
Protein Metabolism	Protein biosynthesis	Translation termination factors bacterial	Peptide chain release factor 3	0.084951	0.065598	0.107302	0.082171	0.060403	0.070621	0.075097	0.095685	0.104876	0.26874	0.155672	0.085700415
Miscellaneous	Plant-Prokaryote DOE project	At5g63420	Peptide deformylase (EC 3.5.1.88)	0.03029	0.038454	0.040877	0.094622	0.049898	0.054061	0.062099	0.069272	0.06021	0.028636	0.042964	0.056555175
Clustering-based subsystems	Carbohydrates	Putative sugar ABC transporter (ytf cluster)	Peptide methionine sulfoxide reductase MsrA (EC 1.8.4.11)	0.062668	0.097265	0.076644	0.099602	0.05909	0.06088	0.084484	0.0912	0.075318	0.068286	0.030389	0.074539247
Miscellaneous	Plant-Prokaryote DOE project	At5g04520 AT1G06240	Peptide methionine sulfoxide reductase MsrB (EC 1.8.4.12)	0.056402	0.074645	0.05876	0.047311	0.062373	0.061854	0.06932	0.076249	0.054518	0.024231	0.045642	0.047760332
Membrane Transport	NULL	Ton and Tol transport systems	Peptidoglycan-associated lipoprotein precursor	0.009748	0.013572	0.015329	0.01245	0.024293	0.020456	0.033938	0.025416	0.015764	0.002203	0.011876	0.014276829
RNA Metabolism	RNA processing and modification	Queuosine-Archaeosine Biosynthesis	Peptidyl-prolyl cis-trans isomerase (EC 5.2.1.8)	0.018801	0.036192	0.028103	0.034861	0.032828	0.036041	0.041881	0.0304	0.025617	0.019825	0.004075	0.029934015
Protein Metabolism	Protein folding	Peptidyl-prolyl cis-trans isomerase	Peptidyl-prolyl cis-trans isomerase ppiA precursor (EC 5.2.1.8)	0.025764	0.004524	0.015329	0.00996	0.011161	0.014124	0.012998	0.014951	0.016859	0.004406	0.013623	0.010885096
RNA Metabolism	RNA processing and modification	Queuosine-Archaeosine Biosynthesis	Peptidyl-prolyl cis-trans isomerase ppiB (EC 5.2.1.8)	0.03029	0.020358	0.017884	0.00747	0.014444	0.020943	0.011553	0.019934	0.019705	0.013217	0.044827	0.021691315
Clustering-based subsystems	NULL	Cell division-ribosomal stress proteins cluster	Peptidyl-tRNA hydrolase (EC 3.1.1.29)	0.043868	0.040716	0.040877	0.02739	0.034797	0.038476	0.025995	0.048341	0.02912	0.024231	0.0248	0.036007583
Respiration	Electron donating reactions	Hydrogenases	Periplasmic [Fe] hydrogenase (EC 1.12.7.2)	0.011837	0.01131	0.007664	0.00996	0.004596	0.009741	0.007943	0.008472	0.004379	0.008811	0.001514	0.010293515
Carbohydrates	Di- and oligosaccharides	Beta-Glucoside Metabolism	Periplasmic beta-glucosidase (EC 3.2.1.21)	0.020541	0.027144	0.017884	0.01992	0.011161	0.015098	0.016608	0.019436	0.040067	0.017622	0.018746	0.023623814
Cofactors, Vitamins, Prosthetic Groups, Pigments	Folate and pterines	Molybdenum cofactor biosynthesis	Periplasmic molybdate-binding domain	0.011489	0.020358	0.025548	0.01992	0.022323	0.015098	0.01372	0.006977	0.005036	0.01542	0.024218	0.009583617
Nitrogen Metabolism	NULL	Nitrate and nitrite ammonification	Periplasmic nitrate reductase precursor (EC 1.7.99.4)	0.018104	0.009048	0.012774	0.039841	0.076817	0.055523	0.042603	0.041364	0.021238	0.008811	0.013972	0.0475237
Miscellaneous	Plant-Prokaryote DOE project	DMT transporter	Permease of the drug/metabolite transporter (DMT) superfamily	0.051876	0.027144	0.028103	0.044821	0.053838	0.060393	0.05199	0.055318	0.067874	0.070489	0.098852	0.106248006
Secondary Metabolism	Plant Hormones	Auxin degradation	Peroxidase (EC 1.11.1.7)	0.21551	0.131195	0.140514	0.131972	0.09717	0.091563	0.078707	0.094688	0.102686	0.05507	0.053909	0.088421689
Stress Response	Oxidative stress	Oxidative stress	Peroxide stress regulator	0.012882	0.006786	0.002555	0.00747	0.011161	0.020456	0.008665	0.003987	0.000657	0.002203	0.000466	0.002405764
Miscellaneous	Plant-Prokaryote DOE project	COG0398	PF00070 family, FAD-dependent NAD(P)-disulphide oxidoreductase	0.004526	0.009048	0.00511	0.00498	0.026262	0.031171	0.027439	0.031397	0.030434	0.008811	0.023636	0.017786878
Phages, Prophages, Transposable elements, Plasmids	Phages, Prophages	Phage integration and excision	Phage integrase	0.029942	0.047502	0.097082	0.032371	0.026262	0.036041	0.033216	0.044354	0.031747	0.046259	0.002678	0.047129312
Phages, Prophages, Transposable elements, Plasmids	Phages, Prophages	r1t-like streptococcal phages	Phage protein	0.121507	0.027144	0.035767	0.01992	0.474033	0.52162	0.627491	0.785914	0.033718	0.030839	0.011411	0.044920742
Stress Response	NULL	Phage shock protein (psp) operon	Phage shock protein A	0.003482	0.004524	0.002555	0.00249	0.015101	0.019969	0.012275	0.009967	0.013356	0.008811	0.00815	0.004456579
Phages, Prophages, Transposable elements, Plasmids	Phages, Prophages	r1t-like streptococcal phages	Phage terminase	0.000696	0.009048	0.012774	0.00747	0.216663	0.20894	0.320605	0.397692	0.019705	0.017622	0.003144	0.017550246
Secondary Metabolism	Bacterial cytostatics, differentiation factors and antibiotics	Phenazine biosynthesis	Phenazine biosynthesis protein PhzF	0.001741	0.009048	0.012774	0.00747	0.016414	0.011689	0.007943	0.006479	0.010947	0.004406	0.000466	0.009228669
Metabolism of Aromatic Compounds	NULL	Phenylacetyl-CoA catabolic pathway (core)	Phenylacetate degradation enoyl-CoA hydratase PaaB (EC 4.2.1.17)	0.004874	0.01131	0.00511	0.00249	0.005909	0.002922	0.007943	0.006977	0.00832	0.004406	0.025615	0.00603413
Metabolism of Aromatic Compounds	NULL	Phenylacetyl-CoA catabolic pathway (core)	Phenylacetate-CoA oxygenase, PaaG subunit	0.01323	0.006786	0.007664	0.01245	0.005252	0.006332	0.011553	0.019436	0.0208	0.052867	0.064854	0.011437239
Amino Acids and Derivatives	Aromatic amino acids and derivatives	Aromatic amino acid interconversions with aryl acids	Phenylacetate-coenzyme A ligase (EC 6.2.1.30)	0.037253	0.092741	0.051096	0.047311	0.03808	0.06916	0.053434	0.0304	0.027149	0.050664	0.030622	0.049298444
Metabolism of Aromatic Compounds	NULL	Phenylacetyl-CoA catabolic pathway (core)	Phenylacetic acid degradation protein PaaN, ring-opening aldehyde dehydrogenase (EC 1.2.1.3)	0.011837	0.006786	0.010219	0.00747	0.00197	0.012663	0.01372	0.029902	0.029558	0.004406	0.007917	0.007493363
Miscellaneous	Plant-Prokaryote DOE project	COG3146 experimental	Phenylalanine-4-hydroxylase (EC 1.14.16.1)	0.007311	0.009048	0.002555	0.00498	0.004596	0.005357	0.010831	0.009967	0.017516	0.019825	0.007568	0.01360637
Protein Metabolism	Protein biosynthesis	tRNA aminoacylation, Phe	Phenylalanyl-tRNA synthetase alpha chain (EC 6.1.1.20)	0.133693	0.099527	0.071534	0.094622	0.102423	0.106662	0.124198	0.094688	0.080572	0.191643	0.077428	0.166392107
Protein Metabolism	Protein biosynthesis	tRNA aminoacylation, Phe	Phenylalanyl-tRNA synthetase beta chain (EC 6.1.1.20)	0.147271	0.131195	0.178836	0.074701	0.158886	0.13491	0.153804	0.183895	0.147789	0.290768	0.113989	0.17262343
Metabolism of Aromatic Compounds	Peripheral pathways for catabolism of aromatic compounds	Phenylpropanoid compound degradation	Phenylpropionate dioxygenase and related ring-hydroxylating dioxygenases, large terminal subunit	0.006963	0.02262	0.012774	0.01992	0.009848	0.008767	0.012275	0.016446	0.019924	0.004406	0.068812	0.01076678
Phosphorus Metabolism	NULL	P uptake (cyanobacteria)	Phosphate ABC transporter, periplasmic phosphate-binding protein PstS (TC 3.A.1.7.1)	0.055357	0.117623	0.097082	0.074701	0.107019	0.097408	0.097481	0.075252	0.071377	0.085909	0.062292	0.080573376
Carbohydrates	Sugar alcohols	Propanediol utilization	Phosphate acetyltransferase (EC 2.3.1.8)	0.040734	0.063336	0.061315	0.039841	0.03808	0.040424	0.036104	0.040367	0.031966	0.01542	0.110729	0.075446338
Carbohydrates	Fermentation	Acetyl-CoA fermentation to Butyrate	Phosphate butyryltransferase (EC 2.3.1.19)	0.00383	0.013572	0.010219	0.00747	0.002626	0.001948	0.006499	0.000997	0.001533	0.008811	0.000466	0.005324232

Phosphorus Metabolism	NULL	High affinity phosphate transporter and control of PHO regulon	Phosphate regulon sensor protein PhoR (SphS) (EC 2.7.13.3)	0.040386	0.047502	0.038322	0.069721	0.056464	0.058932	0.049824	0.040866	0.048825	0.011014	0.032834	0.045078497
Clustering-based subsystems	Phosphate metabolism	PhoR-PhoB two-component regulatory system	Phosphate regulon transcriptional regulatory protein PhoB (SphR)	0.074854	0.099527	0.045986	0.084661	0.070908	0.072082	0.077263	0.052328	0.070501	0.050664	0.044128	0.0671642
Clustering-based subsystems	NULL	tRNA-methylthiotransferase containing cluster	Phosphate starvation-inducible ATPase PhoH with RNA binding motif	0.023675	0.006786	0.020438	0.037351	0.019697	0.01802	0.025995	0.029403	0.02496	0.030839	0.031321	0.020310959
Phosphorus Metabolism	NULL	P uptake (cyanobacteria)	Phosphate starvation-inducible protein PhoH	0.003133	0.004524	0.00511	0.00249	0.008535	0.003896	0.006499	0.005482	0.005036	0.033042	0.001164	0.005718619
Clustering-based subsystems	NULL	CBSS-56780.10.peg.1536	Phosphate starvation-inducible protein PhoH, predicted ATPase	0.024023	0.047502	0.038322	0.057271	0.047929	0.058445	0.05199	0.043357	0.032185	0.116748	0.007335	0.036362532
Phosphorus Metabolism	NULL	High affinity phosphate transporter and control of PHO regulon	Phosphate transport ATP-binding protein PstB (TC 3.A.1.7.1)	0.172338	0.117623	0.12263	0.164343	0.120806	0.097408	0.085206	0.093692	0.079916	0.074895	0.085695	0.099977244
Phosphorus Metabolism	NULL	High affinity phosphate transporter and control of PHO regulon	Phosphate transport system permease protein PstA (TC 3.A.1.7.1)	0.081817	0.05655	0.081754	0.097112	0.085352	0.083771	0.073653	0.08522	0.07072	0.05507	0.063922	0.070437616
Phosphorus Metabolism	NULL	P uptake (cyanobacteria)	Phosphate transport system permease protein PstC (TC 3.A.1.7.1)	0.078684	0.088217	0.094528	0.077191	0.10111	0.084745	0.094593	0.072761	0.087798	0.096923	0.066134	0.073553278
Phosphorus Metabolism	NULL	Phosphate metabolism	Phosphate transport system regulatory protein PhoU	0.030638	0.029406	0.040877	0.042331	0.057777	0.059419	0.036826	0.040367	0.033937	0.046259	0.033533	0.030762229
Fatty Acids, Lipids, and Isoprenoids	Phospholipids	Glycerolipid and Glycerophospholipid Metabolism in Bacteria	Phosphate:acyl-ACP acyltransferase PlsX	0.040386	0.052026	0.045986	0.034861	0.044646	0.045782	0.042603	0.047344	0.056926	0.050664	0.026896	0.04626166
Clustering-based subsystems	Hypothetical lipase related to Phosphatidate metabolism	CBSS-316407.3.peg.1371	Phosphatidate cytidylyltransferase (EC 2.7.7.41)	0.01706	0.024882	0.053651	0.02241	0.038737	0.029222	0.031772	0.037875	0.031747	0.013217	0.03691	0.029855138
Fatty Acids, Lipids, and Isoprenoids	Phospholipids	Glycerolipid and Glycerophospholipid Metabolism in Bacteria	Phosphatidylserine decarboxylase (EC 4.1.1.65)	0.019497	0.009048	0.015329	0.01245	0.009192	0.02143	0.014442	0.020433	0.015545	0.011014	0.005472	0.020784224
Amino Acids and Derivatives	Lysine, threonine, methionine, and cysteine	Cysteine Biosynthesis	Phosphoadenylyl-sulfate reductase [thioredoxin] (EC 1.8.4.8)	0.000348	0.006786	0.010219	0.034861	0.012475	0.011202	0.016608	0.022925	0.022989	0.011014	0.018862	0.024215396
Miscellaneous	NULL	ZZ gjo need homes	Phosphocarrier protein kinase/phosphorylase, nitrogen regulation associated	0.021586	0.049764	0.025548	0.042331	0.027575	0.050165	0.047658	0.052328	0.044665	0.03965	0.057518	0.033089115
Carbohydrates	Central carbohydrate metabolism	Pyruvate metabolism I: anaplerotic reactions, PEP	Phosphoenolpyruvate carboxykinase [ATP] (EC 4.1.1.49)	0.058839	0.05655	0.061315	0.069721	0.06106	0.053087	0.090983	0.090702	0.118888	0.204859	0.188623	0.09883352
Carbohydrates	Central carbohydrate metabolism	Pyruvate metabolism I: anaplerotic reactions, PEP	Phosphoenolpyruvate carboxykinase [GTP] (EC 4.1.1.32)	0.028897	0.095003	0.086863	0.107072	0.107019	0.092051	0.056323	0.046348	0.02912	0.028636	0.00163	0.048351914
Carbohydrates	Fermentation	Fermentations: Mixed acid	Phosphoenolpyruvate carboxylase (EC 4.1.1.31)	0.054661	0.05655	0.074089	0.02739	0.060403	0.065263	0.051268	0.076249	0.083419	0.114545	0.10025	0.095875613
Phosphorus Metabolism	NULL	Phosphoenolpyruvate phosphomutase	Phosphoenolpyruvate phosphomutase (EC 5.4.2.9)	0.008008	0.002262	0.015329	0.00747	0.013788	0.010715	0.005777	0.008472	0.008977	0.004406	0.000233	0.002681835
Carbohydrates	Central carbohydrate metabolism	Glycolysis and Gluconeogenesis, including Archaeal enzymes	Phosphoenolpyruvate synthase (EC 2.7.9.2)	0.120114	0.131195	0.099637	0.196713	0.194997	0.129553	0.142973	0.123593	0.137936	0.072692	0.169644	0.109087596
Membrane Transport	Sugar Phosphotransferase Systems, PTS	Fructose and Mannose Inducible PTS	Phosphoenolpyruvate-protein phosphotransferase of PTS system (EC 2.7.3.9)	0.057794	0.063336	0.066425	0.082171	0.082726	0.08231	0.070042	0.056813	0.052547	0.037447	0.03854	0.051349259
Clustering-based subsystems	Chromosome Replication	SeqA and Co-occurring Genes	Phosphoglucomutase (EC 5.4.2.2)	0.049438	0.049764	0.066425	0.067231	0.031515	0.045782	0.041159	0.040866	0.051452	0.074895	0.040868	0.079784601
Carbohydrates	Central carbohydrate metabolism	Entner-Doudoroff Pathway	Phosphogluconate dehydratase (EC 4.2.1.12)	0.016712	0.004524	0.007664	0.00747	0.011818	0.010228	0.011553	0.019436	0.038316	0.030839	0.123653	0.01265984
Cell Wall and Capsule	Capsular and extracellular polysacchrides	Sialic Acid Metabolism	Phosphoglucosamine mutase (EC 5.4.2.10)	0.164331	0.074645	0.089418	0.092131	0.074191	0.073543	0.085928	0.083226	0.085608	0.121153	0.096989	0.090709136
Carbohydrates	Central carbohydrate metabolism	Glycolysis and Gluconeogenesis, including Archaeal enzymes	Phosphoglycerate kinase (EC 2.7.2.3)	0.115588	0.115361	0.099637	0.161853	0.110958	0.129553	0.116256	0.113128	0.097869	0.242307	0.12668	0.116028817
Carbohydrates	Central carbohydrate metabolism	Glycolysis and Gluconeogenesis, including Archaeal enzymes	Phosphoglycerate mutase (EC 5.4.2.1)	0.020193	0.052026	0.038322	0.0249	0.024293	0.024352	0.024551	0.018439	0.022114	0.022028	0.002562	0.018023511
Carbohydrates	Central carbohydrate metabolism	Glycolate, glyoxylate interconversions	Phosphoglycolate phosphatase (EC 3.1.3.18)	0.024371	0.020358	0.040877	0.00747	0.013788	0.014124	0.01372	0.017443	0.013575	0.006608	0.000466	0.017826317
Clustering-based subsystems	NULL	CBSS-160492.1.peg.550	Phosphoheptose isomerase (EC 5.3.1.-)	0.01706	0.013572	0.033212	0.01494	0.011818	0.018995	0.022385	0.017941	0.015983	0.01542	0.025266	0.014828972
Protein Metabolism	Protein processing and modification	N-linked Glycosylation in Bacteria	Phospholipid-lipopolysaccharide ABC transporter	0.005222	0.002262	0.00511	0.01494	0.004596	0.010228	0.008665	0.00897	0.010728	0.019825	0.001048	0.01774744
Cell Wall and Capsule	Gram-Negative cell wall components	Perosamine Synthesis Vibrio	Phosphomannomutase (EC 5.4.2.8)	0.124292	0.090479	0.130295	0.119522	0.087322	0.079875	0.085206	0.090203	0.096556	0.085909	0.146823	0.098044745
Cofactors, Vitamins, Prosthetic Groups, Pigments	NULL	Thiamin biosynthesis	Phosphomethylpyrimidine kinase (EC 2.7.4.7)	0.019845	0.01131	0.015329	0.032371	0.027575	0.033119	0.028883	0.029902	0.022333	0.013217	0.014438	0.017274175
Cell Wall and Capsule	NULL	Peptidoglycan Biosynthesis	Phospho-N-acetylmuramoyl-pentapeptide-transferase (EC 2.7.8.13)	0.06615	0.085955	0.071534	0.079681	0.089948	0.080362	0.093149	0.095187	0.073128	0.121153	0.047505	0.091695105
Membrane Transport	ABC transporters	ABC transporter alkylphosphonate (TC 3.A.1.9.1)	Phosphonate ABC transporter ATP-binding protein (TC 3.A.1.9.1)	0.009052	0.02262	0.020438	0.00498	0.019697	0.029222	0.027439	0.029902	0.023646	0.004406	0.040985	0.007966629
Membrane Transport	ABC transporters	ABC transporter alkylphosphonate (TC 3.A.1.9.1)	Phosphonate ABC transporter phosphate-binding periplasmic component (TC 3.A.1.9.1)	0.007311	0.013572	0.017884	0.00747	0.028888	0.026787	0.036826	0.034387	0.031747	0.004406	0.079757	0.013133105
Clustering-based subsystems	NULL	CBSS-269801.1.peg.1715	Phosphopantetheine adenylyltransferase (EC 2.7.7.3)	0.050831	0.038454	0.035767	0.01992	0.053181	0.040911	0.046935	0.054321	0.03941	0.026433	0.02678	0.038728857
Cofactors, Vitamins, Prosthetic Groups, Pigments	Coenzyme A	Coenzyme A Biosynthesis	Phosphopantothenoylcysteine decarboxylase (EC 4.1.1.36)	0.030638	0.054288	0.020438	0.037351	0.024949	0.027761	0.035382	0.033889	0.025836	0.099125	0.028177	0.032182024
Cofactors, Vitamins, Prosthetic Groups, Pigments	Coenzyme A	Coenzyme A Biosynthesis	Phosphopantothenoylcysteine synthetase (EC 6.3.2.5)	0.022978	0.029406	0.038322	0.067231	0.03808	0.038476	0.029605	0.038374	0.039848	0.066084	0.030389	0.037940082
Clustering-based subsystems	NULL	Nucleoside Catabolism cluster	Phosphopentomutase (EC 5.4.2.7)	0.035164	0.027144	0.017884	0.01494	0.014444	0.018995	0.023107	0.028905	0.031528	0.022028	0.022472	0.015026166
Nucleosides and Nucleotides	Purines	De Novo Purine Biosynthesis	Phosphoribosylamine--glycine ligase (EC 6.3.4.13)	0.140308	0.088217	0.12263	0.107072	0.12015	0.075978	0.119866	0.108144	0.094366	0.121153	0.092914	0.11117785
Nucleosides and Nucleotides	Purines	De Novo Purine Biosynthesis	Phosphoribosylaminoimidazole carboxylase ATPase subunit (EC 4.1.1.21)	0.032379	0.018096	0.017884	0.01245	0.02101	0.024352	0.033938	0.042361	0.049482	0.03965	0.009897	0.038373909
Nucleosides and Nucleotides	Purines	De Novo Purine Biosynthesis	Phosphoribosylaminoimidazole carboxylase catalytic subunit (EC 4.1.1.21)	0.103055	0.038454	0.022993	0.042331	0.030202	0.026787	0.035382	0.047843	0.02912	0.041853	0.015136	0.036954113
Cofactors, Vitamins, Prosthetic Groups, Pigments	Folate and pterines	5-FCL-like protein	Phosphoribosylaminoimidazolecarboxamide formyltransferase (EC 2.1.2.3)	0.068239	0.083693	0.155843	0.144422	0.089292	0.089128	0.096037	0.102662	0.08101	0.035245	0.135529	0.10707622
Nucleosides and Nucleotides	Purines	De Novo Purine Biosynthesis	Phosphoribosylaminoimidazole-succinocarboxamide synthase (EC 6.3.2.6)	0.074854	0.063336	0.112411	0.181773	0.074191	0.089128	0.093149	0.087213	0.070282	0.110139	0.150432	0.088934392
Cofactors, Vitamins, Prosthetic Groups, Pigments	Folate and pterines	YgfZ	Phosphoribosyl-AMP cyclohydrolase (EC 3.5.4.19)	0.040038	0.03393	0.038322	0.049801	0.022979	0.025813	0.032494	0.033889	0.027368	0.019825	0.012342	0.030959423
Amino Acids and Derivatives	Aromatic amino acids and derivatives	Chorismate: Intermediate for synthesis of Tryptophan, PAPA antibiotics, PABA, 3-hydroxyanthranilate and more.	Phosphoribosylanthranilate isomerase (EC 5.3.1.24)	0.062668	0.01131	0.030658	0.052291	0.022323	0.036041	0.022385	0.03339	0.033718	0.011014	0.028061	0.035573757
Amino Acids and Derivatives	Histidine Metabolism	Histidine Biosynthesis	Phosphoribosyl-ATP pyrophosphatase (EC 3.6.1.31)	0.01706	0.01131	0.028103	0.00996	0.004596	0.007793	0.022385	0.016944	0.019267	0.01542	0.02189	0.020941979
Amino Acids and Derivatives	Aromatic amino acids and derivatives	Chorismate: Intermediate for synthesis of Tryptophan, PAPA antibiotics, PABA, 3-hydroxyanthranilate and more.	Phosphoribosylformimino-5-aminoimidazole carboxamide ribotide isomerase (EC 5.3.1.16)	0.05292	0.027144	0.038322	0.02241	0.047272	0.036041	0.043325	0.053823	0.036126	0.022028	0.005007	0.04878574
Nucleosides and Nucleotides	Purines	De Novo Purine Biosynthesis	Phosphoribosylformylglycinamidine cyclo-ligase (EC 6.3.3.1)	0.059187	0.106313	0.084308	0.087151	0.081413	0.079875	0.091705	0.092197	0.072033	0.057273	0.082552	0.086134241
Nucleosides and Nucleotides	Purines	De Novo Purine Biosynthesis	Phosphoribosylformylglycinamidine synthase, glutamine amidotransferase subunit (EC 6.3.5.3)	0.186613	0.169649	0.158398	0.129482	0.153634	0.13978	0.135752	0.138544	0.151292	0.17402	0.367814	0.209143718
Nucleosides and Nucleotides	Purines	De Novo Purine Biosynthesis	Phosphoribosylformylglycinamidine synthase, PurS subunit (EC 6.3.5.3)	0.014274	0.047502	0.020438	0.01992	0.024293	0.028735	0.01372	0.017941	0.017735	0.011014	0.000349	0.0164854
Nucleosides and Nucleotides	Purines	De Novo Purine Biosynthesis	Phosphoribosylformylglycinamidine synthase, synthetase subunit (EC 6.3.5.3)	0.147619	0.133457	0.102192	0.097112	0.128685	0.102765	0.108313	0.126085	0.102905	0.033042	0.00163	0.074736441
Nucleosides and Nucleotides	Purines	De Novo Purine Biosynthesis	Phosphoribosylglycinamide formyltransferase (EC 2.1.2.2)	0.028549	0.031668	0.028103	0.042331	0.033484	0.038963	0.039715	0.034885	0.038097	0.063881	0.025732	0.037861205
Nucleosides and Nucleotides	Purines	De Novo Purine Biosynthesis	Phosphoribosylglycinamide formyltransferase 2 (EC 2.1.2.-)	0.017408	0.020358	0.015329	0.02241	0.006566	0.01315	0.008665	0.014951	0.012699	0.05507	0.075682	0.032339779
Carbohydrates	CO2 fixation	Calvin-Benson cycle	Phosphoribulokinase (EC 2.7.1.19)	0.031682	0.020358	0.012774	0.01245	0.015757	0.016559	0.015886	0.008472	0.014669	0.030839	0.00326	0.018536215
Amino Acids and Derivatives	Alanine, serine, and glycine	Serine Biosynthesis	Phosphoserine aminotransferase (EC 2.6.1.52)	0.04909	0.099527	0.06898	0.087151	0.069595	0.086693	0.082318	0.093692	0.070939	0.068286	0.107818	0.086410312

Amino Acids and Derivatives	Alanine, serine, and glycine	Glycine and Serine Utilization	Phosphoserine phosphatase (EC 3.1.3.3)	0.038646	0.02262	0.017884	0.042331	0.025606	0.035067	0.036104	0.046348	0.048606	0.077098	0.156021	0.04212059
Metabolism of Aromatic Compounds	Peripheral pathways for catabolism of aromatic compounds	p-Hydroxybenzoate degradation	P-hydroxybenzoate hydroxylase (EC 1.14.13.2)	0.007659	0.004524	0.020438	0.00996	0.012475	0.007306	0.01733	0.014951	0.021238	0.011014	0.104208	0.006744027
Photosynthesis	NULL	Proteorhodopsin	Phytoene dehydrogenase (EC 1.14.99.-)	0.01323	0.029406	0.022993	0.034861	0.032171	0.035067	0.042603	0.05781	0.041381	0.112342	0.002096	0.033286309
Fatty Acids, Lipids, and Isoprenoids	Isoprenoids	Carotenoids	Phytoene dehydrogenase and related proteins	0.011489	0.006786	0.022993	0.01743	0.008535	0.010228	0.010109	0.015948	0.019267	0.008811	0.001397	0.00982025
Fatty Acids, Lipids, and Isoprenoids	Isoprenoids	Carotenoids	Phytoene desaturase (EC 1.14.99.-)	0.004526	0.015834	0.00511	0.00498	0.007879	0.002922	0.010109	0.00897	0.008101	0.004406	0.001397	0.004732651
Fatty Acids, Lipids, and Isoprenoids	Isoprenoids	Carotenoids	Phytoene desaturase, neurosporene or lycopene producing (EC 1.3.-.-)	0.001044	0.002262	0.010219	0.01743	0.013131	0.007793	0.008665	0.011462	0.012042	0.004406	0.000233	0.004022753
Fatty Acids, Lipids, and Isoprenoids	Isoprenoids	Carotenoids	Phytoene synthase (EC 2.5.1.32)	0.033423	0.029406	0.053651	0.032371	0.029545	0.035554	0.031772	0.039869	0.032185	0.050664	0.009548	0.024806977
RNA Metabolism	RNA processing and modification	Polyadenylation bacterial	Poly(A) polymerase (EC 2.7.7.19)	0.014971	0.02262	0.007664	0.01245	0.013788	0.018995	0.033216	0.030898	0.029777	0.017622	0.021191	0.023702692
Carbohydrates	Monosaccharides	D-Galacturonate and D-Glucuronate Utilization	Polygalacturonase (EC 3.2.1.15)	0.000348	0.020358	0.017884	0.037351	0.007879	0.004383	0.006499	0.004984	0.007225	0.008811	0.000233	0.011042851
Fatty Acids, Lipids, and Isoprenoids	NULL	Polyhydroxybutyrate metabolism	Polyhydroxyalkanoic acid synthase	0.043172	0.042978	0.048541	0.037351	0.05909	0.066724	0.067154	0.070269	0.079478	0.046259	0.037608	0.033799013
Cell Wall and Capsule	Gram-Negative cell wall components	Lipid A-Ara4N pathway (Polymyxin resistance)	Polymyxin resistance protein ArnA DH, UDP-glucuronic acid decarboxylase (EC 4.1.1.-)	0.056053	0.013572	0.007664	0.00747	0.000657	0.000487	0.002166	0.001993	0.002846	0.008811	0.000233	0.005087599
Cell Wall and Capsule	Gram-Negative cell wall components	Lipid A-Ara4N pathway (Polymyxin resistance)	Polymyxin resistance protein ArnC, glycosyl transferase (EC 2.4.-.-)	0.025764	0.004524	0.028103	0.01992	0.006566	0.006819	0.010109	0.005482	0.011385	0.006608	0.002329	0.017865756
Cell Wall and Capsule	Gram-Negative cell wall components	Lipid A-Ara4N pathway (Polymyxin resistance)	Polymyxin resistance protein ArnT, undecaprenyl phosphate-alpha-L-Ara4N transferase	0.009052	0.015834	0.048541	0.02241	0.004596	0.005844	0.004333	0.00299	0.014888	0.006608	0.000233	0.010924535
Phosphorus Metabolism	NULL	Phosphate metabolism	Polyphosphate kinase (EC 2.7.4.1)	0.084951	0.117623	0.130295	0.136952	0.122776	0.116403	0.106146	0.137049	0.163115	0.121153	0.109797	0.103999997
Clustering-based subsystems	NULL	Bacterial RNA-metabolizing Zn-dependent hydrolases	Polyribonucleotide nucleotidyltransferase (EC 2.7.7.8)	0.183131	0.232984	0.168617	0.186753	0.187118	0.205531	0.20796	0.203829	0.194644	0.226887	0.201896	0.19798255
Cell Wall and Capsule	Gram-Negative cell wall components	Vibrio Core Oligosaccharide Biosynthesis	Polysaccharide deacetylase	0.018104	0.02262	0.022993	0.02988	0.01904	0.031658	0.018774	0.021928	0.036564	0.011014	0.000931	0.030446719
Cofactors, Vitamins, Prosthetic Groups, Pigments	Tetrapyrroles	Heme and Siroheme Biosynthesis	Porphobilinogen deaminase (EC 2.5.1.61)	0.03029	0.047502	0.056206	0.049801	0.049898	0.045295	0.03466	0.046846	0.042257	0.033042	0.095476	0.052374667
Regulation and Cell signaling	NULL	Zinc regulated enzymes	Porphobilinogen synthase (EC 4.2.1.24)	0.099225	0.067859	0.086863	0.057271	0.075504	0.075004	0.081596	0.084223	0.080791	0.101328	0.081853	0.090866891
Motility and Chemotaxis	NULL	Bacterial Chemotaxis	Positive regulator of CheA protein activity (CheW)	0.017408	0.013572	0.033212	0.01992	0.03808	0.045782	0.041881	0.027908	0.01664	0.01542	0.004192	0.035139931
Miscellaneous	Plant-Prokaryote DOE project	COG0398	Possible sterol desaturase	0.005571	0.01131	0.007664	0.00498	0.025606	0.024352	0.012998	0.023423	0.019705	0.061678	0.002212	0.017510807
Potassium metabolism	NULL	Potassium homeostasis	Potassium efflux system KefA	0.023327	0.018096	0.012774	0.01992	0.030202	0.025813	0.031772	0.021429	0.031747	0.006608	0.025266	0.027015547
Potassium metabolism	NULL	Potassium homeostasis	Potassium efflux system KefA protein	0.017408	0.018096	0.025548	0.01992	0.028888	0.024352	0.024551	0.031895	0.034594	0.013217	0.006287	0.027291618
Potassium metabolism	NULL	Glutathione-regulated potassium-efflux system and associated functions	Potassium efflux system KefA protein / Small-conductance mechanosensitive channel	0.046653	0.042978	0.061315	0.01992	0.071565	0.075978	0.041159	0.058807	0.07488	0.019825	0.061593	0.06113007
Potassium metabolism	NULL	Potassium homeostasis	Potassium uptake protein TrkH	0.10201	0.151553	0.109856	0.134462	0.117523	0.120299	0.109035	0.098177	0.100934	0.01542	0.11003	0.089171025
Potassium metabolism	NULL	Hyperosmotic potassium uptake	Potassium uptake protein, integral membrane component, KtrB	0.011141	0.027144	0.028103	0.039841	0.011161	0.011202	0.016608	0.014452	0.024303	0.006608	0.003609	0.032694728
Potassium metabolism	NULL	Potassium homeostasis	Potassium voltage-gated channel subfamily KQT	0.029593	0.01131	0.028103	0.01494	0.051211	0.040424	0.050546	0.052826	0.031966	0.037447	0.048786	0.039833143
Cofactors, Vitamins, Prosthetic Groups, Pigments	Tetrapyrroles	Heme and Siroheme Biosynthesis	Precorrin-2 oxidase (EC 1.3.1.76)	0.016363	0.020358	0.020438	0.00747	0.016414	0.019969	0.012275	0.012957	0.008101	0.028636	0.025732	0.013764125
Metabolism of Aromatic Compounds	NULL	Benzoate transport and degradation cluster	Predicted 2-keto-4-pentenoate hydratase/2-oxohepta-3-ene-1,7-dioic acid hydratase	0.003133	0.013572	0.00511	0.00747	0.009192	0.007306	0.00361	0.00299	0.003503	0.011014	0.000931	0.005245354
Phosphorus Metabolism	NULL	Phosphate metabolism	Predicted ATPase related to phosphate starvation-inducible protein PhoH	0.038297	0.047502	0.043432	0.032371	0.049898	0.035554	0.03466	0.049338	0.038754	0.030839	0.053909	0.037742889
Membrane Transport	Protein secretion system, Type II	Widespread colonization island	Predicted ATPase with chaperone activity, associated with Flp pilus assembly	0.004178	0.004524	0.007664	0.00498	0.003283	0.002922	0.001444	0.004485	0.008977	0.006608	0.001514	0.003588927
Carbohydrates	Organic acids	Lactate utilization	Predicted D-lactate dehydrogenase, Fe-S protein, FAD/FMN-containing	0.006615	0.018096	0.022993	0.00498	0.013788	0.030684	0.018052	0.00897	0.017954	0.006608	0.144145	0.032418657
Amino Acids and Derivatives	Lysine, threonine, methionine, and cysteine	Threonine and Homoserine Biosynthesis	Predicted functional analog of homoserine kinase (EC 2.7.1.-)	0.007311	0.031668	0.010219	0.01743	0.028232	0.018995	0.009387	0.006977	0.004817	0.004406	0.000349	0.014552901
Carbohydrates	Polysaccharides	Glycogen metabolism	Predicted glycogen synthase, ADP-glucose transglucosylase (EC 2.4.1.21), Actinobacterial type	0.002785	0.004524	0.002555	0.00249	0.007222	0.00487	0.002166	0.001993	0.003941	0.002203	0.002911	0.004101631
Carbohydrates	Organic acids	Lactate utilization	Predicted L-lactate dehydrogenase, Fe-S oxidoreductase subunit YkgE	0.017756	0.013572	0.015329	0.01992	0.022323	0.024352	0.025273	0.02741	0.023427	0.002203	0.01991	0.022282897
Carbohydrates	Monosaccharides	L-rhamnose utilization	Predicted L-lactate dehydrogenase, Iron-sulfur cluster-binding subunit YkgF	0.020193	0.024882	0.030658	0.032371	0.045959	0.04773	0.044769	0.03937	0.037002	0.026433	0.045875	0.037821766
Carbohydrates	Monosaccharides	L-rhamnose utilization	Predicted L-rhamnose ABC transporter, transmembrane component 1	0.007311	0.009048	0.002555	0.00249	0.004596	0.000487	0.002166	0.001993	0.003722	0.006608	0.000931	0.001459234
Carbohydrates	Di- and oligosaccharides	Maltose and Maltodextrin Utilization	Predicted maltose transporter MalT	0.01706	0.006786	0.010219	0.00747	0.003283	0.005844	0.010109	0.017941	0.017735	0.006608	0.005007	0.018930602
Cofactors, Vitamins, Prosthetic Groups, Pigments	NAD and NADP	NAD regulation	Predicted NAD regulator in Alphaproteobacteria	0.000696	0.004524	0.00511	0.00498	0.00197	0.000487	0.002166	0.002492	0.002627	0.002203	0.000233	0.000788775
Carbohydrates	Monosaccharides	Deoxyribose and Deoxynucleoside Catabolism	Predicted nucleoside ABC transporter, ATP-binding component	0.002785	0.004524	0.00511	0.00249	0.003939	0.006819	0.010109	0.008472	0.012918	0.013217	0.001048	0.004101631
Carbohydrates	Monosaccharides	L-rhamnose utilization	Predicted rhamnogalacturonan lyase in rhamnose utilization cluster	0.008704	0.015834	0.00511	0.00498	0.009192	0.004383	0.002888	0.003489	0.005036	0.017622	0.000116	0.007493363
Carbohydrates	Monosaccharides	L-rhamnose utilization	Predicted rhamnulose-1-phosphate aldolase (EC 4.1.2.19)	0.001741	0.013572	0.002555	0.00249	0.007222	0.005844	0.005777	0.011462	0.014888	0.002203	0.001747	0.002484642
Membrane Transport	Protein secretion system, Type II	Predicted secretion system W clustering with cell division proteins	Predicted secretion system W protein GspE-like	0.002437	0.002262	0.00511	0.01743	0.005909	0.002435	0.002888	0.001495	0.003503	0.006608	0.000466	0.003983314
Regulation and Cell signaling	NULL	cAMP signaling in bacteria	Predicted signal-transduction protein containing cAMP-binding and CBS domains	0.008704	0.006786	0.010219	0.00249	0.007879	0.017046	0.012998	0.021429	0.050139	0.008811	0.143912	0.044684109
Carbohydrates	Monosaccharides	Mannose Metabolism	Predicted sodium-dependent mannose transporter	0.008704	0.009048	0.007664	0.00996	0.006566	0.003896	0.012998	0.004984	0.010728	0.008811	0.000699	0.012462647
Amino Acids and Derivatives	Aromatic amino acids and derivatives	Chorismate Synthesis	Prephenate and/or arogenate dehydrogenase (unknown specificity) (EC 1.3.1.12)(EC 1.3.1.43)	0.008356	0.006786	0.002555	0.00747	0.012475	0.003896	0.010109	0.009469	0.004817	0.050664	0.001979	0.019048919
Amino Acids and Derivatives	Aromatic amino acids and derivatives	Chorismate Synthesis	Prephenate dehydratase (EC 4.2.1.51)	0.031682	0.024882	0.043432	0.039841	0.032171	0.038476	0.030328	0.037377	0.028025	0.063881	0.041101	0.045117936
Membrane Transport	Protein translocation across cytoplasmic membrane	HtrA and Sec secretion	Preprotein translocase secY subunit (TC 3.A.5.1.1)	0.125685	0.099527	0.120076	0.119522	0.143786	0.131014	0.13864	0.147515	0.121515	0.116748	0.142282	0.120879784
Membrane Transport	Protein translocation across cytoplasmic membrane	HtrA and Sec secretion	Preprotein translocase subunit SecG (TC 3.A.5.1.1)	0.003133	0.006786	0.00511	0.01494	0.014444	0.010228	0.015164	0.010964	0.007006	0.004406	0.013739	0.013803564
Regulation and Cell signaling	Programmed Cell Death and Toxin-antitoxin Systems	Phd-Doc, YdcE-YdcD toxin-antitoxin (programmed cell death) systems	Prevent host death protein, Phd antitoxin	0.011141	0.006786	0.015329	0.00996	0.008535	0.008767	0.007221	0.004485	0.005036	0.002203	0.000233	0.007453925
Secondary Metabolism	Aromatic amino acids and derivatives	Cinnamic Acid Degradation	Probable 3-phenylpropionic acid transporter	0.009748	0.002262	0.00511	0.00747	0.005909	0.006332	0.005777	0.005482	0.003284	0.002203	0.009548	0.006428517
Amino Acids and Derivatives	Branched-chain amino acids	Isoleucine degradation	Probable acyl-CoA dehydrogenase (EC 1.3.99.3)	0.011141	0.01131	0.017884	0.00747	0.009848	0.00487	0.002888	0.00299	0.014013	0.013217	0.010013	0.007493363
Virulence, Disease and Defense	Resistance to antibiotics and toxic compounds	Cobalt-zinc-cadmium resistance	Probable Co/Zn/Cd efflux system membrane fusion protein	0.036208	0.04524	0.061315	0.039841	0.052524	0.055523	0.038993	0.061298	0.057145	0.090314	0.009082	0.070200984
Cell Wall and Capsule	Gram-Negative cell wall components	Lipopolysaccharide assembly	Probable component of the lipoprotein assembly complex (forms a complex with YaeT, YfgL, and NlpB)	0.008704	0.002262	0.00511	0.00747	0.007222	0.012176	0.007221	0.006977	0.015545	0.011014	0.0333	0.008913159
Protein Metabolism	Protein biosynthesis	Universal GTPases	Probable GTPase related to EngC	0.006267	0.031668	0.038322	0.02988	0.043989	0.050652	0.015164	0.016446	0.012699	0.002203	0.021307	0.044289722
Clustering-based subsystems	NULL	CBSS-316273.3.peg.922	probable iron binding protein from the HesB	0.019149	0.015834	0.022993	0.02241	0.016414	0.025326	0.02094	0.017941	0.020143	0.017622	0.007335	0.019364429

			IscA SufA family												
Phosphorus Metabolism	NULL	Phosphate metabolism	Probable low-affinity inorganic phosphate transporter	0.085299	0.095003	0.071534	0.099602	0.086665	0.090589	0.057045	0.050334	0.053642	0.072692	0.104558	0.103329538
Cell Wall and Capsule	Capsular and extracellular polysacchrides	Alginate metabolism	Probable poly(beta-D-mannuronate) O-acetylase (EC 2.3.1.-)	0.034468	0.049764	0.05876	0.047311	0.03808	0.04773	0.058489	0.050833	0.033499	0.004406	0.00326	0.046340537
Virulence, Disease and Defense	Resistance to antibiotics and toxic compounds	The mdtABCD multidrug resistance cluster	Probable RND efflux membrane fusion protein	0.015667	0.024882	0.020438	0.0249	0.015757	0.027274	0.022385	0.029902	0.054737	0.035245	0.008965	0.032891922
Carbohydrates	CO2 fixation	CO2 uptake, carboxysome	probable RuBisCo-expression protein CbbX	0.004526	0.006786	0.007664	0.00249	0.005909	0.000974	0.002166	0.001993	0.004379	0.006608	0.000582	0.001459234
Iron acquisition and metabolism	NULL	Iron Scavenging cluster in Thermus	Probable thiol oxidoreductase with 2 cytochrome c heme-binding sites	0.000696	0.002262	0.010219	0.00498	0.013788	0.005357	0.012998	0.012957	0.027149	0.006608	0.022122	0.011437239
Respiration	Electron donating reactions	Respiratory dehydrogenases 1	Proline dehydrogenase (EC 1.5.99.8) (Proline oxidase)	0.051876	0.03393	0.043432	0.049801	0.054494	0.056984	0.066432	0.072761	0.084295	0.024231	0.056121	0.04815472
Amino Acids and Derivatives	Proline and 4-hydroxyproline	Proline, 4-hydroxyproline uptake and utilization	Proline iminopeptidase (EC 3.4.11.5)	0.033075	0.01131	0.020438	0.01494	0.025606	0.026787	0.037548	0.025416	0.031747	0.013217	0.042265	0.024846416
Miscellaneous	Plant-Prokaryote DOE project	COG1565	Prolipoprotein diacylglyceryl transferase (EC 2.4.99.-)	0.178953	0.054288	0.038322	0.01743	0.044646	0.053087	0.054878	0.043856	0.04773	0.03965	0.048436	0.056357981
Protein Metabolism	Protein degradation	Serine endopeptidase (EC 3.4.21.-)	Prolyl endopeptidase (EC 3.4.21.26)	0.019845	0.03393	0.025548	0.01743	0.013788	0.01802	0.019496	0.034885	0.06021	0.072692	0.017232	0.057896092
Miscellaneous	Plant-Prokaryote DOE project	Synechocystis experimental	prolyl oligopeptidase family protein	0.019497	0.027144	0.017884	0.01992	0.024949	0.01802	0.014442	0.028407	0.036126	0.013217	0.004657	0.021612438
Protein Metabolism	Protein biosynthesis	tRNA aminoacylation, Pro	Prolyl-tRNA synthetase (EC 6.1.1.15)	0.18522	0.133457	0.12774	0.219124	0.148382	0.150008	0.134308	0.163462	0.143629	0.147587	0.153809	0.152430788
Protein Metabolism	Protein processing and modification	Protein Acetylation and Deacetylation in Bacteria	Propionate--CoA ligase (EC 6.2.1.17)	0.011141	0.024882	0.017884	0.01743	0.014444	0.01315	0.01372	0.013954	0.034813	0.05507	0.207718	0.035376563
Carbohydrates	One-carbon Metabolism	Serine-glyoxylate cycle	Propionyl-CoA carboxylase beta chain (EC 6.4.1.3)	0.009748	0.04524	0.051096	0.0249	0.034141	0.031171	0.030328	0.022925	0.034375	0.013217	0.003609	0.027646567
Carbohydrates	Organic acids	Propionyl-CoA to Succinyl-CoA Module	Propionyl-CoA carboxylase biotin-containing subunit (EC 6.4.1.3)	0.026808	0.02262	0.020438	0.0249	0.012475	0.019482	0.030328	0.029902	0.029996	0.026433	0.003726	0.017510807
Carbohydrates	One-carbon Metabolism	Serine-glyoxylate cycle	Propionyl-CoA carboxylase carboxyl transferase subunit (EC 6.4.1.3)	0.041083	0.04524	0.045986	0.034861	0.028888	0.03945	0.044769	0.050833	0.046855	0.057273	0.00687	0.035849828
Miscellaneous	NULL	ZZ gjo need homes	Proposed peptidoglycan lipid II flippase MurJ	0.014971	0.027144	0.043432	0.02739	0.044646	0.038963	0.046213	0.044354	0.044227	0.019825	0.066717	0.042633294
Protein Metabolism	Protein processing and modification	Protein Acetylation and Deacetylation in Bacteria	Protein acetyltransferase	0.020889	0.006786	0.028103	0.02241	0.02101	0.018995	0.018774	0.010964	0.034813	0.011014	0.000233	0.019482745
Membrane Transport	Protein translocation across cytoplasmic membrane	HtrA and Sec secretion	Protein export cytoplasm chaperone protein (SecB, maintains protein to be exported in unfolded state)	0.006963	0.009048	0.002555	0.00498	0.006566	0.007306	0.005777	0.013954	0.01248	0.002203	0.030506	0.010411831
Clustering-based subsystems	Protein export?	CBSS-393121.3.peg.2760	Protein export cytoplasm protein SecA ATPase RNA helicase (TC 3.A.5.1.1)	0.238836	0.361917	0.245261	0.296315	0.315803	0.333622	0.327826	0.293534	0.246096	0.361257	0.23834	0.266132719
Clustering-based subsystems	NULL	CBSS-316407.3.peg.2816	Protein involved in stability of MscS mechanosensitive channel	0.002089	0.004524	0.00511	0.00498	0.006566	0.006332	0.00361	0.004984	0.008758	0.011014	0.007801	0.011634433
RNA Metabolism	RNA processing and modification	RNA 3'-terminal phosphate cyclase	Protein RtcB	0.100618	0.151553	0.135404	0.109562	0.141816	0.126143	0.120588	0.083226	0.052547	0.022028	0.010013	0.066257108
Clustering-based subsystems	NULL	CBSS-323097.3.peg.2594	Protein YicC	0.078684	0.015834	0.022993	0.02739	0.015101	0.019969	0.023107	0.041364	0.037002	0.118951	0.020842	0.032221463
Clustering-based subsystems	NULL	RNA modification cluster	Protein YidD	0.017756	0.020358	0.010219	0.00747	0.011818	0.006819	0.018052	0.014452	0.013575	0.017622	0.000349	0.010175199
Membrane Transport	Protein translocation across cytoplasmic membrane	HtrA and Sec secretion	Protein-export membrane protein SecD (TC 3.A.5.1.1)	0.089128	0.083693	0.114966	0.119522	0.131968	0.119325	0.157414	0.113626	0.093271	0.116748	0.097804	0.088540005
Membrane Transport	Protein translocation across cytoplasmic membrane	HtrA and Sec secretion	Protein-export membrane protein SecF (TC 3.A.5.1.1)	0.041431	0.05655	0.094528	0.047311	0.07616	0.062341	0.064265	0.071764	0.069187	0.035245	0.099318	0.091182401
Membrane Transport	NULL	Ton and Tol transport systems	Protein-L-isoaspartate O-methyltransferase (EC 2.1.1.77)	0.052572	0.104051	0.081754	0.102092	0.093887	0.105201	0.059211	0.072262	0.07269	0.037447	0.031088	0.078956387
Miscellaneous	Plant-Prokaryote DOE project	YrdC-YciO-Sua5 protein family	Protein-N(5)-glutamine methyltransferase PrmB, methylates LSU ribosomal protein L3p	0.054661	0.009048	0.022993	0.01494	0.011818	0.011689	0.009387	0.011961	0.021019	0.002203	0.045875	0.020665908
Miscellaneous	Plant-Prokaryote DOE project	At4g38090	Protein-N(5)-glutamine methyltransferase PrmC, methylates polypeptide chain release factors RF1 and RF2	0.036557	0.018096	0.025548	0.039841	0.033484	0.026787	0.021663	0.029403	0.027368	0.002203	0.048669	0.038689419
Metabolism of Aromatic Compounds	Metabolism of central aromatic intermediates	Protocatechuate branch of beta-ketoadipate pathway	Protocatechuate 3,4-dioxygenase beta chain (EC 1.13.11.3)	0.002089	0.002262	0.017884	0.00249	0.003283	0.002922	0.008665	0.00897	0.011166	0.011014	0.000815	0.002918468
Metabolism of Aromatic Compounds	Metabolism of central aromatic intermediates	Central meta-cleavage pathway of aromatic compound degradation	Protocatechuate 4,5-dioxygenase beta chain (EC 1.13.11.8)	0.010097	0.002262	0.025548	0.01245	0.015101	0.003409	0.007943	0.005482	0.016859	0.006608	0.123769	0.006783466
Clustering-based subsystems	Putrescine/GABA utilization cluster-temporal,to add to SSs	GABA and putrescine metabolism from cluters	Psp operon transcriptional activator	0.021238	0.02262	0.007664	0.032371	0.02101	0.017046	0.01733	0.007974	0.015983	0.01542	0.004541	0.009425862
Cofactors, Vitamins, Prosthetic Groups, Pigments	Folate and pterines	Pterin biosynthesis	Pterin-4-alpha-carbinolamine dehydratase (EC 4.2.1.96)	0.016363	0.018096	0.010219	0.02241	0.019697	0.014124	0.016608	0.024918	0.0208	0.01542	0.001397	0.009544179
Clustering-based subsystems	NULL	Lipopolysaccharide assembly cluster	PTS system nitrogen-specific IIA component, PtsN	0.005571	0.004524	0.010219	0.00747	0.004596	0.005844	0.018774	0.016446	0.012042	0.006608	0.004774	0.00634964
Carbohydrates	NULL	Sugar utilization in Thermotogales	PTS system, fructose-specific IIB component (EC 2.7.1.69)	0.002785	0.004524	0.00511	0.00747	0.003939	0.00487	0.00361	0.000997	0.000657	0.008811	0.003493	0.002721274
Nucleosides and Nucleotides	Pyrimidines	pyrimidine conversions	Purine nucleoside phosphorylase (EC 2.4.2.1)	0.097136	0.04524	0.061315	0.057271	0.04202	0.036041	0.037548	0.051331	0.042476	0.096923	0.014787	0.053636707
Respiration	Electron accepting reactions	Terminal cytochrome d ubiquinol oxidases	putative Cytochrome bd2, subunit I	0.021238	0.02262	0.033212	0.01992	0.024949	0.032145	0.031772	0.029403	0.042695	0.008811	0.004657	0.011200606
Respiration	NULL	Biogenesis of c-type cytochromes	Putative cytochrome C-type biogenesis protein	0.019149	0.013572	0.017884	0.01494	0.006566	0.007306	0.009387	0.015449	0.013137	0.002203	0.004657	0.066493741
Nitrogen Metabolism	NULL	Nitric oxide synthase	putative cytochrome P450 hydroxylase	0.025416	0.024882	0.071534	0.02988	0.02101	0.023378	0.032494	0.037875	0.044227	0.017622	0.064039	0.024609783
DNA Metabolism	NULL	YcfH	Putative deoxyribonuclease YcfH	0.031682	0.03393	0.015329	0.034861	0.041363	0.029222	0.035382	0.038872	0.03109	0.072692	0.021191	0.042278345
Carbohydrates	Monosaccharides	Deoxyribose and Deoxynucleoside Catabolism	Putative deoxyribose-specific ABC transporter, ATP-binding protein	0.006615	0.01131	0.012774	0.00747	0.008535	0.009254	0.007221	0.003489	0.007444	0.004406	0.000815	0.010332954
Clustering-based subsystems	Pyruvate kinase associated cluster	CBSS-288000.5.peg.1793	putative esterase	0.010445	0.029406	0.020438	0.01743	0.012475	0.014124	0.007943	0.011961	0.016859	0.01542	0.000233	0.012778157
Respiration	NULL	Formate hydrogenase	Putative formate dehydrogenase iron-sulfur subunit (EC 1.2.1.2)	0.003133	0.006786	0.022993	0.00249	0.005252	0.001461	0.005777	0.002492	0.005912	0.006608	0.000931	0.004022753
Respiration	NULL	Formate hydrogenase	Putative formate dehydrogenase oxidoreductase protein	0.020193	0.002262	0.015329	0.00498	0.005252	0.006819	0.01372	0.015449	0.030434	0.035245	0.005589	0.008321577
Potassium metabolism	NULL	Potassium homeostasis	putative Glutathione-regulated potassium-efflux system protein KefB	0.0094	0.002262	0.015329	0.00996	0.014444	0.029222	0.015164	0.009967	0.015107	0.002203	0.044012	0.011161168
Miscellaneous	Plant-Prokaryote DOE project	COG0523	Putative metal chaperone, involved in Zn homeostasis, GTPase of COG0523 family	0.007311	0.004524	0.002555	0.00996	0.022323	0.028735	0.032494	0.035882	0.01445	0.017622	0.010828	0.007375047
Protein Metabolism	Protein processing and modification	G3E family of P-loop GTPases (metallocenter biosynthesis)	putative periplasmic protein kinase ArgK and related GTPases of G3E family	0.027156	0.031668	0.056206	0.037351	0.041363	0.039937	0.032494	0.037377	0.033718	0.035245	0.007102	0.036283655
Miscellaneous	NULL	ZZ gjo need homes	Putative permease often clustered with de novo purine synthesis	0.014274	0.002262	0.002555	0.00747	0.015101	0.011689	0.012998	0.021429	0.013575	0.006608	0.027362	0.011871065
RNA Metabolism	RNA processing and modification	Queuosine-Archaeosine Biosynthesis	Putative preQ0 transporter	0.016363	0.020358	0.043432	0.01992	0.027575	0.015098	0.022385	0.031895	0.020581	0.072692	0.00326	0.016761471
Regulation and Cell signaling	NULL	Orphan regulatory proteins	Putative sensory histidine kinase YfhA	0.053964	0.038454	0.012774	0.02988	0.025606	0.011202	0.014442	0.013954	0.032842	0.004406	0.100715	0.030762229
Miscellaneous	NULL	YbbK	Putative stomatin/prohibitin-family membrane protease subunit aq 911	0.066498	0.049764	0.056206	0.054781	0.081413	0.049678	0.039715	0.02741	0.027587	0.074895	0.006287	0.045867272
Clustering-based subsystems	Probably Ybbk-related hypothetical membrane proteins	CBSS-316057.3.peg.659	Putative stomatin/prohibitin-family membrane protease subunit YbbK	0.042475	0.03393	0.045986	0.049801	0.04005	0.040911	0.03827	0.043357	0.049263	0.072692	0.029341	0.048273036
Carbohydrates	Di- and oligosaccharides	Maltose and Maltodextrin Utilization	Putative sucrose phosphorylase (EC 2.4.1.7)	0.024023	0.029406	0.020438	0.037351	0.028888	0.033119	0.029605	0.028407	0.020362	0.006608	0.108866	0.035968145
Clustering-based subsystems	Translation	CBSS-326442.4.peg.1852	Putative TEGT family carrier/transport protein	0.014623	0.018096	0.007664	0.00498	0.017727	0.012176	0.01733	0.012459	0.016421	0.004406	0.037841	0.015183921
Amino Acids and Derivatives	Arginine; urea cycle, polyamines	Polyamine Metabolism	Putrescine ABC transporter putrescine-binding protein PotF (TC 3.A.1.11.2)	0.019149	0.009048	0.002555	0.01494	0.005909	0.006819	0.00361	0.007475	0.011166	0.017622	0.136344	0.024057641

Amino Acids and Derivatives	Arginine; urea cycle, polyamines	Polyamine Metabolism	Putrescine transport ATP-binding protein PotA (TC 3.A.1.11.1)	0.070676	0.029406	0.053651	0.02739	0.047929	0.041885	0.044047	0.027908	0.059772	0.017622	0.018979	0.035337124
Clustering-based subsystems	Putrescine/GABA utilization cluster-temporal,to add to SSs	GABA and putrescine metabolism from cluters	Putrescine transport ATP-binding protein PotG (TC 3.A.1.11.2)	0.032727	0.015834	0.020438	0.01992	0.011161	0.016072	0.012998	0.011961	0.024741	0.011014	0.068463	0.017037542
Clustering-based subsystems	Putrescine/GABA utilization cluster-temporal,to add to SSs	GABA and putrescine metabolism from cluters	Putrescine transport system permease protein PotH (TC 3.A.1.11.2)	0.001044	0.020358	0.007664	0.00996	0.009848	0.006332	0.011553	0.010466	0.015326	0.011014	0.053676	0.010609025
Nucleosides and Nucleotides	Pyrimidines	Pyrimidine utilization	Pyridine nucleotide-disulphide oxidoreductase associated with reductive pyrimidine catabolism	0.006963	0.002262	0.007664	0.00249	0.003939	0.007793	0.006499	0.00598	0.008977	0.008811	0.001164	0.004219947
Cofactors, Vitamins, Prosthetic Groups, Pigments	Pyridoxine	Pyridoxin (Vitamin B6) Biosynthesis	Pyridoxamine 5'-phosphate oxidase (EC 1.4.3.5)	0.013578	0.024882	0.012774	0.02241	0.015101	0.017046	0.018052	0.03339	0.035688	0.01542	0.004424	0.027212741
Cofactors, Vitamins, Prosthetic Groups, Pigments	Pyridoxine	Pyridoxin (Vitamin B6) Biosynthesis	Pyridoxine 5'-phosphate synthase (EC 2.6.99.2)	0.145878	0.058812	0.048541	0.042331	0.040706	0.037015	0.058489	0.062295	0.042476	0.033042	0.060778	0.058251041
Cofactors, Vitamins, Prosthetic Groups, Pigments	Pyridoxine	Pyridoxin (Vitamin B6) Biosynthesis	Pyridoxine biosynthesis glutamine amidotransferase, synthase subunit (EC 2.4.2.-)	0.014971	0.027144	0.074089	0.039841	0.032828	0.028248	0.037548	0.012957	0.017954	0.017622	0.005822	0.016643155
Carbohydrates	Monosaccharides	Deoxyribose and Deoxynucleoside Catabolism	Pyrimidine-nucleoside phosphorylase (EC 2.4.2.2)	0.008704	0.013572	0.012774	0.01992	0.01707	0.017533	0.005055	0.017941	0.017954	0.013217	0.002096	0.009386424
Phosphorus Metabolism	NULL	Phosphate metabolism	Pyrophosphate-energized proton pump (EC 3.6.1.1)	0.172338	0.255604	0.173726	0.194223	0.245552	0.195303	0.184854	0.158977	0.146038	0.081503	0.005822	0.150734921
Carbohydrates	Central carbohydrate metabolism	Glycolysis and Gluconeogenesis, including Archaeal enzymes	Pyrophosphate--fructose 6-phosphate 1-phosphotransferase, alpha subunit (EC 2.7.1.90)	0.019497	0.052026	0.051096	0.062251	0.055151	0.050165	0.040437	0.026413	0.008101	0.006608	0.000116	0.022953355
Miscellaneous	Plant-Prokaryote DOE project	YggW	Pyrroline-5-carboxylate reductase (EC 1.5.1.2)	0.02263	0.03393	0.056206	0.01992	0.026919	0.027761	0.036826	0.032892	0.034813	0.052867	0.024451	0.03707243
Carbohydrates	Central carbohydrate metabolism	Pyruvate metabolism I: anaplerotic reactions, PEP	Pyruvate carboxyl transferase (EC 6.4.1.1)	0.037601	0.031668	0.043432	0.034861	0.033484	0.046756	0.059933	0.05482	0.058897	0.024231	0.009664	0.034784982
Carbohydrates	Central carbohydrate metabolism	Pyruvate metabolism I: anaplerotic reactions, PEP	Pyruvate carboxyl transferase subunit A (EC 6.4.1.1)	0.023675	0.02262	0.020438	0.034861	0.019697	0.019969	0.016608	0.023921	0.028244	0.092517	0.091284	0.045078497
Carbohydrates	Central carbohydrate metabolism	Pyruvate metabolism I: anaplerotic reactions, PEP	Pyruvate carboxyl transferase subunit B (EC 6.4.1.1)	0.067891	0.058812	0.051096	0.047311	0.040706	0.04286	0.021663	0.021429	0.022989	0.026433	0.095825	0.043264314
Cofactors, Vitamins, Prosthetic Groups, Pigments	Folate and pterines	5-FCL-like protein	Pyruvate dehydrogenase E1 component (EC 1.2.4.1)	0.108625	0.126671	0.166062	0.082171	0.082726	0.102765	0.134308	0.114623	0.161145	0.066084	0.224019	0.120169886
Carbohydrates	Central carbohydrate metabolism	Pyruvate metabolism II: acetyl-CoA, acetogenesis from pyruvate	Pyruvate dehydrogenase E1 component alpha subunit (EC 1.2.4.1)	0.028201	0.009048	0.038322	0.02988	0.032828	0.029709	0.054878	0.054321	0.043133	0.116748	0.015719	0.033680697
Carbohydrates	Central carbohydrate metabolism	Dehydrogenase complexes	Pyruvate dehydrogenase E1 component beta subunit (EC 1.2.4.1)	0.038994	0.047502	0.053651	0.047311	0.045302	0.044321	0.052712	0.077246	0.080135	0.116748	0.014205	0.051507014
Carbohydrates	Fermentation	Butanol Biosynthesis	Pyruvate formate-lyase (EC 2.3.1.54)	0.075898	0.133457	0.084308	0.131972	0.113584	0.109097	0.076541	0.050833	0.016421	0.033042	0.022937	0.089880923
Carbohydrates	Fermentation	Fermentations: Mixed acid	Pyruvate formate-lyase activating enzyme (EC 1.97.1.4)	0.007659	0.027144	0.010219	0.01743	0.011161	0.024352	0.015886	0.008472	0.000876	0.013217	0.005705	0.015578308
Carbohydrates	Organic acids	Glycerate metabolism	Pyruvate kinase (EC 2.7.1.40)	0.099225	0.104051	0.125185	0.126992	0.122776	0.098869	0.104702	0.126085	0.103781	0.140978	0.157884	0.113859686
Carbohydrates	Central carbohydrate metabolism	Pyruvate metabolism II: acetyl-CoA, acetogenesis from pyruvate	Pyruvate oxidase [ubiquinone, cytochrome] (EC 1.2.2.2)	0.002437	0.002262	0.00511	0.00498	0.013131	0.018508	0.004333	0.007974	0.011604	0.017622	0.001048	0.004929844
Cell Wall and Capsule	Capsular and extracellular polysacchrides	O-Methyl Phosphoramidate Capsule Modification in Campylobacter	Pyruvate phosphate dikinase	0.01706	0.038454	0.033212	0.039841	0.023636	0.0263	0.022385	0.010466	0.008758	0.006608	0.000116	0.029184679
Carbohydrates	Central carbohydrate metabolism	Glycolysis and Gluconeogenesis	Pyruvate,phosphate dikinase (EC 2.7.9.1)	0.232918	0.269176	0.158398	0.201693	0.216007	0.228909	0.197129	0.167947	0.143191	0.074895	0.03004	0.173293889
Carbohydrates	Central carbohydrate metabolism	Pyruvate:ferredoxin oxidoreductase	Pyruvate:ferredoxin oxidoreductase, alpha subunit (EC 1.2.7.1)	0.034816	0.036192	0.025548	0.054781	0.053181	0.032145	0.03466	0.006479	0.01248	0.008811	0.002096	0.036520287
Carbohydrates	Central carbohydrate metabolism	Pyruvate:ferredoxin oxidoreductase	Pyruvate:ferredoxin oxidoreductase, beta subunit (EC 1.2.7.1)	0.037949	0.054288	0.038322	0.057271	0.053181	0.035067	0.032494	0.008472	0.014232	0.026433	0.001164	0.034508911
Amino Acids and Derivatives	Lysine, threonine, methionine, and cysteine	Methionine Degradation	Pyruvate-flavodoxin oxidoreductase (EC 1.2.7.-)	0.279919	0.325726	0.229932	0.281375	0.217976	0.211375	0.208682	0.116616	0.125238	0.0793	0.174651	0.233990133
RNA Metabolism	RNA processing and modification	Queuosine-Archaeosine Biosynthesis	Queuosine Biosynthesis QueC ATPase	0.028201	0.015834	0.071534	0.039841	0.063686	0.041885	0.047658	0.035384	0.027806	0.035245	0.027711	0.032418657
Miscellaneous	Plant-Prokaryote DOE project	COG3146 experimental	Queuosine biosynthesis QueD, PTPS-I	0.007659	0.013572	0.017884	0.01743	0.015757	0.01802	0.01733	0.018938	0.006787	0.022028	0.003377	0.022953355
RNA Metabolism	RNA processing and modification	tRNA modification Bacteria	Queuosine Biosynthesis QueE Radical SAM	0.026112	0.02262	0.022993	0.0249	0.034141	0.023378	0.035382	0.043856	0.028463	0.006608	0.010945	0.033956768
Cofactors, Vitamins, Prosthetic Groups, Pigments	NAD and NADP	NAD regulation	Quinolinate phosphoribosyltransferase [decarboxylating] (EC 2.4.2.19)	0.085647	0.040716	0.051096	0.049801	0.031515	0.050165	0.063543	0.058308	0.040943	0.017622	0.053909	0.052966248
Cofactors, Vitamins, Prosthetic Groups, Pigments	NAD and NADP	NAD and NADP cofactor biosynthesis global	Quinolinate synthetase (EC 2.5.1.72)	0.042475	0.063336	0.038322	0.079681	0.06106	0.037015	0.061377	0.059803	0.063495	0.024231	0.066484	0.058842622
Respiration	NULL	Quinone oxidoreductase family	Quinone oxidoreductase (EC 1.6.5.5)	0.030638	0.024882	0.076644	0.01992	0.039393	0.038963	0.038993	0.036879	0.0624	0.026433	0.003493	0.025635191
Respiration	Electron donating reactions	Hydrogenases	Quinone-reactive Ni/Fe-hydrogenase large chain (EC 1.12.5.1)	0.012882	0.013572	0.020438	0.01992	0.013788	0.00828	0.002166	0.003987	0.005912	0.006608	0.000931	0.024530906
Cofactors, Vitamins, Prosthetic Groups, Pigments	Tetrapyrroles	Heme biosynthesis orphans	Radical SAM domain heme biosynthesis protein	0.031334	0.049764	0.030658	0.02739	0.044646	0.040911	0.033216	0.014452	0.009415	0.011014	0.003842	0.02725218
Stress Response	Heat shock	Heat shock dnaK gene cluster extended	Radical SAM family enzyme, similar to coproporphyrinogen III oxidase, oxygen-independent, clustered with nucleoside-triphosphatase RdgB	0.021238	0.02262	0.022993	0.054781	0.030858	0.016559	0.021663	0.029902	0.02912	0.01542	0.055306	0.021691315
Cell Wall and Capsule	NULL	Peptidoglycan Biosynthesis	Rare lipoprotein A precursor	0.008356	0.004524	0.015329	0.01494	0.007879	0.01802	0.025273	0.020433	0.015764	0.006608	0.02154	0.012896473
Miscellaneous	Plant-Prokaryote DOE project	Competence or DNA damage-inducible protein CinA and related protein families	RecA protein	0.124989	0.137981	0.117521	0.107072	0.152321	0.103252	0.155248	0.144525	0.114509	0.136573	0.120742	0.110586269
DNA Metabolism	DNA repair	DNA repair, bacterial RecBCD pathway	RecD-like DNA helicase YrrC	0.026112	0.079169	0.074089	0.067231	0.079443	0.088641	0.05199	0.042361	0.031309	0.008811	0.001281	0.051783085
Clustering-based subsystems	Recombination related cluster	CBSS-198094.1.peg.4426	Recombination inhibitory protein MutS2	0.014623	0.047502	0.035767	0.067231	0.03808	0.031171	0.011553	0.023423	0.029996	0.081503	0.007685	0.059986346
DNA Metabolism	DNA replication	DNA-replication	Recombination protein RecR	0.034468	0.042978	0.048541	0.034861	0.040706	0.041885	0.047658	0.039869	0.043789	0.030839	0.031204	0.052295789
DNA Metabolism	DNA replication	DNA-replication	Replicative DNA helicase (EC 3.6.1.-)	0.054661	0.024882	0.061315	0.02988	0.06106	0.086693	0.106868	0.139043	0.051015	0.081503	0.035279	0.052650738
DNA Metabolism	DNA replication	DNA-replication	Replicative DNA helicase (EC 3.6.1.-) [SA14-24]	0.004526	0.02262	0.012774	0.01245	0.016414	0.017046	0.01372	0.013954	0.006131	0.002203	0.000233	0.007217292
Nitrogen Metabolism	NULL	Nitrate and nitrite ammonification	Respiratory nitrate reductase alpha chain (EC 1.7.99.4)	0.014971	0.013572	0.045986	0.01743	0.042676	0.028735	0.03105	0.028905	0.027587	0.006608	0.005822	0.020113765
Nitrogen Metabolism	NULL	Nitrate and nitrite ammonification	Respiratory nitrate reductase beta chain (EC 1.7.99.4)	0.009748	0.004524	0.00511	0.01245	0.015757	0.010228	0.01372	0.011462	0.017078	0.013217	0.002562	0.010490709
Virulence, Disease and Defense	Resistance to antibiotics and toxic compounds	Zinc resistance	Response regulator of zinc sigma-54-dependent two-component system	0.122552	0.128933	0.137959	0.102092	0.147068	0.116403	0.091705	0.056813	0.122172	0.004406	0.093263	0.143202119
RNA Metabolism	NULL	Group II intron-associated genes	Retron-type reverse transcriptase	0.068935	0.110837	0.153288	0.087151	0.207472	0.154392	0.116978	0.068275	0.056926	0.008811	0.021075	0.066612057
Carbohydrates	Monosaccharides	L-rhamnose utilization	rhamnogalacturonan acetylesterase	0.001741	0.031668	0.007664	0.00747	0.005909	0.002435	0.00361	0.004984	0.008101	0.017622	0.000582	0.012107698
Carbohydrates	Monosaccharides	D-Galacturonate and D-Glucuronate Utilization	Rhamnogalacturonides degradation protein RhiN	0.004178	0.027144	0.017884	0.01992	0.011161	0.006332	0.005055	0.004984	0.013794	0.006608	0.000466	0.018181266
Cofactors, Vitamins, Prosthetic Groups, Pigments	Folate and pterines	YgfZ	Rhodanese-related sulfurtransferase	0.021586	0.020358	0.033212	0.00996	0.032171	0.031171	0.028161	0.024918	0.024741	0.006608	0.001514	0.027370496
Cofactors, Vitamins, Prosthetic Groups, Pigments	Riboflavin, FMN, FAD	Riboflavin, FMN and FAD metabolism	Riboflavin kinase (EC 2.7.1.26)	0.008704	0.006786	0.012774	0.0249	0.009192	0.01315	0.008665	0.010466	0.013575	0.017622	0.010363	0.014395146
Cofactors, Vitamins, Prosthetic Groups, Pigments	Riboflavin, FMN, FAD	Riboflavin, FMN and FAD metabolism	Riboflavin synthase alpha chain (EC 2.5.1.9)	0.033423	0.03393	0.015329	0.037351	0.030202	0.033606	0.044047	0.045849	0.030871	0.024231	0.020492	0.043816457
Carbohydrates	Monosaccharides	Deoxyribose and Deoxynucleoside Catabolism	Ribokinase (EC 2.7.1.15)	0.021586	0.054288	0.048541	0.01743	0.047272	0.050652	0.036826	0.055816	0.03744	0.037447	0.004424	0.034430033
Clustering-based subsystems	NULL	CBSS-176280.1.peg.1561	Ribonuclease BN (EC 3.1.-.-)	0.10201	0.042978	0.033212	0.054781	0.049898	0.036528	0.025273	0.032892	0.040286	0.013217	0.03493	0.03991202
RNA Metabolism	RNA processing and modification	tRNA processing	Ribonuclease D (EC 3.1.26.3)	0.01706	0.027144	0.012774	0.02241	0.022979	0.019969	0.026717	0.023423	0.027368	0.006608	0.03691	0.016761471
Protein Metabolism	Protein biosynthesis	Ribosome biogenesis bacterial	Ribonuclease E (EC 3.1.26.12)	0.045609	0.072383	0.071534	0.047311	0.060403	0.058932	0.046213	0.045351	0.059554	0.026433	0.114687	0.047563138

RNA Metabolism	RNA processing and modification	Ribonuclease H	Ribonuclease HI (EC 3.1.26.4)	0.047001	0.02262	0.038322	0.042331	0.029545	0.026787	0.033938	0.035882	0.032185	0.026433	0.030622	0.033522942
DNA Metabolism	DNA replication	DNA replication, archaeal	Ribonuclease HII (EC 3.1.26.4)	0.027853	0.027144	0.053651	0.039841	0.047929	0.046269	0.053434	0.058308	0.032842	0.035245	0.029923	0.042672733
Clustering-based subsystems	Probably GTP or GMP signaling related	CBSS-176299.4.peg.1292	Ribonuclease III (EC 3.1.26.3)	0.027156	0.018096	0.05876	0.042331	0.035454	0.035067	0.046935	0.029403	0.027587	0.074895	0.05356	0.047247628
RNA Metabolism	RNA processing and modification	tRNA processing	Ribonuclease PH (EC 2.7.7.56)	0.063713	0.047502	0.066425	0.02988	0.04202	0.037015	0.04838	0.046348	0.055831	0.01542	0.049484	0.029302995
RNA Metabolism	RNA processing and modification	tRNA processing	Ribonuclease Z (EC 3.1.26.11)	0.006615	0.01131	0.002555	0.039841	0.013131	0.015098	0.012275	0.011462	0.014669	0.017622	0.00163	0.027212741
Nucleosides and Nucleotides	NULL	Ribonucleotide reduction	Ribonucleotide reductase of class Ia (aerobic), alpha subunit (EC 1.17.4.1)	0.023327	0.036192	0.066425	0.044821	0.085352	0.151469	0.16969	0.174426	0.079478	0.279754	0.203992	0.104315507
Clustering-based subsystems	NULL	Ribonucleotide reductase cluster	Ribonucleotide reductase of class Ia (aerobic), beta subunit (EC 1.17.4.1)	0.047698	0.018096	0.043432	0.032371	0.043333	0.073543	0.109757	0.090702	0.038097	0.297376	0.087092	0.056791807
Nucleosides and Nucleotides	NULL	Ribonucleotide reduction	Ribonucleotide reductase of class II (coenzyme B12-dependent) (EC 1.17.4.1)	0.232221	0.23977	0.229932	0.256474	0.387368	0.366254	0.44697	0.429088	0.229894	0.143181	0.017232	0.167811902
Nucleosides and Nucleotides	NULL	Ribonucleotide reduction	Ribonucleotide reductase of class III (anaerobic), large subunit (EC 1.17.4.2)	0.022978	0.058812	0.061315	0.064741	0.102423	0.085232	0.080873	0.048341	0.006131	0.033042	0.010945	0.059670836
Nucleosides and Nucleotides	NULL	Ribonucleotide reduction	Ribonucleotide reductase transcriptional regulator NrdR	0.032031	0.013572	0.038322	0.01992	0.036111	0.038963	0.028161	0.0304	0.03109	0.008811	0.01339	0.026581721
Carbohydrates	CO2 fixation	Calvin-Benson cycle	Ribose 5-phosphate isomerase A (EC 5.3.1.6)	0.071721	0.020358	0.022993	0.02241	0.02101	0.018508	0.023107	0.021429	0.026274	0.033042	0.035279	0.021336366
Carbohydrates	CO2 fixation	Calvin-Benson cycle	Ribose 5-phosphate isomerase B (EC 5.3.1.6)	0.034119	0.02262	0.012774	0.01494	0.022979	0.021917	0.020218	0.029902	0.014232	0.033042	0.00163	0.024057641
Carbohydrates	NULL	Sugar utilization in Thermotogales	Ribose ABC transport system, ATP-binding protein RbsA (TC 3.A.1.2.1)	0.021934	0.024882	0.025548	0.047311	0.021666	0.020456	0.023107	0.024918	0.034375	0.011014	0.002329	0.031314371
Carbohydrates	NULL	Sugar utilization in Thermotogales	Ribose ABC transport system, periplasmic ribose-binding protein RbsB (TC 3.A.1.2.1)	0.00383	0.02262	0.010219	0.00996	0.003283	0.006332	0.005777	0.004984	0.005693	0.002203	0.014554	0.007177853
Carbohydrates	Monosaccharides	D-ribose utilization	Ribose ABC transport system, permease protein RbsC (TC 3.A.1.2.1)	0.020193	0.038454	0.053651	0.059761	0.04202	0.038476	0.016608	0.036879	0.049701	0.044056	0.049717	0.029736821
Carbohydrates	Monosaccharides	D-ribose utilization	Ribose operon repressor	0.000348	0.004524	0.002555	0.00996	0.004596	0.001948	0.00361	0.00299	0.007225	0.002203	0.000233	0.004732651
Carbohydrates	Central carbohydrate metabolism	Pentose phosphate pathway	Ribose-phosphate pyrophosphokinase (EC 2.7.6.1)	0.154582	0.106313	0.125185	0.092131	0.101766	0.092538	0.098926	0.125587	0.097869	0.09472	0.094311	0.101436478
Cell Division and Cell Cycle	NULL	Two cell division clusters relating to chromosome partitioning	Ribosomal large subunit pseudouridine synthase B (EC 4.2.1.70)	0.025067	0.027144	0.028103	0.02739	0.023636	0.029709	0.028883	0.023921	0.02912	0.022028	0.049019	0.035889267
RNA Metabolism	RNA processing and modification	RNA pseudouridine syntheses	Ribosomal large subunit pseudouridine synthase C (EC 4.2.1.70)	0.005571	0.013572	0.002555	0.01494	0.015101	0.006819	0.030328	0.030898	0.028244	0.017622	0.063573	0.014828972
Protein Metabolism	Protein biosynthesis	Ribosome biogenesis bacterial	Ribosomal large subunit pseudouridine synthase D (EC 4.2.1.70)	0.098181	0.079169	0.086863	0.079681	0.046615	0.059419	0.054878	0.070269	0.061086	0.030839	0.061011	0.071344708
RNA Metabolism	RNA processing and modification	RNA pseudouridine syntheses	Ribosomal large subunit pseudouridine synthase E (EC 4.2.1.70)	0.02263	0.002262	0.007664	0.00498	0.004596	0.006332	0.010109	0.010964	0.01248	0.008811	0.015602	0.009780811
Stress Response	Heat shock	Heat shock dnaK gene cluster extended	Ribosomal protein L11 methyltransferase (EC 2.1.1.-)	0.051876	0.02262	0.045986	0.044821	0.020353	0.037015	0.028883	0.021429	0.033499	0.112342	0.036444	0.042041713
Protein Metabolism	Protein processing and modification	Ribosomal protein S12p Asp methylthiotransferase	Ribosomal protein S12p Asp88 (E. coli) methylthiotransferase	0.069283	0.095003	0.051096	0.067231	0.055807	0.061854	0.068598	0.048839	0.055831	0.088112	0.024335	0.087199087
Protein Metabolism	Protein biosynthesis	Ribosome biogenesis bacterial	Ribosomal protein S6 glutaminyl transferase	0.084254	0.036192	0.043432	0.034861	0.052524	0.044808	0.073653	0.072262	0.07269	0.03965	0.107119	0.072251799
Clustering-based subsystems	NULL	Conserved gene cluster associated with Met-tRNA formyltransferase	Ribosomal RNA large subunit methyltransferase N (EC 2.1.1.-)	0.037601	0.036192	0.025548	0.037351	0.022979	0.027274	0.021663	0.041364	0.022989	0.033042	0.044943	0.045512323
Clustering-based subsystems	NULL	Conserved gene cluster associated with Met-tRNA formyltransferase	Ribosomal RNA small subunit methyltransferase B (EC 2.1.1.-)	0.032727	0.02262	0.017884	0.02241	0.023636	0.016559	0.018052	0.02442	0.029339	0.006608	0.0333	0.019048919
Clustering-based subsystems	NULL	CBSS-269801.1.peg.1715	Ribosomal RNA small subunit methyltransferase D (EC 2.1.1.-)	0.018801	0.024882	0.020438	0.02241	0.012475	0.02143	0.012275	0.024918	0.022114	0.017622	0.005123	0.022322335
Stress Response	Heat shock	Heat shock dnaK gene cluster extended	Ribosomal RNA small subunit methyltransferase E (EC 2.1.1.-)	0.008008	0.020358	0.015329	0.00747	0.012475	0.012176	0.018774	0.022426	0.024084	0.030839	0.02154	0.02342662
Protein Metabolism	Protein biosynthesis	Ribosome biogenesis bacterial	Ribosomal-protein-S5p-alanine acetyltransferase	0.00383	0.002262	0.002555	0.00498	0.005909	0.003896	0.00361	0.00598	0.007225	0.002203	0.001397	0.003391733
Clustering-based subsystems	NULL	CBSS-176299.4.peg.1996B	Ribosome recycling factor	0.03586	0.049764	0.051096	0.064741	0.053838	0.050165	0.043325	0.046846	0.038973	0.154195	0.056354	0.055963593
Clustering-based subsystems	Ribosomal Protein L28P relates to a set of uncharacterized proteins	A Gram-positive cluster that relates ribosomal protein L28P to a set of uncharacterized proteins	Ribosome small subunit-stimulated GTPase EngC	0.016363	0.015834	0.020438	0.01494	0.015101	0.013637	0.018052	0.020931	0.029558	0.105734	0.04669	0.036007583
Clustering-based subsystems	NULL	NusA-TFII Cluster	Ribosome-binding factor A	0.016712	0.01131	0.010219	0.00498	0.009848	0.009741	0.008665	0.012957	0.013137	0.030839	0.000116	0.016919226
Cofactors, Vitamins, Prosthetic Groups, Pigments	NAD and NADP	NAD and NADP cofactor biosynthesis global	Ribosylnicotinamide kinase (EC 2.7.1.22)	0.001393	0.009048	0.00511	0.01245	0.007879	0.006332	0.006499	0.004984	0.005474	0.052867	0.003493	0.01900948
Miscellaneous	Plant-Prokaryote DOE project	COG3533	Ribulokinase (EC 2.7.1.16)	0.037949	0.049764	0.056206	0.094622	0.032828	0.01802	0.018052	0.020931	0.028025	0.035245	0.025965	0.0278832
Carbohydrates	CO2 fixation	Calvin-Benson cycle	Ribulose bisphosphate carboxylase large chain (EC 4.1.1.39)	0.03969	0.004524	0.020438	0.059761	0.011161	0.01315	0.015164	0.009967	0.008977	0.006608	0.001281	0.016248767
Miscellaneous	Plant-Prokaryote DOE project	COG0451	Ribulose-5-phosphate 4-epimerase and related epimerases and aldolases	0.027853	0.024882	0.028103	0.0249	0.01707	0.025326	0.029605	0.034387	0.038754	0.011014	0.022006	0.012502085
Carbohydrates	CO2 fixation	Calvin-Benson cycle	Ribulose-phosphate 3-epimerase (EC 5.1.3.1)	0.042823	0.052026	0.071534	0.087151	0.051211	0.063315	0.057045	0.0608	0.052328	0.072692	0.041567	0.055135379
RNA Metabolism	RNA processing and modification	rRNA modification Bacteria	RNA binding methyltransferase FtsJ like	0.018104	0.018096	0.020438	0.042331	0.019697	0.019969	0.018774	0.014452	0.012699	0.013217	0.002911	0.016722032
Miscellaneous	Plant-Prokaryote DOE project	At3g21300	RNA methyltransferase, TrmA family	0.001741	0.03393	0.017884	0.032371	0.014444	0.029709	0.012998	0.021429	0.017735	0.068286	0.019328	0.033917329
Clustering-based subsystems	Translation	CBSS-326442.4.peg.1852	RNA polymerase associated protein RapA (EC 3.6.1.-)	0.01706	0.006786	0.030658	0.02739	0.017727	0.019482	0.023829	0.03339	0.040286	0.004406	0.102927	0.033877891
Stress Response	Oxidative stress	Regulation of Oxidative Stress Response	RNA polymerase sigma factor	0.100618	0.158339	0.155843	0.174303	0.154947	0.164619	0.125643	0.142531	0.13509	0.222482	0.098503	0.138153959
Motility and Chemotaxis	Flagellar motility in Prokaryota	Flagellar motility	RNA polymerase sigma factor for flagellar operon	0.03029	0.006786	0.022993	0.01494	0.022323	0.01802	0.025995	0.026413	0.022114	0.01542	0.015369	0.020468714
Motility and Chemotaxis	Flagellar motility in Prokaryota	Flagellum	RNA polymerase sigma factor RpoD	0.063365	0.101789	0.109856	0.072211	0.11818	0.088154	0.106146	0.098675	0.092177	0.14979	0.092216	0.080178989
RNA Metabolism	Transcription	Transcription initiation, bacterial sigma factors	RNA polymerase sigma factor RpoE	0.025067	0.015834	0.002555	0.0249	0.012475	0.014124	0.012275	0.017443	0.016202	0.017622	0.02352	0.010214638
Stress Response	Heat shock	Heat shock dnaK gene cluster extended	RNA polymerase sigma factor RpoH	0.040734	0.027144	0.025548	0.047311	0.025606	0.035554	0.029605	0.032393	0.037002	0.0793	0.0248	0.026423966
Clustering-based subsystems	NULL	CBSS-211586.1.peg.3133	RNA polymerase sigma factor RpoS	0.06232	0.006786	0.017884	0.00249	0.015101	0.012663	0.010831	0.017941	0.02912	0.037447	0.058683	0.014040197
Motility and Chemotaxis	Flagellar motility in Prokaryota	Flagellar motility	RNA polymerase sigma-54 factor RpoN	0.080076	0.104051	0.117521	0.067231	0.084696	0.090102	0.091705	0.104157	0.103562	0.090314	0.053909	0.091734544
Clustering-based subsystems	Putative asociate of RNA polymerase sigma-54 factor rpoN	CBSS-316057.3.peg.1308	RNA polymerase sigma-70 factor	0.049438	0.018096	0.043432	0.034861	0.027575	0.033606	0.019496	0.050334	0.084295	0.103531	0.002329	0.045236252
Protein Metabolism	Protein biosynthesis	Ribosome biogenesis bacterial	RNA pseudouridylate synthase, group 1	0.002437	0.013572	0.002555	0.01245	0.003283	0.00828	0.002166	0.011462	0.006568	0.028636	0.000582	0.013724687
Stress Response	NULL	Hfl operon	RNA-binding protein Hfq	0.070676	0.006786	0.00511	0.01494	0.009848	0.011202	0.010831	0.015449	0.009853	0.006608	0.002562	0.007848312
Miscellaneous	NULL	ZZ gjo need homes	RNase L inhibitor	0.000348	0.009048	0.015329	0.01245	0.007222	0.008767	0.02094	0.010964	0.004598	0.01542	0.001164	0.00761168
Virulence, Disease and Defense	Resistance to antibiotics and toxic compounds	Multidrug efflux pump in Campylobacter jejuni (CmeABC operon)	RND efflux system, inner membrane transporter CmeB	0.094699	0.124409	0.071534	0.109562	0.026919	0.035554	0.051268	0.076748	0.114728	0.037447	0.108749	0.107115658
Virulence, Disease and Defense	Resistance to antibiotics and toxic compounds	Multidrug efflux pump in Campylobacter jejuni (CmeABC operon)	RND efflux system, outer membrane lipoprotein CmeC	0.016015	0.024882	0.007664	0.01992	0.007222	0.010715	0.001444	0.006977	0.011166	0.002203	0.001514	0.016958665
Virulence, Disease and Defense	Resistance to antibiotics and toxic compounds	Multidrug Resistance Efflux Pumps	RND efflux system, outer membrane lipoprotein, NodT family	0.000348	0.004524	0.00511	0.00498	0.000657	0.000974	0.002888	0.000997	0.007444	0.013217	0.009664	0.003549488
Cell Division and Cell Cycle	NULL	Bacterial Cytoskeleton	Rod shape-determining protein MreB	0.193228	0.137981	0.153288	0.114542	0.146412	0.110071	0.128531	0.111134	0.088673	0.209265	0.107701	0.108850964
Clustering-based subsystems	NULL	Bacterial Cell Division	Rod shape-determining protein MreC	0.014623	0.013572	0.007664	0.01245	0.019697	0.020943	0.012275	0.019436	0.021676	0.011014	0.014787	0.018536215

Cell Wall and Capsule	NULL	Peptidoglycan Biosynthesis	Rod shape-determining protein RodA	0.072417	0.040716	0.045986	0.062251	0.066312	0.060393	0.057767	0.064787	0.051671	0.046259	0.059847	0.055687522
Clustering-based subsystems	NULL	Conserved gene cluster associated with Met-tRNA formyltransferase	Rossmann fold nucleotide-binding protein Smf possibly involved in DNA uptake	0.053964	0.029406	0.030658	0.034861	0.047929	0.050165	0.03466	0.036879	0.034156	0.017622	0.018047	0.029539627
RNA Metabolism	RNA processing and modification	Ribosome biogenesis archaeal	rRNA biogenesis protein Nop5/Nop56	0.001393	0.009048	0.002555	0.00747	0.003939	0.000974	0.004333	0.003987	0.001095	0.002203	0.000466	0.001025408
RNA Metabolism	RNA processing and modification	RNA methylation	rRNA small subunit 7-methylguanosine (m7G) methyltransferase GidB	0.060231	0.002262	0.022993	0.01245	0.014444	0.014124	0.022385	0.022426	0.025617	0.063881	0.026663	0.028277587
Clustering-based subsystems	NULL	Bacterial Cell Division	rRNA small subunit methyltransferase H	0.071721	0.065598	0.06387	0.059761	0.047272	0.053574	0.056323	0.074754	0.040724	0.052867	0.045526	0.060853999
RNA Metabolism	RNA processing and modification	16S rRNA modification within P site of ribosome	rRNA small subunit methyltransferase I	0.050483	0.04524	0.048541	0.062251	0.033484	0.034093	0.057045	0.048839	0.043133	0.068286	0.038307	0.050875994
Stress Response	Oxidative stress	Rubrerythrin	Rubrerythrin	0.090869	0.137981	0.076644	0.122012	0.107675	0.110558	0.098926	0.052826	0.031528	0.033042	0.005589	0.077221082
Cofactors, Vitamins, Prosthetic Groups, Pigments	Folate and pterines	YgfZ	S-(hydroxymethyl)glutathione dehydrogenase (EC 1.1.1.284)	0.061624	0.018096	0.015329	0.02241	0.011818	0.01802	0.028161	0.031397	0.045103	0.019825	0.067415	0.020508153
Clustering-based subsystems	NULL	CBSS-291331.3.peg.3674	S-adenosyl-L-methionine dependent methyltransferase, similar to cyclopropane-fatty-acyl-phospholipid synthase	0.014971	0.01131	0.038322	0.01992	0.025606	0.036041	0.028161	0.041364	0.038316	0.022028	0.011643	0.023505498
Amino Acids and Derivatives	Arginine; urea cycle, polyamines	Polyamine Metabolism	S-adenosylmethionine decarboxylase proenzyme (EC 4.1.1.50), prokaryotic class 1A	0.016015	0.004524	0.020438	0.00498	0.00197	0.002435	0.00361	0.002492	0.000876	0.004406	0.004541	0.006625711
Amino Acids and Derivatives	Arginine; urea cycle, polyamines	Polyamine Metabolism	S-adenosylmethionine decarboxylase proenzyme (EC 4.1.1.50), prokaryotic class 1B	0.015319	0.004524	0.007664	0.00249	0.007879	0.014124	0.009387	0.017443	0.007006	0.006608	0.000349	0.004298824
Amino Acids and Derivatives	Lysine, threonine, methionine, and cysteine	Methionine Biosynthesis	S-adenosylmethionine synthetase (EC 2.5.1.6)	0.230481	0.158339	0.140514	0.174303	0.170704	0.188484	0.179077	0.155987	0.14757	0.110139	0.107701	0.148447474
RNA Metabolism	RNA processing and modification	tRNA modification Bacteria	S-adenosylmethionine:tRNA ribosyltransferase-isomerase (EC 5.-.-.-)	0.092262	0.063336	0.094528	0.057271	0.068282	0.075978	0.089538	0.081731	0.091739	0.041853	0.050416	0.084122864
Miscellaneous	Plant-Prokaryote DOE project	Niacin-Choline transport and metabolism	Sarcosine dehydrogenase (EC 1.5.99.1)	0.027853	0.024882	0.051096	0.02739	0.043333	0.046756	0.044769	0.041862	0.040724	0.004406	0.000582	0.021770193
Stress Response	Osmotic stress	Choline and Betaine Uptake and Betaine Biosynthesis	Sarcosine oxidase alpha subunit (EC 1.5.3.1)	0.018104	0.020358	0.038322	0.02988	0.036767	0.050652	0.063543	0.084223	0.070063	0.026433	0.449435	0.023702692
Stress Response	Osmotic stress	Choline and Betaine Uptake and Betaine Biosynthesis	Sarcosine oxidase beta subunit (EC 1.5.3.1)	0.051179	0.018096	0.010219	0.00747	0.015757	0.019482	0.033216	0.041364	0.032185	0.028636	0.118413	0.011555555
Miscellaneous	Plant-Prokaryote DOE project	Iron-sulfur cluster assembly	Scaffold protein for [4Fe-4S] cluster assembly ApbC, MRP-like	0.039342	0.018096	0.017884	0.02241	0.018384	0.028248	0.027439	0.03638	0.033937	0.017622	0.022937	0.019719377
Miscellaneous	Plant-Prokaryote DOE project	Scaffold proteins for [4Fe-4S] cluster assembly (MRP family)	Scaffold protein for [4Fe-4S] cluster assembly, MRP-like, similar to chloroplast-targeted plant protein HCF101	0.010793	0.013572	0.007664	0.01494	0.010505	0.006819	0.015886	0.014951	0.016859	0.006608	0.001281	0.006941221
Cell Division and Cell Cycle	NULL	Two cell division clusters relating to chromosome partitioning	Segregation and condensation protein A	0.028897	0.031668	0.028103	0.042331	0.045959	0.040424	0.046213	0.042361	0.037002	0.035245	0.002562	0.028474781
Cell Division and Cell Cycle	NULL	Two cell division clusters relating to chromosome partitioning	Segregation and condensation protein B	0.024023	0.03393	0.038322	0.02988	0.038737	0.0263	0.024551	0.044852	0.02912	0.006608	0.002678	0.020192643
Protein Metabolism	Selenoproteins	Selenocysteine metabolism	Selenide,water dikinase (EC 2.7.9.3)	0.019149	0.036192	0.028103	0.034861	0.030202	0.028735	0.03105	0.02442	0.032842	0.013217	0.007102	0.0278832
Protein Metabolism	Selenoproteins	Selenocysteine metabolism	selenocysteine-containing	0.014971	0.092741	0.056206	0.077191	0.094544	0.082797	0.044769	0.02442	0.01664	0.006608	0.000699	0.064048538
Protein Metabolism	Selenoproteins	Selenocysteine metabolism	Selenocysteine-specific translation elongation factor	0.061276	0.03393	0.043432	0.054781	0.086009	0.039937	0.036826	0.016944	0.015326	0.006608	0.001397	0.035731512
RNA Metabolism	RNA processing and modification	tRNA modification Bacteria	Selenophosphate-dependent tRNA 2-selenouridine synthase	0.012186	0.002262	0.00511	0.01494	0.004596	0.007306	0.007943	0.008472	0.010509	0.011014	0.035396	0.007729996
Protein Metabolism	Selenoproteins	Selenoprotein O	Selenoprotein O and cysteine-containing homologs	0.027853	0.015834	0.010219	0.00996	0.030858	0.051626	0.05199	0.050833	0.036126	0.026433	0.11853	0.048036404
Cell Division and Cell Cycle	NULL	Bacterial Cytoskeleton	Septum formation protein Maf	0.058142	0.01131	0.038322	0.02241	0.042676	0.03458	0.026717	0.023921	0.026274	0.050664	0.02154	0.032063708
Cell Division and Cell Cycle	NULL	Bacterial Cytoskeleton	Septum site-determining protein MinD	0.026112	0.015834	0.030658	0.042331	0.011161	0.013637	0.01733	0.025915	0.032404	0.196048	0.02515	0.05797497
Amino Acids and Derivatives	Lysine, threonine, methionine, and cysteine	Methionine Biosynthesis	Serine acetyltransferase (EC 2.3.1.30)	0.024719	0.047502	0.043432	0.037351	0.037424	0.041885	0.045491	0.043357	0.037221	0.046259	0.037375	0.052414105
Miscellaneous	Plant-Prokaryote DOE project	Experimental - Histidine Degradation	Serine hydroxymethyltransferase (EC 2.1.2.1)	0.123248	0.110837	0.12774	0.129482	0.133281	0.149034	0.142251	0.141036	0.115166	0.215873	0.208882	0.133381869
Stress Response	NULL	SigmaB stress responce regulation	Serine phosphatase RsbU, regulator of sigma subunit	0.047349	0.090479	0.048541	0.049801	0.121463	0.129553	0.080151	0.058308	0.068092	0.01542	0.017931	0.071384146
RNA Metabolism	Transcription	Transcription initiation, bacterial sigma factors	Serine protease precursor MucD/AlgY associated with sigma factor RpoE	0.018801	0.004524	0.017884	0.01494	0.022323	0.011689	0.007221	0.015449	0.025179	0.006608	0.007102	0.016761471
Carbohydrates	Organic acids	Glycerate metabolism	Serine--glyoxylate aminotransferase (EC 2.6.1.45)	0.010793	0.015834	0.020438	0.047311	0.025606	0.033606	0.025995	0.018938	0.046636	0.030839	0.058799	0.013054228
Carbohydrates	One-carbon Metabolism	Serine-glyoxylate cycle	Serine--pyruvate aminotransferase (EC 2.6.1.51)	0.002785	0.004524	0.00511	0.00996	0.003283	0.006332	0.005777	0.003489	0.000657	0.002203	0.000233	0.004101631
Protein Metabolism	Protein biosynthesis	tRNA aminoacylation, Ser	Seryl-tRNA synthetase (EC 6.1.1.11)	0.102707	0.131195	0.135404	0.102092	0.110958	0.114941	0.146583	0.129574	0.090644	0.273146	0.076613	0.116068256
Stress Response	Detoxification	Glutathione-dependent pathway of formaldehyde detoxification	S-formylglutathione hydrolase (EC 3.1.2.12)	0.008704	0.006786	0.010219	0.00249	0.008535	0.00828	0.02094	0.029403	0.027806	0.017622	0.038889	0.008992036
Amino Acids and Derivatives	Aromatic amino acids and derivatives	Common Pathway For Synthesis of Aromatic Compounds (DAHP synthase to chorismate)	Shikimate 5-dehydrogenase I alpha (EC 1.1.1.25)	0.043172	0.03393	0.033212	0.01992	0.045959	0.035554	0.040437	0.043357	0.039848	0.01542	0.022239	0.041331815
Clustering-based subsystems	NULL	Shikimate kinase containing cluster	Shikimate kinase I (EC 2.7.1.71)	0.022282	0.013572	0.017884	0.0249	0.012475	0.020943	0.02094	0.030898	0.02693	0.022028	0.025965	0.024649222
Fatty Acids, Lipids, and Isoprenoids	NULL	Polyhydroxybutyrate metabolism	Short chain fatty acids transporter	0.002785	0.013572	0.010219	0.01992	0.000657	0.00487	0.00361	0.005482	0.009196	0.035245	0.003726	0.003864998
Sulfur Metabolism	NULL	Galactosylceramide and Sulfatide metabolism	Sialidase (EC 3.2.1.18)	0.001044	0.004524	0.010219	0.00996	0.004596	0.003409	0.004333	0.000498	0.001095	0.017622	0.000116	0.003904437
Membrane Transport	Protein secretion system, Type VIII (Extracellular nucleation/precipitation pathway, ENP)	Fap amyloid fiber secretion system	Sigma-54 dependent transcriptional regulator	0.032379	0.038454	0.033212	0.044821	0.042676	0.031171	0.028883	0.0304	0.056926	0.03965	0.099551	0.080612815
Virulence, Disease and Defense	Adhesion	Streptococcus pyogenes recombinatorial zone	Signal peptidase I	0.033423	0.024882	0.030658	0.01494	0.041363	0.041398	0.027439	0.025915	0.022114	0.013217	0.019561	0.037151307
Clustering-based subsystems	Probably GTP or GMP signaling related	CBSS-176299.4.peg.1292	Signal peptidase I (EC 3.4.21.89)	0.024023	0.027144	0.017884	0.02739	0.036111	0.041398	0.028883	0.029902	0.031747	0.006608	0.00978	0.031511565
Stress Response	Heat shock	Heat shock dnaK gene cluster extended	Signal peptidase-like protein	0.014274	0.01131	0.015329	0.01245	0.018384	0.012176	0.007221	0.007475	0.007663	0.006608	0.000699	0.011121729
Clustering-based subsystems	NULL	Bacterial Cell Division	Signal recognition particle receptor protein FtsY (=alpha subunit) (TC 3.A.5.1.1)	0.060928	0.092741	0.051096	0.069721	0.058433	0.069647	0.08665	0.074256	0.065684	0.136573	0.076148	0.070358739
Cell Division and Cell Cycle	NULL	Two cell division clusters relating to chromosome partitioning	Signal recognition particle, subunit Ffh SRP54 (TC 3.A.5.1.1)	0.129863	0.108575	0.176281	0.119522	0.110958	0.103739	0.101092	0.108144	0.112758	0.127762	0.112359	0.122299579
Motility and Chemotaxis	NULL	Bacterial Chemotaxis	Signal transduction histidine kinase CheA (EC 2.7.3.-)	0.071721	0.065598	0.043432	0.062251	0.128028	0.127117	0.130697	0.126085	0.10816	0.105734	0.081154	0.120879784
Respiration	Electron donating reactions	Hydrogenases	Signal transduction histidine kinase HoxJ (hydrogenase regulation)	0.0094	0.009048	0.002555	0.01245	0.016414	0.015585	0.010109	0.004984	0.007663	0.002203	0.020609	0.01234433
DNA Metabolism	DNA repair	2-phosphoglycolate salvage	Similar to phosphoglycolate phosphatase, clustered with ribosomal large subunit pseudouridine synthase C	0.001044	0.01131	0.002555	0.00498	0.004596	0.005357	0.007221	0.013456	0.011604	0.002203	0.016883	0.006507395
DNA Metabolism	DNA repair	DNA repair, bacterial RecFOR pathway	Single-stranded DNA-binding protein	0.070676	0.038454	0.056206	0.059761	0.078787	0.129553	0.156692	0.214295	0.036345	0.03965	0.025383	0.063930222
DNA Metabolism	DNA replication	DNA-replication	Single-stranded-DNA-specific exonuclease RecJ (EC 3.1.-.-)	0.092958	0.070121	0.061315	0.074701	0.087978	0.066237	0.088816	0.088708	0.077507	0.099125	0.086976	0.085621537
Phages, Prophages, Transposable elements, Plasmids	Phages, Prophages	Phage integration and excision	Site-specific recombinase	0.008008	0.020358	0.038322	0.02988	0.056464	0.022891	0.023829	0.030898	0.03328	0.019825	0.001747	0.020074326
Miscellaneous	Plant-Prokaryote DOE project	At1g26220 At1g32070	Site-specific recombinase XerD	0.02263	0.058812	0.061315	0.034861	0.031515	0.030196	0.036104	0.027908	0.027149	0.008811	0.054957	0.027922639
Phages, Prophages, Transposable elements, Plasmids	Phages, Prophages	r1t-like streptococcal phages	site-specific recombinase, phage integrase family	0.005571	0.015834	0.017884	0.00996	0.004596	0.005844	0.005777	0.006977	0.003503	0.002203	0.000815	0.009780811

Amino Acids and Derivatives	Glutamine, glutamate, aspartate, asparagine; ammonia assimilation	Glutamate and Aspartate uptake in Bacteria	Sodium/glutamate symport protein	0.001741	0.004524	0.015329	0.01743	0.007879	0.011202	0.024551	0.025416	0.045322	0.002203	0.001281	0.025359119
Membrane Transport	Uni- Sym- and Antiporters	NhaA, NhaD and Sodium-dependent phosphate transporters	Sodium-dependent phosphate transporter	0.037601	0.029406	0.040877	0.037351	0.061716	0.068673	0.066432	0.049338	0.022552	0.01542	0.013273	0.047957526
Miscellaneous	Plant-Prokaryote DOE project	COG0451	Soluble lytic murein transglycosylase and related regulatory proteins (some contain LysM/invasin domains)	0.007311	0.01131	0.033212	0.00996	0.017727	0.032632	0.018774	0.014951	0.014669	0.013217	0.010712	0.014158513
Cell Wall and Capsule	NULL	Murein Hydrolases	Soluble lytic murein transglycosylase precursor (EC 3.2.1.-)	0.01323	0.009048	0.022993	0.00747	0.01904	0.01802	0.028161	0.022925	0.03109	0.006608	0.0248	0.01013576
Phosphorus Metabolism	NULL	Phosphate metabolism	Soluble pyridine nucleotide transhydrogenase (EC 1.6.1.1)	0.053268	0.020358	0.030658	0.01992	0.013131	0.016072	0.025995	0.025416	0.052547	0.024231	0.086743	0.021967387
Carbohydrates	Monosaccharides	D-Sorbitol(D-Glucitol) and L-Sorbose Utilization	Sorbitol-6-phosphate 2-dehydrogenase (EC 1.1.1.140)	0.004526	0.006786	0.002555	0.00249	0.002626	0.003409	0.00361	0.001495	0.004817	0.002203	0.000233	0.002997345
DNA Metabolism	DNA repair	DNA repair, bacterial	SOS-response repressor and protease LexA (EC 3.4.21.88)	0.040038	0.031668	0.048541	0.042331	0.035454	0.029222	0.030328	0.046846	0.034375	0.013217	0.020259	0.023268865
Sulfur Metabolism	NULL	Sulfur oxidation	SoxH protein, homolog	0.003133	0.002262	0.010219	0.00498	0.010505	0.01315	0.015164	0.00897	0.005036	0.004406	0.000116	0.007217292
Amino Acids and Derivatives	Arginine; urea cycle, polyamines	Polyamine Metabolism	Spermidine Putrescine ABC transporter permease component PotB (TC 3.A.1.11.1)	0.011837	0.031668	0.022993	0.0249	0.019697	0.027761	0.025273	0.017941	0.036783	0.008811	0.005356	0.016564277
Amino Acids and Derivatives	Arginine; urea cycle, polyamines	Polyamine Metabolism	Spermidine Putrescine ABC transporter permease component potC (TC 3.A.1.11.1)	0.017408	0.009048	0.015329	0.00747	0.014444	0.019969	0.025995	0.021928	0.033937	0.013217	0.007219	0.015814941
Amino Acids and Derivatives	Arginine; urea cycle, polyamines	Polyamine Metabolism	Spermidine synthase (EC 2.5.1.16)	0.02646	0.009048	0.020438	0.032371	0.031515	0.038963	0.042603	0.047843	0.032623	0.022028	0.075566	0.029815699
Cell Division and Cell Cycle	NULL	Bacterial Cytoskeleton	Sporulation initiation inhibitor protein Soj	0.066498	0.038454	0.017884	0.02241	0.026262	0.018995	0.018052	0.016944	0.012261	0.024231	0.003493	0.012147137
Fatty Acids, Lipids, and Isoprenoids	Isoprenoids	Carotenoids	Squalene--hopene cyclase (EC 5.4.99.17)	0.002785	0.01131	0.010219	0.00249	0.005909	0.010715	0.001444	0.003987	0.02277	0.013217	0.001048	0.004969283
Protein Metabolism	Protein biosynthesis	Ribosome SSU bacterial	SSU ribosomal protein S10p (S20e)	0.070676	0.03393	0.038322	0.034861	0.034141	0.034093	0.043325	0.038374	0.032842	0.030839	0.039937	0.036835797
Protein Metabolism	Protein biosynthesis	Ribosome SSU bacterial	SSU ribosomal protein S11p (S14e)	0.040386	0.04524	0.040877	0.042331	0.036767	0.031171	0.06932	0.049338	0.04576	0.044056	0.024102	0.035770951
Protein Metabolism	Protein biosynthesis	Ribosome SSU bacterial	SSU ribosomal protein S12p (S23e)	0.061972	0.049764	0.061315	0.037351	0.04202	0.038963	0.052712	0.043856	0.03328	0.022028	0.031437	0.031866514
Protein Metabolism	Protein biosynthesis	Ribosome SSU bacterial	SSU ribosomal protein S13p (S18e)	0.103403	0.04524	0.051096	0.02739	0.059747	0.050165	0.056323	0.053325	0.038754	0.057273	0.02643	0.037151307
Miscellaneous	Plant-Prokaryote DOE project	COG0523	SSU ribosomal protein S14p (S29e)	0.015319	0.004524	0.022993	0.00996	0.019697	0.018995	0.01733	0.027908	0.022333	0.061678	0.019794	0.018891164
Protein Metabolism	Protein biosynthesis	Ribosome SSU bacterial	SSU ribosomal protein S15p (S13e)	0.063365	0.013572	0.028103	0.01992	0.02101	0.024352	0.047658	0.034387	0.019267	0.011014	0.010945	0.028553659
Clustering-based subsystems	NULL	KH domain RNA binding protein YlqC	SSU ribosomal protein S16p	0.031334	0.036192	0.020438	0.01494	0.015757	0.014124	0.021663	0.019934	0.017516	0.077098	0.031786	0.023584375
Protein Metabolism	Protein biosynthesis	Ribosome SSU bacterial	SSU ribosomal protein S17p (S11e)	0.036905	0.018096	0.022993	0.032371	0.023636	0.019482	0.035382	0.029902	0.02496	0.057273	0.026198	0.02599014
Protein Metabolism	Protein biosynthesis	Ribosome SSU bacterial	SSU ribosomal protein S19p (S15e)	0.26843	0.054288	0.020438	0.067231	0.024949	0.037989	0.046935	0.033889	0.038973	0.0793	0.020027	0.029500189
Protein Metabolism	Protein biosynthesis	Ribosome SSU bacterial	SSU ribosomal protein S1p	0.200539	0.176435	0.13285	0.124502	0.186462	0.175334	0.162469	0.169443	0.144943	0.248915	0.490536	0.164301853
Miscellaneous	Plant-Prokaryote DOE project	At4g10620 At3g57180 At3g47450	SSU ribosomal protein S20p	0.0094	0.015834	0.00511	0.00498	0.011161	0.005844	0.012998	0.013456	0.018829	0.013217	0.020958	0.015933257
Cell Division and Cell Cycle	NULL	Macromolecular synthesis operon	SSU ribosomal protein S21p	0.013926	0.015834	0.022993	0.01743	0.017727	0.012663	0.014442	0.020931	0.017078	0.008811	0.008383	0.019601061
Protein Metabolism	Protein biosynthesis	Ribosome SSU bacterial	SSU ribosomal protein S2p (SAe)	0.114544	0.065598	0.06898	0.052291	0.074191	0.095947	0.062099	0.075252	0.078164	0.158601	0.072655	0.074499808
Protein Metabolism	Protein biosynthesis	Ribosome SSU bacterial	SSU ribosomal protein S3p (S3e)	0.146226	0.070121	0.056206	0.072211	0.070908	0.070134	0.070042	0.080236	0.071158	0.253321	0.06823	0.065626088
Clustering-based subsystems	Cell Division	CBSS-393130.3.peg.794	SSU ribosomal protein S4p (S9e)	0.186961	0.058812	0.053651	0.062251	0.073534	0.052113	0.057767	0.074754	0.05189	0.068286	0.068347	0.057186195
Protein Metabolism	Protein processing and modification	Ribosomal protein S5p acylation	SSU ribosomal protein S5p (S2e)	0.131256	0.067859	0.084308	0.039841	0.046615	0.045295	0.032494	0.072262	0.044227	0.037447	0.04506	0.050402729
Protein Metabolism	Protein biosynthesis	Ribosome SSU bacterial	SSU ribosomal protein S6p	0.135085	0.013572	0.007664	0.02241	0.013131	0.015585	0.01372	0.021928	0.022989	0.008811	0.022705	0.025635191
Protein Metabolism	Protein biosynthesis	Ribosome SSU bacterial	SSU ribosomal protein S7p (S5e)	0.153538	0.042978	0.030658	0.067231	0.043333	0.043347	0.067876	0.055318	0.05408	0.066084	0.037026	0.044881303
Protein Metabolism	Protein biosynthesis	Ribosome SSU bacterial	SSU ribosomal protein S8p (S15Ae)	0.054313	0.029406	0.030658	0.02988	0.036111	0.028248	0.038993	0.039869	0.035031	0.017622	0.028759	0.035376563
Protein Metabolism	Protein biosynthesis	Ribosome SSU bacterial	SSU ribosomal protein S9p (S16e)	0.037601	0.027144	0.05876	0.01245	0.037424	0.041885	0.028883	0.054321	0.032842	0.033042	0.027129	0.032182024
Protein Metabolism	Protein biosynthesis	rRNAs	SSU rRNA	0.002437	0.006786	0.010219	0.00747	0.005252	0.006332	0.007943	0.006479	0.009196	0.006608	0.030739	0.008913159
Cell Division and Cell Cycle	NULL	Bacterial Cytoskeleton	Stage 0 sporulation protein J	0.004178	0.004524	0.002555	0.00498	0.010505	0.00828	0.012998	0.00897	0.007882	0.017622	0.026081	0.008992036
Stress Response	NULL	Carbon Starvation	Starvation sensing protein RspA	0.0094	0.006786	0.007664	0.01245	0.008535	0.005844	0.012998	0.006479	0.010947	0.006608	0.001863	0.005087599
Stress Response	NULL	Carbon Starvation	Stringent starvation protein A	0.019497	0.004524	0.007664	0.00498	0.011161	0.010715	0.019496	0.016446	0.017735	0.006608	0.033067	0.012935912
Membrane Transport	NULL	Transport of Nickel and Cobalt	Substrate-specific component NikM of nickel ECF transporter	0.006615	0.002262	0.007664	0.00498	0.018384	0.010715	0.011553	0.004485	0.006568	0.006608	0.001397	0.00855821
Respiration	Electron donating reactions	Succinate dehydrogenase	Succinate dehydrogenase cytochrome b subunit	0.011141	0.006786	0.017884	0.00747	0.008535	0.011202	0.004333	0.010466	0.007882	0.030839	0.001048	0.007808873
Respiration	Electron donating reactions	Succinate dehydrogenase	Succinate dehydrogenase flavoprotein subunit (EC 1.3.99.1)	0.197057	0.21715	0.229932	0.256474	0.191714	0.231831	0.184854	0.204826	0.183915	0.222482	0.199218	0.207842239
Respiration	Electron donating reactions	Succinate dehydrogenase	Succinate dehydrogenase hydrophobic membrane anchor protein	0.001741	0.002262	0.002555	0.00249	0.002626	0.005357	0.009387	0.008472	0.006131	0.024231	0.000466	0.003312855
Cofactors, Vitamins, Prosthetic Groups, Pigments	Folate and pterines	5-FCL-like protein	Succinate dehydrogenase iron-sulfur protein (EC 1.3.99.1)	0.192531	0.095003	0.114966	0.064741	0.083383	0.097895	0.085928	0.081233	0.073566	0.066084	0.07149	0.084004548
Carbohydrates	Central carbohydrate metabolism	TCA Cycle	Succinyl-CoA ligase [ADP-forming] alpha chain (EC 6.2.1.5)	0.090869	0.085955	0.104747	0.117032	0.091918	0.109584	0.085928	0.102164	0.093709	0.220279	0.08814	0.094574134
Carbohydrates	One-carbon Metabolism	Serine-glyoxylate cycle	Succinyl-CoA ligase [ADP-forming] beta chain (EC 6.2.1.5)	0.182783	0.110837	0.137959	0.136952	0.129998	0.147086	0.145139	0.112131	0.117793	0.17402	0.135878	0.121786875
Metabolism of Aromatic Compounds	Metabolism of central aromatic intermediates	Catechol branch of beta-ketoadipate pathway	Succinyl-CoA:3-ketoacid-coenzyme A transferase subunit A (EC 2.8.3.5)	0.010793	0.009048	0.017884	0.01245	0.009192	0.009254	0.011553	0.020931	0.014888	0.013217	0.0751	0.012383769
Carbohydrates	Di- and oligosaccharides	Fructooligosaccharides(FOS) and Raffinose Utilization	Sucrose phosphorylase (EC 2.4.1.7)	0.023327	0.042978	0.045986	0.02739	0.058433	0.053574	0.033216	0.032892	0.013794	0.006608	0.013972	0.041923397
Carbohydrates	Di- and oligosaccharides	Fructooligosaccharides(FOS) and Raffinose Utilization	Sucrose-6-phosphate hydrolase (EC 3.2.1.26)	0.015319	0.004524	0.010219	0.00747	0.006566	0.006819	0.004333	0.006977	0.006568	0.013217	0.000233	0.004811528
Miscellaneous	Plant-Prokaryote DOE project	COG3533	sugar ABC transporter, permease protein	0.005222	0.020358	0.033212	0.02739	0.018384	0.024352	0.015886	0.013456	0.027806	0.044056	0.002212	0.014474023
Carbohydrates	Fermentation	Fermentations: Mixed acid	Sugar/maltose fermentation stimulation protein homolog	0.005571	0.004524	0.010219	0.01494	0.030202	0.023378	0.035382	0.019934	0.021676	0.002203	0.024218	0.020192643
Clustering-based subsystems	Sulfatases and sulfatase modifying factor 1 (and a hypothetical)	Sulfatases and sulfatase modifying factor 1	Sulfatase	0.057794	0.065598	0.109856	0.074701	0.045302	0.045782	0.046935	0.033889	0.057145	0.008811	0.004424	0.055924155
Sulfur Metabolism	Inorganic sulfur assimilation	Inorganic Sulfur Assimilation	Sulfate adenylyltransferase subunit 1 (EC 2.7.7.4)	0.013926	0.024882	0.040877	0.02241	0.032828	0.026787	0.039715	0.056315	0.058459	0.118951	0.101065	0.062470988
Sulfur Metabolism	Inorganic sulfur assimilation	Inorganic Sulfur Assimilation	Sulfate adenylyltransferase subunit 2 (EC 2.7.7.4)	0.081469	0.031668	0.05876	0.02988	0.020353	0.024352	0.05199	0.058308	0.053204	0.063881	0.128543	0.051861963
Sulfur Metabolism	Inorganic sulfur assimilation	Inorganic Sulfur Assimilation	Sulfate adenylyltransferase, dissimilatory-type (EC 2.7.7.4)	0.068587	0.076907	0.081754	0.087151	0.078787	0.076465	0.059933	0.032393	0.027149	0.019825	0.001863	0.048233597
Stress Response	Detoxification	Uptake of selenate and selenite	Sulfate and thiosulfate import ATP-binding protein CysA (EC 3.6.3.25)	0.019497	0.015834	0.002555	0.01494	0.004596	0.005844	0.008665	0.010466	0.013356	0.002203	0.000582	0.007690557
Amino Acids and Derivatives	Lysine, threonine, methionine, and cysteine	Cysteine Biosynthesis	Sulfate permease	0.057446	0.085955	0.120076	0.072211	0.072221	0.092538	0.088816	0.107147	0.108817	0.072692	0.143214	0.171558584
Sulfur Metabolism	NULL	Sulfur oxidation	Sulfide dehydrogenase [flavocytochrome C] flavoprotein chain precursor (EC 1.8.2.-)	0.009052	0.004524	0.010219	0.00249	0.015101	0.015585	0.007221	0.010964	0.012261	0.013217	0.000233	0.027843761

Sulfur Metabolism	NULL	Sulfur oxidation	Sulfite oxidase	0.008704	0.015834	0.028103	0.01494	0.014444	0.009741	0.016608	0.012459	0.031747	0.006608	0.021075	0.019285551
Amino Acids and Derivatives	Lysine, threonine, methionine, and cysteine	Cysteine Biosynthesis	Sulfite reductase [NADPH] hemoprotein beta-component (EC 1.8.1.2)	0.016712	0.018096	0.010219	0.00498	0.029545	0.024352	0.024551	0.045849	0.050796	0.05507	0.088839	0.018733409
Respiration	Electron accepting reactions	Anaerobic respiratory reductases	Sulfite reductase beta subunit (EC 1.8.99.1)	0.036905	0.02262	0.022993	0.047311	0.028232	0.029222	0.020218	0.012957	0.007663	0.013217	0.001397	0.020389836
Sulfur Metabolism	NULL	Sulfate reduction-associated complexes	Sulfite reduction-associated complex DsrMKJOP iron-sulfur protein DsrO (=HmeA)	0.021586	0.03393	0.030658	0.01992	0.024949	0.021917	0.015164	0.007475	0.003941	0.006608	0.000116	0.015026166
Miscellaneous	Plant-Prokaryote DOE project	COG2363	Sulfur acceptor protein SufE for iron-sulfur cluster assembly	0.002437	0.015834	0.012774	0.00249	0.005252	0.00828	0.007221	0.022426	0.020362	0.011014	0.000582	0.012699279
Cofactors, Vitamins, Prosthetic Groups, Pigments	NULL	Thiamin biosynthesis	Sulfur carrier protein adenylyltransferase ThiF	0.041779	0.036192	0.030658	0.01494	0.036767	0.03458	0.029605	0.028905	0.036564	0.033042	0.013041	0.032142585
Sulfur Metabolism	NULL	Sulfur oxidation	Sulfur oxidation molybdopterin C protein	0.004178	0.002262	0.00511	0.00996	0.004596	0.012176	0.01372	0.012459	0.01445	0.004406	0.001281	0.012699279
Sulfur Metabolism	NULL	Sulfur oxidation	sulfur oxidation protein SoxA	0.0094	0.020358	0.020438	0.00747	0.003939	0.01802	0.008665	0.011462	0.011604	0.004406	0.001281	0.012620402
Sulfur Metabolism	NULL	Sulfur oxidation	Sulfur oxidation protein SoxB	0.030986	0.009048	0.00511	0.02988	0.021666	0.018995	0.01733	0.025416	0.01861	0.022028	0.002794	0.035139931
Sulfur Metabolism	NULL	Sulfur oxidation	Sulfur oxidation protein SoxY	0.009748	0.01131	0.002555	0.00996	0.003283	0.014124	0.008665	0.00897	0.002627	0.004406	0.000466	0.005679181
Clustering-based subsystems	NULL	CBSS-316273.3.peg.2709	Superfamily II DNA/RNA helicases, SNF2 family	0.036557	0.049764	0.035767	0.072211	0.127372	0.079387	0.066432	0.079239	0.049044	0.028636	0.00163	0.040030336
Stress Response	Oxidative stress	Oxidative stress	Superoxide dismutase [Fe] (EC 1.15.1.1)	0.071024	0.042978	0.020438	0.01743	0.022979	0.033606	0.041881	0.037377	0.023208	0.024231	0.028876	0.028908607
Stress Response	Oxidative stress	Oxidative stress	Superoxide reductase (EC 1.15.1.2)	0.013926	0.020358	0.028103	0.02241	0.035454	0.027274	0.023829	0.011462	0.001533	0.006608	0.000815	0.013685248
Protein Metabolism	Protein folding	Peptidyl-prolyl cis-trans isomerase	Survival protein SurA precursor (Peptidyl-prolyl cis-trans isomerase SurA) (EC 5.2.1.8)	0.011489	0.015834	0.033212	0.00996	0.011818	0.010715	0.012275	0.023423	0.028025	0.03965	0.020725	0.029657944
Clustering-based subsystems	Choline bitartrate degradation, putative	CBSS-344610.3.peg.2335	Tartrate decarboxylase (EC 4.1.1.73)	0.001393	0.004524	0.007664	0.01245	0.005909	0.005844	0.01372	0.004984	0.009853	0.008811	0.000931	0.002563519
Clustering-based subsystems	Choline bitartrate degradation, putative	CBSS-344610.3.peg.2335	Tartrate dehydrogenase (EC 1.1.1.93)	0.002437	0.004524	0.002555	0.00498	0.001313	0.000487	0.004333	0.00299	0.001971	0.006608	0.000116	0.00063102
Sulfur Metabolism	Organic sulfur assimilation	Taurine Utilization	Taurine transport ATP-binding protein TauB	0.004526	0.006786	0.007664	0.00498	0.003283	0.005844	0.004333	0.00299	0.005912	0.008811	0.000233	0.003233978
Clustering-based subsystems	NULL	CBSS-176280.1.peg.1561	Teichoic acid export ATP-binding protein TagH (EC 3.6.3.40)	0.003482	0.006786	0.00511	0.00249	0.005252	0.002435	0.002888	0.00299	0.003941	0.004406	0.000699	0.005600303
Miscellaneous	Plant-Prokaryote DOE project	COG0451	Tetraacyldisaccharide 4'-kinase (EC 2.7.1.130)	0.022282	0.020358	0.033212	0.01743	0.028888	0.028248	0.025273	0.029403	0.020143	0.006608	0.004308	0.029736821
Protein Metabolism	Protein folding	Thermosome, archaeal	Thermosome subunit	0.00383	0.020358	0.002555	0.00996	0.026262	0.010715	0.033938	0.012957	0.005693	0.03965	0.002678	0.012541524
Protein Metabolism	Protein degradation	Metallocarboxypeptidases (EC 3.4.17.-)	Thermostable carboxypeptidase 1 (EC 3.4.17.19)	0.013578	0.020358	0.010219	0.0249	0.022323	0.024352	0.022385	0.029403	0.026055	0.004406	0.002096	0.014750094
Cofactors, Vitamins, Prosthetic Groups, Pigments	NULL	Thiamin biosynthesis	Thiamin ABC transporter, ATPase component	0.004874	0.01131	0.007664	0.00249	0.003283	0.002922	0.002888	0.003489	0.003722	0.006608	0.003027	0.004890406
Miscellaneous	Plant-Prokaryote DOE project	Iron-sulfur cluster assembly	Thiamin biosynthesis lipoprotein ApbE	0.010097	0.052026	0.033212	0.01245	0.037424	0.027761	0.032494	0.0304	0.03744	0.011014	0.023636	0.04720819
Miscellaneous	Plant-Prokaryote DOE project	COG2363	Thiamin biosynthesis protein ThiC	0.091566	0.079169	0.081754	0.087151	0.087978	0.0789	0.074375	0.082728	0.080135	0.057273	0.167898	0.095007961
Clustering-based subsystems	Cell Division	CBSS-393130.3.peg.794	Thiamine biosynthesis protein ThiI	0.002785	0.006786	0.002555	0.01494	0.009192	0.006819	0.01372	0.014452	0.02496	0.030839	0.080106	0.014631778
Cofactors, Vitamins, Prosthetic Groups, Pigments	NULL	Thiamin biosynthesis	Thiamine-monophosphate kinase (EC 2.7.4.16)	0.015667	0.027144	0.033212	0.02241	0.025606	0.022891	0.01372	0.020931	0.032842	0.132167	0.016766	0.038452786
Miscellaneous	Plant-Prokaryote DOE project	At1g54520	Thiamin-phosphate pyrophosphorylase (EC 2.5.1.3)	0.018452	0.020358	0.025548	0.02739	0.026919	0.036041	0.016608	0.021928	0.011604	0.011014	0.026896	0.0196405
Miscellaneous	Plant-Prokaryote DOE project	COG2363	Thiazole biosynthesis protein ThiG	0.050831	0.036192	0.030658	0.034861	0.04202	0.042372	0.046213	0.040367	0.04357	0.019825	0.036677	0.038058399
Cofactors, Vitamins, Prosthetic Groups, Pigments	NULL	Thiamin biosynthesis	Thiazole biosynthesis protein ThiH	0.005919	0.015834	0.012774	0.00996	0.012475	0.015585	0.019496	0.013954	0.003941	0.002203	0.00652	0.019364429
Clustering-based subsystems	NULL	CBSS-176280.1.peg.1561	ThiJ/PfpI family protein	0.029245	0.027144	0.015329	0.034861	0.023636	0.023378	0.019496	0.025416	0.046198	0.033042	0.087325	0.02819871
Miscellaneous	Plant-Prokaryote DOE project	At5g04520 AT1G06240	Thiol peroxidase, Bcp-type (EC 1.11.1.15)	0.037949	0.024882	0.030658	0.034861	0.020353	0.035067	0.046935	0.034387	0.036783	0.070489	0.036793	0.039478194
Sulfur Metabolism	NULL	Thioredoxin-disulfide reductase	Thiol peroxidase, Tpx-type (EC 1.11.1.15)	0.015667	0.027144	0.015329	0.02241	0.023636	0.0263	0.011553	0.013954	0.009196	0.024231	0.009548	0.029973454
Respiration	NULL	Biogenesis of c-type cytochromes	Thiol:disulfide oxidoreductase TlpA	0.003133	0.002262	0.002555	0.00498	0.00197	0.002922	0.00361	0.00598	0.004817	0.004406	0.000349	0.001419795
Miscellaneous	Plant-Prokaryote DOE project	DOE COG2016	Thioredoxin	0.140656	0.176435	0.150733	0.141932	0.109645	0.125169	0.161747	0.166452	0.155671	0.074895	0.070442	0.170533176
RNA Metabolism	RNA processing and modification	Wyeosine-MimG Biosynthesis	Thioredoxin reductase (EC 1.8.1.9)	0.095743	0.133457	0.135404	0.117032	0.093231	0.092538	0.099648	0.098675	0.092396	0.121153	0.061943	0.097137653
Clustering-based subsystems	NULL	CBSS-316273.3.peg.227	Thiosulfate sulfurtransferase, rhodanese (EC 2.8.1.1)	0.018104	0.018096	0.033212	0.037351	0.028888	0.026787	0.035382	0.040866	0.033718	0.008811	0.007335	0.021178611
Amino Acids and Derivatives	Lysine, threonine, methionine, and cysteine	Threonine degradation	Threonine dehydratase (EC 4.3.1.19)	0.020889	0.03393	0.025548	0.02988	0.02101	0.01802	0.032494	0.020931	0.025398	0.01542	0.013623	0.025911262
Amino Acids and Derivatives	Branched-chain amino acids	Branched-Chain Amino Acid Biosynthesis	Threonine dehydratase biosynthetic (EC 4.3.1.19)	0.025416	0.020358	0.05876	0.01992	0.024293	0.026787	0.025995	0.024918	0.03328	0.011014	0.102578	0.043737579
Amino Acids and Derivatives	Lysine, threonine, methionine, and cysteine	Threonine degradation	Threonine dehydratase, catabolic (EC 4.3.1.19)	0.035512	0.02262	0.015329	0.054781	0.051868	0.05601	0.036826	0.051829	0.057583	0.006608	0.047272	0.03072279
Amino Acids and Derivatives	Lysine, threonine, methionine, and cysteine	Threonine degradation	Threonine dehydrogenase and related Zn-dependent dehydrogenases	0.03029	0.038454	0.028103	0.00498	0.013788	0.014611	0.018774	0.015948	0.037878	0.002203	0.055422	0.012935912
Amino Acids and Derivatives	Lysine, threonine, methionine, and cysteine	Threonine and Homoserine Biosynthesis	Threonine synthase (EC 4.2.3.1)	0.101662	0.110837	0.109856	0.129482	0.139846	0.138806	0.134308	0.093193	0.103124	0.074895	0.144611	0.114096318
Protein Metabolism	Protein biosynthesis	tRNA aminoacylation, Thr	Threonyl-tRNA synthetase (EC 6.1.1.3)	0.136826	0.235246	0.222268	0.191733	0.206815	0.180692	0.192074	0.194361	0.181288	0.262132	0.192698	0.197548724
Phages, Prophages, Transposable elements, Plasmids	Phages, Prophages	Phage DNA synthesis	Thymidine kinase	0.001393	0.015834	0.002555	0.02241	0.006566	0.008767	0.011553	0.012957	0.013575	0.006608	0.001979	0.012107698
Nucleosides and Nucleotides	Pyrimidines	pyrimidine conversions	Thymidine kinase (EC 2.7.1.21)	0.006267	0.004524	0.010219	0.01743	0.009192	0.006332	0.007943	0.00897	0.005036	0.011014	0.002096	0.009425862
Nucleosides and Nucleotides	Pyrimidines	pyrimidine conversions	Thymidine phosphorylase (EC 2.4.2.4)	0.018104	0.013572	0.022993	0.032371	0.009192	0.024352	0.028883	0.033889	0.052328	0.035245	0.011178	0.021770193
Clustering-based subsystems	NULL	CBSS-393133.3.peg.2787	Thymidylate kinase (EC 2.7.4.9)	0.160153	0.079169	0.13285	0.119522	0.097827	0.102278	0.087372	0.101167	0.112539	0.052867	0.05519	0.114845654
Cofactors, Vitamins, Prosthetic Groups, Pigments	Folate and pterines	Folate Biosynthesis	Thymidylate synthase (EC 2.1.1.45)	0.216554	0.042978	0.030658	0.039841	0.081413	0.083284	0.098926	0.104656	0.052985	0.24451	0.061943	0.063496395
Cofactors, Vitamins, Prosthetic Groups, Pigments	Folate and pterines	Folate Biosynthesis	Thymidylate synthase thyX (EC 2.1.1.-)	0.013926	0.058812	0.035767	0.0249	0.066312	0.110558	0.149471	0.105652	0.015545	0.004406	0.000466	0.018930602
Protein Metabolism	Protein degradation	Putative TldE-TldD proteolytic complex	TldD family protein, Beta/Gamma-proteobacterial subgroup	0.012186	0.006786	0.022993	0.00996	0.001313	0.010228	0.021663	0.002492	0.005036	0.004406	0.000582	0.006310201
Clustering-based subsystems	NULL	CBSS-316057.3.peg.563	TldD protein, part of proposed TldE/TldD proteolytic complex (PMID 12029038)	0.122203	0.040716	0.017884	0.059761	0.029545	0.033606	0.067154	0.044354	0.050796	0.026433	0.008849	0.036401971
Cell Wall and Capsule	Gram-Negative cell wall components	Lipopolysaccharide-related cluster in Alphaproteobacteria	TldE/PmbA protein, part of proposed TldE/TldD proteolytic complex (PMID 12029038)	0.025416	0.009048	0.012774	0.01494	0.01904	0.023865	0.026717	0.029902	0.044227	0.035245	0.006404	0.017905195
Stress Response	Heat shock	Heat shock dnaK gene cluster extended	tmRNA-binding protein SmpB	0.045957	0.070121	0.053651	0.062251	0.03808	0.034093	0.043325	0.043856	0.038535	0.030839	0.030971	0.039399316
Membrane Transport	NULL	Ton and Tol transport systems	tolB protein precursor, periplasmic protein involved in the tonb-independent uptake of group A colicins	0.085299	0.081431	0.035767	0.062251	0.069595	0.063315	0.059211	0.084721	0.092177	0.01542	0.019561	0.05986803
Membrane Transport	NULL	Ton and Tol transport systems	TonB-dependent receptor	0.169205	0.158339	0.143069	0.166833	0.179896	0.244981	0.249119	0.436065	0.383376	0.129965	0.265003	0.300641629
Cofactors, Vitamins, Prosthetic Groups, Pigments	Folate and pterines	YgfZ-Iron	TonB-dependent receptor; Outer membrane receptor for ferrienterochelin and colicins	0.001393	0.01131	0.002555	0.00498	0.015101	0.012663	0.012275	0.018938	0.023208	0.004406	0.024917	0.013172544
Virulence, Disease and Defense	Resistance to antibiotics and toxic compounds	Resistance to fluoroquinolones	Topoisomerase IV subunit A (EC 5.99.1.-)	0.145182	0.067859	0.051096	0.104582	0.108332	0.102765	0.109757	0.139541	0.126113	0.348041	0.203876	0.131094422

DNA Metabolism	DNA replication	DNA topoisomerases, Type II, ATP-dependent	Topoisomerase IV subunit B (EC 5.99.1.-)	0.125337	0.090479	0.076644	0.077191	0.082726	0.084745	0.109757	0.113626	0.101153	0.259929	0.14333	0.112361013
Clustering-based subsystems	NULL	Aerotolerance operon in Bacteroides and potentially orthologous operons in other organisms	TPR domain protein in aerotolerance operon	0.005222	0.006786	0.012774	0.00996	0.005909	0.006332	0.007943	0.013456	0.020362	0.002203	0.000699	0.013685248
Carbohydrates	Central carbohydrate metabolism	Pentose phosphate pathway	Transaldolase (EC 2.2.1.2)	0.065106	0.101789	0.099637	0.092131	0.071565	0.05601	0.06571	0.06379	0.072909	0.145384	0.067066	0.082545314
Cell Division and Cell Cycle	NULL	Macromolecular synthesis operon	Transamidase GatB domain protein	0.010445	0.002262	0.010219	0.02241	0.017727	0.014611	0.014442	0.02442	0.010947	0.01542	0.036677	0.016524838
Clustering-based subsystems	NULL	CBSS-393131.3.peg.612	Transcription accessory protein (S1 RNA-binding domain)	0.037253	0.070121	0.05876	0.042331	0.050555	0.037989	0.046935	0.053823	0.056488	0.03965	0.042382	0.101081529
RNA Metabolism	Transcription	Transcription factors bacterial	Transcription antitermination protein NusG	0.105492	0.052026	0.051096	0.059761	0.038737	0.053087	0.061377	0.070269	0.046417	0.028636	0.037375	0.059039816
RNA Metabolism	Transcription	Transcription factors bacterial	Transcription elongation factor GreA	0.019497	0.02262	0.038322	0.037351	0.041363	0.032145	0.050546	0.051331	0.038316	0.072692	0.037608	0.050166096
RNA Metabolism	Transcription	Transcription factors bacterial	Transcription elongation factor GreB	0.012186	0.006786	0.017884	0.00747	0.013131	0.011202	0.015886	0.013456	0.015545	0.011014	0.031321	0.010885096
RNA Metabolism	Transcription	RNA polymerase archaeal initiation factors	Transcription initiation factor B	0.000348	0.009048	0.002555	0.00996	0.01904	0.007793	0.029605	0.016446	0.010947	0.017622	0.000233	0.006704588
RNA Metabolism	Transcription	Transcription factors bacterial	Transcription termination factor Rho	0.153189	0.147029	0.12774	0.124502	0.142473	0.120786	0.137196	0.154492	0.139469	0.187237	0.222971	0.13176488
Clustering-based subsystems	NULL	CBSS-350688.3.peg.1509	Transcription termination protein NusA	0.102358	0.156077	0.125185	0.112052	0.134594	0.123708	0.141529	0.123593	0.102905	0.13437	0.187109	0.115397797
RNA Metabolism	Transcription	Transcription factors bacterial	Transcription termination protein NusB	0.050135	0.01131	0.00511	0.02988	0.010505	0.027761	0.024551	0.024918	0.026712	0.061678	0.016534	0.028040955
Carbohydrates	Fermentation	Acetoin, butanediol metabolism	Transcriptional activator of acetoin dehydrogenase operon AcoR	0.004874	0.002262	0.00511	0.00498	0.003939	0.002435	0.000722	0.000997	0.003722	0.004406	0.030156	0.006152446
Amino Acids and Derivatives	Branched-chain amino acids	Branched chain amino acid degradation regulons	Transcriptional regulator BkdR of isoleucine and valine catabolism operon	0.014623	0.006786	0.015329	0.00747	0.005909	0.004383	0.002166	0.00299	0.004598	0.01542	0.000349	0.01013576
Stress Response	Oxidative stress	Oxidative stress	transcriptional regulator, Crp/Fnr family	0.011489	0.015834	0.017884	0.01992	0.01707	0.017046	0.012275	0.023921	0.017954	0.022028	0.002794	0.036007583
Metabolism of Aromatic Compounds	Metabolism of central aromatic intermediates	Homogentisate pathway of aromatic compound degradation	Transcriptional regulator, IclR family	0.01706	0.020358	0.010219	0.01992	0.010505	0.010228	0.007221	0.014951	0.017078	0.013217	0.000815	0.010372393
Virulence, Disease and Defense	Resistance to antibiotics and toxic compounds	Cobalt-zinc-cadmium resistance	Transcriptional regulator, MerR family	0.025764	0.038454	0.022993	0.02988	0.034797	0.04286	0.024551	0.033889	0.036126	0.028636	0.056703	0.035179369
Dormancy and Sporulation	NULL	Sporulation Cluster	Transcription-repair coupling factor	0.18522	0.160601	0.135404	0.191733	0.143129	0.167055	0.202184	0.193862	0.187637	0.158601	0.174185	0.209656421
Carbohydrates	Central carbohydrate metabolism	Pentose phosphate pathway	Transketolase (EC 2.2.1.1)	0.211332	0.194531	0.201829	0.161853	0.156917	0.14806	0.133586	0.148013	0.15589	0.101328	0.156836	0.140125896
Carbohydrates	CO2 fixation	Calvin-Benson cycle	Transketolase, C-terminal section (EC 2.2.1.1)	0.003482	0.049764	0.028103	0.00996	0.033484	0.025813	0.029605	0.02741	0.01445	0.033042	0.007452	0.035849828
Carbohydrates	Central carbohydrate metabolism	Pentose phosphate pathway	Transketolase, N-terminal section (EC 2.2.1.1)	0.023675	0.081431	0.038322	0.057271	0.055807	0.054061	0.049824	0.035384	0.028463	0.125559	0.016883	0.045630639
Protein Metabolism	Protein biosynthesis	Translation elongation factors eukaryotic and archaeal	Translation elongation factor 1 alpha subunit	0.006963	0.01131	0.002555	0.02241	0.010505	0.009254	0.018052	0.00299	0.001752	0.002203	0.001048	0.004456579
Protein Metabolism	Protein biosynthesis	Universal GTPases	Translation elongation factor 2	0.004526	0.01131	0.010219	0.02739	0.016414	0.015098	0.03105	0.013456	0.004598	0.011014	0.000582	0.010017444
Protein Metabolism	Protein biosynthesis	Translation elongation factor G family	Translation elongation factor G	0.663937	0.398109	0.31935	0.440737	0.425448	0.419828	0.358154	0.313967	0.265801	0.515453	0.424518	0.357039049
Protein Metabolism	Protein biosynthesis	Translation elongation factors bacterial	Translation elongation factor G paralog	0.010445	0.002262	0.00511	0.00498	0.000657	0.006332	0.007221	0.003987	0.001752	0.019825	0.065552	0.009662495
Protein Metabolism	Protein biosynthesis	Translation elongation factor G family	Translation elongation factor G-related protein	0.041431	0.049764	0.040877	0.047311	0.03808	0.031171	0.029605	0.028407	0.026274	0.004406	0.00326	0.033483503
Protein Metabolism	Protein biosynthesis	Universal GTPases	Translation elongation factor LepA	0.263556	0.221674	0.199274	0.184263	0.167422	0.180205	0.192796	0.192367	0.175157	0.299579	0.210862	0.193052706
Protein Metabolism	Protein biosynthesis	Translation elongation factors eukaryotic and archaeal	Translation elongation factor P	0.048394	0.036192	0.040877	0.034861	0.052524	0.036041	0.059933	0.048839	0.039848	0.050664	0.057402	0.059986346
Protein Metabolism	Protein biosynthesis	Translation elongation factors bacterial	Translation elongation factor Ts	0.119418	0.061074	0.035767	0.047311	0.047929	0.059906	0.082318	0.084721	0.065027	0.074895	0.102345	0.074105421
Protein Metabolism	Protein biosynthesis	Universal GTPases	Translation elongation factor Tu	0.243014	0.23977	0.242706	0.249004	0.315803	0.232805	0.249841	0.231738	0.206686	0.471397	0.281071	0.200388314
Protein Metabolism	Protein biosynthesis	Translation initiation factors bacterial	Translation initiation factor 1	0.158064	0.024882	0.028103	0.049801	0.028888	0.0263	0.028161	0.032393	0.021676	0.017622	0.002329	0.025516874
Clustering-based subsystems	NULL	CBSS-138119.3.peg.2719	Translation initiation factor 2	0.153886	0.201316	0.229932	0.186753	0.214037	0.201635	0.214459	0.198347	0.165086	0.17402	0.175582	0.192106175
Protein Metabolism	Protein biosynthesis	Translation initiation factors bacterial	Translation initiation factor 3	0.066498	0.058812	0.048541	0.042331	0.053838	0.038963	0.051268	0.052826	0.039848	0.077098	0.053443	0.051230943
Protein Metabolism	Protein biosynthesis	Translation initiation factors eukaryotic and archaeal	Translation initiation factor SUI1-related protein	0.001393	0.004524	0.002555	0.00498	0.009848	0.003896	0.009387	0.008472	0.008101	0.050664	0.00524	0.009741372
Respiration	Electron accepting reactions	Terminal cytochrome d ubiquinol oxidases	Transport ATP-binding protein CydD	0.000348	0.004524	0.007664	0.00747	0.004596	0.009254	0.00361	0.00598	0.010509	0.013217	0.006637	0.008952597
Clustering-based subsystems	Tricarboxylate transporter	CBSS-49338.1.peg.459	TRAP-type C4-dicarboxylate transport system, large permease component	0.109322	0.158339	0.217158	0.194223	0.226512	0.275665	0.196407	0.162964	0.271275	0.048461	0.32357	0.184139547
Clustering-based subsystems	Tricarboxylate transporter	CBSS-49338.1.peg.459	TRAP-type C4-dicarboxylate transport system, periplasmic component	0.035512	0.070121	0.066425	0.097112	0.119493	0.132475	0.08665	0.11562	0.114728	0.052867	0.220409	0.089920361
Cell Wall and Capsule	Capsular and extracellular polysacchrides	Sialic Acid Metabolism	TRAP-type transport system, large permease component, predicted N-acetylneuraminate transporter	0.002785	0.015834	0.025548	0.01992	0.020353	0.025813	0.012998	0.007974	0.008101	0.006608	0.000349	0.012817595
Cell Wall and Capsule	Capsular and extracellular polysacchrides	Sialic Acid Metabolism	TRAP-type transport system, periplasmic component, predicted N-acetylneuraminate-binding protein	0.005571	0.006786	0.002555	0.00747	0.005252	0.00487	0.000722	0.002492	0.005255	0.004406	0.000815	0.005994691
Clustering-based subsystems	Tricarboxylate transporter	CBSS-49338.1.peg.459	TRAP-type uncharacterized transport system, fused permease component	0.063713	0.065598	0.094528	0.112052	0.117523	0.15634	0.096037	0.10017	0.095461	0.041853	0.077778	0.074065982
Carbohydrates	Di- and oligosaccharides	Trehalose Biosynthesis	Trehalose phosphorylase (EC 2.4.1.64)	0.001044	0.009048	0.017884	0.00996	0.005909	0.009741	0.005777	0.017443	0.013794	0.077098	0.005007	0.029894576
Carbohydrates	Di- and oligosaccharides	Trehalose Biosynthesis	Trehalose synthase (EC 5.4.99.16)	0.053964	0.092741	0.066425	0.057271	0.038737	0.027274	0.028161	0.014452	0.057583	0.022028	0.003842	0.034666666
Membrane Transport	NULL	Tricarboxylate transport system	Tricarboxylate transport membrane protein TctA	0.06998	0.061074	0.099637	0.094622	0.057777	0.091076	0.057045	0.057311	0.089987	0.044056	0.141118	0.056436858
Membrane Transport	NULL	Tricarboxylate transport system	Tricarboxylate transport protein TctC	0.02263	0.015834	0.022993	0.01743	0.023636	0.030196	0.015886	0.022925	0.024522	0.01542	0.129125	0.015775502
Carbohydrates	Central carbohydrate metabolism	Glycolysis and Gluconeogenesis	Triosephosphate isomerase (EC 5.3.1.1)	0.079032	0.054288	0.066425	0.119522	0.082726	0.056497	0.05199	0.064787	0.058678	0.026433	0.03819	0.066612057
Potassium metabolism	NULL	Potassium homeostasis	Trk system potassium uptake protein TrkA	0.118722	0.038454	0.05876	0.092131	0.09914	0.095947	0.082318	0.079239	0.077069	0.037447	0.117831	0.066690935
RNA Metabolism	RNA processing and modification	tRNA modification Bacteria	tRNA (5-methoxyuridine) 34 synthase	0.014274	0.01131	0.00511	0.00498	0.011818	0.010228	0.012998	0.015449	0.015545	0.01542	0.004541	0.019601061
RNA Metabolism	RNA processing and modification	mnm5U34 biosynthesis bacteria	tRNA (5-methylaminomethyl-2-thiouridylate)-methyltransferase (EC 2.1.1.61)	0.058491	0.040716	0.061315	0.039841	0.061716	0.059906	0.08665	0.074754	0.070282	0.081503	0.085462	0.076274552
RNA Metabolism	RNA processing and modification	tRNA modification Archaea	tRNA (adenine57/58-N1)-methyltransferase (EC 2.1.1.36)	0.006963	0.006786	0.015329	0.00498	0.012475	0.004383	0.010109	0.008472	0.009634	0.024231	0.005356	0.011634433
RNA Metabolism	RNA processing and modification	tRNA modification Bacteria	tRNA (cytosine34-2'-O-)-methyltransferase (EC 2.1.1.-)	0.021934	0.004524	0.010219	0.032371	0.022323	0.017533	0.020218	0.023423	0.015545	0.017622	0.01339	0.016958665
RNA Metabolism	RNA processing and modification	tRNA modification yeast mitochondrial	tRNA (Guanine37-N1) -methyltransferase (EC 2.1.1.31)	0.184524	0.065598	0.097082	0.074701	0.06106	0.065263	0.075819	0.070767	0.056707	0.107937	0.06334	0.06113007
RNA Metabolism	RNA processing and modification	RNA methylation	tRNA (guanine46-N7-)-methyltransferase (EC 2.1.1.33)	0.040038	0.009048	0.053651	0.034861	0.017727	0.016072	0.023107	0.03937	0.030434	0.011014	0.056587	0.040897989
RNA Metabolism	RNA processing and modification	tRNA modification Bacteria	tRNA (uridine-5-oxyacetic acid methyl ester) 34 synthase	0.012534	0.004524	0.020438	0.00498	0.009192	0.008767	0.009387	0.012459	0.016859	0.002203	0.008034	0.014316268
RNA Metabolism	RNA processing and modification	tRNA modification Bacteria	tRNA delta(2)-isopentenylpyrophosphate transferase (EC 2.5.1.8)	0.11524	0.038454	0.040877	0.047311	0.055151	0.041885	0.039715	0.067279	0.046855	0.03965	0.035978	0.05608191
RNA Metabolism	RNA processing and modification	tRNA modification Archaea	tRNA dihydrouridine synthase B (EC 1.-.-.-)	0.042127	0.038454	0.051096	0.042331	0.041363	0.058445	0.064988	0.058807	0.053204	0.070489	0.076613	0.068347362

Miscellaneous	Plant-Prokaryote DOE project	At2g33980 At1g28960	tRNA nucleotidyltransferase (EC 2.7.7.21) (EC 2.7.7.25)	0.042127	0.040716	0.043432	0.074701	0.051211	0.047243	0.077263	0.069272	0.058021	0.118951	0.042615	0.073868788
RNA Metabolism	RNA processing and modification	Polyadenylation bacterial	tRNA nucleotidyltransferase, A-adding (EC 2.7.7.25)	0.041083	0.047502	0.022993	0.062251	0.039393	0.043347	0.03466	0.012459	0.008539	0.002203	0.000582	0.022006825
RNA Metabolism	RNA processing and modification	tRNA modification yeast cytoplasmic	tRNA pseudouridine 13 synthase (EC 4.2.1.-)	0.005919	0.01131	0.012774	0.00249	0.011818	0.010715	0.007221	0.00897	0.014669	0.004406	0.021773	0.025201364
RNA Metabolism	RNA processing and modification	tRNA modification yeast mitochondrial	tRNA pseudouridine synthase A (EC 4.2.1.70)	0.028201	0.036192	0.025548	0.01743	0.053181	0.038963	0.037548	0.048341	0.047074	0.022028	0.030273	0.044053089
Clustering-based subsystems	NULL	CBSS-350688.3.peg.1509	tRNA pseudouridine synthase B (EC 4.2.1.70)	0.061972	0.054288	0.040877	0.069721	0.049898	0.050652	0.062099	0.052328	0.043133	0.037447	0.010595	0.051546453
RNA Metabolism	RNA processing and modification	RNA pseudouridine syntheses	tRNA pseudouridine synthase C (EC 4.2.1.70)	0.005571	0.01131	0.00511	0.00249	0.004596	0.007793	0.007943	0.00598	0.009415	0.002203	0.020842	0.011161168
Clustering-based subsystems	NULL	RNA modification and chromosome partitioning cluster	tRNA uridine 5-carboxymethylaminomethyl modification enzyme GidA	0.208198	0.151553	0.145624	0.119522	0.142473	0.128579	0.143695	0.150505	0.12896	0.301782	0.127262	0.15266742
RNA Metabolism	RNA processing and modification	tRNA modification Archaea	tRNA(Cytosine32)-2-thiocytidine synthetase	0.018104	0.03393	0.020438	0.034861	0.030858	0.027761	0.014442	0.028905	0.030215	0.068286	0.047971	0.028277587
Clustering-based subsystems	NULL	Cell division-ribosomal stress proteins cluster	tRNA(Ile)-lysidine synthetase	0.030638	0.009048	0.025548	0.02988	0.016414	0.018508	0.024551	0.02442	0.026493	0.002203	0.008733	0.037269623
RNA Metabolism	RNA processing and modification	tRNA modification Archaea	tRNA:Cm32/Um32 methyltransferase	0.018452	0.027144	0.010219	0.02241	0.025606	0.030196	0.033216	0.018439	0.024084	0.002203	0.033533	0.019443306
Clustering-based subsystems	NULL	Conserved gene cluster associated with Met-tRNA formyltransferase	tRNA:m(5)U-54 MTase gid	0.029942	0.02262	0.030658	0.064741	0.01904	0.020456	0.025995	0.036879	0.030653	0.017622	0.002212	0.01774744
RNA Metabolism	RNA processing and modification	tRNA modification Bacteria	tRNA-guanine transglycosylase (EC 2.4.2.29)	0.063713	0.124409	0.112411	0.141932	0.109645	0.101791	0.12131	0.119108	0.098964	0.114545	0.123071	0.111414483
RNA Metabolism	RNA processing and modification	tRNA modification Archaea	tRNA-i(6)A37 methylthiotransferase	0.117329	0.074645	0.112411	0.062251	0.09717	0.090589	0.089538	0.107147	0.100059	0.077098	0.123071	0.097532041
RNA Metabolism	RNA processing and modification	tRNAmodification position 34	tRNA-specific adenosine-34 deaminase (EC 3.5.4.-)	0.013926	0.029406	0.028103	0.02739	0.031515	0.032145	0.041881	0.036879	0.028025	0.028636	0.01665	0.030565035
Secondary Metabolism	Plant Hormones	Auxin biosynthesis	Tryptophan synthase alpha chain (EC 4.2.1.20)	0.016363	0.049764	0.043432	0.02988	0.042676	0.042372	0.03466	0.037377	0.042038	0.01542	0.071141	0.052453544
Secondary Metabolism	Plant Hormones	Auxin biosynthesis	Tryptophan synthase beta chain (EC 4.2.1.20)	0.075202	0.095003	0.120076	0.124502	0.092574	0.119812	0.096037	0.111134	0.104438	0.178426	0.093613	0.115121726
Amino Acids and Derivatives	Aromatic amino acids and derivatives	Chorismate: Intermediate for synthesis of Tryptophan, PAPA antibiotics, PABA, 3-hydroxyanthranilate and more.	Tryptophan synthase beta chain like (EC 4.2.1.20)	0.054313	0.147029	0.107302	0.102092	0.066312	0.064289	0.062821	0.035384	0.031966	0.037447	0.066134	0.081993172
Amino Acids and Derivatives	Aromatic amino acids and derivatives	Aromatic amino acid degradation	Tryptophanase (EC 4.1.99.1)	0.04909	0.070121	0.051096	0.064741	0.039393	0.043347	0.045491	0.033889	0.023208	0.006608	0.001281	0.05194084
Protein Metabolism	Protein biosynthesis	tRNA aminoacylation, Trp	Tryptophanyl-tRNA synthetase (EC 6.1.1.2)	0.07555	0.110837	0.12263	0.114542	0.104392	0.106662	0.1177	0.109639	0.091082	0.178426	0.098387	0.112163819
Respiration	NULL	Formate hydrogenase	tungsten-containing formate dehydrogenase alpha subunit	0.016015	0.009048	0.045986	0.0249	0.028232	0.0263	0.032494	0.02741	0.052109	0.002203	0.00163	0.011910504
Respiration	NULL	Formate hydrogenase	tungsten-containing formate dehydrogenase beta subunit	0.010793	0.01131	0.012774	0.00747	0.015101	0.015098	0.010831	0.018439	0.018829	0.013217	0.000931	0.00729617
Membrane Transport	Protein translocation across cytoplasmic membrane	Twin-arginine translocation system	Twin-arginine translocation protein TatB	0.005571	0.009048	0.015329	0.01494	0.007222	0.006819	0.006499	0.015948	0.009853	0.006608	0.000349	0.012265453
Membrane Transport	Protein translocation across cytoplasmic membrane	Twin-arginine translocation system	Twin-arginine translocation protein TatC	0.032379	0.029406	0.033212	0.032371	0.03808	0.04286	0.030328	0.038374	0.041162	0.03965	0.062758	0.045788395
Regulation and Cell signaling	NULL	Two-component regulatory systems in Campylobacter	Two-component system response regulator	0.043172	0.05655	0.056206	0.059761	0.036767	0.039937	0.050546	0.045849	0.090425	0.099125	0.011993	0.16217216
Respiration	Electron accepting reactions	Terminal cytochrome C oxidases	Type cbb3 cytochrome oxidase biogenesis protein CcoG, involved in Cu oxidation	0.048742	0.029406	0.045986	0.02739	0.030858	0.040911	0.036826	0.077246	0.052547	0.092517	0.065902	0.052138034
Respiration	NULL	Biogenesis of cbb3-type cytochrome c oxidases	Type cbb3 cytochrome oxidase biogenesis protein CcoI	0.007659	0.015834	0.007664	0.00996	0.009848	0.014611	0.024551	0.025416	0.010509	0.008811	0.024451	0.022282897
DNA Metabolism	NULL	Type I Restriction-Modification	Type I restriction-modification system, DNA-methyltransferase subunit M (EC 2.1.1.72)	0.050831	0.104051	0.066425	0.107072	0.110301	0.104714	0.101814	0.127082	0.11648	0.147587	0.136228	0.149946146
DNA Metabolism	NULL	Type I Restriction-Modification	Type I restriction-modification system, restriction subunit R (EC 3.1.21.3)	0.085647	0.153815	0.137959	0.134462	0.15823	0.138806	0.12131	0.153993	0.142534	0.143181	0.320659	0.202951833
DNA Metabolism	NULL	Type I Restriction-Modification	Type I restriction-modification system, specificity subunit S (EC 3.1.21.3)	0.020193	0.036192	0.020438	0.032371	0.036111	0.023378	0.03105	0.033889	0.02912	0.011014	0.048786	0.041331815
Virulence, Disease and Defense	Resistance to antibiotics and toxic compounds	Multidrug Resistance Efflux Pumps	Type I secretion outer membrane protein, TolC precursor	0.005919	0.004524	0.010219	0.00498	0.015757	0.013637	0.01733	0.025915	0.026493	0.006608	0.006055	0.011003413
Membrane Transport	Protein secretion system, Type II	Widespread colonization island	Type II/IV secretion system ATP hydrolase TadA/VirB11/CpaF, TadA subfamily	0.041431	0.063336	0.053651	0.042331	0.041363	0.029222	0.041159	0.043856	0.055612	0.03965	0.004774	0.035494879
Membrane Transport	Protein secretion system, Type II	Widespread colonization island	Type II/IV secretion system ATPase TadZ/CpaE, associated with Flp pilus assembly	0.002089	0.01131	0.010219	0.01245	0.003283	0.004383	0.004333	0.010964	0.010947	0.004406	0.001048	0.003983314
Membrane Transport	Protein secretion system, Type II	Widespread colonization island	Type II/IV secretion system protein TadC, associated with Flp pilus assembly	0.006267	0.013572	0.030658	0.00996	0.006566	0.009741	0.009387	0.014452	0.009415	0.002203	0.001747	0.005955252
Membrane Transport	Protein secretion system, Type II	Widespread colonization island	Type II/IV secretion system secretin RcpA/CpaC, associated with Flp pilus assembly	0.006615	0.01131	0.012774	0.01245	0.007879	0.014124	0.005055	0.014452	0.01029	0.006608	0.000815	0.005718619
DNA Metabolism	NULL	Restriction-Modification System	Type III restriction-modification system methylation subunit (EC 2.1.1.72)	0.010793	0.029406	0.017884	0.01494	0.029545	0.014124	0.029605	0.022426	0.026274	0.068286	0.043663	0.020074326
Miscellaneous	Plant-Prokaryote DOE project	At1g26220 At1g32070	Tyrosine recombinase XerC	0.020193	0.015834	0.015329	0.01992	0.019697	0.018995	0.011553	0.019436	0.025179	0.011014	0.002794	0.015657186
Protein Metabolism	Protein biosynthesis	tRNA aminoacylation, Tyr	Tyrosyl-tRNA synthetase (EC 6.1.1.1)	0.048394	0.151553	0.112411	0.169323	0.136564	0.104227	0.13864	0.129075	0.114509	0.200454	0.174069	0.132159268
Respiration	Electron accepting reactions	Ubiquinone Menaquinone-cytochrome c reductase complexes	ubiquinol cytochrome C oxidoreductase, cytochrome C1 subunit	0.025416	0.009048	0.033212	0.00996	0.012475	0.031171	0.026717	0.022426	0.027149	0.013217	0.03167	0.020705346
Respiration	Electron accepting reactions	Ubiquinone Menaquinone-cytochrome c reductase complexes	Ubiquinol-cytochrome C reductase iron-sulfur subunit (EC 1.10.2.2)	0.055009	0.018096	0.015329	0.01992	0.019697	0.019969	0.023107	0.025416	0.024741	0.002203	0.023403	0.017313613
Respiration	Electron accepting reactions	Ubiquinone Menaquinone-cytochrome c reductase complexes	Ubiquinol--cytochrome c reductase, cytochrome B subunit (EC 1.10.2.2)	0.051179	0.029406	0.06898	0.054781	0.03808	0.03945	0.049102	0.058807	0.065684	0.046259	0.085579	0.057738337
Cofactors, Vitamins, Prosthetic Groups, Pigments	Quinone cofactors	Ubiquinone Biosynthesis	Ubiquinone biosynthesis monooxygenase UbiB	0.107581	0.108575	0.074089	0.082171	0.103736	0.119325	0.08665	0.112131	0.108379	0.129965	0.117831	0.079390214
Miscellaneous	Plant-Prokaryote DOE project	Experimental-Ubiquinone BiosynthesisVDC	Ubiquinone/menaquinone biosynthesis methyltransferase UbiE (EC 2.1.1.-)	0.059535	0.061074	0.043432	0.069721	0.047929	0.049191	0.044769	0.064787	0.055612	0.110139	0.035047	0.052098595
Cell Wall and Capsule	Gram-Negative cell wall components	KDO2-Lipid A biosynthesis	UDP-2,3-diacylglucosamine hydrolase (EC 3.6.1.-)	0.038994	0.013572	0.007664	0.037351	0.026262	0.032632	0.035382	0.034885	0.038535	0.03965	0.007452	0.041883958
Cell Wall and Capsule	Gram-Negative cell wall components	KDO2-Lipid A biosynthesis	UDP-3-O-[3-hydroxymyristoyl] glucosamine N-acyltransferase (EC 2.3.1.-)	0.036208	0.058812	0.048541	0.037351	0.043989	0.03458	0.042603	0.055318	0.045541	0.233496	0.042033	0.067676904
Cell Wall and Capsule	Gram-Negative cell wall components	KDO2-Lipid A biosynthesis	UDP-3-O-[3-hydroxymyristoyl] N-acetylglucosamine deacetylase (EC 3.5.1.-)	0.021934	0.031668	0.040877	0.054781	0.062373	0.049191	0.041159	0.038374	0.04576	0.041853	0.067066	0.043303753
Cell Wall and Capsule	Gram-Negative cell wall components	Lipid A-Ara4N pathway (Polymyxin resistance)	UDP-4-amino-4-deoxy-L-arabinose--oxoglutarate aminotransferase (EC 2.6.1.-)	0.023327	0.05655	0.035767	0.044821	0.056464	0.036528	0.033938	0.020433	0.060429	0.006608	0.033999	0.06590216
Cell Wall and Capsule	Gram-Negative cell wall components	LOS core oligosaccharide biosynthesis	UDP-galactopyranose mutase (EC 5.4.99.9)	0.001741	0.020358	0.012774	0.00498	0.004596	0.003409	0.002166	0.006479	0.00416	0.004406	0.001281	0.005481987
Cell Wall and Capsule	Capsular and extracellular polysacchrides	Pseudaminic Acid Biosynthesis	UDP-GlcNAc-specific C4,6 dehydratase/C5 epimerase (PseB, first step of pseudaminic acid biosynthesis)	0.002089	0.002262	0.00511	0.00249	0.003283	0.003896	0.008665	0.003987	0.001095	0.01542	0.000233	0.00698066
Clustering-based subsystems	NULL	CBSS-296591.1.peg.2330	UDP-glucose 4-epimerase (EC 5.1.3.2)	0.152145	0.208102	0.240151	0.231574	0.270501	0.225012	0.236122	0.199843	0.201212	0.196048	0.093263	0.188004545
Cell Wall and Capsule	Gram-Negative cell wall components	Lipid A-Ara4N pathway (Polymyxin resistance)	UDP-glucose dehydrogenase (EC 1.1.1.22)	0.120811	0.140243	0.160952	0.209163	0.155604	0.130527	0.141529	0.139541	0.168151	0.191643	0.126098	0.193052706
Cell Wall and Capsule	NULL	UDP-N-acetylmuramate from Fructose-6-phosphate Biosynthesis	UDP-N-acetylenolpyruvoylglucosamine reductase (EC 1.1.1.158)	0.036557	0.031668	0.028103	0.054781	0.040706	0.033119	0.051268	0.050334	0.052766	0.022028	0.004541	0.049140689
Clustering-based subsystems	Clustering-based subsystems	Sporulation-related Hypotheticals	UDP-N-acetylglucosamine 1-carboxyvinyltransferase (EC 2.5.1.7)	0.114892	0.099527	0.097082	0.072211	0.102423	0.099356	0.111923	0.120603	0.098964	0.220279	0.106537	0.113899124
Virulence, Disease and Defense	NULL	Streptococcus agalactiae virulome	UDP-N-acetylglucosamine 2-epimerase (EC 5.1.3.14)	0.103055	0.067859	0.051096	0.082171	0.133937	0.109097	0.099648	0.068774	0.060429	0.114545	0.020958	0.111966625
Virulence, Disease and Defense	NULL	C jejuni colonization of chick caeca	UDP-N-acetylglucosamine 4,6-dehydratase (EC 4.2.1.-)	0.027156	0.070121	0.066425	0.032371	0.065656	0.056984	0.062099	0.059305	0.049044	0.05507	0.104441	0.070595371
Miscellaneous	Plant-Prokaryote DOE project	COG2302	UDP-N-acetylglucosamine--N-acetylmuramyl-(pentapeptide) pyrophosphoryl-undecaprenol	0.036557	0.038454	0.048541	0.02739	0.03808	0.04286	0.041159	0.043357	0.034813	0.050664	0.013506	0.054622676

			N-acetylglucosamine transferase (EC 2.4.1.227)												
Cell Wall and Capsule	NULL	Recycling of Peptidoglycan Amino Acids	UDP-N-acetylmuramate:L-alanyl-gamma-D-glutamyl-meso-diaminopimelate ligase (EC 6.3.2.-)	0.059187	0.042978	0.051096	0.02739	0.025606	0.04286	0.031772	0.036879	0.033061	0.022028	0.050649	0.030446719
Cell Wall and Capsule	NULL	Peptidoglycan biosynthesis--gjo	UDP-N-acetylmuramate--alanine ligase (EC 6.3.2.8)	0.118026	0.092741	0.048541	0.119522	0.10111	0.102765	0.088816	0.088708	0.078821	0.127762	0.075566	0.089802045
Cell Wall and Capsule	NULL	Peptidoglycan Biosynthesis	UDP-N-acetylmuramoylalanine--D-glutamate ligase (EC 6.3.2.9)	0.055705	0.072383	0.038322	0.067231	0.070908	0.072082	0.067876	0.066282	0.057364	0.063881	0.03167	0.081441029
Cell Wall and Capsule	NULL	Peptidoglycan Biosynthesis	UDP-N-acetylmuramoylalanyl-D-glutamate--2,6-diaminopimelate ligase (EC 6.3.2.13)	0.15493	0.106313	0.112411	0.122012	0.068938	0.076465	0.098926	0.076748	0.073347	0.057273	0.029807	0.081598784
Virulence, Disease and Defense	Resistance to antibiotics and toxic compounds	Methicillin resistance in Staphylococci	UDP-N-acetylmuramoylalanyl-D-glutamyl-2,6-diaminopimelate--D-alanyl-D-alanine ligase (EC 6.3.2.10)	0.020193	0.063336	0.06898	0.042331	0.043989	0.054548	0.04838	0.055816	0.056707	0.048461	0.040985	0.069648841
Cell Wall and Capsule	Gram-Negative cell wall components	Lipopolysaccharide assembly	Uncharacterized ABC transporter, ATP-binding protein YrbF	0.013578	0.015834	0.015329	0.00996	0.007222	0.01315	0.007221	0.009967	0.016202	0.008811	0.033882	0.015144482
Cell Wall and Capsule	Gram-Negative cell wall components	Lipopolysaccharide assembly	Uncharacterized ABC transporter, permease component YrbE	0.005919	0.009048	0.017884	0.00996	0.013131	0.00487	0.007943	0.005482	0.013575	0.017622	0.030855	0.013211983
Carbohydrates	Sugar alcohols	Inositol catabolism	uncharacterized domain	0.0094	0.018096	0.017884	0.00249	0.011161	0.011689	0.011553	0.00598	0.01445	0.011014	0.001048	0.007572241
Amino Acids and Derivatives	Glutamine, glutamate, aspartate, asparagine; ammonia assimilation	Aspartate aminotransferase	Uncharacterized PLP-dependent aminotransferase YfdZ	0.029942	0.013572	0.035767	0.01743	0.012475	0.012663	0.019496	0.011961	0.007663	0.011014	0.056238	0.01360637
Membrane Transport	Protein secretion system, Type VI	Type VI secretion systems	Uncharacterized protein ImpC	0.024371	0.004524	0.012774	0.032371	0.025606	0.025813	0.033216	0.02442	0.028025	0.105734	0.106071	0.030880545
Clustering-based subsystems	NULL	Conserved gene cluster associated with Met-tRNA formyltransferase	Uncharacterized protein with LysM domain, COG1652	0.005919	0.01131	0.012774	0.00996	0.010505	0.010228	0.016608	0.013456	0.011823	0.004406	0.004308	0.011121729
Clustering-based subsystems	Clustering-based subsystems	Putative diaminopropionate ammonia-lyase cluster	Uncharacterized protein YgeA of aspartate/glutamate/hydantoin racemase family	0.004178	0.013572	0.012774	0.00498	0.009848	0.00828	0.012998	0.006977	0.003065	0.006608	0.02189	0.008124384
Miscellaneous	Plant-Prokaryote DOE project	COG1836	Undecaprenyl pyrophosphate synthetase (EC 2.5.1.31)	0.099921	0.070121	0.104747	0.082171	0.080756	0.060393	0.06932	0.076748	0.067436	0.103531	0.046224	0.073987104
Miscellaneous	Plant-Prokaryote DOE project	COG0451	Undecaprenyl-diphosphatase (EC 3.6.1.27)	0.058491	0.04524	0.015329	0.037351	0.038737	0.041398	0.044769	0.062295	0.041819	0.03965	0.035745	0.043816457
Cell Wall and Capsule	Capsular and extracellular polysacchrides	Exopolysaccharide Biosynthesis	Undecaprenyl-phosphate galactosephosphotransferase (EC 2.7.8.6)	0.002785	0.018096	0.028103	0.01992	0.013788	0.019482	0.018052	0.012459	0.022989	0.011014	0.001514	0.020192643
Cell Wall and Capsule	Gram-Positive cell wall components	Teichoic and lipoteichoic acids biosynthesis	Undecaprenyl-phosphate N-acetylglucosaminyl 1-phosphate transferase (EC 2.7.8.-)	0.014623	0.029406	0.033212	0.02739	0.030202	0.037989	0.028161	0.015948	0.019924	0.006608	0.01013	0.019088357
Clustering-based subsystems	Two related proteases	CBSS-257314.1.peg.676	Unspecified monosaccharide ABC transport system, ATP-binding protein	0.001044	0.009048	0.012774	0.01245	0.007879	0.014611	0.007943	0.006479	0.006568	0.006608	0.000699	0.010017444
Miscellaneous	NULL	YaaA	UPF0246 protein YaaA	0.001741	0.002262	0.012774	0.00498	0.007222	0.006819	0.020218	0.02442	0.015545	0.050664	0.039355	0.026345088
Respiration	Electron donating reactions	Hydrogenases	Uptake hydrogenase large subunit (EC 1.12.99.6)	0.057794	0.090479	0.066425	0.042331	0.047929	0.054061	0.054878	0.022925	0.027806	0.048461	0.160912	0.069530525
Respiration	Electron donating reactions	Hydrogenases	Uptake hydrogenase small subunit precursor (EC 1.12.99.6)	0.071024	0.04524	0.025548	0.034861	0.015101	0.015585	0.016608	0.011462	0.009415	0.035245	0.077079	0.028593097
Nucleosides and Nucleotides	Pyrimidines	De Novo Pyrimidine Synthesis	Uracil permease	0.002785	0.004524	0.015329	0.01245	0.014444	0.012663	0.010831	0.00598	0.007006	0.011014	0.074867	0.019285551
Nucleosides and Nucleotides	Pyrimidines	De Novo Pyrimidine Synthesis	Uracil phosphoribosyltransferase (EC 2.4.2.9)	0.055009	0.029406	0.033212	0.044821	0.034141	0.033119	0.033216	0.034885	0.051671	0.050664	0.063107	0.042514978
DNA Metabolism	DNA repair	DNA Repair Base Excision	Uracil-DNA glycosylase, family 1	0.012534	0.009048	0.010219	0.00498	0.011818	0.014124	0.026717	0.023423	0.025398	0.030839	0.031321	0.025122487
DNA Metabolism	DNA repair	Uracil-DNA glycosylase	Uracil-DNA glycosylase, family 3	0.00383	0.004524	0.002555	0.00747	0.008535	0.007306	0.008665	0.001993	0.004598	0.011014	0.000233	0.004850967
DNA Metabolism	DNA repair	Uracil-DNA glycosylase	Uracil-DNA glycosylase, family 4	0.01323	0.03393	0.028103	0.034861	0.038737	0.038476	0.044769	0.044852	0.021895	0.013217	0.002096	0.019522184
DNA Metabolism	DNA repair	Uracil-DNA glycosylase	Uracil-DNA glycosylase, family 5	0.00383	0.004524	0.012774	0.01494	0.011818	0.008767	0.012275	0.012957	0.011823	0.002203	0.001397	0.007177853
Amino Acids and Derivatives	Arginine; urea cycle, polyamines	Urea decomposition	Urea ABC transporter, ATPase protein UrtD	0.01323	0.013572	0.00511	0.01245	0.011161	0.011689	0.016608	0.010964	0.034813	0.008811	0.089072	0.018970041
Amino Acids and Derivatives	Arginine; urea cycle, polyamines	Urea decomposition	Urea ABC transporter, ATPase protein UrtE	0.005222	0.004524	0.00511	0.00498	0.007222	0.01802	0.015164	0.013954	0.025398	0.004406	0.068812	0.014552901
Amino Acids and Derivatives	Arginine; urea cycle, polyamines	Urea decomposition	Urea ABC transporter, permease protein UrtC	0.014623	0.018096	0.012774	0.01992	0.008535	0.011689	0.012998	0.015948	0.040724	0.01542	0.064039	0.015933257
Amino Acids and Derivatives	Arginine; urea cycle, polyamines	Urea decomposition	Urea ABC transporter, urea binding protein	0.03029	0.027144	0.022993	0.02241	0.021666	0.019482	0.024551	0.02442	0.047074	0.008811	0.098387	0.024136518
Amino Acids and Derivatives	Arginine; urea cycle, polyamines	Urea decomposition	Urea carboxylase-related ABC transporter, ATPase protein	0.003133	0.006786	0.002555	0.01494	0.007879	0.005357	0.002888	0.013456	0.010947	0.002203	0.000466	0.011673871
Amino Acids and Derivatives	Arginine; urea cycle, polyamines	Urea decomposition	Urea carboxylase-related ABC transporter, periplasmic substrate-binding protein	0.008356	0.01131	0.00511	0.00249	0.006566	0.007793	0.02094	0.00897	0.015107	0.008811	0.002212	0.010490709
Amino Acids and Derivatives	Arginine; urea cycle, polyamines	Urea decomposition	Urea carboxylase-related ABC transporter, permease protein	0.002785	0.004524	0.002555	0.00249	0.003939	0.005357	0.006499	0.00598	0.013794	0.008811	0.000233	0.009978005
Protein Metabolism	Protein processing and modification	G3E family of P-loop GTPases (metallocenter biosynthesis)	Urease accessory protein UreG	0.017408	0.01131	0.015329	0.00498	0.010505	0.010715	0.006499	0.013456	0.020143	0.002203	0.047389	0.010609025
Amino Acids and Derivatives	Arginine; urea cycle, polyamines	Urea decomposition	Urease alpha subunit (EC 3.5.1.5)	0.035164	0.013572	0.020438	0.01992	0.026262	0.020456	0.027439	0.024918	0.056488	0.019825	0.126331	0.027962077
Nucleosides and Nucleotides	Pyrimidines	Novel non-oxidative pathway of Uracil catabolism	Urease beta subunit (EC 3.5.1.5)	0.047349	0.004524	0.00511	0.01245	0.002626	0.005844	0.009387	0.005482	0.007006	0.008811	0.015136	0.003588927
Nucleosides and Nucleotides	Pyrimidines	Novel non-oxidative pathway of Uracil catabolism	Uridine kinase (EC 2.7.1.48)	0.030986	0.040716	0.017884	0.01245	0.008535	0.015098	0.01372	0.024918	0.013575	0.008811	0.008849	0.02599014
Nucleosides and Nucleotides	Pyrimidines	pyrimidine conversions	Uridine kinase (EC 2.7.1.48) [C1]	0.000696	0.004524	0.00511	0.00249	0.00197	0.001461	0.001444	0.004984	0.002627	0.002203	0.000233	0.002326887
Nucleosides and Nucleotides	Pyrimidines	pyrimidine conversions	Uridine phosphorylase (EC 2.4.2.3)	0.002437	0.004524	0.007664	0.00498	0.002626	0.004383	0.002888	0.000498	0.003284	0.004406	0.002911	0.001971938
Clustering-based subsystems	NULL	CBSS-176299.4.peg.1996B	Uridylate kinase (EC 2.7.4.-)	0.085995	0.061074	0.094528	0.049801	0.052524	0.05601	0.054878	0.072761	0.058459	0.112342	0.037725	0.064206293
Miscellaneous	Plant-Prokaryote DOE project	Experimental - Histidine Degradation	Urocanate hydratase (EC 4.2.1.49)	0.055705	0.076907	0.097082	0.064741	0.064342	0.086206	0.08665	0.071764	0.107503	0.121153	0.162192	0.092523319
Carbohydrates	Monosaccharides	D-Galacturonate and D-Glucuronate Utilization	Uronate isomerase (EC 5.3.1.12)	0.040386	0.02262	0.022993	0.054781	0.022323	0.033119	0.018774	0.030898	0.021019	0.013217	0.00163	0.024018202
Cofactors, Vitamins, Prosthetic Groups, Pigments	Tetrapyrroles	Heme and Siroheme Biosynthesis	Uroporphyrinogen III decarboxylase (EC 4.1.1.37)	0.02646	0.018096	0.007664	0.039841	0.022979	0.026787	0.032494	0.043856	0.03941	0.0793	0.03819	0.039044367
Cofactors, Vitamins, Prosthetic Groups, Pigments	Tetrapyrroles	Coenzyme B12 biosynthesis	Uroporphyrinogen-III methyltransferase (EC 2.1.1.107)	0.045957	0.063336	0.043432	0.02739	0.051868	0.047243	0.041881	0.03638	0.037221	0.041853	0.056005	0.040188091
Clustering-based subsystems	NULL	Nucleoside Catabolism cluster	UTP--glucose-1-phosphate uridylyltransferase (EC 2.7.7.9)	0.048394	0.038454	0.051096	0.082171	0.039393	0.054548	0.044769	0.053325	0.044227	0.011014	0.027013	0.043224875
Carbohydrates	Central carbohydrate metabolism	Pyruvate Alanine Serine Interconversions	Valine--pyruvate aminotransferase (EC 2.6.1.66)	0.002437	0.004524	0.002555	0.00249	0.00197	0.003896	0.008665	0.006977	0.016859	0.004406	0.000931	0.005363671
Protein Metabolism	Protein biosynthesis	tRNA aminoacylation, Val	Valyl-tRNA synthetase (EC 6.1.1.9)	0.231873	0.235246	0.311686	0.271414	0.290198	0.270307	0.257062	0.30699	0.259671	0.176223	0.313323	0.271969654
Regulation and Cell signaling	Programmed Cell Death and Toxin-antitoxin Systems	Toxin-antitoxin systems (other than RelBE and MazEF)	VapC toxin protein	0.002089	0.004524	0.012774	0.00498	0.009848	0.020456	0.010831	0.011961	0.007225	0.006608	0.000349	0.003707243
Stress Response	Detoxification	Uptake of selenate and selenite	Various polyols ABC transporter, ATP-binding component	0.00383	0.009048	0.00511	0.00498	0.005909	0.009741	0.005777	0.009469	0.016202	0.004406	0.035279	0.004929844
Stress Response	Detoxification	Uptake of selenate and selenite	Various polyols ABC transporter, periplasmic substrate-binding protein	0.00383	0.006786	0.00511	0.00249	0.011161	0.011689	0.009387	0.00598	0.015107	0.002203	0.001164	0.003273417
Clustering-based subsystems	NULL	USS-DB-7	VgrG protein	0.024371	0.020358	0.020438	0.034861	0.038737	0.027274	0.020218	0.022426	0.039629	0.033042	0.133084	0.029224117
Cofactors, Vitamins, Prosthetic Groups, Pigments	Tetrapyrroles	Coenzyme B12 biosynthesis	Vitamin B12 ABC transporter, ATPase component BtuD	0.002785	0.013572	0.00511	0.00498	0.010505	0.007306	0.007221	0.004485	0.007225	0.008811	0.001863	0.007532802
Cofactors, Vitamins, Prosthetic Groups, Pigments	Tetrapyrroles	Coenzyme B12 biosynthesis	Vitamin B12 ABC transporter, B12-binding component BtuF	0.011489	0.015834	0.012774	0.01743	0.024293	0.015585	0.019496	0.012957	0.014888	0.006608	0.001048	0.017313613
Cofactors, Vitamins, Prosthetic Groups, Pigments	Tetrapyrroles	Coenzyme B12 biosynthesis	Vitamin B12 ABC transporter, permease component BtuC	0.010793	0.02262	0.020438	0.02241	0.022979	0.022404	0.022385	0.022426	0.014013	0.013217	0.00326	0.024254834

Potassium metabolism	NULL	Potassium homeostasis	Voltage-gated potassium channel	0.014623	0.006786	0.012774	0.00747	0.011161	0.01315	0.009387	0.00897	0.012261	0.011014	0.000116	0.008676526
Respiration	ATP synthases	V-Type ATP synthase	V-type ATP synthase subunit A (EC 3.6.3.14)	0.051527	0.070121	0.079199	0.057271	0.074191	0.066724	0.06932	0.037875	0.016421	0.011014	0.003144	0.041607886
Respiration	ATP synthases	V-Type ATP synthase	V-type ATP synthase subunit B (EC 3.6.3.14)	0.029593	0.058812	0.033212	0.034861	0.053838	0.061854	0.058489	0.030898	0.018173	0.037447	0.004541	0.038926051
Protein Metabolism	Protein degradation	Aminopeptidases (EC 3.4.11.-)	Xaa-Pro aminopeptidase (EC 3.4.11.9)	0.058839	0.049764	0.079199	0.064741	0.059747	0.072569	0.076541	0.084223	0.083419	0.068286	0.066018	0.113780808
Nucleosides and Nucleotides	Purines	Purine Utilization	Xanthine dehydrogenase iron-sulfur subunit (EC 1.17.1.4)	0.029942	0.067859	0.040877	0.057271	0.052524	0.043834	0.030328	0.028905	0.034813	0.01542	0.042731	0.033010238
Nucleosides and Nucleotides	Purines	Purine Utilization	Xanthine dehydrogenase, FAD binding subunit (EC 1.17.1.4)	0.008704	0.015834	0.030658	0.02241	0.016414	0.022404	0.018052	0.019436	0.017735	0.004406	0.02678	0.008282139
Clustering-based subsystems	Clustering-based subsystems	Putative diaminopropionate ammonia-lyase cluster	Xanthine dehydrogenase, molybdenum binding subunit (EC 1.17.1.4)	0.07555	0.144767	0.084308	0.154382	0.11818	0.138806	0.101814	0.074256	0.090863	0.046259	0.156953	0.071029198
Nucleosides and Nucleotides	Purines	Purine Utilization	Xanthine/uracil/thiamine/ascorbate permease family protein	0.005571	0.013572	0.007664	0.02241	0.013788	0.009741	0.018774	0.014951	0.026712	0.026433	0.071374	0.021572999
Nucleosides and Nucleotides	Purines	Purine conversions	Xanthine-guanine phosphoribosyltransferase (EC 2.4.2.22)	0.004874	0.01131	0.002555	0.00747	0.006566	0.016559	0.011553	0.014452	0.011385	0.006608	0.001747	0.004850967
Carbohydrates	Monosaccharides	Xylose utilization	Xylonate dehydratase (EC 4.2.1.82)	0.0094	0.002262	0.00511	0.00249	0.009192	0.000487	0.010831	0.003987	0.013575	0.004406	0.000815	0.00189306
Carbohydrates	Monosaccharides	Xylose utilization	Xylose ABC transporter, periplasmic xylose-binding protein XylF	0.006963	0.002262	0.012774	0.01743	0.005909	0.004383	0.002888	0.009469	0.009634	0.004406	0.00163	0.005127038
Carbohydrates	Monosaccharides	Xylose utilization	Xylose ABC transporter, permease protein XylH	0.005571	0.002262	0.002555	0.00249	0.007879	0.005844	0.00361	0.006977	0.01029	0.011014	0.000582	0.004456579
Carbohydrates	Monosaccharides	Xylose utilization	Xylose isomerase (EC 5.3.1.5)	0.028549	0.058812	0.045986	0.037351	0.024949	0.020943	0.021663	0.027908	0.030653	0.026433	0.004541	0.028474781
Carbohydrates	NULL	Sugar utilization in Thermotogales	Xylulose kinase (EC 2.7.1.17)	0.020889	0.024882	0.051096	0.02241	0.032171	0.026787	0.029605	0.010964	0.027149	0.011014	0.004774	0.025437997
Carbohydrates	Central carbohydrate metabolism	Pentose phosphate pathway	Xylulose-5-phosphate phosphoketolase (EC 4.1.2.9)	0.033423	0.031668	0.017884	0.052291	0.013788	0.010228	0.008665	0.004984	0.042914	0.008811	0.001048	0.022835039
Miscellaneous	Plant-Prokaryote DOE project	YrdC-YciO-Sua5 protein family	YciO family	0.010445	0.020358	0.017884	0.01743	0.015757	0.014124	0.023829	0.014452	0.019048	0.013217	0.051697	0.024491467
Miscellaneous	Plant-Prokaryote DOE project	At5g63420	YgjD/Kae1/Qri7 family, required for N6-threonylcarbamoyl adenosine t(6)A37 modification in tRNA	0.077639	0.095003	0.081754	0.077191	0.098483	0.078413	0.064988	0.075252	0.067436	0.05507	0.069395	0.084359497
Respiration	NULL	Quinone oxidoreductase family	YhdH, a putative quinone oxidoreductase	0.008008	0.02262	0.012774	0.00996	0.011161	0.020943	0.023107	0.018938	0.019048	0.037447	0.043779	0.011121729
Cell Wall and Capsule	NULL	YjeE	YjeF protein, function unknown	0.052224	0.063336	0.053651	0.034861	0.072878	0.064776	0.06571	0.05482	0.040505	0.022028	0.039122	0.052414105
Miscellaneous	NULL	Broadly distributed proteins not in subsystems	YpfJ protein, zinc metalloprotease superfamily	0.013578	0.027144	0.012774	0.01992	0.004596	0.007306	0.007943	0.003987	0.008539	0.008811	0.002445	0.008203261
Miscellaneous	Plant-Prokaryote DOE project	COG0523	Zinc ABC transporter, ATP-binding protein ZnuC	0.065454	0.036192	0.020438	0.02988	0.056464	0.035067	0.045491	0.026413	0.018173	0.024231	0.004774	0.029934015
Miscellaneous	Plant-Prokaryote DOE project	COG0523	Zinc ABC transporter, inner membrane permease protein ZnuB	0.030638	0.072383	0.040877	0.01992	0.055151	0.042372	0.050546	0.033889	0.020581	0.006608	0.042149	0.032142585
Miscellaneous	Plant-Prokaryote DOE project	COG0523	Zinc ABC transporter, periplasmic-binding protein ZnuA	0.002785	0.024882	0.010219	0.02988	0.041363	0.039937	0.024551	0.020433	0.012042	0.002203	0.003493	0.020429275
Clustering-based subsystems	NULL	Conserved gene cluster associated with Met-tRNA formyltransferase	Zn-dependent hydrolase, RNA-metabolising	0.002437	0.018096	0.028103	0.01494	0.014444	0.007793	0.007943	0.003489	0.006349	0.01542	0.001747	0.019877133
Miscellaneous	Plant-Prokaryote DOE project	At5g63420	Zn-dependent hydrolase, RNA-metabolising, CPSF 100 kDa analog	0.033771	0.036192	0.022993	0.01494	0.029545	0.037015	0.020218	0.00897	0.006131	0.008811	0.000349	0.020468714
Carbohydrates	Central carbohydrate metabolism	Glycolate, glyoxylate interconversions	(S)-2-hydroxy-acid oxidase (EC 1.1.3.15)	0.019497	0.006786	0.007664	0.01743	0.008535	0.008767	0.006499	0.002492	0.002189	0	0.000116	0.01774744
Protein Metabolism	Protein processing and modification	G3E family of P-loop GTPases (metallocenter biosynthesis)	[NiFe] hydrogenase metallocenter assembly protein HypC	0.014274	0.009048	0.010219	0.01245	0.007222	0.014124	0.012998	0.003489	0.005255	0	0.000815	0.012541524
Carbohydrates	Polysaccharides	Cellulosome	1,4-alpha-glucan branching enzyme (EC 2.4.1.18)	0.003482	0.02262	0.015329	0.00747	0.011818	0.011689	0.012998	0.015948	0.014888	0	0.003609	0.02662116
Carbohydrates	Central carbohydrate metabolism	Glycolysis and Gluconeogenesis, including Archaeal enzymes	2,3-bisphosphoglycerate-independent phosphoglycerate mutase, archaeal type (EC 5.4.2.1)	0.016712	0.038454	0.025548	0.01743	0.014444	0.023378	0.022385	0.007974	0.005036	0	0.000116	0.011871065
Carbohydrates	Fermentation	Acetoin, butanediol metabolism	2,3-butanediol dehydrogenase, R-alcohol forming, (R)- and (S)-acetoin-specific (EC 1.1.1.4)	0.001393	0.002262	0.00511	0	0.002626	0.001461	0.001444	0.001495	0.000438	0.002203	0.000233	0.002050815
Amino Acids and Derivatives	Aromatic amino acids and derivatives	Common Pathway For Synthesis of Aromatic Compounds (DAHP synthase to chorismate)	2-amino-3,7-dideoxy-D-threo-hept-6-ulosonate synthase (EC 2.5.1.-)	0.008704	0.002262	0	0.01245	0.008535	0.006332	0.005055	0.000498	0.001752	0.002203	0.000582	0.008084945
Amino Acids and Derivatives	Aromatic amino acids and derivatives	Tryptophan catabolism	2-amino-3-carboxymuconate-6-semialdehyde decarboxylase (EC 4.1.1.45)	0.007311	0.002262	0.00511	0.00747	0.000657	0.001948	0.001444	0.002492	0.003503	0	0.000233	0.002011377
Cell Wall and Capsule	Gram-Positive cell wall components	Teichoic and lipoteichoic acids biosynthesis	2-C-methyl-D-erythritol 4-phosphate cytidylyltransferase (EC 2.7.7.60)	0.016015	0.036192	0.012774	0.01992	0.022323	0.026787	0.028883	0.029403	0.016859	0	0.002678	0.022874478
Metabolism of Aromatic Compounds	Anaerobic degradation of aromatic compounds	Anaerobic benzoate metabolism	2-hydroxycyclohexanecarboxyl-CoA dehydrogenase (EC 1.1.1.-)	0	0.004524	0.00511	0.00498	0.005909	0.001461	0.007221	0.005482	0.006131	0.002203	0.000116	0.00252408
Carbohydrates	Monosaccharides	L-fucose utilization	2-keto-3-deoxy-L-fuconate dehydrogenase	0.00383	0	0.010219	0.01494	0.006566	0.006819	0.008665	0.004984	0.007882	0.019825	0.001514	0.002090254
Metabolism of Aromatic Compounds	NULL	Benzoate transport and degradation cluster	2-keto-4-pentenoate hydratase/2-oxohepta-3-ene-1,7-dioic acid hydratase (catechol pathway)	0.000696	0.004524	0.012774	0	0.005252	0.003409	0.006499	0.007974	0.010072	0.008811	0.001048	0.004219947
Carbohydrates	Organic acids	Propionate-CoA to Succinate Module	2-methylcitrate dehydratase FeS dependent (EC 4.2.1.79)	0.003482	0.006786	0.010219	0	0.004596	0.010715	0.011553	0.010466	0.035907	0.066084	0.192116	0.029105801
Miscellaneous	Plant-Prokaryote DOE project	Experimental-Ubiquinone BiosynthesisVDC	2-octaprenyl-6-methoxyphenol hydroxylase (EC 1.14.13.-)	0.019497	0	0.010219	0.0249	0.007222	0.014124	0.020218	0.029403	0.021676	0.008811	0.002212	0.013133105
Miscellaneous	Plant-Prokaryote DOE project	At1g52510 AT4G12830 (COG0596)	3-beta hydroxysteroid dehydrogenase/isomerase family protein in hypothetical gene cluster	0.004178	0.015834	0.017884	0.01992	0.013788	0.01802	0.014442	0.004984	0.004379	0.004406	0	0.005955252
Metabolism of Aromatic Compounds	Peripheral pathways for catabolism of aromatic compounds	Quinate degradation	3-dehydroquinate dehydratase I (EC 4.2.1.10)	0.007311	0.004524	0.002555	0	0.004596	0.001948	0.001444	0.004485	0.000657	0.002203	0.000233	0.001025408
Fatty Acids, Lipids, and Isoprenoids	Isoprenoids	Acyclic terpenes utilization	3-hydroxy-3isohexenylglutaryl-CoA:acetate lyase	0.001393	0	0.002555	0.00498	0.00197	0.000974	0.005055	0.009967	0.021457	0.006608	0.000233	0.002957907
Cofactors, Vitamins, Prosthetic Groups, Pigments	NAD and NADP	NAD and NADP cofactor biosynthesis global	3-hydroxyanthranilate 3,4-dioxygenase (EC 1.13.11.6)	0.002785	0.002262	0	0.00498	0.003283	0.00487	0.002888	0.007475	0.007663	0.006608	0.005938	0.002879029
Amino Acids and Derivatives	Lysine, threonine, methionine, and cysteine	Lysine fermentation	3-keto-5-aminohexanoate cleavage enzyme	0.009052	0.01131	0.010219	0.01743	0.007222	0.006819	0.005055	0.004485	0.000876	0.002203	0	0.005994691
Fatty Acids, Lipids, and Isoprenoids	Fatty acids	Phospholipid and Fatty acid biosynthesis related cluster	3-oxoacyl-[ACP] reductase (EC 1.1.1.100)	0.00383	0.004524	0.00511	0.00498	0.003283	0.000487	0.002166	0.007974	0.006787	0	0.001747	0.003352294
Fatty Acids, Lipids, and Isoprenoids	Fatty acids	Phospholipid and Fatty acid biosynthesis related cluster	3-oxoacyl-[ACP] synthase	0.016712	0.006786	0.010219	0.01245	0.005909	0.00487	0.008665	0.003987	0.006131	0	0.002562	0.005915813
DNA Metabolism	NULL	DNA phosphorothioation	3'-phosphoadenosine 5'-phosphosulfate sulfurtransferase DndC	0.000696	0	0.002555	0.00249	0.004596	0.008767	0.005777	0.002492	0.007006	0.002203	0.001979	0.009662495
Metabolism of Aromatic Compounds	Peripheral pathways for catabolism of aromatic compounds	p-Hydroxybenzoate degradation	4-hydroxybenzoate transporter	0.002785	0.004524	0.007664	0.01494	0.001313	0.001461	0.002888	0.000997	0.002627	0.002203	0	0.002800152
Metabolism of Aromatic Compounds	Metabolism of central aromatic intermediates	4-Hydroxyphenylacetic acid catabolic pathway	4-hydroxyphenylacetate 3-monooxygenase (EC 1.14.13.3)	0.015667	0.02262	0.012774	0.02739	0.019697	0.0263	0.015164	0.013954	0.015983	0	0.00163	0.011555555
Amino Acids and Derivatives	Aromatic amino acids and derivatives	Aromatic amino acid degradation	5-carboxymethyl-2-oxo-hex-3- ene-1,7-dioate decarboxylase (EC 4.1.1.68)	0.006615	0	0.00511	0.00249	0.003283	0.003409	0.005777	0.00299	0.004817	0.002203	0.001863	0.005087599
Carbohydrates	Central carbohydrate metabolism	Entner-Doudoroff Pathway	6-phosphogluconolactonase (EC 3.1.1.31)	0	0.004524	0.00511	0.00747	0.003283	0.008767	0.007221	0.007974	0.010509	0.004406	0.000815	0.004022753
Membrane Transport	ABC transporters	Periplasmic-Binding-Protein-Dependent Transport System for -Glucosides	ABC alpha-glucoside transporter, inner membrane subunit AglF	0	0.002262	0.00511	0.00249	0.011161	0.006332	0.001444	0.00598	0.007225	0.006608	0.009082	0.003549488
Miscellaneous	Plant-Prokaryote DOE project	COG3533	ABC transport system, sugar-binding protein	0.002089	0.002262	0.002555	0.00996	0.009848	0.002435	0.000722	0.002492	0.005693	0	0.000582	0.002997345
Clustering-based subsystems	NULL	USS-DB-6	ABC transporter ATP-binding protein USSDB6B	0.002437	0.004524	0	0.01494	0.00197	0.001461	0.002166	0.00598	0.004379	0.002203	0.000582	0.00252408
Clustering-based subsystems	Molybdopterin oxidoreductase	CBSS-269799.3.peg.2220	ABC-type Fe3+ transport system protein	0.01323	0.013572	0.00511	0.00747	0.003283	0.005357	0.000722	0.009967	0.004598	0	0.005356	0.003825559

Sulfur Metabolism	Organic sulfur assimilation	Alkanesulfonate assimilation	ABC-type nitrate/sulfonate/bicarbonate transport system, permease component	0.001393	0.004524	0.002555	0.00249	0.007222	0.002922	0	0.006479	0.008101	0.008811	0.02678	0.004180508
Sulfur Metabolism	Organic sulfur assimilation	Alkanesulfonate assimilation	ABC-type nitrate/sulfonate/bicarbonate transport systems, periplasmic components	0.002785	0.009048	0.010219	0.00747	0.007222	0.010228	0.00361	0.003987	0.006349	0	0.000233	0.00347061
Miscellaneous	Plant-Prokaryote DOE project	COG3533	ABC-type sugar transport system, permease component	0.005222	0.01131	0	0.00747	0.011818	0.00487	0.005055	0.002492	0.008977	0.011014	0.00978	0.004259386
Membrane Transport	ABC transporters	ABC transporter tungstate (TC 3.A.1.6.2)	ABC-type tungstate transport system, ATP-binding protein	0.000696	0.013572	0.012774	0.00747	0.011161	0.007793	0.004333	0.007974	0.008758	0.004406	0	0.005403109
Metabolism of Aromatic Compounds	Peripheral pathways for catabolism of aromatic compounds	Benzoate catabolism	Acetyl-CoA C-acyltransferase (EC 2.3.1.16)	0.004874	0.006786	0	0.00498	0.005909	0.003409	0.005055	0.013954	0.010072	0.002203	0.000931	0.003273417
Carbohydrates	Fermentation	Acetone Butanol Ethanol Synthesis	Acetyl-CoA:acetoacetyl-CoA transferase, alpha subunit (EC 2.8.3.8)	0.000348	0.002262	0	0.00249	0.00197	0.004383	0.006499	0.00299	0.007444	0.002203	0.014438	0.006744027
Clustering-based subsystems	Monosaccharides	Unspecified monosaccharide transport cluster	ACT domain protein	0.001044	0.009048	0.00511	0.00498	0	0.001948	0.001444	0.000498	0.001971	0.002203	0.005007	0.004298824
Virulence, Disease and Defense	NULL	C jejuni colonization of chick caeca	acyl-acyl carrier protein synthetase (EC 6.2.1.20)	0.012186	0.024882	0.00511	0.00996	0.010505	0.009741	0.006499	0.012459	0.003941	0	0.047389	0.020034888
Protein Metabolism	Protein degradation	Omega peptidases (EC 3.4.19.-)	Acylamino-acid-releasing enzyme (EC 3.4.19.1)	0.020541	0.013572	0.017884	0.01245	0.021666	0.018995	0.018774	0.027908	0.046417	0.024231	0	0.012738718
Miscellaneous	Plant-Prokaryote DOE project	At1g52510 AT4G12830 (COG0596)	Acyl-CoA synthetase (AMP-forming)/AMP-acid ligase/Peptide synthase	0.014971	0.009048	0.00511	0.00996	0.022979	0.020943	0.025273	0.010964	0.003503	0	0.000116	0.009662495
Cofactors, Vitamins, Prosthetic Groups, Pigments	Tetrapyrroles	Cobalamin synthesis	Adenosylcobinamide-phosphate synthase	0.011489	0.01131	0	0.00996	0.022323	0.01802	0.014442	0.014452	0.008101	0.006608	0.001048	0.009583617
Sulfur Metabolism	Inorganic sulfur assimilation	Inorganic Sulfur Assimilation	Adenylyl-sulfate reductase [thioredoxin] (EC 1.8.4.10)	0	0.004524	0.002555	0.00249	0.009192	0.004383	0.005055	0.010466	0.006568	0.011014	0.017931	0.004732651
Respiration	Electron accepting reactions	Anaerobic respiratory reductases	Adenylylsulfate reductase beta-subunit (EC 1.8.99.2)	0.009052	0.018096	0.010219	0.01245	0.025606	0.017046	0.015886	0.00299	0.001971	0	0.000699	0.010687903
Nucleosides and Nucleotides	Detoxification	Nudix proteins (nucleoside triphosphate hydrolases)	ADP compounds hydrolase NudE (EC 3.6.1.-)	0.002089	0.006786	0.002555	0	0.004596	0.002435	0.000722	0.004984	0.004379	0.002203	0.016068	0.004141069
Amino Acids and Derivatives	Arginine; urea cycle, polyamines	Arginine and Ornithine Degradation	Agmatine deiminase (EC 3.5.3.12)	0.004526	0.03393	0.020438	0.02988	0.014444	0.009741	0.011553	0.021429	0.025179	0	0.003726	0.024018202
Carbohydrates	Central carbohydrate metabolism	Pyruvate Alanine Serine Interconversions	Alanine racemase, catabolic (EC 5.1.1.1)	0.000348	0.004524	0.002555	0.00996	0.003283	0.002435	0.005777	0.001993	0.003065	0	0.001514	0.002642397
Carbohydrates	NULL	Sugar utilization in Thermotogales	Alcohol dehydrogenase, zinc-containing	0.011837	0.002262	0.00511	0	0.011818	0.006819	0.010109	0.008472	0.009415	0.006608	0.001397	0.005284793
Sulfur Metabolism	Organic sulfur assimilation	Alkanesulfonate assimilation	Alkanesulfonates ABC transporter ATP-binding protein	0.003133	0.009048	0.007664	0.00747	0.003939	0.004383	0.00361	0.003987	0.005474	0	0.000349	0.003943875
Nitrogen Metabolism	NULL	Allantoin Utilization	Allantoicase (EC 3.5.3.4)	0.000348	0.002262	0	0.00498	0.001313	0.003409	0.00361	0.004485	0.002189	0.004406	0.120975	0.002248009
Clustering-based subsystems	NULL	Possible Ammonia conversion cluster	Allophanate hydrolase 2 subunit 2 (EC 3.5.1.54)	0.001741	0.004524	0	0.01494	0.013131	0.012176	0.010831	0.013954	0.015326	0.008811	0.000466	0.012896473
Clustering-based subsystems	NULL	CBSS-316273.3.peg.227	Alpha-2-macroglobulin	0.006963	0.006786	0.007664	0.00498	0.002626	0.002435	0.002888	0.001993	0.006787	0	0.000466	0.006152446
Protein Metabolism	Protein degradation	Dipeptidases (EC 3.4.13.-)	Alpha-aspartyl dipeptidase Peptidase E (EC 3.4.13.21)	0	0.006786	0.010219	0.00747	0.002626	0.000974	0.002166	0.006479	0.005912	0.030839	0.002329	0.010332954
Cell Wall and Capsule	Capsular and extracellular polysacchrides	Rhamnose containing glycans	Alpha-D-GlcNAc alpha-1,2-L-rhamnosyltransferase (EC 2.4.1.-)	0.006615	0.006786	0.007664	0.00498	0.004596	0.004383	0.000722	0.00598	0.001533	0	0.000931	0.004377702
Sulfur Metabolism	NULL	Galactosylceramide and Sulfatide metabolism	Alpha-galactosidase precursor (EC 3.2.1.22)	0.005222	0.036192	0.028103	0.01743	0.005909	0.003409	0.004333	0.001993	0.00832	0	0.000116	0.012423208
Cofactors, Vitamins, Prosthetic Groups, Pigments	NAD and NADP	NAD and NADP cofactor biosynthesis global	Amidases related to nicotinamidase	0.003133	0.01131	0.00511	0.00249	0.004596	0.005844	0.000722	0.001495	0.003941	0	0.000699	0.003746682
Amino Acids and Derivatives	Aromatic amino acids and derivatives	Aromatic amino acid interconversions with aryl acids	Amino acid-binding ACT	0.006963	0.020358	0.010219	0.00249	0.013131	0.017046	0.002888	0.003987	0.000876	0	0.000116	0.005205916
Amino Acids and Derivatives	Aromatic amino acids and derivatives	Chorismate: Intermediate for synthesis of Tryptophan, PAPA antibiotics, PABA, 3-hydroxyanthranilate and more.	Aminodeoxychorismate lyase (EC 4.1.3.38)	0.009748	0.002262	0.010219	0	0.011161	0.015585	0.015886	0.014951	0.01029	0.008811	0.007685	0.02153356
Protein Metabolism	Protein degradation	Protein degradation	Aminopeptidase C (EC 3.4.22.40)	0.008704	0.015834	0.00511	0.00498	0.00197	0.004383	0.004333	0.002492	0.002846	0	0.001048	0.011989382
Clustering-based subsystems	NULL	CBSS-176280.1.peg.1561	Aminopeptidase S (Leu, Val, Phe, Tyr preference) (EC 3.4.11.24)	0.002785	0.004524	0.002555	0	0.005909	0.000974	0.002166	0.002492	0.002189	0.002203	0.000699	0.005481987
Cofactors, Vitamins, Prosthetic Groups, Pigments	Folate and pterines	YgfZ	Anaerobic dimethyl sulfoxide reductase chain B (EC 1.8.99.-)	0.004178	0.020358	0.015329	0.02241	0.020353	0.020456	0.015164	0.010964	0.007225	0	0.000582	0.011003413
Respiration	Electron donating reactions	Respiratory dehydrogenases 1	Anaerobic glycerol-3-phosphate dehydrogenase subunit C (EC 1.1.5.3)	0.000348	0.013572	0.00511	0.00996	0.010505	0.015585	0.015164	0.008472	0.004598	0	0.000349	0.003943875
Cell Wall and Capsule	NULL	Recycling of Peptidoglycan Amino Sugars	Anhydro-N-acetylmuramic acid kinase (EC 2.7.1.-)	0.044564	0	0.030658	0.01245	0.022979	0.021917	0.027439	0.028407	0.029996	0.057273	0.008383	0.024688661
Amino Acids and Derivatives	Arginine; urea cycle, polyamines	Arginine and Ornithine Degradation	Arginase (EC 3.5.3.1)	0.009748	0.015834	0.017884	0.01245	0.011161	0.013637	0.010831	0.014951	0.015764	0	0.000699	0.005600303
Protein Metabolism	Protein degradation	Protein degradation	Arginine-tRNA-protein transferase (EC 2.3.2.8)	0.006267	0.002262	0.010219	0	0.003939	0.005357	0.002888	0.007974	0.011604	0.004406	0.000233	0.005363671
Virulence, Disease and Defense	Resistance to antibiotics and toxic compounds	Arsenic resistance	Arsenical resistance operon repressor	0.000348	0.006786	0.007664	0.00249	0.005909	0.002922	0.004333	0.003489	0.005912	0.011014	0	0.006270762
Amino Acids and Derivatives	Glutamine, glutamate, aspartate, asparagine; ammonia assimilation	Glutamine, Glutamate, Aspartate and Asparagine Biosynthesis	Aspartate racemase (EC 5.1.1.13)	0.001741	0.006786	0.00511	0.00249	0.003939	0.00487	0.000722	0.003987	0.002189	0	0.000349	0.004535457
Respiration	ATP synthases	F0F1-type ATP synthase	ATP synthase delta chain (EC 3.6.3.14)	0.022282	0.01131	0.00511	0.00996	0.031515	0.018995	0.015886	0.019436	0.024741	0	0.022006	0.018733409
Respiration	ATP synthases	F0F1-type ATP synthase	ATP synthase protein I	0.001044	0.009048	0.010219	0.00249	0.011161	0.003409	0.005777	0.00897	0.008977	0	0.000349	0.005481987
Membrane Transport	Protein and nucleoprotein secretion system, Type IV	Vir-like type 4 secretion system	ATPase required for both assembly of type IV secretion complex and secretion of T-DNA complex, VirB4	0.003133	0.002262	0.007664	0.00249	0.005252	0.000974	0.004333	0.00299	0.002189	0	0.000466	0.002169132
Clustering-based subsystems	NULL	CBSS-393121.3.peg.1913	ATP-dependent DNA helicase pcrA (EC 3.6.1.-)	0.011489	0.01131	0.002555	0.01992	0.030858	0.027761	0.026717	0.015449	0.019924	0.011014	0	0.012817595
DNA Metabolism	DNA repair	DNA repair, bacterial RecFOR pathway	ATP-dependent DNA helicase, RecQ family	0.006963	0.02262	0.007664	0	0.013131	0.015098	0.010109	0.010466	0.014013	0.030839	0.105838	0.024175957
DNA Metabolism	DNA repair	ATP-dependent Nuclease	ATP-dependent nuclease, subunit A	0.002785	0.004524	0.00511	0.00249	0.005252	0.001461	0.004333	0.002492	0.005255	0	0.000116	0.004811528
RNA Metabolism	RNA processing and modification	ATP-dependent RNA helicases, bacterial	ATP-dependent RNA helicase Atu1833	0.006267	0.002262	0.00511	0	0.011161	0.016559	0.015164	0.014452	0.011385	0.008811	0.001048	0.003391733
RNA Metabolism	RNA processing and modification	ATP-dependent RNA helicases, bacterial	ATP-dependent RNA helicase NGO0650	0.003133	0.006786	0.00511	0.00249	0.002626	0.000974	0.002166	0.001993	0.002189	0	0.000699	0.00252408
Regulation and Cell signaling	Quorum sensing and biofilm formation	Autoinducer 2 (AI-2) transport and processing (lsrACDBFGE operon)	Autoinducer 2 (AI-2) aldolase LsrF (EC 4.2.1.-)	0.016363	0.027144	0.025548	0.01992	0.01707	0.022404	0.012275	0.000997	0.002408	0	0.000349	0.009346985
Clustering-based subsystems	Biosynthesis of galactoglycans and related lipopolysacharides	CBSS-376686.6.peg.291	Bacterial sugar transferase	0.0094	0.01131	0.012774	0	0.009192	0.00487	0.004333	0.007475	0.01029	0.041853	0.005822	0.013409177
Regulation and Cell signaling	Quorum sensing and biofilm formation	Quorum sensing regulation in Pseudomonas	BarA-associated response regulator UvrY (= GacA = SirA)	0.011837	0.01131	0.002555	0.00996	0.008535	0.01315	0.010831	0.007974	0.007225	0	0.0085	0.011042851
Clustering-based subsystems	NULL	Aerotolerance operon in Bacteroides and potentially orthologous operons in other organisms	BatD	0.003133	0.004524	0	0.00249	0.001313	0.001948	0.001444	0.00598	0.012261	0.019825	0.002445	0.020468714
Metabolism of Aromatic Compounds	Peripheral pathways for catabolism of aromatic compounds	Benzoate degradation	Benzoate transport protein	0.002785	0.006786	0.007664	0	0.001313	0.002435	0.007221	0.003987	0.007006	0.002203	0.027828	0.005324232
Metabolism of Aromatic Compounds	NULL	Benzoate transport and degradation cluster	Benzoate transport, inner-membrane translocator	0.002437	0.013572	0.002555	0.00996	0.002626	0.006332	0.00361	0.006977	0.009196	0	0.013972	0.002918468
Metabolism of Aromatic Compounds	NULL	Benzoate transport and degradation cluster	Benzoate-CoA ligase (EC 6.2.1.25)	0.004526	0.002262	0.012774	0.00996	0.014444	0.00828	0.007943	0.012957	0.032404	0	0.000466	0.00634964
Metabolism of Aromatic Compounds	NULL	Benzoate transport and degradation cluster	Benzoyl-CoA oxygenase component B	0.017756	0	0.007664	0.01743	0.011818	0.00828	0.015164	0.015449	0.022989	0.002203	0.000116	0.004062192
Metabolism of Aromatic Compounds	Anaerobic degradation of aromatic compounds	Anaerobic benzoate metabolism	Benzoyl-CoA reductase subunit BadE (EC 1.3.99.15)	0.02263	0.024882	0.017884	0.01245	0.025606	0.024839	0.023107	0.004984	0.007225	0	0.000233	0.01171331
Cell Wall and Capsule	Gram-Negative cell wall components	LOS core oligosaccharide biosynthesis	Beta-1,3-glucosyltransferase	0.006267	0.002262	0.002555	0.00996	0.013788	0.009254	0.008665	0.005482	0.005036	0	0.000699	0.007572241

Stress Response	Osmotic stress	Choline and Betaine Uptake and Betaine Biosynthesis	Betaine aldehyde dehydrogenase (EC 1.2.1.8)	0.002089	0.009048	0.002555	0	0.008535	0.005844	0.005777	0.011462	0.036126	0.013217	0.151946	0.014119074
Carbohydrates	Di- and oligosaccharides	Maltose and Maltodextrin Utilization	Beta-phosphoglucomutase (EC 5.4.2.6)	0.003482	0.009048	0.012774	0.00249	0.003283	0.002922	0.005777	0.003987	0.010072	0	0.001747	0.013330299
Membrane Transport	Protein secretion system, Type V	Type Vc secretion systems	bifunctional outer membrane translocase / extracellular lipase, PlpD	0.005222	0.013572	0.015329	0.01245	0.007222	0.007306	0.005777	0.006479	0.015764	0.002203	0	0.00918923
Clustering-based subsystems	NULL	Allophanate hydrolase 2 and Biotin carboxylase cluster	Biotin carboxylase (EC 6.3.4.14)	0.011141	0	0.00511	0.00498	0.003283	0.001461	0.004333	0.009469	0.012261	0.004406	0.00163	0.006389078
Miscellaneous	Plant-Prokaryote DOE project	Biotin biosynthesis Experimental	Biotin synthesis protein bioC	0	0.002262	0.010219	0.01743	0.005909	0.00487	0.006499	0.002492	0.003722	0.002203	0.00326	0.008637087
Nucleosides and Nucleotides	Pyrimidines	pyrimidine conversions	Bis(5'-nucleosyl)-tetraphosphatase (asymmetrical) (EC 3.6.1.17)	0.002785	0.004524	0.007664	0.00747	0.005252	0.005844	0.007943	0.002492	0.007225	0	0.000349	0.003864998
Clustering-based subsystems	NULL	EC49-61	Bis(5'-nucleosyl)-tetraphosphatase, symmetrical (EC 3.6.1.41)	0.011489	0.009048	0.007664	0.00996	0.013131	0.009254	0.01372	0.011462	0.021238	0	0.018164	0.016682593
Amino Acids and Derivatives	Branched-chain amino acids	Valine degradation	Branched-chain acyl-CoA dehydrogenase (EC 1.3.99.12)	0.006963	0.002262	0	0.00498	0.008535	0.010715	0.005777	0.016446	0.026712	0.01542	0.144145	0.013093667
Cell Wall and Capsule	Capsular and extracellular polysacchrides	Capsular Polysaccharides Biosynthesis and Assembly	Capsular polysaccharide biosynthesis protein CapD	0.002785	0	0.00511	0.00747	0.000657	0.006819	0.005055	0.003489	0.006349	0.048461	0.0085	0.015065604
Nucleosides and Nucleotides	Pyrimidines	De Novo Pyrimidine Synthesis	Carbamoyl-phosphate synthase large chain B (EC 6.3.5.5)	0.000348	0.009048	0	0.00249	0.002626	0.00487	0.001444	0.000997	0.001314	0.004406	0.000233	0.00220857
Fatty Acids, Lipids, and Isoprenoids	Isoprenoids	Carotenoids	Carotenoid cis-trans isomerase (EC 5.2.-.-)	0.017756	0.009048	0.00511	0.00498	0.006566	0.006332	0.005777	0.016446	0.014232	0	0.000466	0.005718619
Metabolism of Aromatic Compounds	Metabolism of central aromatic intermediates	Central meta-cleavage pathway of aromatic compound degradation	Catechol 2,3-dioxygenase (EC 1.13.11.2)	0.004178	0	0.015329	0.00249	0.00197	0.000487	0.000722	0.002492	0.009634	0.004406	0.216916	0.00918923
Miscellaneous	Plant-Prokaryote DOE project	COG0451	CDP-6-deoxy-delta-3,4-glucoseen reductase-like	0.006963	0.002262	0.017884	0.00747	0.003283	0.004383	0.001444	0.002492	0.008758	0.008811	0	0.006389078
Clustering-based subsystems	NULL	Bacterial Cell Division	Cell division protein BolA	0.002089	0.004524	0.00511	0.01743	0.001313	0.005357	0.010109	0.005482	0.003722	0	0.000116	0.005639742
Carbohydrates	Di- and oligosaccharides	Beta-Glucoside Metabolism	Cellobiose phosphorylase (EC 2.4.1.-)	0.027853	0.029406	0.028103	0.042331	0.017727	0.010228	0.010109	0.014452	0.013356	0.008811	0	0.032418657
Motility and Chemotaxis	Flagellar motility in Prokaryota	Flagellar motility	Chemotaxis protein CheV (EC 2.7.3.-)	0.001741	0	0.002555	0.00249	0.028232	0.007793	0.014442	0.015948	0.022333	0.068286	0.021307	0.036954113
Motility and Chemotaxis	NULL	Bacterial Chemotaxis	Chemotaxis protein CheX	0	0.002262	0.002555	0.00249	0.000657	0.000487	0.002166	0.002492	0.007225	0.006608	0.011061	0.002326887
Carbohydrates	Aminosugars	(GlcNAc)2 Catabolic Operon	Chitobiose phosphorylase (EC 2.4.1.-)	0.006267	0.006786	0	0.00498	0.000657	0.003896	0.001444	0.000498	0.001314	0.011014	0.015253	0.004456579
Cofactors, Vitamins, Prosthetic Groups, Pigments	Tetrapyrroles	Coenzyme B12 biosynthesis	ChlD component of cobalt chelatase involved in B12 biosynthesis	0.016015	0.009048	0.020438	0.01992	0.002626	0.002922	0.002888	0.001993	0.001095	0	0.000116	0.004298824
Cofactors, Vitamins, Prosthetic Groups, Pigments	Tetrapyrroles	Coenzyme B12 biosynthesis	ChlI component of cobalt chelatase involved in B12 biosynthesis	0.001741	0.018096	0.00511	0.02241	0.001313	0.007306	0.00361	0.001993	0.003503	0	0.000699	0.005679181
Virulence, Disease and Defense	Resistance to antibiotics and toxic compounds	Resistance to chromium compounds	Chromate transport protein ChrA	0.005571	0.006786	0	0.00747	0.005909	0.004383	0.005777	0.011462	0.017078	0.017622	0.045526	0.009899127
Clustering-based subsystems	NULL	Bacterial Cell Division	Chromosome partitioning protein ParA	0.009052	0.006786	0	0.00249	0.00197	0.005357	0.005055	0.011961	0.009853	0.006608	0.012458	0.005442548
Cell Division and Cell Cycle	NULL	Cyanobacterial Circadian Clock	Circadian phase modifier	0.014971	0.024882	0.010219	0.0249	0.025606	0.027274	0.018774	0.009469	0.009415	0	0.00163	0.012147137
Respiration	NULL	Carbon monoxide induced hydrogenase	CO dehydrogenase/acetyl-CoA synthase subunit alpha, CO dehydrogenase subcomplex (EC 1.2.99.2)	0.011141	0.009048	0.017884	0.01992	0.005252	0.001948	0.00361	0	0.000876	0.004406	0.000116	0.007335608
Cofactors, Vitamins, Prosthetic Groups, Pigments	Tetrapyrroles	Cobalamin synthesis	Cobalamin synthase	0.005919	0	0.00511	0.00249	0.016414	0.010715	0.006499	0.006479	0.006787	0.004406	0.000815	0.006941221
Cofactors, Vitamins, Prosthetic Groups, Pigments	Tetrapyrroles	Coenzyme B12 biosynthesis	Cobalt-precorrin-2 C20-methyltransferase (EC 2.1.1.130)	0.002089	0.002262	0.00511	0.00249	0.010505	0.00828	0.007943	0.005482	0.005693	0	0.000116	0.002129693
Cofactors, Vitamins, Prosthetic Groups, Pigments	Tetrapyrroles	Coenzyme B12 biosynthesis	Cobalt-precorrin-3b C17-methyltransferase	0.001044	0	0.002555	0.00498	0.011818	0.010228	0.009387	0.010466	0.009196	0.004406	0.001048	0.003864998
Cofactors, Vitamins, Prosthetic Groups, Pigments	Tetrapyrroles	Cobalamin synthesis	Cobalt-precorrin-8x methylmutase (EC 5.4.1.2)	0	0.009048	0.002555	0.00249	0.011818	0.011202	0.012275	0.004984	0.008539	0.004406	0.000931	0.002760713
Respiration	Electron donating reactions	H2:CoM-S-S-HTP oxidoreductase	CoB--CoM heterodisulfide reductase subunit C (EC 1.8.98.1)	0.013926	0.02262	0.035767	0.02988	0.013131	0.016072	0.002888	0.004485	0.005912	0	0.000116	0.009938566
Respiration	Electron accepting reactions	Anaerobic respiratory reductases	CoB--CoM heterodisulfide reductase subunit D (EC 1.8.98.1)	0.014971	0.058812	0.017884	0.01992	0.015101	0.016072	0.014442	0.003987	0.001752	0	0.000116	0.010924535
Respiration	Electron accepting reactions	Anaerobic respiratory reductases	CoB--CoM-reducing hydrogenase (Cys) delta subunit	0.010445	0.01131	0.007664	0.00747	0.003283	0.005844	0.002166	0.000498	0.000438	0	0.000116	0.003943875
Respiration	Electron donating reactions	H2:CoM-S-S-HTP oxidoreductase	CoB--CoM-reducing hydrogenase (Cys) gamma subunit	0.004526	0.009048	0.00511	0.01245	0.004596	0.004383	0.000722	0.001993	0.001095	0	0.000116	0.003076223
Carbohydrates	Glycoside hydrolases	Predicted carbohydrate hydrolases	COG1306 predicted glycoside hydrolase	0.000348	0.006786	0.002555	0.00249	0.005252	0.005357	0.005777	0.003987	0.001971	0.004406	0	0.003391733
Carbohydrates	Glycoside hydrolases	Predicted carbohydrate hydrolases	COG1649 predicted glycoside hydrolase	0	0.004524	0.010219	0.01743	0.000657	0.005844	0.001444	0.007475	0.007882	0.002203	0.000349	0.004535457
Miscellaneous	Plant-Prokaryote DOE project	COG3380 COG2907	COG1683: Uncharacterized conserved protein	0.009748	0.013572	0.007664	0.01494	0.014444	0.010715	0.01372	0.007974	0.007663	0.006608	0	0.013330299
RNA Metabolism	RNA processing and modification	tRNA modification Archaea	COG1720: Uncharacterized conserved protein	0.016015	0.088217	0.038322	0.047311	0.017727	0.023865	0.016608	0.013456	0.012261	0	0.010828	0.022401213
Miscellaneous	Plant-Prokaryote DOE project	COG2078	COG2078: Uncharacterized ACR	0.021934	0.020358	0.025548	0.0249	0.025606	0.02143	0.023829	0.012459	0.004817	0	0.000116	0.02090254
Miscellaneous	Plant-Prokaryote DOE project	Experimental-Ubiquinone BiosynthesisVDC	COG3536: Uncharacterized protein conserved in bacteria	0.007659	0.004524	0.017884	0.01245	0.006566	0.009741	0.006499	0.018439	0.007444	0	0.008849	0.00855821
Stress Response	Cold shock	Cold shock, CspA family of proteins	Cold shock protein CspC	0.008356	0.013572	0.012774	0.00747	0.007879	0.00828	0.005055	0.010466	0.008539	0	0.000699	0.009228669
Stress Response	Cold shock	Cold shock, CspA family of proteins	Cold shock protein CspD	0.028897	0.006786	0.00511	0.00996	0.005909	0.002435	0.005777	0.003489	0.007225	0	0.000116	0.004298824
Miscellaneous	Plant-Prokaryote DOE project	At5g04520 AT1G06240	Colicin V production protein	0.003482	0	0.002555	0.00747	0.005252	0.005844	0.004333	0.007475	0.01029	0.002203	0.019212	0.004298824
Clustering-based subsystems	NULL	Glutaredoxin 3 containing cluster 2	Competence protein F homolog, phosphoribosyltransferase domain	0.001044	0	0.015329	0.00996	0.012475	0.013637	0.01372	0.013456	0.005912	0.002203	0.000349	0.012541524
RNA Metabolism	RNA processing and modification	tRNA modification Archaea	conserved hypothetical protein [Pyrococcus horikoshii]; COG2102: Predicted ATPases of PP-loop superfamily; IPR002761: Domain of unknown function DUF71	0.002785	0.004524	0.00511	0.00249	0.008535	0.00487	0.010109	0.00598	0.00832	0	0.020725	0.004811528
Virulence, Disease and Defense	Resistance to antibiotics and toxic compounds	Copper homeostasis	CopG protein	0.003133	0.002262	0.007664	0.00498	0.007879	0.001948	0.010831	0.007475	0.004379	0	0.000233	0.004219947
Miscellaneous	Plant-Prokaryote DOE project	COG0523	Creatinine amidohydrolase (EC 3.5.2.10)	0.002785	0.01131	0	0.00996	0.014444	0.016559	0.01372	0.02442	0.016202	0.002203	0.000466	0.009978005
Fatty Acids, Lipids, and Isoprenoids	Fatty acids	Carnitine Metabolism in Microorganisms	Crotonobetaine carnitine-CoA ligase (EC 6.3.2.-)	0.003482	0.009048	0.010219	0.00249	0.003283	0.00487	0.002888	0.000997	0.002627	0.002203	0	0.002681835
Amino Acids and Derivatives	Arginine; urea cycle, polyamines	Cyanophycin Metabolism	Cyanophycinase (EC 3.4.15.6)	0.00383	0	0.00511	0.00996	0.000657	0.003409	0.00361	0.00299	0.006568	0.004406	0.000815	0.001380356
Regulation and Cell signaling	NULL	cAMP signaling in bacteria	Cyclic AMP receptor protein	0.002089	0.004524	0.015329	0.00249	0.007879	0.005357	0.009387	0.010964	0.011604	0	0.005007	0.015026166
Clustering-based subsystems	NULL	CBSS-291331.3.peg.3674	Cyclopropane-fatty-acyl-phospholipid synthase-like protein, clusters with FIG005069	0.017408	0.009048	0.015329	0.01743	0.016414	0.023378	0.015164	0.011462	0.007444	0	0.002794	0.009780811
Respiration	NULL	Biogenesis of c-type cytochromes	Cytochrome c heme lyase subunit CcmL	0.01323	0	0.010219	0.00498	0.003283	0.00828	0.010831	0.012957	0.009853	0.013217	0.016534	0.005758058
Respiration	NULL	Soluble cytochromes and functionally related electron carriers	Cytochrome c551/c552	0	0.004524	0.010219	0.00249	0.002626	0.004383	0.002888	0.007475	0.00832	0.002203	0.000233	0.003273417
Respiration	NULL	Soluble cytochromes and functionally related electron carriers	Cytochrome c553	0.014274	0.009048	0.007664	0.00498	0.008535	0.007306	0	0.002492	0.003941	0.006608	0.000233	0.010175199
Respiration	NULL	Biogenesis of c-type cytochromes	Cytochrome c-type biogenesis protein CcdA (DsbD analog)	0.006963	0.004524	0.015329	0.00996	0.005252	0.005844	0.008665	0.00897	0.007663	0	0.000582	0.007177853
Respiration	NULL	Biogenesis of c-type cytochromes	Cytochrome c-type biogenesis protein Ccs1/ResB	0.015667	0.006786	0.00511	0.01494	0.015757	0.01802	0.007221	0.006977	0.003503	0	0.000699	0.009701934
Respiration	NULL	Biogenesis of cytochrome c oxidases	Cytochrome oxidase biogenesis protein Cox11-CtaG, copper delivery to Cox1	0.00383	0.006786	0.012774	0.00498	0.008535	0.013637	0.015886	0.017941	0.015326	0	0.000815	0.007690557

Nucleosides and Nucleotides	Purines	Purine Utilization	Cytosine/purine/uracil/thiamine/allantoin permease family protein	0	0.009048	0.007664	0.00498	0.00197	0.002922	0.002166	0.000997	0.007225	0.024231	0.077545	0.004338263
Respiration	Electron donating reactions	Respiratory dehydrogenases 1	D-amino acid dehydrogenase small subunit (EC 1.4.99.1)	0.003482	0	0.007664	0.01992	0.003283	0.013637	0.022385	0.013954	0.038097	0.008811	0.042033	0.021572999
Regulation and Cell signaling	Programmed Cell Death and Toxin-antitoxin Systems	Phd-Doc, YdcE-YdcD toxin-antitoxin (programmed cell death) systems	Death on curing protein, Doc toxin	0.021238	0.02262	0.00511	0.00747	0.030858	0.024352	0.016608	0.012957	0.008977	0	0.001514	0.008676526
Miscellaneous	Plant-Prokaryote DOE project	COG0398	DedA family protein, putative	0.002785	0.002262	0	0.00249	0.002626	0.00487	0.005055	0.003489	0.002189	0.011014	0.000466	0.003549488
Membrane Transport	Protein translocation across cytoplasmic membrane	Twin-arginine translocation system	Deoxyribonuclease TatD	0.001044	0.004524	0.00511	0.00249	0.005909	0.001461	0.002888	0.005482	0.009415	0	0.020027	0.004456579
Cell Wall and Capsule	Capsular and extracellular polysacchrides	Capsular heptose biosynthesis	D-glycero-D-manno-heptose 1-phosphate guanosyltransferase	0.024023	0.013572	0.00511	0.02988	0.010505	0.013637	0.010831	0.007974	0.007882	0	0.001164	0.018181266
Membrane Transport	Uni- Sym- and Antiporters	Proton-dependent Peptide Transporters	Di/tripeptide permease YjdL	0.008008	0.002262	0.017884	0.00249	0.000657	0.002922	0.007221	0.010964	0.017297	0	0.017232	0.007256731
Secondary Metabolism	Biosynthesis of phenylpropanoids	Biflavanoid biosynthesis	Dihydroflavonol-4-reductase (EC 1.1.1.219)	0.005919	0.02262	0.010219	0.01494	0.010505	0.006819	0.007221	0.006479	0.009853	0	0.000349	0.007256731
Carbohydrates	Fermentation	Acetoin, butanediol metabolism	Dihydrolipoamide dehydrogenase of acetoin dehydrogenase (EC 1.8.1.4)	0.001044	0.009048	0.00511	0.00498	0.005909	0.001461	0.005777	0.001993	0.001752	0.002203	0	0.004062192
Cofactors, Vitamins, Prosthetic Groups, Pigments	Folate and pterines	YgfZ	Dihydroorotate dehydrogenase, catalytic subunit (EC 1.3.3.1)	0.037949	0.072383	0.045986	0.044821	0.068938	0.061854	0.054878	0.024918	0.017516	0	0.001863	0.039714826
Cofactors, Vitamins, Prosthetic Groups, Pigments	Folate and pterines	Pterin biosynthesis	Dihydropteridine reductase (EC 1.5.1.34)	0.003482	0.006786	0.00511	0.01245	0.014444	0.011202	0.006499	0.010466	0.005693	0	0.029225	0.028317026
Nucleosides and Nucleotides	Pyrimidines	Novel non-oxidative pathway of Uracil catabolism	Dihydropyrimidine dehydrogenase [NADP+] (EC 1.3.1.2)	0.018452	0	0.010219	0.01494	0.008535	0.009254	0.011553	0.006479	0.014888	0.002203	0.002562	0.006152446
Miscellaneous	Plant-Prokaryote DOE project	At1g48360	DinG family ATP-dependent helicase CPE1197	0	0.015834	0.012774	0.00498	0.015757	0.012176	0.012998	0.010964	0.010728	0.008811	0.000349	0.011871065
Regulation and Cell signaling	NULL	Global Two-component Regulator PrrBA in Proteobacteria	Dna binding response regulator PrrA (RegA)	0.014623	0.004524	0	0.00249	0.005909	0.003409	0.010109	0.013456	0.007882	0.011014	0.01502	0.005205916
DNA Metabolism	DNA replication	DNA Helicase of Unknown Function	DNA helicase (Rad25 homolog)	0.001044	0.004524	0.002555	0.00996	0.007879	0.00487	0.002888	0.004485	0.006349	0	0.000349	0.001538111
Clustering-based subsystems	Recombination related cluster	CBSS-198094.1.peg.4426	DNA polymerase X family	0.003482	0.004524	0.00511	0.00249	0.010505	0.006819	0.010831	0.010964	0.013137	0	0.000931	0.010569586
DNA Metabolism	DNA repair	DNA repair, bacterial	DNA polymerase-like protein PA0670	0.003133	0.006786	0.010219	0.00498	0.002626	0.005357	0.005777	0.008472	0.009415	0	0.000233	0.002997345
DNA Metabolism	DNA recombination	DNA recombination, archaeal	DNA repair and recombination protein RadA	0.002785	0.004524	0	0.00249	0.011818	0.011202	0.016608	0.004485	0.004598	0.004406	0.002562	0.005718619
DNA Metabolism	DNA repair	DNA repair, bacterial	DNA repair exonuclease family protein YhaO	0.007311	0.013572	0	0.00747	0.011818	0.009741	0.009387	0.007974	0.003065	0.006608	0.000931	0.005127038
DNA Metabolism	DNA replication	DNA topoisomerases, Type II, ATP-dependent	DNA topoisomerase VI subunit A (EC 5.99.1.3)	0.008008	0.015834	0.007664	0.00747	0.013788	0.010715	0.005777	0.004984	0.003941	0	0.001164	0.004180508
RNA Metabolism	Transcription	RNA polymerase archaeal	DNA-directed RNA polymerase subunit B (EC 2.7.7.6)	0.005571	0	0.020438	0.02241	0.008535	0.006332	0.023107	0.004485	0.003722	0.002203	0.000699	0.005915813
Cell Wall and Capsule	Capsular and extracellular polysacchrides	dTDP-rhamnose synthesis	dTDP-Rha:A-D-GlcNAc-diphosphoryl polyprenol, A-3-L-rhamnosyl transferase WbbL	0.002437	0.002262	0.007664	0.00249	0.003283	0.004383	0.005055	0.003489	0.002627	0.002203	0	0.005639742
Carbohydrates	NULL	Sugar utilization in Thermotogales	Endoglucanase (EC 3.2.1.4)	0.011837	0	0.00511	0.00498	0.00197	0.003896	0.005777	0.002492	0.005036	0.002203	0.000466	0.004850967
Fatty Acids, Lipids, and Isoprenoids	Fatty acids	Fatty Acid Biosynthesis FASII	Enoyl-[acyl-carrier-protein] reductase [NADPH] (EC 1.3.1.10)	0	0.009048	0.012774	0.00747	0.004596	0.009741	0.008665	0.00897	0.011823	0.002203	0.000233	0.005797497
Carbohydrates	Central carbohydrate metabolism	Dehydrogenase complexes	Enoyl-CoA hydratase [branched-chain amino acid degradation] (EC 4.2.1.17)	0.003482	0	0.007664	0.00249	0.004596	0.00487	0.00361	0.004984	0.006787	0.004406	0.002911	0.005442548
DNA Metabolism	DNA repair	DNA repair, bacterial UmuCD system	Error-prone, lesion bypass DNA polymerase V (UmuC)	0.005571	0.004524	0.015329	0.00996	0.007879	0.004383	0.010109	0.008472	0.011823	0	0.016068	0.011831626
Cofactors, Vitamins, Prosthetic Groups, Pigments	Pyridoxine	Pyridoxin (Vitamin B6) Biosynthesis	Erythronate-4-phosphate dehydrogenase (EC 1.1.1.290)	0.004526	0	0.010219	0.00747	0.006566	0.002435	0.002888	0.008472	0.011166	0.004406	0.021773	0.008913159
Carbohydrates	Sugar alcohols	Ethanolamine utilization	Ethanolamine ammonia-lyase heavy chain (EC 4.3.1.7)	0	0.006786	0.00511	0.00498	0.009192	0.003409	0.002166	0.004485	0.009196	0.028636	0.070326	0.007808873
Carbohydrates	Sugar alcohols	Ethanolamine utilization	Ethanolamine utilization protein similar to PduL	0.002089	0.004524	0.002555	0.00249	0.00197	0.003409	0.000722	0.00299	0.001095	0	0.000582	0.002405764
DNA Metabolism	DNA repair	DNA repair, UvrABC system	Excinuclease ABC subunit A paralog in greater Bacteroides group	0.016015	0.024882	0.012774	0.01494	0.006566	0.014124	0.008665	0.015948	0.017735	0.004406	0	0.024412589
DNA Metabolism	DNA repair	DNA repair, UvrABC system	Excinuclease ABC subunit A paralog of unknown function	0.004874	0.004524	0.00511	0.00498	0.001313	0.001461	0.002166	0.002492	0.005912	0.004406	0	0.003588927
RNA Metabolism	RNA processing and modification	RNA processing and degradation, bacterial	Exoribonuclease II (EC 3.1.13.1)	0.001044	0.006786	0.007664	0.00498	0.00197	0.003409	0.000722	0.00598	0.003503	0	0.000699	0.005403109
Virulence, Disease and Defense	Toxins and superantigens	Streptolysin S Biosynthesis and Transport	Export ABC transporter ATP-binding protein	0.014971	0.006786	0	0.00498	0.005909	0.001461	0.000722	0.00299	0.008101	0.004406	0.000931	0.007966629
Miscellaneous	Plant-Prokaryote DOE project	Iron-sulfur cluster assembly	Ferredoxin, 2Fe-2S	0.019845	0.013572	0.00511	0.00747	0.010505	0.007306	0.01372	0.015449	0.007663	0	0.024568	0.008084945
Iron acquisition and metabolism	NULL	Campylobacter Iron Metabolism	Ferric uptake regulation protein	0.000348	0.004524	0.00511	0.00249	0.00197	0.002435	0.004333	0.00299	0.001752	0	0.001281	0.007808873
Iron acquisition and metabolism	NULL	Iron acquisition in Vibrio	Ferrichrome-iron receptor	0	0.004524	0.00511	0.00747	0.008535	0.014611	0.011553	0.041364	0.031309	0.004406	0.022122	0.010372393
Stress Response	Oxidative stress	Rubrerythrin	Fe-S oxidoreductase-like protein in Rubrerythrin cluster	0.006267	0.002262	0.012774	0.01743	0.000657	0.001461	0.00361	0.000997	0.005255	0	0.000582	0.003115662
Respiration	Electron accepting reactions	Anaerobic respiratory reductases	Fe-S-cluster-containing hydrogenase components 1	0.04909	0.029406	0.071534	0.049801	0.046615	0.062341	0.030328	0.032892	0.037002	0	0.001863	0.055214257
RNA Metabolism	RNA processing and modification	rRNA modification Archaea	Fibrillarin	0	0.004524	0.002555	0.00249	0.001313	0.002435	0.005055	0.001495	0.000876	0.002203	0.000233	0.00189306
Miscellaneous	Plant-Prokaryote DOE project	COG3760	FIG000875: Thioredoxin domain-containing protein EC-YbbN	0.004526	0.006786	0.002555	0.00249	0.003939	0.002435	0.005055	0.000997	0.005255	0	0.000233	0.001419795
Clustering-based subsystems	NULL	Cluster containing CofD-like protein and co-occuring with DNA repair	FIG002813: LPPG:FO 2-phospho-L-lactate transferase like, CofD-like	0.008008	0	0.012774	0.044821	0.011818	0.007793	0.008665	0.006977	0.009853	0.011014	0.000349	0.006428517
Amino Acids and Derivatives	Lysine, threonine, methionine, and cysteine	Threonine degradation	FIG003492: Threonine dehydrogenase and related Zn-dependent dehydrogenases	0.003133	0.002262	0.010219	0	0.011161	0.012176	0.011553	0.017443	0.014232	0.004406	0.001863	0.001971938
Miscellaneous	Plant-Prokaryote DOE project	At2g33980 At1g28960	FIG003620: Proteophosphoglycan precursor (Fragment)	0.00383	0.004524	0.002555	0.00249	0	0.001948	0.006499	0.009967	0.006787	0.006608	0.000349	0.005363671
Miscellaneous	Plant-Prokaryote DOE project	At4g10620 At3g57180 At3g47450	FIG004454: RNA binding protein	0.008008	0.002262	0.002555	0.00498	0.001313	0.001948	0.002888	0.005482	0.005912	0	0.001863	0.004338263
Clustering-based subsystems	NULL	CBSS-291331.3.peg.3674	FIG005069: Hypothetical protein	0.0094	0.01131	0.00511	0.01743	0.008535	0.007306	0.008665	0.007974	0.003722	0	0.000349	0.006073568
Regulation and Cell signaling	Programmed Cell Death and Toxin-antitoxin Systems	Phd-Doc, YdcE-YdcD toxin-antitoxin (programmed cell death) systems	FIG022160: hypothetical toxin	0.001044	0.002262	0.010219	0	0.007222	0.00828	0.005777	0.004485	0.003503	0.002203	0.000233	0.003588927
Clustering-based subsystems	NULL	Cell wall related cluster	FIG022606: AAA ATPase	0.008704	0.013572	0.015329	0.00747	0.011818	0.011202	0.00361	0.005482	0.005036	0	0.000699	0.009307546
Clustering-based subsystems	Sulfatases and sulfatase modifying factor 1 (and a hypothetical)	Sulfatases and sulfatase modifying factor 1	FIG068086: hypothetical protein	0.002089	0.01131	0.00511	0.00498	0.000657	0.002435	0.002166	0.001495	0.014013	0.002203	0	0.013054228
Membrane Transport	Protein secretion system, Type II	Predicted secretion system W clustering with cell division proteins	FIG073201: glycosyl transferase	0	0.004524	0.002555	0.00249	0.002626	0.001461	0.002888	0.001495	0.002627	0.006608	0.000815	0.003076223
Clustering-based subsystems	NULL	CBSS-176299.4.peg.1996A	FIG110192: hypothetical protein	0.013578	0	0.00511	0.00996	0.010505	0.006819	0.014442	0.013954	0.012261	0.006608	0.001747	0.002129693
Fatty Acids, Lipids, and Isoprenoids	Fatty acids	Phospholipid and Fatty acid biosynthesis related cluster	FIG143263: Glycosyl transferase	0.002785	0.002262	0.002555	0.00498	0.002626	0.000974	0.000722	0.000997	0.001314	0	0.000582	0.001616989
Clustering-based subsystems	NULL	CBSS-138119.3.peg.2719	FIG146085: 3'-to-5' oligoribonuclease A, Bacillus type	0.001044	0.006786	0.00511	0.00498	0.011818	0.012663	0.010831	0.002492	0.005036	0.022028	0	0.011476678
Protein Metabolism	Protein folding	Peptidyl-prolyl cis-trans isomerase	FKBP-type peptidyl-prolyl cis-trans isomerase FkpA precursor (EC 5.2.1.8)	0.012882	0	0.002555	0.00249	0.005252	0.003409	0.004333	0.008472	0.013794	0.002203	0.016068	0.00982025
Motility and Chemotaxis	Flagellar motility in Prokaryota	Flagellar motility	Flagellar basal-body rod modification protein FlgD	0	0.004524	0.00511	0.00747	0.007879	0.012176	0.011553	0.00897	0.015107	0.006608	0.005938	0.00698066
Motility and Chemotaxis	Flagellar motility in Prokaryota	Flagellum	Flagellar basal-body rod protein FlgF	0	0.002262	0.020438	0.00249	0.01904	0.014611	0.014442	0.018938	0.015326	0.002203	0.024917	0.01076678
Motility and Chemotaxis	Flagellar motility in Prokaryota	Flagellum	Flagellar biosynthesis protein FliC	0.004874	0.002262	0.002555	0	0.007879	0.001948	0.00361	0.006977	0.00416	0.013217	0.000349	0.003904437

Motility and Chemotaxis	Flagellar motility in Prokaryota	Flagellum	Flagellar hook-associated protein FlgL	0	0.006786	0.002555	0.00498	0.013788	0.00828	0.006499	0.010466	0.012042	0.002203	0.000582	0.003549488
Motility and Chemotaxis	Flagellar motility in Prokaryota	Flagellum	Flagellar L-ring protein FlgH	0	0.002262	0.00511	0.00498	0.019697	0.017046	0.012275	0.017941	0.019048	0.006608	0.007917	0.01297535
Motility and Chemotaxis	Flagellar motility in Prokaryota	Flagellum	Flagellar protein FlgJ [peptidoglycan hydrolase] (EC 3.2.1.-)	0.004874	0.009048	0	0.00249	0.009848	0.007306	0.004333	0.009967	0.01029	0.006608	0.017349	0.006152446
Motility and Chemotaxis	Flagellar motility in Prokaryota	Flagellum	Flagellin protein FlaB	0.011489	0.004524	0.00511	0	0.011161	0.012176	0.012275	0.014452	0.012918	0.002203	0.029807	0.005955252
Miscellaneous	Plant-Prokaryote DOE project	COG1565	Folate biosynthesis protein PTPS-III, catalyzes a reaction that bypasses dihydroneopterin aldolase (FolB)	0.003133	0.002262	0.00511	0.00249	0.003939	0.001461	0.002166	0.001495	0.000219	0.002203	0	0.001380356
Cofactors, Vitamins, Prosthetic Groups, Pigments	Folate and pterines	YgfZ	Folate-dependent protein for Fe/S cluster synthesis/repair in oxidative stress	0.011141	0.002262	0.007664	0	0.010505	0.015585	0.010109	0.012957	0.020362	0.002203	0.011178	0.009307546
Membrane Transport	Protein translocation across cytoplasmic membrane	EcsAB transporter affecting expression and secretion of secretory preproteins	Foldase protein PrsA precursor (EC 5.2.1.8)	0.009748	0.006786	0.015329	0.01743	0.013131	0.010715	0.010109	0.003489	0.005474	0	0.000233	0.009110352
Carbohydrates	One-carbon Metabolism	Formaldehyde assimilation: Ribulose monophosphate pathway	Formaldehyde activating enzyme	0.004874	0.002262	0.012774	0.00498	0.003939	0.001461	0.002888	0.001993	0.002189	0.004406	0	0.001183163
Respiration	Electron donating reactions	Formate dehydrogenase	Formate dehydrogenase alpha subunit (EC 1.2.1.2)	0.012882	0.004524	0.012774	0.00498	0.015101	0.019969	0.002888	0.000997	0.001752	0	0.000466	0.009386424
Virulence, Disease and Defense	NULL	Bacterial cyanide production and tolerance mechanisms	Formate dehydrogenase O beta subunit (EC 1.2.1.2)	0.012534	0.006786	0.017884	0.00498	0.005252	0.006819	0.002888	0.001993	0.004598	0	0.001048	0.006152446
Respiration	NULL	Formate hydrogenase	Formate dehydrogenase-O, iron-sulfur subunit (EC 1.2.1.2)	0.01706	0.009048	0.010219	0.00996	0.006566	0.005357	0.007221	0.004485	0.006349	0	0.000116	0.004219947
Miscellaneous	Plant-Prokaryote DOE project	Experimental - Histidine Degradation	Formiminoglutamic iminohydrolase (EC 3.5.3.13)	0.005571	0	0.010219	0.00249	0.009848	0.004383	0.004333	0.005482	0.011166	0.004406	0.000466	0.002248009
Cofactors, Vitamins, Prosthetic Groups, Pigments	Folate and pterines	5-FCL-like protein	Formiminotetrahydrofolate cyclodeaminase (EC 4.3.1.4)	0.002089	0.006786	0.00511	0.01743	0.003939	0.005844	0.005055	0.001495	0.002408	0	0.000116	0.003983314
Carbohydrates	Central carbohydrate metabolism	Glycolysis and Gluconeogenesis	Fructose-1,6-bisphosphatase, Bacillus type (EC 3.1.3.11)	0	0.002262	0.002555	0.00498	0.005252	0.002922	0.005055	0.00299	0.003941	0.008811	0.000815	0.00347061
Stress Response	Oxidative stress	Oxidative stress	Fumarate and nitrate reduction regulatory protein	0	0.002262	0.002555	0.00747	0.007879	0.003896	0.004333	0.005482	0.008977	0.008811	0.035629	0.009268107
Respiration	Electron donating reactions	Succinate dehydrogenase	Fumarate reductase flavoprotein subunit (EC 1.3.99.1)	0.012186	0.024882	0.022993	0.00249	0.017727	0.006332	0.002888	0.004485	0.013137	0	0.000582	0.006862343
Carbohydrates	Monosaccharides	D-galactonate catabolism	Galactonate dehydratase (EC 4.2.1.6)	0.008704	0	0.007664	0.00249	0.003283	0.007793	0.008665	0.009967	0.009853	0.008811	0.000699	0.003076223
Carbohydrates	Di- and oligosaccharides	Lactose utilization	Galactoside O-acetyltransferase (EC 2.3.1.18)	0.002089	0.002262	0.002555	0.00498	0.00197	0.003896	0.005055	0.003987	0.004817	0	0.004774	0.005915813
Cell Wall and Capsule	Capsular and extracellular polysacchrides	Alginate metabolism	GDP-mannose 6-dehydrogenase (EC 1.1.1.132)	0.000696	0.009048	0.002555	0.00747	0.008535	0.009741	0.000722	0.004984	0.014013	0.019825	0	0.006783466
Phages, Prophages, Transposable elements, Plasmids	Gene Transfer Agent (GTA)	Gene Transfer Agent	Gene Transfer Agent host specificity protein	0.001741	0	0.007664	0.00498	0.133937	0.009254	0.018052	0.022925	0.015764	0.008811	0.000116	0.003076223
Phages, Prophages, Transposable elements, Plasmids	Gene Transfer Agent (GTA)	Gene Transfer Agent	Gene Transfer Agent terminase protein	0.008704	0	0.002555	0.00249	0.024949	0.032632	0.032494	0.042361	0.004379	0.002203	0.001164	0.001735305
Membrane Transport	Protein secretion system, Type II	General Secretion Pathway	General secretion pathway protein D	0.083558	0.024882	0.028103	0.052291	0.064999	0.045295	0.041881	0.034885	0.053642	0	0.066251	0.044762987
Fatty Acids, Lipids, and Isoprenoids	Isoprenoids	Acyclic terpenes utilization	Geranyl-CoA carboxylase biotin-containing subunit	0.005222	0.009048	0	0.00498	0.003283	0.003409	0.002888	0.008472	0.012261	0.006608	0.002212	0.002918468
Stress Response	Osmotic stress	Synthesis of osmoregulated periplasmic glucans	Glucans biosynthesis glucosyltransferase H (EC 2.4.1.-)	0.001044	0.020358	0.022993	0	0.007222	0.001461	0.002888	0.006479	0.015107	0.01542	0.019561	0.002642397
Carbohydrates	NULL	Sugar utilization in Thermotogales	Gluconokinase (EC 2.7.1.12)	0.000348	0.01131	0.002555	0	0.003283	0.00487	0.007221	0.00299	0.009196	0.004406	0.000349	0.002879029
Miscellaneous	Plant-Prokaryote DOE project	Experimental - Histidine Degradation	Glutamate formyltransferase	0.005571	0.006786	0.007664	0	0.007222	0.011689	0.002166	0.002492	0.001314	0.004406	0.000233	0.003352294
Nitrogen Metabolism	NULL	Ammonia assimilation	Glutamate synthase [NADPH] putative GlxC chain (EC 1.4.1.13)	0.002785	0.004524	0	0.00498	0.003939	0.001461	0.002888	0.004984	0.005255	0.008811	0.015369	0.001538111
RNA Metabolism	RNA processing and modification	tRNA modification Bacteria	glutamyl-Q-tRNA synthetase	0.013926	0.015834	0.010219	0.01245	0.022979	0.019482	0.023829	0.03638	0.014013	0	0.00978	0.015105043
Protein Metabolism	Protein biosynthesis	tRNA aminoacylation, Glu and Gln	Glutamyl-tRNA(Gln) amidotransferase asparaginase subunit (EC 6.3.5.7)	0.008704	0.018096	0.040877	0.00996	0.014444	0.011202	0.015164	0.005482	0.002408	0.006608	0	0.009465301
Protein Metabolism	Protein biosynthesis	tRNA aminoacylation, Glu and Gln	Glutamyl-tRNA(Gln) amidotransferase transferase subunit (EC 6.3.5.7)	0.005222	0.004524	0.020438	0.01494	0.018384	0.005357	0.009387	0.004984	0.007225	0	0.000582	0.007532802
Clustering-based subsystems	NULL	Glutaredoxin 3 and Glutathione synthetase cluster	Glutaredoxin 3	0.002785	0.002262	0.002555	0.00996	0.003283	0.003896	0.00361	0.000997	0.001533	0	0.005007	0.004141069
Stress Response	Oxidative stress	Glutathione: Redox cycle	Glutathione peroxidase (EC 1.11.1.9)	0.005919	0.015834	0	0.01494	0.003283	0.007306	0.012998	0.016944	0.014232	0.002203	0.004075	0.007335608
Stress Response	Oxidative stress	Glutathione: Non-redox reactions	Glutathione S-transferase, unnamed subgroup (EC 2.5.1.18)	0.003133	0.002262	0	0.00249	0.007222	0.006332	0.009387	0.009967	0.013137	0.002203	0.00163	0.003707243
Potassium metabolism	NULL	Glutathione-regulated potassium-efflux system and associated functions	Glutathione-regulated potassium-efflux system ATP-binding protein	0.018801	0.009048	0.002555	0.00996	0.006566	0.009741	0.011553	0.016446	0.02277	0	0.103743	0.023032233
Amino Acids and Derivatives	Alanine, serine, and glycine	Glycine and Serine Utilization	Glycerate kinase (EC 2.7.1.31)	0.002785	0.01131	0.00511	0.00498	0.008535	0.00487	0.006499	0.004485	0.004379	0	0.002096	0.011516116
Carbohydrates	Sugar alcohols	Glycerol and Glycerol-3-phosphate Uptake and Utilization	Glycerol uptake facilitator protein	0	0.004524	0.007664	0.00747	0.003939	0.002435	0.00361	0.004984	0.010728	0.004406	0.000931	0.004693212
Fatty Acids, Lipids, and Isoprenoids	Phospholipids	Glycerolipid and Glycerophospholipid Metabolism in Bacteria	Glycerol-1-phosphate dehydrogenase [NAD(P)] (EC 1.1.1.261)	0.001741	0.002262	0	0.00747	0.001313	0.000974	0.004333	0.002492	0.001971	0.004406	0.000116	0.002011377
Carbohydrates	Sugar alcohols	Glycerol and Glycerol-3-phosphate Uptake and Utilization	Glycerol-3-phosphate regulon repressor, DeoR family	0.000696	0	0.002555	0.00747	0.007879	0.015585	0.007221	0.010964	0.011823	0.008811	0.045642	0.004298824
Carbohydrates	Sugar alcohols	Glycerol and Glycerol-3-phosphate Uptake and Utilization	Glycerol-3-phosphate transporter	0.001044	0	0.002555	0.00498	0.004596	0.005844	0.000722	0.001993	0.001095	0.002203	0.000699	0.002721274
Stress Response	Osmotic stress	Choline and Betaine Uptake and Betaine Biosynthesis	Glycine betaine ABC transport system, permease protein OpuAB	0.006963	0.006786	0.002555	0.00498	0.001313	0.002922	0.002166	0.003987	0.006349	0	0.002911	0.001222601
Carbohydrates	Polysaccharides	Glycogen metabolism	Glycogen [starch] synthase, eukaryotic (EC 2.4.1.11)	0.003133	0.009048	0.007664	0.00747	0.003939	0.006332	0.006499	0.00299	0.003722	0.002203	0	0.004180508
Cell Wall and Capsule	Capsular and extracellular polysacchrides	Exopolysaccharide Biosynthesis	Glycosyl transferase, group 1 family protein	0.007659	0.013572	0	0.00747	0.007879	0.008767	0.009387	0.009469	0.023208	0.006608	0.000233	0.01076678
Carbohydrates	Organic acids	Glycerate metabolism	Glyoxylate carboligase (EC 4.1.1.47)	0.004526	0	0.002555	0.00747	0.009848	0.008767	0.002166	0.005482	0.011385	0.002203	0.000582	0.00126204
Stress Response	Osmotic stress	Osmotic stress cluster	Heat shock (predicted periplasmic) protein YciM, precursor	0.012882	0	0.002555	0.00498	0.004596	0.006332	0.005055	0.00897	0.014232	0.002203	0.000466	0.005876374
Virulence, Disease and Defense	Resistance to antibiotics and toxic compounds	Cobalt-zinc-cadmium resistance	Heavy metal RND efflux outer membrane protein, CzcC family	0.011141	0.009048	0.007664	0.01494	0.005909	0.003409	0.00361	0.004984	0.010728	0.002203	0	0.01076678
Respiration	NULL	Biogenesis of cytochrome c oxidases	Heme A synthase, cytochrome oxidase biogenesis protein Cox15-CtaA	0.028897	0.01131	0.040877	0.034861	0.017727	0.01802	0.037548	0.032892	0.038754	0	0.001514	0.018812286
Iron acquisition and metabolism	NULL	Hemin transport system	Hemin ABC transporter, permease protein	0.004178	0.004524	0	0.00498	0.001313	0.001461	0.00361	0.011961	0.008977	0.002203	0.007801	0.003983314
Miscellaneous	Plant-Prokaryote DOE project	At5g37530	HesA/MoeB/ThiF family protein related to EC-YgdL	0.008356	0.002262	0.002555	0.00996	0.003283	0.002922	0	0.002492	0.005912	0.002203	0.028526	0.008361016
Respiration	Electron accepting reactions	Anaerobic respiratory reductases	heterodisulfide reductase, iron-sulfur binding subunit, putative	0.013578	0.013572	0.015329	0.00498	0.020353	0.009254	0.006499	0.001993	0.000876	0.004406	0	0.003943875
Respiration	Electron accepting reactions	Anaerobic respiratory reductases	heterodisulfide reductase, subunit A/methylviologen reducing hydrogenase, subunit delta	0.143093	0.339297	0.25037	0.316235	0.302672	0.327778	0.157414	0.05482	0.016421	0.006608	0	0.155625327
Stress Response	Osmotic stress	Choline and Betaine Uptake and Betaine Biosynthesis	High-affinity choline uptake protein BetT	0.014623	0.01131	0.007664	0	0.047929	0.067212	0.059933	0.0912	0.082105	0.030839	0.132385	0.03009177
Amino Acids and Derivatives	Histidine Metabolism	Histidine Biosynthesis	Histidinol-phosphatase [alternative form] (EC 3.1.3.15)	0.009052	0.006786	0.00511	0.01245	0.003939	0.006332	0.004333	0.009967	0.008977	0	0.000466	0.004456579
RNA Metabolism	RNA processing and modification	mcm5s2U biosynthesis in tRNA	histone acetyltransferase, ELP3 family	0.013926	0.027144	0.028103	0.01245	0.015101	0.006819	0.016608	0.010964	0.002627	0	0.000349	0.005915813
Membrane Transport	NULL	Transport of Nickel and Cobalt	HupE-UreJ family metal transporter	0.000348	0.002262	0.002555	0.00996	0.003283	0.007306	0.002166	0.005482	0.004817	0.002203	0	0.004259386
Nucleosides and Nucleotides	NULL	Hydantoin metabolism	Hydantoin racemase (EC 5.1.99.-)	0.004178	0	0.002555	0.00498	0.005909	0.007306	0.000722	0.001495	0.006131	0.011014	0.035279	0.001498673

Respiration	Electron donating reactions	Hydrogenases	hydrogenase/sulfur reductase, delta subunit	0.009748	0.006786	0.007664	0.00996	0.007879	0.006819	0.007943	0.003489	0.005693	0.006608	0	0.006073568
Miscellaneous	Plant-Prokaryote DOE project	At1g52510 AT4G12830 (COG0596)	Hydrolase, alpha/beta fold family protein, At1g52510/AT4G12830 homolog	0.016363	0.009048	0.025548	0.01494	0.010505	0.01802	0.009387	0.021928	0.024084	0	0.000116	0.006625711
Amino Acids and Derivatives	Branched-chain amino acids	Leucine Degradation and HMG-CoA Metabolism	Hydroxymethylglutaryl-CoA synthase (EC 2.3.3.10)	0.025416	0.063336	0.040877	0.044821	0.041363	0.023378	0.018052	0.014452	0.014013	0	0.040403	0.02184907
Cofactors, Vitamins, Prosthetic Groups, Pigments	NULL	Thiamin biosynthesis	Hydroxymethylpyrimidine ABC transporter, ATPase component	0.006267	0.004524	0.002555	0	0.007879	0.005357	0.007943	0.002492	0.007663	0.006608	0.000931	0.003549488
Miscellaneous	Plant-Prokaryote DOE project	At2g33980 At1g28960	Hypothetical nudix hydrolase YeaB	0.012534	0.009048	0.007664	0.00498	0.005909	0.00828	0.019496	0.019436	0.012918	0	0.001979	0.011555555
Clustering-based subsystems	NULL	CBSS-316279.3.peg.746	Hypothetical protein of L-Asparaginase type 2-like superfamily	0.006963	0.004524	0	0.00249	0.003283	0.006332	0.00361	0.008472	0.00832	0.002203	0.002329	0.001222601
Carbohydrates	NULL	VC0266	Hypothetical protein VC0266 (sugar utilization related?)	0.001393	0.01131	0	0.00747	0.005252	0.008767	0.007943	0.015449	0.011604	0.017622	0.011643	0.005758058
Miscellaneous	Plant-Prokaryote DOE project	At1g26220 At1g32070	Hypothetical protein, contains HAS barrel docking domain and DUF87 domain	0.001393	0.01131	0.017884	0.00498	0.009848	0.006332	0.005055	0.001993	0.009634	0	0.001514	0.00252408
Cofactors, Vitamins, Prosthetic Groups, Pigments	Tetrapyrroles	Heme and Siroheme Biosynthesis	Hypothetical radical SAM family enzyme in heat shock gene cluster, similarity with CPO of BS HemN-type	0.002437	0.013572	0.00511	0.01245	0.007879	0.008767	0.006499	0.00598	0.006349	0	0.000466	0.008913159
Cofactors, Vitamins, Prosthetic Groups, Pigments	Tetrapyrroles	Heme and Siroheme Biosynthesis	Hypothetical radical SAM family enzyme, NOT coproporphyrinogen III oxidase, oxygen-independent	0	0.004524	0.015329	0.00747	0.007222	0.014611	0.006499	0.011462	0.008101	0.046259	0.000466	0.01774744
Respiration	Electron donating reactions	Respiratory Complex I	Hypothetical transmembrane protein coupled to NADH-ubiquinone oxidoreductase chain 5 homolog	0.029245	0.047502	0.033212	0.062251	0.013788	0.021917	0.020218	0.032393	0.046636	0.008811	0	0.042672733
Nucleosides and Nucleotides	Purines	Purine conversions	Inosine-uridine preferring nucleoside hydrolase (EC 3.2.2.1)	0.004178	0.006786	0	0.00747	0.011818	0.012663	0.007221	0.010466	0.013794	0.006608	0.001397	0.004772089
Carbohydrates	Sugar alcohols	Inositol catabolism	Inosose dehydratase (EC 4.2.1.44)	0.001044	0	0.00511	0.01245	0.004596	0.002922	0.005777	0.004984	0.008101	0.006608	0.001164	0.002129693
Miscellaneous	Plant-Prokaryote DOE project	COG2302	Integral membrane protein YggT, involved in response to extracytoplasmic stress (osmotic shock)	0.010793	0.004524	0.007664	0.00498	0.011818	0.020943	0.014442	0.016446	0.01664	0	0.004075	0.014040197
Phages, Prophages, Transposable elements, Plasmids	NULL	Integrons	Integron integrase	0.036557	0.02262	0.025548	0.01494	0.015101	0.01315	0.015164	0.013456	0.010728	0	0.000116	0.009859689
Clustering-based subsystems	Pyruvate kinase associated cluster	CBSS-288000.5.peg.1793	InterPro IPR001440 COGs COG0457	0.007659	0.006786	0.012774	0.00747	0.004596	0.007306	0	0.000498	0.004817	0.004406	0.000233	0.004219947
Iron acquisition and metabolism	Siderophores	Siderophore Yersiniabactin Biosynthesis	iron aquisition yersiniabactin synthesis enzyme (Irp2)	0.002785	0.002262	0.002555	0.00249	0.00197	0.000487	0.002166	0.010466	0.004379	0.002203	0	0.00126204
Amino Acids and Derivatives	Alanine, serine, and glycine	Alanine biosynthesis	Iron binding protein IscA for iron-sulfur cluster assembly	0.011141	0.01131	0	0.00747	0.003939	0.009741	0.007221	0.006977	0.006568	0.002203	0.019328	0.00792719
Iron acquisition and metabolism	NULL	Iron(III) dicitrate transport system Fec	Iron(III) dicitrate transport system permease protein FecD (TC 3.A.1.14.1)	0.002089	0.002262	0	0.00996	0.003283	0.003409	0.004333	0.001993	0.006787	0.002203	0.003027	0.00698066
Iron acquisition and metabolism	NULL	Transport of Iron	Iron-regulated protein A precursor	0.000696	0	0.002555	0.00498	0.007879	0.004383	0.007943	0.013954	0.026493	0.017622	0.062758	0.01171331
Stress Response	Oxidative stress	Oxidative stress	Iron-responsive regulator Irr	0.003133	0.002262	0.002555	0	0.005252	0.004383	0.005055	0.004984	0.004817	0.002203	0.000349	0.002681835
RNA Metabolism	Transcription	Rrf2 family transcriptional regulators	Iron-sulfur cluster regulator IscR	0.022978	0	0.020438	0.02988	0.020353	0.020456	0.022385	0.020433	0.015326	0.013217	0.010479	0.019127796
Iron acquisition and metabolism	NULL	Transport of Iron	Iron-uptake factor PiuC	0.005919	0.002262	0.00511	0	0.004596	0.003896	0.002888	0.004485	0.008101	0.004406	0.020492	0.005245354
Protein Metabolism	Protein degradation	Omega peptidases (EC 3.4.19.-)	Isoaspartyl aminopeptidase (EC 3.4.19.5)	0.004526	0.002262	0.007664	0.00747	0.002626	0.002922	0.007943	0.010964	0.015545	0	0.001281	0.01234433
Amino Acids and Derivatives	Aromatic amino acids and derivatives	Chorismate: Intermediate for synthesis of Tryptophan, PAPA antibiotics, PABA, 3-hydroxyanthranilate and more.	Isochorismate synthase (EC 5.4.4.2)	0.001044	0.004524	0.007664	0.00747	0.000657	0.001461	0.004333	0.00299	0.003284	0	0.000116	0.008597649
Phages, Prophages, Transposable elements, Plasmids	Transposable elements	CBSS-203122.12.peg.188	ISPsy4, transposition helper protein	0.022282	0.002262	0.00511	0.00747	0.001313	0.003409	0.005777	0.001993	0.001752	0.002203	0	0.005403109
Amino Acids and Derivatives	Branched-chain amino acids	Ketoisovalerate oxidoreductase	Ketoisovalerate oxidoreductase subunit VorB (EC 1.2.7.7)	0.006963	0.004524	0.010219	0.00249	0.003283	0.007306	0.002166	0.001993	0.000657	0.002203	0	0.007532802
DNA Metabolism	DNA repair	Nonhomologous End-Joining in Bacteria	Ku domain protein	0.003133	0.004524	0.002555	0	0.000657	0.002922	0.002166	0.001993	0.004598	0.008811	0.000233	0.001419795
Cofactors, Vitamins, Prosthetic Groups, Pigments	NAD and NADP	NAD and NADP cofactor biosynthesis global	Kynurenine formamidase, bacterial (EC 3.5.1.9)	0.006615	0.002262	0.007664	0.00747	0.005252	0.003896	0.002888	0.003489	0.005036	0	0.000116	0.003233978
Miscellaneous	Plant-Prokaryote DOE project	COG3533	L-arabinose-specific 1-epimerase (mutarotase)	0.007311	0.020358	0.017884	0.01494	0.005909	0.006819	0.007943	0.007475	0.007225	0	0.000349	0.00887372
Miscellaneous	Plant-Prokaryote DOE project	COG3533	L-arabonate dehydratase (EC 4.2.1.25)	0.006615	0.013572	0.007664	0	0.007222	0.004383	0.008665	0.003489	0.017297	0.004406	0.000582	0.004456579
Miscellaneous	Plant-Prokaryote DOE project	At5g04520 AT1G06240	Large exoproteins involved in heme utilization or adhesion	0.002785	0.01131	0.00511	0	0.004596	0.00828	0.005777	0.011462	0.011604	0.002203	0.000233	0.005127038
Stress Response	Osmotic stress	Ectoine biosynthesis and regulation	L-ectoine synthase (EC 4.2.1.-)	0.004178	0.004524	0	0.01494	0.006566	0.003896	0.01733	0.0304	0.009853	0.002203	0.012575	0.007177853
Carbohydrates	Monosaccharides	L-fucose utilization temp	L-fuconate dehydratase (EC 4.2.1.68)	0.005571	0.006786	0.002555	0.00747	0.000657	0.000487	0	0.003987	0.00416	0.002203	0.000466	0.001459234
Cell Wall and Capsule	Gram-Negative cell wall components	KDO2-Lipid A biosynthesis	Lipid A biosynthesis lauroyl acyltransferase (EC 2.3.1.-)	0.01323	0.015834	0.017884	0.00747	0.007879	0.016072	0.009387	0.017941	0.021238	0	0.001164	0.017984072
Miscellaneous	Plant-Prokaryote DOE project	Competence or DNA damage-inducible protein CinA and related protein families	Lipoate-protein ligase A	0.008008	0.01131	0.010219	0.00498	0.009192	0.003896	0.007943	0.006977	0.005255	0.006608	0	0.007690557
Sulfur Metabolism	NULL	Sulfur oxidation	Lipocalin-related protein and Bos/Can/Equ allergen	0.003133	0.006786	0.00511	0.01494	0.007879	0.010715	0.005777	0.016944	0.007444	0	0.000116	0.01265984
Cell Wall and Capsule	Gram-Negative cell wall components	Lipoprotein sorting system	Lipoprotein releasing system transmembrane protein LolE	0.018104	0.006786	0.030658	0.0249	0.017727	0.022404	0.012275	0.021928	0.021019	0	0.016301	0.02058703
Respiration	Electron donating reactions	Respiratory dehydrogenases 1	L-lactate dehydrogenase (EC 1.1.2.3)	0.025764	0	0.028103	0.00996	0.014444	0.012663	0.018052	0.022925	0.035031	0.011014	0.030971	0.010648464
Carbohydrates	Central carbohydrate metabolism	Ethylmalonyl-CoA pathway of C2 assimilation	L-malyl-CoA/beta-methylmalyl-CoA lyase (EC 4.1.3.-)	0.006615	0.002262	0.002555	0	0.003939	0.007306	0.009387	0.019934	0.005912	0.004406	0.000582	0.002642397
Carbohydrates	Monosaccharides	L-rhamnose utilization	L-rhamnose isomerase (EC 5.3.1.14)	0.07555	0.040716	0.010219	0.034861	0.017727	0.011202	0.005777	0.012957	0.005036	0	0.001164	0.015065604
Protein Metabolism	Protein biosynthesis	Ribosome LSU bacterial	LSU ribosomal protein L32p	0.061276	0.009048	0.010219	0.00498	0.003283	0.006819	0.011553	0.013456	0.005255	0	0.000815	0.014552901
Cofactors, Vitamins, Prosthetic Groups, Pigments	Coenzyme M	coenzyme M biosynthesis	L-sulfolactate dehydrogenase (EC 1.1.1.272)	0.005571	0.009048	0.007664	0.00996	0.002626	0.005357	0.006499	0.002492	0.003503	0	0.000349	0.003707243
Cofactors, Vitamins, Prosthetic Groups, Pigments	Tetrapyrroles	Cobalamin synthesis	L-threonine 3-O-phosphate decarboxylase (EC 4.1.1.81)	0.005919	0	0.002555	0.01245	0.006566	0.006332	0.002166	0.005482	0.006349	0.002203	0.000349	0.002484642
Amino Acids and Derivatives	Lysine, threonine, methionine, and cysteine	Lysine biosynthesis AAA pathway 2	Lysine biosynthesis protein LysX	0.000696	0.006786	0	0.00249	0.008535	0.007306	0.005055	0.001993	0.007006	0.004406	0.000349	0.006073568
Clustering-based subsystems	Putative associate of RNA polymerase sigma-54 factor rpoN	CBSS-316057.3.peg.1308	macromolecule metabolism	0.011837	0.002262	0.025548	0	0.007222	0.008767	0.012998	0.00598	0.013794	0.002203	0.003377	0.009228669
Clustering-based subsystems	Clustering-based subsystems	CBSS-292415.3.peg.2341	Major facilitator superfamily (MFS) transporter	0.001393	0.006786	0.007664	0.00498	0.000657	0.000974	0.002166	0.003987	0.005255	0	0.000233	0.002602958
Miscellaneous	Plant-Prokaryote DOE project	COG3146 experimental	Maleylacetoacetate isomerase (EC 5.2.1.2)	0.013926	0.002262	0.00511	0.00996	0.002626	0.004383	0.005055	0.017941	0.017516	0	0.019328	0.003549488
Carbohydrates	Di- and oligosaccharides	Maltose and Maltodextrin Utilization	Maltose O-acetyltransferase (EC 2.3.1.79)	0.001393	0.004524	0.002555	0.00498	0.004596	0.001461	0.004333	0.00299	0.005693	0	0.000582	0.005284793
Carbohydrates	Polysaccharides	Alpha-Amylase locus in Streptocococcus	Maltose/maltodextrin ABC transporter, substrate binding periplasmic protein MalE	0.00383	0.01131	0.015329	0.01992	0.005252	0.003896	0.00361	0.005482	0.011166	0	0.001979	0.005166477
Membrane Transport	NULL	Transport of Manganese	Manganese ABC transporter, ATP-binding protein SitB	0.002785	0.009048	0.002555	0.00996	0.017727	0.014611	0.014442	0.018938	0.007006	0	0.002096	0.006941221
Virulence, Disease and Defense	Resistance to antibiotics and toxic compounds	Mercury resistance operon	Mercuric ion reductase (EC 1.16.1.1)	0.014971	0.01131	0.022993	0.02241	0.041363	0.045782	0.033938	0.040866	0.031747	0	0.001281	0.014513462
Phosphorus Metabolism	NULL	Alkylphosphonate utilization	Metal-dependent hydrolases of the beta-lactamase superfamily I; PhnP protein	0.0094	0.009048	0	0.00498	0.003939	0.00487	0.007943	0.010466	0.006568	0.013217	0.000233	0.011161168
Virulence, Disease and Defense	Resistance to antibiotics and toxic compounds	Beta-lactamase	Metal-dependent hydrolases of the beta-lactamase superfamily II	0.004178	0.009048	0.00511	0.00249	0.011161	0.00828	0.007221	0.003489	0	0.002203	0.000233	0.004850967

Amino Acids and Derivatives	Lysine, threonine, methionine, and cysteine	Methionine Salvage	Methionine aminotransferase, PLP-dependent	0.004526	0	0.007664	0.00498	0.003283	0.005844	0.00361	0.007974	0.003941	0.004406	0.050882	0.010727341
Carbohydrates	Central carbohydrate metabolism	Methylglyoxal Metabolism	Methylglyoxal synthase (EC 4.2.3.3)	0.002437	0.015834	0.015329	0	0.007222	0.007306	0.006499	0.009967	0.012261	0.011014	0.020842	0.00918923
Respiration	Sodium Ion-Coupled Energetics	Na+ translocating decarboxylases and related biotin-dependent enzymes	Methylmalonyl-CoA:Pyruvate transcarboxylase 12S subunit (EC 2.1.3.1)	0	0.004524	0.002555	0.00249	0.00197	0.001948	0.001444	0.000498	0.002627	0.002203	0.000349	0.001774744
Carbohydrates	Central carbohydrate metabolism	Ethylmalonyl-CoA pathway of C2 assimilation	Methylsuccinyl-CoA dehydrogenase, predicted by (Erb et al, 2007)	0.009748	0	0.010219	0.01992	0.008535	0.01315	0.015886	0.023921	0.017297	0.013217	0.002445	0.003549488
Cofactors, Vitamins, Prosthetic Groups, Pigments	Tetrapyrroles	Chlorophyll Biosynthesis	Mg-protoporphyrin IX monomethyl ester oxidative cyclase (anaerobic) (EC 1.14.13.81)	0.003133	0.01131	0.00511	0.00996	0.011818	0.018508	0.015164	0.013954	0.016202	0	0.00326	0.002997345
Miscellaneous	NULL	ZZ gjo need homes	Mitochondrial processing peptidase-like protein (EC 3.4.24.64)	0.004178	0.004524	0	0.00249	0.009848	0.013637	0.02094	0.018938	0.020143	0.002203	0.001397	0.004101631
Clustering-based subsystems	Probably GTP or GMP signaling related	CBSS-176299.4.peg.1292	Mll7752 protein	0.007659	0.002262	0.002555	0	0.002626	0.006819	0.005777	0.00598	0.005255	0.004406	0.001164	0.001025408
Membrane Transport	NULL	Transport of Manganese	Mn-dependent transcriptional regulator MntR	0.005222	0.006786	0	0.00996	0.011818	0.002922	0.007943	0.012459	0.013794	0.083706	0.002096	0.010648464
Cofactors, Vitamins, Prosthetic Groups, Pigments	Folate and pterines	Molybdenum cofactor biosynthesis	Molybdenum ABC transporter, periplasmic molybdenum-binding protein ModA (TC 3.A.1.8.1)	0.017756	0.036192	0.022993	0.02988	0.014444	0.010228	0.009387	0.012459	0.015545	0	0.041334	0.017905195
Clustering-based subsystems	Molybdopterin oxidoreductase	CBSS-269799.3.peg.2220	Molybdopterin oxidoreductase subunit, predicted	0.004526	0.013572	0.010219	0	0.007879	0.008767	0.012998	0.011961	0.017735	0.004406	0.000233	0.018457337
Clustering-based subsystems	Molybdopterin oxidoreductase	CBSS-269799.3.peg.2220	Molybdopterin oxidoreductase, iron-sulfur binding subunit (EC 1.2.7.-)	0.026808	0.03393	0.045986	0.02988	0.034141	0.032145	0.036826	0.036879	0.044884	0	0.003377	0.046458853
Clustering-based subsystems	Clustering-based subsystems	Sporulation-related Hypotheticals	MreB-like protein (Mbl protein)	0.002437	0.002262	0.00511	0	0.004596	0.003409	0.002166	0.003489	0.002189	0.006608	0.000699	0.003510049
Cell Wall and Capsule	NULL	Peptidoglycan Biosynthesis	Murein-DD-endopeptidase (EC 3.4.99.-)	0.008704	0.002262	0	0.01245	0.000657	0.003896	0.00361	0.001993	0.006568	0.002203	0.030506	0.004732651
Nucleosides and Nucleotides	Detoxification	Nudix proteins (nucleoside triphosphate hydrolases)	Mutator mutT protein (7,8-dihydro-8-oxoguanine-triphosphatase) (EC 3.6.1.-)	0.005919	0.004524	0	0.00249	0.013788	0.008767	0.008665	0.00598	0.00832	0.002203	0.00489	0.008163822
Virulence, Disease and Defense	NULL	Streptococcus pyogenes Virulome	N protein	0.0094	0.009048	0.007664	0	0.013788	0.014611	0.010831	0.021429	0.018829	0.011014	0.006287	0.015893818
Membrane Transport	Uni- Sym- and Antiporters	Multi-subunit cation antiporter	Na(+) H(+) antiporter subunit G	0.009052	0.009048	0.007664	0.01743	0.007222	0.015098	0.010109	0.008472	0.009196	0	0.007335	0.00666515
Cell Wall and Capsule	Gram-Positive cell wall components	Teichoic and lipoteichoic acids biosynthesis	N-acetylmannosaminyltransferase (EC 2.4.1.187)	0.005571	0.002262	0.00511	0.00249	0.004596	0.005844	0.00361	0.001495	0.005693	0	0.000233	0.003115662
Cell Wall and Capsule	NULL	Recycling of Peptidoglycan Amino Acids	N-acetylmuramoyl-L-alanine amidase (EC 3.5.1.28) AmpD	0.002785	0.002262	0.00511	0	0.001313	0.001461	0.000722	0.002492	0.003503	0.006608	0.005822	0.002129693
Cell Wall and Capsule	Capsular and extracellular polysacchrides	CMP-N-acetylneuraminate Biosynthesis	N-Acetylneuraminate cytidylyltransferase (EC 2.7.7.43)	0.010097	0.02262	0.015329	0.00996	0.031515	0.019482	0.025995	0.018439	0.006131	0	0.001397	0.022204019
Cofactors, Vitamins, Prosthetic Groups, Pigments	Riboflavin, FMN, FAD	Flavodoxin	NAD(P)H oxidoreductase YRKL (EC 1.6.99.-)	0.003482	0.004524	0	0.00498	0.004596	0.002922	0.004333	0.000997	0.003065	0.004406	0.000349	0.002642397
RNA Metabolism	RNA processing and modification	eukaryotic rRNA modification and related functions	NADH dehydrogenase subunit 5	0.002437	0.002262	0.002555	0.00249	0.002626	0.003896	0.000722	0.001993	0.003722	0	0.000699	0.001656428
Respiration	Electron donating reactions	Hydrogenases	NAD-reducing hydrogenase hoxS alpha subunit (EC 1.12.1.2)	0.000696	0.009048	0.007664	0.00747	0.005252	0.007306	0.009387	0.005482	0.000876	0	0.043197	0.007335608
Respiration	Electron donating reactions	Hydrogenases	NAD-reducing hydrogenase subunit HoxU (EC 1.12.1.2)	0.006615	0.024882	0	0.00996	0.002626	0.012176	0.005055	0.00299	0.002846	0.002203	0.063224	0.01360637
Amino Acids and Derivatives	Proline and 4-hydroxyproline	Proline, 4-hydroxyproline uptake and utilization	NAD-specific glutamate dehydrogenase (EC 1.4.1.2), large	0.004874	0.004524	0.002555	0	0.002626	0.001461	0.005777	0.011462	0.005912	0.004406	0.046341	0.004614334
Nucleosides and Nucleotides	NULL	Hydantoin metabolism	N-carbamoyl-L-amino acid hydrolase (EC 3.5.1.87)	0.002785	0.002262	0.007664	0	0.00197	0.004383	0.002888	0.009469	0.011604	0.013217	0.000931	0.002918468
Respiration	Electron donating reactions	Hydrogenases	Ni,Fe-hydrogenase I cytochrome b subunit	0.015667	0.006786	0.00511	0.00747	0.004596	0.007793	0.007943	0.003489	0.006349	0	0.052395	0.015578308
Respiration	Electron donating reactions	Hydrogenases	Ni,Fe-hydrogenase III large subunit	0.001044	0.004524	0.010219	0.00249	0.005252	0.000974	0	0.000997	0.002627	0.006608	0.000466	0.002681835
Respiration	Electron donating reactions	Hydrogenases	Ni/Fe-hydrogenase 2 B-type cytochrome subunit	0.029942	0.040716	0.017884	0.01245	0.03808	0.037989	0.025995	0.00897	0.007882	0	0.000349	0.022322335
Metabolism of Aromatic Compounds	NULL	Aromatic Amin Catabolism	Nitrilotriacetate monooxygenase component B (EC 1.14.13.-)	0.004874	0.004524	0.002555	0	0.000657	0.003409	0.00361	0.00598	0.013575	0.006608	0.007801	0.008952597
Nitrogen Metabolism	NULL	Nitrate and nitrite ammonification	Nitrite reductase probable [NAD(P)H] subunit (EC 1.7.1.4)	0.002089	0.002262	0	0.02988	0.01904	0.01802	0.006499	0.004984	0.006131	0.008811	0.005938	0.006467956
Nitrogen Metabolism	NULL	Nitrogen fixation	Nitrogenase (iron-iron) transcriptional regulator	0.003482	0.006786	0.007664	0.00498	0.008535	0.010228	0.009387	0.004485	0.00416	0.022028	0	0.007493363
Nitrogen Metabolism	NULL	Denitrification	Nitrous oxide reductase maturation transmembrane protein NosY	0.001044	0.004524	0.00511	0	0.002626	0.000487	0.002888	0.003987	0.004817	0.004406	0.002096	0.011318923
Nitrogen Metabolism	NULL	Denitrification	Nitrous-oxide reductase (EC 1.7.99.6)	0.012882	0.009048	0.010219	0	0.010505	0.011689	0.018774	0.018439	0.023865	0.019825	0.003726	0.039438755
Clustering-based subsystems	Protein export?	CBSS-393121.3.peg.2760	NLP/P60 family protein	0.004526	0.002262	0.00511	0.00249	0.00197	0.002922	0.004333	0.003489	0.007006	0.002203	0	0.006152446
Amino Acids and Derivatives	Branched-chain amino acids	Ketoisovalerate oxidoreductase	nonspecific lipid-transfer protein (acethyl CoA synthetase)	0.006963	0.004524	0.012774	0.00996	0.005252	0.004383	0.001444	0.000997	0.005036	0.002203	0	0.005481987
Carbohydrates	Di- and oligosaccharides	Maltose and Maltodextrin Utilization	Oligo-1,6-glucosidase (EC 3.2.1.10)	0.001393	0.004524	0.00511	0.00249	0.003939	0.001461	0.002888	0.004485	0.006568	0	0.001747	0.004772089
Fatty Acids, Lipids, and Isoprenoids	Fatty acids	Polyunsaturated Fatty Acids synthesis	omega-3 polyunsaturated fatty acid synthase subunit, PfaB	0.002785	0.036192	0.010219	0.00747	0.030858	0.035554	0.026717	0.014951	0.004817	0	0.000233	0.013724687
Cofactors, Vitamins, Prosthetic Groups, Pigments	Quinone cofactors	Menaquinone and Phylloquinone Biosynthesis	O-succinylbenzoate-CoA synthase (EC 4.2.1.-)	0.001044	0.01131	0.015329	0	0.004596	0.003896	0.00361	0.008472	0.007663	0.030839	0.007801	0.012147137
Cofactors, Vitamins, Prosthetic Groups, Pigments	Quinone cofactors	Menaquinone and Phylloquinone Biosynthesis	O-succinylbenzoic acid--CoA ligase (EC 6.2.1.26)	0.0094	0.015834	0.012774	0.00996	0.013131	0.010228	0.005777	0.013456	0.017297	0	0.003144	0.022440652
Cell Wall and Capsule	Gram-Negative cell wall components	Lipoprotein sorting system	Outer membrane lipoprotein carrier protein LolA	0.001393	0.004524	0.007664	0.00747	0.005252	0.002435	0.002166	0.00897	0.009196	0	0.005356	0.005600303
Cell Wall and Capsule	Gram-Negative cell wall components	Lipopolysaccharide assembly	Outer membrane lipoprotein SmpA, a component of the essential YaeT outer-membrane protein assembly complex	0.005919	0.004524	0.002555	0.00498	0.006566	0.001461	0.002888	0.003489	0.004598	0	0.006055	0.003194539
Cell Wall and Capsule	Gram-Negative cell wall components	Major Outer Membrane Proteins	Outer membrane protein A precursor	0.008704	0.020358	0.015329	0.00996	0.005252	0.011202	0.008665	0.005482	0.013137	0	0.000931	0.012620402
Cell Wall and Capsule	Gram-Negative cell wall components	Major Outer Membrane Proteins	Outer membrane protein H precursor	0.003482	0.004524	0.002555	0	0.003283	0.005357	0.002166	0.004984	0.009415	0.017622	0.0085	0.006783466
Cell Wall and Capsule	Gram-Negative cell wall components	Lipopolysaccharide assembly	Outer membrane protein NlpB, lipoprotein component of the protein assembly complex (forms a complex with YaeT, YfiO, and YfgL)	0.00383	0.004524	0.010219	0.00498	0.002626	0.005844	0.002888	0.004485	0.006349	0	0.013623	0.001735305
Iron acquisition and metabolism	NULL	Hemin transport system	Outer membrane receptor proteins, mostly Fe transport	0.009052	0.015834	0.010219	0.02739	0.01904	0.014124	0.037548	0.029902	0.0208	0	0.011643	0.015933257
Stress Response	Periplasmic Stress	Periplasmic Stress Response	Outer membrane stress sensor protease DegQ, serine protease	0.00383	0.002262	0.007664	0.00747	0.002626	0.00487	0.004333	0.00299	0.012042	0	0.002445	0.005048161
Cofactors, Vitamins, Prosthetic Groups, Pigments	Coenzyme A	Coenzyme A Biosynthesis	Pantothenate kinase (EC 2.7.1.33)	0.001393	0.002262	0	0.00249	0.000657	0.000974	0.002166	0.003987	0.003503	0.002203	0.000931	0.001419795
Carbohydrates	Monosaccharides	L-rhamnose utilization	Pedicted L-rhamnose permease, NCS1 Family	0.008008	0.006786	0.010219	0.00498	0.007879	0.009254	0.005777	0.00598	0.006349	0	0.002562	0.004850967
Clustering-based subsystems	NULL	CBSS-316273.3.peg.227	Penicillin-insensitive transglycosylase (EC 2.4.2.-) & transpeptidase PBP-1C	0	0.009048	0.007664	0.00249	0.003939	0.001948	0.002166	0.00299	0.002189	0.002203	0.000466	0.00761168
Protein Metabolism	Protein degradation	Aminopeptidases (EC 3.4.11.-)	Peptidase B (EC 3.4.11.23)	0.003133	0	0.010219	0.00747	0.022323	0.01315	0.019496	0.016944	0.021019	0.008811	0.005589	0.004338263
Protein Metabolism	Protein folding	Peptidyl-prolyl cis-trans isomerase	Peptidyl-prolyl cis-trans isomerase ppiD (EC 5.2.1.8)	0.003482	0.009048	0.015329	0.00996	0.011161	0.017046	0.012275	0.018938	0.023865	0	0.009431	0.017550246
Respiration	Electron donating reactions	Hydrogenases	Periplasmic [Fe] hydrogenase large subunit (EC 1.12.7.2)	0.016712	0.038454	0.025548	0.037351	0.020353	0.019482	0.016608	0.018439	0.005693	0	0.000815	0.028948046
Carbohydrates	Di- and oligosaccharides	Maltose and Maltodextrin Utilization	Periplasmic alpha-amylase (EC 3.2.1.1)	0.003133	0	0.00511	0.00498	0.003939	0.00487	0.005055	0.001495	0.002627	0.004406	0.000466	0.01837846
Cofactors, Vitamins, Prosthetic Groups, Pigments	NULL	Molybdopterin cytosine dinucleotide	Periplasmic aromatic aldehyde oxidoreductase, iron-sulfur subunit YagT	0.005222	0.004524	0.00511	0.00996	0.006566	0.00487	0.002888	0.001993	0.010509	0	0.000466	0.007177853

Cofactors, Vitamins, Prosthetic Groups, Pigments	NULL	Molybdopterin cytosine dinucleotide	Periplasmic aromatic aldehyde oxidoreductase, molybdenum binding subunit YagR	0.024023	0.020358	0.015329	0.01494	0.011818	0.016072	0.010109	0.007475	0.019486	0	0.000931	0.010056883
Virulence, Disease and Defense	Resistance to antibiotics and toxic compounds	Copper homeostasis: copper tolerance	Periplasmic divalent cation tolerance protein cutA	0.000696	0.009048	0.00511	0.01245	0.003939	0.00828	0.004333	0.007974	0.005474	0	0.000116	0.005324232
Protein Metabolism	Protein folding	Periplasmic disulfide interchange	Periplasmic thiol:disulfide oxidoreductase DsbB, required for DsbA reoxidation	0.006963	0.002262	0	0.00249	0.003939	0.003409	0.009387	0.00897	0.007882	0.002203	0.000815	0.003391733
Phages, Prophages, Transposable elements, Plasmids	Phages, Prophages	Phage capsid proteins	Phage major capsid protein	0	0.002262	0.007664	0.00249	0.066312	0.043347	0.074375	0.101666	0.006349	0.002203	0.002329	0.00378612
Metabolism of Aromatic Compounds	NULL	Phenylacetyl-CoA catabolic pathway (core)	Phenylacetate-CoA oxygenase, PaaI subunit	0	0.004524	0.00511	0.00747	0.003939	0.003896	0.009387	0.012957	0.012699	0.070489	0.023869	0.005245354
Metabolism of Aromatic Compounds	NULL	Phenylacetyl-CoA catabolic pathway (core)	Phenylacetate-CoA oxygenase, PaaJ subunit	0.002437	0.006786	0	0.00249	0.000657	0.003896	0.012998	0.004984	0.005255	0.006608	0.001164	0.003391733
Metabolism of Aromatic Compounds	NULL	Phenylacetyl-CoA catabolic pathway (core)	Phenylacetate-coenzyme A ligase (EC 6.2.1.30) PaaF	0.005919	0.002262	0.007664	0.00249	0	0.003409	0.002166	0.003489	0.004817	0.022028	0.04506	0.005639742
Metabolism of Aromatic Compounds	NULL	Phenylacetyl-CoA catabolic pathway (core)	Phenylacetic acid degradation protein PaaE, ketothiolase	0.024023	0	0.002555	0.00249	0.000657	0.000974	0.011553	0.016446	0.019048	0.006608	0.025266	0.007020098
Phosphorus Metabolism	NULL	Phosphate metabolism	Phosphate transport regulator (distant homolog of PhoU)	0.010445	0.013572	0.012774	0.00996	0.016414	0.010715	0.008665	0.012459	0.011823	0	0.024218	0.011121729
Fatty Acids, Lipids, and Isoprenoids	Phospholipids	Glycerolipid and Glycerophospholipid Metabolism in Bacteria	Phosphatidylglycerophosphatase A (EC 3.1.3.27)	0.012186	0.009048	0.010219	0.02241	0.014444	0.013637	0.011553	0.021928	0.016202	0	0.004774	0.014158513
Regulation and Cell signaling	NULL	The Chv regulatory system of Alphaproteobacteria	Phosphocarrier protein, nitrogen regulation associated	0.002785	0.004524	0.007664	0	0.004596	0.005844	0.004333	0.00598	0.01248	0.004406	0.013041	0.005048161
Carbohydrates	Central carbohydrate metabolism	Dihydroxyacetone kinases	Phosphoenolpyruvate-dihydroxyacetone phosphotransferase (EC 2.7.1.121), dihydroxyacetone binding subunit DhaK	0.004178	0	0.00511	0.00498	0.005909	0.001948	0.001444	0.00299	0.004817	0.004406	0.000931	0.003233978
Cell Wall and Capsule	Capsular and extracellular polysacchrides	Capsular heptose biosynthesis	Phosphoheptose isomerase 1 (EC 5.3.1.-)	0.013578	0.01131	0.007664	0.00747	0.015757	0.023378	0.036826	0.016944	0.009853	0	0.015136	0.016998103
Phosphorus Metabolism	NULL	Alkylphosphonate utilization	Phosphonates transport ATP-binding protein PhnL	0.001044	0.004524	0.007664	0	0.00197	0.006332	0.007943	0.010466	0.005036	0.002203	0.025965	0.001380356
Stress Response	Oxidative stress	Oxidative stress	Phytochrome, two-component sensor histidine kinase (EC 2.7.3.-)	0.011489	0.02262	0.035767	0.01992	0.038737	0.029222	0.025995	0.021429	0.054518	0	0.001048	0.032852483
Clustering-based subsystems	Methylamine utilization	CBSS-265072.7.peg.546	PlcB, ORFX, ORFP, ORFB, ORFA, ldh gene	0.014274	0.004524	0.002555	0.01992	0.012475	0.015098	0.01372	0.014452	0.008539	0	0.000931	0.007335608
Miscellaneous	NULL	ZZ gjo need homes	Pole remodelling regulatory diguanylate cyclase	0.010097	0.015834	0.020438	0.01494	0.028888	0.020456	0.023829	0.022925	0.015764	0	0.000233	0.036007583
Cell Wall and Capsule	Capsular and extracellular polysacchrides	Alginate metabolism	poly(beta-D-mannuronate) lyase (EC 4.2.2.3)	0.002437	0.004524	0	0.00249	0.000657	0.006332	0.00361	0.004984	0.000438	0.006608	0.001164	0.001853621
Cell Wall and Capsule	Gram-Negative cell wall components	Lipid A-Ara4N pathway (Polymyxin resistance)	Polymyxin resistance protein ArnA FT, UDP-4-amino-4-deoxy-L-arabinose formylase (EC 2.1.2.-)	0.013926	0.015834	0.007664	0.0249	0.001313	0.000974	0.002888	0.001495	0.004598	0	0.000349	0.008321577
Carbohydrates	Central carbohydrate metabolism	Entner-Doudoroff Pathway	Polyphosphate glucokinase (EC 2.7.1.63)	0.007311	0.009048	0.007664	0.01494	0.002626	0.003409	0.005055	0.007974	0.010947	0	0.000931	0.013172544
Carbohydrates	Monosaccharides	L-fucose utilization	Possible fucose ABC transporter, ATP-binding component	0.002785	0.02262	0.007664	0.00996	0.00197	0.001948	0.002888	0.001993	0.003503	0	0.000233	0.004377702
Nucleosides and Nucleotides	Purines	Purine conversions	Possible hypoxanthine oxidase XdhD (EC 1.-.-.-)	0.037949	0.002262	0.002555	0.00498	0.005252	0.012176	0.006499	0.007974	0.008101	0.004406	0	0.00378612
Cell Wall and Capsule	Cell wall of Mycobacteria	mycolic acid synthesis	POSSIBLE LINOLEOYL-CoA DESATURASE (DELTA(6)-DESATURASE)	0.00383	0.002262	0.002555	0	0.001313	0.000974	0.002166	0.000498	0.005912	0.004406	0.023287	0.00126204
Potassium metabolism	NULL	Potassium homeostasis	Potassium channel protein	0.022978	0.020358	0.028103	0.032371	0.055807	0.048217	0.03105	0.03339	0.021238	0	0.011527	0.037190746
Carbohydrates	Di- and oligosaccharides	Beta-Glucoside Metabolism	Predicted beta-glucoside transporter, GPH family	0.009052	0.009048	0.007664	0.00747	0.002626	0.002922	0.005055	0.000997	0.010947	0	0.016184	0.003628365
Carbohydrates	Di- and oligosaccharides	Beta-Glucoside Metabolism	Predicted beta-glucoside-regulated ABC transport system, sugar binding component, COG1653	0.002437	0.004524	0.007664	0.00249	0.001313	0.000487	0.000722	0.001993	0.000438	0	0.000116	0.00252408
Carbohydrates	Monosaccharides	D-Galacturonate and D-Glucuronate Utilization	Predicted D-mannonate epimerase	0.010097	0.009048	0.028103	0.02241	0.009192	0.013637	0.006499	0.007974	0.005474	0.022028	0	0.009268107
Carbohydrates	Central carbohydrate metabolism	Glycolysis and Gluconeogenesis	Predicted Fructose-bisphosphate aldolase (EC 4.1.2.13) in Geobacter	0.014971	0.015834	0.007664	0.00747	0.034141	0.025326	0.014442	0.002492	0.002189	0.033042	0	0.020310959
Carbohydrates	Monosaccharides	L-rhamnose utilization	Predicted lactaldehyde dehydrogenase (EC 1.2.1.22)	0.002437	0.013572	0.007664	0.00249	0.00197	0.003896	0.00361	0.009967	0.019048	0	0.003842	0.002957907
Carbohydrates	Organic acids	Lactate utilization	Predicted L-lactate dehydrogenase, hypothetical protein subunit YkgG	0.008356	0.006786	0.015329	0.00249	0.013788	0.01315	0.008665	0.00598	0.007663	0.004406	0	0.007217292
Carbohydrates	Monosaccharides	L-rhamnose utilization	Predicted L-rhamnose ABC transporter, ATP-binding component	0.00383	0.020358	0.010219	0.01743	0.005909	0.003409	0.005055	0.003489	0.015764	0.002203	0	0.006744027
Carbohydrates	Monosaccharides	L-rhamnose utilization	Predicted L-rhamnose ABC transporter, substrate-binding component	0.009748	0.004524	0.002555	0.01245	0.003939	0.000974	0.001444	0.003489	0.00832	0.011014	0	0.002800152
Carbohydrates	Monosaccharides	L-rhamnose utilization	Predicted L-rhamnose isomerase RhaI (EC 5.3.1.14)	0.008704	0.018096	0.007664	0.00498	0.008535	0.005357	0.004333	0.010466	0.01248	0	0.00489	0.004417141
Membrane Transport	ABC transporters	ATP-dependent efflux pump transporter Ybh	Predicted membrane fusion protein (MFP) component of efflux pump, membrane anchor protein YbhG	0.016363	0.029406	0.012774	0.01992	0.004596	0.003896	0.004333	0.003987	0.008539	0	0.000582	0.01202882
Carbohydrates	Monosaccharides	Deoxyribose and Deoxynucleoside Catabolism	Predicted nucleoside ABC transporter, permease 1 component	0.002785	0.002262	0.002555	0	0.002626	0.00487	0.004333	0.00897	0.006568	0.004406	0.001048	0.002326887
Cell Wall and Capsule	Capsular and extracellular polysacchrides	Sialic Acid Metabolism	Predicted sialic acid transporter	0.011489	0.018096	0.012774	0.00498	0.007222	0.010715	0.002888	0.00598	0.007006	0.002203	0	0.011358361
Fatty Acids, Lipids, and Isoprenoids	Fatty acids	Fatty acid degradation regulons	Predicted transcriptional regulator for fatty acid degradation FadQ, TetR family	0.003482	0	0.002555	0.00498	0.00197	0.002435	0.002888	0.003489	0.010947	0.002203	0.000815	0.003431172
Amino Acids and Derivatives	Branched-chain amino acids	Branched chain amino acid degradation regulons	Predicted transcriptional regulator LiuR of leucine degradation pathway, MerR family	0.008008	0.004524	0.010219	0	0.002626	0.001461	0.002888	0.010466	0.011823	0.002203	0.001048	0.002642397
Cofactors, Vitamins, Prosthetic Groups, Pigments	Pyridoxine	Pyridoxin (Vitamin B6) Biosynthesis	Predicted transcriptional regulator of pyridoxine metabolism	0.001044	0.002262	0	0.00498	0.002626	0.000974	0.000722	0.001495	0.001095	0.004406	0.002562	0.003352294
Membrane Transport	Protein translocation across cytoplasmic membrane	HtrA and Sec secretion	Preprotein translocase subunit SecE (TC 3.A.5.1.1)	0.019497	0.004524	0.012774	0.00249	0.008535	0.009741	0.005055	0.014452	0.00832	0	0.011061	0.014907849
Miscellaneous	Plant-Prokaryote DOE project	At2g23840	Probable 3-methyladenine DNA glycosylase/8-oxoguanine DNA glycosylase	0.000696	0.002262	0.017884	0.00996	0.005252	0.005357	0.001444	0.006479	0.005474	0	0.012691	0.002090254
Protein Metabolism	Protein biosynthesis	Programmed frameshift	programmed frameshift-containing	0.012882	0	0.007664	0.00996	0.008535	0.00828	0.010109	0.008472	0.008101	0.017622	0.026314	0.013330299
Amino Acids and Derivatives	Proline and 4-hydroxyproline	Proline, 4-hydroxyproline uptake and utilization	Proline/sodium symporter PutP (TC 2.A.21.2.1)	0.004526	0.006786	0.020438	0.01245	0.005909	0.00828	0.006499	0.008472	0.008758	0	0.054142	0.018575654
DNA Metabolism	DNA repair	DNA repair, bacterial RecBCD pathway	Protease III precursor (EC 3.4.24.55)	0.004178	0.004524	0.012774	0	0.00197	0.004383	0.004333	0.013456	0.032623	0.011014	0.045409	0.006113007
Clustering-based subsystems	Proteasome related clusters	Proteasome subunit alpha archaeal cluster	Proteasome subunit alpha (EC 3.4.25.1), archaeal	0.001741	0	0.00511	0.00747	0.003283	0.003409	0.012998	0.00299	0.002189	0.002203	0.000233	0.003746682
Clustering-based subsystems	NULL	USS-DB-7	Protein ImpG/VasA	0.012186	0.004524	0.040877	0.01494	0.015101	0.010715	0.007943	0.006977	0.013356	0	0.014554	0.00950474
DNA Metabolism	DNA uptake, competence	DNA processing cluster	Protein involved in catabolism of external DNA	0.002785	0	0.010219	0.00747	0.002626	0.001948	0.001444	0.00299	0.009196	0.006608	0.025615	0.005442548
Miscellaneous	NULL	ZZ gjo need homes	Protein of unknown function DUF81	0.028201	0.006786	0	0.00996	0.005252	0.006819	0.007943	0.010466	0.019924	0.011014	0.04832	0.022361774
Miscellaneous	NULL	ZZ gjo need homes	Protein of unknown function YceH	0.0094	0.006786	0.007664	0.00249	0.001313	0.006819	0.000722	0.003489	0.001971	0	0.033533	0.005560864
Sulfur Metabolism	NULL	Sulfate reduction-associated complexes	Protein similar to glutamate synthase [NADPH] small chain, clustered with sulfite reductase	0.053268	0.040716	0.038322	0.0249	0.018384	0.020456	0.030328	0.010964	0.006568	0	0.000116	0.022322335
RNA Metabolism	RNA processing and modification	Polyadenylation specificity factor	Protein similar to polyadenylation specificity factor, MJ1236 type	0.002785	0.013572	0.002555	0.00996	0.013788	0.011689	0.026717	0.007475	0.005912	0	0.000233	0.008794842
Clustering-based subsystems	NULL	Cell wall related cluster	Protein-tyrosine kinase (EC 2.7.1.112)	0.01323	0.004524	0.010219	0.01992	0.009192	0.007306	0.000722	0.005482	0.007882	0.008811	0	0.006744027
Metabolism of Aromatic Compounds	Metabolism of central aromatic intermediates	Protocatechuate branch of beta-ketoadipate pathway	Protocatechuate 3,4-dioxygenase alpha chain (EC 1.13.11.3)	0.002785	0	0.002555	0.00498	0.00197	0.00487	0.000722	0.005482	0.005474	0.002203	0.000815	0.001498673
Metabolism of Aromatic Compounds	Metabolism of central aromatic intermediates	Central meta-cleavage pathway of aromatic compound degradation	Protocatechuate 4,5-dioxygenase alpha chain (EC 1.13.11.8)	0.010445	0	0.007664	0.00498	0.002626	0.001948	0.001444	0.00299	0.003503	0.006608	0.027944	0.001656428

Cofactors, Vitamins, Prosthetic Groups, Pigments	Tetrapyrroles	Chlorophyll Biosynthesis	Protoporphyrin IX Mg-chelatase subunit H (EC 6.6.1.1)	0.002437	0.027144	0	0.00747	0.009848	0.035067	0.02094	0.035384	0.046636	0.004406	0.002212	0.003352294
Cofactors, Vitamins, Prosthetic Groups, Pigments	Tetrapyrroles	Heme and Siroheme Biosynthesis	Protoporphyrinogen IX oxidase, novel form, HemJ (EC 1.3.-.-)	0.007659	0.004524	0.002555	0	0.010505	0.007306	0.018052	0.015948	0.023865	0.088112	0.000699	0.016327645
Carbohydrates	Organic acids	Methylcitrate cycle	PrpF protein involved in 2-methylcitrate cycle	0.007311	0	0.00511	0.00249	0.007222	0.006819	0.005055	0.008472	0.015545	0.022028	0.102811	0.015972696
Cell Wall and Capsule	Capsular and extracellular polysacchrides	Pseudaminic Acid Biosynthesis	Pseudaminic acid synthase (EC 4.1.3.-)	0.002437	0.006786	0.012774	0	0.010505	0.005844	0.004333	0.004984	0.002408	0.006608	0.015253	0.004456579
Carbohydrates	Di- and oligosaccharides	Maltose and Maltodextrin Utilization	Pullulanase (EC 3.2.1.41)	0	0.009048	0.002555	0.00249	0.002626	0.003896	0.005055	0.003987	0.00416	0.024231	0.001048	0.006467956
Amino Acids and Derivatives	Aromatic amino acids and derivatives	Aromatic amino acid degradation	Putative 5-carboxymethyl-2-hydroxymuconate semialdehyde dehydrogenase oxidoreductase protein (EC 1.2.1.60)	0.006615	0	0.002555	0.00249	0.00197	0.002435	0.005055	0.001993	0.006131	0.017622	0.14333	0.009662495
Sulfur Metabolism	Organic sulfur assimilation	Alkanesulfonate assimilation	Putative arylsulfatase regulatory protein	0.001741	0.02262	0.00511	0.00498	0.001313	0.002922	0.002888	0.005482	0.003941	0.004406	0	0.006113007
Respiration	Electron accepting reactions	Terminal cytochrome oxidases	putative Cytochrome bd2, subunit II	0.000348	0.006786	0.00511	0.00498	0.004596	0.005844	0.002888	0.005482	0.008101	0	0.000349	0.002405764
Clustering-based subsystems	Clustering-based subsystems	Putative diaminopropionate ammonia-lyase cluster	Putative deacetylase YgeY	0.290364	0.015834	0.002555	0.01245	0.00197	0.005357	0.005055	0.002492	0	0.004406	0.000349	0.003746682
Carbohydrates	Monosaccharides	Deoxyribose and Deoxynucleoside Catabolism	Putative deoxyribose-specific ABC transporter, permease protein	0.002089	0.004524	0	0.00996	0.011161	0.011202	0.007221	0.009967	0.005912	0.006608	0.02841	0.006546833
Respiration	NULL	Soluble cytochromes and functionally related electron carriers	Putative diheme cytochrome c-553	0.005571	0.009048	0	0.01494	0.011818	0.007793	0.005777	0.018439	0.016421	0.013217	0.000815	0.005048161
DNA Metabolism	NULL	Restriction-Modification System	Putative DNA-binding protein in cluster with Type I restriction-modification system	0	0.006786	0.002555	0.00249	0.007879	0.006332	0.007221	0.005482	0.005693	0.013217	0.030506	0.011003413
Respiration	NULL	Formate hydrogenase	Putative formate dehydrogenase-specific chaperone	0	0.004524	0.007664	0.00498	0.002626	0.00487	0.002166	0.00598	0.006568	0.002203	0.000349	0.002721274
Carbohydrates	Di- and oligosaccharides	Trehalose Biosynthesis	Putative glucanase glgE (EC 3.2.1.-)	0.000348	0.004524	0.015329	0.00249	0	0.000487	0.000722	0.000498	0.002846	0.002203	0.000466	0.000749336
Sulfur Metabolism	Organic sulfur assimilation	Utilization of glutathione as a sulphur source	Putative glutathione transporter, ATP-binding component	0.002437	0	0.007664	0.00747	0.009848	0.003896	0.011553	0.011462	0.011385	0.008811	0.000815	0.004732651
Sulfur Metabolism	Organic sulfur assimilation	Utilization of glutathione as a sulphur source	Putative glutathione transporter, permease component	0	0.006786	0.00511	0.00249	0.005909	0.005357	0.007943	0.005482	0.01029	0.008811	0.000116	0.007532802
Carbohydrates	Polysaccharides	Glycogen metabolism	Putative glycogen debranching enzyme, archaeal type, TIGR01561	0.026112	0.018096	0.017884	0.059761	0.01904	0.0263	0.020218	0.007475	0.017516	0.006608	0	0.017037542
Clustering-based subsystems	NULL	CBSS-296591.1.peg.2330	Putative glycosyltransferase	0.005222	0.002262	0.002555	0.00249	0.003939	0.001461	0.002166	0.003489	0.002846	0	0.000233	0.002879029
Miscellaneous	NULL	ZZ gjo need homes	Putative GTP-binding protien TM0445	0.001741	0.009048	0.00511	0.00249	0.001313	0.006819	0.005055	0.003489	0.002627	0	0.001164	0.007769435
Clustering-based subsystems	Hypothetical protein possible functionally linked with Alanyl-tRNA synthetase	CBSS-257314.1.peg.488	Putative Holliday junction resolvase	0.006615	0.004524	0.012774	0.00249	0.003283	0.006332	0.007221	0.009469	0.010072	0	0.006404	0.006901782
Miscellaneous	Plant-Prokaryote DOE project	Iron-sulfur cluster assembly	Putative iron-sulfur cluster assembly scaffold protein for SUF system, SufE2	0.010097	0.004524	0.010219	0.00996	0.010505	0.002435	0.010831	0.016446	0.015326	0	0.000233	0.006113007
Miscellaneous	NULL	YbbK	Putative membrane-bound ClpP-class protease associated with aq 911	0.028201	0.03393	0.033212	0.057271	0.053181	0.043347	0.042603	0.028905	0.021457	0.006608	0	0.024491467
Cell Wall and Capsule	Gram-Positive cell wall components	Teichuronic acid biosynthesis	Putative N-acetylgalactosaminyl-diphosphoundecaprenol glucuronosyltransferase	0.002437	0.002262	0	0.00747	0.004596	0.002435	0.002888	0.00299	0.004817	0.01542	0.000466	0.008913159
Respiration	NULL	Quinone oxidoreductase family	Putative NADP-dependent oxidoreductase PA1648	0.004526	0.002262	0.007664	0.00498	0.005909	0.003896	0.005055	0.004485	0.00416	0	0.000931	0.003036784
Miscellaneous	NULL	Broadly distributed proteins not in subsystems	Putative oxidoreductase YncB	0.000348	0.002262	0.00511	0	0.001313	0.000974	0.007943	0.007475	0.007225	0.01542	0.033882	0.003746682
Miscellaneous	NULL	ZZ gjo need homes	Putative permease PerM (= YfgO)	0.008008	0.002262	0.010219	0.00747	0.010505	0.019969	0.010831	0.013954	0.023646	0	0.030156	0.016288206
Stress Response	Osmotic stress	Ectoine biosynthesis and regulation	Putative regulatory protein associated with the ectoine operon	0.001393	0.002262	0.007664	0.00249	0.003939	0.006332	0.010109	0.006479	0.005693	0.008811	0	0.004022753
Regulation and Cell signaling	NULL	Orphan regulatory proteins	Putative sensor-like histidine kinase YfhK	0.002437	0.002262	0.002555	0	0.003939	0.001461	0.002888	0.001495	0.002846	0.002203	0.000116	0.002602958
Carbohydrates	CO2 fixation	CO2 uptake, carboxysome	putative sodium-dependent bicarbonate transporter	0.002785	0.009048	0.002555	0	0.003939	0.012663	0.007943	0.029403	0.010728	0.004406	0.00163	0.0031551
Miscellaneous	NULL	YbbK	Putative stomatin/prohibitin-family membrane protease subunit PA4582	0.009052	0.006786	0.007664	0.00249	0.010505	0.006819	0.00361	0.00299	0.005693	0.004406	0	0.00252408
Sulfur Metabolism	Inorganic sulfur assimilation	Inorganic Sulfur Assimilation	Putative sulfate permease	0.004526	0.004524	0	0.00249	0.005252	0.003409	0.005055	0.001495	0.004817	0.024231	0.073004	0.01202882
Cell Wall and Capsule	Gram-Negative cell wall components	LOS core oligosaccharide biosynthesis	Putative two-domain glycosyltransferase	0.006615	0.006786	0.002555	0	0.003939	0.002922	0.001444	0.001495	0.002408	0.006608	0.000233	0.003391733
Respiration	NULL	Quinone oxidoreductase family	Putative Zn-dependent oxidoreductase PA5234	0.004874	0.006786	0.015329	0.01494	0.007879	0.004383	0.009387	0.016446	0.01248	0	0.003493	0.003904437
Amino Acids and Derivatives	Arginine; urea cycle, polyamines	Polyamine Metabolism	Putrescine transport system permease protein PotI (TC 3.A.1.11.2)	0.020541	0.009048	0.017884	0	0.003283	0.008767	0.004333	0.007974	0.012699	0.002203	0.046108	0.011752749
Miscellaneous	Plant-Prokaryote DOE project	Experimental-COG2515	pyridoxal phosphate-dependent deaminase, putative	0.040386	0.015834	0.040877	0.01743	0.020353	0.02143	0.022385	0.012957	0.013575	0	0.000233	0.017944634
Cofactors, Vitamins, Prosthetic Groups, Pigments	Pyridoxine	Pyridoxin (Vitamin B6) Biosynthesis	Pyridoxine biosynthesis glutamine amidotransferase, glutaminase subunit (EC 2.4.2.-)	0.014274	0.024882	0.020438	0.00249	0.008535	0.005844	0.005777	0.005482	0.006349	0	0.000815	0.005363671
Carbohydrates	Central carbohydrate metabolism	Glycolysis and Gluconeogenesis	Pyrophosphate--fructose 6-phosphate 1-phosphotransferase, beta subunit (EC 2.7.1.90)	0.014971	0.013572	0.015329	0.01245	0.024949	0.018995	0.018774	0.011961	0.003941	0.011014	0	0.010017444
Carbohydrates	Central carbohydrate metabolism	Pyruvate:ferredoxin oxidoreductase	Pyruvate:ferredoxin oxidoreductase, gamma subunit (EC 1.2.7.1)	0.019149	0.018096	0.022993	0.034861	0.015757	0.017046	0.012275	0.004984	0.00416	0	0.000582	0.015657186
DNA Metabolism	DNA repair	DNA repair, bacterial RecBCD pathway	RecD-like DNA helicase Atu2026	0	0.002262	0.033212	0.00498	0.043333	0.01802	0.019496	0.024918	0.015107	0.050664	0.001979	0.025043609
Stress Response	Oxidative stress	Oxidative stress	Redox-sensitive transcriptional regulator (AT-rich DNA-binding protein)	0.004526	0.002262	0.00511	0.01245	0.005909	0.003896	0.006499	0.003489	0.005693	0	0.000116	0.005442548
Virulence, Disease and Defense	Resistance to antibiotics and toxic compounds	BlaR1 Family Regulatory Sensor-transducer Disambiguation	Regulatory sensor-transducer, BlaR1/MecR1 family	0.004178	0.006786	0	0.00747	0.001313	0.003409	0.001444	0.004984	0.003503	0.004406	0.000582	0.004811528
DNA Metabolism	DNA replication	DNA replication, archaeal	Replication factor C small subunit	0.002785	0.002262	0.00511	0.00498	0.007879	0.00487	0.01372	0.004984	0.002408	0	0.000582	0.003036784
Virulence, Disease and Defense	Resistance to antibiotics and toxic compounds	The mdtABCD multidrug resistance cluster	Response regulator BaeR	0.008704	0.002262	0.00511	0.01245	0.008535	0.004383	0.00361	0.004984	0.003503	0	0.017232	0.004456579
Miscellaneous	Plant-Prokaryote DOE project	Single-Rhodanese-domain proteins	Rhodanese domain protein UPF0176	0.003482	0.018096	0	0.00249	0.006566	0.007306	0.014442	0.027908	0.031528	0.01542	0.069627	0.012068259
RNA Metabolism	RNA processing and modification	tRNA modification Archaea	Rhodanese-like domain protein	0.008008	0.006786	0.010219	0.00498	0.014444	0.013637	0.006499	0.012459	0.005912	0.044056	0	0.016722032
RNA Metabolism	RNA processing and modification	RNA processing and degradation, bacterial	Ribonuclease E inhibitor RraA	0.005919	0.009048	0	0.00249	0.005909	0.006332	0.010109	0.007974	0.011385	0.004406	0.008616	0.009780811
RNA Metabolism	RNA processing and modification	tRNA processing	Ribonuclease T (EC 3.1.13.-)	0.010097	0	0.007664	0.00498	0.009192	0.005357	0.022385	0.012459	0.0208	0.004406	0.03982	0.015381114
Motility and Chemotaxis	NULL	Bacterial Chemotaxis	Ribose ABC transporter, periplasmic ribose-binding protein RbsB (TC 3.A.1.2.1)	0.002089	0.004524	0	0.00249	0.006566	0.009254	0.002166	0.00897	0.017516	0.011014	0.011294	0.007453925
RNA Metabolism	RNA processing and modification	RNA methylation	Ribosomal RNA large subunit methyltransferase E (EC 2.1.1.-)	0.021238	0	0.015329	0.01245	0.010505	0.014124	0.009387	0.010466	0.01664	0.002203	0.013506	0.009110352
RNA Metabolism	RNA processing and modification	RNA pseudouridine syntheses	Ribosomal small subunit pseudouridine synthase A (EC 4.2.1.70)	0.000348	0	0.00511	0.00498	0.006566	0.00828	0.005777	0.003489	0.005912	0.017622	0.059614	0.016406522
Protein Metabolism	Protein biosynthesis	Ribosome activity modulation	Ribosomal subunit interface protein	0.012186	0.020358	0.010219	0	0.009848	0.011689	0.008665	0.014951	0.010072	0.002203	0.004657	0.011003413
Protein Metabolism	Protein biosynthesis	Ribosome biogenesis bacterial	Ribosomal-protein-S18p-alanine acetyltransferase (EC 2.3.1.-)	0.012186	0.006786	0.017884	0.00996	0.009192	0.007793	0.010109	0.007974	0.009196	0	0.000466	0.007729996
Clustering-based subsystems	NULL	Lipopolysaccharide assembly cluster	Ribosome hibernation protein YhbH	0.001741	0	0.007664	0.00498	0.005252	0.007793	0.007221	0.00598	0.007444	0.011014	0.021191	0.011871065
Clustering-based subsystems	NULL	Cell division-ribosomal stress proteins cluster	Ribosome-associated heat shock protein implicated in the recycling of the 50S subunit (S4 paralog)	0.028549	0	0.007664	0.00249	0.007879	0.005357	0.007221	0.009967	0.011823	0.004406	0.015719	0.010924535

Carbohydrates	CO2 fixation	CO2 uptake, carboxysome	Ribulose bisphosphate carboxylase (EC 4.1.1.39)	0.004178	0.002262	0.010219	0	0.007879	0.006819	0.004333	0.000498	0.001095	0.004406	0.002329	0.006941221
Carbohydrates	CO2 fixation	Calvin-Benson cycle	Ribulose bisphosphate carboxylase small chain (EC 4.1.1.39)	0.008356	0	0.007664	0.01494	0.002626	0.007306	0.006499	0.003489	0.005036	0.004406	0.002096	0.007453925
RNA Metabolism	RNA processing and modification	RNA 3'-terminal phosphate cyclase	RNA 3'-terminal phosphate cyclase (EC 6.5.1.4)	0.001393	0.015834	0.007664	0.00498	0.007879	0.007306	0.01372	0.004485	0.007663	0	0.001397	0.007335608
RNA Metabolism	RNA processing and modification	eukaryotic rRNA modification and related functions	RNA helicase, putative	0	0.004524	0.007664	0.00249	0.00197	0.001461	0.006499	0.00299	0.001533	0.004406	0.000931	0.003983314
Virulence, Disease and Defense	Resistance to antibiotics and toxic compounds	Multidrug efflux pump in Campylobacter jejuni (CmeABC operon)	RND efflux system, membrane fusion protein CmeA	0.005571	0.013572	0.025548	0.00747	0.001313	0.005357	0.000722	0.00598	0.011604	0	0.002678	0.006704588
Virulence, Disease and Defense	NULL	C jejuni colonization of chick caeca	Rrf2 family transcriptional regulator	0.000696	0	0.00511	0.00249	0.005252	0.003896	0.002888	0.001495	0.002846	0.002203	0.000582	0.008203261
Carbohydrates	CO2 fixation	CO2 uptake, carboxysome	Rubisco activation protein CbbO	0.028549	0.006786	0.015329	0.02241	0.017727	0.008767	0.012998	0.010964	0.004817	0	0.01013	0.020350398
Carbohydrates	CO2 fixation	CO2 uptake, carboxysome	Rubisco activation protein CbbQ	0.01323	0.006786	0.010219	0.01494	0.013788	0.010715	0.015886	0.006479	0.003284	0	0.011527	0.019837694
Carbohydrates	CO2 fixation	CO2 uptake, carboxysome	RuBisCO operon transcriptional regulator	0.011837	0	0.002555	0.01743	0.006566	0.010228	0.009387	0.00897	0.006568	0.004406	0.003144	0.012935912
Stress Response	Oxidative stress	Rubrerythrin	Rubredoxin	0.021934	0.02262	0.017884	0.0249	0.028232	0.027761	0.023107	0.018439	0.010947	0.002203	0	0.022322335
Clustering-based subsystems	NULL	Cell division-ribosomal stress proteins cluster	S1 RNA binding domain protein	0.0094	0.009048	0.020438	0.00996	0.021666	0.015585	0.014442	0.012957	0.013575	0	0.001747	0.015223359
Amino Acids and Derivatives	Lysine, threonine, methionine, and cysteine	Lysine degradation	Saccharopine dehydrogenase (EC 1.5.1.9)	0.000696	0.004524	0.002555	0.00498	0.00197	0.000974	0.002888	0.001495	0.001752	0.041853	0	0.008952597
Amino Acids and Derivatives	Lysine, threonine, methionine, and cysteine	Lysine degradation	Saccharopine dehydrogenase [NADP , L-glutamate-forming] (EC 1.5.1.10)	0.001393	0.013572	0.012774	0.00498	0.001313	0.007793	0.004333	0.004485	0.003941	0	0.000815	0.018615092
Metabolism of Aromatic Compounds	Peripheral pathways for catabolism of aromatic compounds	Salicylate ester degradation	Salicylate hydroxylase (EC 1.14.13.1)	0.002089	0	0.00511	0.00249	0.00197	0.001948	0.002888	0.003987	0.007225	0.006608	0.000815	0.001616989
Clustering-based subsystems	NULL	Glutaredoxin 3 containing cluster 2	SAM-dependent methyltransferase, BioC-like	0.005919	0	0.002555	0.00249	0.005909	0.003896	0.006499	0.011961	0.002408	0.004406	0.000815	0.000867653
Regulation and Cell signaling	NULL	Global Two-component Regulator PrrBA in Proteobacteria	Sensor histidine kinase PrrB (RegB) (EC 2.7.3.-)	0.006615	0.002262	0.010219	0.00498	0.006566	0.006819	0.004333	0.017941	0.013575	0	0.001397	0.004377702
Virulence, Disease and Defense	Resistance to antibiotics and toxic compounds	Zinc resistance	Sensor protein of zinc sigma-54-dependent two-component system	0.003133	0.054288	0.015329	0.01245	0.01707	0.030684	0.018774	0.009967	0.010728	0.004406	0	0.021060295
Clustering-based subsystems	NULL	Conserved gene cluster associated with Met-tRNA formyltransferase	Serine/threonine protein kinase PrkC, regulator of stationary phase	0.061624	0.036192	0.048541	0.049801	0.026262	0.026787	0.020218	0.009469	0.056488	0	0.000349	0.030446719
Stress Response	NULL	SigmaB stress responce regulation	Serine-protein kinase rsbW (EC 2.7.11.1)	0.002437	0.002262	0.015329	0.00249	0.003939	0.000487	0.000722	0.001495	0.003503	0	0.000116	0.001301479
Carbohydrates	One-carbon Metabolism	Serine-glyoxylate cycle	Serine-pyruvate aminotransferase/archaeal aspartate aminotransferase	0.003482	0	0.002555	0.00498	0.003939	0.003409	0.002166	0.002492	0.006349	0.002203	0.000116	0.004141069
Miscellaneous	Plant-Prokaryote DOE project	At3g50560	Short-chain dehydrogenase, associated with 2-hydroxychromene-2-carboxylate isomerase family protein	0.004178	0.004524	0	0.00249	0.004596	0.003409	0.002888	0.00299	0.002627	0.002203	0.000116	0.000749336
Miscellaneous	Plant-Prokaryote DOE project	Competence or DNA damage-inducible protein CinA and related protein families	Similar to N-terminal domain of competence/damage-inducible protein CinA, molybdopterin binding motif	0.001044	0.006786	0.00511	0.01494	0.004596	0.001461	0.007221	0.007475	0.012261	0	0.000815	0.001814183
Phages, Prophages, Transposable elements, Plasmids	Phages, Prophages	Phage integration and excision	Site-specific tyrosine recombinase	0.014274	0.024882	0.012774	0.00747	0.015101	0.01315	0.005777	0.006977	0.003722	0.002203	0	0.007098976
Amino Acids and Derivatives	Alanine, serine, and glycine	Glycine cleavage system	Sodium/glycine symporter GlyP	0.021238	0.01131	0.002555	0.00249	0.009848	0.004383	0.008665	0.005482	0.001533	0	0.000349	0.009741372
Dormancy and Sporulation	NULL	Spore Core Dehydration	Spore maturation protein A	0.002437	0.004524	0.017884	0.00996	0.010505	0.005844	0.001444	0.00598	0.006131	0	0.000699	0.004969283
Protein Metabolism	Protein biosynthesis	Ribosome SSU bacterial	SSU ribosomal protein S18p	0.105492	0.020358	0.045986	0.01992	0.02101	0.017533	0.018052	0.032393	0.020143	0	0.002212	0.022519529
Stress Response	NULL	Carbon Starvation	Stringent starvation protein B	0	0.002262	0.00511	0.00747	0.00197	0.002435	0.010109	0.007475	0.009415	0.002203	0.01665	0.005994691
Cofactors, Vitamins, Prosthetic Groups, Pigments	Biotin	Biotin biosynthesis	Substrate-specific component BioY of biotin ECF transporter	0.002089	0.01131	0.002555	0.01743	0.006566	0.011202	0.006499	0.003489	0.004379	0	0.000582	0.003233978
Respiration	Electron donating reactions	Succinate dehydrogenase	Succinate dehydrogenase cytochrome b-556 subunit	0.002785	0.002262	0.00511	0.00249	0.004596	0.000974	0.005777	0.009967	0.011604	0	0.000815	0.001380356
Clustering-based subsystems	NULL	CBSS-83333.1.peg.946	Succinyl-CoA synthetase, alpha subunit-related enzymes	0.000696	0.002262	0.00511	0	0.006566	0.005357	0.001444	0.003489	0.006349	0.006608	0.011993	0.004574896
Amino Acids and Derivatives	Branched-chain amino acids	Branched chain amino acid degradation regulons	Succinyl-CoA:3-ketoacid-coenzyme A transferase subunit B (EC 2.8.3.5)	0.011141	0.01131	0.015329	0	0.005909	0.009254	0.010109	0.012957	0.011823	0.070489	0.039238	0.008439894
Clustering-based subsystems	Sulfatases and sulfatase modifying factor 1 (and a hypothetical)	Sulfatases and sulfatase modifying factor 1	Sulfatase modifying factor 1 precursor (C-alpha-formyglycine- generating enzyme 1)	0.003482	0.006786	0.012774	0.00996	0.004596	0.005844	0.004333	0.004984	0.005036	0	0.000349	0.006625711
Sulfur Metabolism	Inorganic sulfur assimilation	Inorganic Sulfur Assimilation	Sulfate permease, Trk-type	0.010445	0.01131	0.00511	0.00249	0.00197	0.004383	0.001444	0.012957	0.018173	0	0.04506	0.008400455
Amino Acids and Derivatives	Lysine, threonine, methionine, and cysteine	Cysteine Biosynthesis	Sulfite reductase [NADPH] flavoprotein alpha-component (EC 1.8.1.2)	0.000696	0	0.00511	0.00747	0.002626	0.001461	0.002888	0.007974	0.004817	0.008811	0.02352	0.003943875
Respiration	Electron accepting reactions	Anaerobic respiratory reductases	Sulfite reductase alpha subunit (EC 1.8.99.1)	0.029942	0.020358	0.043432	0.042331	0.020353	0.017046	0.024551	0.011462	0.005255	0.002203	0	0.016130451
Respiration	Electron accepting reactions	Anaerobic respiratory reductases	Sulfite reductase, dissimilatory-type gamma subunit (EC 1.8.99.3)	0.009052	0.031668	0.015329	0.01245	0.005252	0.014124	0.008665	0.005482	0.002846	0	0.001164	0.009346985
Sulfur Metabolism	NULL	Sulfate reduction-associated complexes	Sulfite reduction-associated complex DsrMKJOP protein DsrK (=HmeD)	0.082513	0.067859	0.06387	0.064741	0.077474	0.073056	0.037548	0.016446	0.010509	0.024231	0	0.044644671
Sulfur Metabolism	NULL	Sulfate reduction-associated complexes	Sulfite reduction-associated complex DsrMKJOP protein DsrM (= HmeC)	0.018452	0.015834	0.010219	0.00747	0.010505	0.019482	0.005055	0.00598	0.001314	0.002203	0	0.008163822
Sulfur Metabolism	NULL	Sulfur oxidation	Sulfur oxidation protein SoxX	0.003482	0.004524	0.007664	0.00498	0.004596	0.004383	0.00361	0.003987	0.001971	0	0.000931	0.003391733
Stress Response	Oxidative stress	Oxidative stress	Superoxide dismutase [Mn] (EC 1.15.1.1)	0.003133	0.006786	0.002555	0	0.003939	0.001461	0.00361	0.001993	0.00416	0.002203	0.000233	0.001459234
Membrane Transport	Protein secretion system, Type I	Type I secretion system for aggregation	T1SS associated transglutaminase-like cysteine proteinase (LapP)	0.005919	0	0.002555	0.00249	0.001313	0.000487	0.001444	0.000498	0.003503	0.006608	0.00326	0.002090254
RNA Metabolism	Transcription	RNA polymerase archaeal initiation factors	TATA-box binding protein	0.002785	0.006786	0.00511	0.00747	0.003939	0.005844	0.007943	0.002492	0.000219	0	0.000349	0.003352294
Carbohydrates	Organic acids	Tricarballylate Utilization	TcuA: flavoprotein used to oxidize tricarballylate to cis-aconitate	0.000696	0.006786	0.010219	0.00747	0.005909	0.011202	0.005055	0.00598	0.008539	0	0.059847	0.012107698
Cofactors, Vitamins, Prosthetic Groups, Pigments	NULL	Thiamin biosynthesis	Thiamin ABC transporter, substrate-binding component	0.005919	0.002262	0.00511	0.00498	0.003283	0.002435	0.007221	0.006479	0.007882	0	0.001979	0.00698066
Cofactors, Vitamins, Prosthetic Groups, Pigments	NULL	Thiamin biosynthesis	Thiamin ABC transporter, transmembrane component	0.002437	0.01131	0.007664	0.00996	0.006566	0.006332	0.005055	0.005482	0.004817	0.004406	0	0.005127038
Nucleosides and Nucleotides	Detoxification	Nudix proteins (nucleoside triphosphate hydrolases)	Thiamin-phosphate pyrophosphorylase-like protein	0.002089	0.006786	0.00511	0	0.001313	0.002435	0.000722	0.003489	0.001533	0.002203	0.000233	0.003431172
Cofactors, Vitamins, Prosthetic Groups, Pigments	NULL	Thiamin biosynthesis	Thiamin-regulated outer membrane receptor Omr1	0.005919	0.009048	0	0.00498	0.000657	0.003409	0.002166	0.009469	0.007444	0.083706	0.000349	0.04212059
Protein Metabolism	Protein degradation	Protein degradation	Thimet oligopeptidase (EC 3.4.24.15)	0	0.01131	0.015329	0.00249	0.006566	0.000974	0.000722	0.004485	0.003722	0.002203	0.000116	0.002011377
Cofactors, Vitamins, Prosthetic Groups, Pigments	Folate and pterines	YgfZ	Thiol:disulfide interchange protein DsbC	0.002785	0.013572	0	0.00747	0.004596	0.002435	0.00361	0.006977	0.006787	0.004406	0.000233	0.005955252
Sulfur Metabolism	NULL	Sulfur oxidation	thioredoxin SoxW	0.002785	0.002262	0	0.00747	0.002626	0.003409	0.005055	0.00299	0.001971	0.006608	0.000233	0.001222601
Protein Metabolism	Protein degradation	Putative TldE-TldD proteolytic complex	TldE/PmbA family protein, Beta/Gamma-proteobacterial subgroup	0.002785	0.009048	0.002555	0.00996	0.002626	0.005844	0.004333	0.003489	0.005036	0	0.000699	0.004219947
Membrane Transport	NULL	Ton and Tol transport systems	Tol biopolymer transport system, TolR protein	0.005919	0.01131	0.002555	0.00249	0.003939	0.006332	0.005777	0.007475	0.009196	0	0.004075	0.004772089
Membrane Transport	NULL	Ton and Tol transport systems	TolA protein	0.011837	0.004524	0.012774	0.02739	0.01904	0.010715	0.011553	0.013954	0.007225	0	0.000233	0.008439894
Metabolism of Aromatic Compounds	Peripheral pathways for catabolism of aromatic compounds	Toluene degradation	toluenesulfonate zinc-independent alcohol dehydrogenase	0.00383	0.004524	0	0.00996	0.000657	0.000974	0.005777	0.000498	0.005693	0.002203	0.000815	0.001143724
Membrane Transport	NULL	Ton and Tol transport systems	TPR domain protein, putative component of TonB system	0.033075	0.029406	0.043432	0.01992	0.01904	0.020456	0.020218	0.017941	0.040505	0	0.02841	0.016840348

Membrane Transport	NULL	Ton and Tol transport systems	TPR repeat containing exported protein	0.004178	0.004524	0.00511	0.00747	0.014444	0.010715	0.00361	0.007974	0.017297	0	0.000815	0.009662495
Miscellaneous	Plant-Prokaryote DOE project	At3g21300	Transcription regulator [contains diacylglycerol kinase catalytic domain]	0.003482	0.006786	0.002555	0.00249	0.005909	0.005357	0.010831	0.003987	0.004379	0	0.000815	0.006507395
Virulence, Disease and Defense	Resistance to antibiotics and toxic compounds	BlaR1 Family Regulatory Sensor-transducer Disambiguation	Transcriptional repressor, BlaI/MecI family	0.00383	0.006786	0.002555	0.00249	0.002626	0.005357	0.001444	0.00299	0.002846	0	0.006287	0.001616989
Membrane Transport	NULL	Transport of Nickel and Cobalt	Transmembrane component NikQ of energizing module of nickel ECF transporter	0.00383	0.004524	0.002555	0.00498	0.009192	0.003409	0.006499	0.003489	0.002627	0	0.000349	0.003352294
Respiration	Electron accepting reactions	Terminal cytochrome oxidases	Transport ATP-binding protein CydC	0.003133	0.024882	0.017884	0.00249	0.012475	0.012176	0.007221	0.003987	0.022114	0	0.008965	0.009780811
Carbohydrates	Di- and oligosaccharides	Trehalose Biosynthesis	Trehalose-6-phosphate phosphatase (EC 3.1.3.12)	0.013926	0.006786	0.017884	0.01992	0.024293	0.012176	0.010831	0.007974	0.016421	0	0.000466	0.007651118
Cofactors, Vitamins, Prosthetic Groups, Pigments	Folate and pterines	YgfZ-Iron	Tricarboxylate transport protein TctB	0.00383	0.002262	0.00511	0.00498	0.007222	0.008767	0.00361	0.002492	0.011166	0	0.096407	0.009149791
Respiration	Electron accepting reactions	trimethylamine N-oxide (TMAO) reductase	Trimethylamine-N-oxide reductase (Cytochrome c) (EC 1.7.2.3)	0.000348	0.01131	0.002555	0.00498	0.005909	0.002922	0.006499	0.006977	0.00832	0.002203	0	0.002366325
RNA Metabolism	RNA processing and modification	RNA methylation	tRNA (adenine37-N(6)-)-methyltransferase TrmN6	0.001044	0.004524	0	0.00498	0.005252	0.007306	0.004333	0.005482	0.002408	0.019825	0.002911	0.014986727
RNA Metabolism	RNA processing and modification	RNA methylation	tRNA (Guanosine18-2'-O-) -methyltransferase (EC 2.1.1.34)	0.007311	0.002262	0.002555	0.00747	0.006566	0.00487	0.007943	0.006479	0.016421	0	0.013041	0.013448615
Clustering-based subsystems	Translation	CBSS-326442.4.peg.1852	tRNA 2-thiouridine synthesizing protein E (EC 2.8.1.-)	0.006267	0.01131	0.00511	0.01494	0.004596	0.008767	0.002888	0.004984	0.006568	0	0.001048	0.006389078
Clustering-based subsystems	Translation	CBSS-326442.4.peg.1852	tRNA 5-methylaminomethyl-2-thiouridine synthase TusC	0.005571	0.006786	0.007664	0.00249	0.005252	0.00487	0.002166	0.002492	0.001971	0	0.002678	0.004259386
RNA Metabolism	RNA processing and modification	mnm5U34 biosynthesis bacteria	tRNA 5-methylaminomethyl-2-thiouridine synthase TusD	0	0.006786	0.007664	0.00747	0.007222	0.005844	0.010831	0.004485	0.00416	0.019825	0.015136	0.006625711
Cofactors, Vitamins, Prosthetic Groups, Pigments	NAD and NADP	NAD and NADP cofactor biosynthesis global	Tryptophan 2,3-dioxygenase (EC 1.13.11.11)	0.006267	0.013572	0	0.032371	0.010505	0.007793	0.018052	0.028407	0.032842	0.046259	0.000233	0.006822905
Regulation and Cell signaling	NULL	Orphan regulatory proteins	Two-component oxygen-sensor histidine kinase FixL	0.004526	0.01131	0.00511	0.01992	0.005252	0.005844	0.006499	0.001993	0.007663	0.01542	0	0.007493363
Virulence, Disease and Defense	Bacteriocins, ribosomally synthesized antibacterial peptides	Tolerance to colicin E2	Two-component response regulator CreB	0.000696	0.002262	0.002555	0.00747	0.009848	0.00487	0.001444	0.003987	0.003503	0	0.000116	0.004614334
Iron acquisition and metabolism	NULL	Heme, hemin uptake and utilization systems in GramPositives	Two-component response regulator SA14-24	0.004178	0.015834	0.007664	0.00249	0.003283	0.004383	0.005055	0.001495	0.005036	0.006608	0	0.01013576
Cell Wall and Capsule	Capsular and extracellular polysacchrides	Capsular Polysaccharides Biosynthesis and Assembly	Tyrosine-protein kinase Wzc (EC 2.7.10.2)	0.048046	0	0.015329	0.00498	0.004596	0.005844	0.009387	0.010466	0.021676	0.068286	0.017931	0.032694728
Clustering-based subsystems	NULL	CBSS-296591.1.peg.2330	UDP-glucuronate 5'-epimerase (EC 5.1.3.12)	0.004178	0.002262	0.007664	0.00249	0.003283	0.001461	0.002888	0.00598	0.008977	0	0.000699	0.004456579
Cell Wall and Capsule	Capsular and extracellular polysacchrides	Vibrio Polysaccharide (VPS) Biosynthesis	UDP-N-acetyl-D-mannosaminuronate dehydrogenase	0.048742	0.015834	0.012774	0.01245	0.026262	0.025326	0.024551	0.008472	0.010947	0	0.014321	0.021967387
Stress Response	Oxidative stress	Glutaredoxins	Uncharacterized monothiol glutaredoxin ycf64-like	0.004874	0.004524	0	0.00249	0.007222	0.002922	0.008665	0.008472	0.008101	0.002203	0.000582	0.001971938
Iron acquisition and metabolism	NULL	Encapsulating protein for DyP-type peroxidase and ferritin-like protein oligomers	Uncharacterized protein COG3461	0.008356	0.013572	0.002555	0.0249	0.006566	0.007793	0.012275	0.013456	0.007444	0	0.000233	0.005994691
Membrane Transport	Protein secretion system, Type VI	Type VI secretion systems	Uncharacterized protein ImpB	0	0.004524	0.00511	0.00747	0.007222	0.009741	0.004333	0.008472	0.006349	0.006608	0.013972	0.007177853
Carbohydrates	NULL	Unknown sugar utilization (cluster yphABCDEFG)	Uncharacterized protein YphG, TPR-domain containing	0.005919	0.006786	0.00511	0.01743	0.004596	0.005357	0.00361	0.001495	0.003722	0	0.000116	0.008439894
Protein Metabolism	Protein degradation	Proteasome bacterial	Uncharacterized protein, similar to the N-terminal domain of Lon protease	0.011837	0.002262	0.002555	0.00498	0.00197	0.010715	0.005777	0.011462	0.015983	0	0.000815	0.005521426
Stress Response	NULL	Universal stress protein family	Universal stress protein family	0.012186	0.015834	0.012774	0.032371	0.011818	0.019482	0.015164	0.018938	0.018173	0	0.006637	0.013882442
Miscellaneous	Plant-Prokaryote DOE project	Competence or DNA damage-inducible protein CinA and related protein families	UPF0125 protein yfjF	0.006615	0.006786	0.007664	0.00249	0.003939	0.004383	0.00361	0.003987	0.007444	0	0.021657	0.006152446
Clustering-based subsystems	NULL	CBSS-320372.3.peg.6046	UPF0301 protein YqgE	0.013926	0	0.010219	0.00996	0.014444	0.013637	0.010109	0.009967	0.013137	0.004406	0.011061	0.011871065
Nucleosides and Nucleotides	Purines	Purine Utilization	Uracil-xanthine permease	0.002089	0	0.002555	0.00249	0.004596	0.002922	0.001444	0.003489	0.003941	0.004406	0.000582	0.00094653
Amino Acids and Derivatives	Arginine; urea cycle, polyamines	Urea decomposition	Urea ABC transporter, permease protein UrtB	0.012186	0.02262	0	0.01743	0.015101	0.015585	0.014442	0.014452	0.041162	0.013217	0.047272	0.015775502
Amino Acids and Derivatives	Arginine; urea cycle, polyamines	Urea decomposition	Urea carboxylase-related aminomethyltransferase (EC 2.1.2.10)	0.013926	0.002262	0.002555	0.00747	0.007222	0.005357	0.008665	0.010964	0.007663	0	0.001747	0.009701934
Protein Metabolism	Protein processing and modification	G3E family of P-loop GTPases (metallocenter biosynthesis)	Urease accessory protein UreD	0.000348	0.002262	0.00511	0	0.000657	0.002922	0.002166	0.008472	0.004379	0.004406	0.000233	0.002090254
Protein Metabolism	Protein processing and modification	G3E family of P-loop GTPases (metallocenter biosynthesis)	Urease accessory protein UreF	0.004526	0.004524	0.002555	0.00498	0.001313	0.001461	0.007943	0.003489	0.003722	0.002203	0	0.004693212
Cofactors, Vitamins, Prosthetic Groups, Pigments	Tetrapyrroles	Heme and Siroheme Biosynthesis	Uroporphyrinogen-III synthase (EC 4.2.1.75)	0.008356	0.01131	0.015329	0.01494	0.014444	0.013637	0.008665	0.006479	0.008101	0	0.012575	0.008597649
Respiration	Electron accepting reactions	Anaerobic respiratory reductases	Vanillate O-demethylase oxidoreductase (EC 1.14.13.-)	0.001044	0.004524	0	0.00747	0.007222	0.003409	0.007943	0.003987	0.006568	0.006608	0.037957	0.002602958
Metabolism of Aromatic Compounds	Peripheral pathways for catabolism of aromatic compounds	Phenylpropanoid compound degradation	Vanillate O-demethylase oxygenase subunit (EC 1.14.13.82)	0.002437	0	0.00511	0.00249	0.008535	0.001948	0.00361	0.003987	0.006131	0.011014	0.039238	0.00157755
Stress Response	Detoxification	Uptake of selenate and selenite	Various polyols ABC transporter, permease component 2	0.003482	0	0.007664	0.00498	0.009848	0.009254	0.002166	0.006977	0.009415	0.006608	0.000931	0.002760713
DNA Metabolism	DNA repair	DNA repair, bacterial	Very-short-patch mismatch repair endonuclease (G-T specific)	0.000348	0.006786	0.010219	0	0.004596	0.002922	0.00361	0.000997	0.005255	0.004406	0.000233	0.0031551
Respiration	ATP synthases	V-Type ATP synthase	V-type ATP synthase subunit D (EC 3.6.3.14)	0.001044	0.018096	0.015329	0	0.011161	0.005357	0.008665	0.013456	0.006349	0.002203	0.001048	0.006744027
Miscellaneous	Plant-Prokaryote DOE project	Iojap	V-type ATP synthase subunit K (EC 3.6.3.14)	0.010097	0.020358	0.010219	0.01245	0.013788	0.016072	0.005777	0.005482	0.003722	0.002203	0	0.006231323
Nucleosides and Nucleotides	Purines	Purine Utilization	Xanthine and CO dehydrogenases maturation factor, XdhC/CoxF family	0.018801	0.015834	0.033212	0.01245	0.018384	0.018508	0.025273	0.010964	0.00832	0	0.000349	0.011437239
Nucleosides and Nucleotides	Purines	Purine Utilization	Xanthine permease	0	0.002262	0.00511	0.00498	0.00197	0.002922	0.001444	0.002492	0.005693	0.041853	0.133084	0.003904437
Carbohydrates	Monosaccharides	Xylose utilization	Xylanase	0.000696	0.015834	0.002555	0	0.003283	0.002435	0.007221	0.001993	0.016202	0.002203	0.000699	0.008992036
Carbohydrates	Monosaccharides	Xylose utilization	Xylose-responsive transcription regulator, ROK family	0.002785	0.006786	0.002555	0	0.003939	0.003409	0.001444	0.001993	0.002627	0.002203	0.000116	0.001222601
Membrane Transport	Protein and nucleoprotein secretion system, Type IV	Conjugative transfer	Yac	0.001741	0.004524	0.00511	0.00249	0.004596	0.001948	0.002888	0.001495	0.002627	0	0.005822	0.002287448
Miscellaneous	NULL	Broadly distributed proteins not in subsystems	YbbL ABC transporter ATP-binding protein	0.001393	0.004524	0.002555	0	0.004596	0.003409	0.004333	0.001495	0.005474	0.002203	0.000931	0.001656428
Miscellaneous	NULL	Broadly distributed proteins not in subsystems	YbbM seven transmembrane helix protein	0.018104	0.01131	0.012774	0.01245	0.019697	0.018995	0.011553	0.017443	0.013575	0	0.00163	0.010411831
Miscellaneous	NULL	Broadly distributed proteins not in subsystems	YciL protein	0.005571	0.006786	0.020438	0.00498	0.004596	0.005357	0.002888	0.004984	0.005693	0	0.011061	0.006507395
Membrane Transport	Protein and nucleoprotein secretion system, Type IV	Conjugative transfer	Ync	0	0.02262	0.00511	0.00498	0.010505	0.010228	0.012998	0.009469	0.012042	0.011014	0.017349	0.006191885
Stress Response	Oxidative stress	Oxidative stress	Zinc uptake regulation protein ZUR	0.00383	0.002262	0.002555	0	0.009848	0.002922	0.007943	0.009469	0.007225	0.011014	0.014205	0.003352294
Membrane Transport	NULL	Transport of Zinc	Zinc-regulated TonB-dependent outer membrane receptor	0.003482	0.01131	0.002555	0.00498	0.003283	0.002435	0.002888	0.003987	0.001971	0	0.000233	0.005521426
Clustering-based subsystems	NULL	Disulphide related cluster	Zn-dependent hydroxyacylglutathione hydrolase	0.002437	0.004524	0.015329	0.00249	0.003939	0.003896	0.002888	0.006479	0.008101	0	0.000116	0.002484642
Fatty Acids, Lipids, and Isoprenoids	Isoprenoids	Archaeal lipids	(S)-3-O-geranylgeranylglyceryl phosphate synthase	0.002089	0.01131	0	0.00498	0.003939	0.003896	0.007943	0.006977	0.002627	0	0.000582	0.010175199
Respiration	Electron donating reactions	Hydrogenases	[Fe] hydrogenase, HymB subunit, putative	0.025416	0.02262	0.00511	0.032371	0.011818	0.010228	0.011553	0.001993	0.001095	0	0	0.01108229
Amino Acids and Derivatives	Lysine, threonine, methionine, and cysteine	Methionine Salvage	1,2-dihydroxy-3-keto-5-methylthiopentene dioxygenase (EC 1.13.11.54)	0.003482	0	0.002555	0	0.000657	0.000487	0.002166	0.001495	0.003065	0.01542	0.006753	0.005008722

Clustering-based subsystems	NULL	Conserved gene cluster associated with Met-tRNA formyltransferase	16S rRNA m(5)C 967 methyltransferase (EC 2.1.1.-)	0.001393	0	0.002555	0.00747	0.004596	0.005357	0.002888	0.009967	0.004598	0.002203	0	0.001064846
Iron acquisition and metabolism	NULL	Iron acquisition in Vibrio	2,3-dihydroxybenzoate-AMP ligase (EC 2.7.7.58)	0.001044	0.004524	0.002555	0	0.003283	0.000974	0.000722	0.00299	0.002408	0	0.000349	0.001222601
Carbohydrates	Monosaccharides	L-fucose utilization temp	2,4-diketo-3-deoxy-L-fuconate hydrolase	0.025764	0	0.00511	0.00996	0	0.002435	0.004333	0.003489	0.004817	0.008811	0.001048	0.001616989
Clustering-based subsystems	NULL	CBSS-160492.1.peg.550	21 kDa hemolysin precursor	0	0.004524	0.002555	0.00249	0.003283	0.000974	0.00361	0.003987	0.003722	0	0.007568	0.001340918
RNA Metabolism	RNA processing and modification	RNA methylation	23S rRNA (guanine-N-2-) -methyltransferase rlmL EC 2.1.1.-)	0.046305	0.01131	0	0.01743	0.007879	0.014124	0.01372	0.016944	0.022552	0	0.010595	0.018141827
Cofactors, Vitamins, Prosthetic Groups, Pigments	Tetrapyrroles	Chlorophyll Biosynthesis	2-desacetyl-2-hydroxyethyl bacteriochlorophyllide A dehydrogenase BchC	0.006963	0.004524	0	0.00747	0.003283	0.00828	0.008665	0.011462	0.012261	0	0.000466	0.000276071
Metabolism of Aromatic Compounds	NULL	carbazol degradation cluster	2-hydroxy-6-oxo-6-phenylhexa-2,4-dienoate hydrolase (EC 3.7.1.-)	0.002785	0	0	0.00249	0.003283	0.002435	0.002888	0.006479	0.010509	0.077098	0.044943	0.012423208
Metabolism of Aromatic Compounds	Peripheral pathways for catabolism of aromatic compounds	Biphenyl Degradation	2-keto-4-pentenoate hydratase (EC 4.2.1.-)	0	0.004524	0	0.00498	0.000657	0.002435	0.005055	0.00299	0.001314	0.013217	0.077545	0.005127038
Carbohydrates	Organic acids	2-methylcitrate to 2-methylaconitate metabolism cluster	2-Methylcitrate dehydratase AcnD	0.003133	0.002262	0.002555	0	0.002626	0.000974	0.002888	0.002492	0.002408	0	0.000116	0.000709898
Clustering-based subsystems	NULL	CBSS-342610.3.peg.1794	3',5'-cyclic-nucleotide phosphodiesterase (EC 3.1.4.17)	0.003133	0.006786	0	0.00249	0.004596	0.006332	0.00361	0.009469	0.007225	0	0.001281	0.002011377
Metabolism of Aromatic Compounds	Metabolism of central aromatic intermediates	Protocatechuate branch of beta-ketoadipate pathway	3-carboxy-cis,cis-muconate cycloisomerase (EC 5.5.1.2)	0.003133	0.002262	0	0	0.00197	0.000487	0.005055	0.005482	0.00832	0.011014	0.000466	0.00189306
Amino Acids and Derivatives	Branched-chain amino acids	Branched chain amino acid degradation regulons	3-hydroxyisobutyryl-CoA hydrolase (EC 3.1.2.4)	0	0.004524	0.033212	0.00747	0.002626	0.00487	0.005055	0.015449	0.02277	0	0.029691	0.006783466
Miscellaneous	Plant-Prokaryote DOE project	At1g52510 AT4G12830 (COG0596)	3-oxoacyl-[acyl-carrier-protein] synthase, KASIII in hypothetical gene cluster	0.005919	0	0.007664	0.00249	0.005252	0.012663	0.005777	0.006977	0.001752	0	0.000349	0.005758058
Cofactors, Vitamins, Prosthetic Groups, Pigments	Folate and pterines	Pterin biosynthesis	4-amino-4-deoxy-L-arabinose transferase and related glycosyltransferases of PMT family	0.000348	0.002262	0	0	0.003939	0.000487	0.00361	0.000498	0.005255	0.004406	0.000116	0.002287448
Metabolism of Aromatic Compounds	Metabolism of central aromatic intermediates	Protocatechuate branch of beta-ketoadipate pathway	4-carboxymuconolactone decarboxylase (EC 4.1.1.44)	0.012186	0.01131	0	0.01245	0.009192	0.004383	0.007221	0.005482	0.007663	0	0.002445	0.009110352
Metabolism of Aromatic Compounds	NULL	Benzoate transport and degradation cluster	4-hydroxybenzoyl-CoA reductase, beta subunit (EC 1.3.99.20)	0.000696	0.004524	0	0.00747	0.00197	0.001461	0.001444	0.004485	0.002189	0	0.000233	0.001025408
Metabolism of Aromatic Compounds	NULL	Benzoate transport and degradation cluster	4-oxalocrotonate decarboxylase (EC 4.1.1.77)	0	0	0.002555	0.01245	0.003283	0.001948	0.000722	0.001495	0.003284	0.006608	0.045293	0.005127038
RNA Metabolism	RNA processing and modification	tRNA modification Bacteria	5-methylaminomethyl-2-thiouridine-forming enzyme mnmC	0.001393	0	0.002555	0	0.001313	0.005357	0.001444	0.010466	0.010947	0.002203	0.047389	0.006467956
Nucleosides and Nucleotides	Detoxification	Nudix proteins (nucleoside triphosphate hydrolases)	5-methyl-dCTP pyrophosphohydrolase (EC 3.6.1.-)	0.001393	0.002262	0.00511	0.00249	0.005909	0.005357	0.012275	0.007475	0.005474	0	0	0.002090254
Respiration	NULL	Carbon monoxide induced hydrogenase	5-methyltetrahydrofolate:corrinoid iron-sulfur protein methyltransferase	0.008704	0.013572	0.00511	0.01494	0.023636	0.01315	0.012998	0.003489	0.000438	0	0	0.007572241
Carbohydrates	Central carbohydrate metabolism	Glycolysis and Gluconeogenesis	6-phosphofructokinase, eukaryotic type (EC 2.7.1.11)	0.004526	0.009048	0.00511	0.00498	0.003939	0.003409	0.00361	0.004485	0.002627	0	0	0.004141069
Cofactors, Vitamins, Prosthetic Groups, Pigments	Folate and pterines	Methanopterin biosynthesis2	6-pyruvoyl tetrahydrobiopterin synthase (EC 4.2.3.12)	0.01323	0	0.002555	0.00249	0.004596	0.005357	0.006499	0.007475	0.008977	0	0.000582	0.012226014
Cofactors, Vitamins, Prosthetic Groups, Pigments	Folate and pterines	methanopterin biosynthesis	7,8 dihydropteroate synthase (methanopterin)	0.009748	0.006786	0.002555	0.02739	0.002626	0.006819	0.001444	0.001993	0.001752	0	0	0.004101631
Clustering-based subsystems	Protein export?	CBSS-393121.3.peg.2760	ABC transporter ATP-binding protein uup	0	0.009048	0.00511	0	0.001313	0.001461	0.00361	0.00299	0.011166	0.004406	0.016766	0.008045506
Miscellaneous	Plant-Prokaryote DOE project	COG0398	ABC transporter, periplasmic substrate-binding protein YnjB	0.003133	0.002262	0	0	0.011161	0.008767	0.007221	0.013954	0.007663	0.019825	0.000349	0.003273417
Sulfur Metabolism	Inorganic sulfur assimilation	Inorganic Sulfur Assimilation	ABC-type probable sulfate transporter, ATPase component	0.002089	0.002262	0	0.00249	0.001313	0.001948	0.006499	0.001495	0.002408	0	0.000466	0.001340918
Metabolism of Aromatic Compounds	Peripheral pathways for catabolism of aromatic compounds	Biphenyl Degradation	Acetaldehyde dehydrogenase, acetylating, (EC 1.2.1.10) in gene cluster for degradation of phenols, cresols, catechol	0.002437	0.009048	0	0	0.004596	0.001948	0.002166	0.004485	0.007663	0.008811	0.126913	0.00855821
Respiration	NULL	Carbon monoxide induced hydrogenase	Acetyl-CoA synthase corrinoid iron-sulfur protein, large subunit	0.025416	0.047502	0.035767	0.062251	0.063029	0.052113	0.032494	0.006977	0.002189	0	0	0.021415244
Respiration	NULL	Carbon monoxide induced hydrogenase	Acetyl-CoA synthase corrinoid iron-sulfur protein, small subunit	0.015319	0.049764	0.040877	0.01743	0.036767	0.030196	0.015164	0.008472	0.002189	0	0	0.014119074
Carbohydrates	Central carbohydrate metabolism	Pyruvate metabolism II: acetyl-CoA, acetogenesis from pyruvate	Acetyl-CoA synthetase (ADP-forming) beta chain (EC 6.2.1.13)	0.006615	0.004524	0.015329	0.00498	0.004596	0.011689	0.004333	0.000498	0.002189	0	0	0.011673871
Clustering-based subsystems	NULL	CBSS-176280.1.peg.1561	Acyl-CoA hydrolase (EC 3.1.2.20)	0.008356	0	0.007664	0	0.003939	0.001948	0.00361	0.001495	0.003284	0.004406	0.000233	0.003352294
Carbohydrates	Central carbohydrate metabolism	Pyruvate metabolism II: acetyl-CoA, acetogenesis from pyruvate	Acylphosphate phosphohydrolase (EC 3.6.1.7), putative	0.009748	0.015834	0.012774	0.00747	0.016414	0.012176	0.007943	0.011462	0.003941	0	0	0.005442548
Miscellaneous	Plant-Prokaryote DOE project	At1g54520	Adenosylcobinamide-phosphate guanylyltransferase (EC 2.7.7.62)	0.000696	0.009048	0	0.00747	0.007879	0.012663	0.005777	0.010466	0.005693	0	0.002212	0.007217292
Regulation and Cell signaling	NULL	cAMP signaling in bacteria	Adenylate/guanylate cyclase (EC 4.6.1.2)	0.014623	0.002262	0.010219	0.00249	0.002626	0.008767	0.002888	0.003987	0.008539	0	0	0.004496018
Sulfur Metabolism	Inorganic sulfur assimilation	Inorganic Sulfur Assimilation	Adenylylsulfate reductase alpha-subunit (EC 1.8.99.2)	0.110714	0.074645	0.074089	0.049801	0.063029	0.055036	0.044769	0.017443	0.014013	0	0	0.044408038
Virulence, Disease and Defense	Adhesion	Adhesins in Staphylococcus	Adhesin of unknown specificity SdrC	0.002437	0.002262	0.002555	0.00249	0.00197	0.001461	0.002166	0.000498	0.000219	0	0	0.000985969
Protein Metabolism	Protein biosynthesis	tRNA aminoacylation, Ala	Alanyl-tRNA synthetase domain protein	0.007659	0.009048	0.012774	0.00996	0.013788	0.014611	0.010109	0.013954	0.006349	0	0	0.007335608
Protein Metabolism	Protein biosynthesis	tRNA aminoacylation, Ala	Alanyl-tRNA synthetase family protein	0.003482	0.002262	0.007664	0.00498	0.003283	0.001461	0.002166	0.000498	0.001095	0	0	0.001656428
Nitrogen Metabolism	NULL	Allantoin Utilization	Allantoinase (EC 3.5.2.5)	0.005222	0	0	0.00996	0.00197	0.001948	0.000722	0.00299	0.006787	0.006608	0.000349	0.00220857
Carbohydrates	Polysaccharides	Glycogen metabolism	Alpha-glucosidase, family 31 of glycosyl hydrolases, COG1501	0	0	0.002555	0.01245	0.002626	0.004383	0.001444	0.009469	0.006787	0.044056	0.023752	0.042160029
Carbohydrates	NULL	Sugar utilization in Thermotogales	Alpha-glucuronidase (EC 3.2.1.139)	0.004526	0.002262	0	0.00249	0.003283	0.000974	0	0.00299	0.009196	0.002203	0.000233	0.003391733
Cofactors, Vitamins, Prosthetic Groups, Pigments	Tetrapyrroles	Cobalamin synthesis	Alpha-ribazole-5'-phosphate phosphatase (EC 3.1.3.73)	0.002089	0.004524	0	0.00249	0.005252	0.003409	0.005777	0.001495	0.002408	0	0.000582	0.003076223
Protein Metabolism	Protein degradation	Aminopeptidases (EC 3.4.11.-)	Aminopeptidase Y (Arg, Lys, Leu preference) (EC 3.4.11.15)	0.002089	0.002262	0	0.00249	0.002626	0.005357	0.004333	0.000498	0.007006	0	0.010013	0.003352294
Nitrogen Metabolism	NULL	Ammonia assimilation	ammonium/methylammonium permease	0.001393	0.002262	0	0.00249	0.002626	0.000974	0.002166	0.001495	0.004817	0	0.000116	0.003588927
Respiration	Electron accepting reactions	Anaerobic respiratory reductases	Anaerobic dimethyl sulfoxide reductase chain C (EC 1.8.99.-)	0.031334	0.002262	0.007664	0.01494	0.007879	0.009254	0.010109	0.011961	0.005255	0	0	0.00761168
Virulence, Disease and Defense	Adhesion	Adhesins in Staphylococcus	Antiadhesin Pls, binding to squamous nasal epithelial cells	0.002785	0.020358	0.00511	0.00249	0.004596	0.005844	0.00361	0.002492	0.001095	0	0	0.002248009
Carbohydrates	Monosaccharides	L-Arabinose utilization	Arabinan endo-1,5-alpha-L-arabinosidase (EC 3.2.1.99)	0.001044	0.006786	0.010219	0	0.002626	0.001948	0.005055	0.001495	0.008539	0	0.00524	0.007138415
RNA Metabolism	RNA processing and modification	tRNA modification Archaea	archaeal ATPase, fused to C-terminal DUF234 domain	0.000696	0.002262	0.002555	0	0.003939	0.002435	0.000722	0.000997	0.001533	0	0.000233	0.00220857
Amino Acids and Derivatives	Arginine; urea cycle, polyamines	Arginine and Ornithine Degradation	Arginine deiminase (EC 3.5.3.6)	0.008704	0.006786	0	0.00249	0.002626	0.006819	0.009387	0.008472	0.013356	0	0.000233	0.003391733
Amino Acids and Derivatives	Arginine; urea cycle, polyamines	Arginine and Ornithine Degradation	Arginine N-succinyltransferase (EC 2.3.1.109)	0.035164	0.002262	0.00511	0	0.00197	0	0.00361	0.00299	0.018392	0.011014	0.054375	0.01108229
Amino Acids and Derivatives	Arginine; urea cycle, polyamines	Arginine Biosynthesis extended	Arginine pathway regulatory protein ArgR, repressor of arg regulon	0.011141	0	0	0.00498	0.00197	0.000974	0.00361	0.000997	0.01029	0.002203	0.000931	0.00283959
Amino Acids and Derivatives	Arginine; urea cycle, polyamines	Arginine and Ornithine Degradation	Arginine/ornithine ABC transporter, ATP-binding protein AotP	0	0	0.002555	0.00498	0.005252	0.002922	0.004333	0.00598	0.009415	0.002203	0.001863	0.002721274
Cofactors, Vitamins, Prosthetic Groups, Pigments	Folate and pterines	Pterin carbinolamine dehydratase	Aromatic-amino-acid aminotransferase (EC 2.6.1.57)	0.00383	0.004524	0	0	0.000657	0.002435	0.002888	0.001495	0.003941	0.006608	0.001164	0.000828214
DNA Metabolism	DNA replication	DNA-replication	Arrested fork binding	0.001044	0.006786	0	0.00249	0.005252	0.006819	0.012998	0.00897	0.004598	0	0.004424	0.002169132
Amino Acids and Derivatives	Glutamine, glutamate, aspartate, asparagine; ammonia assimilation	Glutamine, Glutamate, Aspartate and Asparagine Biosynthesis	Asparagine synthetase [glutamine-hydrolyzing] (EC 6.3.5.4) AsnH	0.010793	0.02262	0.025548	0.00747	0.028888	0.018995	0.019496	0.012459	0.015545	0	0	0.014316268

Respiration	ATP synthases	F0F1-type ATP synthase	ATP synthase B' chain (EC 3.6.3.14)	0.004526	0	0	0.00249	0.003939	0.000974	0.00361	0.006479	0.003284	0.002203	0.000349	0.003194539
Cofactors, Vitamins, Prosthetic Groups, Pigments	Tetrapyrroles	Coenzyme B12 biosynthesis	ATPase component CbiO of energizing module of cobalt ECF transporter	0	0.004524	0.002555	0	0.004596	0.000974	0.000722	0.001495	0.000219	0.002203	0.000233	0.003707243
Protein Metabolism	Protein degradation	Proteolysis in bacteria, ATP-dependent	ATPase, AFG1 family	0.006615	0.004524	0	0	0.005252	0.00487	0.010109	0.011961	0.017735	0.008811	0.040635	0.012778157
Protein Metabolism	Protein degradation	Proteasome bacterial	ATP-dependent protease LonB Type II	0	0.002262	0.00511	0.00498	0.003939	0.007306	0.004333	0.006479	0.001533	0	0.000699	0.003194539
RNA Metabolism	RNA processing and modification	ATP-dependent RNA helicases, bacterial	ATP-dependent RNA helicase Bcep18194 A5658	0.004874	0.002262	0.002555	0.00498	0.00197	0.001948	0.002166	0.000498	0.002189	0	0	0.001419795
Regulation and Cell signaling	Programmed Cell Death and Toxin-antitoxin Systems	Murein hydrolase regulation and cell death	Autolysis response regulater LytR	0.003133	0	0.002555	0.00249	0.001313	0.002435	0.002166	0.002492	0.004379	0.013217	0	0.008715965
Protein Metabolism	Protein processing and modification	G3E family of P-loop GTPases (metallocenter biosynthesis)	B12 binding domain of Methylmalonyl-CoA mutase (EC 5.4.99.2)	0.001741	0.004524	0.007664	0	0.002626	0.003896	0.00361	0.001495	0.003065	0.004406	0	0.003233978
Miscellaneous	Plant-Prokaryote DOE project	Iron-sulfur cluster assembly	Believed to be involved in assembly of Fe-S clusters	0.008356	0.004524	0.002555	0	0.003939	0.004383	0.006499	0.002492	0.001095	0	0.000116	0.003076223
Carbohydrates	Di- and oligosaccharides	Beta-Glucoside Metabolism	Beta-glucanase precursor (EC 3.2.1.73)	0.002785	0.01131	0	0.01245	0.00197	0.006819	0.005055	0.011961	0.021457	0	0.016068	0.01869397
Amino Acids and Derivatives	Lysine, threonine, methionine, and cysteine	Lysine degradation	Beta-lysine acetyltransferase (EC 2.3.1.-)	0	0.004524	0.007664	0.00747	0.003939	0.005357	0.009387	0.004485	0.000876	0	0.000116	0.004732651
Virulence, Disease and Defense	Resistance to antibiotics and toxic compounds	Cadmium resistance	Cadmium efflux system accessory protein	0.003133	0.004524	0	0	0.005909	0.00487	0.004333	0.001993	0.000657	0.002203	0.000466	0.003233978
Regulation and Cell signaling	NULL	cAMP signaling in bacteria	CAMP phosphodiesterases class-II:Metallo-beta-lactamase superfamily	0.000348	0.002262	0.007664	0	0.00197	0.000487	0.001444	0.000498	0.001314	0.004406	0	0.002366325
Cell Wall and Capsule	Capsular and extracellular polysacchrides	Capsular Polysaccharide (CPS) of Campylobacter	Capsular polysaccharide ABC transporter, ATP-binding protein KpsT	0.001393	0	0.00511	0	0.001313	0.002435	0.002166	0.005482	0.005036	0.002203	0.015835	0.001735305
Cell Wall and Capsule	Capsular and extracellular polysacchrides	Exopolysaccharide Biosynthesis	Capsular polysaccharide synthesis enzyme CpsD, exopolysaccharide synthesis	0.00383	0.002262	0	0.00498	0.002626	0.003896	0.000722	0.003489	0.001752	0	0.000233	0.00252408
Nucleosides and Nucleotides	Pyrimidines	De Novo Pyrimidine Synthesis	Carbamoyl-phosphate synthase large chain A (EC 6.3.5.5)	0.000696	0.002262	0	0.00747	0.004596	0.000974	0.004333	0.00299	0.001533	0.002203	0	0.001932499
Respiration	NULL	Carbon monoxide dehydrogenase maturation factors	Carbon monoxide dehydrogenase F protein	0.012534	0.004524	0.017884	0.00249	0.003283	0.007793	0.008665	0.006479	0.01248	0	0	0.004456579
Respiration	NULL	Carbon monoxide dehydrogenase maturation factors	carbon monoxide dehydrogenase G protein	0.007659	0.004524	0.002555	0	0.007222	0.001948	0.006499	0.009967	0.005912	0	0.000349	0.000985969
Stress Response	NULL	Carbon Starvation	Carbon storage regulator	0.009748	0.002262	0.015329	0.01245	0.015101	0.017533	0.018052	0.017443	0.006568	0	0	0.006704588
Carbohydrates	CO2 fixation	CO2 uptake, carboxysome	carboxysome shell protein CsoS3	0.004526	0.002262	0.00511	0.00747	0.009848	0.007793	0.008665	0	0.000657	0.011014	0	0.010924535
Cofactors, Vitamins, Prosthetic Groups, Pigments	Folate and pterines	p-Aminobenzoyl-Glutamate Utilization	Catalyzes the cleavage of p-aminobenzoyl-glutamate to p-aminobenzoate and glutamate, subunit B	0.002089	0	0.002555	0.00996	0.002626	0.002922	0.005055	0.001993	0.002627	0.002203	0	0.002760713
Metabolism of Aromatic Compounds	Metabolism of central aromatic intermediates	Catechol branch of beta-ketoadipate pathway	Catechol 1,2-dioxygenase (EC 1.13.11.1)	0.001741	0	0.002555	0	0.000657	0.000487	0.002166	0.000498	0.005255	0.004406	0.000582	0.000354949
Cell Wall and Capsule	NULL	YjeE	CBS domain protein sometimes clustered with YjeE	0.002089	0.004524	0	0.00249	0.004596	0.009254	0.006499	0	0.001314	0.004406	0.000931	0.003864998
Clustering-based subsystems	NULL	Bacterial Cell Division	Cell division protein FtsQ	0.009052	0	0.010219	0.00249	0.000657	0.005844	0.002888	0.005482	0.013356	0	0.013506	0.005245354
Miscellaneous	NULL	ZZ gjo need homes	Cephalosporin hydroxylase	0.004526	0.002262	0	0.00498	0.00197	0.003409	0.008665	0.000498	0.001971	0	0.000699	0.002050815
RNA Metabolism	RNA processing and modification	tRNA modification Bacteria	Chaperone protein HscB	0.001044	0.006786	0.00511	0.00498	0	0.002922	0.002888	0.000498	0.000219	0	0.000116	0.002957907
Clustering-based subsystems	Chemotaxis, response regulators	CBSS-323850.3.peg.3142	Chemotaxis protein CheC -- inhibitor of MCP methylation	0.005919	0	0.002555	0	0.009848	0.005844	0.002888	0.00598	0.01248	0.004406	0.033649	0.009149791
Metabolism of Aromatic Compounds	Peripheral pathways for catabolism of aromatic compounds	Phenylpropanoid compound degradation	Chlorogenate esterase	0.001741	0	0.00511	0.00498	0.002626	0.004383	0.00361	0.001495	0.003503	0	0.000116	0.003391733
Cell Wall and Capsule	Capsular and extracellular polysacchrides	Phosphorylcholine incorporation in LPS	Choline kinase (EC 2.7.1.32)	0.002089	0	0	0.00498	0.003939	0.002922	0.002166	0.002492	0.005693	0.002203	0.000466	0.000867653
Amino Acids and Derivatives	Aromatic amino acids and derivatives	Chorismate Synthesis	Chorismate mutase II (EC 5.4.99.5)	0	0	0.002555	0.00498	0.005909	0.003896	0.004333	0.001495	0.001533	0.002203	0.000699	0.000709898
Fatty Acids, Lipids, and Isoprenoids	Isoprenoids	Acyclic terpenes utilization	Citronellol and citronellal dehydrogenase	0.003133	0	0	0.00498	0.00197	0.000974	0.002888	0.00299	0.005255	0.002203	0.000116	0.000985969
Respiration	NULL	Carbon monoxide induced hydrogenase	CO dehydrogenase/acetyl-CoA synthase, acetyl-CoA synthase subunit (EC 2.3.1.169)	0.028549	0.067859	0.043432	0.059761	0.051211	0.048217	0.049102	0.004485	0.005474	0	0	0.028671975
Respiration	NULL	Carbon monoxide induced hydrogenase	CO dehydrogenase/acetyl-CoA synthase, CO dehydrogenase subunit (EC 1.2.99.2)	0.027853	0.05655	0.038322	0.037351	0.05712	0.056984	0.016608	0.009469	0.005036	0	0	0.030170648
Cofactors, Vitamins, Prosthetic Groups, Pigments	Tetrapyrroles	Coenzyme B12 biosynthesis	Cob(II)alamin reductase	0.003482	0.002262	0.007664	0.01245	0.006566	0.004383	0.001444	0.001993	0.002189	0	0	0.002918468
Cofactors, Vitamins, Prosthetic Groups, Pigments	Tetrapyrroles	Cobalamin synthesis	Cobalamin biosynthesis protein CbiG	0.002437	0	0.002555	0	0.005252	0.00487	0.009387	0.006977	0.002846	0.008811	0.000233	0.002642397
Virulence, Disease and Defense	Resistance to antibiotics and toxic compounds	Cobalt-zinc-cadmium resistance	Cobalt/zinc/cadmium efflux RND transporter, membrane fusion protein, CzcB family	0.008008	0.009048	0	0.00747	0.012475	0.010228	0.010831	0.009967	0.01248	0	0.017232	0.011200606
Miscellaneous	Plant-Prokaryote DOE project	COG0523	Cobalt-containing nitrile hydratase subunit alpha (EC 4.2.1.84)	0	0.004524	0.007664	0	0.003283	0.008767	0.001444	0.004485	0.01029	0.004406	0.000466	0.000867653
Miscellaneous	Plant-Prokaryote DOE project	COG0523	Cobalt-containing nitrile hydratase subunit beta (EC 4.2.1.84)	0	0	0.00511	0.00249	0.00197	0.002435	0.000722	0.000997	0.001314	0.002203	0.000349	0.000433826
Cofactors, Vitamins, Prosthetic Groups, Pigments	Tetrapyrroles	Coenzyme B12 biosynthesis	Cobalt-precorrin-6 synthase, anaerobic	0	0.002262	0.007664	0.01494	0.009848	0.01315	0.008665	0.011961	0.008539	0	0.002445	0.003864998
Cofactors, Vitamins, Prosthetic Groups, Pigments	Tetrapyrroles	Cobalamin synthesis	Cobalt-precorrin-6x reductase (EC 1.3.1.54)	0.002089	0	0.00511	0.00498	0.002626	0.002435	0.001444	0.002492	0.001095	0	0.000582	0.00031551
Respiration	Electron accepting reactions	Anaerobic respiratory reductases	CoB--CoM heterodisulfide reductase 2 iron-sulfur subunit D (EC 1.8.98.1)	0.010445	0.006786	0.002555	0.01992	0.011818	0.006819	0.006499	0.000997	0.000438	0	0	0.005521426
Respiration	Electron donating reactions	H2:CoM-S-S-HTP oxidoreductase	CoB--CoM-reducing hydrogenase (Cys) alpha subunit	0.027504	0.013572	0.00511	0.02739	0.011818	0.010228	0.006499	0.002492	0.002408	0	0	0.007690557
Respiration	Electron donating reactions	Hydrogenases	Coenzyme F420-reducing hydrogenase, beta subunit	0.016712	0.01131	0.010219	0.01494	0.009848	0.023865	0.006499	0.006479	0.000876	0	0	0.007572241
Clustering-based subsystems	NULL	CBSS-350688.3.peg.1509	COG2740: Predicted nucleic-acid-binding protein implicated in transcription termination	0.002785	0.004524	0.00511	0.00249	0	0.005357	0.002888	0.002492	0.002408	0	0.000466	0.001064846
Stress Response	Cold shock	Cold shock, CspA family of proteins	Cold shock protein CspG	0.014971	0.018096	0.012774	0	0.011818	0.01315	0.012275	0.009967	0.007006	0.002203	0	0.017786878
Membrane Transport	Protein and nucleoprotein secretion system, Type IV	Conjugative transfer	Conjugative transfer protein TrbE	0	0	0.007664	0.00747	0.003939	0.003409	0.00361	0.003987	0.002846	0.006608	0.000116	0.001735305
Respiration	NULL	Biogenesis of cytochrome c oxidases	Copper metallochaperone, bacterial analog of Cox17 protein	0	0.002262	0.015329	0.00249	0.004596	0.001948	0.002166	0.00598	0.005693	0	0.000699	0.003036784
Virulence, Disease and Defense	Resistance to antibiotics and toxic compounds	Copper homeostasis	Copper resistance protein B	0.002437	0	0	0.00249	0.003283	0.000974	0.002888	0.001495	0.005693	0.002203	0.000466	0.005284793
Amino Acids and Derivatives	NULL	Creatine and Creatinine Degradation	Creatinase (EC 3.5.3.3)	0.002785	0.002262	0	0	0.000657	0.003409	0.012275	0.012957	0.00832	0.008811	0.001164	0.001143724
DNA Metabolism	CRISPs	CRISPRs	CRISPR-associated protein Cas2	0	0.004524	0	0.00498	0.005909	0.003409	0.00361	0.003987	0.003503	0.002203	0.000349	0.002760713
DNA Metabolism	CRISPs	CRISPRs	CRISPR-associated protein, CT1975 family	0	0.009048	0.002555	0	0.015757	0.005844	0.004333	0.004984	0.002189	0.008811	0.080689	0.003312855
Nitrogen Metabolism	NULL	Cyanate hydrolysis	Cyanate ABC transporter, substrate binding protein	0.002437	0.004524	0	0.00249	0.002626	0.00828	0.005055	0.010964	0.008539	0	0.002911	0.003076223
Stress Response	NULL	Bacterial hemoglobins	Cyanoglobin	0.003133	0.004524	0.00511	0	0.002626	0.000487	0.004333	0.003987	0.01029	0	0.000233	0.004298824
Amino Acids and Derivatives	Lysine, threonine, methionine, and cysteine	Cysteine Biosynthesis	Cys regulon transcriptional activator CysB	0.029593	0	0.00511	0	0.004596	0.002922	0.008665	0.006479	0.015983	0.028636	0.043081	0.011358361
Virulence, Disease and Defense	Resistance to antibiotics and toxic compounds	Copper homeostasis	Cytochrome c heme lyase subunit CcmH	0.002785	0	0.010219	0	0.005252	0.002435	0.002888	0.00598	0.007225	0.002203	0.013972	0.005363671
Respiration	Electron accepting reactions	Terminal cytochrome C oxidases	Cytochrome c oxidase (B(O/a)3-type) chain I (EC 1.9.3.1)	0.004178	0	0.010219	0.00747	0.02101	0.016559	0.021663	0.013456	0.01029	0.002203	0	0.004022753

Respiration	NULL	Biogenesis of cytochrome c oxidases	Cytochrome oxidase biogenesis protein Surf1, facilitates heme A insertion	0.009052	0.002262	0.015329	0.00249	0.000657	0.002435	0.000722	0.003987	0.012261	0	0	0.002484642
Carbohydrates	One-carbon Metabolism	Serine-glyoxylate cycle	cytosolic long-chain acyl-CoA thioester hydrolase family protein	0.009052	0	0.00511	0.00747	0.001313	0.001461	0.00361	0.006479	0.011823	0	0.007452	0.005758058
Cell Wall and Capsule	NULL	Peptidoglycan biosynthesis--gjo	D-alanine--D-alanine ligase A (EC 6.3.2.4)	0.000696	0	0.002555	0.00498	0.00197	0.000974	0	0.000997	0.003065	0.004406	0.000349	0.00189306
Cell Wall and Capsule	NULL	Peptidoglycan biosynthesis--gjo	D-alanine--D-alanine ligase B (EC 6.3.2.4)	0.009748	0.002262	0	0.00249	0.001313	0.001948	0.002166	0.006977	0.006787	0	0.000815	0.004693212
Carbohydrates	One-carbon Metabolism	Formaldehyde assimilation: Ribulose monophosphate pathway	D-arabino-3-hexulose 6-phosphate formaldehyde lyase	0.003133	0.002262	0.002555	0.00249	0.00197	0	0.001444	0	0.000438	0.004406	0.000116	0.00189306
Fatty Acids, Lipids, and Isoprenoids	NULL	Polyhydroxybutyrate metabolism	D-beta-hydroxybutyrate permease	0.000348	0	0.002555	0.00249	0.001313	0.005844	0.002888	0.003987	0.008977	0	0.002329	0.001301479
Cofactors, Vitamins, Prosthetic Groups, Pigments	Pyridoxine	Pyridoxin (Vitamin B6) Biosynthesis	D-erythrose-4-phosphate dehydrogenase (EC 1.2.1.72)	0	0.002262	0.002555	0	0.00197	0.001948	0.007943	0.007974	0.015764	0.002203	0.053443	0.008755404
Carbohydrates	Monosaccharides	D-Galacturonate and D-Glucuronate Utilization	D-galactarate dehydratase (EC 4.2.1.42)	0.000696	0.002262	0.017884	0.00747	0	0.001461	0	0.001495	0.004817	0.004406	0.000466	0.002248009
Miscellaneous	Plant-Prokaryote DOE project	Niacin-Choline transport and metabolism	DgcA Dimethylglycine demethylase subunit A	0	0.004524	0.00511	0.00747	0.00197	0.000974	0.000722	0.000997	0.003284	0	0.109448	0.002129693
Miscellaneous	Plant-Prokaryote DOE project	Niacin-Choline transport and metabolism	DgcB Dimethylglycine demethylase subunit B	0.002785	0.002262	0.002555	0	0.00197	0.000487	0.001444	0.000997	0.001752	0	0.030156	0.00094653
Carbohydrates	Organic acids	Glycerate metabolism	D-glycerate 3-kinase (EC 2.7.1.31), plant type	0	0	0.00511	0.00498	0.00197	0.002435	0.004333	0.003489	0.005693	0.004406	0.000233	0.001656428
Carbohydrates	Monosaccharides	D-galactarate, D-glucarate and D-glycerate catabolism	D-glycerate transporter (predicted)	0.008008	0.01131	0	0.00498	0.002626	0.002922	0.000722	0.003489	0.003284	0	0.000116	0.004574896
Carbohydrates	Central carbohydrate metabolism	Dihydroxyacetone kinases	DHA-specific EI component	0.005571	0.004524	0	0.00249	0.001313	0.000974	0.001444	0.000498	0.001314	0.002203	0	0.001538111
Nucleosides and Nucleotides	NULL	Hydantoin metabolism	D-hydantoinase (EC 3.5.2.2)	0.003133	0	0.00511	0.00249	0.001313	0.002435	0.002888	0.004485	0.008758	0	0.001397	0.001932499
Fatty Acids, Lipids, and Isoprenoids	Phospholipids	Glycerolipid and Glycerophospholipid Metabolism in Bacteria	Dihydroxyacetone kinase family protein	0.010793	0.01131	0.017884	0.02241	0.003939	0.003896	0.00361	0.00299	0.002627	0	0	0.004850967
Carbohydrates	One-carbon Metabolism	Methanogenesis from methylated compounds	Dimethylamine methyltransferase corrinoid protein	0.001741	0.009048	0.007664	0	0.004596	0.002435	0.00361	0.000997	0.000438	0.006608	0	0.002800152
Fatty Acids, Lipids, and Isoprenoids	Isoprenoids	Archaeal lipids	Diphosphomevalonate decarboxylase (EC 4.1.1.33)	0.000348	0.002262	0.010219	0	0.000657	0.001948	0.00361	0.003987	0.001314	0	0.000116	0.013133105
Cofactors, Vitamins, Prosthetic Groups, Pigments	Tetrapyrroles	Chlorophyll Biosynthesis	Divinyl protochlorophyllide a 8-vinyl-reductase (EC 1.3.1.75)	0.003133	0	0.00511	0.00498	0.000657	0.003409	0.002888	0.004984	0.002189	0	0.001164	0.001064846
Sulfur Metabolism	Organic sulfur assimilation	DMSP breakdown	DMSP demethylase EC 2.1.2.10	0	0.004524	0	0.00498	0.003283	0.001948	0.006499	0.00598	0.008101	0.004406	0.088024	0.002326887
Phages, Prophages, Transposable elements, Plasmids	Phages, Prophages	Phage replication	DNA helicase, phage-associated	0	0.006786	0.002555	0	0.031515	0.049678	0.063543	0.075751	0.002408	0.006608	0.000699	0.002405764
DNA Metabolism	DNA replication	DNA-replication	DNA polymerase III chi subunit (EC 2.7.7.7)	0.004874	0.002262	0.002555	0	0.005909	0.005844	0.001444	0.010466	0.00832	0	0.000349	0.001971938
DNA Metabolism	DNA repair	DNA repair, bacterial	DNA recombination-dependent growth factor C	0.026808	0	0	0.00498	0.00197	0.000974	0.00361	0.001993	0.01248	0.008811	0.006404	0.004496018
DNA Metabolism	DNA replication	DNA topoisomerases, Type II, ATP-dependent	DNA topoisomerase VI subunit B (EC 5.99.1.3)	0.016712	0.009048	0.033212	0.01494	0.009848	0.012176	0.007221	0.002492	0.010072	0	0	0.006862343
DNA Metabolism	NULL	DNA structural proteins, bacterial	DNA-binding protein HU-alpha	0.000348	0	0	0.00996	0.003283	0.003409	0.002166	0.002492	0.001752	0.004406	0.000233	0.004456579
RNA Metabolism	Transcription	RNA polymerase archaeal	DNA-directed RNA polymerase subunit E' (EC 2.7.7.6)	0.002785	0.002262	0	0.00249	0.001313	0.001948	0.005777	0.00299	0.000438	0.002203	0	0.001380356
Fatty Acids, Lipids, and Isoprenoids	Isoprenoids	Dolichol metabolism	Dolichol-phosphate mannosyltransferase (EC 2.4.1.83)	0.002089	0.004524	0.010219	0	0.009848	0.006819	0.007943	0.005482	0.009853	0	0.000349	0.012817595
Miscellaneous	NULL	Broadly distributed proteins not in subsystems	DUF1022 domain-containing protein	0.004874	0.002262	0.007664	0.00249	0.005252	0.005357	0.007943	0.010466	0.005693	0	0	0.001735305
DNA Metabolism	DNA repair	DNA repair, bacterial UmuCD system	Error-prone repair protein UmuD	0	0	0.00511	0.00249	0.001313	0.001948	0.00361	0.00598	0.002627	0.002203	0.008267	0.00634964
Protein Metabolism	Protein biosynthesis	Translation elongation factors eukaryotic and archaeal	Eukaryotic peptide chain release factor subunit 1	0	0	0.002555	0.00498	0.014444	0.003409	0.006499	0.005482	0.002189	0.011014	0.000582	0.003510049
Clustering-based subsystems	Proteasome related clusters	Proteasome subunit alpha archaeal cluster	Exosome complex exonuclease 1	0.000348	0.009048	0.002555	0	0.003283	0.005844	0.008665	0.000498	0	0.002203	0.000233	0.001419795
Nitrogen Metabolism	NULL	Nitrosative stress	Ferredoxin 3 fused to uncharacterized domain	0.010097	0.024882	0.00511	0	0.013788	0.01315	0.004333	0.005482	0.000219	0.006608	0	0.00698066
Sulfur Metabolism	Inorganic sulfur assimilation	Inorganic Sulfur Assimilation	Ferredoxin--NADP(+) reductase (EC 1.18.1.2)	0.004874	0	0.002555	0	0.001313	0.001948	0.00361	0.004485	0.00832	0.002203	0.026896	0.003510049
Iron acquisition and metabolism	NULL	Encapsulating protein for DyP-type peroxidase and ferritin-like protein oligomers	Ferritin-like di-iron-carboxylate protein	0.000696	0.01131	0.00511	0.00249	0.003283	0.001461	0.000722	0.000498	0	0	0.000466	0.002090254
Miscellaneous	Plant-Prokaryote DOE project	Iron-sulfur cluster assembly	Ferritin-like protein 2	0	0.009048	0.007664	0.00249	0.005909	0.009741	0.005777	0.004485	0.001971	0	0.000349	0.016248767
Stress Response	Oxidative stress	Oxidative stress	Ferroxidase (EC 1.16.3.1)	0	0	0.002555	0.00249	0.00197	0.001948	0.00361	0.007974	0.007444	0.002203	0.00652	0.002011377
Metabolism of Aromatic Compounds	Peripheral pathways for catabolism of aromatic compounds	Phenylpropanoid compound degradation	Feruloyl-CoA synthetase	0.003133	0.002262	0.012774	0	0.005909	0.001461	0.005055	0.00598	0.009196	0	0.042848	0.003312855
Respiration	Electron donating reactions	Hydrogenases	Fe-S-cluster-containing hydrogenase components 2	0.007659	0.009048	0.007664	0.00498	0.008535	0.010228	0.004333	0.001495	0.001095	0	0	0.002169132
Virulence, Disease and Defense	Adhesion	Adhesion of Campylobacter	Fibronectin/fibrinogen-binding protein	0.001044	0.01131	0.002555	0.00498	0.005252	0.003896	0.002888	0.006479	0.00416	0	0	0.00761168
Clustering-based subsystems	alpha-proteobacterial cluster of hypotheticals	CBSS-52598.3.peg.2843	FIG003603: membrane protein, putative	0.005919	0	0.012774	0.01245	0.008535	0.007793	0.01372	0.021928	0.013137	0	0.000466	0.001932499
Clustering-based subsystems	proteosome related	Cluster-based Subsystem Grouping Hypotheticals - perhaps Proteosome Related	FIG005666: putative helicase	0	0.013572	0.007664	0.00747	0	0.001461	0.000722	0.001495	0.000219	0.004406	0.000931	0.001380356
Clustering-based subsystems	NULL	CBSS-316273.3.peg.2378	FIG006126: DNA helicase, restriction/modification system component YeeB	0.002437	0.009048	0	0.00747	0.002626	0.002922	0.005055	0.001495	0.005474	0.059475	0	0.002287448
Fatty Acids, Lipids, and Isoprenoids	Fatty acids	Phospholipid and Fatty acid biosynthesis related cluster	FIG022199: FAD-binding protein	0.004874	0	0.002555	0.00498	0.000657	0.000974	0.000722	0.001993	0.00832	0	0.000931	0.003864998
Iron acquisition and metabolism	NULL	Heme, hemin uptake and utilization systems in GramPositives	FIG039061: hypothetical protein related to heme utilization	0	0.002262	0.002555	0.00498	0.005252	0.004383	0.007221	0.003489	0.007444	0	0.006055	0.003115662
Miscellaneous	Plant-Prokaryote DOE project	COG3760	FIG042921: similarity to aminoacyl-tRNA editing enzymes YbaK, ProX	0.001741	0.004524	0.002555	0.00498	0	0.004383	0.002166	0.003987	0.001971	0	0.000116	0.001104285
Clustering-based subsystems	NULL	Cell wall related cluster	FIG123464: Polysaccharide export protein	0.008008	0.01131	0	0.00498	0.005909	0.007306	0.006499	0.006977	0.008977	0	0.002096	0.007651118
Clustering-based subsystems	Lysine Biosynthesis	A Glutathione-dependent Thiol Reductase Associated with a Step in Lysine Biosynthesis	FIG138056: a glutathione-dependent thiol reductase	0.002785	0	0	0.01245	0.001313	0.001461	0.004333	0.003489	0.002408	0.004406	0.012109	0.004772089
Fatty Acids, Lipids, and Isoprenoids	Fatty acids	Phospholipid and Fatty acid biosynthesis related cluster	FIG138576: 3-oxoacyl-[ACP] synthase (EC 2.3.1.41)	0.002785	0.002262	0.002555	0	0.00197	0.003896	0.002166	0.001495	0.003503	0	0.000233	0.001814183
Clustering-based subsystems	NULL	CBSS-211586.1.peg.3133	FIG139438: lipoprotein B	0.00383	0.002262	0.007664	0	0.006566	0.004383	0.001444	0.00299	0.002189	0	0.007685	0.005008722
Clustering-based subsystems	Fatty acid metabolic cluster	COG1399	FIG146278: Maf/YceF/YhdE family protein	0.036208	0.004524	0	0.00747	0.002626	0.005357	0.00361	0.006479	0.007225	0	0.014321	0.009583617
Protein Metabolism	Protein folding	Peptidyl-prolyl cis-trans isomerase	FKBP-type peptidyl-prolyl cis-trans isomerase slpA (EC 5.2.1.8)	0	0.002262	0.002555	0	0.000657	0.003409	0.002166	0.001993	0.007225	0.002203	0.027944	0.004850967
Motility and Chemotaxis	Flagellar motility in Prokaryota	Flagellum	Flagellar biosynthesis protein FliQ	0	0.004524	0.010219	0.01992	0.013788	0.007306	0.010109	0.021928	0.00832	0	0.00978	0.007848312
Motility and Chemotaxis	Flagellar motility in Prokaryota	Flagellum in Campylobacter	Flagellar hook-basal body complex protein FliE	0.002785	0	0.002555	0	0.006566	0.005844	0.008665	0.004485	0.005912	0.004406	0.009897	0.006704588
DNA Metabolism	DNA repair	DNA Repair Base Excision	Flap structure-specific endonuclease (EC 3.-.-.-)	0	0	0.017884	0.00498	0.007879	0.006819	0.005055	0.003987	0.002189	0.011014	0.000233	0.004062192
Respiration	Electron donating reactions	Formate dehydrogenase	Formate dehydrogenase H (EC 1.2.1.2)	0.002785	0.002262	0.002555	0.00249	0.004596	0.003896	0.002166	0.000498	0.001314	0	0	0.003352294

Virulence, Disease and Defense	NULL	Bacterial cyanide production and tolerance mechanisms	Formate dehydrogenase O gamma subunit (EC 1.2.1.2)	0.001044	0.002262	0	0.00498	0.001313	0.000487	0.000722	0.000997	0.000438	0	0.000233	0.00094653
Respiration	NULL	Formate hydrogenase	Formate dehydrogenase -O, gamma subunit (EC 1.2.1.2)	0.007311	0	0.002555	0.00249	0.00197	0.003896	0.005055	0.008472	0.011604	0	0.000233	0.002997345
Respiration	NULL	Formate hydrogenase	Formate hydrogenlyase subunit 4	0.008704	0.002262	0.012774	0.00249	0.00197	0.000487	0	0.000498	0.008539	0.004406	0	0.003588927
Carbohydrates	Monosaccharides	Fructose utilization	Fructose ABC transporter, ATP-binding component FrcA	0.003133	0.009048	0	0.00498	0	0.000974	0.000722	0.001993	0.005912	0.002203	0.030971	0.001735305
Carbohydrates	Monosaccharides	Fructose utilization	Fructose ABC transporter, substrate-binding component FrcB	0.001044	0.006786	0	0.00498	0	0.003409	0.001444	0.001993	0.005474	0.011014	0.02317	0.001814183
Carbohydrates	Central carbohydrate metabolism	Glycolysis and Gluconeogenesis, including Archaeal enzymes	Fructose-1,6-bisphosphatase, type V, archaeal (EC 3.1.3.11)	0.009748	0.009048	0	0.00996	0.012475	0.011202	0.009387	0.000498	0.000657	0	0.000931	0.005442548
Carbohydrates	Central carbohydrate metabolism	Glycolysis and Gluconeogenesis	Fructose-bisphosphate aldolase, archaeal class I (EC 4.1.2.13)	0.002785	0.004524	0	0	0.00197	0.001948	0.002888	0.000997	0.001314	0.004406	0.000233	0.001498673
Carbohydrates	Central carbohydrate metabolism	TCA Cycle	Fumarate hydratase class I (EC 4.2.1.2)	0.002785	0.009048	0.010219	0.00498	0.003283	0.000974	0.002166	0.002492	0.001095	0	0	0.004377702
Respiration	Electron donating reactions	Succinate dehydrogenase	Fumarate reductase cytochrome b subunit	0.000696	0.004524	0.00511	0.00498	0.000657	0.001461	0	0.000498	0.001752	0.004406	0	0.005403109
Clustering-based subsystems	Putative GGDEF domain protein related to agglutinin secretion	CBSS-323850.3.peg.3284	GAF domain/GGDEF domain/EAL domain protein	0.002785	0	0.002555	0	0.001313	0.000974	0.001444	0.005482	0.009634	0.013217	0.000349	0.005324232
Amino Acids and Derivatives	Arginine; urea cycle, polyamines	Putrescine utilization pathways	Gamma-glutamyl-aminobutyraldehyde dehydrogenase (EC 1.2.1.-)	0.000696	0.002262	0	0.00249	0	0.000974	0.002166	0.000498	0.003503	0.002203	0.011411	0.002366325
Clustering-based subsystems	Putrescine/GABA utilization cluster-temporal,to add to SSs	GABA and putrescine metabolism from cluters	Gamma-glutamyl-putrescine synthetase (EC 6.3.1.11)	0	0	0.002555	0.00498	0.002626	0.003409	0.006499	0.004485	0.003941	0.002203	0.002212	0.00126204
Phages, Prophages, Transposable elements, Plasmids	Gene Transfer Agent (GTA)	Gene Transfer Agent	Gene Transfer Agent capsid protein	0	0	0.002555	0.00747	0.00197	0.003896	0.002888	0.009469	0.002627	0.002203	0.000233	0.000512704
Membrane Transport	Protein secretion system, Type II	General Secretion Pathway	General secretion pathway protein C	0.003482	0	0.002555	0	0.002626	0.001461	0.001444	0.003489	0.006131	0.002203	0.000233	0.002366325
Membrane Transport	Protein secretion system, Type II	General Secretion Pathway	General secretion pathway protein K	0.002437	0.004524	0.012774	0	0.003939	0.002922	0.002166	0.003489	0.009196	0	0.000116	0.002602958
Metabolism of Aromatic Compounds	NULL	Gentisare degradation	Gentisate 1,2-dioxygenase (EC 1.13.11.4)	0	0	0.002555	0.00498	0.003283	0.006332	0.007221	0.007475	0.008101	0.004406	0.000582	0.001971938
Fatty Acids, Lipids, and Isoprenoids	Isoprenoids	Acyclic terpenes utilization	Geranyl-CoA carboxylase carboxyl transferase subunit	0.000696	0	0.015329	0.00249	0.001313	0.003896	0.010109	0.009469	0.012699	0.008811	0	0.001616989
Fatty Acids, Lipids, and Isoprenoids	Isoprenoids	Polyprenyl Diphosphate Biosynthesis	Geranylgeranyl reductase (EC 1.3.1.83)	0	0.002262	0.007664	0.00498	0.005252	0.004383	0.006499	0.004485	0.007444	0	0.000815	0.001538111
Stress Response	Osmotic stress	Synthesis of osmoregulated periplasmic glucans	Glucans biosynthesis protein G precursor	0.001393	0.006786	0	0.01494	0.003283	0.001461	0.004333	0.003489	0.008758	0	0.002562	0.004614334
Carbohydrates	Di- and oligosaccharides	Beta-Glucoside Metabolism	Glucokinase, ROK family (EC 2.7.1.2)	0.000348	0.004524	0.002555	0	0.000657	0.001461	0.005777	0.000498	0.002189	0	0.000466	0.003746682
Carbohydrates	Monosaccharides	D-gluconate and ketogluconates metabolism	Gluconate permease	0.013578	0.002262	0.00511	0.00249	0.000657	0.001948	0.000722	0.001993	0.005474	0	0	0.001104285
Respiration	Electron donating reactions	Respiratory dehydrogenases 1	Glycerol dehydrogenase (EC 1.1.1.6)	0.003482	0.009048	0.007664	0	0.003939	0.007306	0.001444	0.002492	0.004598	0	0.000815	0.003036784
Cell Wall and Capsule	Capsular and extracellular polysacchrides	Rhamnose containing glycans	Glycerol-3-phosphate cytidylyltransferase (EC 2.7.7.39)	0	0.002262	0	0.00498	0.001313	0.000974	0.002166	0.000997	0.000657	0.002203	0.002329	0.002681835
Stress Response	Osmotic stress	Choline and Betaine Uptake and Betaine Biosynthesis	Glycine betaine ABC transport system, ATP-binding protein OpuAA (EC 3.6.3.32)	0.003482	0	0.007664	0	0.003939	0.006819	0.005055	0.013954	0.006787	0.002203	0.017582	0.004062192
Miscellaneous	Plant-Prokaryote DOE project	Niacin-Choline transport and metabolism	Glycine betaine transporter OpuD	0.002089	0.004524	0	0	0.009192	0.006332	0.00361	0.011462	0.004379	0.002203	0.001164	0.002957907
Regulation and Cell signaling	NULL	Orphan regulatory proteins	Glycine cleavage system transcriptional activator GcvA	0.002785	0	0	0.00498	0.003939	0.005844	0.007221	0.005482	0.013356	0.004406	0.00489	0.001814183
Regulation and Cell signaling	NULL	Orphan regulatory proteins	Glycine cleavage system transcriptional antiactivator GcvR	0.006615	0.01131	0.002555	0.00498	0.007879	0.010715	0.005055	0.00299	0.001971	0	0	0.005363671
Nucleosides and Nucleotides	Purines	Purine conversions	GMP reductase	0.000696	0.002262	0.00511	0.00747	0.002626	0	0.006499	0.004485	0.003503	0	0.000466	0.008637087
Nucleosides and Nucleotides	Purines	Purine conversions	GMP reductase (EC 1.7.1.7)	0	0.002262	0.00511	0.00249	0	0.003409	0.007221	0.00598	0.002627	0.002203	0.000349	0.007729996
Cell Division and Cell Cycle	NULL	Cyanobacterial Circadian Clock	Group 2 RNA polymerase sigma factor	0.002437	0.002262	0.002555	0	0.003939	0.002435	0.002166	0.000997	0.001752	0	0.001048	0.000709898
Nucleosides and Nucleotides	Pyrimidines	Novel non-oxidative pathway of Uracil catabolism	GTP cyclohydrolase II homolog	0.003133	0.002262	0.00511	0.00996	0	0.001461	0.001444	0.00299	0.005036	0.004406	0	0.000512704
Miscellaneous	NULL	ZZ gjo need homes	GTP-binding protein RBG1	0	0.002262	0.00511	0	0.00197	0.000974	0.002888	0.003489	0.000876	0.002203	0.000582	0.001340918
DNA Metabolism	DNA replication	DNA-replication	Helicase loader DnaI	0.004526	0.013572	0.007664	0.01245	0.007222	0.002922	0.002166	0	0.000657	0.002203	0	0.003194539
Cofactors, Vitamins, Prosthetic Groups, Pigments	Tetrapyrroles	Heme biosynthesis orphans	Heme d1 biosynthesis protein NirJ	0	0.006786	0.012774	0.00747	0.005252	0.005357	0.006499	0.00299	0.005912	0	0.000699	0.010254076
Cofactors, Vitamins, Prosthetic Groups, Pigments	Tetrapyrroles	Heme biosynthesis orphans	Heme d1 biosynthesis protein NirL	0.000696	0.004524	0.002555	0	0.005252	0.002435	0.005055	0.001993	0.001314	0	0.000349	0.001222601
Iron acquisition and metabolism	NULL	Heme, hemin uptake and utilization systems in GramPositives	Heme-degrading monooxygenase IsdG (EC 1.14.99.3)	0.002785	0	0.002555	0.00747	0.00197	0.002922	0.007221	0.001993	0.002189	0	0.00687	0.002287448
Virulence, Disease and Defense	NULL	Streptococcus agalactiae virulome	Hemolysin III	0.002785	0.002262	0.002555	0	0.000657	0.001948	0.000722	0.003987	0.003503	0.002203	0	0.002681835
Respiration	Electron accepting reactions	Anaerobic respiratory reductases	heterodisulfide reductase, subunit E, putative	0.004526	0.009048	0.007664	0.00747	0.015101	0.017046	0.012998	0.005482	0.001533	0	0	0.006822905
Carbohydrates	CO2 fixation	CO2 uptake, carboxysome	High-affnity carbon uptake protein Hat/HatR	0.003482	0.006786	0.002555	0	0.008535	0.006819	0.00361	0.003987	0.021238	0	0.000466	0.006152446
Amino Acids and Derivatives	Arginine; urea cycle, polyamines	Arginine and Ornithine Degradation	Histidine ABC transporter, permease protein HisM (TC 3.A.1.3.1)	0.000348	0.002262	0.002555	0	0.001313	0.002922	0.004333	0.001495	0.007006	0	0.001281	0.001656428
Amino Acids and Derivatives	Lysine, threonine, methionine, and cysteine	Lysine biosynthesis AAA pathway 2	Homoaconitase (EC 4.2.1.36)	0.004178	0.002262	0.002555	0.00249	0.00197	0.002922	0.002888	0.000997	0.000438	0	0	0.002800152
Nucleosides and Nucleotides	NULL	Hydantoin metabolism	hydantoin racemase	0.001044	0.002262	0	0.00249	0	0.005357	0.007943	0.005482	0.003941	0.002203	0.007568	0.003707243
Nucleosides and Nucleotides	NULL	Hydantoin metabolism	Hydantoinase	0.005222	0.002262	0.002555	0	0.009192	0.012176	0.007943	0.009469	0.002408	0	0.001164	0.003233978
Respiration	Electron donating reactions	Hydrogenases	hydrogenase/sulfur reductase, alpha subunit	0.005571	0.002262	0.012774	0.01494	0.009848	0.01315	0.015886	0.006479	0.009196	0	0	0.008479332
Regulation and Cell signaling	NULL	cAMP signaling in bacteria	Hydrolase, alpha/beta fold family functionally coupled to Phosphoribulokinase	0.008704	0.004524	0.002555	0.00498	0	0.005844	0.006499	0.00598	0.004598	0	0.011993	0.005481987
Fatty Acids, Lipids, and Isoprenoids	Isoprenoids	Carotenoids	Hydroxyneurosporene methyltransferase (EC 2.1.1.-)	0.002785	0.006786	0	0.00996	0.003283	0.012663	0.007221	0.007475	0.005693	0	0.000582	0.000354949
Miscellaneous	Plant-Prokaryote DOE project	COG3533	Hypothetical glycoside hydrolase, family 43, similar to arabinosidase	0	0.015834	0.00511	0.00249	0.001313	0.002435	0.002166	0.000498	0.002846	0	0.00524	0.002760713
Cofactors, Vitamins, Prosthetic Groups, Pigments	NAD and NADP	NAD regulation	Hypothetical Nudix-like regulator	0.001393	0.006786	0.00511	0	0.003283	0.001461	0.005777	0.006977	0.011823	0	0.000233	0.014079636
Membrane Transport	Protein secretion system, Type VI	Type VI secretion systems	IcmF-related protein	0.009052	0	0.007664	0.00498	0.007222	0.007793	0.004333	0.006977	0.015326	0.004406	0	0.007769435
Amino Acids and Derivatives	Aromatic amino acids and derivatives	Aromatic amino acid degradation	Indoleacetamide hydrolase (EC 3.5.1.-)	0.004526	0	0	0.00249	0.001313	0.000974	0.000722	0.003489	0.002189	0.002203	0.000582	0.001340918
Clustering-based subsystems	NULL	Cluster with second YidC in Bacilli	Inner membrane protein translocase component YidC, short form OxaI-like	0.001393	0.002262	0	0.00996	0.002626	0.001948	0.002888	0.005482	0.002627	0	0.000815	0.001538111
Phages, Prophages, Transposable elements, Plasmids	NULL	Integrons	Integron integrase IntIPac	0.002089	0.018096	0.012774	0.01992	0.015101	0.010715	0.012275	0.010466	0.00832	0	0	0.008992036
Virulence, Disease and Defense	Invasion and intracellular resistance	Listeria surface proteins: Internalin-like proteins	internalin, putative	0.004526	0.020358	0.007664	0.00498	0.008535	0.009254	0.007221	0.018938	0.011823	0	0	0.010569586
Cell Division and Cell Cycle	NULL	Intracellular septation in Enterobacteria	Intracellular septation protein IspA	0.005919	0.002262	0	0.00747	0.000657	0.003896	0.002166	0.000498	0.005036	0	0.015719	0.006389078
Carbohydrates	NULL	Sugar utilization in Thermotogales	Iron-containing alcohol dehydrogenase	0	0.01131	0.00511	0.00747	0.004596	0.003409	0.007221	0.007974	0.003722	0	0.000815	0.00918923
Potassium metabolism	NULL	Potassium homeostasis	Kef-type K+ transport systems (NAD-binding component fused to domain related to exopolyphosphatase)	0.003133	0.004524	0	0.00249	0.012475	0.010715	0.008665	0.001495	0.005474	0	0.000349	0.003628365

Amino Acids and Derivatives	Branched-chain amino acids	Ketoisovalerate oxidoreductase	Ketoisovalerate oxidoreductase subunit VorA (EC 1.2.7.7)	0.00383	0.01131	0.00511	0.01494	0.013788	0.007793	0.002166	0.003987	0.003722	0	0	0.004929844
Cofactors, Vitamins, Prosthetic Groups, Pigments	NAD and NADP	NAD and NADP cofactor biosynthesis global	Kynurenine 3-monooxygenase (EC 1.14.13.9)	0.008356	0.002262	0	0.00498	0.003939	0.00487	0.008665	0.012459	0.012699	0	0.007801	0.004890406
Cofactors, Vitamins, Prosthetic Groups, Pigments	Coenzyme F420	Coenzyme F420 synthesis	Lactyl (2) diphospho-(5')guanosine:7,8-didemethyl-8-hydroxy-5-deazariboflavin 2-phospho-L-lactate transferase	0.001393	0.004524	0	0.00249	0.007222	0.001948	0.002888	0.005482	0.005912	0.006608	0	0.002760713
Cell Wall and Capsule	NULL	Recycling of Peptidoglycan Amino Acids	L-alanine-DL-glutamate epimerase	0.006267	0.006786	0.010219	0	0.014444	0.022404	0.015164	0.010466	0.015764	0	0.000582	0.005403109
Amino Acids and Derivatives	Lysine, threonine, methionine, and cysteine	Threonine degradation	L-allo-threonine aldolase (EC 2.1.2.1)	0	0.002262	0.002555	0.00249	0.001313	0.000974	0	0.001993	0.002846	0.013217	0.001048	0.00666515
Amino Acids and Derivatives	Glutamine, glutamate, aspartate, asparagine; ammonia assimilation	Glutamine, Glutamate, Aspartate and Asparagine Biosynthesis	L-asparaginase I, cytoplasmic (EC 3.5.1.1)	0	0.006786	0	0.01245	0.000657	0.007306	0.004333	0.007974	0.015326	0.017622	0.030389	0.02311111
Cofactors, Vitamins, Prosthetic Groups, Pigments	Tetrapyrroles	Chlorophyll Biosynthesis	Light-independent protochlorophyllide reductase iron-sulfur ATP-binding protein ChlL (EC 1.18.-.-)	0	0.01131	0.00511	0.00249	0.003939	0.010715	0.008665	0.010964	0.013137	0	0.003726	0.000709898
Clustering-based subsystems	NULL	Cell wall related cluster	Lipopolysaccharide biosynthesis chain length determinant protein	0.005919	0.009048	0.002555	0.00498	0.006566	0.005844	0.005777	0.003987	0.009196	0	0	0.005797497
Cell Wall and Capsule	Capsular and extracellular polysacchrides	Colanic acid biosynthesis	Lipopolysaccharide biosynthesis protein WzxC	0.001044	0.002262	0	0.00249	0.00197	0.001461	0.000722	0.000997	0.002189	0	0.000349	0.002248009
Cell Wall and Capsule	Gram-Negative cell wall components	Vibrio Core Oligosaccharide Biosynthesis	Lipopolysaccharide heptosyltransferase I (EC 2.4.1.-)	0.002785	0.002262	0.007664	0	0.005909	0.003896	0.010831	0.009967	0.003065	0	0.013273	0.006704588
Fatty Acids, Lipids, and Isoprenoids	Fatty acids	Fatty acid degradation regulons	Long-chain fatty acid transport protein	0.012186	0.002262	0.002555	0	0.007879	0.003409	0.004333	0.003489	0.017078	0	0.002911	0.007375047
Regulation and Cell signaling	Programmed Cell Death and Toxin-antitoxin Systems	Murein hydrolase regulation and cell death	LrgA-associated membrane protein LrgB	0	0	0.012774	0.01743	0.00197	0.007793	0.004333	0.003987	0.009196	0.002203	0.009664	0.005245354
Carbohydrates	Monosaccharides	L-rhamnose utilization	L-rhamnose mutarotase	0.000696	0.004524	0.020438	0.00249	0.003939	0.002435	0.000722	0.003987	0.003941	0	0	0.003549488
Protein Metabolism	Protein biosynthesis	Ribosome LSU bacterial	LSU ribosomal protein L30p (L7e)	0.007659	0.01131	0	0.01245	0.012475	0.009254	0.018774	0.014452	0.015107	0	0.000116	0.010963974
Protein Metabolism	Protein biosynthesis	Ribosome LSU bacterial	LSU ribosomal protein L33p	0.032379	0.018096	0.012774	0.01992	0.00197	0.008767	0.010831	0.016944	0.015107	0	0	0.015578308
Miscellaneous	Plant-Prokaryote DOE project	COG0523	LSU ribosomal protein L36p	0.041431	0.009048	0.002555	0.00498	0.004596	0.00487	0.011553	0.007475	0.005474	0	0	0.005324232
Fatty Acids, Lipids, and Isoprenoids	Fatty acids	Phospholipid and Fatty acid biosynthesis related cluster	Lysophospholipid acyltransferase	0.004178	0.004524	0.002555	0.00249	0.000657	0.001461	0.001444	0.000498	0.000657	0	0	0.001538111
Regulation and Cell signaling	NULL	DNA-binding regulatory proteins, strays	LysR family transcriptional regulator PA5437	0.000348	0.004524	0.002555	0.00249	0	0.000487	0.002166	0.001993	0.000219	0	0.000116	0.004377702
Clustering-based subsystems	Putative asociate of RNA polymerase sigma-54 factor rpoN	CBSS-316057.3.peg.1308	macromolecule synthesis, modification	0	0.002262	0.002555	0.00249	0.000657	0.002922	0.005777	0.00598	0.003722	0	0.000349	0.001656428
Carbohydrates	Central carbohydrate metabolism	Pyruvate metabolism I: anaplerotic reactions, PEP	Malate Na(+) symporter	0.004874	0.002262	0.007664	0	0.005909	0.004383	0.002166	0.012459	0.010728	0.002203	0	0.002445203
Carbohydrates	Central carbohydrate metabolism	Pyruvate metabolism I: anaplerotic reactions, PEP	Malate permease	0.000348	0.002262	0.00511	0.00249	0.001313	0.000974	0.001444	0.000498	0.001314	0	0	0.002800152
Carbohydrates	Central carbohydrate metabolism	TCA Cycle	Malate:quinone oxidoreductase (EC 1.1.99.16)	0.003133	0	0.002555	0	0.001313	0.003896	0.005777	0.006977	0.024741	0.008811	0.129591	0.023623814
Membrane Transport	NULL	Transport of Manganese	Manganese ABC transporter, periplasmic-binding protein SitA	0	0.013572	0.010219	0.00249	0.011818	0.012176	0.012275	0.013456	0.007444	0	0.002445	0.003864998
Cell Wall and Capsule	Capsular and extracellular polysacchrides	Exopolysaccharide Biosynthesis	Manganese-dependent protein-tyrosine phosphatase (EC 3.1.3.48)	0.005222	0.002262	0.00511	0.00249	0.00197	0.000487	0.002888	0.002492	0.000438	0	0	0.004219947
Miscellaneous	NULL	YbbK	Membrane protease family protein Swol 1659	0.002437	0.01131	0.00511	0.00996	0.003283	0.006819	0.001444	0.003489	0.004379	0	0	0.004496018
Respiration	Electron accepting reactions	Ubiquinone Menaquinone-cytochrome c reductase complexes	Menaquinone-cytochrome c reductase, cytochrome B subunit	0.005919	0	0.010219	0.00249	0.000657	0.000974	0	0.000997	0.004379	0.002203	0.000349	0.002445203
Cofactors, Vitamins, Prosthetic Groups, Pigments	Quinone cofactors	Menaquinone and Phylloquinone Biosynthesis	Menaquinone-specific isochorismate synthase (EC 5.4.4.2)	0.001044	0.004524	0.00511	0	0.00197	0.000487	0.000722	0.004984	0.003503	0.002203	0	0.00220857
Miscellaneous	Plant-Prokaryote DOE project	Omega-amidase	Methylthioribulose-1-phosphate dehydratase (EC 4.2.1.109)	0.000696	0.004524	0	0.00249	0.001313	0.003409	0	0.003987	0.004379	0.006608	0.009664	0.006191885
Membrane Transport	NULL	Transport of Molybdenum	Molybdate-binding domain of ModE	0.014971	0.009048	0	0	0.000657	0.001948	0.000722	0.000498	0.000438	0.006608	0.000116	0.001025408
Cofactors, Vitamins, Prosthetic Groups, Pigments	Folate and pterines	Molybdenum cofactor biosynthesis	Molybdopterin biosynthesis enzyme	0.002785	0.002262	0	0.00249	0.006566	0.006819	0.007221	0.003987	0.003503	0	0.000116	0.002011377
Fatty Acids, Lipids, and Isoprenoids	Triacylglycerols	Triacylglycerol metabolism	Monoglyceride lipase (EC 3.1.1.23)	0	0.002262	0.00511	0.00249	0.003939	0.005357	0.010109	0.005482	0.005693	0.002203	0	0.003115662
Metabolism of Aromatic Compounds	Metabolism of central aromatic intermediates	Catechol branch of beta-ketoadipate pathway	Muconolactone isomerase (EC 5.3.3.4)	0.000348	0.004524	0	0.00249	0.006566	0.009741	0.007943	0.002492	0.002846	0	0.000466	0.002090254
Membrane Transport	Uni- Sym- and Antiporters	Multi-subunit cation antiporter	Na(+) H(+) antiporter subunit F	0.007659	0.004524	0	0	0.003939	0.002922	0.005055	0.003489	0.004379	0.002203	0.000116	0.00220857
Carbohydrates	Aminosugars	Chitin and N-acetylglucosamine utilization	N-Acetyl-D-glucosamine ABC transport system, permease protein 2	0.004526	0.009048	0.010219	0	0.001313	0.003896	0.005055	0.00598	0.00832	0	0.000699	0.005915813
Clustering-based subsystems	Sulfatases and sulfatase modifying factor 1 (and a hypothetical)	Sulfatases and sulfatase modifying factor 1	N-acetylgalactosamine 6-sulfate sulfatase (GALNS)	0.003482	0.024882	0.010219	0.00747	0.013131	0.015098	0.011553	0.007475	0.008101	0	0	0.005481987
Amino Acids and Derivatives	Lysine, threonine, methionine, and cysteine	Lysine biosynthesis AAA pathway 2	N-acetyl-gamma-aminoadipyl-phosphate reductase (EC 1.2.1.-)	0.003133	0.006786	0.00511	0.00249	0.003939	0.003409	0.00361	0.001993	0.008101	0	0	0.003549488
Cell Wall and Capsule	NULL	UDP-N-acetylmuramate from Fructose-6-phosphate Biosynthesis	N-acetylglucosamine-1-phosphate uridyltransferase eukaryotic (EC 2.7.7.23)	0.004178	0.006786	0.00511	0.00249	0.000657	0.004383	0.002166	0.003987	0.002189	0	0	0.001301479
Carbohydrates	CO2 fixation	Calvin-Benson cycle	NAD(P)-dependent glyceraldehyde 3-phosphate dehydrogenase archaeal (EC 1.2.1.59)	0.001393	0.006786	0.00511	0.00249	0.00197	0.001948	0.005777	0.00299	0.001752	0	0	0.001340918
Respiration	Electron donating reactions	Respiratory Complex I	NADH dehydrogenase subunit 2	0.002785	0	0.00511	0.00747	0.009848	0.000487	0.000722	0.001993	0.005693	0	0.001979	0.001183163
Respiration	Electron donating reactions	Respiratory Complex I	NADH dehydrogenase, subunit 5	0.011141	0.009048	0.010219	0.01743	0.004596	0.016072	0.015886	0.015948	0.018829	0	0	0.017313613
Carbohydrates	Central carbohydrate metabolism	Pyruvate metabolism II: acetyl-CoA, acetogenesis from pyruvate	NAD-independent protein deacetylase AcuC	0.006267	0.004524	0.002555	0	0.000657	0.001461	0.001444	0.000997	0.002189	0.004406	0	0.004219947
Miscellaneous	Plant-Prokaryote DOE project	Biotin biosynthesis Experimental	NfuA Fe-S protein maturation	0.006963	0	0	0.00249	0.005909	0.002435	0.010831	0.007974	0.008758	0.004406	0.032369	0.006901782
Membrane Transport	NULL	Transport of Nickel and Cobalt	Nickel responsive regulator NikR	0.005571	0.01131	0	0.00747	0.008535	0.00828	0.00361	0.001495	0.001752	0.011014	0	0.005521426
Respiration	Electron donating reactions	Hydrogenases	Nickel-dependent hydrogenase, large subunit	0.000696	0.020358	0	0.01245	0.008535	0.009741	0.009387	0.000997	0.000876	0.002203	0	0.005915813
Miscellaneous	Plant-Prokaryote DOE project	Iojap	Nicotinamide-nucleotide adenylyltransferase, NadM family (EC 2.7.7.1)	0.003482	0.002262	0	0.00498	0.00197	0.002435	0.008665	0.003987	0.001971	0	0.000466	0.001419795
Nitrogen Metabolism	NULL	Nitrate and nitrite ammonification	Nitrate/nitrite response regulator protein	0.004526	0	0.020438	0.00498	0.007879	0.007306	0.002166	0.007475	0.006787	0.006608	0	0.009780811
Nitrogen Metabolism	NULL	Denitrification	Nitric oxide reductase activation protein NorD	0.021934	0	0.007664	0.01992	0.004596	0.007306	0.005055	0.00598	0.001314	0	0.017465	0.01202882
Nitrogen Metabolism	NULL	Nitrate and nitrite ammonification	Nitrite reductase [NAD(P)H] small subunit (EC 1.7.1.4)	0.000696	0.004524	0.002555	0	0.002626	0.001461	0.006499	0.003987	0.004598	0	0.005705	0.005008722
Nitrogen Metabolism	NULL	Denitrification	Nitrite reductase accessory protein NirV	0.000696	0.002262	0.002555	0.00747	0.000657	0.001948	0.002888	0.00299	0.002627	0	0	0.001774744
Nitrogen Metabolism	NULL	Nitrate and nitrite ammonification	Nitrite transporter from formate/nitrite family	0.018452	0	0.012774	0	0.002626	0.001461	0.001444	0.003987	0.008101	0.004406	0.050299	0.009544179
Stress Response	Oxidative stress	Oxidative stress	Nitrite-sensitive transcriptional repressor NsrR	0.003133	0.004524	0.007664	0	0.003939	0	0.008665	0.006479	0.008977	0.01542	0.007335	0.00252408
Nitrogen Metabolism	NULL	Ammonia assimilation	Nitrogen regulation protein NR(II) (EC 2.7.3.-)	0.002785	0	0.002555	0.00498	0.003939	0.003896	0.006499	0.004984	0.016202	0	0.008383	0.010924535
Nitrogen Metabolism	NULL	Denitrification	Nitrous oxide reductase maturation protein NosD	0	0.002262	0.002555	0	0.016414	0.010228	0.002888	0.003489	0.007882	0.057273	0.000349	0.012935912
Nitrogen Metabolism	NULL	Denitrification	Nitrous oxide reductase maturation protein NosR	0.019497	0	0.002555	0.00747	0.005252	0.00828	0.008665	0.00897	0.013356	0	0.030971	0.004614334
Cell Wall and Capsule	Gram-Negative cell wall components	Vibrio Core Oligosaccharide Biosynthesis	Nucleoside-diphosphate-sugar pyrophosphorylase involved in lipopolysaccharide biosynthesis/translation	0.002785	0.004524	0.002555	0	0.005909	0.004383	0.007943	0.008472	0.00416	0	0.005007	0.004022753

			initiation factor 2B, gamma/epsilon subunits (eIF-2Bgamma/eIF-2Bepsilon)												
Nucleosides and Nucleotides	Detoxification	Nudix proteins (nucleoside triphosphate hydrolases)	Nudix-like NDP and NTP phosphohydrolase YmfB	0.003133	0.002262	0.002555	0	0.00197	0.000974	0.002166	0.001495	0.000876	0	0.015486	0.002879029
Miscellaneous	Plant-Prokaryote DOE project	Omega-amidase	Omega amidase (Nit2 homolog)	0.000348	0.002262	0.007664	0	0.003939	0.00487	0.002888	0.000997	0.001533	0	0.016883	0.004969283
DNA Metabolism	DNA replication	DNA replication, archaeal	Origin of replication recognition protein	0.002785	0.015834	0	0	0.005252	0.005357	0.005055	0.003489	0.005255	0.008811	0.001397	0.002879029
Cell Wall and Capsule	Gram-Negative cell wall components	Major Outer Membrane Proteins	Outer membrane protein W precursor	0.001741	0	0	0.00249	0.005252	0.005844	0.001444	0.007974	0.004817	0.002203	0.01013	0.001932499
Membrane Transport	NULL	Ton and Tol transport systems	Outer membrane receptor for ferric coprogen and ferric-rhodotorulic acid	0.000348	0	0.002555	0	0.00197	0.002922	0.002166	0.006479	0.025179	0.002203	0.000349	0.000591581
Regulation and Cell signaling	Programmed Cell Death and Toxin-antitoxin Systems	Toxin-antitoxin systems (other than RelBE and MazEF)	ParD protein (antitoxin to ParE)	0.001044	0.006786	0.002555	0	0.000657	0.000974	0.004333	0.003489	0.005693	0	0.000466	0.004969283
Membrane Transport	ABC transporters	ABC transporter peptide (TC 3.A.1.5.5)	Peptide transport system permease protein sapC (TC 3.A.1.5.5)	0.004178	0.002262	0.00511	0	0.00197	0.000487	0	0.004485	0.007882	0.004406	0.000233	0.000985969
Protein Metabolism	Protein folding	Peptidyl-prolyl cis-trans isomerase	Peptidyl-prolyl cis-trans isomerase ppiC (EC 5.2.1.8)	0.004178	0	0.002555	0	0.003939	0.004383	0.00361	0.004485	0.007444	0.002203	0.012691	0.007059537
Respiration	NULL	Biogenesis of c-type cytochromes	Periplasmic thiol:disulfide interchange protein DsbA	0.004874	0.002262	0.007664	0	0.005909	0.006332	0.008665	0.009469	0.01445	0	0.009431	0.006191885
Phages, Prophages, Transposable elements, Plasmids	Phages, Prophages	Phage entry and exit	Phage portal	0.002089	0	0.00511	0	0.04005	0.040424	0.041881	0.065784	0.004598	0.004406	0.000815	0.00252408
Phages, Prophages, Transposable elements, Plasmids	Phages, Prophages	r1t-like streptococcal phages	Phage portal protein	0.001741	0.002262	0.002555	0	0.027575	0.023865	0.014442	0.029902	0.000876	0	0.000582	0.00252408
Phages, Prophages, Transposable elements, Plasmids	Phages, Prophages	Phage tail fiber proteins	Phage tail fiber protein	0.002785	0.002262	0.002555	0	0.022323	0.018995	0.030328	0.022426	0.003065	0	0.000116	0.006191885
Metabolism of Aromatic Compounds	NULL	Phenylacetyl-CoA catabolic pathway (core)	Phenylacetate degradation enoyl-CoA hydratase PaaA (EC 4.2.1.17)	0	0.002262	0.007664	0	0.000657	0.003896	0.002888	0.003987	0.005912	0.004406	0.02643	0.004338263
Metabolism of Aromatic Compounds	NULL	Phenylacetyl-CoA catabolic pathway (core)	Phenylacetate-CoA oxygenase/reductase, PaaK subunit	0.002089	0	0.007664	0.01245	0	0.002922	0.002166	0.006977	0.009415	0.002203	0.00978	0.007098976
Metabolism of Aromatic Compounds	NULL	Phenylacetyl-CoA catabolic pathway (core)	Phenylacetic acid degradation protein PaaD, thioesterase	0.004874	0.004524	0	0.00498	0.000657	0.001948	0.00361	0.004485	0.007006	0	0.003959	0.005915813
Metabolism of Aromatic Compounds	NULL	Phenylacetyl-CoA catabolic pathway (core)	Phenylacetic acid degradation protein PaaY	0.002785	0.002262	0.002555	0	0.000657	0.004383	0.002166	0.00897	0.007006	0	0.020027	0.003825559
Phosphorus Metabolism	NULL	Phosphate metabolism	PhoQ	0	0.006786	0.00511	0	0.003283	0.005844	0.007221	0.007974	0.009196	0.004406	0.000466	0.00918923
Stress Response	Osmotic stress	Synthesis of osmoregulated periplasmic glucans	Phosphoglycerol transferase I (EC 2.7.8.20)	0.005919	0	0.002555	0.02241	0.000657	0.000974	0.002166	0.001495	0.005474	0	0.000582	0.003825559
Fatty Acids, Lipids, and Isoprenoids	Triacylglycerols	Triacylglycerol metabolism	Phospholipase A1 precursor (EC 3.1.1.32, EC 3.1.1.4)	0.005919	0.004524	0	0.00498	0.007879	0.006819	0.005055	0.010964	0.012699	0	0.000116	0.008006067
Membrane Transport	ABC transporters	ABC transporter alkylphosphonate (TC 3.A.1.9.1)	Phosphonate ABC transporter permease protein phnE (TC 3.A.1.9.1)	0.003133	0	0.007664	0	0.006566	0.010715	0.016608	0.012957	0.013137	0.013217	0.081154	0.004929844
Membrane Transport	ABC transporters	ABC transporter alkylphosphonate (TC 3.A.1.9.1)	Phosphonate ABC transporter permease protein phnE2 (TC 3.A.1.9.1)	0	0.006786	0.00511	0.00498	0.003283	0.012663	0.010831	0.014452	0.011823	0	0.009315	0.003549488
Phosphorus Metabolism	NULL	Alkylphosphonate utilization	Phosphonates transport ATP-binding protein PhnK	0.001741	0	0.002555	0.00249	0.007222	0.007793	0.007221	0.014951	0.006568	0	0.025965	0.00220857
Cofactors, Vitamins, Prosthetic Groups, Pigments	Coenzyme A	Coenzyme A Biosynthesis	Phosphopantothenate synthetase, archaeal	0.002785	0.01131	0	0.00498	0	0.000974	0.002166	0.000997	0.001971	0.002203	0.000582	0.001380356
Nucleosides and Nucleotides	Purines	De Novo Purine Biosynthesis	Phosphoribosylaminoimidazolecarboxamide formyltransferase [alternate form]	0	0	0.00511	0.00747	0.003283	0.001948	0.005777	0.002492	0.001095	0.002203	0.000349	0.002050815
Nitrogen Metabolism	NULL	Nitrate and nitrite ammonification	Polyferredoxin NapH (periplasmic nitrate reductase)	0.003133	0.002262	0.00511	0	0.006566	0.007793	0.007221	0.000997	0.000876	0	0.001281	0.00666515
Clustering-based subsystems	NULL	CBSS-224911.1.peg.435	Possible divergent polysaccharide deacetylase	0.004874	0.009048	0.002555	0.01245	0.003939	0.006819	0.009387	0.005482	0.007882	0	0	0.004890406
Carbohydrates	Monosaccharides	L-Arabinose utilization	Possible GPH family transporter (TC 2.A.2) for arabinosides	0.004526	0.002262	0	0.00996	0.001313	0.000487	0.002166	0.000997	0.002408	0	0.00326	0.003312855
Carbohydrates	Monosaccharides	L-rhamnose utilization	Predicted alpha-L-rhamnosidase	0.002437	0.013572	0.002555	0.01494	0.003283	0.007306	0.002166	0.000997	0.002189	0	0	0.005758058
Carbohydrates	NULL	Sugar utilization in Thermotogales	Predicted D-tagaturonate epimerase	0.008704	0.018096	0.007664	0.01245	0.009192	0.009741	0.008665	0.003489	0.009415	0	0	0.008400455
Cell Wall and Capsule	Gram-Negative cell wall components	KDO2-Lipid A biosynthesis	Predicted hydrolase of the metallo-beta-lactamase superfamily, clustered with KDO2-Lipid A biosynthesis genes	0.009052	0.009048	0	0.01245	0.011818	0.007306	0.001444	0.010964	0.008758	0.004406	0	0.007177853
Cofactors, Vitamins, Prosthetic Groups, Pigments	NULL	Thiamin biosynthesis	Predicted hydroxymethylpyrimidine transporter CytX	0.002785	0	0.002555	0.00249	0.001313	0.001461	0.001444	0.000498	0.000438	0	0.000466	0.000907091
Clustering-based subsystems	NULL	Conserved cluster around acetyltransferase YpeA in Enterobacteria	Predicted iron-dependent peroxidase, Dyp-type family	0.005222	0.006786	0	0.00249	0	0.002435	0.001444	0.004485	0.008539	0.002203	0.027828	0.005679181
Carbohydrates	Monosaccharides	Deoxyribose and Deoxynucleoside Catabolism	Predicted nucleoside ABC transporter, substrate-binding component	0.002785	0	0	0.00498	0.003283	0.003409	0.002888	0.00598	0.010947	0.004406	0.003493	0.001301479
Carbohydrates	Monosaccharides	L-rhamnose utilization	Predicted rhamnose oligosaccharide ABC transport system, permease component	0.005222	0.006786	0	0.00249	0.003939	0.002922	0.004333	0.003489	0.007225	0	0.000233	0.005639742
Membrane Transport	Protein secretion system, Type II	Predicted secretion system X	Predicted secretion system X protein GspE-like	0.005571	0.002262	0	0.00498	0.001313	0.002435	0.001444	0.000498	0.007882	0.004406	0	0.003115662
Carbohydrates	Aminosugars	Chitin and N-acetylglucosamine utilization	Predicted transcriptional regulator of N-Acetylglucosamine utilization, GntR family	0.000348	0	0.002555	0	0.005252	0.003409	0.004333	0.007475	0.004379	0.002203	0.000349	0.002760713
Carbohydrates	Monosaccharides	Xylose utilization	predicted xylose isomerase	0	0.009048	0.002555	0.00249	0.00197	0.000974	0.002888	0.000498	0.000219	0	0.000815	0.001301479
Stress Response	Oxidative stress	Rubrerythrin	Probable peroxiredoxin (EC 1.11.1.15)	0.009052	0.004524	0.00511	0.00249	0	0.005357	0.004333	0.002492	0.001314	0	0.000815	0.005403109
Amino Acids and Derivatives	Alanine, serine, and glycine	Alanine biosynthesis	Probable valine-pyruvate aminotransferase (EC 2.6.1.66)	0.000348	0.002262	0.00511	0	0.000657	0.000974	0.000722	0.000498	0.000657	0.002203	0	0.000591581
Amino Acids and Derivatives	Proline and 4-hydroxyproline	Proline, 4-hydroxyproline uptake and utilization	Proline racemase (EC 5.1.1.4)	0	0.002262	0.00511	0.00498	0.002626	0.001948	0.000722	0.001495	0.002627	0	0.000815	0.001104285
Cell Wall and Capsule	Gram-Negative cell wall components	KDO2-Lipid A biosynthesis	Protein of unknown function DUF1009 clustered with KDO2-Lipid A biosynthesis genes	0.001044	0.002262	0.00511	0.00249	0.005252	0.004383	0.002166	0.006977	0.001095	0	0	0.002445203
Miscellaneous	Plant-Prokaryote DOE project	At1g01770	Protein of unknown function DUF1446	0.004178	0	0.002555	0.00249	0.006566	0.003896	0.00361	0.005482	0.011385	0	0.001164	0.003391733
Clustering-based subsystems	NULL	Conserved gene cluster associated with Met-tRNA formyltransferase	Protein of unknown function Smg	0.006267	0.004524	0.00511	0.00996	0.001313	0	0.001444	0.003987	0.004817	0.002203	0	0.003194539
Clustering-based subsystems	NULL	Conserved gene cluster associated with Met-tRNA formyltransferase	Protein serine/threonine phosphatase PrpC, regulation of stationary phase	0.005919	0.004524	0.002555	0.00747	0.00197	0.003896	0.002166	0.003489	0.007006	0	0	0.006428517
Miscellaneous	NULL	ZZ gjo need homes	Proton/glutamate symport protein	0.001393	0.002262	0.002555	0	0.002626	0.000974	0.002888	0.003489	0.001971	0.002203	0	0.002169132
Cofactors, Vitamins, Prosthetic Groups, Pigments	Tetrapyrroles	Chlorophyll Biosynthesis	Protoporphyrin IX Mg-chelatase subunit I (EC 6.6.1.1)	0.005571	0.01131	0	0.01743	0.003283	0.007793	0.010831	0.011462	0.012918	0	0.001281	0.003194539
Cofactors, Vitamins, Prosthetic Groups, Pigments	Tetrapyrroles	Heme and Siroheme Biosynthesis	Protoporphyrinogen IX oxidase, aerobic, HemY (EC 1.3.3.4)	0.008704	0	0.012774	0.0249	0.009192	0.007793	0.002888	0.009469	0.007663	0	0.000116	0.00666515
Cell Wall and Capsule	Capsular and extracellular polysacchrides	Pseudaminic Acid Biosynthesis	Pseudaminic acid cytidylyltransferase (EC 2.7.7.43)	0.001044	0.004524	0	0.00249	0.007222	0.004383	0.00361	0.001993	0.002408	0	0.006753	0.005324232
Protein Metabolism	Protein degradation	Metalloendopeptidases (EC 3.4.24.-)	Pseudolysin, extracellular zinc protease (EC 3.4.24.26)	0.001044	0	0.002555	0	0.003283	0.000974	0.001444	0.001495	0.000876	0.004406	0.000233	0.001104285
Nucleosides and Nucleotides	NULL	Pseudouridine catabolism	Pseudouridine 5'-phosphate glycosidase	0	0.002262	0.007664	0.00747	0.004596	0.005844	0.00361	0.000498	0.003941	0	0.00163	0.004456579
Carbohydrates	NULL	Sugar utilization in Thermotogales	PTS system, fructose-specific IIC component (EC 2.7.1.69)	0.007659	0.002262	0.002555	0	0.000657	0.000974	0.000722	0.000498	0.001314	0	0.00163	0.002248009
Carbohydrates	Di- and oligosaccharides	Trehalose Uptake and Utilization	PTS system, glucose-specific IIA component (EC 2.7.1.69)	0	0.002262	0.002555	0.00249	0.002626	0.000974	0.000722	0.000498	0.00416	0	0.001048	0.001616989
Protein Metabolism	Protein degradation	Proteasome bacterial	Pup ligase PafA' paralog, possible component of postulated heterodimer PafA-PafA'	0.002785	0	0.010219	0	0.003283	0.000974	0.000722	0.001495	0.003065	0.004406	0.000233	0.001143724
Carbohydrates	CO2 fixation	CO2 uptake, carboxysome	putative carboxysome peptide A	0.004874	0	0.00511	0.00249	0.001313	0.001948	0.001444	0.001993	0.000438	0.004406	0	0.003707243

Carbohydrates	Central carbohydrate metabolism	Dihydroxyacetone kinases	Putative dihydroxyacetone kinase (EC 2.7.1.29), dihydroxyacetone binding subunit	0.011141	0.002262	0.002555	0	0.002626	0.007793	0.000722	0.001495	0.001533	0	0.000466	0.002366325
Sulfur Metabolism	Organic sulfur assimilation	Utilization of glutathione as a sulphur source	Putative gluthatione transporter,solute-binding component	0	0.004524	0.002555	0.00747	0.006566	0.009254	0.007221	0.001993	0.004817	0	0.000582	0.001814183
Miscellaneous	NULL	ZZ gjo need homes	Putative heat shock protein YegD	0	0.002262	0.00511	0	0.001313	0.000487	0.001444	0.001495	0.003503	0.011014	0.002562	0.001143724
Clustering-based subsystems	Hypothetical protein possible functionally linked with Alanyl-tRNA synthetase	CBSS-257314.1.peg.488	Putative Holliday junction resolvase (EC 3.1.-.-)	0.002785	0	0.002555	0.00249	0.00197	0.003409	0.000722	0.005482	0.004598	0	0.003377	0.004811528
Metabolism of Aromatic Compounds	NULL	Gentisare degradation	Putative n-hydroxybenzoate hydroxylase	0.003482	0	0.002555	0.00249	0	0.003896	0.002888	0.000498	0.00416	0.002203	0.000349	0.000433826
Virulence, Disease and Defense	Invasion and intracellular resistance	Listeria surface proteins: LPXTG motif	Putative peptidoglycan bound protein (LPXTG motif) Lmo1799 homolog	0.00383	0.004524	0.012774	0.00498	0.001313	0.007306	0.002888	0.001993	0.000438	0	0	0.001656428
Carbohydrates	Central carbohydrate metabolism	Glycolysis and Gluconeogenesis	Putative phosphoenolpyruvate synthase/pyruvate phosphate dikinase, N-terminal domain	0.000348	0	0.007664	0.00747	0.003939	0.001461	0.001444	0	0.000657	0.008811	0.000233	0.000867653
DNA Metabolism	NULL	Restriction-Modification System	Putative predicted metal-dependent hydrolase	0.001741	0	0.002555	0.01245	0.005252	0.003896	0.004333	0.006479	0.003284	0	0.000116	0.004022753
Clustering-based subsystems	NULL	Putative sulfate assimilation cluster	Putative reductase (alkanesulfonate metabolism)	0.002785	0.002262	0	0	0.002626	0.000974	0.001444	0.000498	0.005693	0.006608	0.000466	0.002681835
Nucleosides and Nucleotides	Pyrimidines	Pyrimidine utilization	Pyrimidine ABC transporter, ATP-binding protein	0.002785	0	0.015329	0	0.00197	0.003896	0.002166	0.004485	0.007882	0.002203	0.000349	0.002642397
Nucleosides and Nucleotides	Pyrimidines	Pyrimidine utilization	Pyrimidine ABC transporter, substrate-binding component	0	0.006786	0.00511	0.00249	0.005252	0.003409	0.004333	0.006977	0.006131	0	0.000466	0.00347061
Nucleosides and Nucleotides	Pyrimidines	Pyrimidine utilization	Pyrimidine ABC transporter, transmembrane component 2	0.002437	0.004524	0	0.00249	0.001313	0.003409	0.000722	0.00299	0.003065	0	0.000349	0.000867653
Cofactors, Vitamins, Prosthetic Groups, Pigments	Riboflavin, FMN, FAD	Riboflavin, FMN and FAD metabolism	Pyrimidine deaminase archaeal predicted (EC 3.5.4.26)	0.000348	0.002262	0	0.00747	0.005252	0.00487	0.002166	0.001993	0.003503	0	0.000233	0.003233978
Nucleosides and Nucleotides	Pyrimidines	De Novo Pyrimidine Synthesis	Pyrimidine operon regulatory protein PyrR	0.00383	0	0.010219	0.00498	0.005252	0.001461	0.001444	0.001495	0.001533	0	0.000233	0.002800152
Carbohydrates	Central carbohydrate metabolism	Pyruvate metabolism II: acetyl-CoA, acetogenesis from pyruvate	Pyruvate decarboxylase (EC 4.1.1.1)	0.000696	0.004524	0.002555	0	0.004596	0.000974	0.002888	0.002492	0.002189	0.004406	0	0.00189306
Carbohydrates	Central carbohydrate metabolism	Pyruvate:ferredoxin oxidoreductase	Pyruvate:ferredoxin oxidoreductase, delta subunit (EC 1.2.7.1)	0	0.009048	0.00511	0.00249	0.004596	0.005357	0.000722	0.001495	0.001095	0	0.000233	0.004574896
Amino Acids and Derivatives	Aromatic amino acids and derivatives	Chorismate Synthesis	Quinate/shikimate 5-dehydrogenase I delta (EC 1.1.1.25)	0.006615	0.002262	0.00511	0.00249	0.001313	0.000974	0.002888	0.00299	0.001971	0	0	0.000867653
Cofactors, Vitamins, Prosthetic Groups, Pigments	Folate and pterines	Pterin metabolism 3	Related to Dihydropteroate synthase	0.002785	0.006786	0	0.00249	0.005252	0.001461	0	0.001993	0.002189	0.004406	0.000116	0.002248009
Nitrogen Metabolism	NULL	Nitrate and nitrite ammonification	Respiratory nitrate reductase gamma chain (EC 1.7.99.4)	0.003482	0.009048	0	0	0.003283	0.001948	0.00361	0.005482	0.005693	0.004406	0.000699	0.001616989
Virulence, Disease and Defense	Bacteriocins, ribosomally synthesized antibacterial peptides	Bacitracin Stress Response	Response regulator LiaR	0.00383	0.002262	0.002555	0.00249	0.00197	0.001461	0.001444	0	0.000219	0	0.000233	0.004062192
Nitrogen Metabolism	NULL	Nitrate and nitrite ammonification	Response regulator NasT	0.001393	0.002262	0.00511	0	0.005252	0.001948	0.001444	0.003987	0.008977	0	0.003493	0.004101631
Miscellaneous	Plant-Prokaryote DOE project	At1g14345	Ribonuclease P protein component (EC 3.1.26.5)	0	0.013572	0.002555	0	0.006566	0.003896	0.004333	0.007475	0.008539	0.01542	0.019444	0.008006067
Nucleosides and Nucleotides	NULL	Ribonucleotide reduction	Ribonucleotide reductase of class III (anaerobic), activating protein (EC 1.97.1.4)	0.003482	0.006786	0	0.01494	0.009192	0.009741	0.007943	0.00299	0.000219	0	0.002212	0.006073568
RNA Metabolism	RNA processing and modification	RNA pseudouridine syntheses	Ribosomal large subunit pseudouridine synthase A (EC 4.2.1.70)	0	0.002262	0	0.00996	0.000657	0.005357	0.008665	0.008472	0.01248	0.026433	0.010945	0.006231323
RNA Metabolism	RNA processing and modification	RNA pseudouridine syntheses	Ribosomal large subunit pseudouridine synthase F (EC 4.2.1.70)	0.000348	0	0	0.00249	0.003283	0.004383	0.007221	0.00299	0.007225	0.037447	0.065669	0.021967387
Carbohydrates	CO2 fixation	Calvin-Benson cycle	Ribulose-1,5-bisphosphate carboxylase, Type III (EC 4.1.1.39)	0	0.002262	0.010219	0.00498	0.007222	0.001461	0.006499	0	0.001971	0.002203	0.000582	0.00283959
Miscellaneous	Plant-Prokaryote DOE project	At1g14345	RNA-binding protein Jag	0.002437	0.002262	0.00511	0.00249	0.006566	0.003896	0.007221	0.001495	0.002189	0	0	0.007848312
Clustering-based subsystems	TldD cluster	CBSS-354.1.peg.2917	Rod shape-determining protein MreD	0	0.004524	0.007664	0.00249	0.000657	0.002922	0	0.003987	0.007663	0.002203	0.000233	0.002169132
Stress Response	Oxidative stress	Rubrerythrin	rubredoxin-oxygen oxidoreductase	0.012882	0.018096	0.010219	0.00498	0.011161	0.023865	0.000722	0.003987	0.001314	0	0	0.00982025
Metabolism of Aromatic Compounds	Metabolism of central aromatic intermediates	Salicylate and gentisate catabolism	salicylate esterase	0.000696	0.006786	0	0.00249	0.001313	0.000974	0.001444	0.00299	0.002189	0	0.000116	0.001025408
Stress Response	Osmotic stress	Betaine biosynthesis from glycine	Sarcosine N-methyltransferase	0.002089	0.015834	0.00511	0	0.015757	0.011689	0.024551	0.019934	0.009196	0.004406	0	0.002602958
Virulence, Disease and Defense	Resistance to antibiotics and toxic compounds	The mdtABCD multidrug resistance cluster	Sensory histidine kinase BaeS	0.059883	0.004524	0	0	0.006566	0.002922	0.002888	0.002492	0.003503	0.002203	0.005822	0.004811528
Clustering-based subsystems	NULL	CBSS-269801.1.peg.1715	Serine protease (EC 3.4.21.-)	0.002785	0.004524	0	0.00249	0.001313	0.002435	0.002888	0.002492	0.003065	0.004406	0	0.006507395
Membrane Transport	Protein translocation across cytoplasmic membrane	HtrA and Sec secretion	Serine protease, DegP/HtrA, do-like (EC 3.4.21.-)	0.002437	0.006786	0.00511	0.00747	0.003939	0.006332	0.005055	0.001993	0.005255	0	0	0.004772089
Cofactors, Vitamins, Prosthetic Groups, Pigments	Quinone cofactors	Coenzyme PQQ synthesis	Similar to coenzyme PQQ synthesis protein B	0.003482	0.004524	0.00511	0.00498	0.002626	0.003896	0.004333	0.00598	0.003722	0	0	0.001498673
DNA Metabolism	DNA repair	2-phosphoglycolate salvage	Similar to phosphoglycolate phosphatase, clustered with ubiquinone biosynthesis SAM-dependent O-methyltransferase	0.008008	0.002262	0.007664	0.00249	0.005252	0.005357	0.004333	0.008472	0.006568	0	0	0.006783466
Cofactors, Vitamins, Prosthetic Groups, Pigments	Tetrapyrroles	Coenzyme B12 biosynthesis	Sirohydrochlorin cobaltochelatase (EC 4.99.1.3)	0.003482	0	0.002555	0.01245	0.002626	0.006332	0.012275	0.011961	0.010728	0	0.001747	0.001340918
Dormancy and Sporulation	NULL	Spore Core Dehydration	Spore maturation protein A-like protein	0.004874	0.002262	0.002555	0.00747	0.003283	0.00487	0.002888	0.00598	0.003941	0	0	0.002129693
Cofactors, Vitamins, Prosthetic Groups, Pigments	Folate and pterines	YgfZ	Spore photoproduct lyase (EC 4.1.99.-)	0.002437	0.009048	0.00511	0.00249	0.007222	0.012663	0.004333	0.002492	0.001533	0	0	0.005876374
Clustering-based subsystems	Clustering-based subsystems	Putative diaminopropionate ammonia-lyase cluster	SsnA protein	0.000696	0.004524	0.010219	0	0.001313	0.002435	0.00361	0.001993	0.000438	0	0.000233	0.001538111
Protein Metabolism	Protein biosynthesis	Ribosome SSU eukaryotic and archaeal	SSU ribosomal protein S14e (S11p)	0	0	0.007664	0.00498	0.004596	0.002922	0.001444	0.004984	0.000657	0.01542	0.000233	0.002366325
Protein Metabolism	Protein biosynthesis	Ribosome SSU eukaryotic and archaeal	SSU ribosomal protein S20e (S10p)	0	0.002262	0	0.00747	0.002626	0.000487	0.000722	0.000498	0.000438	0.002203	0.000466	0.000985969
Protein Metabolism	Protein biosynthesis	Ribosome SSU eukaryotic and archaeal	SSU ribosomal protein S2e (S5p)	0.002785	0.009048	0.00511	0	0.002626	0.002922	0.00361	0.001495	0.000876	0	0.000116	0.002011377
Protein Metabolism	Protein biosynthesis	Ribosome SSU eukaryotic and archaeal	SSU ribosomal protein SAe (S2p)	0.002785	0	0.00511	0.00249	0.002626	0.002922	0.006499	0.004485	0.001095	0	0.000466	0.001616989
Phages, Prophages, Transposable elements, Plasmids	Phages, Prophages	r1t-like streptococcal phages	structural protein	0.00383	0	0	0.00498	0.039393	0.043347	0.033938	0.053325	0.005693	0.011014	0.000349	0.002050815
Miscellaneous	Plant-Prokaryote DOE project	At5g38900	Substrate-binding periplasmic component of uncharacterized ABC transporter	0.006267	0	0.007664	0.00747	0.003283	0.002435	0.000722	0.003489	0.005693	0.002203	0	0.002287448
Amino Acids and Derivatives	Arginine; urea cycle, polyamines	Arginine and Ornithine Degradation	Succinylglutamic semialdehyde dehydrogenase (EC 1.2.1.71)	0.002785	0	0.010219	0	0.001313	0.004383	0.010831	0.010964	0.022114	0.008811	0.118064	0.012068259
Sulfur Metabolism	NULL	Sulfur oxidation	Sulfite dehydrogenase cytochrome subunit SoxD	0.007659	0	0	0.00249	0.001313	0.008767	0.012275	0.010466	0.003941	0.004406	0.001514	0.002405764
Sulfur Metabolism	NULL	Sulfate reduction-associated complexes	Sulfite reduction-associated complex DsrMKJOP protein DsrP (= HmeB)	0.025067	0.031668	0.017884	0.00996	0.018384	0.029709	0.019496	0.00897	0.002627	0	0	0.014276829
Sulfur Metabolism	NULL	Sulfur oxidation	Sulfur oxidation protein SoxZ	0.005571	0.002262	0.00511	0	0.006566	0.003896	0.005777	0.00299	0.003065	0	0.000815	0.006113007
Carbohydrates	Polysaccharides	Cellulosome	SusC, outer membrane protein involved in starch binding	0.00383	0.01131	0.012774	0.00747	0.004596	0.005844	0.005777	0.019436	0.009853	0	0	0.031196055
Membrane Transport	Protein secretion system, Type I	Type I secretion system for aggregation	T1SS secreted agglutinin (RTX)	0.000348	0.004524	0.010219	0.00747	0.011161	0.019969	0.028883	0.012957	0.016202	0	0	0.014040197
Carbohydrates	Organic acids	Tricarballylate Utilization	TcuB: works with TcuA to oxidize tricarballylate to cis-aconitate	0	0.002262	0.00511	0	0.004596	0.004383	0.002166	0.004485	0.003503	0.002203	0.03167	0.005324232
Respiration	Electron accepting reactions	Tetrathionate respiration	Tetrathionate reductase subunit B	0.015319	0.013572	0.012774	0.01245	0.018384	0.010228	0.005777	0.000997	0.002627	0	0	0.008321577
Clustering-based subsystems	Cytochrome biogenesis	CBSS-196164.1.peg.461	Thiol:disulfide oxidoreductase related to ResA	0.00383	0.004524	0.010219	0.00996	0.006566	0.006819	0.008665	0.004984	0.006131	0	0	0.005481987

Clustering-based subsystems	NULL	CBSS-316273.3.peg.448	TldD family protein, Actinobacterial subgroup	0.006267	0.006786	0.010219	0	0.005252	0.006819	0.007943	0.005482	0.004817	0	0.001397	0.004259386
Membrane Transport	NULL	Ton and Tol transport systems	TonB system biopolymer transport component	0.00383	0	0	0.00498	0.002626	0.001461	0.012275	0.00897	0.015326	0.002203	0.015602	0.00283959
Amino Acids and Derivatives	Lysine, threonine, methionine, and cysteine	Methionine Biosynthesis	Transcriptional activator MetR	0	0.006786	0	0.00747	0.001313	0.004383	0.005777	0.006479	0.005912	0.008811	0.044128	0.008518771
Clustering-based subsystems	NULL	Maltose utilization cluster	Transcriptional activator of maltose regulon, MalT	0.018452	0.03393	0.028103	0.0249	0.032171	0.010715	0.012275	0.004984	0.030653	0	0	0.027686006
Clustering-based subsystems	Methylamine utilization	CBSS-265072.7.peg.546	Transcriptional regulator containing an amidase domain and an AraC-type DNA-binding HTH domain	0.010097	0	0	0.00498	0.009192	0.003896	0.002888	0.013456	0.010509	0.004406	0.026081	0.003707243
Carbohydrates	Di- and oligosaccharides	Maltose and Maltodextrin Utilization	Transcriptional regulator of maltose utilization, LacI family	0.002785	0.002262	0.00511	0	0	0.001948	0.001444	0.001993	0.002189	0.002203	0.000116	0.001340918
Sulfur Metabolism	NULL	Sulfur oxidation	transcriptional regulator SoxR	0.000696	0.006786	0.002555	0	0.000657	0.002435	0.002166	0.000498	0.004379	0	0.000466	0.001025408
Virulence, Disease and Defense	Resistance to antibiotics and toxic compounds	BlaR1 Family Regulatory Sensor-transducer Disambiguation	Transcriptional regulator, MecI family	0.012186	0	0	0.00498	0.003283	0.000974	0.004333	0.006977	0.006568	0.013217	0.00163	0.018023511
Miscellaneous	Plant-Prokaryote DOE project	COG3380 COG2907	Transcriptional regulator, MerR family, associated with photolyase	0.00383	0.006786	0.007664	0.00249	0.002626	0.000974	0	0.000498	0.000876	0.002203	0	0.002760713
RNA Metabolism	RNA processing and modification	RNA 3'-terminal phosphate cyclase	Transcriptional regulatory protein RtcR	0.006267	0.006786	0.00511	0	0.003939	0.003896	0.005777	0.005482	0.001971	0	0.004541	0.00347061
Carbohydrates	Di- and oligosaccharides	Trehalose Uptake and Utilization	Trehalase (EC 3.2.1.28)	0.008008	0.002262	0	0	0.00197	0.002922	0.005055	0.004984	0.005036	0.008811	0.000466	0.003707243
Carbohydrates	Di- and oligosaccharides	Trehalose Uptake and Utilization	Trehalose synthase, nucleoside diphosphate glucose dependent	0.003482	0.004524	0.002555	0.00498	0.001313	0.000974	0.001444	0.000498	0.003722	0	0	0.003667804
Carbohydrates	Di- and oligosaccharides	Trehalose Uptake and Utilization	Trehalose-6-phosphate hydrolase (EC 3.2.1.93)	0.002089	0.004524	0	0	0.000657	0.003409	0.000722	0.004485	0.004598	0.004406	0.002212	0.003076223
Carbohydrates	One-carbon Metabolism	Methanogenesis from methylated compounds	Trimethylamine:corrinoid methyltransferase	0.01323	0.01131	0.00511	0.01743	0.036767	0.045295	0.020218	0.010964	0.004598	0	0	0.00761168
Respiration	Electron accepting reactions	trimethylamine N-oxide (TMAO) reductase	Trimethylamine-N-oxide reductase (EC 1.6.6.9)	0.047698	0.158339	0.109856	0.097112	0.092574	0.030196	0.014442	0.006479	0.002627	0	0	0.030131209
Miscellaneous	Plant-Prokaryote DOE project	At5g37530	tRNA (5-methylaminomethyl-2-thiouridylate)-methyltransferase(EC:2.1.1.61)	0.007311	0.002262	0	0.00249	0.001313	0.000974	0.001444	0.001495	0.001314	0	0.000349	0.002918468
RNA Metabolism	RNA processing and modification	tRNA modification Archaea	tRNA pseudouridine 55 synthase (EC 4.2.1.70)	0	0.006786	0.002555	0	0.005252	0.00487	0.004333	0.001495	0.000219	0.002203	0.000931	0.004850967
Clustering-based subsystems	proteosome related	Cluster-based Subsystem Grouping Hypotheticals - perhaps Proteosome Related	Twin-arginine translocation protein TatA	0.029593	0.009048	0.025548	0.01992	0.015101	0.021917	0.029605	0.025416	0.024084	0	0	0.025122487
Amino Acids and Derivatives	Glutamine, glutamate, aspartate, asparagine; ammonia assimilation	Glutamate and Aspartate uptake in Bacteria	Two component, Sigma-54 Specific, central transcriptional regulator of acidic amino acid uptake	0.002089	0.004524	0.002555	0	0.002626	0.001948	0.001444	0.000997	0.005255	0	0.013856	0.006783466
Regulation and Cell signaling	NULL	Orphan regulatory proteins	Two-component nitrogen fixation transcriptional regulator FixJ	0.005222	0	0.002555	0.00996	0.001313	0.001461	0.004333	0.003987	0.005474	0.002203	0	0.004969283
Virulence, Disease and Defense	Bacteriocins, ribosomally synthesized antibacterial peptides	Tolerance to colicin E2	Two-component response regulator CreC	0	0	0.00511	0.00747	0.003939	0.005844	0.002166	0.002492	0.000876	0.004406	0.000349	0.002879029
Cell Wall and Capsule	Gram-Negative cell wall components	Major Outer Membrane Proteins	Two-component system response regulator OmpR	0.002785	0.002262	0.002555	0.00249	0	0.000974	0	0.000498	0.005036	0.004406	0.000466	0.001538111
Membrane Transport	Protein secretion system, Type I	Type I secretion system for aggregation	type I secretion system ATPase, LssB family (LapB)	0	0	0.002555	0.00249	0.004596	0.002922	0.004333	0.001993	0.001533	0.008811	0.078011	0.004614334
Cell Wall and Capsule	Gram-Negative cell wall components	LOS core oligosaccharide biosynthesis	UDP-glucose:(heptosyl) LPS alpha1,3-glucosyltransferase WaaG (EC 2.4.1.-)	0.000696	0.01131	0.007664	0	0.002626	0.00487	0.002888	0.003489	0.001533	0	0.000349	0.00094653
Cell Wall and Capsule	Gram-Negative cell wall components	Lipopolysaccharide assembly	Uncharacterized ABC transporter, periplasmic component YrbD	0.008008	0	0.00511	0.00249	0.003939	0.004383	0.002888	0.003489	0.009415	0	0.014088	0.007651118
Miscellaneous	NULL	ZZ gjo need homes	Uncharacterized ATP-dependent helicase MJ0294	0	0.01131	0.010219	0.00498	0.021666	0.007793	0.007221	0.003489	0.007444	0	0.000116	0.007177853
Miscellaneous	Plant-Prokaryote DOE project	COG0398	Uncharacterized protein DUF547	0	0.002262	0	0.00498	0.013131	0.011689	0.006499	0.012957	0.007882	0.004406	0.000931	0.004535457
Membrane Transport	Protein secretion system, Type VI	Type VI secretion systems	Uncharacterized protein ImpA	0.007659	0	0.002555	0	0.008535	0.000974	0.000722	0.001993	0.005912	0.004406	0.001281	0.00126204
Clustering-based subsystems	NULL	USS-DB-7	Uncharacterized protein ImpJ/VasE	0	0.006786	0.010219	0.00498	0.006566	0.001948	0.002166	0.003987	0.006568	0	0.000466	0.003510049
Carbohydrates	NULL	Sugar utilization in Thermotogales	Unknown pentose isomerase TM0951	0.011489	0.02262	0.015329	0.01245	0.011161	0.007793	0.005777	0.00598	0.002189	0	0	0.006467956
Clustering-based subsystems	Monosaccharides	Unspecified monosaccharide transport cluster	Unspecified monosaccharide ABC transport system, permease component 2	0.002437	0.006786	0.015329	0.00498	0.002626	0.00487	0.002888	0.00299	0.002627	0	0	0.003588927
Clustering-based subsystems	NULL	CBSS-630.2.peg.3360	UPF0235 protein VC0458	0.001393	0.002262	0.002555	0	0.000657	0.000487	0.002888	0.000997	0.000219	0	0.004774	0.001459234
Protein Metabolism	Protein processing and modification	G3E family of P-loop GTPases (metallocenter biosynthesis)	Urease gamma subunit (EC 3.5.1.5)	0.026112	0	0.010219	0.01245	0.00197	0.003896	0.006499	0.004984	0.008101	0	0.000931	0.007887751
Virulence, Disease and Defense	Resistance to antibiotics and toxic compounds	Resistance to Vancomycin	Vancomycin B-type resistance protein VanW	0	0.002262	0.010219	0.00249	0.005909	0.002922	0.000722	0.000997	0.001752	0	0.000349	0.001419795
Regulation and Cell signaling	Programmed Cell Death and Toxin-antitoxin Systems	Toxin-antitoxin systems (other than RelBE and MazEF)	VapB protein (antitoxin to VapC)	0.000348	0.002262	0.007664	0.00249	0.005909	0.003409	0.006499	0.002492	0.001533	0	0	0.002997345
Stress Response	Detoxification	Uptake of selenate and selenite	Various polyols ABC transporter, permease component 1	0.000348	0	0.00511	0	0.003939	0.005357	0.004333	0.006977	0.008977	0.008811	0.00163	0.0031551
Protein Metabolism	Protein degradation	Metalloendopeptidases (EC 3.4.24.-)	Vibriolysin, extracellular zinc protease (EC 3.4.24.25)	0.000696	0	0.002555	0	0.001313	0.006819	0.002166	0.00299	0.003284	0.002203	0.003842	0.001932499
Respiration	ATP synthases	V-Type ATP synthase	V-type ATP synthase subunit I (EC 3.6.3.14)	0.010793	0.013572	0.012774	0.01743	0.013788	0.012176	0.018052	0.010964	0.005474	0	0	0.007453925
Stress Response	Heat shock	Heat shock dnaK gene cluster extended	Xanthosine/inosine triphosphate pyrophosphatase	0.001393	0.006786	0	0.00498	0.002626	0.002435	0.00361	0.001495	0.002189	0	0.001281	0.002090254
Clustering-based subsystems	NULL	CBSS-316273.3.peg.2378	YeeC-like protein	0	0	0.002555	0.00498	0.001313	0.002922	0.002166	0.001495	0.001971	0.002203	0.000233	0.001025408
Regulation and Cell signaling	Programmed Cell Death and Toxin-antitoxin Systems	Toxin-antitoxin systems (other than RelBE and MazEF)	YefM protein (antitoxin to YoeB)	0.003133	0.01131	0	0.00249	0.005909	0.005844	0.007221	0.002492	0.002627	0.006608	0	0.002681835
Cofactors, Vitamins, Prosthetic Groups, Pigments	Folate and pterines	5-FCL-like protein	YjbQ (alternate ThiE)	0.008008	0.002262	0.010219	0.00249	0.005909	0.005844	0.002166	0.000498	0.001971	0	0	0.002800152
Membrane Transport	NULL	Transport of Zinc	Zinc transporter, ZIP family	0.004526	0.004524	0.022993	0.00996	0.006566	0.006819	0.008665	0.001495	0.003503	0	0	0.008992036
Membrane Transport	NULL	Transport of Zinc	Zinc-regulated outer membrane receptor	0.002785	0	0.002555	0.00249	0.005909	0.007793	0.010831	0.016944	0.013137	0	0.000349	0.002602958
Carbohydrates	Aminosugars	(GlcNAc)2 Catabolic Operon	(GlcNAc)2 ABC transporter, ATP-binding component 1	0.000348	0.006786	0	0.00747	0	0.001948	0.001444	0.000997	0.002408	0	0.000233	0.001143724
Carbohydrates	Central carbohydrate metabolism	Dehydrogenase complexes	(Pyruvate) Oxoisovalerate Dehydrogenase Alpha & Beta Fusion like	0.036905	0	0.002555	0.00249	0.000657	0	0.001444	0.001993	0.001971	0	0.000116	0.000828214
Carbohydrates	Central carbohydrate metabolism	Glyoxylate bypass	(R)-2-hydroxyacid dehydrogenase, similar to L-sulfolactate dehydrogenase (EC 1.1.1.272)	0	0.002262	0	0.00249	0.001313	0	0.000722	0.000997	0.000657	0.002203	0.000116	0.000473265
Fatty Acids, Lipids, and Isoprenoids	Isoprenoids	Archaeal lipids	(S)-2,3-di-O-geranylgeranylglyceryl phosphate synthase	0.001044	0.004524	0	0	0.002626	0.001948	0.000722	0.00299	0.00416	0.002203	0	0.011161168
Protein Metabolism	Protein processing and modification	G3E family of P-loop GTPases (metallocenter biosynthesis)	[NiFe] hydrogenase nickel incorporation protein HypA	0	0.015834	0	0.01743	0.003283	0.006819	0.005777	0.001993	0.002408	0	0.016999	0.006507395
Carbohydrates	NULL	Sugar utilization in Thermotogales	1-phosphofructokinase (EC 2.7.1.56)	0.000696	0.009048	0	0.00498	0.001313	0.001461	0.000722	0.002492	0.000876	0	0	0.001932499
Carbohydrates	Fermentation	Acetoin, butanediol metabolism	2,3-butanediol dehydrogenase, S-alcohol forming, (S)-acetoin-specific (EC 1.1.1.76)	0	0.004524	0	0.00249	0.001313	0.001948	0.000722	0.003987	0.00416	0	0.000466	0.001380356
Secondary Metabolism	Aromatic amino acids and derivatives	Cinnamic Acid Degradation	2,3-dihydroxyphenylpropionate 1,2-dioxygenase (EC 1.13.11.-)	0	0	0.002555	0.00249	0.001313	0.000487	0.000722	0.000997	0.001971	0	0.029923	0.000907091
Carbohydrates	Monosaccharides	D-galactonate catabolism	2-dehydro-3-deoxygalactonokinase (EC 2.7.1.58)	0	0	0.002555	0	0.002626	0.001461	0.001444	0.003987	0.001971	0.004406	0.000116	0.000157755
Carbohydrates	Monosaccharides	D-galactarate, D-glucarate and D-glycerate catabolism	2-dehydro-3-deoxyglucarate aldolase (EC 4.1.2.20)	0.004526	0.004524	0.002555	0	0.000657	0.001948	0.00361	0.000997	0.001752	0	0	0.001064846

Carbohydrates	Monosaccharides	D-galactonate catabolism	2-dehydro-3-deoxyphosphogalactonate aldolase (EC 4.1.2.21)	0	0.002262	0.00511	0.00249	0.003939	0.00487	0.005055	0.003987	0.004598	0	0	0.00094653
Miscellaneous	Plant-Prokaryote DOE project	At5g38900	2-hydroxychromene-2-carboxylate isomerase/DsbA-like thioredoxin domain	0.002785	0	0	0.00249	0.000657	0.001461	0	0.00299	0.004379	0.002203	0.000349	0.001380356
Metabolism of Aromatic Compounds	Metabolism of central aromatic intermediates	Central meta-cleavage pathway of aromatic compound degradation	2-hydroxymuconic semialdehyde hydrolase (EC 3.7.1.9)	0	0.006786	0	0.00747	0.001313	0	0.002166	0.000498	0.005255	0.002203	0.000349	0.001735305
Metabolism of Aromatic Compounds	Anaerobic degradation of aromatic compounds	Anaerobic benzoate metabolism	2-ketocyclohexanecarboxyl-CoA hydrolase (EC 4.1.3.36)	0.008356	0.009048	0	0	0.004596	0.011202	0.005777	0.000997	0.001971	0.004406	0	0.004219947
Carbohydrates	Monosaccharides	L-Arabinose utilization	2-ketoglutaric semialdehyde dehydrogenase (EC 1.2.1.26)	0	0	0.007664	0	0.003939	0.003409	0.002888	0.003489	0.008758	0.004406	0.000466	0.001419795
Carbohydrates	Organic acids	2-methylcitrate to 2-methylaconitate metabolism cluster	2-methylaconitate racemase	0.002089	0.002262	0	0.00498	0.00197	0.007306	0.004333	0.006977	0.008758	0	0	0.005008722
Metabolism of Aromatic Compounds	NULL	Benzoate transport and degradation cluster	2-oxo-hepta-3-ene-1,7-dioic acid hydratase (EC 4.2.-.-)	0.001393	0	0.002555	0	0.002626	0.002435	0.001444	0.000498	0.007663	0	0.032951	0.007453925
Secondary Metabolism	Aromatic amino acids and derivatives	Cinnamic Acid Degradation	3-(3-hydroxy-phenyl)propionate hydroxylase (EC 1.14.13.-)	0.000348	0.002262	0.00511	0	0	0.000487	0.001444	0.000498	0.003065	0	0.000466	0.000907091
Fatty Acids, Lipids, and Isoprenoids	Fatty acids	Phospholipid and Fatty acid biosynthesis related cluster	3-oxoacyl-[ACP] synthase (EC 2.3.1.41) FabV like	0	0	0.00511	0.00249	0.003283	0.001461	0.002166	0.00299	0.003503	0	0.000116	0.002366325
Secondary Metabolism	Biosynthesis of phenylpropanoids	Flavanone biosynthesis	4-coumarate--CoA ligase 1 (EC 6.2.1.12)	0.000696	0	0.002555	0	0.000657	0.001948	0.001444	0.00299	0.00416	0	0.000233	0.00126204
Cofactors, Vitamins, Prosthetic Groups, Pigments	Tetrapyrroles	Coenzyme B12 biosynthesis	5,6-dimethylbenzimidazole synthase, flavin destructase family	0	0	0.00511	0	0.003283	0.000974	0.002888	0.001495	0.002408	0.008811	0.000233	0.003707243
Miscellaneous	Plant-Prokaryote DOE project	COG0523	50S ribosomal subunit maturation GTPase RbgA (B. subtilis YlqF)	0.002785	0	0	0	0.00197	0.000974	0.005777	0.005482	0.009634	0.01542	0.034697	0.006822905
Metabolism of Aromatic Compounds	Metabolism of central aromatic intermediates	4-Hydroxyphenylacetic acid catabolic pathway	5-carboxymethyl-2-hydroxymuconate delta-isomerase (EC 5.3.3.10)	0	0.004524	0.002555	0	0.005252	0.003896	0.001444	0.006977	0.006131	0.002203	0	0.005008722
Nucleosides and Nucleotides	NULL	Adenosyl nucleosidases	5'-methylthioadenosine nucleosidase (EC 3.2.2.16)	0	0	0.002555	0.00249	0.002626	0	0.002166	0.002492	0.001971	0.006608	0.019328	0.003391733
Carbohydrates	Di- and oligosaccharides	Beta-Glucoside Metabolism	6-phospho-beta-glucosidase (EC 3.2.1.86)	0.010097	0.004524	0.00511	0.01245	0.003283	0.000974	0.000722	0.000498	0	0	0	0.003312855
Miscellaneous	Plant-Prokaryote DOE project	COG0398	ABC transporter, permease protein YnjC	0.002785	0	0.00511	0	0.003283	0.003409	0.005055	0.007974	0.005912	0	0.001164	0.001183163
Iron acquisition and metabolism	NULL	Heme, hemin uptake and utilization systems in GramNegatives	ABC-type Fe3+-siderophore transport system permease component	0	0.002262	0	0.00249	0.000657	0.000487	0.002166	0.001495	0.001752	0	0.000116	0.004141069
Iron acquisition and metabolism	NULL	Heme, hemin uptake and utilization systems in GramNegatives	ABC-type hemin transport system, ATPase component	0.001044	0.002262	0.00511	0	0.000657	0.002922	0.002888	0.002492	0.003941	0	0	0.001814183
Clustering-based subsystems	NULL	USS-DB-6	ABC-type transport system involved in resistance to organic solvents, permease component USSDB6A	0.000348	0	0	0	0.002626	0.003409	0.000722	0.008472	0.010072	0.013217	0.000349	0.004456579
Metabolism of Aromatic Compounds	Anaerobic degradation of aromatic compounds	Acetophenone carboxylase 1	Acetophenone carboxylase subunit Apc3	0.002437	0.002262	0.00511	0.00249	0.000657	0.000487	0	0	0.001095	0	0.000349	0.001380356
Carbohydrates	Monosaccharides	Xylose utilization	Acetyl xylan esterase (EC 3.1.1.41)	0.006615	0.009048	0	0	0.00197	0.000487	0.002166	0.000498	0.001095	0.004406	0	0.001656428
Carbohydrates	Central carbohydrate metabolism	TCA Cycle	Aconitate hydratase X, predicted	0	0.006786	0	0.01245	0.004596	0.001461	0.002166	0.000498	0.000219	0	0.000582	0.001183163
Carbohydrates	Central carbohydrate metabolism	Ethylmalonyl-CoA pathway of C2 assimilation	Acyltransferase family protein associated with ethylmalonyl-CoA pathway	0.003482	0.004524	0.010219	0	0.007222	0.006332	0.004333	0.006977	0.002846	0	0	0.002405764
Membrane Transport	NULL	Transport of Nickel and Cobalt	Additional periplasmic component NikK of nickel ECF transporter	0.025416	0.01131	0	0.01245	0.010505	0.017046	0.008665	0.00299	0.000657	0	0	0.005324232
RNA Metabolism	RNA processing and modification	tRNA splicing	ADP-ribose 1"-phosphate phophatase	0.001044	0	0	0.00249	0.003283	0.003896	0.005055	0.001495	0.002627	0	0.000233	0.002681835
Motility and Chemotaxis	NULL	Bacterial Chemotaxis	Aerotaxis sensor receptor protein	0.034468	0	0	0.00498	0.002626	0.004383	0.000722	0.001495	0.009415	0	0.027129	0.015893818
Sulfur Metabolism	Organic sulfur assimilation	Alkanesulfonates Utilization	Alkanesulfonates-binding protein	0.000696	0	0	0.00249	0.000657	0.001948	0.000722	0.000498	0.000876	0	0.000116	0.002011377
DNA Metabolism	DNA repair	DNA repair, bacterial	Alkylated DNA repair protein AlkB	0	0	0.002555	0	0.003283	0.004383	0.002888	0.00598	0.01029	0.004406	0.000466	0.00283959
Regulation and Cell signaling	Proteolytic pathway	Coagulation cascade	Alpha-2-macroglobulin, protease inhibitor I39.001	0.002437	0.01131	0.00511	0	0.007879	0.003896	0	0.001993	0.002189	0.008811	0	0.00157755
Respiration	Electron accepting reactions	Terminal cytochrome C oxidases	Alternative cytochrome c oxidase polypeptide CoxM (EC 1.9.3.1)	0	0	0.002555	0.00747	0.003283	0.000974	0.007943	0.004984	0.004817	0.002203	0	0.002169132
Respiration	Electron accepting reactions	Terminal cytochrome C oxidases	Alternative cytochrome c oxidase polypeptide CoxP (EC 1.9.3.1)	0	0.01131	0.002555	0	0.00197	0.002435	0.00361	0.00897	0.012699	0.01542	0	0.016091012
Cofactors, Vitamins, Prosthetic Groups, Pigments	Folate and pterines	p-Aminobenzoyl-Glutamate Utilization	Aminobenzoyl-glutamate transport protein	0.004874	0.004524	0	0	0.001313	0.007793	0.008665	0.011462	0.011823	0	0.015835	0.003904437
Nitrogen Metabolism	NULL	Ammonia assimilation	Ammonium transporter family	0.001393	0.002262	0	0.00249	0.002626	0.003409	0	0.003489	0.002189	0	0.000582	0.002248009
Nucleosides and Nucleotides	NULL	AMP to 3-phosphoglycerate	AMP phosphohydrolase	0.001393	0.002262	0	0.00498	0.003283	0.003409	0.002166	0	0.000438	0	0.000349	0.002050815
Nitrogen Metabolism	NULL	Nitrosative stress	Anaerobic nitric oxide reductase flavorubredoxin	0.0094	0.004524	0	0.00747	0.005909	0.002435	0	0.000997	0.00832	0	0.000233	0.005994691
Regulation and Cell signaling	Programmed Cell Death and Toxin-antitoxin Systems	Murein hydrolase regulation and cell death	Antiholin-like protein LrgA	0	0	0.002555	0.00498	0.000657	0.000974	0.000722	0.003489	0.006131	0	0.007452	0.001222601
Stress Response	NULL	SigmaB stress responce regulation	anti-sigma B factor RsbT	0.009748	0.002262	0.00511	0	0.003283	0.004383	0.005777	0	0.000438	0	0.000116	0.001064846
Carbohydrates	Monosaccharides	L-Arabinose utilization	Arabinose-proton symporter	0	0.004524	0	0.00498	0.000657	0.003409	0.008665	0.003987	0.002408	0	0.000116	0.00634964
DNA Metabolism	DNA replication	DNA replication, archaeal	Archaeal DNA polymerase I (EC 2.7.7.7)	0.002089	0.006786	0	0.00498	0.003283	0.000487	0.005777	0.001993	0.002627	0	0	0.001183163
RNA Metabolism	RNA processing and modification	Queuosine-Archaeosine Biosynthesis	archaeosine tRNA-ribosyltransferase (EC 2.4.2.-) type 1	0.002785	0	0.002555	0	0.003939	0.00487	0.001444	0.000997	0.002408	0	0.000466	0.001498673
Clustering-based subsystems	Clustering-based subsystems	Putative diaminopropionate ammonia-lyase cluster	Aspartate/ornithine carbamoyltransferase family protein protein YgeW	0.04352	0	0.007664	0.00249	0.000657	0.002435	0.002888	0.000997	0.000876	0	0	0.002957907
Stress Response	Osmotic stress	Ectoine biosynthesis and regulation	Aspartokinase (EC 2.7.2.4) associated with ectoine biosynthesis	0.004178	0	0.007664	0	0.004596	0.001948	0.005777	0.015449	0.012918	0	0.064737	0.006862343
Clustering-based subsystems	NULL	CBSS-176299.4.peg.1996A	ATP/GTP-binding site motif A	0.005571	0	0.00511	0	0.001313	0.002435	0.001444	0.004485	0.003503	0.002203	0	0.002918468
Cofactors, Vitamins, Prosthetic Groups, Pigments	Biotin	Biotin biosynthesis	ATPase component BioM of energizing module of biotin ECF transporter	0	0.004524	0.00511	0	0.002626	0.002435	0.002166	0.002492	0.001095	0	0.000349	0.001025408
Membrane Transport	NULL	ECF class transporters	ATPase component of general energizing module of ECF transporters	0.005222	0	0	0.00747	0.001313	0.003409	0.004333	0.003987	0.003941	0	0.000699	0.006546833
Membrane Transport	Protein secretion system, Type II	Widespread colonization island	ATPase with chaperone activity, associated with Flp pilus assembly	0.011141	0.004524	0	0	0.002626	0.001461	0	0.004984	0.005693	0.002203	0.000931	0.003667804
Membrane Transport	Protein secretion system, Type II	Widespread colonization island	ATP-dependent helicase, DEAD/DEAH box family, associated with Flp pilus assembly	0.002785	0	0.00511	0	0.003283	0.007793	0.004333	0.010964	0.009415	0	0.001281	0.002445203
Regulation and Cell signaling	Quorum sensing and biofilm formation	Autoinducer 2 (AI-2) transport and processing (lsrACDBFGE operon)	Autoinducer 2 (AI-2) modifying protein LsrG	0.002785	0.006786	0	0.00747	0.00197	0.000974	0.002166	0.000997	0.001314	0	0	0.002602958
Metabolism of Aromatic Compounds	Peripheral pathways for catabolism of aromatic compounds	Benzoate catabolism	Benzoate 1,2-dioxygenase (EC 1.14.12.10)	0.002089	0	0	0.00249	0.001313	0.003409	0.00361	0.003489	0.002627	0	0.000466	0.00094653
Metabolism of Aromatic Compounds	NULL	Benzoate transport and degradation cluster	Benzoate transport, ATPase component	0	0.002262	0.002555	0	0.000657	0.000974	0.000722	0.002492	0.003284	0	0.000233	0.001222601
Metabolism of Aromatic Compounds	NULL	Benzoate transport and degradation cluster	Benzoate transport, inner-membrane translocator precursor	0.002785	0	0.00511	0	0.005252	0.000974	0.001444	0.002492	0.002189	0	0.000233	0.000394388
Cell Wall and Capsule	Gram-Negative cell wall components	LOS core oligosaccharide biosynthesis	Beta-1,4-galactosyltransferase	0.002437	0.002262	0	0.00498	0.001313	0	0.001444	0.000997	0.000657	0	0.000233	0.00189306
Carbohydrates	Monosaccharides	D-Galacturonate and D-Glucuronate Utilization	Beta-glucuronidase (EC 3.2.1.31)	0.003133	0.004524	0.002555	0.00498	0.000657	0.000974	0	0.001495	0.002408	0	0	0.003391733
Cofactors, Vitamins, Prosthetic Groups, Pigments	Biotin	Biotin biosynthesis	Biotin operon repressor	0.000696	0	0.002555	0	0.003939	0.000974	0.002888	0.00299	0.002408	0.002203	0	0.002287448
Metabolism of Aromatic Compounds	Peripheral pathways for catabolism of aromatic compounds	Biphenyl Degradation	Biphenyl-2,3-diol 1,2-dioxygenase (EC 1.13.11.39)	0.00383	0.002262	0.010219	0.00498	0.000657	0	0.000722	0.000997	0.004379	0	0	0.001380356

Metabolism of Aromatic Compounds	Peripheral pathways for catabolism of aromatic compounds	Biphenyl Degradation	biphenyl-2,3-diol 1,2-dioxygenase III-related protein	0.029942	0	0	0.00249	0.002626	0.002922	0.000722	0.00299	0.002189	0	0.012109	0.002129693
Amino Acids and Derivatives	Lysine, threonine, methionine, and cysteine	Lysine fermentation	Butyrate-acetoacetate CoA-transferase subunit B (EC 2.8.3.9)	0	0.002262	0.00511	0.00249	0	0.000487	0.000722	0.001495	0	0.008811	0.000815	0.002326887
Cell Wall and Capsule	Capsular and extracellular polysacchrides	Serotype determining Capsular polysaccharide biosynthesis in Staphylococcus	Capsular polysaccharide synthesis enzyme Cap5F	0	0.004524	0	0.00249	0.000657	0.000487	0.000722	0	0.000438	0.002203	0.000116	0.002169132
Cell Wall and Capsule	Gram-Negative cell wall components	Core Oligosaccharide Glycosylation in Pseudomonas	Carbamoyltransferase in large core OS assembly cluster	0	0.006786	0.00511	0	0.001313	0.002922	0.001444	0.000997	0.000876	0	0.000233	0.003628365
Clustering-based subsystems	NULL	CBSS-314269.3.peg.1840	Carbon monoxide dehydrogenase large chain (EC 1.2.99.2) parolog without usual motifs	0.005222	0.002262	0.002555	0	0.002626	0.000974	0.002888	0.000997	0.005255	0	0	0.001419795
Miscellaneous	Plant-Prokaryote DOE project	At5g04520 AT1G06240	Carbonic anhydrases/acetyltransferases, isoleucine patch superfamily	0.001393	0.004524	0	0	0.00197	0.000487	0.005777	0.003489	0.006131	0	0.024568	0.003273417
Clustering-based subsystems	NULL	CBSS-314269.3.peg.1840	Carbon-monoxide dehydrogenase form II, large subunit (EC 1.2.99.2)	0.004526	0	0.017884	0.00249	0.005252	0.012176	0.005055	0.014452	0.02496	0	0	0.001932499
Carbohydrates	CO2 fixation	CO2 uptake, carboxysome	carboxysome shell protein CsoS1	0.025764	0.002262	0	0.00249	0.001313	0.002435	0.002166	0.000997	0	0	0.000116	0.004969283
Carbohydrates	CO2 fixation	CO2 uptake, carboxysome	carboxysome shell protein CsoS2	0.014971	0.01131	0.007664	0	0.006566	0.005844	0.005055	0.003987	0.000438	0	0	0.008992036
DNA Metabolism	DNA replication	DNA replication, archaeal	Cell division control protein 6	0	0.002262	0	0	0.007879	0.005844	0.007221	0.004984	0.003065	0.004406	0.000233	0.001971938
Clustering-based subsystems	NULL	Bacterial Cell Division	Cell division initiation protein DivIVA	0.005571	0.002262	0.002555	0	0.001313	0.001948	0.001444	0.002492	0.000219	0	0	0.000985969
Cell Division and Cell Cycle	NULL	Bacterial Cytoskeleton	Cell division protein FtsB	0.001393	0	0	0	0.003283	0.001461	0.000722	0.00299	0.001095	0.002203	0.00978	0.001774744
Clustering-based subsystems	NULL	Bacterial Cell Division	Cell division topological specificity factor MinE	0	0.002262	0	0.00249	0.00197	0.001948	0	0.001993	0.004817	0.004406	0.000815	0.001695866
Motility and Chemotaxis	NULL	Bacterial Chemotaxis	Chemotaxis response - phosphatase CheZ	0	0	0	0.00249	0.005909	0.003896	0.004333	0.006977	0.007006	0.022028	0.006637	0.004969283
Carbohydrates	Aminosugars	(GlcNAc)2 Catabolic Operon	Chitin catabolic cascade sensor histidine kinase ChiS	0.001393	0.002262	0.00511	0	0.003283	0.000974	0.002888	0.002492	0.002408	0	0	0.000985969
Cofactors, Vitamins, Prosthetic Groups, Pigments	Tetrapyrroles	Chlorophyll Biosynthesis	Chlorophyll a synthase ChlG (EC 2.5.1.62)	0.002785	0	0.00511	0	0.001313	0.009741	0.011553	0.013954	0.012918	0	0.000815	0.000552143
Cofactors, Vitamins, Prosthetic Groups, Pigments	Tetrapyrroles	Chlorophyll Biosynthesis	Chlorophyllide reductase subunit BchY (EC 1.18.-.-)	0	0.006786	0	0	0.00197	0.01315	0.009387	0.010466	0.020143	0.002203	0.000815	0.000512704
Cofactors, Vitamins, Prosthetic Groups, Pigments	Tetrapyrroles	Chlorophyll Biosynthesis	Chlorophyllide reductase subunit BchZ (EC 1.18.-.-)	0	0.004524	0.00511	0	0.007879	0.019969	0.007943	0.021928	0.023865	0.004406	0	0.000473265
Cell Division and Cell Cycle	NULL	Cyanobacterial Circadian Clock	Circadian clock protein KaiC	0	0.006786	0.010219	0	0.005252	0.010715	0.011553	0.006977	0.010728	0	0.002678	0.007335608
Cofactors, Vitamins, Prosthetic Groups, Pigments	Tetrapyrroles	Coenzyme B12 biosynthesis	Cobalt-precorrin-6y C15-methyltransferase [decarboxylating] (EC 2.1.1.-)	0	0.009048	0.00511	0	0.003283	0.004383	0.008665	0.007475	0.005474	0	0.001281	0.002445203
Cofactors, Vitamins, Prosthetic Groups, Pigments	Tetrapyrroles	Coenzyme B12 biosynthesis	Cobalt-precorrin-6y C5-methyltransferase (EC 2.1.1.-)	0	0.006786	0.007664	0	0.008535	0.007306	0.00361	0.007974	0.006568	0	0.000233	0.001814183
Respiration	Electron donating reactions	H2:CoM-S-S-HTP oxidoreductase	CoB--CoM-reducing hydrogenase (Sec) delta subunit	0	0.006786	0.00511	0.00249	0.002626	0.003896	0.000722	0.000997	0.000438	0	0	0.002287448
Respiration	Electron donating reactions	Hydrogenases	Coenzyme F420-reducing hydrogenase, alpha subunit	0.001393	0.006786	0	0.00996	0.003283	0.001948	0.00361	0.001495	0.001971	0	0	0.001538111
RNA Metabolism	RNA processing and modification	tRNA modification Archaea	COG1293, Predicted RNA-binding protein homologous to eukaryotic snRNP	0	0.002262	0.00511	0.00249	0.004596	0.001461	0.007221	0.001993	0.002846	0	0	0.001419795
Clustering-based subsystems	Fatty acid metabolic cluster	COG1399	COG1399 protein, clustered with ribosomal protein L32p	0	0.002262	0.002555	0	0.004596	0.000974	0.004333	0.002492	0.006787	0	0.007335	0.002248009
Miscellaneous	Plant-Prokaryote DOE project	COG1836	COG1836	0.001044	0.004524	0.00511	0.00249	0.002626	0.000974	0	0.000498	0.002408	0	0	0.00094653
Membrane Transport	NULL	Ton and Tol transport systems	Colicin I receptor precursor	0.002437	0.002262	0.002555	0	0.005909	0.005357	0.000722	0.00598	0.001971	0	0	0.001853621
Virulence, Disease and Defense	Bacteriocins, ribosomally synthesized antibacterial peptides	Tolerance to colicin E2	Conserved uncharacterized protein CreA	0	0.002262	0	0.00747	0.007879	0.001461	0.000722	0.001495	0.001752	0	0.019328	0.0031551
Virulence, Disease and Defense	Resistance to antibiotics and toxic compounds	Copper homeostasis	Copper chaperone	0.001393	0.002262	0	0	0.00197	0.002435	0.000722	0.000997	0.000219	0	0.000116	0.001301479
Nitrogen Metabolism	NULL	Denitrification	Copper-containing nitrite reductase (EC 1.7.2.1)	0.009052	0	0	0	0.001313	0.001461	0.008665	0.011462	0.014013	0.006608	0.001281	0.00189306
DNA Metabolism	CRISPs	CRISPRs	CRISPR-associated helicase Cas3, protein	0.001393	0.004524	0	0	0.015757	0.009254	0.007221	0.004984	0.003722	0.004406	0	0.00252408
DNA Metabolism	CRISPs	CRISPRs	CRISPR-associated protein, CT1134 family	0	0	0.007664	0.00249	0.006566	0.003409	0.006499	0.003987	0.002846	0	0.000815	0.002602958
DNA Metabolism	CRISPs	CRISPRs	CRISPR-associated protein, TM1801 family	0	0.002262	0	0	0.012475	0.007306	0.002888	0.005482	0.003503	0.002203	0.000116	0.003628365
DNA Metabolism	CRISPs	CRISPRs	CRISPR-associated RecB family exonuclease Cas4b	0	0.004524	0.002555	0	0.005909	0.003896	0.001444	0.003489	0.007006	0	0.001048	0.002445203
Carbohydrates	One-carbon Metabolism	Serine-glyoxylate cycle	crotonyl-CoA reductase	0.002437	0.002262	0.00511	0	0.003939	0.006332	0.002888	0.003489	0.004817	0	0	0.000591581
Miscellaneous	Plant-Prokaryote DOE project	COG3380 COG2907	Cryptochrome	0	0	0	0.00249	0.001313	0.011689	0.007943	0.032393	0.032185	0.026433	0.001397	0.005284793
Membrane Transport	Protein secretion system, Type VIII (Extracellular nucleation/precipitation pathway, ENP)	Curli production	Curli production assembly/transport component CsgG	0	0	0.002555	0	0.000657	0.001948	0.002166	0.004984	0.00832	0.004406	0.000116	0.0082427
Nitrogen Metabolism	NULL	Cyanate hydrolysis	Cyanate ABC transporter, ATP-binding protein	0.002785	0	0.002555	0	0.002626	0.003896	0.002888	0.001993	0.004379	0	0.001514	0.001459234
Nitrogen Metabolism	NULL	Cyanate hydrolysis	Cyanate ABC transporter, permease protein	0	0.006786	0.00511	0	0.005252	0.005844	0.00361	0.005482	0.006787	0	0.000466	0.002721274
Amino Acids and Derivatives	Arginine; urea cycle, polyamines	Cyanophycin Metabolism	Cyanophycinase 2 (EC 3.4.15.6)	0	0	0.002555	0.00498	0.005252	0.005357	0.005055	0.001993	0.003722	0.002203	0	0.001853621
Regulation and Cell signaling	NULL	cAMP signaling in bacteria	cyclolysin secretion ATP-binding protein	0.001044	0.002262	0	0.00498	0.003939	0	0.002888	0.000498	0.002627	0	0.005938	0.001380356
Miscellaneous	Plant-Prokaryote DOE project	At5g37530	Cysteine desulfurase CsdA-CsdE (EC 2.8.1.7), main protein CsdA	0	0.002262	0.002555	0	0.003283	0.001948	0.004333	0.002492	0.002189	0	0.025499	0.001538111
Protein Metabolism	Protein biosynthesis	tRNA aminoacylation, Cys	Cysteinyl-tRNA synthetase paralog 2	0.003133	0.002262	0	0.00498	0.004596	0.006332	0.002166	0.001495	0.000219	0	0	0.002800152
Respiration	NULL	Cytochrome B6-F complex	Cytochrome b6-f complex iron-sulfur subunit PetC1 (Rieske iron sulfur protein EC 1.10.99.1)	0	0	0.002555	0.00249	0.001313	0.000487	0.000722	0.000498	0.001533	0	0.000233	0.00031551
Nitrogen Metabolism	NULL	Nitrate and nitrite ammonification	Cytochrome c nitrite reductase, small subunit NrfH	0	0.004524	0.007664	0.00498	0.004596	0.004383	0.001444	0.00299	0.000219	0	0	0.000828214
Respiration	NULL	Soluble cytochromes and functionally related electron carriers	Cytochrome c552	0	0	0	0.00249	0.002626	0.000974	0.00361	0.003489	0.002627	0.004406	0.000116	0.00252408
Respiration	NULL	Soluble cytochromes and functionally related electron carriers	Cytochrome c552 precursor (EC 1.7.2.2)	0	0.009048	0	0.00249	0.006566	0.010228	0.005777	0.006479	0.001533	0	0.000233	0.008992036
Respiration	NULL	Biogenesis of c-type cytochromes	Cytochrome c-type biogenesis protein ResA	0.007311	0.006786	0.00511	0	0.00197	0.002922	0.000722	0.000997	0.001314	0	0	0.001538111
Respiration	Electron accepting reactions	trimethylamine N-oxide (TMAO) reductase	Cytochrome c-type protein NapC	0	0.002262	0.007664	0	0.005252	0.005357	0.005777	0.003987	0.002627	0	0.001747	0.006744027
Respiration	Electron accepting reactions	Terminal cytochrome O ubiquinol oxidase	Cytochrome O ubiquinol oxidase subunit III (EC 1.10.3.-)	0.005919	0.002262	0.002555	0	0.001313	0	0	0.000997	0.004598	0.008811	0.000466	0.003510049
Respiration	Electron donating reactions	Hydrogenases	cytochrome-c3 hydrogenase alpha chain	0.003482	0.006786	0.017884	0	0.003283	0.007793	0.004333	0.006977	0.010509	0	0	0.004141069
Respiration	Electron donating reactions	Respiratory dehydrogenases 1	D-amino acid dehydrogenase (EC 1.4.99.1) family protein in hydroxy-L-proline catabolic cluster	0	0.002262	0.002555	0	0.003939	0.000487	0.00361	0.003987	0.002846	0	0.000349	0.008637087
Fatty Acids, Lipids, and Isoprenoids	Isoprenoids	Archaeal lipids	Digeranylgeranylglycerophospholipid reductase	0.003133	0.002262	0	0.00747	0.005252	0.001948	0.010109	0.006479	0.006787	0	0	0.002879029
Carbohydrates	Central carbohydrate metabolism	Dihydroxyacetone kinases	Dihydroxyacetone kinase, ATP-dependent (EC 2.7.1.29)	0	0.002262	0	0.00498	0.000657	0.003409	0.000722	0.001993	0.001971	0	0.000233	0.002011377
Nucleosides and Nucleotides	Detoxification	Housecleaning nucleoside triphosphate pyrophosphatases	Dimeric dUTPase (EC 3.6.1.23)	0	0.002262	0	0.00498	0.004596	0.003409	0.007221	0.008472	0.002846	0.002203	0	0.007217292

Miscellaneous	Plant-Prokaryote DOE project	At5g04520 AT1G06240	Disulfide bond chaperones of the HSP33 family	0.002785	0.004524	0.010219	0.00249	0.001313	0.000487	0	0.000997	0.000876	0	0	0.001498673
Clustering-based subsystems	Oxidative stress	CoA-disulfide reductase (EC 1.8.1.14) containing cluster	Disulfide bond regulator	0.003482	0.004524	0.00511	0.00249	0.001313	0.000487	0	0	0.000876	0.002203	0	0.001419795
Carbohydrates	Central carbohydrate metabolism	Glycolate, glyoxylate interconversions	D-Lactate dehydrogenase, cytochrome c-dependent (EC 1.1.2.4)	0.010793	0	0.010219	0	0.005252	0.01315	0.010831	0.016944	0.021238	0	0.000582	0.009701934
Phages, Prophages, Transposable elements, Plasmids	Phages, Prophages	Phage replication	DNA polymerase, phage-associated	0.005222	0	0.002555	0	0.026262	0.059906	0.075819	0.069272	0.001533	0	0.000349	0.00126204
DNA Metabolism	NULL	DNA phosphorothioation	DNA sulfur modification protein DndB	0.001044	0	0.00511	0	0.00197	0.003409	0.002166	0.001993	0.004817	0.002203	0	0.006744027
DNA Metabolism	NULL	DNA phosphorothioation	DNA sulfur modification protein DndD	0.005222	0.009048	0	0	0.001313	0.001461	0.002166	0.001495	0.001095	0	0.000582	0.006783466
Miscellaneous	Plant-Prokaryote DOE project	At1g69340 At2g40600	DNA/RNA-binding protein Alba	0.001044	0	0.002555	0.01245	0.001313	0.001461	0.009387	0.000997	0.000657	0	0	0.001459234
DNA Metabolism	NULL	DNA structural proteins, bacterial	DNA-binding protein Fis	0.012882	0.002262	0	0	0.00197	0.000974	0.007943	0.006479	0.007663	0	0.010595	0.003628365
DNA Metabolism	NULL	DNA structural proteins, bacterial	DNA-binding protein HBsu	0	0.004524	0.00511	0	0.000657	0.002435	0.002166	0.005482	0.003284	0	0.000466	0.004022753
Carbohydrates	Monosaccharides	D-Tagatose and Galactitol Utilization	D-tagatose 3-epimerase (EC 5.3.1.-)	0.002437	0.004524	0.00511	0	0.00197	0.000974	0.001444	0.000498	0.002846	0	0	0.001380356
Iron acquisition and metabolism	NULL	Encapsulating protein for DyP-type peroxidase and ferritin-like protein oligomers	Encapsulating protein for a DyP-type peroxidase or ferritin-like protein oligomers	0	0.004524	0	0.00249	0.00197	0.001461	0.002888	0.001495	0.001095	0.002203	0	0.00094653
Respiration	Reverse electron transport	Archaeal membrane bound hydrogenases	Energy-conserving hydrogenase (ferredoxin), subunit A	0	0.004524	0.002555	0	0.000657	0.000974	0.002888	0.000997	0.000438	0.004406	0	0.003233978
Clustering-based subsystems	NULL	Cell wall related cluster	EpsI protein	0.005919	0.009048	0	0	0.000657	0.002435	0.002166	0.00299	0.000876	0	0.000233	0.002050815
Carbohydrates	Sugar alcohols	Ethanolamine utilization	Ethanolamine utilization polyhedral-body-like protein EutN	0.003133	0	0.002555	0.00249	0.00197	0.001461	0.000722	0.004485	0.002408	0	0	0.001025408
Fatty Acids, Lipids, and Isoprenoids	Fatty acids	Fatty acid degradation regulons	Fatty acid degradation regulator YsiA, TetR family	0.011489	0	0.002555	0.00498	0.007879	0.009741	0.005777	0.005482	0.000657	0	0	0.003273417
Respiration	Electron accepting reactions	Anaerobic respiratory reductases	Ferredoxin reductase	0	0	0.00511	0	0.003283	0.005357	0.000722	0.003987	0.007006	0.002203	0.019677	0.002129693
Iron acquisition and metabolism	NULL	Iron acquisition in Vibrio	Ferrichrome transport ATP-binding protein FhuC (TC 3.A.1.14.3)	0	0.006786	0.002555	0	0.00197	0.000487	0.005777	0.003489	0.003722	0	0.000466	0.002800152
Iron acquisition and metabolism	NULL	Transport of Iron	Ferrous iron transport protein A	0.005919	0.002262	0.010219	0	0.004596	0.005357	0.002888	0.00299	0.000876	0	0	0.001971938
RNA Metabolism	RNA processing and modification	tRNA modification Archaea	Fe-S OXIDOREDUCTASE (1.8.-.-) Wyeosine biosynthesis	0	0	0	0.00996	0.006566	0.001948	0.00361	0.00299	0.000219	0.004406	0.000349	0.002602958
Clustering-based subsystems	TldD cluster	CBSS-354.1.peg.2917	FIG005080: Possible exported protein	0.001741	0.004524	0	0	0.00197	0.000974	0.00361	0.003489	0.024303	0	0.000116	0.004022753
Dormancy and Sporulation	NULL	Sporulation Cluster	FIG006789: Stage V sporulation protein	0	0.004524	0.00511	0.00249	0.000657	0.000487	0.001444	0	0.000219	0	0.000233	0.00189306
Miscellaneous	Plant-Prokaryote DOE project	COG3380 COG2907	FIG024285: Hypothetical protein	0.007659	0.002262	0	0	0.001313	0.000487	0.002166	0.001495	0.002408	0.002203	0	0.000394388
Clustering-based subsystems	NULL	CBSS-291331.3.peg.3674	FIG026291: Hypothetical periplasmic protein	0	0.006786	0.002555	0.00249	0.001313	0.003896	0.002888	0.001993	0.006131	0	0	0.001498673
Clustering-based subsystems	NULL	CBSS-316273.3.peg.922	FIG037127: Two-component system sensor protein	0.011141	0.004524	0	0.00249	0.003939	0.001461	0.002166	0.002492	0.002408	0	0	0.003588927
Cell Wall and Capsule	Gram-Negative cell wall components	Lipopolysaccharide-related cluster in Alphaproteobacteria	FIG062860: Dehydrogenases with different specificities (related to short-chain alcohol dehydrogenases)	0	0	0.00511	0.00249	0.00197	0.002435	0.001444	0.007974	0.004817	0	0.000349	0.000788775
Clustering-based subsystems	NULL	CBSS-291331.3.peg.3674	FIG097052: Sugar transporter	0.001044	0.006786	0	0	0.016414	0.019482	0.019496	0.015948	0.008758	0	0.000116	0.005127038
Protein Metabolism	Protein degradation	Putative TldE-TldD proteolytic complex	FIG138315: Putative alpha helix protein	0	0.002262	0.002555	0.00498	0.003283	0.004383	0	0.000997	0.004817	0	0.000116	0.003667804
Virulence, Disease and Defense	NULL	C jejuni colonization of chick caeca	Flagellar biosynthesis protein FliR	0.002437	0	0	0.00249	0.006566	0.015585	0.007943	0.012957	0.012699	0	0.0085	0.0113978
Respiration	NULL	Flavocytochrome C	Flavocytochrome c flavin subunit	0.000696	0.004524	0	0.00498	0.002626	0.013637	0.005777	0.000498	0	0	0.000815	0.000591581
Membrane Transport	Protein secretion system, Type II	Widespread colonization island	Flp pilus assembly protein, pilin Flp	0.005919	0.002262	0.002555	0	0.000657	0.001461	0.002166	0.00598	0.005912	0	0	0.000749336
Respiration	NULL	Formate hydrogenase	Formate dehydrogenase related protein	0.00383	0	0.002555	0.00747	0.003283	0.000974	0.000722	0	0.001752	0.002203	0	0.00220857
Miscellaneous	Plant-Prokaryote DOE project	Experimental - Histidine Degradation	Formiminoglutamase (EC 3.5.3.8)	0	0.004524	0.002555	0	0.00197	0.002922	0	0.004984	0.011604	0.026433	0.020492	0.004969283
Carbohydrates	One-carbon Metabolism	Methanogenesis	Formylmethanofuran dehydrogenase subunit A (EC 1.2.99.5)	0	0.006786	0.007664	0.00498	0.008535	0.005844	0.002166	0.001993	0.003284	0	0	0.004496018
Carbohydrates	One-carbon Metabolism	Methanogenesis	Formylmethanofuran dehydrogenase subunit B (EC 1.2.99.5)	0.001393	0	0.002555	0	0.000657	0.000487	0	0.000498	0.000219	0.002203	0.000116	0.000749336
Carbohydrates	One-carbon Metabolism	Methanogenesis	Formylmethanofuran--tetrahydromethanopterin N-formyltransferase (EC 2.3.1.101)	0.002785	0	0.010219	0.00747	0.002626	0.003409	0.002166	0.001495	0.002189	0	0	0.001616989
DNA Metabolism	DNA repair	Uracil-DNA glycosylase	G:T/U mismatch-specific uracil/thymine DNA-glycosylase	0	0.004524	0.00511	0.00747	0.000657	0.002435	0.001444	0.000997	0.005255	0	0	0.010332954
Clustering-based subsystems	Putrescine/GABA utilization cluster-temporal,to add to SSs	GABA and putrescine metabolism from cluters	Gamma-glutamyl-GABA hydrolase	0.000348	0	0	0	0.001313	0.001461	0.002166	0.000997	0.002846	0.004406	0.000233	0.00031551
Phages, Prophages, Transposable elements, Plasmids	Gene Transfer Agent (GTA)	Gene Transfer Agent	Gene Transfer Agent (GTA) ORFG12	0	0.002262	0	0.00249	0.024293	0.001948	0.00361	0.006479	0.004598	0	0.000582	0.00094653
Phages, Prophages, Transposable elements, Plasmids	Gene Transfer Agent (GTA)	Gene Transfer Agent	Gene Transfer Agent NlpC/P60 family peptidase	0	0.002262	0.00511	0	0.001313	0.000487	0.002166	0.002492	0	0.004406	0.000349	0.000276071
Phages, Prophages, Transposable elements, Plasmids	Gene Transfer Agent (GTA)	Gene Transfer Agent	Gene Transfer Agent portal protein	0.001044	0	0	0.00249	0.004596	0.007793	0.007943	0.018439	0.004598	0	0.001863	0.001340918
Miscellaneous	Plant-Prokaryote DOE project	COG1836	Geranylgeranyl hydrogenase BchP	0	0.009048	0	0	0.003939	0.012663	0.005055	0.006479	0.006568	0.002203	0.000815	0.000512704
Clustering-based subsystems	Putative GGDEF domain protein related to agglutinin secretion	CBSS-323850.3.peg.3284	GGDEF and EAL domain proteins	0.002437	0	0.002555	0	0.00197	0.002922	0.001444	0.001495	0.005474	0.004406	0	0.001932499
Carbohydrates	Aminosugars	(GlcNAc)2 Catabolic Operon	GlcNAc phosphomutase (EC 5.4.2.3)	0.000348	0.006786	0.002555	0	0.000657	0.000974	0	0.000498	0	0.002203	0.00326	0.001222601
Carbohydrates	Central carbohydrate metabolism	Glycolysis and Gluconeogenesis	Glucose-6-phosphate isomerase, archaeal II (EC 5.3.1.9)	0.002785	0.006786	0.002555	0.00249	0.00197	0	0.005055	0.006977	0.001752	0	0	0.003391733
Amino Acids and Derivatives	Glutamine, glutamate, aspartate, asparagine; ammonia assimilation	Glutamate and Aspartate uptake in Bacteria	Glutamate Aspartate periplasmic binding protein precursor GltI (TC 3.A.1.3.4)	0	0	0.00511	0	0.000657	0.002435	0.000722	0.000997	0.001095	0.002203	0.000931	0.000709898
Amino Acids and Derivatives	Glutamine, glutamate, aspartate, asparagine; ammonia assimilation	Glutamate and Aspartate uptake in Bacteria	Glutamate Aspartate transport system permease protein GltK (TC 3.A.1.3.4)	0	0.004524	0	0.00249	0.002626	0.005844	0.00361	0.000997	0.004598	0	0.035629	0.004732651
Nitrogen Metabolism	NULL	Ammonia assimilation	Glutamine amidotransferase protein GlxB (EC 2.4.2.-)	0	0	0	0.00249	0.005252	0.001461	0.005055	0.00598	0.003941	0.002203	0.033766	0.001853621
Nitrogen Metabolism	NULL	Ammonia assimilation	Glutamine amidotransferase, class-II	0	0.01131	0	0.00747	0.005252	0.006332	0.002888	0.002492	0.003284	0.002203	0	0.005560864
Miscellaneous	Plant-Prokaryote DOE project	Biotin biosynthesis Experimental	Glutaredoxin 3 (Grx2)	0	0	0	0.00249	0.001313	0.004383	0.00361	0.001495	0.003065	0.002203	0.001979	0.001301479
Carbohydrates	Central carbohydrate metabolism	Entner-Doudoroff Pathway	Glyceraldehyde-3-phosphate: ferredoxin oxidoreductase (EC 1.2.7.6)	0.011489	0.009048	0.00511	0	0.005252	0.001461	0.002166	0.001993	0.000438	0	0	0.004929844
Regulation and Cell signaling	NULL	Orphan regulatory proteins	Glycine cleavage system transcriptional activator	0	0	0.002555	0	0.003283	0.001461	0.002888	0.004485	0.011604	0.004406	0.013273	0.002090254
Protein Metabolism	Selenoproteins	Glycine reductase, sarcosine reductase and betaine reductase	Glycine reductase component B gamma subunit (EC 1.21.4.2)	0	0.002262	0.010219	0	0.004596	0.002435	0.001444	0.00299	0.000438	0	0.000116	0.001222601
Carbohydrates	Central carbohydrate metabolism	Glycolate, glyoxylate interconversions	Glyoxylate reductase (EC 1.1.1.79)	0.002785	0.002262	0	0	0.006566	0.001461	0.002166	0.001993	0.003503	0.002203	0	0.001419795
Stress Response	NULL	Bacterial hemoglobins	Hemoglobin-like protein HbO	0	0	0.002555	0	0.00197	0.002922	0.002888	0.001495	0.003065	0.004406	0.000931	0.004180508
Amino Acids and Derivatives	Arginine; urea cycle, polyamines	Arginine and Ornithine Degradation	Histidine ABC transporter, ATP-binding protein HisP (TC 3.A.1.3.1)	0	0	0.007664	0.00747	0.002626	0.005844	0.004333	0.011462	0.010509	0	0.039587	0.006586272
Clustering-based subsystems	NULL	CBSS-176279.3.peg.1262	Histidine triad (HIT) nucleotide-binding protein,	0	0.009048	0.002555	0.00249	0.007879	0.004383	0.001444	0.002492	0.002627	0	0	0.002405764

			similarity with At5g48545 and yeast YDL125C (HNT1)												
Stress Response	Osmotic stress	Choline and Betaine Uptake and Betaine Biosynthesis	HTH-type transcriptional regulator BetI	0	0	0.002555	0.00747	0.000657	0.002435	0.000722	0.003489	0.00416	0	0.000699	0.004259386
Respiration	NULL	Formate hydrogenase	Hydrogenase-4 component F (EC 1.-.-.-)	0.002437	0	0	0	0.00197	0.000487	0.001444	0.000498	0.005693	0.01542	0.000116	0.003273417
Respiration	NULL	Formate hydrogenase	Hydrogenase-4 transcriptional activator	0.005222	0.004524	0.007664	0	0.006566	0.00487	0.004333	0.003987	0.006131	0	0	0.005048161
Carbohydrates	Aminosugars	Chitin and N-acetylglucosamine utilization	hypothetical oxidoreductase related to N-acetylglucosamine utilization	0.000348	0	0.00511	0.00249	0.005909	0.004383	0.000722	0.002492	0.001752	0	0	0.001064846
Clustering-based subsystems	NULL	CBSS-370552.3.peg.1240	Hypothetical protein DUF194, DegV family	0.034816	0.01131	0.002555	0.00249	0.001313	0	0.000722	0.001495	0.001971	0	0	0.00157755
Miscellaneous	Plant-Prokaryote DOE project	At1g48360	Hypothetical protein, restriction endonuclease-like VRR-NUC domain	0.003133	0	0	0.00249	0.000657	0.001948	0.00361	0.00299	0.005912	0	0.000466	0.003825559
Carbohydrates	NULL	Unknown sugar utilization (cluster yphABCDEFG)	Hypothetical zinc-type alcohol dehydrogenase-like protein YphC	0	0.006786	0	0.00249	0.001313	0.002435	0.001444	0.000498	0.000438	0	0.000116	0.001616989
Clustering-based subsystems	NULL	CBSS-316273.3.peg.922	Integral membrane protein CcmA involved in cell shape determination	0.001393	0	0	0.01743	0.005909	0.007793	0.007221	0.012957	0.004817	0	0.002329	0.003273417
Phages, Prophages, Transposable elements, Plasmids	Phages, Prophages	Staphylococcal phi-Mu50B-like prophages	Integrase [SA bacteriophages 11, Mu50B]	0.002437	0.004524	0.002555	0.00249	0.002626	0.001948	0.001444	0.000997	0	0	0	0.000828214
Phages, Prophages, Transposable elements, Plasmids	Phages, Prophages	Phage integration and excision	Integrase/recombinase (XerC/CodV family)	0.002089	0.002262	0	0.00249	0.007222	0.000974	0.002888	0.001495	0.000876	0	0	0.002366325
Iron acquisition and metabolism	Siderophores	Siderophore Yersiniabactin Biosynthesis	iron aquisition yersiniabactin synthesis enzyme (Irp1,polyketide synthetase)	0.001393	0	0	0.00249	0.001313	0.000974	0.002166	0.003987	0.001095	0.004406	0	0.001814183
Iron acquisition and metabolism	NULL	Heme, hemin uptake and utilization systems in GramPositives	Iron-dependent repressor IdeR/DtxR	0	0.002262	0.007664	0.00249	0.000657	0.000487	0.000722	0	0.001752	0	0.000349	0.001301479
Miscellaneous	Plant-Prokaryote DOE project	Iron-sulfur cluster assembly	Iron-sulfur cluster assembly scaffold protein IscU/NifU-like	0.000348	0.006786	0.00511	0	0.006566	0.005357	0.005055	0.000498	0	0	0.000116	0.003194539
Phages, Prophages, Transposable elements, Plasmids	Transposable elements	CBSS-203122.12.peg.188	ISPsy4, transposase	0.001741	0.002262	0.007664	0	0.006566	0.001461	0.002888	0.00299	0.002627	0	0	0.003076223
Carbohydrates	Monosaccharides	L-Arabinose utilization	L-2-keto-3-deoxyarabonate dehydratase (EC 4.2.1.43)	0.000348	0.002262	0.007664	0	0.000657	0	0.002888	0.000498	0.003722	0	0.000349	0.000512704
Carbohydrates	Monosaccharides	L-Arabinose utilization	L-arabinose-binding periplasmic protein precursor AraF (TC 3.A.1.2.2)	0.002437	0.002262	0	0.00249	0	0.000974	0	0.001495	0.004379	0.004406	0.000116	0.003076223
DNA Metabolism	DNA uptake, competence	Late competence	Late competence protein ComEA, DNA receptor	0.00383	0.002262	0	0	0.003939	0.000487	0.001444	0.000997	0.002408	0	0.000233	0.003036784
DNA Metabolism	DNA uptake, competence	Gram Positive Competence	Late competence protein ComEC, DNA transport	0.004526	0.002262	0.002555	0	0.000657	0.003896	0.00361	0.001495	0.002627	0	0	0.001380356
Amino Acids and Derivatives	Lysine, threonine, methionine, and cysteine	Lysine fermentation	L-beta-lysine 5,6-aminomutase alpha subunit (EC 5.4.3.3)	0	0.004524	0.007664	0	0.00197	0.001948	0.001444	0.001993	0.001314	0	0.000582	0.001853621
Cell Wall and Capsule	Capsular and extracellular polysacchrides	Legionaminic Acid Biosynthesis	Legionaminic acid biosynthesis protein PtmG	0	0.002262	0	0.00747	0.014444	0.00828	0.014442	0.011462	0.00416	0	0.000582	0.004535457
Amino Acids and Derivatives	Glutamine, glutamate, aspartate, asparagine; ammonia assimilation	Glutamine, Glutamate, Aspartate and Asparagine Biosynthesis	Leucine-responsive regulatory protein, regulator for leucine (or lrp) regulon and high-affinity branched-chain amino acid transport system	0	0.004524	0	0.00249	0.003283	0.000974	0.004333	0.009967	0.012042	0	0.005472	0.003194539
Carbohydrates	Monosaccharides	L-fucose utilization temp	L-fuco-beta-pyranose dehydrogenase	0	0.004524	0.007664	0	0	0.002435	0.002166	0.002492	0.005474	0.004406	0.001514	0.002326887
Carbohydrates	Monosaccharides	L-fucose utilization	L-fucose mutarotase	0.001741	0	0.007664	0	0.003283	0.005844	0.002888	0.001495	0.001095	0.002203	0	0.002287448
Carbohydrates	Monosaccharides	L-fucose utilization	L-fuculokinase (EC 2.7.1.51)	0.002437	0.004524	0.00511	0.00249	0	0.001948	0.000722	0.000498	0.000438	0	0	0.001183163
Carbohydrates	Monosaccharides	D-gluconate and ketogluconates metabolism	L-idonate 5-dehydrogenase (EC 1.1.1.264)	0.001741	0	0.002555	0.00498	0.001313	0.001948	0.000722	0	0.001971	0	0.000233	0.000985969
Cofactors, Vitamins, Prosthetic Groups, Pigments	Tetrapyrroles	Chlorophyll Biosynthesis	Light-independent protochlorophyllide reductase subunit B (EC 1.18.-.-)	0	0.01131	0.010219	0	0.002626	0.016559	0.008665	0.019436	0.030871	0	0.001281	0.00157755
Amino Acids and Derivatives	Lysine, threonine, methionine, and cysteine	Threonine degradation	low-specificity D-threonine aldolase	0.005571	0	0.020438	0.00498	0.002626	0.00487	0.000722	0.004485	0.005036	0	0	0.001971938
Clustering-based subsystems	NULL	CBSS-160492.1.peg.550	LppC putative lipoprotein	0.003482	0	0	0	0.003939	0.002922	0.002888	0.002492	0.008977	0.004406	0.007335	0.004456579
Carbohydrates	Monosaccharides	L-rhamnose utilization	L-rhamnose-proton symporter	0.001044	0	0.002555	0.01245	0.003939	0.004383	0.002888	0.001495	0.000876	0	0	0.003746682
Nitrogen Metabolism	NULL	Nitrogen fixation	LRV (FeS)4 cluster domain protein clustered with nitrogenase cofactor synthesis	0.002785	0.002262	0	0.00249	0.00197	0.000487	0.000722	0	0	0.004406	0.021307	0.001932499
Amino Acids and Derivatives	Alanine, serine, and glycine	Glycine and Serine Utilization	L-serine dehydratase, alpha subunit (EC 4.3.1.17)	0	0.002262	0	0.00498	0.000657	0.00487	0.001444	0.001495	0.000876	0	0.000349	0.001498673
Carbohydrates	Monosaccharides	D-Sorbitol(D-Glucitol) and L-Sorbose Utilization	L-sorbose 1-phosphate reductase (EC 1.1.1.-)	0.036557	0.004524	0.007664	0.00249	0.00197	0.001461	0.001444	0	0.000219	0	0	0.002721274
Protein Metabolism	Protein biosynthesis	Ribosome LSU eukaryotic and archaeal	LSU ribosomal protein L1e (L4p)	0	0.002262	0.002555	0	0.001313	0.004383	0.002888	0.000997	0.002846	0.002203	0	0.001498673
Miscellaneous	Plant-Prokaryote DOE project	At1g14345	LSU ribosomal protein L34p	0.010445	0	0.002555	0.01245	0.004596	0.007793	0.007221	0.020931	0.015326	0	0	0.007651118
Protein Metabolism	Protein biosynthesis	Ribosome LSU eukaryotic and archaeal	LSU ribosomal protein L3e (L3p)	0	0	0.002555	0.01743	0.007879	0.001948	0.002888	0.000997	0.000876	0	0.000582	0.002326887
RNA Metabolism	RNA processing and modification	rRNA modification Archaea	LSU ribosomal protein L7Ae	0	0.01131	0	0.01245	0.006566	0.005844	0.008665	0.001993	0	0.002203	0.000233	0.001853621
RNA Metabolism	RNA processing and modification	RNA methylation	LSU rRNA 2'-O-methyl-C2498 methyltransferase RlmM	0.004874	0	0.002555	0	0.001313	0	0.001444	0.002492	0.008758	0.017622	0.018513	0.001695866
Fatty Acids, Lipids, and Isoprenoids	Isoprenoids	Carotenoids	Lycopene cyclase	0.000696	0	0.00511	0	0.003283	0.000974	0.001444	0.00299	0.005036	0	0.000116	0.003431172
Amino Acids and Derivatives	Lysine, threonine, methionine, and cysteine	Lysine degradation	Lysine/cadaverine antiporter membrane protein CadB	0.009748	0.006786	0.002555	0	0	0.001948	0.000722	0.00299	0.000657	0	0.000116	0.002090254
Fatty Acids, Lipids, and Isoprenoids	Triacylglycerols	Triacylglycerol metabolism	Lysophospholipase (EC 3.1.1.5)	0	0.013572	0.00511	0	0.011818	0.010228	0.005055	0.013954	0.013137	0	0.000116	0.003076223
Membrane Transport	NULL	Transport of Manganese	Manganese ABC transporter, inner membrane permease protein SitC	0	0	0.002555	0.00498	0.015757	0.013637	0.012275	0.018439	0.008101	0	0.000582	0.002800152
Clustering-based subsystems	NULL	CBSS-243277.1.peg.4359	Membrane-bound lytic murein transglycosylase B (EC 3.2.1.-)	0.000348	0.002262	0	0	0.001313	0.003896	0.00361	0.005482	0.011604	0	0.008616	0.00189306
Cofactors, Vitamins, Prosthetic Groups, Pigments	Quinone cofactors	Menaquinone Biosynthesis via Futalosine -- gjo	Menaquinone via futalosine step 1	0	0.002262	0.007664	0	0.001313	0.003409	0.000722	0.000997	0.000876	0.002203	0	0.004811528
Phosphorus Metabolism	NULL	Alkylphosphonate utilization	Metal-dependent hydrolase involved in phosphonate metabolism	0.003482	0	0	0.00498	0.006566	0.011689	0.02094	0.013456	0.008101	0	0.017931	0.002326887
Clustering-based subsystems	NULL	PA0057 cluster	Metallo-beta-lactamase superfamily protein PA0057	0.001044	0	0.007664	0	0.001313	0.001948	0.002166	0.000997	0	0.002203	0.050998	0.001774744
Clustering-based subsystems	NULL	Shikimate kinase containing cluster	Methyl-directed repair DNA adenine methylase (EC 2.1.1.72)	0.002785	0.002262	0	0	0.004596	0.001948	0.007221	0.003489	0.01029	0	0.009548	0.003667804
Cofactors, Vitamins, Prosthetic Groups, Pigments	Tetrapyrroles	Chlorophyll Biosynthesis	Mg-protoporphyrin O-methyltransferase (EC 2.1.1.11)	0	0.004524	0.002555	0	0.003939	0.006332	0.00361	0.000498	0.004379	0	0.000466	0.000552143
Clustering-based subsystems	NULL	CBSS-290633.1.peg.1906	Mlr7403 protein	0.003133	0	0	0.00249	0.00197	0.000974	0.000722	0.004984	0.007663	0	0.000466	0.002326887
Clustering-based subsystems	Flagella protein?	CBSS-323098.3.peg.2823	MotA/TolQ/ExbB proton channel family protein, probably associated with flagella	0.044216	0	0	0	0.007222	0.002922	0.012998	0.008472	0.01445	0.006608	0.000233	0.001656428
Virulence, Disease and Defense	Resistance to antibiotics and toxic compounds	Multidrug Resistance Efflux Pumps	Multidrug efflux pump component MtrF	0	0	0.007664	0	0.003939	0.005357	0.00361	0.009967	0.001752	0.006608	0.000466	0.003076223
Virulence, Disease and Defense	Resistance to antibiotics and toxic compounds	Copper homeostasis	Multidrug resistance transporter, Bcr/CflA family	0.001044	0	0	0	0.00197	0.001461	0.002888	0.004984	0.014232	0.002203	0.020958	0.002681835
Cell Wall and Capsule	Gram-Negative cell wall components	Lipopolysaccharide-related cluster in Alphaproteobacteria	Murein endopeptidase	0.004526	0.01131	0	0.00747	0.005909	0.000487	0	0.003489	0.004379	0	0.000233	0.00157755
Carbohydrates	Sugar alcohols	Inositol catabolism	Myo-inositol 2-dehydrogenase 2 (EC 1.1.1.18)	0.002089	0	0.007664	0	0.000657	0.000487	0.000722	0.000498	0.001533	0	0.000466	0.00063102
Amino Acids and Derivatives	Lysine, threonine, methionine, and cysteine	Lysine biosynthesis AAA pathway 2	N-acetyl-lysine deacetylase (EC 3.5.1.-)	0	0.002262	0	0.00747	0.004596	0.002435	0.005055	0.00299	0.003284	0.008811	0	0.002484642

Amino Acids and Derivatives	Arginine; urea cycle, polyamines	Arginine Biosynthesis extended	N-acetylornithine carbamoyltransferase (EC 2.1.3.9)	0.002437	0.002262	0	0	0.000657	0.001461	0.001444	0.004485	0.004598	0	0.000582	0.001932499
Respiration	Electron donating reactions	Respiratory Complex I	NAD(P)H-quinone oxidoreductase chain 1	0.008008	0	0.002555	0	0.00197	0.003409	0.000722	0.001993	0.008101	0	0.001514	0.004101631
Stress Response	Oxidative stress	NADPH:quinone oxidoreductase 2	NADPH:quinone oxidoreductase 2	0	0.009048	0.002555	0	0.006566	0.000974	0	0.001993	0.001533	0.006608	0.001281	0.001183163
Membrane Transport	NULL	Transport of Nickel and Cobalt	Nicel/Cobalt-specific TonB-dependent outer membrane receptor	0.002437	0.002262	0	0	0.002626	0.000487	0.001444	0.001495	0.001095	0.002203	0	0.000276071
Cofactors, Vitamins, Prosthetic Groups, Pigments	NAD and NADP	NAD and NADP cofactor biosynthesis global	Nicotinamidase/isochorismatase family protein	0.005919	0	0.002555	0	0.001313	0.004383	0.006499	0.007475	0.001752	0	0.000815	0.001301479
Nitrogen Metabolism	NULL	Nitrogen fixation	NifX-associated protein	0	0.004524	0	0.00249	0	0.003409	0.005777	0.001993	0.001752	0.013217	0.018164	0.003352294
Nitrogen Metabolism	NULL	Denitrification	Nitric-oxide reductase subunit C (EC 1.7.99.7)	0.002437	0.006786	0	0	0.003283	0.000487	0.000722	0.004485	0	0.011014	0.006404	0.00603413
Secondary Metabolism	Plant Hormones	Auxin biosynthesis	Nitrilase 1 (EC 3.5.5.1)	0.003133	0.002262	0.007664	0	0.001313	0.000974	0.001444	0.000997	0.002189	0	0	0.004259386
Nitrogen Metabolism	NULL	Dissimilatory nitrite reductase	Nitrite reductase associated c-type cytochorome NirN	0.0094	0	0.002555	0.00498	0.010505	0.003896	0.01372	0.007475	0.010947	0	0	0.00666515
Nitrogen Metabolism	NULL	Nitrogen fixation	Nitrogenase (vanadium-iron) transcriptional regulator VnfA	0.000696	0.002262	0	0.00498	0.001313	0.000974	0.000722	0.001495	0.001752	0	0	0.001774744
Nitrogen Metabolism	NULL	Nitrogen fixation	Nitrogenase FeMo-cofactor carrier protein NifX	0.000696	0.002262	0	0	0.002626	0.001461	0.000722	0.001495	0.000657	0	0.001048	0.00252408
Nitrogen Metabolism	NULL	Denitrification	NnrU family protein, required for expression of nitric oxide and nitrite reductases (Nir and Nor)	0	0.004524	0.007664	0	0.002626	0.001461	0	0.005482	0.003065	0.004406	0.00978	0.001143724
Carbohydrates	Central carbohydrate metabolism	Glycolysis and Gluconeogenesis	Non-phosphorylating glyceraldehyde-3-phosphate dehydrogenase (NADP) (EC 1.2.1.9)	0.002089	0	0	0	0.002626	0.000974	0.000722	0.001495	0.000438	0.002203	0.000466	0.003115662
Iron acquisition and metabolism	Siderophores	Siderophore Pyoverdine	Non-ribosomal peptide synthetase modules, pyoverdine	0.011837	0.004524	0	0.00249	0.003283	0.002922	0.011553	0.006977	0.008539	0	0	0.003628365
Membrane Transport	Protein secretion system, Type VI	Type VI secretion systems	Outer membrane protein ImpK/VasF, OmpA/MotB domain	0.000696	0.006786	0	0	0.001313	0.000487	0.001444	0.002492	0.004817	0	0.000233	0.001616989
Clustering-based subsystems	Probably Ybbk-related hypothetical membrane proteins	CBSS-316057.3.peg.659	Outer membrane protein/protective antigen OMA87	0	0	0.007664	0.00498	0.000657	0.003896	0.000722	0.001495	0.001314	0	0.000233	0.001459234
Sulfur Metabolism	Inorganic sulfur assimilation	Inorganic Sulfur Assimilation	Oxidoreductase probably involved in sulfite reduction	0.000348	0	0	0	0.00197	0.002435	0.006499	0.010466	0.006787	0.006608	0.000349	0.002879029
Carbohydrates	Monosaccharides	D-Galacturonate and D-Glucuronate Utilization	Pectate lyase precursor (EC 4.2.2.2)	0.002437	0	0.002555	0.00249	0.001313	0.002435	0.008665	0.001495	0.001533	0	0	0.002011377
Cofactors, Vitamins, Prosthetic Groups, Pigments	NULL	Molybdopterin cytosine dinucleotide	Periplasmic aromatic aldehyde oxidoreductase, FAD binding subunit YagS	0	0.002262	0	0.00249	0.00197	0.002922	0.002166	0.000997	0.002408	0	0.000582	0.001498673
Phages, Prophages, Transposable elements, Plasmids	Phages, Prophages	Phage tail fiber proteins	Phage tail fibers	0	0.002262	0	0.00249	0.011818	0.012176	0.007943	0.012459	0.002189	0	0.000116	0.00126204
Phages, Prophages, Transposable elements, Plasmids	Phages, Prophages	Phage packaging machinery	Phage terminase, large subunit	0	0.004524	0	0	0.03808	0.039937	0.036826	0.069272	0.002846	0.004406	0.000116	0.001971938
Metabolism of Aromatic Compounds	NULL	Phenylacetyl-CoA catabolic pathway (core)	Phenylacetate-CoA oxygenase, PaaH subunit	0.002089	0.002262	0.007664	0	0	0.001948	0.005777	0.007974	0.004817	0	0.018746	0.001932499
Cofactors, Vitamins, Prosthetic Groups, Pigments	Folate and pterines	Pterin metabolism 3	Phenylalanine-4-hydroxylase (EC 1.14.16.1) - Long	0	0.009048	0	0.00249	0.00197	0.001948	0.008665	0.003489	0.002846	0.006608	0	0.011240045
Phosphorus Metabolism	NULL	Alkylphosphonate utilization	PhnI protein	0.001044	0	0	0	0.005252	0.023378	0.007943	0.018439	0.005693	0.002203	0.030273	0.002563519
Miscellaneous	NULL	ZZ gjo need homes	Phosphatidylcholine synthase (EC 2.7.8.24)	0.002785	0	0	0.00498	0.004596	0.001461	0.000722	0.003489	0.002408	0	0.000815	0.00094653
Membrane Transport	ABC transporters	ABC transporter alkylphosphonate (TC 3.A.1.9.1)	Phosphonate ABC transporter permease protein phnE1 (TC 3.A.1.9.1)	0.003482	0	0.002555	0	0.007879	0.011202	0.005777	0.013954	0.005474	0	0.00163	0.003312855
Phosphorus Metabolism	NULL	Phosphoenolpyruvate phosphomutase	Phosphonopyruvate decarboxylase (EC 4.1.1.82)	0.001044	0.002262	0.010219	0	0.005252	0.003896	0.004333	0.004984	0.007882	0	0	0.001971938
Cofactors, Vitamins, Prosthetic Groups, Pigments	Coenzyme M	coenzyme M biosynthesis	Phosphosulfolactate synthase (EC 4.4.1.19)	0	0.002262	0.00511	0	0.000657	0.001948	0.000722	0.007475	0.005036	0.026433	0	0.001222601
Photosynthesis	Electron transport and photophosphorylation	Photosystem II-type photosynthetic reaction center	Photosynthetic reaction center M subunit	0	0.004524	0	0.00249	0.003939	0.002922	0.008665	0.009469	0.005255	0	0.000466	0.000749336
Photosynthesis	Electron transport and photophosphorylation	Photosystem I	photosystem I biogenesis protein BtpA	0	0.006786	0	0	0.00197	0.002922	0.000722	0.001993	0.000657	0.002203	0.000233	0.000670459
Photosynthesis	Electron transport and photophosphorylation	Photosystem II	photosystem II protein D1 (PsbA)	0	0	0	0.00249	0.006566	0.005844	0.000722	0.002492	0.006131	0.008811	0.001397	0.000552143
Fatty Acids, Lipids, and Isoprenoids	Isoprenoids	Carotenoids	Phytoene desaturase, pro-zeta-carotene producing (EC 1.-.-.-)	0.000696	0	0	0.00747	0.000657	0.000974	0.001444	0.001993	0.003941	0	0.000699	0.001498673
DNA Metabolism	DNA replication	Plasmid replication	Plasmid replication protein RepA	0.000348	0	0	0	0.000657	0.002435	0.000722	0.00299	0.01029	0.008811	0.00489	0.003036784
Cell Wall and Capsule	Gram-Positive cell wall components	Teichoic and lipoteichoic acids biosynthesis	Poly(glycerol-phosphate) alpha-glucosyltransferase (EC 2.4.1.52)	0	0.004524	0.015329	0.00996	0.005252	0.001461	0.00361	0.005482	0.003722	0	0	0.005087599
Respiration	Electron accepting reactions	Anaerobic respiratory reductases	polysulfide reductase, subunit A	0.000696	0	0.00511	0.00498	0.005252	0.006332	0.005777	0.003489	0.000657	0	0	0.002287448
Carbohydrates	Monosaccharides	L-fucose utilization	Possible fucose ABC transporter, substrate-binding component	0.002437	0	0.002555	0.00249	0	0.001461	0	0.000997	0.000219	0.004406	0.000349	0.000433826
Nucleosides and Nucleotides	Purines	De Novo Purine Biosynthesis	Possible subunit variant of phosphoribosylaminoimidazolecarboxamide formyltransferase [alternate form]	0.001044	0	0.002555	0.00249	0.00197	0.003409	0.002166	0.001495	0.000219	0	0	0.001538111
Dormancy and Sporulation	NULL	Sporulation Cluster	possible tetrapyrrole methyltransferase domain	0	0.006786	0	0	0.006566	0.002435	0.00361	0.000997	0.001971	0.002203	0.000466	0.00189306
Carbohydrates	Di- and oligosaccharides	Beta-Glucoside Metabolism	Predicted beta-glucoside specific TonB-dependent outer membrane receptor	0.001741	0	0	0.00249	0.001313	0.002435	0.001444	0.004485	0.03328	0	0.019794	0.000552143
Carbohydrates	Di- and oligosaccharides	Beta-Glucoside Metabolism	Predicted beta-glucoside-regulated ABC transport system, permease component 2, COG0395	0	0.002262	0.002555	0	0.002626	0.001948	0	0.000997	0.000876	0.002203	0.000466	0.001301479
Clustering-based subsystems	NULL	CBSS-160492.1.peg.550	Predicted endonuclease distantly related to archaeal Holliday junction resolvase	0	0.002262	0.007664	0	0.003283	0.001948	0.000722	0.00598	0.00416	0	0.006287	0.002681835
Carbohydrates	Monosaccharides	D-gluconate and ketogluconates metabolism	Predicted gluconate TRAP family transporter, DctM subunit	0.000348	0	0	0.00249	0.003939	0.00487	0.002888	0.000498	0.006787	0.013217	0	0.001301479
Carbohydrates	NULL	Sugar utilization in Thermotogales	Predicted glycosylase TM1225	0	0.009048	0	0	0.000657	0.000974	0.000722	0.000997	0.000657	0.004406	0.000466	0.000670459
Carbohydrates	NULL	Sugar utilization in Thermotogales	Predicted inosose dehydrogenase	0.003482	0.004524	0	0.00498	0.003283	0.000974	0.000722	0	0.000219	0	0.000233	0.001459234
RNA Metabolism	RNA processing and modification	tRNA modification Archaea	Predicted P-loop ATPase fused to an acetyltransferase COG1444	0.005571	0	0	0	0.002626	0.002922	0.006499	0.00598	0.010509	0.002203	0.000233	0.004417141
Carbohydrates	Sugar alcohols	Inositol catabolism	Predicted transcriptional regulator of the myo-inositol catabolic operon	0.001393	0	0.002555	0.00249	0.001313	0	0.000722	0.000997	0.001533	0.002203	0	0.00094653
Carbohydrates	Fermentation	Acetyl-CoA fermentation to Butyrate	Probable electron transfer flavoprotein-quinone oxidoreductase FixC (EC 1.5.5.-)	0.006963	0	0	0.00249	0.005252	0.002922	0.002166	0.010466	0.005912	0	0.000349	0.00252408
Clustering-based subsystems	Proteasome related clusters	Proteasome subunit alpha archaeal cluster	Probable exosome complex exonuclease 2 (EC 3.1.13.-)	0	0	0	0.00249	0.003283	0.003409	0.002166	0.001495	0.000657	0.011014	0.000349	0.002050815
Carbohydrates	Sugar alcohols	Glycerol and Glycerol-3-phosphate Uptake and Utilization	Probable glycerol transport protein	0.003482	0.006786	0.00511	0	0.00197	0.00487	0.002888	0.000498	0.000876	0	0	0.001774744
Nitrogen Metabolism	NULL	Nitrogen fixation	probable iron binding protein from the HesB IscA SufA family in Nif operon	0.001044	0	0.002555	0	0.001313	0.000974	0.000722	0.000498	0.001095	0	0.008034	0.00094653
Metabolism of Aromatic Compounds	Peripheral pathways for catabolism of aromatic compounds	Phenylpropanoid compound degradation	Probable VANILLIN dehydrogenase oxidoreductase (EC 1.-.-.-)	0.005222	0	0.002555	0.00498	0	0.002435	0.002166	0.001495	0.003722	0	0.000116	0.001616989
Regulation and Cell signaling	Programmed Cell Death and Toxin-antitoxin Systems	MazEF toxin-antitoxing (programmed cell death) system	Programmed cell death toxin MazF	0	0.006786	0.015329	0.00249	0.003283	0.006332	0.002166	0.001495	0.001533	0	0	0.002169132
Regulation and Cell signaling	Programmed Cell Death and Toxin-antitoxin Systems	MazEF toxin-antitoxing (programmed cell death) system	Programmed cell death toxin YdcE	0.000696	0	0	0.00996	0.007879	0.005357	0.004333	0.000997	0.003722	0	0.000815	0.002721274
Carbohydrates	Sugar alcohols	Propanediol utilization	Propanediol dehydratase large subunit (EC 4.2.1.28)	0.004526	0.004524	0	0.00249	0.004596	0.001461	0.000722	0	0.001314	0	0.000931	0.002129693
Carbohydrates	Organic acids	Methylcitrate cycle	Propionate catabolism operon regulatory protein PrpR	0	0.002262	0	0	0.000657	0.002435	0.001444	0.001993	0.003284	0.002203	0.001048	0.003076223

Carbohydrates	Organic acids	Methylcitrate cycle	Propionate catabolism operon transcriptional regulator of GntR family [predicted]	0	0	0.00511	0.00249	0.003283	0.002435	0.001444	0.000997	0.009853	0	0.027129	0.005836936
Protein Metabolism	Protein degradation	Proteasome archaeal	Proteasome subunit beta (EC 3.4.25.1), bacterial	0.003133	0	0.002555	0.00249	0.000657	0	0.002166	0.001495	0.001971	0.002203	0	0.000197194
Protein Metabolism	Protein degradation	Proteasome archaeal	Proteasome-activating AAA-ATPase (PAN), archaeal	0.00383	0.004524	0	0	0.004596	0.001948	0	0.001993	0.00416	0.022028	0.000116	0.003115662
Stress Response	NULL	Bacterial hemoglobins	Protein containing plastocyanin/azurin family domain	0.002437	0	0.007664	0	0.000657	0.001461	0.000722	0.001495	0.005036	0	0.001747	0.00094653
Metabolism of Aromatic Compounds	Peripheral pathways for catabolism of aromatic compounds	Phenylpropanoid compound degradation	Protein involved in meta-pathway of phenol degradation	0	0.006786	0	0.00249	0.000657	0.000487	0.002166	0.000997	0	0.01542	0.012226	0.00220857
Clustering-based subsystems	Pyruvate kinase associated cluster	CBSS-288000.5.peg.1793	protein of unknown function DUF1244	0	0	0.002555	0	0.000657	0.000487	0.000722	0.002492	0.00416	0.002203	0.014205	0.00189306
Clustering-based subsystems	Hypothetical in Lysine biosynthetic cluster	CBSS-323850.3.peg.3269	Protein of unknown function DUF484	0.012534	0	0	0.00249	0.001313	0.001461	0.001444	0.004485	0.00832	0.004406	0	0.002445203
Dormancy and Sporulation	NULL	Sporulation-associated proteins with broader functions	Protein of unknown function identified by role in sporulation (SpoVG)	0	0.01131	0.007664	0.00498	0.000657	0.003896	0.001444	0.001495	0.001314	0	0	0.002011377
Miscellaneous	NULL	ZZ gjo need homes	Protein secretion chaperonin CsaA	0.001393	0	0	0	0.003939	0.006819	0.007943	0.010466	0.007444	0.019825	0.000466	0.00729617
Cofactors, Vitamins, Prosthetic Groups, Pigments	Tetrapyrroles	Chlorophyll Biosynthesis	Protoporphyrin IX Mg-chelatase subunit D (EC 6.6.1.1)	0	0.015834	0	0.00498	0.00197	0.00828	0.005055	0.004984	0.007663	0	0.000815	0.000354949
Cell Division and Cell Cycle	NULL	Two cell division clusters relating to chromosome partitioning	Pseudouridine synthase family protein	0.003482	0	0	0.00498	0.00197	0.001948	0	0.001495	0.001971	0.002203	0.000349	0.001222601
Miscellaneous	Plant-Prokaryote DOE project	Experimental-PTPS	PTPS-like type 4	0	0	0.00511	0.00498	0.001313	0.003409	0.001444	0.003987	0.005693	0	0.000466	0.000907091
Carbohydrates	NULL	Sugar utilization in Thermotogales	PTS system, fructose-specific IIA component (EC 2.7.1.69)	0	0.002262	0	0.00249	0.005909	0.002435	0.004333	0.001495	0.003722	0	0.002212	0.003076223
Carbohydrates	Sugar alcohols	Mannitol Utilization	PTS system, mannitol-specific IIC component (EC 2.7.1.69)	0.000696	0	0	0.00747	0.002626	0.001461	0.002888	0.002492	0.000876	0	0.000233	0.001380356
Clustering-based subsystems	proteosome related	Cluster-based Subsystem Grouping Hypotheticals - perhaps Proteosome Related	Pup ligase PafA, possible component of postulated heterodimer PafA-PafA'	0.003482	0.002262	0	0.00249	0.00197	0.000974	0	0.000498	0.003065	0	0.000116	0.000788775
Clustering-based subsystems	Probably Ybbk-related hypothetical membrane proteins	CBSS-316057.3.peg.659	Putative activity regulator of membrane protease YbbK	0.001741	0.002262	0.00511	0	0.001313	0.000487	0	0.000498	0.001533	0	0.001979	0.00347061
Metabolism of Aromatic Compounds	Peripheral pathways for catabolism of aromatic compounds	Chlorobenzoate degradation	Putative benzaldehyde dehydrogenase oxidoreductase protein (EC 1.2.1.28)	0	0	0.007664	0	0.007222	0.001461	0.001444	0.000498	0.002189	0.006608	0.053676	0.001616989
Clustering-based subsystems	NULL	USS-DB-7	Putative cytoplasmic protein USSDB7A	0	0.004524	0	0.00249	0.004596	0.001461	0	0.000997	0.001533	0.008811	0.000116	0.001143724
DNA Metabolism	NULL	YcfH	Putative deoxyribonuclease similar to YcfH, type 2	0	0.004524	0.002555	0	0.000657	0.000974	0.000722	0.001993	0.001971	0.002203	0	0.000788775
DNA Metabolism	NULL	YcfH	Putative deoxyribonuclease YjjV	0.000696	0	0.010219	0	0.00197	0.000974	0.005055	0.00299	0.004598	0	0.001281	0.004693212
Amino Acids and Derivatives	Aromatic amino acids and derivatives	Aromatic amino acid degradation	Putative fumarylacetoacetate (FAA) hydrolase	0	0	0.002555	0.00249	0.003939	0.000487	0.000722	0.000997	0.001752	0.002203	0	0.00063102
Carbohydrates	Central carbohydrate metabolism	Glycolysis and Gluconeogenesis	Putative phosphoenolpyruvate synthase/pyruvate phosphate dikinase, C-terminal domain	0.006615	0.004524	0.007664	0.01245	0.00197	0.00487	0	0.000498	0.001752	0	0	0.00189306
Stress Response	NULL	SigmaB stress responce regulation	Putative SigmaB asociated two-component system sensor protein	0	0	0.00511	0.00249	0.003939	0.003409	0.002166	0.002492	0.001314	0.002203	0	0.002287448
Clustering-based subsystems	Carbohydrates	Cluster Ytf and putative sugar transporter	Putative sugar ABC transport system, ATP-binding protein YtfR (EC 3.6.3.17)	0.012882	0.009048	0.002555	0	0.001313	0.003896	0	0.00299	0.003284	0	0.000116	0.004338263
Clustering-based subsystems	Carbohydrates	Cluster Ytf and putative sugar transporter	Putative sugar ABC transport system, permease protein YjfF	0.002437	0.004524	0.00511	0	0.000657	0	0.001444	0.001495	0.000876	0	0.000233	0.002445203
Clustering-based subsystems	Oxidative stress	CoA-disulfide reductase (EC 1.8.1.14) containing cluster	Putative sulfide reductase	0	0.006786	0.00511	0	0.007222	0.004383	0.00361	0.000498	0.001533	0	0.000116	0.00157755
Cofactors, Vitamins, Prosthetic Groups, Pigments	Pyridoxine	Pyridoxin(Vitamin B6) Degradation Pathway	Pyridoxine 4-oxidase (EC 1.1.3.12)	0.003133	0	0	0	0.000657	0.000487	0.000722	0.000997	0.002189	0.002203	0.000116	0.000749336
Regulation and Cell signaling	Programmed Cell Death and Toxin-antitoxin Systems	Toxin-antitoxin replicon stabilization systems	RelE/StbE replicon stabilization toxin	0.000348	0.006786	0	0	0.00197	0.002922	0.00361	0.003987	0.000438	0.002203	0	0.003746682
Nitrogen Metabolism	NULL	Nitrate and nitrite ammonification	Respiratory nitrate reductase delta chain (EC 1.7.99.4)	0	0.009048	0.002555	0	0.00197	0.000974	0.00361	0.000498	0.003503	0	0.000116	0.000867653
Carbohydrates	Monosaccharides	L-rhamnose utilization	Rhamnulokinase (EC 2.7.1.5)	0	0.009048	0.040877	0.01494	0.007879	0.017533	0.001444	0.00598	0.007006	0	0	0.010175199
Carbohydrates	Monosaccharides	L-rhamnose utilization	Rhamnulokinase RhaK in alpha-proteobacteria (EC 2.7.1.5)	0.000696	0.002262	0	0	0.003283	0.002922	0.002166	0.005482	0.002846	0.002203	0	0.001301479
Miscellaneous	Plant-Prokaryote DOE project	Single-Rhodanese-domain proteins	Rhodanese domain protein, Enterobacterial subgroup, YceA homolog	0	0.002262	0.002555	0	0.000657	0.003896	0.00361	0.00299	0.006787	0.154195	0	0.004929844
RNA Metabolism	Transcription	Transcription initiation, bacterial sigma factors	RNA polymerase sigma factor SigB	0.001741	0	0	0.00498	0.00197	0	0.002166	0.000498	0.000876	0.002203	0.000233	0.001064846
Stress Response	Oxidative stress	Rubrerythrin	Rubredoxin-NAD(+) reductase (EC 1.18.1.1)	0.000696	0	0.00511	0	0.003939	0.006332	0.000722	0.003489	0.00416	0	0.000233	0.001735305
Amino Acids and Derivatives	Lysine, threonine, methionine, and cysteine	Lysine degradation	Saccharopine dehydrogenase [NAD+, L-lysine-forming] (EC 1.5.1.7)	0	0.002262	0	0.00249	0.001313	0.001461	0.001444	0.000997	0.000219	0	0.000233	0.001064846
Stress Response	Osmotic stress	Choline and Betaine Uptake and Betaine Biosynthesis	Sarcosine oxidase delta subunit (EC 1.5.3.1)	0.001044	0	0.007664	0	0.001313	0.004383	0.001444	0.006977	0.005255	0	0.018047	0.001340918
Regulation and Cell signaling	NULL	The Chv regulatory system of Alphaproteobacteria	Sensor histidine kinase ChvG (EC 2.7.3.-)	0.012882	0	0.002555	0	0.013788	0.013637	0.015886	0.022426	0.009634	0	0.001747	0.002681835
Miscellaneous	Plant-Prokaryote DOE project	COG0398	Sensor histidine kinase in cluster with mercury reductase	0.001044	0	0.002555	0	0.000657	0.004383	0.005055	0.00598	0.006568	0.004406	0	0.001143724
Regulation and Cell signaling	NULL	Orphan regulatory proteins	Sensory histidine kinase QseC	0	0.009048	0.002555	0.00498	0.003939	0.000974	0.002166	0.000498	0.003284	0	0	0.002011377
Cell Division and Cell Cycle	NULL	Bacterial Cytoskeleton	Septum site-determining protein MinC	0	0.006786	0.002555	0	0.003939	0.00487	0.005777	0.001993	0.007006	0	0.000699	0.003983314
Membrane Transport	Protein and nucleoprotein secretion system, Type IV	Type 4 conjugative transfer system, IncI1 type	Shufflon-specific DNA recombinase	0.002437	0.004524	0	0.00498	0.006566	0.004383	0.002166	0.003987	0.001533	0	0	0.002957907
Iron acquisition and metabolism	Siderophores	Siderophore assembly kit	Siderophore biosynthesis non-ribosomal peptide synthetase modules	0.002437	0.002262	0	0	0.005252	0.00487	0.000722	0.002492	0.007006	0	0.000233	0.002169132
Stress Response	Periplasmic Stress	Periplasmic Stress Response	Sigma factor RpoE negative regulatory protein RseB precursor	0	0	0.002555	0.00498	0.00197	0.001948	0.001444	0.001495	0.006349	0.002203	0	0.000907091
Regulation and Cell signaling	NULL	Two-component regulatory systems in Campylobacter	Signal-transduction regulatory protein FlgR	0.005919	0	0.002555	0.00249	0	0.000487	0	0.000498	0.001752	0.008811	0.001048	0.006152446
Stress Response	Oxidative stress	Glutathione: Non-redox reactions	Similar to Hydroxyacylglutathione hydrolase, but in an organism lacking glutathione biosynthesis	0	0	0.00511	0.00498	0.001313	0.001461	0.000722	0.003489	0.001095	0	0.000116	0.002011377
Cofactors, Vitamins, Prosthetic Groups, Pigments	Tetrapyrroles	Coenzyme B12 biosynthesis	Sirohydrochlorin cobaltochelatase CbiK (EC 4.99.1.3)	0.004178	0.002262	0.00511	0	0.003939	0.003409	0.00361	0.000997	0.000876	0	0	0.002326887
Miscellaneous	Plant-Prokaryote DOE project	At1g01770	Small uncharacterized protein Bpro 4170	0.004874	0.002262	0	0.00249	0	0.000487	0.001444	0.004984	0.003941	0	0.000116	0.000867653
Clustering-based subsystems	NULL	Spore Coat	Spore coat protein A	0.004178	0.004524	0	0.00249	0.004596	0.005357	0.002166	0.000498	0.00832	0	0	0.013961319
Dormancy and Sporulation	NULL	Spore Core Dehydration	Spore maturation protein B	0.000348	0	0.015329	0.00747	0.001313	0.000974	0	0.001495	0.001533	0	0.000699	0.00157755
Protein Metabolism	Protein biosynthesis	Ribosome SSU eukaryotic and archaeal	SSU ribosomal protein S11e (S17p)	0.002785	0.002262	0	0	0.001313	0.001461	0.000722	0.000997	0.000657	0	0.000116	0.00063102
Protein Metabolism	Protein biosynthesis	Ribosome SSU eukaryotic and archaeal	SSU ribosomal protein S23e (S12p)	0.002785	0	0	0.00249	0.004596	0.002435	0.005055	0.000498	0	0.004406	0.000931	0.00157755
Protein Metabolism	Protein biosynthesis	Ribosome SSU eukaryotic and archaeal	SSU ribosomal protein S5e (S7p)	0	0.004524	0	0.00249	0.001313	0.002435	0.005055	0.00299	0.001095	0.008811	0	0.001222601
RNA Metabolism	RNA processing and modification	16S rRNA modification within P site of ribosome	Stage V sporulation protein D (Sporulation-specific penicillin-binding protein)	0.000348	0.006786	0.010219	0.00249	0.005909	0.001948	0	0.000997	0.001971	0	0	0.000907091
Secondary Metabolism	Biologically active compounds in metazoan cell defence and differentiation	Steroid sulfates	Steryl-sulfatase precursor (EC 3.1.6.2)	0.004526	0.002262	0	0.00249	0.000657	0.001461	0.000722	0.000997	0.000219	0	0	0.001340918
Protein Metabolism	Protein biosynthesis	tRNA aminoacylation, Met	Structure-specific tRNA-binding protein	0.003482	0.002262	0	0.00747	0.003283	0.005844	0.006499	0.004485	0	0	0.001979	0.00126204

Amino Acids and Derivatives	Arginine; urea cycle, polyamines	Arginine and Ornithine Degradation	Succinylarginine dihydrolase (EC 3.5.3.23)	0	0	0	0.00498	0.00197	0.000974	0.005777	0.011961	0.026712	0.008811	0.031437	0.012580963
Amino Acids and Derivatives	Arginine; urea cycle, polyamines	Arginine and Ornithine Degradation	Succinylornithine transaminase (EC 2.6.1.81)	0	0.002262	0	0	0.001313	0.000974	0.002888	0.00299	0.007882	0.004406	0.022006	0.004811528
Sulfur Metabolism	NULL	Sulfate reduction-associated complexes	Sulfite reduction-associated complex DsrMKJOP multiheme protein DsrJ (=HmeF)	0.004526	0.004524	0.00511	0.00249	0.002626	0.007793	0.002166	0.000498	0	0	0	0.001143724
Sulfur Metabolism	Organic sulfur assimilation	Taurine Utilization	Taurine transport system permease protein TauC	0.000348	0	0	0.00249	0.00197	0.002922	0.000722	0.003489	0.004817	0	0.000349	0.001971938
Cofactors, Vitamins, Prosthetic Groups, Pigments	NULL	Thiamin biosynthesis	Thiazole biosynthetic enzyme Thi4	0	0.004524	0.002555	0.01245	0.00197	0.005357	0.004333	0.00299	0.000438	0	0	0.002050815
Protein Metabolism	Protein degradation	Putative TldE-TldD proteolytic complex	TldD-domain protein	0	0.006786	0.007664	0.00249	0.002626	0.001948	0.001444	0.003489	0.001314	0	0	0.00126204
Clustering-based subsystems	NULL	CBSS-316273.3.peg.448	TldE/PmbA family protein, Actinobacterial subgroup	0	0.002262	0.002555	0.00498	0.001313	0.000974	0	0.002492	0.002846	0	0.001048	0.001419795
RNA Metabolism	Transcription	Transcription factors bacterial	Transcriptional activator RfaH	0.002785	0	0.002555	0.00249	0.003283	0.003896	0.000722	0.004485	0.003503	0	0	0.00220857
Nucleosides and Nucleotides	Pyrimidines	Pyrimidine utilization	Transcriptional regulator RutR of pyrimidine catabolism (TetR family)	0.002785	0	0	0	0.000657	0.001461	0.00361	0.00598	0.002627	0.008811	0.001164	0.001380356
Regulation and Cell signaling	NULL	Orphan regulatory proteins	Transcriptional regulatory protein CitB, DpiA	0.000348	0	0.002555	0	0.000657	0.000487	0.000722	0.000498	0.000876	0	0.016068	0.002050815
Miscellaneous	Plant-Prokaryote DOE project	At4g17370	TRAP-type C4-dicarboxylate transport system, possibly of alkanesulfonates, large permease component	0	0.004524	0	0.00498	0.005252	0.006819	0.002888	0.003987	0.003722	0	0.001979	0.00283959
Cell Wall and Capsule	Capsular and extracellular polysacchrides	Sialic Acid Metabolism	TRAP-type transport system, small permease component, predicted N-acetylneuraminate transporter	0	0	0	0.00996	0.000657	0.000974	0.002166	0.001495	0.002408	0.004406	0.001048	0.003273417
Carbohydrates	Di- and oligosaccharides	Trehalose Uptake and Utilization	Trehalose 6-phosphate phosphorylase (EC 2.4.1.216)	0	0	0.012774	0	0.009192	0.00828	0.010831	0.003489	0.001095	0.002203	0.000582	0.005521426
Carbohydrates	One-carbon Metabolism	Methanogenesis from methylated compounds	Trimethylamine methyltransferase corrinoid protein	0.000348	0.004524	0	0.00249	0.003283	0.002922	0.000722	0.001495	0.000657	0	0	0.003233978
RNA Metabolism	RNA processing and modification	tRNA modification yeast cytoplasmic	tRNA (Uracil54-C5-)-methyltransferase (EC 2.1.1.35)	0.000348	0	0	0	0.001313	0.001948	0.002888	0.009469	0.015764	0.002203	0.042033	0.01329086
RNA Metabolism	RNA processing and modification	tRNA modification Bacteria	tRNA 5-methylaminomethyl-2-thiouridine synthase TusA	0.001044	0.002262	0	0	0.005252	0.005844	0.006499	0.003987	0.006787	0.006608	0	0.005403109
RNA Metabolism	RNA processing and modification	mnm5U34 biosynthesis bacteria	tRNA 5-methylaminomethyl-2-thiouridine synthase TusB	0.000696	0.004524	0	0.01245	0.001313	0.002435	0.001444	0.000498	0.000438	0	0	0.001498673
RNA Metabolism	RNA processing and modification	tRNA modification Archaea	tRNA m5C48-49 methylase	0.001044	0	0.002555	0.00498	0.001313	0.001948	0.002166	0.000498	0.001971	0	0	0.001853621
RNA Metabolism	RNA processing and modification	tRNA modification Bacteria	tRNA-(ms[2]io[6]A)-hydroxylase (EC 1.-.-.-)	0	0	0.007664	0	0.005909	0.00487	0.008665	0.013954	0.013575	0.024231	0.006986	0.01171331
Miscellaneous	NULL	Archease2	tRNA/RNA cytosine-C5-methylase (EC 2.1.1.-)	0.002089	0	0	0.00747	0.002626	0.001461	0.000722	0.000997	0.002408	0	0.000116	0.001774744
Regulation and Cell signaling	Programmed Cell Death and Toxin-antitoxin Systems	Murein hydrolase regulation and cell death	tRNA-dihydrouridine synthase C (EC 1.-.-.-)	0	0.009048	0	0	0.001313	0.002922	0.002166	0.006479	0.005912	0.01542	0.030855	0.005797497
Iron acquisition and metabolism	NULL	Heme, hemin uptake and utilization systems in GramPositives	Two-component sensor kinase SA14-24	0.001393	0.006786	0	0.00249	0.007879	0.001948	0.004333	0.001495	0.006349	0	0	0.006191885
Membrane Transport	Protein secretion system, Type I	Type I secretion system for aggregation	type I secretion system, membrane fusion protein, HlyD family (LapC)	0	0	0	0.00498	0.001313	0.005844	0.000722	0.001993	0.003284	0.002203	0.04995	0.007414486
Virulence, Disease and Defense	NULL	Streptococcus agalactiae virulome	Tyrosine-protein kinase EpsD (EC 2.7.10.2)	0	0.006786	0	0.00498	0.00197	0.003409	0.00361	0.004485	0.006131	0	0.000349	0.004417141
Clustering-based subsystems	NULL	Cell wall related cluster	UDP-N-acetylglucosamine 2-epimerase (EC 5.1.3.14) 2	0.006267	0	0	0.00747	0.00197	0.002922	0.001444	0.002492	0.003284	0.002203	0	0.002957907
Cell Wall and Capsule	NULL	Peptidoglycan biosynthesis--gjo	UDP-N-acetylmuramoylalanyl-D-glutamate--L,L-2,6-diaminopimelate ligase	0.003482	0	0.002555	0.00249	0.003939	0.001948	0.000722	0.001495	0.001533	0	0	0.000828214
Stress Response	Oxidative stress	Glutathione: Non-redox reactions	Uncharacterized glutathione S-transferase-like protein	0	0.004524	0.007664	0.00498	0	0.002435	0.00361	0.004984	0.007444	0	0.000699	0.001064846
Clustering-based subsystems	NULL	USS-DB-7	Uncharacterized protein ImpH/VasB	0.000348	0	0	0.00996	0.000657	0.001948	0.000722	0.000498	0.00416	0	0.000233	0.002248009
Clustering-based subsystems	Monosaccharides	Unspecified monosaccharide transport cluster	Unspecified monosaccharide ABC transport system, permease component Ib (FIG143636)	0.004178	0	0.002555	0.00747	0.002626	0.002922	0.001444	0.000498	0.003065	0	0	0.002681835
Miscellaneous	Plant-Prokaryote DOE project	COG3760	UPF0434 protein YcaR	0.005571	0.002262	0.007664	0	0.008535	0.005357	0.007221	0.009469	0.007444	0	0	0.004535457
Amino Acids and Derivatives	Arginine; urea cycle, polyamines	Urea decomposition	Urea carboxylase (EC 6.3.4.6)	0.004526	0.002262	0.002555	0	0.002626	0.003896	0.002166	0.000997	0.002189	0	0	0.003391733
Amino Acids and Derivatives	Arginine; urea cycle, polyamines	Urease subunits	Urease accessory protein UreE	0.014623	0	0.007664	0	0.004596	0.001948	0.002888	0.007974	0.005255	0	0.021307	0.005166477
Carbohydrates	Monosaccharides	D-Galacturonate and D-Glucuronate Utilization	Uronate isomerase, family BH0493 (EC 5.3.1.12)	0.002437	0	0.002555	0.00498	0.003939	0.003896	0.004333	0.000997	0.000657	0	0	0.000433826
Nucleosides and Nucleotides	Purines	Purine Utilization	XdhC protein (assists in molybdopterin insertion into xanthine dehydrogenase)	0	0.01131	0	0.00996	0.001313	0	0.001444	0.003987	0.005255	0.01542	0.031321	0.002681835
Carbohydrates	Monosaccharides	Xylose utilization	Xylose ABC transporter, substrate-binding component	0.000348	0.002262	0.002555	0.00747	0	0.000487	0.000722	0	0.001533	0.004406	0	0.001222601
Cofactors, Vitamins, Prosthetic Groups, Pigments	Folate and pterines	YgfZ	YgfY COG2938	0.001044	0	0	0	0.001313	0.001461	0.001444	0.004984	0.007444	0.004406	0.011411	0.002445203
Regulation and Cell signaling	Programmed Cell Death and Toxin-antitoxin Systems	Toxin-antitoxin systems (other than RelBE and MazEF)	YoeB toxin protein	0.002785	0.006786	0	0.00498	0.005909	0.004383	0.010109	0.002492	0.00416	0	0	0.00252408
Miscellaneous	NULL	Broadly distributed proteins not in subsystems	YrbA protein	0.011141	0.01131	0	0.00249	0.00197	0.003896	0.005777	0.007475	0.003941	0	0	0.002602958
Virulence, Disease and Defense	Resistance to antibiotics and toxic compounds	Cobalt-zinc-cadmium resistance	Zinc transporter ZitB	0	0.006786	0	0.00249	0.002626	0.000974	0.001444	0.001495	0.001095	0	0.00687	0.001340918
Iron acquisition and metabolism	NULL	Heme, hemin uptake and utilization systems in GramPositives	Zn-dependent hydrolase YycJ/WalJ, required for cell wall metabolism and coordination of cell division with DNA replication	0	0.004524	0	0.00498	0.003939	0.003409	0.001444	0.001993	0.000876	0	0.000349	0.003194539
Cell Division and Cell Cycle	NULL	Bacterial Cytoskeleton	Z-ring-associated protein ZapA	0.006267	0.004524	0.00511	0	0.003939	0.005357	0.000722	0.008472	0.008539	0	0	0.004062192
Respiration	Electron donating reactions	Hydrogenases	[Fe] hydrogenase, HymA subunit, putative	0.00383	0.004524	0	0.00498	0.002626	0.00487	0.004333	0.000997	0	0	0	0.002997345
Metabolism of Aromatic Compounds	Peripheral pathways for catabolism of aromatic compounds	Biphenyl Degradation	2,3-dihydroxybiphenyl 1,2-dioxygenase	0.002089	0	0.002555	0	0.001313	0.001948	0.002888	0.001993	0.002846	0	0	0.00094653
RNA Metabolism	RNA processing and modification	rRNA modification Bacteria	23S rRNA (guanine-N-2-) -methyltransferase rlmG (EC 2.1.1.-)	0	0	0	0.00249	0.000657	0.001948	0.001444	0.000498	0.000438	0	0.014205	0.003628365
Amino Acids and Derivatives	Arginine; urea cycle, polyamines	Anaerobic Oxidative Degradation of L-Ornithine	2-amino-4-ketopentanoate thiolase, beta subunit	0	0.004524	0	0	0.000657	0.004383	0.002888	0.001495	0.000438	0	0.000815	0.002366325
Carbohydrates	Monosaccharides	2-Ketogluconate Utilization	2-ketogluconate 6-phosphate reductase (EC 1.1.1.43)	0	0.004524	0.002555	0	0.00197	0.000487	0	0	0.001095	0.002203	0.000349	0.000552143
Amino Acids and Derivatives	Lysine, threonine, methionine, and cysteine	Methionine Degradation	2-Oxobutyrate oxidase, putative	0.002785	0	0	0.00249	0.001313	0.002435	0.001444	0.004485	0.008758	0	0	0.000433826
Carbohydrates	CO2 fixation	Photorespiration (oxidative C2 cycle)	2-oxoglutarate/malate translocator	0	0	0.002555	0	0.004596	0.005844	0.00361	0.001495	0.001095	0	0.002794	0.00252408
Fatty Acids, Lipids, and Isoprenoids	Isoprenoids	Carotenoids	2-vinyl bacteriochlorophyllide hydratase BchF (EC 4.2.1.-)	0	0.002262	0	0.00747	0.00197	0.003896	0.005055	0.007974	0.004817	0	0	3.94388E-05
Metabolism of Aromatic Compounds	NULL	Phenylacetyl-CoA catabolic pathway (core)	3-hydroxyacyl-CoA dehydrogenase PaaC (EC 1.1.1.-)	0	0	0.010219	0	0.000657	0.006819	0.005055	0.010466	0.014013	0	0.037375	0.005087599
Fatty Acids, Lipids, and Isoprenoids	Isoprenoids	Carotenoids	4,4'-diapolycopene oxidase	0	0.002262	0.002555	0	0.002626	0.001461	0.00361	0.003489	0.001314	0	0	0.001064846
Metabolism of Aromatic Compounds	NULL	Cresol degradation	4-cresol dehydrogenase [hydroxylating] flavoprotein subunit (EC 1.17.99.1)	0.000348	0.002262	0	0	0.000657	0.000487	0.001444	0.000498	0.000438	0	0	0.001380356
Metabolism of Aromatic Compounds	Peripheral pathways for catabolism of aromatic compounds	Phenylpropanoid compound degradation	4-hydroxycinnamoyl CoA hydratase/lyase (Enoyl-CoA hydratase/lyase) (EC 4.2.1.17)	0	0	0.002555	0.00498	0.00197	0	0.000722	0.000997	0.001533	0.006608	0	0.000433826
Metabolism of Aromatic Compounds	Metabolism of central aromatic intermediates	Central meta-cleavage pathway of aromatic compound degradation	4-oxalocrotonate tautomerase (EC 5.3.2.-)	0.002785	0.004524	0	0	0.001313	0.002435	0.004333	0.001993	0.003503	0	0	0.002602958

Fatty Acids, Lipids, and Isoprenoids	Fatty acids	Fatty Acid Biosynthesis FASII	4'-phosphopantetheinyl transferase (EC 2.7.8.-)	0	0.002262	0	0	0.001313	0.001461	0.00361	0.001495	0.003722	0	0.000116	0.00094653
Clustering-based subsystems	NULL	CBSS-176279.3.peg.868	50S ribosomal protein acetyltransferase	0	0	0.002555	0.00249	0.000657	0	0.001444	0.003489	0.000876	0	0.000233	0.000236633
Amino Acids and Derivatives	Lysine, threonine, methionine, and cysteine	Lysine degradation	5-aminopentanamidase (EC 3.5.1.30)	0.003133	0.002262	0	0	0.003283	0.001948	0.001444	0.001993	0.012918	0	0	0.003943875
Amino Acids and Derivatives	Lysine, threonine, methionine, and cysteine	Lysine degradation	5-aminovalerate aminotransferase (EC 2.6.1.48)	0.002785	0	0.00511	0	0.002626	0.001461	0	0.000997	0.001971	0	0.000466	0.000354949
Iron acquisition and metabolism	NULL	ABC transporter [iron.B12.siderophore.hemin]	ABC transporter (iron.B12.siderophore.hemin) , ATP-binding component	0.002437	0	0	0	0.000657	0.001461	0.001444	0.000498	0.000876	0	0.000699	0.001459234
Miscellaneous	Plant-Prokaryote DOE project	COG0398	ABC transporter, ATP-binding protein YnjD	0	0.002262	0	0.00249	0.00197	0.001461	0.002888	0.004485	0.004598	0	0	0.000591581
Iron acquisition and metabolism	NULL	ABC-type iron transport system	ABC-type spermidine/putrescine transport systems, ATPase components	0.000348	0	0	0	0.003283	0.003409	0.002888	0.003987	0.008758	0	0.009315	0.002484642
Amino Acids and Derivatives	Lysine, threonine, methionine, and cysteine	Lysine biosynthesis AAA pathway 2	Acetylaminoadipate kinase (EC 2.7.2.-)	0	0.006786	0.00511	0.00249	0.003283	0.001948	0.002166	0	0.001752	0	0	0.001538111
Amino Acids and Derivatives	Arginine; urea cycle, polyamines	Arginine Biosynthesis extended	Acetylornithine aminotransferase 2 (EC 2.6.1.11)	0.002785	0.006786	0	0.00996	0	0.002435	0.000722	0.001495	0.000876	0	0	0.001064846
Clustering-based subsystems	NULL	CBSS-257314.1.peg.752	Adenine-specific methyltransferase (EC 2.1.1.72)	0.004874	0	0	0	0.032828	0.050165	0.090261	0.068774	0.008101	0	0.000466	0.006389078
Stress Response	Oxidative stress	Regulation of Oxidative Stress Response	Aerobic respiration control sensor protein arcB (EC 2.7.3.-)	0	0	0.00511	0.00249	0.002626	0.005357	0	0.000997	0.002408	0	0.022588	0.005008722
Clustering-based subsystems	Putative GGDEF domain protein related to agglutinin secretion	CBSS-323850.3.peg.3284	Agglutination protein	0.001393	0	0	0.00249	0	0.001948	0.002166	0.00299	0.002189	0	0.000349	0.008084945
Carbohydrates	Central carbohydrate metabolism	Methylglyoxal Metabolism	Aldehyde dehydrogenase A (EC 1.2.1.22)	0.002785	0	0	0	0.006566	0.004383	0.004333	0.005482	0.006568	0	0.031204	0.00220857
Cell Wall and Capsule	Capsular and extracellular polysacchrides	Alginate metabolism	Alginate lyase precursor (EC 4.2.2.3)	0	0	0	0.00249	0.00197	0.009254	0.009387	0.006977	0.001095	0	0.000349	0.004377702
Sulfur Metabolism	Organic sulfur assimilation	Alkanesulfonates Utilization	Alkanesulfonates transport system permease protein	0	0	0.00511	0	0.000657	0.000974	0	0.000997	0.002846	0.002203	0.000116	0.00126204
Nitrogen Metabolism	NULL	Amidase clustered with urea and nitrile hydratase functions	Amidase clustered with urea ABC transporter and nitrile hydratase functions	0.004526	0	0.002555	0	0.000657	0.002922	0	0.000498	0.005255	0.008811	0	0.000788775
Sulfur Metabolism	Organic sulfur assimilation	DMSP breakdown	Aminotransferase involved in DMSP breakdown	0.001741	0	0.002555	0	0.00197	0.001948	0.002166	0.004485	0.003065	0	0	0.000867653
Carbohydrates	Sugar alcohols	Glycerol and Glycerol-3-phosphate Uptake and Utilization	Anaerobic glycerol-3-phosphate dehydrogenase subunit B (EC 1.1.5.3)	0	0	0	0.00249	0.005252	0.009254	0.00361	0.000997	0.000219	0	0.000349	0.001498673
Respiration	Electron accepting reactions	Anaerobic respiratory reductases	Anaerobic sulfite reductase subunit B	0	0.004524	0	0.00249	0.000657	0.001461	0.000722	0.000498	0.000438	0	0	0.000552143
DNA Metabolism	DNA replication	DNA replication, archaeal	Archaeal DNA polymerase II small subunit (EC 2.7.7.7)	0	0.006786	0	0.00249	0.000657	0.000974	0.005777	0.001495	0.001752	0	0	0.002050815
Amino Acids and Derivatives	Lysine, threonine, methionine, and cysteine	Methionine Biosynthesis	Archaeal S-adenosylmethionine synthetase (EC 2.5.1.6)	0	0	0.00511	0	0.001313	0.001948	0	0.000997	0.001752	0.004406	0.000233	0.002721274
RNA Metabolism	RNA processing and modification	tRNA modification Archaea	Archease	0.002785	0	0	0.01494	0.004596	0.000487	0.002888	0.002492	0.001533	0	0	0.003352294
Miscellaneous	Plant-Prokaryote DOE project	At1g14345	ATPase of the AAA+ family protein associated with FIG137771 hypothetical protein	0.001044	0.006786	0	0	0.00197	0.002922	0.001444	0.001993	0.006349	0	0	0.0031551
Membrane Transport	Protein and nucleoprotein secretion system, Type IV	Vir-like type 4 secretion system	ATPase required for both assembly of type IV secretion complex and secretion of T-DNA complex, VirB11	0	0	0	0.00249	0.000657	0.001461	0	0.000997	0.000876	0.002203	0.000349	0.001695866
Regulation and Cell signaling	NULL	The Chv regulatory system of Alphaproteobacteria	ATP-binding protein ManX	0.002785	0	0	0	0.002626	0.001461	0.000722	0.006977	0.003722	0	0.000815	0.000552143
DNA Metabolism	DNA repair	DNA repair, bacterial DinG and relatives	ATP-dependent helicase DinG/Rad3	0	0	0.002555	0	0.002626	0.001461	0.005055	0.011961	0.009853	0	0.020143	0.009110352
Clustering-based subsystems	NULL	Aerotolerance operon in Bacteroides and potentially orthologous operons in other organisms	BatE	0	0	0.002555	0	0.000657	0.001461	0.002166	0.003489	0.000657	0.006608	0	0.006625711
Metabolism of Aromatic Compounds	NULL	Benzoate transport and degradation cluster	benzoate degradation ring-cleavage hydrolase	0.001044	0.002262	0	0	0.00197	0.005357	0.002888	0.004485	0.003065	0	0	0.001735305
Metabolism of Aromatic Compounds	NULL	Benzoate transport and degradation cluster	Benzoate transport, ATP binding protein	0	0	0.002555	0	0.000657	0.000974	0.000722	0	0.003503	0.008811	0.020609	0.00283959
Metabolism of Aromatic Compounds	NULL	Benzoate transport and degradation cluster	Benzoyl-CoA oxygenase component A	0	0.004524	0	0.00498	0.004596	0.002435	0.001444	0.00299	0.004379	0	0	0.001814183
Metabolism of Aromatic Compounds	Anaerobic degradation of aromatic compounds	Anaerobic benzoate metabolism	Benzoyl-CoA reductase subunit BadF (EC 1.3.99.15)	0	0	0.00511	0	0.002626	0.000974	0.004333	0.002492	0.003065	0.004406	0	0.001735305
Fatty Acids, Lipids, and Isoprenoids	Isoprenoids	Carotenoids	Beta-carotene hydroxylase	0	0	0	0	0.00197	0.003896	0.001444	0.004485	0.003284	0.004406	0.000233	0.005166477
Miscellaneous	Plant-Prokaryote DOE project	Niacin-Choline transport and metabolism	Betaine--homocysteine S-methyltransferase (EC 2.1.1.5)	0	0.006786	0	0	0.010505	0.008767	0.006499	0.009967	0.006131	0.008811	0	0.002721274
Clustering-based subsystems	tRNA sulfuration	CBSS-89187.3.peg.2957	Bifunctional PLP-dependent enzyme with beta-cystathionase and maltose regulon repressor activities	0.000696	0	0	0	0.001313	0.003409	0.001444	0.003489	0.004379	0	0.001281	0.001064846
Regulation and Cell signaling	Quorum sensing and biofilm formation	Biofilm Adhesin Biosynthesis	Biofilm PGA synthesis N-glycosyltransferase PgaC (EC 2.4.-.-)	0	0	0.00511	0.00249	0	0.002922	0.000722	0.000997	0.000657	0	0.000233	0.001498673
Cofactors, Vitamins, Prosthetic Groups, Pigments	Biotin	Biotin biosynthesis	Biotin synthesis protein bioH	0	0	0	0	0.000657	0.003896	0.00361	0.000997	0.003941	0.006608	0.000233	0.002366325
Sulfur Metabolism	NULL	Sulfur oxidation	Blr3520 protein homolog, hypothetical protein	0	0.004524	0	0.00498	0	0.001948	0.005055	0.00299	0.000657	0.002203	0	0.002287448
Virulence, Disease and Defense	Resistance to antibiotics and toxic compounds	Copper homeostasis	Blue copper oxidase CueO precursor	0.002437	0	0	0.00249	0.003283	0.002922	0.004333	0.001495	0.002846	0	0	0.00126204
Regulation and Cell signaling	NULL	cAMP signaling in bacteria	cAMP-dependent Kef-type K+ transport system	0	0	0.002555	0	0.00197	0.000974	0.001444	0.002492	0.002189	0.002203	0	0.00094653
Fatty Acids, Lipids, and Isoprenoids	Isoprenoids	Isoprenoid Biosynthesis	Candidate gene for the hypothesized phosphomevalonate decarboxylase	0	0.002262	0.00511	0.00498	0.006566	0.000487	0.002166	0.000997	0	0	0	0.00094653
Cell Wall and Capsule	Capsular and extracellular polysacchrides	Capsular Polysaccharide (CPS) of Campylobacter	Capsular polysaccharide export system inner membrane protein KpsE	0	0.002262	0	0.00249	0.000657	0.000487	0	0.00299	0.001752	0	0.012342	0.000788775
Cell Wall and Capsule	Capsular and extracellular polysacchrides	Capsular Polysaccharides Biosynthesis and Assembly	Capsular polysaccharide export system protein KpsS	0	0	0	0.00747	0.001313	0.002922	0.002166	0.003489	0.001314	0.004406	0	0.001340918
Cell Wall and Capsule	Capsular and extracellular polysacchrides	Serotype determining Capsular polysaccharide biosynthesis in Staphylococcus	Capsular polysaccharide synthesis enzyme Cap8D	0.000348	0.002262	0.00511	0.00249	0.000657	0	0.000722	0	0.000876	0	0	0.001143724
Cell Wall and Capsule	Capsular and extracellular polysacchrides	Vibrio Polysaccharide (VPS) Biosynthesis	Capsular polysaccharide synthesis enzyme CpsC, polysaccharide export	0	0.002262	0	0	0.00197	0.002435	0.000722	0.00299	0.000438	0	0.000699	0.00094653
Clustering-based subsystems	NULL	CBSS-314269.3.peg.1840	Carbon monoxide dehydrogenase form I, large chain(EC:1.2.99.2)	0.005571	0.002262	0	0.00249	0	0.001948	0.002166	0	0.004598	0	0.000349	0.00094653
Protein Metabolism	Protein degradation	Metallocarboxypeptidases (EC 3.4.17.-)	Carboxypeptidase T (EC 3.4.17.18)	0	0.002262	0	0.00498	0.001313	0.001461	0.00361	0.000498	0.000438	0	0	0.001025408
Cofactors, Vitamins, Prosthetic Groups, Pigments	Folate and pterines	p-Aminobenzoyl-Glutamate Utilization	Catalyzes the cleavage of p-aminobenzoyl-glutamate to p-aminobenzoate and glutamate, subunit A	0	0	0	0	0.000657	0.000974	0.001444	0.000997	0.000219	0.004406	0.000116	0.000828214
Miscellaneous	Plant-Prokaryote DOE project	At1g24340	Catechol 1,2-dioxygenase 1 (EC 1.13.11.1)	0.00383	0	0	0.00498	0	0.001461	0.002888	0.001495	0.000876	0	0.000233	0.000709898
Cell Division and Cell Cycle	NULL	Bacterial Cytoskeleton	Cell division protein FtsL	0.002089	0	0	0	0.000657	0.004383	0.000722	0.000498	0.005474	0	0.000466	0.000867653
Secondary Metabolism	Biosynthesis of phenylpropanoids	Flavanone biosynthesis	Chalcone synthase (EC 2.3.1.74)	0	0	0	0.00249	0.001313	0.001461	0.002166	0.004984	0.006568	0	0.001979	0.001143724
Cofactors, Vitamins, Prosthetic Groups, Pigments	Tetrapyrroles	Chlorophyll Biosynthesis	Chlorophyllide reductase subunit BchX (EC 1.18.-.-)	0	0.006786	0	0	0.004596	0.005357	0.004333	0.010964	0.011166	0	0.000466	0.00031551
Clustering-based subsystems	Choline bitartrate degradation, putative	CBSS-344610.3.peg.2335	Choline-glycine betaine transporter	0	0.002262	0	0	0.00197	0.000487	0.00361	0.001993	0.004379	0	0.014088	0.00126204
Clustering-based subsystems	NULL	Bile hydrolysis related cluster	Choloylglycine hydrolase (EC 3.5.1.24)	0	0	0.002555	0	0.000657	0.000487	0	0.000498	0.001095	0.004406	0.000116	0.001340918
Virulence, Disease and Defense	Adhesion	Adhesins in Staphylococcus	Clumping factor ClfB, fibrinogen binding protein	0	0.002262	0	0.00498	0.00197	0.000974	0.000722	0.003489	0.000876	0	0	0.000552143

Respiration	NULL	Carbon monoxide induced hydrogenase	CO dehydrogenase/acetyl-CoA synthase subunit beta, acetyl-CoA synthase (EC 2.3.1.169)	0.002785	0.002262	0	0.00747	0.005252	0.000974	0.001444	0	0	0.004406	0	0.003431172
Respiration	NULL	Carbon monoxide induced hydrogenase	CO dehydrogenase/acetyl-CoA synthase subunit delta, corrinoid iron-sulfur subcomplex small subunit	0.008704	0.004524	0.002555	0.00498	0.003939	0	0.000722	0.000498	0	0	0	0.001853621
Respiration	Electron donating reactions	CO Dehydrogenase	CO dehydrogenases maturation factor, CoxF family	0.000348	0	0.002555	0	0	0.001948	0.000722	0.001495	0.001971	0.002203	0	0.00031551
Carbohydrates	Sugar alcohols	Propanediol utilization	Cob(III)alamin reductase	0.000348	0.002262	0.00511	0.00996	0	0.000974	0	0.000997	0.001314	0	0	0.001301479
Cofactors, Vitamins, Prosthetic Groups, Pigments	Tetrapyrroles	Cobalamin synthesis	Cobalamin biosynthesis protein BluB	0	0	0	0	0.005909	0.003896	0.001444	0.000997	0.001971	0.002203	0.000233	0.002642397
Clustering-based subsystems	Oxidative stress	CoA-disulfide reductase (EC 1.8.1.14) containing cluster	Coenzyme A disulfide reductase	0	0.009048	0.007664	0.00996	0	0.005844	0.002888	0.002492	0.000876	0	0	0.004850967
Miscellaneous	Plant-Prokaryote DOE project	COG0523	COG0523: Putative GTPases (G3E family)	0	0.004524	0.002555	0	0.001313	0.001461	0	0.000997	0.002627	0	0.000466	0.001104285
Clustering-based subsystems	Catabolism of an unknown compound	CBSS-262316.1.peg.2929	COG2071: predicted glutamine amidotransferases in hypothetical Actinobacterial gene cluster	0	0.002262	0.010219	0	0.000657	0.000974	0.001444	0.000498	0.001971	0	0	0.000512704
Miscellaneous	Plant-Prokaryote DOE project	COG2363	COG2363	0.002437	0	0.007664	0	0.005909	0.004383	0.00361	0.011961	0.010728	0	0	0.011279484
Cell Wall and Capsule	Capsular and extracellular polysacchrides	Colanic acid biosynthesis	Colanic acid biosynthesis acetyltransferase WcaF (EC 2.3.1.-)	0	0	0	0	0.001313	0.000487	0.000722	0.001993	0.000657	0.002203	0.000349	0.001143724
Cell Wall and Capsule	Capsular and extracellular polysacchrides	Colanic acid biosynthesis	Colanic acid biosynthesis glycosyl transferase WcaE	0.000696	0.002262	0	0	0.000657	0.001461	0	0.003489	0.000657	0	0.000233	0.002681835
Cell Wall and Capsule	Capsular and extracellular polysacchrides	Colanic acid biosynthesis	Colanic acid biosynthsis UDP-glucose lipid carrier transferase WcaJ	0	0	0	0	0.001313	0.000487	0.002166	0.001993	0.003722	0.002203	0.001514	0.00189306
Stress Response	Cold shock	Cold shock, CspA family of proteins	Cold shock protein CspB	0.002785	0	0	0	0.002626	0	0.002888	0.002492	0.001533	0.004406	0.000116	0.001735305
Membrane Transport	Protein and nucleoprotein secretion system, Type IV	Conjugative transfer	Conjugative transfer protein TrbF	0	0	0	0.00249	0.001313	0.000974	0.000722	0.000498	0.000876	0	0.000116	0.000157755
Regulation and Cell signaling	NULL	Orphan regulatory proteins	Copper sensory histidine kinase CpxA	0.007311	0	0	0.00996	0.000657	0.001461	0	0.001993	0.002408	0	0.000233	0.001932499
Clustering-based subsystems	CRISPRs and associated hypotheticals	CBSS-216592.1.peg.3534	corresponds to STY3070 from Accession AL513382: Salmonella typhi CT18	0	0	0	0.00498	0.002626	0.003409	0.000722	0.001993	0.001752	0	0.000233	0.001301479
Membrane Transport	Protein and nucleoprotein secretion system, Type IV	pVir Plasmid of Campylobacter	Coupling protein VirD4, ATPase required for T-DNA transfer	0	0	0	0.00249	0.00197	0.001461	0.002166	0.001993	0.001971	0	0.000931	0.000907091
Amino Acids and Derivatives	Aromatic amino acids and derivatives	Phenylalanine and Tyrosine Branches from Chorismate	Cyclohexadienyl dehydratase (EC 4.2.1.51)(EC 4.2.1.91)	0	0.006786	0	0.00249	0.002626	0.007793	0.00361	0.000997	0.001533	0	0	0.005876374
Respiration	NULL	Soluble cytochromes and functionally related electron carriers	Cytochrome c2	0	0.002262	0	0	0.001313	0.002435	0.002166	0.007974	0.004379	0	0.000116	0.000749336
Virulence, Disease and Defense	NULL	C jejuni colonization of chick caeca	Cytochrome C551 peroxidase (EC 1.11.1.5)	0.002437	0	0.002555	0	0.003939	0.001461	0.002166	0.002492	0.001314	0	0	0.000670459
Respiration	Electron donating reactions	Hydrogenases	cytochrome-c3 hydrogenase gamma chain	0.000348	0	0	0.00498	0.002626	0.005844	0.00361	0.002492	0.001752	0	0	0.003115662
Virulence, Disease and Defense	Resistance to antibiotics and toxic compounds	Copper homeostasis: copper tolerance	Cytoplasmic copper homeostasis protein cutC	0.002437	0.002262	0	0	0	0.001948	0.002166	0.003987	0.000219	0	0.000815	0.002129693
Carbohydrates	Di- and oligosaccharides	Trehalose Uptake and Utilization	Cytoplasmic trehalase (EC 3.2.1.28)	0.000348	0	0	0	0.000657	0.000487	0	0.000997	0.000219	0.017622	0.000233	0.00063102
Amino Acids and Derivatives	Proline and 4-hydroxyproline	Proline, 4-hydroxyproline uptake and utilization	D-amino-acid oxidase (EC 1.4.3.3)	0.001044	0.006786	0	0.00249	0.002626	0	0.000722	0.001495	0.001314	0	0	0.000394388
Carbohydrates	Monosaccharides	L-fucose utilization	dehydrogenase clustered with L-fuconate utilization genes	0.002437	0	0	0	0	0.000487	0.000722	0.000997	0.001314	0.002203	0.000116	0.000276071
Membrane Transport	Uni- Sym- and Antiporters	Proton-dependent Peptide Transporters	Di/tripeptide permease DtpT	0.002437	0	0	0	0.000657	0.004383	0.002166	0.001993	0.003065	0.004406	0	0.004180508
Miscellaneous	Plant-Prokaryote DOE project	Experimental-PTPS	Dihydroneopterin triphosphate epimerase	0	0	0.002555	0	0.00197	0.002922	0.002888	0	0.003284	0.004406	0.016068	0.002879029
Cofactors, Vitamins, Prosthetic Groups, Pigments	Folate and pterines	Folate Biosynthesis	Dihydroneopterin triphosphate pyrophosphohydrolase type 2 (nudB)	0	0.002262	0	0	0.000657	0.000974	0.002166	0.000997	0.000876	0	0.000116	0.000867653
Phages, Prophages, Transposable elements, Plasmids	Phages, Prophages	Phage DNA synthesis	DNA adenine methyltransferase, phage-associated	0	0	0	0.00249	0.034141	0.015098	0.028161	0.021928	0.000219	0.004406	0	0.001498673
DNA Metabolism	DNA repair	DNA repair, bacterial UvrD and related helicases	DNA helicase IV	0	0.002262	0	0.00249	0.005252	0.002435	0	0.000498	0.000876	0	0.000815	0.002326887
RNA Metabolism	Transcription	RNA polymerase bacterial	DNA-directed RNA polymerase gamma subunit (EC 2.7.7.6)	0	0	0	0.00498	0.00197	0.001461	0.002166	0.001495	0.002408	0	0.001164	0.000907091
Amino Acids and Derivatives	Arginine; urea cycle, polyamines	Anaerobic Oxidative Degradation of L-Ornithine	D-Ornithine 4,5-aminomutase S subunit (EC 5.4.3.5)	0.002785	0	0.00511	0.00498	0	0.000487	0.000722	0.000997	0	0	0.000116	0.000788775
Clustering-based subsystems	NULL	Nucleoside Catabolism cluster	D-Ribose 1,5-phosphomutase (EC 5.4.2.7)	0	0.006786	0	0	0.000657	0.001461	0.000722	0.000498	0.003503	0	0.001979	0.00220857
Membrane Transport	NULL	ECF class transporters	Duplicated ATPase component CbrU of energizing module of predicted cobalamin ECF transporter	0.002437	0.006786	0	0.00747	0.000657	0.001948	0	0	0.001314	0.002203	0	0.000867653
Membrane Transport	NULL	ECF class transporters	Duplicated ATPase component MtsB of energizing module of methionine-regulated ECF transporter	0	0.01131	0.00511	0	0.002626	0.000974	0	0.000997	0.000438	0.002203	0	0.000788775
Stress Response	Osmotic stress	Ectoine biosynthesis and regulation	Ectoine hydroxylase (EC 1.17.-.-)	0	0	0	0.00249	0.005252	0.003896	0.011553	0.010466	0.013575	0	0.012808	0.003904437
Clustering-based subsystems	NULL	Cell wall related cluster	Eight transmembrane protein EpsH	0.003133	0	0.007664	0.01494	0.009848	0.004383	0	0.00299	0.001314	0	0	0.003549488
Carbohydrates	Monosaccharides	Mannose Metabolism	Endo-1,4-beta-mannosidase	0	0	0.002555	0.00249	0.003283	0.000974	0.005055	0.001495	0.001752	0	0	0.001695866
Respiration	Reverse electron transport	Archaeal membrane bound hydrogenases	Energy-conserving hydrogenase (ferredoxin), subunit B	0	0.002262	0.00511	0	0.000657	0.003896	0	0.001993	0.000657	0	0.001747	0.002445203
Clustering-based subsystems	Chromosome Replication	SeqA and Co-occurring Genes	Esterase ybfF (EC 3.1.-.-)	0.002089	0	0.002555	0.00249	0	0.000487	0.002166	0.001993	0.002189	0	0	0.001814183
Protein Metabolism	Protein biosynthesis	Translation initiation factors eukaryotic and archaeal	Eukaryotic translation initiation factor 2 beta subunit	0.001393	0.002262	0	0	0	0.001461	0.000722	0.000997	0.000219	0.002203	0	0.00031551
Sulfur Metabolism	Inorganic sulfur assimilation	Inorganic Sulfur Assimilation	Ferredoxin--sulfite reductase (EC 1.8.7.1)	0	0	0.002555	0	0.001313	0.000487	0.001444	0.000997	0.002408	0.004406	0	0.00094653
Iron acquisition and metabolism	Siderophores	Siderophore assembly kit	Ferric hydroxamate ABC transporter (TC 3.A.1.14.3), ATP-binding protein FhuC	0.000696	0	0	0	0.000657	0.000487	0.000722	0.000997	0.001533	0	0.000349	0.00031551
Iron acquisition and metabolism	NULL	Iron acquisition in Vibrio	Ferric hydroxamate outer membrane receptor FhuA	0	0.002262	0	0	0.000657	0.000487	0.000722	0.000498	0.000219	0	0.000233	0.000118316
Clustering-based subsystems	NULL	Cell wall related cluster	FIG004655: Polysaccharide deacetylase	0	0	0	0	0.001313	0.001461	0.002166	0.000997	0.001533	0.002203	0.000233	0.000907091
Clustering-based subsystems	DNA polymerase III epsilon cluster	CBSS-342610.3.peg.1536	FIG005121: SAM-dependent methyltransferase (EC 2.1.1.-)	0.004874	0	0.002555	0	0.000657	0.000974	0.002166	0.003489	0.006131	0	0	0.002366325
Clustering-based subsystems	NULL	Divergent RNA modification related clusters	FIG011178: rRNA methylase	0.002437	0	0	0.00249	0.000657	0.001461	0.00361	0.001495	0.001971	0	0	0.003115662
Clustering-based subsystems	Three hypotheticals linked to lipoprotein biosynthesis	CBSS-188.1.peg.9880	FIG018229: hypothetical protein	0.005571	0	0.002555	0	0.001313	0.000974	0.000722	0.005482	0.001314	0	0	0.000394388
Fatty Acids, Lipids, and Isoprenoids	Fatty acids	Phospholipid and Fatty acid biosynthesis related cluster	FIG021862: membrane protein, exporter	0.003133	0.006786	0.002555	0	0.001313	0	0	0	0.003503	0.002203	0.000233	0.001695866
Clustering-based subsystems	NULL	CBSS-316273.3.peg.448	FIG033680: Hypothetical protein	0.003482	0	0	0	0.000657	0	0.001444	0.001993	0.000657	0.002203	0.000815	0.000354949
Cell Wall and Capsule	Gram-Negative cell wall components	Lipopolysaccharide-related cluster in Alphaproteobacteria	FIG043197: Inositol monophosphatase family protein	0.001741	0.002262	0	0.00249	0	0.001948	0	0.003489	0.002627	0	0.000116	0.001143724
Clustering-based subsystems	alpha-proteobacterial cluster of hypotheticals	CBSS-52598.3.peg.2843	FIG139612: Possible conserved membrane protein	0	0	0	0	0.002626	0.00487	0.006499	0.004984	0.006787	0.002203	0.000466	0.000788775
Clustering-based subsystems	Putative GGDEF domain protein related to agglutinin secretion	CBSS-323850.3.peg.3284	FIGfam010717	0.000348	0.002262	0	0	0.000657	0.000487	0.000722	0	0.000438	0	0.000815	0.00094653
Fatty Acids, Lipids, and Isoprenoids	Fatty acids	Phospholipid and Fatty acid biosynthesis related cluster	FIGfam138462: Acyl-CoA synthetase, AMP-(fatty) acid ligase	0.000696	0	0	0.00249	0.00197	0.000974	0	0.000997	0.001533	0	0.000233	0.00157755

Carbohydrates	NULL	Carbon storage regulator	Flagellar assembly factor FliW	0	0	0.002555	0	0.000657	0.000974	0.002166	0.00299	0.000219	0	0.000116	0.002169132
Motility and Chemotaxis	Flagellar motility in Prokaryota	Flagellum	Flagellar basal-body rod protein FlgB	0.002785	0.009048	0	0	0.012475	0.005357	0.008665	0.009469	0.008101	0	0	0.008361016
Motility and Chemotaxis	Flagellar motility in Prokaryota	Flagellum	Flagellar biosynthesis protein FliL	0	0	0.002555	0	0.001313	0.005844	0.002888	0.005482	0.008101	0	0.00815	0.003312855
Motility and Chemotaxis	Flagellar motility in Prokaryota	Flagellum	Flagellar biosynthesis protein FliO	0	0	0.002555	0	0.000657	0.000974	0.001444	0.001495	0.001533	0	0.000116	0.00126204
Motility and Chemotaxis	Flagellar motility in Prokaryota	Flagellum	Flagellar biosynthesis protein FliS	0	0	0	0.00498	0.002626	0.007793	0.005777	0.004984	0.003284	0	0.000349	0.004574896
Cofactors, Vitamins, Prosthetic Groups, Pigments	Riboflavin, FMN, FAD	Flavodoxin	Flavodoxin 1	0.002785	0	0	0	0.001313	0.000974	0.000722	0.003489	0.004379	0	0.000815	0.003312855
Stress Response	NULL	Bacterial hemoglobins	Flavohemoprotein	0	0	0	0.00249	0.000657	0.000974	0	0.000997	0.000876	0.002203	0.040752	0.002484642
Membrane Transport	Protein secretion system, Type II	Widespread colonization island	Flp pilus assembly protein TadD, contains TPR repeat	0.001044	0	0	0	0.002626	0.002922	0.002166	0.007974	0.005693	0	0.002329	0.001656428
Respiration	Electron donating reactions	Succinate dehydrogenase	fumarate reductase/succinate dehydrogenase flavoprotein, N-terminal:FAD dependent oxidoreductase	0.005919	0	0	0.00249	0.003939	0.002922	0.002166	0.002492	0.001314	0	0	0.001774744
Carbohydrates	Monosaccharides	D-Tagatose and Galactitol Utilization	Galactitol-1-phosphate 5-dehydrogenase (EC 1.1.1.251)	0.000348	0.004524	0.00511	0	0	0.000974	0	0.001495	0.001314	0	0.000116	0.001419795
Carbohydrates	Di- and oligosaccharides	Lactose and Galactose Uptake and Utilization	Galactose/methyl galactoside ABC transport system, ATP-binding protein MglA (EC 3.6.3.17)	0	0.004524	0.002555	0	0.000657	0.000974	0.000722	0	0.001314	0	0.000699	0.002405764
Cofactors, Vitamins, Prosthetic Groups, Pigments	Quinone cofactors	Tocopherol Biosynthesis	Gamma-tocopherol C-methyltransferase (EC 2.1.1.95)	0	0	0	0.00249	0.00197	0.001948	0.002166	0.000997	0.002189	0	0.000466	0.001853621
Membrane Transport	Protein secretion system, Type II	General Secretion Pathway	General secretion pathway protein I	0.003133	0	0	0.00249	0.001313	0.000487	0	0.000498	0.001095	0	0.000582	0.000828214
Membrane Transport	Protein secretion system, Type II	General Secretion Pathway	General secretion pathway protein J	0.005571	0	0	0	0.001313	0.001948	0.002166	0.000498	0.008977	0	0.000582	0.001459234
Stress Response	Osmotic stress	Synthesis of osmoregulated periplasmic glucans	Glucans biosynthesis protein C (EC 2.1.-.-)	0.002089	0.002262	0	0	0.00197	0.003409	0.000722	0.004485	0.001533	0	0	0.002563519
Amino Acids and Derivatives	Glutamine, glutamate, aspartate, asparagine; ammonia assimilation	Glutamate and Aspartate uptake in Bacteria	Glutamate Aspartate transport ATP-binding protein GltL (TC 3.A.1.3.4)	0	0	0	0	0.000657	0.002922	0.001444	0.000498	0.003503	0.011014	0.000466	0.002918468
Clustering-based subsystems	tRNA sulfuration	CBSS-89187.3.peg.2957	Glutathione S-transferase domain protein	0.000696	0	0	0	0.001313	0.000974	0.000722	0.00299	0.000657	0	0.000349	0.001932499
Potassium metabolism	NULL	Potassium homeostasis	Glutathione-regulated potassium-efflux system ancillary protein KefG	0	0	0	0.00498	0.008535	0.015098	0.002166	0.007974	0.001752	0	0.000466	0.002681835
Nitrogen Metabolism	NULL	Nitrosative stress	Hcp transcriptional regulator HcpR (Crp/Fnr family)	0.006963	0	0.007664	0.00498	0.007879	0.00828	0.002166	0.002492	0	0	0	0.004417141
Cofactors, Vitamins, Prosthetic Groups, Pigments	Tetrapyrroles	Heme biosynthesis orphans	Heme biosynthesis protein related to NirD and NirG	0	0.002262	0	0	0.00197	0.005357	0.001444	0.003987	0.000438	0.002203	0	0.005994691
Nitrogen Metabolism	NULL	Dissimilatory nitrite reductase	Heme d1 biosynthesis protein NirD	0	0.002262	0.010219	0	0.002626	0.000974	0.002166	0.001495	0.001752	0	0	0.000552143
Cofactors, Vitamins, Prosthetic Groups, Pigments	Tetrapyrroles	Heme biosynthesis orphans	Heme d1 biosynthesis protein NirG	0.000348	0	0.002555	0	0.00197	0.002435	0.002888	0	0.002189	0	0.000349	0.001301479
Fatty Acids, Lipids, and Isoprenoids	Isoprenoids	Polyprenyl Diphosphate Biosynthesis	Heptaprenyl diphosphate synthase component II (EC 2.5.1.30)	0	0.002262	0	0.00249	0.001313	0.000974	0.001444	0.000498	0.001095	0	0	0.001498673
Amino Acids and Derivatives	Histidine Metabolism	Histidine Degradation	Histidine utilization repressor	0.002437	0.004524	0	0	0	0.001461	0.001444	0.003987	0.007225	0	0.015835	0.000867653
Miscellaneous	Plant-Prokaryote DOE project	COG3146 experimental	Histidinol-phosphate aminotransferase (EC 2.6.1.9) @ Aromatic-amino-acid aminotransferase (EC 2.6.1.57)	0	0.002262	0	0.00249	0.002626	0.001948	0.002888	0.003489	0.003065	0	0	0.000512704
Amino Acids and Derivatives	Lysine, threonine, methionine, and cysteine	Lysine biosynthesis AAA pathway 2	Homoisocitrate dehydrogenase (EC 1.1.1.87)	0	0	0	0.00249	0.000657	0	0.002888	0.000997	0.000876	0.002203	0.000116	0.000354949
Carbohydrates	Monosaccharides	Hexose Phosphate Uptake System	Homolog of fucose/glucose/galactose permeases	0	0	0.002555	0	0.003283	0.000487	0.001444	0.001495	0.004817	0	0.000233	0.001695866
Stress Response	Heat shock	Heat shock dnaK gene cluster extended	HspR, transcriptional repressor of DnaK operon	0.003482	0.004524	0.00511	0.00498	0.00197	0.001948	0	0	0.001314	0	0	0.002957907
Amino Acids and Derivatives	Alanine, serine, and glycine	Alanine biosynthesis	HTH-type transcriptional regulator IlvY	0.002089	0	0	0	0.00197	0	0.002166	0.001993	0.003503	0.008811	0.02189	0.004850967
Nucleosides and Nucleotides	NULL	Hydantoin metabolism	Hydantoin permease	0.000348	0	0.002555	0	0.000657	0	0.000722	0.000997	0.001752	0	0.03819	0.00063102
Respiration	NULL	Formate hydrogenase	Hydrogenase-4 component E (EC 1.-.-.-)	0.002437	0.002262	0.007664	0	0.002626	0	0	0	0.007225	0.006608	0.000116	0.002090254
Miscellaneous	Plant-Prokaryote DOE project	COG3533	Hypothetical periplasmic glycoside hydrolase, family DUF1680	0.004178	0	0.002555	0.00498	0.000657	0.001461	0	0.000997	0.004817	0	0	0.003352294
Carbohydrates	Central carbohydrate metabolism	TCA Cycle	hypothetical protein that often co-occurs with aconitase	0	0.004524	0	0.00498	0.002626	0.003896	0.005055	0.00299	0.003065	0	0	0.000670459
Carbohydrates	One-carbon Metabolism	Formaldehyde assimilation: Ribulose monophosphate pathway	Hypothetical protein with distant similarity to Ribonuclease E inhibitor RraA (former MenG)	0.000348	0.004524	0	0.00996	0.002626	0.003896	0.00361	0	0.000219	0	0	0.001695866
Membrane Transport	Protein and nucleoprotein secretion system, Type IV	Conjugative transfer	IncF plasmid conjugative transfer pilus assembly protein TraC	0	0.015834	0.00511	0.00249	0.002626	0.000487	0.001444	0	0.000876	0	0	0.001143724
Clustering-based subsystems	NULL	Conjugative transfer related cluster	IncW plasmid conjugative relaxase protein TrwC (TraI homolog)	0	0	0.00511	0	0.002626	0.000487	0	0.000997	0.002627	0.004406	0.000233	0.000276071
Phages, Prophages, Transposable elements, Plasmids	NULL	Integrons	Integron integrase IntI2	0	0	0	0.00249	0.001313	0.002435	0.002166	0.001993	0.001314	0	0.000699	0.001064846
Fatty Acids, Lipids, and Isoprenoids	NULL	Polyhydroxybutyrate metabolism	Intracellular PHB depolymerase (EC 3.1.1.-)	0	0	0.002555	0.00249	0.000657	0	0.000722	0.000997	0.001971	0.002203	0	0.001301479
Miscellaneous	Plant-Prokaryote DOE project	Iron-sulfur cluster assembly	Iron binding protein SufA for iron-sulfur cluster assembly	0.006267	0	0	0	0.001313	0.002435	0	0.003489	0.007663	0.002203	0.001048	0.00094653
Iron acquisition and metabolism	NULL	Iron(III) dicitrate transport system Fec	Iron(III) dicitrate transport ATP-binding protein FecE (TC 3.A.1.14.1)	0	0.002262	0.002555	0	0.00197	0.000487	0	0.001993	0.002408	0	0.002794	0.004456579
Carbohydrates	One-carbon Metabolism	Serine-glyoxylate cycle	isobutyryl-CoA dehydrogenase	0	0	0	0.00249	0.001313	0.003409	0.004333	0.001495	0.003503	0	0.000466	0.000788775
Fatty Acids, Lipids, and Isoprenoids	Isoprenoids	Archaeal lipids	Isopentenyl phosphate kinase	0.001044	0.002262	0	0	0.000657	0.000487	0.002888	0.000997	0.000876	0	0	0.000118316
Amino Acids and Derivatives	Lysine, threonine, methionine, and cysteine	Threonine degradation	L-allo-threonine aldolase	0	0.002262	0	0.00498	0.001313	0.002435	0.001444	0.003987	0.006131	0	0	0.002129693
Carbohydrates	Monosaccharides	L-Arabinose utilization	L-arabinose transport system permease protein (TC 3.A.1.2.2)	0	0.006786	0.00511	0	0.000657	0.001948	0	0.002492	0.001971	0	0.000815	0.002721274
Cofactors, Vitamins, Prosthetic Groups, Pigments	NAD and NADP	NAD and NADP cofactor biosynthesis global	L-Aspartate dehydrogenase (EC 1.4.1.21)	0	0.002262	0	0.01245	0.003283	0.001461	0.002888	0.001495	0.001314	0	0	0.000433826
Stress Response	Oxidative stress	Glutathione analogs: mycothiol	L-cysteine:1D-myo-inositol 2-amino-2-deoxy-alpha-D-glucopyranoside ligase MshC	0.003482	0	0.00511	0	0.000657	0.000487	0	0.000498	0.001095	0	0.000116	0.000552143
Sulfur Metabolism	Organic sulfur assimilation	L-Cystine Uptake and Metabolism	L-cystine uptake protein TcyP	0	0	0.002555	0	0.001313	0.001461	0.00361	0.000997	0.001314	0	0.000116	0.001538111
Cell Wall and Capsule	Capsular and extracellular polysacchrides	Legionaminic Acid Biosynthesis	Legionaminic acid cytidylyltransferase (EC 2.7.7.43)	0	0.002262	0.002555	0	0.007222	0.002435	0.005055	0.002492	0.001533	0	0	0.002642397
Carbohydrates	Monosaccharides	L-fucose utilization temp	L-fuconolactone hydrolase	0.000696	0	0	0	0.003939	0.000974	0.00361	0.001993	0.003941	0	0.000233	0.002484642
Cofactors, Vitamins, Prosthetic Groups, Pigments	Tetrapyrroles	Chlorophyll Biosynthesis	Light-independent protochlorophyllide reductase subunit N (EC 1.18.-.-)	0	0.004524	0	0	0.003283	0.003896	0.006499	0.009469	0.01029	0	0.003959	0.000473265
Carbohydrates	Monosaccharides	D-gluconate and ketogluconates metabolism	Low-affinity gluconate/H+ symporter GntU	0.000696	0.004524	0.002555	0	0	0.000487	0.002166	0.005482	0.003503	0	0	0.001301479
Protein Metabolism	Protein biosynthesis	Ribosome LSU eukaryotic and archaeal	LSU ribosomal protein L10Ae (L1p)	0	0.002262	0	0	0.002626	0.000487	0.000722	0.000498	0.000219	0	0.000116	0.000394388
Protein Metabolism	Protein biosynthesis	Ribosome LSU eukaryotic and archaeal	LSU ribosomal protein L12a (P1/P2)	0.001044	0	0.002555	0.00498	0.004596	0.002922	0.002166	0.000997	0	0	0	0.001616989
Protein Metabolism	Protein biosynthesis	Ribosome LSU eukaryotic and archaeal	LSU ribosomal protein L12e (L11p)	0	0	0.00511	0	0.002626	0.002435	0.001444	0.000997	0.001095	0.004406	0	0.001616989
Protein Metabolism	Protein biosynthesis	Ribosome LSU eukaryotic and archaeal	LSU ribosomal protein L19e	0.001393	0	0	0.00249	0.001313	0.001461	0	0.001993	0.001971	0	0.000233	0.001064846

Protein Metabolism	Protein biosynthesis	Ribosome LSU eukaryotic and archaeal	LSU ribosomal protein L23e (L14p)	0	0.004524	0	0.00249	0.001313	0.000974	0.007943	0.000498	0	0	0.000116	0.00126204
Protein Metabolism	Protein biosynthesis	Ribosome LSU eukaryotic and archaeal	LSU ribosomal protein L7e (L30p)	0	0	0.002555	0	0.001313	0.001461	0.000722	0.001993	0.000438	0.002203	0	0.001301479
Fatty Acids, Lipids, and Isoprenoids	Isoprenoids	Carotenoids	Lycopene beta cyclase (EC 1.14.-.-)	0.000696	0	0	0	0.000657	0.002435	0.000722	0.001495	0.001314	0	0.000349	0.000749336
Miscellaneous	Plant-Prokaryote DOE project	Synechocystis experimental	Lysophospholipase L2 (EC 3.1.1.5)	0	0.004524	0	0	0.002626	0.001461	0.002166	0.003489	0.004598	0	0.000349	0.000828214
Regulation and Cell signaling	NULL	DNA-binding regulatory proteins, strays	LysR family transcriptional regulator PA3398	0	0	0.002555	0	0.000657	0.003896	0.005777	0.00598	0.005255	0	0.027944	0.005481987
Protein Metabolism	Protein degradation	Serine endopeptidase (EC 3.4.21.-)	Lysyl endopeptidase (EC 3.4.21.50)	0.000348	0.004524	0	0	0.001313	0.004383	0.002888	0.001495	0.000876	0	0	0.001380356
Cell Wall and Capsule	Gram-Negative cell wall components	Major Outer Membrane Proteins	Maltoporin (maltose/maltodextrin high-affinity receptor, phage lambda receptor protein)	0	0	0	0.00498	0.00197	0.000974	0.002166	0.000498	0.003503	0	0.000116	0.001419795
Membrane Transport	NULL	Transport of Manganese	Manganese uptake regulation protein MUR	0	0	0	0.00498	0.00197	0.001948	0.00361	0.002492	0.002189	0	0.000233	0.000433826
Carbohydrates	One-carbon Metabolism	Methanogenesis from methylated compounds	Methanol:corrinoid methyltransferase	0	0.004524	0.002555	0	0.00197	0.002435	0.002888	0.001495	0	0	0.000116	0.001340918
Respiration	Electron donating reactions	Respiratory dehydrogenases 1	Methylamine dehydrogenase heavy chain precursor (EC 1.4.99.3)	0.002437	0	0.002555	0	0.000657	0.000974	0.001444	0.001495	0.001314	0	0	0.000394388
Carbohydrates	One-carbon Metabolism	Methanogenesis from methylated compounds	Methylcobalamin:coenzyme M methyltransferase, methanol-specific	0.002785	0	0	0.00747	0.004596	0.003896	0.000722	0.000997	0	0.002203	0	0.001143724
Carbohydrates	One-carbon Metabolism	One-carbon metabolism by tetrahydropterines	Methylene tetrahydromethanopterin dehydrogenase (EC 1.5.99.9)	0.00383	0.009048	0	0	0.000657	0.000487	0.000722	0.000498	0.002189	0	0	0.000907091
Carbohydrates	One-carbon Metabolism	Serine-glyoxylate cycle	Methylmalonyl-CoA mutase, small subunit (EC 5.4.99.2)	0.006267	0	0	0	0	0.000974	0.001444	0.004485	0.003722	0.002203	0.000233	0.005205916
Respiration	Sodium Ion-Coupled Energetics	Na+ translocating decarboxylases and related biotin-dependent enzymes	Methylmalonyl-CoA:Pyruvate transcarboxylase 5S subunit (EC 2.1.3.1)	0	0.002262	0.002555	0.00249	0	0.001948	0.000722	0.001495	0	0	0.000466	0.000276071
Cofactors, Vitamins, Prosthetic Groups, Pigments	Folate and pterines	Molybdenum cofactor biosynthesis	Molybdenum cofactor biosynthesis protein MoaD	0.001044	0	0	0	0.00197	0.000487	0.002888	0.001495	0.001752	0	0.000233	0.002484642
Clustering-based subsystems	Sulfatases and sulfatase modifying factor 1 (and a hypothetical)	Sulfatases and sulfatase modifying factor 1	Mucin-desulfating sulfatase MdsA precursor (EC 3.1.6.14)	0.002089	0.002262	0.007664	0	0	0.001461	0.00361	0.001993	0.001752	0	0	0.00252408
Carbohydrates	Di- and oligosaccharides	Fructooligosaccharides(FOS) and Raffinose Utilization	Multiple sugar ABC transporter, membrane-spanning permease protein MsmG	0	0.004524	0	0.00498	0.000657	0.001461	0.000722	0.001495	0.001533	0	0	0.001340918
Carbohydrates	One-carbon Metabolism	Serine-glyoxylate cycle	N(5),N(10)-methenyltetrahydromethanopterin cyclohydrolase (EC 3.5.4.27)	0.002785	0	0	0	0.002626	0.000487	0.001444	0.001993	0.005255	0	0.000233	0.00094653
Carbohydrates	Aminosugars	Chitin and N-acetylglucosamine utilization	N-Acetyl-D-glucosamine ABC transport system ATP-binding protein	0.040038	0	0.002555	0.00747	0.000657	0.000974	0	0	0.001752	0	0.000233	0.000591581
Amino Acids and Derivatives	Lysine, threonine, methionine, and cysteine	Lysine Biosynthesis DAP Pathway	N-acetyl-L,L-diaminopimelate aminotransferase (EC 2.6.1.-)	0	0	0.00511	0.00249	0.001313	0.000974	0.000722	0.001495	0.001314	0	0	0.001025408
Regulation and Cell signaling	Quorum sensing and biofilm formation	Quorum sensing in Yersinia	N-acyl homoserine lactone hydrolase	0.006963	0	0	0.00249	0.000657	0.001461	0.001444	0.000498	0.001314	0	0	0.002248009
Respiration	Electron donating reactions	Respiratory Complex I	NAD(P)H-quinone oxidoreductase chain 5 (EC 1.6.5.2)	0	0	0.002555	0.00249	0.00197	0.000487	0	0.000498	0.001095	0.006608	0	0.000433826
Respiration	Electron donating reactions	Respiratory Complex I	NAD(P)H-quinone oxidoreductase chain J (EC 1.6.5.2)	0.001741	0	0.012774	0.00747	0	0.002922	0.004333	0.005482	0.003503	0	0	0.002129693
Respiration	Electron donating reactions	Respiratory Complex I	NADH dehydrogenase subunit 4L	0.001741	0	0	0.00747	0.003283	0	0.002166	0	0.001971	0.006608	0.000116	0.000867653
Respiration	Electron donating reactions	Respiratory Complex I	NADH-quinone oxidoreductase chain F 2 (EC 1.6.99.5)	0	0.009048	0.00511	0.00249	0.004596	0.002435	0.000722	0.000498	0	0	0	0.002011377
Stress Response	Oxidative stress	Glutathione analogs: mycothiol	NADPH-dependent mycothiol reductase Mtr	0.005919	0.006786	0.007664	0.00498	0.000657	0	0.000722	0	0.000876	0	0	0.00063102
Cofactors, Vitamins, Prosthetic Groups, Pigments	NAD and NADP	NAD and NADP cofactor biosynthesis global	Nicotinamide phosphoribosyltransferase (EC 2.4.2.12)	0	0	0	0	0.014444	0.015098	0.037548	0.034387	0.001314	0.002203	0.001397	0.000867653
DNA Metabolism	NULL	DNA phosphorothioation	NifS-like cysteine desulfurase DndA	0	0.004524	0.002555	0	0.000657	0	0.001444	0.002492	0.000657	0	0.000233	0.000867653
Nitrogen Metabolism	NULL	Nitrate and nitrite ammonification	Nitrate reductase cytochrome c550-type subunit	0.000696	0	0	0.00249	0.000657	0.001948	0.001444	0	0.001533	0	0.000349	0.003667804
Respiration	Electron accepting reactions	trimethylamine N-oxide (TMAO) reductase	Nitrate/TMAO reductases, membrane-bound tetraheme cytochrome c subunit	0	0.002262	0.010219	0	0.00197	0.00487	0	0.000997	0.001752	0	0.000116	0.004417141
Nitrogen Metabolism	NULL	Denitrification	Nitric oxide -responding transcriptional regulator NnrR (Crp/Fnr family)	0	0	0	0.00249	0.00197	0.001461	0.001444	0.002492	0.001752	0	0.000349	0.00063102
Nitrogen Metabolism	NULL	Nitrogen fixation	Nitrogenase FeMo-cofactor synthesis molybdenum delivery protein NifQ	0	0	0.002555	0	0.001313	0.000487	0.001444	0.001495	0.001752	0.004406	0	0.001380356
Nitrogen Metabolism	NULL	Nitrate and nitrite ammonification	NrfC protein	0.000348	0.004524	0.002555	0.00996	0	0.000974	0.000722	0.000498	0	0	0	0.002760713
Amino Acids and Derivatives	Lysine, threonine, methionine, and cysteine	Lysine Biosynthesis DAP Pathway	N-succinyl-L,L-diaminopimelate aminotransferase (EC 2.6.1.17)	0	0	0	0	0.000657	0.001948	0.002888	0.001495	0.00416	0.002203	0.007452	0.003549488
Clustering-based subsystems	Probably organic hydroperoxide resistance related hypothetical protein	CBSS-269482.1.peg.1294	Organic hydroperoxide resistance transcriptional regulator	0	0	0	0	0.000657	0.001948	0.001444	0.00299	0.004379	0.004406	0.001979	0.0031551
Amino Acids and Derivatives	Arginine; urea cycle, polyamines	Polyamine Metabolism	Ornithine decarboxylase (EC 4.1.1.17)	0.000348	0.002262	0.002555	0	0	0	0.000722	0.001993	0.000876	0	0.000466	0.001064846
Cell Wall and Capsule	Gram-Negative cell wall components	Major Outer Membrane Proteins	Osmolarity sensory histidine kinase EnvZ	0.000348	0	0.00511	0.00996	0	0.000974	0	0.000997	0.015983	0	0.013856	0.000709898
Carbohydrates	Monosaccharides	D-Galacturonate and D-Glucuronate Utilization	Pectin degradation protein KdgF	0.000696	0	0	0	0.000657	0.003896	0.002166	0.001495	0.000657	0	0.000349	0.001735305
Phages, Prophages, Transposable elements, Plasmids	Phages, Prophages	Phage capsid proteins	Phage capsid and scaffold	0	0	0	0.00498	0.012475	0.048704	0.049102	0.047843	0.000876	0	0.000116	0.00094653
Phages, Prophages, Transposable elements, Plasmids	Phages, Prophages	Prophage lysogenic conversion modules	Phage lysin	0	0	0	0	0.002626	0.00487	0.002166	0.00598	0.001095	0.002203	0.000466	0.001538111
Phages, Prophages, Transposable elements, Plasmids	Phages, Prophages	Phage regulation of gene expression	Phage repressor	0.002437	0	0	0	0.001313	0.001948	0.001444	0.00299	0.001533	0	0.000116	0.000788775
Clustering-based subsystems	NULL	CBSS-159087.4.peg.2189	Phage T7 exclusion protein	0	0	0	0	0.001313	0.001461	0.002888	0.001993	0.003722	0.004406	0.000699	0.003036784
Phages, Prophages, Transposable elements, Plasmids	Phages, Prophages	Phage packaging machinery	Phage terminase large subunit	0	0.004524	0	0	0.041363	0.048704	0.070042	0.074754	0.000657	0	0.000466	0.001340918
Cell Wall and Capsule	Cell wall of Mycobacteria	mycolic acid synthesis	PHENOLPTHIOCEROL SYNTHESIS TYPE-I POLYKETIDE SYNTHASE PPSC	0.001741	0.004524	0	0.00249	0.000657	0.000487	0.000722	0	0.003503	0	0	0.001971938
Metabolism of Aromatic Compounds	NULL	Phenylacetyl-CoA catabolic pathway (core)	Phenylacetic acid degradation operon negative regulatory protein PaaX	0.001044	0	0	0	0.00197	0.001461	0.002166	0.000498	0.000438	0	0.028876	0.003943875
Phosphorus Metabolism	NULL	Alkylphosphonate utilization	PhnJ protein	0	0.004524	0	0	0.007222	0.007793	0.009387	0.020433	0.006568	0	0.042265	0.003273417
Phosphorus Metabolism	NULL	Phosphate metabolism	Phosphatase, Ppx/GppA family	0.000696	0	0	0.00249	0.004596	0.003409	0.000722	0.003987	0.000657	0	0	0.000749336
Phosphorus Metabolism	NULL	Phosphonate metabolism	Phosphonoacetaldehyde hydrolase (EC 3.11.1.1)	0	0	0	0.00249	0.003939	0.004383	0.002166	0	0.000876	0.024231	0.006171	0.001104285
Photosynthesis	Electron transport and photophosphorylation	Photosystem II-type photosynthetic reaction center	Photosynthetic reaction center H subunit	0	0	0.002555	0	0.003283	0.003409	0.004333	0.00299	0.007444	0	0.000349	0.000473265
Photosynthesis	Electron transport and photophosphorylation	Photosystem I	photosystem I P700 chlorophyll a apoprotein subunit Ib (PsaB)	0	0	0.00511	0.00249	0.00197	0.000487	0.000722	0	0.011166	0	0.000699	0.001025408
Photosynthesis	Electron transport and photophosphorylation	Photosystem II	Photosystem II CP43 protein (PsbC)	0	0.002262	0	0.00498	0.001313	0	0.002888	0.000997	0.000876	0	0.002212	0.000197194
Stress Response	Oxidative stress	Glutathione: Non-redox reactions	Phytochelatin synthase (EC 2.3.2.15), bacterial type	0.002089	0	0	0.00249	0.000657	0.000974	0	0.001993	0.000219	0	0.000349	0.000118316
Membrane Transport	Protein and nucleoprotein secretion system, Type IV	pVir Plasmid of Campylobacter	Plasmid partitioning protein ParA	0.000348	0	0	0	0.00197	0.003409	0.002888	0.000498	0.002408	0.004406	0	0.00063102
DNA Metabolism	DNA replication	Plasmid replication	Plasmid replication protein RepB	0.005571	0.004524	0	0	0.000657	0.002922	0	0.001495	0.001971	0	0.000815	0.000867653
Fatty Acids, Lipids, and Isoprenoids	NULL	Polyhydroxybutyrate metabolism	Poly(3-hydroxyalkanoate) depolymerase	0	0	0.002555	0.00249	0.001313	0.000974	0.002166	0.000997	0.001971	0	0	0.000749336
Cell Wall and Capsule	Gram-Negative cell wall components	Lipid A-Ara4N pathway (Polymyxin resistance)	Polymyxin resistance protein PmrJ, predicted deacetylase	0.001044	0.01131	0.010219	0.00996	0	0	0	0	0.003284	0.002203	0.000233	0.002405764

Cell Wall and Capsule	Capsular and extracellular polysacchrides	Vibrio Polysaccharide (VPS) Biosynthesis	polysaccharide biosynthesis protein, putative	0.001741	0	0	0.00249	0.002626	0.001461	0	0.000498	0.001533	0	0.000466	0.004850967
Stress Response	Oxidative stress	CoA disulfide thiol-disulfide redox system	Polysulfide binding and transferase domain	0.004178	0.004524	0	0.00498	0.000657	0	0.002166	0.000997	0.000438	0	0	0.001735305
Clustering-based subsystems	NULL	Disulphide related cluster	Polysulfide binding protein	0	0	0.007664	0	0.001313	0.002435	0.000722	0.007974	0.009415	0	0.001164	0.001222601
Respiration	Electron accepting reactions	Anaerobic respiratory reductases	Polysulfide reductase, subunit A, putative	0	0.002262	0.00511	0	0.001313	0.001948	0.004333	0.000997	0.000438	0	0	0.001616989
Phages, Prophages, Transposable elements, Plasmids	Phages, Prophages	Phage packaging machinery	Portal protein	0	0.002262	0	0	0.006566	0.012176	0.037548	0.030898	0.002408	0	0.000582	0.001656428
Nucleosides and Nucleotides	Pyrimidines	Pyrimidine utilization	Possible pyrimidine permease in reductive pathway	0	0	0.010219	0.00249	0	0.000487	0	0.000498	0.003503	0.01542	0.000582	0.001222601
Nucleosides and Nucleotides	Pyrimidines	Novel non-oxidative pathway of Uracil catabolism	Possible pyrimidine-degrading protein DUF1688	0.002785	0	0.002555	0	0.00197	0.000487	0.001444	0.001993	0.002189	0	0	0.000118316
Potassium metabolism	NULL	Potassium homeostasis	Potassium uptake protein, integral membrane component, KtrA	0	0	0.007664	0	0	0.002435	0.000722	0.001993	0.007006	0.006608	0.002562	0.00855821
Carbohydrates	Monosaccharides	Xylose utilization	predicted 2-keto-3-deoxyxylonate dehydratase	0.002785	0	0	0	0.001313	0.000974	0.001444	0.001495	0.001752	0	0.000815	0.001064846
Virulence, Disease and Defense	Adhesion	Adhesins in Staphylococcus	Predicted cell-wall-anchored protein SasA (LPXTG motif)	0	0.002262	0.010219	0	0.007879	0.001948	0.01733	0.004984	0.002846	0	0	0.001735305
Carbohydrates	Aminosugars	N-Acetyl-Galactosamine and Galactosamine Utilization	Predicted galactosamine-6-phosphate isomerase (EC 5.3.1.-)	0	0	0	0.00249	0	0.000487	0.000722	0.000997	0.000219	0.002203	0.002212	0.000512704
Carbohydrates	Monosaccharides	Hexose Phosphate Uptake System	Predicted glucose transporter in maltodextrin utilization gene cluster	0.002785	0.004524	0	0	0.001313	0.000974	0	0.000997	0.001533	0	0.002562	0.001695866
Carbohydrates	Di- and oligosaccharides	Maltose and Maltodextrin Utilization	Predicted maltose-specific TonB-dependent receptor	0.005222	0	0	0	0	0.000974	0.001444	0.001993	0.001533	0.002203	0.045293	0.001025408
Membrane Transport	NULL	Transport of Manganese	Predicted manganese transporter, 11 TMS	0.001393	0	0	0.01245	0.000657	0.002922	0.002166	0.004485	0.001314	0	0	0.001656428
Carbohydrates	Monosaccharides	D-ribose utilization	Predicted nucleoside ABC transporter, permease 2 component	0.003133	0	0	0.00249	0.003939	0.002435	0.005777	0.006479	0.005255	0	0	0.001616989
Membrane Transport	Protein secretion system, Type II	Predicted secretion system X	Predicted secretion system X protein GspD-like	0	0	0.00511	0.00249	0.000657	0.000487	0	0.000997	0.001752	0	0.000233	0.000394388
Motility and Chemotaxis	Flagellar motility in Prokaryota	Flagellar motility	Predicted signal transduction protein	0.000348	0	0	0	0.009192	0.00487	0.007221	0.005482	0.017735	0	0.019328	0.022756162
Respiration	NULL	Soluble cytochromes and functionally related electron carriers	Probable cytochrome c2	0.001044	0.004524	0	0	0.00197	0.002435	0.002888	0.000498	0.000438	0	0	0.001301479
Amino Acids and Derivatives	Arginine; urea cycle, polyamines	Polyamine Metabolism	Probable two-component sensor, near polyamine transporter	0.002089	0	0	0.00498	0.000657	0	0.00361	0.000498	0.001752	0.002203	0	0.00252408
Carbohydrates	Sugar alcohols	Propanediol utilization	Propanediol utilization polyhedral body protein PduT	0.000348	0	0.002555	0.00249	0.000657	0	0.001444	0.000997	0	0	0.000116	0.001340918
Regulation and Cell signaling	NULL	cAMP signaling in bacteria	Prophage Clp protease-like protein	0	0	0	0.00747	0.012475	0.012663	0.020218	0.030898	0.004379	0	0.000699	0.001183163
Protein Metabolism	Protein degradation	Proteasome archaeal	Proteasome subunit beta (EC 3.4.25.1), archaeal	0	0.002262	0	0.00249	0.00197	0.000974	0.005777	0.001495	0.003065	0	0	0.001695866
RNA Metabolism	RNA processing and modification	Polyadenylation specificity factor	Protein similar to polyadenylation specificity factor, MJ0047 type	0.001044	0.002262	0	0.00249	0.001313	0	0.004333	0.003987	0.001533	0	0	0.000749336
Nucleosides and Nucleotides	NULL	Pseudouridine catabolism	Pseudouridine kinase (EC 2.7.1.83)	0.000348	0.002262	0.002555	0	0.001313	0.000487	0	0.000498	0.000438	0	0	0.001301479
Carbohydrates	Di- and oligosaccharides	Trehalose Uptake and Utilization	PTS system, glucose-specific IIB component (EC 2.7.1.69)	0	0	0	0	0.000657	0.000487	0.000722	0.000498	0.000219	0.002203	0.000116	0.00063102
Carbohydrates	Di- and oligosaccharides	Trehalose Uptake and Utilization	PTS system, glucose-specific IIC component (EC 2.7.1.69)	0	0.004524	0	0	0.000657	0.000487	0.000722	0.001495	0.001314	0	0.000699	0.00063102
Carbohydrates	Monosaccharides	Mannose Metabolism	PTS system, mannose-specific IIA component (EC 2.7.1.69)	0.000348	0	0.002555	0.00498	0.000657	0.002922	0	0.000498	0.000438	0	0	0.001814183
Amino Acids and Derivatives	Aromatic amino acids and derivatives	Aromatic amino acid degradation	Putative 2,4-dihydroxyhept-2-ene-1,7-dioic acid aldolase	0.001044	0	0.010219	0	0.00197	0.000974	0.001444	0.003987	0.000876	0	0	0.001064846
Protein Metabolism	Protein degradation	Proteolysis in bacteria, ATP-dependent	Putative ATP:guanido phosphotransferase YacI (EC 2.7.3.-)	0	0.009048	0.002555	0	0.001313	0.000974	0.002888	0.001993	0.001314	0	0	0.001774744
Carbohydrates	Central carbohydrate metabolism	Ethylmalonyl-CoA pathway of C2 assimilation	Putative cyclic di-GMP phosphodiesterase, EAL domain protein	0	0	0	0.00498	0	0.001461	0.000722	0.001993	0.003503	0.002203	0.001048	0.000473265
Carbohydrates	Central carbohydrate metabolism	Dihydroxyacetone kinases	Putative dihydroxyacetone kinase (EC 2.7.1.29), ADP-binding subunit	0	0.002262	0	0.00249	0.001313	0.001461	0.001444	0.000498	0.000657	0	0	0.000670459
Miscellaneous	Plant-Prokaryote DOE project	Niacin-Choline transport and metabolism	Putative Dimethylglycine oxidase	0	0.004524	0	0.00747	0.003939	0.002435	0.001444	0.000997	0.005255	0	0	0.000670459
Metabolism of Aromatic Compounds	Metabolism of central aromatic intermediates	Salicylate and gentisate catabolism	putative facilitator of salicylate uptake	0.002089	0.004524	0	0.00747	0.002626	0.001461	0	0.000997	0.000438	0	0	0.002484642
Iron acquisition and metabolism	NULL	Heme, hemin uptake and utilization systems in GramNegatives	Putative heme iron utilization protein	0	0	0	0.00249	0.00197	0.000974	0	0.002492	0.003722	0.002203	0.00163	0.001340918
Fatty Acids, Lipids, and Isoprenoids	Fatty acids	Phospholipid and Fatty acid biosynthesis related cluster	Putative histidine ammonia-lyase protein	0	0	0.015329	0	0.001313	0	0.002166	0.00299	0.007225	0.004406	0.000582	0.000709898
Motility and Chemotaxis	Flagellar motility in Prokaryota	Flagellar motility	Putative lipoprotein required for motility	0.000348	0	0.002555	0	0.00197	0.002922	0.002888	0.000997	0.000876	0	0	0.001183163
Miscellaneous	NULL	ZZ gjo need homes	Putative membrane protein YfcA	0.000696	0	0	0	0.002626	0.004383	0.004333	0.000498	0.000657	0	0.008383	0.001814183
Respiration	NULL	Quinone oxidoreductase family	Putative oxidoreductase SMc00968	0.000348	0	0	0	0.000657	0.001948	0.001444	0.003489	0.000876	0.006608	0	0.000236633
Miscellaneous	Plant-Prokaryote DOE project	COG0398	Putative phosphatidylglycerophosphate synthase	0	0	0	0.00249	0	0.001948	0.00361	0.001495	0.00416	0.008811	0.002911	0.000828214
Respiration	Electron donating reactions	Succinate dehydrogenase	Putative succinate dehydrogenase cytochrome b subunit	0	0	0.010219	0	0.002626	0	0.001444	0.002492	0.000219	0.008811	0.000349	0.00378612
Clustering-based subsystems	Carbohydrates	Putative sugar ABC transporter (ytf cluster)	Putative sugar ABC transport system, periplasmic binding protein YtfQ precursor	0.000696	0.002262	0	0.00249	0	0.000974	0.000722	0	0.001095	0	0.004541	0.002287448
RNA Metabolism	Transcription	Transcription elongation factors, archaeal	Putative transcription antitermination protein NusG	0.003133	0	0	0	0.003939	0.002922	0.002888	0.001993	0.000876	0	0.000233	0.001104285
Regulation and Cell signaling	NULL	Two-component regulatory systems in Campylobacter	Putative two-component response regulator	0.000348	0	0	0	0.000657	0	0.000722	0.001495	0.000876	0.004406	0.000233	0.012462647
Regulation and Cell signaling	NULL	Two-component regulatory systems in Campylobacter	Putative two-component sensor histidine kinase	0.005571	0	0.007664	0	0.00197	0.003409	0.002888	0.002492	0.000657	0	0	0.034272278
Miscellaneous	Plant-Prokaryote DOE project	At5g63420	Putative Zn-dependent hydrolase in polyisoprenoid biosynthetic cluster	0.002785	0.002262	0.002555	0	0.00197	0	0.000722	0.001993	0.000438	0	0	0.000749336
Amino Acids and Derivatives	Arginine; urea cycle, polyamines	Putrescine utilization pathways	Putrescine aminotransferase (EC 2.6.1.82)	0.000348	0.004524	0.002555	0	0.00197	0.005357	0	0.000498	0	0.008811	0	0.003233978
Iron acquisition and metabolism	Siderophores	Siderophore Pyoverdine	Pyoverdine chromophore precursor synthetase PvdL	0	0.002262	0.002555	0	0.00197	0.000974	0.004333	0.004984	0.003941	0	0	0.002721274
Iron acquisition and metabolism	Siderophores	Siderophore Pyoverdine	Pyoverdine efflux carrier and ATP binding protein	0.002437	0.002262	0	0	0.000657	0.000974	0.002166	0.000498	0.001752	0	0	0.000985969
Cofactors, Vitamins, Prosthetic Groups, Pigments	Pyridoxine	Pyridoxin (Vitamin B6) Biosynthesis	Pyridoxal kinase (EC 2.7.1.35)	0	0	0	0.00747	0.001313	0.002435	0.002166	0.00299	0.000657	0	0.000233	0.001616989
Protein Metabolism	Protein biosynthesis	Pyrrolysine	pyrrolysine-containing	0	0.004524	0.00511	0.00249	0.009192	0.014124	0.002888	0.000997	0	0	0	0.002090254
Cell Wall and Capsule	Gram-Positive cell wall components	Teichoic and lipoteichoic acids biosynthesis	Regulation of D-alanyl-lipoteichoic acid biosynthesis, DltR	0	0.006786	0.002555	0	0.000657	0	0.000722	0	0.000657	0.006608	0.000349	0.002879029
Regulation and Cell signaling	Quorum sensing and biofilm formation	Quorum-sensing in Vibrio	Regulatory protein LuxO	0.001393	0	0	0	0.000657	0.000487	0.000722	0.002492	0.000657	0	0.001979	0.001183163
Metabolism of Aromatic Compounds	NULL	Benzoate transport and degradation cluster	Regulatory protein of benzoate catabolism	0	0.004524	0.002555	0	0.003939	0.004383	0.00361	0.003987	0.003941	0	0	0.000788775
Clustering-based subsystems	recX and regulatory cluster	CBSS-261594.1.peg.788	Regulatory protein RecX	0	0.006786	0.007664	0	0.001313	0.000487	0.001444	0.002492	0.003284	0	0	0.001656428
Miscellaneous	Plant-Prokaryote DOE project	Single-Rhodanese-domain proteins	Rhodanese domain protein UPF0176, cyanobacterial/alphaproteobacterial subgroup	0.007659	0.002262	0	0	0	0.001948	0.00361	0.003987	0.002846	0	0.000233	0.000907091
Carbohydrates	CO2 fixation	Calvin-Benson cycle	Ribose 1,5-bisphosphate or 5-ribose-1,2-cyclic phosphate dehydrogenase	0	0.004524	0.002555	0.00498	0.002626	0.002922	0.002166	0	0.000438	0	0	0.002287448

Carbohydrates	NULL	Sugar utilization in Thermotogales	Ribose ABC transport system, high affinity permease RbsD (TC 3.A.1.2.1)	0.000696	0.002262	0	0	0.000657	0.000487	0.000722	0	0.000219	0	0.000116	0.000473265
RNA Metabolism	RNA processing and modification	rRNA modification Bacteria	Ribosomal RNA small subunit methyltransferase C (EC 2.1.1.52)	0	0	0	0	0.001313	0.002435	0.005055	0.003987	0.007225	0.006608	0.011643	0.003746682
Cofactors, Vitamins, Prosthetic Groups, Pigments	NAD and NADP	NAD regulation	Ribosyl nicotinamide transporter, PnuC-like	0	0	0.002555	0	0	0.002922	0.002166	0.002492	0.005474	0.004406	0.005007	0.00729617
RNA Metabolism	Transcription	Transcription initiation, bacterial sigma factors	RNA polymerase sigma factor SigW	0.000696	0.004524	0	0.00249	0.005252	0.001461	0	0.003489	0.003284	0	0	0.002366325
Cell Wall and Capsule	Capsular and extracellular polysacchrides	Alginate metabolism	RNA polymerase sigma-H factor AlgT	0.008704	0	0	0.00498	0.001313	0.000974	0	0	0.000876	0.004406	0.007917	0.001498673
RNA Metabolism	RNA processing and modification	tRNA splicing	RNA:NAD 2'-phosphotransferase	0.001044	0	0	0	0.006566	0.00487	0.000722	0.001495	0.002627	0	0.000582	0.002405764
Stress Response	NULL	SigmaB stress responce regulation	RsbR, positive regulator of sigma-B	0	0	0	0.00498	0.007222	0.003409	0.00361	0.001495	0.001752	0	0.000349	0.002760713
Amino Acids and Derivatives	Lysine, threonine, methionine, and cysteine	Methionine Biosynthesis	S-adenosylhomocysteine nucleosidase (EC 3.2.2.9)	0	0	0.002555	0.00249	0.000657	0	0.001444	0.000498	0.000657	0	0.016999	0.001104285
Clustering-based subsystems	Methylamine utilization	Glutamate-mediated methylamine utilization pathway	Sarcosine oxidase gamma subunit (EC 1.5.3.1)	0	0.002262	0	0	0.001313	0	0.001444	0.002492	0.001095	0.002203	0.00524	0.000788775
Regulation and Cell signaling	NULL	Orphan regulatory proteins	Sensor kinase CitA, DpiB (EC 2.7.3.-)	0.001393	0	0	0.00498	0.001313	0.000487	0.001444	0.000498	0.000876	0	0	0.004338263
Cell Wall and Capsule	Gram-Negative cell wall components	Lipid A modifications	Sensor protein PhoQ (EC 2.7.13.3)	0.001393	0	0	0	0.000657	0.001948	0.005777	0.000997	0.001752	0	0.008383	0.002405764
Respiration	Electron accepting reactions	trimethylamine N-oxide (TMAO) reductase	Sensor protein torS (EC 2.7.3.-)	0	0.002262	0.002555	0	0	0	0.000722	0.003987	0.002846	0.004406	0.000582	0.002366325
Membrane Transport	Protein secretion system, Type VI	Type VI secretion systems	Serine/threonine protein kinase (EC 2.7.11.1) PpkA	0.013926	0.004524	0	0	0.005252	0.002435	0.002166	0.003987	0.007882	0	0	0.005915813
Clustering-based subsystems	Catabolism of an unknown compound	CBSS-262316.1.peg.2929	Short-chain dehydrogenase/reductase in hypothetical Actinobacterial gene cluster	0.000696	0	0.00511	0	0.00197	0.000974	0	0.000498	0.002846	0.002203	0	0.001301479
Clustering-based subsystems	Proteasome related clusters	Proteasome subunit alpha archaeal cluster	Shwachman-Bodian-Diamond syndrome protein	0	0	0	0.00747	0.001313	0.002435	0.00361	0.000498	0.001095	0.002203	0	0.001143724
Protein Metabolism	Protein biosynthesis	Ribosome SSU eukaryotic and archaeal	SSU ribosomal protein S13e (S15p)	0	0	0	0.00249	0.000657	0.000487	0.005777	0	0.000876	0.002203	0.000582	0.001222601
Protein Metabolism	Protein biosynthesis	Ribosome SSU eukaryotic and archaeal	SSU ribosomal protein S15Ae (S8p)	0	0.002262	0	0	0.001313	0.001461	0.001444	0.000997	0.001314	0.002203	0	0.001853621
Protein Metabolism	Protein biosynthesis	Ribosome SSU eukaryotic and archaeal	SSU ribosomal protein S15e (S19p)	0	0	0	0.00498	0.002626	0.002435	0.007943	0.001993	0.001314	0	0.000116	0.002326887
Protein Metabolism	Protein biosynthesis	Ribosome SSU eukaryotic and archaeal	SSU ribosomal protein S28e	0	0.004524	0	0.00498	0.003939	0.000974	0.006499	0.001495	0.001095	0	0	0.00094653
Protein Metabolism	Protein biosynthesis	Ribosome SSU eukaryotic and archaeal	SSU ribosomal protein S6e	0.000348	0.004524	0	0	0	0.001461	0.000722	0.001495	0.000657	0	0.000116	0.000828214
Protein Metabolism	Protein biosynthesis	Ribosome SSU eukaryotic and archaeal	SSU ribosomal protein S8e	0.001393	0	0	0.00249	0.00197	0	0.002166	0.00299	0.000438	0	0.000116	0.000867653
Protein Metabolism	Protein biosynthesis	Ribosome SSU eukaryotic and archaeal	SSU ribosomal protein S9e (S4p)	0.002785	0.002262	0	0	0.008535	0.001948	0.00361	0	0.001533	0	0.000116	0.001538111
Clustering-based subsystems	Biosynthesis of galactoglycans and related lipopolysacharides	CBSS-258594.1.peg.3339	STRUCTURAL ELEMENTS; Cell Exterior; surface polysaccharides/antigens	0	0	0.00511	0	0.002626	0.001948	0.005055	0.004984	0.007882	0	0.000466	0.002169132
Virulence, Disease and Defense	NULL	Bacterial cyanide production and tolerance mechanisms	Sulfate transporter family protein in cluster with carbonic anhydrase	0	0	0	0	0.00197	0.002435	0.002166	0.002492	0.001314	0.002203	0.000349	0.000236633
Respiration	Electron donating reactions	Hydrogenases	Sulfhydrogenase II subunit d	0.002089	0.002262	0.00511	0	0.00197	0.000487	0.001444	0.000498	0	0	0	0.00031551
Sulfur Metabolism	Organic sulfur assimilation	Alkanesulfonate assimilation	sulfonate monooxygenase	0	0	0	0	0.000657	0.002922	0.006499	0.002492	0.007006	0.006608	0.000466	0.002800152
Miscellaneous	Plant-Prokaryote DOE project	At1g54520	Sulfur carrier protein ThiS	0	0.002262	0	0	0.00197	0.000974	0.000722	0.001495	0.003065	0	0.000116	0.001459234
Stress Response	Oxidative stress	Protection from Reactive Oxygen Species	Superoxide dismutase [Cu-Zn] precursor (EC 1.15.1.1)	0	0	0.002555	0	0	0.002435	0.000722	0.004485	0.004817	0.002203	0.000466	0.001932499
Carbohydrates	Polysaccharides	Cellulosome	SusD, outer membrane protein	0	0.002262	0	0	0.000657	0.001948	0.002166	0.003987	0.002408	0.022028	0	0.014316268
Carbohydrates	Di- and oligosaccharides	Lactose and Galactose Uptake and Utilization	Tagatose 1,6-diphosphate aldolase (EC 4.1.2.40)	0.002437	0	0	0	0.003283	0.002922	0.000722	0.002492	0.001971	0	0.000116	0.000985969
Membrane Transport	Sugar Phosphotransferase Systems, PTS	Galactose-inducible PTS	Tagatose-6-phosphate kinase (EC 2.7.1.144) / 1-phosphofructokinase (EC 2.7.1.56)	0.002437	0.002262	0	0	0.00197	0.000974	0	0.000997	0.001314	0	0.000116	0.00063102
Respiration	Electron accepting reactions	Tetrathionate respiration	Tetrathionate reductase subunit C	0	0.004524	0.007664	0	0.003939	0.002922	0.001444	0.000498	0.002627	0	0	0.004693212
Cofactors, Vitamins, Prosthetic Groups, Pigments	Folate and pterines	5-FCL-like protein	Thiaminase II (EC 3.5.99.2)	0	0	0.007664	0	0.007222	0.005357	0.006499	0.003489	0.006349	0	0.000582	0.001064846
Iron acquisition and metabolism	Siderophores	Siderophore assembly kit	TonB-dependent hemin , ferrichrome receptor	0.000348	0	0	0	0.000657	0.000487	0.005055	0.003987	0.000219	0	0.000233	0.000552143
Regulation and Cell signaling	NULL	CytR regulation	Transcriptional (co)regulator CytR	0.000696	0.002262	0	0	0.000657	0.000974	0.000722	0.000997	0.000657	0	0	0.001104285
Membrane Transport	ABC transporters	Periplasmic-Binding-Protein-Dependent Transport System for -Glucosides	Transcriptional regulator AglR, LacI family	0	0.006786	0.002555	0	0.002626	0.000974	0.000722	0.000498	0.001752	0	0	0.000749336
Carbohydrates	Monosaccharides	Fructose utilization	Transcriptional regulator FrcR for fructose utilization, ROK family	0.000348	0.002262	0	0	0.00197	0.000487	0	0.000498	0.002189	0.002203	0	0.000985969
Carbohydrates	Monosaccharides	L-rhamnose utilization	Transcriptional regulator of rhamnose utilization, DeoR family	0	0.004524	0	0.00249	0.000657	0.000974	0.000722	0.002492	0.001314	0	0	0.000907091
Cofactors, Vitamins, Prosthetic Groups, Pigments	Folate and pterines	YgfZ-Iron	Transcriptional regulator, FUR family	0.002437	0.002262	0.00511	0	0	0.000974	0.002888	0.001495	0.000219	0	0	0.000709898
Regulation and Cell signaling	NULL	Orphan regulatory proteins	Transcriptional regulatory protein RstA	0	0.002262	0	0	0.002626	0.000487	0.000722	0.001495	0.003284	0.002203	0	0.004929844
Nitrogen Metabolism	NULL	Nitrogen fixation	Transcriptional repressor of nif and glnA operons	0.00383	0.002262	0.002555	0.00249	0.000657	0.000487	0	0.000498	0	0	0	0.000552143
Amino Acids and Derivatives	Aromatic amino acids and derivatives	Common Pathway For Synthesis of Aromatic Compounds (DAHP synthase to chorismate)	Transcriptional repressor protein TyrR	0.03969	0	0	0	0	0.001461	0.000722	0.002492	0.014013	0.006608	0.000466	0.001419795
Cell Wall and Capsule	Gram-Negative cell wall components	Vibrio Core Oligosaccharide Biosynthesis	transferase, hexapeptide repeat family	0	0.002262	0.002555	0	0.000657	0.000487	0.000722	0.000498	0.001533	0	0	0.001616989
Membrane Transport	NULL	Tricarboxylate transport system	Tricarboxylate transport transcriptional regulator TctD	0.004526	0.002262	0	0	0.001313	0.000974	0	0.000498	0.002408	0	0.000116	0.003036784
RNA Metabolism	RNA processing and modification	tRNA modification Archaea	tRNA cytosine methylase Pos56	0.004178	0	0	0	0.00197	0.001461	0.000722	0.00299	0.001314	0.004406	0	0.000512704
RNA Metabolism	RNA processing and modification	Polyadenylation bacterial	tRNA nucleotidyltransferase, CC-adding (EC 2.7.7.21)	0	0	0.022993	0	0.00197	0.001461	0.00361	0.000498	0.001752	0	0.000699	0.000552143
RNA Metabolism	RNA processing and modification	tRNA processing	tRNAHis-5'-guanylyltransferase	0	0	0.002555	0	0.003283	0.002922	0.005055	0.000997	0.000876	0	0.000116	0.000985969
Regulation and Cell signaling	NULL	Staphylococcal accessory gene regulator system	Two component transcriptional regulator VraR	0.004178	0.002262	0	0.00498	0.001313	0.000974	0.000722	0	0.001533	0	0	0.002879029
Regulation and Cell signaling	NULL	Orphan regulatory proteins	Two-component sensor histidine kinase PleC	0.006963	0	0.002555	0	0.010505	0.01315	0.015886	0.021429	0.016421	0	0	0.007020098
Regulation and Cell signaling	NULL	Orphan regulatory proteins	Two-component system response regulator QseB	0.002785	0	0.00511	0.00249	0	0.000974	0	0.000498	0.003941	0	0.0085	0.003983314
Respiration	Electron accepting reactions	Terminal cytochrome C oxidases	Type cbb3 cytochrome oxidase biogenesis protein CcoS, involved in heme b insertion	0	0.004524	0.00511	0	0.005252	0.003409	0.000722	0.007974	0.007225	0	0	0.007690557
Membrane Transport	Protein secretion system, Type III	Type III secretion systems	Type III effector	0	0.004524	0	0	0.001313	0.000974	0.000722	0	0.001314	0.002203	0.014671	0.002484642
Membrane Transport	Protein secretion system, Type III	Type III secretion system	Type III secretion cytoplasmic ATP synthase (EC 3.6.3.14, YscN,SpaL,MxiB,HrcN,EscN)	0	0	0.00511	0	0.003939	0.002435	0.005055	0.003489	0.002846	0	0.000699	0.001774744
Membrane Transport	Protein secretion system, Type III	Type III secretion system	Type III secretion inner membrane protein (YscR,SpaR,HrcR,EscR,homologous to flagellar export components)	0	0	0.007664	0	0.005252	0.000487	0.002166	0.000997	0.000657	0	0.000116	0.001064846
Cell Wall and Capsule	Capsular and extracellular polysacchrides	Legionaminic Acid Biosynthesis	UDP-Bac2Ac4Ac hydrolyzing 2-epimerase NeuC homolog	0	0	0	0	0.002626	0.007306	0.005777	0.00299	0.003065	0.006608	0.000233	0.005758058

Cell Wall and Capsule	NULL	Peptidoglycan biosynthesis--gjo	UDP-N-acetylmuramoylalanyl-D-glutamate--L-ornithine ligase	0.009052	0.002262	0.00511	0	0	0.001948	0.000722	0.001495	0.000657	0	0	0.001025408
Respiration	NULL	Methanogenesis strays	Uncharacterized HTH-type transcriptional regulator MJ0272	0.000348	0.002262	0	0	0.000657	0.000487	0.001444	0.001495	0.001752	0	0	0.002326887
Membrane Transport	Protein secretion system, Type VI	Type VI secretion systems	Uncharacterized protein ImpD	0.010445	0.004524	0	0.00249	0.00197	0	0	0	0.001314	0.002203	0.000116	0.002050815
Clustering-based subsystems	NULL	USS-DB-7	Uncharacterized protein ImpI/VasC	0	0.002262	0	0	0	0.001461	0.002166	0.001993	0.004598	0.002203	0.000582	0.000907091
Carbohydrates	NULL	Sugar utilization in Thermotogales	Unknown pentose kinase TM0952	0	0	0.002555	0	0.002626	0.002435	0.000722	0.001495	0.001095	0	0.000116	0.001538111
Clustering-based subsystems	Monosaccharides	Unspecified monosaccharide transport cluster	Unspecified monosaccharide ABC transport system, substrate-binding component	0.001393	0.002262	0	0	0.00197	0.001461	0.000722	0.000997	0.000657	0	0	0.001774744
Clustering-based subsystems	Pyruvate kinase associated cluster	CBSS-288000.5.peg.1793	UPF0335 protein R02793	0	0	0	0.00249	0.001313	0.001948	0.007221	0.003489	0.001752	0.004406	0	0.000157755
Nitrogen Metabolism	NULL	Allantoin Utilization	Ureidoglycolate dehydrogenase (EC 1.1.1.154)	0.001044	0	0.010219	0	0.001313	0.001948	0.001444	0.004485	0.00416	0	0	0.001222601
Nitrogen Metabolism	NULL	Allantoin Utilization	Ureidoglycolate hydrolase (EC 3.5.3.19)	0	0	0	0.00249	0	0.002435	0.002888	0.001993	0.002408	0.004406	0.050067	0.001814183
Clustering-based subsystems	NULL	ClpS like protein and vWFA-like protein cluster	vWFA-like protein with metal ion dependent adhesion motif (MIDAS)	0	0	0	0.00249	0.004596	0.002922	0.002888	0.009469	0.005255	0	0.000233	0.000709898
Miscellaneous	Plant-Prokaryote DOE project	At4g38090	Xaa-Pro dipeptidase PepQ (EC 3.4.13.9)	0	0	0	0.00249	0.000657	0.001948	0.002166	0.006977	0.018173	0	0.000233	0.003233978
Carbohydrates	NULL	Sugar utilization in Thermotogales	Xylan oligosaccharide ABC transporter, permease component 2	0.000348	0.004524	0.002555	0	0.000657	0.002435	0.002888	0	0.002627	0	0	0.003194539
Carbohydrates	Monosaccharides	Xylose utilization	Xylose ABC transporter, ATP-binding component	0.000348	0	0.007664	0	0.000657	0.001461	0	0.001993	0.000219	0.002203	0	0.001183163
Regulation and Cell signaling	Programmed Cell Death and Toxin-antitoxin Systems	Toxin-antitoxin systems (other than RelBE and MazEF)	YafQ toxin protein	0	0.004524	0.00511	0	0.003939	0.003409	0	0.001495	0.000219	0	0.000233	0.001498673
Clustering-based subsystems	NULL	CBSS-243277.1.peg.4359	YcgN (Fragment)	0	0	0.00511	0	0.001313	0.001948	0.000722	0.002492	0.006568	0	0.012458	0.002879029
Clustering-based subsystems	NULL	NusA-TFII Cluster	YlxP-like protein	0	0	0.002555	0	0.004596	0.003409	0.000722	0.000997	0.001095	0	0.000116	0.00157755
RNA Metabolism	RNA processing and modification	tRNA modification Bacteria	YrdC/Sua5 family protein, required for threonylcarbamoyladenosine (t(6)A) formation in tRNA	0	0	0	0	0.000657	0.000487	0.001444	0.000997	0.001971	0.002203	0.000349	0.001656428
Carbohydrates	Aminosugars	(GlcNAc)2 Catabolic Operon	(GlcNAc)2 ABC transporter, periplasmic substrate-binding protein	0	0	0.007664	0	0.001313	0	0.001444	0.000498	0.001971	0	0.000116	0.000512704
Amino Acids and Derivatives	Arginine; urea cycle, polyamines	Anaerobic Oxidative Degradation of L-Ornithine	2,4-diaminopentanoate dehydrogenase (EC 1.4.1.12)	0	0	0	0.00249	0	0.000487	0.00361	0.002492	0.001314	0	0.000815	0.002879029
Cofactors, Vitamins, Prosthetic Groups, Pigments	Quinone cofactors	Menaquinone and Phylloquinone Biosynthesis	2-heptaprenyl-1,4-naphthoquinone methyltransferase (EC 2.1.1.-)	0.002785	0	0	0	0.003283	0.004383	0.002166	0.002492	0.001314	0	0	0.001971938
Miscellaneous	Plant-Prokaryote DOE project	At3g50560	2-hydroxychromene-2-carboxylate isomerase family protein, glutathione-dependent	0.002785	0.002262	0	0	0.000657	0.000487	0	0.000997	0.000657	0	0	0.000276071
Cofactors, Vitamins, Prosthetic Groups, Pigments	Quinone cofactors	Menaquinone and Phylloquinone Biosynthesis	2-succinyl-6-hydroxy-2,4-cyclohexadiene-1-carboxylate synthase (EC 4.2.99.20)	0	0	0.002555	0	0.000657	0.000487	0	0.001495	0.000876	0	0.000116	0.000157755
Metabolism of Aromatic Compounds	Metabolism of central aromatic intermediates	Central meta-cleavage pathway of aromatic compound degradation	3,4-dihydroxyphenylacetate 2,3-dioxygenase	0	0	0.002555	0	0.00197	0	0	0.000997	0.001971	0.004406	0.012808	0.005481987
Amino Acids and Derivatives	Lysine, threonine, methionine, and cysteine	Lysine fermentation	3,5-diaminobexanoate dehydrogenase (EC 1.4.1.11)	0.002785	0	0	0.00498	0	0.001948	0.001444	0.000997	0	0	0.000699	0.001616989
Metabolism of Aromatic Compounds	NULL	Benzoate transport and degradation cluster	4-hydroxybenzoyl-CoA reductase, gamma subunit (EC 1.3.99.20)	0.001741	0.002262	0	0	0.005909	0.002922	0	0.000997	0.004817	0	0	0.003036784
Carbohydrates	Sugar alcohols	Inositol catabolism	5-keto-2-deoxy-D-gluconate-6 phosphate aldolase (EC 4.1.2.29)	0	0.004524	0.00511	0	0	0.001461	0.001444	0.000997	0.000438	0	0	0.000354949
Amino Acids and Derivatives	Lysine, threonine, methionine, and cysteine	Methionine Salvage	5-methylthioribose kinase (EC 2.7.1.100)	0	0.002262	0	0	0.001313	0.004383	0	0.001495	0.003284	0	0.002678	0.002287448
Respiration	Electron accepting reactions	Terminal AA3-600 quinol oxidase	AA3-600 quinol oxidase subunit I	0.006615	0	0.002555	0	0	0	0.000722	0.001495	0.001533	0	0.000116	0.000433826
Membrane Transport	Protein secretion system, Type I	Type I protein secretion systems	ABC exporter for hemopore HasA, ATP-binding component HasD	0.000348	0	0	0	0.000657	0.000487	0.005055	0.00299	0.001752	0	0	0.001971938
Iron acquisition and metabolism	NULL	ABC-type iron transport system	ABC-type Fe3+ transport system, periplasmic component	0.002437	0.002262	0.00511	0.00249	0	0.001461	0	0	0.000657	0	0	0.001814183
Iron acquisition and metabolism	Siderophores	Siderophore assembly kit	ABC-type Fe3+-siderophore transport system, permease component	0	0	0	0.00498	0.001313	0.001461	0	0.001495	0.002189	0.002203	0	0.000867653
Membrane Transport	Protein secretion system, Type I	Type I protein secretion systems	ABC-type protease exporter, ATP-binding component PrtD/AprD	0	0.004524	0	0	0	0.000487	0.002166	0.001993	0.003941	0	0.000349	0.002366325
Carbohydrates	Di- and oligosaccharides	Maltose and Maltodextrin Utilization	ABC-type sugar transport system, periplasmic binding protein YcjN	0	0.002262	0	0	0.001313	0.000974	0.000722	0.001993	0.000876	0	0	0.000197194
Carbohydrates	Fermentation	Butanol Biosynthesis	Acetaldehyde dehydrogenase (acetylating) (EC 1.2.1.10)	0	0.002262	0	0	0.001313	0.000487	0.000722	0.000997	0.000876	0	0	0.000828214
Carbohydrates	Fermentation	Acetone Butanol Ethanol Synthesis	Acetoacetate decarboxylase (EC 4.1.1.4)	0.003133	0	0.002555	0	0	0.000974	0.000722	0	0.000438	0	0.000116	0.000236633
Carbohydrates	NULL	Acetone carboxylase	Acetone carboxylase, alpha subunit (EC 6.4.1.6)	0	0.002262	0	0	0.000657	0.000487	0	0	0.000657	0.002203	0.13646	0.00252408
Carbohydrates	Fermentation	Acetone Butanol Ethanol Synthesis	Acetyl-CoA:acetoacetyl-CoA transferase, beta subunit (EC 2.8.3.8)	0.000348	0	0.002555	0	0	0	0.000722	0	0.001752	0.002203	0.000116	0.001853621
Amino Acids and Derivatives	Arginine; urea cycle, polyamines	Arginine Biosynthesis extended	Acetylornithine aminotransferase 1 (EC 2.6.1.11)	0	0	0.00511	0	0.000657	0.000487	0	0.000498	0.000876	0	0.000466	0.000591581
Membrane Transport	NULL	Transport of Nickel and Cobalt	Additional substrate-specific component NikN of nickel ECF transporter	0.005919	0.004524	0.002555	0	0.001313	0	0	0	0.001752	0.004406	0	0.001656428
Amino Acids and Derivatives	Arginine; urea cycle, polyamines	Polyamine Metabolism	Agmatine/putrescine antiporter, associated with agmatine catabolism	0	0	0.002555	0	0.00197	0	0.000722	0.000997	0.000219	0	0.000116	0.00031551
Carbohydrates	Fermentation	Butanol Biosynthesis	Alcohol dehydrogenase (EC 1.1.1.1); Acetaldehyde dehydrogenase (EC 1.2.1.10)	0	0.002262	0	0	0.001313	0.001948	0.001444	0.000498	0.000438	0	0	0.001656428
Clustering-based subsystems	Catabolism of an unknown compound	CBSS-262316.1.peg.2929	Aldehyde dehydrogenase in hypothetical Actinobacterial gene cluster	0	0.002262	0.002555	0	0.00197	0	0.000722	0	0.001533	0	0.000116	0.000512704
Cofactors, Vitamins, Prosthetic Groups, Pigments	Folate and pterines	Pterin biosynthesis	Aldo-keto reductase family 1 member B10 (EC 1.1.1.-)	0.004178	0	0	0	0.000657	0.000487	0.002888	0.005482	0.002408	0	0	0.001380356
Protein Metabolism	Protein processing and modification	N-linked Glycosylation in Bacteria	Alpha-1,4-N-acetylgalactosamine transferase PglJ (EC 2.4.1.-)	0	0	0.002555	0	0.000657	0.001461	0.001444	0.00299	0.002408	0	0	0.001459234
Miscellaneous	Plant-Prokaryote DOE project	COG3533	Alpha-arabinosides ABC transport system, permease protein 1	0.000348	0.004524	0.002555	0	0	0.001948	0.002166	0	0.000438	0	0	0.000433826
Membrane Transport	ABC transporters	Periplasmic-Binding-Protein-Dependent Transport System for -Glucosides	Alpha-glucosidase AglA	0.000348	0.002262	0	0	0.000657	0.006332	0	0.008472	0.007225	0	0	0.001774744
Carbohydrates	Polysaccharides	Cellulosome	Alpha-glucosidase SusB (EC 3.2.1.20)	0	0.002262	0.002555	0.00498	0	0.001948	0.000722	0	0.001533	0	0	0.002721274
Carbohydrates	Aminosugars	Chitin and N-acetylglucosamine utilization	Alpha-N-acetylglucosaminidase (EC 3.2.1.50)	0	0.004524	0	0	0	0.000487	0	0.000498	0.000219	0.004406	0.001164	0.000749336
Carbohydrates	Monosaccharides	L-Arabinose utilization	Alternative L-arabinose isomerase (EC 5.3.1.4)	0.001393	0	0	0	0.000657	0.000974	0.001444	0.000498	0.000438	0	0	0.001695866
Carbohydrates	Monosaccharides	D-Galacturonate and D-Glucuronate Utilization	Altronate oxidoreductase (EC 1.1.1.58)	0.001044	0	0	0	0.001313	0	0.002888	0.002492	0.004817	0	0.000699	0.005955252
Membrane Transport	NULL	Tricarboxylate transport system	Ammonia monooxygenase	0	0.002262	0	0.00249	0	0.000974	0.000722	0.000498	0.000876	0	0	0.000118316
Carbohydrates	Central carbohydrate metabolism	TCA Cycle	Archaeal succinyl-CoA ligase [ADP-forming] alpha chain (EC 6.2.1.5)	0.002437	0.002262	0	0	0.002626	0.005357	0	0.001495	0.000219	0	0	0.001932499
Carbohydrates	Central carbohydrate metabolism	TCA Cycle	Archaeal succinyl-CoA ligase [ADP-forming] beta chain (EC 6.2.1.5)	0.000696	0.002262	0	0	0.004596	0.001461	0.001444	0	0	0	0.000116	0.00126204
Amino Acids and Derivatives	Arginine; urea cycle, polyamines	Arginine and Ornithine Degradation	Arginine/ornithine ABC transporter, permease protein AotQ	0	0.002262	0	0	0.002626	0.002922	0.000722	0.000498	0.002846	0	0	0.001143724
Amino Acids and Derivatives	Aromatic amino acids and derivatives	Phenylalanine and Tyrosine Branches from Chorismate	Arogenate dehydratase (EC 4.2.1.91)	0.003482	0.002262	0	0.00498	0	0.001461	0	0	0.000438	0	0.000116	0.000907091

Nucleosides and Nucleotides	Pyrimidines	De Novo Pyrimidine Synthesis	Aspartate carbamoyltransferase regulatory chain (PyrI)	0	0	0	0	0.00197	0.000974	0.004333	0.001993	0.002846	0	0.000233	0.001735305
Protein Metabolism	Protein degradation	Proteasome bacterial	ATP-dependent protease La (EC 3.4.21.53) LonB Type I	0.000348	0.002262	0	0	0.001313	0.002435	0.002166	0.001993	0	0	0	0.001735305
RNA Metabolism	RNA processing and modification	ATP-dependent RNA helicases, bacterial	ATP-dependent RNA helicase SrmB	0	0.004524	0	0	0	0.000487	0.001444	0.005482	0.007444	0	0.027478	0.004811528
RNA Metabolism	RNA processing and modification	ATP-dependent RNA helicases, bacterial	ATP-dependent RNA helicase VVA0939	0	0	0.002555	0	0.000657	0	0.000722	0	0.000219	0.006608	0.06334	0.00189306
Clustering-based subsystems	proteosome related	Cluster-based Subsystem Grouping Hypothetics - perhaps Proteosome Related	Bacterial proteasome-activating AAA-ATPase (PAN)	0	0.002262	0	0.00249	0.003283	0.003409	0	0	0.001314	0.008811	0	0.00094653
Clustering-based subsystems	NULL	Aerotolerance operon in Bacteroides and potentially orthologous operons in other organisms	BatC	0	0.002262	0	0	0.000657	0.001948	0	0.003987	0.001095	0.006608	0	0.007098976
Metabolism of Aromatic Compounds	Peripheral pathways for catabolism of aromatic compounds	Benzoate degradation	Benzoylformate decarboxylase (EC 4.1.1.7)	0.000348	0.004524	0	0	0.000657	0.001461	0.002888	0	0.004379	0	0	0.000828214
Carbohydrates	Di- and oligosaccharides	Lactose and Galactose Uptake and Utilization	Beta-galactosidase (EC 3.2.1.23), LacA family	0	0	0	0	0.000657	0.001461	0.001444	0.000997	0.000438	0.002203	0	0.000394388
RNA Metabolism	RNA processing and modification	16S rRNA modification within P site of ribosome	BH2577 unknown conserved protein in B. subtilis	0	0.002262	0.002555	0	0.000657	0.002435	0	0.004485	0.002408	0	0	0.000788775
Cell Wall and Capsule	Capsular and extracellular polysacchrides	Rhamnose containing glycans	Capsular polysaccharide biosynthesis/export periplasmic protein WcbA	0.000696	0.002262	0	0	0	0.001461	0	0.001993	0.003941	0.013217	0	0.000473265
Cell Wall and Capsule	Capsular and extracellular polysacchrides	Capsular Polysaccharide (CPS) of Campylobacter	Capsular polysaccharide export system periplasmic protein KpsD	0	0	0	0	0.002626	0.000487	0.002166	0.000498	0.001533	0	0.022705	0.001380356
Cell Wall and Capsule	Capsular and extracellular polysacchrides	Rhamnose containing glycans	Capsular polysaccharide export system protein KpsC	0	0	0	0.00996	0.000657	0.000974	0.000722	0.00299	0.000657	0	0	0.001104285
Cell Wall and Capsule	Capsular and extracellular polysacchrides	Serotype determining Capsular polysaccharide biosynthesis in Staphylococcus	Capsular polysaccharide synthesis enzyme Cap8O	0	0.002262	0	0.00249	0	0	0.001444	0.000498	0.001095	0.002203	0	0.000276071
Cell Wall and Capsule	Capsular and extracellular polysacchrides	Exopolysaccharide Biosynthesis	Capsular polysaccharide synthesis enzyme CpsA, sugar transferase	0	0.002262	0.007664	0	0	0.000974	0	0.001495	0.002627	0	0.032718	0.004219947
Amino Acids and Derivatives	Arginine; urea cycle, polyamines	Polyamine Metabolism	Carboxynorspermidine dehydrogenase, putative (EC 1.1.1.-)	0.006615	0.002262	0	0	0	0.001461	0	0.000498	0.001752	0	0.000815	0.003036784
Regulation and Cell signaling	NULL	HPr catabolite repression system	Catabolite control protein A	0.003133	0	0	0.00498	0.000657	0.001461	0	0	0.001752	0	0.000116	0.001104285
Cell Wall and Capsule	Gram-Positive cell wall components	Teichoic and lipoteichoic acids biosynthesis	CDP-glycerol: N-acetyl-beta-D-mannosaminyl-1,4-N-acetyl-D-glucosaminyldiphosphoundecaprenyl glycerophosphotransferase	0	0	0.002555	0	0	0.001948	0.000722	0	0.001533	0.004406	0.004774	0.001301479
Carbohydrates	Aminosugars	Chitin and N-acetylglucosamine utilization	Chitodextrinase precursor (EC 3.2.1.14)	0	0	0.002555	0	0	0	0.000722	0.000498	0.001971	0.026433	0.002562	0.00094653
Membrane Transport	NULL	Choline Transport	Choline ABC transporter ATP-binding protein	0	0.002262	0	0.00249	0	0.000974	0	0	0.001095	0.002203	0.000349	0.000709898
Secondary Metabolism	Biosynthesis of phenylpropanoids	Caffeic acid derivatives	Cinnamoyl-CoA reductase (EC 1.2.1.44)	0.000348	0	0	0.00249	0	0.000487	0.001444	0.001495	0.000876	0	0	0.001104285
Cell Division and Cell Cycle	NULL	Cyanobacterial Circadian Clock	Circadian oscillation regulator KaiB	0	0	0	0	0.001313	0.002435	0.002166	0	0.001095	0.002203	0.000116	0.000670459
Respiration	Electron donating reactions	H2:CoM-S-S-HTP oxidoreductase	CoB--CoM-reducing hydrogenase (Sec) alpha subunit	0.004874	0.002262	0	0.00249	0.00197	0.002922	0	0.000997	0	0	0	0.001183163
Respiration	Electron donating reactions	H2:CoM-S-S-HTP oxidoreductase	CoB--CoM-reducing hydrogenase (Sec) gamma subunit	0.000348	0.002262	0	0	0.000657	0.000974	0.002166	0.000498	0	0	0	0.000394388
Cofactors, Vitamins, Prosthetic Groups, Pigments	Coenzyme B	Coenzyme B synthesis	Coenzyme B synthesis from 2-oxoglutarate: steps 4, 7, 8, 11, and 12 [large subunit]	0.001741	0.004524	0	0	0.002626	0.002435	0.002166	0.001495	0	0	0	0.002129693
Respiration	Electron accepting reactions	Anaerobic respiratory reductases	Coenzyme F420-dependent oxidoreductase	0	0	0	0.00747	0.002626	0.002435	0.000722	0.002492	0.000219	0	0	0.001380356
Respiration	Electron donating reactions	Hydrogenases	Coenzyme F420-reducing hydrogenase, gamma subunit	0	0.002262	0	0	0.000657	0.000487	0.002166	0.000498	0.002189	0	0	0.001735305
Cofactors, Vitamins, Prosthetic Groups, Pigments	Quinone cofactors	Coenzyme PQQ synthesis	Coenzyme PQQ synthesis protein C	0	0	0	0	0.000657	0.001461	0.007221	0.004984	0.004598	0	0.029225	0.009425862
Cofactors, Vitamins, Prosthetic Groups, Pigments	Quinone cofactors	Coenzyme PQQ synthesis	Coenzyme PQQ synthesis protein D	0	0	0	0	0.001313	0.000974	0.001444	0	0.002189	0.004406	0.00326	0.001419795
RNA Metabolism	RNA processing and modification	tRNA modification Archaea	COG2047: Uncharacterized protein (ATP-grasp superfamily)	0	0.004524	0	0	0.001313	0.002435	0.00361	0.001993	0.001314	0	0	0.000709898
Cell Wall and Capsule	Capsular and extracellular polysacchrides	Colanic acid biosynthesis	Colanic acid biosysnthesis glycosyl transferase WcaI	0	0	0.00511	0.00249	0	0.000974	0.00361	0.000498	0.000438	0	0	0.001183163
Membrane Transport	Protein and nucleoprotein secretion system, Type IV	Conjugative transfer	Conjugative transfer protein TrbI	0	0	0.002555	0	0.000657	0.000974	0	0.000997	0.001314	0.008811	0	0.000552143
Virulence, Disease and Defense	Resistance to antibiotics and toxic compounds	Copper homeostasis	Copper tolerance protein	0	0	0.007664	0	0	0.001461	0.000722	0.000498	0.001752	0	0.000349	0.001419795
Virulence, Disease and Defense	Resistance to antibiotics and toxic compounds	Cobalt-zinc-cadmium resistance	Copper-sensing two-component system response regulator CusR	0.003482	0	0	0	0	0.001461	0	0.000498	0.003065	0.004406	0.000582	0.002957907
Clustering-based subsystems	CRISPRs and associated hypotheticals	CBSS-216592.1.peg.3534	CRISPR-associated protein, CT1974	0	0	0.002555	0	0	0.001461	0.000722	0.001495	0.000657	0	0.000116	0.000512704
Clustering-based subsystems	CRISPRs and associated hypotheticals	CBSS-216592.1.peg.3534	CRISPR-associated protein, CT1976	0	0	0	0.00498	0.00197	0.000487	0.000722	0.002492	0.000438	0	0	0.00094653
Regulation and Cell signaling	NULL	DNA-binding regulatory proteins, strays	Cyn operon transcriptional activator	0.010097	0	0	0	0.001313	0.000974	0.000722	0.001495	0.001752	0	0	0.000552143
Respiration	Electron accepting reactions	Terminal cytochrome C oxidases	Cytochrome c oxidase (B(O/a)3-type) chain II (EC 1.9.3.1)	0	0	0	0	0.003283	0.003409	0.005777	0.003489	0.001971	0	0.000233	0.000552143
Fatty Acids, Lipids, and Isoprenoids	NULL	Polyhydroxybutyrate metabolism	D(-)-3-hydroxybutyrate oligomer hydrolase (EC 3.1.1.22)	0	0	0.002555	0	0.002626	0.002922	0.000722	0.001495	0.004817	0	0	0.000473265
Miscellaneous	Plant-Prokaryote DOE project	Experimental-COG2515	D-cysteine desulfhydrase (EC 4.4.1.15)	0.000348	0.002262	0	0	0.001313	0.000487	0.005055	0	0.001533	0	0	0.001419795
Amino Acids and Derivatives	NULL	Creatine and Creatinine Degradation	Dimethylglycine dehydrogenase (EC 1.5.99.2)	0	0	0.002555	0	0.00197	0.000974	0.00361	0.001993	0.009634	0	0	0.001695866
Stress Response	Osmotic stress	Betaine biosynthesis from glycine	Dimethylglycine N-methyltransferase	0.003133	0	0	0	0.011818	0.010228	0.01733	0.011961	0.006349	0	0	0.002287448
Respiration	Electron accepting reactions	Anaerobic respiratory reductases	Dissimilatory sulfite reductase (desulfoviridin), alpha and beta subunits	0.000348	0	0.002555	0	0.007222	0.007793	0	0.000498	0.000438	0	0	0.001853621
Carbohydrates	Organic acids	Lactate utilization	D-Lactate dehydrogenase (EC 1.1.2.5)	0	0	0.002555	0	0.001313	0.000487	0	0.00299	0.001095	0	0.000466	0.000433826
Carbohydrates	Monosaccharides	Mannose Metabolism	D-mannose isomerase (EC 5.3.1.7)	0	0	0	0.00498	0	0.000487	0.002166	0.001993	0.002627	0	0.003842	0.000157755
Clustering-based subsystems	Shiga toxin cluster	CBSS-194948.1.peg.143	DNA packaging	0	0	0	0	0.005909	0.011689	0.022385	0.025416	0.001533	0	0.000349	0.003431172
Phages, Prophages, Transposable elements, Plasmids	Phages, Prophages	r1t-like streptococcal phages	DNA replication protein DnaC	0.000696	0	0.002555	0	0.000657	0.001461	0	0.000498	0.000219	0	0	0.001380356
RNA Metabolism	Transcription	RNA polymerase bacterial	DNA-directed RNA polymerase alpha subunit domain	0	0.004524	0	0	0	0.003896	0.004333	0.005482	0.003503	0.004406	0	0.001656428
RNA Metabolism	Transcription	RNA polymerase bacterial	DNA-directed RNA polymerase delta (= beta'') subunit (EC 2.7.7.6)	0	0	0.00511	0	0.000657	0.007306	0	0.003987	0.007006	0	0.000349	0.001025408
RNA Metabolism	Transcription	RNA polymerase archaeal	DNA-directed RNA polymerase subunit A (EC 2.7.7.6)	0	0	0.002555	0	0.009848	0.000487	0.01372	0.005482	0.002189	0	0	0.000552143
Clustering-based subsystems	NULL	EC699-706	Endonuclease VIII	0.000348	0	0	0.00249	0.003283	0.00487	0.002888	0.001495	0.002846	0	0	0
Regulation and Cell signaling	Proteolytic pathway	Regulatory Intramembrane Proteolysis Pathways	Endoplasmic reticulum chaperon grp78 (BiP)	0	0	0	0	0.000657	0.000487	0.001444	0.000997	0	0.013217	0.000699	0.000512704
Respiration	Reverse electron transport	Archaeal membrane bound hydrogenases	Energy-conserving hydrogenase (ferredoxin), subunit C	0.000696	0.002262	0	0.00249	0.000657	0.001461	0	0	0.000657	0	0	0.003667804
Carbohydrates	Sugar alcohols	Erythritol utilization	Erythritol phosphate dehydrogenase EryB	0	0	0	0.00249	0.000657	0.001461	0.002166	0	0.000219	0.002203	0	3.94388E-05

Carbohydrates	Sugar alcohols	Ethanolamine utilization	Ethanolamine ammonia-lyase light chain (EC 4.3.1.7)	0	0	0	0	0	0.000487	0.006499	0.000498	0.002846	0.002203	0.002911	0.001735305
Carbohydrates	Sugar alcohols	Ethanolamine utilization	Ethanolamine utilization polyhedral-body-like protein EutM	0.163634	0.002262	0.00511	0	0.000657	0	0.002888	0.000997	0	0	0	0.001971938
Carbohydrates	Sugar alcohols	Ethanolamine utilization	Ethanolamine utilization protein EutJ	0.001393	0	0.00511	0	0.000657	0.001461	0.001444	0	0.000876	0	0	0.000828214
Metabolism of Aromatic Compounds	Anaerobic degradation of aromatic compounds	Anaerobic toluene and ethylbenzene degradation	Ethylbenzene dehydrogenase alpha subunit (EC 1.17.99.2)	0.002437	0	0.002555	0.00249	0.00197	0.000487	0.000722	0	0	0	0	0.001774744
Carbohydrates	Di- and oligosaccharides	Lactose utilization	Evolved beta-D-galactosidase, alpha subunit	0.000348	0	0	0	0.000657	0.000487	0.001444	0	0.000438	0	0.000349	0.001774744
Cell Wall and Capsule	Capsular and extracellular polysacchrides	Exopolysaccharide Biosynthesis	Exopolysaccharide biosynthesis glycosyltransferase EpsF (EC 2.4.1.-)	0	0	0.012774	0	0.003939	0.001461	0.004333	0.000997	0.000657	0	0	0.001498673
Miscellaneous	Plant-Prokaryote DOE project	At1g24340	FAD-binding monooxygenase, PheA/TfdB family, similarity to 2,4-dichlorophenol 6-monooxygenase	0	0	0.00511	0	0.000657	0.000974	0	0.000997	0.001971	0.002203	0	0.000394388
Cell Wall and Capsule	Gram-Negative cell wall components	Lipid A modifications	Fe(2+)/alpha-ketoglutarate-dependent dioxygenase LpxO	0.000696	0	0.00511	0	0	0.000974	0	0	0.003503	0.011014	0.000815	7.88775E-05
Respiration	NULL	Cytochrome B6-F complex	Ferredoxin-NADP(+) reductase (EC 1.18.1.2)	0	0	0	0.00249	0.001313	0.000487	0.000722	0.000997	0.001752	0	0	0.000118316
Virulence, Disease and Defense	Adhesion	Streptococcus pyogenes recombinatorial zone	Fibronectin-binding protein	0	0.002262	0	0	0.000657	0.002435	0.000722	0.000997	0.000438	0	0	0.00031551
Miscellaneous	Plant-Prokaryote DOE project	COG3380 COG2907	FIG002577: Putative lipoprotein precursor	0	0	0	0	0.002626	0.006332	0.011553	0.009469	0.009196	0	0.000466	0.003864998
Clustering-based subsystems	NULL	CBSS-291331.3.peg.3674	FIG002994: Putative transcriptional regulator	0	0	0.00511	0	0	0.000487	0.002166	0.000498	0.004598	0	0.000233	0.000591581
Clustering-based subsystems	NULL	CBSS-323097.3.peg.2594	FIG003307: hypothetical protein	0	0.002262	0.002555	0	0.003283	0.000974	0.002888	0.000997	0	0	0	0.001419795
Fatty Acids, Lipids, and Isoprenoids	Fatty acids	Phospholipid and Fatty acid biosynthesis related cluster	FIG018329: 1-acyl-sn-glycerol-3-phosphate acyltransferase	0.002437	0.002262	0	0.00498	0	0	0	0	0.000657	0.002203	0.000233	0.000788775
Respiration	ATP synthases	F0F1-type ATP synthase	FIG048548: ATP synthase protein I2	0	0.002262	0	0	0.001313	0.002435	0.002166	0.000498	0.000657	0	0	0.00094653
Clustering-based subsystems	NULL	CBSS-214092.1.peg.3450	FIG139552: Putative protease	0.054313	0	0	0.00249	0	0.000487	0	0.000498	0.001971	0	0.002329	0.002090254
Clustering-based subsystems	NULL	Cell wall related cluster	FIG140336: TPR domain protein	0.007659	0.002262	0.002555	0	0.000657	0	0	0.000997	0.000657	0	0	0.000828214
Motility and Chemotaxis	Flagellar motility in Prokaryota	Flagellum	Flagellar assembly protein FliH	0	0	0	0.00249	0.000657	0.001948	0.000722	0.00299	0.005693	0	0	0.004614334
Motility and Chemotaxis	Flagellar motility in Prokaryota	Flagellum	Flagellar basal-body P-ring formation protein FlgA	0	0	0.002555	0	0.000657	0.000974	0	0.001495	0.004817	0	0.000233	0.000828214
Motility and Chemotaxis	Flagellar motility in Prokaryota	Flagellum	Flagellar protein FlbD	0	0	0	0.00249	0.000657	0.001948	0.000722	0.000997	0.000438	0	0	0.000788775
Motility and Chemotaxis	Flagellar motility in Prokaryota	Archaeal Flagellum	Flagellin FlaB	0	0.004524	0	0.00498	0.005252	0.001948	0.002888	0.001495	0	0	0	0.001104285
Iron acquisition and metabolism	NULL	Heme, hemin uptake and utilization systems in GramNegatives	Flavin reductase (EC 1.5.1.30)	0	0	0	0	0.00197	0.002922	0.001444	0.004485	0.000657	0.002203	0	0.000788775
Stress Response	NULL	Bacterial hemoglobins	Flavohemoprotein (Hemoglobin-like protein) (Flavohemoglobin) (Nitric oxide dioxygenase) (EC 1.14.12.17)	0	0	0	0	0	0.001461	0.000722	0.001495	0.000438	0.002203	0.000349	0.001459234
Respiration	NULL	Formate hydrogenase	Formate hydrogenlyase subunit 7	0	0	0.010219	0.00498	0.003283	0.001461	0	0	0.003722	0.008811	0	0.00252408
Respiration	NULL	Biogenesis of cytochrome c oxidases	Frataxin homolog CyaY, facilitates iron supply for heme A synthesis or Fe-S cluster assembly	0.002089	0	0	0.00249	0	0.000974	0.001444	0.000498	0.000438	0	0	0.001538111
Carbohydrates	Monosaccharides	Fructose utilization	Fructose ABC transporter, permease component FrcC	0	0	0	0	0.001313	0.003409	0	0.001495	0.004817	0.004406	0.041683	0.001064846
Carbohydrates	Di- and oligosaccharides	Lactose and Galactose Uptake and Utilization	Galactose operon repressor, GalR-LacI family of transcriptional regulators	0	0.002262	0	0	0	0.000487	0.000722	0.000498	0.001314	0	0.000233	0.000670459
Virulence, Disease and Defense	NULL	Streptococcus agalactiae virulome	Galactosyl transferase CpsE (EC 2.7.8.-)	0	0.004524	0	0	0.001313	0.000487	0.002166	0.000498	0.000657	0	0	0.001853621
Carbohydrates	Aminosugars	N-Acetyl-Galactosamine and Galactosamine Utilization	GALNS arylsulfatase regulator (Fe-S oxidoreductase)	0	0.006786	0	0.00249	0	0.001948	0.000722	0.004984	0.000657	0	0	0.002800152
Phages, Prophages, Transposable elements, Plasmids	Gene Transfer Agent (GTA)	Gene Transfer Agent	Gene Transfer Agent prohead protease	0.001393	0	0	0	0.007879	0.005844	0.000722	0.004984	0.001752	0	0	0.000473265
Phages, Prophages, Transposable elements, Plasmids	Gene Transfer Agent (GTA)	Gene Transfer Agent	Gene Transfer Agent tail protein	0	0	0.00511	0	0.000657	0.000974	0	0.005482	0.001752	0	0.000582	0.000433826
Phages, Prophages, Transposable elements, Plasmids	Gene Transfer Agent (GTA)	Gene Transfer Agent	Gene Transfer Agent tail tape measure	0	0	0	0	0.003283	0.00487	0.004333	0.013456	0.002846	0	0.000699	7.88775E-05
Membrane Transport	Protein secretion system, Type II	General Secretion Pathway	General secretion pathway protein L	0	0	0.002555	0	0	0.000974	0.000722	0.001993	0.007663	0	0.000116	0.000828214
Carbohydrates	NULL	beta-glucuronide utilization	Glucuronide transporter UidB	0	0	0.00511	0	0	0.000974	0.001444	0.001495	0	0.004406	0.000466	0.001025408
Nitrogen Metabolism	NULL	Ammonia assimilation	Glutamate synthase, alpha subunit domain protein	0	0.004524	0.010219	0.00249	0.003939	0.000974	0	0	0	0	0.000116	0.000907091
Amino Acids and Derivatives	Glutamine, glutamate, aspartate, asparagine; ammonia assimilation	Glutamine, Glutamate, Aspartate and Asparagine Biosynthesis	Glutamine amidotransferase class-I (EC 6.3.5.2)	0.002089	0	0.00511	0	0.00197	0.002922	0.000722	0	0.000438	0	0	0.003115662
Miscellaneous	Plant-Prokaryote DOE project	Omega-amidase	Glutamine-dependent 2-keto-4-methylthiobutyrate transaminase	0	0.002262	0	0	0	0.000487	0	0.000498	0.001314	0.002203	0.004774	0.001695866
Stress Response	Oxidative stress	Glutaredoxins	Glutaredoxin-related protein	0	0	0.002555	0.00249	0	0.001948	0.000722	0.000997	0.001095	0	0	0.000788775
Stress Response	Osmotic stress	Choline and Betaine Uptake and Betaine Biosynthesis	Glycine betaine ABC transport system, glycine betaine-binding protein OpuAC	0	0	0	0	0.002626	0.001461	0.002888	0.007974	0.003503	0	0.000116	0.000788775
Protein Metabolism	Selenoproteins	Glycine reductase, sarcosine reductase and betaine reductase	Glycine/sarcosine/betaine reductase component C chain 1	0	0.002262	0.00511	0.00249	0.005909	0.003896	0	0	0.001533	0	0	0.002760713
Clustering-based subsystems	NULL	Cell wall related cluster	Glycine-rich cell wall structural protein precursor	0.004178	0.002262	0	0	0.00197	0.001461	0	0.002492	0.001533	0	0	0.001064846
Carbohydrates	Central carbohydrate metabolism	Glycolate, glyoxylate interconversions	Glycolate oxidase (EC 1.1.3.15)	0	0	0	0	0.000657	0.002435	0.002166	0.002492	0.003284	0.002203	0	0.000709898
Cofactors, Vitamins, Prosthetic Groups, Pigments	Folate and pterines	methanopterin biosynthesis	GTP cyclohydrolase III (methanopterin)	0	0	0.002555	0	0.00197	0.000487	0.011553	0.003987	0.002627	0	0	0.001025408
Stress Response	NULL	Hfl operon	GTP-binding protein related to HflX	0	0	0.002555	0	0.000657	0	0.001444	0.000498	0.000657	0	0.000116	0.000354949
Membrane Transport	NULL	Ton and Tol transport systems	Haemin uptake system outer membrane receptor	0	0.002262	0	0	0.003939	0.003409	0.000722	0.001495	0.000657	0	0	0.008045506
Cofactors, Vitamins, Prosthetic Groups, Pigments	Tetrapyrroles	Heme biosynthesis orphans	Heme biosynthesis protein related to NirD	0	0.002262	0	0	0.000657	0.001461	0	0.000498	0.000657	0	0.000116	0.001183163
Cofactors, Vitamins, Prosthetic Groups, Pigments	Tetrapyrroles	Heme biosynthesis orphans	Heme biosynthesis protein related to NirL and NirH	0	0.002262	0.00511	0	0.002626	0.004383	0.00361	0.001993	0	0	0	0.00220857
Cofactors, Vitamins, Prosthetic Groups, Pigments	Tetrapyrroles	Heme biosynthesis orphans	Heme d1 biosynthesis protein NirH	0.005919	0	0	0.00498	0	0.000974	0.007221	0.001495	0.002846	0	0	0.002563519
Carbohydrates	Monosaccharides	D-Galacturonate and D-Glucuronate Utilization	Hexuronate utilization operon transcriptional repressor ExuR	0	0	0	0	0.000657	0.000487	0.000722	0.000498	0.000657	0	0.000349	0.003864998
Miscellaneous	Plant-Prokaryote DOE project	At5g48545 and At3g56490 At1g31160	Histidine triad (HIT) nucleotide-binding protein, cyanobacterial subgroup	0	0	0	0.00249	0.008535	0.001948	0.00361	0.000997	0.002408	0	0	0.001616989
Phages, Prophages, Transposable elements, Plasmids	Phages, Prophages	Phage introns	HNH homing endonuclease	0	0.004524	0	0	0.005909	0.012663	0.010109	0.014951	0.001314	0	0	0.000276071
Metabolism of Aromatic Compounds	Metabolism of central aromatic intermediates	4-Hydroxyphenylacetic acid catabolic pathway	Homoprotocatechuate degradative operon repressor	0	0	0	0.00249	0.002626	0.000487	0	0.001993	0.001971	0	0.008267	0.00094653
Cofactors, Vitamins, Prosthetic Groups, Pigments	Tetrapyrroles	Coenzyme B12 biosynthesis	HupE-UreJ family cobalt transporter	0	0.004524	0	0	0	0.000974	0.00361	0.000997	0.000219	0	0.000116	0.000118316
Cell Wall and Capsule	Capsular and extracellular polysacchrides	Streptococcal Hyaluronic Acid Capsule	Hyaluronan synthase (EC 2.4.1.212)	0.000696	0.004524	0	0.00747	0	0	0	0.001495	0.000219	0	0.002096	0.001143724
Regulation and Cell signaling	NULL	Orphan regulatory proteins	Hybrid sensory histidine kinase in two-component regulatory system with EvgA	0	0.002262	0	0.00249	0.000657	0	0.000722	0.000498	0.000438	0	0	0.00063102

Miscellaneous	Plant-Prokaryote DOE project	Iojap	Hydrolase (HAD superfamily), YqeK	0	0.004524	0	0	0.000657	0.000487	0.000722	0.000498	0.000438	0	0	0.000157755
Carbohydrates	Sugar alcohols	Mannitol Utilization	Hydrolase in polyol utilization gene cluster, haloacid dehalogenase-like family	0	0.004524	0.002555	0	0.000657	0.000487	0	0.001495	0.002408	0	0	0.000473265
Miscellaneous	Plant-Prokaryote DOE project	At3g50560	Hypothetical protein in cluster with At3g50560-like gene family	0	0	0	0	0.000657	0.002922	0.002888	0.000997	0.003065	0.006608	0	0.000197194
Clustering-based subsystems	Probably GTP or GMP signaling related	CBSS-176299.4.peg.1292	hypothetical protein NAS141 09886	0	0	0	0	0	0.000974	0.000722	0.003987	0.001752	0.004406	0.000233	0.000236633
Carbohydrates	NULL	Sugar utilization in Thermotogales	Hypothetical protein TM0950	0.000348	0	0	0.00249	0.001313	0.004383	0	0.002492	0.002189	0	0	0.001380356
Miscellaneous	Plant-Prokaryote DOE project	At1g69340 At2g40600	Hypothetical protein YmdC, PLD phosphodiesterase domains	0.000696	0	0.002555	0.00249	0	0.002922	0.001444	0	0.000657	0	0	0.001143724
Carbohydrates	Aminosugars	N-Acetyl-Galactosamine and Galactosamine Utilization	Hypothetical secreted sugar oxidoreductase, Gfo/Idh/MocA family, GalNAc-related	0	0.009048	0.002555	0	0	0.000974	0	0.000997	0.001095	0	0.000349	0.001222601
Regulation and Cell signaling	NULL	Sex pheromones in Enterococcus faecalis and other Firmicutes	Hypothetical similar to thiamin biosynthesis lipoprotein ApbE	0	0.004524	0	0	0.001313	0.001461	0.000722	0.001495	0.000657	0	0	0.001814183
Nucleosides and Nucleotides	Purines	Xanthine Metabolism in Bacteria	Hypoxanthine/guanine permease PbuG	0	0	0.002555	0.00249	0.000657	0.000487	0.001444	0	0	0	0.000116	0.000276071
Membrane Transport	Protein and nucleoprotein secretion system, Type IV	Dot-Icm type IV secretion system	IcmO (DotL) protein	0	0	0.002555	0	0.000657	0.001948	0.00361	0.003987	0.001533	0	0	0.000197194
Clustering-based subsystems	NULL	Conjugative transfer related cluster	IncF plasmid conjugative transfer pilus assembly protein TraH	0	0.002262	0.002555	0	0.000657	0.000974	0.001444	0	0	0	0.000466	0.000236633
Clustering-based subsystems	NULL	Conjugative transfer related cluster	IncF plasmid conjugative transfer protein TraD	0.001044	0	0.012774	0.00747	0	0.000974	0.001444	0	0.000876	0	0	0.00094653
Secondary Metabolism	Plant Hormones	Auxin biosynthesis	Indole-3-pyruvate decarboxylase (EC 4.1.1.74)	0	0.002262	0	0	0.001313	0.000487	0	0.000498	0.000657	0.006608	0	0.000236633
Membrane Transport	Protein and nucleoprotein secretion system, Type IV	Vir-like type 4 secretion system	Inner membrane protein of type IV secretion of T-DNA complex, TonB-like, VirB10	0	0	0	0.00498	0	0.000974	0.001444	0.000498	0.000657	0	0.000466	0.000512704
Carbohydrates	Sugar alcohols	Inositol catabolism	Inositol oxygenase (EC 1.13.99.1)	0.002437	0.004524	0.002555	0	0.000657	0	0.000722	0	0.001533	0	0	0.000394388
Iron acquisition and metabolism	Siderophores	Iron siderophore sensor & receptor system	Iron siderophore sensor protein	0.004874	0	0	0	0	0.000487	0.000722	0.000498	0.000438	0	0.000466	0.001459234
Iron acquisition and metabolism	NULL	Iron(III) dicitrate transport system Fec	Iron(III) dicitrate transport protein FecA	0	0	0	0	0.000657	0.000487	0.002166	0.013456	0.013137	0	0.000466	0.001340918
Iron acquisition and metabolism	NULL	Transport of Iron	Iron-responsive repressor RirA	0	0	0	0	0.000657	0.000487	0.000722	0.001495	0.000657	0.002203	0	7.88775E-05
Protein Metabolism	Protein degradation	Protein degradation	Isoaspartyl dipeptidase (EC 3.4.19.5)	0	0	0.002555	0	0	0.000974	0.000722	0.002492	0.000219	0	0.000116	0.000354949
Stress Response	Osmotic stress	Ectoine biosynthesis and regulation	L-2,4-diaminobutyric acid acetyltransferase (EC 2.3.1.-)	0	0	0	0	0.005909	0.001461	0.007943	0.011462	0.007882	0	0.002212	0.002760713
Carbohydrates	Monosaccharides	L-rhamnose utilization	Lactaldehyde reductase (EC 1.1.1.77)	0.003482	0	0	0.00249	0.001313	0.000974	0	0.001495	0.000438	0	0	0.002681835
Carbohydrates	Organic acids	Lactate utilization	Lactate-responsive regulator LldR in Enterobacteria, GntR family	0	0	0	0	0.001313	0.002435	0.001444	0.000997	0.000657	0	0.002911	0.002602958
Carbohydrates	NULL	Sugar utilization in Thermotogales	Laminarinase (EC 3.2.1.39)	0.000348	0	0.002555	0	0	0.000974	0.000722	0.00299	0.001971	0	0	0.002366325
Carbohydrates	Monosaccharides	L-Arabinose utilization	L-arabinose 1-dehydrogenase (EC 1.1.1.46)	0	0	0	0	0.002626	0.000974	0.002166	0.001993	0.003722	0	0.000349	0.001104285
DNA Metabolism	DNA uptake, competence	Gram Positive Competence	Late competence protein ComGA, access of DNA to ComEA	0	0	0.002555	0	0.001313	0.000974	0.001444	0.001993	0.001752	0	0	0.00157755
Amino Acids and Derivatives	Lysine, threonine, methionine, and cysteine	Lysine degradation	L-beta-lysine 5,6-aminomutase beta subunit (EC 5.4.3.3)	0	0.002262	0	0	0.001313	0.001461	0.004333	0.001495	0	0	0.001281	0.001695866
Carbohydrates	Monosaccharides	L-fucose utilization	L-fuculose phosphate aldolase (EC 4.1.2.17)	0.004178	0	0.002555	0	0.000657	0.000974	0	0	0.000876	0	0.000116	0.000552143
Virulence, Disease and Defense	Invasion and intracellular resistance	Cytolysin and Lipase operon in Vibrio	Lipase precursor (EC 3.1.1.3)	0	0	0.002555	0	0	0.000974	0	0.001495	0.007225	0.002203	0.002911	0.001301479
Cell Wall and Capsule	Gram-Negative cell wall components	LOS core oligosaccharide biosynthesis	Lipopolysaccharide core biosynthesis protein WaaP (EC 2.7.-.-), heptosyl-I-kinase	0	0.006786	0.002555	0	0.003939	0.002435	0.002888	0.002492	0	0	0	0.001025408
Clustering-based subsystems	Clustering-based subsystems	CBSS-280355.3.peg.2835	Lmbe-related protein	0	0.002262	0	0	0.000657	0.001461	0.000722	0.000498	0.000438	0	0	0.000670459
Protein Metabolism	Protein biosynthesis	Ribosome LSU eukaryotic and archaeal	LSU ribosomal protein L10e (L16p)	0	0	0	0	0.001313	0.002435	0.005055	0.002492	0.001752	0	0.000233	0.000867653
Protein Metabolism	Protein biosynthesis	Ribosome LSU eukaryotic and archaeal	LSU ribosomal protein L8e (L2p)	0	0.004524	0	0	0.003283	0.002922	0.002166	0.001993	0.002189	0	0	0.001419795
Protein Metabolism	Protein biosynthesis	Ribosome LSU eukaryotic and archaeal	LSU ribosomal protein L9e (L6p)	0.003133	0	0	0	0.00197	0.001948	0.00361	0.00299	0.002846	0	0	0.001340918
Protein Metabolism	Protein biosynthesis	Ribosome LSU eukaryotic and archaeal	LSU ribosomal protein P0 (L10p)	0	0	0	0	0.002626	0.000974	0.001444	0.000997	0.000219	0.004406	0	0.000670459
Amino Acids and Derivatives	Arginine; urea cycle, polyamines	Arginine and Ornithine Degradation	Lysine-arginine-ornithine-binding periplasmic protein precursor (TC 3.A.1.3.1)	0	0.002262	0	0	0.000657	0.002435	0.000722	0	0.006568	0	0.009548	0.001104285
Regulation and Cell signaling	NULL	DNA-binding regulatory proteins, strays	LysR family transcriptional regulator YnfL	0.007311	0.002262	0	0.00249	0.001313	0	0	0.000498	0.000219	0	0	7.88775E-05
Carbohydrates	Central carbohydrate metabolism	Pyruvate metabolism I: anaplerotic reactions, PEP	Malolactic enzyme (EC 1.-.-.-)	0.002089	0	0	0.00249	0.001313	0.000487	0	0.001495	0.000219	0	0	0.001380356
Carbohydrates	Organic acids	Malonate decarboxylase	Malonate transporter, MadM subunit	0.002437	0.002262	0	0	0	0	0.002166	0.000997	0.000657	0	0.000233	0.001025408
Carbohydrates	Monosaccharides	Mannose Metabolism	Mannoside ABC transport system, permease protein 1	0.002437	0.002262	0	0	0.000657	0	0	0.000997	0	0.004406	0.000233	0.000433826
Respiration	Electron accepting reactions	Terminal cytochrome C oxidases	membrane c-type cytochrome cy	0	0	0	0.00249	0.002626	0.001948	0.006499	0.002492	0.005474	0	0	0.002090254
Virulence, Disease and Defense	Resistance to antibiotics and toxic compounds	Copper homeostasis: copper tolerance	Membrane protein, suppressor for copper-sensitivity ScsB	0	0	0	0	0.001313	0.000974	0.00361	0.002492	0.005036	0	0.000116	0.002800152
Cell Wall and Capsule	NULL	Murein Hydrolases	Membrane-bound lytic murein transglycosylase C precursor (EC 3.2.1.-)	0	0	0	0	0.003283	0.001948	0.002888	0.003987	0.001752	0	0.000116	0.010609025
Cell Wall and Capsule	NULL	Murein Hydrolases	Membrane-bound lytic murein transglycosylase E (EC 3.2.1.-)	0.000696	0	0.002555	0.00249	0.001313	0.001948	0.002888	0	0	0	0	0.001222601
Virulence, Disease and Defense	Resistance to antibiotics and toxic compounds	Mercury resistance operon	Mercuric resistance operon regulatory protein	0	0	0.002555	0	0.000657	0.000487	0	0.000498	0.003722	0	0.000466	0.001104285
Amino Acids and Derivatives	Lysine, threonine, methionine, and cysteine	Methionine Biosynthesis	Methionine transporter MetT	0	0	0	0	0.00197	0.000974	0	0.000997	0.010509	0.002203	0.054491	0.005560864
Protein Metabolism	Protein biosynthesis	tRNA aminoacylation, Met	Methionyl-tRNA synthetase-related protein	0	0	0.002555	0	0.001313	0.000974	0.002888	0.001495	0.000438	0	0	0.00031551
Fatty Acids, Lipids, and Isoprenoids	Isoprenoids	Carotenoids	Methoxyneurosporene dehydrogenase (EC 1.14.99.-)	0	0	0.002555	0	0.00197	0.010715	0.002888	0.00598	0.006131	0	0	0.000670459
Motility and Chemotaxis	NULL	Bacterial Chemotaxis	Methyl-accepting chemotaxis protein IV (dipeptide chemoreceptor protein)	0	0	0	0.00249	0.000657	0.000487	0	0	0.000657	0.006608	0.000233	0.000236633
Cofactors, Vitamins, Prosthetic Groups, Pigments	Tetrapyrroles	Chlorophyll Biosynthesis	Mg protoporphyrin IX monomethyl ester oxidative cyclase (aerobic) (EC 1.14.13.81)	0	0	0	0	0.005252	0.020943	0.015886	0.007475	0.018173	0	0.000931	0.000828214
Metabolism of Aromatic Compounds	Metabolism of central aromatic intermediates	Catechol branch of beta-ketoadipate pathway	Muconolactone isomerase (EC 5.3.3.4),putative	0	0	0.002555	0	0.001313	0.003409	0.004333	0.000498	0.002846	0	0	0.001064846
Carbohydrates	Aminosugars	Chitin and N-acetylglucosamine utilization	N-acetyl glucosamine transporter, NagP	0	0.004524	0	0	0.00197	0.001948	0	0.000498	0.001095	0	0.001863	0.026581721
Carbohydrates	Aminosugars	Chitin and N-acetylglucosamine utilization	N-acetylglucosamine regulated methyl-accepting chemotaxis protein	0	0	0	0	0.000657	0.000487	0	0.001993	0.00832	0.008811	0.001747	0.008637087
Carbohydrates	NULL	Lacto-N-Biose I and Galacto-N-Biose Metabolic Pathway	N-acetylhexosamine 1-kinase	0.001741	0	0	0.00747	0	0.001948	0	0.000498	0.000657	0	0.000116	0.001656428
Respiration	Electron donating reactions	Respiratory Complex I	NAD(P)H-quinone oxidoreductase chain I (EC 1.6.5.2)	0	0	0.00511	0	0	0.000487	0.002888	0.000997	0.000219	0	0.000349	0.000276071
Respiration	Electron donating reactions	Respiratory Complex I	NAD(P)H-quinone oxidoreductase subunit 3 (EC 1.6.5.2)	0	0.004524	0.002555	0	0.001313	0.000974	0	0	0.002189	0	0.000349	0.001104285
Respiration	Electron donating reactions	Respiratory Complex I	NADH:ubiquinone oxidoreductase 17.2 kD subunit	0	0	0	0	0.001313	0.002435	0.008665	0.007475	0.001752	0.002203	0	0.001301479

Respiration	Electron donating reactions	Hydrogenases	NADP-reducing hydrogenase, subunit B	0	0.006786	0	0.00249	0.00197	0.003896	0.004333	0.003489	0	0	0	0.003825559
Amino Acids and Derivatives	NULL	Creatine and Creatinine Degradation	N-carbamoylsarcosine amidase (EC 3.5.1.59)	0	0	0.002555	0	0	0.001461	0.002888	0.005482	0.002408	0.004406	0	0.000512704
Virulence, Disease and Defense	Resistance to antibiotics and toxic compounds	Beta-lactamase	Negative regulator of beta-lactamase expression	0.003482	0	0	0	0	0.002435	0.002166	0.005482	0.001752	0	0.002678	0.000788775
Sulfur Metabolism	NULL	Galactosylceramide and Sulfatide metabolism	Neuraminidase NanP	0	0.002262	0.002555	0	0.00197	0.000974	0.000722	0	0.000219	0	0	0.00094653
Respiration	Electron donating reactions	Hydrogenases	Ni,Fe-hydrogenase maturation factor	0	0.002262	0.002555	0.00747	0	0.000487	0	0	0.000438	0	0.012808	0.00063102
Cofactors, Vitamins, Prosthetic Groups, Pigments	NAD and NADP	NAD and NADP cofactor biosynthesis global	Niacin transporter NiaP	0.004526	0	0.002555	0	0	0.001461	0.001444	0.000498	0.000876	0	0	0.000276071
Nitrogen Metabolism	NULL	Nitrosative stress	Nitric oxide-dependent regulator DnrN or NorA	0.005222	0	0	0	0	0.000974	0.007221	0.001993	0.006349	0	0.000931	0.006546833
Nitrogen Metabolism	NULL	Nitrogen fixation	Nitrogenase cofactor carrier protein NafY	0.000696	0.002262	0	0	0.001313	0.001461	0.001444	0.000997	0	0	0	0.001301479
Nitrogen Metabolism	NULL	Nitrogen fixation	Nitrogenase stabilizing/protective protein NifW	0	0.002262	0	0	0.000657	0.001461	0.000722	0.000997	0.001971	0	0	0.002129693
Nitrogen Metabolism	NULL	Nitrogen fixation	Nitrogenase-associated protein NifO	0.002089	0	0	0	0.001313	0.000974	0	0.000997	0.000438	0.002203	0	0.002405764
Nitrogen Metabolism	NULL	Nitrate and nitrite ammonification	NrfD protein	0.002785	0	0	0.00498	0.002626	0.005844	0.001444	0.000498	0	0	0	0.011200606
RNA Metabolism	RNA processing and modification	tRNAmodification position 34	n-type ATP pyrophosphatase superfamily / TilS and TtcA-like	0	0	0.002555	0	0.001313	0.000974	0.004333	0.001495	0.000657	0	0	0.000197194
Cell Wall and Capsule	Capsular and extracellular polysacchrides	Capsular Polysaccharides Biosynthesis and Assembly	O-antigen flippase Wzx	0	0.002262	0	0.00249	0	0	0.000722	0.000997	0.000438	0	0.000233	0.00252408
Cell Wall and Capsule	Gram-Negative cell wall components	LOS core oligosaccharide biosynthesis	O-antigen ligase	0	0	0	0	0.000657	0.001461	0.001444	0.001993	0.001533	0	0.000116	0.001222601
Miscellaneous	Plant-Prokaryote DOE project	COG1836	Octaprenyl-diphosphate synthase (EC 2.5.1.-) / Dimethylallyltransferase (EC 2.5.1.1) / Geranyltranstransferase (EC 2.5.1.10) / Geranylgeranyl pyrophosphate synthetase (EC 2.5.1.29)	0.000696	0	0	0	0.00197	0.000974	0	0.001993	0.000657	0	0.000466	0.002050815
Carbohydrates	Monosaccharides	D-Galacturonate and D-Glucuronate Utilization	Oligogalacturonate lyase (EC 4.2.2.6)	0	0.002262	0	0.00249	0	0.001948	0.00361	0.001495	0.000876	0	0	0.00189306
Protein Metabolism	Protein processing and modification	N-linked Glycosylation in Bacteria	Oligosaccharyltransferase PglB (EC 2.4.1.119)	0	0	0.002555	0.00249	0	0.000974	0.001444	0.000498	0	0	0.000349	0.004259386
Stress Response	Osmotic stress	Synthesis of osmoregulated periplasmic glucans	OpgC protein	0	0	0	0.00249	0.000657	0	0.004333	0.002492	0.002408	0	0.000466	0.000670459
Stress Response	Oxidative stress	Oxidative stress	Organic hydroperoxide resistance protein	0	0	0	0	0.001313	0	0.000722	0.000997	0.003722	0.002203	0.007917	0.00283959
Amino Acids and Derivatives	Arginine; urea cycle, polyamines	Anaerobic Oxidative Degradation of L-Ornithine	Ornithine racemase (EC 5.1.1.12)	0	0.006786	0.002555	0	0.00197	0	0.000722	0.000498	0.001314	0	0	0.002721274
Stress Response	Osmotic stress	Choline and Betaine Uptake and Betaine Biosynthesis	Osmotically activated L-carnitine/choline ABC transporter, ATP-binding protein OpuCA	0.000696	0	0	0.00249	0.001313	0	0	0.001993	0.001314	0	0.000466	0.000354949
Phages, Prophages, Transposable elements, Plasmids	Plasmid related functions	Plasmid-encoded T-DNA transfer	Outer membrane and periplasm component of type IV secretion of T-DNA complex, has secretin-like domain, VirB9	0.002785	0	0	0	0.000657	0.000487	0.000722	0.000997	0.000657	0	0	0.000433826
Miscellaneous	Plant-Prokaryote DOE project	At4g17370	Oxidoreductase, NAD(P)-dependent, weak similarity to Myo-inositol 2-dehydrogenase	0.000348	0	0	0	0.000657	0	0.001444	0.002492	0.003722	0	0.000116	0.000276071
Metabolism of Aromatic Compounds	Metabolism of central aromatic intermediates	Protocatechuate branch of beta-ketoadipate pathway	Pca regulon regulatory protein PcaR	0	0.002262	0	0	0.00197	0.000487	0.000722	0.000997	0.001971	0	0	0.000433826
Carbohydrates	Monosaccharides	D-Galacturonate and D-Glucuronate Utilization	Pectinesterase (EC 3.1.1.11)	0.002785	0	0.002555	0.00249	0.00197	0	0	0.000997	0.003941	0	0	0.001735305
Iron acquisition and metabolism	NULL	Transport of Iron	Periplasmic protein p19 involved in high-affinity Fe2+ transport	0	0	0.002555	0	0.000657	0.000487	0.000722	0	0.000438	0.004406	0	3.94388E-05
Phages, Prophages, Transposable elements, Plasmids	Phages, Prophages	Phage baseplate proteins	Phage baseplate	0	0	0	0	0	0.000487	0.004333	0.001993	0.001095	0.002203	0.000233	0.001932499
Phages, Prophages, Transposable elements, Plasmids	Phages, Prophages	Phage capsid proteins	Phage capsid protein	0	0	0	0	0.004596	0.012663	0.008665	0.012459	0.000657	0	0.000233	0.00063102
Phages, Prophages, Transposable elements, Plasmids	Phages, Prophages	Phage packaging machinery	Phage DNA packaging	0	0	0	0	0.00197	0.011202	0.004333	0.011462	0.000438	0	0.000466	0.000512704
Clustering-based subsystems	Shiga toxin cluster	CBSS-194948.1.peg.143	Phage endolysin	0	0	0	0.00249	0.00197	0.002922	0.002166	0.003987	0	0.002203	0	0.000867653
Phages, Prophages, Transposable elements, Plasmids	Phages, Prophages	Phage capsid proteins	Phage head maturation protease	0.002785	0	0	0	0.003939	0.011689	0.011553	0.003987	0.000438	0	0	0.000236633
Phages, Prophages, Transposable elements, Plasmids	Phages, Prophages	Phage entry and exit	Phage peptidoglycan binding endopeptidase	0.000696	0	0	0	0.001313	0.001948	0.002166	0	0.000438	0	0.000582	0.00031551
Stress Response	NULL	Phage shock protein (psp) operon	Phage shock protein C	0.002785	0	0	0	0.00197	0.001461	0	0.001993	0.002408	0	0.001979	0.000709898
Cell Wall and Capsule	Cell wall of Mycobacteria	mycolic acid synthesis	Phenolpthiocerol synthesis polyketide synthase ppsB	0	0.002262	0	0.00498	0	0	0.000722	0.000997	0.001095	0	0.000233	0.001301479
Metabolism of Aromatic Compounds	NULL	Phenylacetyl-CoA catabolic pathway (core)	Phenylacetic acid degradation protein PaaN2, ring-opening aldehyde dehydrogenase (EC 1.2.1.3)	0	0	0.002555	0	0.00197	0	0.002166	0.000997	0.005036	0	0.058566	0.005008722
Phosphorus Metabolism	NULL	Alkylphosphonate utilization	PhnB protein	0	0	0.017884	0	0	0	0.000722	0.004984	0.01445	0.006608	0.000699	0.005679181
Phosphorus Metabolism	NULL	Alkylphosphonate utilization	PhnG protein	0	0	0.002555	0	0.000657	0.000974	0.001444	0.007475	0.000876	0	0	0.000157755
Phosphorus Metabolism	NULL	Phosphate metabolism	Phosphate-specific outer membrane porin OprP	0	0	0.002555	0	0.001313	0.000487	0	0.000498	0.000876	0	0.002212	0.000591581
Fatty Acids, Lipids, and Isoprenoids	Phospholipids	Glycerolipid and Glycerophospholipid Metabolism in Bacteria	Phosphatidylglycerophosphatase B (EC 3.1.3.27)	0.000348	0	0	0	0	0.000974	0.000722	0.000997	0.002846	0.006608	0	0.000670459
Carbohydrates	Central carbohydrate metabolism	Dihydroxyacetone kinases	Phosphoenolpyruvate-dihydroxyacetone phosphotransferase (EC 2.7.1.121), ADP-binding subunit DhaL	0.002785	0	0	0	0.004596	0.000974	0.002166	0	0.002189	0	0.000466	0.001183163
Clustering-based subsystems	Clustering-based subsystems	CBSS-262719.3.peg.410	Phosphoesterase, DHH family protein	0	0	0	0	0.00197	0.001461	0.002888	0.000997	0.000876	0	0.003493	0.007138415
Carbohydrates	One-carbon Metabolism	Serine-glyoxylate cycle	Phosphoglyceromutase	0	0.004524	0	0	0	0.000487	0.000722	0.002492	0.000438	0	0.000116	0.000197194
Cell Wall and Capsule	Gram-Negative cell wall components	LOS core oligosaccharide biosynthesis	Phosphoheptose isomerase 2 (EC 5.3.1.-)	0.002437	0.002262	0	0.00249	0	0.000487	0	0.000498	0.000657	0	0	0.001025408
Clustering-based subsystems	NULL	CBSS-235.1.peg.567	Phosphohistidine phosphatase SixA	0	0	0	0.00498	0.003283	0.001948	0.000722	0.003987	0.006787	0	0	0.004456579
Miscellaneous	Plant-Prokaryote DOE project	At2g23840	Phospholipase/carboxylesterase family protein (EC 3.1.-.-)	0.010793	0	0	0.01245	0	0.000487	0	0.007475	0.00416	0.004406	0	0.00063102
Photosynthesis	Electron transport and photophosphorylation	Photosystem II-type photosynthetic reaction center	Photosynthetic reaction center cytochrome c subunit	0	0.004524	0	0	0.002626	0.006819	0.00361	0.00598	0.006787	0	0	0.000394388
Photosynthesis	Electron transport and photophosphorylation	Photosystem II-type photosynthetic reaction center	Photosynthetic reaction center L subunit	0	0	0	0	0.000657	0.010715	0.00361	0.002492	0.007006	0	0.000349	0.000670459
Photosynthesis	Electron transport and photophosphorylation	Photosystem I	photosystem I P700 chlorophyll a apoprotein subunit Ia (PsaA)	0	0	0	0	0.001313	0.000487	0	0.001495	0.007225	0.011014	0.001747	0.000591581
Photosynthesis	Electron transport and photophosphorylation	Photosystem II	Photosystem II CP47 protein (PsbB)	0	0	0	0.00249	0.00197	0	0	0.000498	0.002408	0.002203	0.003609	0.000157755
Photosynthesis	Electron transport and photophosphorylation	Photosystem II	photosystem II protein D2 (PsbD)	0	0	0	0	0.001313	0	0.001444	0.001495	0.003941	0.002203	0.004075	0.000670459
Amino Acids and Derivatives	Glutamine, glutamate, aspartate, asparagine; ammonia assimilation	Poly-gamma-glutamate biosynthesis	Poly-gamma-glutamate synthase subunit PgsB/CapB (EC 6.3.2.-)	0	0.004524	0	0	0.003283	0.002435	0.002166	0.004485	0.012261	0	0	0.001380356
Amino Acids and Derivatives	Glutamine, glutamate, aspartate, asparagine; ammonia assimilation	Poly-gamma-glutamate biosynthesis	Poly-gamma-glutamate synthase subunit PgsC/CapC (EC 6.3.2.-)	0	0.002262	0	0	0.00197	0.000974	0.000722	0.00299	0.006349	0	0	0.000157755
Carbohydrates	Monosaccharides	L-fucose utilization	Possible fucose ABC transporter, permease component	0	0	0	0	0	0.000487	0.000722	0.000498	0.000876	0.004406	0.000233	0.001183163

Carbohydrates	CO2 fixation	CO2 uptake, carboxysome	Possible pterin-4 alpha-carbinolamine dehydratase-like protein	0.004178	0	0	0.00498	0.000657	0.000487	0.001444	0.001495	0	0	0	0.001538111
Potassium metabolism	NULL	Potassium homeostasis	Potassium-transporting ATPase A chain (EC 3.6.3.12) (TC 3.A.3.7.1)	0	0.002262	0.00511	0	0	0	0.000722	0	0.015545	0.013217	0.001048	0.002405764
Potassium metabolism	NULL	Potassium homeostasis	Potassium-transporting ATPase B chain (EC 3.6.3.12) (TC 3.A.3.7.1)	0	0	0	0.02739	0	0.000974	0.001444	0	0.016202	0.048461	0.001164	0.002169132
Cofactors, Vitamins, Prosthetic Groups, Pigments	Tetrapyrroles	Coenzyme B12 biosynthesis	Predicted cobalt transporter CbtA	0.016015	0	0	0	0.000657	0	0.002166	0.001993	0.001314	0.002203	0	0.00031551
Carbohydrates	Sugar alcohols	Erythritol utilization	Predicted erythritol ABC transporter 1, permease component 2	0	0	0	0.00249	0.000657	0.000487	0.000722	0	0.000438	0	0.000116	0.000157755
Carbohydrates	Monosaccharides	D-gluconate and ketogluconates metabolism	Predicted gluconate TRAP family transporter, DctP subunit	0.000348	0	0	0	0.00197	0.001948	0.001444	0.001993	0.003722	0	0	7.88775E-05
Miscellaneous	Plant-Prokaryote DOE project	COG3533	Predicted L-arabinose ABC transport system, periplasmic arabinose-binding protein	0.000348	0	0	0	0.000657	0.000487	0.000722	0	0.001095	0	0.001979	0.001143724
Carbohydrates	Monosaccharides	L-rhamnose utilization	Predicted L-rhamnose ABC transporter, transmembrane component 2	0.002785	0.004524	0	0.01245	0.008535	0	0.00361	0	0.003503	0	0	0.001025408
Carbohydrates	Sugar alcohols	Mannitol Utilization	Predicted mannitol permease	0	0.002262	0.010219	0	0.002626	0.001461	0	0.002492	0.001533	0	0	0.00094653
Carbohydrates	Monosaccharides	Mannose Metabolism	Predicted mannose transporter, GGP family	0	0	0	0	0.000657	0.000487	0.000722	0.000997	0.002189	0	0.000349	0.000867653
Nucleosides and Nucleotides	Pyrimidines	Pyrimidine utilization	Predicted reductase RutE in novel pyrimidine catabolism pathway	0.000348	0.002262	0	0	0.001313	0.000974	0	0.004485	0.001533	0	0	0.001143724
RNA Metabolism	RNA processing and modification	tRNA modification Archaea	Predicted RNA-binding protein COG1491	0	0.006786	0	0.00249	0.000657	0	0.005777	0.000997	0.000219	0	0	0.000828214
Membrane Transport	Protein secretion system, Type II	Predicted secretion system X	Predicted secretion system X protein GspG-like	0	0	0.00511	0	0.001313	0.000487	0.000722	0	0.001095	0	0.000349	0.000433826
RNA Metabolism	Transcription	Rrf2 family transcriptional regulators	Predicted transcriptional regulator of cysteine synthase, Rrf2 family	0	0	0.002555	0	0.00197	0.002435	0.001444	0.00299	0.002189	0	0	0.000749336
Carbohydrates	Di- and oligosaccharides	Trehalose Uptake and Utilization	Predicted trehalose ABC transporter, permease component 1, Archaea-type	0.00383	0	0.00511	0	0.00197	0.000974	0	0.000997	0.000438	0	0	0.000907091
Carbohydrates	Di- and oligosaccharides	Trehalose Uptake and Utilization	Predicted trehalose ABC transporter, substrate-binding component, Archaea-type	0	0	0	0.00498	0.001313	0.000974	0.000722	0.000997	0.000438	0	0	0.000473265
Respiration	Electron accepting reactions	Terminal cytochrome C oxidases	probable cytochrome oxidase (cbb3-type)	0	0	0.002555	0	0.00197	0.001461	0.001444	0.002492	0.001752	0	0	0.000473265
Clustering-based subsystems	Proteasome related clusters	Proteasome subunit alpha archaeal cluster	Probable exosome complex RNA-binding protein 1	0	0.004524	0	0	0.002626	0.002435	0.002166	0.001495	0.000219	0	0	0.00094653
Stress Response	Acid stress	Acid resistance mechanisms	Probable glutamate/gamma-aminobutyrate antiporter	0.001044	0	0	0	0.000657	0.000974	0	0.000498	0.000219	0	0.000116	0.000552143
Stress Response	Oxidative stress	Glutaredoxins	Probable monothiol glutaredoxin GrlA	0.005571	0	0.002555	0.00249	0	0.000487	0.000722	0	0.001095	0	0	0.001064846
Amino Acids and Derivatives	Arginine; urea cycle, polyamines	Anaerobic Oxidative Degradation of L-Ornithine	Probable reactivating factor for D-ornithine aminomutase	0	0.004524	0.00511	0	0.00197	0.001948	0.000722	0.001495	0	0	0	0.001774744
Carbohydrates	Sugar alcohols	Propanediol utilization	Propanediol dehydratase medium subunit (EC 4.2.1.28)	0	0.002262	0.002555	0	0.000657	0.001461	0.000722	0.000498	0	0	0	0.000236633
Carbohydrates	Sugar alcohols	Propanediol utilization	Propanediol utilization polyhedral body protein PduA	0	0.002262	0.00511	0	0.00197	0.000487	0	0	0.000876	0	0.000116	0.00063102
Clustering-based subsystems	proteosome related	Cluster-based Subsystem Grouping Hypotheticals - perhaps Proteosome Related	Proteasome subunit alpha (EC 3.4.25.1), bacterial	0	0	0.00511	0.00498	0	0	0.000722	0	0.001971	0.002203	0.000466	0.000394388
Protein Metabolism	Protein degradation	Proteasome bacterial	proteasome-activating AAA-ATPase (PAN)	0.000348	0	0	0	0.002626	0	0.000722	0.002492	0.002846	0	0.000116	0.000907091
Phages, Prophages, Transposable elements, Plasmids	Phages, Prophages	Listeria phi-A118-like prophages	Protein gp47, recombination-related [Bacteriophage A118]	0	0	0	0	0.000657	0.000487	0.00361	0.002492	0.001095	0	0.000233	0.001932499
Membrane Transport	Protein secretion system, Type VI	Type VI secretion systems	Protein of avirulence locus ImpE	0.001741	0	0	0	0.001313	0.000487	0.000722	0.000997	0.001314	0	0	0.001695866
Cell Wall and Capsule	Gram-Negative cell wall components	Lipopolysaccharide-related cluster in Alphaproteobacteria	Protein of unknown function DUF374	0.000696	0	0.002555	0	0	0.000487	0.001444	0.000997	0.000657	0	0	0.00220857
Regulation and Cell signaling	NULL	Rcs phosphorelay signal transduction pathway	Protein RcsF	0	0	0.002555	0	0.001313	0.003409	0.002888	0.001993	0.001533	0	0	0.000828214
Clustering-based subsystems	NULL	CBSS-243277.1.peg.4359	Protein YcgL	0	0	0	0.00249	0	0.001461	0.001444	0.000997	0.002846	0	0.009082	0.001971938
Cofactors, Vitamins, Prosthetic Groups, Pigments	Tetrapyrroles	Heme and Siroheme Biosynthesis	Protoporphyrinogen IX oxidase, oxygen-independent, HemG (EC 1.3.-.-)	0.008356	0	0.007664	0	0	0.004383	0	0.000997	0.000219	0.019825	0	0.005994691
Regulation and Cell signaling	NULL	The Chv regulatory system of Alphaproteobacteria	PTS system permease (IIAMan), nitrogen regulatory IIA protein	0.004178	0	0.002555	0	0	0.002922	0.002166	0.00598	0.004598	0	0	0.002326887
Carbohydrates	Monosaccharides	D-Tagatose and Galactitol Utilization	PTS system, galactitol-specific IIC component (EC 2.7.1.69)	0	0.002262	0	0	0.001313	0.000487	0.000722	0	0	0.004406	0.000116	0.000670459
Cell Wall and Capsule	Capsular and extracellular polysacchrides	Sialic Acid Metabolism	PTS system, N-acetylglucosamine-specific IIB component (EC 2.7.1.69)	0.005222	0	0	0	0	0	0.000722	0.000498	0.000438	0.011014	0.000582	0.000394388
Metabolism of Aromatic Compounds	NULL	Gentisare degradation	putative 4-hydroxybenzoyl-CoA thioesterase	0	0	0	0	0.003939	0.003409	0.005777	0.005482	0.003941	0	0.000466	0.001301479
Respiration	NULL	Biogenesis of cbb3-type cytochrome c oxidases	Putative analog of CcoH, COG3198	0.004874	0	0	0	0.000657	0.001948	0.002166	0.003489	0.004598	0	0	0.000552143
Stress Response	NULL	Bacterial hemoglobins	Putative bacterial haemoglobin	0	0	0	0	0.003283	0.000487	0.005055	0.002492	0.003284	0.004406	0	0.002681835
Carbohydrates	One-carbon Metabolism	Serine-glyoxylate cycle	putative isobutyryl-CoA mutase, chain B	0.000348	0	0	0.00249	0	0	0.000722	0.000498	0.000657	0	0.000582	0.000197194
Fatty Acids, Lipids, and Isoprenoids	Triacylglycerols	Triacylglycerol metabolism	Putative lipase in cluster with Phosphatidate cytidylyltransferase	0	0	0.002555	0	0.005252	0.001461	0	0.000498	0.001533	0	0.000233	0.001538111
Carbohydrates	Central carbohydrate metabolism	TCA Cycle	Putative malate dehydrogenase (EC 1.1.1.37), similar to archaeal MJ1425	0	0.002262	0	0.00747	0	0.003896	0	0	0.000657	0.004406	0.000233	0.001735305
Miscellaneous	Plant-Prokaryote DOE project	COG0523	putative metal chaperone, involved in Fe-nitrile hydratase activation, GTPase of COG0523 family	0	0	0	0.00249	0	0.000487	0.000722	0.000997	0.002189	0	0.000116	0.000354949
Carbohydrates	NULL	Unknown carbohydrate utilization (cluster Yeg)	Putative nucleoside transporter YegT	0	0	0	0.00747	0.003939	0.000487	0.001444	0.001495	0.005474	0	0	0.002287448
DNA Metabolism	DNA repair	2-phosphoglycolate salvage	Putative phosphatase YieH	0.002437	0	0.002555	0	0.001313	0.000974	0.001444	0	0.000657	0	0	0.000512704
RNA Metabolism	RNA processing and modification	rRNA modification Archaea	Putative RNA-binding protein COG1094	0	0	0	0.00249	0.003283	0.000974	0.00361	0.001993	0.000876	0	0	0.000709898
RNA Metabolism	RNA processing and modification	tRNA modification Bacteria	Putative sulfite reductase, gamma subunit (EC 1.8.99.3)	0.002437	0	0.002555	0.00996	0.000657	0	0.002888	0	0.000876	0	0	0.001459234
Clustering-based subsystems	Molybdopterin oxidoreductase	CBSS-269799.3.peg.2220	Putative uncharacterized protein TTHA1760	0	0.002262	0	0	0.001313	0.002922	0.001444	0.002492	0.003941	0	0	0.00252408
Amino Acids and Derivatives	Proline and 4-hydroxyproline	Proline, 4-hydroxyproline uptake and utilization	PutR, transcriptional activator of PutA and PutP	0	0	0	0	0.001313	0.000487	0	0.00299	0.005036	0.004406	0.023869	0.002957907
Iron acquisition and metabolism	Siderophores	Siderophore Pyoverdine	PvdO, pyoverdine responsive serine/threonine kinase (predicted by OlgaV)	0.001741	0	0.00511	0	0.001313	0.001461	0	0.004485	0.003065	0	0	0.001538111
Iron acquisition and metabolism	Siderophores	Siderophore Pyoverdine	Pyoverdine sidechain non-ribosomal peptide synthetase PvdJ	0.005222	0	0	0	0.001313	0.001948	0.002888	0.00598	0.003284	0	0	0.001419795
Nucleosides and Nucleotides	Pyrimidines	Pyrimidine utilization	Pyrimidine ABC transporter, transmembrane component 1	0	0.006786	0	0	0	0.002922	0.002888	0.005482	0.005255	0	0.001164	0.001143724
Amino Acids and Derivatives	Aromatic amino acids and derivatives	Chorismate Synthesis	Quinate/shikimate dehydrogenase [Pyrroloquinoline-quinone] (EC 1.1.99.25)	0	0	0.002555	0	0.004596	0.000974	0.002888	0.00299	0.005255	0	0	0.000867653
Respiration	Electron donating reactions	Hydrogenases	quinone-reactive Ni/Fe-hydrogenase, small subunit	0	0.002262	0	0.00249	0	0	0.000722	0	0.000219	0.002203	0.000233	0.010490709
Phosphorus Metabolism	NULL	Phosphate-binding DING proteins	RecA/RadA recombinase	0.001741	0	0.00511	0.00249	0	0	0.001444	0.001993	0.007882	0	0	0.00063102
Stress Response	Oxidative stress	Regulation of Oxidative Stress Response	Redox-sensitive transcriptional activator SoxR	0	0	0	0	0.004596	0.000974	0.004333	0	0.002627	0.004406	0.002911	0.00347061
Fatty Acids, Lipids, and Isoprenoids	Isoprenoids	Carotenoids	Regulator of carotenoid biosynthesis	0	0.009048	0	0	0.00197	0.003896	0	0.007974	0.003941	0	0.000233	0.000473265

DNA Metabolism	DNA replication	DNA replication, archaeal	Replication factor C large subunit	0	0	0	0.00249	0.002626	0.001948	0.004333	0.001993	0.002189	0	0	0.001222601
Phosphorus Metabolism	NULL	Phosphate metabolism	response regulator in two-component regulatory system with PhoQ	0	0.006786	0	0.00498	0.003283	0.001948	0	0.001495	0.005036	0	0	0.004811528
Nucleosides and Nucleotides	NULL	Ribonucleotide reduction	Ribonucleotide reductase of class Ib (aerobic), alpha subunit (EC 1.17.4.1)	0	0	0	0	0.01904	0.015098	0.015886	0.018938	0.000876	0	0.001747	0.000749336
Nucleosides and Nucleotides	NULL	AMP to 3-phosphoglycerate	Ribose-1,5-bisphosphate isomerase	0.000348	0	0	0	0.000657	0.001461	0.001444	0.000997	0.001314	0	0	0.001498673
Clustering-based subsystems	NULL	NusA-TFII Cluster	ribosomal protein L7Ae family protein	0.000696	0	0	0	0	0.002435	0.000722	0.002492	0.000438	0	0.000116	3.94388E-05
RNA Metabolism	RNA processing and modification	rRNA modification Archaea	Ribosomal RNA large subunit methyltransferase J (EC 2.1.1.-)	0	0	0.002555	0	0.000657	0.000487	0.000722	0.001495	0.001095	0	0	0.000512704
RNA Metabolism	Transcription	Transcription initiation, bacterial sigma factors	RNA polymerase principal sigma factor HrdD	0	0.002262	0	0	0.001313	0.000487	0	0.000498	0.000876	0.002203	0	0.000157755
Clustering-based subsystems	Putative asociate of RNA polymerase sigma-54 factor rpoN	CBSS-316057.3.peg.1308	RNA polymerase sigma-E factor	0.006963	0	0	0	0.002626	0.003896	0.002888	0.003987	0.003284	0	0	0.002169132
Carbohydrates	CO2 fixation	CO2 uptake, carboxysome	RuBisCO operon transcriptional regulator CbbR	0.012882	0	0	0.00249	0	0.001948	0	0.001495	0.000657	0.002203	0	0.002248009
Membrane Transport	Protein secretion system, Type I	Type I protein secretion systems	Secreted alkaline metalloproteinase (EC 3.4.24.-), PrtA/B/C/G homolog	0	0.004524	0	0	0	0.000487	0.000722	0.001993	0.001095	0	0.00326	0.000394388
Phosphorus Metabolism	NULL	Phosphate metabolism	secreted alkaline phosphatase	0	0	0	0	0.002626	0.001948	0.001444	0.001495	0.003722	0	0.000349	0.000788775
Carbohydrates	CO2 fixation	CO2 uptake, carboxysome	Sensory subunit of low CO2-induced protein complex, putative	0.000348	0	0	0	0.001313	0.002435	0.002888	0.002492	0.006568	0	0	0.002090254
Amino Acids and Derivatives	Aromatic amino acids and derivatives	Chorismate Synthesis	Shikimate 5-dehydrogenase I gamma (EC 1.1.1.25)	0.000696	0.002262	0	0	0.001313	0.000487	0.000722	0	0.000219	0	0	0.001340918
RNA Metabolism	Transcription	Transcription initiation, bacterial sigma factors	Sigma factor RpoE regulatory protein RseC	0.000696	0	0	0	0	0.000487	0.001444	0.000997	0.001095	0.002203	0	0.000709898
Amino Acids and Derivatives	Glutamine, glutamate, aspartate, asparagine; ammonia assimilation	Glutamate and Aspartate uptake in Bacteria	Signal transduction histidine kinase regulating C4-dicarboxylate transport system (EC 2.7.13.3)	0	0	0.002555	0	0.000657	0.000487	0.001444	0.000997	0.002846	0	0	0.002050815
Protein Metabolism	Protein biosynthesis	Ribosome biogenesis bacterial	Similar to ribosomal large subunit pseudouridine synthase D, Bacillus subtilis YjbO type	0	0	0.002555	0	0.001313	0.000487	0.000722	0.000498	0.000657	0	0	0.000394388
Regulation and Cell signaling	Quorum sensing and biofilm formation	Autoinducer 2 (AI-2) transport and processing (lsrACDBFGE operon)	S-ribosylhomocysteine lyase (EC 4.4.1.21)	0.003133	0	0.00511	0.00249	0	0	0	0.000498	0.001533	0	0.035978	0.005087599
Protein Metabolism	Protein biosynthesis	Ribosome SSU eukaryotic and archaeal	SSU ribosomal protein S19e	0	0	0	0	0.00197	0.001461	0.005777	0.001993	0.000219	0.004406	0	0.001025408
Protein Metabolism	Protein biosynthesis	Ribosome SSU eukaryotic and archaeal	SSU ribosomal protein S3Ae	0	0	0	0.00498	0.000657	0.000487	0.001444	0	0.002189	0.002203	0	0.000354949
Protein Metabolism	Protein biosynthesis	Ribosome SSU eukaryotic and archaeal	SSU ribosomal protein S3e (S3p)	0	0.002262	0.002555	0	0	0	0.000722	0.000498	0.001533	0	0.000349	0.000788775
Protein Metabolism	Protein biosynthesis	Ribosome SSU eukaryotic and archaeal	SSU ribosomal protein S4e	0	0	0	0	0.001313	0.001948	0.000722	0.000498	0.001095	0.008811	0	0.000788775
Dormancy and Sporulation	NULL	Sporulation gene orphans	Stage V sporulation protein E	0	0	0.010219	0.00249	0.000657	0.005844	0.002888	0.000997	0	0	0	0.000552143
Regulation and Cell signaling	NULL	Staphylococcal accessory gene regulator system	Staphylococcal respiratory response protein SrrB	0	0.002262	0	0.00249	0.001313	0.000487	0.001444	0	0.000438	0	0	0.000473265
Amino Acids and Derivatives	Lysine, threonine, methionine, and cysteine	Cysteine Biosynthesis	Sulfate transport system permease protein CysT	0.001044	0	0	0	0.00197	0.000487	0.001444	0	0.002189	0	0.001048	0.001932499
Stress Response	Oxidative stress	Regulation of Oxidative Stress Response	Superoxide dismutase [Cu-Zn] (EC 1.15.1.1)	0	0	0	0	0.00197	0.001461	0.000722	0.002492	0.002408	0	0.000466	0.001340918
Carbohydrates	Aminosugars	N-Acetyl-Galactosamine and Galactosamine Utilization	Tagatose-6-phosphate kinase AgaZ (EC 2.7.1.144)	0.000348	0	0	0	0	0.001948	0.001444	0.001993	0.001095	0	0.003027	0.001419795
Sulfur Metabolism	Organic sulfur assimilation	Taurine Utilization	Taurine-binding periplasmic protein TauA	0.003133	0	0	0.00498	0	0.003409	0.000722	0.003987	0.004379	0	0	0.000473265
Fatty Acids, Lipids, and Isoprenoids	Fatty acids	Acyl-CoA thioesterase II	TesB-like acyl-CoA thioesterase 3	0.001393	0	0	0	0.001313	0.001461	0.002166	0.002492	0.000438	0	0	0.000197194
Respiration	NULL	Reductive Dechlorination	Tetrachloroethene reductive dehalogenase PceA (EC 1.97.1.8)	0	0	0.002555	0	0.003939	0.008767	0.002166	0.000997	0	0	0.000116	0.000433826
Respiration	NULL	Reductive Dechlorination	Tetrachloroethene reductive dehalogenase TceA	0	0	0	0.00498	0.001313	0.002922	0.002888	0.000498	0.000219	0	0	0.000473265
RNA Metabolism	Transcription	Transcription elongation factors, archaeal	Transcription factor S	0	0.002262	0	0	0.000657	0.000487	0.001444	0.001495	0.000219	0	0	0.000236633
Miscellaneous	Plant-Prokaryote DOE project	At4g38090	Transcriptional regulator DegU, LuxR family	0.004526	0	0.012774	0.00249	0	0.002922	0	0.000997	0.001752	0	0	0.001104285
Fatty Acids, Lipids, and Isoprenoids	Fatty acids	Fatty acid degradation regulons	Transcriptional regulator for fatty acid degradation FadR, GntR family	0.002785	0.004524	0	0	0	0.000974	0	0.001495	0.006787	0	0.003842	0.001183163
Carbohydrates	Monosaccharides	D-Galacturonate and D-Glucuronate Utilization	Transcriptional regulator KdgR, KDG operon repressor	0	0	0	0.00249	0	0.000487	0.000722	0.000498	0.000438	0.004406	0	0.000354949
Phosphorus Metabolism	NULL	Alkylphosphonate utilization	Transcriptional regulator PhnF	0.000348	0	0	0	0.00197	0.000487	0	0.001993	0.002189	0	0.000466	0.000828214
Membrane Transport	ABC transporters	ATP-dependent efflux pump transporter Ybh	Transcriptional regulator YbiH, TetR family	0.000696	0	0	0	0.001313	0.002435	0.000722	0.000997	0.000219	0	0	0.000276071
Stress Response	Detoxification	Glutathione-dependent pathway of formaldehyde detoxification	Transcriptional regulator, LysR family, in formaldehyde detoxification operon	0	0.004524	0	0	0	0	0.001444	0.000997	0.006787	0.006608	0.04832	0.004062192
Regulation and Cell signaling	NULL	Oxygen and light sensor PpaA-PpsR	Transcriptional regulator, PpsR	0.002089	0.004524	0	0	0.000657	0.002922	0	0.003987	0.001752	0	0	0.000118316
Cell Wall and Capsule	Gram-Negative cell wall components	Lipid A modifications	Transcriptional regulatory protein PhoP	0	0	0.00511	0.00249	0	0.000487	0	0.000498	0.001095	0	0.000349	0.00157755
Clustering-based subsystems	Tricarboxylate transporter	CBSS-49338.1.peg.459	TRAP dicarboxylate transporter, DctM subunit, unknown substrate 8	0	0	0.002555	0	0.002626	0.000487	0.002166	0.003489	0.001752	0	0	0.001774744
Miscellaneous	Plant-Prokaryote DOE project	At4g17370	TRAP-type C4-dicarboxylate transport system, possibly of alkanesulfonates, periplasmic component	0.000696	0	0.002555	0	0	0	0.000722	0.001993	0.004817	0	0.000582	0.000591581
RNA Metabolism	RNA processing and modification	tRNA modification Bacteria	tRNA dihydrouridine synthase A (EC 1.-.-.-)	0	0	0.002555	0	0.000657	0.000487	0	0	0.002189	0.002203	0.000116	0.000828214
Cell Wall and Capsule	NULL	tRNA-dependent amino acid transfers	tRNA-dependent lipid II--amino acid ligase	0	0	0.00511	0	0.00197	0.001948	0.000722	0.001495	0.000876	0	0	0.002366325
Respiration	NULL	Biogenesis of cbb3-type cytochrome c oxidases	Type cbb3 cytochrome oxidase biogenesis protein CcoH	0	0	0.002555	0	0	0.002922	0.001444	0.003489	0.001095	0	0.001281	0.000867653
Membrane Transport	Protein secretion system, Type III	Type III secretion system	Type III secretion inner membrane channel protein (LcrD,HrcV,EscV,SsaV)	0	0	0	0.00498	0.004596	0.000974	0.002888	0	0.004817	0	0.000582	0.002050815
Membrane Transport	Protein secretion system, Type II	Widespread colonization island	Type IV prepilin peptidase TadV/CpaA	0.005222	0	0	0	0.00197	0.000974	0.001444	0.003987	0.001095	0	0	0.001301479
Cell Wall and Capsule	Capsular and extracellular polysacchrides	Capsular Polysaccharides Biosynthesis and Assembly	UDP-N-acetylglucosamine 4-epimerase (EC 5.1.3.7)	0	0.002262	0	0	0.000657	0.000487	0	0.000498	0.000438	0	0.00978	0.000394388
Cell Wall and Capsule	NULL	Peptidoglycan biosynthesis--gjo	UDP-N-acetylmuramoylalanyl-D-glutamate--L-lysine ligase (EC 6.3.2.7)	0.000348	0	0	0.00498	0	0.000487	0	0.000997	0.000657	0	0.000116	0.000788775
Cell Wall and Capsule	Gram-Negative cell wall components	Lipopolysaccharide assembly	Uncharacterized ABC transporter, auxiliary component YrbC	0.000696	0	0.002555	0	0	0.001461	0.00361	0.001495	0.012042	0	0	0.001932499
Respiration	NULL	Methanogenesis strays	Uncharacterized ferredoxin oxidoreductase MJ0100	0	0.004524	0	0.00498	0	0.00487	0.001444	0	0	0.006608	0.000233	0.003352294
Respiration	Electron donating reactions	CO Dehydrogenase	Uncharacterized MobA-related protein	0.005222	0	0	0	0.00197	0.002922	0.000722	0.00299	0.005036	0	0	0.000709898
Miscellaneous	Plant-Prokaryote DOE project	COG0398	Uncharacterized protein DUF1284, possibly iron-sulphur binding	0.004178	0.002262	0	0	0.006566	0.001948	0.001444	0.002492	0	0	0	0.001301479
Clustering-based subsystems	NULL	Yfa cluster	Uncharacterized protein YfaU	0	0	0.002555	0	0.000657	0.000487	0	0.000498	0.000219	0	0.003842	0.000157755
Stress Response	NULL	Universal stress protein family	Universal stress protein family 1	0	0	0	0.00249	0.004596	0.00487	0.002166	0.002492	0.000876	0	0	0.000591581
Clustering-based subsystems	Two related proteases	CBSS-257314.1.peg.676	Unspecified monosaccharide ABC transport system, permease component I	0.001393	0.004524	0.002555	0	0	0.001948	0	0.000498	0.001752	0	0	0.000394388
Nucleosides and Nucleotides	Purines	Purine conversions	Xanthine phosphoribosyltransferase (EC 2.4.2.22)	0	0	0.00511	0	0.000657	0.000974	0	0	0.000438	0.002203	0.000349	0.001301479

Cell Wall and Capsule	NULL	YjeE	YjeF protein, C-terminal domain	0	0	0	0	0.000657	0.000974	0.000722	0.000997	0.000219	0	0.005589	0.000670459
Carbohydrates	Aminosugars	(GlcNAc)2 Catabolic Operon	(GlcNAc)2 ABC transporter, permease component 1	0	0	0.002555	0	0.000657	0	0	0.002492	0.001095	0	0.003959	0.001025408
Carbohydrates	Aminosugars	(GlcNAc)2 Catabolic Operon	(GlcNAc)2 ABC transporter, permease component 2	0	0	0	0	0	0.000487	0	0.000498	0.000438	0.006608	0.000699	0.00031551
Secondary Metabolism	Biosynthesis of phenylpropanoids	Phenylpropionate Degradation	1,2-dihydroxycyclohexa-3,5-diene-1-carboxylate dehydrogenase (EC 1.3.1.25)	0	0	0	0	0	0.000487	0.001444	0	0.001314	0.004406	0.032834	0.001025408
Carbohydrates	Di- and oligosaccharides	Unknown oligosaccharide utilization Sde 1396	1st GPH family transporter in unknown oligosaccharide utilization Sde 1396	0.000696	0.002262	0	0	0.000657	0.000974	0	0.000997	0	0	0	0.000552143
Amino Acids and Derivatives	Lysine, threonine, methionine, and cysteine	Methionine Salvage	2,3-diketo-5-methylthiopentyl-1-phosphate enolase-phosphatase (EC 3.1.3.77)	0	0	0	0	0.000657	0.001461	0	0.001993	0.000876	0	0.022821	0.004496018
Metabolism of Aromatic Compounds	Anaerobic degradation of aromatic compounds	Anaerobic toluene and ethylbenzene degradation	2-[hydroxy(phenyl)methyl]-succinyl-CoA dehydrogenase beta subunit (EC 1.1.1.35)	0	0	0	0.00249	0.001313	0.001948	0	0	0.000876	0	0.000116	3.94388E-05
RNA Metabolism	RNA processing and modification	RNA processing orphans	2'-5' RNA ligase	0.000696	0	0.002555	0	0.003283	0	0	0.006479	0.001752	0	0	0.000512704
DNA Metabolism	DNA repair	2-phosphoglycolate salvage	2-deoxyglucose-6-phosphate hydrolase YniC	0	0	0	0	0	0.000487	0.000722	0.000498	0.000438	0	0.000466	0.000867653
Carbohydrates	Monosaccharides	D-gluconate and ketogluconates metabolism	2-ketoaldonate reductase, broad specificity (EC 1.1.1.215) (EC 1.1.1.-)	0	0.002262	0	0	0	0.001461	0.001444	0.001495	0.001095	0	0	0.000709898
Carbohydrates	Monosaccharides	D-gluconate and ketogluconates metabolism	2-Keto-D-gluconate dehydrogenase (EC 1.1.99.4), membrane-bound, flavoprotein	0	0.002262	0	0	0.000657	0	0.000722	0.000498	0.001752	0	0	3.94388E-05
Cofactors, Vitamins, Prosthetic Groups, Pigments	Coenzyme M	coenzyme M biosynthesis	2-phosphosulfolactate phosphatase (EC 3.1.3.71)	0.000348	0	0	0	0	0.000974	0.001444	0.000997	0.002408	0	0	0.001025408
Amino Acids and Derivatives	Aromatic amino acids and derivatives	Aromatic amino acid degradation	3,4-dihydroxyphenylacetate 2,3-dioxygenase (EC 1.13.11.15)	0	0	0.002555	0	0.001313	0	0.002166	0	0.002846	0	0.066717	0.005363671
Carbohydrates	Fermentation	Acetyl-CoA fermentation to Butyrate	3-hydroxybutyrate dehydrogenase (EC 1.1.1.30)	0	0	0	0.00249	0	0.000487	0.001444	0	0.001314	0	0.000116	0.000354949
Metabolism of Aromatic Compounds	Metabolism of central aromatic intermediates	Protocatechuate branch of beta-ketoadipate pathway	3-oxoadipate enol-lactone hydrolase/4-carboxymuconolactone decarboxylase	0	0.002262	0	0	0.000657	0	0	0.000997	0.000438	0.002203	0	0.000197194
Protein Metabolism	Protein processing and modification	N-linked Glycosylation in Bacteria	4-amino-6-deoxy-N-Acetyl-D-hexosaminyl-(Lipid carrier) acetyltrasferase	0	0	0	0	0.001313	0.003409	0.002166	0.001993	0.001314	0	0	0.003864998
Cofactors, Vitamins, Prosthetic Groups, Pigments	Pyridoxine	Pyridoxin(Vitamin B6) Degradation Pathway	4-pyridoxolactonase (EC 3.1.1.27)	0	0.002262	0.002555	0	0	0.000487	0	0.000997	0.000657	0	0	0.001971938
Carbohydrates	Monosaccharides	D-Galacturonate and D-Glucuronate Utilization	5-dehydro-4-deoxyglucarate dehydratase (EC 4.2.1.41)	0	0	0	0	0	0.000974	0.002166	0.000997	0.003503	0	0.000116	0.000197194
Amino Acids and Derivatives	Aromatic amino acids and derivatives	Aromatic amino acid degradation	5-oxopent-3-ene-1,2,5-tricarboxylate decarboxylase.	0	0	0.002555	0	0	0.000487	0.000722	0.000997	0.002846	0	0	0.000749336
Carbohydrates	One-carbon Metabolism	Formaldehyde assimilation: Ribulose monophosphate pathway	6-phospho-3-hexuloisomerase	0	0.002262	0	0	0.001313	0.000974	0	0	0.000219	0	0.000116	0.000512704
Iron acquisition and metabolism	NULL	ABC transporter [iron.B12.siderophore.hemin]	ABC transporter (iron.B12.siderophore.hemin) , permease component	0	0	0	0	0.00197	0	0.000722	0.000498	0.000657	0	0.000466	0.000394388
Iron acquisition and metabolism	Siderophores	Siderophore assembly kit	ABC-type Fe3+-siderophore transport system, ATPase component	0	0	0	0.00249	0	0.001461	0	0.001993	0.003065	0	0.001397	0.00063102
Membrane Transport	Protein secretion system, Type I	Type I protein secretion systems	ABC-type protease exporter, membrane fusion protein (MFP) family component PrtE/AprE	0	0.002262	0	0	0.000657	0	0.001444	0.000498	0.000876	0	0	0.002445203
Carbohydrates	NULL	Acetone carboxylase	Acetone carboxylase, gamma subunit (EC 6.4.1.6)	0	0.002262	0.002555	0	0	0.000487	0	0	0.000219	0	0.005589	0.000552143
Cofactors, Vitamins, Prosthetic Groups, Pigments	Tetrapyrroles	Coenzyme B12 biosynthesis	Adenosylcobinamide amidohydrolase (EC 3.5.1.90)	0.004874	0	0	0	0.001313	0.000974	0	0.000997	0.000657	0	0	0.000354949
Nitrogen Metabolism	NULL	Allantoin Utilization	Allantoate amidohydrolase (EC 3.5.3.9)	0	0	0.007664	0	0	0.000974	0.000722	0.000498	0.000876	0	0	0.000591581
Cell Wall and Capsule	Capsular and extracellular polysacchrides	Rhamnose containing glycans	Alpha-L-Rha alpha-1,3-L-rhamnosyltransferase (EC 2.4.1.-)	0	0.002262	0	0	0.00197	0	0.000722	0.001495	0.000657	0	0	0.001183163
Cofactors, Vitamins, Prosthetic Groups, Pigments	Folate and pterines	5-FCL-like protein	Alternative dihydrofolate reductase 2	0	0	0	0	0.000657	0.000487	0	0.000997	0.000876	0	0.000116	0.004377702
Protein Metabolism	Protein processing and modification	Inteins	Amino-terminal intein-mediated trans-splice	0	0	0	0	0	0.000974	0.001444	0.000498	0.000657	0	0.000349	0.000473265
Cofactors, Vitamins, Prosthetic Groups, Pigments	Folate and pterines	Methanopterin biosynthesis2	Archaeal flavoprotein COG1036	0.005571	0	0	0.00498	0	0	0	0.000498	0.000438	0.004406	0	0.00094653
RNA Metabolism	RNA processing and modification	Queuosine-Archaeosine Biosynthesis	archaeosine tRNA-ribosyltransferase (EC 2.4.2.-) type 5	0.000348	0.006786	0	0	0.000657	0.001461	0	0	0.001095	0	0	0.000670459
Amino Acids and Derivatives	Arginine; urea cycle, polyamines	Arginine and Ornithine Degradation	Arginine ABC transporter, permease protein ArtQ	0	0	0	0	0	0.000487	0	0.000498	0.002189	0.011014	0.025965	0.002563519
Amino Acids and Derivatives	Arginine; urea cycle, polyamines	Polyamine Metabolism	Arginine/ornithine antiporter	0	0	0	0.00249	0	0.000974	0.000722	0	0.002408	0	0.001514	0.001735305
Amino Acids and Derivatives	Arginine; urea cycle, polyamines	Arginine Deiminase Pathway	Arginine/ornithine antiporter ArcD	0	0.004524	0	0	0.001313	0.001948	0	0.001993	0.005255	0	0	0.003194539
Regulation and Cell signaling	NULL	DNA-binding regulatory proteins, strays	Aromatic hydrocarbon utilization transcriptional regulator CatR (LysR family)	0	0	0	0	0.001313	0.000487	0	0.000997	0.003065	0	0.046341	0.001656428
Virulence, Disease and Defense	Resistance to antibiotics and toxic compounds	Arsenic resistance	Arsenic resistance protein ArsH	0	0.004524	0	0	0	0.000487	0	0.005482	0.003065	0.004406	0	0.000985969
DNA Metabolism	DNA repair	DNA repair, bacterial RecBCD pathway	ATP-dependent DNA helicase SCO5184	0	0	0.002555	0	0.00197	0.000487	0.000722	0	0.000219	0	0	0.000157755
RNA Metabolism	RNA processing and modification	ATP-dependent RNA helicases, bacterial	ATP-dependent RNA helicase PA3950	0	0	0	0	0.001313	0.000487	0.000722	0.000498	0	0	0.000233	0.000552143
RNA Metabolism	RNA processing and modification	ATP-dependent RNA helicases, bacterial	ATP-dependent RNA helicase VC1407	0	0	0.002555	0	0.001313	0	0.000722	0.000498	0	0	0.001979	0.007532802
RNA Metabolism	RNA processing and modification	ATP-dependent RNA helicases, bacterial	ATP-dependent RNA helicase VCA0768	0	0	0	0	0.000657	0	0	0.000498	0.000438	0.004406	0.063573	0.003825559
Cell Wall and Capsule	Capsular and extracellular polysacchrides	Legionaminic Acid Biosynthesis	Bacillosamine/Legionaminic acid biosynthesis aminotransferase PglE	0	0	0.002555	0	0.001313	0.006332	0	0.001993	0.002627	0	0	0.00378612
Dormancy and Sporulation	NULL	Exosporium	BclA protein	0	0	0	0.00249	0.00197	0.001461	0.001444	0.001495	0	0	0	0.000197194
Metabolism of Aromatic Compounds	Peripheral pathways for catabolism of aromatic compounds	Benzoate degradation	Benzoate 1,2-dioxygenase alpha subunit (EC 1.14.12.10)	0.001393	0	0	0	0	0.000487	0	0	0.004817	0.004406	0.097222	0.001538111
Metabolism of Aromatic Compounds	Peripheral pathways for catabolism of aromatic compounds	Benzoate catabolism	Benzoate 1,2-dioxygenase beta subunit (EC 1.14.12.10)	0	0	0	0	0.000657	0	0.000722	0	0.000438	0.002203	0.024218	0.000394388
Metabolism of Aromatic Compounds	Peripheral pathways for catabolism of aromatic compounds	Benzoate degradation	benzoate dioxygenase, ferredoxin reductase component	0	0	0	0	0.000657	0	0.002166	0	0.001752	0.004406	0.042731	0.000512704
Carbohydrates	NULL	Sugar utilization in Thermotogales	Beta-galactosidase (EC 3.2.1.23), LacZ family	0.006267	0	0	0	0.001313	0.000487	0.00361	0.000498	0	0	0	0.002050815
Carbohydrates	Aminosugars	(GlcNAc)2 Catabolic Operon	Beta-N-acetylhexosaminidase, (GlcNAc)2 catabolism	0	0	0	0	0.000657	0.000487	0	0	0.000657	0.002203	0.000233	0.000236633
Cofactors, Vitamins, Prosthetic Groups, Pigments	Folate and pterines	methanopterin biosynthesis	beta-ribofuranosylaminobenzene 5'-phosphate synthase	0	0	0.002555	0	0.001313	0.000487	0	0.000997	0.000438	0	0	0.000867653
Fatty Acids, Lipids, and Isoprenoids	Fatty acids	Fatty Acid Biosynthesis FASI	Biotin carboxylase of ACAC1 (EC 6.3.4.14)	0.000696	0	0.00511	0	0	0.000487	0	0.000997	0.000219	0	0	0.000157755
Carbohydrates	Fermentation	Acetyl-CoA fermentation to Butyrate	Butyrate-acetoacetate CoA-transferase subunit A (EC 2.8.3.9)	0.005571	0.002262	0	0	0.000657	0.001948	0	0	0	0	0.000116	0.000709898
Cell Wall and Capsule	Capsular and extracellular polysacchrides	Capsular Polysaccharides Biosynthesis and Assembly	Capsular polysaccharide biosynthesis/export periplasmic protein WcbC	0.002437	0	0	0	0	0.000487	0	0.002492	0.001533	0	0.000116	0.000276071
Cell Wall and Capsule	Capsular and extracellular polysacchrides	Serotype determining Capsular polysaccharide biosynthesis in Staphylococcus	Capsular polysaccharide synthesis enzyme Cap8F	0	0.002262	0	0	0.000657	0	0.000722	0.001495	0.000219	0	0	0.000985969
Fatty Acids, Lipids, and Isoprenoids	Fatty acids	Carnitine Metabolism in Microorganisms	Carnitine operon protein CaiE	0	0	0	0.00249	0	0.000974	0.000722	0	0.001533	0	0.009198	0.000473265
Regulation and Cell signaling	NULL	HPr catabolite repression system	Catabolite repression HPr-like protein Crh	0	0	0.002555	0	0.001313	0	0.001444	0.000498	0.000438	0	0	0.000354949
Cell Division and Cell Cycle	NULL	Bacterial Cytoskeleton	Cell division protein ZipA	0	0.002262	0	0	0	0.000974	0.000722	0.006479	0.010072	0	0	0.001301479

Carbohydrates	Di- and oligosaccharides	Beta-Glucoside Metabolism	Cellobiose phosphotransferase system YdjC-like protein	0	0.006786	0.00511	0	0.001313	0.000974	0	0	0.000219	0	0	0.001656428
Miscellaneous	Plant-Prokaryote DOE project	At4g38090	Choline monooxygenase-like protein with aromatic-ring-hydroxylating dioxygenase domain	0.000696	0.002262	0	0	0	0	0.001444	0.002492	0.005474	0	0	0.001656428
Cell Division and Cell Cycle	NULL	Cyanobacterial Circadian Clock	Clock-associated two-component sensor histidine kinase SasA	0.000348	0	0.007664	0	0.001313	0.003409	0	0	0.001533	0	0	0.002090254
DNA Metabolism	DNA repair	DNA repair, bacterial MutL-MutS system	Clostridial MutS2-related protein	0	0	0	0.00249	0.000657	0.000487	0	0.000997	0.000876	0	0	0.000512704
Respiration	NULL	Carbon monoxide induced hydrogenase	CO dehydrogenase iron-sulfur protein CooF (EC 1.2.99.2)	0	0	0.00511	0	0.002626	0.000487	0.001444	0.000498	0	0	0	0.000867653
Cofactors, Vitamins, Prosthetic Groups, Pigments	Coenzyme B	Coenzyme B synthesis	Coenzyme B synthesis from 2-oxoglutarate: steps 4, 7, 8, 11, and 12 [small subunit]	0	0.002262	0	0	0	0.000487	0.000722	0.000498	0.000219	0	0	0.00063102
Cofactors, Vitamins, Prosthetic Groups, Pigments	Coenzyme B	Coenzyme B synthesis	Coenzyme B synthesis from 2-oxoglutarate: steps 5, 9, and 13	0.002785	0	0	0	0	0	0.000722	0.000997	0.000219	0	0.000116	0.000354949
Respiration	Electron donating reactions	Coenzyme F420-H2 dehydrogenase (methanophenazine)	Coenzyme F(420)H(2) dehydrogenase (methanophenazine) subunit FpoL	0.003482	0	0.002555	0	0.000657	0	0	0.000498	0.000657	0	0	0.000552143
Respiration	Electron donating reactions	Coenzyme F420-H2 dehydrogenase (methanophenazine)	Coenzyme F(420)H(2) dehydrogenase (methanophenazine) subunit FpoM	0.000348	0	0	0	0.000657	0.000487	0	0.000498	0.000219	0	0	0.000394388
Miscellaneous	Plant-Prokaryote DOE project	COG3380 COG2907	COG3380: Amine oxidase, flavin-containing	0	0	0	0	0.001313	0.003896	0.002888	0.002492	0.000657	0	0	0.00063102
Cell Wall and Capsule	Capsular and extracellular polysacchrides	Colanic acid biosynthesis	Colanic acid biosynthesis glycosyl transferase WcaL	0.001044	0	0	0	0.000657	0.002435	0	0.002492	0.001752	0	0	0.001025408
Stress Response	Cold shock	Cold shock, CspA family of proteins	Cold shock protein CspF	0.002785	0	0.002555	0	0.000657	0	0.000722	0	0.000219	0	0	0.000473265
Membrane Transport	Protein and nucleoprotein secretion system, Type IV	Conjugative transfer	Conjugative transfer protein TrbB	0	0	0	0	0.002626	0.001461	0	0.000498	0.002189	0	0.000349	0.000433826
Membrane Transport	Protein and nucleoprotein secretion system, Type IV	Conjugative transfer	Conjugative transfer protein TrbL	0	0	0	0	0.003283	0.000974	0	0.001495	0.001095	0	0.000233	0.000276071
Clustering-based subsystems	NULL	CBSS-316057.3.peg.563	conserved hypothetical protein in cyt c oxidase gene clusters	0	0	0.00511	0	0	0.000974	0.000722	0.003987	0.002189	0	0	0.000157755
Amino Acids and Derivatives	Branched-chain amino acids	Ketoisovalerate oxidoreductase	conserved protein associated with acetyl-CoA C-acyltransferase	0.006615	0	0	0	0.007879	0	0.001444	0.000498	0.000438	0	0	0.001380356
Virulence, Disease and Defense	Resistance to antibiotics and toxic compounds	Copper homeostasis	Copper resistance protein D	0	0	0	0	0.002626	0	0.005777	0.001993	0.002189	0	0.000116	0.000433826
DNA Metabolism	CRISPs	CRISP Cmr Cluster	CRISPR-associated protein NE0113	0	0.002262	0	0	0.001313	0.000974	0.000722	0	0	0	0.000233	0.00031551
Clustering-based subsystems	CRISPRs and associated hypotheticals	CBSS-216592.1.peg.3534	CRISPR-associated protein, Cas2	0	0	0	0	0.003939	0.002435	0.00361	0	0.001752	0	0.011178	0.000867653
DNA Metabolism	CRISPs	CRISPRs	CRISPR-associated protein, Csn1 family	0	0.002262	0	0	0	0.000974	0.000722	0.004485	0.002846	0	0	0.00063102
DNA Metabolism	CRISPs	CRISPRs	CRISPR-associated protein, CT1133 family	0	0	0	0	0.009192	0.004383	0.00361	0.002492	0.001533	0	0	0.002248009
DNA Metabolism	CRISPs	CRISP Cmr Cluster	CRISPR-associated RAMP Cmr2	0	0	0	0	0.002626	0.000487	0.002166	0.000997	0.001752	0	0	0.000473265
DNA Metabolism	CRISPs	CRISP Cmr Cluster	CRISPR-associated RAMP Cmr6	0	0.002262	0	0	0	0.000487	0.001444	0.000498	0.001095	0	0	0.000157755
Cofactors, Vitamins, Prosthetic Groups, Pigments	NULL	Molybdopterin cytosine dinucleotide	CTP:molybdopterin cytidylyltransferase	0.000696	0	0	0	0.001313	0.001948	0	0	0.001752	0.002203	0	0.001183163
Virulence, Disease and Defense	Resistance to antibiotics and toxic compounds	Copper homeostasis	Cu(I)-responsive transcriptional regulator	0	0	0	0	0.001313	0.000487	0.002888	0.010964	0.002846	0	0	0.001064846
Carbohydrates	Polysaccharides	Alpha-Amylase locus in Streptocococcus	Cyclomaltodextrin glucanotransferase (EC 2.4.1.19)	0	0	0	0	0	0.000974	0.002888	0.004485	0.003503	0	0.000466	0.001064846
Protein Metabolism	Protein biosynthesis	tRNA aminoacylation, Cys	Cysteinyl-tRNA synthetase paralog 1	0.001044	0.002262	0	0	0.00197	0.001461	0	0.000997	0	0	0	0.000157755
Respiration	NULL	Cytochrome B6-F complex	Cytochrome b6-f complex subunit IV (PetD)	0	0	0	0.00249	0	0	0.001444	0.000997	0.001095	0	0.000233	0.000118316
Respiration	NULL	Cytochrome B6-F complex	Cytochrome b6-f complex subunit, cytochrome b6	0	0.002262	0.002555	0	0	0	0	0.000498	0.005255	0	0.000349	0.000788775
Respiration	Electron accepting reactions	Terminal cytochrome C oxidases	Cytochrome c oxidase polypeptide IV(EC 1.9.3.1)	0	0	0	0	0.00197	0	0.000722	0.002492	0.000438	0.002203	0	0.000473265
Respiration	Electron accepting reactions	Terminal cytochrome C oxidases	Cytochrome c oxidase subunit CcoQ (EC 1.9.3.1)	0	0	0.010219	0	0.001313	0.002435	0	0.002492	0.002627	0	0	0.002484642
Nitrogen Metabolism	NULL	Dissimilatory nitrite reductase	Cytochrome c55X precursor NirC	0	0	0.010219	0	0.000657	0	0.002888	0.000498	0.000438	0	0	0.000670459
Carbohydrates	Di- and oligosaccharides	Maltose and Maltodextrin Utilization	Cytoplasmic alpha-amylase (EC 3.2.1.1)	0	0	0	0	0.004596	0.001461	0.00361	0.001495	0.004817	0	0	0.008597649
Fatty Acids, Lipids, and Isoprenoids	Isoprenoids	Polyprenyl Diphosphate Biosynthesis	Dehydrosqualene desaturase (EC 1.14.99.-) (Diapophytoene desaturase) (4,4'-diapophytoene desaturase)	0	0	0	0	0.000657	0.000974	0.000722	0.000997	0.000219	0	0	0.000197194
Amino Acids and Derivatives	Lysine, threonine, methionine, and cysteine	Lysine degradation	Delta 1-piperideine-2-carboxylate reductase (EC 1.5.1.21)	0	0.002262	0	0.00249	0	0.001948	0	0.000997	0.000219	0	0	0.000354949
DNA Metabolism	DNA repair	DNA repair, bacterial photolyase	Deoxyribodipyrimidine photolyase, single-strand-specific	0	0	0	0	0	0.000487	0	0.000498	0.003503	0.011014	0.000699	0.001301479
Sulfur Metabolism	NULL	Release of Dimethyl Sulfide (DMS) from Dimethylsulfoniopropionate (DMSP)	Dimethylsulfoniopropionate (DMSP) acyl CoA transferase DddD	0	0	0	0	0.001313	0.002435	0.002888	0.001495	0.000438	0	0	0.000118316
Amino Acids and Derivatives	Proline and 4-hydroxyproline	Proline, 4-hydroxyproline uptake and utilization	Dipeptidyl peptidase IV in 4-hydroxyproline catabolic gene cluster	0.001741	0	0.002555	0.00249	0	0	0.002166	0	0.000219	0	0	0.00126204
Phages, Prophages, Transposable elements, Plasmids	Phages, Prophages	Phage replication	DNA maturase, phage-associated	0	0	0	0	0.008535	0.007793	0.005055	0.004984	0	0.011014	0	0.000749336
DNA Metabolism	DNA repair	DNA repair, bacterial	DNA mismatch repair endonuclease MutH	0.000348	0	0	0	0	0	0.001444	0.00299	0.005255	0	0.014088	0.000867653
DNA Metabolism	DNA replication	DNA replication, archaeal	DNA primase small subunit (EC 2.7.7.-)	0	0	0.00511	0	0	0.001948	0.001444	0.000997	0.000657	0	0	0.000985969
DNA Metabolism	DNA replication	DNA topoisomerases, Type I, ATP-independent	DNA topoisomerase I, eukaryotic-type (EC 5.99.1.2)	0	0	0	0	0.003283	0.000487	0.005055	0.000997	0.000219	0	0	0.000907091
DNA Metabolism	DNA replication	DNA topoisomerases, Type I, ATP-independent	DNA topoisomerase IB (poxvirus type) (EC 5.99.1.2)	0	0	0	0	0.00197	0.002922	0.002166	0.00299	0.005255	0	0	0.000749336
RNA Metabolism	Transcription	RNA polymerase II	DNA-directed RNA polymerase II largest subunit (EC 2.7.7.6)	0	0	0.002555	0	0	0.001948	0.001444	0.001993	0	0	0.000233	0.000394388
RNA Metabolism	Transcription	RNA polymerase II	DNA-directed RNA polymerase II second largest subunit (EC 2.7.7.6)	0	0	0	0	0.00197	0.000487	0.00361	0.001495	0.000438	0	0	0.000276071
RNA Metabolism	Transcription	RNA polymerase archaeal	DNA-directed RNA polymerase subunit N (EC 2.7.7.6)	0	0	0.002555	0	0.000657	0.000487	0.00361	0	0.000438	0	0	0.001064846
DNA Metabolism	DNA repair	Uracil-DNA glycosylase	Domain often clustered or fused with uracil-DNA glycosylase	0.000348	0	0	0	0	0.004383	0	0.002492	0.001752	0	0.000466	0.001104285
Carbohydrates	Central carbohydrate metabolism	Pyruvate Alanine Serine Interconversions	D-serine dehydratase (EC 4.3.1.18)	0	0	0	0	0.001313	0	0.001444	0.001495	0.000876	0	0.000349	0.000828214
Carbohydrates	Monosaccharides	Xylose utilization	D-xylose-specific 1-epimerase (mutarotase)	0.001393	0.004524	0	0	0	0	0.001444	0.000997	0.000219	0	0	0.001222601
Carbohydrates	Polysaccharides	Cellulosome	Endoglucanase J, the largest catalytic component of the cellulosome	0	0	0	0	0.000657	0.000487	0.000722	0.000498	0.000657	0	0	0.000709898
Respiration	Electron donating reactions	Energy-conserving hydrogenase (ferredoxin) Ech	Energy-conserving hydrogenase (ferredoxin), subunit E	0	0.009048	0	0.00747	0	0.002435	0	0.000498	0.000876	0	0	0.004062192
Metabolism of Aromatic Compounds	Peripheral pathways for catabolism of aromatic compounds	n-Phenylalkanoic acid degradation	enoyl-CoA hydratase, R-specific	0	0	0	0	0.000657	0.000974	0	0.000498	0.002408	0.006608	0	0.001932499
Carbohydrates	Sugar alcohols	Ethanolamine utilization	Ethanolamine permease	0	0	0.002555	0.00249	0	0.000487	0	0.001993	0.000657	0	0	0.000907091
Carbohydrates	Sugar alcohols	Ethanolamine utilization	Ethanolamine utilization protein similar to PduT	0.002437	0	0	0	0.000657	0.000487	0	0.000498	0.000219	0	0	0.000433826
Protein Metabolism	Protein biosynthesis	Translation initiation factors eukaryotic and archaeal	Eukaryotic translation initiation factor 2 alpha subunit	0	0	0	0.00498	0.000657	0.002922	0.002166	0	0.002627	0	0	0.001853621

Protein Metabolism	Protein biosynthesis	Translation initiation factors eukaryotic and archaeal	Eukaryotic translation initiation factor 5	0	0	0	0.01245	0	0	0.000722	0.000498	0.000219	0	0.000116	0.000552143
Protein Metabolism	Protein biosynthesis	Translation initiation factors eukaryotic and archaeal	Eukaryotic translation initiation factor 5A	0	0.004524	0	0	0.000657	0	0.000722	0.000498	0.000219	0	0	0.000197194
Protein Metabolism	Protein biosynthesis	Translation initiation factors eukaryotic and archaeal	Eukaryotic translation initiation factor 6	0	0	0	0.00249	0.000657	0	0.002888	0.001495	0.000219	0	0	0.000591581
Iron acquisition and metabolism	NULL	Iron acquisition in Vibrio	Ferric hydroxamate ABC transporter (TC 3.A.1.14.3), permease component FhuB	0	0	0	0	0.000657	0	0.002166	0.001495	0.006568	0	0.000116	0.000354949
Stress Response	Oxidative stress	Regulation of Oxidative Stress Response	ferric uptake regulator	0	0	0.00511	0	0.002626	0.00487	0.001444	0.005482	0	0	0	0.000749336
Fatty Acids, Lipids, and Isoprenoids	Fatty acids	Phospholipid and Fatty acid biosynthesis related cluster	FIG002571: 4-hydroxybenzoyl-CoA thioesterase domain protein	0	0	0.002555	0	0.002626	0	0.000722	0	0.000219	0	0.000815	0.00094653
Clustering-based subsystems	NULL	DNA replication cluster 1	FIG002958: hypothetical protein	0	0.002262	0	0	0.000657	0.001461	0.001444	0	0.000657	0	0	0.001932499
Clustering-based subsystems	Hypothetical Related to Dihydroorate Dehydrogenase	Hypothetical Related to Dihydroorotate dehydrogenase	FIG005495: hypothetical protein	0	0	0	0	0.001313	0.000974	0.004333	0.004984	0.001533	0	0	0.000236633
Clustering-based subsystems	Monosaccharides	Unspecified monosaccharide transport cluster	FIG009210: peptidase, M16 family	0	0	0	0	0.001313	0.000487	0.000722	0	0.000219	0	0.000349	0.000473265
Motility and Chemotaxis	Flagellar motility in Prokaryota	Flagellum	Flagellar protein FlbB	0	0	0	0	0.002626	0.002922	0.000722	0.003489	0.001971	0	0	7.88775E-05
Cofactors, Vitamins, Prosthetic Groups, Pigments	Riboflavin, FMN, FAD	Flavodoxin	Flavodoxin 2	0	0	0	0	0.001313	0.000487	0.002888	0.004984	0.004817	0	0	0.001301479
Membrane Transport	Protein secretion system, Type II	Widespread colonization island	Flp pilus assembly protein CpaD	0.000696	0.004524	0	0.00249	0.000657	0	0	0	0.000219	0	0	0.000197194
Cofactors, Vitamins, Prosthetic Groups, Pigments	Riboflavin, FMN, FAD	Riboflavin, FMN and FAD metabolism	FMN adenylyltransferase, type 3 archaeal (EC 2.7.7.2)	0	0	0	0	0.00197	0.002435	0.000722	0	0.000219	0.002203	0	0.000433826
Respiration	Electron donating reactions	Formate dehydrogenase	Formate dehydrogenase beta subunit (EC 1.2.1.2)	0.002437	0.002262	0.002555	0.01245	0	0.001948	0	0	0	0	0	0.000473265
Respiration	NULL	Formate hydrogenase	Formate dehydrogenase putative subunit (EC 1.2.1.2)	0.001044	0.002262	0.002555	0	0.003283	0	0	0	0.000876	0	0	0.000670459
Carbohydrates	Monosaccharides	Fructose utilization	Fructokinase FrcK (EC 2.7.1.4)	0	0	0	0	0.000657	0.000487	0	0.000997	0.000876	0	0.000116	0.000197194
Clustering-based subsystems	Putrescine/GABA utilization cluster-temporal,to add to SSs	GABA and putrescine metabolism from cluters	GABA aminotransferase (EC 2.6.1.19)	0	0	0.002555	0	0.000657	0	0.000722	0	0.000219	0	0.000349	0.000197194
Clustering-based subsystems	Putrescine/GABA utilization cluster-temporal,to add to SSs	GABA and putrescine metabolism from cluters	Gamma-glutamyl-putrescine oxidase (EC1.4.3.-)	0	0	0	0	0	0.000487	0	0.000498	0.002846	0.002203	0.048553	0.007690557
Miscellaneous	Plant-Prokaryote DOE project	Niacin-Choline transport and metabolism	GbcB Glycine betaine demethylase subunit B	0	0	0	0	0.000657	0	0	0.000498	0.001971	0.008811	0.019212	0.000512704
Carbohydrates	One-carbon Metabolism	Serine-glyoxylate cycle	GDSL-like lipase/acylhydrolase	0	0	0	0	0	0.000487	0.000722	0.000997	0.001095	0	0.000116	0.000867653
Phages, Prophages, Transposable elements, Plasmids	Gene Transfer Agent (GTA)	Gene Transfer Agent	Gene Transfer Agent associated protein Pden 3078	0	0	0	0	0.00197	0.000487	0.002888	0.002492	0.002189	0	0	0.000118316
Phages, Prophages, Transposable elements, Plasmids	Gene Transfer Agent (GTA)	Gene Transfer Agent	Gene Transfer Agent FAD/FMN-containing dehydrogenase	0	0	0	0	0.036111	0.004383	0.005055	0.001495	0.003941	0	0	0.000670459
Stress Response	Osmotic stress	Synthesis of osmoregulated periplasmic glucans	Glucans biosynthesis protein D precursor	0	0	0.002555	0	0	0	0.001444	0.001495	0.003284	0	0.000116	0.001616989
Carbohydrates	Monosaccharides	D-gluconate and ketogluconates metabolism	Gluconate transporter family protein	0.000348	0	0	0.00249	0	0	0.001444	0.000498	0.002189	0	0	0.000473265
Carbohydrates	Monosaccharides	D-gluconate and ketogluconates metabolism	Gluconate utilization system Gnt-I transcriptional repressor	0	0	0	0	0.000657	0	0.000722	0.000997	0.001533	0	0.000931	0.000236633
Carbohydrates	Central carbohydrate metabolism	Peripheral Glucose Catabolism Pathways	Glucose ABC transporter, ATP-binding subunit (EC 3.6.3.-)	0	0	0	0	0.000657	0.000974	0.000722	0.000498	0.001314	0	0	0.000591581
Carbohydrates	Di- and oligosaccharides	Trehalose Uptake and Utilization	Glucose/mannose:H+ symporter GlcP	0.002785	0.002262	0	0.00249	0	0	0	0.001993	0.001533	0	0	0.00126204
Amino Acids and Derivatives	Glutamine, glutamate, aspartate, asparagine; ammonia assimilation	Glutamate and Aspartate uptake in Bacteria	Glutamate-aspartate carrier protein	0	0	0	0.00498	0.000657	0.000487	0	0.000997	0.000438	0	0	0.000354949
Stress Response	Oxidative stress	Glutathione: Biosynthesis and gamma-glutamyl cycle	Glutamate--cysteine ligase (EC 6.3.2.2), divergent, of Alpha- and Beta-proteobacteria type	0	0	0	0.00249	0	0.001948	0.000722	0.000997	0.000657	0	0	0.000985969
Stress Response	Oxidative stress	Glutathione: Non-redox reactions	Glutathione S-transferase, zeta (EC 2.5.1.18)	0	0	0.00511	0	0	0	0	0.000498	0.001095	0.002203	0.001164	0.000276071
Carbohydrates	Sugar alcohols	Glycerol and Glycerol-3-phosphate Uptake and Utilization	Glycerophosphoryl diester phosphodiesterase, periplasmic (EC 3.1.4.46)	0	0	0	0	0	0.003896	0.002166	0.003489	0.004379	0	0.000233	0.010254076
Stress Response	Osmotic stress	Betaine biosynthesis from glycine	Glycine N-methyltransferase (EC 2.1.1.20)	0	0	0.002555	0	0.000657	0.001461	0.001444	0	0.000657	0	0	0.000473265
Carbohydrates	Central carbohydrate metabolism	Glycolate, glyoxylate interconversions	Glycolate permease	0	0.002262	0	0	0.000657	0	0.001444	0.000498	0.000438	0	0	0.001222601
Nucleosides and Nucleotides	Purines	Purine Utilization	Guanine-hypoxanthine permease	0	0.002262	0	0	0.000657	0	0.000722	0	0.000876	0	0.000116	0.001143724
Cofactors, Vitamins, Prosthetic Groups, Pigments	Folate and pterines	5-FCL-like protein	H4MPt-dependent SHMT	0	0.002262	0	0	0.001313	0.000487	0.002166	0.000498	0	0	0	0.000512704
Iron acquisition and metabolism	NULL	Iron Scavenging cluster in Thermus	Heme ABC transporter, ATPase component HmuV	0	0	0	0	0.00197	0.000974	0.002166	0.000997	0.000876	0	0	0.000236633
Cofactors, Vitamins, Prosthetic Groups, Pigments	Tetrapyrroles	Heme and Siroheme Biosynthesis	Hemoprotein HemQ, essential component of heme biosynthetic pathway in Gram-positive bacteria	0	0	0.007664	0.00249	0.000657	0	0	0.000997	0.001314	0	0	0.000907091
Carbohydrates	Monosaccharides	Hexose Phosphate Uptake System	Hexose phosphate uptake regulatory protein UhpC	0	0.004524	0	0	0.001313	0.000974	0.001444	0	0	0	0.000466	0.000276071
Clustering-based subsystems	NULL	CBSS-393121.3.peg.1913	His repressor	0.003133	0	0	0	0.00197	0.001461	0	0	0.002189	0	0.000233	0.000354949
Amino Acids and Derivatives	Arginine; urea cycle, polyamines	Arginine and Ornithine Degradation	Histidine ABC transporter, permease protein HisQ (TC 3.A.1.3.1)	0	0.002262	0	0	0	0.000487	0.001444	0	0.003941	0	0.001397	0.000552143
Amino Acids and Derivatives	Lysine, threonine, methionine, and cysteine	Lysine biosynthesis AAA pathway 2	Homoaconitase large subunit (EC 4.2.1.36)	0	0.015834	0	0	0.001313	0	0.000722	0.003987	0.000876	0	0	0.000985969
Virulence, Disease and Defense	NULL	Streptococcus agalactiae virulome	Hyaluronate lyase	0	0	0	0	0.005909	0.005357	0.006499	0.003489	0	0	0.000233	0.000552143
Respiration	Electron donating reactions	Hydrogenases	hydrogenase/sulfur reductase, gamma subunit	0	0.002262	0.002555	0	0.004596	0.00487	0	0	0.000219	0	0	0.00063102
Respiration	NULL	Formate hydrogenase	Hydrogenase-4 component G (EC 1.-.-.-)	0	0.004524	0.00511	0	0	0.000487	0	0	0.000219	0	0.000116	0.001025408
Miscellaneous	NULL	ZZ gjo need homes	Hydroxylamine oxidoreductase precursor (EC 1.7.3.4)	0	0.002262	0	0	0.000657	0	0.001444	0.000997	0	0.002203	0	0.000118316
Fatty Acids, Lipids, and Isoprenoids	Isoprenoids	Carotenoids	Hydroxyneurosporene dehydrogenase (EC 1.-.-.-)	0	0	0	0	0.000657	0.001948	0.002166	0.00299	0.003065	0	0	0.000197194
Photosynthesis	NULL	Proteorhodopsin	Hydroxyneurosporene synthase	0	0	0	0	0.000657	0.001948	0	0.003489	0.001314	0	0.000116	0.000118316
DNA Metabolism	DNA replication	DNA Helicase of Unknown Function	Hypothetical protein (associated with DNA helicase - Rad25 homolog)	0	0	0	0	0.000657	0	0	0.000498	0.002846	0.002203	0.000815	0.000157755
Clustering-based subsystems	Clustering-based subsystems	CBSS-280355.3.peg.2835	hypothetical protein BH3604	0.001393	0	0	0.00249	0	0	0.001444	0.001993	0.000438	0	0	0.000552143
Nucleosides and Nucleotides	Purines	Purine conversions	IMP dehydrogenase related 2 (EC 1.7.1.7)	0.005571	0.002262	0	0	0.005252	0.000974	0	0	0.000219	0	0	0.000788775
Clustering-based subsystems	NULL	Conjugative transfer related cluster	IncF plasmid conjugative transfer protein TraG	0.002785	0.009048	0	0	0	0	0.000722	0.000498	0.000219	0	0	0.001340918
Carbohydrates	Di- and oligosaccharides	Maltose and Maltodextrin Utilization	Inner membrane ABC transporter permease protein YcjO	0	0	0.002555	0	0.00197	0	0.000722	0.000498	0.000657	0	0	0.000867653
Phages, Prophages, Transposable elements, Plasmids	Plasmid related functions	Plasmid-encoded T-DNA transfer	Inner membrane protein of type IV secretion of T-DNA complex, VirB6	0.002785	0	0	0	0.000657	0	0.000722	0	0.000657	0	0.000349	0.000788775
Carbohydrates	Sugar alcohols	Inositol catabolism	Inositol transport system sugar-binding protein	0	0	0	0	0.000657	0.003896	0.000722	0.000997	0.001095	0	0	0.00063102
Clustering-based subsystems	Cytochrome biogenesis	CBSS-196164.1.peg.1690	Iron-sulfur cluster regulator SufR	0	0.002262	0	0	0	0.000487	0	0.000498	0.001533	0	0.000233	0.000197194

Iron acquisition and metabolism	NULL	Transport of Iron	Iron-uptake factor PiuB	0	0.002262	0	0	0	0.000487	0	0.001495	0.006131	0	0.000116	0.003431172
Potassium metabolism	NULL	Potassium homeostasis	Kef-type transport system 2 (probable substrate potassium), subunit 1	0	0.004524	0	0	0	0.000487	0.000722	0	0.000657	0	0.000116	0.000394388
Carbohydrates	Organic acids	Lactate utilization	Lactate-responsive regulator LldR in Firmicutes, GntR family	0	0.002262	0	0	0.000657	0.002435	0	0	0.001095	0.002203	0	0.000709898
Photosynthesis	Electron transport and photophosphorylation	Photosystem II-type photosynthetic reaction center	Light-harvesting LHI, alpha subunit	0	0	0.002555	0	0	0.001948	0.000722	0.004485	0.005912	0	0	0.000197194
Photosynthesis	Electron transport and photophosphorylation	Photosystem II-type photosynthetic reaction center	Light-harvesting LHI, beta subunit	0	0.004524	0	0	0	0.001948	0.001444	0.00598	0.000438	0	0	0.000197194
Metabolism of Aromatic Compounds	Peripheral pathways for catabolism of aromatic compounds	Phenylpropanoid compound degradation	lignostilbene-alpha,beta-dioxygenase	0	0	0.002555	0	0.000657	0	0.000722	0	0.000876	0	0.000233	0.000197194
Cell Wall and Capsule	Gram-Negative cell wall components	KDO2-Lipid A biosynthesis	Lipid A biosynthesis (KDO) 2-(lauroyl)-lipid IVA acyltransferase (EC 2.3.1.-)	0	0	0.002555	0	0	0.000974	0.002166	0.001495	0.001095	0	0	0.001183163
Fatty Acids, Lipids, and Isoprenoids	Fatty acids	Fatty acid metabolism cluster	Long-chain-acyl-CoA dehydrogenase (EC 1.3.99.13)	0	0	0.002555	0	0.001313	0	0.002166	0.001495	0.002408	0	0	0.002997345
Amino Acids and Derivatives	Proline and 4-hydroxyproline	Proline, 4-hydroxyproline uptake and utilization	L-Proline/Glycine betaine transporter ProP	0	0	0.002555	0	0	0.001948	0.002166	0.001495	0.007663	0	0	0.00157755
Cell Wall and Capsule	Gram-Negative cell wall components	KDO2-Lipid A biosynthesis	LPS-assembly lipoprotein RlpB precursor (Rare lipoprotein B)	0	0	0	0.00249	0	0.000487	0	0.001993	0.003722	0.002203	0	0.000354949
Carbohydrates	Central carbohydrate metabolism	Pyruvate Alanine Serine Interconversions	L-serine dehydratase, beta subunit (EC 4.3.1.17)	0	0	0	0	0	0.000974	0.000722	0.000498	0.000219	0	0.000233	0.000749336
Protein Metabolism	Protein biosynthesis	Ribosome LSU eukaryotic and archaeal	LSU ribosomal protein L11e (L5p)	0	0	0	0	0.001313	0.001461	0.000722	0.002492	0	0	0.000233	0.001459234
Protein Metabolism	Protein biosynthesis	Ribosome LSU eukaryotic and archaeal	LSU ribosomal protein L13Ae (L13p)	0	0	0	0.00249	0.001313	0.001461	0	0.001495	0.000438	0	0	0.00063102
Protein Metabolism	Protein biosynthesis	Ribosome LSU chloroplast	LSU ribosomal protein L16p (L10e), chloroplast	0	0	0.002555	0.00249	0	0	0.000722	0.000498	0.001095	0	0	7.88775E-05
Protein Metabolism	Protein biosynthesis	Ribosome LSU eukaryotic and archaeal	LSU ribosomal protein L17e (L22p)	0	0.002262	0	0	0	0.000487	0	0.000997	0.000657	0	0.000116	0.000512704
Protein Metabolism	Protein biosynthesis	Ribosome LSU eukaryotic and archaeal	LSU ribosomal protein L18e	0	0	0	0.00249	0	0.001461	0.000722	0.000997	0.001095	0	0	0.00031551
Protein Metabolism	Protein biosynthesis	Ribosome LSU eukaryotic and archaeal	LSU ribosomal protein L26e (L24p)	0	0	0	0.00498	0.00197	0.000974	0.001444	0	0.000876	0	0	0.000473265
Protein Metabolism	Protein biosynthesis	Ribosome LSU eukaryotic and archaeal	LSU ribosomal protein L32e	0	0.002262	0	0.00498	0.001313	0.000974	0	0	0.000219	0	0	0.000512704
Protein Metabolism	Protein biosynthesis	Ribosome LSU eukaryotic and archaeal	LSU ribosomal protein L5e (L18p)	0	0.002262	0	0.00249	0.000657	0	0	0	0.001095	0.002203	0	0.001222601
Amino Acids and Derivatives	Lysine, threonine, methionine, and cysteine	Lysine degradation	Lysine decarboxylase (EC 4.1.1.18)	0	0	0	0.00498	0	0.000487	0.001444	0	0.003941	0.002203	0	0.002326887
Virulence, Disease and Defense	Bacteriocins, ribosomally synthesized antibacterial peptides	Marinocine, a broad-spectrum antibacterial protein	Lysine-epsilon oxidase (EC 1.4.3.20) antimicrobial protein LodA	0	0	0	0	0.000657	0.001461	0.000722	0.001495	0.002408	0	0	0.000394388
Metabolism of Aromatic Compounds	Metabolism of central aromatic intermediates	Salicylate and gentisate catabolism	Maleate cis-trans isomerase (EC 5.2.1.1)	0	0	0	0	0.001313	0.000487	0.000722	0.004485	0.001095	0	0	0.000276071
Carbohydrates	Organic acids	Malonate decarboxylase	Malonate transporter, MadL subunit	0.002437	0	0	0	0	0.000974	0.002166	0.000997	0.000876	0	0	0.000236633
Carbohydrates	Di- and oligosaccharides	Maltose and Maltodextrin Utilization	Maltose operon transcriptional repressor MalR, LacI family	0	0	0	0.00249	0.00197	0.001948	0	0.000997	0.002627	0	0	0.001340918
Carbohydrates	Monosaccharides	Mannose Metabolism	Mannoside ABC transport system, ATP-binding protein 2	0	0.006786	0	0	0.001313	0.000974	0.001444	0	0.000438	0	0	0.001183163
Clustering-based subsystems	Clustering-based subsystems	CBSS-280355.3.peg.2835	MazG nucleotide pyrophosphohydrolase	0	0	0	0	0	0.000487	0.002166	0.001495	0.001095	0	0.000349	0.000473265
Phages, Prophages, Transposable elements, Plasmids	Pathogenicity islands	Staphylococcal pathogenicity islands SaPI	Methionine ABC transporter substrate-binding protein	0	0	0	0	0	0	0.001444	0.000997	0.003284	0.004406	0.000815	0.000709898
Protein Metabolism	Protein biosynthesis	tRNA aminoacylation, Met	Methionyl-tRNA synthetase-related protein 2	0	0	0	0	0.000657	0.000487	0.002166	0.000498	0.000219	0	0	0.000197194
Carbohydrates	One-carbon Metabolism	Methanogenesis from methylated compounds	Methylthiol:coenzyme M methyltransferase	0	0	0.012774	0	0.00197	0.001461	0	0.000498	0.000438	0	0	0.001695866
Protein Metabolism	Protein degradation	Metalloendopeptidases (EC 3.4.24.-)	Microbial collagenase (EC 3.4.24.3)	0.002785	0	0	0	0.00197	0.002435	0.001444	0	0.000438	0	0	0.001459234
Amino Acids and Derivatives	Proline and 4-hydroxyproline	Proline, 4-hydroxyproline uptake and utilization	Microbial collagenase, secreted (EC 3.4.24.3)	0	0	0	0	0.001313	0.001461	0.001444	0.001495	0.003284	0	0	0.004732651
Clustering-based subsystems	NULL	Allophanate hydrolase 2 and Biotin carboxylase cluster	Mn2+/Fe2+ transporter, NRAMP family	0	0	0	0.00249	0.000657	0	0	0.000498	0.001095	0.013217	0	0.000907091
Secondary Metabolism	Plant Hormones	Auxin biosynthesis	Monoamine oxidase (1.4.3.4)	0	0	0.00511	0	0.000657	0.000487	0	0.000997	0.002846	0	0	0.000512704
Virulence, Disease and Defense	Resistance to antibiotics and toxic compounds	Multidrug Resistance Efflux Pumps	Multidrug efflux RND transporter MexD	0.003133	0	0.002555	0	0	0	0.001444	0	0.001314	0	0.004541	0.001301479
Virulence, Disease and Defense	Resistance to antibiotics and toxic compounds	Multidrug Resistance Efflux Pumps	Multidrug-efflux transporter, major facilitator superfamily (MFS) (TC 2.A.1)	0.004526	0.002262	0	0	0	0.001948	0.000722	0	0.000438	0	0	0.001932499
DNA Metabolism	DNA repair	DNA repair, bacterial MutL-MutS system	MutS domain protein, family 5	0.002437	0.002262	0	0	0.000657	0.000487	0	0	0.000438	0	0	0.000394388
Carbohydrates	One-carbon Metabolism	Methanogenesis	N5-methyltetrahydromethanopterin:coenzyme M methyltransferase subunit A (EC 2.1.1.86)	0	0	0	0.00249	0.000657	0.000974	0	0	0.000657	0	0.000116	0.000276071
Carbohydrates	Aminosugars	Chitin and N-acetylglucosamine utilization	N-acetylglucosamine-regulated TonB-dependent outer membrane receptor	0.002437	0	0	0	0	0.000974	0	0.000498	0.001533	0	0.001514	0.000749336
Cofactors, Vitamins, Prosthetic Groups, Pigments	Quinone cofactors	Ubiquinone Biosynthesis	NAD(P)H-flavin reductase (EC 1.5.1.29) (EC 1.16.1.3)	0	0	0	0	0	0.000487	0	0.001495	0.004817	0.002203	0.000116	0.00063102
Respiration	Electron donating reactions	Respiratory Complex I	NADH dehydrogenase I subunit 4, Involved in photosystem-1 cyclic electron flow	0	0	0	0	0.001313	0.002435	0.006499	0	0.004379	0	0.002329	0.000867653
Respiration	Electron donating reactions	Respiratory Complex I	NADH dehydrogenase subunit 1	0.001044	0	0	0	0	0	0.000722	0.000498	0.000219	0	0.000233	0.000157755
Respiration	Electron donating reactions	Respiratory Complex I	NADH dehydrogenase subunit 4 (EC:1.6.99.3)	0	0	0	0	0.001313	0.001461	0	0	0.001314	0.002203	0.003493	0.004890406
Carbohydrates	CO2 fixation	CO2 uptake, carboxysome	NADH dehydrogenase subunit 4, Involved in CO2 fixation	0	0	0	0	0.003283	0.001461	0.000722	0	0.003284	0	0.001164	0.000197194
Respiration	Electron donating reactions	Hydrogenases	NADP-reducing hydrogenase, subunit A	0	0	0	0.00996	0.000657	0.002435	0.002888	0.000997	0	0	0	0.002129693
Membrane Transport	NULL	Transport of Nickel and Cobalt	Nickel transport system permease protein NikB (TC 3.A.1.5.3)	0.000348	0.002262	0	0	0.000657	0	0	0.000997	0.001095	0	0	0.001183163
Membrane Transport	NULL	Transport of Nickel and Cobalt	Nickel transport system permease protein NikC (TC 3.A.1.5.3)	0	0	0	0	0.000657	0.001948	0.00361	0.001993	0.000657	0	0	0.000433826
Cofactors, Vitamins, Prosthetic Groups, Pigments	NAD and NADP	NAD and NADP cofactor biosynthesis global	Nicotinamidase family protein YcaC	0	0	0	0	0	0.001461	0.000722	0.001495	0.002846	0	0.000582	0.000867653
Nitrogen Metabolism	NULL	Nitrogen fixation	NifT protein	0	0	0.002555	0	0.001313	0	0.000722	0.000997	0.001095	0	0	0.000788775
Nitrogen Metabolism	NULL	Nitrogen fixation	NifZ protein	0	0	0.002555	0	0.007879	0.002435	0	0.001495	0.000438	0	0	0.002366325
Nitrogen Metabolism	NULL	Denitrification	Nitric oxide -responding transcriptional regulator Dnr (Crp/Fnr family)	0	0	0.002555	0.00747	0.001313	0	0.000722	0	0.000876	0	0	0.001538111
Nitrogen Metabolism	NULL	Denitrification	Nitrous oxide reductase maturation protein, outer-membrane lipoprotein NosL	0	0	0	0	0	0.000487	0.001444	0.000997	0.000219	0	0.000931	0.000985969
Protein Metabolism	Protein processing and modification	N-linked Glycosylation in Bacteria	N-linked glycosylation glycosyltransferase PglG	0.002785	0	0	0	0.00197	0.002435	0	0.001993	0.000657	0	0	0.002681835
Iron acquisition and metabolism	Siderophores	Siderophore Pyoverdine	Non-ribosomal peptide synthetase modules, pyoverdine??	0	0	0	0.00249	0	0.000974	0.000722	0.006479	0.003065	0	0	0.000512704
Nucleosides and Nucleotides	Detoxification	Housecleaning nucleoside triphosphate pyrophosphatases	Nucleotidase YfbR, HD superfamily	0	0	0	0.00249	0	0.000974	0.000722	0.001495	0.003065	0	0	0.000788775
Protein Metabolism	Protein degradation	Proteolysis in bacteria, ATP-dependent	Nucleotide excision repair protein, with UvrB/UvrC motif	0	0.002262	0	0	0.003283	0	0.001444	0.00299	0.000438	0	0	0.000473265
Cofactors, Vitamins, Prosthetic Groups, Pigments	NAD and NADP	NAD regulation	Nudix-related transcriptional regulator NrtR	0.000348	0	0	0	0	0.004383	0.002166	0	0.00416	0	0.000699	0.002602958

Cell Wall and Capsule	Gram-Negative cell wall components	Perosamine Synthesis Vibrio	O-antigen export system, ATP-binding protein	0	0	0	0	0.000657	0.001461	0.002888	0.004984	0.002846	0	0	0.001853621
Secondary Metabolism	Plant Octadecanoids	Octadecanoids	Oxidase	0	0	0	0	0.000657	0	0.000722	0.000498	0.000438	0	0.001747	0.002563519
Cell Division and Cell Cycle	NULL	YgjD and YeaZ	p53-regulating protein kinase (human PRPK/yeast YGR262c)	0	0	0	0.00498	0.001313	0	0.001444	0.000997	0.000438	0	0	0.000670459
Protein Metabolism	Protein biosynthesis	Translation termination factors bacterial	Peptide chain release factor homolog	0.000696	0	0	0	0.001313	0	0	0.001993	0.000219	0	0.000116	0.000157755
Cell Wall and Capsule	Capsular and extracellular polysacchrides	Polysaccharide deacetylases	Peptidoglycan N-acetylglucosamine deacetylase (EC 3.5.1.-)	0	0.002262	0	0.00498	0.000657	0.002922	0	0	0.000219	0	0	0.000473265
Miscellaneous	NULL	ZZ gjo need homes	Peptidyl-tRNA hydrolase, archaeal type (EC 3.1.1.29)	0	0	0	0	0.004596	0.002435	0.00361	0.001993	0.000876	0	0	0.001025408
Respiration	Electron accepting reactions	Fe(III) respiration - Shewanella type	periplasmic decaheme cytochrome c, MtrA	0	0	0.002555	0	0	0.000487	0.000722	0.000498	0.000438	0	0	0.00063102
Cell Wall and Capsule	Gram-Negative cell wall components	Perosamine Synthesis Vibrio	Perosamine synthetase	0.002437	0	0	0	0.00197	0.001948	0	0	0.002627	0.004406	0	0.002760713
Phages, Prophages, Transposable elements, Plasmids	Phages, Prophages	Phage regulation of gene expression	Phage antirepressor protein	0.002089	0	0	0	0	0.000487	0.000722	0.000997	0.000219	0	0	0.000394388
Phages, Prophages, Transposable elements, Plasmids	Phages, Prophages	Phage integration and excision	Phage endonuclease	0	0	0	0	0.009848	0.013637	0.005055	0.006977	0	0	0.000349	0.000276071
Phages, Prophages, Transposable elements, Plasmids	Phages, Prophages	Phage integration and excision	Phage exonuclease	0	0	0	0	0.004596	0.015098	0.015164	0.024918	0.000657	0	0	0.000433826
Phages, Prophages, Transposable elements, Plasmids	Phages, Prophages	Phage entry and exit	Phage lysozyme	0	0.002262	0	0	0.000657	0.001461	0.002888	0.001495	0	0	0	0.000236633
Phages, Prophages, Transposable elements, Plasmids	Phages, Prophages	Phage capsid proteins	Phage prohead core scaffold protein and protease	0	0	0	0	0.000657	0.001948	0.000722	0.003489	0.000219	0	0	0.000197194
Phages, Prophages, Transposable elements, Plasmids	Phages, Prophages	Phage tail proteins 2	Phage tail length tape-measure protein	0	0	0	0	0.019697	0.017533	0.015886	0.029403	0.001752	0	0	0.003943875
Phages, Prophages, Transposable elements, Plasmids	Phages, Prophages	Phage tail proteins 2	Phage tail sheath monomer	0	0	0	0	0.003939	0.003409	0.010831	0.011462	0.001533	0	0	0.001616989
Phages, Prophages, Transposable elements, Plasmids	Phages, Prophages	Phage tail proteins 2	Phage tape measure	0	0	0	0.00249	0	0.002435	0.005055	0.00299	0	0	0.000233	0.000276071
Phages, Prophages, Transposable elements, Plasmids	Phages, Prophages	Phage packaging machinery	Phage terminase, small subunit	0	0	0	0	0.003283	0.00487	0.005777	0.011462	0.000219	0	0	0.000197194
Metabolism of Aromatic Compounds	Peripheral pathways for catabolism of aromatic compounds	Phenol hydroxylase	Phenol hydroxylase, P3 oxygenase component DmpN (EC 1.14.13.7)	0	0	0.00511	0	0	0.000487	0	0.001495	0.004379	0	0.072189	0.006113007
Cell Wall and Capsule	Cell wall of Mycobacteria	mycolic acid synthesis	Phenolpthiocerol synthesis polyketide synthase ppsA	0	0	0.00511	0	0.000657	0.000487	0	0.001495	0.000219	0	0	0.000354949
Metabolism of Aromatic Compounds	NULL	Aromatic Amin Catabolism	Phenylacetaldehyde dehydrogenase (EC 1.2.1.39)	0	0	0	0	0	0.000487	0.000722	0.000997	0.003065	0	0.000466	0.000473265
Carbohydrates	Central carbohydrate metabolism	Pyruvate metabolism I: anaplerotic reactions, PEP	Phosphoenolpyruvate carboxylase, archaeal (EC 4.1.1.31)	0	0.009048	0.002555	0	0.002626	0.001461	0	0.001495	0	0	0	0.000433826
Phosphorus Metabolism	NULL	Phosphonate metabolism	Phosphonoacetate hydrolase (EC 3.11.1.2)	0	0	0.012774	0	0	0.001948	0.002888	0.003987	0.006568	0	0	0.001419795
Membrane Transport	Protein secretion system, Type VI	Type VI secretion systems	Phosphoprotein phosphatase PppA	0.001044	0	0	0.00249	0	0.001948	0.000722	0	0.001095	0	0	0.00126204
Metabolism of Aromatic Compounds	NULL	Benzoate transport and degradation cluster	P-hydroxylaminobenzoate lyase	0	0.002262	0	0	0.000657	0	0.000722	0.000498	0.001533	0	0	0.000236633
Miscellaneous	Plant-Prokaryote DOE project	COG1836	phytol kinase	0	0.002262	0	0	0.001313	0.000974	0	0.000498	0.001971	0	0	0.000709898
Virulence, Disease and Defense	Resistance to antibiotics and toxic compounds	Polymyxin Synthetase Gene Cluster in Bacillus	Polymyxin synthetase PmxA	0	0.002262	0	0	0	0.000487	0.001444	0.000498	0.001752	0	0	0.000118316
Cell Wall and Capsule	Capsular and extracellular polysacchrides	Colanic acid biosynthesis	Polysaccharide export lipoprotein Wza	0	0	0	0	0.000657	0.000487	0	0.000997	0.004817	0	0.001514	0.001380356
Clustering-based subsystems	Probably Ybbk-related hypothetical membrane proteins	CBSS-316057.3.peg.659	Polysialic acid capsule sugar isomerase, KpsF/GutQ family	0	0	0.00511	0	0	0	0.000722	0.001495	0.000876	0	0.000116	0.00063102
Metabolism of Aromatic Compounds	Peripheral pathways for catabolism of aromatic compounds	Phenol hydroxylase	Positive regulator of phenol hydroxylase	0	0	0	0.00249	0	0	0.005055	0.000498	0.003503	0	0.10025	0.006191885
Miscellaneous	Plant-Prokaryote DOE project	At1g26220 At1g32070	Possible alpha/beta hydrolase superfamily, slr1916 homolog	0	0.002262	0	0	0	0.002435	0	0.000498	0.001533	0	0.000931	0.000197194
Cofactors, Vitamins, Prosthetic Groups, Pigments	Folate and pterines	Folate Biosynthesis	PqqC-like protein	0	0	0.002555	0	0.000657	0	0.001444	0.000498	0.000219	0	0	0.000197194
Carbohydrates	Di- and oligosaccharides	Beta-Glucoside Metabolism	Predicted beta-glucoside-regulated ABC transport system, permease component 1, COG1175	0	0.002262	0.002555	0.00249	0	0	0	0.000498	0.000438	0	0	0.000552143
Cofactors, Vitamins, Prosthetic Groups, Pigments	Tetrapyrroles	Coenzyme B12 biosynthesis	Predicted cobalt transporter in sulfate-reducing delta-proteobacteria	0	0	0.002555	0	0.004596	0.00487	0.002166	0.001495	0	0	0	0.001419795
Carbohydrates	Monosaccharides	D-galactarate, D-glucarate and D-glycerate catabolism	Predicted D-glucarate or D-galactorate regulator, GntR family	0.000696	0	0	0	0	0.000487	0.000722	0.000498	0.000438	0	0	0.000197194
Carbohydrates	NULL	Sugar utilization in Thermotogales	Predicted keto-inosose hydrolase	0.008008	0.006786	0.00511	0.00996	0.000657	0	0	0	0	0	0	0.001459234
Carbohydrates	Monosaccharides	L-Arabinose utilization	Predicted L-arabinose ABC transport system, ATP-binding protein	0	0.009048	0.002555	0	0	0.000487	0.001444	0	0.001314	0	0	0.000709898
Carbohydrates	Organic acids	Lactate utilization	Predicted L-lactate dehydrogenase, hypothetical protein subunit SO1518	0	0	0	0	0.000657	0.000974	0	0.001993	0.000657	0	0.000116	0.00063102
Clustering-based subsystems	Clustering-based subsystems	Putative diaminopropionate ammonia-lyase cluster	Predicted oxidoreductase, Fe-S subunit	0.004874	0.004524	0	0	0	0.001461	0.001444	0.000498	0	0	0	0.00126204
Carbohydrates	Monosaccharides	L-rhamnose utilization	Predicted rhamnose oligosaccharide ABC transport system, permease component 2	0.003133	0.002262	0	0	0.00197	0	0.000722	0	0.000219	0	0	0.000512704
Carbohydrates	Monosaccharides	L-rhamnose utilization	Predicted rhamnose oligosaccharide ABC transport system, substrate-binding component	0	0.006786	0	0.00498	0	0.000974	0.00361	0	0.00416	0	0	0.002918468
RNA Metabolism	RNA processing and modification	tRNA modification Archaea	Predicted RNA methylase COG2263	0	0	0	0	0.000657	0.000487	0.000722	0.000997	0.000657	0	0	0.000354949
Membrane Transport	Protein secretion system, Type II	Predicted secretion system W clustering with cell division proteins	Predicted secretion system W protein GspG-like	0	0	0	0.00498	0.001313	0.000974	0.000722	0	0.000657	0	0	0.00031551
Carbohydrates	NULL	Sugar utilization in Thermotogales	Predicted trehalose ABC transporter, permease component 2, Archaea-type	0.000696	0	0	0.00498	0	0.000974	0	0.000997	0.000657	0	0	0.000512704
DNA Metabolism	DNA replication	DNA-replication	Primosomal protein I	0	0	0	0	0.004596	0.001461	0.012275	0.006479	0.000219	0	0	0.000118316
Carbohydrates	Monosaccharides	L-ascorbate utilization (and related gene clusters)	Probable L-ascorbate-6-phosphate lactonase UlaG (EC 3.1.1.-) (L-ascorbate utilization protein G)	0.080773	0	0.002555	0	0.000657	0.000974	0	0	0	0	0.000116	0.000473265
Protein Metabolism	Protein biosynthesis	Pyrrolysine	Proline 2-methylase for pyrrolysine biosynthesis	0.001044	0	0.002555	0	0.002626	0.002922	0.001444	0	0	0	0	0.000788775
Stress Response	NULL	Dimethylarginine metabolism	Protein arginine N-methyltransferase 1 (EC 2.1.1.-)	0	0	0	0	0.000657	0.000487	0.000722	0	0.000438	0.002203	0	0.000118316
Clustering-based subsystems	NULL	CBSS-393133.3.peg.2787	protein from nitrogen regulatory protein P-II (GLNB) family, ortholog YAAQ B. subtilis	0.002437	0.002262	0.002555	0.00249	0	0	0	0	0.000219	0	0	0.000749336
Clustering-based subsystems	Shiga toxin cluster	CBSS-194948.1.peg.143	Protein NinG	0	0	0	0	0	0.005357	0.007943	0.012957	0.000219	0	0.000233	0.000118316
RNA Metabolism	RNA processing and modification	RNA 3'-terminal phosphate cyclase	Protein with similarity to RtcB	0	0	0	0.00498	0.000657	0	0.001444	0.000997	0	0	0.000466	0.000709898
Clustering-based subsystems	NULL	CBSS-562.2.peg.5158 SK3 including	Protein YaiI	0	0	0	0.00498	0	0	0.000722	0	0.002627	0.002203	0.002911	0.00220857
Cofactors, Vitamins, Prosthetic Groups, Pigments	Quinone cofactors	Ubiquinone Biosynthesis	Protein YigP (COG3165) clustered with ubiquinone biosynthetic genes	0	0	0	0.00498	0	0.000487	0	0.001993	0.004817	0	0.002096	0.000788775
Fatty Acids, Lipids, and Isoprenoids	Isoprenoids	Carotenoids	Pro-zeta-carotene desaturase, prolycopene producing (EC 1.-.-.-)	0	0	0	0	0.002626	0.000487	0	0.000997	0.006568	0	0.000233	3.94388E-05
Carbohydrates	Sugar alcohols	Mannitol Utilization	PTS system, mannitol-specific IIA component (EC 2.7.1.69)	0	0	0	0.00747	0.00197	0	0	0.000997	0.000219	0	0.000233	0.000985969
Carbohydrates	Aminosugars	Chitin and N-acetylglucosamine utilization	PTS system, N-acetylglucosamine-specific IIC component (EC 2.7.1.69)	0.00383	0	0	0	0	0	0	0.000498	0.000657	0.019825	0.000582	0.000197194

Nucleosides and Nucleotides	Purines	Purine nucleotide synthesis regulator	Purine nucleotide synthesis repressor	0	0	0	0.00747	0.001313	0.000487	0.001444	0	0.001533	0	0	0.000236633
Carbohydrates	CO2 fixation	CO2 uptake, carboxysome	putative carboxysome peptide B	0.002785	0	0	0	0.003283	0.000487	0.002166	0.000997	0	0	0	0.001498673
Cell Wall and Capsule	Gram-Positive cell wall components	Teichoic and lipoteichoic acids biosynthesis	Putative CDP-glycerol:glycerophosphate glycerophosphotransferase (EC 2.7.8.-)	0	0.002262	0	0	0.001313	0	0.000722	0.000997	0	0	0.000116	0.00094653
Carbohydrates	Di- and oligosaccharides	Trehalose Uptake and Utilization	Putative cytoplasmic protein clustered with trehalase	0	0.002262	0	0	0.000657	0.000487	0	0.000498	0.002189	0	0	0.001340918
Clustering-based subsystems	Biosynthesis of galactoglycans and related lipopolysacharides	CBSS-258594.1.peg.3339	putative glycosyltransferase protein	0.000348	0	0.002555	0	0	0	0.002166	0.001495	0.001533	0	0	0.000236633
Regulation and Cell signaling	NULL	cAMP signaling in bacteria	Putative Heme-regulated two-component response regulator	0	0.002262	0	0	0	0.000487	0.001444	0.001993	0.001095	0	0	0.002366325
Iron acquisition and metabolism	NULL	Campylobacter Iron Metabolism	Putative high-affinity iron permease	0	0	0.002555	0	0.000657	0	0.000722	0	0.000657	0	0.000233	0.000433826
Stress Response	NULL	Hfl operon	Putative inner membrane protein YjeT (clustered with HflC)	0	0	0	0	0.000657	0.000974	0.001444	0.000498	0.000876	0	0	0.000907091
Membrane Transport	Protein secretion system, Type V	Two partner secretion pathway (TPS)	Putative large exoprotein involved in heme utilization or adhesion of ShlA/HecA/FhaA family	0	0	0	0	0.001313	0.001461	0.001444	0.000498	0.001095	0	0	0.002405764
Clustering-based subsystems	Lysine, threonine, methionine, and cysteine	YeiH	Putative membrane protein YeiH	0.004178	0	0	0.00249	0	0	0.004333	0	0.003065	0	0.001863	0.008715965
Amino Acids and Derivatives	Proline and 4-hydroxyproline	Proline, 4-hydroxyproline uptake and utilization	Putative oxidoreductase in 4-hydroxyproline catabolic gene cluster	0	0	0.002555	0.00249	0.000657	0.001461	0	0	0.000219	0	0	0.000552143
DNA Metabolism	DNA repair	2-phosphoglycolate salvage	Putative phosphatase YqaB	0.038646	0	0	0	0.001313	0	0	0.000498	0.000438	0	0.000233	0.002484642
Virulence, Disease and Defense	Resistance to antibiotics and toxic compounds	Cobalt-zinc-cadmium resistance	Putative silver efflux pump	0	0.002262	0	0	0	0.000974	0	0.000498	0.000876	0.002203	0	0.000394388
Clustering-based subsystems	Carbohydrates	Putative sugar ABC transporter (ytf cluster)	Putative sugar ABC transport system, permease protein YtfT	0	0.004524	0	0.00249	0.001313	0.000487	0	0	0.000438	0	0	0.001814183
Cell Wall and Capsule	Capsular and extracellular polysacchrides	O-Methyl Phosphoramidate Capsule Modification in Campylobacter	Putative sugar nucleotidyltransferase	0	0	0	0	0.000657	0.000487	0.000722	0.000997	0.000438	0	0	0.003115662
Amino Acids and Derivatives	Arginine; urea cycle, polyamines	Polyamine Metabolism	Putrescine carbamoyltransferase (EC 2.1.3.6)	0	0	0	0.00249	0.001313	0	0	0.000498	0	0.004406	0.000116	0.000512704
Iron acquisition and metabolism	Siderophores	Siderophore Pyoverdine	Pyoverdine sidechain non-ribosomal peptide synthetase PvdD	0	0	0	0	0.002626	0.002922	0.012998	0.005482	0.005036	0	0	0.002248009
Iron acquisition and metabolism	Siderophores	Siderophore Pyoverdine	Pyoverdine sidechain non-ribosomal peptide synthetase PvdI	0	0	0.002555	0	0	0.000974	0.007221	0.001993	0.001752	0	0	0.001380356
Phosphorus Metabolism	NULL	Phosphate metabolism	Pyrophosphate-specific outer membrane porin OprO	0	0	0	0.00498	0.000657	0	0.000722	0.000498	0.000438	0	0	0.000394388
Protein Metabolism	Protein degradation	Omega peptidases (EC 3.4.19.-)	Pyrrolidone-carboxylate peptidase (EC 3.4.19.3)	0	0	0.002555	0	0.00197	0.000487	0	0.000498	0.001752	0	0	0.00157755
Respiration	Electron donating reactions	Hydrogenases	Quinone-reactive Ni/Fe-hydrogenase small chain precursor (EC 1.12.5.1)	0	0.01131	0	0	0.003939	0.001461	0.001444	0	0.001752	0	0	0.007375047
Phages, Prophages, Transposable elements, Plasmids	Phages, Prophages	r1t-like streptococcal phages	Recombinational DNA repair protein RecT (prophage associated)	0	0	0	0	0.006566	0.007793	0.016608	0.033889	0.000219	0	0	0.001183163
Stress Response	Oxidative stress	Oxidative stress	Regulatory protein SoxS	0.002785	0	0	0	0	0.001461	0.002166	0.003987	0.000876	0	0	3.94388E-05
DNA Metabolism	DNA replication	DNA replication, archaeal	Replication factor A (ssDNA-binding protein)	0	0	0	0.00249	0.000657	0	0.001444	0.001495	0.001533	0	0	7.88775E-05
Motility and Chemotaxis	Social motility and nonflagellar swimming in bacteria	Rhamnolipids in Pseudomonas	Rhamnolipids biosynthesis 3-oxoacyl-[acyl-carrier-protein] reductase RhlG (EC 1.1.1.100)	0	0	0.012774	0.00249	0	0.001461	0	0.001495	0.001095	0	0	0.000591581
Cofactors, Vitamins, Prosthetic Groups, Pigments	Riboflavin, FMN, FAD	Riboflavin, FMN and FAD metabolism	Riboflavin synthase archaeal (EC 2.5.1.9)	0	0.006786	0	0	0.001313	0.001461	0.000722	0	0	0	0.000349	0.00063102
Clustering-based subsystems	Recombination related cluster	CBSS-198094.1.peg.4426	Ribonuclease HIII (EC 3.1.26.4)	0	0	0	0	0	0.000974	0.001444	0.00299	0.001752	0	0.000116	0.000552143
RNA Metabolism	RNA processing and modification	Ribonuclease H	Ribonuclease HI-related protein	0.000348	0	0	0	0.000657	0	0	0.000498	0.000657	0.011014	0	0.002129693
Protein Metabolism	Protein biosynthesis	Ribosome biogenesis bacterial	Ribosomal-protein-L7p-serine acetyltransferase	0	0	0	0.00249	0	0.002435	0.004333	0.000997	0	0.004406	0	0.001380356
Clustering-based subsystems	Putative asociate of RNA polymerase sigma-54 factor rpoN	CBSS-316057.3.peg.1308	RNA polymerase sigma-70 factor, ECF family	0	0.002262	0	0	0.001313	0.002922	0	0.001993	0.000657	0	0	0.000276071
Miscellaneous	Plant-Prokaryote DOE project	Conserved gene cluster possibly involved in RNA metabolism	RNA polymerase sporulation specific sigma factor SigH	0.027853	0.002262	0.00511	0	0	0	0	0.001495	0.000657	0	0	7.88775E-05
Stress Response	Oxidative stress	Glutathione: Non-redox reactions	SAM-dependent methyltransferase 2, in cluster with Hydroxyacylglutathione hydrolase (EC 3.1.2.6)	0	0	0	0	0.000657	0.000974	0.002166	0.001495	0.001314	0	0	0.000157755
Membrane Transport	Protein secretion system, Type VI	Type VI secretion systems	Secreted protein Hcp	0	0.002262	0.00511	0	0	0.000974	0.001444	0	0	0	0.028992	0.000867653
Regulation and Cell signaling	Regulation of virulence	Two-component Response Regulator of Virulence ResDE	Sensor histidine kinase ResE (EC 2.7.3.-)	0	0.004524	0.002555	0	0.000657	0.001461	0	0	0.001533	0	0	0.001183163
Regulation and Cell signaling	NULL	Orphan regulatory proteins	Sensory histidine kinase AtoS	0	0	0	0	0.003283	0.002435	0.000722	0.000498	0.000438	0	0	0.004732651
Miscellaneous	Plant-Prokaryote DOE project	Experimental-PTPS	Sepiapterin reductase (EC 1.1.1.153)	0	0	0	0	0	0.002435	0.000722	0.001495	0.002189	0	0.000116	0.000552143
Clustering-based subsystems	Putative asociate of RNA polymerase sigma-54 factor rpoN	CBSS-316057.3.peg.1308	Sigma-70 region 2:Sigma-70 region 4	0.002437	0	0	0	0	0.000974	0.001444	0	0.001314	0	0.000349	0.00063102
Iron acquisition and metabolism	NULL	Iron acquisition in Vibrio	Similar to coproporphyrinogen III oxidase, oxygen-independent (EC 1.3.99.22)	0	0.002262	0	0	0.001313	0	0.000722	0.000997	0.000657	0	0	0.000828214
DNA Metabolism	DNA replication	DNA topoisomerases, Type I, ATP-independent	Similar to C-terminal Zn-finger domain of DNA topoisomerase I	0.002785	0.002262	0	0	0.000657	0	0	0.001495	0.003065	0	0	0.000433826
Amino Acids and Derivatives	Proline and 4-hydroxyproline	Proline, 4-hydroxyproline uptake and utilization	Similar to eukaryotic Peptidyl prolyl 4-hydroxylase, alpha subunit (EC 1.14.11.2)	0	0.002262	0	0	0	0.000487	0	0.000997	0.000219	0	0.000116	0.000197194
Protein Metabolism	Protein biosynthesis	Ribosome biogenesis bacterial	Similar to ribosomal large subunit pseudouridine synthase A	0	0.004524	0	0	0.000657	0	0.001444	0.000498	0.000876	0	0	0.001143724
DNA Metabolism	DNA repair	DNA repair, bacterial RecFOR pathway	Single-stranded-DNA-specific exonuclease RecJ, cyanobacterial paralog	0	0.002262	0	0	0.00197	0	0	0.001495	0.001314	0	0.000116	0.000118316
Cofactors, Vitamins, Prosthetic Groups, Pigments	Tetrapyrroles	Heme and Siroheme Biosynthesis	Sirohydrochlorin ferrochelatase (EC 4.99.1.4)	0	0	0	0	0.002626	0.000974	0	0.001993	0.000876	0.002203	0	0.001380356
Virulence, Disease and Defense	Adhesion	Streptococcus pyogenes recombinatorial zone	Sortase	0	0	0	0.00498	0	0.000487	0.000722	0	0.001314	0	0.000233	0.000354949
Dormancy and Sporulation	NULL	SpoVS protein family	SpoVS-related protein, type 2	0	0	0.002555	0	0.000657	0.000487	0	0	0.000219	0	0.000116	0.000552143
Protein Metabolism	Protein biosynthesis	Ribosome SSU eukaryotic and archaeal	SSU ribosomal protein S17e	0	0	0	0	0	0.001948	0.000722	0.000997	0.000438	0	0.000466	0.000394388
Protein Metabolism	Protein biosynthesis	Ribosome SSU eukaryotic and archaeal	SSU ribosomal protein S18e (S13p)	0	0	0	0.00249	0.00197	0	0.005055	0.00299	0.000876	0	0	0.000473265
Protein Metabolism	Protein biosynthesis	Ribosome SSU eukaryotic and archaeal	SSU ribosomal protein S27Ae	0	0	0.002555	0	0.000657	0	0.000722	0.000997	0	0	0.000233	0.00031551
Dormancy and Sporulation	NULL	Sporulation gene orphans	Stage V sporulation protein required for dehydratation of the spore core and assembly of the coat (SpoVS)	0.016015	0	0	0	0	0.000487	0	0.000498	0.000876	0	0.000349	0.00094653
Cofactors, Vitamins, Prosthetic Groups, Pigments	NULL	Thiamin biosynthesis	Substrate-specific component ThiW of predicted thiazole ECF transporter	0.004874	0	0	0	0.00197	0.000974	0.000722	0	0	0	0.000116	0.000709898
Carbohydrates	Central carbohydrate metabolism	TCA Cycle	Succinyl-CoA ligase [GDP-forming] beta chain (EC 6.2.1.4)	0	0.002262	0.002555	0.00249	0	0	0	0	0.000438	0	0.000116	0.000670459
Amino Acids and Derivatives	Arginine; urea cycle, polyamines	Arginine and Ornithine Degradation	Succinylglutamate desuccinylase (EC 3.5.1.96)	0	0	0	0.00249	0	0.000487	0	0.001993	0.013794	0	0.008849	0.000907091
Clustering-based subsystems	NULL	USS-DB-1	Sugar ABC transporter, sugar permease protein 1 USSDB1C	0	0	0.00511	0.00249	0	0.000487	0.000722	0	0.001314	0	0	0.000552143
Regulation and Cell signaling	Quorum sensing and biofilm formation	Symbiotic colonization and sigma-dependent biofilm formation gene cluster	Sugar transferase SypR involved in lipopolysaccharide synthesis	0	0	0.002555	0	0	0.000974	0	0.000498	0.000219	0	0.001397	0.000473265
Amino Acids and Derivatives	Lysine, threonine, methionine, and cysteine	Cysteine Biosynthesis	Sulfate transport system permease protein CysW	0	0.009048	0.002555	0.00249	0	0	0	0	0.001971	0	0.001281	0.001538111

Amino Acids and Derivatives	Lysine, threonine, methionine, and cysteine	Cysteine Biosynthesis	Sulfate transporter, CysZ-type	0	0.002262	0	0	0.001313	0	0	0.004485	0.007663	0	0.000466	0.003746682
Sulfur Metabolism	Inorganic sulfur assimilation	Inorganic Sulfur Assimilation	Sulfate-binding protein Sbp	0	0	0	0	0	0.000487	0.000722	0	0.000219	0.002203	0.000233	0.000985969
Cofactors, Vitamins, Prosthetic Groups, Pigments	Coenzyme M	coenzyme M biosynthesis	Sulfopyruvate decarboxylase - alpha subunit (EC 4.1.1.79)	0	0	0	0.00249	0.000657	0	0.002888	0.000498	0.000876	0	0	0.000118316
Cofactors, Vitamins, Prosthetic Groups, Pigments	Coenzyme M	coenzyme M biosynthesis	Sulfopyruvate decarboxylase - beta subunit (EC 4.1.1.79)	0	0.002262	0.002555	0	0.00197	0.001948	0	0	0.001314	0	0	0.000670459
Phages, Prophages, Transposable elements, Plasmids	Phages, Prophages	T7-like phage core proteins	T7-like phage DNA Polymerase (EC 2.7.7.7)	0	0	0.002555	0	0.005909	0.018995	0.012275	0.006479	0	0	0	7.88775E-05
Phages, Prophages, Transposable elements, Plasmids	Phages, Prophages	T7-like phage core proteins	T7-like phage endonuclease (EC 3.1.21.2)	0	0	0	0	0.004596	0.004383	0.001444	0.00299	0.000219	0	0	3.94388E-05
Membrane Transport	Sugar Phosphotransferase Systems, PTS	Galactose-inducible PTS	Tagatose-1,6-bisphosphate aldolase AgaY (EC 4.1.2.-)	0	0.004524	0	0	0	0.000487	0.000722	0.000498	0.000219	0	0	0.000512704
Respiration	Electron accepting reactions	Tetrathionate respiration	Tetrathionate reductase sensory transduction histidine kinase	0.003133	0	0	0	0	0.001461	0.001444	0	0.000219	0	0.000233	0.003628365
Respiration	Electron accepting reactions	Tetrathionate respiration	Tetrathionate reductase subunit A	0	0	0	0	0.002626	0.001461	0.001444	0.001495	0	0	0.000233	0.00157755
Clustering-based subsystems	Ribosomal Protein L28P relates to a set of uncharacterized proteins	A Gram-positive cluster that relates ribosomal protein L28P to a set of uncharacterized proteins	Thiamin pyrophosphokinase (EC 2.7.6.2)	0	0	0.002555	0	0.00197	0.000974	0	0.001993	0.000657	0	0	0.003510049
Carbohydrates	Sugar alcohols	Glycerol and Glycerol-3-phosphate Uptake and Utilization	Thiosulfate sulfurtransferase GlpE (EC 2.8.1.1)	0.014623	0	0	0	0	0	0.002166	0.000997	0.004598	0	0.010828	0.001380356
Protein Metabolism	Protein biosynthesis	tRNA aminoacylation, Thr	Threonyl-tRNA synthetase fragment	0.000348	0	0	0	0.001313	0.000487	0	0.000498	0.000657	0	0	0.00063102
Metabolism of Aromatic Compounds	Peripheral pathways for catabolism of aromatic compounds	Phenylpropanoid compound degradation	Transcriptional regulator for ferulate or vanillate catabolism	0	0	0	0	0.001313	0.000974	0.001444	0	0.000657	0	0.009897	0.000433826
Amino Acids and Derivatives	Lysine, threonine, methionine, and cysteine	Threonine degradation	Transcriptional regulator GabR of GABA utilization (GntR family with aminotransferase-like domain)	0.001044	0	0.002555	0	0	0	0.002888	0.001495	0.000438	0	0	0.000157755
Carbohydrates	Di- and oligosaccharides	Beta-Glucoside Metabolism	Transcriptional regulator of beta-glucosides utilization, LacI family	0.004178	0	0.002555	0	0	0	0	0.001993	0.004379	0	0.00978	0.00063102
Carbohydrates	Monosaccharides	L-rhamnose utilization	Transcriptional regulator of rhamnose utilization, GntR family	0	0	0	0	0.000657	0.000974	0	0.000997	0.000438	0.004406	0	0.000394388
Clustering-based subsystems	Putrescine/GABA utilization cluster-temporal,to add to SSs	GABA and putrescine metabolism from cluters	Transcriptional regulator, GABA/putrescine utiliation cluster 3	0	0	0	0.00249	0.003283	0.003896	0	0	0.000438	0	0.007801	0.001143724
Amino Acids and Derivatives	Branched-chain amino acids	Ketoisovalerate oxidoreductase	Transcriptional regulator, Hth-3 family	0.002785	0	0	0	0.000657	0	0.000722	0.000498	0.000219	0	0	0.001459234
Carbohydrates	Central carbohydrate metabolism	Glycolate, glyoxylate interconversions	Transcriptional regulator, LysR family, in glycolate utilization operon	0	0	0	0	0.00197	0.000487	0.001444	0.000498	0.001752	0	0	0.000354949
Carbohydrates	Monosaccharides	Hexose Phosphate Uptake System	Transcriptional regulatory protein UhpA	0	0	0	0.00498	0.00197	0.000487	0.000722	0	0.005036	0	0	0.004772089
Carbohydrates	Aminosugars	N-Acetyl-Galactosamine and Galactosamine Utilization	Transcriptional repressor of aga operon	0	0	0	0	0.001313	0	0.000722	0.000997	0.001533	0	0.000815	0.000354949
Protein Metabolism	Protein biosynthesis	Translation elongation factors eukaryotic and archaeal	Translation elongation factor 1 alpha-related protein	0	0	0	0	0.002626	0	0.001444	0.001495	0.000876	0.006608	0	0.00063102
Protein Metabolism	Protein biosynthesis	Translation elongation factor G family	Translation elongation factor G, mitochondrial	0.002785	0	0	0.00249	0.001313	0.000487	0	0.000498	0	0	0	0.000433826
Protein Metabolism	Protein biosynthesis	Translation elongation factors bacterial	Translation elongation factor P-related protein	0	0	0.002555	0	0	0.000974	0.000722	0.00299	0	0	0.003377	0.002800152
Clustering-based subsystems	Putative asociate of RNA polymerase sigma-54 factor rpoN	CBSS-316057.3.peg.1308	Transmembrane regulator protein PrtR	0	0	0	0.00249	0.001313	0.000487	0.002888	0	0.002189	0	0	0.000394388
Respiration	Electron accepting reactions	Terminal cytochrome oxidases	Transport ATP-binding protein CydCD	0	0	0	0	0	0.000487	0.000722	0	0.001095	0.002203	0.000466	0.001222601
Phages, Prophages, Transposable elements, Plasmids	Transposable elements	CBSS-203122.12.peg.188	Transposase OrfAB, subunit B	0	0	0.002555	0.00249	0.003939	0.002435	0	0	0.001752	0	0	0.001459234
Membrane Transport	NULL	Tricarboxylate transport system	Tricarboxylate transport sensor protein TctE	0	0.002262	0	0	0.002626	0.000974	0	0.000997	0.000876	0	0	0.00094653
Cofactors, Vitamins, Prosthetic Groups, Pigments	Folate and pterines	Methanopterin biosynthesis2	Triphosphoribosyl-dephospho-CoA synthetase	0	0	0.002555	0	0	0	0.001444	0.000498	0.001533	0.002203	0	0.000828214
Miscellaneous	NULL	Archease2	tRNA and rRNA cytosine-C5-methylase Type 1	0	0.002262	0	0.00249	0.000657	0.000974	0	0	0.000219	0	0	0.000394388
RNA Metabolism	RNA processing and modification	tRNA modification Archaea	tRNA N2,N2-dimethyl(Guanine26-N2)-methyltransferase (EC 2.1.1.32)	0	0.006786	0	0	0.000657	0.001948	0	0	0.002408	0	0.000116	0.000512704
RNA Metabolism	RNA processing and modification	tRNA nucleotidyltransferase	tRNA nucleotidyltransferase, archaeal type (EC 2.7.7.21) (EC 2.7.7.25)	0	0	0	0	0.002626	0.002435	0.000722	0.001993	0.001752	0	0	0.000276071
RNA Metabolism	RNA processing and modification	tRNA modification Archaea	tRNA-(G10-N2) methyltransferase; tRNA-(G10-N2) dimethyltransferase	0	0	0	0	0	0.000487	0	0.000498	0.000438	0.004406	0.000116	0.000749336
RNA Metabolism	RNA processing and modification	tRNA modification Archaea	tRNA(U54)-2-thioribothymidine synthetase	0.002785	0.004524	0.00511	0	0.000657	0	0	0	0.000876	0	0	0.001143724
RNA Metabolism	RNA processing and modification	tRNA splicing	tRNA-intron endonuclease (EC 3.1.27.9)	0	0.004524	0	0.00498	0	0	0.000722	0.001495	0.001095	0	0	0.000473265
Amino Acids and Derivatives	Aromatic amino acids and derivatives	Aromatic amino acid degradation	Tryptophan 2-monooxygenase (EC 1.13.12.3)	0	0	0	0.00249	0	0.000487	0.002166	0	0.001095	0.002203	0	0.000394388
Regulation and Cell signaling	NULL	Two-component regulatory systems in Campylobacter	Two-component system histidine kinase	0.002089	0	0.007664	0	0.001313	0.000487	0	0.001495	0	0	0	0.01869397
Membrane Transport	Protein secretion system, Type III	Type III secretion systems	Type III effector HrpW, hairpin with pectate lyase domain	0	0.004524	0	0	0.00197	0	0.000722	0.000498	0.000219	0	0	7.88775E-05
DNA Metabolism	NULL	Restriction-Modification System	Type III restriction-modification system DNA endonuclease res (EC 3.1.21.5)	0	0.002262	0.002555	0	0	0.000487	0	0	0.001533	0.002203	0	0.000591581
Respiration	Electron accepting reactions	Ubiquinone Menaquinone-cytochrome c reductase complexes	Ubiquinol-cytochrome C reductase complex core protein I, mitochondrial precursor (EC 1.10.2.2)	0	0	0.00511	0	0.000657	0.000487	0.001444	0.000997	0	0	0	0.000670459
Cofactors, Vitamins, Prosthetic Groups, Pigments	Quinone cofactors	Ubiquinone Biosynthesis	Ubiquinone biosynthesis methyltransferase COQ5, mitochondrial precursor (EC 2.1.1.-)	0	0	0	0	0	0.001461	0.000722	0.000498	0.001095	0	0.000349	0.000276071
Respiration	NULL	Methanogenesis strays	Uncharacterized ABC transporter ATP-binding protein MJ0121	0	0.002262	0.00511	0	0	0.000487	0.000722	0.000498	0	0	0	0.000788775
Cell Wall and Capsule	Gram-Negative cell wall components	Lipopolysaccharide-related cluster in Alphaproteobacteria	Uncharacterized protein, Bsl7517 homolog	0	0	0	0	0.001313	0	0.001444	0.001993	0.001752	0	0.000233	0.000354949
Miscellaneous	Plant-Prokaryote DOE project	At1g01770	Uncharacterized transporter, similarity to citrate transporter	0	0.004524	0	0.00249	0	0	0	0	0.001971	0.002203	0.000349	0.001695866
Stress Response	NULL	Universal stress protein family	Universal stress protein E	0	0	0	0	0	0.001461	0.002166	0.002492	0.008977	0	0.002445	0.000276071
Stress Response	NULL	Universal stress protein family	Universal stress protein family COG0589	0	0	0	0	0.000657	0.001461	0.002166	0.002492	0.000219	0	0	0.001025408
Clustering-based subsystems	Two related proteases	CBSS-257314.1.peg.676	Unspecified monosaccharide ABC transport system, permease component Ia (FIG025991)	0.002785	0.002262	0	0	0.000657	0.002435	0	0	0.000438	0	0	0.000828214
Clustering-based subsystems	DNA metabolism	CBSS-269801.1.peg.2186	UPF0118 membrane protein YrrI	0	0	0.002555	0	0.000657	0.000487	0.000722	0	0.000657	0	0	0.000354949
Carbohydrates	NULL	Unknown carbohydrate utilization (cluster Ydj)	UPF0229 protein YeaH	0	0.002262	0	0	0	0.000974	0	0.000498	0.001752	0	0.000699	0.00126204
Amino Acids and Derivatives	Proline and 4-hydroxyproline	Proline, 4-hydroxyproline uptake and utilization	Ureidoglycolate/malate/sulfolactate dehydrogenase family (EC 1.1.1.-)	0	0	0	0.00249	0.000657	0	0	0.000997	0.000219	0	0.000466	0.002405764
Carbohydrates	Central carbohydrate metabolism	Dehydrogenase complexes	Valine dehydrogenase (EC 1.4.1.-)	0	0.004524	0	0	0.000657	0	0.000722	0	0.000657	0	0.000233	0.000197194
Respiration	ATP synthases	V-Type ATP synthase	V-type ATP synthase subunit C (EC 3.6.3.14)	0	0	0	0	0.001313	0.000974	0.005055	0.000997	0.002189	0	0	0.000473265
Respiration	ATP synthases	V-Type ATP synthase	V-type ATP synthase subunit E (EC 3.6.3.14)	0	0	0	0	0.000657	0.000974	0.002166	0.002492	0.002408	0	0	0.000670459
Nucleosides and Nucleotides	Purines	Xanthosine utilization (xap region)	Xanthosine phosphorylase (EC 2.4.2.1)	0	0	0	0	0.000657	0	0.000722	0.00299	0.000438	0	0.000233	7.88775E-05
Carbohydrates	NULL	Sugar utilization in Thermotogales	Xylan oligosaccharide ABC transporter, ATP-binding protein 2	0	0.002262	0	0	0	0.000974	0.001444	0.000498	0.001095	0	0	0.001419795

Carbohydrates	NULL	Sugar utilization in Thermotogales	Xylose ABC transporter, permease component	0	0.002262	0	0	0.000657	0.001461	0.002166	0	0.000438	0	0	0.001222601
Carbohydrates	Monosaccharides	Xylose utilization	Xylose oligosaccharides ABC transporter, ATP-binding protein 2	0.002437	0.002262	0	0	0.001313	0	0.000722	0	0.000876	0	0	0.000985969
Miscellaneous	Plant-Prokaryote DOE project	COG3533	Xyloside transporter XynT	0	0.004524	0	0	0	0.001461	0.000722	0.000498	0.003065	0	0	0.001616989
Fatty Acids, Lipids, and Isoprenoids	Isoprenoids	Carotenoids	Zeaxanthin glucosyl transferase	0.002437	0.002262	0	0	0.000657	0	0	0	0.000438	0	0.000349	0.000118316
Fatty Acids, Lipids, and Isoprenoids	Fatty acids	Phospholipid and Fatty acid biosynthesis related cluster	(3R)-hydroxymyristoyl-[ACP] dehydratase (EC 4.2.1.-)	0	0	0	0.00249	0	0.000974	0	0.000997	0.000219	0	0	0.000709898
Carbohydrates	Aminosugars	(GlcNAc)2 Catabolic Operon	(GlcNAc)2 ABC transporter, ATP-binding component 2	0	0	0	0.00249	0	0.000974	0	0	0.000876	0	0.000349	0.000276071
Carbohydrates	Sugar alcohols	Glycerol fermenation to 1,3-propanediol	1,3-propanediol dehydrogenase (EC 1.1.1.202)	0	0.002262	0	0.00249	0.002626	0	0	0.000997	0	0	0	0.000276071
Virulence, Disease and Defense	Adhesion	Mediator of hyperadherence YidE in Enterobacteria and its conserved region	16 kDa heat shock protein B	0	0	0	0	0	0.000487	0	0.000997	0.001095	0	0.017116	0.000591581
Miscellaneous	Plant-Prokaryote DOE project	At1g24340	2,4-dichlorophenol 6-monooxygenase (EC 1.14.13.20)	0.001393	0	0	0	0	0.002435	0	0.003489	0.002846	0	0.000233	0
Carbohydrates	Monosaccharides	D-gluconate and ketogluconates metabolism	2,5-diketo-D-gluconate reductase A (EC 1.1.1.274)	0	0	0.002555	0	0	0.000974	0	0	0.000219	0	0.000116	0.000236633
Amino Acids and Derivatives	Aromatic amino acids and derivatives	Tryptophan catabolism	2-aminomuconate semialdehyde dehydrogenase (EC 1.2.1.32)	0	0	0	0	0	0	0.000722	0.001495	0.001314	0	0.000466	0.000591581
Carbohydrates	Monosaccharides	D-gluconate and ketogluconates metabolism	2-Keto-D-gluconate dehydrogenase (EC 1.1.99.4), membrane-bound, cytochrome c	0.000348	0	0	0	0.000657	0	0.000722	0	0.000438	0	0	0.000157755
Amino Acids and Derivatives	Lysine, threonine, methionine, and cysteine	Lysine fermentation	3-aminobutyrl-CoA ammonia lyase (EC 4.3.1.14)	0	0	0.00511	0	0.002626	0.000487	0.000722	0	0	0	0	0.000670459
Carbohydrates	Monosaccharides	L-ascorbate utilization (and related gene clusters)	3-dehydro-L-gulonate 2-dehydrogenase (EC 1.1.1.130)	0	0	0.00511	0	0	0.001461	0.000722	0	0.000438	0	0	0.000591581
Fatty Acids, Lipids, and Isoprenoids	Fatty acids	Phospholipid and Fatty acid biosynthesis related cluster	3-hydroxydecanoyl-[ACP] dehydratase (EC 4.2.1.60)	0.002437	0	0	0	0	0.000487	0	0.000498	0.000657	0	0	0.000670459
Carbohydrates	Sugar alcohols	Inositol catabolism	5-keto-2-deoxy-D-gluconate-6 phosphate aldolase [form 2] (EC 4.1.2.29)	0	0	0	0.00249	0	0	0.001444	0.001495	0	0.002203	0	0.000197194
Nucleosides and Nucleotides	Detoxification	Housecleaning nucleoside triphosphate pyrophosphatases	5'-nucleotidase YjjG (EC 3.1.3.5)	0	0	0	0	0	0.000487	0	0.001495	0.005912	0	0.007335	0.005245354
Respiration	NULL	Carbon monoxide induced hydrogenase	5-tetrahydromethanopterin:corrinoid iron-sulfur protein methyltransferase	0	0.004524	0.00511	0.00249	0.001313	0	0	0	0	0	0	0.001064846
Iron acquisition and metabolism	NULL	ABC transporter [iron.B12.siderophore.hemin]	ABC transporter (iron.B12.siderophore.hemin) , periplasmic substrate-binding component	0	0	0.002555	0	0	0.000487	0	0	0.000219	0	0.000815	0.000157755
Sulfur Metabolism	Inorganic sulfur assimilation	Inorganic Sulfur Assimilation	ABC-type probable sulfate transporter, permease protein	0	0	0.002555	0	0.001313	0	0.001444	0.000498	0	0	0	7.88775E-05
Carbohydrates	Fermentation	Acetoin, butanediol metabolism	Acetoin catabolism protein X	0	0	0	0	0.000657	0.000487	0.001444	0	0.001314	0	0	0.001143724
Carbohydrates	Central carbohydrate metabolism	Ethylmalonyl-CoA pathway of C2 assimilation	Acetyl-CoA acetyltransferase (EC 2.3.1.9) of ethylmalonyl-CoA pathway	0.002089	0	0	0	0	0.000487	0	0.001993	0.000657	0	0	7.88775E-05
Miscellaneous	Plant-Prokaryote DOE project	At1g26220 At1g32070	Acetyltransferase (GNAT) family, Syn7942 2240 homolog	0	0.002262	0	0	0.000657	0	0	0	0.000876	0	0.000582	0.000197194
Fatty Acids, Lipids, and Isoprenoids	Isoprenoids	Acyclic terpenes utilization	Acyclic terpenes utilization regulator AtuR, TetR family	0	0	0.00511	0	0	0	0.002888	0	0.001314	0.004406	0	0.000354949
Cell Division and Cell Cycle	NULL	Intracellular septation in Enterobacteria	Acyl-CoA thioesterase YciA, involved in membrane biogenesis	0.002437	0.002262	0	0	0	0	0	0.001993	0.000219	0	0	0.000512704
Membrane Transport	NULL	Transport of Nickel and Cobalt	Additional component NikL of nickel ECF transporter	0	0	0	0	0.001313	0.000487	0.001444	0	0.000438	0	0	3.94388E-05
Phages, Prophages, Transposable elements, Plasmids	Phages, Prophages	Phage DNA synthesis	Adenine DNA methyltransferase, phage-associated	0	0	0	0	0.000657	0.001948	0.000722	0.002492	0	0	0	3.94388E-05
Cell Wall and Capsule	Gram-Negative cell wall components	Vibrio Core Oligosaccharide Biosynthesis	ADP-heptose--lipooligosaccharide heptosyltransferase, putative	0	0	0.002555	0.00747	0.000657	0	0.001444	0	0	0	0	0.000670459
Carbohydrates	Central carbohydrate metabolism	Pyruvate metabolism I: anaplerotic reactions, PEP	Aerobic C4-dicarboxylate transporter for fumarate, L-malate, D-malate, succunate, aspartate	0	0	0	0	0	0.000487	0.000722	0	0.000438	0.006608	0	0.00063102
Stress Response	Oxidative stress	Regulation of Oxidative Stress Response	Aerobic respiration control protein arcA	0	0	0	0	0	0	0	0.001495	0.003722	0.002203	0.010363	0.000354949
Cell Wall and Capsule	Capsular and extracellular polysacchrides	Alginate metabolism	Alginate biosynthesis protein Alg8	0	0.004524	0	0	0	0.001461	0	0.000997	0.000219	0	0	0.000236633
Clustering-based subsystems	CRISPRs and associated hypotheticals	CBSS-216592.1.peg.3534	Alkaline phosphatase isozyme conversion protein precursor (EC 3.4.11.-)	0	0	0	0.00249	0.000657	0.000487	0	0	0.000219	0	0	0.000276071
Phosphorus Metabolism	NULL	Phosphate metabolism	Alkaline phosphatase like protein	0	0	0.002555	0	0.00197	0.002922	0	0	0.001752	0	0	0.000512704
Regulation and Cell signaling	NULL	DNA-binding regulatory proteins, strays	Alkanesulfonate utilization operon LysR-family regulator Cbl	0	0.002262	0	0.00249	0.000657	0	0	0	0.000219	0	0	0.000709898
Sulfur Metabolism	NULL	Thioredoxin-disulfide reductase	Alkylhydroperoxidase protein D	0	0	0	0	0.00197	0.000487	0	0.003987	0.007006	0	0	0.000433826
Clustering-based subsystems	Biosynthesis of galactoglycans and related lipopolysacharides	CBSS-376686.6.peg.291	Alpha-1,4-N-acetylgalactosamine transferase PglH (EC 2.4.1.-)	0	0	0	0.00249	0	0	0.000722	0.000498	0.000876	0	0	0.006546833
Carbohydrates	Organic acids	Alpha-acetolactate operon	Alpha-acetolactate decarboxylase (EC 4.1.1.5)	0.003482	0	0	0	0	0.000487	0	0.000498	0.001752	0	0	0.002169132
Carbohydrates	Polysaccharides	Cellulosome	Alpha-amylase (Neopullulanase) SusA (EC 3.2.1.135)	0	0	0	0	0	0	0.000722	0.001993	0.000876	0	0.000466	0.006941221
Miscellaneous	Plant-Prokaryote DOE project	COG3533	Alpha-arabinosides ABC transport system, permease protein 2	0	0	0	0.00249	0	0	0.000722	0.000498	0.000657	0	0	0.00063102
Respiration	Electron accepting reactions	Terminal cytochrome C oxidases	Alternative cytochrome c oxidase polypeptide CoxO (EC 1.9.3.1)	0	0	0	0	0.001313	0	0.000722	0.000498	0.001095	0	0	0.006901782
Miscellaneous	Plant-Prokaryote DOE project	COG4319	Alternative dihydrofolate reductase 3	0	0	0.00511	0	0.000657	0	0	0.001495	0.000657	0	0	0.000591581
Clustering-based subsystems	NULL	CBSS-235.1.peg.567	Amino acid regulated cytosolic protein	0	0	0.002555	0	0	0	0.000722	0	0.001095	0	0.000466	0.000197194
Carbohydrates	Monosaccharides	L-Arabinose utilization	Arabinose operon regulatory protein	0	0	0.002555	0.00249	0	0	0	0.000498	0.000219	0	0	0.000670459
RNA Metabolism	RNA processing and modification	tRNA modification Archaea	archaeosine tRNA-ribosyltransferase (EC 2.4.2.-) PUA domain	0	0	0	0	0.001313	0.001948	0.001444	0	0.000876	0	0	0.000276071
RNA Metabolism	RNA processing and modification	Queuosine-Archaeosine Biosynthesis	archaeosine tRNA-ribosyltransferase (EC 2.4.2.-) type 2	0	0.006786	0	0	0	0.001461	0	0.001495	0.000876	0	0	0.000394388
Amino Acids and Derivatives	Arginine; urea cycle, polyamines	Arginine and Ornithine Degradation	Arginine/ornithine ABC transporter, periplasmic arginine/ornithine binding protein	0	0	0	0	0	0.000974	0.000722	0.000498	0.001314	0	0	0.000354949
Amino Acids and Derivatives	Arginine; urea cycle, polyamines	Arginine and Ornithine Degradation	Arginine/ornithine ABC transporter, permease protein AotM	0.000696	0	0	0	0.001313	0.000974	0	0	0	0.004406	0	0.000157755
Amino Acids and Derivatives	Aromatic amino acids and derivatives	Chorismate Synthesis	Arogenate dehydrogenase (EC 1.3.1.43)	0	0	0	0	0	0.002922	0.000722	0	0.001095	0	0.000116	0.000236633
Protein Metabolism	Protein degradation	Proteolysis in bacteria, ATP-dependent	ATP-dependent protease domain protein (EC 3.4.21.-)	0	0	0	0	0	0	0.000722	0	0.000876	0.002203	0.000233	7.88775E-05
Regulation and Cell signaling	Quorum sensing and biofilm formation	Quorum-sensing in Vibrio	Autoinducer 2 sensor kinase/phosphatase LuxQ (EC 2.7.3.-) (EC 3.1.3.-)	0	0	0	0	0	0	0.000722	0.000997	0.000219	0	0.000233	0.001025408
Regulation and Cell signaling	Quorum sensing and biofilm formation	Acyl Homoserine Lactone (AHL) Autoinducer Quorum Sensing	Autoinducer synthesis protein LuxI	0	0	0	0	0.001313	0.000487	0	0.00299	0.000219	0	0	0.000394388
Iron acquisition and metabolism	Siderophores	Bacillibactin Siderophore	Bacillibactin synthetase component F (EC 2.7.7.-)	0	0.002262	0	0	0	0	0	0.000997	0.000876	0.002203	0	0.00063102
Clustering-based subsystems	Methylamine utilization	CBSS-265072.7.peg.546	Bacillolysin (EC 3.4.24.28)	0	0	0.002555	0	0	0.000487	0.000722	0	0.000219	0	0	0.000433826
Metabolism of Aromatic Compounds	NULL	Benzoate transport and degradation cluster	Benzoate transport related protein	0	0	0	0	0	0.001461	0.000722	0.000498	0.001314	0	0	0.000709898
Carbohydrates	NULL	Sugar utilization in Thermotogales	Beta-glucoside ABC transport system, ATP-binding protein 2	0	0.004524	0	0.00498	0	0	0	0	0.000438	0	0.000116	0.000197194

Carbohydrates	Monosaccharides	D-gluconate and ketogluconates metabolism	Broad-specificity glycerol dehydrogenase (EC 1.1.99.22), subunit SldA	0	0	0	0	0.000657	0.000487	0	0.000498	0.001314	0	0	0.00031551
Cell Wall and Capsule	Capsular and extracellular polysacchrides	Vibrio Polysaccharide (VPS) Biosynthesis	CapK protein, putative	0	0	0	0.00747	0	0.000487	0	0	0.000219	0.002203	0	0.000433826
Cell Wall and Capsule	Capsular and extracellular polysacchrides	Serotype determining Capsular polysaccharide biosynthesis in Staphylococcus	Capsular polysaccharide synthesis enzyme Cap8E	0	0	0	0.00249	0	0	0.000722	0	0.000438	0	0.000466	0.00063102
Clustering-based subsystems	Clustering-based subsystems	Putative diaminopropionate ammonia-lyase cluster	Carbamate kinase-like protein YqeA	0	0	0.002555	0	0.000657	0	0.000722	0	0.000219	0	0	0.000591581
Miscellaneous	Plant-Prokaryote DOE project	COG0523	Carbon starvation protein A paralog	0	0	0	0.00249	0	0.000974	0	0.001495	0.000438	0	0	0.000985969
Regulation and Cell signaling	NULL	Zinc regulated enzymes	Carbonic anhydrase, gamma class (EC 4.2.1.1)	0	0.004524	0	0	0	0.000487	0	0.001495	0	0	0.000582	0.000236633
Carbohydrates	CO2 fixation	CO2 uptake, carboxysome	Carboxysome protein CcmM	0	0	0	0.00249	0.000657	0.000974	0	0	0.002408	0	0.000116	0
Protein Metabolism	Protein processing and modification	Inteins	Carboxy-terminal intein-mediated trans-splice	0	0	0	0	0	0.000487	0.000722	0	0.000657	0	0.000931	0.00031551
Miscellaneous	Plant-Prokaryote DOE project	At4g38090	Cell envelope-associated transcriptional attenuator LytR-CpsA-Psr, subfamily F2 (as in PMID19099556)	0	0.002262	0	0	0	0	0	0.000498	0.000657	0.002203	0	0.000354949
Regulation and Cell signaling	NULL	Cell envelope-associated LytR-CpsA-Psr transcriptional attenuators	Cell envelope-associated transcriptional attenuator LytR-CpsA-Psr, subfamily M (as in PMID19099556)	0.002785	0	0	0	0	0.002435	0	0	0.000657	0	0.001164	0.00031551
Cell Wall and Capsule	Gram-Positive cell wall components	Sortase	Cell wall surface anchor family protein	0	0	0.002555	0	0.000657	0	0	0.000498	0.000438	0	0	0.00031551
Protein Metabolism	Protein folding	Protein chaperones	Chaperone-modulator protein CbpM	0	0	0	0	0.000657	0	0.000722	0.000498	0.003503	0	0	0.000709898
Carbohydrates	Aminosugars	Chitin and N-acetylglucosamine utilization	Chitin binding protein	0	0	0	0	0.00197	0	0.006499	0.000997	0.002189	0	0	0.002050815
Stress Response	Osmotic stress	Choline and Betaine Uptake and Betaine Biosynthesis	Choline ABC transport system, ATP-binding protein OpuBA	0	0	0	0	0	0	0.000722	0.000997	0.000657	0.002203	0	0.000236633
Miscellaneous	Plant-Prokaryote DOE project	COG3146 experimental	Chorismate--pyruvate lyase (EC 4.1.3.40)	0.000696	0	0	0	0.001313	0	0	0.000997	0.002189	0	0	0.000276071
Clustering-based subsystems	NULL	RNA modification and chromosome partitioning cluster	Chromosome (plasmid) partitioning protein ParB-2	0.000696	0	0.002555	0	0	0	0	0.000498	0	0.002203	0	0.000197194
Cell Division and Cell Cycle	NULL	MukBEF Chromosome Condensation	Chromosome partition protein MukB	0	0	0	0	0.000657	0.000487	0.001444	0	0.000438	0	0	0.000197194
Respiration	Electron donating reactions	Coenzyme F420-H2 dehydrogenase (methanophenazine)	Coenzyme F(420)H(2) dehydrogenase (methanophenazine) subunit FpoN	0	0	0.002555	0	0	0.000487	0.001444	0	0.001095	0	0	0.000236633
RNA Metabolism	RNA processing and modification	rRNA modification Archaea	COG2106 Methylase	0	0	0	0	0.000657	0	0.000722	0.000997	0.001533	0	0	3.94388E-05
Virulence, Disease and Defense	NULL	Streptococcus pyogenes Virulome	Collagen-like surface protein	0	0	0	0	0.000657	0.002435	0.002888	0.001495	0	0	0	0.00031551
Membrane Transport	Protein and nucleoprotein secretion system, Type IV	Conjugative transfer	Conjugative transfer protein TrbG	0	0	0	0	0.000657	0.001461	0	0	0.000438	0	0.000116	0.000473265
Phages, Prophages, Transposable elements, Plasmids	Transposable elements	Conjugative transposon, Bacteroidales	Conjugative transposon protein TraG	0	0	0	0	0	0.000974	0	0.000997	0.003941	0	0.000233	0.003588927
Phages, Prophages, Transposable elements, Plasmids	Transposable elements	Conjugative transposon, Bacteroidales	Conjugative transposon protein TraM	0	0	0.002555	0	0	0	0	0	0.000219	0.006608	0.000233	0.000670459
Sulfur Metabolism	Inorganic sulfur assimilation	Inorganic Sulfur Assimilation	Conserved hypothetical protein probably involved in assimilatory sulfate reduction	0	0.002262	0	0	0	0	0.001444	0.001495	0.001095	0	0	0.003904437
Virulence, Disease and Defense	Resistance to antibiotics and toxic compounds	Cobalt-zinc-cadmium resistance	Copper sensory histidine kinase CusS	0	0	0.002555	0	0.000657	0	0	0.000498	0.000219	0	0	0.001025408
Cofactors, Vitamins, Prosthetic Groups, Pigments	Tetrapyrroles	Heme and Siroheme Biosynthesis	Coproporphyrinogen III oxidase, oxygen-independent (EC 1.3.99.22), divergent, putative	0	0.002262	0	0	0	0	0.000722	0.000997	0.000219	0	0	0.00031551
DNA Metabolism	CRISPs	CRISP Cmr Cluster	CRISPR-associated RAMP Cmr4	0	0	0	0	0.000657	0	0.000722	0.000498	0.003065	0	0	0.00031551
Fatty Acids, Lipids, and Isoprenoids	Fatty acids	Carnitine Metabolism in Microorganisms	Crotonobetainyl-CoA dehydrogenase (EC 1.3.99.-)	0	0	0.00511	0	0	0.000487	0	0.000498	0.000876	0	0	0.00031551
Membrane Transport	Protein secretion system, Type VIII (Extracellular nucleation/precipitation pathway, ENP)	Curli production	Curli production assembly/transport component CsgF	0	0	0	0	0	0.000487	0.000722	0.001495	0.000657	0	0	0.002129693
RNA Metabolism	Transcription	Transcription factors cyanobacterial RpoD-like sigma factors	Cyanobacteria-specific RpoD-like sigma factor, type-1	0	0	0	0.00498	0.003283	0.000974	0	0	0.001314	0	0	7.88775E-05
Amino Acids and Derivatives	Lysine, threonine, methionine, and cysteine	Cysteine Biosynthesis	Cysteine synthase A (EC 2.5.1.47)	0	0	0.002555	0	0.001313	0	0.002888	0	0.001533	0	0	0.000867653
Sulfur Metabolism	Organic sulfur assimilation	L-Cystine Uptake and Metabolism	Cystine ABC transporter, ATP-binding protein	0	0	0	0	0	0.000974	0.000722	0	0.000438	0.002203	0	0.001064846
Photosynthesis	Electron transport and photophosphorylation	Photosystem II	Cytochrome b559 alpha chain (PsbE)	0	0	0	0	0.001313	0	0	0.001993	0.000219	0	0.000466	0.000118316
Respiration	NULL	Soluble cytochromes and functionally related electron carriers	Cytochrome C550 (Soluble cytochrome C)	0	0	0	0	0.001313	0	0	0.000498	0.000876	0	0.005822	0.002405764
Respiration	NULL	Soluble cytochromes and functionally related electron carriers	Cytochrome C553 (soluble cytochrome f)	0	0	0	0.00249	0	0	0	0.000498	0.000219	0	0.000233	0.002326887
Respiration	NULL	Biogenesis of c-type cytochromes	Cytochrome c-type biogenesis protein CcmD, interacts with CcmCE	0.002785	0	0	0	0.000657	0.000487	0	0	0.002189	0	0	0.000788775
Nitrogen Metabolism	NULL	Nitrate and nitrite ammonification	Cytochrome c-type heme lyase subunit nrfE, nitrite reductase complex assembly	0	0	0	0	0.000657	0	0.000722	0.000498	0.000657	0	0	0.000473265
Respiration	Electron accepting reactions	Terminal cytochrome oxidases	Cytochrome O ubiquinol oxidase subunit IV (EC 1.10.3.-)	0.004874	0	0	0	0	0	0.000722	0.001495	0.001095	0	0	0.000433826
Miscellaneous	NULL	DedA family of inner membrane proteins	DedA family inner membrane protein YdjZ	0	0	0.002555	0	0.000657	0.000974	0.000722	0	0	0	0	0.000354949
Cofactors, Vitamins, Prosthetic Groups, Pigments	Coenzyme A	Coenzyme A Biosynthesis	Dephospho-CoA kinase archaeal, predicted (EC 2.7.1.24)	0	0	0	0.00249	0.002626	0	0	0	0.002189	0	0.000233	0.000512704
Carbohydrates	Monosaccharides	D-galactonate catabolism	D-galactonate transporter	0	0.004524	0	0	0	0.000487	0	0.000498	0.004817	0	0	0.001025408
Iron acquisition and metabolism	Siderophores	Siderophore pyochelin	Dihydroaeruginoate synthetase PchE, non-ribosomal peptide synthetase modules	0	0.002262	0	0	0	0.000487	0	0.000498	0.000219	0	0	0.000157755
Sulfur Metabolism	NULL	Release of Dimethyl Sulfide (DMS) from Dimethylsulfoniopropionate (DMSP)	Dimethylsulfoniopropionate (DSMP) lyase DddL	0.003133	0	0	0	0	0	0.002166	0.003489	0.001314	0	0	0.00094653
Virulence, Disease and Defense	Toxins and superantigens	Diphtheria toxin	diptheria toxin resistance protein required for diphthamide biosynthesis (Saccharomyces)-like 1	0	0.002262	0	0	0.000657	0	0	0.000498	0.001095	0	0	0.000276071
Sulfur Metabolism	Organic sulfur assimilation	DMSP breakdown	DMSP demthylase transcriptional regulator	0	0	0	0	0	0.001461	0.001444	0.000997	0.000219	0	0	0.00189306
DNA Metabolism	DNA replication	DNA replication, archaeal	DNA polymerase sliding clamp protein PCNA	0	0	0	0	0.001313	0	0.001444	0.000997	0.001095	0	0	0.000354949
DNA Metabolism	DNA replication	DNA replication, archaeal	DNA primase large subunit (EC 2.7.7.-)	0	0	0	0	0	0	0.001444	0.000498	0.000657	0	0.000116	7.88775E-05
DNA Metabolism	DNA recombination	DNA recombination, archaeal	DNA repair and recombination protein RadB	0	0	0	0	0.000657	0.000974	0.001444	0	0.000438	0	0	0.000354949
DNA Metabolism	DNA replication	DNA topoisomerases, Type II, ATP-dependent	DNA topoisomerase II (EC 5.99.1.3)	0	0	0	0	0.001313	0.002435	0.005777	0.003987	0	0	0	0.000749336
DNA Metabolism	NULL	Nucleoid-associated proteins in Bacteria	DNA-binding protein H-NS	0	0	0	0	0	0.000974	0	0.00299	0.003722	0	0.000116	0.000276071
RNA Metabolism	Transcription	RNA polymerase III	DNA-directed RNA polymerase III largest subunit (EC 2.7.7.6)	0	0	0	0.00249	0.001313	0	0.000722	0	0	0	0.000116	0.000236633
RNA Metabolism	Transcription	RNA polymerase archaeal	DNA-directed RNA polymerase subunit D (EC 2.7.7.6)	0	0	0	0	0.005252	0.001461	0	0.001495	0.000438	0	0	0.00094653
RNA Metabolism	Transcription	RNA polymerase archaeal	DNA-directed RNA polymerase subunit H (EC 2.7.7.6)	0	0	0	0	0	0.000487	0.000722	0	0.000219	0	0.000233	0.000354949
Membrane Transport	Protein and nucleoprotein secretion system, Type IV	Dot-Icm type IV secretion system	DotB protein	0	0	0	0	0.001313	0.000487	0.000722	0.001495	0	0	0	3.94388E-05

Membrane Transport	NULL	ECF class transporters	Duplicated ATPase component of energizing module of predicted tryptophan ECF transporter	0.000348	0	0	0	0	0.001461	0.001444	0	0.000219	0	0	0.00031551
Membrane Transport	NULL	ECF class transporters	Duplicated ATPase component TTE1589 of energizing module of predicted methylthioadenosine ECF transporter	0.004526	0	0.002555	0.00249	0	0	0.000722	0	0	0	0	0.000552143
Membrane Transport	NULL	ECF class transporters	Duplicated ATPase component YkoD of energizing module of thiamin-regulated ECF transporter for HydroxyMethylPyrimidine	0	0	0.002555	0	0.000657	0	0	0	0.001314	0	0.000116	0.000197194
Clustering-based subsystems	Putative asociate of RNA polymerase sigma-54 factor rpoN	CBSS-316057.3.peg.1308	ECF sigma factor	0	0.002262	0	0.00249	0	0.000487	0	0	0.000219	0	0	0.000433826
Carbohydrates	Di- and oligosaccharides	Beta-Glucoside Metabolism	Endo-beta-1,3-1,4 glucanase (Licheninase) (EC 3.2.1.73)	0	0	0.002555	0	0.000657	0	0.002166	0	0	0	0.000233	0.000473265
Respiration	Reverse electron transport	Mebrane bound hydrogenases	Energy conserving hydrogenase Ehb protein Q	0	0.002262	0	0.00498	0	0	0.000722	0	0.000438	0	0	0.000118316
Respiration	Electron donating reactions	Energy-conserving hydrogenase (ferredoxin) Ech	Energy-conserving hydrogenase (ferredoxin), subunit D	0	0	0.002555	0.00747	0	0.000487	0	0.000498	0	0	0	0.00157755
Carbohydrates	Sugar alcohols	Erythritol utilization	Erythritol kinase EryA (EC 2.7.1.27)	0	0	0	0.00249	0	0.000487	0	0	0.000438	0	0.000233	0.00031551
Carbohydrates	Sugar alcohols	Erythritol utilization	Erythritol transcriptional regulator EryD	0	0	0	0	0	0.001461	0.000722	0	0.000438	0	0.000233	0.000197194
Miscellaneous	Plant-Prokaryote DOE project	Synechocystis experimental	Esterase/lipase, sll0644 homolog	0	0.002262	0	0	0	0.000487	0	0	0.000219	0.004406	0	0.000749336
Protein Metabolism	Protein biosynthesis	Translation initiation factors eukaryotic and archaeal	Eukaryotic translation initiation factor 3 110 kDa subunit	0	0	0	0	0.000657	0	0	0.000498	0.000876	0	0.000233	0.000157755
Clustering-based subsystems	Biosynthesis of galactoglycans and related lipopolysacharides	CBSS-258594.1.peg.3339	Exopolysaccharide production protein ExoZ	0	0	0.002555	0	0	0	0.000722	0	0.001095	0	0.000116	7.88775E-05
Nitrogen Metabolism	NULL	Nitrate and nitrite ammonification	Ferredoxin-type protein NapG (periplasmic nitrate reductase)	0	0	0.002555	0	0.002626	0.000974	0	0.000498	0	0	0	0.002366325
Iron acquisition and metabolism	NULL	Campylobacter Iron Metabolism	Ferric receptor CfrA	0	0	0	0	0.003939	0.002922	0.000722	0.001993	0	0	0	0.004417141
Iron acquisition and metabolism	NULL	Iron acquisition in Vibrio	Ferrichrome transport system permease protein FhuB (TC 3.A.1.14.3)	0	0	0	0	0	0.000487	0	0.000498	0.001095	0	0.000582	0.001104285
Iron acquisition and metabolism	NULL	Ferrous iron transporter EfeUOB, low-pH-induced	Ferrous iron transport permease EfeU	0	0	0	0	0.00197	0.000487	0.001444	0	0	0.004406	0	0.000670459
Virulence, Disease and Defense	NULL	Streptococcus pyogenes Virulome	Fibrinogen-binding protein	0	0.006786	0	0	0.003283	0	0.00361	0.000498	0	0	0	0.001616989
Clustering-based subsystems	NULL	CBSS-87626.3.peg.3639	FIG001590: Putative conserved exported protein precursor	0	0	0	0	0	0.000487	0	0.001495	0.003722	0	0.000699	0.000591581
Cell Division and Cell Cycle	NULL	Control of cell elongation - division cycle in Bacilli	FIG001721: Predicted N6-adenine-specific DNA methylase	0	0	0	0	0.00197	0	0.000722	0	0.000438	0	0.000466	0.000591581
Clustering-based subsystems	Ribosome-related cluster	A Gammaproteobacteria Cluster Relating to Translation	FIG002082: Protein sirB2	0	0	0	0.00249	0	0	0	0.001495	0.001533	0	0.002678	0.000473265
Clustering-based subsystems	NULL	Disulphide related cluster	FIG002984: FAD-dependent pyridine nucleotide-disulphide oxidoreductase	0	0	0.007664	0	0	0	0.001444	0.000498	0.003065	0	0	0.006389078
Clustering-based subsystems	DNA polymerase III epsilon cluster	CBSS-342610.3.peg.1536	FIG004599: Hypothetical protein	0	0	0	0	0	0	0	0.000498	0.003065	0.004406	0.000699	0.001143724
Iron acquisition and metabolism	Siderophores	Siderophore Pyoverdine	FIG006220: Hypothetical MbtH-like protein	0	0	0	0	0.000657	0.000487	0	0.000997	0.001314	0	0	3.94388E-05
Clustering-based subsystems	NULL	CBSS-159087.4.peg.2189	FIG014574: hypothetical protein	0	0	0	0	0.000657	0	0.000722	0.000498	0.001314	0	0	0.000236633
Iron acquisition and metabolism	Siderophores	Siderophore Pyoverdine	FIG014801: Cation ABC transporter, periplasmic cation-binding protein	0	0	0	0	0.000657	0.000974	0.000722	0	0.000219	0	0	3.94388E-05
Clustering-based subsystems	NULL	CBSS-316273.3.peg.448	FIG037995: Hypothetical protein	0	0	0	0.00249	0.000657	0.000487	0	0	0.000438	0	0	0.000118316
Miscellaneous	Plant-Prokaryote DOE project	At4g17370	FIG092115: Oxidoreductase, aldo/keto reductase family	0	0	0	0	0	0.000974	0	0.003489	0.001095	0	0.000582	0.000236633
Metabolism of Aromatic Compounds	NULL	Benzoate transport and degradation cluster	FIG094199: Fumarylacetoacetate hydrolase	0	0	0	0	0	0.000487	0.002888	0.000997	0	0	0.008616	0.001143724
Clustering-based subsystems	NULL	Glutaredoxin 3 containing cluster	FIG136845: Rhodanese-related sulfurtransferase	0	0	0	0	0.000657	0.000487	0.001444	0	0.003065	0	0	0.001419795
Respiration	Electron accepting reactions	Terminal cytochrome C oxidases	FixO3 cytochrome-c oxidase subunit	0.000348	0	0	0	0.000657	0.000487	0	0	0.000657	0	0	0.000394388
Motility and Chemotaxis	Flagellar motility in Prokaryota	Flagellum	Flagellar protein FliJ	0	0	0	0	0	0	0.001444	0.002492	0.001971	0	0.00489	0.000670459
Motility and Chemotaxis	Flagellar motility in Prokaryota	Archaeal Flagellum	Flagellin FlaB2	0	0	0	0	0.000657	0	0.001444	0.000498	0.000219	0	0	3.94388E-05
Sulfur Metabolism	Organic sulfur assimilation	Alkanesulfonate assimilation	FMN reductase (EC 1.5.1.29)	0	0	0	0	0.001313	0	0.002888	0	0.001314	0	0.000116	0.000197194
Carbohydrates	One-carbon Metabolism	Methanogenesis	Formylmethanofuran dehydrogenase (molybdenum) operon gene E	0	0	0	0	0.001313	0.000487	0.001444	0.000498	0	0	0	0.000473265
Carbohydrates	Sugar alcohols	Mannitol Utilization	Fructokinase in mannitol utilization gene cluster (EC 2.7.1.4)	0	0	0	0	0.000657	0	0.000722	0.000997	0	0	0.000233	0.00094653
Membrane Transport	Protein translocation across cytoplasmic membrane	ESAT-6 proteins secretion system in Firmicutes	FtsK/SpoIIIE family protein, putative secretion system component EssC/YukA	0	0.002262	0	0	0.003939	0.000487	0	0	0.000438	0	0	0.001340918
Carbohydrates	One-carbon Metabolism	Serine-glyoxylate cycle	Fumarate hydratase class I, N-terminal domain	0	0	0.002555	0	0.000657	0.001461	0	0	0.000657	0	0	0.000118316
Amino Acids and Derivatives	Arginine; urea cycle, polyamines	Putrescine utilization pathways	Gamma-glutamyl-GABA hydrolase (EC 3.5.1.94)	0	0	0	0	0	0.000974	0.000722	0.000498	0.000657	0	0	0.000709898
Stress Response	Osmotic stress	Choline and Betaine Uptake and Betaine Biosynthesis	GbcA Glycine betaine demethylase subunit A	0	0	0	0	0	0	0.001444	0	0.001314	0.002203	0.002212	0.000512704
Nucleosides and Nucleotides	Detoxification	Nudix proteins (nucleoside triphosphate hydrolases)	GDP-mannose mannosyl hydrolase (EC 3.6.1.-)	0.002785	0	0	0	0	0.000487	0	0.000997	0.002846	0	0	0.000749336
Nucleosides and Nucleotides	Detoxification	Nudix proteins (nucleoside triphosphate hydrolases)	GDP-mannose pyrophosphatase YffH	0	0	0	0	0	0	0.000722	0.000498	0.000219	0.002203	0	0.006428517
Membrane Transport	Protein secretion system, Type II	General Secretion Pathway	General secretion pathway protein H	0	0	0.002555	0.00249	0	0	0	0.00299	0.001314	0	0	0.000197194
Membrane Transport	Protein secretion system, Type II	General Secretion Pathway	General secretion pathway protein M	0	0	0.002555	0	0	0	0	0.000498	0.001533	0.002203	0	0.000354949
Carbohydrates	Monosaccharides	D-gluconate and ketogluconates metabolism	Gluconate 2-dehydrogenase (EC 1.1.99.3), membrane-bound, flavoprotein	0	0	0	0	0.002626	0	0	0.001993	0.001314	0.004406	0	0.000354949
Carbohydrates	Monosaccharides	D-gluconate and ketogluconates metabolism	Gluconate 2-dehydrogenase (EC 1.1.99.3), membrane-bound, gamma subunit	0.002089	0.002262	0	0	0	0	0	0	0.001314	0	0.000233	7.88775E-05
Carbohydrates	Aminosugars	(GlcNAc)2 Catabolic Operon	Glucosamine-link cellobiase (EC 3.2.1.21)	0	0.015834	0	0.00249	0	0	0	0	0.000438	0	0.000233	0.000118316
Carbohydrates	Central carbohydrate metabolism	Peripheral Glucose Catabolism Pathways	Glucose ABC transport system, inner membrane component 1	0	0	0	0	0.000657	0.000974	0	0	0.000657	0	0.002096	0.000157755
Carbohydrates	Central carbohydrate metabolism	Peripheral Glucose Catabolism Pathways	Glucose ABC transport system, inner membrane component 2	0	0	0	0	0	0.000487	0.000722	0	0.000876	0	0.015253	0.000433826
Respiration	Sodium Ion-Coupled Energetics	Na+ translocating decarboxylases and related biotin-dependent enzymes	Glutaconyl-CoA decarboxylase alpha chain (EC 4.1.1.70)	0.001044	0.002262	0.002555	0	0	0	0	0	0.000219	0	0	0.000197194
Protein Metabolism	Protein degradation	Metallocarboxypeptidases (EC 3.4.17.-)	Glutamate carboxypeptidase (EC 3.4.17.11)	0	0	0.002555	0	0.000657	0	0	0	0.000876	0	0.000116	0.000276071
Cofactors, Vitamins, Prosthetic Groups, Pigments	Folate and pterines	YgfZ	Glutamate--cysteine ligase eukaryotic (EC 6.3.2.2)	0.000348	0	0	0	0	0	0.000722	0	0.000438	0	0.000116	3.94388E-05
Clustering-based subsystems	NULL	CBSS-193567.1.peg.90	Glutamyl aminopeptidase (EC 3.4.11.7)	0	0	0.002555	0.00249	0.001313	0	0	0	0.000876	0	0	0.000749336
Clustering-based subsystems	NULL	Glutaredoxin 3 containing cluster	Glutaredoxin 3 (Grx3)	0	0	0	0	0.000657	0.000974	0	0.000498	0.000219	0	0	0.000197194
Potassium metabolism	NULL	Glutathione-regulated potassium-efflux system and associated functions	Glutathione-regulated potassium-efflux system ancillary protein KefF	0	0.004524	0.002555	0	0.000657	0	0.000722	0	0	0	0	7.88775E-05
Stress Response	Oxidative stress	Glutathionylspermidine and Trypanothione	Glutathionylspermidine amidohydrolase (EC 3.5.1.78)	0	0.004524	0.00511	0	0	0	0	0	0.003941	0	0.090935	0.00220857

Stress Response	Oxidative stress	Glutathionylspermidine and Trypanothione	Glutathionylspermidine synthase (EC 6.3.1.8)	0.000348	0	0.007664	0	0	0	0	0	0.002846	0.008811	0	0.00220857
Protein Metabolism	Selenoproteins	Glycine reductase, sarcosine reductase and betaine reductase	Glycine reductase component B alpha subunit (EC 1.21.4.2)	0	0	0.002555	0	0.000657	0.000487	0	0.000498	0	0	0	0.00063102
Protein Metabolism	Selenoproteins	Glycine reductase, sarcosine reductase and betaine reductase	Glycine/sarcosine/betaine reductase component C chain 2	0	0	0	0	0.000657	0	0	0	0.000438	0.004406	0.000116	0.001025408
Carbohydrates	Central carbohydrate metabolism	Glycolate, glyoxylate interconversions	Glycolate utilization operon transcriptional activator GlcC	0	0	0	0.00249	0	0	0	0.000498	0.002627	0	0.000116	0.000276071
Virulence, Disease and Defense	Resistance to antibiotics and toxic compounds	Cobalt-zinc-cadmium resistance	Heavy metal sensor histidine kinase	0.000348	0	0	0.00249	0.000657	0	0	0	0.000876	0	0	0.001380356
Iron acquisition and metabolism	NULL	Iron Scavenging cluster in Thermus	Heme ABC transporter, permease protein HmuU	0	0	0	0	0	0.000487	0.000722	0.000498	0.000219	0	0	0.00063102
Cofactors, Vitamins, Prosthetic Groups, Pigments	Tetrapyrroles	Heme biosynthesis orphans	Heme biosynthesis protein related to NirL	0	0	0.002555	0	0.000657	0.000974	0.005055	0	0	0	0	0.000670459
Iron acquisition and metabolism	NULL	Heme, hemin uptake and utilization systems in GramPositives	Heme efflux system ATPase HrtA	0.000348	0	0	0	0	0.000487	0	0	0.000219	0	0.000116	0.000512704
Iron acquisition and metabolism	NULL	Heme, hemin uptake and utilization systems in GramNegatives	Hemin transport protein	0	0	0	0	0.000657	0	0	0.001495	0.000657	0.004406	0	0.000512704
Iron acquisition and metabolism	NULL	Heme, hemin uptake and utilization systems in GramPositives	Hemin transport protein HmuS	0	0	0	0	0	0.000487	0.002166	0.002492	0.000219	0	0	0.000118316
Carbohydrates	Monosaccharides	D-gluconate and ketogluconates metabolism	High-affinity gluconate transporter GntT	0	0	0	0.00249	0	0.000974	0	0	0.000876	0	0.000582	0.000828214
Phages, Prophages, Transposable elements, Plasmids	Phages, Prophages	Prophage lysogenic conversion modules	Holin	0	0	0	0	0	0.001461	0.002166	0.001993	0.000219	0	0	7.88775E-05
Membrane Transport	NULL	Transport of Nickel and Cobalt	HoxN/HupN/NixA family nickel/cobalt transporter	0.000696	0	0	0	0	0	0.000722	0	0.000438	0.004406	0	0.000985969
Miscellaneous	Plant-Prokaryote DOE project	DOE COG3533	Hydrolase ass w/ COG3533Xanth	0	0	0	0	0.000657	0	0	0	0.000876	0.002203	0.000116	0.000433826
Carbohydrates	NULL	Uncharacterized sugar kinase cluster (ygc)	Hypothetical oxidoreductase YgcW (EC 1.-.-.-)	0	0	0.00511	0	0	0.000974	0.000722	0.000997	0	0	0	3.94388E-05
Clustering-based subsystems	NULL	USS-DB-1	Hypothetical protein USSDB1A	0	0.002262	0	0	0.000657	0	0	0.000498	0.002408	0	0	0.000473265
Membrane Transport	Protein and nucleoprotein secretion system, Type IV	Dot-Icm type IV secretion system	IcmB (DotO) protein	0	0	0	0	0.002626	0.002922	0.00361	0.00897	0	0	0	0.000236633
Amino Acids and Derivatives	Aromatic amino acids and derivatives	Aromatic amino acid interconversions with aryl acids	Indolepyruvate ferredoxin oxidoreductase, alpha and beta subunits like	0	0	0	0.00249	0	0.000487	0.000722	0	0.001095	0	0	0.000197194
Amino Acids and Derivatives	Aromatic amino acids and derivatives	Aromatic amino acid interconversions with aryl acids	Indolepyruvate oxidoreductase subunit IorB II (EC 1.2.7.8)	0	0.002262	0	0	0	0	0.005055	0.00299	0.001971	0	0	0.000354949
Virulence, Disease and Defense	Bacteriocins, ribosomally synthesized antibacterial peptides	Tolerance to colicin E2	Inner membrane protein CreD-like protein	0	0	0	0	0.000657	0	0	0.000997	0.000876	0	0.000815	0.00094653
Clustering-based subsystems	NULL	Divergent RNA modification related clusters	Inner membrane protein translocase component YidC, OxaA protein	0	0	0	0	0.001313	0	0.000722	0.000498	0.000219	0	0.000233	0
Nucleosides and Nucleotides	Detoxification	Housecleaning nucleoside triphosphate pyrophosphatases	Inosine/xanthosine triphosphatase (EC 3.6.1.-)	0	0	0	0	0	0.000487	0	0.000997	0.000219	0	0.001863	0.000394388
Nucleosides and Nucleotides	Purines	Purine conversions	Inosine-guanosine kinase (EC 2.7.1.73)	0	0	0	0	0.001313	0	0.001444	0.000498	0	0	0.015951	0.000907091
Phages, Prophages, Transposable elements, Plasmids	NULL	Integrons	Integron integrase IntI4	0	0.002262	0	0	0.001313	0	0	0	0.000657	0	0.000233	0.001853621
Iron acquisition and metabolism	NULL	Iron(III) dicitrate transport system Fec	Iron(III) dicitrate transport system permease protein FecC (TC 3.A.1.14.1)	0	0	0	0	0	0.000487	0	0.000997	0.000657	0	0.001048	7.88775E-05
Fatty Acids, Lipids, and Isoprenoids	Isoprenoids	Acyclic terpenes utilization	Isohexenylglutaconyl-CoA hydratase	0	0	0	0	0	0.000974	0.000722	0.003987	0.003284	0	0	0.000512704
Cell Wall and Capsule	NULL	Peptidoglycan Crosslinking of Peptide Stems	L,D-transpeptidase YcbB	0	0	0.002555	0	0	0	0	0.000498	0.000219	0	0.000233	0.00189306
Virulence, Disease and Defense	Bacteriocins, ribosomally synthesized antibacterial peptides	Bacteriocin-like peptides Blp	Lactacin F ABC transporter permease component	0	0	0	0	0.000657	0	0	0.000498	0.000219	0.002203	0	0.000157755
Carbohydrates	Central carbohydrate metabolism	Pyruvate metabolism II: acetyl-CoA, acetogenesis from pyruvate	Lactate 2-monooxygenase (EC 1.13.12.4)	0	0	0	0	0	0.001948	0	0.000997	0.001752	0	0.000116	0.000118316
Carbohydrates	Monosaccharides	L-Arabinose utilization	L-arabinolactonase (EC 3.1.1.15)	0	0	0	0	0.000657	0	0.001444	0.000498	0.000657	0	0	0.000236633
Carbohydrates	NULL	Sugar utilization in Thermotogales	L-fucose isomerase, putative	0	0	0	0.00498	0.001313	0.000487	0	0.001495	0	0	0	0.000236633
Cofactors, Vitamins, Prosthetic Groups, Pigments	Tetrapyrroles	Chlorophyll Biosynthesis	Light-dependent protochlorophyllide reductase (EC 1.3.1.33)	0	0	0	0	0	0.000974	0	0.001495	0.001095	0	0.000466	7.88775E-05
Fatty Acids, Lipids, and Isoprenoids	NULL	Cholesterol catabolic operon in Mycobacteria	Lipid carrier protein IgrF	0	0	0	0	0.000657	0.000974	0.000722	0	0.001095	0	0	0.001616989
Amino Acids and Derivatives	Lysine, threonine, methionine, and cysteine	Lysine degradation	L-pipecolate oxidase (1.5.3.7)	0	0	0	0	0.000657	0.000487	0	0	0	0.002203	0.011876	0.000512704
Clustering-based subsystems	NULL	Lipopolysaccharide assembly cluster	LptA, protein essential for LPS transport across the periplasm	0	0	0	0	0.000657	0.000487	0	0.001495	0.005693	0	0	0.000354949
Protein Metabolism	Protein biosynthesis	Ribosome LSU eukaryotic and archaeal	LSU ribosomal protein L15e	0	0	0	0	0	0.00487	0.007221	0.00299	0.001095	0	0	0.001853621
Protein Metabolism	Protein biosynthesis	Ribosome LSU eukaryotic and archaeal	LSU ribosomal protein L23Ae (L23p)	0	0	0	0	0.00197	0.000487	0	0.000498	0.000219	0	0	0.000670459
Protein Metabolism	Protein biosynthesis	Ribosome LSU eukaryotic and archaeal	LSU ribosomal protein L27Ae (L15p)	0	0.002262	0	0	0	0.000487	0.000722	0.000997	0	0	0	0.000157755
Protein Metabolism	Protein biosynthesis	Ribosome LSU eukaryotic and archaeal	LSU ribosomal protein L30e	0	0	0	0	0	0.000487	0.000722	0.000498	0.000219	0	0	0.00031551
Protein Metabolism	Protein biosynthesis	Ribosome LSU eukaryotic and archaeal	LSU ribosomal protein L31e	0.002785	0	0	0	0.000657	0	0.000722	0.000498	0	0	0	0.000157755
Protein Metabolism	Protein biosynthesis	Ribosome LSU eukaryotic and archaeal	LSU ribosomal protein L44e	0	0	0	0	0.001313	0.000974	0.000722	0	0.001095	0	0	0.000354949
Regulation and Cell signaling	NULL	DNA-binding regulatory proteins, strays	LysR family transcriptional regulator PA0133	0	0	0	0	0	0	0.000722	0.000997	0.001095	0	0.059265	0.003312855
Regulation and Cell signaling	NULL	DNA-binding regulatory proteins, strays	LysR family transcriptional regulator YbhD	0	0	0	0	0.001313	0.000487	0	0.000498	0.002189	0	0	0.000157755
Carbohydrates	Organic acids	Malonate decarboxylase	Malonate decarboxylase alpha subunit	0.002785	0	0	0	0	0	0	0	0.000438	0.004406	0.000349	0.003628365
Clustering-based subsystems	NULL	Maltose utilization cluster	Maltodextrin phosphorylase (EC 2.4.1.1)	0.000696	0	0	0	0	0	0	0.000498	0.000876	0	0.000233	0.001222601
Carbohydrates	Monosaccharides	Mannose Metabolism	Mannoside ABC transport system, ATP-binding protein 1	0.002437	0.006786	0	0	0.000657	0	0	0	0.000876	0	0	0.000591581
Miscellaneous	Plant-Prokaryote DOE project	Synechocystis experimental	Menaquinone biosynthesis related protein, putative DHNA-CoA thioesterase	0	0.002262	0.007664	0	0.000657	0	0	0	0.001533	0	0	7.88775E-05
Cofactors, Vitamins, Prosthetic Groups, Pigments	Quinone cofactors	Menaquinone Biosynthesis via Futalosine -- gjo	Menaquinone via futalosine step 2	0	0	0	0.00249	0.000657	0.001948	0	0	0.000219	0	0	0.006191885
Virulence, Disease and Defense	Resistance to antibiotics and toxic compounds	Mercury resistance operon	Mercuric transport protein, MerT	0	0	0	0	0	0.000487	0	0	0.000876	0.008811	0.000699	0.000512704
Miscellaneous	Plant-Prokaryote DOE project	COG0523	Methanol methyltransferase corrinoid protein	0	0	0.002555	0	0.003939	0	0	0.000997	0.000219	0	0	0.000354949
Amino Acids and Derivatives	Lysine, threonine, methionine, and cysteine	Methionine Biosynthesis	Methionine repressor MetJ	0	0	0	0	0	0.001461	0.001444	0	0.003722	0	0.002329	3.94388E-05
Amino Acids and Derivatives	Lysine, threonine, methionine, and cysteine	Methionine Biosynthesis	Methionine synthase activation domain (EC 2.1.1.13)	0.001393	0.004524	0	0	0	0	0.001444	0.000997	0	0	0	0.00031551
Respiration	Electron donating reactions	Respiratory dehydrogenases 1	Methylamine dehydrogenase light chain precursor (EC 1.4.99.3)	0.002785	0.002262	0	0	0	0.000974	0	0	0.000219	0	0.000116	0
Carbohydrates	Sugar alcohols	Inositol catabolism	Minor myo-inositol transporter IolF	0.000696	0	0	0.00249	0	0.000974	0.000722	0	0	0	0	0.000512704
Cofactors, Vitamins, Prosthetic Groups, Pigments	Folate and pterines	Molybdenum cofactor biosynthesis	Molybdopterin binding domain MoeA-like	0.002437	0	0	0	0.007222	0.002435	0.000722	0	0	0	0	0.00157755
RNA Metabolism	RNA processing and modification	rRNA modification Archaea	N1-specific pseudouridine methyltransferase NEP1, biosynthesis of m1acp3-psi in 18S/16S	0	0	0	0	0.00197	0.000487	0	0.001495	0	0	0.000233	7.88775E-05

			rRNA in Eukaryotes and Archaea												
Carbohydrates	Aminosugars	Chitin and N-acetylglucosamine utilization	N-acetylglucosamine kinase of eukaryotic type (EC 2.7.1.59)	0	0	0	0	0	0.000487	0	0.000997	0.000438	0	0.004192	0.000197194
Cell Wall and Capsule	Capsular and extracellular polysacchrides	Sialic Acid Metabolism	N-acetylmannosamine-6-phosphate 2-epimerase (EC 5.1.3.9)	0	0	0	0.00498	0.000657	0.000487	0	0	0.000438	0	0	0.000118316
Cell Wall and Capsule	Capsular and extracellular polysacchrides	Sialic Acid Metabolism	N-acetylneuraminate lyase (EC 4.1.3.3)	0.000348	0.004524	0	0	0.001313	0	0	0	0.001095	0	0	0.002326887
Miscellaneous	NULL	Muconate lactonizing enzyme family	N-acylamino acid racemase	0	0	0	0	0	0.000974	0.001444	0.000997	0.002627	0	0	0.001340918
Respiration	Electron donating reactions	Respiratory Complex I	NAD(P)H-quinone oxidoreductase chain K (EC 1.6.5.2)	0	0	0	0	0	0	0.000722	0.000997	0.002408	0	0.000931	0.000354949
Stress Response	Oxidative stress	Redox-dependent regulation of nucleus processes	NAD-dependent deacetylase	0	0	0	0	0.000657	0	0.000722	0.000498	0.000219	0	0	0.000197194
Respiration	Electron donating reactions	Respiratory Complex I	NADH-ubiquinone oxidoreductase chain G2 (EC 1.6.5.3)	0.000696	0	0.002555	0	0.000657	0	0	0	0.001095	0	0	0.000236633
Virulence, Disease and Defense	Adhesion	Streptococcus pyogenes recombinatorial zone	Negative transcriptional regulator	0	0	0	0	0	0.000974	0.001444	0.001495	0.000657	0	0	0.000354949
Respiration	Electron donating reactions	Hydrogenases	Ni,Fe-hydrogenase III small subunit	0	0	0.00511	0.00498	0.001313	0	0	0	0.000657	0	0	0.000433826
Respiration	NULL	Methanogenesis strays	Ni2+-binding GTPase involved in regulation of expression and maturation of hydrogenase	0	0	0	0.00249	0.001313	0.000974	0	0.002492	0	0	0	0.001340918
Membrane Transport	NULL	Transport of Nickel and Cobalt	Nickel transporter UreH	0	0	0	0	0.000657	0.001461	0.001444	0.00299	0	0	0	7.88775E-05
Cofactors, Vitamins, Prosthetic Groups, Pigments	NAD and NADP	NAD and NADP cofactor biosynthesis global	Nicotinamide-nucleotide adenylyltransferase, NadR family (EC 2.7.7.1)	0	0	0	0	0	0.000487	0	0.000997	0.000438	0	0.000233	0.00063102
Nitrogen Metabolism	NULL	Nitrogen fixation	Nitrogenase (vanadium-iron) reductase and maturation protein VnfH	0	0	0.002555	0	0	0	0.001444	0.000498	0.000219	0	0	0.000433826
Cofactors, Vitamins, Prosthetic Groups, Pigments	NAD and NADP	NAD and NADP cofactor biosynthesis global	NMN 5'-nucleotidase, extracellular (EC 3.1.3.5)	0	0	0	0	0.001313	0.000974	0.001444	0	0.000219	0	0	0.000394388
Carbohydrates	Monosaccharides	Xylose utilization	Novel Xylose regulator from LacI family	0	0	0	0	0	0.000974	0.002888	0.000498	0.001971	0	0	0.000709898
Secondary Metabolism	Bacterial cytostatics, differentiation factors and antibiotics	Nonribosomal peptide synthetases (NRPS) in Frankia sp. Ccl3	NRPS module 4 PG-Ser-Gly-Thr	0	0	0.002555	0	0	0	0.002166	0.001495	0.001314	0	0	0.000591581
RNA Metabolism	Transcription	Transcription elongation factors, archaeal	NusA protein homolog, archaeal	0	0.002262	0	0	0	0	0.00361	0.000997	0.001533	0	0	0.000197194
Miscellaneous	Plant-Prokaryote DOE project	At1g52510 AT4G12830 (COG0596)	omega-3 polyunsaturated fatty acid synthase module PfaB/C	0	0	0	0	0	0.000974	0.000722	0.000498	0.000876	0	0	0.000276071
Metabolism of Aromatic Compounds	Peripheral pathways for catabolism of aromatic compounds	Benzoate degradation	Ortho-halobenzoate 1,2-dioxygenase alpha-ISP protein OhbB	0	0	0	0	0	0.000487	0	0.000997	0.000219	0	0.000233	0.000157755
Stress Response	Osmotic stress	Osmoprotectant ABC transporter YehZYXW of Enterobacteriales	Osmoprotectant ABC transporter ATP-binding subunit YehX	0	0	0.002555	0	0	0	0.000722	0	0.000438	0.002203	0	0.000276071
Stress Response	Osmotic stress	Choline and Betaine Uptake and Betaine Biosynthesis	Osmotically activated L-carnitine/choline ABC transporter, permease protein OpuCB	0	0	0	0	0	0	0.000722	0.000498	0.001971	0.002203	0	0.000670459
Stress Response	Osmotic stress	Choline and Betaine Uptake and Betaine Biosynthesis	Osmotically activated L-carnitine/choline ABC transporter, permease protein OpuCD	0	0	0	0	0	0.000487	0	0.000498	0.000438	0.002203	0	0.000157755
Cell Wall and Capsule	Gram-Negative cell wall components	Lipopolysaccharide assembly	Outer membrane chaperone Skp (OmpH) precursor	0.002437	0	0	0.00498	0	0	0	0	0.000219	0	0.000116	0.000433826
Clustering-based subsystems	NULL	Staphylococcus aureus hypothetical repetitive gene loci	Oxidoreductase ion channel	0	0.002262	0	0	0.002626	0.000974	0	0.000498	0	0	0	0.000552143
Metabolism of Aromatic Compounds	NULL	p-cymene degradation	p-cumate dioxygenase large subunit (CmtAb)	0	0.002262	0	0	0	0	0.000722	0.001495	0.000657	0	0	0.000157755
Regulation and Cell signaling	Quorum sensing and biofilm formation	Symbiotic colonization and sigma-dependent biofilm formation gene cluster	Periplasmic protein SypC involved in polysaccharide export	0	0	0	0	0	0.000974	0.002166	0.001993	0	0	0.008849	0.000157755
Respiration	Electron accepting reactions	trimethylamine N-oxide (TMAO) reductase	Periplasmic protein torT precursor	0	0	0	0	0.002626	0.001948	0.000722	0.000997	0	0	0	0.00031551
Stress Response	Oxidative stress	Oxidative stress	Peroxide stress regulator PerR, FUR family	0.000348	0.002262	0	0	0.00197	0.000974	0	0	0	0	0	0.000394388
Potassium metabolism	NULL	pH adaptation potassium efflux system	pH adaptation potassium efflux system a	0	0	0	0	0	0.000487	0.000722	0.002492	0.001095	0	0	0.000118316
Potassium metabolism	NULL	pH adaptation potassium efflux system	pH adaption potassium efflux system protein PhaD	0	0	0	0	0	0	0.002888	0.003987	0.001752	0	0.000116	0.00031551
Phages, Prophages, Transposable elements, Plasmids	Phages, Prophages	Phage regulation of gene expression	Phage antirepressor	0	0	0	0	0	0.000974	0.000722	0.001495	0.000219	0	0	0.000394388
Phages, Prophages, Transposable elements, Plasmids	Phages, Prophages	Phage neck proteins	Phage collar	0	0	0	0	0.00197	0.002922	0.002888	0.000997	0	0	0	0.000118316
Phages, Prophages, Transposable elements, Plasmids	Phages, Prophages	Phage introns	Phage intron	0	0	0	0	0	0.001461	0.001444	0.00299	0.000438	0	0	0.000118316
Phages, Prophages, Transposable elements, Plasmids	Phages, Prophages	r1t-like streptococcal phages	Phage minor tail protein	0	0	0	0	0	0.003409	0.002888	0.001993	0.000219	0	0	0.000118316
Phages, Prophages, Transposable elements, Plasmids	Phages, Prophages	Phage nin genes - N-independent survival	Phage NinG rap recombination	0	0	0	0	0.000657	0.000487	0.001444	0.002492	0	0	0	3.94388E-05
Phages, Prophages, Transposable elements, Plasmids	Phages, Prophages	Phage entry and exit	Phage rIIA lysis inhibitor	0	0	0	0	0.00197	0.003896	0.000722	0.004485	0	0	0	7.88775E-05
Stress Response	NULL	Phage shock protein (psp) operon	Phage shock protein E	0	0	0	0	0	0.000487	0	0.001495	0.001314	0	0.000116	3.94388E-05
Phages, Prophages, Transposable elements, Plasmids	Phages, Prophages	Phage tail proteins 2	Phage tail assembly	0	0	0	0	0.001313	0.001948	0.000722	0.000997	0	0	0	7.88775E-05
Phages, Prophages, Transposable elements, Plasmids	Phages, Prophages	Phage tail proteins 2	Phage tail length tape-measure protein 1	0	0	0	0	0.000657	0.000487	0.000722	0.004485	0	0	0	3.94388E-05
Metabolism of Aromatic Compounds	Peripheral pathways for catabolism of aromatic compounds	Phenol hydroxylase	Phenol hydroxylase, P1 oxygenase component DmpL (EC 1.14.13.7)	0	0	0	0	0	0	0	0.000997	0.000657	0.008811	0.009664	0.002484642
Metabolism of Aromatic Compounds	Peripheral pathways for catabolism of aromatic compounds	Phenol hydroxylase	Phenol hydroxylase, P2 regulatory component DmpM (EC 1.14.13.7)	0	0	0	0.00249	0	0	0	0.000498	0.001314	0	0.026663	0.001695866
Cell Wall and Capsule	Cell wall of Mycobacteria	mycolic acid synthesis	PHENOLPTHIOCEROL SYNTHESIS TYPE-I POLYKETIDE SYNTHASE PPSD	0	0	0.00511	0	0	0	0.002166	0	0.001314	0	0.000233	0.000433826
Cell Wall and Capsule	Cell wall of Mycobacteria	mycolic acid synthesis	PHENOLPTHIOCEROL SYNTHESIS TYPE-I POLYKETIDE SYNTHASE PPSE	0.003133	0	0	0	0.000657	0.000487	0	0	0.000657	0	0	0.000118316
Amino Acids and Derivatives	Aromatic amino acids and derivatives	Aromatic amino acid degradation	Phenylalanine hydroxylase transcriptional activator PhhR	0.000696	0	0	0	0	0	0	0	0.001752	0.004406	0.000466	0.003904437
Phosphorus Metabolism	NULL	Alkylphosphonate utilization	PhnH protein	0	0	0	0	0.000657	0.000487	0	0.001495	0.000219	0	0	0.000276071
Carbohydrates	Aminosugars	Chitin and N-acetylglucosamine utilization	Phosphatase NagD predicted to act in N-acetylglucosamine utilization subsystem	0	0.004524	0	0	0	0	0.000722	0.001993	0.001314	0	0	0.000552143
Membrane Transport	NULL	Phosphoglycerate transport system	Phosphoglycerate transport system transcriptional regulatory protein PgtA	0.000348	0	0	0	0	0	0	0.000498	0.000219	0	0.000349	0.000749336
Photosynthesis	Electron transport and photophosphorylation	Photosystem I	photosystem I assembly related protein Ycf3	0	0	0	0	0	0	0	0.000498	0.001533	0.002203	0.000699	7.88775E-05
Respiration	NULL	Soluble cytochromes and functionally related electron carriers	Photosystem II protein PsbV, cytochrome c550	0	0	0	0	0	0.000487	0	0.000498	0.000219	0	0.001281	3.94388E-05
Photosynthesis	Light-harvesting complexes	Phycobilisome	Phycocyanin alpha phycocyanobilin lyase related protein NblB	0	0	0	0	0.000657	0.001461	0	0.000498	0.001971	0	0.000582	0
Miscellaneous	Plant-Prokaryote DOE project	At2g23840	Phytoene/squalene synthetase-like protein	0	0	0	0	0.002626	0	0.000722	0	0.000657	0	0.000233	0.000118316
Amino Acids and Derivatives	Glutamine, glutamate, aspartate, asparagine; ammonia assimilation	Poly-gamma-glutamate biosynthesis	Poly-gamma-glutamate synthase subunit PgsA/CapA (EC 6.3.2.-)	0	0	0	0	0.000657	0.000487	0.001444	0	0.000219	0	0	0.000394388
Dormancy and Sporulation	NULL	Spore pigment biosynthetic cluster in Actinomycetes	Polyketide beta-ketoacyl synthase WhiE-KS	0	0	0	0	0.000657	0.000487	0.001444	0	0.000219	0	0	3.94388E-05
Dormancy and Sporulation	NULL	Spore pigment biosynthetic cluster in Actinomycetes	Polyketide hydroxylase WhiE VIII	0.000348	0	0.002555	0.00249	0	0	0	0	0.000219	0	0	3.94388E-05
Miscellaneous	Plant-Prokaryote DOE project	Synechocystis experimental	Possible alpha/beta hydrolase superfamily, slr1235 homolog	0	0	0	0	0.001313	0.000487	0	0.001993	0.003065	0	0.001397	0

Miscellaneous	Plant-Prokaryote DOE project	Synechocystis experimental	Possible alpha/beta hydrolase superfamily, slr1827 homolog	0	0	0	0	0.000657	0	0.000722	0	0.001314	0	0.000233	3.94388E-05
Carbohydrates	Monosaccharides	Xylose utilization	Possible alpha-xyloside ABC transporter, permease component	0	0	0	0.00249	0.000657	0.000487	0	0	0.000876	0	0	0.001064846
Carbohydrates	CO2 fixation	CO2 uptake, carboxysome	Possible carbon dioxide concentrating mechanism protein CcmK	0	0	0.002555	0	0	0.000974	0	0	0.001971	0	0.000233	0.000118316
Carbohydrates	Sugar alcohols	Erythritol utilization	Possible D-erythrulose 4-phosphate dehydrogenase EryC (EC 1.1.1.-)	0	0	0	0	0	0.000487	0	0.000498	0.000657	0.004406	0	0.000276071
Nucleosides and Nucleotides	Pyrimidines	Novel non-oxidative pathway of Uracil catabolism	Possible malonate-semialdehyde dehydrogenase in pyrimidine catabolism	0.002437	0	0	0	0.001313	0	0.000722	0.000997	0	0	0	0.000197194
Nucleosides and Nucleotides	Pyrimidines	Pyrimidine utilization	Predicted amidohydrolase RutB in novel pyrimidine catabolism pathway	0.002437	0	0	0.00249	0.000657	0.000974	0	0	0	0	0	0.000985969
Membrane Transport	NULL	Transport of Nickel and Cobalt	Predicted cobalt transporter CbtC	0	0	0	0	0.005252	0.001948	0	0.000498	0.000657	0	0	0.000433826
Carbohydrates	Sugar alcohols	Erythritol utilization	Predicted erythritol ABC transporter 1, substrate-binding component	0	0.002262	0	0	0	0.000487	0	0	0.001095	0	0.000116	0.000197194
Carbohydrates	NULL	Uncharacterized sugar kinase cluster (ygc)	Predicted FAD containing dehydrogenase	0	0	0	0	0.000657	0.002922	0	0	0.000219	0	0.000233	0.000552143
Carbohydrates	Monosaccharides	D-gluconate and ketogluconates metabolism	Predicted gluconate TRAP family transporter, DctQ subunit	0	0	0	0	0	0.001461	0	0.000498	0.000657	0	0.000116	0.000394388
Carbohydrates	Aminosugars	Chitin and N-acetylglucosamine utilization	Predicted N-acetyl-glucosamine kinase 2, ROK family (EC 2.7.1.59)	0.001393	0	0.002555	0	0.000657	0	0	0	0.000438	0	0	0.000591581
Amino Acids and Derivatives	Proline and 4-hydroxyproline	Proline, 4-hydroxyproline uptake and utilization	Predicted regulator PutR for proline utilization, GntR family	0	0	0	0	0.000657	0.000487	0	0.000997	0.000219	0	0	0.00220857
Membrane Transport	NULL	Transport of Zinc	Predicted zinc-binding protein	0	0	0	0	0.000657	0.000487	0.002888	0.000498	0	0	0	0.000118316
RNA Metabolism	RNA processing and modification	Spliceosome	Pre-mRNA splicing factor PRP8	0	0	0	0	0.000657	0	0.001444	0	0.000657	0	0.001281	0.001025408
Amino Acids and Derivatives	Aromatic amino acids and derivatives	Chorismate Synthesis	Prephenate dehydrogenase (EC 1.3.1.12)	0	0	0	0.00249	0.000657	0.000487	0	0.000997	0	0	0	0.000157755
DNA Metabolism	DNA replication	DNA-replication	Primosomal replication protein N	0	0	0	0	0	0.000487	0.001444	0.000498	0.001971	0	0	0.000118316
Clustering-based subsystems	NULL	CBSS-83332.1.peg.3803	Probable acyl-CoA dehydrogenase fadE35 (EC 1.3.99.-)	0	0.002262	0.002555	0	0	0	0	0.000498	0.002189	0	0	0.000433826
Respiration	Electron donating reactions	Na(+)-translocating NADH-quinone oxidoreductase and rnf-like group of electron transport complexes	probable exported protein YPO3233	0	0	0	0	0	0.000487	0.000722	0.001495	0.002189	0	0	0.001419795
Metabolism of Aromatic Compounds	Peripheral pathways for catabolism of aromatic compounds	Phenylpropanoid compound degradation	Probable vanillate O-demethylase oxygenase subunit oxidoreductase protein (EC 1.14.13.-)	0	0	0	0	0.001313	0	0	0.000498	0.005693	0	0.015486	0.000552143
Regulation and Cell signaling	Programmed Cell Death and Toxin-antitoxin Systems	MazEF toxin-antitoxing (programmed cell death) system	Programmed cell death antitoxin YdcD	0	0	0	0	0.00197	0	0	0.001495	0.001971	0.004406	0	0.00031551
Virulence, Disease and Defense	NULL	Streptococcus pyogenes Virulome	Protein F	0	0.002262	0	0	0	0	0	0.000997	0.003284	0	0.000233	0.00094653
RNA Metabolism	RNA processing and modification	Ribonuclease H	Protein often found in Actinomycetes clustered with signal peptidase and/or RNaseHII	0	0.004524	0	0	0	0.000487	0.000722	0.000498	0	0	0	0.000236633
Clustering-based subsystems	NULL	USS-DB-7	Protein phosphatase ImpM	0.002089	0	0	0.00249	0.002626	0	0	0	0.000219	0	0	0.000512704
Clustering-based subsystems	NULL	PFGI-1-like cluster 1	Protein with ParB-like nuclease domain in PFGI-1-like cluster	0	0	0.00511	0	0.001313	0.000487	0	0	0.001095	0	0	0.00063102
Photosynthesis	NULL	Proteorhodopsin	Proteorhodopsin	0	0	0	0	0	0.001461	0	0.001495	0.000219	0	0.000116	0.000118316
Cell Wall and Capsule	Capsular and extracellular polysacchrides	Pseudaminic Acid Biosynthesis	Pseudaminic acid biosynthesis protein PseA, possible Pse5Ac7Ac acetamidino synthase	0	0	0.00511	0	0.000657	0	0.000722	0.000997	0	0	0	0.001340918
Carbohydrates	Sugar alcohols	Mannitol Utilization	PTS system, mannitol-specific IIB component (EC 2.7.1.69)	0.000348	0	0	0	0.001313	0	0.001444	0	0	0	0.000582	0.001104285
Clustering-based subsystems	NULL	Ycd cluster (putative 2-hydroxyacid dehydrogenase)	Putative 2-hydroxyacid dehydrogenase YcdW (EC 1.-.-.-)	0.000696	0	0	0	0	0.000487	0	0.000997	0.000657	0.002203	0	0
Clustering-based subsystems	Clustering-based subsystems	Putative diaminopropionate ammonia-lyase cluster	Putative diaminopropionate ammonia-lyase (EC 4.3.1.15)	0	0	0	0	0.000657	0.001948	0.001444	0.001993	0	0	0	0.001104285
Clustering-based subsystems	NULL	CBSS-342610.3.peg.1794	Putative esterase, FIGfam005057	0	0.006786	0	0	0	0	0	0.001495	0.000438	0	0.007568	0.002800152
Sulfur Metabolism	Organic sulfur assimilation	Utilization of glutathione as a sulphur source	Putative glutathione transporter,solute-binding component	0.002785	0.002262	0	0	0	0.001948	0	0	0.002189	0	0	0.000867653
Cell Wall and Capsule	Capsular and extracellular polysacchrides	Rhamnose containing glycans	putative glycosyltransferase - possibly involved in cell wall localization and side chain formation of rhamnose-glucose polysaccharide	0	0.004524	0	0	0.000657	0.000487	0	0	0.001533	0	0	0.000788775
Clustering-based subsystems	Biosynthesis of galactoglycans and related lipopolysacharides	CBSS-258594.1.peg.3339	Putative glycosyltransferase protein	0	0	0	0.00249	0.001313	0	0.000722	0	0.000219	0	0	0.000670459
Clustering-based subsystems	Putative Isoquinoline 1-oxidoreductase subunit	CBSS-314267.3.peg.390	Putative Isoquinoline 1-oxidoreductase subunit, Mll3835 protein	0	0	0.00511	0	0	0	0	0.002492	0.002189	0	0.000116	0.000276071
Carbohydrates	Central carbohydrate metabolism	Pyruvate metabolism II: acetyl-CoA, acetogenesis from pyruvate	Putative membrane protein, clustering with ActP	0	0.002262	0	0	0.001313	0.000487	0	0.000997	0	0	0	0.002484642
Phages, Prophages, Transposable elements, Plasmids	Phages, Prophages	r1t-like streptococcal phages	putative methylase	0.002785	0	0	0	0	0.001948	0.000722	0	0.000657	0	0	0.00126204
Phages, Prophages, Transposable elements, Plasmids	Transposable elements	Conjugative transposon, Bacteroidales	Putative mobilization protein BF0133	0	0	0.002555	0	0.000657	0	0	0.000498	0	0	0.000582	0.000433826
Nucleosides and Nucleotides	Detoxification	Nudix proteins (nucleoside triphosphate hydrolases)	Putative Nudix hydrolase YfcD (EC 3.6.-.-)	0	0	0	0	0.003283	0.001948	0.001444	0.001993	0	0	0	0.000512704
Membrane Transport	NULL	Ton and Tol transport systems	Putative OMR family iron-siderophore receptor precursor	0	0	0	0	0	0.000487	0.000722	0.000498	0.000657	0	0	0.000473265
Carbohydrates	NULL	Unknown carbohydrate utilization (cluster Ydj)	Putative oxidoreductase YeaE, aldo/keto reductase family	0	0	0	0	0.000657	0	0.000722	0	0.000219	0	0.000116	3.94388E-05
Metabolism of Aromatic Compounds	Peripheral pathways for catabolism of aromatic compounds	Phenylpropanoid compound degradation	Putative phthalate 4,5-dioxygenase oxygenase subunit (OhpA2)	0	0	0	0	0	0	0.000722	0.000498	0.001314	0.002203	0	0.000276071
Miscellaneous	Plant-Prokaryote DOE project	DOE COG2016	Putative snRNP Sm-like protein	0	0	0.002555	0.00249	0	0	0.005055	0.000498	0	0	0	0.00031551
Cofactors, Vitamins, Prosthetic Groups, Pigments	Pyridoxine	Pyridoxin(Vitamin B6) Degradation Pathway	Pyridoxal 4-dehydrogenase (EC 1.1.1.107)	0	0.002262	0	0	0	0	0.000722	0.000997	0.000876	0	0	0.000236633
Protein Metabolism	Protein biosynthesis	Pyrrolysine	Pyrrolysyl-tRNA synthetase (EC 6.1.1.26)	0.006963	0	0	0.00249	0.00197	0.003409	0	0	0	0	0	0.001025408
Regulation and Cell signaling	Programmed Cell Death and Toxin-antitoxin Systems	Toxin-antitoxin replicon stabilization systems	RelB/StbD replicon stabilization protein (antitoxin to RelE/StbE)	0	0	0	0	0.00197	0.002435	0	0.000498	0.000438	0	0	0.003312855
Phages, Prophages, Transposable elements, Plasmids	Transposable elements	Tn552	Resolvase/integrase Bin	0	0	0	0	0.001313	0.000974	0	0	0.000219	0	0.000349	0.002445203
Phages, Prophages, Transposable elements, Plasmids	Transposable elements	CBSS-203122.12.peg.188	Resolvase/Integrase TinR protein	0	0	0	0	0.00197	0.000487	0	0	0	0.002203	0.000116	0.000433826
Clustering-based subsystems	NULL	USS-DB-2	Response regulator, ompR family [USSDB2A]	0.005919	0.002262	0	0	0	0	0.001444	0.000498	0	0	0	0.000433826
Miscellaneous	Plant-Prokaryote DOE project	Single-Rhodanese-domain proteins	Rhodanese domain protein UPF0176, Firmicutes subgroup	0	0	0	0	0	0.000974	0.000722	0.00299	0.001314	0	0	0.000591581
RNA Metabolism	Transcription	Transcription initiation, bacterial sigma factors	RNA polymerase principal sigma factor HrdC	0.000348	0	0	0	0	0	0.000722	0.000498	0.000219	0	0	3.94388E-05
Phages, Prophages, Transposable elements, Plasmids	Phages, Prophages	Phage replication	RNA polymerase, phage-associated	0	0	0	0	0.005252	0.003409	0.001444	0.001495	0	0	0	0.00031551
Stress Response	NULL	SigmaB stress responce regulation	RsbS, negative regulator of sigma-B	0	0	0	0	0	0.001461	0.000722	0.000997	0.001533	0	0	7.88775E-05
Phages, Prophages, Transposable elements, Plasmids	Phages, Prophages	Prophage-encoded Rst operon	RstR phage-related transcriptional repressor	0	0	0	0	0.002626	0.000974	0.001444	0.000498	0	0	0	0.000157755
Clustering-based subsystems	Biosynthesis of galactoglycans and related lipopolysacharides	CBSS-258594.1.peg.3339	Secretion protein, HlyD family	0	0	0	0	0	0.000487	0.001444	0.000498	0.000219	0	0	0.000118316
Carbohydrates	Monosaccharides	Hexose Phosphate Uptake System	Sensor histidine protein kinase UhpB, glucose-6-phosphate specific (EC 2.7.13.3)	0	0	0	0	0.001313	0.000974	0	0.000997	0.000876	0	0	0.001340918

Regulation and Cell signaling	NULL	Orphan regulatory proteins	Sensor protein basS/pmrB (EC 2.7.3.-)	0	0	0.002555	0.00249	0	0	0	0	0.000876	0.002203	0	0.001735305
Membrane Transport	Protein secretion system, Type VII (Chaperone/Usher pathway, CU)	sigma-Fimbriae	Sigma-fimbriae tip adhesin	0.002785	0	0	0	0	0.000487	0.000722	0	0	0.002203	0	3.94388E-05
Membrane Transport	Protein secretion system, Type II	Widespread colonization island	Similar to secretin RcpA/CpaC, associated with Flp pilus assembly	0	0	0	0	0	0	0	0.000498	0.000219	0.004406	0.000116	7.88775E-05
Miscellaneous	Plant-Prokaryote DOE project	At4g17370	Similarity to Na+/myo-inositol cotransporter	0	0	0.002555	0	0.000657	0	0	0	0.001095	0.004406	0	0.000276071
Stress Response	Oxidative stress	Glutathionylspermidine and Trypanothione	Similarity with glutathionylspermidine synthase (EC 6.3.1.8), group 1	0	0	0	0	0	0.000974	0	0.000498	0	0.013217	0.000233	0.000118316
Stress Response	Oxidative stress	Glutathionylspermidine and Trypanothione	Similarity with glutathionylspermidine synthase (EC 6.3.1.8), group 2	0	0	0	0	0.000657	0.000487	0.000722	0	0	0	0.000233	0.007414486
DNA Metabolism	DNA repair	DNA Repair Base Excision	Single-stranded-DNA-specific exonuclease RecJ, clostridial paralog	0.000348	0	0	0.00498	0	0	0.001444	0.000498	0	0	0	0.000394388
Stress Response	Oxidative stress	Glutathione analogs: mycothiol	S-nitrosomycothiol reductase MscR	0	0	0	0.00747	0.001313	0.001461	0	0	0.001533	0	0	0.00094653
Carbohydrates	Sugar alcohols	Inositol catabolism	Sodium/myo-inositol cotransporter	0.000348	0.002262	0	0	0	0	0	0.000498	0.000438	0	0	0.000907091
Fatty Acids, Lipids, and Isoprenoids	Isoprenoids	Polyprenyl Diphosphate Biosynthesis	Solanesyl diphosphate synthase (EC 2.5.1.11)	0.002785	0	0	0	0.000657	0	0	0	0.000876	0	0.000466	0.000157755
Respiration	NULL	Soluble cytochromes and functionally related electron carriers	soluble [2Fe-2S] ferredoxin	0.000696	0	0	0	0	0	0	0.000997	0.004379	0	0.000233	3.94388E-05
Photosynthesis	NULL	Proteorhodopsin	Spheroidene monooxygenase	0	0	0	0	0.000657	0.001948	0	0.000997	0.001971	0	0	0.000197194
Regulation and Cell signaling	NULL	WhiB and WhiB-type regulatory proteins	Sporulation regulatory protein WhiB	0	0	0	0.00498	0	0	0.000722	0.000997	0.000438	0	0	3.94388E-05
Protein Metabolism	Protein biosynthesis	Ribosome SSU eukaryotic and archaeal	SSU ribosomal protein S27e	0.001044	0	0	0.00249	0	0	0.001444	0.000498	0	0	0	0.000354949
Dormancy and Sporulation	NULL	Sporulation draft	Stage 0 sporulation protein YaaT	0	0	0	0	0.000657	0	0.000722	0.000498	0	0	0.000116	0.00031551
Dormancy and Sporulation	NULL	Sporulation Cluster	Stage II sporulation protein D (SpoIID)	0	0	0	0	0	0.000974	0.001444	0.000498	0.001095	0	0	0.000867653
Stress Response	NULL	Carbon Starvation	Starvation lipoprotein Slp	0	0	0	0.00249	0	0	0.000722	0	0.002408	0.002203	0	0.000118316
Membrane Transport	NULL	ECF class transporters	Substrate-specific component ThiT of thiamin ECF transporter	0	0	0.00511	0	0	0.000487	0.000722	0.000997	0	0	0	0.000276071
Metabolism of Aromatic Compounds	Anaerobic degradation of aromatic compounds	Anaerobic toluene and ethylbenzene degradation	Succinyl-CoA:(R)-benzylsuccinate CoA-transferase subunit BbsE (EC 2.8.3.15)	0	0	0	0.00249	0	0.001461	0	0	0.000219	0	0.000116	0.000670459
Clustering-based subsystems	NULL	USS-DB-1	Sugar ABC transporter, sugar permease protein 2 USSDB1D	0.002437	0	0	0	0.000657	0	0	0.000997	0.001533	0	0	0.00126204
Sulfur Metabolism	Inorganic sulfur assimilation	Inorganic Sulfur Assimilation	Sulfate and thiosulfate binding protein CysP	0	0	0.002555	0	0	0	0.000722	0	0.000438	0	0.000582	0.001814183
Respiration	Electron donating reactions	Hydrogenases	Sulfhydrogenase II subunit a	0	0.002262	0	0	0.000657	0.000487	0	0.000498	0	0	0	0.000157755
Respiration	Electron donating reactions	Hydrogenases	Sulfhydrogenase II subunit b	0.002785	0	0	0	0.000657	0.000974	0	0.000498	0	0	0	0.000276071
Virulence, Disease and Defense	Resistance to antibiotics and toxic compounds	Resistance to chromium compounds	Superoxide dismutase SodM-like protein ChrF	0	0	0.00511	0	0	0.000487	0	0	0.002189	0.002203	0	0.00126204
Phages, Prophages, Transposable elements, Plasmids	Phages, Prophages	Phage capsid proteins	T7-like phage head-to-tail joining protein	0	0	0	0	0.004596	0.002435	0.002888	0.002492	0	0	0	0.000157755
Carbohydrates	Monosaccharides	D-Tagatose and Galactitol Utilization	Tagatose-1,6-bisphosphate aldolase GatY (EC 4.1.2.-)	0	0.002262	0	0	0	0.000487	0.001444	0	0.000219	0	0	0.000394388
Carbohydrates	Di- and oligosaccharides	Lactose and Galactose Uptake and Utilization	Tagatose-6-phosphate kinase (EC 2.7.1.144)	0	0	0	0	0.003283	0.001461	0	0.000498	0.000657	0	0	0.000473265
Carbohydrates	Monosaccharides	D-Tagatose and Galactitol Utilization	Tagatose-6-phosphate kinase GatZ (EC 2.7.1.144)	0	0	0	0	0.000657	0	0	0.000498	0.000219	0	0.000582	0.000236633
Phages, Prophages, Transposable elements, Plasmids	Phages, Prophages	Phage DNA synthesis	Thioredoxin, phage-associated	0	0	0	0	0.00197	0.000974	0.002888	0.000997	0	0	0	7.88775E-05
Respiration	Electron accepting reactions	Anaerobic respiratory reductases	Thiosulfate reductase cytochrome B subunit	0.004178	0	0	0.00249	0	0	0.001444	0	0.000657	0	0	0.000394388
RNA Metabolism	RNA processing and modification	tRNA modification Archaea	TilS and TtcA-like	0	0	0	0	0	0.000487	0.002888	0.001495	0.000438	0	0	0.000552143
Iron acquisition and metabolism	Siderophores	Siderophore assembly kit	TonB-dependent hemin receptor	0	0	0	0	0.000657	0.000487	0	0.000498	0.001314	0	0	0.000197194
Membrane Transport	NULL	Ton and Tol transport systems	TonB-dependent siderophore receptor	0	0	0	0	0	0.000487	0.001444	0.000997	0.000657	0	0	0.00063102
Virulence, Disease and Defense	Resistance to antibiotics and toxic compounds	Multidrug Resistance Efflux Pumps	Transcription regulator of multidrug efflux pump operon, TetR (AcrR) family	0	0	0	0	0.002626	0	0.000722	0.000997	0.000657	0	0	0.001538111
Membrane Transport	Uni- Sym- and Antiporters	NhaA, NhaD and Sodium-dependent phosphate transporters	Transcriptional activator HlyU	0	0	0	0	0	0	0.001444	0.000498	0.000438	0	0.002212	0.001459234
Cell Wall and Capsule	Capsular and extracellular polysacchrides	Sialic Acid Metabolism	Transcriptional regulator NanR	0	0	0	0	0	0.001461	0.000722	0.001495	0.000657	0	0	0.000118316
Carbohydrates	Monosaccharides	L-rhamnose utilization	Transcriptional regulator of rhamnose utilization, LacI family	0	0.002262	0.00511	0	0	0	0	0.000498	0.000876	0	0	0.000197194
Clustering-based subsystems	Putrescine/GABA utilization cluster-temporal,to add to SSs	GABA and putrescine metabolism from cluters	Transcriptional regulator, GABA/putrescine utiliation cluster 1	0.000348	0	0	0	0	0.000974	0	0.000498	0.000438	0	0	0.000118316
Carbohydrates	Monosaccharides	L-Arabinose utilization	Transcriptional repressor of arabinoside utilization operon, GntR family	0	0	0	0	0.000657	0.000487	0	0.000997	0.000438	0	0	0.000118316
Carbohydrates	Monosaccharides	Fructose utilization	Transcriptional repressor of the fructose operon, DeoR family	0	0	0	0	0	0	0.002166	0.001495	0.001314	0.002203	0	0.000433826
Membrane Transport	NULL	ECF class transporters	Transmembrane component of general energizing module of ECF transporters	0	0	0	0	0.000657	0	0.000722	0.000498	0	0	0.000116	0.001183163
Potassium metabolism	NULL	Potassium homeostasis	TrkA-N:Sodium/hydrogen exchanger	0	0	0	0	0.001313	0.001461	0.002888	0	0.003065	0	0	0.000788775
RNA Metabolism	RNA processing and modification	Methylthiotransferases	tRNA N6-threonylcarbamoyladenosine 2-methylthiotransferase (putative)	0	0	0	0.00249	0	0.000974	0	0.000997	0.000657	0	0	0.000394388
RNA Metabolism	RNA processing and modification	tRNA modification Archaea	tRNA pseudouridine 55 synthase Archaea	0.002437	0	0	0	0	0	0.000722	0.001993	0.001314	0	0	0.017313613
Carbohydrates	Central carbohydrate metabolism	Pyruvate metabolism I: anaplerotic reactions, PEP	Two-component sensor histidine kinase, malate (EC 2.7.3.-)	0.002089	0	0	0	0	0.000487	0	0	0.000438	0	0.000116	0.000236633
Membrane Transport	Protein secretion system, Type I	Type I secretion system for aggregation	type I secretion system, outer membrane component (LapE)	0	0	0	0	0	0.001461	0	0.001993	0.000219	0	0.007917	0.000394388
Miscellaneous	NULL	ZZ gjo need homes	Tyrosine-specific transport protein	0	0	0	0	0.001313	0.000487	0.000722	0	0	0	0.000582	0.000433826
Cofactors, Vitamins, Prosthetic Groups, Pigments	Folate and pterines	Pterin biosynthesis	UDP-glucose:tetrahydrobiopterin glucosyltransferase [EC:2.4.1.-]	0	0	0	0	0.001313	0.000487	0.001444	0	0.000438	0	0.000349	0
Clustering-based subsystems	NULL	USS-DB-7	Uncharacterized protein ImpF	0	0	0	0	0.000657	0.000487	0	0.000498	0.001314	0	0	0.000828214
Respiration	NULL	Methanogenesis strays	Uncharacterized protein MJ0282	0.001044	0.004524	0	0	0	0.000487	0	0	0	0	0.000233	0.000236633
RNA Metabolism	RNA processing and modification	tRNA modification Archaea	Uncharacterized protein MJ0319	0.000348	0	0	0	0.001313	0.000974	0	0	0.000438	0	0	0.001025408
Cofactors, Vitamins, Prosthetic Groups, Pigments	Folate and pterines	Methanopterin biosynthesis2	Uncharacterized protein MJ1487	0.002785	0	0	0.00249	0.000657	0.000974	0	0	0	0	0	0.001340918
Clustering-based subsystems	Carbohydrates	Putative sugar ABC transporter (ytf cluster)	Uncharacterized protein YtfN	0	0.004524	0	0	0	0	0	0	0.000438	0.004406	0.000699	0.000197194
Stress Response	NULL	Universal stress protein family	Universal stress protein family 3	0.001044	0	0	0	0	0.000487	0.001444	0.001993	0	0	0	3.94388E-05
DNA Metabolism	DNA repair	Uracil-DNA glycosylase	Uracil-DNA glycosylase, putative family 6	0	0	0	0	0.000657	0.001461	0	0.000498	0.001752	0	0	0.000394388
Miscellaneous	Plant-Prokaryote DOE project	COG0523	Uroporphyrinogen decarboxylase (URO-D)	0	0.002262	0.002555	0	0	0.000487	0	0	0.000219	0	0	0.00063102
Regulation and Cell signaling	NULL	WhiB and WhiB-type regulatory proteins	WhiB-type transcriptional regulator	0	0	0.002555	0	0.000657	0.000974	0	0.000498	0	0	0	3.94388E-05

Carbohydrates	NULL	Sugar utilization in Thermotogales	Xylan oligosaccharide ABC transporter, permease component 1	0	0	0	0.00249	0	0.001461	0.002166	0	0.000657	0	0	0.00126204
Carbohydrates	Monosaccharides	Xylose utilization	Xylose oligosaccharides ABC transporter, ATP-binding protein 1	0	0	0	0	0.000657	0	0.000722	0	0.000876	0	0.000349	0.00094653
Nucleosides and Nucleotides	Purines	De Novo Purine Biosynthesis	YabJ, a purine regulatory protein and member of the highly conserved YjgF family	0	0.002262	0.002555	0.00249	0	0	0	0.000997	0	0	0	0.000433826
Membrane Transport	Protein and nucleoprotein secretion system, Type IV	Conjugative transfer	Ynd	0	0	0	0	0.002626	0.001461	0	0.001993	0.000219	0	0	0.00063102
Miscellaneous	Plant-Prokaryote DOE project	At5g63420	Zn-dependent hydrolase, RNA-metabolising, CPSF 73 kDa analog	0	0	0	0	0	0.000487	0.002888	0.000498	0.000219	0	0	0.000197194
Metabolism of Aromatic Compounds	Anaerobic degradation of aromatic compounds	Anaerobic toluene and ethylbenzene degradation	(R)-benzylsuccinyl-CoA dehydrogenase (EC 1.3.99.21)	0	0	0	0.00249	0.000657	0.000487	0	0	0	0	0	0.000552143
Carbohydrates	Monosaccharides	D-gluconate and ketogluconates metabolism	2,5-diketo-D-gluconic acid reductase B (EC 1.1.1.274)	0	0	0	0	0	0	0	0.000997	0.003284	0	0.001164	0.000236633
Amino Acids and Derivatives	Aromatic amino acids and derivatives	Tryptophan catabolism	2-aminomuconate deaminase (EC 3.5.99.5)	0	0	0	0	0.001313	0	0	0.000498	0.000438	0	0	0.000907091
Amino Acids and Derivatives	Lysine, threonine, methionine, and cysteine	Methionine Salvage	2-hydroxy-3-keto-5-methylthiopentenyl-1-phosphate phosphatase	0	0	0	0	0.001313	0	0.000722	0	0.000219	0	0	0.000197194
Cofactors, Vitamins, Prosthetic Groups, Pigments	Quinone cofactors	Tocopherol Biosynthesis	2-methyl-6-phytyl-1,4-benzoquinone cyclase	0	0	0	0	0.001313	0.000487	0	0.000997	0.000219	0	0	0
Cofactors, Vitamins, Prosthetic Groups, Pigments	Quinone cofactors	Tocopherol Biosynthesis	2-methyl-6-phytyl-1,4-benzoquinone methyltransferase	0	0	0	0	0	0	0.000722	0.000498	0.001533	0	0.000815	0
Amino Acids and Derivatives	Lysine, threonine, methionine, and cysteine	Methionine Degradation	2-Oxobutyrate dehydrogenase E1 (EC:1.2.4.1)	0	0	0	0	0.000657	0	0	0.000498	0.000438	0	0	0.000157755
Cell Wall and Capsule	Cell wall of Mycobacteria	mycolic acid synthesis	3-OXOACYL-[ACYL-CARRIER-PROTEIN] SYNTHASE III FABH (BETA-KETOACYL-ACP SYNTHASE III) (KAS III)	0	0	0	0	0	0	0.000722	0.000498	0	0.006608	0	0.000512704
Metabolism of Aromatic Compounds	Metabolism of central aromatic intermediates	Catechol branch of beta-ketoadipate pathway	3-oxoadipate enol-lactone hydrolase	0	0	0	0	0.000657	0	0	0.000498	0.000876	0	0	0.000907091
Secondary Metabolism	Aromatic amino acids and derivatives	Cinnamic Acid Degradation	3-phenylpropionate dioxygenase ferredoxin subunit	0	0	0	0	0	0.000487	0	0.000498	0.000657	0	0	0.000118316
Phages, Prophages, Transposable elements, Plasmids	Phages, Prophages	Phage DNA synthesis	3'-phosphatase, 5'-polynucleotide kinase, phage-associated	0	0	0	0	0	0.001461	0.000722	0.00299	0	0	0	3.94388E-05
RNA Metabolism	RNA processing and modification	tRNA modification Archaea	5-carboxymethyl uridine and 5-carboxymethyl 2-thiouridine methyltransferase	0	0	0	0	0.000657	0.000487	0	0	0.001095	0	0	0.000197194
Carbohydrates	One-carbon Metabolism	One-carbon metabolism by tetrahydropterines	5-FCL-like protein	0	0	0	0	0	0	0.001444	0.000997	0.002189	0	0	7.88775E-05
Virulence, Disease and Defense	Bacteriocins, ribosomally synthesized antibacterial peptides	Bacitracin Stress Response	ABC transporter ATP-binding protein YvcR	0	0.002262	0	0	0	0	0.000722	0	0.001971	0	0	0.000867653
Miscellaneous	Plant-Prokaryote DOE project	At5g48545 and At3g56490 At1g31160	ABC transporter, ATP-binding protein EcsA	0.000348	0	0	0	0	0	0.001444	0	0.000219	0	0	0.000118316
Sulfur Metabolism	Inorganic sulfur assimilation	Inorganic Sulfur Assimilation	ABC-type probable sulfate transporter, periplasmic binding protein	0	0	0	0	0	0	0.002166	0.000498	0.000438	0	0	7.88775E-05
Clustering-based subsystems	NULL	USS-DB-6	ABC-type transport system involved in resistance to organic solvents, periplasmic component USSDB6C	0	0	0	0	0	0	0	0.000997	0.002189	0	0.000116	0.00031551
Carbohydrates	Fermentation	Acetoin, butanediol metabolism	Acetoin (diacetyl) reductase (EC 1.1.1.5)	0	0.002262	0	0	0	0	0.000722	0.000498	0	0	0	7.88775E-05
Carbohydrates	Fermentation	Acetoin, butanediol metabolism	Acetolactate synthase, catabolic (EC 2.2.1.6)	0	0	0	0	0	0	0.000722	0.001495	0.000438	0	0	0.000157755
Metabolism of Aromatic Compounds	Anaerobic degradation of aromatic compounds	Acetophenone carboxylase 1	Acetophenone carboxylase subunit Apc1	0	0	0	0.00249	0	0	0	0.001495	0.000219	0	0	0.000788775
Metabolism of Aromatic Compounds	Anaerobic degradation of aromatic compounds	Acetophenone carboxylase 1	Acetophenone carboxylase subunit Apc4	0	0.002262	0.002555	0	0	0	0	0	0	0.002203	0	0.001183163
Amino Acids and Derivatives	Aromatic amino acids and derivatives	Tryptophan synthesis	Acting phosphoribosylanthranilate isomerase (EC 5.3.1.24)	0	0.002262	0	0.00498	0	0	0	0.000498	0	0	0	0.000197194
Fatty Acids, Lipids, and Isoprenoids	NULL	Cholesterol catabolic operon in Mycobacteria	Acyl-CoA dehydrogenase IgrC	0.060928	0	0	0	0	0	0.000722	0	0.000219	0	0	0.000788775
Nucleosides and Nucleotides	Purines	Purine conversions	Adenosine kinase (EC 2.7.1.20)	0.003133	0	0	0	0.001313	0	0	0	0.000657	0	0	0.000709898
Stress Response	Osmotic stress	Choline and Betaine Uptake and Betaine Biosynthesis	Alcohol dehydrogenase GbsB (type III), essential for the utilization of choline (EC 1.1.1.1)	0	0.004524	0	0	0	0.001461	0.000722	0	0.000219	0	0	0
Cell Wall and Capsule	Capsular and extracellular polysacchrides	Alginate metabolism	alginate biosynthesis protein AlgJ	0	0	0	0	0.000657	0	0.001444	0.001495	0.000219	0	0	0
Miscellaneous	Plant-Prokaryote DOE project	At4g17370	Aliphatic sulfonate monooxygenase family, FMNH2- or F420-dependent	0	0	0	0	0	0	0	0.000498	0.000438	0	0.000233	7.88775E-05
Sulfur Metabolism	Organic sulfur assimilation	Alkanesulfonate assimilation	Alkanesulfonate monooxygenase (EC 1.14.14.5)	0	0	0	0	0	0	0.000722	0.000498	0.001971	0	0	7.88775E-05
Photosynthesis	Light-harvesting complexes	Phycobilisome	Allophycocyanin alpha chain	0	0	0	0	0.001313	0	0	0.000498	0.000876	0	0.000582	0
Amino Acids and Derivatives	Lysine, threonine, methionine, and cysteine	Lysine biosynthesis AAA pathway 2	Alpha-aminoadipate aminotransferase (EC 2.6.1.39)	0	0	0	0	0.000657	0.001461	0	0	0.000219	0	0	3.94388E-05
Clustering-based subsystems	Catabolism of an unknown compound	CBSS-262316.1.peg.2929	Amino acid permease in hypothetical Actinobacterial gene cluster	0	0	0.002555	0	0	0	0	0.000498	0.000219	0	0	0.000433826
Motility and Chemotaxis	Social motility and nonflagellar swimming in bacteria	Control of Swarming in Vibrio and Shewanella species	Aminotransferase ScrA	0	0.004524	0	0.00498	0.001313	0	0	0	0	0	0	0.000985969
Miscellaneous	NULL	ZZ gjo need homes	AMP/CMP kinase AK6	0	0	0	0.00498	0.000657	0	0	0	0.000219	0	0	0.000118316
RNA Metabolism	Transcription	RNA polymerase archaeal initiation factors	Archaeal transcription factor E	0	0	0	0	0.000657	0	0	0.001993	0.000657	0	0	0.000118316
Metabolism of Aromatic Compounds	Peripheral pathways for catabolism of aromatic compounds	Salicylate ester degradation	AreB (Aryl-alcohol dehydrogenase) (EC 1.1.1.90)	0.000696	0	0	0	0.000657	0.000487	0	0	0	0	0	0.000907091
Amino Acids and Derivatives	Arginine; urea cycle, polyamines	Arginine and Ornithine Degradation	Arginine decarboxylase, catabolic (EC 4.1.1.19)	0	0.002262	0	0	0	0	0	0	0.000438	0	0.000699	0.000236633
Amino Acids and Derivatives	Arginine; urea cycle, polyamines	Arginine and Ornithine Degradation	Arginine utilization protein RocB	0	0	0.002555	0	0	0.000974	0	0	0.000219	0	0	0.000433826
Membrane Transport	NULL	ECF class transporters	ATPase component STY3232 of energizing module of queuosine-regulated ECF transporter	0.000348	0	0	0	0.001313	0	0	0	0.000438	0	0	0.000118316
Membrane Transport	NULL	ECF class transporters	ATPase component STY3233 of energizing module of queuosine-regulated ECF transporter	0	0.002262	0	0	0	0.000974	0	0	0.000657	0	0	0.000433826
DNA Metabolism	DNA repair	DNA repair, bacterial RecBCD pathway	ATP-dependent DNA helicase SCO5183	0.000348	0.002262	0	0	0	0	0	0	0	0.002203	0	3.94388E-05
DNA Metabolism	DNA repair	DNA repair, bacterial UvrD and related helicases	ATP-dependent DNA helicase UvrD/PcrA/Rep	0	0	0	0	0	0	0.000722	0.000997	0.000438	0	0	0.000670459
DNA Metabolism	DNA repair	ATP-dependent Nuclease	ATP-dependent nuclease, subunit B	0	0.002262	0	0	0.001313	0	0	0	0.000876	0	0	0.000433826
RNA Metabolism	RNA processing and modification	ATP-dependent RNA helicases, bacterial	ATP-dependent RNA helicase BA2475	0	0	0	0	0	0	0.000722	0	0.000657	0	0.000233	0.001025408
RNA Metabolism	RNA processing and modification	ATP-dependent RNA helicases, bacterial	ATP-dependent RNA helicase YqfR	0	0	0	0.00249	0	0.000487	0	0	0.000438	0	0	0.000276071
Regulation and Cell signaling	Quorum sensing and biofilm formation	Autoinducer 2 (AI-2) transport and processing (lsrACDBFGE operon)	Autoinducer 2 (AI-2) kinase LsrK (EC 2.7.1.-)	0	0.002262	0	0	0	0	0.002888	0	0	0	0.000233	0.009465301
Metabolism of Aromatic Compounds	Peripheral pathways for catabolism of aromatic compounds	Benzoate degradation	benABC operon transcriptional activator BenR	0	0	0.002555	0	0	0	0	0	0.001095	0	0.003609	0.000591581
Metabolism of Aromatic Compounds	Peripheral pathways for catabolism of aromatic compounds	Benzoate degradation	benzoate-specific porin	0	0.002262	0.002555	0	0	0	0	0.000997	0	0	0	0.00031551
Virulence, Disease and Defense	Resistance to antibiotics and toxic compounds	Beta-lactamase	Beta-lactamase (Cephalosporinase) (EC 3.5.2.6)	0	0	0.002555	0	0	0.000487	0.000722	0	0.000876	0	0	0
Regulation and Cell signaling	NULL	cAMP signaling in bacteria	bifunctional hemolysin-adenylate cyclase precursor	0	0	0	0	0	0.002435	0	0.000997	0.001971	0	0	0.000552143
Virulence, Disease and Defense	Resistance to antibiotics and toxic compounds	Bile hydrolysis	Bile acid 7-alpha dehydratase BaiE (EC 4.2.1.106)	0	0	0.002555	0	0	0.000487	0	0	0.000438	0	0	0.00031551

Respiration	Sodium Ion-Coupled Energetics	Na+ translocating decarboxylases and related biotin-dependent enzymes	Biotin carboxyl carrier protein of methylmalonyl-CoA decarboxylase	0.001044	0	0	0.00249	0	0.000487	0	0	0	0	0	0.00157755
Photosynthesis	NULL	Bacteriorhodopsin	Brp-like protein	0	0	0	0	0	0.000487	0.000722	0	0.000657	0	0	3.94388E-05
Cell Wall and Capsule	Capsular and extracellular polysacchrides	Pseudaminic Acid Biosynthesis	C4 aminotransferase specific for PseB product (PseC, second step of pseudaminic acid biosynthesis)	0	0	0	0	0	0	0.000722	0	0.000219	0	0.000233	0.001735305
Membrane Transport	NULL	Transport of Zinc	Candidate Zn-dependent transcriptional regulator, MntR/DtxR family	0	0	0	0	0	0	0.000722	0	0.001095	0	0.000116	0.000197194
Cell Wall and Capsule	Capsular and extracellular polysacchrides	Capsular Polysaccharides Biosynthesis and Assembly	Capsular polysaccharide ABC transporter, permease protein KpsM	0	0	0	0	0	0	0.000722	0.000498	0.000219	0	0	0.000276071
Cell Wall and Capsule	Capsular and extracellular polysacchrides	Capsular Polysaccharides Biosynthesis and Assembly	Capsular polysaccharide biosynthesis glycosyltransferase	0	0	0	0	0	0	0.000722	0.000498	0	0.002203	0	7.88775E-05
Cell Wall and Capsule	Capsular and extracellular polysacchrides	Serotype determining Capsular polysaccharide biosynthesis in Staphylococcus	Capsular polysaccharide synthesis enzyme Cap5E	0	0	0	0	0.000657	0	0.000722	0.000498	0	0	0	0.000157755
Cell Wall and Capsule	Capsular and extracellular polysacchrides	Serotype determining Capsular polysaccharide biosynthesis in Staphylococcus	Capsular polysaccharide synthesis enzyme Cap8C	0	0	0.002555	0	0	0	0	0	0.000219	0	0.002212	0.001419795
Respiration	NULL	Carbon monoxide induced hydrogenase	Carbon monoxide-induced hydrogenase large subunit CooH	0	0	0	0	0.000657	0.000487	0	0.000498	0	0	0	0.000394388
Respiration	NULL	Carbon monoxide induced hydrogenase	Carbon monoxide-induced hydrogenase membrane protein CooM	0	0	0	0.00498	0	0.000487	0.000722	0	0	0	0	0.000236633
Respiration	NULL	Carbon monoxide induced hydrogenase	Carbon monoxide-induced hydrogenase proton translocating subunit CooK	0	0	0.002555	0	0.001313	0.001461	0	0	0	0	0	0.000433826
Amino Acids and Derivatives	Arginine; urea cycle, polyamines	Polyamine Metabolism	Carboxynorspermidine decarboxylase, putative (EC 4.1.1.-)	0	0	0	0	0	0.000487	0.000722	0	0.000219	0	0	0.000394388
Fatty Acids, Lipids, and Isoprenoids	Fatty acids	Carnitine Metabolism in Microorganisms	Carnitinyl-CoA dehydratase (EC 4.2.1.-)	0	0	0	0	0	0	0.001444	0.00299	0.000438	0	0	0.000157755
Cofactors, Vitamins, Prosthetic Groups, Pigments	Tetrapyrroles	Coenzyme B12 biosynthesis	CblX, a non-orthologous displasment for Alpha-ribazole-5'-phosphate phosphatase	0	0	0	0	0	0.000487	0.000722	0	0.000219	0	0	7.88775E-05
Cofactors, Vitamins, Prosthetic Groups, Pigments	Tetrapyrroles	Coenzyme B12 biosynthesis	CblY, a non-orthologous displasment for Alpha-ribazole-5'-phosphate phosphatase	0	0	0	0	0	0.000974	0	0.000498	0.000657	0	0	0.000118316
Clustering-based subsystems	Cytochrome biogenesis	CBSS-196164.1.peg.461	Ccs1/ResB-related putative cytochrome C-type biogenesis protein	0	0.002262	0.002555	0.00498	0	0	0	0	0	0	0	0.000118316
Regulation and Cell signaling	NULL	Cell envelope-associated LytR-CpsA-Psr transcriptional attenuators	Cell envelope-associated transcriptional attenuator LytR-CpsA-Psr, subfamily A1 (as in PMID19099556)	0.014623	0	0	0	0	0.000487	0	0	0.000219	0	0	0.000118316
Photosynthesis	Light-harvesting complexes	Chlorosome	Chlorosome protein I, 2Fe-2S ferredoxin	0	0	0.002555	0	0.00197	0	0	0.000997	0	0	0	0.00094653
Cell Wall and Capsule	Capsular and extracellular polysacchrides	Phosphorylcholine incorporation in LPS	Cholinephosphate cytidylyltransferase (EC 2.7.7.15)	0.024371	0	0	0	0	0.000487	0	0	0.000219	0	0	3.94388E-05
Miscellaneous	Plant-Prokaryote DOE project	At4g17370	Clavaminate synthase-like protein At3g21360 (EC 1.-.-.-)	0	0	0	0	0.001313	0	0.001444	0	0.001752	0	0	0.000157755
RNA Metabolism	RNA processing and modification	Polyadenylation specificity factor	Cleavage and polyadenylation specificity factor, 73 kDa subunit	0	0.004524	0	0	0	0.000974	0	0	0.000219	0	0	7.88775E-05
Protein Metabolism	Protein degradation	Proteolysis in bacteria, ATP-dependent	ClpXP protease specificity-enhancing factor	0	0.002262	0	0	0	0	0	0	0.000657	0	0.000931	0.000552143
Respiration	NULL	Carbon monoxide induced hydrogenase	CO dehydrogenase/acetyl-CoA synthase subunit gamma, corrinoid iron-sulfur subcomplex large subunit	0	0	0	0	0.001313	0	0.000722	0	0	0.006608	0	0.000709898
Cofactors, Vitamins, Prosthetic Groups, Pigments	Tetrapyrroles	Coenzyme B12 biosynthesis	Cobalamin biosynthesis protein CobG	0	0	0	0	0.000657	0.000487	0	0	0.001533	0.002203	0	0
Cofactors, Vitamins, Prosthetic Groups, Pigments	Coenzyme F420	Coenzyme F420 synthesis	Coenzyme gamma-F420-2:L-glutamate ligase	0	0	0	0	0	0	0.001444	0.000997	0.001095	0	0	0.00031551
Cofactors, Vitamins, Prosthetic Groups, Pigments	Quinone cofactors	Coenzyme PQQ synthesis	Coenzyme PQQ synthesis protein F (EC 3.4.99.-)	0.003482	0	0	0	0	0	0.000722	0	0	0	0.024917	0.001064846
Clustering-based subsystems	Fatty acid metabolic cluster	COG1399	COG1399 protein in cluster with ribosomal protein L32p, Bacteroidetes/Chlorobi subfamily	0	0	0	0	0	0.000974	0	0	0.001095	0	0.000931	0.005008722
RNA Metabolism	RNA processing and modification	tRNA modification Archaea	COG1537: Predicted RNA-binding proteins	0	0	0	0	0.001313	0	0	0.001495	0.001533	0	0	0.000394388
RNA Metabolism	RNA processing and modification	tRNA modification Archaea	COG3269, Predicted RNA-binding protein, contains TRAM domain	0	0	0	0	0	0.000974	0	0.000997	0.000219	0	0	0.000276071
Miscellaneous	Plant-Prokaryote DOE project	COG3533	COG5434 Endopygalactorunase	0	0	0	0	0.000657	0.001461	0.000722	0	0	0	0	0.000118316
Cell Wall and Capsule	Capsular and extracellular polysacchrides	Colanic acid biosynthesis	Colanic acid biosysnthesis protein WcaK	0	0	0	0	0.00197	0.000487	0	0.000498	0	0	0	0.00031551
Protein Metabolism	Protein biosynthesis	Nucleolar protein complex	Collagen alpha 1(I) chain precursor	0	0	0.002555	0	0	0	0	0	0.000438	0	0.000116	0.000157755
RNA Metabolism	RNA processing and modification	tRNA modification Archaea	conserved protein with predicted RNA binding PUA domain	0	0	0	0	0.001313	0	0	0.000997	0.000438	0	0	0.000354949
Regulation and Cell signaling	Regulation of virulence	A conserved operon linked to TyrR and possibly involved in virulence	Conserved protein YcjX with nucleoside triphosphate hydrolase domain	0	0	0	0	0.000657	0	0	0.000498	0.005912	0	0	0.000591581
DNA Metabolism	CRISPs	CRISPRs	CRISPR-associated negative autoregulator	0	0	0	0	0.000657	0	0	0	0.000876	0	0.000582	7.88775E-05
DNA Metabolism	CRISPs	CRISPRs	CRISPR-associated protein, CT1972 family	0	0	0	0	0.003283	0	0	0.000498	0.000219	0	0	0.000236633
DNA Metabolism	CRISPs	CRISP Cmr Cluster	CRISPR-associated RAMP Cmr3	0	0	0	0	0	0.000487	0.000722	0.001495	0.000219	0	0	0
Virulence, Disease and Defense	NULL	Bacterial cyanide production and tolerance mechanisms	Cyanide insensitive terminal oxidase, subunit II	0.005571	0	0.002555	0	0	0	0	0	0.001095	0	0	0.000118316
Metabolism of Aromatic Compounds	Anaerobic degradation of aromatic compounds	Anaerobic benzoate metabolism	Cyclohex-1-ene-1-carboxyl-CoA hydratase (EC 4.2.1.17)	0.00383	0	0	0	0	0	0	0.000498	0.001095	0	0	0.000354949
Clustering-based subsystems	Related to Menaquinone-cytochrome C reductase	CBSS-393130.3.peg.129	Cytochrome b/b6, N-terminal domain	0	0	0	0	0.000657	0.000974	0	0	0.000438	0	0	0.000552143
Amino Acids and Derivatives	NULL	Creatine and Creatinine Degradation	Cytosine permease	0	0	0	0	0	0	0	0	0.001752	0.006608	0.067881	0.004456579
Virulence, Disease and Defense	Resistance to antibiotics and toxic compounds	Methicillin resistance in Staphylococci	D-alanyl transfer protein DltB	0	0.002262	0	0	0	0	0	0	0.000219	0	0.000116	3.94388E-05
Carbohydrates	Monosaccharides	D-allose utilization	D-allose ABC transporter, ATPase component	0	0	0.00511	0	0	0.000487	0	0	0.000438	0	0	0.000512704
Cofactors, Vitamins, Prosthetic Groups, Pigments	Folate and pterines	Methanopterin biosynthesis2	delta 1-pyrroline-5-carboxylate synthetase	0	0.002262	0	0	0	0	0	0.000997	0.000219	0	0	0.000197194
Nucleosides and Nucleotides	Pyrimidines	pyrimidine conversions	Deoxycytidylate 5-hydroxymethyltransferase (EC 2.1.2.8)	0	0	0.002555	0	0	0	0	0.000498	0	0	0.000116	3.94388E-05
Carbohydrates	Monosaccharides	D-galactarate, D-glucarate and D-glycerate catabolism	D-galactarate permease	0	0	0	0	0.000657	0	0	0.000498	0.000219	0	0	0.000236633
Carbohydrates	Monosaccharides	D-galactarate, D-glucarate and D-glycerate catabolism	D-glucarate permease	0	0	0.002555	0	0	0	0	0.000498	0.000657	0	0	0.000157755
Regulation and Cell signaling	NULL	Signal transduction module [RsbQ hydrolase - PAS domain]	Diguanylate cyclase/phosphodiesterase (GGDEF/EAL domains) associated with [RsbQ - PAS domain] sensing module	0	0.002262	0	0	0	0	0.001444	0	0.000438	0	0	0.00031551
Stress Response	Oxidative stress	Glutathione: Non-redox reactions	Distant similarity with leukotriene C4 synthase (microsomal glutathione S-transferase)	0	0	0	0.00249	0	0.001461	0.000722	0	0	0	0	0.000197194
Phages, Prophages, Transposable elements, Plasmids	Phages, Prophages	Phage DNA synthesis	DNA methyl transferase, phage-associated	0	0	0	0	0	0	0.002166	0.004984	0.000219	0	0	0.000118316
DNA Metabolism	DNA replication	DNA topoisomerases, Type I, ATP-independent	DNA topoisomerase III, TraE-type (EC 5.99.1.2)	0.000696	0	0	0	0	0.000974	0	0	0.000438	0	0	0.000157755
RNA Metabolism	RNA processing and modification	tRNA modification Archaea	DNA-binding protein MTH1615	0	0	0	0.00249	0	0	0.000722	0	0.000876	0	0	0.000276071
DNA Metabolism	DNA repair	DNA repair, bacterial	DNA-damage-inducible protein J	0	0	0	0	0	0.000974	0	0.001495	0	0	0.000116	0.000354949

RNA Metabolism	Transcription	RNA polymerase chloroplast	DNA-directed RNA polymerase beta subunit (EC 2.7.7.6), chloroplast	0	0	0	0	0.000657	0	0	0	0.000219	0	0.000116	0.00031551
RNA Metabolism	Transcription	RNA polymerase chloroplast	DNA-directed RNA polymerase delta (= beta'') subunit (EC 2.7.7.6), chloroplast	0	0	0	0	0	0.000487	0	0.000498	0.000438	0	0	0.000157755
RNA Metabolism	Transcription	RNA polymerase chloroplast	DNA-directed RNA polymerase gamma subunit (EC 2.7.7.6), chloroplast	0.000348	0	0	0	0.000657	0	0	0	0.001971	0	0	0.000197194
RNA Metabolism	Transcription	RNA polymerase III	DNA-directed RNA polymerase III second largest subunit (EC 2.7.7.6)	0.000348	0	0	0	0.000657	0	0	0	0	0	0.000116	0.000197194
Amino Acids and Derivatives	Proline and 4-hydroxyproline	Proline, 4-hydroxyproline uptake and utilization	D-proline reductase, 26 kDa subunit (EC 1.21.4.1)	0	0	0	0.00249	0	0.000974	0	0.000498	0	0	0	0.000157755
Amino Acids and Derivatives	Alanine, serine, and glycine	Glycine and Serine Utilization	D-serine/D-alanine/glycine transporter	0	0	0	0	0.00197	0	0	0	0.000438	0	0.000349	0.000354949
Cofactors, Vitamins, Prosthetic Groups, Pigments	Folate and pterines	Methanopterin biosynthesis2	duf556 family protein	0	0	0	0	0	0.000487	0	0	0.000438	0.002203	0	0.000473265
Membrane Transport	NULL	ECF class transporters	Duplicated ATPase component Cce 1530 of energizing module of predicted ECF transporter	0	0	0.002555	0	0	0	0	0	0.000219	0	0.000116	0.000433826
Membrane Transport	NULL	ECF class transporters	Duplicated ATPase component Kcr 0336 of energizing module of predicted ECF transporter	0	0	0	0.00249	0	0	0	0	0.000219	0	0.000116	0.000276071
Membrane Transport	NULL	ECF class transporters	Duplicated ATPase component of energizing module of predicted B12-regulated ECF transporter for dimethylbenzimidazole	0.001044	0	0	0	0.000657	0.000974	0	0	0	0	0	7.88775E-05
Carbohydrates	Monosaccharides	Xylose utilization	D-xylose 1-dehydrogenase (EC 1.1.1.175)	0	0	0	0	0	0	0.000722	0.000997	0.000219	0	0	7.88775E-05
Clustering-based subsystems	Putative asociate of RNA polymerase sigma-54 factor rpoN	CBSS-316057.3.peg.1308	ECF family sigma factor	0	0	0	0	0.000657	0	0.000722	0	0.001533	0	0	0.000118316
Regulation and Cell signaling	NULL	cAMP signaling in bacteria	ElaA protein	0	0	0	0	0	0.002435	0	0.000997	0.002627	0	0	0.005521426
Carbohydrates	NULL	Sugar utilization in Thermotogales	Endo-mannanase (EC 3.2.1.78)	0	0.004524	0	0	0.002626	0	0	0.000997	0	0	0	0.001183163
Respiration	Reverse electron transport	Energy conserving hydrogenase, Methanococcales-Methanobacteriales-Methanopyrales	Energy conserving hydrogenase Eha large subunit homolog (protein O)	0	0	0	0.00249	0	0	0.000722	0.000498	0	0	0	3.94388E-05
Iron acquisition and metabolism	NULL	Iron acquisition in Vibrio	Enterobactin receptor VctA	0	0	0	0	0	0.000487	0	0.002492	0.001095	0	0	0.000552143
Secondary Metabolism	Biologically active compounds in metazoan cell defence and differentiation	Steroid sulfates	Estrogen sulfotransferase (EC 2.8.2.4)	0	0	0	0	0.000657	0	0.000722	0.000997	0	0	0	3.94388E-05
Carbohydrates	Sugar alcohols	Ethanolamine utilization	Ethanolamine operon regulatory protein	0	0	0	0	0	0	0	0.000498	0.000219	0	0.000349	0.000867653
Metabolism of Aromatic Compounds	Anaerobic degradation of aromatic compounds	Anaerobic toluene and ethylbenzene degradation	Ethylbenzene dehydrogenase beta subunit (EC 1.17.99.2)	0	0	0	0	0.001313	0	0	0.000997	0.000219	0	0	0.000354949
Protein Metabolism	Protein biosynthesis	Translation initiation factors eukaryotic and archaeal	Eukaryotic translation initiation factor 4A	0	0	0	0	0	0.000487	0.000722	0	0	0	0.000233	0.000118316
Carbohydrates	Monosaccharides	D-Galacturonate and D-Glucuronate Utilization	Exopolygalacturonate lyase (EC 4.2.2.9)	0	0.002262	0	0	0.000657	0	0	0	0.000219	0	0	0.000157755
Miscellaneous	Plant-Prokaryote DOE project	COG2078	Extradiol ring-cleavage dioxygenase, class III subunit B-like domain	0	0.002262	0	0	0.00197	0	0	0.000997	0	0	0	0.00031551
Stress Response	Oxidative stress	Oxidative stress	Fe2+/Zn2+ uptake regulation proteins	0	0	0	0	0.000657	0.000974	0	0	0	0	0.000233	0.000118316
Carbohydrates	Sugar alcohols	Inositol catabolism	Fe-containing alcohol dehydrogenase	0	0.002262	0	0	0	0	0.001444	0	0.000438	0	0	0.000118316
Sulfur Metabolism	Inorganic sulfur assimilation	Inorganic Sulfur Assimilation	Ferredoxin--NADP(+) reductase, actinobacterial (eukaryote-like) type (EC 1.18.1.2)	0	0	0	0	0	0	0.001444	0.002492	0.000876	0	0	0.000433826
Nitrogen Metabolism	NULL	Nitrate and nitrite ammonification	Ferredoxin--nitrite reductase (EC 1.7.7.1)	0	0	0	0	0.000657	0	0	0.000997	0.002189	0	0	0.000118316
Iron acquisition and metabolism	NULL	Ferrous iron transporter EfeUOB, low-pH-induced	Ferrous iron transport periplasmic protein EfeO, contains peptidase-M75 domain and (frequently) cupredoxin-like domain	0	0	0.002555	0.00249	0	0	0	0	0.000657	0	0	0.000157755
Iron acquisition and metabolism	NULL	Ferrous iron transporter EfeUOB, low-pH-induced	Ferrous iron transport peroxidase EfeB	0.002437	0	0.012774	0	0	0	0	0	0.000438	0	0	0.00031551
Clustering-based subsystems	Cell Division	Cell Division Cluster	FIG001583: hypothetical protein, contains S4-like RNA binding domain	0	0	0	0	0.001313	0	0	0	0.002627	0	0.000582	7.88775E-05
Clustering-based subsystems	NULL	CBSS-316279.3.peg.746	FIG017608: hypothetical membrane protein, specific for cyanobacteria	0	0	0	0	0.001313	0.000974	0	0	0.001533	0	0.000233	0
Cell Wall and Capsule	Gram-Negative cell wall components	Lipopolysaccharide-related cluster in Alphaproteobacteria	FIG081201: hypothetical protein	0	0	0	0	0	0	0.001444	0	0.000876	0	0.000116	0.000276071
Motility and Chemotaxis	Flagellar motility in Prokaryota	Flagellum in Campylobacter	Flagellar hook subunit protein	0	0	0	0	0	0.000487	0.001444	0.000498	0	0	0	0.00063102
Motility and Chemotaxis	Flagellar motility in Prokaryota	Additional flagellar genes in Vibrionales	Flagellar protein FlgP	0	0	0	0	0	0	0	0.000498	0.003722	0	0.000116	0.000197194
Motility and Chemotaxis	Flagellar motility in Prokaryota	Archaeal Flagellum	Flagella-related protein FlaJ	0	0	0	0	0.000657	0	0	0	0.001095	0.002203	0	3.94388E-05
Motility and Chemotaxis	Flagellar motility in Prokaryota	Flagellum	Flagellin protein FlaG	0	0	0	0	0.000657	0.000487	0	0	0.000219	0	0	0.000433826
Miscellaneous	Plant-Prokaryote DOE project	At4g17370	Flavin reductase family, FMN-binding	0	0	0	0	0	0	0.000722	0.000997	0.000219	0	0	3.94388E-05
Clustering-based subsystems	NULL	CBSS-176279.3.peg.1262	Foldase clustered with pyrimidine conversion	0	0.002262	0	0	0	0	0	0.000997	0.000219	0	0	0.000197194
Carbohydrates	One-carbon Metabolism	Methanogenesis	Formylmethanofuran dehydrogenase (tungsten) subunit B (EC 1.2.99.5)	0.002437	0	0	0	0	0.000487	0	0	0.000657	0	0	0.00063102
Carbohydrates	One-carbon Metabolism	Methanogenesis	Formylmethanofuran dehydrogenase (tungsten) subunit C (EC 1.2.99.5)	0	0.002262	0	0.00249	0	0	0	0	0.000219	0	0	0.000236633
Virulence, Disease and Defense	Resistance to antibiotics and toxic compounds	Fosfomycin resistance	Fosfomycin resistance protein FosX	0	0	0	0	0	0.001948	0.000722	0	0.000657	0	0	0.000985969
Carbohydrates	Monosaccharides	Fructose utilization	Fructose repressor FruR, LacI family	0	0	0	0	0	0	0	0.000498	0	0.004406	0.001514	0.000236633
Membrane Transport	Sugar Phosphotransferase Systems, PTS	Fructose and Mannose Inducible PTS	fructose response regulator of fruA and EII fructose/mannose	0	0	0	0	0	0	0.000722	0.001495	0.000219	0	0	0.00063102
Metabolism of Aromatic Compounds	Metabolism of central aromatic intermediates	Salicylate and gentisate catabolism	Fumarylacetoacetate (FAA) hydrolase (EC 3.7.1.5)	0	0	0	0	0	0	0	0.000997	0.000876	0	0.000116	7.88775E-05
Clustering-based subsystems	Putrescine/GABA utilization cluster-temporal,to add to SSs	GABA and putrescine metabolism from cluters	GABA aminotransferase	0	0	0	0	0	0.000487	0	0.000498	0.001752	0	0	0.000197194
Fatty Acids, Lipids, and Isoprenoids	Isoprenoids	Carotenoids	gamma-carotene desaturase, putative	0	0.002262	0	0.00498	0	0	0	0	0.000438	0	0	7.88775E-05
Amino Acids and Derivatives	Glutamine, glutamate, aspartate, asparagine; ammonia assimilation	Poly-gamma-glutamate biosynthesis	Gamma-glutamyltranspeptidase PgsD/CapD (EC 2.3.2.2), catalyses PGA anchorage to peptidoglycan	0	0	0	0	0.000657	0.000974	0	0.000498	0	0	0	0.000118316
Phages, Prophages, Transposable elements, Plasmids	Gene Transfer Agent (GTA)	Gene Transfer Agent	Gene Transfer Agent (GTA) ORFG06	0.002785	0	0	0	0	0.001461	0	0.001993	0	0	0	0.000236633
Membrane Transport	Protein secretion system, Type II	General Secretion Pathway	General secretion pathway protein N	0	0	0	0	0	0	0.000722	0.000498	0.001752	0	0	0.000118316
Photosynthesis	NULL	Bacteriorhodopsin	Geranylgeranyl-diphosphate geranylgeranyl-transferase	0	0	0	0	0	0.000974	0.001444	0	0.000657	0	0	0.000197194
Carbohydrates	Sugar alcohols	Glycerol and Glycerol-3-phosphate Uptake and Utilization	GlpG protein (membrane protein of glp regulon)	0	0	0	0	0	0.000487	0	0.001993	0.006131	0	0	0.00252408
Carbohydrates	Di- and oligosaccharides	Fructooligosaccharides(FOS) and Raffinose Utilization	Glucan 1,6-alpha-glucosidase (EC 3.2.1.70)	0	0.002262	0	0	0	0	0	0.001495	0.000876	0	0	0.000788775
Carbohydrates	Monosaccharides	D-gluconate and ketogluconates metabolism	Gluconate permease, Bsu4004 homolog	0	0	0	0	0	0.000974	0	0.000997	0.001314	0	0	0.000552143
Carbohydrates	Central carbohydrate metabolism	Peripheral Glucose Catabolism Pathways	Glucose ABC transport system, periplasmic sugar-binding protein	0	0	0	0	0.000657	0	0	0	0.002627	0	0.015835	0.000512704
Carbohydrates	NULL	Sugar utilization in Thermotogales	Glucose oligosaccharide ABC transport system, ATP-binding protein 1	0	0	0	0	0	0.000487	0	0.000498	0.000438	0	0	0.000118316

Carbohydrates	Central carbohydrate metabolism	Glycolysis and Gluconeogenesis, including Archaeal enzymes	Glucose-6-phosphate isomerase, archaeal (EC 5.3.1.9)	0	0.002262	0	0	0	0.000974	0	0.001993	0	0	0	0.000433826
Respiration	Sodium Ion-Coupled Energetics	Na+ translocating decarboxylases and related biotin-dependent enzymes	Glutaconyl-CoA decarboxylase beta chain (EC 4.1.1.70)	0	0	0	0	0	0	0.000722	0.000498	0	0	0.000116	7.88775E-05
Amino Acids and Derivatives	Glutamine, glutamate, aspartate, asparagine; ammonia assimilation	Glutamate and Aspartate uptake in Bacteria	Glutamate Aspartate transport system permease protein GltJ (TC 3.A.1.3.4)	0	0	0	0	0	0.000487	0	0	0.001314	0	0.000116	0.000552143
Stress Response	Acid stress	Glutamate transporter involved in acid tolerance in Streptococcus	Glutamate transport ATP-binding protein	0	0.002262	0	0	0.000657	0	0	0	0.001752	0	0	0.000276071
Amino Acids and Derivatives	Alanine, serine, and glycine	Alanine biosynthesis	Glutamate-pyruvate aminotransferase (EC 2.6.1.2)	0	0	0	0	0.000657	0	0	0.001993	0.000219	0	0	0.000512704
Amino Acids and Derivatives	Glutamine, glutamate, aspartate, asparagine; ammonia assimilation	Glutamine, Glutamate, Aspartate and Asparagine Biosynthesis	Glutamine synthetase family protein in hypothetical Actinobacterial gene cluster	0	0	0	0	0	0.000487	0	0	0.000657	0	0.000116	0.00126204
Stress Response	Oxidative stress	Glutathione: Biosynthesis and gamma-glutamyl cycle	Glutathione biosynthesis bifunctional protein gshF (EC 6.3.2.2)(EC 6.3.2.3)	0.003482	0	0	0	0	0	0.001444	0.000498	0	0	0	0.000236633
Stress Response	Oxidative stress	Glutathione: Non-redox reactions	Glutathione S-transferase, phi (EC 2.5.1.18)	0	0	0	0	0.000657	0.000974	0.000722	0	0.000438	0	0	0
Stress Response	Detoxification	Glutathione-dependent pathway of formaldehyde detoxification	Glutathione-dependent formaldehyde-activating enzyme (EC 4.4.1.22)	0	0	0	0	0.001313	0	0.000722	0.000997	0.002189	0	0	0
Carbohydrates	Sugar alcohols	Inositol catabolism	Glyceraldehyde-3-phosphate ketol-isomerase (EC 5.3.1.1)	0	0	0.002555	0	0	0	0	0	0.001971	0	0.000233	0.000867653
Carbohydrates	Sugar alcohols	Glycerol fermenation to 1,3-propanediol	Glycerol dehydratase large subunit (EC 4.2.1.30)	0.000348	0.002262	0	0	0	0.000974	0	0	0	0	0	0.000552143
Carbohydrates	CO2 fixation	Photorespiration (oxidative C2 cycle)	Glycine decarboxylase L-protein 2 (EC 1.8.1.4), mitochondrial	0	0	0	0	0	0.000487	0.000722	0	0.000876	0	0	0.00031551
Protein Metabolism	Selenoproteins	Glycine reductase, sarcosine reductase and betaine reductase	Glycine reductase component B beta subunit (EC 1.21.4.2)	0	0	0	0	0.001313	0	0	0.000498	0	0	0.000116	0.000591581
Protein Metabolism	Selenoproteins	Glycine reductase, sarcosine reductase and betaine reductase	Glycine/sarcosine/betaine reductase protein A	0	0.004524	0	0	0	0.000974	0	0	0.000438	0	0	0.000354949
Cell Wall and Capsule	Gram-Positive cell wall components	Anthrose Biosynthesis	Glycosyl transferase ,group 2 family, anthrose biosynthesis	0	0	0	0	0	0	0.000722	0.000498	0.000876	0	0	0.000709898
Miscellaneous	NULL	ZZ gjo need homes	GTP-binding protein, gtp1/obg family	0	0	0	0	0.001313	0	0	0.001993	0.000876	0	0	0.000907091
Regulation and Cell signaling	NULL	Stringent Response, (p)ppGpp metabolism	Guanosine-5'-triphosphate,3'-diphosphate pyrophosphatase (EC 3.6.1.40)	0	0	0	0	0.000657	0.000974	0	0	0	0	0.000931	0.00094653
Cofactors, Vitamins, Prosthetic Groups, Pigments	Tetrapyrroles	Bilin Biosynthesis	Heme oxygenase (EC 1.14.99.3)	0	0	0	0	0	0.001948	0	0	0.005474	0	0.000815	3.94388E-05
Clustering-based subsystems	NULL	Putative hemin transporter	Heme-regulated cyclic AMP phosphodiesterase (EC 3.1.4.-)	0	0	0	0	0	0	0	0.00299	0.005474	0	0.000116	0.003115662
Iron acquisition and metabolism	NULL	Hemin transport system	Hemin-binding lipoprotein HbpA	0	0	0	0.00249	0	0	0	0	0.000876	0	0.000349	0.000236633
Protein Metabolism	Protein processing and modification	Ubiquitin-like archaeal modifier proteins (SAMPs)	HesA/MoeB/ThiF family protein, possibly E1-enzyme activating SAMPs for protein conjugation	0	0	0	0.00249	0	0	0.000722	0	0.000438	0	0	7.88775E-05
Cell Division and Cell Cycle	NULL	Heterocyst formation in cyanobacteria	Heterocyst differentiation protein HetC	0	0	0	0	0.001313	0	0	0.000498	0.001314	0	0	7.88775E-05
Cell Wall and Capsule	Capsular and extracellular polysacchrides	Rhamnose containing glycans	Heteropolysaccharide repeat unit export protein	0	0	0	0	0.001313	0	0.001444	0	0	0	0.000699	0.000236633
Clustering-based subsystems	Biosynthesis of galactoglycans and related lipopolysacharides	CBSS-376686.6.peg.291	Hexapeptide transferase family protein	0	0	0	0.00498	0.000657	0	0	0	0.001971	0	0	0.000354949
Carbohydrates	Central carbohydrate metabolism	Entner-Doudoroff Pathway	Hexokinase (EC 2.7.1.1)	0	0	0	0	0	0.000487	0.000722	0	0	0	0.000116	0.00031551
Amino Acids and Derivatives	Arginine; urea cycle, polyamines	Arginine and Ornithine Degradation	Histidine ABC transporter, histidine-binding periplasmic protein precursor HisJ (TC 3.A.1.3.1)	0	0	0	0	0	0	0.000722	0.000498	0.000219	0	0	0.000197194
Miscellaneous	Plant-Prokaryote DOE project	At5g48545 and At3g56490 At1g31160	Histidine triad (HIT) nucleotide-binding protein, possible regulatory role based on hydrolysis of lysyl-AMP or LysRS:lysyl-AMP	0	0.002262	0	0	0	0.000487	0.000722	0	0	0	0	0.000433826
Miscellaneous	Plant-Prokaryote DOE project	At5g48545 and At3g56490 At1g31160	Histidine triad nucleotide-binding protein 1 (HINT1)	0.000348	0	0	0.00498	0	0	0	0	0.000438	0	0	0.000236633
Protein Metabolism	Protein biosynthesis	tRNA aminoacylation, His	Histidyl-tRNA synthetase, archaeal-type paralog (EC 6.1.1.21)	0	0	0	0	0.000657	0	0.002166	0.000498	0	0	0	0.000670459
Respiration	Electron donating reactions	Hydrogenases	hydrogenase, methyl-viologen-reducing type, delta subunit	0.000348	0	0	0	0.001313	0.000487	0	0	0	0	0	0.000749336
Cofactors, Vitamins, Prosthetic Groups, Pigments	Riboflavin, FMN, FAD	Flavodoxin	Hypothetical flavoprotein YqcA (clustered with tRNA pseudouridine synthase C)	0	0	0	0	0	0	0.000722	0.000997	0.003065	0	0	0.000197194
Respiration	Electron donating reactions	Na(+)-translocating NADH-quinone oxidoreductase and rnf-like group of electron transport complexes	Hypothetical protein in ApbE locus	0	0	0	0	0	0	0	0.000498	0.000438	0.004406	0	0.004574896
Stress Response	NULL	Bacterial hemoglobins	Hypothetical protein in Cyanoglobin locus	0	0	0	0	0	0	0.000722	0.001993	0.002846	0	0	0.000354949
Clustering-based subsystems	NULL	PFGI-1-like cluster 2	hypothetical protein in PFGI-1-like cluster	0.002089	0	0	0	0	0	0.000722	0	0	0	0.000116	0.000157755
Amino Acids and Derivatives	Glutamine, glutamate, aspartate, asparagine; ammonia assimilation	Poly-gamma-glutamate biosynthesis	Hypothetical protein in predicted poly-gamma-glutamate synthase operon	0	0.002262	0	0	0	0	0.002166	0	0.006787	0	0	0.000473265
Clustering-based subsystems	NULL	USS-DB-1	Hypothetical protein USSDB1E	0	0.004524	0.012774	0	0	0	0	0	0.001533	0	0	0.001222601
Miscellaneous	Plant-Prokaryote DOE project	Synechocystis experimental	Hypothetical protein, slr1506/slr1944 homolog	0	0	0	0	0	0.000487	0	0.000498	0.001971	0	0.000582	0
Clustering-based subsystems	Cytochrome biogenesis	CBSS-196164.1.peg.461	Hypothetical, related to broad specificity phosphatases COG0406	0	0	0.002555	0	0	0.000974	0	0	0	0.004406	0	0.000157755
Membrane Transport	Protein and nucleoprotein secretion system, Type IV	Dot-Icm type IV secretion system	IcmE (DotG) protein	0	0	0	0	0.000657	0.000487	0.004333	0.000498	0	0	0	0
Membrane Transport	Protein and nucleoprotein secretion system, Type IV	Conjugative transfer	IncF plasmid conjugative transfer pilus assembly protein TraB	0	0	0	0	0.001313	0	0	0.000498	0	0	0.000233	0.000552143
Membrane Transport	Protein and nucleoprotein secretion system, Type IV	Conjugative transfer	IncF plasmid conjugative transfer pilus assembly protein TraF	0	0	0	0	0.000657	0.000974	0	0	0.000657	0	0	7.88775E-05
Clustering-based subsystems	NULL	Conjugative transfer related cluster	IncF plasmid conjugative transfer pilus assembly protein TraU	0	0	0	0.00249	0.001313	0.000974	0	0	0	0	0	0.000709898
Membrane Transport	Protein and nucleoprotein secretion system, Type IV	Conjugative transfer	IncF plasmid conjugative transfer protein TraN	0	0	0	0	0	0.000487	0	0	0.000438	0	0.000233	0.000394388
Membrane Transport	Protein and nucleoprotein secretion system, Type IV	Conjugative transfer	IncQ plasmid conjugative transfer protein TraB	0	0	0	0	0	0.000974	0.001444	0	0.000219	0	0	3.94388E-05
Cofactors, Vitamins, Prosthetic Groups, Pigments	NAD and NADP	NAD and NADP cofactor biosynthesis global	Indoleamine 2,3-dioxygenase (EC 1.13.11.52)	0	0	0	0	0.000657	0	0	0.001495	0.000438	0	0	0.000118316
Carbohydrates	Di- and oligosaccharides	Maltose and Maltodextrin Utilization	Inner membrane ABC transporter permease protein YcjP	0	0	0	0	0	0	0.000722	0.000498	0.001314	0	0	0.000670459
Virulence, Disease and Defense	Invasion and intracellular resistance	Listeria surface proteins: Internalin-like proteins	Internalin C	0	0.002262	0.002555	0	0	0	0	0.000498	0	0	0	0.000197194
Virulence, Disease and Defense	Invasion and intracellular resistance	Listeria surface proteins: Internalin-like proteins	Internalin-like protein (LPXTG motif) Lmo0331 homolog	0.001044	0.002262	0	0	0	0	0	0	0.000438	0	0	0.000512704
Membrane Transport	NULL	Ton and Tol transport systems	iron-chelator utilization protein	0	0	0.002555	0	0	0	0.000722	0	0.000438	0	0	0.000157755
Carbohydrates	Polysaccharides	Glycogen metabolism	Isoamylase-type starch debranching enzyme (3.2.1.-)	0	0	0	0	0	0	0	0.000498	0.000219	0.002203	0	3.94388E-05
Amino Acids and Derivatives	Aromatic amino acids and derivatives	Chorismate: Intermediate for synthesis of Tryptophan, PAPA antibiotics, PABA, 3-hydroxyanthranilate and more.	Isochorismate pyruvate-lyase (EC 4.-.-.-)	0	0	0	0	0	0	0	0.000997	0.000657	0.002203	0	0.000788775
Carbohydrates	Central carbohydrate metabolism	TCA Cycle	Isocitrate dehydrogenase 3 subunit alpha, mitochondrial precursor (EC 1.1.1.41)	0.000348	0	0	0	0	0	0.000722	0.000997	0	0	0	7.88775E-05
Secondary Metabolism	Biosynthesis of phenylpropanoids	Phytoalexin biosynthesis	Isoflavone reductase homolog P3 (EC 1.3.1.-)	0	0	0	0.00249	0	0.000974	0	0	0.000438	0	0	0.000197194
Potassium metabolism	NULL	Potassium homeostasis	Kef-type transport system 2 (probable substrate potassium), subunit 2	0.001741	0	0	0	0.000657	0	0	0	0.000657	0	0	3.94388E-05

Amino Acids and Derivatives	Branched-chain amino acids	Ketoisovalerate oxidoreductase	Ketoisovalerate oxidoreductase subunit VorD (EC 1.2.7.7)	0	0.006786	0	0	0	0	0.00361	0	0.000219	0	0	0.000394388
Amino Acids and Derivatives	Branched-chain amino acids	Ketoisovalerate oxidoreductase	Ketoisovalerate oxidoreductase subunit VorG (EC 1.2.7.7)	0.000348	0.002262	0	0	0.000657	0	0	0	0	0	0	0.000512704
Amino Acids and Derivatives	NULL	L-2-amino-thiazoline-4-carboxylic acid-Lcysteine conversion	L-2-amino-thiazoline-4-carboxylic acid hydrolase (EC 3.5.2.-)	0	0.004524	0	0.00249	0	0	0	0.000498	0	0	0	0.00031551
Carbohydrates	Monosaccharides	L-rhamnose utilization	Lactaldehyde dehydrogenase involved in fucose or rhamnose utilization (EC 1.2.1.22)	0	0	0	0	0.000657	0.000487	0	0	0.000219	0	0	0.000118316
Carbohydrates	NULL	Lacto-N-Biose I and Galacto-N-Biose Metabolic Pathway	Lacto-N-biose phosphorylase (EC 2.4.1.211)	0	0.006786	0	0.00747	0	0.001461	0	0	0	0	0	0.000749336
Cell Wall and Capsule	Gram-Negative cell wall components	Vibrio Core Oligosaccharide Biosynthesis	Lipopolysaccharide biosynthesis glycosyltransferase	0	0	0	0	0	0.001461	0	0	0.000657	0	0.000116	0.000709898
Cell Wall and Capsule	Gram-Negative cell wall components	LOS core oligosaccharide biosynthesis	Lipopolysaccharide heptosyltransferase III (EC 2.4.1.-)	0	0	0	0	0	0.000487	0.000722	0	0.000219	0	0	0.001025408
Respiration	Electron donating reactions	Respiratory dehydrogenases 1	L-lactate dehydrogenase (FMN-dependent) and related alpha-hydroxy acid dehydrogenase	0.000696	0	0	0.00249	0	0.000487	0	0	0	0	0	0.00031551
Carbohydrates	Central carbohydrate metabolism	Ethylmalonyl-CoA pathway of C2 assimilation	L-malyl-CoA/beta-methylmalyl-CoA lyase (EC 4.1.3.-), chloroflexus type	0	0	0.002555	0	0	0.000487	0	0	0.000438	0	0	0.000276071
Carbohydrates	CO2 fixation	CO2 uptake, carboxysome	Low-affinity CO2 hydration protein CphX	0	0	0	0	0	0.000974	0	0.001495	0.00416	0	0.001048	0
Phosphorus Metabolism	NULL	Phosphate metabolism	Low-affinity inorganic phosphate transporter	0	0	0	0.00498	0	0.000974	0	0	0.000438	0	0	7.88775E-05
Carbohydrates	Monosaccharides	L-ascorbate utilization (and related gene clusters)	L-ribulose-5-phosphate 4-epimerase UlaF (EC 5.1.3.4) (L-ascorbate utilization protein F)	0	0	0	0	0.000657	0.000974	0	0	0	0	0.001747	0.000591581
Amino Acids and Derivatives	Alanine, serine, and glycine	Glycine and Serine Utilization	L-serine dehydratase 1 (EC 4.3.1.17)	0	0	0	0	0	0	0.000722	0	0.000219	0	0.004541	0.000552143
Protein Metabolism	Protein biosynthesis	Ribosome LSU eukaryotic and archaeal	LSU ribosomal protein L40e	0	0.002262	0	0	0	0	0.000722	0.000498	0	0	0	7.88775E-05
Carbohydrates	Monosaccharides	L-ascorbate utilization (and related gene clusters)	L-xylulose 5-phosphate 3-epimerase (EC 5.1.3.-)	0	0	0	0	0.001313	0	0.000722	0	0	0	0.000233	0.000197194
Fatty Acids, Lipids, and Isoprenoids	Isoprenoids	Myxoxanthophyll biosynthesis in Cyanobacteria	Lycopene cyclase, CruA type	0	0	0	0	0.000657	0	0	0.001495	0.006131	0	0.000466	0
Regulation and Cell signaling	Programmed Cell Death and Toxin-antitoxin Systems	Murein hydrolase regulation and cell death	LysR family regulatory protein CidR	0	0	0	0	0.001313	0	0	0	0.000219	0	0.000233	0.000118316
Regulation and Cell signaling	NULL	DNA-binding regulatory proteins, strays	LysR family transcriptional regulator near succinyl-CoA:3-ketoacid-coenzyme A transferase	0	0	0	0	0	0.000487	0	0	0.000219	0	0.00815	0.000591581
Clustering-based subsystems	NULL	PA0057 cluster	LysR-family transcriptional regulator clustered with PA0057	0	0.002262	0	0	0	0	0	0	0.000657	0	0.037492	0.000867653
Carbohydrates	Sugar alcohols	Mannitol Utilization	Mannitol-1-phosphate 5-dehydrogenase (EC 1.1.1.17)	0	0	0	0	0.000657	0.000487	0	0.000498	0	0	0	0.000433826
Membrane Transport	NULL	Agrobacterium opine transport	Mannopine transporter permease protein MotD	0	0	0	0	0	0	0	0	0.000219	0.004406	0.000233	7.88775E-05
Carbohydrates	Monosaccharides	Mannose Metabolism	Mannoside ABC transport system, sugar-binding protein	0	0	0.002555	0	0.000657	0	0	0	0.000438	0	0	0.000157755
Carbohydrates	Di- and oligosaccharides	Melibiose Utilization	Melibiose operon regulatory protein	0	0	0	0	0	0	0.000722	0.000997	0.000438	0	0	7.88775E-05
Clustering-based subsystems	NULL	USS-DB-6	Membrane lipoprotein lipid attachment site containing protein USSDB6D	0	0	0	0	0	0	0.001444	0.001993	0.001095	0	0	0.000118316
Miscellaneous	NULL	YbbK	Membrane protease family protein HP0248	0	0	0	0.00747	0	0.000974	0	0.000498	0	0	0	0.00855821
Respiration	Sodium Ion-Coupled Energetics	Na+ translocating decarboxylases and related biotin-dependent enzymes	Membrane protein associated with methylmalonyl-CoA decarboxylase	0.004178	0	0	0	0	0	0.000722	0	0.000876	0	0	0.001143724
Miscellaneous	Plant-Prokaryote DOE project	At1g54520	Membrane protein PxcA, involved in light-induced proton extrusion	0	0	0	0	0	0	0.002888	0.000997	0.000876	0	0.000582	0
Regulation and Cell signaling	Regulation of virulence	A conserved operon linked to TyrR and possibly involved in virulence	Membrane protein YcjF	0	0	0	0	0	0	0.001444	0.000498	0.003065	0	0	3.94388E-05
Virulence, Disease and Defense	Invasion and intracellular resistance	Cytolysin and Lipase operon in Vibrio	Metalloprotease, putative zinc-binding domain	0	0	0	0	0	0	0.000722	0.000498	0.001095	0	0	0.000433826
Amino Acids and Derivatives	Lysine, threonine, methionine, and cysteine	Methionine Biosynthesis	Methionine ABC transporter permease protein	0	0	0	0	0	0	0.001444	0	0.002846	0	0.000349	0.000354949
Respiration	NULL	Methanogenesis strays	Methyl coenzyme M reductase system component A2	0	0.002262	0	0	0.000657	0	0	0	0.000657	0	0	0.000591581
Clustering-based subsystems	Methylamine utilization	CBSS-265072.7.peg.546	Methylamine utilization protein MauD	0	0	0.002555	0	0	0	0.000722	0	0.001314	0	0	0.000236633
Carbohydrates	One-carbon Metabolism	Methanogenesis from methylated compounds	Methylcobalamin:coenzyme M methyltransferase, methylamine-specific	0	0	0	0.00249	0	0.000487	0	0	0.000219	0	0	0.000118316
Secondary Metabolism	Aromatic amino acids and derivatives	Cinnamic Acid Degradation	Mhp operon transcriptional activator	0	0	0	0	0	0.000487	0	0	0.000438	0	0.034464	0.000749336
Virulence, Disease and Defense	Resistance to antibiotics and toxic compounds	MexE-MexF-OprN Multidrug Efflux System	Multidrug efflux membrane fusion protein MexE	0	0	0	0	0	0	0.000722	0.000498	0.000876	0	0	7.88775E-05
Virulence, Disease and Defense	Resistance to antibiotics and toxic compounds	Multidrug Resistance Efflux Pumps	Multidrug efflux RND membrane fusion protein MexC	0	0.004524	0	0.00498	0	0	0	0	0.000657	0	0	0.000591581
Virulence, Disease and Defense	Resistance to antibiotics and toxic compounds	The mdtABCD multidrug resistance cluster	Multidrug transporter MdtC	0	0.002262	0	0	0	0	0	0.000498	0.002627	0	0	0.000394388
Carbohydrates	One-carbon Metabolism	Methanogenesis	N5-methyltetrahydromethanopterin:coenzyme M methyltransferase subunit H (EC 2.1.1.86)	0.001393	0	0	0.00249	0	0	0.000722	0	0	0	0	0.000749336
Carbohydrates	Organic acids	Propionyl-CoA to Succinyl-CoA Module	Na+/H+-dicarboxylate symporters	0	0	0	0	0.000657	0	0	0	0.000219	0	0.000116	0.000788775
Carbohydrates	Aminosugars	Chitin and N-acetylglucosamine utilization	N-acetylglucosamine-6P-responsive transcriptional repressor NagC, ROK family	0	0.002262	0	0	0	0	0	0.000498	0	0	0.005123	0.000354949
Amino Acids and Derivatives	Lysine, threonine, methionine, and cysteine	Lysine biosynthesis AAA pathway 2	N-acetyl-lysine aminotransferase (EC 2.6.1.-)	0	0	0	0	0.000657	0.000487	0	0	0.000219	0	0	0.000552143
Cell Wall and Capsule	Capsular and extracellular polysacchrides	Sialic Acid Metabolism	N-acetylmannosamine kinase (EC 2.7.1.60)	0	0	0	0	0.000657	0	0	0.001495	0.000876	0	0	0.000552143
Respiration	Electron donating reactions	Respiratory Complex I	NAD(P)H-quinone oxidoreductase chain H (EC 1.6.5.2)	0	0.002262	0	0	0.001313	0	0	0	0.001971	0	0	0.001222601
Amino Acids and Derivatives	Glutamine, glutamate, aspartate, asparagine; ammonia assimilation	Glutamate dehydrogenases	NAD-specific glutamate dehydrogenase (EC 1.4.1.2), eukaryotic type	0.000348	0	0	0	0	0.000487	0.000722	0	0	0	0	0.000157755
Motility and Chemotaxis	Flagellar motility in Prokaryota	Flagellum	Negative regulator of flagellin synthesis	0	0	0	0	0.000657	0	0	0.000498	0.002846	0	0	0.00031551
Fatty Acids, Lipids, and Isoprenoids	Isoprenoids	Carotenoids	Neurosporene desaturase (EC 1.-.-.-)	0	0	0	0	0	0.000487	0.000722	0	0.001314	0	0	0.000788775
Membrane Transport	NULL	Transport of Nickel and Cobalt	Nickel ABC transporter, periplasmic nickel-binding protein NikA (TC 3.A.1.5.3)	0	0	0	0	0.000657	0.000974	0	0	0.000438	0	0	0.001104285
Nitrogen Metabolism	NULL	Nitrogen fixation	NifM protein	0	0	0	0	0.000657	0.001948	0	0	0.000219	0	0	0.000749336
Nitrogen Metabolism	NULL	Denitrification	Nitric oxide reductase activation protein NorE	0	0.004524	0	0	0	0.000487	0.006499	0	0	0	0	0.00189306
Nitrogen Metabolism	NULL	Denitrification	Nitrous oxide reductase maturation periplasmic protein NosX	0	0	0	0	0	0.000487	0	0.000997	0.000219	0	0	3.94388E-05
Membrane Transport	NULL	Agrobacterium opine transport	Nopaline transporter ATP-binding protein NocP	0	0	0	0	0	0.002435	0.001444	0	0.000438	0	0	7.88775E-05
Membrane Transport	NULL	Agrobacterium opine transport	Nopaline transporter permease protein NocM	0	0	0	0	0.002626	0.001461	0.001444	0	0.001971	0	0	0
Cell Wall and Capsule	Capsular and extracellular polysacchrides	Capsular Polysaccharides Biosynthesis and Assembly	Oligosaccharide repeat unit polymerase Wzy	0	0	0	0	0	0.000487	0	0.000997	0	0.002203	0	0.004101631
Carbohydrates	Central carbohydrate metabolism	Entner-Doudoroff Pathway	OpcA, an allosteric effector of glucose-6-phosphate dehydrogenase, actinobacterial	0.002785	0.002262	0	0	0	0	0	0	0.000219	0	0	3.94388E-05
Cofactors, Vitamins, Prosthetic Groups, Pigments	Tetrapyrroles	Coenzyme B12 biosynthesis	Optional hypothetical component of the B12 transporter BtuN	0	0	0	0	0	0	0.000722	0.000498	0.000657	0	0	0.000118316
Virulence, Disease and Defense	Adhesion	Adhesion of Campylobacter	Outer membrane fibronectin-binding protein	0	0	0	0.00498	0	0	0.000722	0	0.000438	0	0	0.002681835

Cell Wall and Capsule	Gram-Negative cell wall components	Lipoprotein sorting system	Outer membrane lipoprotein LolB precursor	0	0	0	0	0	0	0	0.000498	0.002846	0	0.000466	0.000512704
Cofactors, Vitamins, Prosthetic Groups, Pigments	Quinone cofactors	Coenzyme PQQ synthesis	Paralog of coenzyme PQQ synthesis protein C	0	0	0	0	0	0	0	0.001993	0.001314	0	0.000233	7.88775E-05
Membrane Transport	ABC transporters	ABC transporter peptide (TC 3.A.1.5.5)	Peptide transport system ATP-binding protein SapD (TC 3.A.1.5.5)	0	0	0	0	0	0	0.000722	0	0.008101	0	0.000699	0.000354949
Membrane Transport	ABC transporters	ABC transporter peptide (TC 3.A.1.5.5)	Peptide transport system ATP-binding protein sapF (TC 3.A.1.5.5)	0	0	0	0	0	0	0	0.000498	0.007006	0	0.007801	0.000276071
Clustering-based subsystems	Protein export?	CBSS-393121.3.peg.2760	peptidoglycan lytic protein P45	0.002437	0	0	0	0	0	0.000722	0.000498	0	0	0	0.000118316
Respiration	Electron accepting reactions	fumarate respiration, periplasmic	periplasmic fumarate reductase, FccA (EC 1.3.99.1)	0	0	0	0	0.00197	0.000974	0	0	0.000438	0	0	0.000749336
Iron acquisition and metabolism	Siderophores	Siderophore assembly kit	Periplasmic hemin-binding protein	0	0	0	0	0.000657	0	0.001444	0.001495	0	0	0	0.000552143
Phages, Prophages, Transposable elements, Plasmids	Phages, Prophages	Phage entry and exit	Phage holin	0	0	0	0	0.00197	0.000974	0	0.001993	0	0	0	3.94388E-05
Phages, Prophages, Transposable elements, Plasmids	Phages, Prophages	Phage integration and excision	Phage Holliday junction resolvase	0	0	0	0	0.000657	0.000487	0	0.003489	0	0	0	7.88775E-05
Phages, Prophages, Transposable elements, Plasmids	Phages, Prophages	Phage neck proteins	Phage neck	0	0	0	0	0	0	0.001444	0.001495	0.000438	0	0	3.94388E-05
Phages, Prophages, Transposable elements, Plasmids	Phages, Prophages	Phage nin genes - N-independent survival	Phage NinB DNA recombination	0	0	0	0	0.011161	0	0.001444	0.003489	0	0	0	3.94388E-05
Phages, Prophages, Transposable elements, Plasmids	Phages, Prophages	Phage capsid proteins	Phage prohead core protein	0	0	0	0	0	0.000974	0	0.001993	0	0	0.000116	0.000118316
Phages, Prophages, Transposable elements, Plasmids	Phages, Prophages	Phage entry and exit	Phage rIIB lysis inhibitor	0	0	0	0	0.002626	0.003409	0.006499	0.004485	0	0	0	0
Phages, Prophages, Transposable elements, Plasmids	Phages, Prophages	Phage capsid proteins	Phage scaffold prohead core protein	0	0	0	0	0.002626	0.000487	0	0.000498	0	0	0	0.000236633
Phages, Prophages, Transposable elements, Plasmids	Phages, Prophages	Phage integration and excision	Phage-associated recombinase	0	0	0	0	0	0.003409	0.001444	0.004984	0	0	0	3.94388E-05
Metabolism of Aromatic Compounds	Peripheral pathways for catabolism of aromatic compounds	Phenol hydroxylase	Phenol hydroxylase, FAD- and [2Fe-2S]-containing reductase component DmpP	0	0	0	0	0	0	0	0.000997	0.003284	0	0.09338	0.004772089
Metabolism of Aromatic Compounds	Anaerobic degradation of aromatic compounds	Anaerobic toluene and ethylbenzene degradation	Phenylitaconyl-CoA hydratase (EC 4.2.1.-)	0.000696	0	0	0	0.000657	0	0.000722	0	0	0	0	0.000433826
Cell Wall and Capsule	Gram-Negative cell wall components	Lipid A modifications	Phosphoethanolamine transferase EptA specific for the 1 phosphate group of core-lipid A	0.002785	0	0	0	0	0	0	0	0.000657	0.004406	0	0.002681835
Cofactors, Vitamins, Prosthetic Groups, Pigments	Coenzyme A	Coenzyme A Biosynthesis	Phosphopantetheine adenylyltransferase, type II eukaryotic (EC 2.7.7.3)	0	0	0	0	0.001313	0.000974	0	0	0.000438	0	0	0.000276071
Carbohydrates	CO2 fixation	Calvin-Benson cycle	Phosphoribulokinase (EC 2.7.1.19) homolog, function unknown	0	0	0	0	0	0	0.000722	0	0.002846	0	0.000931	0.003115662
Photosynthesis	Electron transport and photophosphorylation	Photosystem I	photosystem I subunit II (PsaD)	0	0	0	0	0	0.000974	0.001444	0	0.002627	0	0.000466	0
Photosynthesis	Light-harvesting complexes	Phycobilisome	Phycobilisome rod-core linker polypeptide, phycocyanin-associated	0	0	0	0	0	0.000974	0	0.001495	0.000876	0	0.000349	0
Phages, Prophages, Transposable elements, Plasmids	Plasmid related functions	Plasmid-encoded T-DNA transfer	Plant-inducible protein PinF2, cytochrome P450-like, contributes to virulence	0	0.002262	0	0	0	0	0	0	0.000876	0	0.000699	0.000394388
DNA Metabolism	DNA replication	Plasmid replication	Plasmid replication protein RepC	0	0	0	0	0.000657	0	0.002166	0	0	0	0.001747	0.001064846
Cell Wall and Capsule	Capsular and extracellular polysacchrides	Alginate metabolism	Poly(beta-D-mannuronate) C5 epimerase precursor (EC 5.1.3.-)	0	0	0	0	0	0.000487	0.000722	0	0.001314	0	0	7.88775E-05
Respiration	Electron accepting reactions	Anaerobic respiratory reductases	Polysulfide reductase, subunit C, putative	0.002785	0	0	0	0	0.000487	0	0.000498	0	0	0	0.000512704
Potassium metabolism	NULL	Potassium homeostasis	Potassium-transporting ATPase C chain (EC 3.6.3.12) (TC 3.A.3.7.1)	0	0	0	0.00498	0	0	0	0	0.004817	0.002203	0	0.000552143
Cofactors, Vitamins, Prosthetic Groups, Pigments	Tetrapyrroles	Coenzyme B12 biosynthesis	Precorrin-6A synthase (EC 2.1.1.152)	0	0	0	0	0	0	0.000722	0.000498	0.000438	0	0	0.000552143
Amino Acids and Derivatives	Arginine; urea cycle, polyamines	Arginine Biosynthesis extended	Predicted arginine uptake transporter	0	0	0	0	0	0.000974	0	0	0.001095	0	0.010363	0.008124384
Carbohydrates	Di- and oligosaccharides	Beta-Glucoside Metabolism	Predicted cellobiose ABC transport system, permease protein 1	0.000348	0	0	0	0.000657	0	0	0	0	0.002203	0	7.88775E-05
Carbohydrates	Aminosugars	Chitin and N-acetylglucosamine utilization	Predicted chitobiose ABC transport system II, permease protein 1	0	0	0	0	0	0.000974	0	0.000997	0.000438	0	0	0.000197194
Carbohydrates	Sugar alcohols	Erythritol utilization	Predicted erythritol ABC transporter 1, permease component 1	0	0	0	0.00249	0	0	0	0.000498	0.000219	0	0	3.94388E-05
Carbohydrates	Sugar alcohols	Erythritol utilization	Predicted erythritol ABC transporter 2, permease component	0	0	0	0	0	0.000974	0	0.000997	0.000657	0	0	7.88775E-05
Carbohydrates	Monosaccharides	Hexose Phosphate Uptake System	Predicted glucose transporter in beta-glucoside utilization gene cluster	0	0	0	0	0.000657	0	0	0	0.012918	0	0.005356	0.001616989
Miscellaneous	Plant-Prokaryote DOE project	COG3533	Predicted glycerol-1-phosphate dehydrogenase, arabinose operon	0	0	0.002555	0	0.000657	0.000487	0	0	0	0	0	7.88775E-05
Carbohydrates	Organic acids	Lactate utilization	Predicted Lactate-responsive regulator, IclR family	0	0	0	0	0	0.000487	0.000722	0	0	0.002203	0	0.000394388
Carbohydrates	Organic acids	Lactate utilization	Predicted Lactate-responsive regulator, LysR family	0	0	0	0	0	0	0.000722	0	0.001314	0.002203	0	0.000157755
Carbohydrates	Monosaccharides	L-rhamnose utilization	Predicted L-rhamnose permease RhaY	0	0	0.002555	0.00249	0	0	0	0	0.000876	0	0	0.000197194
Carbohydrates	Monosaccharides	Mannose Metabolism	Predicted regulator of mannoside utilization, LacI family	0	0	0	0	0	0.000487	0	0	0.000438	0	0.000233	0.000118316
Amino Acids and Derivatives	Lysine, threonine, methionine, and cysteine	Methionine Biosynthesis	Predicted regulator of methionine metabolism, ArsR family	0	0	0	0	0.002626	0	0.001444	0.001495	0	0	0	0.000670459
Membrane Transport	Protein secretion system, Type II	Predicted secretion system W clustering with cell division proteins	Predicted secretion system W protein GspD-like	0	0	0	0	0.001313	0	0	0.000498	0	0.006608	0	0.000788775
Membrane Transport	Protein secretion system, Type II	Predicted secretion system X	Predicted secretion system X protein GspF-like	0	0	0	0	0	0	0	0.000498	0.001314	0.002203	0	0.000276071
Membrane Transport	Protein secretion system, Type II	Predicted secretion system X	Predicted secretion system X protein GspG-like 3	0	0	0	0	0.001313	0	0	0.000498	0.002408	0	0	0.000236633
Carbohydrates	Di- and oligosaccharides	Sucrose utilization Shewanella	Predicted sucrose-specific TonB-dependent receptor	0	0	0	0	0	0.001461	0	0	0.000219	0	0.018397	0.00094653
Fatty Acids, Lipids, and Isoprenoids	Fatty acids	Fatty acid degradation regulons	Predicted transcriptional regulator for fatty acid degradation FadP, TetR family	0	0	0	0.00249	0.000657	0	0	0	0.000219	0	0	0.000197194
RNA Metabolism	Transcription	Rrf2 family transcriptional regulators	Predicted transcriptional regulator of sulfate transport, Rrf2 family	0	0	0	0	0	0	0.000722	0	0.000219	0.002203	0	0.001301479
Carbohydrates	Di- and oligosaccharides	Trehalose Uptake and Utilization	Predicted trehalose permease, MFS family	0	0	0	0	0.000657	0	0	0.000498	0.001095	0	0	0.000552143
Carbohydrates	Di- and oligosaccharides	Trehalose Uptake and Utilization	Predicted trehalose permease, MFS family, FucP subfamily	0	0	0	0	0.000657	0	0	0.000997	0.002189	0	0	0.000276071
Clustering-based subsystems	NULL	CBSS-393124.3.peg.2657	Probable carboxyvinyl-carboxyphosphonate phosphorylmutase (EC 2.7.8.23)	0	0	0	0	0.000657	0	0	0.003987	0.001971	0	0	0.000512704
Clustering-based subsystems	proteosome related	Cluster-based Subsystem Grouping Hypotheticals - perhaps Proteosome Related	Prokaryotic ubiquitin-like protein Pup	0.003133	0	0.00511	0	0	0	0	0	0.000657	0	0	0.000433826
Protein Metabolism	Protein biosynthesis	tRNA aminoacylation, Pro	Prolyl-tRNA synthetase related protein	0	0	0	0	0.000657	0	0.000722	0	0.000657	0	0	0.000197194
Stress Response	Osmotic stress	Osmoregulation	Propanediol diffusion facilitator	0	0	0	0	0	0.000487	0	0.000498	0.000219	0	0	0.000118316
Carbohydrates	Sugar alcohols	Propanediol utilization	Propanediol utilization polyhedral body protein PduJ	0	0	0	0.00249	0.000657	0	0	0	0.000219	0	0	0.000236633
Carbohydrates	Sugar alcohols	Propanediol utilization	Propanediol utilization protein PduL	0	0	0.002555	0	0	0.000487	0	0	0.000219	0	0	0.000157755
Cofactors, Vitamins, Prosthetic Groups, Pigments	Lipoic acid	Lipoic acid metabolism	Proposed lipoate regulatory protein YbeD	0.003482	0.004524	0	0	0	0	0	0	0.004817	0	0	0.001814183

Protein Metabolism	Protein degradation	Proteasome eukaryotic	proteasome regulatory subunit Rpt1	0	0	0	0	0	0	0	0	0.000219	0.004406	0.000116	0.000276071
Protein Metabolism	Protein degradation	Proteasome eukaryotic	proteasome regulatory subunit Rpt4	0	0	0	0	0.000657	0	0	0	0.000219	0.002203	0	0.000157755
Carbohydrates	Di- and oligosaccharides	Trehalose Uptake and Utilization	PTS system, trehalose-specific IIB component (EC 2.7.1.69)	0	0	0	0	0	0	0.000722	0.000997	0	0	0.000349	7.88775E-05
RNA Metabolism	RNA processing and modification	tRNA modification Archaea	PUA-PAPS reductase like fusion	0	0	0	0	0	0.000487	0.000722	0.000997	0	0	0	0.000236633
Metabolism of Aromatic Compounds	Peripheral pathways for catabolism of aromatic compounds	Phenylpropanoid compound degradation	Putative 3-alpha-hydroxysteroid dehydrogenase/carbonyl reductase oxidoreductase protein (EC 1.1.1.50)	0	0	0	0	0.000657	0.000487	0	0	0.001314	0	0	0.000433826
Fatty Acids, Lipids, and Isoprenoids	NULL	Polyhydroxybutyrate metabolism	Putative 3-hydroxyacyl-coa dehydrogenase	0	0	0	0	0	0.001461	0	0.000498	0.000219	0	0	0.000433826
Phosphorus Metabolism	NULL	Phosphate metabolism	putative alkaline phosphatase-like protein	0	0	0	0	0	0.000974	0.000722	0.000498	0.000219	0	0	0
Cofactors, Vitamins, Prosthetic Groups, Pigments	Tetrapyrroles	Chlorophyll Biosynthesis	Putative chaperon-like protein Ycf39 for quinone binding in Photosystem II	0	0	0	0	0	0.000487	0	0	0.003065	0	0.000233	7.88775E-05
DNA Metabolism	NULL	YcfH	Putative deoxyribonuclease similar to YcfH, type 3	0	0	0	0	0.000657	0	0.001444	0	0.000219	0	0	3.94388E-05
Clustering-based subsystems	NULL	CBSS-159087.4.peg.2189	Putative deoxyribonuclease similar to YcfH, type 4	0	0	0	0	0.001313	0	0	0.000997	0.000657	0	0	0.000788775
Clustering-based subsystems	NULL	Putative hemin transporter	putative hemin permease	0.001393	0	0	0	0.000657	0	0	0	0.002189	0	0	0.000985969
Iron acquisition and metabolism	NULL	Campylobacter Iron Metabolism	Putative iron-uptake ABC transport system ATP-binding protein	0	0	0.002555	0	0	0	0	0	0.001314	0	0.003609	0.003746682
Regulation and Cell signaling	NULL	Trans-envelope signaling system VreARI in Pseudomonas	Putative outer membrane TonB-dependent receptor associated with haemagglutinin family outer membrane protein	0	0	0	0	0	0.000487	0.000722	0	0.000438	0	0	7.88775E-05
Carbohydrates	Central carbohydrate metabolism	Glycolate, glyoxylate interconversions	Putative phosphoglycolate phosphatase (EC 3.1.3.18)	0	0	0	0	0.000657	0.000487	0	0.000498	0	0	0	3.94388E-05
Photosynthesis	Electron transport and photophosphorylation	Photosystem II-type photosynthetic reaction center	Putative photosynthetic complex assembly protein	0	0	0	0.00249	0	0	0	0.00299	0.002627	0	0	0.000118316
Regulation and Cell signaling	NULL	Staphylococcal accessory gene regulator system	Putative response regulator ArlR	0	0	0	0	0	0	0.00361	0.000498	0	0.002203	0	0.000867653
Miscellaneous	Plant-Prokaryote DOE project	COG0398	Putative thiosulfate sulfurtransferase ynjE (EC 2.8.1.1)	0	0	0	0	0.000657	0	0.000722	0	0.000219	0	0	0.000354949
Membrane Transport	NULL	Ton and Tol transport systems	Putative TolA protein	0	0	0	0	0	0.001461	0.000722	0.001495	0	0	0	0.00063102
Carbohydrates	Monosaccharides	Xylose utilization	Putative xylulose kinase (EC 2.7.1.17)	0	0	0	0	0.000657	0	0.000722	0	0.000657	0	0	0.00031551
Clustering-based subsystems	Putrescine/GABA utilization cluster-temporal,to add to SSs	GABA and putrescine metabolism from cluters	Putrescine utilization regulator	0	0	0.002555	0.00249	0	0	0	0	0	0	0.001514	0.000197194
Amino Acids and Derivatives	Arginine; urea cycle, polyamines	Polyamine Metabolism	Putrescine/proton symporter, putrescine/ornithine antiporter PotE	0.003133	0	0	0	0	0	0	0	0.000219	0	0.000466	0.000157755
Respiration	Electron donating reactions	Respiratory dehydrogenases 1	quinoprotein alcohol dehydrogenase	0	0	0	0	0	0.000974	0	0	0.003722	0	0.110845	0.007059537
Stress Response	Oxidative stress	NADPH:quinone oxidoreductase 2	Redox-sensing transcriptional regulator QorR	0	0	0	0	0.000657	0.000974	0	0	0	0	0.000815	0.000276071
Clustering-based subsystems	NULL	PFGI-1-like cluster 1	Replicative DNA helicase (EC 3.6.1.-) in PFGI-1-like cluster	0	0	0	0	0	0.000487	0.000722	0.00299	0.000219	0	0	0
Respiration	Electron accepting reactions	Tetrathionate respiration	Respiratory arsenate reductase subunit B	0	0.002262	0	0	0.001313	0.000487	0	0	0	0	0	0.000867653
RNA Metabolism	RNA processing and modification	RNA processing and degradation, bacterial	Ribonuclease E inhibitor RraB	0	0	0	0	0	0.000974	0	0.001495	0.003284	0	0	0.000512704
RNA Metabolism	RNA processing and modification	Ribonuclease H	Ribonuclease HI-related protein 3	0.000696	0	0	0	0	0	0	0.000997	0.001095	0	0	0.001538111
RNA Metabolism	RNA processing and modification	RNA processing and degradation, bacterial	Ribonuclease I precursor (EC 3.1.27.6)	0	0	0	0	0	0.001461	0.000722	0	0	0	0.000349	0.00031551
Nucleosides and Nucleotides	NULL	Ribonucleotide reduction	Ribonucleotide reductase of class Ib (aerobic), beta subunit (EC 1.17.4.1)	0	0	0	0	0.001313	0	0	0	0.000657	0	0.000466	0.000157755
Carbohydrates	Monosaccharides	D-ribose utilization	Ribose/xylose/arabinose/galactoside ABC-type transport systems, permease component 1	0	0	0	0	0	0.001948	0.000722	0	0.000438	0	0	3.94388E-05
RNA Metabolism	RNA processing and modification	RNA methylation	Ribosomal RNA large subunit methyltransferase A (EC 2.1.1.51)	0	0	0	0	0	0	0	0.000498	0.005036	0	0.001397	0.002800152
RNA Metabolism	RNA processing and modification	RNA methylation	Ribosomal RNA small subunit methyltransferase F (EC 2.1.1.-)	0	0	0	0	0	0.001461	0.001444	0	0	0	0.000349	0.000197194
Dormancy and Sporulation	NULL	Sporulation Cluster	RNA binding protein, contains ribosomal protein S1 domain	0	0.002262	0	0	0	0.000487	0	0.000498	0	0	0	0.000197194
RNA Metabolism	Transcription	Transcription initiation, bacterial sigma factors	RNA polymerase sigma factor SigZ	0	0	0	0	0.002626	0.000487	0	0.000498	0	0	0	0.000118316
Amino Acids and Derivatives	Arginine; urea cycle, polyamines	Polyamine Metabolism	S-adenosylmethionine decarboxylase proenzyme (EC 4.1.1.50), eukaryotic	0	0	0	0	0	0.000487	0.000722	0.000997	0.000219	0	0	0
Respiration	NULL	Methanogenesis strays	Selenophosphate synthetase-related protein MJ0640	0.002785	0	0	0	0	0	0.000722	0	0	0	0.000233	0.00063102
Clustering-based subsystems	Chromosome Replication	SeqA and Co-occurring Genes	SeqA protein, negative modulator of initiation of replication	0	0	0	0	0	0	0.000722	0.000997	0.000657	0	0	0.000473265
Clustering-based subsystems	Hypothetical lipase related to Phosphatidate metabolism	CBSS-316407.3.peg.1371	Ser/Thr and Tyr protein phosphatase (dual specificity)	0	0	0	0	0	0.000487	0.000722	0	0.000876	0	0	0.000394388
Amino Acids and Derivatives	Alanine, serine, and glycine	Glycine and Serine Utilization	Serine transporter	0.000348	0	0	0	0	0.001461	0	0	0	0	0.008849	0.000433826
Amino Acids and Derivatives	Aromatic amino acids and derivatives	Chorismate Synthesis	Shikimate kinase II (EC 2.7.1.71)	0	0	0.002555	0	0	0	0.001444	0	0.000219	0	0	0.000354949
Amino Acids and Derivatives	Aromatic amino acids and derivatives	Common Pathway For Synthesis of Aromatic Compounds (DAHP synthase to chorismate)	Shikimate/quinate 5-dehydrogenase I beta (EC 1.1.1.282)	0	0	0	0.00249	0.001313	0.002435	0	0	0	0	0	0.001222601
Cell Wall and Capsule	Capsular and extracellular polysacchrides	Sialic Acid Metabolism	Sialic acid transporter (permease) NanT	0.002785	0	0.002555	0	0	0	0	0.000498	0	0	0	3.94388E-05
Cell Wall and Capsule	Capsular and extracellular polysacchrides	Sialic Acid Metabolism	Sialic acid utilization regulator, RpiR family	0	0	0	0	0	0.000487	0.000722	0	0.000219	0	0	3.94388E-05
Iron acquisition and metabolism	Siderophores	Siderophore assembly kit	Siderophore biosynthesis diaminobutyrate--2-oxoglutarate aminotransferase (EC 2.6.1.76)	0	0	0	0	0	0.000487	0	0	0.000219	0.002203	0	0.000118316
RNA Metabolism	Transcription	Transcription initiation, bacterial sigma factors	Sigma factor RpoE negative regulatory protein RseA	0	0	0	0	0	0	0	0.000498	0.001971	0	0.000233	0.000433826
Regulation and Cell signaling	Quorum sensing and biofilm formation	Symbiotic colonization and sigma-dependent biofilm formation gene cluster	Signal transduction histidine kinase SypF	0	0	0	0.00249	0	0.000487	0	0	0.000657	0	0	0.000552143
Motility and Chemotaxis	Flagellar motility in Prokaryota	Flagellar motility	Signal transduction protein CetB, mediates an energy taxis response	0	0.002262	0	0	0	0.000487	0	0	0.000657	0	0	0.011555555
Amino Acids and Derivatives	Alanine, serine, and glycine	Glycine cleavage system	Similar to Glycine cleavage system H protein	0	0.002262	0	0	0	0.000487	0	0	0.000438	0	0	0.00031551
Protein Metabolism	Protein biosynthesis	Ribosome biogenesis bacterial	Similar to ribosomal large subunit pseudouridine synthase D, Bacillus subtilis YhcT type	0	0	0	0	0	0.000487	0	0.000498	0.000657	0	0	0.000236633
Membrane Transport	Protein secretion system, Type II	Widespread colonization island	Similar to TadZ/CpaE, associated with Flp pilus assembly	0	0.002262	0	0	0.000657	0.000487	0	0	0	0	0	0.00063102
Dormancy and Sporulation	NULL	SpoVS protein family	SpoVS-related protein, type 5	0	0	0	0	0.000657	0	0	0	0.000219	0	0.000116	0.000276071
Fatty Acids, Lipids, and Isoprenoids	Isoprenoids	Carotenoids	Squalene desaturase	0	0	0	0	0	0.001948	0	0.000997	0.000876	0	0	0.000354949
Protein Metabolism	Protein biosynthesis	Ribosome SSU chloroplast	SSU ribosomal protein S3p (S3e), chloroplast	0.000348	0	0	0	0	0	0	0	0.000219	0.002203	0	0.000118316
Dormancy and Sporulation	NULL	Sporulation gene orphans	Stage V sporulation protein involved in spore cortex synthesis (SpoVR)	0	0	0	0.00249	0	0.000487	0	0	0.000657	0	0	0.000394388
Membrane Transport	NULL	ECF class transporters	Substrate-specific component CbiM of cobalt	0	0	0	0	0.000657	0	0	0.000498	0	0	0.000466	0.001143724

			ECF transporter												
Membrane Transport	NULL	ECF class transporters	Substrate-specific component MtsA of methionine-regulated ECF transporter	0.002089	0	0	0	0	0	0	0	0.000876	0	0.000233	0.000236633
Respiration	Electron donating reactions	Succinate dehydrogenase	Succinate dehydrogenase cytochrome b558 subunit	0.002785	0	0	0.00249	0	0	0	0	0.000219	0	0	0.000394388
Carbohydrates	Central carbohydrate metabolism	TCA Cycle	Succinyl-CoA ligase [GDP-forming] alpha chain (EC 6.2.1.4)	0	0	0	0	0	0	0	0.000498	0.000657	0	0.000116	0.000394388
Respiration	Electron donating reactions	Hydrogenases	Sulfhydrogenase II subunit g	0.001393	0.002262	0	0.00249	0	0	0	0	0	0	0	0.000473265
Phages, Prophages, Transposable elements, Plasmids	Phages, Prophages	T7-like phage core proteins	T7-like phage primase/helicase protein	0	0	0	0	0	0.000974	0.007943	0.000498	0	0	0	0.000197194
Fatty Acids, Lipids, and Isoprenoids	Fatty acids	Acyl-CoA thioesterase II	TesB-like acyl-CoA thioesterase 5	0	0	0	0	0	0	0.000722	0.000997	0.000438	0	0	7.88775E-05
Amino Acids and Derivatives	Lysine, threonine, methionine, and cysteine	Threonine degradation	threonine dehydrogenase	0	0	0	0	0	0.000487	0.000722	0	0.000219	0	0	0.000473265
Protein Metabolism	Protein biosynthesis	tRNA aminoacylation, Thr	Threonyl-tRNA synthetase-related protein	0	0	0	0	0.000657	0	0	0.000498	0.001095	0	0	0.000118316
Phages, Prophages, Transposable elements, Plasmids	Transposable elements	Tn552	Tn552 transposase	0	0	0	0.00249	0	0	0.000722	0	0.000438	0	0	0.000197194
Phages, Prophages, Transposable elements, Plasmids	Transposable elements	CBSS-203122.12.peg.188	TniB NTP-binding protein	0	0	0	0	0	0.000487	0	0	0.000219	0.002203	0	0.000828214
Photosynthesis	NULL	Bacteriorhodopsin	Transcription regulator Bat, regulates bacteriorhodopsin synthesis	0	0	0	0	0.000657	0	0	0.000498	0.000657	0	0	0.000118316
Virulence, Disease and Defense	Resistance to antibiotics and toxic compounds	Multidrug Resistance Efflux Pumps	Transcription repressor of multidrug efflux pump acrAB operon, TetR (AcrR) family	0	0	0	0	0	0.000487	0	0.000498	0.000876	0	0	0.000473265
Membrane Transport	Uni- Sym- and Antiporters	NhaA, NhaD and Sodium-dependent phosphate transporters	Transcriptional activator NhaR	0	0	0	0	0	0.001461	0	0.000997	0	0	0.000349	0.000354949
Miscellaneous	NULL	Luciferases	Transcriptional activator protein LuxR	0	0	0	0	0	0.000487	0	0.002492	0.000438	0	0	0.000670459
Carbohydrates	One-carbon Metabolism	Formaldehyde assimilation: Ribulose monophosphate pathway	Transcriptional regulator HxlR, formaldehyde assimilation	0.002437	0	0	0	0	0.000974	0	0	0.000219	0	0	0.001932499
Carbohydrates	Monosaccharides	L-fucose utilization	Transcriptional regulator of fucose utilization, GntR family	0	0	0	0	0	0.000487	0	0.000498	0	0	0.002794	0.00157755
Carbohydrates	Sugar alcohols	Mannitol Utilization	Transcriptional regulator of mannitol utilization, DeoR family protein	0	0	0.002555	0	0	0	0	0	0.001533	0	0.002911	0.000236633
Miscellaneous	Plant-Prokaryote DOE project	COG3533	Transcriptional regulator of the arabinose operon in Shewanella, GntR family	0	0	0	0	0.000657	0	0.000722	0	0.001095	0	0	3.94388E-05
Cell Wall and Capsule	Gram-Negative cell wall components	Lipid A modifications	Transcriptional regulatory protein basR/pmrA	0	0	0.00511	0	0	0	0	0	0.000438	0	0.000582	0.00031551
Membrane Transport	NULL	ECF class transporters	Transmembrane component BioN of energizing module of biotin ECF transporter	0	0	0	0	0.001313	0	0	0.000498	0	0	0.000116	0.000197194
Membrane Transport	NULL	ECF class transporters	Transmembrane component Cce 1531 of energizing module of predicted ECF transporter	0	0	0	0	0.002626	0	0	0	0.000219	0	0.000116	0.000118316
Miscellaneous	Plant-Prokaryote DOE project	At4g17370	TRAP-type C4-dicarboxylate transport system, possibly of alkanesulfonates, small permease component	0	0	0	0.00249	0.000657	0	0	0	0.001095	0	0.000466	0
Carbohydrates	Di- and oligosaccharides	Trehalose Uptake and Utilization	Trehalose-regulated TonB-dependent outer membrane receptor	0	0	0	0.00249	0	0	0	0	0.001314	0	0.019212	3.94388E-05
Regulation and Cell signaling	NULL	Signal transduction module [RsbQ hydrolase - PAS domain]	Two-component hybrid sensor and regulator associated with [RsbQ - PAS domain] sensing module	0	0	0	0	0	0.002435	0	0.001495	0.002189	0	0	0.001064846
Iron acquisition and metabolism	NULL	Heme, hemin uptake and utilization systems in GramPositives	Two-component response regulator colocalized with HrtAB transporter	0	0	0	0	0.000657	0.000487	0	0	0.000219	0	0	7.88775E-05
Phages, Prophages, Transposable elements, Plasmids	Plasmid related functions	Plasmid-encoded T-DNA transfer	Two-component sensor kinase of vir regulon, VirA	0	0	0	0	0	0.000487	0	0.000498	0.000657	0	0	0.000867653
Clustering-based subsystems	NULL	USS-DB-2	Two-component system sensor protein [USSDB2B]	0	0	0	0.00249	0	0.000487	0	0	0.000438	0	0	0.000749336
Membrane Transport	Protein secretion system, Type III	Type III secretion systems	Type III secretion bridge between inner and outermembrane lipoprotein (YscJ,HrcJ,EscJ, PscJ)	0	0.002262	0	0	0	0	0	0	0.000657	0	0.000116	7.88775E-05
Membrane Transport	Protein secretion system, Type III	Type III secretion system	Type III secretion inner membrane protein (YscS,homologous to flagellar export components)	0	0	0.002555	0	0	0.000487	0	0	0.000219	0	0	7.88775E-05
Membrane Transport	Protein secretion system, Type III	Type III secretion systems	Type III secretion inner membrane protein (YscU,SpaS,EscU,HrcU,SsaU, homologous to flagellar export components)	0	0	0	0	0	0.000487	0	0.000498	0.001533	0	0	0.000354949
Membrane Transport	Protein secretion system, Type VI	Type VI secretion systems	Type VI secretion lipoprotein/VasD	0	0	0	0	0.001313	0	0	0	0.001752	0	0.000233	0.000276071
Cell Wall and Capsule	Capsular and extracellular polysacchrides	Extracellular Polysaccharide Biosynthesis of Streptococci	Tyrosine-protein kinase transmembrane modulator EpsC	0	0	0	0	0.00197	0.000487	0	0	0.000219	0	0	0.000197194
Miscellaneous	Plant-Prokaryote DOE project	Experimental-Ubiquinone BiosynthesisVDC	Ubiquinone biosynthesis enzyme COQ7	0	0	0	0	0	0	0.000722	0.000498	0.000219	0	0	0.000118316
Miscellaneous	Plant-Prokaryote DOE project	At5g37530	Uncharacterized ATPase (AAA family) associated with cysteine desulfurase	0	0	0	0	0	0	0.001444	0	0.001752	0	0.000116	0.000591581
Stress Response	Oxidative stress	Glutathionylspermidine and Trypanothione	Uncharacterized GST-like protein yghU associated with glutathionylspermidine synthetase/amidase	0	0	0	0	0	0	0	0.000498	0.000219	0	0.008616	0.000157755
Cell Wall and Capsule	Gram-Negative cell wall components	Lipopolysaccharide assembly	Uncharacterized protein YrbK clustered with lipopolysaccharide transporters	0	0	0	0	0	0.000487	0	0.001495	0.001971	0	0	7.88775E-05
Clustering-based subsystems	Carbohydrates	Cluster Ytf and putative sugar transporter	Uncharacterized protein YtfM precursor	0.000696	0	0	0	0	0	0	0	0.001095	0	0.000699	0.000433826
Clustering-based subsystems	Clustering-based subsystems	Putative diaminopropionate ammonia-lyase cluster	Uncharacterized sigma-54-dependent transcriptional regulator YgeV	0	0	0	0.00249	0	0	0.000722	0	0.001095	0	0	0.000433826
Stress Response	NULL	Universal stress protein family	Universal stress protein family 4	0	0	0	0	0.000657	0	0	0.00299	0.002846	0	0	0.000118316
Respiration	NULL	Methanogenesis strays	UPF0129 protein MJ1474	0	0	0	0	0.000657	0.000974	0	0.000997	0.000438	0	0	0
Nucleosides and Nucleotides	Detoxification	Nudix proteins (nucleoside triphosphate hydrolases)	Uridine diphosphate glucose pyrophosphatase (EC 3.6.1.45)	0	0	0	0.00249	0.000657	0	0	0.000498	0	0	0	0.003312855
Carbohydrates	Monosaccharides	D-Galacturonate and D-Glucuronate Utilization	Uronate dehydrogenase (EC 1.1.1.203)	0.002785	0	0	0	0.000657	0	0	0	0.001314	0	0	0.000433826
Iron acquisition and metabolism	NULL	Iron acquisition in Vibrio	Utilization protein for unknown catechol-siderophore X	0	0	0	0	0	0	0	0.002492	0.000657	0	0.000582	0.000157755
Protein Metabolism	Protein biosynthesis	tRNA aminoacylation, Val	Valyl-tRNA synthetase (EC 6.1.1.9), mitochondrial	0	0	0	0.00249	0	0	0	0	0.000219	0	0.000116	0.000118316
Respiration	ATP synthases	V-Type ATP synthase	V-type ATP synthase subunit F (EC 3.6.3.14)	0	0	0	0	0.00197	0.000487	0	0	0.001314	0	0	0.000157755
Nucleosides and Nucleotides	Purines	Purine Utilization	Xanthine oxidase (EC 1.17.3.2)	0	0	0	0	0.000657	0.000487	0	0	0.000219	0	0	0.00031551
Nucleosides and Nucleotides	Purines	Xanthosine utilization (xap region)	Xanthosine permease	0.002437	0	0	0	0	0	0	0	0	0.004406	0.000349	0.006625711
Carbohydrates	Monosaccharides	Xylose utilization	Xylonolactonase (EC 3.1.1.68)	0	0	0	0	0.001313	0	0.000722	0	0.000876	0	0	0.000197194
Carbohydrates	Monosaccharides	Xylose utilization	Xylose oligosaccharides ABC transporter, permease protein 2	0	0.002262	0	0.00249	0	0	0	0.000498	0	0	0	0.000788775
Carbohydrates	Monosaccharides	Xylose utilization	Xylose oligosaccharides ABC transporter, sugar-binding protein	0	0.002262	0	0.00249	0	0	0	0	0.000219	0	0	0.000907091
Cell Wall and Capsule	Capsular and extracellular polysacchrides	YjbEFGH Locus Involved in Exopolysaccharide Production	YjbH outer membrane lipoprotein	0	0.002262	0	0	0	0	0	0.000498	0.005036	0	0	0.00031551
Phages, Prophages, Transposable elements, Plasmids	Pathogenicity islands	Listeria Pathogenicity Island LIPI-1 extended	Zinc metalloproteinase precursor (EC 3.4.24.29)	0.005571	0	0	0	0	0	0.000722	0	0.000876	0	0	0.00063102

Virulence, Disease and Defense	Resistance to antibiotics and toxic compounds	Zinc resistance	Zinc resistance-associated protein	0.001741	0.002262	0	0	0.000657	0	0	0	0	0	0	0.000788775
Membrane Transport	NULL	Transport of Zinc	Zinc-regulated outer membrane porin	0	0	0	0	0.001313	0.000487	0	0	0.003284	0	0.001048	0
Virulence, Disease and Defense	Resistance to antibiotics and toxic compounds	Cobalt-zinc-cadmium resistance	Zn(II) and Co(II) transmembrane diffusion facilitator	0	0	0.00511	0	0	0	0	0.000498	0.000438	0	0	0.000354949
Iron acquisition and metabolism	NULL	Iron acquisition in Vibrio	2,3-dihydro-2,3-dihydroxybenzoate dehydrogenase (EC 1.3.1.28)	0	0	0	0	0	0	0	0.000498	0.000438	0	0	0.000276071
Metabolism of Aromatic Compounds	Peripheral pathways for catabolism of aromatic compounds	Naphtalene and antracene degradation	2,3-dihydroxy-2,3-dihydro-phenylpropionate dehydrogenase (EC 1.3.1.-)	0	0	0	0	0	0.000487	0	0	0.000219	0	0	7.88775E-05
Metabolism of Aromatic Compounds	NULL	p-cymene degradation	2,3-dihydroxy-p-cumate-3,4-dioxygenase (CmtC)	0	0	0	0	0	0	0.000722	0.000997	0	0	0	0.000118316
Amino Acids and Derivatives	Arginine; urea cycle, polyamines	Anaerobic Oxidative Degradation of L-Ornithine	2-amino-4-ketopentanoate thiolase, alpha subunit	0	0	0	0	0.000657	0	0	0	0.000219	0	0	0.000197194
Metabolism of Aromatic Compounds	Peripheral pathways for catabolism of aromatic compounds	Biphenyl Degradation	2-hydroxypenta-2,4-dienoate hydratase	0	0	0.002555	0	0	0	0	0	0.000657	0	0	0.000512704
Carbohydrates	Monosaccharides	D-Galacturonate and D-Glucuronate Utilization	2-keto-3-deoxygluconate permease (KDG permease)	0.005571	0	0	0	0	0	0.001444	0	0	0	0	0.000276071
Carbohydrates	Monosaccharides	2-Ketogluconate Utilization	2-ketogluconate transporter	0.001393	0	0	0	0	0	0	0	0	0.006608	0	0.000433826
Cofactors, Vitamins, Prosthetic Groups, Pigments	Quinone cofactors	Plastoquinone Biosynthesis	2-methyl-6-solanyl-1,4-benzoquinone methyltransferase	0	0	0	0	0	0	0	0	0.001533	0	0.000116	0.00031551
Carbohydrates	Di- and oligosaccharides	Unknown oligosaccharide utilization Sde 1396	2nd GPH family transporter in unknown oligosaccharide utilization Sde 1396	0	0	0	0	0	0	0	0.001495	0.001095	0	0	0.000433826
Fatty Acids, Lipids, and Isoprenoids	Isoprenoids	Myxoxanthophyll biosynthesis in Cyanobacteria	2'-O-glycosyltransferase CruG	0	0	0	0	0.001313	0	0	0	0.000876	0	0.000582	0
Metabolism of Aromatic Compounds	NULL	Benzoate transport and degradation cluster	3-hydroxyacyl-CoA dehydrogenase (PaaH) (EC 1.1.1.157)	0	0	0	0	0	0	0.000722	0	0.000438	0	0	0.000236633
Clustering-based subsystems	Two related proteases	CBSS-257314.1.peg.676	3-oxoacyl-[acyl-carrier protein] reductase paralog (EC 1.1.1.100) in cluster with unspecified monosaccharide transporter	0	0	0	0	0	0.000974	0	0.000498	0	0	0	0.000354949
Metabolism of Aromatic Compounds	Peripheral pathways for catabolism of aromatic compounds	Benzoate catabolism	3-Oxoadipate enol-lactonase (EC 3.1.1.24)	0	0	0	0	0	0.000487	0	0	0.000219	0	0	3.94388E-05
Stress Response	NULL	Flavohaemoglobin	3-phenylpropionate dioxygenase, alpha subunit (EC 1.14.12.19)	0	0	0	0	0	0	0	0.000498	0.000219	0	0	0.000512704
RNA Metabolism	RNA processing and modification	RNA processing and degradation, bacterial	3'-to-5' oligoribonuclease B, Bacillus type	0.002437	0	0	0	0	0	0	0	0	0	0.000116	0.006270762
Amino Acids and Derivatives	Aromatic amino acids and derivatives	Chorismate: Intermediate for synthesis of Tryptophan, PAPA antibiotics, PABA, 3-hydroxyanthranilate and more.	4-amino-4-deoxychorismate synthase, amidotransferase component , aminase component (EC 2.6.1.-)	0	0	0	0	0	0	0	0.000997	0	0.002203	0	7.88775E-05
Metabolism of Aromatic Compounds	Metabolism of central aromatic intermediates	4-Hydroxyphenylacetic acid catabolic pathway	4-hydroxyphenylacetate 3-monooxygenase, reductase component (EC 1.6.8.-)	0	0	0	0	0	0	0	0.000498	0	0	0.032252	0.000473265
Iron acquisition and metabolism	Siderophores	Siderophore assembly kit	ABC-type Fe3+-siderophore transport system, periplasmic iron-binding component	0	0	0	0	0	0.000487	0	0	0.000438	0	0	0.000276071
Membrane Transport	ABC transporters	ABC transporter tungstate (TC 3.A.1.6.2)	ABC-type vanadate transport system, permease protein	0	0.002262	0	0	0	0	0.000722	0	0	0	0	0.000157755
Carbohydrates	Central carbohydrate metabolism	Ethylmalonyl-CoA pathway of C2 assimilation	Acetoacetyl-CoA reductase (EC 1.1.1.36) of ethylmalonyl-CoA pathway	0	0	0	0	0.000657	0	0	0	0.000876	0	0	3.94388E-05
Carbohydrates	NULL	Acetone carboxylase	Acetone carboxylase, beta subunit (EC 6.4.1.6)	0	0	0	0	0	0	0	0	0.000219	0	0.006986	0.001301479
Metabolism of Aromatic Compounds	Anaerobic degradation of aromatic compounds	Anaerobic toluene and ethylbenzene degradation	Acetophenone carboxylase subunit Apc2	0	0	0.007664	0	0	0	0	0	0.000438	0	0	0.00031551
Stress Response	Oxidative stress	Glutathione analogs: mycothiol	Acetyl-CoA:Cys-GlcN-Ins acetyltransferase, mycothiol synthase MshD	0	0.004524	0.002555	0	0	0	0	0	0	0	0	0.00031551
Clustering-based subsystems	NULL	Conserved cluster around acetyltransferase YpeA in Enterobacteria	Acetyltransferase YpeA	0	0.002262	0	0	0.000657	0	0	0	0	0	0	3.94388E-05
Iron acquisition and metabolism	Siderophores	Siderophore Pyoverdine	Acyl-homoserine lactone acylase PvdQ (EC 3.5.1.-), quorum-quenching	0	0.002262	0	0	0	0	0	0.000997	0.001095	0	0	0
Iron acquisition and metabolism	Siderophores	Siderophore Aerobactin	Aerobactin siderophore receptor IutA	0	0	0	0	0	0.000487	0	0	0.000657	0	0	7.88775E-05
Membrane Transport	NULL	Agrobacterium opine transport	Agropine synthesis reductase (EC 1.-.-.-)	0	0	0	0	0	0	0.002166	0	0.000219	0	0	3.94388E-05
Membrane Transport	NULL	Agrobacterium opine transport	Agropinic acid transporter ATP-binding protein AgaD	0	0	0	0	0	0	0.000722	0	0.000219	0	0	0.000118316
Carbohydrates	Central carbohydrate metabolism	Methylglyoxal Metabolism	Aldehyde dehydrogenase, mitochondrial precursor (EC 1.2.1.3)	0	0	0	0.00249	0	0	0	0	0.000219	0.002203	0	0
Cell Wall and Capsule	Capsular and extracellular polysacchrides	Alginate metabolism	Alginate lyase (EC 4.2.2.3)	0	0	0	0	0	0.001461	0.000722	0	0	0	0	0.000394388
Cell Wall and Capsule	Gram-Negative cell wall components	Core Oligosaccharide Glycosylation in Pseudomonas	alpha-1,3-rhamnosyltransferase	0	0	0	0	0.000657	0.000487	0	0	0	0	0	0.000473265
Miscellaneous	Plant-Prokaryote DOE project	COG3533	Alpha-arabinosides ABC transport system, substrate-binding protein	0	0	0	0.00249	0	0	0	0.000498	0	0	0	0.000354949
Stress Response	Dessication stress	O-antigen capsule important for environmental persistence	Alpha-glucosyltransferase YihQ	0	0	0.002555	0	0	0.000487	0.000722	0	0	0	0	0
Amino Acids and Derivatives	Aromatic amino acids and derivatives	Aromatic amino acid degradation	Anthranilate dioxygenase reductase	0	0	0.002555	0	0	0	0	0.000498	0	0	0	0.000354949
Regulation and Cell signaling	Regulation of virulence	Streptococcal Mga Regulon	Antiphagocytic M protein	0	0	0	0	0	0	0	0.000498	0.000438	0	0	0.000197194
Carbohydrates	Monosaccharides	L-Arabinose utilization	Arabinose-regulated TonB-dependent outer membrane receptor	0	0	0	0	0	0	0.000722	0	0.000876	0	0	0.00031551
Protein Metabolism	Protein biosynthesis	tRNA aminoacylation, Ser	Archaeal seryl-tRNA synthetase-related sequence	0	0	0	0	0	0	0	0	0.000219	0.002203	0	0.000512704
Amino Acids and Derivatives	Arginine; urea cycle, polyamines	Arginine and Ornithine Degradation	Arginine ABC transporter, periplasmic arginine-binding protein ArtI	0	0	0	0	0	0	0.000722	0	0	0.004406	0	0.000118316
Stress Response	Acid stress	Acid resistance mechanisms	Arginine/agmatine antiporter	0	0	0	0	0	0	0	0.000498	0.000219	0	0	0.000394388
Amino Acids and Derivatives	Aromatic amino acids and derivatives	Aromatic amino acid degradation	Aromatic amino acid transport protein AroP	0	0	0	0	0	0.000487	0	0.000498	0	0	0	3.94388E-05
Virulence, Disease and Defense	Resistance to antibiotics and toxic compounds	Arsenic resistance	Arsenic efflux pump protein	0	0	0	0	0	0	0	0	0.000219	0	0.000349	0.009780811
RNA Metabolism	RNA processing and modification	tRNA modification Archaea	Asparagine--tRNA ligase related protein	0	0.002262	0	0	0	0	0	0.000997	0	0	0	0.000354949
Protein Metabolism	Protein biosynthesis	tRNA aminoacylation, Asp and Asn	Asparaginyl-tRNA synthetase (EC 6.1.1.22), chloroplast	0	0	0	0	0	0	0.000722	0	0.000219	0	0	0.000197194
Protein Metabolism	Protein biosynthesis	tRNA aminoacylation, Asp and Asn	Aspartyl-tRNA synthetase (EC 6.1.1.12), mitochondrial	0	0	0	0	0	0	0.000722	0.000498	0	0	0	0.000118316
Amino Acids and Derivatives	Histidine Metabolism	Histidine Biosynthesis	ATP phosphoribosyltransferase regulatory subunit, divergent variant (EC 2.4.2.17)	0	0	0.002555	0	0	0.000487	0	0	0	0	0	0.007335608
RNA Metabolism	RNA processing and modification	ATP-dependent RNA helicases, bacterial	ATP-dependent RNA helicase SO1501	0	0	0	0	0	0.000487	0	0	0.000657	0	0	0.000276071
RNA Metabolism	RNA processing and modification	ATP-dependent RNA helicases, bacterial	ATP-dependent RNA helicase VCA0061	0	0	0	0	0	0	0	0	0	0.008811	0.073237	0.001419795
RNA Metabolism	RNA processing and modification	ATP-dependent RNA helicases, bacterial	ATP-dependent RNA helicase VF1437	0	0	0	0	0	0.000974	0	0	0	0	0.001863	0.002879029
RNA Metabolism	RNA processing and modification	ATP-dependent RNA helicases, bacterial	ATP-dependent RNA helicase YxiN	0	0	0	0	0	0	0.000722	0	0.000219	0	0	0.000670459
Regulation and Cell signaling	Quorum sensing and biofilm formation	Autoinducer 2 (AI-2) transport and processing (lsrACDBFGE operon)	Autoinducer 2 (AI-2) ABC transport system, fused AI2 transporter subunits and ATP-binding component	0	0.002262	0	0	0	0	0	0	0.000876	0	0	0.000394388
Regulation and Cell signaling	Quorum sensing and biofilm formation	Autoinducer 2 (AI-2) transport and processing (lsrACDBFGE operon)	Autoinducer 2 (AI-2) ABC transport system, periplasmic AI-2 binding protein LsrB	0	0	0	0	0	0	0.000722	0	0	0.002203	0	0.000552143

Regulation and Cell signaling	Programmed Cell Death and Toxin-antitoxin Systems	Murein hydrolase regulation and cell death	Autolysis histidine kinase LytS	0	0	0.002555	0	0	0	0	0	0.000657	0	0	0.000907091
Cell Wall and Capsule	Capsular and extracellular polysacchrides	Legionaminic Acid Biosynthesis	Bacillosamine/Legionaminic acid biosynthesis N-acetyltrasferase PglD (PseH and NeuD homolog)	0	0	0	0	0	0.000487	0	0.000498	0	0	0	0.000709898
Amino Acids and Derivatives	Aromatic amino acids and derivatives	Bacilysin biosynthesis	Bacilysin biosynthesis oxidoreductase BacC	0	0.002262	0	0	0	0	0.001444	0	0	0	0	0.000236633
Cofactors, Vitamins, Prosthetic Groups, Pigments	Tetrapyrroles	Chlorophyll Biosynthesis	Bacteriochlorophyllide c C8 methyltransefase BchQ	0.000348	0.002262	0	0	0	0	0	0	0	0	0	0.000197194
Metabolism of Aromatic Compounds	Peripheral pathways for catabolism of aromatic compounds	Benzoate degradation	benzoate MFS transporter BenK	0	0	0	0	0	0	0	0.000498	0.002189	0	0	0.000276071
Stress Response	Osmotic stress	Synthesis of osmoregulated periplasmic glucans	Beta-(1-->2)glucan export ATP-binding/permease protein NdvA (EC 3.6.3.42)	0	0	0	0	0	0	0	0	0.001314	0	0.000116	0.000591581
Carbohydrates	Di- and oligosaccharides	Trehalose Biosynthesis	Beta-amylase (EC 3.2.1.2)	0	0	0	0	0	0	0	0.000498	0.000876	0	0	0.000157755
Protein Metabolism	Selenoproteins	Glycine reductase, sarcosine reductase and betaine reductase	Betaine reductase component B alpha subunit (EC 1.21.4.4)	0	0	0	0	0	0	0	0.000498	0.000657	0	0	0.000512704
Carbohydrates	CO2 fixation	CO2 uptake, carboxysome	Bicarbonate transporter, bicarbonate binding protein	0	0	0.002555	0	0	0	0	0	0.001752	0	0	0.000788775
Carbohydrates	CO2 fixation	CO2 uptake, carboxysome	Bicarbonate transporter, bicarbonate binding protein 2	0	0	0	0	0	0	0	0.000498	0.001752	0	0	0.000788775
Amino Acids and Derivatives	Branched-chain amino acids	HMG CoA Synthesis	Biotin carboxyl carrier protein of methylcrotonyl-CoA carboxylase	0.001393	0	0	0	0	0	0	0.000997	0.000438	0	0	0
Fatty Acids, Lipids, and Isoprenoids	Isoprenoids	Myxoxanthophyll biosynthesis in Cyanobacteria	C-1'-hydroxylase CruF	0	0	0	0	0.000657	0	0.002166	0	0.002627	0	0	0
Membrane Transport	NULL	Transport of Zinc	Candidate zinc-binding lipoprotein ZinT	0	0	0	0	0	0	0	0.000498	0	0	0.000116	0.000552143
Cell Wall and Capsule	Capsular and extracellular polysacchrides	Serotype determining Capsular polysaccharide biosynthesis in Staphylococcus	Capsular polysaccharide synthesis enzyme Cap8M	0	0.002262	0	0	0	0	0	0	0.000219	0	0	0.000118316
Carbohydrates	CO2 fixation	Carboxysome	Carbon dioxide concentrating mechanism protein CcmM	0	0	0	0	0.000657	0.000487	0	0	0.000438	0	0	0
Fatty Acids, Lipids, and Isoprenoids	Fatty acids	Carnitine Metabolism in Microorganisms	Carnitine racemase (EC 5.-.-.-)	0	0	0	0	0.000657	0	0	0	0.001971	0	0	7.88775E-05
Cell Division and Cell Cycle	NULL	Bacterial Cytoskeleton	Cell division protein FtsN	0	0	0	0	0	0	0	0.000498	0.001533	0	0	0.000197194
Virulence, Disease and Defense	Adhesion	Streptococcus pyogenes recombinatorial zone	Chaperonin (heat shock protein 33)	0	0	0	0	0	0	0.002888	0	0.000219	0	0	0.000197194
DNA Metabolism	NULL	DNA structural proteins, bacterial	Chromosome partition protein MukF	0	0	0	0	0	0.000487	0	0	0.000657	0	0.000116	0
Clustering-based subsystems	NULL	PFGI-1-like cluster 1	Chromosome partitioning ATPase in PFGI-1-like cluster, ParA-like	0	0	0	0	0.000657	0.000487	0	0	0	0	0	7.88775E-05
Secondary Metabolism	Bacterial cytostatics, differentiation factors and antibiotics	Clavulanic acid biosynthesis	clavaldehyde dehydrogenase	0	0	0	0	0.00197	0	0	0	0.000438	0	0	0.000433826
Cell Wall and Capsule	Capsular and extracellular polysacchrides	Capsular Polysaccharides Biosynthesis and Assembly	CMP-Kdo synthetase, KpsU	0	0	0	0	0	0	0.000722	0.001495	0	0	0	0.00031551
Miscellaneous	Plant-Prokaryote DOE project	COG0523	Cobalamin synthesis protein/P47K family protein	0	0.002262	0	0	0	0	0.000722	0	0	0	0	7.88775E-05
Respiration	Electron donating reactions	Coenzyme F420-H2 dehydrogenase (methanophenazine)	Coenzyme F(420)H(2) dehydrogenase (methanophenazine) subunit FpoA	0	0	0	0	0.000657	0.000974	0	0	0	0	0	7.88775E-05
Respiration	Electron donating reactions	Coenzyme F420-H2 dehydrogenase (methanophenazine)	Coenzyme F(420)H(2) dehydrogenase (methanophenazine) subunit FpoC	0	0	0	0	0.000657	0.000487	0	0	0.000438	0	0	0
Respiration	Electron donating reactions	Coenzyme F420-H2 dehydrogenase (methanophenazine)	Coenzyme F(420)H(2) dehydrogenase (methanophenazine) subunit FpoJ	0	0	0	0.00249	0	0	0	0.000498	0	0	0	0.000197194
Respiration	Electron donating reactions	Coenzyme F420 hydrogenase	coenzyme F420-reducing hydrogenase, beta subunit homolog	0	0	0	0	0	0.000487	0	0	0	0	0.000116	0.000236633
Cofactors, Vitamins, Prosthetic Groups, Pigments	Folate and pterines	Methanopterin biosynthesis2	COG1548 family protein	0.000696	0	0	0	0	0.000487	0	0	0	0	0	0.000552143
Clustering-based subsystems	NULL	Conjugative transfer related cluster	Conjugative signal peptidase TrhF	0	0	0	0	0	0	0.000722	0.000498	0	0	0	0.000118316
DNA Metabolism	CRISPs	CRISPRs	CRISPR-associated protein, TM1800 family	0	0	0	0	0	0	0	0.000498	0.000219	0	0	3.94388E-05
DNA Metabolism	CRISPs	CRISPRs	CRISPR-associated RecB family exonuclease Cas4a	0	0	0	0	0	0	0	0.000498	0.000438	0	0	0.000236633
Fatty Acids, Lipids, and Isoprenoids	Isoprenoids	Carotenoids	CrtV-methyltransferase-like protein	0.002785	0	0	0	0	0	0.000722	0	0	0	0	0.000197194
Cofactors, Vitamins, Prosthetic Groups, Pigments	Riboflavin, FMN, FAD	Riboflavin, FMN and FAD metabolism	CTP-dependent archaeal riboflavin kinase	0	0	0	0	0	0	0.000722	0	0.001095	0	0	0.000236633
Virulence, Disease and Defense	NULL	Bacterial cyanide production and tolerance mechanisms	Cyanide insensitive terminal oxidase, subunit I	0	0	0	0	0	0	0	0	0.000438	0	0.000233	7.88775E-05
RNA Metabolism	Transcription	Transcription factors cyanobacterial RpoD-like sigma factors	Cyanobacteria-specific RpoD-like sigma factor, type-7	0	0	0	0	0.000657	0.000974	0	0	0	0	0	0.000157755
Amino Acids and Derivatives	Arginine; urea cycle, polyamines	Cyanophycin Metabolism	Cyanophycin synthase II	0	0.002262	0	0	0	0	0	0	0.000438	0	0	3.94388E-05
Cofactors, Vitamins, Prosthetic Groups, Pigments	Folate and pterines	YgfZ	Cystathionine gamma-synthase (EC 2.5.1.48) fungal type	0	0	0	0	0	0	0	0.000498	0.000438	0	0	7.88775E-05
Protein Metabolism	Protein biosynthesis	tRNA aminoacylation, Cys	Cysteinyl-tRNA synthetase (EC 6.1.1.16), mitochondrial	0	0	0	0	0	0.000974	0	0.000997	0	0	0	0.000118316
Miscellaneous	Plant-Prokaryote DOE project	At1g54520	Cytochrome b6-f complex subunit, apocytochrome f	0	0	0	0	0	0	0	0	0.000438	0	0.000349	7.88775E-05
Clustering-based subsystems	Cytochrome biogenesis	CBSS-196164.1.peg.1690	cytochrome oxidase assembly protein	0	0	0	0	0	0	0	0.000498	0.000876	0	0	0.000157755
Virulence, Disease and Defense	Resistance to antibiotics and toxic compounds	Methicillin resistance in Staphylococci	D-alanine--poly(phosphoribitol) ligase subunit 1 (EC 6.1.1.13)	0	0	0	0	0	0	0	0	0.000219	0	0.000116	0.000236633
Virulence, Disease and Defense	Resistance to antibiotics and toxic compounds	Bile hydrolysis	DamX, an inner membrane protein involved in bile resistance	0	0	0	0	0	0	0	0.000997	0.000876	0	0	0.000433826
Virulence, Disease and Defense	Bacteriocins, ribosomally synthesized antibacterial peptides	Marinocine, a broad-spectrum antibacterial protein	Dehydrogenase flavoprotein LodB	0	0	0	0	0.000657	0	0	0	0.000657	0	0	3.94388E-05
Carbohydrates	Monosaccharides	Deoxyribose and Deoxynucleoside Catabolism	Deoxyribose operon repressor, DeoR family	0	0	0	0	0.000657	0	0	0	0.000219	0	0	7.88775E-05
Carbohydrates	Central carbohydrate metabolism	Dihydroxyacetone kinases	DHA-specific IIA component	0	0	0	0	0.000657	0	0	0	0.000438	0	0	0.00031551
Carbohydrates	Central carbohydrate metabolism	Dihydroxyacetone kinases	DHA-specific phosphocarrier protein HPr	0.002089	0	0	0.00498	0	0	0	0	0	0	0	0.000512704
Clustering-based subsystems	NULL	EC699-706	Di/tripeptide permease YbgH	0	0	0	0	0	0	0.001444	0	0.000438	0	0	3.94388E-05
Cell Wall and Capsule	Gram-Positive cell wall components	Teichoic and lipoteichoic acids biosynthesis	diglucosyldiacylglycerol synthase (LTA membrane anchor synthesis)	0	0	0	0	0	0	0.001444	0	0.000438	0	0	7.88775E-05
Carbohydrates	One-carbon Metabolism	Methanogenesis from methylated compounds	Dimethylamine:corrinoid methyltransferase	0	0	0	0	0.000657	0.000487	0	0	0	0	0	0.000197194
Carbohydrates	Central carbohydrate metabolism	Peripheral Glucose Catabolism Pathways	DNA-binding response regulator GltR, controls specific porins for the entry of glucose	0	0	0	0	0.000657	0	0	0	0.001314	0	0	0.000118316
Amino Acids and Derivatives	Arginine; urea cycle, polyamines	Polyamine Metabolism	DNA-binding response regulator, LuxR family, near polyamine transporter	0	0	0	0	0.00197	0	0	0	0.000219	0	0	0.000354949
RNA Metabolism	Transcription	RNA polymerase archaeal	DNA-directed RNA polymerase subunit F (EC 2.7.7.6)	0	0	0	0	0	0	0	0.000498	0.001314	0	0	0.000118316
RNA Metabolism	Transcription	RNA polymerase archaeal	DNA-directed RNA polymerase subunit K (EC 2.7.7.6)	0	0	0	0	0	0	0.000722	0	0.000657	0	0	0.00063102
Membrane Transport	Protein and nucleoprotein secretion system, Type IV	Dot-Icm type IV secretion system	DotA protein	0	0	0	0	0.001313	0	0.000722	0.001993	0	0	0	0
Amino Acids and Derivatives	Alanine, serine, and glycine	Glycine and Serine Utilization	D-serine dehydratase transcriptional activator	0	0	0	0	0	0	0.000722	0.000498	0.001314	0	0	0

Cell Wall and Capsule	Capsular and extracellular polysacchrides	dTDP-rhamnose synthesis	dTDP-rhamnosyl transferase RfbF (EC 2.-.-.-)	0	0	0	0.00249	0	0	0	0	0.001095	0	0	0.000236633
Phages, Prophages, Transposable elements, Plasmids	Phages, Prophages	Phage DNA synthesis	dTMP thymidylate synthase	0	0	0	0	0	0	0.002166	0.000997	0	0	0	7.88775E-05
Cofactors, Vitamins, Prosthetic Groups, Pigments	Folate and pterines	Methanopterin biosynthesis2	DUF447 family protein	0	0	0	0.00249	0	0	0	0	0.000219	0	0	0.000157755
Membrane Transport	NULL	ECF class transporters	Duplicated ATPase component BL0693 of energizing module of predicted ECF transporter	0	0	0	0	0.000657	0.000487	0	0	0	0	0	0.000354949
Membrane Transport	NULL	ECF class transporters	Duplicated ATPase component glr2054 of energizing module of predicted ECF transporter	0	0	0	0	0	0	0.000722	0	0.000657	0	0	3.94388E-05
Membrane Transport	NULL	ECF class transporters	Duplicated ATPase component of energizing module of riboflavin ECF transporter	0	0	0	0	0	0.000487	0	0	0.000438	0	0	7.88775E-05
Respiration	Reverse electron transport	Mebrane bound hydrogenases	Energy conserving hydrogenase Eha associated protein (protein S)	0	0.004524	0	0.00249	0	0	0	0	0	0	0	0.00031551
Respiration	Reverse electron transport	Archaeal membrane bound hydrogenases	Energy conserving hydrogenase Ehb protein I	0.000696	0	0	0	0	0.000487	0	0	0	0	0	0.000197194
Fatty Acids, Lipids, and Isoprenoids	Fatty acids	Fatty Acid Biosynthesis FASI	Enoyl-[acyl-carrier-protein] reductase of FASI (EC 1.3.1.9)	0	0.002262	0	0	0	0	0	0	0.000219	0	0	7.88775E-05
Iron acquisition and metabolism	NULL	Iron acquisition in Vibrio	Enterobactin receptor IrgA	0	0	0	0	0	0	0	0.000498	0.000219	0	0.000116	0
Carbohydrates	Sugar alcohols	Ethanolamine utilization	Ethanolamine utilization polyhedral-body-like protein EutL	0	0.002262	0	0	0	0	0	0	0.000438	0	0	0.000236633
Regulation and Cell signaling	Proteolytic pathway	Regulatory Intramembrane Proteolysis Pathways	Eukaryotic translation initiation factor 2-alpha kinase 3 (PERK)	0	0	0	0	0.000657	0	0	0	0.000219	0	0	7.88775E-05
Regulation and Cell signaling	Proteolytic pathway	Regulatory Intramembrane Proteolysis Pathways	Eukaryotic translation initiation factor 2-alpha kinase 4 (GCN2)	0	0	0	0	0	0	0.000722	0.000498	0	0	0	0.000118316
Phages, Prophages, Transposable elements, Plasmids	Phages, Prophages	Phage Dual Exonuclease Exclusion	Exodeoxyribonuclease X (EC 3.1.11.-)	0	0	0	0	0.000657	0.000487	0	0	0	0	0	0.002405764
Motility and Chemotaxis	Social motility and nonflagellar swimming in bacteria	Control of Swarming in Vibrio and Shewanella species	Extracellular solute binding protein ScrB	0	0	0	0	0	0.000487	0	0	0	0	0.000116	0.001183163
Carbohydrates	One-carbon Metabolism	Methanogenesis	F420-dependent methylenetetrahydromethanopterin dehydrogenase (EC 1.5.99.9)	0	0.004524	0	0	0	0	0	0.000997	0	0	0	0.00031551
Carbohydrates	Di- and oligosaccharides	Unknown oligosaccharide utilization Sde 1396	Family 3 glycosyl hydrolase in unknown oligosaccharide utilization Sde 1396	0	0	0	0	0.000657	0	0	0	0.002627	0	0	0.000394388
Secondary Metabolism	Lipid-derived mediators	Cannabinoid biosynthesis	Fatty-acid amide hydrolase (EC 3.1.-.-)	0.000348	0	0	0	0	0.000487	0	0	0	0	0	0.000118316
Iron acquisition and metabolism	NULL	Iron acquisition in Vibrio	Ferric vibriobactin receptor ViuA	0	0	0	0	0	0	0	0.000498	0	0	0.000116	0.000276071
Iron acquisition and metabolism	NULL	Iron acquisition in Vibrio	Ferric vulnibactin receptor VuuA	0	0	0	0	0	0	0	0.000498	0	0	0.000466	0.000157755
Iron acquisition and metabolism	NULL	Iron acquisition in Vibrio	Ferrichrome-binding periplasmic protein precursor (TC 3.A.1.14.3)	0	0	0	0	0	0	0	0.000498	0.000438	0	0	0.000394388
Clustering-based subsystems	NULL	Cluster containing CofD-like protein and co-occuring with DNA repair	FIG001886: Cytoplasmic hypothetical protein	0	0	0	0.00498	0	0	0	0	0.001533	0	0	0.000709898
Clustering-based subsystems	NULL	PFGI-1-like cluster 1	FIG004780: hypothetical protein in PFGI-1-like cluster	0	0	0	0	0.000657	0	0.000722	0	0	0	0	0.000197194
Clustering-based subsystems	proteosome related	Cluster-based Subsystem Grouping Hypotheticals - perhaps Proteosome Related	FIG005453: Putative DeoR-family transcriptional regulator	0	0	0	0	0.001313	0	0.000722	0	0	0	0	3.94388E-05
Miscellaneous	Plant-Prokaryote DOE project	At4g38090	FIG005590: DegV family protein	0	0.002262	0	0	0	0	0	0.000498	0	0	0	7.88775E-05
Miscellaneous	Plant-Prokaryote DOE project	At4g10620 At3g57180 At3g47450	FIG007079: UPF0348 protein family	0	0	0	0.00249	0	0	0	0.000498	0	0	0	0.000788775
Clustering-based subsystems	NULL	Disulphide related cluster	FIG007303: uncharacterized protein	0	0	0	0	0	0.000487	0	0	0.000438	0	0	7.88775E-05
Fatty Acids, Lipids, and Isoprenoids	Fatty acids	Phospholipid and Fatty acid biosynthesis related cluster	FIG017861: hypothetical protein	0	0	0	0	0	0	0	0.001495	0.000876	0	0	0.000276071
Clustering-based subsystems	proteosome related	Cluster-based Subsystem Grouping Hypotheticals - perhaps Proteosome Related	FIG019733: possible DNA-binding protein	0	0	0.002555	0	0	0	0	0	0.000438	0	0	7.88775E-05
Fatty Acids, Lipids, and Isoprenoids	Fatty acids	Phospholipid and Fatty acid biosynthesis related cluster	FIG027190: Putative transmembrane protein	0.000348	0.002262	0	0	0	0	0	0	0	0	0	0.000394388
Clustering-based subsystems	NULL	CBSS-316273.3.peg.448	FIG042594: DUF1550 domain-containing protein	0	0	0	0	0	0.000487	0	0.000498	0	0	0	3.94388E-05
Clustering-based subsystems	NULL	CBSS-316273.3.peg.448	FIG045085: Hypothetical protein	0	0.004524	0	0	0	0	0	0	0.000438	0	0	7.88775E-05
Clustering-based subsystems	NULL	CBSS-316273.3.peg.2709	FIG046709: Hypothetical protein	0	0	0	0	0.001313	0	0	0.000498	0	0	0	7.88775E-05
Clustering-based subsystems	NULL	CBSS-214092.1.peg.3450	FIG138517: Putative lipid carrier protein	0	0	0	0.00249	0.000657	0	0	0	0	0	0	0.000394388
Clustering-based subsystems	NULL	CBSS-214092.1.peg.3450	FIG139928: Putative protease	0	0	0	0	0	0	0	0	0.001095	0	0.000233	0.000394388
Clustering-based subsystems	NULL	PFGI-1-like cluster 1	FIG141751: hypothetical protein in PFGI-1-like cluster	0	0	0	0	0.000657	0.000487	0	0	0	0	0	0.00094653
Phages, Prophages, Transposable elements, Plasmids	Transposable elements	CBSS-203122.12.peg.188	FIGfam050825	0	0	0	0	0	0	0	0	0.000438	0.002203	0	0.000118316
Phosphorus Metabolism	NULL	Phosphate-binding DING proteins	Filamentous haemagglutinin family outer membrane protein associated with VreARI signalling system	0.001044	0	0	0	0.000657	0	0	0	0	0	0	0.00031551
Motility and Chemotaxis	Flagellar motility in Prokaryota	Additional flagellar genes in Vibrionales	Flagellar protein FlgT	0	0	0	0	0	0	0	0.001993	0.00416	0	0	0.000157755
Regulation and Cell signaling	NULL	Two-component regulatory systems in Campylobacter	Flagellar sensory histidine kinase FlgS	0	0	0.002555	0	0	0.000974	0	0	0	0	0	0.001222601
Motility and Chemotaxis	Flagellar motility in Prokaryota	Flagellum	Flagellar trans-acting factor FliX	0	0	0	0	0	0.000974	0	0.000498	0	0	0	3.94388E-05
Motility and Chemotaxis	Flagellar motility in Prokaryota	Archaeal Flagellum	Flagellin FlaB1	0	0	0	0	0	0.000487	0	0.000498	0	0	0	7.88775E-05
Cofactors, Vitamins, Prosthetic Groups, Pigments	Riboflavin, FMN, FAD	Flavodoxin	Flavoprotein MioC	0	0	0	0.00498	0	0	0	0	0.002627	0	0	0.000118316
Cofactors, Vitamins, Prosthetic Groups, Pigments	Riboflavin, FMN, FAD	riboflavin to FAD	FMN adenylyltransferase, type 2 eukaryotic (EC 2.7.7.2)	0	0	0	0	0	0.001461	0	0	0.000219	0	0	0.000157755
Stress Response	Oxidative stress	Glutathione analogs: mycothiol	Formaldehyde dehydrogenase MscR, NAD/mycothiol-dependent (EC 1.2.1.66)	0	0.004524	0	0	0	0	0	0	0.000657	0	0	0.001025408
Carbohydrates	One-carbon Metabolism	Methanogenesis	Formylmethanofuran dehydrogenase (molybdenum) subunit C (EC 1.2.99.5)	0	0	0	0.00498	0	0.000487	0	0	0	0	0	0.000276071
Virulence, Disease and Defense	Resistance to antibiotics and toxic compounds	Fosfomycin resistance	Fosfomycin resistance protein FosA	0	0	0	0	0.000657	0.000974	0	0	0	0	0	0.000197194
Carbohydrates	Monosaccharides	Mannose Metabolism	Fructokinase in mannoside utilization gene cluster (EC 2.7.1.4)	0	0	0	0.00249	0	0	0	0	0.001533	0	0	0.000197194
Carbohydrates	Monosaccharides	Fructose utilization	Fructose-specific phosphocarrier protein HPr (EC 2.7.1.69)	0	0	0	0	0.000657	0	0	0	0	0	0.001281	3.94388E-05
Phages, Prophages, Transposable elements, Plasmids	Gene Transfer Agent (GTA)	Gene Transfer Agent	Gene Transfer Agent (GTA) ORFG08	0	0	0	0	0	0.000974	0	0.001993	0	0	0	0.000118316
Phages, Prophages, Transposable elements, Plasmids	Gene Transfer Agent (GTA)	Gene Transfer Agent	Gene Transfer Agent (GTA) ORFG10	0	0	0	0	0	0	0.000722	0.001495	0	0	0	7.88775E-05
Membrane Transport	Protein secretion system, Type II	General Secretion Pathway	General secretion pathway protein B	0	0	0	0	0	0	0	0.000498	0.002846	0	0	0.000512704
Miscellaneous	Plant-Prokaryote DOE project	COG1836	Geranylgeranyl/isoprenyl reductase	0	0	0	0	0	0.001461	0	0	0.002189	0	0	0.000433826
Carbohydrates	Monosaccharides	D-gluconate and ketogluconates metabolism	Glucose dehydrogenase (EC 1.1.99.10), membrane-bound, cytochrome c	0	0	0	0	0.001313	0	0.001444	0	0	0	0	3.94388E-05

Carbohydrates	Polysaccharides	Xyloglucan Utilization	Glucose oligosaccharide ABC transport system, permease protein 1	0	0.004524	0	0	0	0	0	0	0.000438	0	0	3.94388E-05
Carbohydrates	Polysaccharides	Xyloglucan Utilization	Glucose oligosaccharide ABC transport system, permease protein 2	0	0.002262	0	0	0	0	0	0	0.000438	0	0	7.88775E-05
Protein Metabolism	Protein biosynthesis	tRNA aminoacylation, Glu and Gln	Glutamyl-tRNA synthetase (EC 6.1.1.17), mitochondrial	0	0	0	0	0	0	0	0	0.000438	0	0.000233	0.000197194
Stress Response	Oxidative stress	Glutathione: Non-redox reactions	Glutathione S-transferase, unnamed subgroup 2 (EC 2.5.1.18)	0	0	0	0	0	0	0	0	0.000876	0.022028	0.001048	0
Stress Response	Oxidative stress	Redox-dependent regulation of nucleus processes	Glyceraldehyde-3-phosphate dehydrogenase (EC 1.2.1.12) (GAPDH)	0	0	0	0	0	0	0	0.000498	0.000438	0	0	0.000236633
Carbohydrates	Sugar alcohols	Glycerol fermenation to 1,3-propanediol	Glycerol dehydratase small subunit (EC 4.2.1.30)	0	0	0	0	0	0.000487	0	0.000498	0	0	0	7.88775E-05
Amino Acids and Derivatives	Arginine; urea cycle, polyamines	Putrescine utilization pathways	Glycine/D-amino acid oxidase (deaminating) in putrescine utilization cluster	0	0	0	0	0.000657	0.000487	0	0	0	0	0	0.000236633
Iron acquisition and metabolism	NULL	Campylobacter Iron Metabolism	Haemin uptake system ATP-binding protein	0	0	0	0	0	0.000974	0	0.000997	0	0	0	3.94388E-05
Iron acquisition and metabolism	NULL	Campylobacter Iron Metabolism	Haemin uptake system permease protein	0	0.002262	0	0	0.000657	0	0	0	0	0	0	0.00031551
Clustering-based subsystems	NULL	CBSS-630.2.peg.3360	HAM1-like protein	0	0	0	0	0	0	0.001444	0	0.000438	0	0	0.000236633
Metabolism of Aromatic Compounds	NULL	p-cymene degradation	HCOMODA decarboxylase	0	0	0	0	0.000657	0.000487	0	0	0	0	0	7.88775E-05
Virulence, Disease and Defense	Resistance to antibiotics and toxic compounds	Cobalt-zinc-cadmium resistance	Heavy metal resistance transcriptional regulator HmrR	0	0	0	0	0	0	0	0.000498	0.000219	0	0	7.88775E-05
Iron acquisition and metabolism	NULL	Iron Scavenging cluster in Thermus	Heme ABC transporter, cell surface heme and hemoprotein receptor HmuT	0	0	0	0	0.000657	0	0.001444	0	0	0	0	7.88775E-05
Iron acquisition and metabolism	NULL	Heme, hemin uptake and utilization systems in GramPositives	Heme ABC type transporter HtsABC, permease protein HtsC	0	0	0	0.00498	0	0.000487	0	0	0.000219	0	0	0
Iron acquisition and metabolism	NULL	Hemin transport system	hemin ABC transporter, permease protein	0.000348	0	0	0	0	0	0	0	0.000876	0	0	3.94388E-05
Phosphorus Metabolism	NULL	Phosphate-binding DING proteins	Hemolysin activation/secretion protein associated with VreARI signalling system	0	0	0	0	0	0.000487	0	0	0.000876	0	0	0.000433826
Regulation and Cell signaling	NULL	Sex pheromones in Enterococcus faecalis and other Firmicutes	Heptaprenyl diphosphate synthase component I (EC 2.5.1.30)	0	0	0	0	0	0.000487	0	0	0	0	0.000233	0.000749336
Cofactors, Vitamins, Prosthetic Groups, Pigments	Quinone cofactors	Tocopherol Biosynthesis	Homogentisate prenyltransferase	0	0	0	0	0	0.000974	0	0	0.001971	0	0	0.000354949
Nucleosides and Nucleotides	NULL	Hydantoin metabolism	Hydantoin utilization protein A	0	0	0.002555	0	0	0	0	0	0.000219	0	0	0.000118316
Respiration	NULL	Formate hydrogenase	hydrogenase, HycC subunit, putative	0	0	0	0	0.000657	0	0	0	0.000219	0	0	0.000197194
Respiration	NULL	Formate hydrogenase	Hydrogenase-4 component C (EC 1.-.-.-)	0	0	0	0.00249	0	0.000487	0	0	0.000219	0	0	0
RNA Metabolism	RNA processing and modification	Wyeosine-MimG Biosynthesis	Hypothetical ATP-binding protein, containing DUF265 domain	0	0.002262	0	0	0.001313	0	0	0	0	0	0	0.000197194
Miscellaneous	Plant-Prokaryote DOE project	At1g54520	Hypothetical glycine rich membrane protein DUF1517	0	0	0	0	0	0.000974	0	0	0.000438	0	0.000233	0
Clustering-based subsystems	Clustering-based subsystems	Putative diaminopropionate ammonia-lyase cluster	Hypothetical hydrolase YgeZ (EC 3.-.-.-)	0	0	0	0	0.000657	0.002435	0	0	0	0	0	0.000157755
Miscellaneous	Plant-Prokaryote DOE project	Conserved gene cluster possibly involved in RNA metabolism	Hypothetical protein DUF901, similar to C-terminal domain of ribosome protection-type Tc-resistance proteins	0	0	0	0	0	0	0	0	0.000438	0	0.000349	7.88775E-05
Clustering-based subsystems	Biosynthesis of galactoglycans and related lipopolysacharides	CBSS-376686.6.peg.291	hypothetical protein FP1254	0	0	0	0	0	0	0	0	0.000438	0	0.002445	0.003273417
Carbohydrates	Central carbohydrate metabolism	Ethylmalonyl-CoA pathway of C2 assimilation	Hypothetical protein in cluster with Mesaconyl-CoA hydratase	0	0	0	0	0	0.000487	0.000722	0.000498	0	0	0	0
Virulence, Disease and Defense	Resistance to antibiotics and toxic compounds	Cobalt-zinc-cadmium resistance	Hypothetical protein involved in heavy metal export	0	0	0	0	0	0.000487	0	0	0.001971	0	0	0.001064846
Clustering-based subsystems	Probably Pyrimidine biosynthesis-related	CBSS-306254.1.peg.1508	Hypothetical protein probably associated with Carbamoyl-phosphate synthase	0	0	0	0	0	0	0	0.000997	0	0	0.000233	0.003707243
Phages, Prophages, Transposable elements, Plasmids	Phages, Prophages	Staphylococcal phi-Mu50B-like prophages	Hypothetical protein, PV83 orf 20 homolog [SA bacteriophages 11, Mu50B]	0	0	0	0	0	0	0.002888	0.000498	0	0	0	7.88775E-05
Membrane Transport	Protein and nucleoprotein secretion system, Type IV	Conjugative transfer	IncF plasmid conjugative transfer DNA-nicking and unwinding protein TraI	0	0	0	0	0.000657	0.000487	0	0	0	0	0	3.94388E-05
Membrane Transport	Protein and nucleoprotein secretion system, Type IV	Conjugative transfer	IncQ plasmid conjugative transfer protein TraP	0	0	0	0	0	0.000487	0	0	0	0	0.000116	3.94388E-05
Phages, Prophages, Transposable elements, Plasmids	Plasmid related functions	Plasmid-encoded T-DNA transfer	Inner membrane protein forms channel for type IV secretion of T-DNA complex, VirB3	0	0	0	0	0	0.000487	0	0	0.000657	0	0	0.00031551
Membrane Transport	Protein and nucleoprotein secretion system, Type IV	pVir Plasmid of Campylobacter	Inner membrane protein forms channel for type IV secretion of T-DNA complex, VirB8	0	0	0	0.00249	0	0	0	0.000997	0	0	0	0.00063102
Clustering-based subsystems	NULL	CBSS-83333.1.peg.946	Inner membrane protein YccF	0	0	0	0	0	0	0	0.000498	0.001971	0	0	0.000433826
Clustering-based subsystems	NULL	Conserved cluster in Enterobacteriaceae downstream from YqjA, a DedA family protein	Inner membrane protein YqjF	0.002437	0	0	0	0	0	0	0	0.000219	0	0	0.000552143
Carbohydrates	Central carbohydrate metabolism	Peripheral Glucose Catabolism Pathways	Integral membrane sensor signal transduction histidine kinase (EC 2.7.13.3), glucose catabolism cluster	0	0	0	0	0	0	0	0	0.001314	0	0.002562	0.000276071
Virulence, Disease and Defense	Invasion and intracellular resistance	Listeria surface proteins: Internalin-like proteins	Internalin E (LPXTG motif)	0	0.002262	0	0	0.00197	0	0	0	0	0	0	0.000394388
Virulence, Disease and Defense	Invasion and intracellular resistance	Listeria surface proteins: Internalin-like proteins	Internalin-like protein (LPXTG motif) Lmo0333 homolog	0	0	0	0	0	0.000974	0	0.000498	0.000219	0	0	0
Metabolism of Aromatic Compounds	Metabolism of central aromatic intermediates	Central meta-cleavage pathway of aromatic compound degradation	Intradiol ring-cleavage dioxygenase (EC 1.13.11.1)	0	0	0	0	0	0.000487	0	0	0.000438	0	0	7.88775E-05
Cofactors, Vitamins, Prosthetic Groups, Pigments	Folate and pterines	YgfZ-Iron	iron (metal) dependent repressor, DtxR family	0	0	0	0	0	0	0	0.000498	0	0	0.000233	0.000157755
Iron acquisition and metabolism	Siderophores	Siderophore Yersiniabactin Biosynthesis	iron aquisition yersiniabactin synthesis enzyme (YbtT,resembles thioesterases)	0	0	0	0	0.000657	0	0	0	0.000438	0	0	3.94388E-05
Iron acquisition and metabolism	NULL	Transport of Iron	Iron starvation-induced chlorophyll a(b) binding protein IsiA, photosystem II CP43 protein (PsbC) homolog	0	0	0	0	0	0	0	0.000498	0.001752	0	0.001164	0
Iron acquisition and metabolism	NULL	Iron(III) dicitrate transport system Fec	Iron(III) dicitrate transport system, periplasmic iron-binding protein FecB (TC 3.A.1.14.1)	0	0	0	0	0	0.000487	0	0	0	0	0.000233	3.94388E-05
Carbohydrates	Central carbohydrate metabolism	TCA Cycle	Isocitrate dehydrogenase [NAD] subunit I, mitochondrial precursor (EC 1.1.1.41)	0	0	0	0	0.001313	0	0	0	0	0.004406	0	0.000157755
Amino Acids and Derivatives	Branched-chain amino acids	Ketoisovalerate oxidoreductase	Ketoisovalerate oxidoreductase subunit VorC (EC 1.2.7.7)	0	0	0	0	0	0.000974	0	0	0.000219	0	0	7.88775E-05
Carbohydrates	Organic acids	Lactate utilization	Lactate-responsive regulator LldR in Actinobacteria, GntR family	0	0	0	0	0	0.000974	0	0	0.000219	0	0	7.88775E-05
Amino Acids and Derivatives	Lysine, threonine, methionine, and cysteine	Lysine degradation	L-aminoadipate-semialdehyde dehydrogenase large subunit (EC 1.2.1.31)	0	0	0.00511	0	0	0.000487	0	0	0	0	0	0.000157755
Cell Wall and Capsule	Capsular and extracellular polysacchrides	Legionaminic Acid Biosynthesis	Legionaminic acid biosynthesis protein PtmA	0	0	0	0	0	0.000974	0.000722	0	0	0	0	0.000828214
Cell Division and Cell Cycle	NULL	Cyanobacterial Circadian Clock	Light dependent period modulator LdpA	0	0	0	0	0.000657	0	0	0	0.000438	0	0	7.88775E-05
Cofactors, Vitamins, Prosthetic Groups, Pigments	Lipoic acid	Lipoic acid metabolism	Lipoate synthase, cyanobacterial paralog	0	0	0.002555	0	0	0	0	0	0.000438	0	0	0.000118316
Cell Wall and Capsule	Capsular and extracellular polysacchrides	Phosphorylcholine incorporation in LPS	Lipopolysaccharide cholinephosphotransferase LicD1 (EC 2.7.8.-)	0	0	0	0	0	0.000487	0.000722	0	0	0	0	0.000157755
Protein Metabolism	Protein degradation	Proteasome bacterial	Lon-like protease with PDZ domain	0	0	0	0.00249	0	0	0	0	0.000657	0	0	0.000236633
Cell Wall and Capsule	Capsular and extracellular polysacchrides	Colanic acid biosynthesis	Low molecular weight protein-tyrosine-phosphatase Wzb (EC 3.1.3.48)	0	0	0	0	0	0.000487	0	0	0.000876	0	0	0.000512704

Regulation and Cell signaling	Quorum sensing and biofilm formation	Autoinducer 2 (AI-2) transport and processing (lsrACDBFGE operon)	LsrR, transcriptional repressor of lsr operon	0	0	0	0	0.001313	0.000487	0	0	0	0	0	0.000157755
Protein Metabolism	Protein biosynthesis	Ribosome LSU eukaryotic and archaeal	LSU ribosomal protein L21e	0	0	0	0	0	0	0.000722	0	0.000657	0	0	0.00031551
Protein Metabolism	Protein biosynthesis	Ribosome LSU chloroplast	LSU ribosomal protein L27p, chloroplast	0	0	0	0.00249	0	0	0	0.000498	0	0.002203	0	0
Protein Metabolism	Protein biosynthesis	Ribosome LSU eukaryotic and archaeal	LSU ribosomal protein L35Ae	0	0	0	0	0.001313	0.000487	0	0	0	0	0	0.000157755
Protein Metabolism	Protein biosynthesis	Ribosome LSU eukaryotic and archaeal	LSU ribosomal protein L35e (L29p)	0	0	0	0	0	0	0	0.000498	0.000657	0	0	3.94388E-05
Protein Metabolism	Protein biosynthesis	Ribosome LSU chloroplast	LSU ribosomal protein L7/L12 (L23e), chloroplast	0	0	0	0.00249	0	0	0	0.000997	0	0	0	3.94388E-05
Carbohydrates	Monosaccharides	L-ascorbate utilization (and related gene clusters)	L-xylulose 5-phosphate 3-epimerase (EC 5.1.3.-) homolog	0	0	0	0	0.000657	0.000487	0	0	0	0	0	3.94388E-05
Amino Acids and Derivatives	Lysine, threonine, methionine, and cysteine	Lysine degradation	Lysine decarboxylase 2, constitutive (EC 4.1.1.18)	0	0	0	0	0	0.000974	0	0.000498	0	0	0	7.88775E-05
Clustering-based subsystems	NULL	CBSS-316407.3.peg.2816	Lysine efflux permease	0	0	0	0	0	0.000487	0	0	0.000876	0	0	0.000236633
Amino Acids and Derivatives	Lysine, threonine, methionine, and cysteine	Lysine degradation	Lysine ketoglutarate reductase (EC 1.5.1.8) (LOR) (LKR)	0	0	0	0	0	0	0	0.000997	0.000219	0	0	0.000709898
Regulation and Cell signaling	NULL	DNA-binding regulatory proteins, strays	LysR family transcriptional regulator PA2877	0	0	0	0	0	0.000487	0	0	0.000876	0	0	0.000276071
Regulation and Cell signaling	NULL	DNA-binding regulatory proteins, strays	LysR family transcriptional regulator STM2281	0	0.004524	0	0	0	0	0	0.001495	0	0	0	7.88775E-05
Regulation and Cell signaling	NULL	DNA-binding regulatory proteins, strays	LysR family transcriptional regulator YcjZ	0	0	0	0	0	0	0	0	0.000438	0	0.003609	0.000473265
Carbohydrates	Sugar alcohols	Inositol catabolism	Major myo-inositol transporter IolT	0	0	0	0	0	0.000487	0	0	0.000657	0	0	0.000512704
Carbohydrates	NULL	Sugar utilization in Thermotogales	Maltose/maltodextrin ABC transporter 2, permease protein MalF2	0	0	0	0	0.000657	0	0.000722	0.000498	0	0	0	0
Membrane Transport	NULL	Agrobacterium opine transport	Mannopinic acid transporter permease protein MoaB	0	0	0	0	0.000657	0	0	0	0.000219	0	0	7.88775E-05
Carbohydrates	Monosaccharides	Mannose Metabolism	Mannoside ABC transport system, permease protein 2	0	0.002262	0	0	0	0	0	0	0	0.008811	0	0.000394388
Cell Wall and Capsule	Gram-Negative cell wall components	Vibrio Core Oligosaccharide Biosynthesis	Mannosyltransferase OCH1 and related enzymes	0	0	0	0	0	0	0.000722	0	0.000219	0	0	3.94388E-05
Nucleosides and Nucleotides	Detoxification	Nucleoside triphosphate pyrophosphohydrolase MazG	MazG-related protein	0	0	0	0	0	0	0.000722	0.000498	0.000876	0	0	0
Carbohydrates	Di- and oligosaccharides	Melibiose Utilization	Melibiose carrier protein, Na+/melibiose symporter	0	0	0	0	0	0	0.000722	0	0.000219	0	0	0.000552143
Miscellaneous	NULL	YbbK	Membrane protease family protein y2843	0	0	0	0	0	0	0.007943	0.003489	0.000438	0	0	0
Regulation and Cell signaling	Quorum sensing and biofilm formation	Symbiotic colonization and sigma-dependent biofilm formation gene cluster	Membrane protein SypL involved in exopolysaccharide production	0	0	0	0	0.000657	0	0.000722	0	0.000438	0	0	0
Amino Acids and Derivatives	Lysine, threonine, methionine, and cysteine	Lysine Biosynthesis DAP Pathway	Meso-diaminopimelate D-dehydrogenase (EC 1.4.1.16)	0	0.002262	0	0	0	0	0	0	0	0	0.000233	0.000236633
Carbohydrates	Central carbohydrate metabolism	Soluble methane monooxygenase (sMMO)	Methane monooxygenase component A beta chain (EC 1.14.13.25)	0	0	0	0	0	0	0	0.000498	0.000438	0	0	3.94388E-05
Protein Metabolism	Protein biosynthesis	tRNA aminoacylation, Met	Methionyl-tRNA synthetase, clostridial paralog	0.002785	0	0.002555	0	0	0	0	0	0	0	0	0.000197194
Carbohydrates	One-carbon Metabolism	Methanogenesis	Methyl coenzyme M reductase associated protein	0	0.002262	0	0	0	0	0	0	0	0.004406	0	0.00031551
Motility and Chemotaxis	NULL	Bacterial Chemotaxis	Methyl-accepting chemotaxis protein III (ribose and galactose chemoreceptor protein)	0	0	0	0	0	0	0	0	0	0.002203	0.000349	0.000236633
Miscellaneous	Plant-Prokaryote DOE project	At5g38900	MFS family multidrug efflux protein in Burkholderiaceae, unknown substrate	0	0.002262	0	0	0	0	0	0	0.000219	0	0	7.88775E-05
Phages, Prophages, Transposable elements, Plasmids	Plasmid related functions	Plasmid-encoded T-DNA transfer	Minor pilin of type IV secretion complex, VirB5	0	0	0	0	0	0	0	0.000498	0.000657	0	0	0.000788775
Carbohydrates	Di- and oligosaccharides	Maltose and Maltodextrin Utilization	Mlc, transcriptional repressor of MalT (the transcriptional activator of maltose regulon) and manXYZ operon	0	0	0	0	0	0.000487	0	0	0.000219	0	0	0.000709898
Virulence, Disease and Defense	Resistance to antibiotics and toxic compounds	Multidrug Resistance Operon mdtRP of Bacillus	Multidrug efflux transporter MdtP	0	0	0	0.00249	0	0	0	0	0.000219	0	0	0.000552143
Virulence, Disease and Defense	Resistance to antibiotics and toxic compounds	The mdtABCD multidrug resistance cluster	Multidrug transporter MdtB	0	0	0	0	0	0	0.000722	0	0.001314	0	0	0.000552143
DNA Metabolism	DNA repair	DNA repair, bacterial MutL-MutS system	MutS domain protein, family 4	0	0	0.002555	0	0	0	0	0	0.002627	0	0	0.000157755
Stress Response	Oxidative stress	Glutathione analogs: mycothiol	Mycothiol S-conjugate amidase Mca	0	0	0	0	0	0	0	0.000498	0.000219	0	0	0.000157755
Respiration	NULL	Formate hydrogenase	NAD-dependent formate dehydrogenase delta subunit	0	0	0	0	0	0.000487	0	0	0.000438	0	0	0.000118316
Miscellaneous	Plant-Prokaryote DOE project	At1g10830	NADH dehydrogenase subunit 5, Involved in CO2 fixation	0	0	0	0	0	0.000487	0	0	0.000438	0	0	0.000394388
Respiration	Electron donating reactions	Respiratory Complex I	NADH dehydrogenase subunit 6	0	0	0	0	0	0	0	0.000498	0.000219	0	0	0.000157755
Miscellaneous	Plant-Prokaryote DOE project	At4g17370	NADPH-dependent aldo-keto reductase, similarity to GRE3, stress-induced in yeast	0	0	0	0	0	0	0	0.000498	0.000438	0	0	0.000512704
Carbohydrates	Central carbohydrate metabolism	Methylglyoxal Metabolism	NADPH-dependent methylglyoxal reductase (D-lactaldehyde dehydrogenase)	0	0	0	0	0	0	0	0.000498	0.000657	0	0	0.000236633
Membrane Transport	NULL	Transport of Nickel and Cobalt	Nickel transport ATP-binding protein NikE (TC 3.A.1.5.3)	0	0	0	0	0	0.000487	0	0	0.000438	0	0	7.88775E-05
Nitrogen Metabolism	NULL	Nitrogen fixation	NifY protein	0	0	0	0	0	0.000974	0.000722	0	0	0	0	0.000354949
Nitrogen Metabolism	NULL	Nitrosative stress	Nitric oxide reductase FlRd-NAD(+) reductase (EC 1.18.1.-)	0	0	0	0.00249	0	0	0	0	0	0	0.000349	0.000276071
Secondary Metabolism	Plant Hormones	Auxin biosynthesis	Nitrilase 2 (EC 3.5.5.1)	0.000696	0	0	0.00249	0	0	0	0	0	0	0	0.001419795
Nitrogen Metabolism	NULL	Nitrate and nitrite ammonification	Nitrite reductase probable electron transfer 4Fe-S subunit (EC 1.7.1.4)	0	0	0	0	0.000657	0	0	0	0.000438	0	0	0.000354949
Nitrogen Metabolism	NULL	Nitrogen fixation	Nitrogenase vanadium-cofactor synthesis protein VnfE	0.002437	0	0	0	0	0	0.001444	0	0	0	0.000233	0
Carbohydrates	Central carbohydrate metabolism	Ethylmalonyl-CoA pathway of C2 assimilation	NnrU family protein in cluster with Mesaconyl-CoA hydratase	0	0	0	0	0	0	0	0.001993	0.001314	0	0	0.000157755
Iron acquisition and metabolism	NULL	Campylobacter Iron Metabolism	Nonheme iron-containing ferritin	0	0	0	0	0.000657	0.000487	0	0	0	0	0	0.000236633
Carbohydrates	Central carbohydrate metabolism	Entner-Doudoroff Pathway	Non-phosphorylating glyceraldehyde-3-phosphate dehydrogenase (NAD)	0	0	0	0	0.000657	0	0	0	0	0.004406	0	0.000236633
Membrane Transport	NULL	Agrobacterium opine transport	Nopaline transporter periplasmic substrate-binding protein NocT	0	0	0	0	0.000657	0.001948	0	0	0	0	0	7.88775E-05
Membrane Transport	NULL	Agrobacterium opine transport	Nopaline transporter permease protein NocQ	0	0	0	0	0.001313	0.001948	0	0	0.000876	0	0	0
Secondary Metabolism	Bacterial cytostatics, differentiation factors and antibiotics	Nonribosomal peptide synthetases (NRPS) in Frankia sp. Ccl3	NRPS module 3 PG-PG	0.002785	0	0	0	0	0	0	0.000997	0.000876	0	0	0
Carbohydrates	Aminosugars	Neotrehalosadiamine (NTD) Biosynthesis Operon	NTD biosynthesis operon putative oxidoreductase NtdC (EC 1.-.-.-)	0	0	0	0	0.000657	0	0.002888	0	0	0	0	0.000276071
Nucleosides and Nucleotides	Detoxification	Nudix proteins (nucleoside triphosphate hydrolases)	Nudix family (d)NDPase DR0975	0	0	0	0	0	0	0.000722	0.000997	0.000438	0	0	0
Protein Metabolism	Protein biosynthesis	tRNA aminoacylation, Cys	O-phosphoseryl-tRNA(Cys) synthetase	0.002785	0	0	0	0	0	0.000722	0	0	0	0	0.000433826
Protein Metabolism	Selenoproteins	Selenocysteine metabolism	O-phosphoseryl-tRNA(Sec) selenium transferase	0	0.002262	0	0	0.000657	0	0	0	0	0	0	0.000157755
Metabolism of Aromatic Compounds	Peripheral pathways for catabolism of aromatic compounds	Benzoate degradation	Ortho-halobenzoate 1,2-dioxygenase beta-ISP protein OhbA	0	0	0	0	0	0	0	0.000997	0.000657	0	0	3.94388E-05

Stress Response	Osmotic stress	Osmoprotectant ABC transporter YehZYXW of Enterobacteriales	Osmoprotectant ABC transporter inner membrane protein YehW	0	0	0	0	0.000657	0	0	0	0.000219	0	0	7.88775E-05
Stress Response	Osmotic stress	Osmoprotectant ABC transporter YehZYXW of Enterobacteriales	Osmoprotectant ABC transporter permease protein YehY	0	0	0	0	0	0	0	0.000498	0.000438	0	0	0.000118316
Stress Response	Osmotic stress	Choline and Betaine Uptake and Betaine Biosynthesis	Osmotically activated L-carnitine/choline ABC transporter, substrate-binding protein OpuCC	0	0	0	0	0.001313	0	0	0	0.000219	0	0	0.000157755
Iron acquisition and metabolism	Siderophores	Siderophore Pyoverdine	Outer membrane ferripyoverdine receptor	0	0	0	0	0	0	0	0.000498	0.000219	0	0	0.000118316
Virulence, Disease and Defense	Resistance to antibiotics and toxic compounds	MexE-MexF-OprN Multidrug Efflux System	Outer membrane protein OprN	0	0	0	0	0.000657	0	0	0	0.001314	0	0	0.000276071
Stress Response	Dessication stress	O-antigen capsule important for environmental persistence	Oxidoreductase YihU	0	0	0	0	0	0.000487	0	0.000997	0	0	0	0.000276071
Cofactors, Vitamins, Prosthetic Groups, Pigments	Coenzyme A	Coenzyme A Biosynthesis	Pantoate kinase, archaeal (EC 2.7.1.-)	0	0.002262	0	0	0	0	0	0.000997	0	0	0	3.94388E-05
Regulation and Cell signaling	Programmed Cell Death and Toxin-antitoxin Systems	Toxin-antitoxin systems (other than RelBE and MazEF)	ParE toxin protein	0	0	0	0	0	0.000974	0	0	0	0.002203	0	0.001380356
Metabolism of Aromatic Compounds	NULL	p-cymene degradation	p-cumic alcohol dehydrogenase (CymB)	0	0	0	0	0	0.000487	0	0	0.000219	0	0	0.000118316
Virulence, Disease and Defense	Resistance to antibiotics and toxic compounds	Methicillin resistance in Staphylococci	Penicillin-binding protein 1A/1B (PBP1)	0	0	0	0.00498	0	0	0	0	0.000438	0	0	0.00031551
RNA Metabolism	RNA processing and modification	16S rRNA modification within P site of ribosome	Penicillin-binding protein 2B	0	0	0	0.00498	0	0	0	0	0.000438	0	0	3.94388E-05
Cell Division and Cell Cycle	NULL	Bacterial Cytoskeleton	Penicillin-binding protein PBP2	0	0	0	0	0.000657	0.000974	0.000722	0	0	0	0	0
Clustering-based subsystems	Isoprenoid/cell wall biosynthesis: PREDICTED UNDECAPRENYL DIPHOSPHATE PHOSPHATASE	CBSS-83331.1.peg.3039	penicillin-binding protein, putative	0	0	0	0	0	0	0.000722	0	0.000219	0	0	0.000276071
Membrane Transport	ABC transporters	ABC transporter peptide (TC 3.A.1.5.5)	Peptide transport system permease protein sapB (TC 3.A.1.5.5)	0	0	0	0	0	0	0	0	0.000219	0	0.000466	0.000709898
Membrane Transport	Protein and nucleoprotein secretion system, Type IV	Vir-like type 4 secretion system	Peptidoglycan hydrolase VirB1, involved in T-DNA transfer	0	0	0	0	0.000657	0	0.000722	0	0.000219	0	0	0
Cell Wall and Capsule	NULL	Recycling of Peptidoglycan Amino Acids	Periplasmic Murein Peptide-Binding Protein MppA	0	0	0	0	0	0	0	0.000498	0.000657	0	0	0.000157755
Potassium metabolism	NULL	pH adaptation potassium efflux system	pH adaption potassium efflux system protein PhaE	0	0	0	0	0	0	0.002166	0.001993	0	0	0	3.94388E-05
Potassium metabolism	NULL	pH adaptation potassium efflux system	pH adaption potassium efflux system protein PhaG	0	0	0	0	0	0.000974	0.000722	0	0.000657	0	0	0
Phages, Prophages, Transposable elements, Plasmids	Phages, Prophages	Phage baseplate proteins	Phage baseplate hub	0	0	0	0	0	0.000487	0.000722	0.002492	0	0	0	0
Phages, Prophages, Transposable elements, Plasmids	Phages, Prophages	Phage Dual Exonuclease Exclusion	Phage DNA binding ATPase	0	0	0	0	0.001313	0.001461	0	0.001993	0	0	0	0
Phages, Prophages, Transposable elements, Plasmids	Phages, Prophages	Phage packaging machinery	Phage DNA binding protein	0	0	0	0	0	0.000974	0	0.001993	0	0	0	3.94388E-05
Phages, Prophages, Transposable elements, Plasmids	Phages, Prophages	Phage capsid proteins	Phage minor capsid protein	0	0	0	0	0.000657	0	0.000722	0.000997	0	0	0	0
Phages, Prophages, Transposable elements, Plasmids	Phages, Prophages	Phage neck proteins	Phage neck protein	0	0	0	0	0	0.000487	0	0.000997	0	0	0	7.88775E-05
Stress Response	NULL	Phage shock protein (psp) operon	Phage shock protein B	0	0	0	0	0.001313	0	0	0	0.003722	0	0	0.000236633
Clustering-based subsystems	NULL	CBSS-159087.4.peg.2189	Phage T7 exclusion protein associated hypothetical protein	0	0	0	0	0	0	0.000722	0	0.000657	0	0	0.000157755
Phages, Prophages, Transposable elements, Plasmids	Phages, Prophages	Phage tail proteins 2	Phage tail assembly protein	0	0	0	0	0.001313	0.000487	0	0.000498	0	0	0	0
Phages, Prophages, Transposable elements, Plasmids	Phages, Prophages	Phage tail proteins 2	Phage tail connector	0	0	0.007664	0	0	0	0.002888	0	0	0	0	0.000276071
Phages, Prophages, Transposable elements, Plasmids	Phages, Prophages	Phage packaging machinery	Phage terminase small subunit	0	0	0	0	0	0.000487	0	0.002492	0	0	0	0.000157755
Phages, Prophages, Transposable elements, Plasmids	Phages, Prophages	Prophage lysogenic conversion modules	Phage-associated cell wall hydrolase	0	0	0	0	0.000657	0.000487	0	0.000997	0	0	0	0
Phages, Prophages, Transposable elements, Plasmids	Phages, Prophages	Phage packaging machinery	Phage-related capsid packaging protein	0	0	0	0	0	0.000487	0	0	0.000438	0	0	0.000118316
Metabolism of Aromatic Compounds	Peripheral pathways for catabolism of aromatic compounds	Phenol hydroxylase	Phenol hydroxylase, assembly protein DmpK	0	0	0	0	0	0	0	0.000498	0.000876	0	0	0.000749336
Cofactors, Vitamins, Prosthetic Groups, Pigments	Tetrapyrroles	Chlorophyll Degradation	Pheophorbide a oxygenase (EC 1.14.-.-)	0	0	0	0	0	0.000487	0	0	0.003065	0	0.000233	0
Regulation and Cell signaling	NULL	Sex pheromones in Enterococcus faecalis and other Firmicutes	Pheromone response surface protein PrgC	0	0	0	0	0	0	0.002166	0	0.000438	0	0	3.94388E-05
Regulation and Cell signaling	NULL	Sex pheromones in Enterococcus faecalis and other Firmicutes	Pheromone shutdown protein TraB/PrgY	0	0	0	0.00249	0	0	0	0	0.000219	0	0	7.88775E-05
Clustering-based subsystems	Phosphate metabolism	PhoR-PhoB two-component regulatory system	Phosphate regulon metal ion transporter containing CBS domains	0	0	0.00511	0	0.000657	0	0	0	0	0	0	0.00031551
Carbohydrates	Central carbohydrate metabolism	Dihydroxyacetone kinases	Phosphoenolpyruvate-dihydroxyacetone phosphotransferase (EC 2.7.1.121), subunit DhaM	0	0	0	0	0	0.001948	0	0	0.000657	0	0	0.000354949
Cell Wall and Capsule	Gram-Negative cell wall components	Lipid A modifications	Phosphoethanolamine transferase specific for the outer Kdo residue of lipopolysaccharide	0	0	0	0	0	0	0	0	0.000219	0.002203	0	0.000118316
Membrane Transport	NULL	Phosphoglycerate transport system	Phosphoglycerate transport system sensor protein PgtB (EC 2.7.3.-)	0	0	0	0	0	0	0.000722	0	0.000219	0	0	0.000276071
Membrane Transport	NULL	Phosphoglycerate transport system	Phosphoglycerate transporter protein PgtP	0	0	0	0	0	0	0.000722	0.000498	0	0	0	0.00031551
Carbohydrates	Central carbohydrate metabolism	Glycolate, glyoxylate interconversions	Phosphoglycolate phosphatase, archaeal type (EC 3.1.3.18)	0	0.002262	0	0	0	0	0	0	0.000438	0	0	7.88775E-05
Amino Acids and Derivatives	NULL	Phosphonoalanine utilization	Phosphonopyruvate hydrolase (EC 3.11.1.3)	0	0	0	0	0	0	0.000722	0	0.000219	0	0	7.88775E-05
Miscellaneous	Plant-Prokaryote DOE project	At1g54520	photosystem I assembly related protein Ycf4	0	0	0	0	0	0	0	0	0.001752	0	0.000233	3.94388E-05
Photosynthesis	Electron transport and photophosphorylation	Photosystem I	photosystem I subunit III precursor, plastocyanin (cyt c553) docking protein (PsaF)	0	0	0	0	0	0.000487	0	0	0.000657	0	0	7.88775E-05
Photosynthesis	Electron transport and photophosphorylation	Photosystem II	Photosystem II 12 kDa extrinsic protein (PsbU)	0	0	0	0	0	0.000974	0	0	0.001095	0	0.000233	0
Photosynthesis	Electron transport and photophosphorylation	Photosystem II	Photosystem II 13 kDa protein Psb28 (PsbW)	0	0	0	0	0	0	0	0.000498	0.001971	0	0.000116	0
Photosynthesis	Light-harvesting complexes	Phycobilisome	Phycobilisome core-membrane linker polypeptide	0	0	0	0	0	0	0.002166	0.000997	0.00416	0	0	0
Photosynthesis	Light-harvesting complexes	Phycobilisome	Phycobilisome rod linker polypeptide, phycocyanin-associated	0	0	0	0	0	0	0	0.001993	0.003284	0	0.000116	0
Photosynthesis	Light-harvesting complexes	Phycobilisome	Phycobilisome small core linker polypeptide	0	0	0	0	0	0.000974	0.000722	0	0.000219	0	0	0
Photosynthesis	Light-harvesting complexes	Phycobilisome	Phycocyanin alpha chain	0	0	0	0	0	0.000487	0	0	0.001533	0	0.000466	0
Cofactors, Vitamins, Prosthetic Groups, Pigments	Tetrapyrroles	Bilin Biosynthesis	Phycocyanobilin lyase alpha subunit	0	0	0	0	0	0.000487	0	0	0.000876	0	0	3.94388E-05
Cofactors, Vitamins, Prosthetic Groups, Pigments	Tetrapyrroles	Bilin Biosynthesis	Phycocyanobilin lyase beta subunit	0	0	0	0	0	0.001948	0	0	0.000876	0	0	0.000118316
Cofactors, Vitamins, Prosthetic Groups, Pigments	Tetrapyrroles	Bilin Biosynthesis	Phycocyanobilin:ferredoxin oxidoreductase PcyA (EC 1.3.7.5)	0	0	0	0	0	0.000974	0	0	0.001095	0	0.000116	0
Nitrogen Metabolism	NULL	Nitrilase	Plant-induced nitrilase (EC 3.5.5.1), hydrolyses beta-cyano-L-alanine	0	0	0	0	0	0	0.000722	0	0	0	0.000699	0.000670459
Phages, Prophages, Transposable elements, Plasmids	Plasmid related functions	F plasmid replication	Plasmid partition protein A	0	0	0	0	0.000657	0	0	0	0	0	0.000116	0.000907091

Stress Response	Oxidative stress	Redox-dependent regulation of nucleus processes	Poly [ADP-ribose] polymerase-1 (EC 2.4.2.30)	0	0	0	0	0.000657	0	0.000722	0	0	0	0	0.000118316
Fatty Acids, Lipids, and Isoprenoids	NULL	Polyhydroxybutyrate metabolism	Polyhydroxyalkanoate granule-associated protein PhaF	0	0	0	0	0	0	0.001444	0	0.001533	0	0	0.000118316
Cell Wall and Capsule	Capsular and extracellular polysacchrides	Extracellular Polysaccharide Biosynthesis of Streptococci	Polysaccharide biosynthesis protein CpsF	0	0.002262	0	0	0	0	0	0	0.000219	0	0	0.000118316
Cell Wall and Capsule	Capsular and extracellular polysacchrides	Extracellular Polysaccharide Biosynthesis of Streptococci	Polysaccharide biosynthesis protein CpsM(V)	0	0	0	0	0	0	0	0	0.000219	0.002203	0	7.88775E-05
Cell Wall and Capsule	Capsular and extracellular polysacchrides	Vibrio Polysaccharide (VPS) Biosynthesis	polysaccharide export protein, putative	0.004526	0	0	0.00249	0	0	0	0	0	0	0	0.000118316
Respiration	Electron accepting reactions	Anaerobic respiratory reductases	polysulfide reductase, subunit B	0.000696	0	0	0	0	0	0	0	0.000219	0	0	0.000788775
Respiration	Electron accepting reactions	Anaerobic respiratory reductases	Polysulfide reductase, subunit B, putative	0	0	0	0	0.001313	0.000487	0	0	0	0	0	0.000907091
Carbohydrates	Central carbohydrate metabolism	Pyruvate metabolism I: anaplerotic reactions, PEP	Positive regulator of Tartrate dehydrogenase/decarboxylase/D-malic enzyme	0	0	0	0	0	0	0	0	0.001971	0	0.000116	0.000157755
Miscellaneous	Plant-Prokaryote DOE project	Synechocystis experimental	Possible alpha/beta hydrolase superfamily, sll0553 homolog	0	0	0	0	0.000657	0.000487	0	0	0.001314	0	0	0
Potassium metabolism	NULL	Potassium homeostasis	Possible potassium-efflux system protein	0	0	0	0	0.000657	0	0	0	0.001095	0	0	0.000473265
Regulation and Cell signaling	NULL	Oxygen and light sensor PpaA-PpsR	PpaA, heme-binding SCHIC domain sensory protein, regulator for photosystem formation	0	0	0	0	0	0	0.000722	0.000997	0.000438	0	0	0
Amino Acids and Derivatives	Proline and 4-hydroxyproline	Proline, 4-hydroxyproline uptake and utilization	Predicted 4-hydroxyproline dipeptidase	0.000696	0	0	0	0	0	0	0	0.000657	0	0	7.88775E-05
Cofactors, Vitamins, Prosthetic Groups, Pigments	Tetrapyrroles	Coenzyme B12 biosynthesis	Predicted alpha-ribazole-5-phosphate synthase CblS for cobalamin biosynthesis	0	0	0	0	0	0.000974	0	0	0.000219	0	0	7.88775E-05
Carbohydrates	Monosaccharides	D-Galacturonate and D-Glucuronate Utilization	Predicted D-glucuronide-specific TRAP transporter, large transmembrane component	0	0	0	0	0.000657	0	0	0	0.001095	0	0	0.000354949
Carbohydrates	Monosaccharides	D-Galacturonate and D-Glucuronate Utilization	Predicted D-glucuronide-specific TRAP transporter, substrate-binding component	0	0	0	0	0	0	0	0.000498	0.000219	0	0	0.000118316
Carbohydrates	Sugar alcohols	Erythritol utilization	Predicted erythritol ABC transporter 2, ATP-binding component	0	0	0	0	0	0.000487	0	0	0.002189	0	0	0.000354949
Carbohydrates	Di- and oligosaccharides	Lactose and Galactose Uptake and Utilization	Predicted galactoside ABC transporter, ATP-binding protein 1	0.000348	0	0	0	0	0	0	0	0.000876	0	0	0.000473265
Carbohydrates	NULL	Sugar utilization in Thermotogales	Predicted glycosylase, COG2152	0	0	0	0	0.000657	0	0	0	0.000438	0	0	0.000473265
Miscellaneous	Plant-Prokaryote DOE project	COG3533	Predicted L-arabinose ABC transport system, permease protein 2	0	0	0	0	0	0	0.002166	0	0.000438	0	0	0.000118316
RNA Metabolism	RNA processing and modification	tRNA modification Archaea	Predicted methyltransferase, DUF43 family	0	0	0	0.00249	0.000657	0	0	0	0	0	0	0.000157755
RNA Metabolism	RNA processing and modification	rRNA modification Archaea	Predicted N6-adenine-specific RNA methylase containing THUMP domain	0	0.002262	0	0	0	0.000487	0	0	0	0	0	0.000394388
Phages, Prophages, Transposable elements, Plasmids	Transposable elements	CBSS-203122.12.peg.188	Predicted nucleotidyltransferases	0	0	0	0	0.002626	0	0	0	0.000657	0	0	0.001380356
Membrane Transport	Protein secretion system, Type II	Predicted secretion system W clustering with cell division proteins	Predicted secretion system W protein GspG-like 2	0	0.002262	0	0	0	0	0.000722	0	0	0	0	0.000670459
Cofactors, Vitamins, Prosthetic Groups, Pigments	NULL	Thiamin biosynthesis	Predicted thiamin transporter PnuT	0	0	0.007664	0	0	0	0	0	0.000219	0	0	0.000512704
RNA Metabolism	Transcription	Rrf2 family transcriptional regulators	Predicted transcriptional regulator of sulfate adenylyltransferase, Rrf2 family	0	0	0	0	0.000657	0	0	0.000498	0	0	0	0.000276071
Respiration	Electron donating reactions	Na(+)-translocating NADH-quinone oxidoreductase and rnf-like group of electron transport complexes	Probable exported or periplasmic protein in ApbE locus	0	0	0	0	0	0	0	0	0.000657	0	0.000116	0.000433826
Stress Response	Oxidative stress	Glutathione: Non-redox reactions	Probable glutathione S-transferase (EC 2.5.1.18), YfcF homolog	0	0	0	0.00498	0	0.000487	0	0	0	0	0	0.000236633
Stress Response	Oxidative stress	Glutathione: Non-redox reactions	Probable glutathione S-transferase (EC 2.5.1.18), YfcG homolog	0	0	0	0	0	0	0	0.000498	0.000438	0	0	3.94388E-05
Cell Wall and Capsule	Cell wall of Mycobacteria	mycolic acid synthesis	PROBABLE MULTIFUNCTIONAL MYCOCEROSIC ACID SYNTHASE MEMBRANE-ASSOCIATED MAS	0	0	0	0	0	0.000487	0	0	0.000438	0	0	7.88775E-05
Regulation and Cell signaling	Programmed Cell Death and Toxin-antitoxin Systems	Phd-Doc, YdcE-YdcD toxin-antitoxin (programmed cell death) systems	Programmed cell death toxin MazF like	0	0	0	0	0	0.000487	0	0	0.001095	0	0	0.000354949
Carbohydrates	Sugar alcohols	Propanediol utilization	Propanediol dehydratase reactivation factor large subunit	0	0	0	0.00249	0	0	0	0.001495	0	0	0	0.000354949
Carbohydrates	Sugar alcohols	Propanediol utilization	Propanediol dehydratase small subunit (EC 4.2.1.28)	0	0	0	0	0	0.000487	0	0.000498	0	0	0	0.000118316
Amino Acids and Derivatives	Proline and 4-hydroxyproline	Proline, 4-hydroxyproline uptake and utilization	ProQ: influences osmotic activation of compatible solute ProP	0	0	0	0	0	0	0	0	0.002408	0	0.000931	0.000473265
Protein Metabolism	Protein degradation	Proteasome eukaryotic	proteasome regulatory subunit Rpt2	0	0	0	0	0	0	0.000722	0	0.000219	0	0	0.000118316
Protein Metabolism	Protein degradation	Proteasome eukaryotic	proteasome regulatory subunit Rpt3	0	0	0	0	0	0.000487	0	0	0.000219	0	0	0.000276071
Protein Metabolism	Protein degradation	Proteasome eukaryotic	proteasome regulatory subunit Rpt5	0	0.002262	0	0	0	0	0	0.000997	0	0	0	3.94388E-05
Protein Metabolism	Protein degradation	Proteasome eukaryotic	proteasome regulatory subunit Rpt6	0	0	0	0	0	0	0	0	0.000438	0	0.000233	0.000118316
Protein Metabolism	Protein degradation	Proteasome eukaryotic	proteasome subunit alpha4 (EC 3.4.25.1)	0	0	0	0	0	0	0	0.000498	0	0	0.000233	3.94388E-05
Protein Metabolism	Protein degradation	Proteasome eukaryotic	proteasome subunit alpha5 (EC 3.4.25.1)	0	0	0	0	0	0	0.000722	0	0	0	0.000116	0.000118316
Cofactors, Vitamins, Prosthetic Groups, Pigments	Tetrapyrroles	Chlorophyll Biosynthesis	Protein BchJ, involved in reduction of C-8 vinyl of divinyl protochlorophyllide	0	0	0	0	0	0	0	0.000997	0.001314	0	0.000116	0
Clustering-based subsystems	Protein export?	CBSS-393121.3.peg.2760	Protein export cytoplasm protein SecA2 ATPase RNA helicase (TC 3.A.5.1.1)	0.000696	0	0	0	0	0	0	0	0.000438	0	0	7.88775E-05
Clustering-based subsystems	Shiga toxin cluster	CBSS-194948.1.peg.143	Protein NinB	0	0	0	0	0	0.007306	0.005055	0.005482	0	0	0	0
Clustering-based subsystems	NULL	CBSS-316407.3.peg.2816	Protein of unknown function DUF541	0	0	0	0	0	0	0	0.000997	0.000876	0	0	0.000709898
Protein Metabolism	Protein biosynthesis	tRNA aminoacylation, Phe	Protein with domain from phenylalanyl-tRNA synthetase alpha chain	0	0	0	0	0	0.000974	0	0	0.000657	0	0	3.94388E-05
Carbohydrates	Di- and oligosaccharides	Beta-Glucoside Metabolism	PTS system, cellobiose-specific IIC component (EC 2.7.1.69)	0	0.002262	0	0	0	0	0	0	0.000876	0	0	0.000591581
Carbohydrates	Monosaccharides	D-Sorbitol(D-Glucitol) and L-Sorbose Utilization	PTS system, glucitol/sorbitol-specific IIB component and second of two IIC components (EC 2.7.1.69)	0	0	0	0	0	0	0.000722	0.000498	0	0	0	0.000157755
Cell Wall and Capsule	Capsular and extracellular polysacchrides	Sialic Acid Metabolism	PTS system, mannose-specific IIB component (EC 2.7.1.69)	0	0	0	0	0	0.000487	0	0	0.000219	0	0	0.000276071
Cell Wall and Capsule	Capsular and extracellular polysacchrides	Sialic Acid Metabolism	PTS system, mannose-specific IID component (EC 2.7.1.69)	0	0	0	0	0	0	0	0.000498	0.000438	0	0	0.000118316
Cell Wall and Capsule	Capsular and extracellular polysacchrides	Sialic Acid Metabolism	PTS system, N-acetylglucosamine-specific IIA component (EC 2.7.1.69)	0.006267	0	0	0	0	0	0	0	0.000219	0	0	0.000433826
Membrane Transport	ABC transporters	ABC transporter of unknown substrate X	Putative ABC transporter of substrate X, ATP-binding subunit	0	0.004524	0	0	0	0	0.000722	0	0	0	0	7.88775E-05
Membrane Transport	ABC transporters	ABC transporter of unknown substrate X	Putative ABC transporter of substrate X, permease subunit I	0	0	0	0	0	0.001461	0	0	0	0	0.000116	7.88775E-05
Membrane Transport	ABC transporters	ABC transporter of unknown substrate X	Putative ABC transporter of substrate X, permease subunit II	0	0	0	0	0.000657	0.001461	0	0	0.000876	0	0	0
Metabolism of Aromatic Compounds	NULL	Benzoate transport and degradation cluster	Putative Acyl-CoA thiolase	0	0	0	0	0	0.000487	0	0	0.000438	0	0	7.88775E-05
Nucleosides and Nucleotides	Purines	Purine conversions	putative Adenosine kinase (EC 2.7.1.20)	0	0	0	0.00498	0	0	0	0	0	0	0.000349	0.00063102
DNA Metabolism	DNA repair	DNA repair, bacterial UvrD and related helicases	putative ATP-dependent DNA helicase YjcD	0	0	0.00511	0	0	0	0.000722	0	0	0	0	0.000552143

Carbohydrates	Polysaccharides	Unknown carbohydrate utilization containing Fructose-bisphosphate aldolase	Putative carbohydrate PTS system, IIC component (EC 2.7.1.69)	0	0	0	0	0	0	0.000722	0	0.000219	0	0	0.000236633
Clustering-based subsystems	NULL	Putative hemin transporter	Putative hemine transporter ATP-binding subunit	0	0	0	0	0.001313	0	0	0	0.000657	0	0	0.000157755
Cofactors, Vitamins, Prosthetic Groups, Pigments	Folate and pterines	YgfZ	Putative hydrolase in cluster with formaldehyde/S-nitrosomycothiol reductase MscR	0	0	0	0.00249	0	0	0	0	0.000219	0	0	0.000236633
Amino Acids and Derivatives	Arginine; urea cycle, polyamines	Putrescine utilization pathways	Putative oxidoreductase in putrescine utilization cluster	0	0	0	0	0	0	0.000722	0	0.001314	0	0	0.000354949
Miscellaneous	Plant-Prokaryote DOE project	Experimental-yggC	Putative oxidoreductase linked to yggC	0	0	0	0.00249	0	0	0	0	0	0	0.000233	0.002602958
DNA Metabolism	DNA repair	2-phosphoglycolate salvage	Putative phosphatase YfbT	0	0	0	0	0	0	0	0.000498	0.000438	0	0	0.000433826
Cell Wall and Capsule	Capsular and extracellular polysacchrides	Rhamnose containing glycans	putative polysialic acid transport protein	0	0	0	0	0.00197	0	0	0.000498	0	0	0	7.88775E-05
Clustering-based subsystems	Putative asociate of RNA polymerase sigma-54 factor rpoN	CBSS-316057.3.peg.1308	putative RNA polymerase sigma factor	0	0	0	0	0.000657	0	0	0	0.000219	0	0	0.002681835
Miscellaneous	Plant-Prokaryote DOE project	DOE COG2016	Putative snRNP Sm-like protein, Archaeal	0	0	0.002555	0	0	0	0.000722	0	0	0	0	0.000118316
Respiration	Electron donating reactions	Succinate dehydrogenase	putative succinate dehydrogenase [membrane anchor subunit] (succinic dehydrogenase)	0	0	0	0	0	0	0.001444	0	0.001752	0	0	7.88775E-05
Cell Wall and Capsule	Gram-Positive cell wall components	Teichuronic acid biosynthesis	Putative teichuronic acid biosynthesis glycosyl transferase TuaC	0	0	0	0	0	0.000487	0	0.000997	0	0	0	0.000354949
Clustering-based subsystems	Tricarboxylate transporter	CBSS-49338.1.peg.459	Putative tricarboxylic transport TctC	0	0	0	0	0	0	0	0.000498	0.001095	0	0	0.00063102
Iron acquisition and metabolism	Siderophores	Siderophore pyochelin	Pyochelin synthetase PchF, non-ribosomal peptide synthetase module	0	0	0	0	0	0	0	0.000498	0.000219	0	0	7.88775E-05
Iron acquisition and metabolism	Siderophores	Siderophore Pyoverdine	Pyoverdin biosynthesis protein PvdN, putative aminotransferase, class V	0	0	0	0.00249	0	0.000487	0	0	0.000219	0	0	0
RNA Metabolism	Transcription	Transcription factors bacterial	Regulator of nucleoside diphosphate kinase	0	0	0	0	0.000657	0	0	0	0.000438	0	0	0.001183163
Photosynthesis	NULL	Proteorhodopsin	Regulatory protein TspO	0	0	0	0	0	0	0	0.000997	0.000219	0	0	3.94388E-05
Carbohydrates	Monosaccharides	L-rhamnose utilization	Rhamnulose-1-phosphate aldolase (EC 4.1.2.19)	0	0	0	0	0	0	0.000722	0	0.000219	0	0	7.88775E-05
Motility and Chemotaxis	Social motility and nonflagellar swimming in bacteria	Rhamnolipids in Pseudomonas	RhlB, TDP-rhamnosyltransferase 1 (EC 2.4.1.-)	0	0	0	0	0.000657	0	0	0	0	0	0.000116	0.000157755
Miscellaneous	Plant-Prokaryote DOE project	Single-Rhodanese-domain proteins	Rhodanese domain protein UPF0176, Betaproteobacterial subgroup	0	0	0	0	0	0	0.001444	0.000498	0	0	0	3.94388E-05
RNA Metabolism	RNA processing and modification	tRNA modification Bacteria	Rhodanese-related sulfurtransferases	0	0	0	0.00249	0	0	0	0.000997	0.000219	0	0	0
RNA Metabolism	RNA processing and modification	Ribonuclease H	Ribonuclease HI-related protein 2	0	0	0	0	0	0	0	0.000498	0	0	0.000233	0.000197194
Carbohydrates	Monosaccharides	D-ribose utilization	Ribose/xylose/arabinose/galactoside ABC-type transport systems, periplasmic sugar binding protein	0	0	0	0.00498	0	0	0	0.000498	0	0	0	0.000118316
RNA Metabolism	RNA processing and modification	RNA methylation	Ribosomal RNA large subunit methyltransferase F (EC 2.1.1.51)	0	0	0	0	0	0	0	0	0	0.002203	0.020376	0.010530148
RNA Metabolism	RNA processing and modification	tRNA modification Archaea	Ribosome biogenesis protein Nop10	0	0	0	0.00249	0	0	0.000722	0	0	0	0	7.88775E-05
Protein Metabolism	Protein biosynthesis	Ribosome activity modulation	Ribosome modulation factor	0	0	0	0	0	0	0	0.003987	0.002846	0	0	0.001104285
Protein Metabolism	Protein biosynthesis	Translation elongation factor G family	Ribosome protection-type tetracycline resistance related proteins	0	0	0	0	0.000657	0.000487	0	0	0	0	0	0.000394388
Metabolism of Aromatic Compounds	Peripheral pathways for catabolism of aromatic compounds	Benzoate degradation	Ring hydroxylating dioxygenase, alpha subunit (EC 1.14.12.13)	0	0	0	0.00249	0.000657	0	0	0	0	0	0	7.88775E-05
Phages, Prophages, Transposable elements, Plasmids	Phages, Prophages	Phage replication	RNA ligase, phage-associated	0	0	0	0	0.000657	0.000974	0.002166	0	0	0	0	0
RNA Metabolism	Transcription	Transcription initiation, bacterial sigma factors	RNA polymerase principal sigma factor HrdA	0.000348	0	0	0	0.000657	0	0	0	0	0	0	0.00031551
RNA Metabolism	Transcription	Transcription initiation, bacterial sigma factors	RNA polymerase sigma factor SigV	0	0	0	0	0	0.000974	0	0.000498	0	0	0	0.000473265
Cell Division and Cell Cycle	NULL	MukBEF Chromosome Condensation	S-adenosylmethionine-dependent methyltransferase Functionally Coupled to the MukBEF Chromosome Partitioning Mechanism	0	0	0	0	0	0	0	0.001993	0.000219	0	0	0.000828214
Clustering-based subsystems	NULL	ClpS and metallo-protease cluster in Cyanos	SAM radical protein, elongator protein	0.002785	0	0	0	0	0	0	0.000498	0.001314	0	0	0
Amino Acids and Derivatives	NULL	Creatine and Creatinine Degradation	Sarcosine dehydrogenase, mitochondrial precursor (EC 1.5.99.1)	0.002089	0	0	0	0	0	0	0	0.000219	0	0	0.000157755
Amino Acids and Derivatives	Arginine; urea cycle, polyamines	Cyanophycin Metabolism	Secreted cyanophycinase CphE (EC 3.4.15.6)	0	0	0	0	0	0	0	0.000498	0.009634	0	0	3.94388E-05
Regulation and Cell signaling	Quorum sensing and biofilm formation	Quorum-sensing in Vibrio	Sensor histidine kinase CqsS	0	0	0	0	0	0	0	0.000498	0.000219	0	0	0.00031551
Virulence, Disease and Defense	Bacteriocins, ribosomally synthesized antibacterial peptides	Bacitracin Stress Response	Sensor histidine kinase LiaS	0	0.002262	0	0	0	0	0	0	0.000219	0	0	0.000394388
Motility and Chemotaxis	Social motility and nonflagellar swimming in bacteria	Control of Swarming in Vibrio and Shewanella species	Sensory box/GGDEF family protein ScrC (involved in swarmer cell regulation)	0.000696	0	0	0	0	0	0	0	0.000438	0	0	0.000907091
Regulation and Cell signaling	NULL	Orphan regulatory proteins	Sensory histidine kinase in two-component regulatory system with RstA	0	0	0	0	0.001313	0	0.001444	0	0	0	0	0.000433826
Iron acquisition and metabolism	Siderophores	Siderophore assembly kit	Siderophore biosynthesis L-2,4-diaminobutyrate decarboxylase	0	0	0	0	0	0	0	0.000997	0.000219	0	0	0.000197194
Iron acquisition and metabolism	Siderophores	Siderophore assembly kit	Siderophore synthetase component, ligase	0	0	0	0	0	0	0	0.001495	0	0	0.000233	0.000118316
Membrane Transport	Protein secretion system, Type VIII (Extracellular nucleation/precipitation pathway, ENP)	Fap amyloid fiber secretion system	sigma-54 specific transcriptional regulator, Fis family	0	0	0	0	0.000657	0	0	0	0.000219	0	0	0.000157755
Cell Division and Cell Cycle	NULL	Two cell division clusters relating to chromosome partitioning	Signal recognition particle associated protein	0	0.002262	0	0	0	0	0	0.000997	0	0	0	0.000157755
Membrane Transport	NULL	Citrate Utilization System (CitAB, CitH, and tctABC)	Signal transduction histidine kinase CitA regulating citrate metabolism	0	0	0	0	0	0	0.000722	0	0.002189	0	0	0.002129693
Respiration	Electron donating reactions	Hydrogenases	Signal transduction histidine kinase for Quinone-reactive Ni/Fe-hydrogenase	0	0	0	0	0	0	0	0	0.003941	0	0.000582	3.94388E-05
Metabolism of Aromatic Compounds	Peripheral pathways for catabolism of aromatic compounds	Biphenyl Degradation	Small subunit naph/bph dioxygenase	0	0	0	0	0	0	0.000722	0.000997	0.001095	0	0	0
Virulence, Disease and Defense	Resistance to antibiotics and toxic compounds	Aminoglycoside adenylyltransferases	Spectinomycin 9-O-adenylyltransferase	0	0	0	0.00249	0	0	0.000722	0.000997	0	0	0	0
Amino Acids and Derivatives	Arginine; urea cycle, polyamines	Polyamine Metabolism	Spermidine N1-acetyltransferase (EC 2.3.1.57)	0	0	0	0	0	0	0.000722	0	0	0	0.000116	0.001143724
Dormancy and Sporulation	NULL	Spore germination	Spore germination protein GerN (inosine-dependent germination), Na+/H+ antiporter	0	0	0	0	0	0.000487	0.001444	0	0	0	0	7.88775E-05
Dormancy and Sporulation	NULL	Sporulation gene orphans	Sporulation initiation phosphotransferase (Spo0F)	0	0	0.002555	0	0	0	0	0.000498	0	0	0	0.00063102
Regulation and Cell signaling	NULL	WhiB and WhiB-type regulatory proteins	Sporulation regulatory protein WhiD	0	0	0	0	0	0	0	0	0.000219	0	0.000233	3.94388E-05
Protein Metabolism	Protein biosynthesis	Ribosome SSU eukaryotic and archaeal	SSU ribosomal protein S16e (S9p)	0	0	0	0	0.002626	0	0	0.001495	0	0	0	0.001380356
Protein Metabolism	Protein biosynthesis	Ribosome SSU eukaryotic and archaeal	SSU ribosomal protein S29e (S14p)	0	0	0	0	0	0.000487	0	0	0.000438	0	0	7.88775E-05
Protein Metabolism	Protein biosynthesis	Ribosome SSU chloroplast	SSU ribosomal protein S7p (S5e), chloroplast	0	0	0	0	0	0.000487	0	0	0.001095	0	0	7.88775E-05
Dormancy and Sporulation	NULL	Sporulation gene orphans	Stage V sporulation protein whose disruption leads to the production of immature spores	0	0	0.002555	0	0	0	0	0	0.000876	0	0	7.88775E-05

			(SpoVK)												
Stress Response	NULL	Carbon Starvation	Starvation lipoprotein Slp paralog	0	0	0.002555	0	0	0	0	0	0.001533	0	0	0.000118316
Stress Response	NULL	Carbon Starvation	Starvation sensing protein RspB	0	0	0.00511	0	0.000657	0	0	0	0	0	0	7.88775E-05
Membrane Transport	NULL	ECF class transporters	Substrate-specific component CbrT of predicted cobalamin ECF transporter	0.002785	0	0	0	0	0.000487	0	0	0	0	0	0.000670459
Membrane Transport	NULL	ECF class transporters	Substrate-specific component STY3230 of queuosine-regulated ECF transporter	0	0	0	0	0	0.000487	0	0	0	0	0.000233	0.000354949
Metabolism of Aromatic Compounds	Anaerobic degradation of aromatic compounds	Anaerobic toluene and ethylbenzene degradation	Succinyl-CoA:(R)-benzylsuccinate CoA-transferase subunit BbsF (EC 2.8.3.15)	0	0.002262	0	0	0	0	0.001444	0	0	0	0	0.000512704
Miscellaneous	Plant-Prokaryote DOE project	PROSC	Succinylornithine transaminase (EC 2.6.1.-)	0	0.004524	0	0	0.001313	0	0	0	0	0	0	0.001498673
Carbohydrates	Di- and oligosaccharides	Sucrose utilization	Sucrose repressor, LacI family, Shewanella subfamily	0	0	0	0	0	0	0	0	0.000438	0	0.010246	0.000354949
Clustering-based subsystems	NULL	USS-DB-1	Sugar ABC transporter, periplasmic sugar-binding protein USSDB1B	0	0	0.00511	0	0.000657	0	0	0	0	0	0	0.000394388
Membrane Transport	Protein secretion system, Type I	Type I secretion system for aggregation	T1SS peptidoglycan-associated lipoprotein, Pal family (LapL)	0	0	0	0	0	0.000974	0	0	0.000876	0	0	0.00063102
Membrane Transport	NULL	Citrate Utilization System (CitAB, CitH, and tctABC)	TctA citrate transporter	0	0	0.002555	0	0	0	0	0	0.000438	0	0	3.94388E-05
Carbohydrates	Organic acids	Tricarballylate Utilization	TcuR: regulates tcuABC genes used in utilization of tricarballylate	0	0	0	0	0	0	0	0.000498	0.001314	0	0	0.000354949
Membrane Transport	Protein and nucleoprotein secretion system, Type IV	Vir-like type 4 secretion system	T-DNA border endonuclease VirD2, RP4 TraG-like relaxase	0	0	0	0	0	0	0	0.000498	0.001095	0	0	0.00126204
Fatty Acids, Lipids, and Isoprenoids	Fatty acids	Acyl-CoA thioesterase II	TesB-like acyl-CoA thioesterase 1	0	0	0	0	0	0	0	0.004485	0.005693	0	0	0.000276071
Fatty Acids, Lipids, and Isoprenoids	Fatty acids	Acyl-CoA thioesterase II	TesB-like acyl-CoA thioesterase 2	0	0.002262	0	0.00249	0	0	0	0	0	0	0	3.94388E-05
Respiration	Electron accepting reactions	Tetrathionate respiration	Tetrathionate reductase two-component response regulator	0.002785	0	0	0	0	0	0	0	0.000219	0	0	0.001932499
Protein Metabolism	Protein folding	Periplasmic disulfide interchange	Thiol:disulfide interchange protein DsbG precursor	0.000348	0	0	0	0	0	0	0	0.000438	0	0	7.88775E-05
Protein Metabolism	Protein processing and modification	Peptide methionine sulfoxide reductase	Thiol:disulfide oxidoreductase associated with MetSO reductase	0	0	0	0	0.000657	0	0	0	0	0	0.002212	0.00063102
Respiration	Electron accepting reactions	Anaerobic respiratory reductases	Thiosulfate reductase precursor (EC 1.-.-.-)	0.001044	0	0	0	0	0	0	0.000498	0	0	0	0.001025408
Protein Metabolism	Protein biosynthesis	tRNA aminoacylation, Thr	Threonyl-tRNA synthetase (EC 6.1.1.3), mitochondrial	0	0.002262	0	0	0	0	0	0	0	0	0.000116	3.94388E-05
Phages, Prophages, Transposable elements, Plasmids	Transposable elements	CBSS-203122.12.peg.188	TniA putative transposase	0	0	0	0	0	0	0	0.001993	0.003284	0	0	0.001222601
Metabolism of Aromatic Compounds	Peripheral pathways for catabolism of aromatic compounds	Toluene degradation	Toluate 1,2-dioxygenase electron transfer component	0	0	0	0	0	0	0	0	0.000219	0	0.003959	3.94388E-05
Metabolism of Aromatic Compounds	NULL	Toluene 4-monooxygenase (T4MO)	Toluene-4-monooxygenase, subunit TmoF	0	0.002262	0	0	0	0	0	0	0.000438	0	0	0.000276071
Regulation and Cell signaling	Programmed Cell Death and Toxin-antitoxin Systems	Toxin-Antitoxin MT1	Toxin 1, PIN domain	0	0	0	0	0	0	0.001444	0	0.000438	0	0	0.000118316
RNA Metabolism	Transcription	RNA polymerase II initiation factors	Transcription initiation factor IIH p80 subunit	0	0	0	0	0	0	0	0	0	0.002203	0.000116	0.000157755
Metabolism of Aromatic Compounds	Metabolism of central aromatic intermediates	4-Hydroxyphenylacetic acid catabolic pathway	Transcriptional activator of 4-hydroxyphenylacetate 3-monooxygenase operon, XylS/AraC family	0	0	0	0	0	0	0	0	0.000657	0	0.013157	0.001104285
Membrane Transport	Protein secretion system, Type VIII (Extracellular nucleation/precipitation pathway, ENP)	Curli production	Transcriptional regulator CsgD for 2nd curli operon	0	0	0	0	0	0.001948	0.000722	0	0	0	0	0.000276071
Virulence, Disease and Defense	Resistance to antibiotics and toxic compounds	MexE-MexF-OprN Multidrug Efflux System	Transcriptional regulator MexT	0.001741	0	0	0	0	0	0	0	0.000219	0	0	0.000118316
Cell Wall and Capsule	Capsular and extracellular polysacchrides	Vibrio Polysaccharide (VPS) Biosynthesis	Transcriptional regulator VpsR	0	0	0	0	0	0	0	0.000498	0.000219	0	0	0.00031551
Stress Response	Oxidative stress	Oxidative stress	transcriptional regulator, Fur family	0	0	0	0	0.000657	0.000487	0	0	0	0	0	0.000197194
Clustering-based subsystems	Putrescine/GABA utilization cluster-temporal,to add to SSs	GABA and putrescine metabolism from cluters	Transcriptional regulator, GABA/putrescine utiliation cluster 2	0.020889	0	0	0.00498	0	0	0	0	0	0	0	0.000157755
Stress Response	Osmotic stress	Osmotic stress cluster	Transcriptional regulatory protein YciT	0	0	0	0.00249	0.000657	0	0	0	0	0	0	0.000157755
Amino Acids and Derivatives	Proline and 4-hydroxyproline	Proline, 4-hydroxyproline uptake and utilization	Transcriptional repressor of PutA and PutP	0.000696	0	0	0	0	0	0	0	0.000438	0	0	0.000118316
Protein Metabolism	Protein biosynthesis	Translation elongation factors eukaryotic and archaeal	Translation elongation factor 1 beta subunit	0	0	0	0	0.000657	0	0.000722	0.000498	0	0	0	0
Protein Metabolism	Protein biosynthesis	Translation initiation factors eukaryotic and archaeal	Translation initiation factor 1A	0	0	0	0	0.000657	0	0	0	0.000438	0	0	0.000236633
Membrane Transport	NULL	ECF class transporters	Transmembrane component of energizing module of ECF transporters in Cyanobacteria	0	0	0	0	0.001313	0	0	0	0.000876	0	0.000233	0
Clustering-based subsystems	NULL	CBSS-83332.1.peg.3803	Transposase for insertion sequence element IS1557	0	0	0	0.00249	0	0	0.000722	0	0	0	0	0.000118316
RNA Metabolism	RNA processing and modification	Wyeosine-MimG Biosynthesis	tRNA methylase Trm12p Wyeosine biosynthesis	0	0	0	0	0	0	0	0.000498	0	0.002203	0	0.000512704
Miscellaneous	Plant-Prokaryote DOE project	At1g33290 At1g73170 At3g10420	Tryptophan-rich protein DUF2389, Ssr2843 homolog	0	0	0	0	0	0.000974	0	0.000997	0.000876	0	0	0
Regulation and Cell signaling	NULL	Staphylococcal accessory gene regulator system	Two component system histidine kinase ArlS (EC 2.7.3.-)	0	0	0	0	0	0	0	0.000498	0.000876	0	0	0.000118316
Carbohydrates	Di- and oligosaccharides	Fructooligosaccharides(FOS) and Raffinose Utilization	Two-component sensor kinase YesM (EC 2.7.3.-)	0	0	0	0	0	0	0	0.001495	0	0	0.000116	0.000552143
Membrane Transport	Protein secretion system, Type VII (Chaperone/Usher pathway, CU)	Type 1 pili (mannose-sensitive fimbriae, gamma-fimbriae)	type 1 fimbriae anchoring protein FimD	0	0	0	0	0	0	0	0.000498	0	0	0.000116	3.94388E-05
Membrane Transport	Protein secretion system, Type III	Type III secretion systems	Type III secretion inner membrane protein (YscT,HrcT,SpaR,EscT,EpaR1,homologous to flagellar export components)	0	0.002262	0	0	0	0	0	0	0.000438	0	0	7.88775E-05
Membrane Transport	Protein secretion system, Type III	Type III secretion system	Type III secretion outermembrane pore forming protein (YscC,MxiD,HrcC, InvG)	0	0	0	0.00249	0	0.000487	0	0	0.000438	0	0	0
Clustering-based subsystems	NULL	CBSS-316279.3.peg.746	Uncharacterized membrane protein Ycf36	0	0	0	0	0.000657	0.000974	0	0	0	0	0.000116	0
Respiration	NULL	Methanogenesis strays	Uncharacterized protein MJ0054	0	0	0	0.00498	0.001313	0	0	0	0	0	0	7.88775E-05
Virulence, Disease and Defense	Adhesion	Mediator of hyperadherence YidE in Enterobacteria and its conserved region	Uncharacterized protein YidR	0	0.002262	0.002555	0	0	0	0	0	0	0	0	0.000236633
Miscellaneous	Plant-Prokaryote DOE project	COG2509	uncharacterized secreted protein, YBBR Bacillus subtilis homolog	0	0.002262	0	0	0	0	0	0	0.000219	0	0	0.000118316
Carbohydrates	NULL	Uncharacterized sugar kinase cluster (ygc)	Uncharacterized sugar kinase YgcE (EC 2.7.1.-)	0	0	0	0	0.000657	0.001461	0	0	0	0	0	7.88775E-05
Clustering-based subsystems	Shiga toxin cluster	CBSS-194948.1.peg.143	unknown protein encoded by bacteriophage BP-933W	0	0	0	0	0	0	0	0.000498	0.000219	0	0	7.88775E-05
Amino Acids and Derivatives	Arginine; urea cycle, polyamines	Urea decomposition	Urea carboxylase-related amino acid permease	0	0	0	0.00249	0	0	0.000722	0	0	0	0	3.94388E-05
Cofactors, Vitamins, Prosthetic Groups, Pigments	Tetrapyrroles	Heme and Siroheme Biosynthesis	Uroporphyrinogen-III synthase, divergent, Flavobacterial type (EC 4.2.1.75)	0	0	0	0	0	0	0	0.001495	0.001533	0	0	0.006744027
Virulence, Disease and Defense	Resistance to antibiotics and toxic compounds	Resistance to Vancomycin	Vancomycin B-type resistance protein VanX	0	0	0	0	0	0.000487	0	0	0.000438	0	0	0.000197194
Virulence, Disease and Defense	Resistance to antibiotics and toxic compounds	Resistance to Vancomycin	Vancomycin response regulator VanR	0	0	0	0	0	0	0.000722	0	0.000657	0	0	0.000354949

Metabolism of Aromatic Compounds	Peripheral pathways for catabolism of aromatic compounds	Phenylpropanoid compound degradation	Vanillin dehydrogenase (Hydroxybenzaldehyde dehydrogenase) (EC 1.2.1.28)	0	0	0.002555	0	0.001313	0	0	0	0	0	0	0.000118316
Metabolism of Aromatic Compounds	Peripheral pathways for catabolism of aromatic compounds	Phenylpropanoid compound degradation	vannilate transporter VanK	0	0	0	0	0.000657	0	0	0	0.000657	0	0	0.00031551
Miscellaneous	Plant-Prokaryote DOE project	COG0523	VEGETATIBLE INCOMPATIBILITY PROTEIN HET-E-1	0	0	0	0	0.000657	0	0.001444	0	0	0	0	0.000118316
Miscellaneous	Plant-Prokaryote DOE project	COG0523	Vegetatible incompatibility protein HET-E-1	0	0	0	0	0	0	0	0	0.000219	0	0.000233	0.000118316
Iron acquisition and metabolism	Siderophores	Vibrioferrin synthesis	Vibrioferrin receptor PvuA	0	0	0	0	0	0	0	0.000498	0	0	0.000233	3.94388E-05
Membrane Transport	Protein secretion system, Type II	Widespread colonization island	Von Willebrand factor type A domain protein, associated with Flp pilus assembly	0	0	0	0	0	0	0	0.000498	0.000219	0	0	0.000591581
Regulation and Cell signaling	NULL	WhiB and WhiB-type regulatory proteins	WhiB-family transcriptional regulator	0	0	0	0	0	0	0	0.000498	0.000219	0	0	7.88775E-05
Cell Wall and Capsule	Capsular and extracellular polysacchrides	Xanthan Exopolysaccharide Biosynthesis and Export	Xanthan biosynthesis glycosyltransferase GumD	0	0.002262	0	0	0	0	0	0.000498	0	0	0	0.000118316
Nucleosides and Nucleotides	Purines	Purine Utilization	Xanthine dehydrogenase, iron-sulfur cluster and FAD-binding subunit A (1.17.1.4)	0	0	0	0	0	0.000487	0	0	0.000876	0	0	0.000197194
Carbohydrates	Monosaccharides	Xylose utilization	Xylose activator XylR (AraC family)	0	0	0	0	0	0	0	0	0.000219	0	0.000582	3.94388E-05
Clustering-based subsystems	Nucleotidyl-phosphate metabolic cluster	CBSS-222523.1.peg.1311	YjbH-like, GTP pyrophosphokinase domain	0	0	0	0	0.000657	0	0.000722	0	0	0	0	3.94388E-05
Membrane Transport	NULL	Transport of Zinc	Zinc-regulated zinc transporting ATPase ZntA	0	0	0	0	0.000657	0	0	0	0.000438	0	0	0.000236633
Fatty Acids, Lipids, and Isoprenoids	Fatty acids	Fatty Acid Biosynthesis FASI	[Acyl-carrier-protein] acetyl transferase of FASI (EC 2.3.1.38)	0	0	0	0	0	0	0	0	0.000219	0	0	3.94388E-05
Respiration	Electron donating reactions	Hydrogenases	[Fe] hydrogenase gamma (EC 1.12.7.2)	0	0	0	0	0	0.000487	0	0.000997	0	0	0	0
Metabolism of Aromatic Compounds	Metabolism of central aromatic intermediates	Central meta-cleavage pathway of aromatic compound degradation	1,2-dihydroxynaphthalene dioxygenase	0	0	0	0.00249	0	0	0	0	0.000219	0	0	0
Secondary Metabolism	Plant Octadecanoids	Octadecanoids	12-oxophytodienoate reductase (OPR3)(DDE1)	0	0	0	0	0	0	0	0	0.000876	0	0	3.94388E-05
Carbohydrates	Fermentation	Acetoin, butanediol metabolism	2,3-butanediol dehydrogenase, S-alcohol forming, (R)-acetoin-specific (EC 1.1.1.4)	0	0	0	0	0	0	0	0	0.000438	0	0	0.000354949
RNA Metabolism	RNA processing and modification	RNA methylation	23S rRNA (Uracil-5-) -methyltransferase rumB (EC 2.1.1.-)	0	0	0	0	0.001313	0	0	0	0	0	0	0.000433826
Amino Acids and Derivatives	Aromatic amino acids and derivatives	Chorismate: Intermediate for synthesis of Tryptophan, PAPA antibiotics, PABA, 3-hydroxyanthranilate and more.	2-Amino-2-deoxy-isochorismate synthase (EC 4.1.3.-)	0	0	0	0	0	0	0.001444	0	0	0	0	3.94388E-05
Metabolism of Aromatic Compounds	Peripheral pathways for catabolism of aromatic compounds	Chlorobenzoate degradation	2-chlorobenzoate 1,2-dioxygenase reductase component	0	0	0	0	0	0	0	0	0.000438	0	0	0.000157755
Carbohydrates	Monosaccharides	D-Galacturonate and D-Glucuronate Utilization	2-dehydro-3-deoxy-D-gluconate 5-dehydrogenase (EC 1.1.1.127)	0	0	0	0	0.001313	0	0	0	0	0	0	0.000157755
Secondary Metabolism	Plant Alkaloids	Alkaloid biosynthesis from L-lysine	2-hydroxyphytanoyl-CoA lyase-related protein [EC:4.1.-.-]	0	0	0	0	0.000657	0	0	0.000498	0	0	0	0
Carbohydrates	Monosaccharides	2-Ketogluconate Utilization	2-ketogluconate utilization repressor PtxS	0	0	0	0	0	0	0	0	0.000219	0	0	7.88775E-05
Cell Wall and Capsule	Gram-Negative cell wall components	Vibrio Core Oligosaccharide Biosynthesis	3-deoxy-D-manno-octulosonic acid kinase (EC 2.7.1.-)	0	0	0	0	0	0	0	0	0.001095	0	0	0.001459234
Carbohydrates	Monosaccharides	L-ascorbate utilization (and related gene clusters)	3-keto-L-gulonate 6-phosphate decarboxylase	0	0	0	0	0	0.000487	0	0	0	0	0	3.94388E-05
Fatty Acids, Lipids, and Isoprenoids	Fatty acids	Fatty Acid Biosynthesis FASI	3-oxoacyl-coenzyme A reductase of elongase (EC 1.1.1.62)	0	0	0	0	0	0	0.000722	0	0	0	0	0.00031551
Fatty Acids, Lipids, and Isoprenoids	Fatty acids	Polyunsaturated Fatty Acids synthesis	4'-phosphopantetheinyl transferase (EC 2.7.8.-), inferred for PFA pathway	0	0.002262	0	0	0	0	0	0	0	0	0	3.94388E-05
Cofactors, Vitamins, Prosthetic Groups, Pigments	Pyridoxine	Pyridoxin(Vitamin B6) Degradation Pathway	4-pyridoxic acid dehydrogenase	0	0	0	0	0	0.000487	0	0	0	0	0	3.94388E-05
Carbohydrates	Di- and oligosaccharides	Lactose and Galactose Uptake and Utilization	6-phospho-beta-galactosidase (EC 3.2.1.85)	0	0	0	0	0	0	0	0.000498	0	0	0.000466	0
Carbohydrates	Di- and oligosaccharides	Beta-Glucoside Metabolism	6-phospho-beta-glucosidase ascB (EC 3.2.1.86)	0	0	0	0	0	0	0	0	0.000219	0	0	7.88775E-05
Membrane Transport	Protein secretion system, Type I	Type I protein secretion systems	ABC exporter for hemopore HasA, membrane fusion protein (MFP) family component HasE	0	0	0	0	0	0	0.000722	0	0.000219	0	0	0
Dormancy and Sporulation	NULL	Bacillus Sporulation Killing Factor A Biosynthetic Cluster	ABC transporter ATP-binding protein SkfF	0	0	0	0.00249	0	0	0	0	0	0	0	7.88775E-05
Iron acquisition and metabolism	Siderophores	Siderophore Pyoverdine	ABC transporter in pyoverdin gene cluster, permease component	0	0	0	0	0	0	0	0	0	0	0.000233	3.94388E-05
Clustering-based subsystems	NULL	Listeria bile tolerance locus BltB	ABC transporter, ATP-binding protein in BtlB locus	0	0	0.002555	0	0	0	0	0	0	0	0	0.000552143
Amino Acids and Derivatives	Arginine; urea cycle, polyamines	Arginine Biosynthesis extended	Acetylornithine aminotransferase, mitochondrial precursor (EC 2.6.1.11) (ACOAT)	0	0	0	0	0	0	0	0.000498	0	0	0	3.94388E-05
Regulation and Cell signaling	Quorum sensing and biofilm formation	Symbiotic colonization and sigma-dependent biofilm formation gene cluster	Acetyltransferase SypM	0	0	0	0	0	0	0	0	0	0	0.002329	0.000157755
Iron acquisition and metabolism	Siderophores	Siderophore Achromobactin	Achromobactin biosynthesis protein AcsA	0	0	0	0	0	0	0	0	0.003284	0	0.000116	0
Iron acquisition and metabolism	Siderophores	Siderophore Achromobactin	Achromobactin biosynthesis protein AcsB, HpcH/HpaI aldolase family	0	0	0	0	0.000657	0	0	0	0.000219	0	0	0
Carbohydrates	Central carbohydrate metabolism	TCA Cycle	Aconitate hydratase large subunit (EC 4.2.1.3)	0.002437	0	0	0	0	0	0	0	0	0	0	3.94388E-05
Fatty Acids, Lipids, and Isoprenoids	Fatty acids	Phospholipid and Fatty acid biosynthesis related cluster	Acyl carrier protein (ACP1)	0	0	0	0	0	0	0.000722	0	0	0	0	0.000157755
Fatty Acids, Lipids, and Isoprenoids	Fatty acids	Phospholipid and Fatty acid biosynthesis related cluster	Acyl carrier protein (ACP2)	0	0	0	0	0	0	0	0.000498	0	0	0	3.94388E-05
Cofactors, Vitamins, Prosthetic Groups, Pigments	NULL	Thiamin biosynthesis	Additional substrate-binding component of thiamin-regulated ECF transporter for HydroxyMethylPyrimidine	0	0	0	0	0.000657	0	0	0	0	0	0	0.000157755
Nucleosides and Nucleotides	Detoxification	Nudix proteins (nucleoside triphosphate hydrolases)	ADP-ribose pyrophosphatase, mitochondrial precursor (EC 3.6.1.13)	0	0	0	0	0	0	0.000722	0	0	0	0	7.88775E-05
Protein Metabolism	Protein biosynthesis	tRNA aminoacylation, Ala	Alanyl-tRNA synthetase (EC 6.1.1.7), mitochondrial	0	0	0	0	0	0	0	0	0.000438	0	0	0.000197194
Secondary Metabolism	Biologically active compounds in metazoan cell defence and differentiation	Steroid sulfates	Alcohol sulfotransferase (EC 2.8.2.2)	0	0	0	0	0	0	0	0.000498	0	0	0	0.000118316
Clustering-based subsystems	Methylamine utilization	CBSS-265072.7.peg.546	Aldehyde dehydrogenase in Methylamine utilization cluster	0	0	0	0	0	0	0	0	0.000876	0	0	0.000394388
Stress Response	Dessication stress	O-antigen capsule important for environmental persistence	Aldolase YihT	0	0	0	0	0.000657	0	0	0	0	0	0	3.94388E-05
Carbohydrates	NULL	Unknown carbohydrate utilization (cluster Ydj)	Aldose 1-epimerase family protein YeaD	0	0	0	0	0	0	0	0	0	0	0.000116	0.000433826
Cell Wall and Capsule	Capsular and extracellular polysacchrides	Alginate metabolism	Alginate biosynthesis protein AlgX	0	0	0	0	0	0.000487	0	0	0	0	0	0.000118316
Cell Wall and Capsule	Capsular and extracellular polysacchrides	Alginate metabolism	Alginate biosynthesis transcriptional activator	0	0	0	0	0	0.000487	0.000722	0	0	0	0	0
Photosynthesis	Light-harvesting complexes	Phycobilisome	Allophycocyanin beta chain	0	0	0	0	0	0.000487	0	0	0.001314	0	0	0
Carbohydrates	NULL	Sugar utilization in Thermotogales	Alpha-1,4-digalacturonate ABC transporter, permease protein 2	0	0	0	0	0.00197	0	0	0	0	0	0	0.000157755
Fatty Acids, Lipids, and Isoprenoids	Phospholipids	Glycerolipid and Glycerophospholipid Metabolism in Bacteria	Alpha-glycerophosphate oxidase (EC 1.1.3.21)	0	0	0	0	0	0	0	0	0.000219	0	0	3.94388E-05
Cell Wall and Capsule	Capsular and extracellular polysacchrides	Rhamnose containing glycans	Alpha-L-Rha alpha-1,2-L-rhamnosyltransferase/alpha-L-Rha alpha-1,3-L-rhamnosyltransferase (EC 2.4.1.-)	0	0	0	0	0	0	0	0	0.000219	0	0	3.94388E-05

Miscellaneous	Plant-Prokaryote DOE project	DOE COG3533	Amino acid permease in 4-hydroxyproline catabolic gene cluster	0	0.004524	0	0	0	0	0	0	0	0	0	7.88775E-05
Virulence, Disease and Defense	Resistance to antibiotics and toxic compounds	Aminoglycoside adenylyltransferases	Aminoglycoside N6'-acetyltransferase (EC 2.3.1.82)	0	0	0	0	0	0	0	0	0.000438	0	0	0.000433826
Carbohydrates	CO2 fixation	Photorespiration (oxidative C2 cycle)	Aminomethyl transferase, THF-dependent (glycine cleavage system T protein) (EC 2.1.2.10), mitochondrial	0	0	0	0	0	0	0	0	0.000219	0	0	3.94388E-05
Respiration	Electron accepting reactions	Anaerobic respiratory reductases	Anaerobic sulfite reductase subunit A	0.000696	0	0	0	0	0	0	0	0	0	0	0.000394388
Respiration	Electron accepting reactions	Anaerobic respiratory reductases	Anaerobic sulfite reductase subunit C (EC 1.8.1.-)	0	0	0	0	0	0	0.000722	0	0.000219	0	0	0
Amino Acids and Derivatives	Aromatic amino acids and derivatives	Aromatic amino acid degradation	Anthranilate dioxygenase large subunit	0	0	0	0	0	0	0	0	0	0	0.022705	0.000512704
Amino Acids and Derivatives	Aromatic amino acids and derivatives	Aromatic amino acid degradation	Anthranilate dioxygenase small subunit	0	0	0	0	0	0	0	0	0	0.002203	0	3.94388E-05
Amino Acids and Derivatives	Arginine; urea cycle, polyamines	Arginine and Ornithine Degradation	Arginine ABC transporter, ATP-binding protein ArtP	0	0	0	0	0	0	0	0	0.000438	0	0	3.94388E-05
Amino Acids and Derivatives	Glutamine, glutamate, aspartate, asparagine; ammonia assimilation	Glutamine, Glutamate, Aspartate and Asparagine Biosynthesis	Asparagine synthetase [glutamine-hydrolyzing] (EC 6.3.5.4) AsnB	0	0.002262	0	0	0	0	0	0	0	0	0	0.000512704
Cofactors, Vitamins, Prosthetic Groups, Pigments	NULL	Thiamin biosynthesis	ATPase component YkoD of energizing module of thiamin-regulated ECF transporter for HydroxyMethylPyrimidine	0	0.002262	0	0	0	0.000487	0	0	0	0	0	0
Phages, Prophages, Transposable elements, Plasmids	Transposable elements	Tn552	ATP-binding protein p271	0	0	0	0	0	0	0	0	0.001533	0	0	3.94388E-05
Phosphorus Metabolism	NULL	Alkylphosphonate utilization	ATP-binding protein PhnN	0	0	0	0	0.000657	0	0	0	0.000438	0	0	0
DNA Metabolism	DNA repair	DNA repair, bacterial UvrD and related helicases	ATP-dependent DNA helicase UvrD/PcrA/Rep, cyanobacterial paralog	0	0	0	0	0.000657	0	0	0	0.000438	0	0	0
Protein Metabolism	Protein degradation	Proteasome bacterial	ATP-dependent protease LonB-like Type I	0	0	0	0	0	0	0.000722	0	0	0	0	3.94388E-05
RNA Metabolism	RNA processing and modification	ATP-dependent RNA helicases, bacterial	ATP-dependent RNA helicase VCA0990	0.002089	0	0	0	0	0	0	0	0	0	0	3.94388E-05
Regulation and Cell signaling	Quorum sensing and biofilm formation	Autoinducer 2 (AI-2) transport and processing (lsrACDBFGE operon)	Autoinducer 2 (AI-2) ABC transport system, membrane channel protein LsrC	0	0	0	0	0	0.000487	0	0	0	0	0	7.88775E-05
Regulation and Cell signaling	Quorum sensing and biofilm formation	Autoinducer 2 (AI-2) transport and processing (lsrACDBFGE operon)	Autoinducer 2-binding periplasmic protein LuxP precursor	0	0	0	0	0	0	0	0	0	0	0.000233	0.000157755
Virulence, Disease and Defense	Bacteriocins, ribosomally synthesized antibacterial peptides	Bacitracin Stress Response	Bacitracin export ATP-binding protein BceA	0	0	0	0	0	0	0	0	0.000219	0	0	0.000433826
Virulence, Disease and Defense	Bacteriocins, ribosomally synthesized antibacterial peptides	Bacitracin Stress Response	Bacitracin export permease protein BceB	0	0	0	0	0	0	0	0	0.000219	0	0	0.000118316
Metabolism of Aromatic Compounds	NULL	Benzoate transport and degradation cluster	Benzoate transport, inner membrane transport component	0	0.002262	0	0	0	0	0	0	0.000219	0	0	0
Metabolism of Aromatic Compounds	Anaerobic degradation of aromatic compounds	Anaerobic toluene and ethylbenzene degradation	Benzoylsuccinyl-CoA thiolase beta subunit (EC:2.3.1.-)	0	0	0	0	0	0.000487	0	0	0	0	0	0.000591581
Metabolism of Aromatic Compounds	Anaerobic degradation of aromatic compounds	Anaerobic toluene and ethylbenzene degradation	Benzylsuccinate synthase alpha subunit (EC 4.1.99.11)	0	0	0	0	0	0.000487	0	0	0	0	0	0.001380356
Carbohydrates	Di- and oligosaccharides	Beta-Glucoside Metabolism	Beta-glucoside ABC transport system, ATP-binding protein 1	0	0	0	0	0.000657	0	0	0	0	0	0	0.000276071
Carbohydrates	Di- and oligosaccharides	Beta-Glucoside Metabolism	Beta-glucoside ABC transport system, permease protein 1	0	0	0.002555	0	0	0	0	0	0	0	0	0.000394388
Carbohydrates	NULL	Sugar utilization in Thermotogales	Beta-glucoside ABC transport system, permease protein 2	0.002437	0	0	0	0	0	0	0	0	0	0	0.000276071
Protein Metabolism	Selenoproteins	Glycine reductase, sarcosine reductase and betaine reductase	Betaine reductase component B beta subunit (EC 1.21.4.4)	0.000696	0	0	0	0	0	0	0	0	0	0	0.00063102
Metabolism of Aromatic Compounds	NULL	Benzoate transport and degradation cluster	Beta-ketoadipyl CoA thiolase (EC 2.3.1.9)	0	0	0	0	0	0	0	0	0.000657	0	0	7.88775E-05
Virulence, Disease and Defense	Resistance to antibiotics and toxic compounds	BlaR1 Family Regulatory Sensor-transducer Disambiguation	Beta-lactamase class A	0	0	0	0	0	0	0	0.000498	0.000657	0	0	0
Nucleosides and Nucleotides	Pyrimidines	Pyrimidine utilization	Beta-ureidopropionase, eukaryotic type (EC 3.5.1.6)	0	0	0	0	0	0	0	0.000997	0	0	0	0.000157755
Carbohydrates	CO2 fixation	CO2 uptake, carboxysome	Bicarbonate transport system permease protein	0	0	0	0	0	0	0	0	0.000876	0	0.000233	0
Carbohydrates	CO2 fixation	CO2 uptake, carboxysome	Bicarbonate utilisation transcription factor, related to RuBisCO operon transcriptional regulator	0	0	0	0	0	0	0	0.000498	0	0	0	0.000236633
Cofactors, Vitamins, Prosthetic Groups, Pigments	Tetrapyrroles	Bilin Biosynthesis	Biliverdin reductase (EC 1.3.1.24)	0	0	0	0	0	0	0	0	0.000876	0	0	3.94388E-05
Regulation and Cell signaling	Quorum sensing and biofilm formation	Biofilm Adhesin Biosynthesis	Biofilm PGA synthesis deacetylase PgaB (EC 3.-)	0	0	0	0	0	0	0	0	0.000219	0	0	0.001459234
Membrane Transport	NULL	Ton and Tol transport systems	Biopolymer transport ExbD protein	0	0	0	0	0	0	0	0.000997	0	0	0	7.88775E-05
Miscellaneous	Plant-Prokaryote DOE project	Biotin biosynthesis Experimental	Biotin biosynthesis protein BioI of cytochrome P450 family, pimeloyl-ACP producing	0	0	0	0	0	0.000487	0.000722	0	0	0	0	0
Carbohydrates	Organic acids	Propionyl-CoA to Succinyl-CoA Module	Biotin carboxylase of Propionyl-CoA carboxylase (EC 6.3.4.14)	0	0	0	0	0	0.000487	0	0.000498	0	0	0	0
Virulence, Disease and Defense	Resistance to antibiotics and toxic compounds	Cadmium resistance	Cadmium resistance protein	0	0	0	0	0	0	0	0	0.000219	0	0	7.88775E-05
Cell Wall and Capsule	Capsular and extracellular polysacchrides	Capsular heptose biosynthesis	Capsular polysaccharide biosynthesis fatty acid synthase WcbR	0	0	0	0	0	0	0	0.000498	0	0	0	0.000157755
Cell Wall and Capsule	Capsular and extracellular polysacchrides	Serotype determining Capsular polysaccharide biosynthesis in Staphylococcus	Capsular polysaccharide synthesis enzyme Cap5H	0	0	0	0	0	0	0	0	0.000219	0	0	0.000394388
Cell Wall and Capsule	Capsular and extracellular polysacchrides	Serotype determining Capsular polysaccharide biosynthesis in Staphylococcus	Capsular polysaccharide synthesis enzyme Cap8J	0	0	0	0	0	0	0	0.000498	0	0	0	3.94388E-05
Cell Wall and Capsule	Capsular and extracellular polysacchrides	Serotype determining Capsular polysaccharide biosynthesis in Staphylococcus	Capsular polysaccharide synthesis enzyme Cap8P	0	0	0	0	0	0	0	0	0.000438	0	0	3.94388E-05
Cell Wall and Capsule	Capsular and extracellular polysacchrides	Vibrio Polysaccharide (VPS) Biosynthesis	Capsular polysaccharide synthesis enzyme CpsB	0	0	0	0	0	0.000487	0	0	0	0	0	3.94388E-05
Carbohydrates	CO2 fixation	CO2 uptake, carboxysome	Carbon dioxide concentrating mechanism protein CcmO	0	0	0	0	0.003283	0	0	0	0	0	0.000699	0
Clustering-based subsystems	NULL	CBSS-314269.3.peg.1840	carbon monoxide dehydrogenase operon C protein	0	0	0	0	0	0.000487	0	0	0.000876	0	0	0
Respiration	NULL	Carbon monoxide induced hydrogenase	Carbon monoxide-responsive transcriptional activator CooA	0	0	0	0	0	0	0.000722	0	0	0	0	7.88775E-05
Virulence, Disease and Defense	Resistance to antibiotics and toxic compounds	Cobalt-zinc-cadmium resistance	Cation efflux system protein CusC precursor	0	0	0	0	0	0	0	0	0	0	0.006055	0.000512704
Regulation and Cell signaling	Programmed Cell Death and Toxin-antitoxin Systems	Toxin-antitoxin systems (other than RelBE and MazEF)	CcdB toxin protein	0	0	0	0	0	0	0.000722	0	0	0	0	7.88775E-05
Virulence, Disease and Defense	Resistance to antibiotics and toxic compounds	Cobalt-zinc-cadmium resistance	Cd(II)/Pb(II)-responsive transcriptional regulator	0	0	0	0	0	0	0	0	0.001095	0	0	0.000907091
Cell Division and Cell Cycle	NULL	Bacterial Cytoskeleton	Cell-shape determining protein MreBH	0	0	0	0	0	0.000487	0	0	0	0	0	3.94388E-05
Membrane Transport	Protein secretion system, Type V	Two partner secretion pathway (TPS)	Channel-forming transporter/cytolysins activator of TpsB family	0	0	0	0	0	0	0	0	0.000219	0	0	3.94388E-05
Membrane Transport	NULL	Choline Transport	Choline ABC transporter permease protein	0	0	0	0	0.000657	0	0	0	0.000219	0	0	0
Membrane Transport	NULL	Choline Transport	Choline-binding lipoprotein	0	0	0	0	0	0	0.000722	0	0	0	0	3.94388E-05
Virulence, Disease and Defense	Resistance to antibiotics and toxic compounds	Resistance to chromium compounds	Chromate resistance protein ChrB	0	0	0	0	0	0	0	0	0.001314	0	0	0.000118316

Cell Division and Cell Cycle	NULL	MukBEF Chromosome Condensation	Chromosome partition protein MukE	0	0	0	0	0.000657	0	0	0	0	0	0	3.94388E-05
Membrane Transport	NULL	Citrate Utilization System (CitAB, CitH, and tctABC)	CitH citrate transporter	0	0	0	0	0	0	0	0	0.000219	0	0	3.94388E-05
Iron acquisition and metabolism	Siderophores	Siderophore Aerobactin	Citrate:6-N-acetyl-6-N-hydroxy-L-lysine ligase, alpha subunit (EC 6.3.2.27), aerobactin biosynthesis protein IucA	0	0	0	0	0	0	0.000722	0.000498	0	0	0	0
Respiration	NULL	Carbon monoxide induced hydrogenase	CO dehydrogenase/acetyl-CoA synthase subunit epsilon, CO dehydrogenase subcomplex (EC 1.2.99.2)	0	0	0.002555	0	0	0	0	0	0	0	0	0.000276071
Respiration	Electron donating reactions	Coenzyme F420-H2 dehydrogenase (methanophenazine)	Coenzyme F(420)H(2) dehydrogenase (methanophenazine) subunit FpoB	0	0	0	0	0	0	0	0	0.000219	0	0	3.94388E-05
Respiration	Electron donating reactions	NiFe hydrogenase maturation	Coenzyme F420 hydrogenase alpha subunit (FrcA) (EC 1.12.98.1)	0	0	0	0.00747	0	0	0	0	0	0	0	0.000276071
Nucleosides and Nucleotides	Detoxification	Housecleaning nucleoside triphosphate pyrophosphatases	Cof protein, HD superfamily hydrolase	0	0	0	0	0	0.000487	0	0	0	0	0	0.000512704
Cell Wall and Capsule	Gram-Positive cell wall components	Teichoic and lipoteichoic acids biosynthesis	COG1887: Putative glycosyl/glycerophosphate transferases involved in teichoic acid biosynthesis TagF/TagB/EpsJ/RodC	0.000348	0	0	0	0.000657	0	0	0	0	0	0	0
Miscellaneous	Plant-Prokaryote DOE project	Conserved gene cluster possibly involved in RNA metabolism	COG1939: Ribonuclease III family protein	0	0	0	0	0	0	0	0	0	0	0.000116	3.94388E-05
RNA Metabolism	RNA processing and modification	tRNA modification Archaea	COG2117: Predicted ATPase of the PP-loop superfamily	0	0	0	0	0	0	0	0	0.000876	0	0	7.88775E-05
Stress Response	Cold shock	Cold shock, CspA family of proteins	Cold shock protein CspI	0	0	0	0	0	0.000487	0	0.000498	0	0	0	0
Virulence, Disease and Defense	Bacteriocins, ribosomally synthesized antibacterial peptides	Tolerance to colicin E2	Colicin E2 tolerance protein CbrC	0	0	0	0	0	0.000974	0	0	0	0	0	0.000118316
DNA Metabolism	DNA uptake, competence	Gram Positive Competence	ComF operon protein A, DNA transporter ATPase	0	0	0	0.00249	0	0	0	0	0	0	0	7.88775E-05
Miscellaneous	Plant-Prokaryote DOE project	Competence or DNA damage-inducible protein CinA and related protein families	Competence/damage-inducible protein CinA family, archaeal branch	0	0.002262	0	0	0	0	0	0	0	0	0	0.000276071
Clustering-based subsystems	NULL	Conjugative transfer related cluster	Conjugative transfer protein TraI, relaxase	0	0	0	0	0	0	0	0	0	0	0.000233	0.000157755
Membrane Transport	Protein and nucleoprotein secretion system, Type IV	Conjugative transfer	Conjugative transfer protein TrbC	0	0	0	0	0	0.000487	0	0	0	0	0	0.00031551
Membrane Transport	Protein and nucleoprotein secretion system, Type IV	Conjugative transfer	Conjugative transfer protein TrbD	0	0	0	0	0.000657	0	0.000722	0	0	0	0	0
Membrane Transport	Protein and nucleoprotein secretion system, Type IV	Conjugative transfer	Conjugative transfer protein TrbJ	0	0	0	0	0.001313	0	0	0	0.000219	0	0	0
Phages, Prophages, Transposable elements, Plasmids	Transposable elements	Conjugative transposon, Bacteroidales	Conjugative transposon protein TraI	0	0	0	0	0	0	0	0	0.000657	0	0	0.000276071
Phages, Prophages, Transposable elements, Plasmids	Transposable elements	Conjugative transposon, Bacteroidales	Conjugative transposon protein TraJ	0	0	0	0	0	0	0	0	0.000219	0	0	0.000394388
Phages, Prophages, Transposable elements, Plasmids	Transposable elements	Conjugative transposon, Bacteroidales	Conjugative transposon protein TraK	0	0	0	0	0	0	0	0	0.000876	0	0	0.000985969
Phages, Prophages, Transposable elements, Plasmids	Transposable elements	Conjugative transposon, Bacteroidales	Conjugative transposon protein TraN	0	0	0	0	0	0	0	0	0.000219	0	0	0.000236633
Phages, Prophages, Transposable elements, Plasmids	Phages, Prophages	r1t-like streptococcal phages	conserved hypothetical protein - phage associated	0	0	0	0	0	0	0	0.000997	0	0	0	3.94388E-05
Sulfur Metabolism	Inorganic sulfur assimilation	Inorganic Sulfur Assimilation	Conserved hypothetical protein probably involved in sulfate reduction	0	0	0	0	0	0	0	0	0.000219	0	0	0.000157755
Miscellaneous	Plant-Prokaryote DOE project	At1g10830	Conserved NnrU/NnuR ortholog membrane enzyme	0	0	0	0	0	0.000974	0	0	0.001752	0	0	0
Virulence, Disease and Defense	Resistance to antibiotics and toxic compounds	Copper homeostasis	Copper-binding periplasmic protein	0	0	0	0	0	0	0	0	0.000219	0	0	3.94388E-05
DNA Metabolism	CRISPs	CRISP Cmr Cluster	CRISPR-associated protein Csx11	0	0	0	0	0	0	0	0.000498	0	0	0	3.94388E-05
DNA Metabolism	CRISPs	CRISP Cmr Cluster	CRISPR-associated RAMP Cmr1	0	0	0	0	0	0.000487	0	0	0.000876	0	0	0
Nitrogen Metabolism	NULL	Cyanate hydrolysis	Cyanate transport protein CynX	0	0	0	0	0	0	0	0	0.000219	0	0.000116	0
RNA Metabolism	Transcription	Transcription factors cyanobacterial RpoD-like sigma factors	Cyanobacteria-specific RpoD-like sigma factor, type-13	0	0	0	0	0	0	0	0	0.000219	0	0.000116	0
RNA Metabolism	Transcription	Transcription factors cyanobacterial RpoD-like sigma factors	Cyanobacteria-specific RpoD-like sigma factor, type-2	0	0	0	0	0.000657	0	0	0	0.000438	0	0	0
Miscellaneous	Plant-Prokaryote DOE project	Iron-sulfur cluster assembly	Cysteine desulfurase CsdA-CsdE, sulfur acceptor protein CsdE	0	0	0	0	0	0	0	0	0.000438	0	0	0.000394388
Virulence, Disease and Defense	Resistance to antibiotics and toxic compounds	Adaptation to d-cysteine	Cystine ABC transporter, permease protein	0	0	0	0	0	0	0	0	0.000438	0	0	0.000433826
Photosynthesis	Electron transport and photophosphorylation	Photosystem II	Cytochrome b559 beta chain (PsbF)	0	0	0	0	0	0.000974	0	0.000997	0	0	0	0
Respiration	NULL	Cytochrome B6-F complex	Cytochrome b6-f complex alternative Rieske iron sulfur protein PetC2	0	0	0	0	0	0	0	0	0.000657	0	0	0.000118316
Respiration	NULL	Cytochrome B6-F complex	Cytochrome b6-f complex subunit V (PetG)	0	0	0	0	0	0	0	0	0.000438	0	0	7.88775E-05
Clustering-based subsystems	Methylamine utilization	CBSS-265072.7.peg.546	Cytochrome c in methylamine utilization cluster	0	0	0	0	0.000657	0.000487	0	0	0	0	0	0
Nitrogen Metabolism	NULL	Dissimilatory nitrite reductase	Cytochrome c551 NirM	0	0.002262	0	0	0	0	0	0	0	0	0	0.000276071
Respiration	NULL	Biogenesis of c-type cytochromes	Cytochrome c-type biogenesis protein, archaeal, distantly related to heme lyase subunit CcmF	0	0	0	0	0	0.000487	0	0	0	0	0	3.94388E-05
Carbohydrates	Monosaccharides	D-allose utilization	D-allose ABC transporter, substrate-binding component	0	0	0	0	0	0.000487	0	0	0	0	0	3.94388E-05
Respiration	Electron donating reactions	Respiratory dehydrogenases 1	D-amino acid dehydrogenase large subunit (EC 1.4.99.1)	0	0	0	0	0.000657	0	0	0	0.000438	0	0	0
Miscellaneous	NULL	DedA family of inner membrane proteins	DedA family inner membrane protein YabI	0	0	0	0	0	0	0	0.000498	0	0	0	3.94388E-05
Miscellaneous	NULL	DedA family of inner membrane proteins	DedA family inner membrane protein YohD	0	0	0	0	0	0	0	0	0.000438	0	0	0.000749336
Carbohydrates	Monosaccharides	Deoxyribose and Deoxynucleoside Catabolism	Deoxyribonucleoside regulator DeoR (transcriptional repressor)	0	0	0.002555	0	0	0	0	0	0	0	0	0.00031551
Iron acquisition and metabolism	Siderophores	Siderophore Desferrioxamine E	Desferrioxamine E biosynthesis protein DesD	0	0	0	0	0	0	0	0	0.000219	0.002203	0	0
Cofactors, Vitamins, Prosthetic Groups, Pigments	Riboflavin, FMN, FAD	Riboflavin, FMN and FAD metabolism	Diaminohydroxyphosphoribosylaminopyrimidine deaminase (EC 3.5.4.26)	0	0	0	0	0	0	0	0.000498	0	0	0	0.000118316
Clustering-based subsystems	Lysine, threonine, methionine, and cysteine	YeiH	DNA binding protein HpkR	0	0	0	0	0	0	0	0	0.000219	0	0	3.94388E-05
Phages, Prophages, Transposable elements, Plasmids	Phages, Prophages	Phage replication	DNA ligase, phage-associated	0	0	0	0	0	0.000487	0	0	0.000876	0	0	0
Phages, Prophages, Transposable elements, Plasmids	Phages, Prophages	Phage replication	DNA polymerase sliding clamp, phage-associated	0	0	0	0.00249	0	0	0	0	0	0	0	3.94388E-05
DNA Metabolism	DNA repair	DNA repair, bacterial	DNA polymerase-like protein MT3142	0	0	0	0	0	0	0	0	0.000219	0	0	0.000157755
Phages, Prophages, Transposable elements, Plasmids	Phages, Prophages	Phage integration and excision	DNA recombinase, phage-associated	0	0	0	0	0	0	0.002888	0.000498	0	0	0	0
DNA Metabolism	DNA repair	DNA repair and recombination eukaryotic	DNA repair protein RAD51	0	0	0	0	0	0.000487	0	0	0	0	0	0.000394388
DNA Metabolism	DNA replication	DNA topoisomerases, Type II, ATP-dependent	DNA reverse gyrase (EC 5.99.1.-)	0	0	0.002555	0	0	0	0	0	0	0	0	0.000118316
DNA Metabolism	NULL	DNA phosphorothioation	DNA sulfur modification protein DndE	0	0	0	0	0	0	0	0	0.001095	0	0	7.88775E-05

Phages, Prophages, Transposable elements, Plasmids	Phages, Prophages	Phage replication	DNA topoisomerase, phage-associated	0	0	0	0	0	0	0.002166	0.001495	0	0	0	0
Clustering-based subsystems	NULL	CBSS-83333.1.peg.946	DNA transformation protein TfoX	0	0	0	0	0	0	0	0	0	0	0.010595	7.88775E-05
Regulation and Cell signaling	Regulation of virulence	Two-component Response Regulator of Virulence ResDE	DNA-binding response regulator ResD	0	0	0	0	0	0	0	0	0.002846	0	0	0.000157755
RNA Metabolism	Transcription	RNA polymerase archaeal	DNA-directed RNA polymerase subunit L (EC 2.7.7.6)	0	0	0	0	0	0.000487	0	0	0.000876	0	0	0
RNA Metabolism	Transcription	RNA polymerase archaeal	DNA-directed RNA polymerase subunit P (EC 2.7.7.6)	0	0	0	0	0	0	0.000722	0	0	0	0	3.94388E-05
RNA Metabolism	Transcription	RNA polymerase III	DNA-directed RNA polymerases I, II, and III 8.3 kDa polypeptide (EC 2.7.7.6)	0	0	0	0	0	0	0.000722	0	0	0	0	7.88775E-05
Membrane Transport	Protein and nucleoprotein secretion system, Type IV	Dot-Icm type IV secretion system	DotC protein	0	0	0	0	0	0	0.001444	0.000997	0	0	0	0
Iron acquisition and metabolism	Siderophores	Siderophore pyochelin	Enantio-pyochelin synthetase PchF, non-ribosomal peptide synthetase module	0	0	0	0	0	0	0	0.000997	0.000219	0	0	0
Carbohydrates	Monosaccharides	Mannose Metabolism	Endo-alpha-mannosidase	0	0	0	0	0	0.000487	0	0	0	0	0	0.000236633
Phages, Prophages, Transposable elements, Plasmids	Phages, Prophages	Listeria phi-A118-like prophages	Endolysin, L-alanyl-D-glutamate peptidase (EC 3.4.-.-) [Bacteriophage A118]	0	0.002262	0	0	0	0	0	0	0	0	0	7.88775E-05
Respiration	Reverse electron transport	Energy conserving hydrogenase, Methanococcales-Methanobacteriales-Methanopyrales	Energy conserving hydrogenase Eha associated protein, ribokinase homolog (protein T)	0.002785	0	0	0	0	0	0	0	0	0	0	0.000394388
Respiration	Reverse electron transport	Archaeal membrane bound hydrogenases	Energy conserving hydrogenase Ehb protein H	0	0	0	0	0	0.000487	0	0	0	0	0	3.94388E-05
Iron acquisition and metabolism	Siderophores	Siderophore Enterobactin	Enterobactin synthetase component F, serine activating enzyme (EC 2.7.7.-)	0	0	0	0	0	0	0	0	0.000219	0	0	0.000157755
Carbohydrates	Sugar alcohols	Ethanolamine utilization	Ethanolamine sensory transduction histidine kinase	0	0	0	0	0	0.000487	0	0	0	0	0	7.88775E-05
Carbohydrates	Sugar alcohols	Ethanolamine utilization	Ethanolamine utilization protein EutA	0	0	0	0	0.000657	0	0	0	0	0	0	0.001340918
Carbohydrates	Sugar alcohols	Ethanolamine utilization	Ethanolamine utilization protein EutG	0	0	0	0	0	0.001461	0	0	0	0	0	0.000394388
Metabolism of Aromatic Compounds	Anaerobic degradation of aromatic compounds	Anaerobic toluene and ethylbenzene degradation	Ethylbenzene dehydrogenase gamma subunit (EC 1.17.99.2)	0	0	0	0	0	0	0	0	0	0	0.000349	0.000157755
Amino Acids and Derivatives	Arginine; urea cycle, polyamines	Urea decomposition	Eukaryotic-type low-affinity urea transporter	0	0	0	0	0	0	0	0	0.000219	0	0	0.000197194
Carbohydrates	Di- and oligosaccharides	Lactose utilization	Evolved beta-D-galactosidase transcriptional repressor	0	0	0	0	0	0	0	0.000498	0	0	0	7.88775E-05
Clustering-based subsystems	Biosynthesis of galactoglycans and related lipopolysacharides	CBSS-258594.1.peg.3339	Exopolysaccharide production protein ExoQ	0	0	0	0	0	0.000487	0	0	0.000219	0	0	0
RNA Metabolism	RNA processing and modification	Exosome	Exosome complex exonuclease RRP4	0	0	0	0	0	0.000487	0	0	0	0	0	3.94388E-05
Dormancy and Sporulation	NULL	Exosporium	Exosporium protein J	0	0.002262	0	0	0	0	0	0.000997	0	0	0	0
DNA Metabolism	DNA uptake, competence	Natural DNA Transformation in Vibrio	Extracellular deoxyribonuclease Dns (EC 3.1.21.-)	0	0	0	0	0	0	0	0	0	0	0.007685	0.00189306
Metabolism of Aromatic Compounds	Metabolism of central aromatic intermediates	Central meta-cleavage pathway of aromatic compound degradation	extradiol dioxygenase large subunit	0	0	0	0	0	0	0	0	0	0	0.000116	3.94388E-05
Carbohydrates	Di- and oligosaccharides	Sucrose utilization	Family 13 glycosyl hydrolase, row 724	0	0	0	0.00249	0	0	0	0	0	0	0	7.88775E-05
Membrane Transport	Protein secretion system, Type VIII (Extracellular nucleation/precipitation pathway, ENP)	Fap amyloid fiber secretion system	Fap amyloid fiber secretin	0	0	0	0	0	0	0	0	0.000438	0	0	7.88775E-05
Fatty Acids, Lipids, and Isoprenoids	Isoprenoids	Archaeal lipids	Farnesylgeranyl pyrophosphate synthetase	0	0	0	0	0	0	0	0	0.000219	0	0	0.000157755
Sulfur Metabolism	Inorganic sulfur assimilation	Inorganic Sulfur Assimilation	Ferredoxin--sulfite reductase, actinobacterial type (EC 1.8.7.1)	0	0.002262	0	0	0	0	0	0	0	0	0.000116	0
Nitrogen Metabolism	NULL	Nitrate and nitrite ammonification	Ferredoxin-type protein NapF (periplasmic nitrate reductase)	0	0	0	0.00249	0	0	0	0	0	0	0	0.000709898
Iron acquisition and metabolism	NULL	Iron acquisition in Vibrio	Ferric hydroxamate ABC transporter (TC 3.A.1.14.3), periplasmic substrate binding protein FhuD	0	0	0	0	0	0	0	0	0.000876	0	0.000233	0
Iron acquisition and metabolism	NULL	Iron acquisition in Vibrio	Ferric vibriobactin, enterobactin transport system, permease protein VctD (TC 3.A.1.14.6)	0	0	0	0	0	0	0	0.000498	0	0	0	3.94388E-05
Iron acquisition and metabolism	Siderophores	Vibrioferrin synthesis	Ferrichrome ABC transporter (ATP binding subunit) PvuE	0	0	0	0	0	0	0	0	0	0	0.000466	3.94388E-05
Clustering-based subsystems	Cell Division	Cell Division Cluster	FIG001960: FtsZ-interacting protein related to cell division	0	0	0	0	0.000657	0	0	0	0	0	0	7.88775E-05
Clustering-based subsystems	Ribosome-related cluster	A Gammaproteobacteria Cluster Relating to Translation	FIG002708: Protein SirB1	0	0	0	0	0	0	0	0	0.002408	0	0	7.88775E-05
Clustering-based subsystems	Cytochrome biogenesis	CBSS-196164.1.peg.1690	FIG020413: transmembrane protein	0	0	0	0	0	0.000487	0.000722	0	0	0	0	0
Fatty Acids, Lipids, and Isoprenoids	Fatty acids	Phospholipid and Fatty acid biosynthesis related cluster	FIG025233: SAM-dependent methyltransferases	0	0	0	0	0.000657	0	0	0	0.000219	0	0	0
Clustering-based subsystems	NULL	CBSS-316273.3.peg.2709	FIG034376: Hypothetical protein	0	0	0	0	0.000657	0	0	0	0	0	0	0.000157755
Clustering-based subsystems	NULL	CBSS-316273.3.peg.922	FIG034602: Probable transmembrane protein	0	0	0	0	0	0	0	0	0.000219	0	0	7.88775E-05
Respiration	NULL	Soluble cytochromes and functionally related electron carriers	FIG135464: Cytochrome c4	0	0	0	0	0	0	0	0	0.000657	0	0	0.001183163
Clustering-based subsystems	NULL	PFGI-1-like cluster 1	FIG141694: hypothetical protein in PFGI-1-like cluster	0	0	0	0	0.000657	0	0	0	0	0	0	0.000788775
Miscellaneous	Plant-Prokaryote DOE project	At4g10620 At3g57180 At3g47450	FIG145533: Methyltransferase (EC 2.1.1.-)	0	0	0	0	0.000657	0	0	0	0	0	0	7.88775E-05
Membrane Transport	NULL	Phosphoglycerate transport system	FIGfam009438: Two-component system DNA-binding response regulator	0.000348	0	0	0	0	0	0	0	0	0	0	0.000670459
Phages, Prophages, Transposable elements, Plasmids	Transposable elements	CBSS-203122.12.peg.188	FIGfam110555	0	0	0	0	0	0	0	0	0.000219	0	0	3.94388E-05
Motility and Chemotaxis	Flagellar motility in Prokaryota	Flagellum	Flagellar biosynthesis protein FlgN	0	0	0	0	0	0	0	0.000997	0.000657	0	0	0
Motility and Chemotaxis	Flagellar motility in Prokaryota	Additional flagellar genes in Vibrionales	Flagellar protein FlgO	0	0	0	0	0	0	0	0.000498	0	0	0	3.94388E-05
Motility and Chemotaxis	Flagellar motility in Prokaryota	Archaeal Flagellum	Flagella-related protein FlaH	0	0	0	0	0	0	0	0	0.000438	0	0	3.94388E-05
Motility and Chemotaxis	Flagellar motility in Prokaryota	Flagellum	Flagellin protein FlaD	0	0	0	0	0	0	0	0	0.000438	0	0	0.000157755
Respiration	NULL	Formate hydrogenase	Formate hydrogenlyase subunit 6	0	0	0	0	0	0	0	0	0.000219	0	0	7.88775E-05
Carbohydrates	One-carbon Metabolism	Methanogenesis	Formylmethanofuran dehydrogenase (molybdenum) subunit B (EC 1.2.99.5)	0	0	0	0	0	0	0	0	0.000438	0	0	0.000197194
Carbohydrates	One-carbon Metabolism	Methanogenesis	Formylmethanofuran dehydrogenase subunit C (EC 1.2.99.5)	0	0	0	0	0	0	0.000722	0	0	0	0	0.000157755
Membrane Transport	Sugar Phosphotransferase Systems, PTS	Fructose and Mannose Inducible PTS	Fructanase (EC 3.2.1.80), FruA / Levanase (EC 3.2.1.65)	0.000696	0	0	0	0	0	0	0	0	0	0	3.94388E-05
Metabolism of Aromatic Compounds	Metabolism of central aromatic intermediates	Salicylate and gentisate catabolism	Fumarylacetoacetate (FAA) hydrolase (EC 4.1.1.68)	0	0	0	0.00249	0	0	0	0	0	0	0	7.88775E-05
Sulfur Metabolism	NULL	Galactosylceramide and Sulfatide metabolism	Galactocerebrosidase precursor (EC 3.2.1.46)	0	0	0	0	0	0	0	0	0	0.002203	0	7.88775E-05
Motility and Chemotaxis	NULL	Bacterial Chemotaxis	Galactose/methyl galactoside ABC transport system, D-galactose-binding periplasmic protein MglB (TC 3.A.1.2.3)	0	0	0	0	0	0.000487	0	0	0	0	0	0.001380356

Carbohydrates	Di- and oligosaccharides	Lactose and Galactose Uptake and Utilization	Galactose/methyl galactoside ABC transport system, permease protein MglC (TC 3.A.1.2.3)	0	0	0	0	0.000657	0	0	0	0	0	0	0.001025408
Carbohydrates	Monosaccharides	D-Galacturonate and D-Glucuronate Utilization	Galacturan 1,4-alpha-galacturonidase (EC 3.2.1.67)	0	0	0	0	0	0	0	0.000498	0	0	0	3.94388E-05
Cell Wall and Capsule	NULL	Recycling of Peptidoglycan Amino Acids	Gamma-D-Glutamyl-meso-Diaminopimelate Amidase	0	0	0	0	0	0.000974	0	0	0	0	0	7.88775E-05
Phages, Prophages, Transposable elements, Plasmids	Gene Transfer Agent (GTA)	Gene Transfer Agent	Gene Transfer Agent (GTA) ORFG07	0	0	0.002555	0	0	0	0	0	0	0	0	0.000118316
Phages, Prophages, Transposable elements, Plasmids	Gene Transfer Agent (GTA)	Gene Transfer Agent	Gene Transfer Agent associated protein Pden 2900	0	0	0	0	0	0	0	0	0.000438	0	0	7.88775E-05
Membrane Transport	Protein secretion system, Type II	General Secretion Pathway	General secretion pathway protein O	0	0	0	0.00249	0	0	0	0.000498	0	0	0	0
Nitrogen Metabolism	NULL	Ammonia assimilation	Global nitrogen regulatory protein, CRP family of transcriptional regulators	0	0	0	0	0	0	0	0	0.001971	0	0.000233	0
Carbohydrates	Monosaccharides	D-Sorbitol(D-Glucitol) and L-Sorbose Utilization	Glucitol operon GutQ protein	0	0	0	0	0	0	0	0	0	0	0.000349	7.88775E-05
Carbohydrates	Monosaccharides	D-gluconate and ketogluconates metabolism	Gluconate 2-dehydrogenase (EC 1.1.99.3), membrane-bound, cytochrome c	0	0	0	0	0	0.000487	0	0	0	0	0	7.88775E-05
Cell Wall and Capsule	NULL	UDP-N-acetylmuramate from Fructose-6-phosphate Biosynthesis	Glucosamine 6-phosphate N-acetyltransferase (EC 2.3.1.4)	0	0	0	0	0	0	0	0.000997	0.000657	0	0	0
Amino Acids and Derivatives	Glutamine, glutamate, aspartate, asparagine; ammonia assimilation	Glutamine, Glutamate, Aspartate and Asparagine Biosynthesis	Glutamine synthetase, clostridia type (EC 6.3.1.2)	0	0	0	0	0	0	0	0	0	0	0.000116	0.000867653
Stress Response	Oxidative stress	Glutathione: Non-redox reactions	Glutathione S-transferase, theta (EC 2.5.1.18)	0	0	0	0	0	0	0	0	0.000438	0	0	7.88775E-05
Carbohydrates	Polysaccharides	Glycogen metabolism	Glycogen biosynthesis protein GlgD, glucose-1-phosphate adenylyltransferase family	0	0	0	0	0	0	0	0	0.000219	0	0	0.00063102
Cell Wall and Capsule	Gram-Positive cell wall components	Teichoic and lipoteichoic acids biosynthesis	Glycosyl transferase involved in teichoic acid biosynthesis	0	0	0	0	0	0	0	0	0.000219	0	0	0.000157755
Clustering-based subsystems	Biosynthesis of galactoglycans and related lipopolysacharides	CBSS-376686.6.peg.291	Glycosyl transferase, group 1 (EC 2.-.-.-)	0	0	0	0	0	0	0	0.000498	0	0	0	0.002800152
Regulation and Cell signaling	Quorum sensing and biofilm formation	Symbiotic colonization and sigma-dependent biofilm formation gene cluster	Glycosyltransferase SypQ	0	0	0	0	0	0	0	0	0	0	0.000116	3.94388E-05
Cofactors, Vitamins, Prosthetic Groups, Pigments	Folate and pterines	Methanopterin biosynthesis2	GTP cyclohydrolase III (EC 3.5.4.29)	0	0	0	0	0	0	0	0.000997	0.002189	0	0	0
Protein Metabolism	Protein biosynthesis	Universal GTPases	GTPase Nop2p which associates with pre-60S ribosomal subunits in the nucleolus and is required for their export and maturation	0	0	0	0	0	0	0	0	0.000219	0	0.001048	0
Clustering-based subsystems	NULL	Conserved gene cluster associated with Met-tRNA formyltransferase	GTP-sensing transcriptional pleiotropic repressor codY	0	0	0	0	0	0	0	0	0	0	0.000466	0.000394388
Virulence, Disease and Defense	Resistance to antibiotics and toxic compounds	Copper homeostasis	Heavy metal-(Cd/Co/Hg/Pb/Zn)-translocating P-type ATPase:Heavy metal translocating P-type ATPase	0	0	0	0	0	0	0	0	0	0	0.000233	7.88775E-05
Iron acquisition and metabolism	NULL	Iron Scavenging cluster in Thermus	Heme-degrading oxygenase, IsdG-like	0	0	0	0	0.000657	0	0	0	0	0	0	0.000197194
Iron acquisition and metabolism	NULL	Heme, hemin uptake and utilization systems in GramPositives	Hemoglobin, heme-dependent two component system response regulator ChrA	0	0	0	0	0.000657	0	0	0	0	0	0	0.000433826
Iron acquisition and metabolism	NULL	Heme, hemin uptake and utilization systems in GramPositives	Hemoglobin-dependent two component system response regulator HrrA	0	0	0	0.00498	0	0	0	0	0	0	0	0.000709898
Cell Wall and Capsule	Capsular and extracellular polysacchrides	Vibrio Polysaccharide (VPS) Biosynthesis	Hemolysin-related protein Vcp	0	0	0	0	0	0	0	0	0.000219	0	0	3.94388E-05
Miscellaneous	Plant-Prokaryote DOE project	At5g48545 and At3g56490 At1g31160	Histidine triad (HIT) nucleotide-binding protein, yeast YDL125C (HNT1) homolog	0	0	0	0	0	0	0	0	0.000219	0	0	0.000118316
RNA Metabolism	Transcription	Rrf2 family transcriptional regulators	Hmc operon transcriptional regulator Rrf2	0	0	0	0	0	0.001461	0	0	0	0	0	0.000236633
Virulence, Disease and Defense	Resistance to antibiotics and toxic compounds	Methicillin resistance in Staphylococci	HmrA protein involved in methicillin resistance	0.000348	0	0	0	0	0	0	0	0	0	0	3.94388E-05
Fatty Acids, Lipids, and Isoprenoids	Fatty acids	Fatty Acid Biosynthesis FASII	Holo-[acyl-carrier protein] synthase, alternative (EC 2.7.8.7)	0	0	0	0	0	0	0	0.000498	0	0.002203	0	0
Amino Acids and Derivatives	Lysine, threonine, methionine, and cysteine	Lysine biosynthesis AAA pathway 2	Homoaconitase small subunit (EC 4.2.1.36)	0	0	0	0	0	0	0	0	0.001095	0	0	0.000354949
Cofactors, Vitamins, Prosthetic Groups, Pigments	Tetrapyrroles	Coenzyme B12 biosynthesis	HoxN/HupN/NixA family cobalt transporter	0	0	0	0	0	0	0	0	0	0.004406	0	0.000236633
Carbohydrates	Monosaccharides	2-Ketogluconate Utilization	HTH-type transcriptional regulator PtxR	0	0	0	0	0	0	0	0	0	0	0.000466	0.000157755
Respiration	NULL	Formate hydrogenase	hydrogenase, group 4, HycE subunit, putative	0.001044	0	0	0	0	0	0	0	0	0	0	0.000118316
Respiration	NULL	Formate hydrogenase	hydrogenase, group 4, HycG subunit, putative	0	0	0	0	0	0	0	0	0.000219	0	0	0.000236633
Respiration	Electron donating reactions	Hydrogenases	hydrogenase/sulfur reductase, beta subunit	0	0.002262	0	0	0	0	0	0	0	0	0	0.001143724
Miscellaneous	Plant-Prokaryote DOE project	Synechocystis experimental	Hydrolase alpha/beta fold family, slr0264 homolog	0	0	0	0	0	0	0	0.000498	0.000438	0	0	0
Regulation and Cell signaling	NULL	Signal transduction module [RsbQ hydrolase - PAS domain]	Hydrolase of unknown specificity RsbQ, part of a novel [RsbQ - PAS domain] bacterial sensing module	0	0	0	0	0	0	0	0	0.000876	0	0	0.000118316
Metabolism of Aromatic Compounds	Anaerobic degradation of aromatic compounds	Hydroxyaromatic decarboxylase family	Hydroxyaromatic non-oxidative decarboxylase protein B (EC 4.1.1.-)	0	0	0	0	0	0.000487	0	0	0	0	0	0.000118316
Metabolism of Aromatic Compounds	Anaerobic degradation of aromatic compounds	Hydroxyaromatic decarboxylase family	Hydroxyaromatic non-oxidative decarboxylase protein C (EC 4.1.1.-)	0.006267	0	0	0	0	0	0	0	0	0	0	0.000197194
Metabolism of Aromatic Compounds	NULL	Benzoate transport and degradation cluster	hydroxybenzoate permease	0	0	0	0	0	0	0	0	0.000219	0	0	3.94388E-05
Respiration	Electron accepting reactions	Terminal cytochrome d ubiquinol oxidases	Hypothetical bd-type Menaquinol oxidase subunit	0	0	0	0	0.000657	0	0.000722	0	0	0	0	0
Miscellaneous	Plant-Prokaryote DOE project	At1g54520	Hypothetical cyanobacterial membrane protein, in cluster with PxcA	0	0	0	0	0	0	0	0.002492	0.000876	0	0	0
Stress Response	Oxidative stress	Rubrerythrin	Hypothetical protein i Rubrerythrin cluster	0.000348	0	0	0	0	0	0	0	0	0	0	0.000118316
Carbohydrates	Central carbohydrate metabolism	Dihydroxyacetone kinases	Hypothetical protein in cluster with dihydroxyacetone kinase in Rhizobia	0	0	0	0	0	0	0	0.000498	0	0	0.000116	0
Iron acquisition and metabolism	Siderophores	Siderophore Pyoverdine	Hypothetical protein PvdX	0	0	0	0	0	0	0	0	0.000438	0	0	0.000276071
Clustering-based subsystems	tRNA sulfuration	CBSS-89187.3.peg.2957	hypothetical protein SKA53 09994	0	0	0	0	0	0	0	0.000498	0	0	0	3.94388E-05
Cofactors, Vitamins, Prosthetic Groups, Pigments	NULL	Molybdopterin cytosine dinucleotide	Hypothetical protein YagQ	0	0	0	0	0.000657	0	0	0	0.000438	0	0	0
Phages, Prophages, Transposable elements, Plasmids	Phages, Prophages	Listeria phi-A118-like prophages	Hypothetical protein, Lmo2313 homolog [Bacteriophage A118]	0	0	0	0	0.001313	0	0	0.000498	0	0	0	0
Membrane Transport	Protein and nucleoprotein secretion system, Type IV	Dot-Icm type IV secretion system	IcmK (DotH) protein	0	0	0	0	0	0.000974	0	0.001993	0	0	0	0
Dormancy and Sporulation	NULL	Bacillus biofilm matrix protein component TasA and homologs	Immune inhibitor A metalloprotease	0	0	0	0	0	0	0	0	0.000438	0	0	3.94388E-05
Nucleosides and Nucleotides	Purines	Purine conversions	IMP dehydrogenase related 1 (EC 1.1.1.205)	0	0	0	0	0	0.000487	0	0	0	0	0	7.88775E-05
Membrane Transport	Protein and nucleoprotein secretion system, Type IV	Conjugative transfer	IncF plasmid conjugative transfer pilus assembly protein TraK	0	0.002262	0	0	0	0	0	0	0	0	0	3.94388E-05
Membrane Transport	Protein and nucleoprotein secretion system, Type IV	Conjugative transfer	IncF plasmid conjugative transfer surface exclusion protein TraT	0	0	0	0	0	0.000487	0	0	0	0	0	0.000157755
Membrane Transport	Protein and nucleoprotein secretion system, Type IV	Type 4 conjugative transfer system, IncI1 type	IncI1 plasmid conjugative transfer integral membrane protein TraY	0	0	0	0	0	0	0	0.000498	0	0	0	0.000118316
Secondary Metabolism	Plant Hormones	Auxin biosynthesis	Indole-acetamide hydrolase	0	0	0	0	0	0.000487	0	0	0	0	0	0.000197194

Clustering-based subsystems	NULL	Conserved cluster in Enterobacteriaceae downstream from YqjA, a DedA family protein	Inner membrane protein YhaH	0	0	0	0	0	0	0	0	0.000657	0	0	0.000118316
Phages, Prophages, Transposable elements, Plasmids	NULL	Integrons	Integron integrase IntI1	0	0	0	0	0	0	0.000722	0	0	0	0	7.88775E-05
Virulence, Disease and Defense	Invasion and intracellular resistance	Listeria surface proteins: Internalin-like proteins	Internalin A (LPXTG motif)	0	0	0	0.00249	0	0	0	0	0	0	0	0.000276071
Virulence, Disease and Defense	Invasion and intracellular resistance	Listeria surface proteins: Internalin-like proteins	Internalin G (LPXTG motif)	0	0	0	0	0	0.000487	0	0	0	0	0	7.88775E-05
Virulence, Disease and Defense	Invasion and intracellular resistance	Listeria surface proteins: Internalin-like proteins	Internalin-like protein (LPXTG motif) Lin0372 homolog	0	0	0	0	0	0.000487	0	0	0	0	0.000116	0
Virulence, Disease and Defense	Invasion and intracellular resistance	Listeria surface proteins: Internalin-like proteins	Internalin-like protein (LPXTG motif) Lmo0409 homolog	0	0.002262	0	0	0	0	0	0	0	0	0	0.000394388
Virulence, Disease and Defense	Invasion and intracellular resistance	Listeria surface proteins: Internalin-like proteins	Internalin-like protein (LPXTG motif) Lmo2821 homolog	0	0	0	0	0	0	0	0	0.000219	0	0.000466	0
Iron acquisition and metabolism	Siderophores	Siderophore Yersiniabactin Biosynthesis	iron aquisition 2,3-dihydroxybenzoate-AMP ligase (EC 2.7.7.58,Irp5)	0	0	0	0	0.000657	0	0	0	0	0	0	0.000118316
Iron acquisition and metabolism	NULL	Iron acquisition in Vibrio	Iron-regulated virulence regulatory protein irgB	0	0	0	0	0	0	0	0	0	0	0.000116	0.000118316
Potassium metabolism	NULL	Potassium homeostasis	K transporter trk	0	0	0	0	0	0	0	0	0.000438	0	0	0.001340918
Amino Acids and Derivatives	Aromatic amino acids and derivatives	Tryptophan catabolism	Kynurenine formamidase (EC 3.5.1.9)	0	0	0	0	0	0	0.000722	0	0	0	0	0.000157755
Cell Wall and Capsule	NULL	Peptidoglycan Crosslinking of Peptide Stems	L,D-transpeptidase YcfS	0	0	0	0	0	0	0	0	0	0	0.000233	3.94388E-05
Cell Wall and Capsule	NULL	Peptidoglycan Crosslinking of Peptide Stems	L,D-transpeptidase YnhG	0	0	0	0	0	0	0	0	0.000438	0	0	7.88775E-05
Carbohydrates	Di- and oligosaccharides	Unknown oligosaccharide utilization Sde 1396	LacI family transcriptional regulator, row 218	0	0	0	0	0	0	0	0	0.000876	0	0	0.000157755
Carbohydrates	Di- and oligosaccharides	Lactose and Galactose Uptake and Utilization	Lactose phosphotransferase system repressor	0	0	0	0	0	0	0	0	0.000438	0	0	7.88775E-05
Metabolism of Aromatic Compounds	Peripheral pathways for catabolism of aromatic compounds	Biphenyl Degradation	Large subunit naph/bph dioxygenase	0	0	0	0	0	0	0	0	0.000657	0	0	0.000591581
DNA Metabolism	DNA uptake, competence	Gram Positive Competence	Late competence protein ComGB, access of DNA to ComEA	0	0	0	0	0	0	0	0	0.000219	0	0	7.88775E-05
Carbohydrates	Monosaccharides	D-gluconate and ketogluconates metabolism	L-idonate, D-gluconate, 5-keto-D-gluconate transporter	0	0	0	0	0	0	0	0	0.000657	0	0	0.000236633
Photosynthesis	Light-harvesting complexes	Bacterial light-harvesting proteins	Light-harvesting LHII, beta subunit B	0	0	0	0	0	0	0	0.000498	0.000219	0	0	0
Miscellaneous	Plant-Prokaryote DOE project	Synechocystis experimental	Lipase (EC 3.1.1.3) family protein, sll1969 homolog	0	0	0	0	0	0	0	0.000498	0	0	0	0.000197194
Cell Wall and Capsule	Gram-Negative cell wall components	LOS core oligosaccharide biosynthesis	Lipopolysaccharide 1,2-N-acetylglucosaminetransferase (EC 2.4.1.56)	0	0	0	0	0	0	0	0	0.000219	0	0	0.00031551
Amino Acids and Derivatives	Lysine, threonine, methionine, and cysteine	Lysine degradation	L-lysine aminomutase regulator	0	0	0	0	0	0.000487	0	0	0	0	0	0.000473265
Protein Metabolism	Protein degradation	Proteasome bacterial	LonB like ATP-ase no protease domain	0	0	0	0	0.002626	0	0	0	0	0	0	0.00063102
Nucleosides and Nucleotides	Detoxification	Nudix proteins (nucleoside triphosphate hydrolases)	Low G+C gram positive nudix hydrolase YtkD (EC 3.6.-.-)	0	0	0	0	0	0	0	0	0.000219	0	0	0.000236633
Carbohydrates	Monosaccharides	L-rhamnose utilization	L-rhamnonate dehydratase (EC 4.2.1.90)	0	0	0	0	0.000657	0	0	0	0	0	0	3.94388E-05
Protein Metabolism	Protein biosynthesis	Ribosome LSU eukaryotic and archaeal	LSU ribosomal protein L14e	0	0	0	0	0	0.000487	0	0	0	0	0	0.000354949
Protein Metabolism	Protein biosynthesis	Ribosome LSU eukaryotic and archaeal	LSU ribosomal protein L18Ae	0	0	0	0	0.000657	0	0	0	0	0	0	0.000197194
Protein Metabolism	Protein biosynthesis	Ribosome LSU chloroplast	LSU ribosomal protein L22p (L17e), chloroplast	0	0	0	0	0	0	0	0	0.000657	0	0	0.000118316
Protein Metabolism	Protein biosynthesis	Ribosome LSU chloroplast	LSU ribosomal protein L23p (L23Ae), chloroplast	0	0	0	0	0	0	0	0	0.000438	0	0	7.88775E-05
Protein Metabolism	Protein biosynthesis	Ribosome LSU eukaryotic and archaeal	LSU ribosomal protein L24e	0	0	0	0	0.000657	0	0	0	0	0	0	0.00031551
Protein Metabolism	Protein biosynthesis	Ribosome LSU chloroplast	LSU ribosomal protein L2p (L8e), chloroplast	0	0	0	0	0.001313	0	0	0	0	0	0	0.000118316
Clustering-based subsystems	Proteasome related clusters	Proteasome subunit alpha archaeal cluster	LSU ribosomal protein L37Ae	0	0	0	0	0	0	0	0	0.000219	0	0	7.88775E-05
Protein Metabolism	Protein biosynthesis	Ribosome LSU eukaryotic and archaeal	LSU ribosomal protein L38e	0	0	0	0	0.000657	0	0	0	0	0	0	3.94388E-05
Protein Metabolism	Protein biosynthesis	Ribosome LSU chloroplast	LSU ribosomal protein L6p (L9e), chloroplast	0	0.002262	0	0	0	0	0	0	0	0	0	7.88775E-05
Cofactors, Vitamins, Prosthetic Groups, Pigments	Riboflavin, FMN, FAD	Riboflavin, FMN and FAD metabolism	Lumazine protein, riboflavin synthase homolog	0	0	0	0	0	0	0	0	0.000219	0	0	0.000907091
Miscellaneous	NULL	Luciferases	LuxC, acyl-CoA reductase (EC 1.2.1.50)	0	0	0	0	0	0	0.000722	0	0	0	0	7.88775E-05
Fatty Acids, Lipids, and Isoprenoids	Isoprenoids	Carotenoids	Lycopene elongase (EC 2.5.1.-)	0	0	0	0	0	0.000487	0	0.000997	0	0	0	0
Amino Acids and Derivatives	Lysine, threonine, methionine, and cysteine	Lysine degradation	Lysine 2-monooxygenase (EC 1.13.12.2)	0	0	0	0	0	0	0	0	0.000876	0	0	0.000236633
Amino Acids and Derivatives	Lysine, threonine, methionine, and cysteine	Lysine degradation	Lysine decarboxylase, inducible (EC 4.1.1.18)	0	0	0	0	0	0	0	0	0.000438	0	0	0.000197194
Clustering-based subsystems	Lysine, threonine, methionine, and cysteine	YeiH	Lysine-specific permease	0	0	0	0	0	0.000487	0.000722	0	0	0	0	0
Regulation and Cell signaling	NULL	DNA-binding regulatory proteins, strays	LysR family transcriptional regulator PA5218	0	0	0	0	0.000657	0	0	0	0	0	0	3.94388E-05
Regulation and Cell signaling	NULL	DNA-binding regulatory proteins, strays	LysR family transcriptional regulator STM3121	0	0	0	0	0	0	0	0	0.001314	0	0	0.000118316
Clustering-based subsystems	NULL	Conserved cluster in Enterobacteriaceae downstream from YqjA, a DedA family protein	LysR-family transcriptional regulator YhaJ	0	0	0	0	0	0	0	0	0.000438	0	0	0.000197194
Membrane Transport	NULL	Agrobacterium opine transport	LysR-type transcriptional regulator for nopaline catabolism NocR	0	0	0	0	0	0	0	0.000997	0	0	0	3.94388E-05
Protein Metabolism	Protein biosynthesis	tRNA aminoacylation, Lys	Lysyl-tRNA synthetase (class II) (EC 6.1.1.6), mitochondrial	0	0	0	0	0	0	0	0	0	0	0.000116	7.88775E-05
Membrane Transport	Protein and nucleoprotein secretion system, Type IV	Vir-like type 4 secretion system	Major pilus subunit of type IV secretion complex, VirB2	0	0	0	0	0	0	0	0.000997	0	0	0	0.000118316
Carbohydrates	Organic acids	Malonate decarboxylase	Malonate decarboxylase beta subunit	0	0	0.002555	0	0	0	0	0	0	0	0	0.00063102
Carbohydrates	Organic acids	Malonate decarboxylase	Malonyl CoA acyl carrier protein transacylase (EC 2.3.1.39)	0	0	0	0	0	0.000487	0	0	0	0	0	3.94388E-05
Carbohydrates	Di- and oligosaccharides	Maltose and Maltodextrin Utilization	Maltose regulon modulator	0	0	0	0	0	0	0.000722	0	0	0	0	3.94388E-05
Carbohydrates	Di- and oligosaccharides	Maltose and Maltodextrin Utilization	Maltose-6'-phosphate glucosidase (EC 3.2.1.122)	0	0	0	0	0	0.000974	0	0	0	0	0	3.94388E-05
Membrane Transport	NULL	Agrobacterium opine transport	Mannopinic acid transporter periplasmic substrate-binding protein MoaA	0	0	0	0	0	0	0	0	0.000219	0	0	3.94388E-05
Virulence, Disease and Defense	NULL	C jejuni colonization of chick caeca	MCP-domain signal transduction protein	0	0	0	0	0	0	0	0	0.000657	0	0	0.002287448
Virulence, Disease and Defense	Adhesion	Mediator of hyperadherence YidE in Enterobacteria and its conserved region	Mediator of hyperadherence YidE	0	0	0	0	0	0	0	0.000498	0	0	0	3.94388E-05
DNA Metabolism	DNA repair	DNA repair and recombination eukaryotic	Meiotic recombination protein DMC1	0	0	0.002555	0	0	0.000487	0	0	0	0	0	0
Membrane Transport	Protein secretion system, Type I	Type I secretion system for aggregation	membrane bound c-di-GMP receptor (LapD)	0	0	0	0	0	0	0	0.000498	0	0	0	7.88775E-05

Cell Division and Cell Cycle	NULL	MukBEF Chromosome Condensation	Membrane Protein Functionally coupled to the MukBEF Chromosome Partitioning Mechanism	0	0	0	0	0	0.001948	0	0	0	0	0	0.00094653
Virulence, Disease and Defense	Resistance to antibiotics and toxic compounds	Copper homeostasis: copper tolerance	Membrane protein, suppressor for copper-sensitivity ScsD	0	0	0	0	0	0	0	0	0.000438	0	0	0.000828214
Regulation and Cell signaling	Proteolytic pathway	Regulatory Intramembrane Proteolysis Pathways	Membrane-bound transcription factor site-1 protease S1P	0	0	0	0	0	0	0	0	0	0	0.000116	0.00031551
Regulation and Cell signaling	Proteolytic pathway	MT1-MMP Pericellular Network	Membrane-type matrix metallopeptidase-1	0	0	0	0	0	0	0	0.000498	0	0	0	3.94388E-05
Cofactors, Vitamins, Prosthetic Groups, Pigments	Quinone cofactors	Menaquinone Biosynthesis via Futalosine -- gjo	Menaquinone via futalosine step 4, possible alternative	0	0	0	0.00498	0	0	0	0	0	0	0	0.000118316
Respiration	Electron accepting reactions	Ubiquinone Menaquinone-cytochrome c reductase complexes	Menaquinone-cytochrome C oxidoreductase, cytochrome C subunit	0	0	0	0	0	0	0	0	0.000876	0	0	0.000157755
Respiration	Electron accepting reactions	Ubiquinone Menaquinone-cytochrome c reductase complexes	Menaquinone-cytochrome C reductase iron-sulfur subunit	0	0	0	0	0	0	0	0	0.000219	0	0	7.88775E-05
Carbohydrates	Central carbohydrate metabolism	Soluble methane monooxygenase (sMMO)	Methane monooxygenase component C (EC 1.14.13.25)	0	0	0	0	0	0	0	0	0.000438	0	0	3.94388E-05
Carbohydrates	One-carbon Metabolism	Methanogenesis	Methyl coenzyme M reductase alpha subunit (EC 2.8.4.1)	0	0.002262	0	0	0	0	0	0	0	0	0	0.000236633
Respiration	Electron donating reactions	Hydrogenases	methyl viologen-reducing hydrogenase, delta subunit homolog FlpD	0.003133	0	0	0	0	0	0	0	0	0	0	0.000354949
Carbohydrates	Di- and oligosaccharides	Beta-Glucoside Metabolism	Methyl-accepting chemotaxis protein co-located with beta-glucan transporter system	0	0	0	0	0	0	0	0	0.000438	0	0	3.94388E-05
Motility and Chemotaxis	NULL	Bacterial Chemotaxis	Methyl-accepting chemotaxis protein II (aspartate chemoreceptor protein)	0	0	0	0	0	0	0.001444	0	0	0	0	0.000118316
Virulence, Disease and Defense	Invasion and intracellular resistance	Cytolysin and Lipase operon in Vibrio	Methyl-accepting chemotaxis protein, hemolysin secretion protein HylB	0	0	0	0	0	0	0	0	0.001314	0	0	0.00063102
Cell Wall and Capsule	Gram-Positive cell wall components	Anthrose Biosynthesis	Methyltransferase, anthrose biosynthesis	0	0	0	0	0	0	0.000722	0	0	0	0	0.000118316
Cell Wall and Capsule	Gram-Positive cell wall components	Teichoic and lipoteichoic acids biosynthesis	Minor teichoic acid biosynthesis protein GgaB	0	0	0	0	0	0.000487	0	0	0	0	0	0.000157755
Membrane Transport	NULL	Agrobacterium opine transport	MocE protein	0	0	0	0	0	0	0.000722	0	0	0	0	3.94388E-05
Cofactors, Vitamins, Prosthetic Groups, Pigments	Folate and pterines	Molybdenum cofactor biosynthesis	Molybdenum transport system protein ModD	0	0.015834	0	0	0	0	0	0	0	0	0	0.00031551
Carbohydrates	One-carbon Metabolism	Methanogenesis from methylated compounds	Monomethylamine permease	0	0	0	0	0	0.000487	0	0	0	0	0	0.000118316
Clustering-based subsystems	Carbohydrates	Predicted mycobacterial monooxygenase	Monooxygenase component C	0	0	0	0	0	0	0	0	0.001095	0	0	3.94388E-05
Clustering-based subsystems	Carbohydrates	Predicted mycobacterial monooxygenase	Monooxygenase, FAD- and [2Fe-2S]-containing component B	0	0	0	0	0	0	0.000722	0	0.000219	0	0	0
Motility and Chemotaxis	Flagellar motility in Prokaryota	Flagellar motility	Motility accessory factor	0	0	0	0	0.000657	0	0	0	0	0	0	0.00063102
Carbohydrates	Di- and oligosaccharides	Fructooligosaccharides(FOS) and Raffinose Utilization	MSM (multiple sugar metabolism) operon regulatory protein	0	0	0	0	0	0	0	0	0.000219	0	0	0.000828214
Virulence, Disease and Defense	Resistance to antibiotics and toxic compounds	Multiple Antibiotic Resistance MAR locus	Multiple antibiotic resistance protein MarC	0	0.002262	0	0	0	0	0	0	0	0	0	3.94388E-05
DNA Metabolism	DNA repair	DNA repair, bacterial MutL-MutS system	MutS domain protein, family 2	0	0	0	0.00249	0	0	0	0	0	0	0	0.000197194
DNA Metabolism	DNA repair	DNA repair, bacterial MutL-MutS system	MutS domain protein, family 6	0	0	0	0	0.000657	0	0	0	0	0	0	3.94388E-05
Carbohydrates	One-carbon Metabolism	Methanogenesis	N5-methyltetrahydromethanopterin:coenzyme M methyltransferase subunit D (EC 2.1.1.86)	0	0	0	0	0	0	0.000722	0	0	0	0	0.000197194
Carbohydrates	One-carbon Metabolism	Methanogenesis	N5-methyltetrahydromethanopterin:coenzyme M methyltransferase subunit E (EC 2.1.1.86)	0	0.004524	0	0	0	0	0	0	0	0	0	7.88775E-05
Miscellaneous	Plant-Prokaryote DOE project	At1g26220 At1g32070	N-acetyltransferase (GNAT) family, Syn7942 0773 homolog, Ycf52 protein	0	0	0	0	0	0.000974	0	0	0.001533	0	0	0
Regulation and Cell signaling	NULL	Sex pheromones in Enterococcus faecalis and other Firmicutes	NADH dehydrogenase (EC 1.6.99.3) in cluster with putative pheromone precursor	0	0	0	0	0	0	0	0.000498	0	0	0	0.000197194
Respiration	Electron donating reactions	Respiratory Complex I	NADH dehydrogenase subunit 3	0	0.002262	0	0	0	0	0	0	0	0	0	3.94388E-05
Virulence, Disease and Defense	NULL	Bacterial cyanide production and tolerance mechanisms	NADH peroxidase Npx (EC 1.11.1.1)	0	0	0	0	0	0	0	0	0	0	0.000116	0.000197194
Respiration	Electron donating reactions	Hydrogenases	NADH-reducing hydrogenase maturation factor	0	0.002262	0	0	0	0	0	0	0	0	0	7.88775E-05
Carbohydrates	Fermentation	Butanol Biosynthesis	NADPH-dependent butanol dehydrogenase (EC 1.1.1.-)	0	0	0	0	0	0	0	0	0.000438	0	0	0.000512704
Metabolism of Aromatic Compounds	Peripheral pathways for catabolism of aromatic compounds	Naphtalene and antracene degradation	naphthalene dioxygenase ferredoxin	0	0	0	0	0	0	0	0	0.000438	0	0	0.000118316
Motility and Chemotaxis	Flagellar motility in Prokaryota	Flagellum	Negative regulator of flagellin synthesis FlgM	0	0	0	0	0	0	0	0	0.001533	0	0	3.94388E-05
Membrane Transport	NULL	Transport of Nickel and Cobalt	Nickel transport ATP-binding protein NikD (TC 3.A.1.5.3)	0	0	0	0	0	0	0	0	0.000219	0	0	3.94388E-05
Respiration	Electron donating reactions	Hydrogenases	Nickel-dependent hydrogenase, small subunit	0	0	0	0	0	0.000974	0	0	0	0	0	0.000591581
Nitrogen Metabolism	NULL	Nitrogen fixation	NifB-domain protein, type 2	0	0	0	0	0	0.000487	0	0	0	0	0	7.88775E-05
Nitrogen Metabolism	NULL	Nitrogen fixation	Nitrogenase (vanadium-iron) alpha chain (EC 1.18.6.1)	0	0	0	0	0	0	0.000722	0	0	0	0	0.000157755
Nitrogen Metabolism	NULL	Nitrogen fixation	Nitrogenase vanadium-cofactor synthesis protein VnfN	0	0	0	0	0	0	0.000722	0	0	0	0	0.000157755
Cell Division and Cell Cycle	NULL	Heterocyst formation in cyanobacteria	Nitrogen-responsive response regulator NrrA	0	0	0	0	0	0	0	0	0	0	0.000349	3.94388E-05
Secondary Metabolism	Bacterial cytostatics, differentiation factors and antibiotics	Nonribosomal peptide synthetases (NRPS) in Frankia sp. Ccl3	NRPS loading module Thr-PG-PG-Thr	0	0	0	0	0.000657	0	0	0	0.000438	0	0	0
Nucleosides and Nucleotides	Detoxification	Nudix proteins (nucleoside triphosphate hydrolases)	Nudix dNTPase DR0329 (EC 3.6.1.-)	0	0.002262	0	0	0	0	0.002888	0	0	0	0	0
Nucleosides and Nucleotides	Detoxification	Nudix proteins (nucleoside triphosphate hydrolases)	Nudix hydrolase family protein YffH	0	0	0	0	0	0	0	0	0.000219	0	0	0.002050815
Carbohydrates	Central carbohydrate metabolism	Entner-Doudoroff Pathway	OpcA, an allosteric effector of glucose-6-phosphate dehydrogenase, cyanobacterial	0	0	0	0	0	0	0	0	0.002846	0	0.000349	0
Stress Response	Osmotic stress	Osmoprotectant ABC transporter YehZYXW of Enterobacteriales	Osmoprotectant ABC transporter binding protein YehZ	0	0	0	0	0	0	0	0	0.000219	0	0	3.94388E-05
Amino Acids and Derivatives	Lysine, threonine, methionine, and cysteine	Lysine degradation	Outer membrane porin, OprD family	0	0	0	0	0	0	0.001444	0	0	0	0	3.94388E-05
Virulence, Disease and Defense	Resistance to antibiotics and toxic compounds	MexA-MexB-OprM Multidrug Efflux System	Outer membrane protein OprM	0.000348	0	0	0	0	0	0	0	0	0	0	3.94388E-05
Respiration	Electron accepting reactions	Fe(III) respiration - Shewanella type	outer membrane protein, MtrB	0	0	0	0.00996	0	0	0	0	0	0	0	7.88775E-05
Iron acquisition and metabolism	Siderophores	Siderophore Pyoverdine	Outer membrane pyoverdine eflux protein	0	0	0	0	0	0	0	0	0.000219	0	0	3.94388E-05
Iron acquisition and metabolism	Siderophores	Siderophore Achromobactin	Outer membrane receptor proteins, likely involved in siderophore uptake	0	0	0	0	0	0	0	0	0.000219	0	0	7.88775E-05
Phosphorus Metabolism	NULL	Phosphate-binding DING proteins	Outer membrane TonB-dependent transducer VreA of trans-envelope signaling system	0	0	0	0	0.000657	0	0	0	0	0	0	3.94388E-05
Respiration	Sodium Ion-Coupled Energetics	Na+ translocating decarboxylases and related biotin-dependent enzymes	Oxaloacetate decarboxylase gamma chain (EC 4.1.1.3)	0	0	0	0	0	0	0	0	0.001314	0	0	0.000276071
Protein Metabolism	Protein biosynthesis	Nucleolar protein complex	p53 inducible protein	0	0	0	0	0	0	0	0	0.000657	0	0	0.000157755
RNA Metabolism	RNA processing and modification	Wyeosine-MimG Biosynthesis	PAB2272 methyltransferase homolog	0	0	0	0	0	0.000487	0	0	0	0	0	0.000157755
Cofactors, Vitamins, Prosthetic Groups, Pigments	Coenzyme A	Coenzyme A Biosynthesis	Pantothenate kinase type II, eukaryotic (EC 2.7.1.33)	0	0	0	0	0	0	0	0	0.000438	0	0	0.000197194

Miscellaneous	Plant-Prokaryote DOE project	Experimental-Ubiquinone BiosynthesisVDC	Para-hydroxybenzoate--polyprenyltransferase, mitochondrial precursor (EC 2.5.1.-)	0	0	0	0	0.000657	0	0	0	0	0	0	7.88775E-05
Carbohydrates	Central carbohydrate metabolism	Particulate methane monooxygenase (pMMO)	Particulate methane monooxygenase B-subunit (EC 1.14.13.25)	0.001393	0	0	0	0	0	0	0	0	0	0	0.000157755
Metabolism of Aromatic Compounds	NULL	p-cymene degradation	P-cymene monooxygenase, reductase subunit(CymAb) (EC 1.17.1.-)	0	0	0	0	0	0	0	0.000498	0	0	0	7.88775E-05
Dormancy and Sporulation	NULL	Spore Core Dehydration	Penicillin-binding protein DacC	0	0	0	0	0	0	0	0	0.000219	0	0	0.000354949
Membrane Transport	ABC transporters	ABC transporter peptide (TC 3.A.1.5.5)	Peptide transport periplasmic protein sapA (TC 3.A.1.5.5)	0	0	0	0	0	0	0	0	0.000438	0	0	0.000394388
Respiration	Electron accepting reactions	Fe(III) respiration - Shewanella type	periplasmic decaheme cytochrome c, MtrD	0	0	0	0	0	0.000974	0	0	0	0	0	0.001064846
Virulence, Disease and Defense	Resistance to antibiotics and toxic compounds	Mercury resistance operon	Periplasmic mercury(+2) binding protein	0	0	0	0	0	0	0	0	0	0	0.000349	3.94388E-05
Nitrogen Metabolism	NULL	Nitrate and nitrite ammonification	Periplasmic nitrate reductase component NapD	0	0	0	0	0	0.000487	0	0	0	0	0	0.000354949
Nitrogen Metabolism	NULL	Nitrate and nitrite ammonification	Periplasmic nitrate reductase component NapL	0	0	0	0	0	0.000487	0	0	0	0	0	0.000473265
Carbohydrates	NULL	Conserved cluster around inner membrane protein gene yghQ, probably involved in polysaccharide biosynthesis	Permease Ygh-P2, YjgP/YjgQ family	0	0	0	0	0	0.000487	0.000722	0	0	0	0	0
Phages, Prophages, Transposable elements, Plasmids	Phages, Prophages	Phage baseplate proteins	Phage baseplate wedge	0	0	0	0	0.000657	0	0	0	0	0.002203	0	0
Phages, Prophages, Transposable elements, Plasmids	Phages, Prophages	Phage capsid proteins	Phage capsid scaffolding protein	0	0	0	0	0	0	0	0	0.000657	0	0	0.000276071
Phages, Prophages, Transposable elements, Plasmids	Phages, Prophages	Phage packaging machinery	Phage DNA-binding protein	0	0	0	0	0	0.001461	0	0.002492	0	0	0	0
Clustering-based subsystems	Shiga toxin cluster	CBSS-194948.1.peg.143	Phage endopeptidase	0	0	0	0	0	0.000974	0	0	0	0	0	3.94388E-05
Phages, Prophages, Transposable elements, Plasmids	Phages, Prophages	Phage integration and excision	Phage exonuclease (EC 3.1.11.3)	0	0	0	0	0.000657	0	0.004333	0	0	0	0	0
Phages, Prophages, Transposable elements, Plasmids	Phages, Prophages	Phage capsid proteins	Phage head completion protein	0	0	0	0	0	0	0	0.001993	0	0	0	0.000118316
Phages, Prophages, Transposable elements, Plasmids	Phages, Prophages	Phage capsid proteins	Phage head completion-stabilization protein	0	0	0	0	0	0	0	0	0.000438	0	0	3.94388E-05
Clustering-based subsystems	NULL	Bacteriophage P4 cluster	Phage integrase, Phage P4-associated	0	0	0	0	0	0	0	0.000498	0	0	0	7.88775E-05
Phages, Prophages, Transposable elements, Plasmids	Phages, Prophages	Phage entry and exit	Phage lysozyme (EC 3.2.1.17)	0	0	0	0	0	0	0	0.000498	0	0	0	3.94388E-05
Phages, Prophages, Transposable elements, Plasmids	Phages, Prophages	Phage tail proteins 2	Phage major tail protein	0	0	0	0	0	0	0.000722	0	0	0	0	0.00031551
Phages, Prophages, Transposable elements, Plasmids	Phages, Prophages	Phage entry and exit	Phage maturase	0	0	0	0	0	0	0	0	0.000219	0	0.000233	0
Phages, Prophages, Transposable elements, Plasmids	Phages, Prophages	Phage nin genes - N-independent survival	Phage NinC	0	0	0	0	0	0	0	0.002492	0	0	0	0.000118316
Miscellaneous	NULL	ZZ gjo need homes	Phage-encoded chromosome degrading nuclease YokF	0	0.002262	0	0	0	0.000487	0	0	0	0	0	0
Secondary Metabolism	Bacterial cytostatics, differentiation factors and antibiotics	Phenazine biosynthesis	Phenazine modifying protein PhzH	0	0	0	0.00249	0	0	0.000722	0	0	0	0	0
Metabolism of Aromatic Compounds	Peripheral pathways for catabolism of aromatic compounds	Phenol hydroxylase	Phenol hydroxylase, P4 oxygenase component DmpO (EC 1.14.13.7)	0	0	0	0	0	0	0	0	0.000438	0	0	0.00063102
Fatty Acids, Lipids, and Isoprenoids	Isoprenoids	Carotenoids	phi-Carotenoid synthase (EC 1.3.-.- and EC 2.1.1-)	0	0	0	0	0	0	0	0	0.000876	0	0	0.000197194
Carbohydrates	Sugar alcohols	Ethanolamine utilization	Phosphate acetyltransferase (EC 2.3.1.8), ethanolamine utilization-specific	0	0	0	0	0	0	0	0	0.000219	0	0	0.000157755
Miscellaneous	NULL	ZZ gjo need homes	Phosphatidyl-N-methylethanolamine N-methyltransferase (EC 2.1.1.71)	0	0	0	0	0	0	0	0	0.000219	0	0	0.000157755
Carbohydrates	Central carbohydrate metabolism	Dihydroxyacetone kinases	Phosphoenolpyruvate-dihydroxyacetone phosphotransferase operon regulatory protein DhaR	0	0	0	0.00498	0	0	0	0	0	0	0	0.000197194
Membrane Transport	NULL	Phosphoglycerate transport system	Phosphoglycerate transport regulatory protein PgtC	0	0.002262	0	0	0	0	0	0	0	0	0	7.88775E-05
Fatty Acids, Lipids, and Isoprenoids	Isoprenoids	Archaeal lipids	Phosphomevalonate kinase (EC 2.7.4.2)	0	0	0	0	0.000657	0	0	0	0	0	0	0.000118316
Amino Acids and Derivatives	NULL	Phosphonoalanine utilization	Phosphonoalanine and/or phosphonopyruvate ABC transporter ATP-binding protein	0	0	0	0	0	0	0	0.000498	0	0	0	7.88775E-05
Photosynthesis	Electron transport and photophosphorylation	Photosystem I	photosystem I subunit XI (PsaL)	0	0.004524	0	0	0	0	0	0	0.001095	0	0	0
Photosynthesis	Electron transport and photophosphorylation	Photosystem II	Photosystem II protein Psb27	0	0	0	0	0	0	0	0	0.000438	0	0.000466	0
Photosynthesis	Electron transport and photophosphorylation	Photosystem II	Photosystem II protein PsbN	0	0	0	0	0	0	0	0.000498	0.000438	0	0	0
Photosynthesis	Electron transport and photophosphorylation	Photosystem II	Photosystem II protein PsbT	0	0	0	0	0	0	0	0	0.000219	0	0	7.88775E-05
Photosynthesis	Electron transport and photophosphorylation	Photosystem II	Photosystem II stability/assembly factor HCF136/Ycf48	0	0	0	0	0	0	0	0	0.000657	0	0.000582	0
Photosynthesis	Light-harvesting complexes	Phycobilisome	Phycobilisome core component	0	0	0	0	0	0	0	0.000498	0.001533	0	0	0
Photosynthesis	Light-harvesting complexes	Phycobilisome	Phycobilisome phycoerythrin-associated linker polypeptide	0	0	0	0	0	0	0	0	0.000438	0	0.000349	0
Photosynthesis	Light-harvesting complexes	Phycobilisome	Phycocyanin beta chain	0	0	0	0	0	0.000487	0	0	0	0	0.000233	0
Photosynthesis	Light-harvesting complexes	Phycobilisome	Phycoerythrin linker protein CpeS homolog	0	0	0	0	0	0.000974	0	0	0.001095	0	0	0
Cofactors, Vitamins, Prosthetic Groups, Pigments	Biotin	Biotin biosynthesis	Pimeloyl-CoA synthase (EC 6.2.1.14)	0	0	0	0	0.000657	0	0	0	0	0	0	7.88775E-05
Iron acquisition and metabolism	Siderophores	Siderophore Pyoverdine	Pirin-related protein, coexpressed with pyoverdine biosynthesis regulon	0	0.002262	0	0	0	0	0	0	0.000219	0	0	0
DNA Metabolism	DNA repair	DNA repair, bacterial UvrD and related helicases	Plasmid conjugative transfer DNA helicase TrhI	0	0	0	0	0	0.000487	0	0	0	0.004406	0	0
Regulation and Cell signaling	Proteolytic pathway	Coagulation cascade	Plasminogen activator inhibitor-1, protease inhibitor I04.020	0	0	0	0	0	0	0	0	0.000438	0	0	0.000709898
Virulence, Disease and Defense	Resistance to antibiotics and toxic compounds	Polymyxin Synthetase Gene Cluster in Bacillus	Polymyxin transporter PmxC	0	0	0	0	0	0.000487	0	0	0	0	0	3.94388E-05
Respiration	Electron accepting reactions	Anaerobic respiratory reductases	polysulfide reductase, subunit C	0	0	0	0	0.000657	0	0	0	0	0	0	0.000433826
Carbohydrates	Monosaccharides	D-gluconate and ketogluconates metabolism	Positive regulator of L-idonate catabolism	0	0	0	0	0	0.000487	0	0	0	0	0	0.000118316
Metabolism of Aromatic Compounds	Metabolism of central aromatic intermediates	Homogentisate pathway of aromatic compound degradation	Possible 3-(3-hydroxy-phenyl)propionate hydroxylase (EC 1.14.13.-)	0	0	0	0	0	0.000487	0	0.000498	0	0	0	0
Membrane Transport	ABC transporters	ABC transporter of unknown substrate X	Possible ABC transporter, periplasmic substrate X binding protein precursor	0	0	0	0.00249	0.000657	0	0	0	0	0	0	0
Miscellaneous	Plant-Prokaryote DOE project	Synechocystis experimental	Possible alpha/beta hydrolase superfamily, sll1129 homolog	0	0	0	0	0	0	0	0	0.000876	0	0.000233	0
Miscellaneous	Plant-Prokaryote DOE project	Synechocystis experimental	Possible alpha/beta hydrolase superfamily, slr1917 homolog	0	0	0	0	0	0	0	0	0.000657	0	0.000233	0
Carbohydrates	Monosaccharides	L-fucose utilization	Possible alternative L-fucose mutarotase	0	0	0	0	0	0	0	0	0.001095	0	0	0.000354949
Iron acquisition and metabolism	NULL	Campylobacter Iron Metabolism	Possible bacterioferritin	0	0.002262	0	0	0	0	0	0	0	0	0	0.000118316
Nucleosides and Nucleotides	Pyrimidines	Pyrimidine utilization	Possible ring-opening amidohydrolase RutC in novel pyrimidine catabolism pathway	0	0	0	0	0	0	0.000722	0	0	0	0	3.94388E-05

Carbohydrates	CO2 fixation	CO2 uptake, carboxysome	Possible RuBisCo chaperonin RbcX	0	0	0	0	0	0.000974	0	0	0.000657	0	0	0
Potassium metabolism	NULL	Potassium homeostasis	POTASSIUM/PROTON ANTIPORTER ROSB	0	0	0	0	0	0	0.001444	0.000498	0	0	0	0
Regulation and Cell signaling	NULL	Pseudomonas quinolone signal PQS	PQS biosynthesis protein PqsA, anthranilate-CoA ligase (EC 6.2.1.32)	0	0	0	0	0	0.000487	0	0	0	0	0	3.94388E-05
Regulation and Cell signaling	NULL	Pseudomonas quinolone signal PQS	PQS biosynthesis protein PqsD, similar to 3-oxoacyl-[acyl-carrier-protein] synthase III	0	0	0.00511	0	0	0	0	0	0	0	0	0.000276071
Cofactors, Vitamins, Prosthetic Groups, Pigments	Tetrapyrroles	Coenzyme B12 biosynthesis	Predicted adenosylcobinamide-phosphate guanylyltransferase CobY (EC 2.7.7.62)	0	0	0	0	0	0	0.002166	0	0	0	0	7.88775E-05
Stress Response	Oxidative stress	Glutathione: Biosynthesis and gamma-glutamyl cycle	Predicted alternative glutathione synthetase (EC 6.3.2.3)	0	0	0	0	0	0	0.000722	0	0	0	0	7.88775E-05
Carbohydrates	Di- and oligosaccharides	Beta-Glucoside Metabolism	Predicted cellobiose ABC transport system, ATP-binding protein 1	0	0	0	0	0.000657	0	0	0	0	0	0	0.00031551
Membrane Transport	NULL	Transport of Nickel and Cobalt	Predicted cobalt transporter in Bacteroides Porphyromonas	0.011489	0	0	0	0	0	0	0	0	0	0	0.006586272
Carbohydrates	Sugar alcohols	Erythritol utilization	Predicted erythritol ABC transporter 1, ATP-binding component 1	0	0	0	0	0	0.000974	0.000722	0	0	0	0	0
Carbohydrates	Sugar alcohols	Erythritol utilization	Predicted erythritol ABC transporter 1, ATP-binding component 2	0	0	0	0	0	0	0	0	0.000657	0	0	3.94388E-05
Nucleosides and Nucleotides	Pyrimidines	Pyrimidine utilization	Predicted flavin reductase RutF in novel pyrimidine catabolism pathway	0	0	0	0	0	0	0	0.000498	0.000219	0	0	0
Carbohydrates	Di- and oligosaccharides	Lactose and Galactose Uptake and Utilization	Predicted galactoside ABC transporter, ATP-binding protein 2	0	0	0	0	0	0	0	0	0	0	0.000116	0.000118316
Carbohydrates	Di- and oligosaccharides	Lactose and Galactose Uptake and Utilization	Predicted galactoside ABC transporter, permease protein 2	0	0	0	0	0	0	0	0	0.000438	0	0	0.000197194
Miscellaneous	Plant-Prokaryote DOE project	COG3533	Predicted L-arabinose ABC transport system, permease protein 1	0	0	0	0	0	0	0	0	0.000657	0	0	7.88775E-05
Nucleosides and Nucleotides	Pyrimidines	Pyrimidine utilization	Predicted monooxygenase RutA in novel pyrimidine catabolism pathway	0	0	0	0	0	0	0	0	0.000876	0	0	0.000394388
Clustering-based subsystems	NULL	Divergent RNA modification related clusters	Predicted nucleoside phosphatase	0	0	0	0	0	0	0	0.000498	0	0	0	3.94388E-05
Membrane Transport	Protein secretion system, Type II	Predicted secretion system W clustering with cell division proteins	Predicted secretion system W protein GspF-like	0	0	0.002555	0	0	0	0	0	0	0	0	0.00031551
Membrane Transport	Protein secretion system, Type II	Predicted secretion system X	Predicted secretion system X protein GspG-like 2	0	0	0	0	0	0	0	0	0.000438	0	0	0.000236633
Carbohydrates	Di- and oligosaccharides	Sucrose utilization Shewanella	Predicted sucrose permease, MFS family, FucP subfamily	0	0	0	0	0	0	0	0	0	0	0.0085	0.002248009
Amino Acids and Derivatives	Branched-chain amino acids	Branched chain amino acid degradation regulons	Predicted transcriptional regulator LiuX of leucine degradation pathway, AcrR family	0	0	0	0	0	0	0	0	0.000657	0	0	3.94388E-05
Amino Acids and Derivatives	Branched-chain amino acids	Valine degradation	Predicted transcriptional regulator of leucine degradation pathway, MerR family	0	0	0	0	0	0.000487	0	0	0	0	0	0.00063102
Respiration	NULL	Biogenesis of c-type cytochromes	Predicted transporter accosiated with cytochrome c biogenesis	0	0	0	0	0	0	0	0	0.000219	0	0	0.000197194
RNA Metabolism	RNA processing and modification	rRNA modification Archaea	Predicted Zn-ribbon RNA-binding protein with a function in translation	0	0	0	0	0	0	0.000722	0	0.000219	0	0	0
Virulence, Disease and Defense	Resistance to antibiotics and toxic compounds	Cobalt-zinc-cadmium resistance	Probable cadmium-transporting ATPase (EC 3.6.3.3)	0	0	0	0	0	0.000974	0	0	0	0	0	0.000433826
Cell Wall and Capsule	Cell wall of Mycobacteria	mycolic acid synthesis	PROBABLE DAUNORUBICIN-DIM-TRANSPORT ATP-BINDING PROTEIN ABC TRANSPORTER DRRA	0	0	0	0	0	0.000974	0	0	0	0	0	0.00031551
Metabolism of Aromatic Compounds	Peripheral pathways for catabolism of aromatic compounds	Phenylpropanoid compound degradation	Probable FERULOYL-CoA synthetase protein	0	0	0	0	0	0.001461	0	0	0.000438	0	0	0
Respiration	Electron donating reactions	Hydrogenases	Probable Ni/Fe-hydrogenase 2 B-type cytochrome subunit	0	0	0	0	0	0.001461	0	0	0	0	0	0.000433826
Clustering-based subsystems	NULL	CBSS-83332.1.peg.3803	Probable polyketide synthase, similar to many. e.g. gp|M63676|SERERYAA 1 S.erythraea first ORF of eryA gene, involved in complex polyketide formation in erythromycin biosynthesis.	0	0	0	0	0	0	0	0	0	0.002203	0	0.000591581
RNA Metabolism	RNA processing and modification	RNA methylation	Probable RNA methyltransferase PA1839	0	0.002262	0	0	0	0	0	0	0	0	0	0.000354949
Virulence, Disease and Defense	Resistance to antibiotics and toxic compounds	Multidrug Resistance Efflux Pumps	Probable transcription regulator protein of MDR efflux pump cluster	0	0	0	0	0	0	0	0	0.000219	0	0	0.000276071
Miscellaneous	NULL	ZZ gjo need homes	probably not quinone oxidoreductase	0	0	0	0	0	0	0.001444	0	0	0	0	3.94388E-05
Regulation and Cell signaling	Programmed Cell Death and Toxin-antitoxin Systems	MazEF toxin-antitoxing (programmed cell death) system	Programmed cell death antitoxin PemI	0.000348	0	0	0	0	0	0	0	0.000219	0	0	0
Regulation and Cell signaling	Programmed Cell Death and Toxin-antitoxin Systems	MazEF toxin-antitoxing (programmed cell death) system	Programmed cell death toxin PemK	0	0	0.002555	0	0	0	0	0	0	0	0	7.88775E-05
Carbohydrates	Sugar alcohols	Propanediol utilization	Propanediol utilization polyhedral body protein PduB	0	0	0	0	0	0	0	0	0	0	0.000582	0.000276071
Carbohydrates	Sugar alcohols	Propanediol utilization	Propanediol utilization polyhedral body protein PduN	0	0.002262	0	0	0.000657	0	0	0	0	0	0	0
Amino Acids and Derivatives	Lysine, threonine, methionine, and cysteine	Threonine anaerobic catabolism gene cluster	Propionate kinase (EC 2.7.2.15)	0	0	0	0	0	0	0	0	0.000219	0	0	7.88775E-05
Protein Metabolism	Protein degradation	Proteasome eukaryotic	proteasome regulatory subunit Rpn2	0	0	0	0	0	0	0	0	0.000219	0	0	3.94388E-05
Protein Metabolism	Protein degradation	Proteasome eukaryotic	proteasome regulatory subunit Rpn6	0	0	0	0	0	0	0	0	0	0	0.000116	7.88775E-05
Protein Metabolism	Protein degradation	Proteasome eukaryotic	proteasome regulatory subunit Rpn8	0	0	0	0	0	0.000974	0	0	0	0	0.000116	0
Protein Metabolism	Protein degradation	Proteasome eukaryotic	proteasome subunit alpha1 (EC 3.4.25.1)	0	0.002262	0	0	0	0	0	0	0	0	0	0.000236633
Protein Metabolism	Protein degradation	Proteasome eukaryotic	proteasome subunit alpha6 (EC 3.4.25.1)	0	0	0	0	0	0	0	0.000997	0	0	0	7.88775E-05
Protein Metabolism	Protein degradation	Proteasome eukaryotic	proteasome subunit beta2 (EC 3.4.25.1)	0	0	0	0	0	0	0	0	0	0	0.000116	7.88775E-05
Miscellaneous	Plant-Prokaryote DOE project	COG3533	Protein AraJ precursor	0	0	0	0	0	0	0	0	0	0.004406	0	0.000197194
Phages, Prophages, Transposable elements, Plasmids	Phages, Prophages	Listeria phi-A118-like prophages	Protein gp49, replication initiation [Bacteriophage A118]	0	0	0	0	0.000657	0.000487	0	0	0	0	0	0
Clustering-based subsystems	Pyruvate kinase associated cluster	CBSS-288000.5.peg.1793	protein of unknown function DUF1036	0	0	0	0.00249	0	0	0	0	0	0	0	3.94388E-05
Clustering-based subsystems	Pyruvate kinase associated cluster	CBSS-288000.5.peg.1793	protein of unknown function DUF882	0	0	0	0	0	0	0	0	0.000657	0	0	0.000433826
Photosynthesis	Light-harvesting complexes	Bacterial light-harvesting proteins	Protein PufQ, involved in assembly of B875 and B800-850 pigment-protein complexes	0	0	0	0	0	0.000974	0	0.001495	0	0	0	0
Clustering-based subsystems	NULL	CBSS-316273.3.peg.227	Protein SseB	0	0	0	0	0	0	0	0	0.000219	0	0	3.94388E-05
Carbohydrates	Di- and oligosaccharides	Maltose and Maltodextrin Utilization	PTS system, maltose and glucose-specific IIB component (EC 2.7.1.69)	0	0	0	0	0	0	0	0.000498	0	0	0	3.94388E-05
Carbohydrates	Di- and oligosaccharides	Sucrose utilization	PTS system, sucrose-specific IIB component (EC 2.7.1.69)	0	0	0	0	0	0	0	0	0	0	0.000116	3.94388E-05
Carbohydrates	Di- and oligosaccharides	Sucrose utilization	PTS system, sucrose-specific IIC component (EC 2.7.1.69)	0	0	0	0	0	0	0	0	0	0	0.000116	0.000118316
Miscellaneous	Plant-Prokaryote DOE project	DOE COG3533	Putative beta-galactosidase in 4-hydroxyproline catabolic gene cluster	0	0	0	0	0	0	0	0	0.000219	0	0	0.000157755
Cell Wall and Capsule	Capsular and extracellular polysacchrides	Capsular Polysaccharides Biosynthesis and Assembly	Putative capsular polysaccharide transport protein YegH	0	0	0	0	0	0	0	0	0.000219	0	0	0.000236633
Cell Wall and Capsule	Gram-Positive cell wall components	Teichoic and lipoteichoic acids biosynthesis	Putative CDP-glycosylpolyol phosphate:glycosylpolyol	0	0	0	0.00249	0	0	0	0	0	0	0	3.94388E-05

			glycosylpolyolphosphotransferase												
Phages, Prophages, Transposable elements, Plasmids	Transposable elements	Conjugative transposon, Bacteroidales	Putative conjugative transposon mobilization protein BF0132	0	0	0	0	0	0	0.000722	0	0	0	0	3.94388E-05
Potassium metabolism	NULL	Potassium homeostasis	Putative cytoplasmic protein ,probably associated with Glutathione-regulated potassium-efflux	0	0	0	0	0	0	0	0	0.000438	0	0	0.000118316
Clustering-based subsystems	Choline bitartrate degradation, putative	CBSS-344610.3.peg.2335	Putative dioxygenase, alpha subunit (EC 1.-.-.-)	0	0	0	0	0	0	0	0	0.000438	0	0	0.000157755
Iron acquisition and metabolism	Siderophores	Siderophore Pyoverdine	Putative dipeptidase, pyoverdin biosynthesis PvdM	0	0	0	0	0	0.000487	0	0	0	0	0	0.000512704
Clustering-based subsystems	NULL	CBSS-83333.1.peg.946	Putative efflux (PET) family inner membrane protein YccS	0	0	0	0	0	0	0	0	0.000219	0	0	7.88775E-05
Respiration	Electron donating reactions	Respiratory dehydrogenases 1	putative Fe-S, FMN containing oxidoreductase	0.002437	0.006786	0	0	0	0	0	0	0	0	0	0
Virulence, Disease and Defense	Adhesion	Adhesion of Campylobacter	Putative fibronectin domain-containing lipoprotein	0	0	0	0	0	0.000487	0	0	0	0	0	0.002484642
Clustering-based subsystems	NULL	Putative hemin transporter	Putative hemin-binding lipoprotein	0	0	0	0	0	0	0	0	0.000657	0	0	0.00031551
Clustering-based subsystems	NULL	Ycd cluster (putative 2-hydroxyacid dehydrogenase)	Putative hydrolase YcdX (EC 3.1.-.-)	0	0	0	0	0	0.000487	0	0	0	0	0	3.94388E-05
Carbohydrates	Sugar alcohols	Propanediol utilization	Putative iron-containing NADPH-dependent propanol dehydrogenase	0	0	0	0	0	0	0	0	0.000219	0	0	7.88775E-05
Miscellaneous	Plant-Prokaryote DOE project	At2g44920 At1g12250	Putative lumenal protein, contains 8 pentapeptide repeats, sll0301 homolog	0	0	0	0	0	0	0	0.000997	0.000219	0	0	0
Cell Wall and Capsule	Gram-Positive cell wall components	Teichoic and lipoteichoic acids biosynthesis	Putative major teichoic acid biosynthesis protein C	0	0	0	0	0	0	0	0	0.000219	0	0	3.94388E-05
Stress Response	Periplasmic Stress	Periplasmic Acid Stress Response in Enterobacteria	Putative membrane transporter ATPase,YhiD	0	0	0	0	0	0	0	0	0.000219	0	0	0.000118316
Regulation and Cell signaling	NULL	Pseudomonas quinolone signal PQS	Putative non-ribosomal peptide synthetase in AHQ biosynthetic operon	0	0	0	0	0	0	0.000722	0	0.000438	0	0	0
Cell Wall and Capsule	Capsular and extracellular polysacchrides	Capsular Polysaccharides Biosynthesis and Assembly	Putative outer membrane lipoprotein YmcA	0	0	0	0	0	0	0	0	0.000438	0	0	7.88775E-05
Carbohydrates	Di- and oligosaccharides	Maltose and Maltodextrin Utilization	Putative oxidoreductase YcjS (EC 1.-.-.-), NADH-binding	0	0	0	0	0	0	0.000722	0	0	0	0	0.000157755
Dormancy and Sporulation	NULL	Spore germination	Putative peptidoglycan hydrolase YvbX, NOT involved in spore germination	0	0	0	0	0	0	0	0	0.000219	0	0	3.94388E-05
Phosphorus Metabolism	NULL	High affinity phosphate transporter and control of PHO regulon	Putative periplasmic phosphate-binding protein PstS (Halobacteriales type)	0	0	0	0	0	0	0	0	0.001095	0	0	3.94388E-05
Phages, Prophages, Transposable elements, Plasmids	Phages, Prophages	Phage integration and excision	Putative phage-encoded enzyme involved in integration-recombination	0	0	0	0	0	0	0.000722	0	0	0	0	7.88775E-05
Metabolism of Aromatic Compounds	Peripheral pathways for catabolism of aromatic compounds	Phenylpropanoid compound degradation	Putative phthalate 4,5-dioxygenase reductase subunit (OhpA1)	0	0	0	0	0.000657	0	0	0	0	0	0	7.88775E-05
Miscellaneous	Plant-Prokaryote DOE project	At5g38900	Putative sodium:solute symporter, similarity with yeast urea transporter DUR3	0	0	0	0	0	0	0.000722	0	0.000219	0	0	0
Clustering-based subsystems	Carbohydrates	Cluster Ytf and putative sugar transporter	Putative sugar transporter	0	0	0	0	0	0	0	0	0.000219	0	0	0.000118316
Amino Acids and Derivatives	Arginine; urea cycle, polyamines	Putrescine utilization pathways	Putative symporter in putrescine utilization cluster	0	0	0	0	0	0	0	0.000498	0	0	0	0.00220857
Miscellaneous	Plant-Prokaryote DOE project	At2g44920 At1g12250	Putative thylakoid membrane protein, contains 8 pentapeptide repeats, sll0274 homolog	0	0	0	0	0.000657	0	0	0	0	0	0	7.88775E-05
Clustering-based subsystems	NULL	CBSS-312309.3.peg.1965	putative; UPF0325 protein yaeH	0	0	0	0	0	0	0	0	0.001314	0	0	0.000433826
Secondary Metabolism	Bacterial cytostatics, differentiation factors and antibiotics	Paerucumarin Biosynthesis	PvcA protein, related to known isonitrile synthases	0	0	0	0	0	0	0	0.000498	0	0	0	0.000236633
Iron acquisition and metabolism	Siderophores	Siderophore Pyoverdine	PvdE, pyoverdine ABC export system, fused ATPase and permease components	0	0	0	0	0	0	0	0	0.000657	0	0	0.000118316
Protein Metabolism	Protein biosynthesis	Pyrrolysine	Pyrrolysine synthetase	0	0.002262	0	0	0	0	0	0	0	0	0	0.000276071
Cofactors, Vitamins, Prosthetic Groups, Pigments	Tetrapyrroles	Heme and Siroheme Biosynthesis	Radical SAM family protein HutW, similar to coproporphyrinogen III oxidase, oxygen-independent, associated with heme uptake	0	0	0	0	0	0.000487	0	0	0	0	0.000233	0
Membrane Transport	Protein translocation across cytoplasmic membrane	ESAT-6 proteins secretion system in Actinobacteria	RD1 region associated protein Rv3879c	0	0	0	0	0	0	0	0	0.001314	0	0	3.94388E-05
Cofactors, Vitamins, Prosthetic Groups, Pigments	Tetrapyrroles	Chlorophyll Degradation	Red chlorophyll catabolite reductase (EC 1.-.-.-)	0	0	0.002555	0	0	0	0	0	0	0	0	3.94388E-05
Cell Wall and Capsule	Gram-Negative cell wall components	KDO2-Lipid A biosynthesis	regulator of length of O-antigen component of lipopolysaccharide chains	0	0	0	0	0	0	0	0	0.000219	0	0	7.88775E-05
Virulence, Disease and Defense	Resistance to antibiotics and toxic compounds	Beta-lactamase	Regulatory protein BlaR1	0	0	0	0	0	0.000487	0	0.000498	0	0	0	0
Carbohydrates	Polysaccharides	Cellulosome	Regulatory protein SusR	0	0	0	0	0	0	0.000722	0	0	0	0	0.000157755
Regulation and Cell signaling	Regulation of virulence	Streptococcus pyogenes virulence regulators	Response regulator CsrR	0	0	0	0	0	0	0	0	0.000438	0	0	3.94388E-05
RNA Metabolism	Transcription	Transcription factors bacterial	Rho-specific inhibitor of transcription termination (YaeO)	0	0	0.002555	0	0	0	0	0	0	0	0	3.94388E-05
Protein Metabolism	Protein biosynthesis	Ribosome biogenesis bacterial	Ribonuclease M5 (EC 3.1.26.8)	0	0	0	0.00498	0	0	0	0	0	0	0	0.000157755
Clustering-based subsystems	Proteasome related clusters	Proteasome subunit alpha archaeal cluster	Ribonuclease P protein component 3 (EC 3.1.26.5)	0	0	0	0	0	0	0	0.001495	0.000219	0	0	0
Nucleosides and Nucleotides	NULL	Ribonucleotide reduction	Ribonucleotide reduction protein NrdI	0	0	0	0	0.000657	0	0	0	0	0.004406	0	0
Carbohydrates	Monosaccharides	D-ribose utilization	Ribose/xylose/arabinose/galactoside ABC-type transport systems, ATP-binding protein (EC 3.6.3.17)	0	0	0	0	0	0	0	0	0.000219	0	0	0.000118316
Carbohydrates	Monosaccharides	D-ribose utilization	Ribose/xylose/arabinose/galactoside ABC-type transport systems, permease component 2	0	0	0.002555	0	0	0	0	0	0	0	0	0.000157755
RNA Metabolism	RNA processing and modification	rRNA modification Bacteria	Ribosomal large subunit pseudouridine synthase E (EC 5.4.99.-)	0	0	0	0	0	0	0.000722	0	0	0	0	0.000552143
Carbohydrates	CO2 fixation	CO2 uptake, carboxysome	ribulose 1,5-bisphosphate carboxylase/oxygenase activase	0	0	0	0	0	0	0	0	0.000438	0	0.000116	0
RNA Metabolism	Transcription	Transcription initiation, bacterial sigma factors	RNA polymerase sigma factor RpoH-related protein RpoH2	0	0	0	0	0	0	0	0	0	0.002203	0	7.88775E-05
Dormancy and Sporulation	NULL	Sporulation gene orphans	RNA polymerase sporulation specific sigma factor SigE	0	0	0	0	0	0	0	0	0.000219	0	0	3.94388E-05
Clustering-based subsystems	NULL	Cell division-ribosomal stress proteins cluster	S4-domain-containing heat shock protein	0	0	0	0	0	0	0	0	0.000438	0	0	0.000197194
Metabolism of Aromatic Compounds	Peripheral pathways for catabolism of aromatic compounds	Naphtalene and antracene degradation	Salicylaldehyde dehydrogenase (EC 1.2.1.65)	0	0	0	0.00249	0	0	0	0	0	0	0	0.000197194
Protein Metabolism	Selenoproteins	Glycine reductase, sarcosine reductase and betaine reductase	Sarcosine reductase component B beta subunit (EC 1.21.4.3)	0	0	0	0	0	0	0	0	0.000219	0	0	3.94388E-05
Virulence, Disease and Defense	Resistance to antibiotics and toxic compounds	Copper homeostasis: copper tolerance	Secreted protein, suppressor for copper-sensitivity ScsC	0	0	0	0	0	0	0	0	0.001533	0	0	0.000157755
Iron acquisition and metabolism	NULL	Heme, hemin uptake and utilization systems in GramPositives	Sensor histidine kinase colocalized with HrtAB transporter	0	0	0	0	0	0	0	0	0.000438	0	0	0.000276071
Virulence, Disease and Defense	Resistance to antibiotics and toxic compounds	Resistance to Vancomycin	Sensor histidine kinase VanS (EC 2.7.3.-)	0	0	0	0	0	0	0	0	0.000438	0	0	0.000118316
Miscellaneous	Plant-Prokaryote DOE project	At4g38090	Sensor protein DegS	0	0	0	0	0	0	0	0	0.000438	0	0	7.88775E-05
Regulation and Cell signaling	Regulation of virulence	VieSAB signal transduction system of Vibrio	Sensory box sensor histidine kinase/response regulator VieS	0	0	0	0	0	0	0.000722	0	0	0	0	0.001104285

Fatty Acids, Lipids, and Isoprenoids	Phospholipids	Sphingolipid biosynthesis	Serine palmitoyltransferase (EC 2.3.1.50)	0	0	0	0	0	0	0	0	0	0	0.000582	0.000197194
Cell Wall and Capsule	Capsular and extracellular polysacchrides	CMP-N-acetylneuraminate Biosynthesis	Sialic acid biosynthesis protein NeuD, O-acetyltransferase	0	0	0	0	0.000657	0	0	0	0	0	0	0.000197194
Cell Wall and Capsule	Capsular and extracellular polysacchrides	Sialic Acid Metabolism	Sialic acid-induced transmembrane protein YjhT(NanM), possible mutarotase	0	0	0	0	0	0.000487	0	0	0	0	0	0.00031551
Iron acquisition and metabolism	Siderophores	Siderophore assembly kit	Siderophore biosynthesis protein, monooxygenase	0	0	0	0	0	0	0	0	0	0	0.000116	3.94388E-05
Regulation and Cell signaling	Quorum sensing and biofilm formation	Symbiotic colonization and sigma-dependent biofilm formation gene cluster	Sigma-54 dependent transcriptional regulator SypG	0	0	0	0	0	0	0	0	0.000219	0	0	0.000394388
Membrane Transport	Protein secretion system, Type VII (Chaperone/Usher pathway, CU)	sigma-Fimbriae	Sigma-fimbriae chaperone protein	0	0	0	0	0	0	0	0	0.000219	0	0	3.94388E-05
Membrane Transport	Protein secretion system, Type VII (Chaperone/Usher pathway, CU)	sigma-Fimbriae	Sigma-fimbriae usher protein	0	0	0	0	0	0	0	0	0.000219	0	0.000116	0
Motility and Chemotaxis	Flagellar motility in Prokaryota	Flagellar motility	Signal transduction protein CetA, mediates an energy taxis response	0	0	0	0	0	0	0	0	0.000438	0	0	0.003115662
Amino Acids and Derivatives	Arginine; urea cycle, polyamines	Putrescine utilization pathways	Similar to gamma-glutamyl-putrescine oxidase	0	0	0	0	0	0	0	0	0.000438	0	0	7.88775E-05
Miscellaneous	Plant-Prokaryote DOE project	Synechocystis experimental	Similar to non-heme chloroperoxidase	0	0	0	0	0	0	0	0	0.00416	0	0	0.000433826
Protein Metabolism	Protein biosynthesis	Ribosome biogenesis bacterial	Similar to ribosomal large subunit pseudouridine synthase D, CAC1266-type	0	0	0	0	0	0	0	0	0.000438	0	0	0.000276071
Protein Metabolism	Protein biosynthesis	Ribosome biogenesis bacterial	Similar to ribosomal large subunit pseudouridine synthase D, TTE1780-type	0	0	0	0	0	0.000974	0	0	0	0	0	3.94388E-05
Phages, Prophages, Transposable elements, Plasmids	Phages, Prophages	Phage replication	Single stranded DNA-binding protein, phage-associated	0	0	0	0.00498	0	0	0	0.000498	0	0	0	0
Membrane Transport	NULL	Choline Transport	Sodium-Choline Symporter	0	0	0	0	0	0	0	0.000498	0	0	0	0.00031551
Respiration	Electron donating reactions	Succinate dehydrogenase	soluble fumarate reductase, cytoplasmic	0	0	0	0	0.000657	0	0	0	0	0	0	0.000118316
Amino Acids and Derivatives	Arginine; urea cycle, polyamines	Polyamine Metabolism	Spermine synthase (EC 2.5.1.22)	0	0	0	0	0	0	0	0	0.000219	0	0	7.88775E-05
Dormancy and Sporulation	NULL	Spore germination	Spore cortex-lytic enzyme, lytic transglycosylase SleB	0	0.002262	0	0	0	0	0	0	0	0	0	0.000236633
Dormancy and Sporulation	NULL	Sporulation draft	Sporulation kinase A (EC 2.7.13.3)	0	0	0	0	0	0	0	0	0.000219	0	0	7.88775E-05
Dormancy and Sporulation	NULL	Sporulation draft	Sporulation kinase B homolog 1	0	0	0	0	0	0.000487	0	0	0.000438	0	0	0
Protein Metabolism	Protein biosynthesis	Ribosome SSU eukaryotic and archaeal	SSU ribosomal protein S24e	0	0	0	0	0	0	0	0	0.000219	0	0	3.94388E-05
Protein Metabolism	Protein biosynthesis	Ribosome SSU eukaryotic and archaeal	SSU ribosomal protein S26e	0	0	0	0	0	0	0.001444	0.000498	0	0	0	0
Dormancy and Sporulation	NULL	Sporulation draft	Stage 0 sporulation two-component response regulator (Spo0A)	0	0	0	0	0	0	0	0	0.000219	0	0	0.000118316
Dormancy and Sporulation	NULL	Sporulation Cluster	Stage II sporulation serine phosphatase for sigma-F activation (SpoIIE)	0	0	0	0	0	0	0	0	0.000876	0	0	3.94388E-05
Dormancy and Sporulation	NULL	Sporulation gene orphans	Stage IV sporulation protein A	0	0	0	0	0	0	0	0	0	0.002203	0	7.88775E-05
Dormancy and Sporulation	NULL	Sporulation gene orphans	Stage V sporulation protein AE (SpoVAE)	0	0	0	0	0	0	0	0	0.000438	0	0	7.88775E-05
Regulation and Cell signaling	NULL	Staphylococcal accessory gene regulator system	Staphylococcal respiratory response protein SrrA	0	0	0	0	0	0	0	0	0.000219	0	0	3.94388E-05
Protein Metabolism	Protein biosynthesis	tRNA aminoacylation, Met	Structure-specific tRNA-binding protein trbp111	0	0	0	0	0	0	0	0.000498	0	0	0	0.000118316
Cofactors, Vitamins, Prosthetic Groups, Pigments	Coenzyme A	Coenzyme A Biosynthesis	Substrate-specific component PanT of predicted pantothenate ECF transporter	0	0	0	0	0	0	0	0.000498	0	0	0	0.000118316
Membrane Transport	NULL	ECF class transporters	Substrate-specific component PdxU2 of predicted pyridoxin-related ECF transporter	0	0	0	0	0	0.000974	0	0	0	0	0	0.000197194
Membrane Transport	NULL	ECF class transporters	Substrate-specific component RibU of riboflavin ECF transporter	0.002437	0	0	0	0	0	0	0	0	0	0	7.88775E-05
Membrane Transport	NULL	ECF class transporters	Substrate-specific component TrpP of tryptophan ECF transporter	0	0	0	0	0	0	0	0	0.000219	0	0	0.000197194
Respiration	Electron donating reactions	Succinate dehydrogenase	Succinate dehydrogenase cytochrome b560 subunit	0	0	0	0	0	0	0	0	0.000219	0	0	7.88775E-05
Membrane Transport	Sugar Phosphotransferase Systems, PTS	Sucrose-specific PTS	Sucrose operon repressor ScrR, LacI family	0	0	0	0	0	0	0	0.000498	0	0	0	0.000276071
Carbohydrates	Monosaccharides	D-galactarate, D-glucarate and D-glycerate catabolism	Sugar diacid utilization regulator SdaR	0	0	0	0	0	0	0	0	0.000438	0	0	0.000157755
Stress Response	Oxidative stress	Oxidative stress	Superoxide dismutase [Mn/Fe] (EC 1.15.1.1)	0	0	0	0	0	0.000974	0	0	0	0	0	0.000118316
Regulation and Cell signaling	NULL	Sex pheromones in Enterococcus faecalis and other Firmicutes	Surface exclusion protein Sea1/PrgA	0	0	0	0	0	0	0	0	0.000438	0	0	3.94388E-05
Phages, Prophages, Transposable elements, Plasmids	Phages, Prophages	Staphylococcal phi-Mu50B-like prophages	Tape measure protein [SA bacteriophages 11, Mu50B]	0	0	0	0	0	0	0	0	0.000438	0	0	3.94388E-05
Cell Wall and Capsule	Gram-Positive cell wall components	Teichoic and lipoteichoic acids biosynthesis	Teichoic acid biosynthesis protein	0	0	0	0	0	0	0.000722	0	0	0	0	0.000512704
Cell Wall and Capsule	Gram-Positive cell wall components	Teichoic and lipoteichoic acids biosynthesis	Teichoic acid translocation permease protein TagG	0	0	0	0	0	0	0	0.000498	0	0	0	0.000236633
Stress Response	Detoxification	Tellurite resistance: Chromosomal determinants	Tellurite resistance protein TrgA	0	0	0	0	0	0.000487	0	0	0	0	0	7.88775E-05
Metabolism of Aromatic Compounds	NULL	Toluene 4-monooxygenase (T4MO)	Toluene-4-monooxygenase, subunit TmoE	0	0	0	0	0	0	0	0	0.000438	0	0	3.94388E-05
Miscellaneous	Plant-Prokaryote DOE project	DOE COG3533	TonB-dependent receptor ass. w/ COG3533Xanth	0	0	0	0	0	0	0	0.000498	0	0	0	3.94388E-05
RNA Metabolism	Transcription	RNA polymerase II initiation factors	Transcription initiation factor IIH p90 subunit	0.000348	0	0	0	0	0	0	0	0	0	0	0.000118316
Amino Acids and Derivatives	Lysine, threonine, methionine, and cysteine	Lysine degradation	Transcriptional activator of cad operon	0	0	0	0	0	0.000487	0	0	0	0	0	0.000197194
Nitrogen Metabolism	NULL	Nitrilase	Transcriptional regulator in custer with plant-induced nitrilase	0	0	0	0	0	0	0	0	0	0	0.000349	3.94388E-05
Carbohydrates	Monosaccharides	Mannose Metabolism	Transcriptional regulator of mannoside utilization, variant 2, LacI family	0	0	0	0	0.000657	0	0	0	0	0	0	3.94388E-05
Carbohydrates	Monosaccharides	L-rhamnose utilization	Transcriptional regulator of rhamnose utilization, AraC family	0.001044	0	0	0	0	0	0	0	0	0	0	3.94388E-05
Carbohydrates	Di- and oligosaccharides	Trehalose Uptake and Utilization	Transcriptional regulator of trehalose utilization, LacI family	0	0	0	0	0	0	0	0.000997	0.000438	0	0	0
Clustering-based subsystems	Catabolism of an unknown compound	CBSS-262316.1.peg.2929	Transcriptional regulator, GntR family, in hypothetical Actinobacterial gene cluster	0	0	0	0	0	0	0	0	0.000219	0	0	0.000118316
Carbohydrates	Di- and oligosaccharides	Lactose and Galactose Uptake and Utilization	Transcriptional repressor of the lac operon	0	0	0	0	0.000657	0	0	0	0	0	0	0.000354949
Amino Acids and Derivatives	Glutamine, glutamate, aspartate, asparagine; ammonia assimilation	Poly-gamma-glutamate biosynthesis	Transition state regulatory protein AbrB	0	0	0	0	0	0	0	0	0.000219	0	0	0.000591581
Membrane Transport	NULL	ECF class transporters	Transmembrane component CbiQ of energizing module of cobalt ECF transporter	0	0.004524	0	0	0	0	0	0	0	0	0	0.000236633
Cofactors, Vitamins, Prosthetic Groups, Pigments	Tetrapyrroles	Coenzyme B12 biosynthesis	Transmembrane component CbrV of energizing module of predicted cobalamin ECF transporter	0.001044	0	0	0	0	0	0	0	0	0	0	3.94388E-05
Amino Acids and Derivatives	Lysine, threonine, methionine, and cysteine	Methionine Biosynthesis	Transmembrane component MtsC of energizing module of methionine-regulated ECF transporter	0	0	0	0	0	0	0	0.000498	0	0	0	0.000157755
Membrane Transport	NULL	ECF class transporters	Transmembrane component TTE1588 of energizing module of predicted methylthioadenosine ECF transporter	0	0	0	0	0	0	0	0	0	0	0.000466	0.000354949

Regulation and Cell signaling	Regulation of virulence	Streptococcus pyogenes virulence regulators	Transmembrane histidine kinase CsrS	0	0	0	0	0	0	0	0.000498	0	0	0	7.88775E-05
Fatty Acids, Lipids, and Isoprenoids	Triacylglycerols	Triacylglycerol metabolism	Triacylglycerol lipase precursor (EC 3.1.1.3)	0	0	0.002555	0	0	0	0	0	0.000438	0	0	0
Potassium metabolism	NULL	Potassium homeostasis	Trk system potassium uptake protein TrkG	0	0	0.002555	0	0	0	0	0	0	0	0	0.000118316
Clustering-based subsystems	NULL	CBSS-193567.1.peg.90	tRNA binding domain protein	0	0	0	0	0	0	0.000722	0	0	0	0	0.000473265
Secondary Metabolism	Aromatic amino acids and derivatives	Pyrrolnitrin biosynthesis	Tryptophan halogenase PrnA	0	0	0	0	0	0	0	0.000498	0	0.002203	0	0
Protein Metabolism	Protein processing and modification	Peptide methionine sulfoxide reductase	Two-component response regulator yesN, associated with MetSO reductase	0	0	0	0	0	0	0	0.000997	0	0	0	0.000236633
Virulence, Disease and Defense	Bacteriocins, ribosomally synthesized antibacterial peptides	Bacitracin Stress Response	Two-component response regulator YvcP	0	0	0	0	0	0.000487	0	0	0	0	0	3.94388E-05
Carbohydrates	Central carbohydrate metabolism	Pyruvate metabolism I: anaplerotic reactions, PEP	Two-component response regulator, malate (EC 2.7.3.-)	0	0	0	0	0	0	0	0	0.000219	0	0	0.000197194
Protein Metabolism	Protein processing and modification	Peptide methionine sulfoxide reductase	Two-component sensor kinase yesM (EC 2.7.3.-), associated with MetSO reductase	0	0	0	0	0	0.000487	0	0	0	0	0	0.00031551
Regulation and Cell signaling	NULL	Rcs phosphorelay signal transduction pathway	Two-component sensor protein RcsC (EC 2.7.3.-)	0	0	0	0	0	0	0	0	0.000219	0	0	0.000512704
Regulation and Cell signaling	NULL	Two-component regulatory systems in Campylobacter	Two-component system response regulator DccR	0	0	0	0	0	0.000487	0	0	0	0	0	0.002090254
Regulation and Cell signaling	NULL	Two-component regulatory systems in Campylobacter	Two-component system response regulator RacR	0	0	0.002555	0	0	0	0	0	0	0	0	0.000276071
Membrane Transport	Protein secretion system, Type III	Type III secretion systems	Type III secretion thermoregulatory protein (LcrF,VirF,transcription regulation of virulence plasmid)	0	0	0	0	0	0	0	0	0	0.002203	0	0.000118316
Cofactors, Vitamins, Prosthetic Groups, Pigments	Quinone cofactors	Ubiquinone Biosynthesis	Ubiquinone biosynthesis monooxgenase COQ6 (EC 1.14.13.-)	0	0	0	0	0	0	0	0.000498	0	0	0	0.000157755
Protein Metabolism	Protein processing and modification	Ubiquitin-like archaeal modifier proteins (SAMPs)	Ubiquitin-like small archaeal modifier protein SAMP1	0	0	0	0	0.000657	0	0	0	0	0	0	3.94388E-05
Respiration	Electron accepting reactions	Anaerobic respiratory reductases	Uncharacterized component of anaerobic dehydrogenases	0	0	0	0	0	0.001461	0	0	0	0	0	3.94388E-05
Stress Response	Oxidative stress	Glutathione: Non-redox reactions	Uncharacterized GST-like protein yncG	0	0	0	0	0	0.000487	0	0	0	0	0	3.94388E-05
Iron acquisition and metabolism	Siderophores	Siderophore Anthrachelin	Uncharacterized iron compound ABC uptake transporter, ATP-binding protein	0	0	0	0	0.000657	0	0	0	0	0	0	3.94388E-05
Respiration	NULL	Methanogenesis strays	Uncharacterized protein MJ0094	0	0	0	0.00249	0	0	0	0	0	0	0	0.000236633
Respiration	NULL	Methanogenesis strays	Uncharacterized protein MJ0308	0	0	0	0	0	0	0	0	0.000219	0	0.000116	0
Respiration	NULL	Methanogenesis strays	Uncharacterized protein MJ0498	0	0	0	0.00249	0	0	0	0	0	0	0	7.88775E-05
Stress Response	Oxidative stress	Glutathione analogs: mycothiol	Uncharacterized protein Rv0487/MT0505 clustered with mycothiol biosynthesis gene	0	0	0	0.00249	0	0	0	0	0	0	0	0.000157755
Clustering-based subsystems	NULL	USS-DB-7	Uncharacterized protein similar to VCA0109	0	0	0	0	0	0	0	0	0.003284	0	0	0.000157755
Clustering-based subsystems	Chromosome Replication	SeqA and Co-occurring Genes	Uncharacterized protein ybfE	0	0	0	0	0	0	0	0	0.000438	0	0	7.88775E-05
Carbohydrates	NULL	Unknown carbohydrate utilization (cluster Ydj)	Uncharacterized protein YeaC	0	0	0	0	0	0	0	0	0.000438	0	0	0.000197194
Clustering-based subsystems	NULL	Yfa cluster	Uncharacterized protein YfaW	0	0	0	0	0	0.000487	0	0	0	0	0	3.94388E-05
Clustering-based subsystems	Clustering-based subsystems	Putative diaminopropionate ammonia-lyase cluster	Uncharacterized protein YqeB	0	0	0	0	0	0	0	0	0.000438	0	0	0.000118316
Stress Response	NULL	Universal stress protein family	Universal stress protein F	0	0.004524	0	0	0	0	0	0.001495	0	0	0	0
Stress Response	NULL	Universal stress protein family	Universal stress protein G	0	0	0	0	0	0	0	0.000997	0.000438	0	0	0
Amino Acids and Derivatives	Arginine; urea cycle, polyamines	Urea decomposition	Urea channel UreI	0	0	0.00511	0	0	0	0	0	0	0	0	3.94388E-05
Miscellaneous	Plant-Prokaryote DOE project	Experimental-yggC	Uridine kinase family protein YggC homolog	0	0.002262	0	0	0	0	0	0	0	0	0	7.88775E-05
Carbohydrates	Monosaccharides	D-Galacturonate and D-Glucuronate Utilization	Uxu operon transcriptional regulator	0	0.002262	0	0	0	0	0	0	0	0	0	0.000473265
Membrane Transport	Protein secretion system, Type VI	Type VI secretion systems	VgrG-3 protein	0	0	0	0	0	0	0	0.000498	0	0	0	3.94388E-05
Phages, Prophages, Transposable elements, Plasmids	Pathogenicity islands	Listeria Pathogenicity Island LIPI-1 extended	virulence cluster protein B VclB	0	0	0	0	0	0	0	0	0.000219	0	0	3.94388E-05
Regulation and Cell signaling	NULL	WhiB and WhiB-type regulatory proteins	WhiB-like transcription regulator	0	0	0	0	0	0	0	0	0.000219	0	0	0.000473265
Regulation and Cell signaling	NULL	WhiB and WhiB-type regulatory proteins	WhiB-like transcriptional regulator	0	0	0	0	0	0	0	0	0	0.002203	0	7.88775E-05
Cell Wall and Capsule	Capsular and extracellular polysacchrides	Xanthan Exopolysaccharide Biosynthesis and Export	Xanthan biosynthesis chain length determinant protein GumC	0	0	0	0	0	0.000974	0	0	0	0	0	0.000276071
Cell Wall and Capsule	Capsular and extracellular polysacchrides	Xanthan Exopolysaccharide Biosynthesis and Export	Xanthan biosynthesis glycosyltransferase GumM	0	0	0	0	0	0	0	0	0.000219	0	0	3.94388E-05
Photosynthesis	NULL	Proteorhodopsin	Xanthorhodopsin	0	0	0	0	0	0	0	0.002492	0.000657	0	0	0
Carbohydrates	NULL	Sugar utilization in Thermotogales	Xylose oligosaccharides ABC transporter, permease protein 1	0	0	0	0	0	0	0	0	0.000876	0	0	0.001301479
Carbohydrates	Di- and oligosaccharides	Maltose and Maltodextrin Utilization	Zinc-type alcohol dehydrogenase YcjQ	0	0	0	0	0	0	0	0.000498	0	0	0	3.94388E-05
Virulence, Disease and Defense	Toxins and superantigens	Cholera toxin	Zona occludens toxin	0	0	0	0	0.000657	0	0	0	0.000219	0	0	0
Metabolism of Aromatic Compounds	Anaerobic degradation of aromatic compounds	Anaerobic toluene and ethylbenzene degradation	(S)-1-phenylethanol dehydrogenase	0	0	0	0	0	0	0.000722	0	0	0	0	0
Fatty Acids, Lipids, and Isoprenoids	Fatty acids	Fatty Acid Biosynthesis FASI	[Acyl-carrier-protein] malonyl transferase of FASI (EC 2.3.1.39)	0	0	0	0	0	0	0	0	0	0	0	3.94388E-05
Fatty Acids, Lipids, and Isoprenoids	Fatty acids	Fatty Acid Biosynthesis FASI	[Acyl-carrier-protein] palmitoyl transferase of FASI (EC 2.3.1.-)	0	0	0	0	0.000657	0	0	0	0	0	0	0
Carbohydrates	Polysaccharides	Glycogen metabolism	1,4-alpha-glucan (starch) branching enzyme, GH-13-type (EC 2.4.1.18)	0	0	0	0	0	0	0	0	0	0	0	7.88775E-05
Iron acquisition and metabolism	Siderophores	Bacillibactin Siderophore	2,3-dihydro-2,3-dihydroxybenzoate dehydrogenase (EC 1.3.1.28) [bacillibactin] siderophore	0	0	0	0	0	0	0	0	0	0	0	3.94388E-05
Metabolism of Aromatic Compounds	NULL	p-cymene degradation	2,3-dihydroxy-2,3-dihydro-p-cumate dehydrogenase(CmtB) (EC 1.1.1.100)	0	0	0	0	0	0	0.000722	0	0	0	0	0
Iron acquisition and metabolism	Siderophores	Siderophore pyochelin	2,3-dihydroxybenzoate-AMP ligase (EC 2.7.7.58) [enantio-pyochelin] siderophore	0	0	0	0	0	0	0	0	0	0	0	7.88775E-05
Iron acquisition and metabolism	Siderophores	Siderophore Enterobactin	2,3-dihydroxybenzoate-AMP ligase (EC 2.7.7.58) [enterobactin] siderophore	0	0	0	0	0	0	0	0	0	0	0	7.88775E-05
Amino Acids and Derivatives	Lysine, threonine, methionine, and cysteine	Methionine Salvage	2,3-diketo-5-methylthiopentyl-1-phosphate enolase-phosphatase related protein	0	0	0	0	0	0	0	0	0	0	0	3.94388E-05
Metabolism of Aromatic Compounds	Anaerobic degradation of aromatic compounds	Anaerobic toluene and ethylbenzene degradation	2-[hydroxy(phenyl)methyl]-succinyl-CoA dehydrogenase alpha subunit (EC 1.1.1.35)	0	0	0	0	0	0	0	0	0	0	0	0.000118316
Phosphorus Metabolism	NULL	Phosphonate metabolism	2-aminoethylphosphonate ABC transporter ATP-binding protein (TC 3.A.1.9.1)	0	0	0	0	0	0	0	0	0	0	0	0.000236633
Phosphorus Metabolism	NULL	Phosphonate metabolism	2-aminoethylphosphonate ABC transporter permease protein I (TC 3.A.1.9.1)	0	0	0	0	0	0	0	0	0	0	0	0.000157755
Metabolism of Aromatic Compounds	Peripheral pathways for catabolism of aromatic compounds	Chlorobenzoate degradation	2-chlorobenzoate 1,2-dioxygenase beta subunit (EC 1.14.12.13)	0	0	0	0	0	0	0	0	0	0	0	0.000118316

Secondary Metabolism	Biosynthesis of phenylpropanoids	Phytosterol biosynthesis	2-cys peroxiredoxin BAS1, chloroplast precursor	0	0	0	0	0	0	0	0	0	0	0	3.94388E-05
RNA Metabolism	RNA processing and modification	RNA processing orphans	2H phosphoesterase superfamily protein Bsu1186 (yjcG)	0	0.004524	0	0	0	0	0	0	0	0	0	0
Secondary Metabolism	Aromatic amino acids and derivatives	Cinnamic Acid Degradation	2-hydroxy-6-ketonona-2,4-dienedioic acid hydrolase (EC 3.7.1.-)	0	0	0	0	0	0	0	0	0	0	0	0.000118316
Cofactors, Vitamins, Prosthetic Groups, Pigments	Pyridoxine	Pyridoxin(Vitamin B6) Degradation Pathway	2-methyl-3-hydroxypyridine-5-carboxylic acid oxygenase (EC 1.14.12.4)	0	0	0	0	0	0	0.000722	0	0	0	0	0
Fatty Acids, Lipids, and Isoprenoids	Phospholipids	Sphingolipid biosynthesis	3-dehydrosphinganine reductase (EC 1.1.1.102)	0	0	0	0	0	0	0	0	0.000219	0	0	0
Metabolism of Aromatic Compounds	Peripheral pathways for catabolism of aromatic compounds	Phenylpropanoid compound degradation	3-hydroxyphenylpropionic acid transporter	0	0	0	0	0	0	0	0	0	0	0	7.88775E-05
Fatty Acids, Lipids, and Isoprenoids	Fatty acids	Fatty acid metabolism cluster	3-oxoacid CoA-transferase (EC 2.8.3.5)	0	0	0	0	0	0	0	0	0	0	0	0.000118316
Fatty Acids, Lipids, and Isoprenoids	Fatty acids	Fatty Acid Biosynthesis FASI	3-oxoacyl-[acyl-carrier-protein] reductase of FASI (EC 1.1.1.100)	0	0	0	0	0	0	0	0	0	0	0	3.94388E-05
Fatty Acids, Lipids, and Isoprenoids	Fatty acids	Fatty Acid Biosynthesis FASI	3-oxoacyl-[acyl-carrier-protein] synthase of FASI (EC 2.3.1.41)	0	0	0	0	0	0	0	0	0	0	0	7.88775E-05
Fatty Acids, Lipids, and Isoprenoids	Fatty acids	Fatty Acid Biosynthesis FASII	3-oxoacyl-[acyl-carrier-protein] synthase, KASI, alternative (EC 2.3.1.41)	0	0	0	0	0	0	0	0.000498	0	0	0	0
Secondary Metabolism	Aromatic amino acids and derivatives	Cinnamic Acid Degradation	3-phenylpropionate dioxygenase ferredoxin--NAD(+) reductase component (EC 1.18.1.3)	0	0	0	0	0	0	0	0	0	0	0	3.94388E-05
Fatty Acids, Lipids, and Isoprenoids	Isoprenoids	Polyprenyl Diphosphate Biosynthesis	4,4'-diaponeurosporenoate glycosyltransferase (EC 2.4.1.-)	0	0	0	0	0	0	0	0.000498	0	0	0	0
Metabolism of Aromatic Compounds	Metabolism of central aromatic intermediates	4-Hydroxyphenylacetic acid catabolic pathway	4-hydroxyphenylacetate symporter, major facilitator superfamily (MFS)	0	0	0	0	0	0	0	0	0.000219	0	0	0
Fatty Acids, Lipids, and Isoprenoids	Fatty acids	Fatty Acid Biosynthesis FASI	4'-phosphopantetheinyl transferase of FASI (EC 2.7.8.7)	0	0	0	0	0	0	0	0	0	0	0	3.94388E-05
RNA Metabolism	RNA processing and modification	eukaryotic rRNA modification and related functions	60S ribosome subunit biogenesis protein NIP7	0	0	0	0	0	0	0	0	0	0	0	3.94388E-05
Iron acquisition and metabolism	Siderophores	Siderophore pyochelin	ABC efflux pump, fused inner membrane and ATPase subunits in pyochelin gene cluster	0	0	0	0	0	0	0	0	0	0	0	0.000197194
Iron acquisition and metabolism	Siderophores	Siderophore Pyoverdine	ABC transporter in pyoverdin gene cluster, ATP-binding component	0	0	0	0	0	0	0	0	0	0	0	0.000118316
Iron acquisition and metabolism	Siderophores	Salmochelin-mediated Iron Acquisition	ABC transporter protein IroC	0	0	0	0	0	0	0	0	0	0	0	3.94388E-05
Virulence, Disease and Defense	Resistance to antibiotics and toxic compounds	Streptococcus pneumoniae Vancomycin Tolerance Locus	ABC transporter, ATP-binding protein Vex2	0	0	0	0	0.000657	0	0	0	0	0	0	0
Cell Wall and Capsule	Capsular and extracellular polysacchrides	Sialic Acid Metabolism	ABC transporter, predicted N-acetylneuraminate transport system permease protein 2	0	0	0	0	0	0	0	0	0	0	0	7.88775E-05
Stress Response	NULL	Flavohaemoglobin	ABC-type Fe3+-siderophore transport system, permease 2 component	0	0	0	0	0	0	0	0	0.000657	0	0	0
Membrane Transport	ABC transporters	ABC transporter tungstate (TC 3.A.1.6.2)	ABC-type vanadate transport system, periplasmic binding protein	0	0	0	0	0	0	0	0	0	0	0	0.000157755
Virulence, Disease and Defense	Adhesion	Accessory colonization factor	Accessory colonization factor AcfA	0	0	0	0	0	0.000487	0	0	0	0	0	0
Regulation and Cell signaling	NULL	Staphylococcal accessory gene regulator system	Accessory gene regulator A (response regulator)	0	0	0	0	0	0	0	0	0	0	0	3.94388E-05
Regulation and Cell signaling	NULL	Staphylococcal accessory gene regulator system	Accessory gene regulator B	0	0	0	0	0	0	0	0	0.000219	0	0	0
Membrane Transport	Protein translocation across cytoplasmic membrane	SecY2-SecA2 Specialized Transport System	Accessory secretory protein Asp1	0	0	0	0	0	0	0	0	0	0	0	7.88775E-05
Membrane Transport	Protein translocation across cytoplasmic membrane	SecY2-SecA2 Specialized Transport System	Accessory secretory protein Asp2	0	0	0	0	0	0	0	0	0	0	0	0.000157755
Metabolism of Aromatic Compounds	NULL	Phenylacetyl-CoA catabolic pathway (core)	Acetate permease ActP (cation/acetate symporter) PaaL	0	0	0	0	0	0	0	0	0	0	0	7.88775E-05
Cofactors, Vitamins, Prosthetic Groups, Pigments	NAD and NADP	NAD consumption	Actin-ADP-ribosyltransferase, toxin SpvB	0	0	0	0	0	0	0	0	0	0	0	0.000118316
Fatty Acids, Lipids, and Isoprenoids	Fatty acids	Fatty Acid Biosynthesis FASI	Acyl carrier protein of FASI	0	0	0	0	0	0	0	0	0	0	0	7.88775E-05
Fatty Acids, Lipids, and Isoprenoids	NULL	Cholesterol catabolic operon in Mycobacteria	Acyl-CoA dehydrogenase IgrB	0	0	0	0	0	0	0	0	0	0.002203	0	0
Cofactors, Vitamins, Prosthetic Groups, Pigments	Tetrapyrroles	Coenzyme B12 biosynthesis	Additional substrate-specific component CbiN of cobalt ECF transporter	0	0	0	0	0	0	0	0	0	0	0	0.000236633
Virulence, Disease and Defense	Adhesion	Adhesins in Staphylococcus	Adhesin of unknown specificity SdrE, similar to bone sialoprotein-binding protein Bbp	0	0	0	0	0	0	0	0	0	0	0	3.94388E-05
Carbohydrates	Central carbohydrate metabolism	Glycolysis and Gluconeogenesis, including Archaeal enzymes	ADP-dependent glucokinase (EC 2.7.1.147)	0	0	0	0	0	0	0	0	0	0	0	7.88775E-05
Membrane Transport	NULL	Agrobacterium opine transport	Agropinic acid transporter periplasmic substrate-binding protein AgaA	0.001393	0	0	0	0	0	0	0	0	0	0	0
Membrane Transport	NULL	Agrobacterium opine transport	Agropinic acid transporter permease protein AgaB	0	0	0	0	0	0.000487	0	0	0	0	0	0
Protein Metabolism	Protein biosynthesis	tRNA aminoacylation, Ala	Alanyl-tRNA synthetase, amino-terminal half (EC 6.1.1.7)	0	0	0	0	0	0	0	0	0	0	0	0.000197194
Amino Acids and Derivatives	Proline and 4-hydroxyproline	Proline, 4-hydroxyproline uptake and utilization	Aldehyde dehydrogenase (EC 1.2.1.3) in 4-hydroxyproline catabolic gene cluster	0	0	0	0	0	0	0	0	0	0	0	3.94388E-05
Carbohydrates	Central carbohydrate metabolism	Methylglyoxal Metabolism	Aldehyde dehydrogenase 2B4, mitochondrial precursor (EC 1.2.1.3) (ALDH2a)	0	0	0	0	0	0	0	0	0	0	0	3.94388E-05
Clustering-based subsystems	Biosynthesis of galactoglycans and related lipopolysacharides	CBSS-258594.1.peg.3339	Aldehyde dehydrogenase-like protein	0	0	0	0	0	0	0	0	0.000219	0	0	0
Clustering-based subsystems	Lysine, threonine, methionine, and cysteine	CBSS-84588.1.peg.1247	All3116 protein	0	0	0	0	0	0	0	0	0.000438	0	0	0
Photosynthesis	Light-harvesting complexes	Phycobilisome	Allophycocyanin-B	0	0	0	0	0	0	0	0	0	0	0.001281	0
Carbohydrates	Monosaccharides	D-Galacturonate and D-Glucuronate Utilization	Alpha-1,4-digalacturonate ABC transporter, permease protein 1	0	0	0	0	0	0	0	0	0	0	0	3.94388E-05
Cell Wall and Capsule	Gram-Negative cell wall components	Core Oligosaccharide Glycosylation in Pseudomonas	alpha-1,6-rhamnosyltransferase	0	0	0	0	0	0	0	0	0	0	0	0.000118316
Regulation and Cell signaling	Proteolytic pathway	Coagulation cascade	Alpha-1-peptidase inhibitor, protease inhibitor I04.001	0	0	0	0	0	0	0	0	0	0	0	3.94388E-05
Carbohydrates	Polysaccharides	Cellulosome	Alpha-amylase SusG (EC 3.2.1.1)	0	0	0	0	0	0	0	0	0	0	0	7.88775E-05
Photosynthesis	Electron transport and photophosphorylation	Photosystem I	alternative photosystem I reaction center subunit X (PsaK2)	0	0	0	0	0	0	0	0	0.000876	0	0	0
Carbohydrates	NULL	Sugar utilization in Thermotogales	alternative Ribulokinase (EC 2.7.1.16)	0	0	0	0	0	0	0	0	0	0	0	7.88775E-05
Virulence, Disease and Defense	Adhesion	Adhesion of Campylobacter	Amino-acid ABC transporter ATP-binding protein PebC	0	0	0	0	0	0	0	0	0	0	0	0.000118316
Secondary Metabolism	Aromatic amino acids and derivatives	Pyrrolnitrin biosynthesis	Aminopyrrolnitrin oxidase PrnD	0	0	0	0	0.000657	0	0	0	0	0	0	0
Miscellaneous	Plant-Prokaryote DOE project	PROSC	AMINOTRANSFERASE CLASS-I (EC 2.6.1.-)	0	0	0	0	0	0	0	0	0	0	0	0.000118316
Cell Wall and Capsule	Gram-Positive cell wall components	Anthrose Biosynthesis	Aminotransferase, anthrose biosynthesis	0	0	0	0	0	0	0	0	0	0	0	3.94388E-05
Iron acquisition and metabolism	Siderophores	Siderophore Anthrachelin	Anthrachelin biosynthesis protein AsbA	0	0	0	0	0.000657	0	0	0	0	0	0	0
Iron acquisition and metabolism	Siderophores	Siderophore Anthrachelin	Anthrachelin biosynthesis protein AsbB	0	0	0	0	0	0	0	0	0	0	0.000116	0
Clustering-based subsystems	NULL	CBSS-83332.1.peg.3803	Antigen 85-A precursor (85A) (Antigen 85 complex A) (Ag85A) (Mycolyl transferase 85A) (EC 2.3.1.-)	0	0	0	0	0	0	0	0	0	0	0	7.88775E-05

Clustering-based subsystems	NULL	CBSS-83332.1.peg.3803	Antigen 85-B precursor (85B) (Extracellular alpha-antigen) (Antigen 85 complex B) (Ag85B) (Mycolyl transferase 85B) (EC 2.3.1.-)	0	0	0	0	0	0	0	0	0	0	0	3.94388E-05
Phages, Prophages, Transposable elements, Plasmids	Phages, Prophages	Listeria phi-A118-like prophages	Antirepressor [Bacteriophage A118]	0	0	0	0	0	0	0.000722	0	0	0	0	0
Regulation and Cell signaling	Proteolytic pathway	Coagulation cascade	Antithrombin III, protease inhibitor I04.018	0	0	0	0	0	0	0	0	0	0	0	3.94388E-05
Regulation and Cell signaling	Programmed Cell Death and Toxin-antitoxin Systems	Toxin-Antitoxin MT1	Antitoxin 1	0	0	0	0	0	0	0	0	0	0	0	3.94388E-05
Regulation and Cell signaling	Programmed Cell Death and Toxin-antitoxin Systems	A toxin-antitoxin module cotranscribed with DinB	Antitoxin YafN	0	0	0	0	0	0	0	0	0	0	0	7.88775E-05
Regulation and Cell signaling	Programmed Cell Death and Toxin-antitoxin Systems	Toxin-antitoxin systems (other than RelBE and MazEF)	Antitoxin YgiT	0	0	0	0	0	0	0	0	0	0	0	7.88775E-05
Clustering-based subsystems	Carbohydrates	Beta-lactamase cluster in Streptococcus	Aquaporin (Major Intrinsic Protein Family)	0	0	0	0	0	0	0	0	0	0	0.000233	0
Amino Acids and Derivatives	Arginine; urea cycle, polyamines	Arginine and Ornithine Degradation	Arginine ABC transporter, periplasmic arginine-binding protein ArtJ	0	0	0	0	0	0	0	0	0	0	0	7.88775E-05
Amino Acids and Derivatives	Arginine; urea cycle, polyamines	Arginine and Ornithine Degradation	Arginine ABC transporter, permease protein ArtM	0	0	0	0	0	0	0	0	0	0	0	3.94388E-05
Clustering-based subsystems	NULL	CBSS-316407.3.peg.2816	Arginine exporter protein ArgO	0	0	0	0	0	0	0	0	0	0	0	7.88775E-05
Amino Acids and Derivatives	Arginine; urea cycle, polyamines	Arginine and Ornithine Degradation	Arginine N-succinyltransferase, beta subunit (EC 2.3.1.109)	0	0	0	0	0	0	0	0	0	0	0	0.000157755
Clustering-based subsystems	NULL	CBSS-562.2.peg.5158 SK3 including	AroM protein	0	0	0	0	0	0	0	0.000498	0	0	0	0
Amino Acids and Derivatives	Aromatic amino acids and derivatives	Aromatic amino acid degradation	Aromatic ring-opening dioxygenase	0	0	0	0	0.000657	0	0	0	0	0	0	0
Virulence, Disease and Defense	Resistance to antibiotics and toxic compounds	Arsenic resistance	Arsenical resistance operon trans-acting repressor ArsD	0	0	0	0	0	0	0	0	0	0	0	0.000354949
Carbohydrates	Monosaccharides	L-ascorbate utilization (and related gene clusters)	Ascorbate utilization transcriptional regulator UlaR, HTH-type	0	0	0	0	0	0	0	0	0	0	0	3.94388E-05
Carbohydrates	Monosaccharides	L-ascorbate utilization (and related gene clusters)	Ascorbate-specific PTS system, EIIA component (EC 2.7.1.-)	0	0	0	0	0	0	0	0	0	0	0	3.94388E-05
Carbohydrates	Monosaccharides	L-ascorbate utilization (and related gene clusters)	Ascorbate-specific PTS system, EIIC component	0	0	0	0	0	0	0	0	0	0	0	3.94388E-05
Amino Acids and Derivatives	Glutamine, glutamate, aspartate, asparagine; ammonia assimilation	Glutamine, Glutamate, Aspartate and Asparagine Biosynthesis	Asparagine synthetase [glutamine-hydrolyzing] (EC 6.3.5.4) YisO	0	0	0	0	0	0	0	0	0.000219	0	0	0
Carbohydrates	Sugar alcohols	Ethanolamine utilization	ATP:Cob(I)alamin adenosyltransferase (EC 2.5.1.17), ethanolamine utilization	0	0	0	0	0	0	0	0	0	0	0	7.88775E-05
Clustering-based subsystems	NULL	ClpS cluster with hypothetical protein in Cyanos	ATP-dependent Clp protease adaptor protein ClpS Cyano2	0	0	0	0	0	0	0	0	0.001095	0	0	0
Clustering-based subsystems	NULL	ClpS like protein and vWFA-like protein cluster	ATP-dependent Clp protease adaptor protein ClpS like	0	0	0	0	0	0	0	0.000498	0	0	0	0
RNA Metabolism	RNA processing and modification	ATP-dependent RNA helicases, bacterial	ATP-dependent RNA helicase YfmL	0	0	0	0	0	0	0	0	0	0	0	0.000276071
Miscellaneous	Plant-Prokaryote DOE project	At5g38900	ATP-energized glutathione S-conjugate pump, putative	0	0	0	0	0	0	0	0	0	0	0	0.000276071
Regulation and Cell signaling	Quorum sensing and biofilm formation	Quorum-sensing in Vibrio	Autoinducer 1 sensor kinase/phosphatase LuxN (EC 2.7.3.-) (EC 3.1.3.-)	0	0	0	0	0	0	0	0	0	0	0	0.000394388
Regulation and Cell signaling	Quorum sensing and biofilm formation	Autoinducer 2 (AI-2) transport and processing (lsrACDBFGE operon)	Autoinducer 2 (AI-2) ABC transport system, membrane channel protein LsrD	0	0	0	0	0	0	0	0.000498	0	0	0	0
Membrane Transport	Protein secretion system, Type V	Autotransporter proteins	autotransporter domain, T5aSS type secretion	0	0	0	0	0	0	0	0	0.000219	0	0	0
Protein Metabolism	Protein degradation	Metalloendopeptidases (EC 3.4.24.-)	Bacillolysin, extracellular neutral metalloprotease (EC 3.4.24.28)	0	0	0	0	0	0	0	0	0	0	0	0.000354949
Sulfur Metabolism	Organic sulfur assimilation	L-Cystine Uptake and Metabolism	Bacterial luciferase family protein YtmO, in cluster with L-cystine ABC transporter	0	0	0	0	0	0	0	0	0	0	0	0.000118316
Virulence, Disease and Defense	Bacteriocins, ribosomally synthesized antibacterial peptides	Bacteriocin-like peptides Blp	Bacteriocin	0	0	0	0	0	0	0	0	0	0	0	0.000512704
Virulence, Disease and Defense	Bacteriocins, ribosomally synthesized antibacterial peptides	Bacteriocin-like peptides Blp	Bacteriocin immunity protein BlpL	0	0	0	0	0	0	0	0	0	0	0	3.94388E-05
Stress Response	Oxidative stress	Oxidative stress	bacteriophytochrome heme oxygenase BphO	0	0	0	0	0	0	0	0	0.000219	0	0	0
Virulence, Disease and Defense	Bacteriocins, ribosomally synthesized antibacterial peptides	Bacteriocin-like peptides Blp	Bactriocin immunity protein BlpY	0	0	0	0	0	0	0	0	0	0	0.000349	0
Miscellaneous	Plant-Prokaryote DOE project	Niacin-Choline transport and metabolism	BAT1-like	0	0	0	0	0	0	0	0	0	0	0	7.88775E-05
Amino Acids and Derivatives	Histidine Metabolism	Histidine Degradation	Believed to be an alternative form of N-formylglutamate deformylase (EC 3.5.1.68), but experimentally invalidated	0	0	0	0	0	0	0	0	0	0	0	0.000118316
Metabolism of Aromatic Compounds	Peripheral pathways for catabolism of aromatic compounds	Benzoate degradation	Benzoate catabolic operon transcription regulator	0	0	0	0	0	0	0	0	0	0	0	7.88775E-05
Metabolism of Aromatic Compounds	Anaerobic degradation of aromatic compounds	Anaerobic toluene and ethylbenzene degradation	Benzoylsuccinyl-CoA thiolase alpha subunit (EC:2.3.1.-)	0	0	0	0	0	0	0	0	0	0	0	0.000118316
Metabolism of Aromatic Compounds	Anaerobic degradation of aromatic compounds	Anaerobic toluene and ethylbenzene degradation	Benzylsuccinate synthase activating enzyme (EC 1.97.1.4)	0	0	0	0	0	0	0	0	0	0	0	0.000276071
Sulfur Metabolism	NULL	Galactosylceramide and Sulfatide metabolism	Beta-galactosidase 3	0	0	0	0	0	0	0	0	0	0	0	7.88775E-05
Sulfur Metabolism	NULL	Galactosylceramide and Sulfatide metabolism	Beta-galactosidase large subunit (EC 3.2.1.23)	0	0	0	0	0	0	0	0	0	0	0	7.88775E-05
Carbohydrates	NULL	Sugar utilization in Thermotogales	Beta-glucoside ABC transport system, sugar-binding protein	0	0.002262	0	0	0	0	0	0	0	0	0	0
Carbohydrates	Di- and oligosaccharides	Beta-Glucoside Metabolism	Beta-glucoside bgl operon antiterminator, BglG family	0	0	0	0	0	0	0	0	0	0	0	3.94388E-05
Virulence, Disease and Defense	Resistance to antibiotics and toxic compounds	Beta-lactamase	Beta-lactamase AmpS	0	0	0	0	0.000657	0	0	0	0	0	0	0
Virulence, Disease and Defense	Resistance to antibiotics and toxic compounds	Beta-lactamase	Beta-lactamase repressor BlaI	0	0	0	0	0	0	0	0	0	0	0	0.000157755
Clustering-based subsystems	Carbohydrates	Beta-lactamase cluster in Streptococcus	Beta-lactamase, class C	0	0	0	0	0.000657	0	0	0	0	0	0	0
Regulation and Cell signaling	Quorum sensing and biofilm formation	Biofilm formation in Staphylococcus	Biofilm operon icaABCD HTH-type negative transcriptional regulator IcaR	0	0	0	0	0	0	0	0	0	0	0	3.94388E-05
Regulation and Cell signaling	Quorum sensing and biofilm formation	Biofilm Adhesin Biosynthesis	Biofilm PGA outer membrane secretin PgaA	0.002437	0	0	0	0	0	0	0	0	0	0	0
Respiration	Sodium Ion-Coupled Energetics	Na+ translocating decarboxylases and related biotin-dependent enzymes	Biotin carboxyl carrier protein of oxaloacetate decarboxylase	0	0	0	0	0	0.000487	0	0	0	0	0	0
Miscellaneous	Plant-Prokaryote DOE project	Biotin biosynthesis Experimental	Biotin synthesis protein bioG	0	0	0	0	0	0	0	0	0	0	0	0.000118316
Miscellaneous	Plant-Prokaryote DOE project	Biotin biosynthesis Experimental	Biotin synthesis protein bioK	0	0	0	0	0	0	0	0.000997	0	0	0	0
Miscellaneous	Plant-Prokaryote DOE project	Biotin biosynthesis Experimental	Biotin synthesis protein bioZ	0	0	0	0	0.000657	0	0	0	0	0	0	0
Metabolism of Aromatic Compounds	Peripheral pathways for catabolism of aromatic compounds	Biphenyl Degradation	Biphenyl dioxygenase beta subunit (EC 1.14.12.18)	0	0	0	0	0	0	0	0	0	0	0	0.000118316
Metabolism of Aromatic Compounds	Peripheral pathways for catabolism of aromatic compounds	Biphenyl Degradation	biphenyl-2,3-diol 1,2-dioxygenase III-related protein VCA0463 VCA0328 VCA0341	0	0	0	0	0	0	0	0	0	0	0	7.88775E-05
Fatty Acids, Lipids, and Isoprenoids	Isoprenoids	Carotenoids	C50 carotenoid epsilon cyclase	0	0	0	0	0	0	0	0	0	0	0	3.94388E-05
Membrane Transport	NULL	ECF class transporters	Candidate substrate-specific domain of ECF transporters in Mycobacteria	0	0	0	0	0	0.000487	0	0	0	0	0	0
Cell Wall and Capsule	Capsular and extracellular polysacchrides	Extracellular Polysaccharide Biosynthesis of Streptococci	Capsular polysaccharide biosynthesis protein Cps4E	0	0	0	0	0	0	0	0	0	0	0	7.88775E-05

Cell Wall and Capsule	Capsular and extracellular polysacchrides	Capsular Polysaccharides Biosynthesis and Assembly	Capsular polysaccharide biosynthesis protein WcbQ	0	0	0	0	0	0	0	0	0	0	0	0.000512704
Cell Wall and Capsule	Capsular and extracellular polysacchrides	Capsular Polysaccharide (CPS) of Campylobacter	Capsular polysaccharide export system protein KpsF	0	0	0	0	0	0	0	0	0	0	0	0.000118316
Cell Wall and Capsule	Capsular and extracellular polysacchrides	Capsular Polysaccharides Biosynthesis and Assembly	Capsular polysaccharide glycosyltransferase biosynthesis protein WcbB	0	0	0	0	0	0	0	0	0	0	0	7.88775E-05
Cell Wall and Capsule	Capsular and extracellular polysacchrides	Serotype determining Capsular polysaccharide biosynthesis in Staphylococcus	Capsular polysaccharide synthesis enzyme Cap5G	0	0	0	0	0	0	0	0	0	0	0	0.000354949
Cell Wall and Capsule	Capsular and extracellular polysacchrides	Serotype determining Capsular polysaccharide biosynthesis in Staphylococcus	Capsular polysaccharide synthesis enzyme Cap5L	0	0	0	0	0	0	0	0	0	0	0	3.94388E-05
Cell Wall and Capsule	Capsular and extracellular polysacchrides	Serotype determining Capsular polysaccharide biosynthesis in Staphylococcus	Capsular polysaccharide synthesis enzyme Cap8G	0	0	0	0	0	0	0	0	0	0	0	7.88775E-05
Cell Wall and Capsule	Capsular and extracellular polysacchrides	Serotype determining Capsular polysaccharide biosynthesis in Staphylococcus	Capsular polysaccharide synthesis enzyme Cap8L	0	0	0	0	0	0	0	0	0	0	0	3.94388E-05
Cell Wall and Capsule	Capsular and extracellular polysacchrides	Exopolysaccharide Biosynthesis	Capsular polysaccharide synthesis enzyme CpsE	0	0	0	0	0	0	0	0	0.000438	0	0	0
Carbohydrates	CO2 fixation	CO2 uptake, carboxysome	Carboxysome protein CcmL	0.001044	0	0	0	0	0	0	0	0	0	0	0
Iron acquisition and metabolism	NULL	Iron acquisition in Vibrio	Catechol siderophore ABC transporter, ATP-binding component	0	0	0	0	0	0	0	0	0	0	0	7.88775E-05
Iron acquisition and metabolism	NULL	Iron acquisition in Vibrio	Catechol siderophore ABC transporter, permease component	0	0	0	0	0	0	0	0	0.000219	0	0	0
Cell Wall and Capsule	Gram-Positive cell wall components	Teichoic and lipoteichoic acids biosynthesis	CDP-glycerol:poly(glycerophosphate) glycerophosphotransferase (EC 2.7.8.12)	0	0	0	0	0	0	0	0	0	0	0	0.00063102
Cell Wall and Capsule	Gram-Positive cell wall components	Teichoic and lipoteichoic acids biosynthesis	CDP-ribitol:poly(ribitol phosphate) ribitol phosphotransferase	0	0	0	0	0	0	0	0	0	0	0	3.94388E-05
Cell Division and Cell Cycle	NULL	Control of cell elongation - division cycle in Bacilli	Cell division protein GpsB, coordinates the switch between cylindrical and septal cell wall synthesis by re-localization of PBP1	0	0	0	0	0	0	0	0	0	0	0	3.94388E-05
Clustering-based subsystems	Cell Division	Cell Division Cluster	Cell division protein YlmG/Ycf19 (putative), YggT family	0	0	0	0	0	0	0	0	0	0	0	0.000157755
Regulation and Cell signaling	NULL	Cell envelope-associated LytR-CpsA-Psr transcriptional attenuators	Cell envelope-associated transcriptional attenuator LytR-CpsA-Psr, subfamily F1 (as in PMID19099556)	0	0	0	0	0	0	0	0	0	0	0	7.88775E-05
Regulation and Cell signaling	NULL	Cell envelope-associated LytR-CpsA-Psr transcriptional attenuators	Cell envelope-associated transcriptional attenuator LytR-CpsA-Psr, subfamily S (as in PMID19099556)	0	0	0.002555	0	0	0	0	0	0	0	0	0
Iron acquisition and metabolism	NULL	Heme, hemin uptake and utilization systems in GramPositives	Cell surface protein IsdA, transfers heme from hemoglobin to apo-IsdC	0	0	0	0	0	0	0	0	0	0	0	7.88775E-05
Iron acquisition and metabolism	NULL	Heme, hemin uptake and utilization systems in GramPositives	Cell surface receptor IsdH for hemoglobin-haptoglobin complexes	0	0	0	0	0	0	0	0	0.000219	0	0	0
Cell Wall and Capsule	Gram-Positive cell wall components	Sortase	Cell wall surface anchor family protein, LPXTG motif	0	0	0	0	0	0.000487	0	0	0	0	0	0
Membrane Transport	Protein secretion system, Type III	Type III secretion systems	Chaperone protein YscY (Yop proteins translocation protein Y)	0	0	0	0	0	0	0	0	0	0	0	0.000118316
Carbohydrates	Aminosugars	Chitin and N-acetylglucosamine utilization	Chitobiose-specific 6-phospho-beta-glucosidase ChbF (EC 3.2.1.86)	0	0	0	0	0	0	0.000722	0	0	0	0	0
Respiration	Electron donating reactions	Respiratory Complex I	chloroplast NADH dehydrogenase ND4	0	0	0	0	0	0	0	0	0	0	0	7.88775E-05
Stress Response	Osmotic stress	Choline and Betaine Uptake and Betaine Biosynthesis	Choline ABC transport system, permease protein OpuBB	0	0	0	0	0	0	0	0	0	0	0	0.000118316
Stress Response	Osmotic stress	Choline and Betaine Uptake and Betaine Biosynthesis	Choline binding protein A	0	0	0	0	0	0	0	0	0	0	0	3.94388E-05
Clustering-based subsystems	Choline bitartrate degradation, putative	CBSS-344610.3.peg.2335	Choline monooxygenase, alpha subunit (EC 1.14.15.7)	0	0	0	0	0	0	0	0	0	0	0	3.94388E-05
Clustering-based subsystems	Choline bitartrate degradation, putative	CBSS-344610.3.peg.2335	Choline monooxygenase, beta subunit (EC 1.14.15.7)	0	0	0	0	0	0	0	0	0	0	0	0.000157755
Carbohydrates	Aminosugars	N-Acetyl-Galactosamine and Galactosamine Utilization	Chondroitinase (chondroitin lyase)	0	0	0	0	0	0	0	0.000498	0	0	0	0
Amino Acids and Derivatives	Aromatic amino acids and derivatives	Chorismate Synthesis	Chorismate mutase III (EC 5.4.99.5)	0	0	0	0	0	0	0	0	0.000219	0	0	0
Regulation and Cell signaling	Programmed Cell Death and Toxin-antitoxin Systems	Murein hydrolase regulation and cell death	CidA-associated membrane protein CidB	0	0	0	0	0	0.000974	0	0	0	0	0	0
Cell Division and Cell Cycle	NULL	Cyanobacterial Circadian Clock	Circadian clock protein KaiA	0	0	0	0	0	0	0	0	0.001314	0	0	0
Cell Division and Cell Cycle	NULL	Cyanobacterial Circadian Clock	Circadian period extender Pex	0	0	0	0	0	0	0	0	0.000657	0	0	0
RNA Metabolism	RNA processing and modification	Polyadenylation specificity factor	Cleavage and polyadenylation specificity factor, 160 kDa subunit	0	0	0	0	0.000657	0	0	0	0	0	0	0
Carbohydrates	Polysaccharides	Cellulosome	Clostridium cellulosome enzyme, dockerin type I precursor	0	0	0	0	0	0	0	0	0	0	0	3.94388E-05
Virulence, Disease and Defense	Adhesion	Adhesins in Staphylococcus	Clumping factor ClfA, fibrinogen-binding protein	0	0	0	0	0	0	0	0	0	0	0	0.000118316
Cell Wall and Capsule	Capsular and extracellular polysacchrides	Sialic Acid Metabolism	CMP-N-acetylneuraminate-beta-galactosamide-alpha-2,3-sialyltransferase (EC 2.4.99.-)	0	0	0	0	0	0	0.000722	0	0	0	0	0
Respiration	Electron accepting reactions	Anaerobic respiratory reductases	CoB--CoM heterodisulfide reductase 2 subunit E (EC 1.8.98.1)	0	0	0	0	0	0	0	0	0	0	0	3.94388E-05
Cofactors, Vitamins, Prosthetic Groups, Pigments	Coenzyme B	Coenzyme B synthesis	Coenzyme B synthesis from 2-oxoglutarate: steps 1, 6, and 10	0	0	0	0	0	0	0	0	0	0	0	7.88775E-05
Respiration	Electron donating reactions	Coenzyme F420-H2 dehydrogenase (methanophenazine)	Coenzyme F(420)H(2) dehydrogenase (methanophenazine) subunit FpoF	0	0	0	0	0	0	0	0	0	0	0	3.94388E-05
Respiration	Electron donating reactions	Coenzyme F420-H2 dehydrogenase (methanophenazine)	Coenzyme F(420)H(2) dehydrogenase (methanophenazine) subunit FpoJ2	0	0	0	0	0	0	0	0	0	0	0	3.94388E-05
Respiration	Electron donating reactions	Coenzyme F420-H2 dehydrogenase (methanophenazine)	Coenzyme F(420)H(2) dehydrogenase (methanophenazine) subunit FpoK	0	0	0	0	0	0	0	0	0	0	0	3.94388E-05
Respiration	Electron donating reactions	Coenzyme F420-H2 dehydrogenase (methanophenazine)	Coenzyme F(420)H(2) dehydrogenase (methanophenazine) subunit FpoO	0	0	0	0	0	0	0.000722	0	0	0	0	0
Respiration	Electron donating reactions	Coenzyme F420 hydrogenase	Coenzyme F420 hydrogenase beta subunit (FrcB) (EC 1.12.98.1)	0	0	0	0	0	0	0	0	0	0	0	0.000118316
Respiration	Electron donating reactions	Coenzyme F420 hydrogenase	Coenzyme F420 hydrogenase beta subunit (FruB) (EC 1.12.98.1)	0	0	0	0	0	0	0	0	0	0	0	3.94388E-05
Cofactors, Vitamins, Prosthetic Groups, Pigments	Quinone cofactors	Pyrroloquinoline Quinone biosynthesis	Coenzyme PQQ synthesis protein A	0	0	0	0	0	0	0	0	0.000219	0	0	0
Cofactors, Vitamins, Prosthetic Groups, Pigments	Folate and pterines	Methanopterin biosynthesis2	COG1821 family protein	0	0	0	0.00249	0	0	0	0	0	0	0	0
Respiration	NULL	Methanogenesis strays	COG4073: protein of unknown function in methanogens	0	0	0	0	0	0	0	0	0	0	0	3.94388E-05
Clustering-based subsystems	Tricarboxylate transporter	CBSS-49338.1.peg.459	COG5591: Uncharacterized conserved protein	0	0.002262	0	0	0	0	0	0	0	0	0	0
Cell Wall and Capsule	Capsular and extracellular polysacchrides	Colanic acid biosynthesis	Colanic acid biosynthesis acetyltransferase WcaB (EC 2.3.1.-)	0	0	0	0	0	0	0	0	0.000219	0	0	0
Cell Wall and Capsule	Capsular and extracellular polysacchrides	Colanic acid biosynthesis	Colanic acid polymerase WcaD	0	0	0	0	0	0	0.000722	0	0	0	0	0
DNA Metabolism	DNA uptake, competence	Late competence	ComF operon protein C	0	0	0	0	0	0	0	0	0	0	0	3.94388E-05
Miscellaneous	Plant-Prokaryote DOE project	Competence or DNA damage-inducible protein CinA and related protein families	Competence/damage-inducible protein CinA family	0	0	0	0	0	0	0	0	0	0	0	0.000276071

Clustering-based subsystems	NULL	PFGI-1-like cluster 2	Conjugative transfer ATPase PilU in PFGI-1-like cluster	0	0	0	0	0	0	0	0	0	0	0	7.88775E-05
Membrane Transport	Protein and nucleoprotein secretion system, Type IV	Conjugative transfer	Conjugative transfer entry exclusion protein TrbK	0	0	0	0	0	0	0	0	0	0	0	3.94388E-05
Clustering-based subsystems	NULL	PFGI-1-like cluster 2	Conjugative transfer pilus-tip adhesin protein PilV in PFGI-1-like cluster	0	0	0	0	0	0	0	0	0	0	0	3.94388E-05
Clustering-based subsystems	NULL	Conjugative transfer related cluster	Conjugative transfer protein TraA	0	0	0	0	0	0	0	0	0	0	0	3.94388E-05
Clustering-based subsystems	NULL	Conjugative transfer related cluster	Conjugative transfer protein TraV	0	0	0	0	0	0	0	0	0	0	0	0.000197194
Membrane Transport	Protein and nucleoprotein secretion system, Type IV	Conjugative transfer	Conjugative transfer protein TrbA	0	0	0	0	0	0	0.000722	0	0	0	0	0
Membrane Transport	Protein and nucleoprotein secretion system, Type IV	Conjugative transfer	Conjugative transfer protein TrbN	0	0	0	0	0	0	0	0	0.000219	0	0	0
Membrane Transport	Protein and nucleoprotein secretion system, Type IV	Conjugative transfer	Conjugative transfer protein TrbO	0	0	0	0	0	0.000487	0	0	0	0	0	0
Membrane Transport	Protein and nucleoprotein secretion system, Type IV	Conjugative transfer	Conjugative transfer protein TrbP (IncF TraX homolog)	0	0	0	0	0	0.000487	0	0	0	0	0	0
Phages, Prophages, Transposable elements, Plasmids	Transposable elements	Conjugative transposon, Bacteroidales	Conjugative transposon protein TraF	0	0	0	0	0	0	0	0	0	0	0	3.94388E-05
DNA Metabolism	DNA repair	DNA repair, bacterial UvrD and related helicases	Conjugative transposon transfer DNA helicase	0	0	0	0	0	0	0	0	0	0	0	3.94388E-05
Fatty Acids, Lipids, and Isoprenoids	NULL	Cholesterol catabolic operon in Mycobacteria	Conserved protein IgrD	0	0	0.002555	0	0	0	0	0	0	0	0	0
Secondary Metabolism	Biosynthesis of phenylpropanoids	Phytoalexin biosynthesis	copalyl diphosphate synthase (CPS) (ent-kaurene synthetase A)	0	0	0	0	0	0	0	0	0	0	0	3.94388E-05
Clustering-based subsystems	NULL	CBSS-196620.1.peg.2477	Copper ion binding protein	0	0	0	0	0	0	0	0	0	0	0	7.88775E-05
Virulence, Disease and Defense	Resistance to antibiotics and toxic compounds	Copper homeostasis	Copper resistance protein C precursor	0	0	0	0	0	0.000487	0	0	0	0	0	0
DNA Metabolism	CRISPs	CRISPRs	CRISPR-associated protein MTH1087	0	0	0	0	0	0	0	0	0.000219	0	0	0
DNA Metabolism	CRISPs	CRISP Cmr Cluster	CRISPR-associated protein TM1812	0	0	0	0	0	0	0	0	0	0	0	3.94388E-05
DNA Metabolism	CRISPs	CRISPRs	CRISPR-associated protein, MJ0381 family	0	0	0	0	0	0	0	0	0	0	0	7.88775E-05
DNA Metabolism	CRISPs	CRISPRs	CRISPR-associated protein, WS1616 family	0	0	0	0	0	0	0	0	0	0	0	3.94388E-05
DNA Metabolism	CRISPs	CRISP Cmr Cluster	CRISPR-associated RAMP Cmr5	0	0	0	0	0	0	0	0	0	0	0	0.000118316
Regulation and Cell signaling	NULL	Staphylococcal accessory gene regulator system	CsbB stress response protein	0	0	0	0	0	0	0	0	0	0	0	3.94388E-05
Stress Response	Oxidative stress	Redox-dependent regulation of nucleus processes	C-terminal binding protein 2	0	0	0	0	0	0	0	0	0	0	0	3.94388E-05
RNA Metabolism	Transcription	Transcription factors cyanobacterial RpoD-like sigma factors	Cyanobacteria-specific RpoD-like sigma factor, type-12	0	0	0	0	0.000657	0	0	0	0	0	0	0
RNA Metabolism	Transcription	Transcription factors cyanobacterial RpoD-like sigma factors	Cyanobacteria-specific RpoD-like sigma factor, type-4	0	0	0	0	0.000657	0	0	0	0	0	0	0
Cell Wall and Capsule	Cell wall of Mycobacteria	mycolic acid synthesis	Cyclopropane-fatty-acyl-phospholipid synthase 2, CmaA2 (EC 2.1.1.79)	0	0	0	0	0	0	0	0	0.000219	0	0	0
Sulfur Metabolism	Organic sulfur assimilation	L-Cystine Uptake and Metabolism	Cystine ABC transporter, periplasmic cystine-binding protein FliY	0	0	0	0	0	0	0	0	0	0	0	7.88775E-05
Sulfur Metabolism	NULL	Sulfur oxidation	cytochrome c subunit of flavocytochrome c sulfide dehydrogenase	0	0	0	0	0	0	0	0	0	0	0	3.94388E-05
Respiration	NULL	Soluble cytochromes and functionally related electron carriers	Cytochrome c-552 precursor	0	0	0	0	0	0	0	0	0	0	0	0.00031551
Respiration	Electron accepting reactions	Terminal cytochrome C oxidases	Cytochrome c553-like	0	0	0	0	0	0	0	0	0	0	0	0.000118316
Protein Metabolism	Protein processing and modification	Peptide methionine sulfoxide reductase	Cytochrome c-type biogenesis protein CcdA homolog, associated with MetSO reductase	0	0	0	0	0	0	0	0	0	0	0	7.88775E-05
Respiration	NULL	Biogenesis of c-type cytochromes	Cytochrome c-type heme lyase CCHL (EC 4.4.1.17)	0	0	0	0	0	0	0	0	0	0	0	3.94388E-05
Respiration	Electron donating reactions	Hydrogenases	cytochrome-c3 hydrogenase beta chain	0	0	0	0	0	0	0	0	0.000219	0	0	0
Phages, Prophages, Transposable elements, Plasmids	Pathogenicity islands	Vibrio pathogenicity island	Cytoplasmic, CoA-independent, aldehyde dehydrogenase (EC 1.2.1.3)	0	0	0	0	0	0	0	0	0	0	0	3.94388E-05
Carbohydrates	Monosaccharides	D-allose utilization	D-allose ABC transporter, permease component	0	0	0	0	0	0	0	0	0	0	0	7.88775E-05
Carbohydrates	Monosaccharides	D-allose utilization	D-allose-6-phosphate isomerase (EC 5.3.1.-)	0	0	0	0	0	0	0	0	0	0	0	3.94388E-05
Carbohydrates	Monosaccharides	D-allose utilization	D-allulose-6-phosphate 3-epimerase (EC 5.1.3.-), row id 846	0	0	0	0	0	0	0	0	0	0	0	3.94388E-05
Miscellaneous	NULL	DedA family of inner membrane proteins	DedA family inner membrane protein YghB	0	0	0	0	0	0	0	0	0	0	0	3.94388E-05
Fatty Acids, Lipids, and Isoprenoids	Isoprenoids	Carotenoids	Dehydrosqualene synthase (EC 2.5.1.-)	0	0	0	0	0.000657	0	0	0	0	0	0	0
Stress Response	Dessication stress	O-antigen capsule important for environmental persistence	DeoR-type transcriptional regulator YihW	0	0	0	0	0	0	0	0.000498	0	0	0	0
Metabolism of Aromatic Compounds	Metabolism of central aromatic intermediates	Protocatechuate branch of beta-ketoadipate pathway	dicarboxylic acid transporter PcaT	0	0	0	0	0	0	0	0	0.000219	0	0	0
Metabolism of Aromatic Compounds	Peripheral pathways for catabolism of aromatic compounds	Naphtalene and antracene degradation	Dihydrodiol dehydrogenase (EC 1.3.1.56)	0	0.002262	0	0	0	0	0	0	0	0	0	0
Cofactors, Vitamins, Prosthetic Groups, Pigments	Folate and pterines	Folate Biosynthesis	Dihydroneopterin triphosphate pyrophosphohydolase	0	0.002262	0	0	0	0	0	0	0	0	0	0
Carbohydrates	One-carbon Metabolism	Methanogenesis from methylated compounds	Dimethylamine permease	0	0	0	0	0	0	0	0.000498	0	0	0	0
Dormancy and Sporulation	Spore DNA protection	Dipicolinate Synthesis	Dipicolinate synthase subunit B	0	0	0	0	0	0	0	0	0	0	0.000116	0
Phages, Prophages, Transposable elements, Plasmids	Phages, Prophages	Phage replication	DNA helicase (EC 3.6.1.-), phage-associated	0	0	0	0	0	0	0.000722	0	0	0	0	0
Stress Response	Oxidative stress	Regulation of Oxidative Stress Response	DNA protection during starvation protein	0	0	0	0	0	0	0	0	0.000219	0	0	0
Phages, Prophages, Transposable elements, Plasmids	Phages, Prophages	Phage replication	DNA replication protein, phage-associated	0	0	0	0	0.000657	0	0	0	0	0	0	0
DNA Metabolism	DNA replication	DNA-replication	DNA replication terminus site-binding protein	0	0	0	0	0	0	0	0	0	0	0	0.000197194
DNA Metabolism	DNA replication	DNA topoisomerases, Type I, ATP-independent	DNA topoisomerase III, Bacteroidales-type (EC 5.99.1.2)	0	0	0	0	0	0	0.000722	0	0	0	0	0
Clustering-based subsystems	NULL	PFGI-1-like cluster 2	DNA/RNA helicase in PFGI-1-like cluster	0	0	0	0	0	0.000487	0	0	0	0	0	0
Cofactors, Vitamins, Prosthetic Groups, Pigments	Riboflavin, FMN, FAD	Riboflavin, FMN and FAD metabolism	DNA-binding HTH domain in riboflavin kinase	0	0	0	0	0	0	0	0	0	0	0	0.000157755
DNA Metabolism	NULL	Nucleoid-associated proteins in Bacteria	DNA-binding protein stpA	0	0	0	0	0	0	0	0.000498	0	0	0	0
RNA Metabolism	Transcription	RNA polymerase chloroplast	DNA-directed RNA polymerase alpha subunit (EC 2.7.7.6), chloroplast	0	0	0	0	0	0	0	0	0	0	0	3.94388E-05
RNA Metabolism	Transcription	RNA polymerase I	DNA-directed RNA polymerase I largest subunit (EC 2.7.7.6)	0	0	0	0	0	0	0	0	0.000219	0	0	0
RNA Metabolism	Transcription	RNA polymerase I	DNA-directed RNA polymerase I second largest subunit (EC 2.7.7.6)	0.000348	0	0	0	0	0	0	0	0	0	0	0
RNA Metabolism	Transcription	RNA polymerase II	DNA-directed RNA polymerase II 13.3 kDa polypeptide (EC 2.7.7.6)	0	0	0	0	0	0	0.000722	0	0	0	0	0

RNA Metabolism	Transcription	RNA polymerase II	DNA-directed RNA polymerase II 45 kDa polypeptide (EC 2.7.7.6)	0	0	0	0	0	0	0.000722	0	0	0	0	0
RNA Metabolism	Transcription	RNA polymerase III	DNA-directed RNA polymerase III 12.5 kDa polypeptide (EC 2.7.7.6)	0	0	0	0	0.000657	0	0	0	0	0	0	0
RNA Metabolism	Transcription	RNA polymerase III	DNA-directed RNA polymerase III 25 kDa polypeptide (EC 2.7.7.6)	0	0	0	0	0	0	0	0	0	0	0	3.94388E-05
RNA Metabolism	Transcription	RNA polymerase archaeal	DNA-directed RNA polymerase subunit E''	0	0	0	0	0	0	0	0	0	0	0	3.94388E-05
RNA Metabolism	Transcription	RNA polymerase III	DNA-directed RNA polymerases I and III 40 kDa polypeptide (EC 2.7.7.6)	0	0	0	0	0	0.000487	0	0	0	0	0	0
RNA Metabolism	Transcription	RNA polymerase I	DNA-directed RNA polymerases I, II, and III 15 kDa polypeptide (EC 2.7.7.6)	0	0	0	0	0	0	0	0	0	0	0	0.000197194
RNA Metabolism	Transcription	RNA polymerase III	DNA-directed RNA polymerases I, II, and III 27 kDa polypeptide (EC 2.7.7.6)	0	0	0	0	0	0	0	0	0	0	0	3.94388E-05
Membrane Transport	Protein and nucleoprotein secretion system, Type IV	Dot-Icm type IV secretion system	DotD	0	0	0	0	0	0	0	0.000498	0	0	0	0
Amino Acids and Derivatives	Proline and 4-hydroxyproline	Proline, 4-hydroxyproline uptake and utilization	D-proline reductase, 23 kDa subunit (EC 1.21.4.1)	0	0	0	0	0	0	0	0	0	0	0	0.00063102
Amino Acids and Derivatives	Proline and 4-hydroxyproline	Proline, 4-hydroxyproline uptake and utilization	D-proline reductase, 45 kDa subunit (EC 1.21.4.1)	0	0	0	0	0	0	0	0	0	0	0	0.000552143
Clustering-based subsystems	NULL	CBSS-196620.1.peg.2477	D-specific D-2-hydroxyacid dehydrogenase ddh homolog (EC 1.1.1.28)	0	0	0	0	0	0	0	0	0	0	0	3.94388E-05
Cofactors, Vitamins, Prosthetic Groups, Pigments	Folate and pterines	YgfZ	DUF1794	0	0	0	0	0	0	0	0	0.000438	0	0	0
Membrane Transport	NULL	ECF class transporters	Duplicated ATPase component of energizing module of predicted ECF transporter in Mycobacteria	0	0	0	0	0	0	0	0	0	0	0	7.88775E-05
Membrane Transport	NULL	ECF class transporters	Duplicated ATPase component of energizing module of predicted pyridoxine-related ECF transporter	0	0	0	0	0	0	0	0.000498	0	0	0	0
Membrane Transport	NULL	ECF class transporters	Duplicated ATPase component SS1136 of energizing module of predicted ECF transporter	0	0	0	0	0	0	0	0	0.000219	0	0	0
Carbohydrates	Monosaccharides	Xylose utilization	D-xylose proton-symporter XylT	0	0	0	0	0	0	0	0	0	0	0	3.94388E-05
Membrane Transport	Protein translocation across cytoplasmic membrane	EcsAB transporter affecting expression and secretion of secretory preproteins	EcsC protein	0	0	0	0	0	0	0	0.000997	0	0	0	0
Virulence, Disease and Defense	Resistance to antibiotics and toxic compounds	Resistance to fluoroquinolones	Efflux pump Lde	0	0	0	0	0	0	0	0	0	0	0	0.000118316
Iron acquisition and metabolism	Siderophores	Siderophore pyochelin	Enantio-pyochelin biosynthetic protein PchC, predicted thioesterase	0	0	0	0	0	0	0	0	0.000219	0	0	0
Carbohydrates	NULL	Sugar utilization in Thermotogales	Endo-1,4-beta-xylanase B (EC 3.2.1.8)	0	0	0	0	0	0	0	0	0	0	0	0.00031551
Carbohydrates	NULL	Sugar utilization in Thermotogales	Endo-1,4-beta-xylanase C precursor (EC 3.2.1.8)	0	0	0	0	0	0	0	0	0	0	0	7.88775E-05
Respiration	Reverse electron transport	Mebrane bound hydrogenases	Energy conserving hydrogenase Eha associated protein (protein R)	0	0	0	0	0	0	0	0	0	0	0	3.94388E-05
Respiration	Reverse electron transport	Archaeal membrane bound hydrogenases	Energy conserving hydrogenase Eha protein M	0	0	0	0	0	0	0	0	0	0	0	7.88775E-05
Respiration	Reverse electron transport	Mebrane bound hydrogenases	Energy conserving hydrogenase Eha proton-sodium antiporter homolog protein H	0	0	0	0	0	0	0	0	0	0	0	3.94388E-05
Respiration	Reverse electron transport	Archaeal membrane bound hydrogenases	Energy conserving hydrogenase Eha small subunit homolog (protein N)	0	0	0	0	0	0	0	0	0	0	0	0.000118316
Respiration	Reverse electron transport	Archaeal membrane bound hydrogenases	Energy conserving hydrogenase Ehb anchor subunit F	0	0	0	0	0	0	0	0	0	0	0	0.000157755
Respiration	Electron donating reactions	Energy-conserving hydrogenase (ferredoxin) Ech	Energy-conserving hydrogenase (ferredoxin), subunit F	0	0	0	0	0	0	0	0	0	0	0	7.88775E-05
Cell Wall and Capsule	Gram-Positive cell wall components	Anthrose Biosynthesis	Enoyl-CoA hydratase (EC 4.2.1.17), anthrose biosynthesis	0	0	0	0	0	0	0	0	0	0	0	3.94388E-05
Iron acquisition and metabolism	NULL	Campylobacter Iron Metabolism	Enterochelin uptake periplasmic binding protein	0	0	0	0	0	0	0	0	0	0	0	0.000197194
Membrane Transport	Protein translocation across cytoplasmic membrane	ESAT-6 proteins secretion system in Firmicutes	ESAT-6/Esx family secreted protein EsxA/YukE	0	0	0	0	0	0	0.000722	0	0	0	0	0
Miscellaneous	Plant-Prokaryote DOE project	Synechocystis experimental	Esterase D (EC 3.1.1.1)	0	0	0	0	0	0	0	0	0.000438	0	0	0
Carbohydrates	Sugar alcohols	Ethanolamine utilization	Ethanolamine two-component response regulator	0	0	0	0	0	0	0	0	0	0	0	0.00031551
Carbohydrates	Sugar alcohols	Ethanolamine utilization	Ethanolamine utilization polyhedral-body-like protein EutK	0	0	0	0	0	0	0	0	0	0	0	7.88775E-05
Carbohydrates	Sugar alcohols	Ethanolamine utilization	Ethanolamine utilization protein EutQ	0	0	0	0	0	0	0	0	0	0	0	0.000433826
Metabolism of Aromatic Compounds	Anaerobic degradation of aromatic compounds	Anaerobic toluene and ethylbenzene degradation	Ethylbenzene dehydrogenase delta subunit (EC 1.17.99.2)	0	0	0	0	0	0	0.000722	0	0	0	0	0
Regulation and Cell signaling	Proteolytic pathway	Regulatory Intramembrane Proteolysis Pathways	Eukaryotic translation initiation factor 2-alpha kinase 1 (HRI)	0.000348	0	0	0	0	0	0	0	0	0	0	0
Regulation and Cell signaling	Proteolytic pathway	Regulatory Intramembrane Proteolysis Pathways	Eukaryotic translation initiation factor 2-alpha kinase 2 (PKR)	0	0	0	0	0	0	0	0	0	0	0	3.94388E-05
Protein Metabolism	Protein biosynthesis	Translation initiation factors eukaryotic and archaeal	Eukaryotic translation initiation factor 3 39 kDa subunit	0	0	0	0	0	0	0	0	0	0	0	3.94388E-05
Protein Metabolism	Protein biosynthesis	Translation initiation factors eukaryotic and archaeal	Eukaryotic translation initiation factor 3 90 kDa subunit	0	0	0	0	0	0	0	0	0	0	0	3.94388E-05
Protein Metabolism	Protein biosynthesis	Translation initiation factors eukaryotic and archaeal	Eukaryotic translation initiation factor 3 RNA-binding subunit	0	0	0	0	0	0	0	0	0	0	0	3.94388E-05
Protein Metabolism	Protein biosynthesis	Translation initiation factors eukaryotic and archaeal	Eukaryotic translation initiation factor 4E	0	0	0	0	0.000657	0	0	0	0	0	0	0
DNA Metabolism	DNA repair	DNA repair, UvrABC system	Excinuclease cho (excinuclease ABC alternative C subunit)	0	0	0	0	0	0	0	0	0	0	0	0.000118316
Cell Wall and Capsule	Capsular and extracellular polysacchrides	Exopolysaccharide Biosynthesis	Exopolysaccharide biosynthesis transcription antiterminator, LytR family	0	0	0	0	0	0	0	0	0	0	0	3.94388E-05
Clustering-based subsystems	Biosynthesis of galactoglycans and related lipopolysacharides	CBSS-258594.1.peg.3339	Exopolysaccharide production protein ExoF precursor	0	0	0	0	0	0	0	0	0	0	0	3.94388E-05
Virulence, Disease and Defense	Toxins and superantigens	Streptolysin S Biosynthesis and Transport	Export ABC transporter permease	0	0	0	0	0	0	0	0	0	0	0	3.94388E-05
DNA Metabolism	DNA uptake, competence	Natural DNA Transformation in Vibrio	Extracellular deoxyribonuclease Xds	0	0	0	0	0	0	0	0	0	0	0	0.000197194
Carbohydrates	Polysaccharides	Xyloglucan Utilization	Extracellular endo-1,4-glucanase	0	0	0	0	0	0	0	0.000498	0	0	0	0
Clustering-based subsystems	Putative asociate of RNA polymerase sigma-54 factor rpoN	CBSS-316057.3.peg.1308	extracytoplasmic sigma factor EcfR	0	0	0	0	0	0	0	0	0	0	0	3.94388E-05
Secondary Metabolism	Bacterial cytostatics, differentiation factors and antibiotics	Phenazine biosynthesis	FAD-dependent monooxygenase PhzS	0	0	0	0	0	0	0	0	0	0	0	0.000236633
Membrane Transport	Protein secretion system, Type VIII (Extracellular nucleation/precipitation pathway, ENP)	Fap amyloid fiber secretion system	Fap amyloid fibril major component	0	0	0	0	0	0	0.001444	0	0	0	0	0
Cell Wall and Capsule	Cell wall of Mycobacteria	mycolic acid synthesis	FATTY-ACID-CoA LIGASE FADD26 (FATTY-ACID-COA SYNTHETASE) (FATTY-ACID-CoA SYNTHASE)	0	0	0	0	0	0	0.000722	0	0	0	0	0
Clustering-based subsystems	NULL	CBSS-83332.1.peg.3803	fatty-acid-CoA ligase FadD32	0	0	0	0	0	0	0	0	0	0	0	7.88775E-05
Iron acquisition and metabolism	Siderophores	Bacillibactin Siderophore	Fe-bacillibactin uptake system FeuD	0	0	0	0	0	0	0	0	0	0	0	3.94388E-05
Virulence, Disease and Defense	Resistance to antibiotics and toxic compounds	Methicillin resistance in Staphylococci	FemC, factor involved in methicillin resistance	0	0	0	0	0	0	0	0	0	0	0	3.94388E-05

Carbohydrates	Fermentation	Acetyl-CoA fermentation to Butyrate	Ferredoxin-like protein	0	0	0	0	0	0	0	0	0	0	0	3.94388E-05
Iron acquisition and metabolism	NULL	Iron acquisition in Vibrio	Ferric aerobactin ABC transporter, periplasmic substrate binding protein	0	0	0	0	0	0	0	0	0	0	0	7.88775E-05
Iron acquisition and metabolism	Siderophores	Siderophore pyochelin	Ferric enantio-pyochelin transport system periplasmic substrate-binding protein	0	0	0	0	0	0	0	0.000498	0	0	0	0
Iron acquisition and metabolism	Siderophores	Siderophore pyochelin	Ferric enantio-pyochelin transport system permease protein	0	0	0	0	0	0	0	0	0	0	0	3.94388E-05
Iron acquisition and metabolism	Siderophores	Siderophore Enterobactin	Ferric enterobactin transport ATP-binding protein FepC (TC 3.A.1.14.2)	0	0	0	0	0	0	0	0	0	0	0	3.94388E-05
Iron acquisition and metabolism	Siderophores	Siderophore Enterobactin	Ferric enterobactin transport system permease protein FepG (TC 3.A.1.14.2)	0	0	0	0	0	0	0	0	0	0	0	7.88775E-05
Iron acquisition and metabolism	Siderophores	Vibrioferrin synthesis	Ferric siderophore receptor PsuA	0	0	0	0	0	0	0	0	0	0	0	0.000157755
Iron acquisition and metabolism	NULL	Campylobacter Iron Metabolism	Ferric siderophore transport system, biopolymer transport protein ExbD	0	0	0	0	0	0	0	0	0	0	0	7.88775E-05
Iron acquisition and metabolism	NULL	Iron acquisition in Vibrio	Ferric vibriobactin, enterobactin transport system, permease protein ViuD (TC 3.A.1.14.6)	0	0	0	0	0	0	0	0	0	0	0	3.94388E-05
Iron acquisition and metabolism	NULL	Iron acquisition in Vibrio	Ferric vibriobactin, enterobactin transport system, substrate-binding protein VctP (TC 3.A.1.14.6)	0	0	0	0	0	0	0	0	0	0	0	7.88775E-05
Iron acquisition and metabolism	Siderophores	Vibrioferrin synthesis	Ferrichrome ABC transporter (permease) PvuC	0	0	0	0	0	0	0	0	0	0	0.000466	0
Iron acquisition and metabolism	Siderophores	Vibrioferrin synthesis	Ferrichrome ABC transporter (permease) PvuD	0	0	0	0	0	0	0.000722	0	0	0	0	0
Iron acquisition and metabolism	Siderophores	Vibrioferrin synthesis	Ferrichrome ABC transporter, periplasmic iron-binding protein PvuB	0	0	0	0	0	0	0	0	0	0	0	7.88775E-05
Clustering-based subsystems	Monosaccharides	Unspecified monosaccharide transport cluster	FIG001621: Zinc protease	0	0	0	0	0	0	0	0	0	0	0	0.000197194
Clustering-based subsystems	Ribosomal Protein L28P relates to a set of uncharacterized proteins	A Gram-positive cluster that relates ribosomal protein L28P to a set of uncharacterized proteins	FIG001802: Putative alkaline-shock protein	0	0	0	0	0	0	0	0	0	0	0	0.000197194
Clustering-based subsystems	NULL	CBSS-214092.1.peg.3450	FIG002208: Acetyltransferase (EC 2.3.1.-)	0	0	0	0	0	0	0	0	0	0	0	0.000118316
Clustering-based subsystems	Oxidative stress	CoA-disulfide reductase (EC 1.8.1.14) containing cluster	FIG002540: Haloacid dehalogenase-like hydrolase	0	0	0	0	0	0.000487	0	0	0	0	0	0
Clustering-based subsystems	NULL	Divergent RNA modification related clusters	FIG005986: HD family hydrolase	0	0	0	0	0	0	0	0	0	0	0	0.000157755
Clustering-based subsystems	NULL	CBSS-350688.3.peg.1509	FIG007013: polysaccharide deacetylase, putative	0	0	0	0	0	0	0	0	0	0	0	0.000197194
Stress Response	Heat shock	Heat shock dnaK gene cluster extended	FIG009886: phosphoesterase	0	0	0	0	0	0	0	0	0	0	0	7.88775E-05
Clustering-based subsystems	NULL	Bacteriophage P4 cluster	FIG033266: Phage DNA binding protein	0	0	0	0	0	0	0	0	0	0	0	0.000118316
Clustering-based subsystems	NULL	CBSS-316273.3.peg.922	FIG035830: Two-component system regulatory protein	0	0	0	0	0	0.000487	0	0	0	0	0	0
Clustering-based subsystems	NULL	PFGI-1-like cluster 1	FIG041388: hypothetical protein in PFGI-1-like cluster	0	0	0	0	0.000657	0	0	0	0	0	0	0
Clustering-based subsystems	NULL	CBSS-316273.3.peg.448	FIG048626: Hypothetical protein	0	0	0	0	0	0	0	0	0	0	0	3.94388E-05
Miscellaneous	Plant-Prokaryote DOE project	At1g14345	FIG052964: hypothetical protein	0	0	0	0	0	0	0	0.000498	0	0	0	0
Miscellaneous	Plant-Prokaryote DOE project	At1g14345	FIG057954: metal-binding, possibly nucleic acid-binding protein	0	0	0	0	0	0	0	0	0.000219	0	0	0
Clustering-based subsystems	NULL	CBSS-291331.3.peg.3674	FIG086212: Putative lipoprotein	0	0	0	0	0	0	0	0	0	0	0	0.000197194
Clustering-based subsystems	NULL	ClpS cluster with hypothetical protein in Cyanos	FIG113347: hypothetical protein	0	0	0	0	0	0	0	0	0.001095	0	0	0
Clustering-based subsystems	NULL	CBSS-214092.1.peg.3450	FIG137864: putative endonuclease containing a URI domain	0	0	0	0	0	0	0	0	0	0	0	0.000552143
Clustering-based subsystems	NULL	CBSS-176279.3.peg.868	FIG139598: Potential ribosomal protein	0	0	0	0	0	0	0	0	0.000219	0	0	0
Clustering-based subsystems	Probably organic hydroperoxide resistance related hypothetical protein	CBSS-269482.1.peg.1294	FIGfam005179	0	0	0	0	0	0	0	0	0	0	0	7.88775E-05
Membrane Transport	Protein secretion system, Type VII (Chaperone/Usher pathway, CU)	The fimbrial Stf cluster	Fimbriae usher protein StfC	0	0	0	0	0	0	0	0.000997	0	0	0	0
Motility and Chemotaxis	Flagellar motility in Prokaryota	Flagellum	Flagellar transcriptional activator FlhC	0	0	0	0	0	0	0	0	0	0	0	0.000157755
Motility and Chemotaxis	Flagellar motility in Prokaryota	Flagellum	Flagellar transcriptional activator FlhD	0	0	0	0	0	0	0	0	0	0	0	3.94388E-05
Motility and Chemotaxis	Flagellar motility in Prokaryota	Archaeal Flagellum	Flagella-related protein FlaE	0	0	0	0	0	0	0	0	0	0	0	7.88775E-05
Motility and Chemotaxis	Flagellar motility in Prokaryota	Archaeal Flagellum	Flagellin FlaA	0	0	0	0	0	0	0	0	0.000219	0	0	0
Motility and Chemotaxis	Flagellar motility in Prokaryota	Flagellum	Flagellin protein FlaF	0	0	0	0	0	0	0	0	0	0	0	0.000118316
Virulence, Disease and Defense	Resistance to antibiotics and toxic compounds	Methicillin resistance in Staphylococci	FmtC (MrpF) protein involved in methicillin resistance	0	0	0	0	0	0	0	0	0	0	0	3.94388E-05
Respiration	NULL	Formate hydrogenase	formate dehydrogenase formation protein FdhE	0	0	0	0	0	0	0	0	0.000219	0	0	0
Respiration	NULL	Formate hydrogenase	Formate dehydrogenase N alpha subunit (EC 1.2.1.2); selenocysteine-containing	0	0	0	0	0	0	0	0	0	0	0	7.88775E-05
Respiration	NULL	Formate hydrogenase	Formate dehydrogenase N gamma subunit (EC 1.2.1.2)	0	0	0	0	0	0	0	0	0	0	0	0.000197194
Respiration	NULL	Formate hydrogenase	Formate hydrogenlyase subunit 2	0	0	0	0	0.000657	0	0	0	0	0	0	0
Carbohydrates	One-carbon Metabolism	Methanogenesis	Formylmethanofuran dehydrogenase (molybdenum) operon gene G	0	0	0	0	0	0	0	0	0	0	0	0.000157755
Carbohydrates	One-carbon Metabolism	Methanogenesis	Formylmethanofuran dehydrogenase (molybdenum) subunit D (EC 1.2.99.5)	0	0	0	0	0	0	0	0	0	0	0	0.000118316
Carbohydrates	One-carbon Metabolism	Methanogenesis	Formylmethanofuran dehydrogenase (tungsten) operon gene F (polyferredoxin)	0	0	0	0	0	0	0	0	0	0	0	0.000354949
Carbohydrates	One-carbon Metabolism	Methanogenesis	Formylmethanofuran dehydrogenase (tungsten) subunit D (EC 1.2.99.5)	0	0	0	0	0	0	0	0	0	0	0	7.88775E-05
Carbohydrates	One-carbon Metabolism	Methanogenesis	Formylmethanofuran dehydrogenase subunit D (EC 1.2.99.5)	0.003133	0	0	0	0	0	0	0	0	0	0	0
Respiration	NULL	Biogenesis of cytochrome c oxidases	Frataxin, mitochondrial precursor, facilitates iron supply for heme A synthesis or Fe-S cluster assembly	0	0	0	0	0	0	0	0	0	0	0	7.88775E-05
Carbohydrates	Polysaccharides	Unknown carbohydrate utilization containing Fructose-bisphosphate aldolase	Fructose-bisphosphate aldolase (EC 4.1.2.13)	0	0	0	0	0	0	0	0.000498	0	0	0	0
Carbohydrates	Aminosugars	Fructoselysine (Amadori product) utilization pathway	Fructoselysine transporter FrlA, cationic amino acid permease	0	0	0	0	0	0	0	0	0	0	0	7.88775E-05
Membrane Transport	Protein translocation across cytoplasmic membrane	ESAT-6 proteins secretion system in Actinobacteria	FtsK/SpoIIIE family protein Rv3871, component of ESX-1 secretion system	0	0	0	0	0	0	0	0	0	0	0	7.88775E-05
Carbohydrates	Monosaccharides	D-Tagatose and Galactitol Utilization	Galactitol utilization operon repressor	0	0	0	0	0	0	0	0	0	0	0	7.88775E-05
Carbohydrates	Aminosugars	N-Acetyl-Galactosamine and Galactosamine Utilization	Galactosamine-6-phosphate isomerase (galactosamine-6-phosphate deaminase) (EC 5.3.1.-)	0	0	0	0	0	0	0	0	0	0	0	7.88775E-05
Cell Wall and Capsule	Gram-Positive cell wall components	Teichoic and lipoteichoic acids biosynthesis	galactosamine-containing minor teichoic acid biosynthesis protein	0	0	0	0	0	0	0	0	0	0	0	3.94388E-05

Carbohydrates	Di- and oligosaccharides	Lactose and Galactose Uptake and Utilization	Galactose permease	0	0	0	0	0	0	0.000722	0	0	0	0	0
Carbohydrates	Di- and oligosaccharides	Lactose and Galactose Uptake and Utilization	Galactose-6-phosphate isomerase, LacA subunit (EC 5.3.1.26)	0	0	0	0	0	0	0	0.000498	0	0	0	0
Membrane Transport	NULL	Ton and Tol transport systems	Galactose-regulated TonB-dependent outer membrane receptor	0	0	0	0	0	0	0	0.000498	0	0	0	0
Amino Acids and Derivatives	Arginine; urea cycle, polyamines	Putrescine utilization pathways	Gamma-aminobutyrate:pyruvate aminotransferase (EC 2.6.1.-)	0	0	0	0	0	0	0.000722	0	0	0	0	0
Fatty Acids, Lipids, and Isoprenoids	Isoprenoids	Carotenoids	gamma-carotene hydroxylase	0	0	0	0	0	0	0	0	0	0	0	7.88775E-05
Stress Response	Oxidative stress	Glutathione: Biosynthesis and gamma-glutamyl cycle	Gamma-glutamyl cyclotransferase (EC 2.3.2.4)	0	0	0	0	0	0	0	0	0	0	0	7.88775E-05
Miscellaneous	Plant-Prokaryote DOE project	Niacin-Choline transport and metabolism	GbcA family protein, putative N1-methylnicotinamide or trigonelline demethylase	0	0	0	0.00249	0	0	0	0	0	0	0	0
Miscellaneous	Plant-Prokaryote DOE project	DOE COG3533	GDB1 like ass w/ COG3533Xanth	0	0	0	0	0	0	0	0	0.000219	0	0	0
Phages, Prophages, Transposable elements, Plasmids	Gene Transfer Agent (GTA)	Gene Transfer Agent	Gene Transfer Agent (GTA) ORFG01	0	0	0	0	0	0	0	0.000498	0	0	0	0
Membrane Transport	Protein translocation across cytoplasmic membrane	SecY2-SecA2 Specialized Transport System	GftB: Glycosyl transferase, family 8	0	0	0	0	0	0	0	0	0	0	0	0.000118316
Regulation and Cell signaling	NULL	DNA-binding regulatory proteins, strays	GltC, transcription activator of glutamate synthase operon	0	0	0	0	0.000657	0	0	0	0	0	0	0
Carbohydrates	Monosaccharides	D-Sorbitol(D-Glucitol) and L-Sorbose Utilization	Glucitol operon activator protein	0	0	0	0	0	0.000487	0	0	0	0	0	0
Carbohydrates	Monosaccharides	D-Sorbitol(D-Glucitol) and L-Sorbose Utilization	Glucitol operon repressor	0	0	0	0	0	0	0	0	0	0	0	0.000118316
Carbohydrates	Monosaccharides	D-gluconate and ketogluconates metabolism	Gluconate operon transcriptional repressor	0	0	0	0	0	0	0	0	0.000219	0	0	0
Carbohydrates	Aminosugars	(GlcNAc)2 Catabolic Operon	Glucosamine kinase GpsK (EC 2.7.1.8)	0	0	0	0	0	0	0	0	0	0	0	3.94388E-05
Carbohydrates	Monosaccharides	D-gluconate and ketogluconates metabolism	Glucose dehydrogenase (EC 1.1.99.10), membrane-bound, flavoprotein	0	0	0	0	0	0	0	0	0	0	0	3.94388E-05
Carbohydrates	NULL	Sugar utilization in Thermotogales	Glucose oligosaccharide ABC transport system, ATP-binding protein 2	0	0	0	0	0	0	0	0	0.000219	0	0	0
Carbohydrates	Monosaccharides	D-Galacturonate and D-Glucuronate Utilization	Glucuronide permease	0	0	0	0	0	0	0	0	0	0	0	7.88775E-05
Stress Response	Oxidative stress	Glutathione: Biosynthesis and gamma-glutamyl cycle	Glutamate--cysteine ligase archaeal (EC 6.3.2.2)	0	0	0	0	0	0	0.000722	0	0	0	0	0
Carbohydrates	CO2 fixation	Photorespiration (oxidative C2 cycle)	Glutamate-glyoxylate aminotransferase (EC 2.6.1.4)	0	0	0.002555	0	0	0	0	0	0	0	0	0
Protein Metabolism	Protein biosynthesis	tRNA aminoacylation, Glu and Gln	Glutaminyl-tRNA synthetase (EC 6.1.1.18), mitochondrial	0	0	0	0	0	0	0	0	0	0	0	3.94388E-05
Protein Metabolism	Protein degradation	Serine endopeptidase (EC 3.4.21.-)	Glutamyl endopeptidase precursor (EC 3.4.21.19), blaSE	0	0	0	0	0	0	0.000722	0	0	0	0	0
Stress Response	Oxidative stress	Glutaredoxins	Glutaredoxin 1	0	0	0	0	0	0	0	0	0.001095	0	0	0
Stress Response	Oxidative stress	Glutaredoxins	Glutaredoxin-like protein NrdH, required for reduction of Ribonucleotide reductase class Ib	0	0	0	0	0	0	0	0	0.000219	0	0	0
Stress Response	Oxidative stress	Glutathione: Non-redox reactions	Glutathione S-transferase, Streptococcal type (EC 2.5.1.18)	0	0	0	0	0.000657	0	0	0	0	0	0	0
Potassium metabolism	NULL	Potassium homeostasis	Glutathione-regulated potassium-efflux system protein KefKL	0	0	0	0	0	0	0.000722	0	0	0	0	0
Carbohydrates	Sugar alcohols	Glycerol fermenation to 1,3-propanediol	Glycerol dehydratase medium subunit (EC 4.2.1.30)	0	0	0	0	0	0	0	0	0	0	0	0.000197194
Carbohydrates	Sugar alcohols	Glycerol fermenation to 1,3-propanediol	Glycerol dehydratase reactivation factor large subunit	0	0	0	0	0	0	0	0	0	0	0	0.000276071
Carbohydrates	Sugar alcohols	Glycerol and Glycerol-3-phosphate Uptake and Utilization	Glycerol uptake operon antiterminator regulatory protein	0	0	0	0	0	0	0	0	0	0	0	0.000157755
Stress Response	Osmotic stress	Choline and Betaine Uptake and Betaine Biosynthesis	Glycine betaine ABC transport system permease protein	0	0	0	0	0	0	0	0	0	0	0	0.000236633
Cell Wall and Capsule	Gram-Negative cell wall components	Core Oligosaccharide Glycosylation in Pseudomonas	Glycosyl transferase in large core OS assembly cluster	0	0	0	0	0	0	0	0	0	0	0	0.000591581
Cell Wall and Capsule	Capsular and extracellular polysacchrides	Capsular Polysaccharides Biosynthesis and Assembly	Glycosyl transferase, group 1 family protein (EC 2.4.1.-)	0	0.004524	0	0	0	0	0	0	0	0	0	0
Fatty Acids, Lipids, and Isoprenoids	Isoprenoids	Polyprenyl Diphosphate Biosynthesis	Glycosyl-4,4'-diaponeurosporenoate acyltransferase precursor (EC 2.3.1.-)	0	0	0	0	0	0	0	0	0	0	0	7.88775E-05
Cell Wall and Capsule	Gram-Negative cell wall components	Core Oligosaccharide Glycosylation in Pseudomonas	Glycosyltransferase (EC 2.4.1.-) in large core OS assembly cluster	0	0	0	0	0	0	0	0	0	0	0	0.000157755
Cell Wall and Capsule	Gram-Positive cell wall components	Polyglycerolphosphate lipoteichoic acid biosynthesis	Glycosyltransferase LafA, responsible for the formation of Glc-DAG	0	0	0	0	0	0	0	0	0	0	0	7.88775E-05
Protein Metabolism	Protein biosynthesis	tRNA aminoacylation, Gly	Glycyl-tRNA synthetase (EC 6.1.1.14), mitochondrial	0	0	0	0	0	0	0	0	0	0	0	7.88775E-05
Protein Metabolism	Protein biosynthesis	Nucleolar protein complex	Growth-arrest-specific protein 8	0	0	0	0	0	0	0	0	0	0	0	3.94388E-05
Protein Metabolism	Protein biosynthesis	Universal GTPases	GTPase Nug1p, which associates with pre-60S ribosomal subunits in the nucleolus and is required for their export from the nucleus	0	0	0	0	0	0	0	0	0	0	0	7.88775E-05
Regulation and Cell signaling	Signal transduction in Eukaryotes	P38 MAP kinase pathways	GTPase Rac1	0	0	0	0	0	0	0	0	0	0	0	3.94388E-05
Iron acquisition and metabolism	NULL	Heme, hemin uptake and utilization systems in GramNegatives	Haemin uptake system periplasmic haemin-binding protein	0	0	0	0	0	0	0	0	0	0	0	3.94388E-05
Secondary Metabolism	Aromatic amino acids and derivatives	Cinnamic Acid Degradation	Hca operon (3-phenylpropionic acid catabolism) transcriptional activator HcaR	0	0	0	0	0	0	0	0	0.000219	0	0	0
Iron acquisition and metabolism	NULL	Heme, hemin uptake and utilization systems in GramPositives	Heme ABC type transporter HtsABC, permease protein HtsB	0	0	0	0	0	0	0	0	0	0	0.000116	0
Iron acquisition and metabolism	NULL	Heme, hemin uptake and utilization systems in GramPositives	Heme transporter analogous to IsdDEF, ATP-binding protein	0	0	0	0	0	0	0	0	0	0	0	7.88775E-05
Iron acquisition and metabolism	NULL	Heme, hemin uptake and utilization systems in GramPositives	Heme transporter IsdDEF, permease component IsdF	0	0	0	0	0	0	0	0	0	0	0	3.94388E-05
Iron acquisition and metabolism	NULL	Heme, hemin uptake and utilization systems in GramNegatives	heme uptake regulator	0	0	0	0	0	0	0	0.000498	0	0	0	0
Iron acquisition and metabolism	NULL	Heme, hemin uptake and utilization systems in GramNegatives	heme uptake transmembrane sensor	0	0	0	0	0	0	0	0	0	0	0	3.94388E-05
Iron acquisition and metabolism	NULL	Campylobacter Iron Metabolism	Hemerythrin-like iron-binding protein	0	0	0	0	0	0	0	0	0.000219	0	0	0
Clustering-based subsystems	NULL	CBSS-83333.1.peg.946	hemimethylated DNA binding protein YccV	0	0	0	0	0	0	0.000722	0	0	0	0	0
Iron acquisition and metabolism	NULL	Hemin transport system	Hemin uptake protein	0	0	0	0	0	0	0	0	0	0	0	3.94388E-05
Iron acquisition and metabolism	NULL	Heme, hemin uptake and utilization systems in GramPositives	Hemoglobin-dependent two component system, sensory histidine kinase HrrS	0	0	0	0	0	0	0	0	0	0	0	3.94388E-05
Cell Wall and Capsule	Capsular and extracellular polysacchrides	Vibrio Polysaccharide (VPS) Biosynthesis	Hemolysin-related protein RbmC	0	0	0	0	0	0	0	0	0	0	0	0.000157755
Cell Division and Cell Cycle	NULL	Heterocyst formation in cyanobacteria	Heterocyst differentiation protein HetP	0	0	0	0	0	0	0	0	0.000438	0	0	0
Respiration	Electron accepting reactions	Anaerobic respiratory reductases	Heterodisulfide reductase, subunit A	0	0	0	0	0.000657	0	0	0	0	0	0	0
Carbohydrates	Monosaccharides	Hexose Phosphate Uptake System	Hexose phosphate transport protein UhpT	0	0	0	0	0	0.000487	0	0	0	0	0	0
Dormancy and Sporulation	NULL	Persister Cells	HipB protein	0	0	0	0	0	0	0	0	0.000219	0	0	0

Miscellaneous	Plant-Prokaryote DOE project	At5g48545 and At3g56490 At1g31160	Histidine triad nucleotide-binding protein 2 (HINT2)	0	0	0	0	0	0	0	0	0	0	0	7.88775E-05
Protein Metabolism	Protein biosynthesis	tRNA aminoacylation, His	Histidyl-tRNA synthetase (EC 6.1.1.21), chloroplast	0	0	0	0	0	0	0	0	0	0	0	3.94388E-05
Phages, Prophages, Transposable elements, Plasmids	Phages, Prophages	r1t-like streptococcal phages	Human platelet-binding protein	0	0	0	0	0	0	0	0	0	0	0	7.88775E-05
Virulence, Disease and Defense	NULL	Bacterial cyanide production and tolerance mechanisms	Hydrogen cyanide synthase HcnB	0	0	0	0.00249	0	0	0	0	0	0	0	0
Respiration	NULL	Formate hydrogenase	Hydrogenase-4 component A (EC 1.-.-.-)	0	0	0	0	0	0	0.000722	0	0	0	0	0
Respiration	NULL	Formate hydrogenase	Hydrogenase-4 component B (EC 1.-.-.-)	0	0	0	0	0	0	0	0	0	0	0	3.94388E-05
Respiration	NULL	Formate hydrogenase	Hydrogenase-4 component H (EC 1.-.-.-)	0	0	0	0	0	0	0	0	0	0	0	7.88775E-05
Metabolism of Aromatic Compounds	NULL	Benzoate transport and degradation cluster	Hydroxybenzoyl-CoA reductase subunit	0	0	0	0	0	0	0	0	0	0	0	0.000236633
Carbohydrates	NULL	Unknown carbohydrate utilization (cluster Ydj)	Hypothetical oxidoreductase YdjG (EC 1.-.-.-)	0	0	0	0	0	0	0	0	0	0	0	0.000118316
Miscellaneous	Plant-Prokaryote DOE project	COG3533	Hypothetical phosphoglucomutase family hydrolase in arabinose utilization cluster	0	0	0	0	0	0	0	0	0.000438	0	0	0
Miscellaneous	Plant-Prokaryote DOE project	DOE COG3533	Hypothetical protein ass w/ COG3533Xanth	0	0	0	0	0	0	0	0	0	0	0	3.94388E-05
Iron acquisition and metabolism	NULL	Heme, hemin uptake and utilization systems in GramPositives	Hypothetical protein GBAA1985 associated with anthrachelin biosynthesis, unique	0	0	0	0	0	0	0.000722	0	0	0	0	0
Carbohydrates	Central carbohydrate metabolism	Glycolate, glyoxylate interconversions	Hypothetical protein GlcG in glycolate utilization operon	0	0	0	0	0	0	0	0	0	0	0	7.88775E-05
Dormancy and Sporulation	NULL	Bacillus biofilm matrix protein component TasA and homologs	Hypothetical protein in cluster with SinR and SinI	0	0	0	0	0	0	0	0	0	0	0	7.88775E-05
Clustering-based subsystems	Hypothetical protein possible functionally linked with Alanyl-tRNA synthetase	CBSS-257314.1.peg.488	Hypothetical protein possible functionally linked with Alanyl-tRNA synthetase	0	0	0	0	0	0	0	0	0	0	0	3.94388E-05
Respiration	NULL	Formate hydrogenase	Hypothetical protein probably related to Formate dehydrogenase function	0	0	0	0	0	0	0	0	0	0	0	7.88775E-05
Clustering-based subsystems	NULL	CBSS-83332.1.peg.3803	hypothetical protein Rv3805c	0	0	0.002555	0	0	0	0	0	0	0	0	0
RNA Metabolism	RNA processing and modification	Ribonuclease H	hypothetical protein ssl1918	0	0	0	0	0.00197	0	0	0	0	0	0	0
Cell Wall and Capsule	Capsular and extracellular polysacchrides	Vibrio Polysaccharide (VPS) Biosynthesis	Hypothetical protein VpsJ	0	0	0	0	0	0	0	0	0	0	0	0.000236633
Phages, Prophages, Transposable elements, Plasmids	Phages, Prophages	Listeria phi-A118-like prophages	Hypothetical protein, Lmo2306 homolog [Bacteriophage A118]	0	0	0	0	0	0	0	0	0.000219	0	0	0
Phages, Prophages, Transposable elements, Plasmids	Phages, Prophages	Staphylococcal phi-Mu50B-like prophages	Hypothetical protein, PV83 orf22 homolog [SA bacteriophages 11, Mu50B]	0	0	0	0	0	0.000487	0	0	0	0	0	0
Iron acquisition and metabolism	NULL	Heme, hemin uptake and utilization systems in GramPositives	Hypothetical similar to CcmC, putative heme lyase for CcmE	0	0	0	0	0	0	0	0	0	0	0	0.000157755
Clustering-based subsystems	NULL	LMPTP YwlE cluster	Hypothetical transmembrane protein ywlD	0	0	0	0	0	0	0	0	0	0	0	0.000552143
Amino Acids and Derivatives	Proline and 4-hydroxyproline	Proline, 4-hydroxyproline uptake and utilization	Hypothetical, similar to sarcosine oxidase alpha subunit, 2Fe-2S domain	0	0	0	0	0	0	0	0	0	0	0	0.000118316
Membrane Transport	Protein and nucleoprotein secretion system, Type IV	Dot-Icm type IV secretion system	IcmD (DotP) protein	0	0	0	0	0	0	0	0.000498	0	0	0	0
Membrane Transport	Protein and nucleoprotein secretion system, Type IV	Dot-Icm type IV secretion system	IcmG protein	0	0	0	0	0	0	0.000722	0	0	0	0	0
Membrane Transport	Protein and nucleoprotein secretion system, Type IV	Dot-Icm type IV secretion system	IcmJ (DotN) protein	0	0	0	0	0	0	0	0	0	0	0.000116	0
Membrane Transport	Protein and nucleoprotein secretion system, Type IV	Dot-Icm type IV secretion system	IcmP (DotM) protein	0	0	0	0	0	0	0	0.000498	0	0	0	0
Membrane Transport	Protein and nucleoprotein secretion system, Type IV	Dot-Icm type IV secretion system	IcmW protein	0	0	0	0	0	0	0	0.000997	0	0	0	0
Membrane Transport	Protein and nucleoprotein secretion system, Type IV	Dot-Icm type IV secretion system	IcmX protein	0	0	0	0	0	0	0	0.000997	0	0	0	0
Nucleosides and Nucleotides	Purines	De Novo Purine Biosynthesis	IMP cyclohydrolase (EC 3.5.4.10) [alternate form]	0	0	0	0	0	0	0	0	0	0	0	0.000118316
Membrane Transport	Protein and nucleoprotein secretion system, Type IV	Conjugative transfer	IncF plasmid conjugative transfer pilus assembly protein TraE	0	0	0	0	0	0	0	0	0.000219	0	0	0
Clustering-based subsystems	NULL	Conjugative transfer related cluster	IncF plasmid conjugative transfer pilus assembly protein TraL	0	0	0	0	0	0	0	0	0	0	0	0.000236633
Clustering-based subsystems	NULL	Conjugative transfer related cluster	IncF plasmid conjugative transfer pilus assembly protein TraW	0	0	0	0	0	0	0	0	0	0	0	0.00031551
Membrane Transport	Protein and nucleoprotein secretion system, Type IV	Conjugative transfer	IncF plasmid conjugative transfer protein TrbC	0	0	0	0	0	0	0	0	0.000219	0	0	0
Membrane Transport	Protein and nucleoprotein secretion system, Type IV	Type 4 conjugative transfer system, IncI1 type	IncI1 plasmid conjugative transfer protein TraU	0	0	0	0	0	0	0	0.000498	0	0	0	0
Membrane Transport	Protein and nucleoprotein secretion system, Type IV	Conjugative transfer	IncQ plasmid conjugative transfer DNA nicking endonuclease TraR (pTi VirD2 homolog)	0	0	0	0	0	0	0	0	0	0	0	0.000118316
Membrane Transport	Protein and nucleoprotein secretion system, Type IV	Conjugative transfer	IncQ plasmid conjugative transfer DNA primase TraO (pTi TraA homolog)	0	0	0	0	0	0	0	0	0	0	0	7.88775E-05
Membrane Transport	Protein and nucleoprotein secretion system, Type IV	Conjugative transfer	IncQ plasmid conjugative transfer protein TraQ (RP4 TrbM homolog)	0	0	0	0	0.000657	0	0	0	0	0	0	0
Membrane Transport	Protein and nucleoprotein secretion system, Type IV	Conjugative transfer	IncW plasmid conjugative protein TrwB (TraD homolog)	0	0	0	0	0	0	0	0	0	0	0	3.94388E-05
Carbohydrates	NULL	Unknown carbohydrate utilization (cluster Ydj)	Inner membrane protein YeaI	0	0	0	0	0	0	0	0	0	0	0	3.94388E-05
Clustering-based subsystems	NULL	Conserved cluster in Enterobacteriaceae downstream from YqjA, a DedA family protein	Inner membrane protein YhaI	0	0	0	0	0	0	0	0	0	0	0	3.94388E-05
Cell Wall and Capsule	Gram-Negative cell wall components	Inner membrane protein YhjD and conserved cluster involved in LPS biosynthesis	Inner membrane protein YhjD	0	0.002262	0	0	0	0	0	0	0	0	0	0
Carbohydrates	NULL	Unknown sugar utilization (cluster yphABCDEFG)	Inner membrane protein YphA	0	0	0	0	0	0	0	0	0.000219	0	0	0
Stress Response	Detoxification	Uptake of selenate and selenite	Inner membrane transport protein YbaT	0	0	0	0	0	0	0	0	0	0	0	0.000236633
Nucleosides and Nucleotides	NULL	Pseudouridine catabolism	Inner membrane transport protein YeiM, in cluster with pseudouridine metabolism operon	0	0	0	0	0	0	0	0	0	0	0	3.94388E-05
Clustering-based subsystems	NULL	Spore Coat	Inner spore coat protein H	0	0	0	0	0	0	0	0	0	0	0	3.94388E-05
Regulation and Cell signaling	NULL	HPr catabolite repression system	Inorganic pyrophospatase PpaX	0	0	0	0	0	0	0	0	0	0	0	0.000236633
Cell Wall and Capsule	Gram-Negative cell wall components	Major Outer Membrane Proteins	Integral membrane protein YfiB	0	0	0	0	0	0	0	0	0.000438	0	0	0
Phages, Prophages, Transposable elements, Plasmids	Pathogenicity islands	Staphylococcal pathogenicity islands SaPI	Integrase, superantigen-encoding pathogenicity islands SaPI	0	0	0	0	0	0	0	0	0	0	0	7.88775E-05
Phages, Prophages, Transposable elements, Plasmids	Transposable elements	CBSS-203122.12.peg.188	Integrase/recombinase clustered with segregation and condensation protein B	0	0	0	0	0	0	0	0	0	0	0	0.000157755
Virulence, Disease and Defense	Invasion and intracellular resistance	Listeria surface proteins: Internalin-like proteins	Internalin H (LPXTG motif)	0	0	0	0	0	0	0	0	0	0	0	3.94388E-05
Virulence, Disease and Defense	Invasion and intracellular resistance	Listeria surface proteins: Internalin-like proteins	Internalin-like protein (LPXTG motif) Lin0290 homolog	0	0	0	0	0	0	0	0	0	0	0	3.94388E-05
Virulence, Disease and Defense	Invasion and intracellular resistance	Listeria surface proteins: Internalin-like proteins	Internalin-like protein (LPXTG motif) Lin1204 homolog	0	0	0	0	0	0	0	0	0	0	0	3.94388E-05
Virulence, Disease and Defense	Invasion and intracellular resistance	Listeria surface proteins: Internalin-like	Internalin-like protein (LPXTG motif) Lmo0732	0	0.004524	0	0	0	0	0	0	0	0	0	0

		proteins	homolog												
Virulence, Disease and Defense	Invasion and intracellular resistance	Listeria surface proteins: Internalin-like proteins	Internalin-like protein (LPXTG motif) Lmo1136 homolog	0	0	0	0	0	0	0	0.000498	0	0	0	0
Virulence, Disease and Defense	Invasion and intracellular resistance	Listeria surface proteins: Internalin-like proteins	Internalin-like protein (LPXTG motif) Lmo1289 homolog	0	0	0	0	0	0	0	0	0	0	0	0.000157755
Virulence, Disease and Defense	Invasion and intracellular resistance	Listeria surface proteins: Internalin-like proteins	Internalin-like protein (LPXTG motif) Lmo2396 homolog	0	0	0	0	0	0	0	0.000498	0	0	0	0
Membrane Transport	Protein secretion system, Type III	Type III secretion systems	Invasion protein IagB precursor	0	0	0	0	0	0	0	0	0	0	0	7.88775E-05
Iron acquisition and metabolism	Siderophores	Siderophore Anthrachelin	Iron compound ABC uptake transporter permease protein	0	0	0	0	0	0	0	0	0	0	0	7.88775E-05
Iron acquisition and metabolism	Siderophores	Iron siderophore sensor & receptor system	Iron siderophore receptor protein	0	0	0	0	0	0	0	0	0	0	0	0.000157755
Iron acquisition and metabolism	Siderophores	Siderophore [Alcaligin-like]	Iron-siderophore [Alcaligin-like] transport system, ATP-binding component	0	0	0	0	0	0	0	0	0	0	0	3.94388E-05
Iron acquisition and metabolism	Siderophores	Siderophore assembly kit	Iron-siderophore transport system, substrate-binding component	0	0	0	0	0	0	0	0	0	0	0	7.88775E-05
Iron acquisition and metabolism	Siderophores	Alcaligin Siderophore	Iron-sulfur protein in siderophore [Alcaligin] cluster	0	0	0	0	0	0	0	0	0	0	0	7.88775E-05
Iron acquisition and metabolism	Siderophores	Siderophore pyochelin	Isochorismate synthase (EC 5.4.4.2) [pyochelin] siderophore	0	0	0	0	0	0	0	0.000498	0	0	0	0
Amino Acids and Derivatives	Aromatic amino acids and derivatives	Chorismate: Intermediate for synthesis of Tryptophan, PAPA antibiotics, PABA, 3-hydroxyanthranilate and more.	Isochorismate synthase (EC 5.4.4.2) of siderophore biosynthesis	0	0	0	0	0	0	0	0	0	0	0	7.88775E-05
Carbohydrates	Central carbohydrate metabolism	TCA Cycle	Isocitrate dehydrogenase [NAD] subunit II, mitochondrial precursor (EC 1.1.1.41)	0	0	0	0	0	0	0	0	0	0	0	7.88775E-05
Cell Wall and Capsule	NULL	Peptidoglycan Crosslinking of Peptide Stems	L,D-transpeptidase ErfK	0	0	0	0	0	0	0	0	0	0	0	7.88775E-05
Cell Wall and Capsule	NULL	Peptidoglycan Crosslinking of Peptide Stems	L,D-transpeptidase YbiS	0	0	0	0	0	0	0	0	0.000219	0	0	0
Carbohydrates	Di- and oligosaccharides	Lactose and Galactose Uptake and Utilization	Lactose and galactose permease, GPH translocator family	0	0	0	0	0	0	0.000722	0	0	0	0	0
DNA Metabolism	DNA uptake, competence	Late competence	Late competence protein ComC, processing protease	0	0	0	0	0	0	0	0	0	0	0	0.000118316
Sulfur Metabolism	Organic sulfur assimilation	L-Cystine Uptake and Metabolism	L-Cystine ABC transporter, ATP-binding protein TcyC	0	0	0	0	0	0	0	0	0	0	0	3.94388E-05
Sulfur Metabolism	Organic sulfur assimilation	L-Cystine Uptake and Metabolism	L-Cystine ABC transporter, ATP-binding protein TcyN	0	0	0	0	0	0	0	0	0	0	0	3.94388E-05
Virulence, Disease and Defense	Resistance to antibiotics and toxic compounds	Adaptation to d-cysteine	L-Cystine ABC transporter, periplasmic cystine-binding protein	0	0	0	0	0	0	0	0	0	0	0	0.000118316
Sulfur Metabolism	Organic sulfur assimilation	L-Cystine Uptake and Metabolism	L-Cystine ABC transporter, periplasmic cystine-binding protein TcyA	0	0	0	0	0	0	0	0	0	0	0	3.94388E-05
Cell Wall and Capsule	Capsular and extracellular polysacchrides	Legionaminic Acid Biosynthesis	Legionaminic acid biosynthesis protein PtmF	0	0	0	0	0	0	0	0	0	0	0	0.000197194
Photosynthesis	Light-harvesting complexes	Bacterial light-harvesting proteins	Light-harvesting LHII, alpha subunit B	0	0	0	0	0	0	0.000722	0	0	0	0	0
Photosynthesis	Light-harvesting complexes	Bacterial light-harvesting proteins	Light-harvesting LHII, alpha subunit E	0	0	0	0	0	0	0	0	0	0	0	7.88775E-05
Photosynthesis	Light-harvesting complexes	Bacterial light-harvesting proteins	Light-harvesting LHII, beta subunit A	0	0	0	0	0	0	0	0	0.000219	0	0	0
Photosynthesis	Light-harvesting complexes	Bacterial light-harvesting proteins	Light-harvesting LHII, beta subunit E	0	0	0	0	0	0	0	0	0	0	0	3.94388E-05
Clustering-based subsystems	Clustering-based subsystems	CBSS-280355.3.peg.2835	Lin2022 protein	0	0	0	0	0	0	0	0	0	0	0	7.88775E-05
Cell Wall and Capsule	Gram-Negative cell wall components	Vibrio Core Oligosaccharide Biosynthesis	lipopolysaccharide biosynthesis protein, putative	0	0	0	0	0	0	0	0	0	0	0	0.000118316
Membrane Transport	Protein and nucleoprotein secretion system, Type IV	Vir-like type 4 secretion system	Lipoprotein of type IV secretion complex that spans outer membrane and periplasm, VirB7	0	0	0	0	0	0.000487	0	0	0	0	0	0
Phages, Prophages, Transposable elements, Plasmids	Pathogenicity islands	Vibrio pathogenicity island	Lipoprotein, ToxR-activated gene, TagA	0	0	0	0	0	0	0	0	0	0	0.004075	0
Cell Wall and Capsule	Gram-Positive cell wall components	Polyglycerolphosphate lipoteichoic acid biosynthesis	Lipoteichoic acid synthase LtaS Type IIIa	0	0	0	0	0	0	0	0	0.000219	0	0	0
Amino Acids and Derivatives	Lysine, threonine, methionine, and cysteine	Lysine degradation	L-lysine permease	0	0	0	0	0	0	0	0	0.000438	0	0	0
Carbohydrates	Central carbohydrate metabolism	Ethylmalonyl-CoA pathway of C2 assimilation	L-malyl-CoA/beta-methylmalyl-CoA lyase (EC 4.1.3.-), actinobacterial type	0	0	0	0	0	0	0	0	0	0	0	7.88775E-05
Iron acquisition and metabolism	NULL	Heme, hemin uptake and utilization systems in GramPositives	Long-chain-fatty-acid--CoA ligase associated with anthrachelin biosynthesis	0	0	0	0	0	0	0	0	0.000438	0	0	0
Iron acquisition and metabolism	Siderophores	Siderophore Pyoverdine	L-ornithine 5-monooxygenase (EC 1.13.12.-), PvdA of pyoverdin biosynthesis	0	0	0	0	0	0	0	0	0	0	0	3.94388E-05
Carbohydrates	Monosaccharides	L-rhamnose utilization	L-rhamnose operon transcriptional activator RhaR	0	0	0	0	0	0	0	0	0	0	0	3.94388E-05
Carbohydrates	Monosaccharides	L-rhamnose utilization	L-rhamnose-1-dehydrogenase (EC 1.1.1.173)	0	0	0	0	0.000657	0	0	0	0	0	0	0
Carbohydrates	Monosaccharides	L-ascorbate utilization (and related gene clusters)	L-ribulose-5-phosphate 3-epimerase UlaE (EC 5.1.3.22) (L-ascorbate utilization protein E)	0	0	0	0	0	0	0	0	0	0	0	7.88775E-05
Protein Metabolism	Selenoproteins	Selenocysteine metabolism	L-seryl-tRNA(Sec) kinase	0	0	0	0	0	0	0.000722	0	0	0	0	0
Protein Metabolism	Protein biosynthesis	Ribosome LSU chloroplast	LSU ribosomal protein L11p (L12e), chloroplast	0	0	0	0	0	0	0	0	0	0	0	3.94388E-05
Protein Metabolism	Protein biosynthesis	Ribosome LSU eukaryotic and archaeal	LSU ribosomal protein L13e	0	0	0	0	0	0	0	0.000498	0	0	0	0
Protein Metabolism	Protein biosynthesis	Ribosome LSU mitochondrial	LSU ribosomal protein L15p (L27Ae), mitochondrial	0	0	0	0	0.000657	0	0	0	0	0	0	0
Protein Metabolism	Protein biosynthesis	Ribosome LSU chloroplast	LSU ribosomal protein L19p, chloroplast	0	0	0	0	0	0	0	0	0.000219	0	0	0
Protein Metabolism	Protein biosynthesis	Ribosome LSU chloroplast	LSU ribosomal protein L24p (L26e), chloroplast	0	0	0	0	0	0	0	0	0.000219	0	0	0
Protein Metabolism	Protein biosynthesis	Ribosome LSU eukaryotic and archaeal	LSU ribosomal protein L27e	0	0	0	0	0	0	0	0	0	0	0	7.88775E-05
Protein Metabolism	Protein biosynthesis	Ribosome LSU chloroplast	LSU ribosomal protein L33p, chloroplast	0	0	0	0	0	0	0	0	0.000438	0	0	0
Protein Metabolism	Protein biosynthesis	Ribosome LSU eukaryotic and archaeal	LSU ribosomal protein L34e	0	0	0	0	0	0	0	0	0	0	0	3.94388E-05
Protein Metabolism	Protein biosynthesis	Ribosome LSU chloroplast	LSU ribosomal protein L34p, chloroplast	0	0	0	0	0	0	0	0	0.000219	0	0	0
Protein Metabolism	Protein biosynthesis	Ribosome LSU eukaryotic and archaeal	LSU ribosomal protein L36e	0	0	0	0	0	0	0	0	0	0	0	3.94388E-05
Miscellaneous	Plant-Prokaryote DOE project	DOE COG2016	LSU ribosomal protein L37e	0	0	0	0	0	0	0	0	0	0	0	3.94388E-05
Protein Metabolism	Protein biosynthesis	Ribosome LSU eukaryotic and archaeal	LSU ribosomal protein L39e	0	0	0	0	0	0	0	0	0	0	0	0.000157755
Protein Metabolism	Protein biosynthesis	Ribosome LSU mitochondrial	LSU ribosomal protein L3p (L3e), mitochondrial	0	0	0	0	0	0	0	0	0	0	0	3.94388E-05
Protein Metabolism	Protein biosynthesis	Ribosome LSU chloroplast	LSU ribosomal protein L5p (L11e), chloroplast	0	0	0	0	0	0	0	0	0.000219	0	0	0
Protein Metabolism	Protein biosynthesis	Ribosome LSU eukaryotic and archaeal	LSU ribosomal protein L6e	0	0	0	0	0	0	0	0	0	0	0	3.94388E-05
Miscellaneous	NULL	Luciferases	LuxE, long-chain-fatty-acid ligase (EC 6.2.1.19)	0	0	0	0	0	0	0	0	0	0	0	0.000197194
Carbohydrates	Monosaccharides	L-ascorbate utilization (and related gene clusters)	L-xylulose/3-keto-L-gulonate kinase (EC 2.7.1.-)	0	0	0	0	0	0	0	0	0.000438	0	0	0

Miscellaneous	NULL	ZZ gjo need homes	LysR family transcriptional regulator clustered with dicarboxylate transport	0	0	0	0	0	0	0	0	0.000438	0	0	0
Regulation and Cell signaling	NULL	DNA-binding regulatory proteins, strays	LysR family transcriptional regulator PA0739	0	0	0	0	0	0	0	0	0	0	0	3.94388E-05
Regulation and Cell signaling	NULL	DNA-binding regulatory proteins, strays	LysR family transcriptional regulator PA2758	0	0	0	0	0	0	0.000722	0	0	0	0	0
Regulation and Cell signaling	NULL	DNA-binding regulatory proteins, strays	LysR family transcriptional regulator YdcI	0	0	0	0	0	0	0	0	0	0	0	3.94388E-05
Cell Wall and Capsule	Gram-Negative cell wall components	Inner membrane protein YhjD and conserved cluster involved in LPS biosynthesis	LysR family transcriptional regulator YhjC	0	0	0	0	0	0	0	0	0	0	0	3.94388E-05
Regulation and Cell signaling	NULL	DNA-binding regulatory proteins, strays	LysR family transcriptional regulator YneJ	0	0	0	0	0	0	0	0.000498	0	0	0	0
Carbohydrates	Central carbohydrate metabolism	Glyoxylate bypass	Malate dehydrogenase (EC 1.1.1.37), chloroplast precursor	0	0	0	0	0	0	0	0	0	0	0	3.94388E-05
Carbohydrates	Central carbohydrate metabolism	Glyoxylate bypass	Malate dehydrogenase (EC 1.1.1.37), peroxisomal	0	0	0	0	0	0	0	0	0.000219	0	0	0
Carbohydrates	One-carbon Metabolism	Serine-glyoxylate cycle	Malate synthase-related protein	0	0	0	0	0	0	0	0	0	0	0.002445	0
Carbohydrates	Organic acids	Malonate decarboxylase	Malonate decarboxylase delta subunit	0	0	0	0	0	0	0	0.000498	0	0	0	0
Carbohydrates	Organic acids	Malonate decarboxylase	Malonate decarboxylase gamma subunit	0	0	0	0	0	0	0	0	0	0	0	0.000473265
Carbohydrates	Di- and oligosaccharides	Maltose and Maltodextrin Utilization	Maltose regulon regulatory protein MalI (repressor for malXY)	0	0	0	0	0	0	0	0	0	0	0	3.94388E-05
Carbohydrates	Di- and oligosaccharides	Maltose and Maltodextrin Utilization	Maltose/maltodextrin ABC transporter 2, permease protein MalG2	0	0	0	0	0	0	0	0	0	0	0	0.000118316
Carbohydrates	Sugar alcohols	Mannitol Utilization	Mannitol operon activator, BglG family	0	0	0	0	0	0	0	0	0	0	0	0.00031551
Membrane Transport	NULL	Agrobacterium opine transport	Mannopine transporter permease protein MotC	0	0	0	0	0	0	0	0	0	0	0	7.88775E-05
Membrane Transport	NULL	Ton and Tol transport systems	Mannosides-regulated TonB-dependent outer membrane receptor	0	0	0	0	0	0	0	0	0	0	0	0.000276071
Regulation and Cell signaling	Signal transduction in Eukaryotes	P38 MAP kinase pathways	MAP kinase ERK5	0	0	0	0	0	0	0	0	0	0	0	3.94388E-05
Regulation and Cell signaling	Signal transduction in Eukaryotes	P38 MAP kinase pathways	MAP kinase JNK1	0	0	0	0	0	0	0	0	0	0	0	7.88775E-05
Regulation and Cell signaling	Signal transduction in Eukaryotes	P38 MAP kinase pathways	MAP kinase kinase MAP2K4	0	0	0	0	0	0	0	0	0	0	0	3.94388E-05
Regulation and Cell signaling	Signal transduction in Eukaryotes	P38 MAP kinase pathways	MAP kinase p38 beta	0	0	0	0	0	0	0	0	0.000438	0	0	0
Regulation and Cell signaling	Signal transduction in Eukaryotes	P38 MAP kinase pathways	MAP kinase p38 delta	0	0	0	0	0	0	0	0	0	0	0	3.94388E-05
Regulation and Cell signaling	Signal transduction in Eukaryotes	P38 MAP kinase pathways	MAP kinase p38 gamma	0	0	0	0	0	0	0	0	0	0	0	0.000157755
Virulence, Disease and Defense	Resistance to antibiotics and toxic compounds	Multidrug Resistance Operon mdtRP of Bacillus	MdtR transcriptional regulator, MarR family	0	0	0	0	0.000657	0	0	0	0	0	0	0
DNA Metabolism	DNA replication	DNA topoisomerases, Type II, ATP-dependent	Meiosis-specific DNA cleavage protein SPO11	0	0	0	0	0	0	0	0	0	0	0	0.00031551
Cell Division and Cell Cycle	NULL	Intracellular septation in Enterobacteria	Membrane protein YciC, linked to IspA	0	0	0	0	0	0.000487	0	0	0	0	0	0
Virulence, Disease and Defense	Resistance to antibiotics and toxic compounds	Lysozyme inhibitors	Membrane-bound lysozyme inhibitor of c-type lysozyme	0	0	0	0	0	0	0	0	0	0	0	3.94388E-05
Virulence, Disease and Defense	Resistance to antibiotics and toxic compounds	Mercury resistance operon	Mercuric resistance operon coregulator	0	0	0	0	0	0	0	0	0	0	0	3.94388E-05
Virulence, Disease and Defense	Resistance to antibiotics and toxic compounds	Mercury resistance operon	Mercuric transport protein, MerC	0	0	0	0	0	0	0	0	0	0	0	0.000118316
Clustering-based subsystems	NULL	ClpS and metallo-protease cluster in Cyanos	Metal-dependent membrane protease, abortive infection protein	0	0	0	0	0	0	0	0	0.000219	0	0	0
Iron acquisition and metabolism	NULL	Heme, hemin uptake and utilization systems in GramPositives	Metallo-beta-lactamase superfamily domain protein in prophage	0	0	0	0	0	0	0	0	0	0	0	7.88775E-05
Carbohydrates	Central carbohydrate metabolism	Soluble methane monooxygenase (sMMO)	Methane monooxygenase component A alpha chain (EC 1.14.13.25)	0	0	0	0	0	0	0	0	0	0	0	0.000157755
Carbohydrates	Central carbohydrate metabolism	Soluble methane monooxygenase (sMMO)	Methane monooxygenase regulatory protein B	0	0	0	0	0	0	0	0.000498	0	0	0	0
Respiration	Electron donating reactions	NiFe hydrogenase maturation	Methanophenazine hydrogenase large subunit (EC 1.12.98.3)	0	0	0	0	0	0	0	0	0	0	0	0.000118316
Respiration	Electron donating reactions	Methanophenazine hydrogenase	Methanophenazine hydrogenase maturation protease (EC 3.4.24.-)	0	0.002262	0	0	0	0	0	0	0	0	0	0
Cell Wall and Capsule	Cell wall of Mycobacteria	mycolic acid synthesis	Methoxy mycolic acid synthase 1 MmaA1 (EC 2.1.1.-)	0	0	0	0	0	0	0	0	0	0	0	3.94388E-05
Carbohydrates	One-carbon Metabolism	Methanogenesis	Methyl coenzyme M reductase beta subunit (EC 2.8.4.1)	0	0	0	0	0	0	0	0	0	0	0	0.00031551
Carbohydrates	One-carbon Metabolism	Methanogenesis	Methyl coenzyme M reductase gamma subunit (EC 2.8.4.1)	0	0	0	0	0	0	0	0	0	0	0	7.88775E-05
Carbohydrates	One-carbon Metabolism	Methanogenesis	Methyl coenzyme M reductase I alpha subunit (EC 2.8.4.1)	0	0	0	0	0	0	0	0	0	0	0	3.94388E-05
Carbohydrates	One-carbon Metabolism	Methanogenesis	Methyl coenzyme M reductase I gamma subunit (EC 2.8.4.1)	0	0	0	0	0	0	0	0	0	0	0	3.94388E-05
Carbohydrates	One-carbon Metabolism	Methanogenesis	Methyl coenzyme M reductase II alpha subunit (EC 2.8.4.1)	0	0	0	0	0	0	0	0	0	0	0	7.88775E-05
Carbohydrates	One-carbon Metabolism	Methanogenesis	Methyl coenzyme M reductase operon protein D	0.001044	0	0	0	0	0	0	0	0	0	0	0
Membrane Transport	NULL	Agrobacterium opine transport	Methylaccepting chemotaxis to opines protein MclA	0	0	0	0	0	0	0	0	0	0	0	3.94388E-05
Miscellaneous	Plant-Prokaryote DOE project	COG0523	methylcobamide:CoM methyltransferase isozyme M	0	0	0	0	0	0	0	0	0	0	0	0.000197194
Miscellaneous	NULL	ZZ gjo need homes	MFS superfamily export protein YceL	0	0	0	0	0	0	0	0	0	0	0	7.88775E-05
Carbohydrates	Di- and oligosaccharides	Lactose and Galactose Uptake and Utilization	Mgl repressor and galactose ultrainduction factor GalS, HTH-type transcriptional regulator	0	0	0	0	0	0	0	0	0	0.002203	0	0
Miscellaneous	NULL	ZZ gjo need homes	Microcompartment protein protein similar to PduA/PduJ	0	0	0	0	0	0	0	0	0	0	0	3.94388E-05
Membrane Transport	Protein secretion system, Type VIII (Extracellular nucleation/precipitation pathway, ENP)	Curli production	Minor curlin subunit CsgB, nucleation component of curlin monomers	0	0	0	0	0	0	0	0	0.000219	0	0	0
Protein Metabolism	Protein degradation	Protein degradation	Mitochondrial intermediate peptidase, mitochondrial precursor (EC 3.4.24.59)	0	0	0	0	0	0	0	0	0	0	0	3.94388E-05
Phages, Prophages, Transposable elements, Plasmids	Transposable elements	CBSS-203122.12.peg.188	Mll9366 protein	0	0	0	0	0	0	0	0	0	0	0	7.88775E-05
Clustering-based subsystems	NULL	CBSS-235.1.peg.567	Mlr0777 protein	0	0	0	0	0	0	0	0	0	0	0	0.000118316
Carbohydrates	One-carbon Metabolism	Methanogenesis from methylated compounds	Monomethylamine:corrinoid methyltransferase	0	0	0	0	0	0	0	0	0	0	0	0.000197194
Clustering-based subsystems	Carbohydrates	Predicted mycobacterial monooxygenase	Monooxygenase component A	0	0	0	0	0	0	0	0	0.001095	0	0	0
Motility and Chemotaxis	Flagellar motility in Prokaryota	Flagellar motility	Motility integral membrane protein	0	0	0	0	0	0	0	0	0	0	0	7.88775E-05
Virulence, Disease and Defense	Resistance to antibiotics and toxic compounds	MexA-MexB-OprM Multidrug Efflux System	Multidrug efflux transporter MexB	0	0	0	0	0	0	0	0	0	0	0	3.94388E-05
Virulence, Disease and Defense	Resistance to antibiotics and toxic compounds	The mdtABCD multidrug resistance cluster	Multidrug transporter MdtD	0	0	0	0	0	0	0	0	0.000219	0	0	0

Carbohydrates	NULL	Sugar utilization in Thermotogales	Myo-inositol ABC transporter, ATP-binding protein InoK	0	0	0	0	0	0	0	0	0	0	0	0.000236633
Carbohydrates	NULL	Sugar utilization in Thermotogales	Myo-inositol ABC transporter, permease protein InoF	0	0	0	0	0	0	0	0	0.000438	0	0	0
Carbohydrates	NULL	Sugar utilization in Thermotogales	Myo-inositol ABC transporter, permease protein InoG	0	0	0	0	0	0	0	0	0	0	0	7.88775E-05
Regulation and Cell signaling	Quorum sensing and biofilm formation	Quorum sensing in Yersinia	N-3-oxododecanoyl-L-homoserine lactone quorum-sensing transcriptional activator	0	0	0	0	0	0	0	0.000498	0	0	0	0
Regulation and Cell signaling	Quorum sensing and biofilm formation	Quorum sensing in Yersinia	N-3-oxohexanoyl-L-homoserine lactone quorum-sensing transcriptional activator	0	0	0	0	0	0	0	0	0	0	0	3.94388E-05
Carbohydrates	One-carbon Metabolism	Methanogenesis	N5-methyltetrahydromethanopterin:coenzyme M methyltransferase subunit B (EC 2.1.1.86)	0	0	0	0.00498	0	0	0	0	0	0	0	0
Carbohydrates	One-carbon Metabolism	Methanogenesis	N5-methyltetrahydromethanopterin:coenzyme M methyltransferase subunit C (EC 2.1.1.86)	0	0	0	0	0	0	0	0	0	0	0	7.88775E-05
Carbohydrates	One-carbon Metabolism	Methanogenesis	N5-methyltetrahydromethanopterin:coenzyme M methyltransferase subunit X	0	0.006786	0	0	0	0	0	0	0	0	0	0
Cell Wall and Capsule	Capsular and extracellular polysacchrides	Sialic Acid Metabolism	N-acetylneuraminate mutarotase	0	0	0.002555	0	0	0	0	0	0	0	0	0
Cell Wall and Capsule	Capsular and extracellular polysacchrides	Sialic Acid Metabolism	N-acetylneuraminic acid outer membrane channel protein NanC	0	0	0	0	0	0.000974	0	0	0	0	0	0
Regulation and Cell signaling	Quorum sensing and biofilm formation	Acyl Homoserine Lactone (AHL) Autoinducer Quorum Sensing	N-acyl-L-homoserine lactone synthetase LasI	0	0	0	0	0	0	0	0	0	0	0	7.88775E-05
Regulation and Cell signaling	Quorum sensing and biofilm formation	Acyl Homoserine Lactone (AHL) Autoinducer Quorum Sensing	N-acyl-L-homoserine lactone synthetase RhlL	0	0	0	0	0	0	0	0	0	0	0	3.94388E-05
Regulation and Cell signaling	Quorum sensing and biofilm formation	Acyl Homoserine Lactone (AHL) Autoinducer Quorum Sensing	N-acyl-L-homoserine lactone synthetase TraI	0	0	0	0	0	0	0	0	0.000219	0	0	0
Stress Response	Oxidative stress	Redox-dependent regulation of nucleus processes	NAD-dependent deacetylase (EC 3.5.1.-)	0	0	0	0	0	0	0	0	0.000219	0	0	0
Respiration	Electron donating reactions	Respiratory Complex I	NADH dehydrogenase I subunit 5, Involved in photosystem-1 cyclic electron flow	0	0	0	0	0	0	0	0.000498	0	0	0	0
Carbohydrates	Fermentation	Acetone Butanol Ethanol Synthesis	NADH-dependent butanol dehydrogenase (EC 1.1.1.-)	0	0	0	0	0	0	0	0	0	0	0	0.000157755
Carbohydrates	Fermentation	Acetone Butanol Ethanol Synthesis	NADH-dependent butanol dehydrogenase B (EC 1.1.1.-)	0	0	0	0	0	0	0	0	0	0	0	0.000867653
Respiration	Electron donating reactions	Respiratory Complex I	NADH-ubiquinone oxidoreductase chain 4 (EC 1.6.5.3)	0	0	0	0	0	0	0	0	0	0	0	3.94388E-05
Nitrogen Metabolism	NULL	Allantoin Utilization	Negative regulator of allantoin and glyoxylate utilization operons	0	0	0	0	0	0	0	0	0.000219	0	0	0
DNA Metabolism	DNA uptake, competence	Gram Positive Competence	Negative regulator of genetic competence MecA	0	0	0	0	0	0	0	0	0.000219	0	0	0
Membrane Transport	NULL	Transport of Nickel and Cobalt	Nickel ABC transporter, periplasmic nickel-binding protein nikA2 (TC 3.A.1.5.3)	0	0.002262	0	0	0	0	0	0	0	0	0	0
Membrane Transport	NULL	Transport of Nickel and Cobalt	Nickel transport ATP-binding protein nikD2 (TC 3.A.1.5.3)	0	0	0	0	0	0	0	0	0	0	0	7.88775E-05
Membrane Transport	NULL	Transport of Nickel and Cobalt	Nickel transport system permease protein nikB2 (TC 3.A.1.5.3)	0	0	0	0	0	0	0	0	0.000219	0	0	0
Membrane Transport	NULL	Transport of Nickel and Cobalt	Nickel transport system permease protein nikC2 (TC 3.A.1.5.3)	0	0	0	0	0	0	0	0	0	0	0	7.88775E-05
Stress Response	Oxidative stress	Redox-dependent regulation of nucleus processes	Nicotinamide-nucleotide adenylyltransferase (EC 2.7.7.1)	0	0	0	0	0	0	0	0	0	0	0	7.88775E-05
Nitrogen Metabolism	NULL	Nitrogen fixation	NifB-domain protein	0	0	0	0	0	0	0	0	0	0	0	0.000788775
Nitrogen Metabolism	NULL	Nitrate and nitrite ammonification	Nitrite transporter NirC	0	0	0	0	0	0	0	0	0	0	0	0.000118316
Regulation and Cell signaling	NULL	DNA-binding regulatory proteins, strays	Nitrogen assimilation regulatory protein Nac	0	0	0	0	0	0	0	0	0.000219	0	0	0
Nitrogen Metabolism	NULL	Nitrogen fixation	Nitrogenase (iron-iron) alpha chain (EC 1.18.6.1)	0	0	0	0	0	0	0	0	0	0	0	0.000197194
Nitrogen Metabolism	NULL	Nitrogen fixation	Nitrogenase (iron-iron) beta chain (EC 1.18.6.1)	0.002089	0	0	0	0	0	0	0	0	0	0	0
Nitrogen Metabolism	NULL	Nitrogen fixation	Nitrogenase (iron-iron) reductase and maturation protein AnfH	0	0	0	0	0	0	0.000722	0	0	0	0	0
Cofactors, Vitamins, Prosthetic Groups, Pigments	NAD and NADP	NAD and NADP cofactor biosynthesis global	NMN synthetase (EC 6.3.1.-)	0	0	0	0	0	0	0	0	0	0	0	7.88775E-05
Nitrogen Metabolism	NULL	Denitrification	NnrT family protein, required for expression of nitric oxide and nitrite reductases (Nir and Nor)	0	0	0	0	0	0	0	0	0	0	0	3.94388E-05
Cell Wall and Capsule	Gram-Positive cell wall components	Sortase	NPQTN specific sortase B	0	0	0	0	0	0	0	0	0	0	0	0.000118316
Secondary Metabolism	Bacterial cytostatics, differentiation factors and antibiotics	Nonribosomal peptide synthetases (NRPS) in Frankia sp. Ccl3	NRPS module 2 PG-PG-Asn	0	0	0	0	0	0	0.000722	0	0	0	0	0
Clustering-based subsystems	NULL	CBSS-196620.1.peg.2477	N-succinyldiaminopimelate aminotransferase (EC 2.6.1.17)	0	0	0	0	0	0	0	0	0	0	0	3.94388E-05
Nucleosides and Nucleotides	Detoxification	Nudix proteins (nucleoside triphosphate hydrolases)	Nudix dNTPase DR0274 (EC 3.6.1.-)	0	0	0	0	0	0	0	0.000498	0	0	0	0
Nucleosides and Nucleotides	Detoxification	Nudix proteins (nucleoside triphosphate hydrolases)	Nudix dNTPase DR1776 (EC 3.6.1.-)	0	0	0	0	0	0	0	0	0.000219	0	0	0
RNA Metabolism	Transcription	Transcription elongation factors, archaeal	NusA protein homolog 2, archaeal	0	0	0	0	0	0	0.001444	0	0	0	0	0
Fatty Acids, Lipids, and Isoprenoids	Fatty acids	Fatty Acid Biosynthesis FASI	Oleoyl-[acyl carrier protein] thioesterase of FASI (EC 3.1.2.14)	0	0	0	0	0	0	0	0	0	0	0	0.000157755
Protein Metabolism	Protein biosynthesis	tRNA aminoacylation, Cys	O-phosphoseryl-tRNA:Cysteinyl-tRNA synthase	0	0	0	0	0	0	0	0	0	0	0	0.000433826
Cofactors, Vitamins, Prosthetic Groups, Pigments	Tetrapyrroles	Coenzyme B12 biosynthesis	Optional hypothetical component of the B12 transporter BtuM	0	0	0	0	0	0	0	0	0	0	0	0.000276071
Clustering-based subsystems	Shiga toxin cluster	CBSS-194948.1.peg.143	ORF-66	0	0	0	0	0	0	0	0	0	0	0	7.88775E-05
Virulence, Disease and Defense	Resistance to antibiotics and toxic compounds	Mercury resistance operon	Organomercurial lyase (EC 4.99.1.2)	0	0	0	0	0	0	0	0	0	0	0	7.88775E-05
Stress Response	Osmotic stress	Osmoregulation	Osmotically inducible protein OsmY	0	0	0	0	0	0	0	0	0	0	0	7.88775E-05
Iron acquisition and metabolism	NULL	ABC transporter [iron.B12.siderophore.hemin]	Outer membrane (iron.B12.siderophore.hemin) receptor	0	0	0	0	0	0	0	0	0	0	0	3.94388E-05
Iron acquisition and metabolism	Siderophores	Siderophore Pyoverdine	Outer membrane ferripyoverdine receptor FpvA, TonB-dependent	0	0	0	0	0	0	0	0	0	0	0	7.88775E-05
Iron acquisition and metabolism	NULL	Heme, hemin uptake and utilization systems in GramNegatives	Outer membrane hemoglobin utilization protein HmbR	0	0	0	0	0	0	0	0	0	0	0.000116	0
Clustering-based subsystems	NULL	USS-DB-2	Outer membrane protein [USSDB2E]	0	0	0	0	0	0	0	0	0.000219	0	0	0
Cell Wall and Capsule	Capsular and extracellular polysacchrides	Alginate metabolism	outer membrane protein AlgE	0	0	0	0	0	0	0.001444	0	0	0	0	0
Cell Wall and Capsule	Gram-Negative cell wall components	Outer membrane	Outer membrane protein F precursor	0	0	0	0	0	0	0	0	0	0	0	7.88775E-05
Cell Wall and Capsule	Gram-Negative cell wall components	Major Outer Membrane Proteins	Outer membrane protein OmpK	0	0	0	0	0	0	0	0	0	0	0	7.88775E-05
Respiration	Electron accepting reactions	Fe(III) respiration - Shewanella type	outer membrane protein, MtrE	0	0	0	0	0	0	0	0	0	0	0.000233	0
Membrane Transport	Protein secretion system, Type VII (Chaperone/Usher pathway, CU)	The usher protein HtrE fimbrial cluster	Outer membrane usher protein HtrE	0	0	0	0	0	0.000487	0	0	0	0	0	0
Carbohydrates	Di- and oligosaccharides	Beta-Glucoside Metabolism	Outer surface protein of unknown function, cellobiose operon	0	0	0	0	0	0	0	0	0	0	0	7.88775E-05
Virulence, Disease and Defense	NULL	C jejuni colonization of chick caeca	Paralysed flagella protein PflA	0	0	0	0	0	0	0	0	0	0	0	0.004811528

Amino Acids and Derivatives	Arginine; urea cycle, polyamines	Urea decomposition	Partial urea carboxylase (EC 6.3.4.6)	0	0	0	0	0	0	0	0	0	0	0	0.000118316
Carbohydrates	Central carbohydrate metabolism	Particulate methane monooxygenase (pMMO)	Particulate methane monooxygenase C-subunit (EC 1.14.13.25)	0	0	0	0	0	0	0	0	0	0	0	3.94388E-05
Metabolism of Aromatic Compounds	NULL	p-cymene degradation	p-cumate dioxygenase small subunit (CmtAc)	0	0	0	0	0	0	0	0	0	0	0	3.94388E-05
Metabolism of Aromatic Compounds	NULL	p-cymene degradation	p-cumic aldehyde dehydrogenase (CymC) [EC:1.2.1.3]	0	0	0	0	0	0	0	0	0	0	0	0.000118316
RNA Metabolism	RNA processing and modification	16S rRNA modification within P site of ribosome	Penicillin-binding protein 3	0	0	0	0	0	0	0	0	0.000219	0	0	0
Cell Wall and Capsule	NULL	Peptidoglycan Crosslinking of Peptide Stems	Penicillin-binding protein AmpH	0	0	0	0	0	0	0	0	0.000219	0	0	0
Amino Acids and Derivatives	Proline and 4-hydroxyproline	Proline, 4-hydroxyproline uptake and utilization	Peptidyl prolyl 4-hydroxylase, alpha subunit (EC 1.14.11.2)	0	0	0	0	0	0	0	0	0	0	0	3.94388E-05
Amino Acids and Derivatives	Aromatic amino acids and derivatives	Phenylalanine and Tyrosine Branches from Chorismate	Periplasmic aromatic amino acid aminotransferase beta precursor (EC 2.6.1.57)	0	0	0	0	0	0	0	0	0	0	0	0.000236633
Iron acquisition and metabolism	Siderophores	Petrobactin-mediated iron uptake system	Petrobactin ABC transporter, ATP-binding protein	0	0	0	0	0	0	0.000722	0	0	0	0	0
Potassium metabolism	NULL	pH adaptation potassium efflux system	pH adaption potassium efflux system protein PhaF	0	0	0	0	0	0	0	0	0.000219	0	0	0
Phages, Prophages, Transposable elements, Plasmids	Phages, Prophages	Phage regulation of gene expression	Phage anti-termination	0	0	0	0	0	0	0	0	0	0	0	3.94388E-05
Phages, Prophages, Transposable elements, Plasmids	Phages, Prophages	Phage tail fiber proteins	Phage long tail fiber proximal subunit	0	0	0	0	0	0	0	0	0.000219	0	0	0
Phages, Prophages, Transposable elements, Plasmids	Phages, Prophages	Phage packaging machinery	Phage terminase, endonuclease subunit	0	0	0	0	0	0	0	0	0	0	0	7.88775E-05
Phages, Prophages, Transposable elements, Plasmids	Phages, Prophages	Staphylococcal phi-Mu50B-like prophages	Phage terminase, small subunit [SA bacteriophages 11, Mu50B]	0	0	0	0	0	0	0	0	0	0	0	3.94388E-05
Metabolism of Aromatic Compounds	Metabolism of central aromatic intermediates	Protocatechuate branch of beta-ketoadipate pathway	PhaK-like protein	0	0	0	0	0	0	0	0	0	0	0	7.88775E-05
Amino Acids and Derivatives	Aromatic amino acids and derivatives	Aromatic amino acid degradation	Phenylalanine-specific permease	0	0	0	0	0	0.000487	0	0	0	0	0	0
Protein Metabolism	Protein biosynthesis	tRNA aminoacylation, Phe	Phenylalanyl-tRNA synthetase alpha chain (EC 6.1.1.20), mitochondrial	0	0	0	0	0.000657	0	0	0	0	0	0	0
Phosphorus Metabolism	NULL	Phosphate metabolism	Phosphate permease PHO89 (Na( )/Pi cotransporter PHO89)	0	0	0	0	0.000657	0	0	0	0	0	0	0
Cell Wall and Capsule	NULL	UDP-N-acetylmuramate from Fructose-6-phosphate Biosynthesis	Phosphoacetylglucosamine mutase (EC 5.4.2.3)	0	0	0	0	0	0	0	0	0.000219	0	0	0
Miscellaneous	Plant-Prokaryote DOE project	Synechocystis experimental	Phospholipase/carboxylesterase family protein, sll1284 homolog	0	0	0	0	0	0	0	0	0	0	0.000233	0
Amino Acids and Derivatives	NULL	Phosphonoalanine utilization	Phosphonoalanine aminotransferase	0	0	0	0	0	0.000487	0	0	0	0	0	0
Cofactors, Vitamins, Prosthetic Groups, Pigments	Folate and pterines	Methanopterin biosynthesis2	Phosphoribosylformimino-5-aminoimidazole carboxamide ribotide isomerase related protein	0	0	0	0	0	0	0	0.000498	0	0	0	0
Amino Acids and Derivatives	Alanine, serine, and glycine	Serine Biosynthesis	Phosphoserine aminotransferase, putative (EC 2.6.1.52)	0	0	0	0	0	0	0	0	0	0	0	3.94388E-05
Miscellaneous	Plant-Prokaryote DOE project	COG3380 COG2907	Photolyase/blue-light receptor	0	0	0	0	0	0	0	0	0	0	0	3.94388E-05
Photosynthesis	Electron transport and photophosphorylation	Photosystem I	photosystem I assembly related protein Ycf37	0	0	0	0	0	0	0	0	0.000219	0	0	0
Photosynthesis	Electron transport and photophosphorylation	Photosystem I	photosystem I subunit IV (PsaE)	0	0	0	0	0	0	0	0	0.001533	0	0	0
Photosynthesis	Electron transport and photophosphorylation	Photosystem I	photosystem I subunit IX (PsaJ)	0	0	0	0	0	0	0	0	0	0	0	3.94388E-05
Photosynthesis	Electron transport and photophosphorylation	Photosystem II	Photosystem II 10 kDa phosphoprotein (PsbH)	0	0	0	0	0	0	0	0	0.001095	0	0	0
Photosynthesis	Electron transport and photophosphorylation	Photosystem II	Photosystem II manganese-stabilizing protein (PsbO)	0	0	0	0	0	0	0	0	0.001095	0	0	0
Photosynthesis	Electron transport and photophosphorylation	Photosystem II	Photosystem II protein PsbM	0	0	0	0	0	0	0	0	0.000438	0	0	0
Photosynthesis	Electron transport and photophosphorylation	Photosystem I-type photosynthetic reaction center	Photosystem P840 reaction center large subunit PscA	0	0.002262	0	0	0	0	0	0	0	0	0	0
Photosynthesis	Light-harvesting complexes	Phycobilisome	Phycobilisome degradation protein NblA	0	0	0	0	0	0	0	0	0.001095	0	0	0
Photosynthesis	Light-harvesting complexes	Phycobilisome	Phycobilisome small rod capping linker polypeptide, phycocyanin-associated	0	0	0	0	0	0	0	0	0.000876	0	0	0
Photosynthesis	Light-harvesting complexes	Phycobilisome	Phycoerythrin alpha chain	0	0	0	0	0	0	0	0	0	0	0.000233	0
Cofactors, Vitamins, Prosthetic Groups, Pigments	Tetrapyrroles	Bilin Biosynthesis	Phycoerythrobilin:ferredoxin oxidoreductase PebB (EC 1.3.7.3)	0	0	0	0	0	0	0	0	0	0	0.000116	0
Stress Response	Oxidative stress	Glutathione: Non-redox reactions	Phytochelatin synthase (EC 2.3.2.15), eukaryotic type	0	0	0	0	0	0	0	0	0	0	0	7.88775E-05
Membrane Transport	Protein and nucleoprotein secretion system, Type IV	Vir-like type 4 secretion system	Plant-inducible protein PinF1, cytochrome P450-like, contributes to virulence	0	0	0	0	0	0	0.000722	0	0	0	0	0
Secondary Metabolism	Bacterial cytostatics, differentiation factors and antibiotics	2-isocapryloyl-3R-hydroxymethyl-gamma-butyrolactone and other bacterial morphogens	Pleiotropic negative regulator for morphological and physiological development in Streptomyces, BldD	0	0	0	0	0	0	0	0	0	0	0	7.88775E-05
Cell Wall and Capsule	Gram-Positive cell wall components	D-Alanyl Lipoteichoic Acid Biosynthesis	Poly(glycerophosphate chain) D-alanine transfer protein DltD	0	0	0	0	0	0	0	0	0	0	0	7.88775E-05
Fatty Acids, Lipids, and Isoprenoids	NULL	Polyhydroxybutyrate metabolism	polyhydroxyalkanoate granule-associated protein PhaI	0	0	0	0	0	0	0	0	0.000219	0	0	0
Dormancy and Sporulation	NULL	Spore pigment biosynthetic cluster in Actinomycetes	Polyketide beta-ketoacyl synthase WhiE-KS paralog	0	0	0	0	0	0	0	0	0	0	0	3.94388E-05
Dormancy and Sporulation	NULL	Spore pigment biosynthetic cluster in Actinomycetes	Polyketide chain length factor WhiE-CLF paralog	0	0	0	0	0.000657	0	0	0	0	0	0	0
Miscellaneous	Plant-Prokaryote DOE project	At1g24340	Polyketide hydroxylase WhiE VII	0	0	0	0	0	0	0	0	0	0	0	7.88775E-05
Cell Wall and Capsule	Gram-Negative cell wall components	Lipid A-Ara4N pathway (Polymyxin resistance)	Polymyxin resistance protein PmrM	0	0	0	0	0	0	0	0	0	0	0	3.94388E-05
Virulence, Disease and Defense	Resistance to antibiotics and toxic compounds	Polymyxin Synthetase Gene Cluster in Bacillus	Polymyxin synthetase PmxB	0	0	0	0	0	0	0	0	0	0	0	3.94388E-05
Cell Wall and Capsule	Capsular and extracellular polysacchrides	Extracellular Polysaccharide Biosynthesis of Streptococci	Polysaccharide biosynthesis glycosyl transferase CpsJ	0	0	0	0	0	0	0	0	0	0	0	3.94388E-05
Cell Wall and Capsule	Capsular and extracellular polysacchrides	Extracellular Polysaccharide Biosynthesis of Streptococci	Polysaccharide biosynthesis glycosyl transferase CpsO	0	0	0	0	0	0.000487	0	0	0	0	0	0
Miscellaneous	Plant-Prokaryote DOE project	Synechocystis experimental	Possible alpha/beta hydrolase superfamily, tll2163 homolog	0	0	0	0	0	0	0	0	0.000219	0	0	0
Nucleosides and Nucleotides	Pyrimidines	Pyrimidine utilization	Possible hydrolase or acyltransferase RutD in novel pyrimidine catabolism pathway	0	0	0.002555	0	0	0	0	0	0	0	0	0
RNA Metabolism	RNA processing and modification	RNA methylation	Possible RNA methyltransferase aq 1528	0	0	0	0	0	0	0	0	0	0	0	3.94388E-05
Potassium metabolism	NULL	Hyperosmotic potassium uptake	Potassium uptake protein, integral membrane component, KtrD	0	0	0	0.00249	0	0	0	0	0	0	0	0
Potassium metabolism	NULL	Potassium homeostasis	Potassium voltage-gated channel subfamily A member 4	0	0	0	0	0	0	0	0	0	0	0	3.94388E-05
Carbohydrates	Monosaccharides	D-Galacturonate and D-Glucuronate Utilization	Predicted 2-keto-3-deoxygluconate-responsive regulator of glucuronate utilization, IclR family	0	0	0	0	0	0	0	0	0	0	0	3.94388E-05
Carbohydrates	NULL	Sugar utilization in Thermotogales	Predicted aldo/keto reductase in CelR regulon, COG4989	0	0	0	0	0	0.000974	0	0	0	0	0	0
Cofactors, Vitamins, Prosthetic Groups, Pigments	Tetrapyrroles	Coenzyme B12 biosynthesis	Predicted alpha-ribazole-5'-phosphate phosphatase CobZ (EC 3.1.3.73)	0	0.002262	0	0	0	0	0	0	0	0	0	0

Amino Acids and Derivatives	Arginine; urea cycle, polyamines	Arginine Biosynthesis extended	Predicted amino-acid acetyltransferase (EC 2.3.1.1) complementing ArgA function in Arginine Biosynthesis pathway	0	0	0	0	0	0	0	0	0	0	0	7.88775E-05
Cofactors, Vitamins, Prosthetic Groups, Pigments	Biotin	Biotin biosynthesis	predicted biotin regulatory protein BioR (GntR family)	0	0	0	0	0	0	0	0	0	0	0	3.94388E-05
Carbohydrates	NULL	Sugar utilization in Thermotogales	Predicted cellobiose ABC transport system, permease protein 2	0	0	0	0	0	0	0	0	0	0	0	3.94388E-05
Carbohydrates	Aminosugars	Chitin and N-acetylglucosamine utilization	Predicted chitobiose ABC transport system II, permease protein 2	0	0	0	0	0	0	0	0	0.000219	0	0	0
Carbohydrates	NULL	Sugar utilization in Thermotogales	Predicted chitobiose ABC transport system, sugar-binding protein	0	0.002262	0	0	0	0	0	0	0	0	0	0
Carbohydrates	Monosaccharides	D-Galacturonate and D-Glucuronate Utilization	Predicted D-glucuronide-specific TRAP transporter, small transmembrane component	0	0	0	0	0	0	0	0	0	0	0	0.000276071
Carbohydrates	Sugar alcohols	Erythritol utilization	Predicted erythritol ABC transporter 2, hypothetical lipoprotein	0	0	0	0	0	0	0	0	0.000219	0	0	0
Carbohydrates	Sugar alcohols	Erythritol utilization	Predicted erythritol ABC transporter 2, substrate-binding component	0	0	0	0	0	0	0	0	0.000657	0	0	0
Carbohydrates	Monosaccharides	D-Tagatose and Galactitol Utilization	Predicted galactitol operon regulator (Transcriptional antiterminator), BglG family	0	0	0	0	0	0	0	0	0	0	0	3.94388E-05
Carbohydrates	NULL	Lacto-N-Biose I and Galacto-N-Biose Metabolic Pathway	Predicted galacto-N-biose-/lacto-N-biose I ABC transporter, periplasmic substrate-binding protein	0	0	0	0	0	0	0	0	0	0	0	3.94388E-05
Carbohydrates	Di- and oligosaccharides	Lactose and Galactose Uptake and Utilization	Predicted galactoside ABC transporter, permease protein 1	0	0	0	0	0	0	0	0	0	0	0	0.000197194
Carbohydrates	Di- and oligosaccharides	Lactose and Galactose Uptake and Utilization	Predicted galactoside ABC transporter, sugar-binding protein	0	0	0	0	0	0	0	0	0	0	0	3.94388E-05
Carbohydrates	Polysaccharides	Glycogen metabolism	Predicted glycogen debranching enzyme (pullulanase-like, but lacking signal peptide)	0	0	0	0	0	0	0	0	0.000657	0	0	0
Carbohydrates	Organic acids	Lactate utilization	Predicted lactate-responsive transcriptional regulator of ykgEFG LDH gene cluster, LysR-type	0	0	0	0	0	0	0	0	0	0	0	3.94388E-05
Carbohydrates	Central carbohydrate metabolism	Pyruvate Alanine Serine Interconversions	Predicted L-serine dehydratase (EC 4.3.1.17) TdcG	0	0	0	0	0	0	0	0	0.000219	0	0	0
Membrane Transport	NULL	Transport of Molybdenum	Predicted molybdate-responsive regulator YvgK in bacilli	0	0	0	0	0	0	0	0	0	0	0	0.000118316
Carbohydrates	Aminosugars	N-Acetyl-Galactosamine and Galactosamine Utilization	Predicted N-acetylgalactosamine kinase, ROK-type (EC 2.7.1.157)	0	0	0	0	0	0	0	0.000498	0	0	0	0
Carbohydrates	Aminosugars	Chitin and N-acetylglucosamine utilization	predicted N-acetylglucosamine kinase, glucokinase-like (EC 2.7.1.59)	0	0	0	0	0	0	0	0	0	0	0	0.000157755
Cofactors, Vitamins, Prosthetic Groups, Pigments	NAD and NADP	NAD regulation	Predicted nicotinate-regulated transporter BH3254	0	0	0	0	0	0	0	0	0	0	0	0.000197194
Cofactors, Vitamins, Prosthetic Groups, Pigments	NAD and NADP	NAD and NADP cofactor biosynthesis global	Predicted N-ribosylNicotinamide CRP-like regulator	0	0	0	0	0	0	0	0	0	0	0	0.000433826
RNA Metabolism	RNA processing and modification	tRNA modification Archaea	predicted nucleic acid-binding protein, containing PIN domain COG1848	0	0.002262	0	0	0	0	0	0	0	0	0	0
Amino Acids and Derivatives	Arginine; urea cycle, polyamines	Polyamine Metabolism	Predicted polyamine sensor NspS, involved in biofilm formation	0	0	0	0	0	0	0	0	0	0	0	7.88775E-05
Carbohydrates	Di- and oligosaccharides	Beta-Glucoside Metabolism	Predicted regulator of cellobiose and glucan utilization, LacI family	0	0	0	0	0	0	0	0	0	0	0	3.94388E-05
Carbohydrates	Monosaccharides	L-rhamnose utilization	Predicted rhamnogalacturonide-specific TRAP-type transporter, large transmembrane component RhiC	0	0	0	0	0	0	0	0	0	0	0	0.000197194
Carbohydrates	Monosaccharides	L-rhamnose utilization	Predicted rhamnogalacturonide-specific TRAP-type transporter, substrate-binding component RhiA	0	0	0	0	0	0	0.002888	0	0	0	0	0
Membrane Transport	Protein secretion system, Type II	Predicted secretion system W clustering with cell division proteins	Predicted secretion system W ATPase PilM-like	0	0	0	0	0	0	0	0	0	0	0	0.000157755
Membrane Transport	Protein secretion system, Type II	Predicted secretion system X	Predicted secretion system X pseudopilin PulG-like	0	0	0.002555	0	0	0	0	0	0	0	0	0
Carbohydrates	NULL	Unknown sugar utilization (cluster yphABCDEFG)	Predicted sugar ABC transport system, permease protein YphD	0	0	0	0	0	0	0	0	0	0	0	3.94388E-05
Amino Acids and Derivatives	Branched-chain amino acids	Branched chain amino acid degradation regulons	Predicted transcriptional regulator LiuQ of leucine degradation pathway, TetR family	0	0	0	0	0	0.000487	0	0	0	0	0	0
Carbohydrates	Aminosugars	Chitin and N-acetylglucosamine utilization	Predicted transcriptional regulator of N-Acetylglucosamine utilization, LacI family	0	0	0	0	0	0	0	0	0	0.002203	0	0
Photosynthesis	NULL	Proteorhodopsin	Predictet Brp-like protein Blh	0	0	0	0	0	0	0	0	0	0	0.000233	0
RNA Metabolism	RNA processing and modification	Spliceosome	Pre-mRNA splicing factor PRP1	0	0	0	0	0	0	0	0	0	0	0	3.94388E-05
Membrane Transport	Protein translocation across cytoplasmic membrane	SecY2-SecA2 Specialized Transport System	Preprotein translocase SecY2 subunit (TC 3.A.5.1.1)	0	0	0	0	0	0	0	0	0	0	0	7.88775E-05
DNA Metabolism	DNA replication	DNA-replication	Primosomal replication protein N prime	0	0	0	0	0	0	0	0	0	0	0	7.88775E-05
Cell Wall and Capsule	Cell wall of Mycobacteria	mycolic acid synthesis	Probable acyl-[acyl-carrier protein] desaturase DESA1 (Acyl-[ACP] desaturase) (Stearoyl-ACP desaturase) (Protein DES) (EC 1.14.19.2)	0	0	0	0	0	0	0	0	0.000438	0	0	0
Sulfur Metabolism	NULL	Galactosylceramide and Sulfatide metabolism	probable beta-D-galactosidase	0	0	0	0	0	0.000487	0	0	0	0	0	0
Virulence, Disease and Defense	Resistance to antibiotics and toxic compounds	Beta-lactamase	Probable beta-lactamase ybxI precursor (EC 3.5.2.6)	0	0	0	0	0	0	0	0	0	0	0	0.000197194
Cell Wall and Capsule	Cell wall of Mycobacteria	mycolic acid synthesis	PROBABLE DAUNORUBICIN-DIM-TRANSPORT INTEGRAL MEMBRANE PROTEIN ABC TRANSPORTER DRRB	0	0.002262	0	0	0	0	0	0	0	0	0	0
Sulfur Metabolism	Organic sulfur assimilation	Alkanesulfonate assimilation	probable dibenzothiophene desulfurization enzyme	0	0	0	0	0	0	0	0	0	0	0	0.000118316
Iron acquisition and metabolism	Siderophores	Siderophore Pyoverdine	Probable hydrolase, coexpressed with pyoverdine biosynthesis regulon	0	0	0	0	0	0	0	0	0	0	0	3.94388E-05
Clustering-based subsystems	Cytochrome biogenesis	CBSS-196164.1.peg.1690	Probable NADPH:quinone reductase (EC 1.6.5.5)	0	0	0	0	0	0	0	0	0	0	0	3.94388E-05
Virulence, Disease and Defense	Resistance to antibiotics and toxic compounds	Multidrug Resistance Efflux Pumps	Probable outer membrane component of multidrug efflux pump	0	0	0	0	0	0	0	0	0	0	0	0.001735305
Nucleosides and Nucleotides	NULL	Pseudouridine catabolism	Probable pyrimidine nucleoside transport protein associated with pseudouridine catabolism	0	0	0	0	0	0	0	0	0.000219	0	0	0
Cell Wall and Capsule	Gram-Negative cell wall components	Core Oligosaccharide Glycosylation in Pseudomonas	Probable transcription regulator Mig-14	0	0	0	0	0	0	0	0	0	0	0	3.94388E-05
Regulation and Cell signaling	Programmed Cell Death and Toxin-antitoxin Systems	MazEF toxin-antitoxing (programmed cell death) system	Programmed cell death antitoxin MazE	0	0	0	0	0	0	0	0	0	0	0	0.000354949
Regulation and Cell signaling	Programmed Cell Death and Toxin-antitoxin Systems	Phd-Doc, YdcE-YdcD toxin-antitoxin (programmed cell death) systems	Programmed cell death antitoxin MazE like	0	0	0	0	0	0	0	0	0	0	0	3.94388E-05
Protein Metabolism	Protein biosynthesis	Pyrrolysine	Proline reductase for pyrrolysine biosynthesis	0	0	0	0	0	0	0	0	0	0	0	7.88775E-05
Carbohydrates	Sugar alcohols	Propanediol utilization	Propanediol utilization polyhedral body protein PduK	0	0	0	0	0	0	0	0	0	0	0	3.94388E-05
Carbohydrates	Sugar alcohols	Propanediol utilization	Propionate kinase, propanediol utilization (EC 2.7.2.1)	0	0	0	0	0	0	0	0	0	0	0	0.000236633
Protein Metabolism	Protein degradation	Proteasome eukaryotic	proteasome regulatory subunit Rpn1	0	0	0	0	0	0	0	0	0	0	0	0.000236633
Protein Metabolism	Protein degradation	Proteasome eukaryotic	proteasome regulatory subunit Rpn10	0	0	0	0	0	0	0	0	0	0	0	3.94388E-05
Protein Metabolism	Protein degradation	Proteasome eukaryotic	proteasome regulatory subunit Rpn11	0	0	0	0	0	0	0	0	0.000219	0	0	0

Protein Metabolism	Protein degradation	Proteasome eukaryotic	proteasome regulatory subunit Rpn5	0	0	0	0	0	0	0	0	0	0	0	3.94388E-05
Protein Metabolism	Protein degradation	Proteasome eukaryotic	proteasome regulatory subunit Rpn7	0	0	0	0	0.000657	0	0	0	0	0	0	0
Protein Metabolism	Protein degradation	Proteasome eukaryotic	proteasome subunit alpha3 (EC 3.4.25.1)	0	0	0	0	0	0	0	0	0	0	0	0.000118316
Protein Metabolism	Protein degradation	Proteasome eukaryotic	proteasome subunit alpha7 (EC 3.4.25.1)	0	0	0	0	0	0	0	0	0	0	0	7.88775E-05
Protein Metabolism	Protein degradation	Proteasome eukaryotic	proteasome subunit beta1 (EC 3.4.25.1)	0	0	0	0	0	0	0	0.000498	0	0	0	0
Protein Metabolism	Protein degradation	Proteasome eukaryotic	proteasome subunit beta4 (EC 3.4.25.1)	0	0	0	0	0	0	0	0	0	0	0	3.94388E-05
Protein Metabolism	Protein degradation	Proteasome eukaryotic	proteasome subunit beta5 (EC 3.4.25.1)	0	0	0	0	0	0	0	0	0.000438	0	0	0
Protein Metabolism	Protein degradation	Proteasome eukaryotic	proteasome subunit beta6 (EC 3.4.25.1)	0	0	0	0	0	0	0	0	0	0	0	3.94388E-05
Stress Response	NULL	Dimethylarginine metabolism	protein arginine methyltransferase type 2 (SDMA formation)	0	0	0	0	0	0	0.001444	0	0	0	0	0
Carbohydrates	CO2 fixation	Calvin-Benson cycle	Protein CP12, regulation of Calvin cycle via association/dissociation of PRK/CP12/GAPDH complex	0	0	0	0	0	0	0.000722	0	0	0	0	0
Virulence, Disease and Defense	NULL	Streptococcus pyogenes Virulome	Protein H	0	0	0	0	0	0	0	0	0	0	0	0.000197194
Phages, Prophages, Transposable elements, Plasmids	Transposable elements	CBSS-203122.12.peg.188	protein of unknown function DUF1403	0	0	0	0	0	0	0	0	0	0	0	0.000276071
RNA Metabolism	RNA processing and modification	tRNA modification Archaea	protein of unknown function DUF358	0	0	0	0	0	0	0	0	0.000438	0	0	0
Miscellaneous	NULL	ZZ gjo need homes	Protein of unknown function DUF81, type 2	0	0	0	0	0	0	0	0	0	0	0	3.94388E-05
RNA Metabolism	RNA processing and modification	Polyadenylation specificity factor	Protein similar to polyadenylation specificity factor, MA3206 type	0	0	0	0	0	0	0	0	0	0	0	0.000157755
RNA Metabolism	RNA processing and modification	Polyadenylation specificity factor	Protein similar to polyadenylation specificity factor, MJ0162 type	0	0	0	0	0	0	0	0	0	0	0	3.94388E-05
RNA Metabolism	RNA processing and modification	Polyadenylation specificity factor	Protein similar to polyadenylation specificity factor, SSO0188 type	0	0	0	0	0	0	0	0	0	0	0	7.88775E-05
Membrane Transport	Protein secretion system, Type II	Widespread colonization island	Protein TadG, associated with Flp pilus assembly	0	0	0	0	0	0	0	0.000498	0	0	0	0
Miscellaneous	Plant-Prokaryote DOE project	COG3146 experimental	Pterin-4-alpha-carbinolamine dehydratase-like	0	0	0	0	0	0	0	0	0.000219	0	0	0
Miscellaneous	Plant-Prokaryote DOE project	COG3146 experimental	Pterin-binding family	0	0	0	0	0	0	0	0	0.000438	0	0	0
Carbohydrates	Di- and oligosaccharides	Beta-Glucoside Metabolism	PTS system, beta-glucoside-specific IIB component (EC 2.7.1.69)	0	0	0	0	0	0	0	0	0	0	0	3.94388E-05
Carbohydrates	Di- and oligosaccharides	Beta-Glucoside Metabolism	PTS system, beta-glucoside-specific IIC component (EC 2.7.1.69)	0	0	0	0	0	0	0	0	0	0	0	3.94388E-05
Carbohydrates	Di- and oligosaccharides	Beta-Glucoside Metabolism	PTS system, cellobiose-specific IIA component (EC 2.7.1.69)	0	0	0	0	0	0	0	0	0	0	0	3.94388E-05
Carbohydrates	Di- and oligosaccharides	Beta-Glucoside Metabolism	PTS system, cellobiose-specific IIB component (EC 2.7.1.69)	0	0	0	0	0	0	0	0	0	0	0	0.000118316
Carbohydrates	Aminosugars	Chitin and N-acetylglucosamine utilization	PTS system, chitobiose-specific IIA component (EC 2.7.1.69)	0	0	0	0	0.001313	0	0	0	0	0	0	0
Carbohydrates	Monosaccharides	D-Tagatose and Galactitol Utilization	PTS system, galactitol-specific IIB component (EC 2.7.1.69)	0	0	0	0	0	0	0	0	0	0	0	3.94388E-05
Carbohydrates	Monosaccharides	D-Sorbitol(D-Glucitol) and L-Sorbose Utilization	PTS system, glucitol/sorbitol-specific IIA component (EC 2.7.1.69)	0	0	0	0	0	0	0	0	0	0	0	3.94388E-05
Carbohydrates	Monosaccharides	D-Sorbitol(D-Glucitol) and L-Sorbose Utilization	PTS system, glucitol/sorbitol-specific IIC component (EC 2.7.1.69)	0	0	0	0	0	0	0	0	0	0	0	0.000236633
Carbohydrates	Di- and oligosaccharides	Maltose and Maltodextrin Utilization	PTS system, maltose and glucose-specific IIC component (EC 2.7.1.69)	0	0	0	0	0	0	0	0	0	0	0	3.94388E-05
Miscellaneous	Plant-Prokaryote DOE project	Experimental-yggC	PTS system, mannitol-specific cryptic IIA component (EC 2.7.1.69)	0	0	0	0	0	0	0	0	0	0	0	0.000197194
Carbohydrates	Sugar alcohols	Mannitol Utilization	PTS system, mannitol-specific cryptic IIB component (EC 2.7.1.69)	0	0	0	0	0	0	0	0	0	0	0	0.000749336
Carbohydrates	Monosaccharides	Mannose Metabolism	PTS system, mannose-specific IIC component (EC 2.7.1.69)	0	0	0	0	0	0	0	0	0	0	0	0.000157755
Carbohydrates	Aminosugars	N-Acetyl-Galactosamine and Galactosamine Utilization	PTS system, N-acetylgalactosamine- and galactosamine-specific IIA component (EC 2.7.1.69)	0	0	0	0	0	0	0	0	0	0	0	3.94388E-05
Cell Wall and Capsule	Capsular and extracellular polysacchrides	Sialic Acid Metabolism	PTS system, N-acetylmuramic acid-specific IIB component (EC 2.7.1.69)	0	0	0	0	0	0	0	0	0	0	0	7.88775E-05
Carbohydrates	Monosaccharides	D-Tagatose and Galactitol Utilization	PTS system, tagatose-specific IIA-TPr component (EC 2.7.1.69)	0	0	0	0	0	0	0	0	0.000219	0	0	0
Clustering-based subsystems	NULL	Putative hemin transporter	puatative hemin transporter subunit	0	0	0	0	0	0	0	0	0	0	0	3.94388E-05
Nucleosides and Nucleotides	Purines	De Novo Purine Biosynthesis	PurR: transcription regulator associated with purine metabolism	0	0	0	0	0	0	0	0	0	0	0	3.94388E-05
Cofactors, Vitamins, Prosthetic Groups, Pigments	Tetrapyrroles	Coenzyme B12 biosynthesis	Putative 2Fe-2S ferredoxin CbiW involved in B12 biosynthesis	0	0.002262	0	0	0	0	0	0	0	0	0	0
Virulence, Disease and Defense	Adhesion	Adhesion of Campylobacter	Putative ABC-type amino-acid transporter permease protein	0	0	0	0	0	0	0	0	0	0	0	3.94388E-05
Carbohydrates	Central carbohydrate metabolism	HPr kinase and hprK operon in Gram-positive organisms	Putative acetyltransferase YvoF	0	0	0	0	0	0	0	0	0	0	0	3.94388E-05
Carbohydrates	NULL	Unknown carbohydrate utilization (cluster Ydj)	Putative aldolase YdjI	0	0	0	0	0	0	0	0	0	0	0	3.94388E-05
Sulfur Metabolism	Organic sulfur assimilation	Alkanesulfonate assimilation	Putative alkylsulfatase (EC 1.14.11.17)	0	0	0	0	0	0	0	0	0.000219	0	0	0
Cell Wall and Capsule	Capsular and extracellular polysacchrides	O-Methyl Phosphoramidate Capsule Modification in Campylobacter	Putative amidotransferase (Type 1 glutamine amidotransferase - GATase1)	0	0	0	0	0	0	0	0	0	0	0	3.94388E-05
Clustering-based subsystems	NULL	CBSS-196620.1.peg.2477	Putative antibiotic transport-associated protein	0	0	0	0	0	0	0	0	0.000438	0	0	0
Miscellaneous	Plant-Prokaryote DOE project	Conserved gene cluster possibly involved in RNA metabolism	Putative anti-sigma factor antagonist	0	0	0	0	0	0	0	0	0.001095	0	0	0
Respiration	NULL	Biogenesis of c-type cytochromes	Putative cytochrome c-type biogenesis protein related to CcmF	0	0	0	0	0	0	0	0	0	0	0	3.94388E-05
Fatty Acids, Lipids, and Isoprenoids	NULL	Cholesterol catabolic operon in Mycobacteria	Putative cytochrome P450 IgrA	0	0	0	0	0	0	0	0	0	0	0	7.88775E-05
Cofactors, Vitamins, Prosthetic Groups, Pigments	Folate and pterines	Folate Biosynthesis	Putative DHNTP pyrophosphatase	0	0	0	0	0.000657	0	0	0	0	0	0	0
Clustering-based subsystems	Choline bitartrate degradation, putative	CBSS-344610.3.peg.2335	Putative dioxygenase, beta subunit (EC 1.-.-.-)	0	0	0	0	0	0	0	0	0	0	0.000349	0
Membrane Transport	Protein translocation across cytoplasmic membrane	ESAT-6 proteins secretion system in Firmicutes	Putative ESAT-secreted protein, BA2187 homolog	0	0	0	0	0	0	0	0	0.000219	0	0	0
Stress Response	Oxidative stress	Glutathione: Biosynthesis and gamma-glutamyl cycle	Putative exported protein clustered with Gamma-glutamyltranspeptidase	0	0	0	0	0	0	0	0	0	0	0	7.88775E-05
Respiration	NULL	Formate hydrogenase	Putative formate dehydrogenase, cytochrome B subunit (EC 1.2.1.2)	0	0	0	0	0	0	0	0	0	0	0	0.00220857
Cell Wall and Capsule	Gram-Positive cell wall components	Teichoic and lipoteichoic acids biosynthesis	Putative glycosyl/glycerophosphate transferase in teichoic acid biosynthesis	0	0	0	0	0	0	0	0	0	0	0	7.88775E-05
Miscellaneous	Plant-Prokaryote DOE project	PROSC	Putative GntR-family regulatory protein and aminotransferase near polyamine transporter	0	0	0	0	0	0	0	0	0	0	0.000116	0
Membrane Transport	Protein secretion system, Type V	Two partner secretion pathway (TPS)	Putative haemolysin/cytolysin secreted via TPS pathway	0	0	0	0	0	0	0	0	0	0	0	0.000157755

Carbohydrates	NULL	Unknown carbohydrate utilization (cluster Ydj)	Putative HTH-type transcriptional regulator YdjF	0	0	0	0	0	0	0	0	0	0	0	0.000118316
Regulation and Cell signaling	NULL	cAMP signaling in bacteria	Putative insecticidal toxin complex	0	0	0	0	0	0	0	0	0	0	0	0.000276071
Protein Metabolism	Protein biosynthesis	tRNA aminoacylation, Lys	Putative membrane protein found fused to lysyl-tRNA synthetase like protein	0	0	0	0	0	0	0.000722	0	0	0	0	0
Amino Acids and Derivatives	NULL	L-2-amino-thiazoline-4-carboxylic acid-Lcysteine conversion	Putative N-carbamyl-L-cysteine amidohydrolase	0	0	0	0	0	0	0	0	0	0	0	3.94388E-05
Miscellaneous	Plant-Prokaryote DOE project	COG3533	Putative oxidoreductase in arabinose utilization cluster	0	0	0	0	0	0	0	0	0	0	0	3.94388E-05
Carbohydrates	One-carbon Metabolism	Formaldehyde assimilation: Ribulose monophosphate pathway	Putative pentose isomerase	0	0	0	0	0	0	0	0	0	0	0	3.94388E-05
Virulence, Disease and Defense	Invasion and intracellular resistance	Listeria surface proteins: LPXTG motif	Putative peptidoglycan bound protein (LPXTG motif) Lmo1666 homolog	0	0	0	0	0	0	0	0	0	0	0	7.88775E-05
Phosphorus Metabolism	NULL	High affinity phosphate transporter and control of PHO regulon	Putative periplasmic phosphate-binding protein PstS (Catenulisporaceae type)	0	0	0	0	0	0	0	0	0	0	0	3.94388E-05
Regulation and Cell signaling	NULL	Sex pheromones in Enterococcus faecalis and other Firmicutes	Putative pheromone precursor lipoprotein	0	0	0	0	0	0	0	0	0	0	0	0.000157755
Cell Wall and Capsule	Gram-Positive cell wall components	Teichoic and lipoteichoic acids biosynthesis	Putative polyribitolphosphotransferase	0	0	0	0	0	0	0	0	0	0	0	3.94388E-05
Regulation and Cell signaling	NULL	Sex pheromones in Enterococcus faecalis and other Firmicutes	Putative prenyltransferase, contains 1,4-dihydroxy-2-naphthoate octaprenyltransferase domain	0	0	0	0	0	0	0	0	0	0	0	0.000197194
Carbohydrates	Monosaccharides	Mannose Metabolism	Putative regulator of the mannose operon, ManO	0	0	0	0	0	0.000487	0	0	0	0	0	0
Iron acquisition and metabolism	Siderophores	Siderophore Achromobactin	Putative siderophore biosynthesis protein, related to 2-demethylmenaquinone methyltransferase	0	0	0	0	0	0	0	0	0.000219	0	0	0
Respiration	Electron donating reactions	Respiratory Complex I	Putative subunit of NAD(P)H:quinone oxidoreductase	0	0	0	0	0	0	0	0	0	0	0.000116	0
Membrane Transport	NULL	Ton and Tol transport systems	putative TolA function	0	0	0	0	0	0	0	0	0	0	0	0.000788775
Membrane Transport	NULL	Ton and Tol transport systems	Putative Ton-B dependent hemine receptor	0	0	0	0	0	0	0	0	0	0	0	7.88775E-05
Fatty Acids, Lipids, and Isoprenoids	Fatty acids	Polyunsaturated Fatty Acids synthesis	putative transcriptional regulator, inferred for PFA pathway	0	0	0	0	0	0	0	0	0	0	0	3.94388E-05
RNA Metabolism	RNA processing and modification	tRNA modification Bacteria	Putative tRNA-m1A22 methylase	0	0	0	0	0	0	0	0	0	0	0	0.000157755
Regulation and Cell signaling	NULL	Orphan regulatory proteins	Putative two-component response regulator and GGDEF family protein YeaJ	0	0	0	0	0.000657	0	0	0	0	0	0	0
Regulation and Cell signaling	NULL	Orphan regulatory proteins	Putative two-component system response regulator YedW	0	0	0	0	0	0	0	0	0	0	0	0.000512704
RNA Metabolism	RNA processing and modification	Queuosine-Archaeosine Biosynthesis	Putative uncharacterized protein ST0479	0	0	0	0	0	0	0	0	0	0	0	3.94388E-05
Nucleosides and Nucleotides	Purines	Xanthosine utilization (xap region)	Putative xanthosine permease	0	0	0	0	0	0	0	0	0	0	0	3.94388E-05
Respiration	NULL	Quinone oxidoreductase family	Putative Zn-dependent oxidoreductase BA2113	0	0.002262	0	0	0	0	0	0	0	0	0	0
Clustering-based subsystems	Putrescine/GABA utilization cluster-temporal,to add to SSs	GABA and putrescine metabolism from cluters	Putrescine importer	0	0	0	0	0	0	0	0	0	0	0	3.94388E-05
Secondary Metabolism	Bacterial cytostatics, differentiation factors and antibiotics	Paerucumarin Biosynthesis	PvcB protein, related to amino acid oxidizing enzymes	0	0	0	0	0	0	0.000722	0	0	0	0	0
Secondary Metabolism	Bacterial cytostatics, differentiation factors and antibiotics	Paerucumarin Biosynthesis	PvcC protein, related to two-component flavin adenine dinucleotide-dependent monooxygenases	0	0	0	0	0	0	0	0	0	0	0	0.00031551
Iron acquisition and metabolism	NULL	Heme, hemin uptake and utilization systems in GramNegatives	Pyridoxamine 5'-phosphate oxidase-related putative heme iron utilization protein	0	0	0	0	0	0	0	0	0	0	0	0.00094653
Cofactors, Vitamins, Prosthetic Groups, Pigments	Riboflavin, FMN, FAD	Riboflavin, FMN and FAD metabolism	Pyrimidine deaminase eukaryotic (EC 3.5.4.26)	0	0	0	0	0	0	0	0	0.000657	0	0	0
Nucleosides and Nucleotides	Detoxification	Nudix proteins (nucleoside triphosphate hydrolases)	Pyrimidine deoxynucleoside triphosphate (dYTP) pyrophosphohydrolase YfoO	0	0	0	0	0.000657	0	0	0	0	0	0	0
Carbohydrates	Central carbohydrate metabolism	HPr kinase and hprK operon in Gram-positive organisms	Pyrophosphatase PpaX (EC 3.6.1.1)	0	0	0	0	0	0	0	0	0	0	0	0.000118316
Carbohydrates	Central carbohydrate metabolism	Pyruvate metabolism II: acetyl-CoA, acetogenesis from pyruvate	Pyruvate oxidase (EC 1.2.3.3)	0	0	0	0	0	0	0	0	0	0	0	3.94388E-05
Carbohydrates	Central carbohydrate metabolism	Pyruvate:ferredoxin oxidoreductase	Pyruvate:ferredoxin oxidoreductase, porF subunit (EC 1.2.7.1)	0	0	0	0	0	0	0	0	0	0	0	3.94388E-05
Respiration	Electron accepting reactions	Terminal cytochrome C oxidases	Quinol oxidase (SoxABC), cytochrome aa3 subunit (SoxB)	0	0	0	0	0	0	0	0	0	0	0	3.94388E-05
Respiration	Electron accepting reactions	Terminal cytochrome C oxidases	quinol oxidase polypeptide I QoxB (EC:1.9.3.-)	0	0	0	0	0	0	0	0	0	0	0	0.000118316
Respiration	Electron donating reactions	Hydrogenases	Quinone-reactive Ni/Fe hydrogenase, cytochrome b subunit	0	0	0	0	0	0	0	0	0	0	0	0.002287448
Cell Wall and Capsule	Capsular and extracellular polysacchrides	Vibrio Polysaccharide (VPS) Biosynthesis	Quorum-sensing regulator of virulence HapR	0	0	0	0	0	0	0	0	0	0	0	0.000197194
Cell Wall and Capsule	Capsular and extracellular polysacchrides	Vibrio Polysaccharide (VPS) Biosynthesis	RbmD, similar to Lipid A core - O-antigen ligase and related enzymes	0	0	0	0	0	0	0	0	0	0	0	7.88775E-05
Membrane Transport	Protein translocation across cytoplasmic membrane	ESAT-6 proteins secretion system in Actinobacteria	RD1 region associated protein Rv3876	0	0	0	0	0	0	0	0	0.000438	0	0	0
Cell Division and Cell Cycle	NULL	Control of cell elongation - division cycle in Bacilli	Recombination protein RecU	0	0	0	0	0	0	0	0	0	0	0	0.000157755
Stress Response	Oxidative stress	NADPH:quinone oxidoreductase 2	Redox-sensing transcriptional regulator QorR, putative	0	0	0	0	0	0	0	0	0	0	0	3.94388E-05
Cell Wall and Capsule	Gram-Positive cell wall components	Teichoic and lipoteichoic acids biosynthesis	Regulation of D-alanyl-lipoteichoic acid biosynthesis, sensor histidine kinase	0	0	0	0	0	0	0	0	0	0	0	0.000118316
Respiration	Electron accepting reactions	Tetrathionate respiration	Respiratory arsenate reductase subunit A	0	0	0	0	0	0	0	0	0	0	0	7.88775E-05
Membrane Transport	NULL	Citrate Utilization System (CitAB, CitH, and tctABC)	Response regulator CitB of citrate metabolism	0	0	0	0	0	0	0	0	0	0	0	3.94388E-05
DNA Metabolism	DNA uptake, competence	Competence in Streptococci	Response regulator of the competence regulon ComE	0	0	0	0	0	0	0	0	0	0	0	3.94388E-05
Regulation and Cell signaling	Regulation of virulence	VieSAB signal transduction system of Vibrio	Response regulator VieA	0	0	0	0	0	0	0	0	0	0	0	0.00031551
Regulation and Cell signaling	Regulation of virulence	VieSAB signal transduction system of Vibrio	Response regulator VieB	0	0	0	0	0	0	0	0	0	0	0	3.94388E-05
Miscellaneous	Plant-Prokaryote DOE project	Single-Rhodanese-domain proteins	Rhodanese domain protein UPF0176, Actinobacterial subgroup	0	0	0.002555	0	0	0	0	0	0	0	0	0
Miscellaneous	Plant-Prokaryote DOE project	Single-Rhodanese-domain proteins	Rhodanese domain protein UPF0176, Rickettsiales subgroup	0	0.002262	0	0	0	0	0	0	0	0	0	0
Cell Wall and Capsule	Gram-Positive cell wall components	Teichoic and lipoteichoic acids biosynthesis	ribitol-5-phosphate cytidylyltransferase (EC 2.7.7.40)	0	0	0	0	0	0	0	0	0	0	0	7.88775E-05
RNA Metabolism	RNA processing and modification	Ribonuclease H	Ribonuclease HI, Vibrio paralog	0	0	0	0	0	0	0	0	0	0	0	0.000157755
RNA Metabolism	RNA processing and modification	Ribonuclease P archaeal and eukaryal	Ribonuclease P protein component 2 (EC 3.1.26.5)	0	0	0	0	0	0	0	0	0.000438	0	0	0
Protein Metabolism	Protein biosynthesis	Ribosome activity modulation	Ribosome hibernation protein YfiA	0	0	0	0	0	0	0	0	0	0	0	3.94388E-05
Stress Response	NULL	Commensurate regulon activation	Right origin-binding protein	0	0	0	0	0	0	0	0	0	0	0	0.000197194
RNA Metabolism	Transcription	RNA polymerase III initiation factors	RNA polymerase III transcription initiation factor B''	0	0	0	0	0	0	0	0	0	0	0	3.94388E-05

Dormancy and Sporulation	NULL	Sporulation gene orphans	RNA polymerase sporulation specific sigma factor SigK	0	0	0	0	0	0	0	0	0.000219	0	0	0
Clustering-based subsystems	NULL	Nucleoside Catabolism cluster	RNA splicing protein	0	0	0	0	0	0	0	0	0	0	0	0.000157755
RNA Metabolism	Transcription	Rrf2 family transcriptional regulators	Rrf2 family transcriptional regulator, group III	0	0	0	0	0	0	0	0	0	0	0.000116	0
Protein Metabolism	Selenoproteins	Glycine reductase, sarcosine reductase and betaine reductase	Sarcosine reductase component B alpha subunit (EC 1.21.4.3)	0	0	0	0	0	0	0	0	0	0	0	7.88775E-05
Protein Metabolism	Selenoproteins	Selenocysteine metabolism	SECIS-binding protein 2	0	0	0	0	0	0	0	0	0.000219	0	0	0
Regulation and Cell signaling	NULL	Orphan regulatory proteins	Sensory histidine kinase CreB	0	0	0	0	0	0.000487	0	0	0	0	0	0
Virulence, Disease and Defense	NULL	Streptococcus pyogenes Virulome	Serine endopeptidase ScpC (EC 3.4.21.-)	0	0	0	0	0	0	0	0	0.000438	0	0	0
Amino Acids and Derivatives	Alanine, serine, and glycine	Serine Biosynthesis	Serine hydroxymethyltransferase 1 (EC 2.1.2.1)	0	0	0	0	0	0	0.000722	0	0	0	0	0
Membrane Transport	Protein secretion system, Type VI	Type VI secretion systems	Serine/threonine-protein kinase ImpN involved in nitrogen fixation	0	0	0	0	0	0	0	0	0.000219	0	0	0
Membrane Transport	Protein secretion system, Type V	Autotransporter proteins	Serine-protease NalP involved in processing of other autotransporters	0	0	0	0	0	0	0	0	0.000219	0	0	0
Virulence, Disease and Defense	Detection	MLST	Shikimate 5-dehydrogenase (EC 1.1.1.25)	0	0	0	0	0.000657	0	0	0	0	0	0	0
Iron acquisition and metabolism	Siderophores	Siderophore [Alcaligin-like]	Siderophore [Alcaligin-like] biosynthesis complex, long chain	0	0	0	0	0	0	0	0	0	0	0	7.88775E-05
Iron acquisition and metabolism	Siderophores	Siderophore [Alcaligin-like]	Siderophore [Alcaligin-like] biosynthesis complex, short chain	0	0	0	0	0	0	0.000722	0	0	0	0	0
Iron acquisition and metabolism	Siderophores	Siderophore [Alcaligin-like]	Siderophore [Alcaligin-like] biosynthetic enzyme (EC 1.14.13.59)	0	0	0	0	0	0	0	0.000498	0	0	0	0
Iron acquisition and metabolism	Siderophores	Siderophore Achromobactin	Siderophore achromobactin ABC transporter, ATPase component	0	0	0	0	0	0	0	0	0	0	0	3.94388E-05
Iron acquisition and metabolism	Siderophores	Siderophore Achromobactin	Siderophore achromobactin ABC transporter, permease protein	0	0	0	0	0	0	0	0	0	0	0.000349	0
Iron acquisition and metabolism	Siderophores	Siderophore Staphylobactin	Siderophore staphylobactin ABC transporter, permease protein SirC, putative	0	0	0	0	0	0	0	0	0	0	0	3.94388E-05
Iron acquisition and metabolism	Siderophores	Siderophore Staphylobactin	Siderophore staphylobactin ABC transporter, substrate-binding protein SirA	0	0	0	0	0	0	0	0	0.000438	0	0	0
Iron acquisition and metabolism	Siderophores	Siderophore assembly kit	Siderophore synthetase large component, acetyltransferase	0	0	0	0	0	0	0	0	0.000876	0	0	0
Iron acquisition and metabolism	Siderophores	Siderophore assembly kit	Siderophore synthetase small component, acetyltransferase	0	0	0	0	0	0	0	0.000498	0	0	0	0
Protein Metabolism	Protein processing and modification	Signal peptidase	Signal peptidase, type IV - prepilin/preflagellin	0	0	0	0	0	0	0	0	0.001095	0	0	0
Respiration	NULL	Biogenesis of c-type cytochromes	Similar to cytochrome c-type biogenesis protein CcsA/ResC	0	0	0	0	0	0	0	0	0	0	0	3.94388E-05
Phages, Prophages, Transposable elements, Plasmids	Phages, Prophages	Listeria phi-A118-like prophages	Similar to terminase small subunit, yqaS homolog	0	0	0	0	0	0	0	0	0	0	0	3.94388E-05
Miscellaneous	Plant-Prokaryote DOE project	DOE COG2016	Similarity with yeast transcription factor IIIC Tau subunit that binds B-block elements of class III promoters	0	0	0	0	0	0	0	0	0	0	0	7.88775E-05
Dormancy and Sporulation	Spore DNA protection	Small acid-soluble spore proteins	Small acid-soluble spore protein, alpha/beta family, SASP 2	0	0	0	0	0	0	0	0	0.000219	0	0	0
Dormancy and Sporulation	Spore DNA protection	Small acid-soluble spore proteins	Small acid-soluble spore protein, alpha/beta family, SASP 6	0	0	0	0	0	0	0	0	0.000438	0	0	0
Miscellaneous	Plant-Prokaryote DOE project	DOE COG2016	Small nuclear ribonucleoprotein F	0	0	0	0	0	0	0.000722	0	0	0	0	0
Carbohydrates	Monosaccharides	D-Sorbitol(D-Glucitol) and L-Sorbose Utilization	Sorbitol operon transcription regulator	0	0	0	0	0	0	0	0	0	0	0	0.000236633
Iron acquisition and metabolism	NULL	Heme, hemin uptake and utilization systems in GramPositives	Sortase A, LPXTG specific	0	0	0	0	0	0	0	0	0	0	0	3.94388E-05
Dormancy and Sporulation	NULL	Sporulation draft	spore peptidoglycan hydrolase (N-acetylglucosaminidase) (EC 3.2.1.-)	0	0	0	0	0	0	0	0	0	0	0	3.94388E-05
Dormancy and Sporulation	NULL	Sporulation gene orphans	Sporulation kinase C (EC 2.7.13.3)	0	0	0	0	0	0	0	0	0	0	0	7.88775E-05
Dormancy and Sporulation	NULL	Sporulation gene orphans	Sporulation sigma-E factor processing peptidase (SpoIIGA)	0	0	0	0	0	0	0	0	0.000657	0	0	0
Dormancy and Sporulation	NULL	SpoVS protein family	SpoVS-related protein, type 4	0	0	0	0	0	0	0	0	0	0	0	0.000276071
Dormancy and Sporulation	NULL	SpoVS protein family	SpoVS-related protein, type 6	0	0	0.00511	0	0	0	0	0	0	0	0	0
Protein Metabolism	Protein biosynthesis	Ribosome SSU chloroplast	SSU ribosomal protein S12p (S23e), chloroplast	0	0	0	0	0	0	0	0	0.000219	0	0	0
Protein Metabolism	Protein biosynthesis	Ribosome SSU mitochondrial	SSU ribosomal protein S13p (S18e), mitochondrial	0	0	0	0	0	0	0	0	0	0	0	3.94388E-05
Protein Metabolism	Protein biosynthesis	Ribosome SSU chloroplast	SSU ribosomal protein S16p, chloroplast	0	0	0	0	0	0	0	0	0	0	0	0.000118316
Protein Metabolism	Protein biosynthesis	Ribosome SSU mitochondrial	SSU ribosomal protein S16p, mitochondrial	0	0	0	0	0	0	0	0	0	0	0	7.88775E-05
Protein Metabolism	Protein biosynthesis	Ribosome SSU chloroplast	SSU ribosomal protein S18p, chloroplast	0	0	0	0	0	0	0	0	0.000219	0	0	0
Protein Metabolism	Protein biosynthesis	Ribosome SSU chloroplast	SSU ribosomal protein S4p (S9e), chloroplast	0	0	0	0	0	0	0	0	0	0	0	3.94388E-05
Protein Metabolism	Protein biosynthesis	Ribosome SSU chloroplast	SSU ribosomal protein S5p (S2e), chloroplast	0	0	0	0	0	0	0	0	0.000438	0	0	0
Protein Metabolism	Protein biosynthesis	Ribosome SSU chloroplast	SSU ribosomal protein S6p, chloroplast	0	0	0	0	0	0	0	0	0.000438	0	0	0
Dormancy and Sporulation	NULL	Sporulation gene orphans	Stage II sporulation protein M (SpoIIM)	0	0	0	0	0	0	0	0	0.000219	0	0	0
Dormancy and Sporulation	NULL	Sporulation gene orphans	Stage II sporulation protein P	0	0	0	0	0	0	0	0	0.000438	0	0	0
Dormancy and Sporulation	NULL	Sporulation Cluster III A	Stage III sporulation protein AC	0	0	0	0	0	0	0	0.000997	0	0	0	0
Dormancy and Sporulation	NULL	Sporulation gene orphans	Stage IV sporulation pro-sigma-K processing enzyme (SpoIVFB)	0	0	0	0	0	0	0	0	0	0	0	7.88775E-05
Dormancy and Sporulation	NULL	Sporulation gene orphans	Stage IV sporulation protein B	0	0	0	0	0	0	0	0	0	0	0	3.94388E-05
Dormancy and Sporulation	NULL	Sporulation gene orphans	Stage IV sporulation protein FA (SpoIVFA)	0	0	0	0	0	0	0	0	0.000219	0	0	0
Virulence, Disease and Defense	Resistance to antibiotics and toxic compounds	Aminoglycoside adenylyltransferases	Streptomycin 3''-O-adenylyltransferase (EC 2.7.7.47)	0	0	0	0	0	0	0	0.000498	0	0	0	0
Virulence, Disease and Defense	Resistance to antibiotics and toxic compounds	Streptothricin resistance	Streptothricin acetyltransferase, Streptomyces lavendulae type	0	0	0	0	0	0	0	0	0	0	0	7.88775E-05
Cofactors, Vitamins, Prosthetic Groups, Pigments	Tetrapyrroles	Coenzyme B12 biosynthesis	Substrate-specific component CblT of predicted B12-regulated ECF transporter for dimethylbenzimidazole	0	0	0	0	0	0	0	0	0	0	0	7.88775E-05
Membrane Transport	NULL	ECF class transporters	Substrate-specific component Cce 1529 of predicted ECF transporter	0	0	0	0	0	0.001461	0	0	0	0	0	0
Membrane Transport	NULL	ECF class transporters	Substrate-specific component QueT (COG4708) of predicted queuosine-regulated ECF transporter	0	0	0	0	0	0	0	0	0	0	0	3.94388E-05
Membrane Transport	NULL	ECF class transporters	Substrate-specific component TTE1586 of predicted methylthioadenosine ECF transporter	0	0	0	0	0	0	0	0	0	0	0	7.88775E-05
Cofactors, Vitamins, Prosthetic Groups, Pigments	NULL	Thiamin biosynthesis	Substrate-specific component YkoE of thiamin-regulated ECF transporter for	0	0	0	0	0	0	0	0	0	0	0.000116	0

			HydroxyMethylPyrimidine												
Dormancy and Sporulation	NULL	Bacillus biofilm matrix protein component TasA and homologs	Subtilase family domain protein	0	0	0	0	0	0	0	0	0	0	0	0.000118316
Stress Response	Oxidative stress	Oxidative stress	superoxide dismutase [Fe-Zn] (EC 1.15.1.1)	0	0	0	0	0	0	0	0	0.000438	0	0	0
Respiration	Electron accepting reactions	Fe(III) respiration - Shewanella type	surface localized decaheme cytochrome c lipoprotein, MtrC	0	0	0	0	0	0	0	0	0	0	0	7.88775E-05
Respiration	Electron accepting reactions	Fe(III) respiration - Shewanella type	surface localized decaheme cytochrome c lipoprotein, MtrF	0	0	0	0	0	0.000487	0	0	0	0	0	0
Respiration	Electron accepting reactions	Fe(III) respiration - Shewanella type	surface localized decaheme cytochrome c lipoprotein, MtrG	0	0	0	0	0	0	0	0	0	0	0	3.94388E-05
Respiration	Electron accepting reactions	Fe(III) respiration - Shewanella type	surface localized undecaheme cytochrome c lipoprotein, UndB	0	0	0	0	0	0	0	0	0	0	0	3.94388E-05
Membrane Transport	Protein secretion system, Type III	Type III secretion systems	Surface presentation of antigens protein SpaP	0	0	0	0	0	0	0	0	0	0	0	7.88775E-05
Phages, Prophages, Transposable elements, Plasmids	Phages, Prophages	T7-like phage core proteins	T7-like phage DNA-directed RNA polymerase (EC 2.7.7.6)	0	0	0	0	0	0	0	0.000997	0	0	0	0
Metabolism of Aromatic Compounds	Peripheral pathways for catabolism of aromatic compounds	Phenylpropanoid compound degradation	Tannase and feruloyl esterase precursor	0	0	0	0	0	0	0	0	0	0	0	7.88775E-05
Sulfur Metabolism	Organic sulfur assimilation	Taurine Utilization	Taurine transporter substrate-binding protein	0	0	0	0	0	0	0	0	0.000657	0	0	0
Cell Wall and Capsule	Gram-Positive cell wall components	Teichuronic acid biosynthesis	Teichuronic acid biosynthesis protein TuaB	0.001044	0	0	0	0	0	0	0	0	0	0	0
Cell Wall and Capsule	Gram-Positive cell wall components	Teichuronic acid biosynthesis	Teichuronic acid biosynthesis protein TuaE, putative secreted polysaccharide polymerase	0	0	0	0	0	0	0	0	0	0	0	7.88775E-05
Virulence, Disease and Defense	Resistance to antibiotics and toxic compounds	Teicoplanin-resistance in Staphylococcus	Teicoplanin resistance associated membrane protein TcaA	0	0	0	0	0	0	0	0	0	0	0	7.88775E-05
Stress Response	Detoxification	Tellurite resistance: Chromosomal determinants	Tellurite resistance protein TehB	0	0	0	0	0	0	0	0	0	0	0	0.000394388
Phages, Prophages, Transposable elements, Plasmids	Phages, Prophages	Listeria phi-A118-like prophages	Terminase large subunit [Bacteriophage A118]	0	0	0	0	0	0	0	0.000498	0	0	0	0
Clustering-based subsystems	NULL	CBSS-196620.1.peg.2477	TetR family regulatory protein	0	0	0	0	0	0	0	0	0	0	0	0.000197194
Protein Metabolism	Protein biosynthesis	Translation elongation factor G family	Tetracycline resistance protein TetQ	0	0	0	0	0	0	0	0	0	0	0	3.94388E-05
Membrane Transport	Protein translocation across cytoplasmic membrane	ESAT-6 proteins secretion system in Firmicutes	Tetratrico-peptide repeat (TPR) protein within ESAT-6 gene cluster	0	0	0	0	0	0	0	0	0	0	0	3.94388E-05
RNA Metabolism	RNA processing and modification	tRNA modification Archaea	Thiamine biosynthesis protein thiI-like	0	0	0	0	0	0	0	0	0	0	0	0.000157755
Phages, Prophages, Transposable elements, Plasmids	Pathogenicity islands	Listeria Pathogenicity Island LIPI-1 extended	Thiol-activated cytolysin	0	0	0	0	0	0	0	0	0.000438	0	0	0
Clustering-based subsystems	NULL	PA0057 cluster	Thioredoxin-like protein clustered with PA0057	0	0	0	0	0	0	0	0	0	0	0	0.000197194
Respiration	Electron accepting reactions	Anaerobic respiratory reductases	Thiosulfate reductase electron transport protein phsB	0	0	0	0	0	0	0	0	0	0	0	0.000276071
Amino Acids and Derivatives	Lysine, threonine, methionine, and cysteine	Threonine anaerobic catabolism gene cluster	Threonine catabolic operon transcriptional activator TdcA	0	0	0	0	0	0.000487	0	0	0	0	0	0
RNA Metabolism	RNA processing and modification	tRNA modification Archaea	TilS-like type 2	0	0	0	0	0	0	0	0	0	0	0	3.94388E-05
Regulation and Cell signaling	Proteolytic pathway	Coagulation cascade	Tissue factor	0	0	0	0	0	0	0	0	0	0	0	3.94388E-05
Phages, Prophages, Transposable elements, Plasmids	Transposable elements	CBSS-203122.12.peg.188	TniQ	0	0	0	0	0	0	0	0	0	0	0	3.94388E-05
Metabolism of Aromatic Compounds	Peripheral pathways for catabolism of aromatic compounds	Toluene degradation	Toluate 1,2-dioxygenase alpha subunit	0	0	0	0	0	0	0	0	0.000438	0	0	0
Metabolism of Aromatic Compounds	NULL	Toluene 4-monooxygenase (T4MO)	Toluene-4-monooxygenase, subunit TmoA	0	0	0	0	0	0	0	0	0	0	0	0.000276071
Carbohydrates	Aminosugars	N-Acetyl-Galactosamine and Galactosamine Utilization	TonB-dependent receptor possibly related to N-acetylgalactosamine utilization	0	0	0	0	0	0	0	0	0	0	0	0.000118316
Phages, Prophages, Transposable elements, Plasmids	Pathogenicity islands	Vibrio pathogenicity island	Toxin co-regulated pilus biosynthesis protein I, chemoreceptor, negative regulator of TcpA	0	0	0	0	0	0	0	0	0	0	0	0.000118316
Regulation and Cell signaling	Programmed Cell Death and Toxin-antitoxin Systems	A toxin-antitoxin module cotranscribed with DinB	Toxin YafO	0	0	0	0	0	0	0	0	0	0	0	7.88775E-05
RNA Metabolism	Transcription	RNA polymerase II initiation factors	Transcription initiation factor IID 90 kDa subunit	0	0	0	0	0	0	0	0	0	0	0	0.000157755
RNA Metabolism	Transcription	RNA polymerase II initiation factors	Transcription initiation factor IIF alpha subunit	0	0	0	0	0	0	0	0	0	0	0	0.000788775
RNA Metabolism	Transcription	RNA polymerase II initiation factors	Transcription initiation factor IIH cyclin-dependent kinase 7	0	0	0	0	0	0	0	0	0	0	0	7.88775E-05
RNA Metabolism	Transcription	RNA polymerase II initiation factors	Transcription initiation factor IIH p44 subunit	0	0	0	0	0	0	0	0	0	0	0	3.94388E-05
RNA Metabolism	Transcription	RNA polymerase III initiation factors	Transcription initiation factor IIIB 70 kDa subunit	0	0	0	0	0	0	0	0	0	0	0	3.94388E-05
Clustering-based subsystems	NULL	CBSS-314269.3.peg.1840	Transcription regulator in CO-DH cluster	0.000348	0	0	0	0	0	0	0	0	0	0	0
Virulence, Disease and Defense	Resistance to antibiotics and toxic compounds	Copper homeostasis	Transcriptional activator protein CopR	0	0	0	0	0	0	0	0	0	0	0	0.000118316
Regulation and Cell signaling	Quorum sensing and biofilm formation	Acyl Homoserine Lactone (AHL) Autoinducer Quorum Sensing	Transcriptional activator protein solR	0	0	0	0	0	0.000487	0	0	0	0	0	0
Virulence, Disease and Defense	Toxins and superantigens	Cholera toxin	Transcriptional activator ToxR	0	0	0	0	0	0	0.000722	0	0	0	0	0
Cell Wall and Capsule	Capsular and extracellular polysacchrides	Vibrio Polysaccharide (VPS) Biosynthesis	Transcriptional regulator CdgA	0	0	0	0	0	0	0	0	0	0	0	7.88775E-05
Regulation and Cell signaling	NULL	DNA-binding regulatory proteins, strays	Transcriptional regulator GbuR	0	0	0	0	0	0	0	0	0	0	0	0.000552143
Clustering-based subsystems	NULL	Listeria bile tolerance locus BltB	Transcriptional regulator in BltB locus	0	0	0	0	0	0	0	0	0	0	0	3.94388E-05
Clustering-based subsystems	NULL	PFGI-1-like cluster 1	Transcriptional regulator in PFGI-1-like cluster	0	0	0	0	0	0	0	0	0	0	0	0.000118316
Regulation and Cell signaling	NULL	Staphylococcal accessory gene regulator system	Transcriptional regulator MgrA (Regulator of autolytic activity)	0	0	0	0	0	0	0	0	0	0	0	7.88775E-05
Regulation and Cell signaling	Quorum sensing and biofilm formation	Biofilm formation in Staphylococcus	Transcriptional regulator of biofilm formation (AraC/XylS family)	0	0	0	0	0	0	0	0	0	0	0	3.94388E-05
Fatty Acids, Lipids, and Isoprenoids	Fatty acids	Fatty Acid Biosynthesis FASII	Transcriptional regulator of fatty acid biosynthesis FabT	0	0	0	0	0	0	0	0	0	0	0	7.88775E-05
Carbohydrates	Monosaccharides	Mannose Metabolism	Transcriptional regulator of mannoside utilization, LacI family	0	0	0	0	0	0	0	0	0	0	0	7.88775E-05
Regulation and Cell signaling	Quorum sensing and biofilm formation	Quorum sensing regulation in Pseudomonas	Transcriptional regulator RhlR	0	0	0	0	0	0	0	0	0	0	0	7.88775E-05
DNA Metabolism	DNA uptake, competence	Competence in Streptococci	Transcriptional regulator SpxA1	0	0	0	0	0	0	0	0	0	0	0	3.94388E-05
Cell Wall and Capsule	Capsular and extracellular polysacchrides	Vibrio Polysaccharide (VPS) Biosynthesis	Transcriptional regulator VpsT	0	0	0	0	0	0	0	0	0	0	0	0.000552143
Carbohydrates	Di- and oligosaccharides	Maltose and Maltodextrin Utilization	Transcriptional regulator YcjW, LacI family, possibly involved in maltodextrin utilization pathway	0	0	0	0	0	0	0	0	0.000219	0	0	0
Amino Acids and Derivatives	Arginine; urea cycle, polyamines	Polyamine Metabolism	Transcriptional regulator, luxR family, associated with agmatine catabolism	0	0	0	0	0	0	0	0	0	0	0	3.94388E-05
Iron acquisition and metabolism	NULL	Iron acquisition in Vibrio	Transcriptional regulator, VCA0231 ortholog	0	0	0	0	0	0	0	0	0	0	0	0.000394388
Virulence, Disease and Defense	Resistance to antibiotics and toxic compounds	Multidrug efflux pump in Campylobacter jejuni (CmeABC operon)	Transcriptional repressor of CmeABC operon, CmeR	0	0	0	0	0	0	0	0	0	0	0	7.88775E-05

Carbohydrates	Sugar alcohols	Inositol catabolism	Transcriptional repressor of the myo-inositol catabolic operon DeoR family	0	0	0	0	0	0	0	0.000498	0	0	0	0
Amino Acids and Derivatives	Aromatic amino acids and derivatives	Common Pathway For Synthesis of Aromatic Compounds (DAHP synthase to chorismate)	Transcriptional repressor protein TrpR	0	0	0	0	0	0	0	0	0	0	0	3.94388E-05
Protein Metabolism	Protein biosynthesis	Translation elongation factors eukaryotic and archaeal	Translation elongation factor 1 gamma subunit	0	0	0	0	0	0	0	0	0	0	0	3.94388E-05
Protein Metabolism	Protein biosynthesis	Translation elongation factors eukaryotic and archaeal	Translation elongation factor 3	0	0	0	0	0	0	0	0	0	0	0	3.94388E-05
Protein Metabolism	Protein biosynthesis	Translation initiation factors eukaryotic and archaeal	Translation initiation factor SUI1	0	0	0	0	0	0	0	0	0	0	0	0.000670459
Membrane Transport	NULL	ECF class transporters	Transmembrane component of energizing module of ECF transporters in Mycobacteria	0	0	0	0	0	0	0	0	0	0	0	3.94388E-05
Membrane Transport	NULL	ECF class transporters	Transmembrane component of energizing module of predicted tryptophan ECF transporter	0	0	0	0	0	0	0	0	0	0	0	3.94388E-05
Membrane Transport	NULL	ECF class transporters	Transmembrane component of energizing module of riboflavin ECF transporter	0	0.002262	0	0	0	0	0	0	0	0	0	0
Carbohydrates	Di- and oligosaccharides	Trehalose Uptake and Utilization	Trehalose operon transcriptional repressor	0	0	0	0	0	0	0	0	0	0	0	0.000236633
Fatty Acids, Lipids, and Isoprenoids	Triacylglycerols	Triacylglycerol metabolism	Triacylglycerol lipase (EC 3.1.1.3)	0	0	0	0	0	0	0	0	0	0	0.000233	0
Carbohydrates	Organic acids	Malonate decarboxylase	Triphosphoribosyl-dephospho-CoA synthetase (EC 2.7.8.25)	0	0	0	0	0	0	0	0	0	0	0	0.000670459
RNA Metabolism	RNA processing and modification	tRNA modification yeast cytoplasmic	tRNA (cytosine-5-)-methyltransferase NCL1 (EC 2.1.1.29) (tRNA methyltransferase 4) (Multisite-specific tRNA:m5C-methyltransferase)	0	0	0	0	0	0	0	0	0	0	0	3.94388E-05
RNA Metabolism	RNA processing and modification	Wyeosine-MimG Biosynthesis	tRNA methylase YGL050w homolog Wyeosine biosynthesis	0	0	0	0	0	0	0	0	0	0	0	0.000118316
Protein Metabolism	Protein biosynthesis	tRNA aminoacylation, Met	tRNA-binding protein YgjH	0	0	0	0	0.000657	0	0	0	0	0	0	0
Cell Wall and Capsule	NULL	tRNA-dependent amino acid transfers	tRNA-dependent lipid II-Ala--L-alanine ligase	0	0	0	0	0	0	0	0	0	0	0.000116	0
RNA Metabolism	RNA processing and modification	tRNA modification yeast cytoplasmic	tRNA-dihydrouridine synthase 2 (EC 1.-.-.-) modifies uridine residues at position 20 of cytoplasmic tRNAs	0	0	0	0	0	0	0	0	0	0	0	3.94388E-05
Protein Metabolism	Protein biosynthesis	tRNAs	tRNA-Met	0	0	0	0	0	0	0	0	0	0	0	3.94388E-05
Stress Response	Detoxification	Uptake of selenate and selenite	TsgA protein homolog	0	0	0	0	0	0	0.000722	0	0	0	0	0
Regulation and Cell signaling	Programmed Cell Death and Toxin-antitoxin Systems	Toxin-antitoxin replicon stabilization systems	TTE0859 replicon stabilization toxin	0	0	0	0	0	0	0	0.000498	0	0	0	0
Membrane Transport	Protein translocation across cytoplasmic membrane	Twin-arginine translocation system	Twin-arginine translocation protein TatE	0	0	0	0	0	0	0	0	0	0	0	0.000118316
Virulence, Disease and Defense	Bacteriocins, ribosomally synthesized antibacterial peptides	Bacitracin Stress Response	Two-component response regulator BceR	0	0	0	0	0	0	0	0	0	0	0	7.88775E-05
Phages, Prophages, Transposable elements, Plasmids	Plasmid related functions	Plasmid-encoded T-DNA transfer	Two-component response regulator of vir regulon, VirG	0	0	0	0	0	0	0	0	0.000438	0	0	0
Virulence, Disease and Defense	Resistance to antibiotics and toxic compounds	Streptococcus pneumoniae Vancomycin Tolerance Locus	Two-component response regulator VncR	0	0	0	0	0	0	0	0	0	0	0	7.88775E-05
Iron acquisition and metabolism	NULL	Iron acquisition in Streptococcus	Two-component response regulator, associated with ferric iron transporter, SPy1062 homolog	0	0	0	0	0	0	0	0	0	0	0	3.94388E-05
Virulence, Disease and Defense	Bacteriocins, ribosomally synthesized antibacterial peptides	Bacitracin Stress Response	Two-component sensor histidine kinase BceS	0	0	0	0	0	0	0	0	0	0	0	3.94388E-05
Virulence, Disease and Defense	Bacteriocins, ribosomally synthesized antibacterial peptides	Bacitracin Stress Response	Two-component sensor kinase YvcQ	0	0	0	0	0	0	0	0	0	0	0	3.94388E-05
Membrane Transport	Protein secretion system, Type VII (Chaperone/Usher pathway, CU)	Type 1 pili (mannose-sensitive fimbriae, gamma-fimbriae)	type 1 fimbriae regulatory protein FimB	0	0	0	0	0.000657	0	0	0	0	0	0	0
Membrane Transport	Protein secretion system, Type VII (Chaperone/Usher pathway, CU)	Type 1 pili (mannose-sensitive fimbriae, gamma-fimbriae)	type 1 fimbriae regulatory protein FimE	0	0	0	0	0	0	0	0	0.000219	0	0	0
Membrane Transport	Protein secretion system, Type III	Type III secretion systems	Type III secretion cytoplasmic LcrG inhibitor (LcrV,secretion and targeting control protein, V antigen)	0	0	0	0	0	0	0	0	0	0.006608	0	0
Membrane Transport	Protein secretion system, Type III	Type III secretion systems	Type III secretion cytoplasmic protein (YscL)	0	0	0	0	0	0	0.000722	0	0	0	0	0
Membrane Transport	Protein secretion system, Type III	Type III secretion systems	Type III secretion host injection and negative regulator protein (YopD)	0	0	0	0	0	0	0	0	0.000219	0	0	0
Membrane Transport	Protein secretion system, Type III	Type III secretion system	Type III secretion inner membrane protein (YscQ,homologous to flagellar export components)	0	0.002262	0	0	0	0	0	0	0	0	0	0
Membrane Transport	Protein secretion system, Type III	Type III secretion systems	Type III secretion protein SctJ	0	0	0	0	0	0	0	0	0.000219	0	0	0
RNA Metabolism	RNA processing and modification	eukaryotic rRNA modification and related functions	Tyrosine-protein kinase BTK (EC 2.7.10.2)	0	0	0	0	0	0	0	0	0	0	0	3.94388E-05
Cell Wall and Capsule	Capsular and extracellular polysacchrides	Extracellular Polysaccharide Biosynthesis of Streptococci	Tyrosine-protein phosphatase CpsB (EC 3.1.3.48)	0	0	0	0	0.000657	0	0	0	0	0	0	0
RNA Metabolism	RNA processing and modification	Spliceosome	U4/U6 small nuclear ribonucleoprotein component PRP3	0	0	0	0	0	0	0	0	0	0	0.000116	0
Cell Wall and Capsule	Capsular and extracellular polysacchrides	Pseudaminic Acid Biosynthesis	UDP-6-deoxy-AltdiNAc hydrolase (PseG, third step of pseudaminic acid biosynthesis)	0	0	0	0	0	0	0	0	0	0	0	0.000907091
Cell Wall and Capsule	Gram-Negative cell wall components	LOS core oligosaccharide biosynthesis	UDP-galactose:(galactosyl) LPS alpha1,2-galactosyltransferase WaaW (EC 2.4.1.-)	0	0	0	0	0	0	0	0	0	0	0	0.000118316
Cell Wall and Capsule	NULL	Peptidoglycan biosynthesis--gjo	UDP-N-acetylmuramoyl-tripeptide--D-alanyl-D-alanine ligase (EC 6.3.2.10)	0	0	0	0	0	0	0	0	0	0	0	7.88775E-05
Regulation and Cell signaling	Quorum sensing and biofilm formation	Protein YjgK cluster linked to biofilm formation	Uncharacterized acetyltransferase YjgM (EC 2.3.1.-)	0	0	0	0	0	0	0	0	0	0	0	3.94388E-05
Respiration	NULL	Methanogenesis strays	Uncharacterized ferredoxin MJ0099	0	0	0	0	0	0	0	0	0	0	0	3.94388E-05
Respiration	NULL	Methanogenesis strays	Uncharacterized HTH-type transcriptional regulator MJ0621	0	0	0	0	0	0	0	0	0	0	0	7.88775E-05
Clustering-based subsystems	Clustering-based subsystems	Putative diaminopropionate ammonia-lyase cluster	Uncharacterized lipoprotein YgeR precursor	0	0	0	0	0	0	0	0	0	0	0	3.94388E-05
RNA Metabolism	RNA processing and modification	Wyeosine-MimG Biosynthesis	Uncharacterized protein conserved in archaea (DUF531)	0	0	0	0	0	0	0	0	0	0	0	7.88775E-05
Respiration	NULL	Methanogenesis strays	Uncharacterized protein MA3991	0	0	0	0	0	0	0	0	0	0	0	0.000118316
Respiration	NULL	Methanogenesis strays	Uncharacterized protein MJ0119	0	0	0	0.00498	0	0	0	0	0	0	0	0
Respiration	NULL	Methanogenesis strays	Uncharacterized protein MJ0296	0	0	0	0	0	0	0	0	0.000219	0	0	0
Respiration	NULL	Methanogenesis strays	Uncharacterized protein MJ0404	0	0	0	0	0	0	0	0	0	0	0	0.000276071
Respiration	NULL	Methanogenesis strays	Uncharacterized protein MJ0405	0	0	0	0	0	0	0	0	0	0	0	7.88775E-05
Respiration	NULL	Methanogenesis strays	Uncharacterized protein MJ0575	0	0	0	0	0	0	0.000722	0	0	0	0	0
Respiration	NULL	Methanogenesis strays	Uncharacterized protein MJ0802	0	0	0	0	0	0	0	0	0	0	0	3.94388E-05
Clustering-based subsystems	NULL	Yfa cluster	Uncharacterized protein YfaD	0	0	0	0	0	0.000487	0	0	0	0	0	0
Iron acquisition and metabolism	Siderophores	Siderophore Achromobactin	Uncharacterized siderophore S biosynthesis protein, AcsC-like	0	0	0	0	0	0	0	0	0.000876	0	0	0

Carbohydrates	NULL	Unknown carbohydrate utilization (cluster Ydj)	Uncharacterized sugar kinase YdjH	0	0	0	0	0	0	0	0	0	0	0	3.94388E-05
Nucleosides and Nucleotides	NULL	Pseudouridine catabolism	Uncharacterized sugar kinase YeiI	0	0	0	0	0	0	0	0	0	0	0	3.94388E-05
Fatty Acids, Lipids, and Isoprenoids	Isoprenoids	polyprenyl synthesis	undecaprenyl diphosphate synthase	0	0	0	0	0	0	0	0	0	0	0	7.88775E-05
Stress Response	NULL	Universal stress protein family	Universal stress protein A	0	0	0	0	0	0	0	0	0	0	0	3.94388E-05
Stress Response	NULL	Universal stress protein family	Universal stress protein family 5	0	0	0	0	0	0	0	0.000498	0	0	0	0
Stress Response	NULL	Universal stress protein family	Universal stress protein family 6	0	0	0	0	0	0	0	0	0	0	0	7.88775E-05
Stress Response	NULL	Universal stress protein family	Universal stress protein family 7	0	0	0	0	0	0	0	0.000997	0	0	0	0
Cell Wall and Capsule	Gram-Negative cell wall components	Lipid A modifications	UPF0141 membrane protein YijP possibly required for phosphoethanolamine modification of lipopolysaccharide	0	0	0	0	0	0	0	0	0	0	0	0.002090254
Respiration	NULL	Methanogenesis strays	UPF0288 protein MJ1412	0	0	0	0	0	0	0	0	0	0	0	0.000118316
DNA Metabolism	DNA repair	Uracil-DNA glycosylase	Uracil DNA glycosylase superfamily protein	0	0	0	0	0	0	0	0	0.001314	0	0	0
Iron acquisition and metabolism	NULL	Iron acquisition in Vibrio	Vibriobactin utilization protein ViuB	0	0	0	0	0	0	0	0.000498	0	0	0	0
Iron acquisition and metabolism	Siderophores	Vibrioferrin synthesis	Vibrioferrin amide bond forming protein PvsB	0	0	0	0	0	0	0	0	0	0	0	7.88775E-05
Iron acquisition and metabolism	Siderophores	Vibrioferrin synthesis	Vibrioferrin amide bond forming protein PvsD	0	0	0	0	0	0	0	0	0	0	0	0.000118316
Iron acquisition and metabolism	Siderophores	Vibrioferrin synthesis	Vibrioferrin decarboxylase protein PvsE	0	0	0	0	0	0	0	0	0	0	0	7.88775E-05
Iron acquisition and metabolism	Siderophores	Vibrioferrin synthesis	Vibrioferrin ligase/carboxylase protein PvsA	0	0	0	0	0	0	0	0	0	0	0.000116	0
Membrane Transport	Protein and nucleoprotein secretion system, Type IV	Vir-like type 4 secretion system	VirC1 protein promotes T-DNA transfer, ParA/MinD-like	0	0	0	0	0	0	0	0	0	0	0	7.88775E-05
Miscellaneous	NULL	ZZ gjo need homes	Virulence factor MviM	0	0	0	0	0	0.000487	0	0	0	0	0	0
Virulence, Disease and Defense	Adhesion	Adhesins in Staphylococcus	Virulence-associated cell-wall-anchored protein SasH (LPXTG motif)	0	0	0	0	0	0	0	0	0	0	0	3.94388E-05
Respiration	ATP synthases	V-Type ATP synthase	V-type ATP synthase subunit G (EC 3.6.3.14)	0	0	0	0	0	0	0	0	0	0	0	0.000118316
Iron acquisition and metabolism	NULL	Iron acquisition in Vibrio	Vulnibactin utilization protein VuuB	0	0	0	0	0	0	0	0.000997	0	0	0	0
Regulation and Cell signaling	NULL	WhiB and WhiB-type regulatory proteins	WhiB-type transcription regulator	0	0	0	0	0	0	0	0	0	0	0	3.94388E-05
Dormancy and Sporulation	NULL	Spore pigment biosynthetic cluster in Actinomycetes	WhiE I protein of unknown function	0	0	0	0	0	0	0	0	0	0	0	7.88775E-05
Clustering-based subsystems	Carbohydrates	Beta-lactamase cluster in Streptococcus	Xaa-Pro dipeptidyl-peptidase (EC 3.4.14.11)	0	0	0	0	0	0	0	0	0	0	0	0.00031551
Cell Wall and Capsule	Capsular and extracellular polysacchrides	Xanthan Exopolysaccharide Biosynthesis and Export	Xanthan biosynthesis exopolysaccharide polymerase GumE	0	0	0	0	0	0	0	0	0	0	0	7.88775E-05
Cell Wall and Capsule	Capsular and extracellular polysacchrides	Xanthan Exopolysaccharide Biosynthesis and Export	Xanthan biosynthesis glucuronosyltransferase GumK	0	0	0	0	0	0	0	0.000498	0	0	0	0
Cell Wall and Capsule	Capsular and extracellular polysacchrides	Xanthan Exopolysaccharide Biosynthesis and Export	Xanthan biosynthesis oligosaccharidyl-lipid flippase GumJ	0	0	0	0	0	0	0	0	0	0	0	0.000118316
Nucleosides and Nucleotides	Purines	Purine Utilization	Xanthine transporter,putative	0	0	0	0	0	0	0	0	0	0	0	7.88775E-05
Nucleosides and Nucleotides	Purines	Purine Utilization	Xanthine/uracil transporter	0	0	0	0	0	0	0	0	0.000219	0	0	0
Miscellaneous	Plant-Prokaryote DOE project	DOE COG3533	Xylosidase/arabinosidase ass w/ COG3533	0	0	0	0	0	0	0	0	0	0	0	0.000118316

Comparative mangrove metagenome reveals global prevalence of heavy metals and antibiotic resistome across different ecosystems

Madangchanok Imchen[1#], Ranjith Kumavath[1#]*, Debmalya Barh[2,3], Aline Vaz[4], Aristóteles Góes-Neto[4], Sandeep Tiwari[5], Preetam Ghosh[6], Alice R. Wattam[7], Vasco Azevedo[5]

Supplementary data 3: Kruskall Wallis comparison for significance among top 25 functional genes abundance from each sample. The differences were statistically significance at *p* value < 0.05 and are indicated in bold. Legend: SA: Saudi Arabia, BR: Brazil, ID: India

Functional Genes	S A vs ID	BR vs SA	BR vs ID
Ribonucleotide reductase of class II (coenzyme B12-dependent) (EC 1.17.4.1)	**0.021**	**0.021**	**0.021**
Aconitate hydratase 2 (EC 4.2.1.3)	**0.021**	**0.043**	**0.021**
Phage terminase	**0.021**	**0.021**	0.149
Phage protein	**0.021**	**0.021**	0.773
2',3'-cyclic-nucleotide 2'-phosphodiesterase (EC 3.1.4.16)	**0.021**	**0.043**	0.248
Asparagine synthetase [glutamine-hydrolyzing] (EC 6.3.5.4)	**0.043**	**0.043**	0.248
Aspartate aminotransferase (EC 2.6.1.1)	**0.043**	0.773	**0.043**
heterodisulfide reductase, subunit A/methylviologen reducing hydrogenase, subunit delta	**0.043**	0.564	**0.043**
5-methyltetrahydrofolate--homocysteine methyltransferase (EC 2.1.1.13)	**0.043**	0.248	**0.043**
Pyruvate-flavodoxin oxidoreductase (EC 1.2.7.-)	0.564	**0.021**	**0.043**
Glycogen phosphorylase (EC 2.4.1.1)	0.386	**0.043**	**0.043**
Excinuclease ABC subunit A	0.149	**0.021**	**0.021**
Arylsulfatase (EC 3.1.6.1)	0.773	**0.021**	**0.021**
Isoleucyl-tRNA synthetase (EC 6.1.1.5)	**0.021**	0.248	0.564
Phosphoribosylformylglycinamidine synthase, glutamine amidotransferase subunit (EC 6.3.5.3)	**0.043**	0.248	0.248
Biotin carboxylase of acetyl-CoA carboxylase (EC 6.3.4.14)	**0.043**	0.564	0.248
TonB-dependent receptor	0.773	**0.021**	0.248
Sarcosine oxidase alpha subunit (EC 1.5.3.1)	0.773	**0.043**	0.248
Adenylate cyclase (EC 4.6.1.1)	0.564	**0.043**	0.149
ATP synthase beta chain (EC 3.6.3.14)	0.248	**0.043**	0.149
decarboxylase	0.248	**0.043**	0.773
Adenosylmethionine-8-amino-7-oxononanoate aminotransferase (EC 2.6.1.62)	0.149	0.248	**0.021**
CoB--CoM heterodisulfide reductase subunit A (EC 1.8.98.1)	0.083	0.149	**0.021**
Topoisomerase IV subunit A (EC 5.99.1.-)	0.083	0.386	0.083
Cysteine desulfurase (EC 2.8.1.7)	0.083	0.564	0.773
Leucyl-tRNA synthetase (EC 6.1.1.4)	0.083	1.000	0.083
Translation elongation factor LepA	0.149	0.083	0.773
DNA polymerase III alpha subunit (EC 2.7.7.7)	0.149	0.248	1.000
Adenosylhomocysteinase (EC 3.3.1.1)	0.149	0.386	0.083
Isocitrate dehydrogenase [NADP] (EC 1.1.1.42)	0.149	0.773	0.149
Cell division protein FtsH (EC 3.4.24.-)	0.149	0.773	0.248
Putative deacetylase YgeY	0.248	0.083	0.083
Type I restriction-modification system, restriction subunit R (EC 3.1.21.3)	0.248	0.248	0.083
Cation efflux system protein CusA	0.248	0.248	0.773
Protein export cytoplasm protein SecA ATPase RNA helicase (TC 3.A.5.1.1)	0.248	0.386	0.773
Heat shock protein 60 family chaperone GroEL	0.248	0.564	0.248
Long-chain-fatty-acid--CoA ligase (EC 6.2.1.3)	0.248	0.773	0.386
Na+/H+ antiporter	0.248	0.773	0.564
DNA polymerase I (EC 2.7.7.7)	0.386	0.083	0.564
Glycosyltransferase	0.386	0.248	0.149
Lysyl-tRNA synthetase (class II) (EC 6.1.1.6)	0.386	0.564	0.564
Acetyl-coenzyme A synthetase (EC 6.2.1.1)	0.564	0.248	0.773
Glutamate synthase [NADPH] large chain (EC 1.4.1.13)	0.564	0.248	1.000
DNA topoisomerase I (EC 5.99.1.2)	0.564	0.564	0.149
ATP-dependent protease La (EC 3.4.21.53) Type I	0.564	0.564	0.564
Methylmalonyl-CoA mutase (EC 5.4.99.2)	0.564	0.564	0.773
Ferredoxin	0.564	1.000	0.564
Carbamoyl-phosphate synthase large chain (EC 6.3.5.5)	0.773	0.248	0.248
ClpB protein	0.773	0.248	0.773
Valyl-tRNA synthetase (EC 6.1.1.9)	0.773	0.564	0.773
SSU ribosomal protein S1p	0.773	0.773	0.248
Glycyl-tRNA synthetase (EC 6.1.1.14)	0.773	0.773	0.773
Copper-translocating P-type ATPase (EC 3.6.3.4)	0.773	0.773	1.000
Chaperone protein DnaK	0.773	1.000	0.773
2,4-dienoyl-CoA reductase [NADPH] (EC 1.3.1.34)	0.773	1.000	1.000
Translation elongation factor Tu	0.773	1.000	1.000
Acriflavin resistance protein	1.000	0.083	0.564
DNA primase (EC 2.7.7.-)	1.000	0.149	0.248
TRAP-type C4-dicarboxylate transport system, large permease component	1.000	0.149	0.564
SSU ribosomal protein S19p (S15e)	1.000	0.248	0.386
Translation elongation factor G	1.000	0.386	0.564
Excinuclease ABC subunit B	1.000	0.386	0.773
DNA gyrase subunit A (EC 5.99.1.3)	1.000	0.564	1.000
DNA-directed RNA polymerase beta' subunit (EC 2.7.7.6)	1.000	0.773	0.564
DNA-directed RNA polymerase beta subunit (EC 2.7.7.6)	1.000	1.000	1.000

Comparative mangrove metagenome reveals global prevalence of heavy metals and antibiotic resistome across different ecosystems

Madangchanok Imchen[1#], Ranjith Kumavath[1#]*, Debmalya Barh[2,3], Aline Vaz[4], Aristóteles Góes-Neto[4], Sandeep Tiwari[5], Preetam Ghosh[6], Alice R. Wattam[7], Vasco Azevedo[5]

Supplementary data 4: A comparative statistical analysis highlighting the significant ($p < 0.05$) changes in Ocean (O), Mangroves (M), and Terrestrial (T) ecosystems.

Subsystems (level3)	O vs. M	O vs. T	M vs. T
Zinc resistance	**<0.001**	**<0.001**	**<0.001**
MexE-MexF-OprN Multidrug Efflux System	**<0.001**	**<0.001**	**0.002**
The mdtABCD multidrug resistance cluster	**<0.001**	**<0.001**	**0.004**
Methicillin resistance in *Staphylococci*	**<0.001**	**<0.001**	**0.005**
Cobalt-zinc-cadmium resistance	**<0.001**	**<0.001**	**0.014**
Resistance to fluoroquinolones	**<0.001**	**<0.001**	0.383
Multidrug efflux pump in *Campylobacter jejuni* (CmeABC operon)	**<0.001**	**<0.001**	0.454
BlaR1 Family Regulatory Sensor-transducer Disambiguation	**<0.001**	**<0.001**	0.835
Copper homeostasis: copper tolerance	**<0.001**	**<0.001**	0.934
Polymyxin Synthetase Gene Cluster in Bacillus	**<0.001**	**0.004**	0.861
Cadmium resistance	**<0.001**	0.087	**<0.001**
Erythromycin resistance	**<0.001**	0.318	0.022
Arsenic resistance	**<0.001**	0.739	**0.002**
Beta-lactamase	**<0.001**	0.868	0.114
Fosfomycin resistance	**0.002**	**0.024**	0.453
Multidrug Resistance Operon mdtRP of Bacillus	**0.002**	0.055	0.248
Bile hydrolysis	**0.003**	0.081	0.145
Aminoglycoside adenylyltransferases	**0.006**	0.109	0.335
Mercury resistance operon	**0.007**	0.834	**0.011**
MexA-MexB-OprM Multidrug Efflux System	**0.008**	**0.010**	0.564
Mercuric reductase	**0.026**	0.124	0.318
Multidrug Resistance Efflux Pumps	**0.029**	**<0.001**	**<0.001**
Streptococcus pneumoniae Vancomycin Tolerance Locus	**0.036**	0.124	0.592
Resistance to Vancomycin	**0.043**	**<0.001**	**<0.001**
Streptothricin resistance	0.074	0.124	0.898
Copper homeostasis	0.090	0.934	0.146
Multiple Antibiotic Resistance MAR locus	0.103	0.523	0.400
Teicoplanin-resistance in *Staphylococcus*	0.151	1.000	0.178
Lysozyme inhibitors	0.342	0.962	0.419
Resistance to chromium compounds	0.522	**0.001**	**0.004**
Adaptation to d-cysteine	0.763	0.23	0.179
MexC-MexD-OprJ Multidrug Efflux System	1.000	0.285	0.285
